# Computational Prediction of One-Electron Oxidation Potentials for Cytosine and Uracil Epigenetic Derivatives

**DOI:** 10.1021/acs.jpca.4c06944

**Published:** 2025-04-08

**Authors:** Vasilii Korotenko, Patrick Langrzyk, Hendrik Zipse

**Affiliations:** † Thermal Separation Processes, TUHH, Denickestraße 22, 21073 Hamburg, Germany; ‡ Laboratory of Asymmetric Catalysis and Synthesis (LACS), 27218 EPFL, CH-1015 Lausanne, Switzerland; § Department of Chemistry, 9183 LMU München, Butenandtstrasse 5-13, 81377 München, Germany

## Abstract

Knowledge of the redox properties of cytosine (C), uracil (U), and their natural derivatives is essential for a deeper understanding of DNA damage, repair, and epigenetic regulation. This study investigates the one-electron oxidation potential (*E*_ox_, V) using DFT (B3LYP-D3) and DLPNO–CCSD­(T) methods with explicit/implicit (SMD) solvation model. Calculations in the gas phase and aprotic solvents such as acetonitrile showed a high correlation with experimental data (0.96–0.98). In aqueous solutions at pH 7, oxidation potentials are significantly influenced by deprotonation equilibria, as acidic molecules like 5caC become easier to oxidize upon deprotonation. The resulting oxidation potentials reflect a complex interplay of substituent effects, acidity, and protonation states. A pH-dependent model based on the Nernst equation for aqueous solutions demonstrated a correlation coefficient of 0.93. The calculated *E*_ox_ values for cytosine epigenetic derivatives in water, accounting for deprotonation effects, follow the trend: d_5caC < 5mC < 5caC < 5hmC < C < 5dhmC < 5fC, where “d_” deprotonated, “5ca” 5-carboxy, “5m” 5-methyl, “5hm” 5-hydroxymethyl, “5dhm” 5-dihydroxymethyl, “5f” 5-formyl.

## Graphical abstract

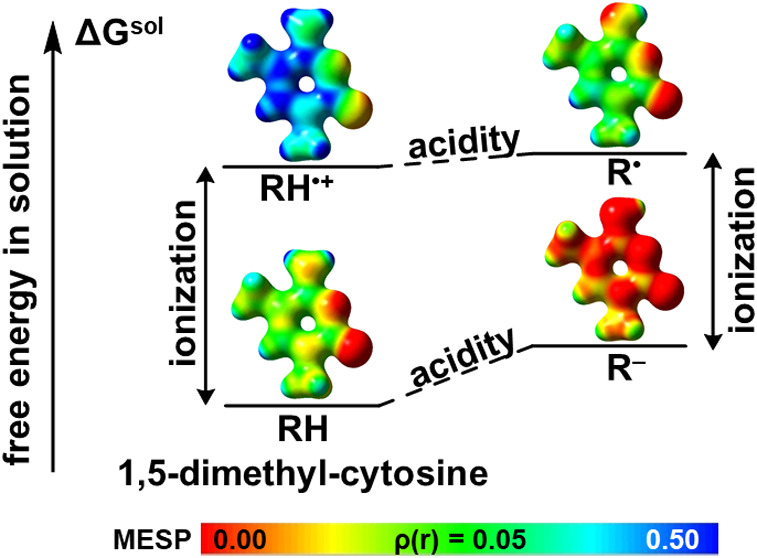

## Introduction

DNA damage, the discordant notes in the symphony of life, manifests in various forms—from single-strand or double-strand breaks in the sugar–phosphate backbone to the jarring cross-linking of DNA and proteins.^1^ These molecular missteps are often orchestrated by the relentless rhythm of metabolism, which produces reactive oxygen species (ROS),^2,3^ reactive nitrogen species (RNS),^4,5^ lipid peroxidation products,^6^ and alkylating agents^7^ that disrupt cellular harmony. Tens of thousands of such events occur in each cell daily.^8,9^ External factors, such as ionizing radiation^10^ or chemical mutagens,^11^ further amplify the disruption—high-energy radiation, for instance, ionizes DNA, triggering oxidative stress.

The oxidative stress theory of aging suggests that life’s melody falters under the relentless accumulation of oxidative damage to macromolecules like DNA. Supporting this idea, genetic interventions such as the overexpression of superoxide dismutase enzymes have been shown to extend lifespan in animal models, highlighting the potential to counteract oxidative stress.^12,13^

This is why studying one-electron oxidation is so crucial for understanding DNA damage. It is the first and most fundamental step, setting off a chain of chemical changes that can alter the structure and function of nucleic acids. By focusing on this initial event, we can better understand how oxidative stress affects DNA and what consequences it may have. Beyond the direct effects of oxidation, an important phenomenon emerges: the migration of the resulting electron hole through the DNA helix. This long-range charge transport—spanning hundreds of Ångströms^14–16^ —can lead to oxidative damage at sites far from the initial oxidation event.

Epigenetic modifications introduce complexity beyond the linear sequence of DNA’s four canonical nucleotides: adenine (A), thymine (T), guanine (G), cytosine (C). Epigenetic modifications of cytosine are recognized for their role in regulating gene expression in human cells and their implications in cancer development and other diseases.^17–20^ Cytosine, undergoes methylation by DNMT (DNA methyltransferase) at its 5-carbon position, creating 5-methylcytosine (5mC). This epigenetic mark can be removed (oxidized) by TET (ten-11 translocation) enzymes, which sequentially convert 5mC into 5-hydroxymethylcytosine (5hmC), 5-formylcytosine (5fC), and 5-carboxylcytosine (5caC).^17,18,21^ DNA repair mechanisms, including the base excision repair (BER) pathway and involving thymidine DNA glycosylase (TDG), process these oxidized derivatives to ensure genomic stability. Alternative pathways, including direct decarboxylation and deformylation of 5caC and 5fC, have also been observed in mammalian cells.^21–24^ (Figure 1) Additionally, pH-dependent hydration of 5fC generates 5-dihydroxymethylcytosine (5dhmC), a potential intermediate in oxidative processes.^25^ A biomimetic iron complex can also accelerate this oxidation in a manner similar to the TET enzyme.^26^ It was also demonstrated that 5-methylcytosine can undergo oxidation in water just by air without any specialized enzymes.^23^

**Figure 1:**
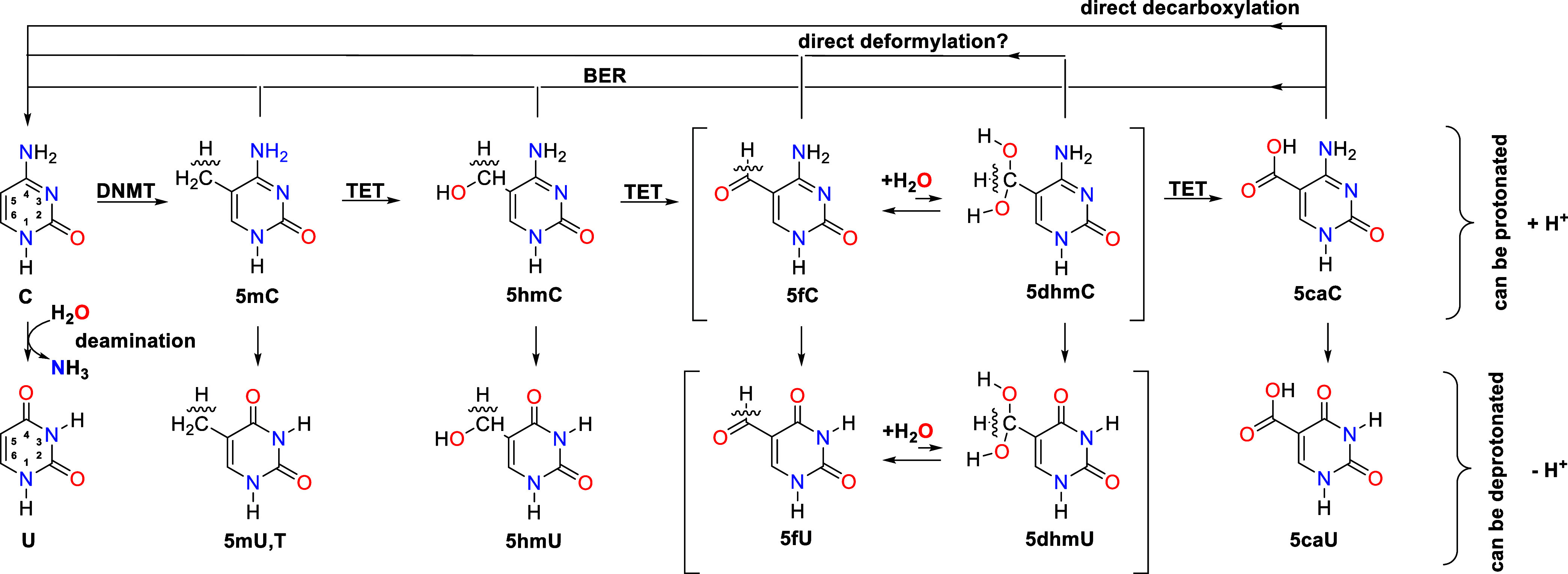
Epigenetic modifications of cytosine and their potential deamination products (uracil derivatives). Pathways for active methylation and oxidative demethylation of cytosine are shown, involving DNA methyltransferases (DNMT), ten-11 translocation enzymes (TET), and base excision repair (BER), along with possible direct deformylation and decarboxylation.^17,18,21^ The square brackets indicate the probable diol form of 5fC, accounting for its hydration to 5dhmC.^25^.

In addition to its role in epigenetic regulation, cytosine is susceptible to spontaneous chemical modifications, among which deamination holds particular significance—a process in which cytosine is converted into uracil, potentially leading to mutations in the genome.^27^ Interestingly, deamination can occur even in the absence of enzymes,^23^ especially at elevated temperatures, as might have been the case four billion years ago when Earth was a hot, dynamic environment with conditions vastly different from those of today.^28^ This suggests that the deamination of cytosine could have served as a mechanism for rapid evolution.^29^ It is important to note that under modern conditions, 5-methylcytosine deamination competes with other processes, such as its oxidation to 5-hydroxymethylcytosine, which can significantly impact epigenetic regulation and DNA repair mechanisms, and may have a potential link to the development of various diseases.^30^ Therefore, we also examined the direct products of epigenetically modified cytosine deamination—uracil (U) derivatives—presented in Figure 1.

The redox properties of cytosine modifications, such as oxidation potential and electron affinity, influence both oxidative damage and charge transport through DNA. Modifications at the 5-carbon position can alter these properties by donating or withdrawing electrons.^31^ This study aims to determine how epigenetic modifications of cytosine (5m-, 5hm-, 5f-, 5dhm-, and 5ca-) impact its redox behavior.

## Methodology

The standard redox potential (E°) quantifies a chemical species’ ability to participate in redox reactions, representing the energy required to transfer an electron under standard conditions (symbol “°” denotes standard conditions): temperature of 298.15 K (25 °C), ion concentration of 1 M and pressure of 1 atm. In redox processes, it is essential to distinguish between reduction and oxidation reactions.

An example of a reduction reaction is the addition of an electron to form a radical anion: RH + e^–^ → RH^–•^, where RH is a molecule containing acidic hydrogens H. This process is characterized by the reduction potential (*E*_red_^°^), which defines the potential at which a substance accepts an electron, transitioning from its oxidized to its reduced form.

The relationship between the free energy and the reduction potential (*E*_red_^°^) is expressed as

1 $$E_{red}^{{}^{\circ}}=\frac{-\Delta G_{red}^{{}^{\circ}}}{nF}-ref$$

where *n* is the number of electrons involved in the reaction, *F* is Faraday’s constant, Δ*G*_red_^°^ is the change in standard free energy under standard conditions, and “ref” is the reference potential. In aqueous solutions, the standard hydrogen electrode (SHE) is often used as the reference, with an absolute potential of +4.281 V at pH = 0 ([H^+^] = 1 M).^32–34^ For experiments in acetonitrile, the saturated calomel electrode (SCE) is commonly used, with an absolute potential of +4.429 V.^33^ The negative sign (−Δ*G*_red_^°^) is necessary because Δ*G*_red_^°^ is typically negative for spontaneous reduction reactions, while the reduction potential is positive when the reduction is thermodynamically favorable.

In this work, we focus on the oxidation potential—the potential at which a substance donates an electron, transitioning from its reduced form to its oxidized form. For example, the one-electron oxidation reaction forming a radical cation: RH → RH^+•^ + e^–^. The relationship between free energy and the oxidation potential is expressed as

2 $$E_{\mathrm{ox}}^{{}^{\circ}}=\frac{\Delta G_{\mathrm{ox}}^{{}^{\circ}}}{\mathrm{nF}}-\mathrm{ref}$$

where the negative sign is not required because Δ*G*_ox_^°^ is positive, as oxidation is thermodynamically unfavorable.

It is important to note that *E*_red_^°^ and *E*_ox_^°^ can be related as *E*_red_^°^ = –*E*_ox_^°^. However, this is valid only for opposite processes, for example, when oxidation occurs as RH → RH^+•^ + e^–^ and the corresponding reduction process is RH^+•^ + e^–^ → RH.

The one-electron oxidation potential can be calculated using the Born–Haber cycle shown in Figure 2. The top part of cycle represents gas phase processes, and the lower part depicts the solvent phase. Oxidation potential is depending on ionization energy (Δ*G*_IE_^gas^), which is defined by the phase change from the neutral state (RH) to its oxidized cation state (RH^•+^)­

3 $$\Delta G_{ox}^{sol}\left(\mathrm{RH}\right)=\Delta G_{IE}^{sol}\left(\mathrm{RH}\right)=\Delta G_{IE}^{gas}+\Delta G^{solv}\left({RH}^{•+}\right)-\Delta G^{solv}\left(\mathrm{RH}\right)$$

where “gas” (^gas^) symbol denotes a standard state of 1 atm in the gas phase and “solution” (^sol^) denotes 1 mol l^–1^ in the solution phase.

**Figure 2:**
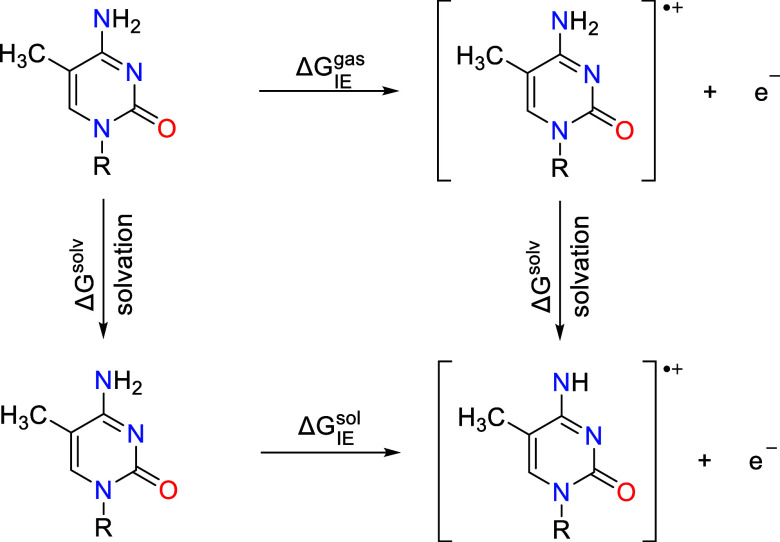
Thermodynamic cycle used in the calculation of one-electron oxidation potential in the solution phase.

Knowing the gas-phase ionization energy (Δ*G*_IE_^gas^) and the solvation energies (ΔG^solv^) of the oxidized and reduced forms, the free energy of oxidation in solution (Δ*G*_ox_^sol^) can be calculated. The ionization energy can be determined using different approaches, such as the adiabatic ionization energy (AIE), which accounts for full geometric optimization, or the vertical ionization energy (VIE), which characterizes the transition from a fixed geometry.

In our work, we used optimized structures of reactants and products, and therefore selected AIE, which is the total energy difference between the fully optimized neutral molecule and its fully optimized cation radical in the gas phase. For the molecule RH, AIE can be calculated^35,36^ using the following equation

4 $$\mathrm{AIE}={E^{\mathrm{gas}}\left({\mathrm{RH}}^{•+}\right)}_{\mathrm{opt}.\mathrm{geom}.}-{E^{\mathrm{gas}}\left(RH\right)}_{\mathrm{opt}.\mathrm{geom}.}$$

where *E* ^gas^(RH^•+^)_opt.geom._ is the total energy of the optimized cation radical, and *E* ^gas^(RH)_opt.geom._ is the total energy of the optimized neutral molecule. The adiabatic ionization potential is experimentally measured using photodissociation spectroscopy or through thermochemical cycles that account for the energies of the reaction products. The vertical ionization potential is determined using photoelectron spectroscopy (PES), which measures the energy required to eject an electron from a molecule in its nonequilibrium geometry.

Everything discussed so far relates to the calculation of the standard oxidation potential, *E*_ox_^°^, which describes the potential under ideal, standard conditions. However, in real systems, where variables such as temperature, concentration, and pH (often around 7 for biological systems) can differ, the behavior of the potential may change significantly. To account for these variations, the Nernst equation is used, linking the standard potential to real-world conditions

5 $$E_{\mathrm{ox}}=E_{\mathrm{ox}}^{{}^{\circ}}+\frac{\mathrm{RT}}{\mathrm{nF}}\ln\frac{\left(\mathrm{product}\;\mathrm{of}\;\mathrm{activities}\;\mathrm{of}\;\mathrm{oxidant}\right)}{\left(\mathrm{product}\;\mathrm{of}\;\mathrm{activities}\;\mathrm{of}\;\mathrm{reductant}\right)}$$

Here, *E*_ox_ is the potential under real conditions, *E*_ox_^°^ is the standard potential, *R* is the universal gas constant 8.31446 in J·mol^–1^·K^–1^, *T* is the temperature in Kelvin, n is the number of electrons involved in the reaction, *F* is Faraday’s constant 96485.3 C/mol, and in parentheses are the product of the activities of the reductant and oxidant. Thus, *E*_ox_^°^ serves as the starting point, while the Nernst equation adapts it to describe the real chemical environment by adding a corrective activities dependent term, bringing the calculations closer to experimental observations. The oxidation potential is experimentally measured using electrochemical methods such as cyclic voltammetry. These experiments employ a three-electrode system comprising a working electrode (e.g., glassy carbon or platinum), a reference electrode (e.g., SHE, Ag/AgCl), and a counter electrode (e.g., platinum wire or mesh). In the Nernst equation, when the activities of the oxidant and reductant are equal, the potential is called the “midpoint”. At this point, *E* corresponds to *E*_h_ (*E*_half‑cell_ or *E*_1/2_), the average potential between oxidation and reduction, describing the equilibrium state of the reaction. In cyclic voltammetry, (Table 1) it corresponds to the peaks on the voltammogram, where the transition between the oxidized and reduced forms occurs.

**Table 1:** Experimental Values of the Oxidation Potential in Aqueous (aq.) and Acetonitrile (ac.) Solution *E*_ox_ (in V), p*K*_a_ Values and Gas Phase Adiabatic Ionization Energy (AIE, in eV) for Chosen Compounds^a^ ^,^ ^b^

									p*K*_a,(aq.)_ multiplicity = 1			
compound		AIE		*E*_ox,(ac.)_		*E*_ox,(aq.)_		pH	*q* = +1; p*K*_a(+1)r_	*q* = 0; p*K*_a(0)r_	*q* = −1; p*K*_a(−1)r_	
adenine	A	8.26	^44^	1.72	^45^	1.32	^46^	7.0	4.2	9.8		^46^
thymine	T	8.87	^44^	1.87	^45^	1.29	^46^	7.0		9.9	>13.0	^46^
guanine	G	7.77	^44^	1.25	^45^	1.04	^46^ ^c^	7.0	3.1 ^35^	9.2	12.2	^46^
						0.89	^46^	10.2				
cytosine	C	8.68	^44^	1.90	^45^	1.44	^46^ ^c^	7.0	4.6	12.2	>13.0	^46^
						1.39	^46^	9.0				
uracil	U	9.32	^44^	>2.15	^45^	1.34	^46^ ^c^	7.0		9.5		^46^
						1.16	^46^	11.6				
guanosine	rG					1.29	^47,48^	7.0	1.8	10.2		^d^
1-methyl-guanine	1mG					1.06	^46^	7.0	3.2	10.4		
xanthine	X					0.93	^46^	7.0	1.2	7.5	11.0	^46^
hypoxanthine	hX					1.16	^46^ ^c^	7.0		8.9	12.1	^46^
						1.05	^46^	9.0				
1-methylindole	1mInd	7.40	^49^			1.23	^50^	7.0				
indole	Ind	7.76	^49^			1.24	^50^	7.0		21.0		^51^
phenol	PhOH	8.51	^52^			1.35	^53^	7.0		10.0		^54^
thioanisole	tAn	7.93	^55^			1.45	^56^	7.0				
4-methylaniline	4 mAnl	7.37	^57^	0.78	^35^				5.1			^58^
anisole	An	8.24	^55^	1.62	^59^							
naphthalene	Napht	8.14	^55^	1.54	^60^							
caffeine	Caf			1.20	^61^	1.45	^62^	6.0	0.7			^63^

a *q* – is the charge of the deprotonating molecule (acid).

b *E*_ox_ (aqueous) values:^46^ Faraggi et al. measured nucleobase potentials by cyclic voltammetry in aqueous solution^47,48^ Steenken et al. measured nucleoside potentials using kinetic rate measurement in aqueous solution^64^ Xie et al. measured nucleobase potentials by cyclic voltammetry in aqueous solution^65^ Fukuzumi et al. measured DNA nucleotide potentials by cyclic voltammetry in aqueous solution.

c Estimated value obtained by extrapolation, assuming a linear increase of 60 mV per pH unit and the p*K*_a_ value of the base. p*K*_a_ values:

d Predicted by ChemAxon AIE values:^44^ Estimated accuracy of ± 0.05 V^49^ two-laser photoionization supersonic jet mass spectrometry^52^ the electric field dependence of the pump–probe photoionization threshold^55,57^ Photoelectron spectroscopy.

In our study, we consider *E*_ox_^°^ as a simplified model for estimating the oxidation potential under ideal conditions, allowing us to focus on the fundamental thermodynamic characteristics of the system (a simplified relationship between free energy and potential). On the other hand, *E*_ox_, which accounts for real conditions such as temperature, concentration, and pH, represents a more comprehensive and complex model. The choice between these approaches depends on the research goals: *E*_ox_^°^ is suitable for analyzing fundamental properties, while *E*_ox_ is used to study system behavior under more realistic conditions.

Authors employing adiabatic^35–38^ and vertical^38–42^ ionization energies to estimate the standard oxidation potential *E*_ox_^°^ report acceptable systematic errors and very strong correlation with experimental data in the gas phase. However, in aqueous solutions, the use of simplified models^35–41,43^ to calculate *E*_ox_^°^ and compare it with experimental *E*_ox_ values (*E*_h_ values measured under nonideal conditions) often results in significant errors, typically overestimated values. Nonetheless, the overall trend in calculated potentials *E*_ox_^°^ often aligns with experimental data, particularly for standard nucleobases such as adenine (A), thymine (T), guanine (G), cytosine (C), and uracil (U).

In the literature, attempts to evaluate correlations between solution phase theoretically calculated oxidation potentials and experimentally measured in aqueous solution *E*_ox_ values using linear regression are notably absent. Figure 3 presents a diagram that compares the experimental AIE values, which refers to gas-phase measurements without accounting for solvation effects, and experimental oxidation potential in (a) acetonitrile and (b) aqueous solution. It should also be considered that in polar solvents, such as water, solvation effects can play an important role in the stabilization of charged particles, such as the radical cation, anion. It is also known, that even in acetonitrile solution solvent molecules can also interact with radical cation and stabilize it.^66^ In the case of the acetonitrile solution, a correlation with the values obtained in the gas phase is visible; that is, the solvent effect of (a) acetonitrile only shifts the energies of single-electron oxidation but does not change the chemistry of the oxidation process shown in Figure 2. In the case of (b) aqueous solution, the absence of correlation indicates that the oxidation chemistry in water differs significantly from that in the gas phase. This may discourage many authors from conducting such analyses, leading them to focus solely on absolute error evaluations. Additionally, this suggests that the factors influencing the single-electron oxidation process in water and in the gas phase are sufficiently different.

**Figure 3:**
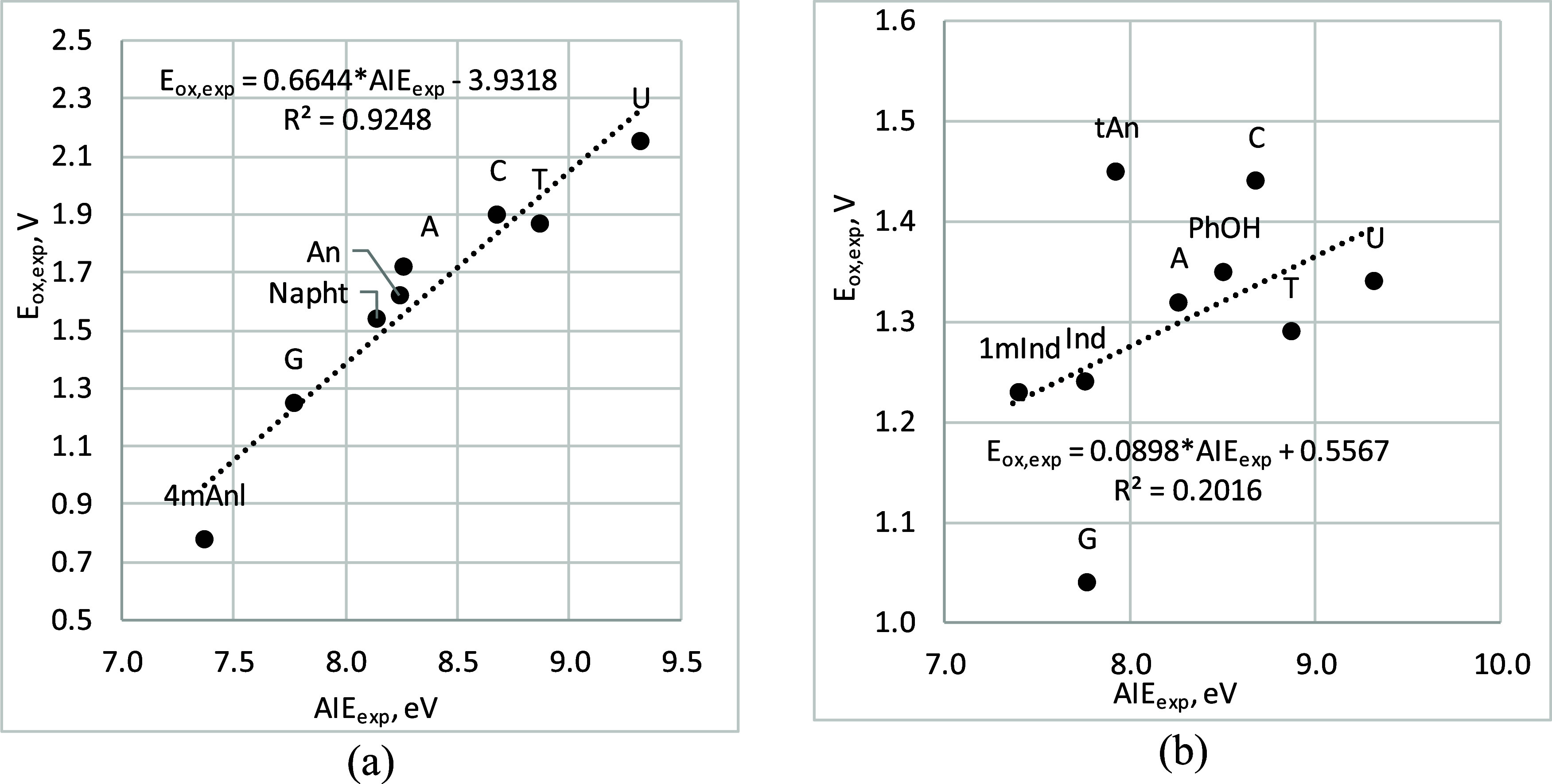
Diagram between experimental values of gas phase AIE and oxidation potential in (a) acetonitrile and (b) aqueous solution.

This also highlights the importance of not only calculating *E*_ox_^°^ but also transitioning to more complex models that incorporate the Nernst eq 5. By accounting for additional effects influencing the measured potential values, such an approach can improve correlation or at least attempt to enhance it. For instance, authors^35,36,67^ applied the Nernst equation and a specific formulation of the activities dependent term by Wardman^68^ and Clark,^69^ incorporating acidity constants (*K*_a_) to accurately describe the activities at pH = 7 (pH/*K*_a_ dependent term), which helped reduce calculation errors. The authors also emphasized the importance of Boltzmann averaging not only across all conformers but also among all possible tautomers, as tautomerism can significantly affect both oxidation potentials and p*K*_a_ values. Unfortunately, no attempt to evaluate the correlation between theory and experiment was made. Therefore, in our work, we will also employ Boltzmann averaging for all conformers and tautomers of each neutral or deprotonated molecule, in both oxidized and reduced states, and we will attempt to assess the correlation between theory and experiment.

Many authors emphasize the importance of accurately determining p*K*_a_ values for properly accounting for the influence of pH on the oxidation potential of DNA bases. Deprotonation is crucial because it significantly impacts the overall standard oxidation potential (*E*_ox_^°^) and can alter the reactivity order among different acidic compounds. Without considering deprotonation, predicted values may deviate from experimental results, particularly in cases where proton loss is strongly coupled with the oxidation process.^35–37,40,67^ Dissociation constants (*K*_a_) can be determined by quantum chemical calculations with satisfactory accuracy at the molecular level, employing either absolute or relative methods.^70–74^ However, achieving chemical accuracy remains challenging, as a computed free energy difference of 5.6 kJ mol^–1^—state-of-the-art for gas-phase energies^75–79^ —leads to an error of one p*K*_a_ unit in de/protonation reactions.

Authors of many studies^79–87^ have conducted successful p*K*_a_ calculations for nucleic acids using various quantum chemical methods and solvation models to achieve results that align well with experimental data. However, this task is technically demanding, often requiring careful selection of methods and validation of multiple approaches to ensure reliable correlation with experiments. Researchers frequently adjust cavity parameters in continuum solvation models^35^ and incorporate explicit solvent molecules, such as one or more water molecules,^88^ for a more accurate description of solvation effects. These effects are particularly critical for protonated or deprotonated species, as charged particles tend to form dense solvation shells due to hydrogen bonding networks.^89^ While mixed solvation models, combining explicit water molecules with implicit continuum methods, are possible, their application requires caution.^90^

### Model 1 (M1)

For convenience, we number the models used in this work. The first model for calculating the standard oxidation potential *E*_ox_^°^ is appropriate for aprotic solvents or even for aqueous solution, if the experimental pH is far enough away from the p*K*_a_ of the molecule under study (neutral reduced species), and if the process depicted in Figure 2 truly reflects the chemistry of the oxidation process

6 $$E_{\mathrm{ox}}^{{}^{\circ}M1}\left({\mathrm{RH}}^{+•}\right|\mathrm{RH})=\frac{\Delta G_{\mathrm{IE}}^{\mathrm{sol}}}{\mathrm{nF}}-\mathrm{ref}$$

### Model 2 (M2)

However, each protic compound has acid/base properties in aqueous solution, characterized by their corresponding p*K*_a_ values, indicating the potential for deprotonation of the studied molecule. As a rule, it is easier to oxidize a negatively charged molecule (the deprotonation product, which is electron-rich) than a neutral one, as this process requires lower energy compared to the oxidation of the corresponding neutral species.

The free energy diagram on Figure 4 helps to visualize the energy changes and intermediates involved in the one-electron oxidation and deprotonation reactions that can happen with nucleic bases in the solution phase. In the case of a dissociating molecule (R-H), there are two possible one-electron oxidation pathways: the free energy of the first pathway includes Δ*G*_IE(0)r_^sol^, while the free energy of the second pathway includes Δ*G*_IE(−1)r_^sol^. The subscript in parentheses indicates the charge of the original molecule that will be oxidized, either zero (neutral) or minus one (deprotonated). The letter “r” indicates that this molecule is in its original, unoxidized, or reduced state. The values of Δ*G*_a(+1)o_^sol^ and Δ*G*_a(0)r_^sol^ (letter “a” stands for acidity) are free energies of deprotonation and are described by the p*K*_a_ values

7 $$\Delta G_{a}^{sol}=-\mathrm{RT}\;\ln K_{a}=2.303\;\mathrm{RT}\;pK_{a}$$

8 $${pK}_{a}=-\log_{10}\;K_{a}=-\log_{10}\frac{(base)\left(H^{+}\right)}{\left(acid\right)}$$

where activities are denoted by parentheses and are unitless (while concentrations are usually represented by square brackets). In the general case, the following holds true: Δ*G*_a(+1)o_^sol^ < Δ*G*_a(0)r_^sol^

**Figure 4:**
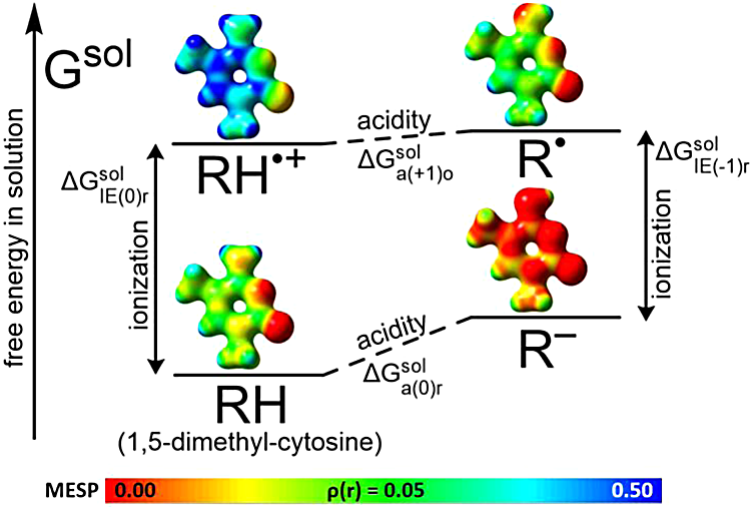
Free energy diagram representing a one-electron oxidation and deprotonation processes in the solution phase. Relative and absolute G^sol^ values of each reactant/product depend on the compound being considered. MESP:^91,92^ The molecular electrostatic potential (0.00–0.50 au) mapped onto the electron density isosurface ρ­(r) at a value of 0.05 au, obtained from SMD/DFT.

Since ΔG_IE(−1)r_^sol^ < Δ*G*_IE(0)r_^sol^ is always, and the measured value of Δ*G*_IE,exp_^sol^ can be somewhere between Δ*G*_IE(−1)r_^sol^ and Δ*G*_IE(0)r_^sol^ values (Δ*G*_IE(−1)r_^sol^ < Δ*G*_IE,exp_^sol^ < Δ*G*_IE(0)r_^sol^) and will depend on the concentrations (or activities) ratio [R^–^] and [RH]. At the thermodynamic equilibrium of the system, the [R^–^]/[RH] ratio at a given pH value can be estimated using the Henderson–Hasselbalch equation

9 $$\mathrm{pH}=pK_{a}+\log_{10}\frac{\left[R^{-}\right]}{\left[\mathrm{RH}\right]}=pK_{a}+\log_{10}\frac{x_{R^{-}}}{x_{\mathrm{RH}}}$$

where *x* is the mole fraction of the corresponding neutral acid RH and ionized base R^–^. If the p*K*_a_ value of the oxidized product is known the eq 9 can be also used to calculate the mole fractions of the cationic radical acid RH^•+^ and neutral deprotonated radical base R^•^.

Equation 9 can be converted (see SI) into an expression for the extent of ionization depending on pH and p*K*_a_ values^93^ as follows

10 $$x_{\mathrm{RH}}=\frac{1}{1{+10}^{\mathrm{pH}-pK_{a}}}$$

11 $$x_{R^{-}}=1-x_{\mathrm{RH}}$$

If we simply assume that the probability of an electron being detached from a particle is equal to its molar fraction value *x* (regardless of the detachment energy), then we can apply *x* as a coefficient contributing to the resulting oxidation potential. Thus, we come to the second model (M2), which depends on the pH value

12 $$E_{\mathrm{pH}}^{{}^{\circ}M2}\left({\mathrm{RH}}^{+•};R^{•}\right|\mathrm{RH};R^{-})=\frac{x_{\mathrm{RH}}*\Delta G_{\mathrm{IE}\left(0\right)r}^{\mathrm{sol}}+x_{R^{-}}*\Delta G_{\mathrm{IE}\left(-1\right)r}^{\mathrm{sol}}}{\mathrm{nF}}-\mathrm{ref}$$

This expression uses the Henderson–Hasselbalch equation, all protonation levels (shown in Figure 4) of the reduced and oxidized species are considered. We notice, if p*K*_a_ > pH, then increasing the value of p*K*_a_ leads to *x*_RH_ → 1 and *x*_R^–^_ → 0. The contribution coefficients x (molar fractions) are directly connected to the free energy of the deprotonation reaction. They reflect the relative population of different molecular forms under specific temperature and conditions, as described by the Boltzmann distribution. Thus, the Boltzmann-averaged value *E*_pH_^°M2^ incorporates the contribution of each molecular form, weighted by its energy state. This approach enables the averaging of *E*° values, providing a more accurate representation of the standard potential for the mixture of forms.

### Model 3 (M3)

According to Wardman^68^ it is important to take into account the coupling of electrons and protons (prototropic equilibrium) in the reaction, which should be included in the Nernst relationship eq 5. In the SI, we define the acid–base dissociation constants of the reductant and oxidant, express their activities in the Nernst equation, and derive an expression for the equilibrium in Figure 4. The obtained activities can then be substituted into the Nernst equation. Let us do this for only one (left) oxidation pathway in Figure 4: RH ⇌ RH^+•^. In this case, we exclude the contribution of the reaction R^–^ ⇌ R^•^ in the resulting value of *E*_h_

13 $$E_{\mathrm{pH}}^{M3}=E^{{}^{\circ}}+\frac{\mathrm{RT}}{F}\ln\frac{\left({\mathrm{RH}}^{+•}\right)}{\left(\mathrm{RH}\right)}=E^{{}^{\circ}}+\frac{\mathrm{RT}}{F}\ln\frac{\left(10^{-\mathrm{pH}}+K_{a\left(0\right)r}\right)}{\left(10^{-\mathrm{pH}}+K_{a\left(+1\right)o}\right)}$$

Thus, M3 provides a correction for the prototropic equilibrium that is added to the *E*° value. Following *E*°, the pH/*K*_a_ dependent term $\frac{\mathrm{RT}}{F}\ln\frac{\left(10^{-\mathrm{pH}}+K_{a\left(0\right)r}\right)}{\left(10^{-\mathrm{pH}}+K_{a\left(+1\right)o}\right)}$ accounts for the main effect of pH and the acidity constants *K*_a_ of the oxidized and reduced states on the redox potential of the left oxidation pathway in Figure 4: RH ⇌ RH^+•^.

For the molecules calculated in this work, the pH/*K*_a_ dependent term <0 V because in all cases, the radical cation is significantly more acidic than the neutral reduced molecule, with *K*_a(0)r_ > *K*_a(+1)o_. For example, with *K*_a(0)r_ = 0 and *K*_a(+1)o_ = 1, the pH/*K*_a_ dependent term = −0.41 V. The more acidic the radical cation, the more negative the value of the term (delta M3) will be.

The specific value of *E*° to be used for calculations depends on the system. For example, if the value of *x*_R^–^_ at a given pH is low, we can use *E* ^°M1^(RH^+•^|RH). For more acidic molecules, we should expect a greater contribution from the deprotonated form of the molecule and a higher *x*_R^–^_ value. In this case, it is appropriate to use *E*_pH_^°M2^ = *E*°(RH^+•^;R^•^|RH;R^–^) or *E* ^°M1^(R^•^|R^–^) when *x*_R^–^_ is close to 1.

### Model 4 (M4)

As shown in Figure 4, both the RH^+•^ cation radical and its deprotonated form R^•^ can act as oxidizing agents, while the neutral RH molecule and its deprotonated form R^–^ can act as reducing agents. In this case, additional active components can be incorporated into the Nernst equation to express the value of *E*_h_

14 $$E_{\mathrm{pH}}^{M4}=E^{{}^{\circ}}+\frac{\mathrm{RT}}{F}\ln\frac{\left({\mathrm{RH}}^{+•}\right)}{\left(\mathrm{RH}\right)}\frac{(R^{•})\left(H^{+}\right)}{(R^{-})\left(H^{+}\right)}=E^{{}^{\circ}}+\frac{\mathrm{RT}}{F}\ln\frac{K_{a\left(+1\right)o}}{K_{a\left(0\right)r}}+2\frac{\mathrm{RT}}{F}\ln\frac{\left(10^{-\mathrm{pH}}+K_{a\left(0\right)r}\right)}{{(10}^{-\mathrm{pH}}+K_{a\left(+1\right)o})}$$

In this formula, following *E*°, two terms can be seen. The first p*K*_a_ dependent term $\frac{\mathrm{RT}}{F}\ln\frac{K_{a\left(+1\right)o}}{K_{a\left(0\right)r}}$ is always greater than 0 V, while the second pH/*K*_a_ dependent term $2\frac{\mathrm{RT}}{F}\ln\frac{\left(10^{-\mathrm{pH}}+K_{a\left(0\right)r}\right)}{{(10}^{-\mathrm{pH}}+K_{a\left(+1\right)o})}$ is always less than 0 V. Unlike M3, in M4, the second term is counted twice after algebraic transformations, as the added reactants (R^•^) and (R^–^) are described by the same acidity constants as (RH^+•^) and (RH), respectively. (See SI)

If we decompose the first term into its components and transform

15 $$\frac{\mathrm{RT}}{F}\ln\frac{K_{a\left(+1\right)o}}{K_{a\left(0\right)r}}=\frac{\mathrm{RT}}{F}\ln\frac{(R^{•})\left(H^{+}\right)}{\left({\mathrm{RH}}^{+•}\right)}\frac{\left(\mathrm{RH}\right)}{(R^{-})\left(H^{+}\right)}=\frac{\mathrm{RT}}{F}\ln\frac{(R^{•})\left(\mathrm{RH}\right)}{({\mathrm{RH}}^{+•})\left(R^{-}\right)}$$

it becomes obvious that it describes the reverse free energy (with a minus sign) of this endergonic acid–base (neutralization) reaction: RH^+•^ + R^–^ ⇌ R^•^ + RH. Thus, this term describes how all the activities in the Nernst equation for *E*_pH_^M4^ interact with each other participating in the neutralization reaction.

## Computational Details

In this work, all geometries were optimized using DFT at the SMD­(H_2_O)/(U)­B3LYP-D3/6–31+G­(d,p) level of theory^35^ with the SMD­(H_2_O) implicit solvent model^94^ using Gaussian09, Revision D.01.^95^ D3 version of Grimme’s dispersion was added to the method of optimization to account the dispersion interactions.^96,97^ Frequency calculations have been carried out to verify that the optimized structures are true minima. If an imaginary frequency appears in the structure, we fully displace the atoms along and against the displacement vectors of this vibrational mode with a negative frequency, save the resulting structures, and perform their reoptimization. Thermochemical corrections to *G* ^gas^ at 298.15 K were calculated in GoodVibes using quasi-harmonic approximation.^98^ For each studied nucleobase, all the stable tautomers and conformers were considered when calculating the Boltzmann-weighted *G* ^sol^ values. The individuality of the found conformers/tautomers was confirmed using an energy criterion (Δ*G* ^sol^ > 10^–7^ au) and comparing geometries by distances between each atom and the centroid point.^99,100^

Since the relative tautomeric stability of the studied molecules is not trivial^101^ and can change due to solvation effects, we systematically considered all possible proton arrangements on relevant oxygen and nitrogen atoms. Tautomers varied across lone pairs, allowing for the possibility of intermolecular proton transfer. Using the expression $C_{n}^{k}=\frac{n!}{\left(n-k\right)!•k!}$ , one can estimate the number of possible tautomers of an asymmetric molecule having n lone pairs and k acidic protons (*k* ⩽ *n*). The initial geometries of tautomers were created manually and then fully optimized.

The SMD­(H_2_O) implicit solvation model, supplemented with one explicit water molecule, was used to calculate redox potential values. The optimized water-complexed geometries were obtained through a stochastic search procedure, which generates structures with randomly arranged water molecules around the target compound.^102,103^ For each studied species, 100 random structures with one explicit water molecule were generated and fully optimized. This “brute force” approach was applied only to the most stable conformers/tautomers that contribute more than 2% to the Boltzmann-averaged *G* ^sol^ values.

The single point energies were also calculated for the optimized geometries by using DLPNO–CCSD­(T).^104–106^ Two-point (cc-pVTZ and cc-pVQZ) extrapolation was employed at the DLPNO–CCSD­(T) level of theory to estimate a result obtained using a complete (infinitely large) basis set (CBS).^105,107^ Standard state solvation free energies Δ*G* ^solv^ were computed as a difference between the solution phase free energy Δ*G* ^sol^ of the solution phase optimized molecule RH (SMD­(H_2_O)/(U)­B3LYP-D3/6–31+G­(d,p)), and the gas phase single point free energy *G* ^gas^ for the same geometry RH ((U)­B3LYP-D3/6–31+G­(d,p)//SMD­(H_2_O)/(U)­B3LYP-D3/6–31+G­(d,p))

16 $${\Delta G}^{\mathrm{solv}}=G^{\mathrm{sol}}\left(\mathrm{RH}\right)-G^{\mathrm{gas}}\left(\mathrm{RH}\right)$$

The solution phase single-point free energies *G* ^sol^ are computed by combining the gas phase free energies with the solvation free energies Δ*G* ^solv^

17 $$G^{\mathrm{sol}}=E_{\mathrm{tot}}^{\mathrm{single}‐\mathrm{point}}+{\mathrm{ZPE}}^{\mathrm{SMD}/\left(U\right)B3\mathrm{LYP}‐D3}+{\Delta G}_{0\;K\to 298\;K}^{\mathrm{SMD}/\left(U\right)B3\mathrm{LYP}‐D3}{+\Delta G}_{0\;K\to 298\;K}^{1\;a\mathrm{tm}\to 1\;M}+{\Delta G}^{\mathrm{solv}}$$

where Δ*G*_0K→298 K_^1 atm→1 M^ = +7.91 kJ mol^–1^ is the free energy difference for converting from the standard state concentration of 1 atm to the standard state concentration of 1 mol l^–1^.

## Results

## Conformational Search

Conformational analysis enabled the identification of multiple conformers in molecules with rotating groups, such as 5hmC, 5dhmC, and their uracil derivatives. For these molecules, potential energy surface (PES) scans were performed by systematically varying the torsion angles, allowing us to explore a broad conformational space and identify the most stable conformers. Furthermore, torsional scans were conducted for nearly all stable tautomers, regardless of their relative stability, ensuring a comprehensive and reliable conformational assessment.

For complexes with a single explicit water molecule, it is important to determine the most favorable position, ideally within the most stable conformer of the most stable tautomer. This task is more challenging, but the stochastic search method, which worked well for our system, made it possible to identify the most stable tautomer, conformer, and water position at the same time. This success is likely due to the molecular electrostatic potential, which creates strongly charged regions, especially where the charge is concentrated in the radical cation or the deprotonated form. When the water molecule is placed near this charged region, it naturally moves into a broad and deep global minimum, stabilizing through electrostatic interactions. With this approach, we identified optimal configurations with stable hydrogen bonds in neutral molecules as well as in radical cations and deprotonated forms.

For example, Figure 5 shows the Molecular Electrostatic Potential (MESP)^91,92^ for the most favorable by G^sol^ structures, with one water molecule. The results indicate that in 1m5mC and d_1m5mC (“d_” deprotonated), the most stable configuration features a hydrogen bond of the OHw···N type (“w” - water), involving an interaction with the lone electron pair on nitrogen N3. The MESP was calculated on an electron density (ED, ρ­(r)) isosurface of 0.05 au, which successfully captures the bonding pathway between water and the lone pair of nitrogen N3 in d_1m5mC. This emphasizes the strength of the hydrogen bond and suggests that the deprotonated form of the molecule is rich in electron density. In contrast, the most favorable structure in rc_1m5mC (“rc_” radical cation) forms a hydrogen bond of the NH···Ow type, whereas in rd_1m5mC (“rd_” radical deprotonated), the bond is of the OHw···O type. These observations highlight the crucial role of electrostatic interactions in stabilizing such configurations.^84^ In all cases, the hydrogen bonds exhibit a pronounced linearity, which is visually evident.

**Figure 5:**
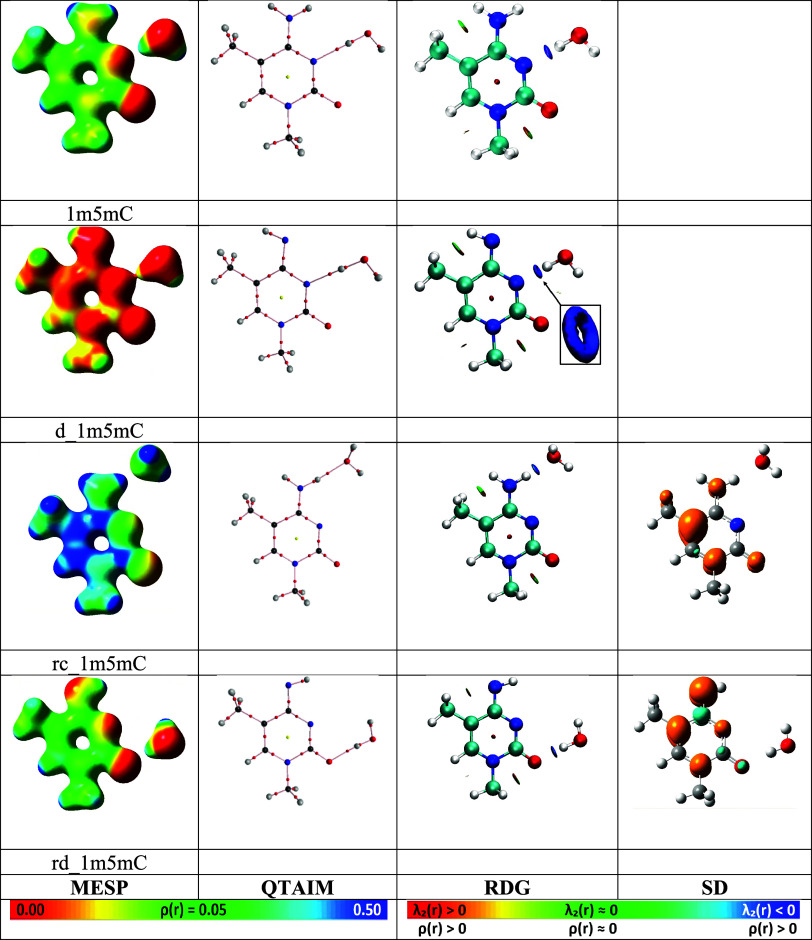
Most stable by G^sol^ values structures with one water molecule, calculated using SMD/DFT. **MESP**:^91,92^ The molecular electrostatic potential (0.00 – 0.50 au, left color scale) mapped onto the electron density isosurface ρ­(r) at a value of 0.05 au **QTAIM**:^108,109^ Analysis performed using AIM2000.^110^ **RDG**:^111^ Reduced Density Gradient analysis with an isovalue of 0.5 au colored (right color scale) by the function sign­[λ_2_(r)]*ρ­(r), where λ_2_(r)^108,109^ is the second eigenvalue of the Hessian matrix of the ED, calculated for ρ­(r) < 0.05 au using Multiwfn^112^ and visualized in VMD.^113^ **SD**: Spin density isosurface at 0.005 au, with orange representing α electrons and blue representing β electrons.

To explore the details of the ED topology of the H-bond, we conducted an analysis using the Quantum Theory of Atoms in Molecules (QTAIM).^108,109^ The results reveal the formation of bond critical points and a noncovalent bonding pathway between the water molecule and the studied molecules, confirming the presence of a well-defined hydrogen bond. Additionally, the ρ­(r) values at the bond critical points were found to be 0.040 au in 1m5mC, 0.052 in d_1m5mC, 0.033 in rc_1m5mC, and 0.037 in rd_1m5mC.

The Reduced Density Gradient (RDG) analysis^111,114–116^ provided additional insights into incomplete^117^ (without a bond critical point) intramolecular interactions that stabilize the molecular conformation. We also observe that in the region of the bond critical point, a characteristic “pancake” shape typical of hydrogen bonds is formed, colored in blue by the function sign­[λ_2_(r)]*ρ­(r), where λ_2_(r) is the second eigenvalue of the Hessian matrix of the ED. For example, in the case of d_1m5mC, instead of a simple “pancake,” a toroidal surface^118^ is formed. This “donut” shape is attributed to the specifics of the RDG calculation, which, by default in Multiwfn, is limited to regions of molecular space where the ED is below 0.05 au Thus, in the central region of the RDG isosurface, where the electron density exceeds the established threshold of 0.05 au, gradient analysis is not performed, resulting in the formation of a hole that serves as an indicator of increased electron density.

We also analyzed the spin density distribution (SD) for rc_1m5mC and rd_1m5mC. In rc_1m5mC, the highest concentration of spin density is observed on the fifth carbon atom C5. In rd_1m5mC, a significant portion of the spin density is localized on the deprotonated amino group, indicating its potential radical reactivity. Although this analysis is based on the SMD/DFT wave function, it is important to note, as highlighted in reference,^119^ that the choice of computational methods can significantly influence the spin density distribution. This was demonstrated in the context of single-electron oxidation of DNA bases in the referenced study. Furthermore, as observed, our radical cation is classified as a π-radical, which, as noted in reference,^120^ is generally more stable than σ-radicals due to its superior delocalization characteristics.

## Benchmark

Let us start with the simplest case—removing a single electron from a molecule in the gas phase using M1. As shown in Table 2, the theoretical gas-phase AIE values align remarkably well with experimental data. The same table also presents a linear regression analysis, described by the following equations (in gas phase)

18 $${\mathrm{AIE}}_{\exp}=\mathrm{slope}\times {\mathrm{AIE}}_{\mathrm{theor}}+\mathrm{intercept}$$

and (in solution phase)

19 $$E_{\exp}=\mathrm{slope}\;\times E_{\mathrm{theor}}+\mathrm{intercept}$$

where the subscript “exp” denotes the experimental values, and “theor” represents the theoretically calculated values. To provide a comprehensive evaluation, we also include key metrics such as the *R* ^2^, slope, intercept, Mean Signed Error (MSE), Mean Unsigned Error (MUE), and Root Mean Square Error (RMSE). Switching from the less expensive SMD/DFT method to the more advanced SMD/CBS approach in the gas phase leads to only a slight improvement in correlation, while noticeably reducing MSE and MUE values. Moving to a more complex system, such as an aprotic acetonitrile solution where protonation/deprotonation effects are minimal, the M1 model based on implicit solvation shows a strong correlation (*R* ^2^ ∼ 0.97, Table 2). We observe that for AIE values, the intercepts are positive for DFT and slightly negative for CBS in the gas phase. When transitioning to acetonitrile solution using SMD­(CH_3_CN), the intercept for DFT remains positive, whereas for CBS, it is approximately zero.

**Table 2:** Experimental and Calculated (M1) Values of the Gas Phase Adiabatic Ionization Energy (AIE, in eV) and Oxidation Potential *E*_ox(ac)_ in Acetonitrile Solution (in V, vs SCE)^a^

	AIE			*E*_ox(ac)_		
compound	DFT	CBS	experiment	SMD/DFT	SMD/CBS	experiment
A	8.01	8.29	8.26	1.42	1.70	1.72
T	8.68	8.92	8.87	1.66	1.90	1.87
G	7.62	7.81	7.77	1.03	1.26	1.25
C	8.44	8.69	8.68	1.54	1.69	1.90
U	9.16	9.35	8.68	2.01	2.21	>2.15
1mInd	7.25	7.52	7.40			
Ind	7.46	7.76	7.76			
PhOH	8.22	8.50	8.51			
tAn	7.65	7.93	7.93			
4 mAnl	7.14	7.44	7.37	0.52	0.83	0.78
An	7.93	8.23	8.24	1.33	1.63	1.62
Napht	7.74	8.08	8.14	1.23	1.57	1.54
***R*** ^**2**^	0.98	0.99		0.98	0.96	
**slope**	0.97	1.02		0.95	1.02	
**intercept**	0.45	–0.23		0.32	–0.02	
**MSE**	–0.25	0.03		–0.26	–0.01	
**MUE**	0.25	0.04		0.26	0.05	
**RMSE**	0.26	0.06		0.27	0.08	

a DFT = (U)­B3LYP-D3/6–31+G­(d,p)//SMD­(H_2_O)/(U)­B3LYP-D3/6–31+G­(d,p); CBS = DLPNO–CCSD­(T)/CBS//SMD­(H_2_O)/(U)­B3LYP-D3/6–31+G­(d,p); SMD/DFT = SMD­(CH_3_CN)/(U)­B3LYP-D3/6–31+G­(d,p); SMD/CBS = SMD­(CH_3_CN)/DLPNO–CCSD­(T)/CBS//SMD­(CH_3_CN)/(U)­B3LYP-D3/6–31+G­(d,p).

While sipping a cup of coffee, we got the idea to analyze the caffeine molecule (1,3,7-trimethylxanthine). For caffeine, calculations of the standard one-electron oxidation potential using M1 (*E* ^°M1^) align exceptionally well with experimental data. The oxidation potential in acetonitrile was calculated as 1.2 V, perfectly matching the experimental value.^61^ In water, the predicted value of 1.5 V also corresponds exactly with experimental results.^62^ This accuracy stems from the absence of acidic protons in caffeine, simplifying its oxidation process and highlighting the suitability of M1 for molecules where protonation–deprotonation dynamics play a negligible role. The consistent results emphasize M1’s effectiveness in capturing the oxidation chemistry of caffeine, as illustrated in Figure 2, or the left (neutral) reaction path of Figure 4: Caf → Caf^+•^ + e^–^.

However, in an aqueous solution, M1 fails to work for other purine derivatives — adenine and guanine — which have acidic protons, unlike caffeine. Thus, for most of the molecules calculated in the benchmark, there is a complete lack of correlation with experimental data, as shown in Table 3. The *R* ^2^ value is close to zero, and the intercept is markedly positive, indicating the absence of a clear correlation between calculated *E*_ox_^°^ value and experimental *E* (in fact, *E*_h_ value). The calculated oxidation potential of the xanthine (X) molecule shifts when switching from M1 to M2, due to its p*K*_a(0)r_ value of 7.5, which closely aligns with the pH value of 7. This also applies to hypoxanthine at pH 9. A drawback of this approach is that M2 requires highly accurate p*K*_a_ values, as even small changes in p*K*_a_ can lead to significant changes in molar fractions.

**Table 3:** Experimental *E*_ox_ and Calculated (*E* ^°M1^ and *E* ^°M2^*) Values of the Oxidation Potential in Aqueous Solution (in V, vs. SHE)^a^

	SMD/DFT		SMD/CBS			
compound		+H_2_O		+H_2_O	experiment	pH
A	1.54	1.51	1.83	1.79	1.32	7.0
T	1.91	1.81	2.15	2.05	1.29	7.0
G	1.25	1.19	1.48	1.45	1.04	7.0
C	1.75	1.72	1.91	1.89	1.44	7.0
U	2.25	2.11	2.44	2.32	1.34	7.0
1mG	1.20	1.18	1.43	1.44	1.06	7.0
**X** ^b^	**1.58**	**1.48**	**1.75**	**1.66**	**0.93**	7.0
**hX** ^b^	**1.82**	**1.74**	**2.04**	**1.98**	**1.16**	7.0
	**1.37**	**1.35**	**1.54**	**1.52**	**1.05**	9.0
1mInd	0.91	0.97	1.19	1.21	1.23	7.0
Ind	1.03	0.99	1.33	1.30	1.24	7.0
PhOH	1.57	1.32	1.84	1.64	1.35	7.0
tAn	1.28	1.34	1.56	1.58	1.45	7.0
Caf	1.54	1.59	1.79	1.81	1.50	7.0
X	1.83	1.69	2.00	1.89		
d_X	0.81	0.78	0.94	0.92		
hX	1.83	1.75	2.05	1.99		
d_hX	1.00	1.04	1.14	1.14		
***R*** ^**2**^	0.02	0.04	0.02	0.05		
**slope**	0.06	0.10	0.08	0.11		
**intercept**	1.17	1.11	1.12	1.07		
**MSE**	0.27	0.22	0.51	0.48		
**MUE**	0.38	0.32	0.52	0.48		
**RMSE**	0.47	0.41	0.63	0.58		

a SMD/DFT = SMD­(H_2_O)/(U)­B3LYP-D3/6–31+G­(d,p); SMD/CBS = SMD­(H_2_O)/DLPNO–CCSD­(T)/CBS//SMD­(H_2_O)/(U)­B3LYP-D3/6–31+G­(d,p); “+H_2_O” = one explicit water molecule was added.

b **M2: Henderson–Hasselbalch averaged values using experimental p** *K*_**a**_ **values**; *x*(X) = 0.76, *x*(d_X) = 0.24 at pH 7; *x*(hX) = 0.99, *x*(d_hX) = 0.01 at pH 7; *x*(hX) = 0.44, *x*(d_hX) = 0.56 at pH 9; *x*(neutral)=1, *x*(deprotonated) = 0 for the other molecules at pH 7.

One might question whether it is appropriate to compare the calculated standard oxidation potential (*E*_ox_^°^) with the half-cell potential (*E*_h_) or simply *E*_ox_, as measured in cyclic voltammetry, given that these are distinct quantities. However, *E*_ox_^°^ constitutes the primary contribution to *E*_ox_ (eq 5), making it a fundamental reference value. If solvation effects are reasonably well described (even if with a systematic error) and deprotonation does not play a significant role (acetonitrile or another aprotic solvent), then the reaction mechanism follows the pathway shown in Figure 2, and a direct comparison between these values is justified. In this case, the activities dependent term in the Nernst equation has a negligible effect on *E*_ox_, and some degree of correlation should be observed. If no correlation emerges, this suggests that these factors do, in fact, influence the system, reinforcing the need to explicitly apply the Nernst equation. Ultimately, the first step is to compute *E*_ox_^°^, as it serves as the foundation for further refinement. Once *E*_ox_^°^ is established, a more precise calculation of *E*_ox_ using the Nernst equation can be performed.

Table 4 shows that M3 improves the correlation between experimental and theoretical results, reducing scatter and significantly increasing the *R* ^2^ value compared to M1. However, at the same level of theory, M4 consistently outperforms all other models considered in this work by *R* ^2^ value. The transition from M1 to M4 introduces a pH/*K*_a_ dependent term that enhances the linear correlation (*R* ^2^) between experimental and calculated values, however, this term does not significantly correct the absolute values, which remain sensitive to the accuracy of solvation energy descriptions. The comparison between M3 and M4 highlights the necessity of applying the Nernst equation to both oxidation pathways, RH ⇌ RH^+•^ and R^–^ ⇌ R^•^ (Figure 4), as evidenced by a significant reduction in the intercept. Notably, within the same model (M3 or M4), the addition of an explicit water molecule consistently improves the *R* ^2^ value for both SMD/DFT and SMD/CBS methods while also decreasing the intercept, indicating a reduction in systematic error associated with solvation effects.

**Table 4:** Experimental *E*_ox_ and Calculated (*E*_pH7_^M3^ and *E*_pH7_^M4^) Values of the Oxidation Potential in Aqueous Solution (in V, vs. SHE)^a^

	SMD/DFT		SMD/DFT + H_2_O		SMD/CBS		SMD/CBS + H_2_O		
compound	M3	M4	M3	M4	M3	M4	M3	M4	experiment
A	1.20	1.62	1.25	1.62	1.40	1.82	1.44	1.83	1.32
T	1.19	1.53	1.23	1.50	1.37	1.79	1.39	1.75	1.29
G	0.94	1.35	0.95	1.31	1.13	1.55	1.15	1.51	1.04
C	1.43	2.02	1.46	1.98	1.57	2.19	1.60	2.17	1.44
U	1.42	1.71	1.45	1.69	1.57	1.96	1.57	1.91	1.34
1mG	0.90	1.34	0.94	1.35	1.11	1.55	1.13	1.55	1.06
X	1.00	1.18	0.95	1.11	1.16	1.38	1.11	1.31	0.93
hX	1.30	1.61	1.29	1.53	1.45	1.76	1.44	1.74	1.16
1mInd^b^									1.23
Ind	0.94	1.84	0.93	1.65	0.97	1.89	0.99	1.78	1.24
***R*** ^**2**^	0.58	0.81	0.67	0.88	0.59	0.85	0.68	0.93	
**slope**	0.59	0.56	0.60	0.60	0.65	0.59	0.67	0.60	
**intercept**	0.52	0.31	0.50	0.30	0.34	0.15	0.31	0.14	
**MSE**	–0.05	0.38	–0.04	0.32	0.12	0.58	0.14	0.56	
**MUE**	0.12	0.38	0.10	0.32	0.14	0.58	0.16	0.56	
**RMSE**	0.14	0.40	0.13	0.34	0.17	0.59	0.18	0.57	

a SMD/DFT = SMD­(H_2_O)/(U)­B3LYP-D3/6–31+G­(d,p); SMD/CBS = SMD­(H_2_O)/DLPNO–CCSD­(T)/CBS//SMD­(H_2_O)/(U)­B3LYP-D3/6–31+G­(d,p); “+H_2_O” = one explicit water molecule was added.

b M3 and M4 are not applicable due to the absence of the corresponding acidic protons.

M3 and M4 depend on the following key variables: (1) the calculated value of *G*_sol_ for the ideal electron removal in water, which is independent of acid–base equilibria and p*K*_a_ values and can be determined using M1 and the SMD model; (2) experimental or calculated p*K*_a_ values. Notably, using theoretically calculated p*K*_a_ values instead of experimental ones significantly improves the correlation. We assume that when theoretically calculated p*K*_a_ values are used, the disadvantages (systematic errors) of the SMD model in describing the free energy of solvation are mitigated through the application of M4.

Tables 3 and 4 show that the calculated oxidation potentials obtained using models such as M4 exhibit significant deviations from experimental data. This is particularly evident in the slope values (e.g., ∼0.50), which indicate a systematic overestimation of calculated values compared to experimental ones. We believe this discrepancy primarily arises from the Δ*G* term used in calculating the standard oxidation potential (*E*_ox_^°^) in Equation M1. Specifically, Table 2 demonstrates that M1 shows excellent correlation with experimental data for gas-phase calculations and acetonitrile solutions. However, when transitioning to aqueous solutions in Table 3, this correlation diminishes, and the errors increase substantially.

To improve agreement with experimental data, it is essential to enhance the accuracy of solvation energy Δ*G* ^solv^ descriptions in the eq 3. This would enable closer alignment not only in relative but also absolute oxidation potential values. Our computational model, which combines the SMD implicit solvation method with a single explicit water molecule, does not fully capture solvation effects, particularly for radical cations and anions. These highly polarized species form complex hydrogen-bond networks with surrounding water molecules, providing greater stabilization than our approach accounts for. Improving accuracy will require more detailed solvation modeling. Similar challenges have been addressed by refining implicit solvent models via cavitation parameter adjustments^35^ or incorporating more explicit water molecules^67^ to enhance p*K*_a_ and oxidation potential predictions. Implementing such refinements could enhance predictive accuracy and will be considered in future work.

### Chemical Abbreviations

In the next section, we will use chemical abbreviations for clarity. Below is a list of these abbreviations, where the chemical name is followed by its corresponding abbreviation.

**Cytosines (Cytosine Derivatives)**

5-Methylcytosine (5mC)

5-Hydroxymethylcytosine (5hmC)

5-Dihydroxymethylcytosine (5dhmC)

5-Formylcytosine (5fC)

5-Carboxylcytosine (5caC)

**1-Methylated Cytosines**

1,5-Dimethylcytosine (1m5mC)

1-Methyl-5-Hydroxymethylcytosine (1m5hmC)

1-Methyl-5-Dihydroxymethylcytosine (1m5dhmC)

1-Methyl-5-Formylcytosine (1m5fC)

1-Methyl-5-Carboxylcytosine (1m5caC)

**Uracils (Deaminated Cytosine Derivatives)**

5-Methyluracil (5mU, Thymine, T)

5-Hydroxymethyluracil (5hmU)

5-Dihydroxymethyluracil (5dhmU)

5-Formyluracil (5fU)

5-Carboxyluracil (5caU)

**1-Methylated Uracils**

1,5-Dimethyluracil (1m5mU)

1-Methyl-5-Hydroxymethyluracil (1m5hmU)

1-Methyl-5-Dihydroxymethyluracil (1m5dhmU)

1-Methyl-5-Formyluracil (1m5fU)

1-Methyl-5-Carboxyluracil (1m5caU)

## Computational Prediction

Table 5 presents the calculated *E* ^°M1^ and *E*_pH_^M4^ values (in volts) for cytosine derivatives in aqueous solution. These values are shown together because *E*_pH_^M4^ is the sum of *E* ^°M1^ and the pH/p*K*_a_-dependent term (eq 14). The results obtained from M4 differ significantly from those of M1, as seen in Table 5. For example, in cytosine derivatives, all M4 values are notably higher and closer to each other, altering some of the trends observed in M1. Notably, experimental data indicate that, unlike in the gas phase, the oxidation potential of cytosine in aqueous solution is higher than that of uracil. However, M1 provides a completely opposite result for both SMD/DFT and SMD/CBS methods. M4 shows more logical result, with the absolute *E*_pH_^M4^ value of cytosine in aqueous solution indeed being greater than that of uracil. However, the calculated values are systematically higher than the experimental ones.

**Table 5:** Calculated **Absolute** Values (in V, vs. SHE) of the Oxidation Potential in Aqueous Solution in V Using M1 and M4 (pH 7)^a^,^b^

	*E* ^°M1^				*E*_pH_^M4^				
compound	SMD/DFT	SMD/DFT + H_2_O	SMD/CBS	SMD/CBS + H_2_O	SMD/DFT	SMD/DFT + H_2_O	SMD/CBS	SMD/CBS + H_2_O	experiment
C	1.75	1.73	1.91	1.90	2.03	2.00	2.20	2.18	**1.44** ^46^ ^b^
5mC	1.50	1.50	1.68	1.71	1.85	1.82	2.05	2.07	
5hmC	1.66	1.63	1.84	1.87	1.90	1.83	2.15	2.14	
5fC	2.15	2.11	2.22	2.24	1.98	1.97	2.18	2.21	
5dhmC	1.79	1.76	1.99	2.00	1.96	1.95	2.25	2.19	
5caC	2.29	2.24	2.62	2.59	1.66	1.62	1.91	2.06	
d_5caC	1.66	1.62	1.90	2.06	1.03	1.00	1.19	1.52	
1mC	1.80	1.84	2.05	2.09	2.64	2.52	2.82	2.74	
1m5mC	1.53	1.60	1.77	1.84	2.49	2.50	2.64	2.70	
1m5hmC	1.67	1.68	1.91	1.92	2.49	2.25	2.80	2.71	
1m5fC	2.26	2.24	2.43	2.44	2.75	2.60	2.91	2.83	
1m5dhmC	1.82	1.79	2.11	2.10	2.12	2.00	2.79	2.60	
1m5caC	2.22	2.15	2.44	2.56	2.64	2.52	2.82	2.74	
d_1m5caC	1.66	1.74	2.25	2.23	1.09	1.34	2.09	1.91	
U	2.25	2.12	2.44	2.36	1.72	1.68	1.97	1.94	**1.34** ^46^ ^b^
T (5mU)	1.92	1.81	2.15	2.11	1.55	1.46	1.82	1.54	
5hmU	2.08	1.96	2.47	2.31	1.62	1.52	1.91	1.80	
5fU	2.47	2.42	2.76	3.09	1.67	1.77	1.92	1.93	
5dhmU	2.25	2.15	2.55	2.50	1.63	2.01	1.95	2.32	
5caU	2.75	2.57	3.03	2.89	1.75	1.76	1.95	1.99	
d_5caU	1.73	1.73	1.77	1.77	0.73	0.91	0.69	0.86	
1mU	2.09	2.00	2.29	2.25	2.21	2.12	2.48	2.45	
1mT (1m5mU)	1.73	1.74	1.98	2.03	1.76	1.89	2.26	2.22	
1m5hmU	1.97	1.94	2.27	2.26	2.03	1.94	2.30	2.19	
1m5fU	2.39	2.33	2.53	2.53	2.27	2.13	2.59	2.51	
1m5dhmU	2.10	2.07	2.38	2.46	1.89	1.94	2.31	2.27	
1m5caU	2.55	2.48	2.69	2.69	1.86	1.81	2.47	2.50	
d_1m5caU	1.67	1.65	2.10	2.13	0.98	0.99	1.88	1.94	

a “SMD/DFT” – SMD­(H_2_O)/(U)­B3LYP-D3/6–31+G­(d,p); “SMD/CBS” – SMD­(H_2_O)/DLPNO–CCSD­(T)/CBS//SMD­(H_2_O)/(U)­B3LYP-D3/6–31+G­(d,p); “+H_2_O“ – one explicit water molecule added.

b Estimated value obtained by extrapolation, assuming a linear increase of 60 mV per pH unit and the p*K*_a_ value of the base.

Considering the *E* ^°M1^ values, there is a clear dependence on the electron-donating or withdrawing nature of the substituent at the fifth position. The electron donation or withdrawal properties of substituent groups are well described by Hammett constants σ_p_, which quantify the electronic effects of substituents relative to hydrogen on a reference compound, typically benzene. These dimensionless constants, derived from reaction rate or equilibrium measurements, provide a measure of electron-donating (negative values) or electron-withdrawing (positive values) tendencies. In this context, we referenced literature values: (m-) −0.14, (hm-) −0.04 ± 0.03, (RO2C−) 0.44, (f-) + 0.57 ± 0.07.^121^ Here, m- corresponds to a generic methyl group, hm- to a hydroxymethyl group, RO2C- to a carboxy-like group, and f- to a formyl group. While these constants are not specific to modified cytosine, they offer a useful comparison for similar substituents, such as the 5-methyl group in cytosine.

For example, according to M1, the 5-methyl group facilitates oxidation due to its electron-donating effect. Moving on to the 5-hydroxymethyl (5hm) group, there is a small electron-withdrawing effect from the hydroxy group on the 5-methyl group, thereby increasing the oxidation potential of 5hmC, bringing it closer to cytosine (C) on the lower side. In principle, the 5dhm group can be viewed as a modification of 5hm to which another electron-withdrawing hydroxy group has been added, thereby gradually increasing the oxidation potential, making it slightly higher than that of C. The 5-formyl group, together with the 5-carboxy group, are the most electron-withdrawing and therefore significantly increase the calculated values of *E* ^°M1^. All the trends described in M1 are observed not only in derivatives of C but also in 1-methylcytosine (1mC), as well as in derivatives of uracil (U) and 1-methyluracil (1mU).

When going from C derivatives to 1mC derivatives, a general increase in the calculated *E*_pH7_^M4^ values of the oxidation potential is observed. This is due to the fact that, in comparison with cytosine in 1mC, one acidic proton is replaced by a 1m group, which is why the calculated absolute p*K*_a(0)r_ values significantly increase at **1mC**, **1m5mC**, **1m5hmC**, **1m5fC**, and slightly increase for 1m5dhmC and **1m5caC**.

In order to improve the description of solvation effects, we have added one explicit water molecule to the optimized geometries. It should be noted that the results both without and with a water molecule are very close. Although, based on our benchmark, we believe that the water molecule results are slightly more accurate.

In Table 5, we observe that the absolute values are consistently higher than the experimental ones, as also indicated by our benchmark analysis for M4 in Table 4.

Since the linear regression slopes for DFT and CBS in water are similar (0.56–0.60 with one explicit water molecule) and also close in acetonitrile (0.95–1.02), the observed difference is primarily due to the solvent model. Therefore, a logical step in Table 6 would be to scale the M4 model values from Table 5 by the corresponding slope coefficient. For example, the calculated value for cytosine in Table 5 is 2.18 V (M4 calculated using SMD/CBS with one explicit water molecule), while the experimental value is 1.44 V. To address this discrepancy, we apply the **slope** of 0.60 from Table 4 as a multiplication factor, yielding 2.18 V × 0.6 = 1.31 V. The resulting value of 1.31 V is 0.13 V lower than the experimental value of 1.44 V, so we shift the scaled values for cytosine by adding an **intercept** of +0.13 V (2.18 V × 0.6 + 0.13 V = 1.44 V). This **linear adjustment** strategy can be applied to a series of cytosine modifications within the same level of theory, as they share a similar chemical structure to regular cytosine. However, for uracil modifications, we will use the same multiplication factor, but with a different intercept (for instance, regular uracil at the same level of theory: 1.94 V × 0.6 + 0.18 V = 1.34 V).

**Table 6:** **Linearly Adjusted** (M4, pH 7) Calculated Values of the Oxidation Potential in Aqueous Solution (in V, vs. SHE)^a^,^b^

	*E*_pH_^M4^			
compound	SMD/DFT	SMD/DFT + H_2_O	SMD/CBS	SMD/CBS + H_2_O
C	**1.44** ^46^ ^b^	**1.44** ^46^ ^b^	**1.44** ^46^ ^b^	**1.44** ^46^ ^b^
5mC	1.34	1.34	1.35	1.37
5hmC	1.36	1.34	1.41	1.42
5fC	1.41	1.43	1.43	1.46
5dhmC	1.40	1.41	1.47	1.45
5caC	1.23	1.21	1.27	1.37
d_5caC	0.88	0.84	0.84	1.04
1mC	1.78	1.75	1.81	1.77
1m5mC	1.70	1.74	1.70	1.75
1m5hmC	1.70	1.59	1.80	1.76
1m5fC	1.84	1.80	1.86	1.83
1m5dhmC	1.49	1.44	1.79	1.69
1m5caC	1.23	1.29	1.49	1.47
d_1m5caC	0.91	1.05	1.37	1.28
U	**1.34** ^46^ ^b^	**1.34** ^46^ ^b^	**1.34** ^46^ ^b^	**1.34** ^46^ ^b^
T (5mU)	1.25	1.21	1.25	1.10
5hmU	1.29	1.24	1.31	1.26
5fU	1.32	1.40	1.31	1.33
5dhmU	1.29	1.54	1.33	1.57
5caU	1.36	1.39	1.33	1.37
d_5caU	0.78	0.88	0.59	0.69
1mU	1.62	1.60	1.64	1.65
1mT (1m5mU)	1.37	1.46	1.51	1.51
1m5hmU	1.51	1.50	1.54	1.49
1m5fU	1.65	1.61	1.71	1.68
1m5dhmU	1.44	1.50	1.54	1.53
1m5caU	1.42	1.42	1.63	1.68
d_1m5caU	0.93	0.93	1.29	1.34

a “SMD/DFT” – SMD­(H_2_O)/(U)­B3LYP-D3/6–31+G­(d,p); “SMD/CBS” – SMD­(H_2_O)/DLPNO–CCSD­(T)/CBS//SMD­(H_2_O)/(U)­B3LYP-D3/6–31+G­(d,p); “+H_2_O“ – one explicit water molecule added.

b Estimated value obtained by extrapolation, assuming a linear increase of 60 mV per pH unit and the p*K*_a_ value of the base.

Unfortunately, it is not possible to apply any linear adjustment strategy to the *E* ^°M1^ values in Table 5, as Table 3 clearly shows a complete lack of linear correlation between the theoretical M1 values and the experimental values in the aqueous solution. However, if desired, according to eq 14, the pH/*K*_a_ dependent term (the difference between *E*_pH_^M4^ and *E* ^°M1^ in Table 5) can be multiplied by the scaling factor (slope) used in Table 6 for the linear adjustment and then subtracted from the *E*_pH_^M4^ values. This allows for an estimation of *E*°. However, experimentally verifying the *E*° value remains challenging.

Among the studied cytosine-derived molecules, it is interesting to consider the most acidic 5caC molecule, which has an acidic carboxy group and can partially be presented as a zwitterion in aqueous solution. Considering the experimental p*K*_a_ values reported in the literature, it can be expected that for 5caC, the value of *x*_R^–^_ approaches 1 at pH 7. For example, the 5caC-nucleoside has experimental p*K*_a_ values for the COOH group at 4.5 and 4.2,^122^ while its N3-protonated form, p_5caC-nucleoside (“p_” protonated), has p*K*_a_ values of 2.4 and 2.1 for the N3–H group.^122^ The 5caC-deoxynucleoside exhibits p*K*_a_ values of 4.28 ^123^ and 4.0 ^124^ for the COOH group, and its N3-protonated form, p_5caC-deoxynucleoside, shows p*K*_a_ values of 2.45 ^123^ and 2.0 ^124^ for the N3–H group. Similarly, 5caU molecule exhibit comparable p*K*_a_ values for its COOH group, indicating its acidic nature. For instance, the 5caU-nucleobase has reported p*K*_a_ values of 425,^125^ and 4.16 ^126^ for the COOH group. Additionally, the 5caU-deoxynucleoside has an experimental p*K*_a_ value of 4.0 ^125^ for the COOH group.

Therefore, using M4 for 5caC, 1m5caC, 5caU, and 1m5caU, it is more appropriate to utilize *E* ^°M1^(R^•^ | R^–^) rather than *E* ^°M1^(RH^+•^ | RH). For the neutral molecule 5caC, the oxidation potential *E* ^°M1^(RH^+•^ | RH) is 2.59 V (calculated using SMD/CBS with one explicit water molecule), while for the deprotonated molecule d_5caC, the oxidation potential *E* ^°M1^ (R^•^ | R^–^) is 2.06 V, which is −0.53 V lower than that of the neutral molecule. (Table 5) The correction for the prototropic equilibrium from M4 remains unchanged, yielding an **absolute** value of *E*_pH7_^M4^(R^•^ | R^–^) = 1.52 V (Table 5), or a **linearly adjusted** value of *E*_pH7_^M4^(R^•^ | R^–^) = 1.04 (Table 6, corrected using linear regression relative to cytosine 1.44 V as the reference). For d_1m5caC, *E* ^°M1^(R^•^ | R^–^) = 2.23 V, which gives *E*_pH7_^M4^(R^•^ | R^–^) = 1.91 V (absolute) and 1.28 V (linearly adjusted). Similarly, using the experimental value for uracil at 1.34 V, one can obtain a linearly adjusted *E*_pH7_^M4^(R^•^ | R^–^) = 0.69 V for d_5caU, and *E*_pH7_^M4^(R^•^ | R^–^) = 1.34 V for d_1m5caU.

Thus, we identified clear trends in the oxidation properties of various cytosine and uracil derivatives (Table 6, Figure 6). According to the form of the Nernst equation, the M4 model at the SMD­(H_2_O)/DLPNO–CCSD­(T)/CBS//SMD­(H_2_O)/(U)­B3LYP-D3/6–31+G­(d,p) level of theory with one explicit water molecule yields the following oxidation potential trend for modified cytosines: d_5caC < 5mC < 5caC < 5hmC < C < 5dhmC < 5fC. This trend reflects the gradual increase in the one-electron oxidation potential, which is simultaneously influenced by the electron-donating/withdrawing properties of the 5-position substituents and the effects of deprotonation at pH 7. The introduction of formyl (5fC) group increases the oxidation potential, whereas the deprotonation (“d_”) of the 5-carboxy group lowers it, which is consistent with the electrochemical nature of these compounds.

**Figure 6:**
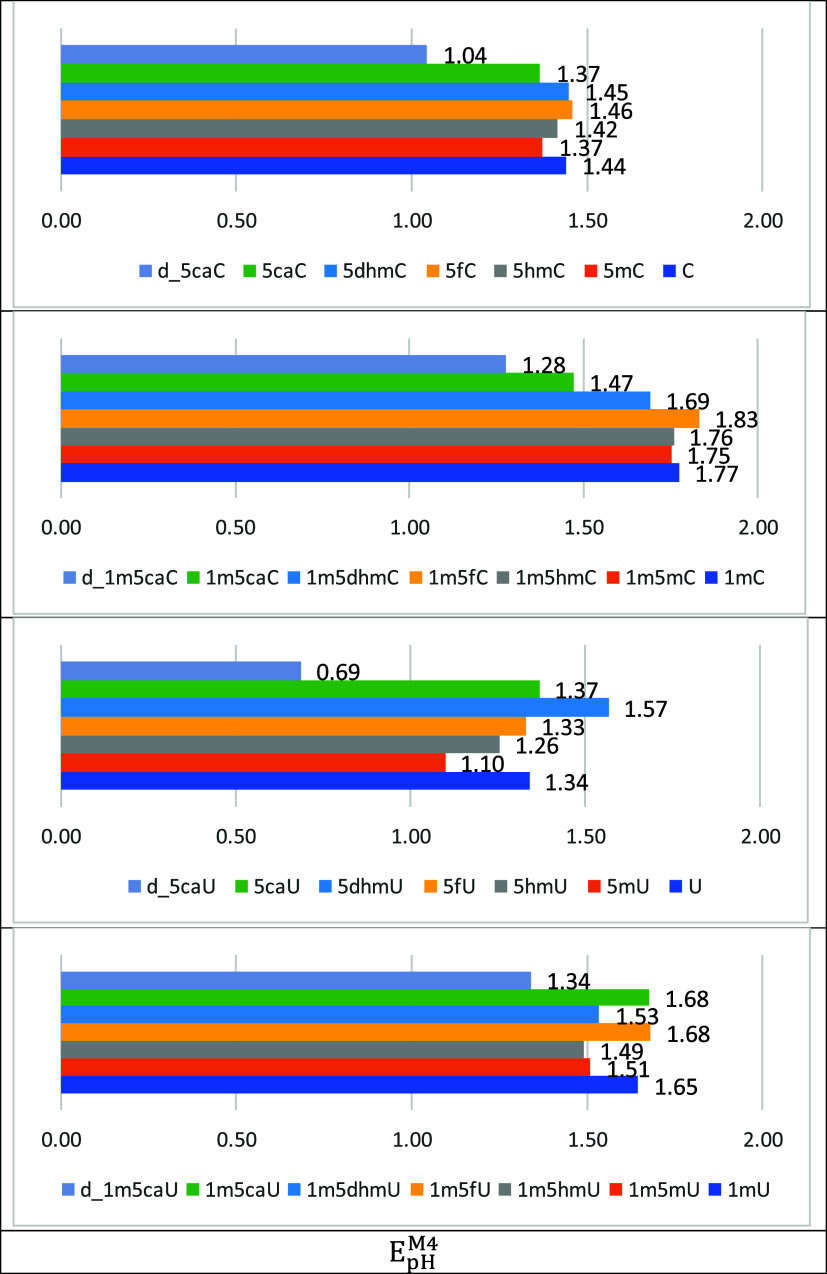
Calculated **linearly adjusted** relative to cytosine (1.44 V^46^) and uracil (1.34 V^46^) *E*_*pH*_^*M4*^ values in aqueous solution (in V vs SHE) using M4 at the SMD­(H_2_O)/DLPNO–CCSD­(T)/CBS//SMD­(H_2_O)/(U)­B3LYP-D3/6–31+G­(d,p) level of theory with one explicit water molecule.

1-Methylated cytosines are likely of greater interest to biochemistry, as they include the effect of the carbon–nitrogen bond at position N1, mimicking the influence of the deoxyribose moiety attached to nucleic acids. The oxidation potential trend for 1-methylated cytosines is as follows: d_1m5caC < 1m5caC < 1m5dhmC < 1m5mC < 1m5hmC < 1mC < 1m5fC. It is evident that the 1-methyl group affects oxidation not only through its weak electron-donating effect but primarily by replacing one of the acidic protons at N1. This substitution reduces the tendency for deprotonation, thereby increasing the oxidation potentials of these cytosines. We observe a systematic increase in oxidation potentials, particularly with the introduction of the strongly electron-withdrawing formyl (5fC) group.

For uracils (deaminated cytosine derivatives), the following oxidation potential trend is observed: d_5caU < T (5mU) < 5hmU < 5fU < U < 5caU < 5dhmU. This order reflects the influence of 5-position substituents on the oxidation properties of uracils. Methylation (as in thymine, T (5mU)) increases electron donation, thereby facilitating oxidation, whereas the introduction of hydroxymethyl (5hmU) and formyl (5fU) groups increases the oxidation potential. Carboxylation (5caU) and particularly its deprotonated form (d_5caU) lower the oxidation potential.

For 1-methylated uracils, a similar trend is observed: d_1m5caU < 1m5hmU < 1mT (1m5mU) < 1m5dhmU < 1mU < 1m5caU < 1m5fU. 1-Methylation at N1 has a similar effect to that observed in cytosines, systematically increasing oxidation potentials by reducing acidity and suppressing deprotonation. Specifically, the introduction of electron-withdrawing groups, such as formyl (1m5fU) and carboxyl (1m5caU), further increases oxidation potentials.

## Conclusions

This study demonstrates that the accuracy of computational models M1–M4 depends on the environment surrounding nucleobases. Model M1 performs well in the gas phase and apolar solvents but becomes less reliable in aqueous solutions. Model M2 is more suitable for highly deprotonated molecules in aqueous environments; however, it requires precise p*K*_a_ values for accurate predictions. Model M3 accounts for prototropic equilibrium, but its effectiveness is limited when the contribution of the deprotonated form is minimal, particularly when p*K*_a_ significantly deviates from physiological pH. Model M4 emerges as the most accurate, as it considers both pH-dependent effects and the mutual influence of protonated and deprotonated forms. This comprehensive approach improves the prediction of oxidation potentials in aqueous solutions, leading to better agreement with experimental data.

The inclusion of explicit water molecules in geometry optimization enhances the description of solvation effects for charged species, such as radical cations and deprotonated forms, and reduces systematic errors associated with solvation energy considerations. However, the overall improvement in correlation remains moderate, suggesting that further refinement of the solvation model is necessary.

Our analysis of chemical modifications on oxidation potential reveals distinct trends. Electron-withdrawing groups (e.g., formyl (5fC) and carboxyl (5caC)) increase oxidation potential, whereas the methyl group (5mC) and deprotonation lower it. The introduction of a 1-methyl group further alters redox properties by reducing nucleobase acidity, making them more comparable to their natural nucleoside forms. The resulting oxidation potential is a combined outcome of these effects and often follows nontrivial dependencies, making its prediction particularly challenging.

Given the complexity of experimental validation, which requires advanced electrochemical setups and specialized reagents, this study focused on computational predictions. While experimental confirmation was beyond the scope of this work, we hope that future studies equipped with the necessary resources will verify our findings and further refine the computational approaches applied.

In summary, this study provides valuable insights into the redox behavior of epigenetic cytosine and uracil derivatives, emphasizing the need for accurate computational models that account for both protonation states and solvation effects. Reliable redox potential predictions are crucial not only for understanding oxidative DNA damage mechanisms but also for broader applications in computational chemistry, particularly in modeling redox behavior across different solvent environments.

## Supplementary Material



## Notes

3D structure images of nucleic acids in various states (neutral, radical cation, deprotonated, and deprotonated radical cation), representing the most stable tautomers and conformers optimized with DFT. Table S1 provides energy and free energy values for oxidation potential calculations, solvation energies, and total energies from SMD/DFT and SMD/CBS methods. Compressed Gaussian output xyz-coordinates (PDF)
