# Supplementary material for: Computational Prediction of One-Electron Oxidation Potentials for Cytosine and Uracil Epigenetic Derivatives
Source: J Phys Chem A. 2025 Apr 8;129(20):4339–56. doi: 10.1021/acs.jpca.4c06944 (PMC12105625; doi:10.1021/acs.jpca.4c06944)
Supplement: Supplementary file 1 [file jp4c06944_si_001.pdf]

Supporting Information

Computational Prediction of One-Electron Oxidation Potentials for Cytosine and Uracil Epigenetic Derivatives

Vasilii Korotenko,[* 1,3] *Patrick Langrzyk,*[2,3] *Hendrik Zipse*[3]

[* 1] Thermal Separation Processes, TUHH, Denickestraße 22, 21073 Hamburg, Germany (E-mail: vasilii.korotenko@tuhh.de)

[2] Laboratory of Asymmetric Catalysis and Synthesis (LACS), EPFL, CH-1015 Lausanne, Switzerland

[3] Department of Chemistry, LMU München, Butenandtstrasse 5-13, 81377 München, Germany

Contents

1 Derivation of formulas M2, M3, M4 2

2 Figures with structures 7

3 Energies 15

4 XYZ data 87

5 XYZ data (1 explicit H_2O) 529

1 Derivation of formulas M2, M3, M4

Model 2 (M2). Each protic compound has acid/base properties in aqueous solution, characterized by their corresponding pK_a values, indicating the potential for deprotonation of the studied molecule. As a rule, it is easier to oxidize a negatively charged molecule (the deprotonation product, which is electron-rich) than a neutral one, as this process requires lower energy compared to the oxidation of the corresponding neutral species.

The free energy diagram on Figure 4 in manuscript helps to visualize the energy changes and intermediates involved in the one-electron oxidation and deprotonation reactions that can happen with nucleic bases in the solution phase. In the case of a dissociating molecule (R-H), there are two possible one-electron oxidation pathways: the free energy of the first pathway includes $\Delta G^{sol}_{IE(0)r}$, while the free energy of the second pathway includes $\Delta G^{sol}_{IE(-1)r}$. The subscript in parentheses indicates the charge of the original molecule that will be oxidized, either zero (neutral) or minus one (deprotonated). The letter 'r' indicates that this molecule is in its original, unoxidized, or reduced state. The values of $\Delta G^{sol}_{a(+1)o}$ and $\Delta G^{sol}_{a(0)r}$ (letter "a" stands for acidity) are free energies of deprotonation and are described by the pK_a values

$$\Delta G^{sol}_{a} = -RT\, lnK_a = 2.303\, RT\, pK_a \quad \text{Equation 1}$$

$$pK_a = -log_{10}K_a = -log_{10}\frac{(Base)(H^+)}{(Acid)} \quad \text{Equation 2}$$

where activities are denoted by parentheses and are unitless (while concentrations are usually represented by square brackets). In the general case, the following holds true: $\Delta G^{sol}_{a(+1)o} < \Delta G^{sol}_{a(0)r}$

Since $\Delta G^{sol}_{IE(-1)r} < \Delta G^{sol}_{IE(0)r}$ is always, and the measured value of $\Delta G^{sol}_{IE,exp}$ can be somewhere between $\Delta G^{sol}_{IE(-1)r}$ and $\Delta G^{sol}_{IE(0)r}$ values ($\Delta G^{sol}_{IE(-1)r} < \Delta G^{sol}_{IE,exp} < \Delta G^{sol}_{IE(0)r}$) and will depend on the concentrations (or activities) ratio $[R^-]$ and [RH]. At the thermodynamic equilibrium of the system, the $[R^-]/[RH]$ ratio at a given pH value can be estimated using the Henderson–Hasselbalch equation

$$pH = pK_a + lg\frac{[R^-]}{[RH]} = pK_a + lg\frac{x_{R^-}}{x_{RH}} \quad \text{Equation 3}$$

where x is the mole fraction of the corresponding neutral acid (RH) and ionized base (R^-). If the pK_a value of the oxidized product is known the Equation 3 can be also used to calculate the mole fractions of the cationic radical acid ($RH^{\bullet+}$) and neutral deprotonated radical base ($R^{\bullet}$).

Equation 3 can be converted into an expression for the extent of ionization depending on pH and pK_a values as follows:

$$x_{RH} = \frac{x_{R^-}}{10^{pH-pK_a}} \quad \text{Equation 4}$$

$x_{R^-} = x_{RH} * 10^{pH-pK_a}$ Equation 5

$x_{RH} + x_{R^-} = 1$ Equation 6

$x_{RH} + x_{RH} * 10^{pH-pK_a} = 1$ Equation 7

$\frac{1}{x_{RH} + x_{RH}*10^{pH-pK_a}} = \frac{1}{1}$ Equation 8

$x_{RH} = \frac{1}{1+10^{pH-pK_a}}$ Equation 9

$x_{R^-} = 1 - x_{RH}$ Equation 10

If we simply assume that the probability of an electron being detached from a particle is equal to its molar fraction value x (regardless of the detachment energy), then we can apply x as a coefficient contributing to the resulting oxidation potential. Thus, we come to the second model (M2), which depends on the pH value

$E^{\circ}(RH^{+\bullet}; R^{\bullet} \mid RH; R^{-}) = E^{\circ M2}_{pH} = \frac{x_{RH} * \Delta G^{sol}_{IE(0)r} + x_{R^-} * \Delta G^{sol}_{IE(-1)r}}{nF} - ref$ Equation 11

This expression uses the Henderson-Hasselbalch equation, all protonation levels (shown in Figure 4 in manuscript) of the reduced and oxidized species are considered. We notice, if $pK_a > pH$, then increasing the value of pK_a leads to $x_{RH} \rightarrow 1$ and $x_{R^-} \rightarrow 0$. The contribution coefficients x (molar fractions) are directly connected to the free energy of the deprotonation reaction. They reflect the relative population of different molecular forms under specific temperature and conditions, as described by the Boltzmann distribution. Thus, the Boltzmann-averaged value $E^{\circ M2}_{pH}$ incorporates the contribution of each molecular form, weighted by its energy state. This approach enables the averaging of E° values, providing a more accurate representation of the standard potential for the mixture of forms.

Model 3 (M3). According to Wardman it is important to consider the coupling of electrons and protons (prototropic equilibrium) in the reaction, which should be included in the Nernst relationship:

$E = E^{\circ}_{ox} + \frac{RT}{nF} \ln \frac{(product\ of\ activities\ of\ oxidant)}{(product\ of\ activities\ of\ reductant)}$ Equation 12

or

$E = E^{\circ}_{ox} - \frac{RT}{nF} \ln \frac{(product\ of\ activities\ of\ reductant)}{(product\ of\ activities\ of\ oxidant)}$ Equation 13

where E_h is the standard potential of the half-cell reaction, including protons.

Now, we will derive an expression for the equilibrium shown in Figure 4 in manuscript. Following Clark, we define the symbol S_r to denote the sum of reductant-activities shown

$$(S_r) = (RH) + (R^-) + (H^+) \quad \text{Equation 14}$$

and define equilibrium constants for the dissociation of the reductant:

$$K_{a(0)r} = \frac{(R^-)(H^+)}{(RH)};\ (R^-) = \frac{K_{a(0)r}(RH)}{(H^+)} \quad \text{Equation 15}$$

Then the sum of reductant-activities can be expressed as follows:

$$(S_r) = (RH) + (R^-) + (H^+) = (RH) + \frac{K_{a(0)r}(RH)}{(H^+)} + (H^+) \quad \text{Equation 16}$$

$$(S_r) = (RH)\left(\frac{1}{1} + \frac{K_{a(0)r}}{(H^+)}\right) + (H^+) = (RH)\left(\frac{(H^+)+K_{a(0)r}}{(H^+)}\right) + (H^+) = (RH)\left(\frac{(H^+)+K_{a(0)r}}{(H^+)}\right) + \frac{(H^+)^2}{(H^+)} = (RH)\left(\frac{(H^+)+K_{a(0)r}+(H^+)^2}{(H^+)}\right)$$
Equation 17

$$(RH) = (S_r)\left(\frac{(H^+)}{(H^+)+K_{a(0)r}+(H^+)^2}\right) \quad \text{Equation 18}$$

Similarly using the symbol S_o to denote the sum of oxidants

$$S_o = RH^{+\bullet} + R^{\bullet} + H^+ \quad \text{Equation 19}$$

we can come to

$$(RH^{+\bullet}) = (S_o)\left(\frac{(H^+)}{(H^+)+K_{a(+1)o}+(H^+)^2}\right) \quad \text{Equation 20}$$

Now we can substitute the derived activities into the Nernst equation. Let's do this for only one (left) oxidation pathway in Figure 4 in manuscript: $RH \rightleftharpoons RH^{+\bullet}$. In this case, we exclude the contribution of the reaction $R^- \rightleftharpoons R^{\bullet}$ in the resulting value of E_h:

$$E_h = E^{\circ} + \frac{RT}{F}\ ln\frac{(RH^{+\bullet})}{(RH)} = E^{\circ} + \frac{RT}{F}\ ln\frac{(S_o)\left(\frac{(H^+)}{(H^+)+K_{a(+1)o}+(H^+)^2}\right)}{(S_r)\left(\frac{(H^+)}{(H^+)+K_{a(0)r}+(H^+)^2}\right)} = E^{\circ} + \frac{RT}{F}\ ln\frac{(S_o)\left((H^+)+K_{a(0)r}+(H^+)^2\right)}{(S_r)\left((H^+)+K_{a(+1)o}+(H^+)^2\right)} = E^{\circ} + \frac{RT}{F}\ ln\frac{(S_o)}{(S_r)} + \frac{RT}{F}\ ln\frac{\left((H^+)+K_{a(0)r}+(H^+)^2\right)}{\left((H^+)+K_{a(+1)o}+(H^+)^2\right)} \quad \text{Equation 21}$$

The values of $(H^+)^2$ at pH 7 are so small that they can be neglected.

When (S_o) = (S_r), E_h can be described as a 'midpoint' potential with symbol E_m:

$$E_m = E_{pH}^{M3} = E^{\circ} + \frac{RT}{F}\ ln\frac{\left(10^{-pH}+K_{a(0)r}+10^{-2pH}\right)}{\left(10^{-pH}+K_{a(+1)o}+10^{-2pH}\right)} \quad \text{Equation 22}$$

Thus, M3 provides a correction for the prototropic equilibrium that is added to the E° value. Following E°, the pH/pK_a dependent term $\frac{RT}{F}\ln\frac{(10^{-pH}+K_{a(0)r})}{(10^{-pH}+K_{a(+1)o})}$ accounts for the main effect of pH and the acidity constants K_a of the oxidized and reduced states on the redox potential of the left oxidation pathway in Figure 4 in manuscript: $RH \rightleftharpoons RH^{+\bullet}$.

For the molecules calculated in this work, the pH/pK_a dependent term < 0 V because in all cases, the radical cation is significantly more acidic than the neutral reduced molecule, with $K_{a(0)r} > K_{a(+1)o}$. For example, with $K_{a(0)r} = 0$ and $K_{a(+1)o} = 1$, the pH/pK_a dependent term = -0.41 V. The more acidic the radical cation, the more negative the value of the term (delta M3) will be.

The specific value of E° to be used for calculations depends on the system. For example, if the value of x_{R^-} at a given pH is low, we can use $E^{\circ M1}(RH^{+\bullet} \mid RH)$. For more acidic molecules, we should expect a greater contribution from the deprotonated form of the molecule and a higher x_{R^-} value. In this case, it is appropriate to use $E^{\circ M2}_{pH} = E^{\circ}(RH^{+\bullet}; R^{\bullet} \mid RH; R^{-})$ or $E^{\circ M1}(R^{\bullet} \mid R^{-})$ when x_{R^-} is close to 1.

Model 4 (M4). As can be seen from Figure 4 in manuscript, both the $RH^{+\bullet}$ cation radical and its deprotonated form $R^{\bullet}$ can act as an oxidizing agent, while the neutral RH molecule and its deprotonated form R^{-} can act as a reducing agent, in this case:

$$K_{a(0)r} = \frac{(R^{-})(H^{+})}{(RH)};\ (R^{-}) = \frac{K_{a(0)r}(RH)}{(H^{+})}$$

$$K_{a(+1)o} = \frac{(R^{\bullet})(H^{+})}{(RH^{+\bullet})};\ (R^{\bullet}) = \frac{K_{a(+1)o}(RH^{+\bullet})}{(H^{+})}$$

and

$$(RH^{+\bullet}) = (S_o)\left(\frac{(H^{+})}{(H^{+}) + K_{a(+1)o}}\right)$$

$$(R^{\bullet}) = (S_o)\left(\frac{K_{a(+1)o}}{(H^{+}) + K_{a(+1)o}}\right)$$

$$(RH) = (S_r)\left(\frac{(H^{+})}{(H^{+}) + K_{a(+1)o}}\right)$$

$$(R^{-}) = (S_r)\left(\frac{K_{a(0)r}}{(H^{+}) + K_{a(0)r}}\right)$$

Now we can substitute the derived activities into the Nernst equation:

$$E_h = E^\circ + \frac{RT}{F}\, ln\frac{(RH^{+\bullet})}{(RH)}\frac{(R^\bullet)(H^+)}{(R^-)(H^+)} = E^\circ + \frac{RT}{F}\, ln\frac{(S_r)\left(\frac{(H^+)}{(H^+)+K_{a(+1)o}}\right)(S_r)\left(\frac{K_{a(+1)o}}{(H^+)+K_{a(+1)o}}\right)}{(S_r)\left(\frac{(H^+)}{(H^+)+K_{a(0)r}}\right)(S_r)\left(\frac{K_{a(0)r}}{(H^+)+K_{a(0)r}}\right)} \quad \text{Equation 23}$$

When $(S_o) = (S_r)$, E_h can be described as a 'midpoint' potential with symbol E_m:

$$E_m = E^\circ + \frac{RT}{F}\, ln\frac{\left((H^+)+K_{a(0)r}\right)}{\left((H^+)+K_{a(+1)o}\right)}\frac{K_{a(+1)o}\left((H^+)+K_{a(0)r}\right)}{K_{a(0)r}\left((H^+)+K_{a(+1)o}\right)} = E^\circ + \frac{RT}{F}\, ln\frac{K_{a(+1)o}}{K_{a(0)r}} + \frac{RT}{F}\, ln\frac{\left((H^+)+K_{a(0)r}\right)^2}{\left((H^+)+K_{a(+1)o}\right)^2} \quad \text{Equation 24}$$

Thus, $E_{pH}^{M4} = E^\circ + \frac{RT}{F}\, ln\frac{K_{a(+1)o}}{K_{a(0)r}} + 2\frac{RT}{F}\, ln\frac{(10^{-pH}+K_{a(0)r})}{(10^{-pH}+K_{a(+1)o})}$ Equation 25

In this formula, following E°, two terms can be seen. The first term $\frac{RT}{F}\, ln\frac{K_{a(+1)o}}{K_{a(0)r}}$ is always greater than 0 V, while the second term $2\frac{RT}{F}\, ln\frac{(10^{-pH}+K_{a(0)r})}{(10^{-pH}+K_{a(+1)o})}$ is always less than 0 V. Unlike M3, in M4 the second term is considered twice because the added reactants $(R^\bullet)$ and (R^-) are described by the same acidity constants as $(RH^{+\bullet})$ and (RH), respectively.

If we decompose the first term into its components and transform:

$$\frac{RT}{F}\, ln\frac{K_{a(+1)o}}{K_{a(0)r}} = \frac{RT}{F}\, ln\frac{(R^\bullet)(H^+)}{(RH^{+\bullet})}\frac{(RH)}{(R^-)(H^+)} = \frac{RT}{F}\, ln\frac{(R^\bullet)(RH)}{(RH^{+\bullet})(R^-)} \quad \text{Equation 26}$$

it becomes obvious that it describes the reverse free energy (with a minus sign) of this endergonic acid-base (neutralization) reaction: $RH^{+\bullet} + R^- \rightleftharpoons R^\bullet + RH$. Thus, this term describes how all the activities in the Nernst equation for E_{pH}^{M4} interact with each other participating in the neutralization reaction.

2 Figures with structures

This set of figures shows the best tautomers/conformers for C, 1mC, U and 1mU, as well as their derivatives in neutral (charge multiplicity “0 1”), radical cationic (“1 2”), deprotonated (“-1 1”) and deprotonated radical cationic (“0 2”) states

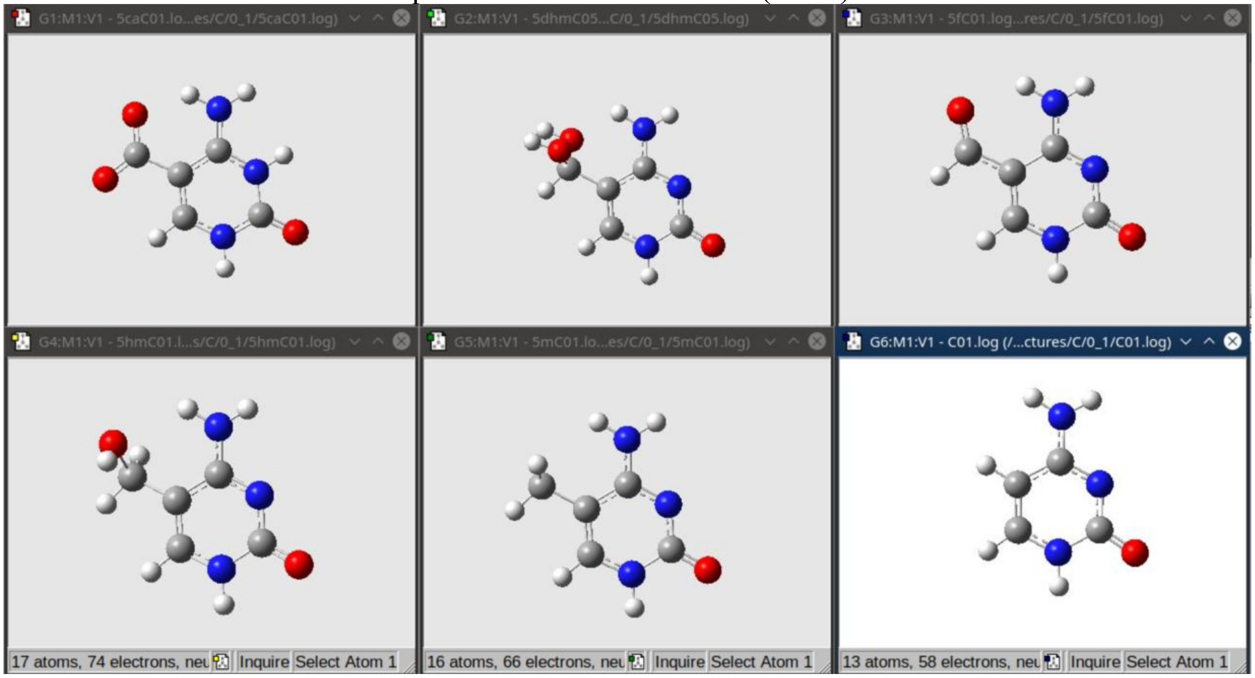

Figure S 1. C derivatives: charge multiplicity “0 1”

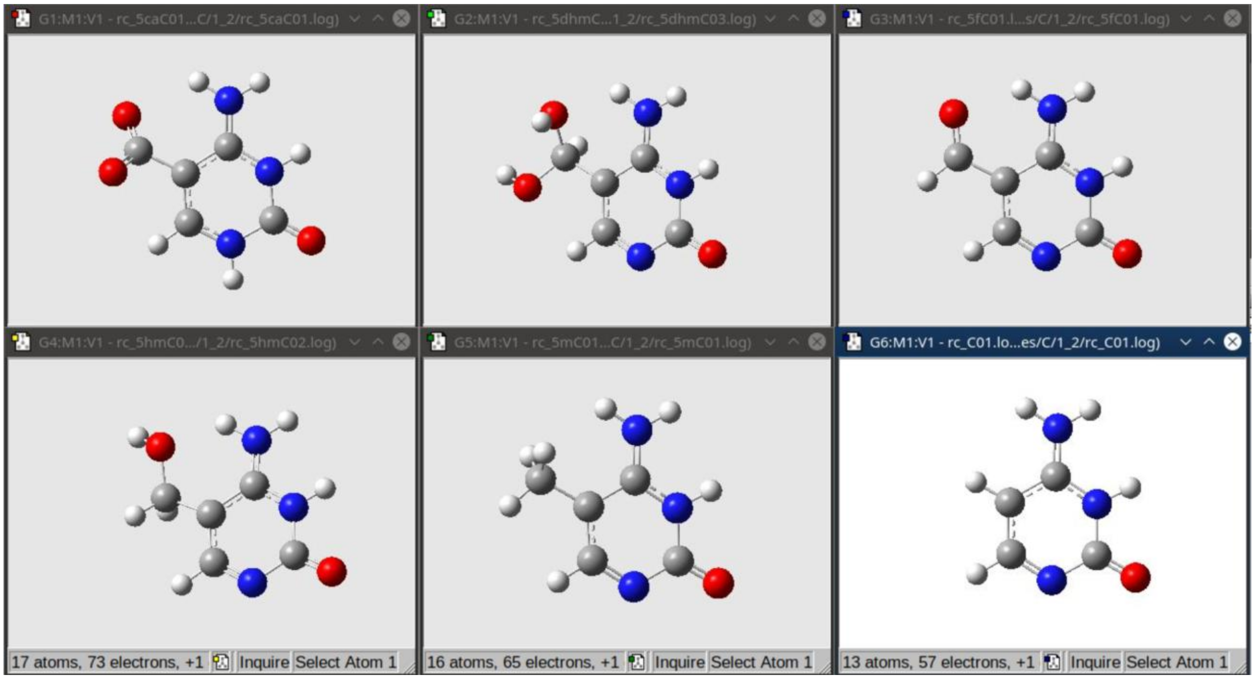

Figure S 2. rc_C derivatives: charge multiplicity “1 2”

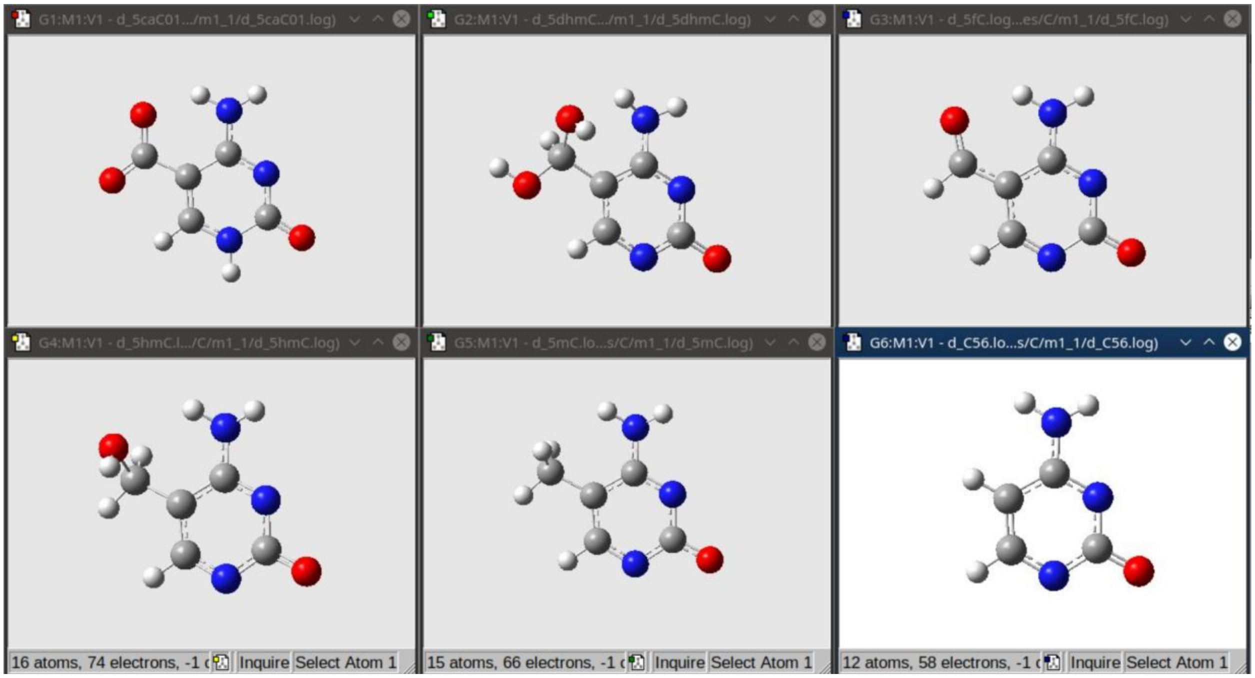

Figure S 3. d_C derivatives: charge multiplicity “-1 1”

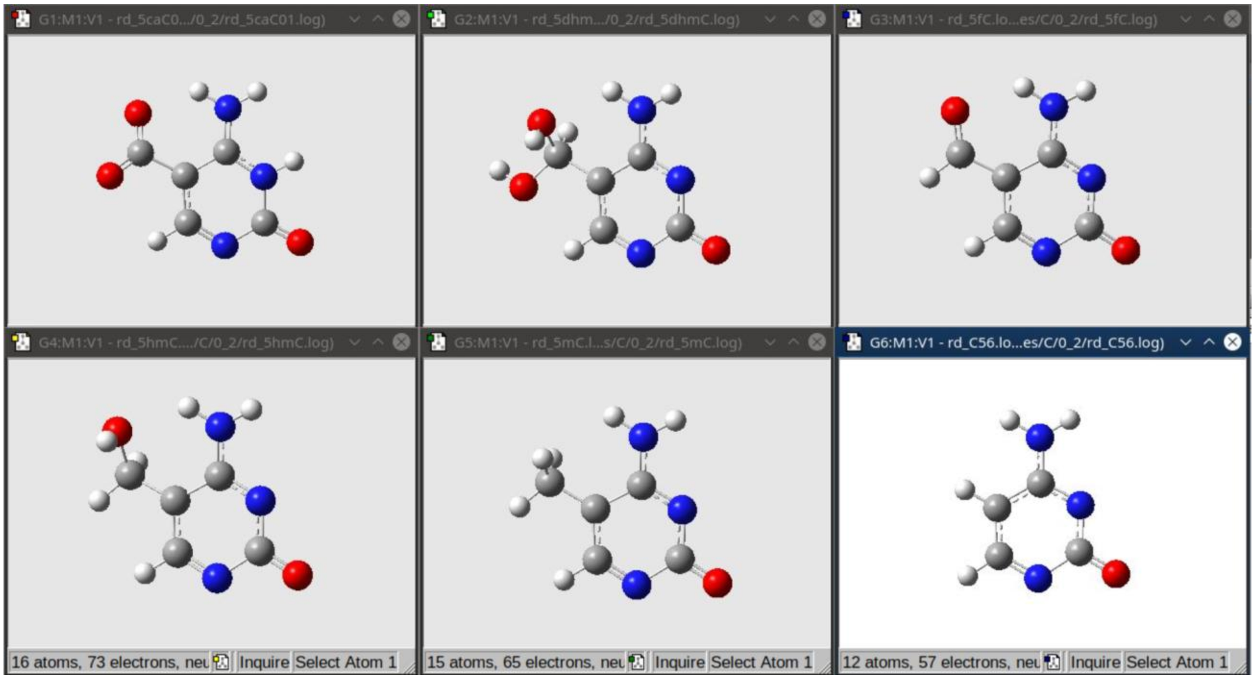

Figure S 4. rd_C derivatives: charge multiplicity “0 2”

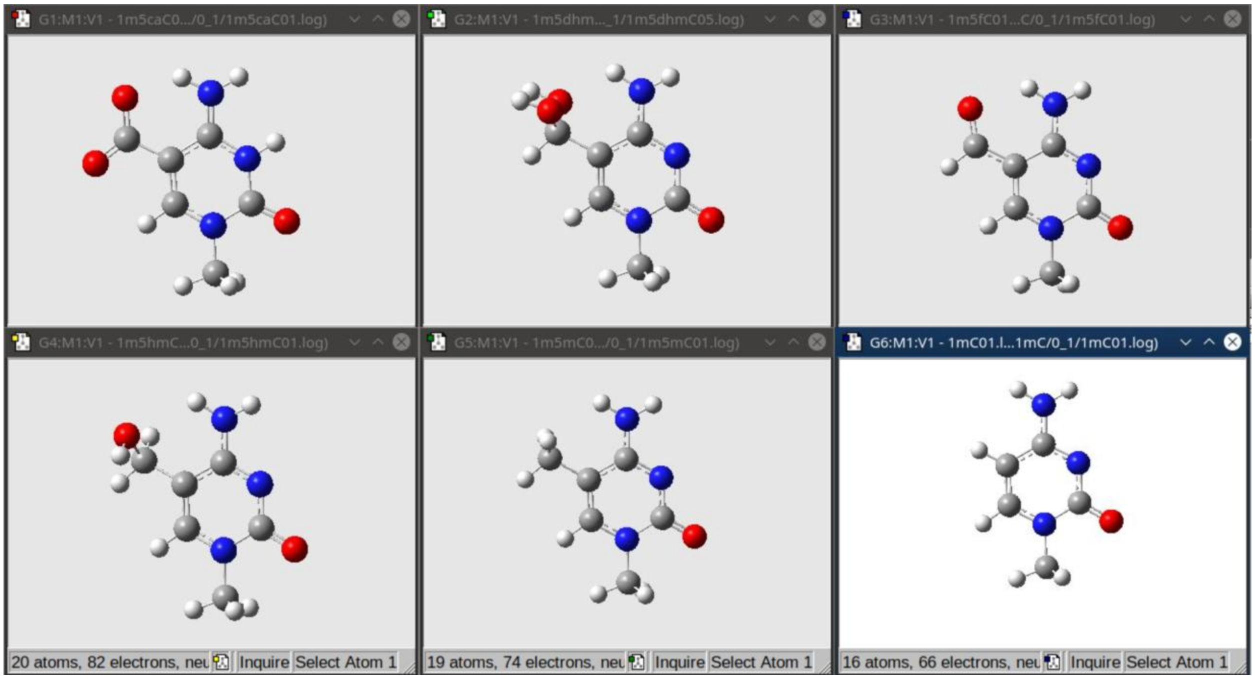

Figure S 5. 1mC derivatives: charge multiplicity “0 1”

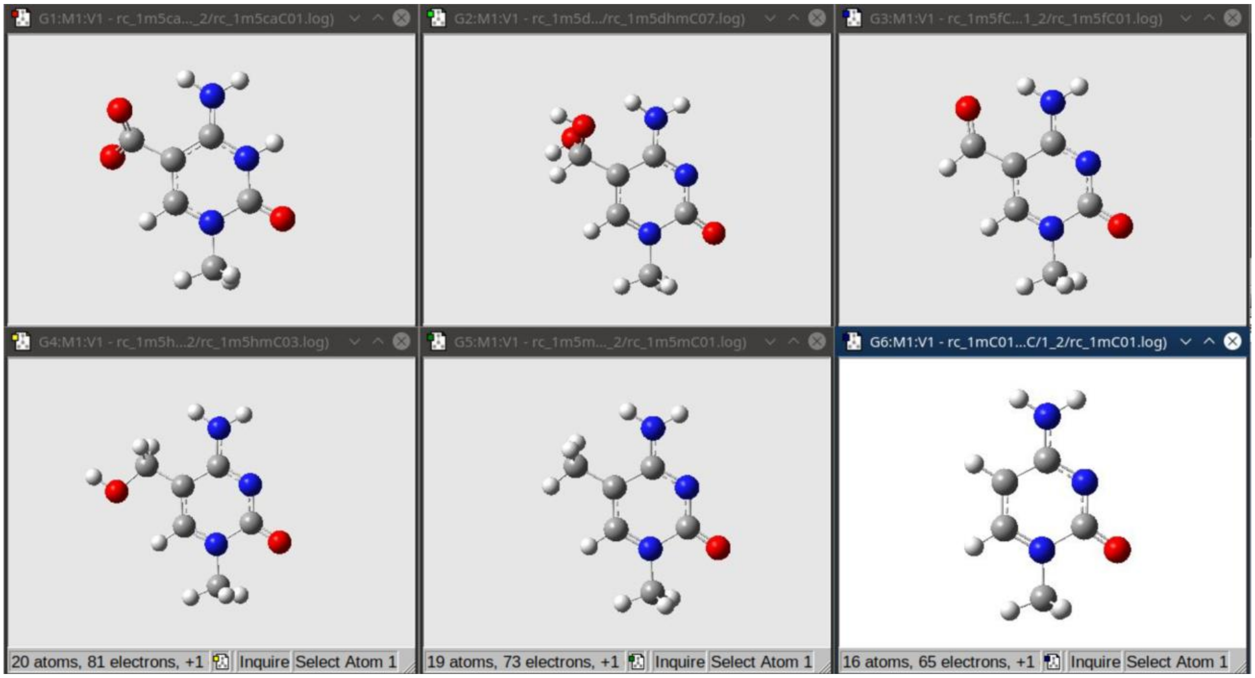

Figure S 6. rc_1mC derivatives: charge multiplicity “1 2”

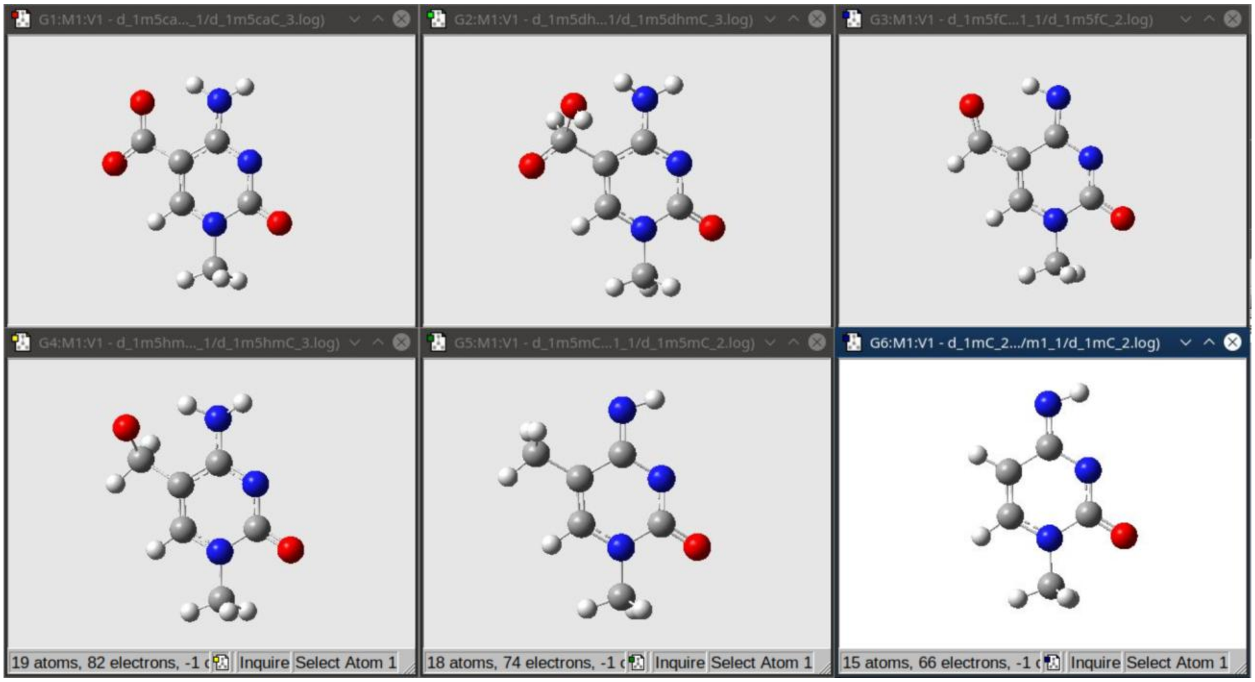

Figure S 7.d_1mC derivatives: charge multiplicity “-1 1”

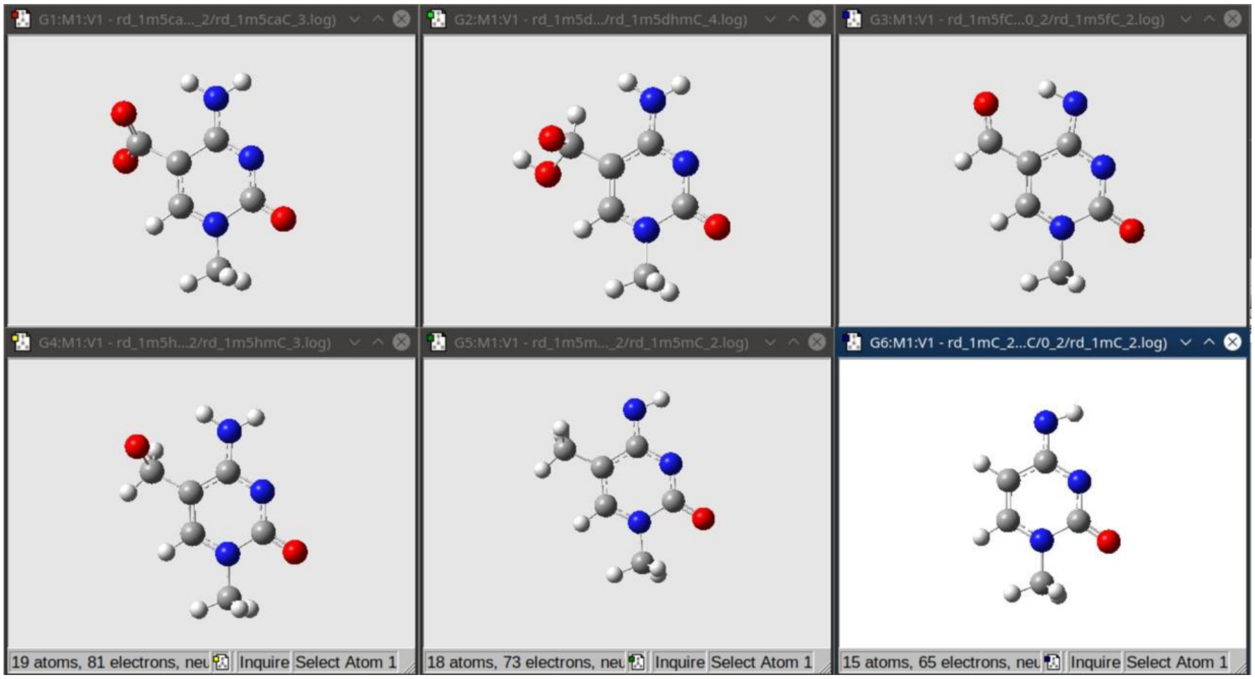

Figure S 8. rd_1mC derivatives: charge multiplicity “0 2”

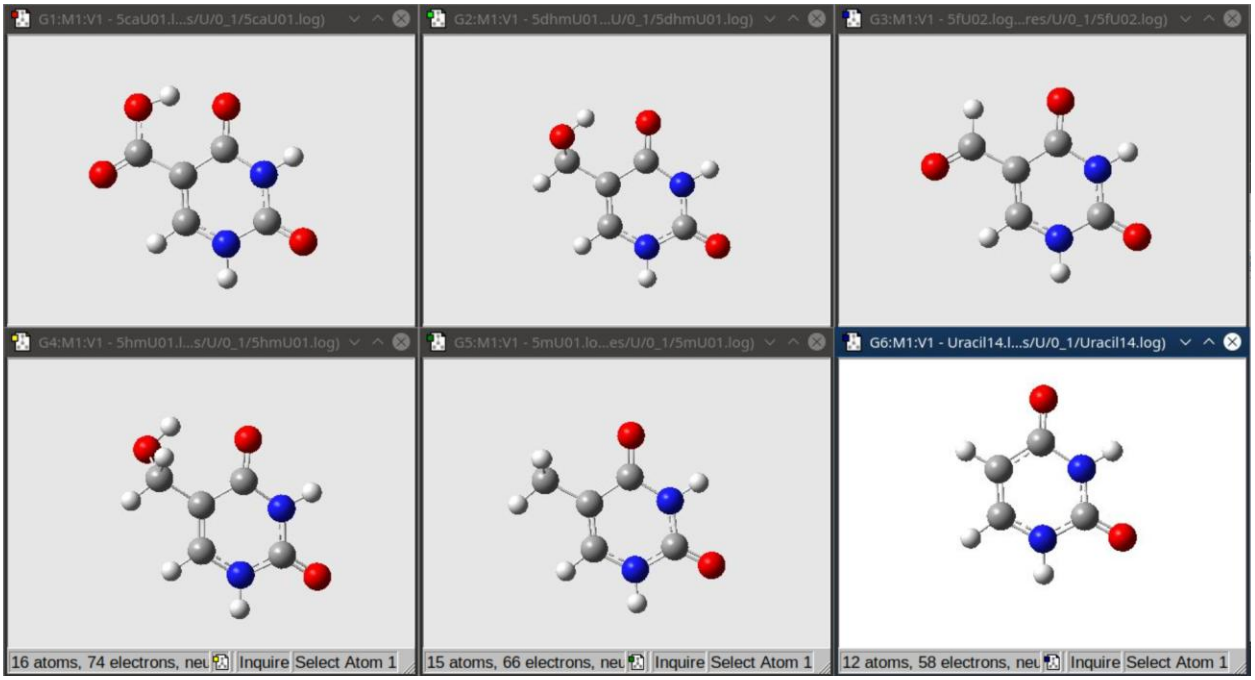

Figure S 9. U derivatives: charge multiplicity “0 1”

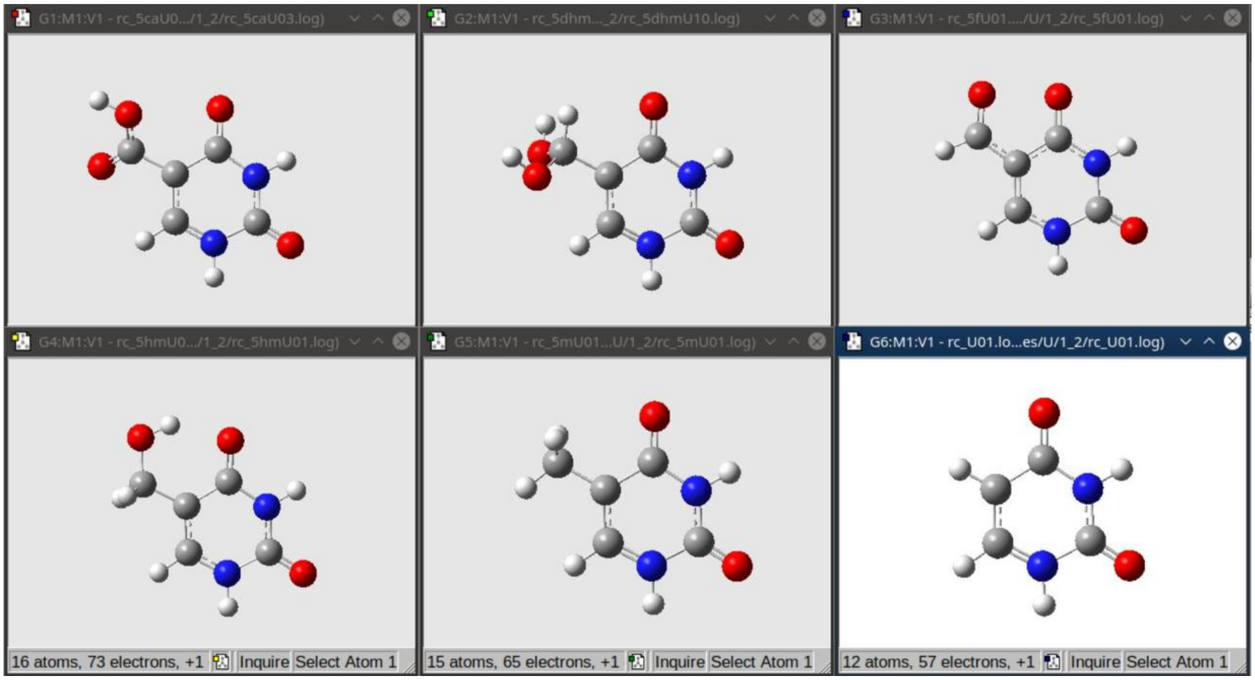

Figure S 10. rc_U derivatives: charge multiplicity “1 2”

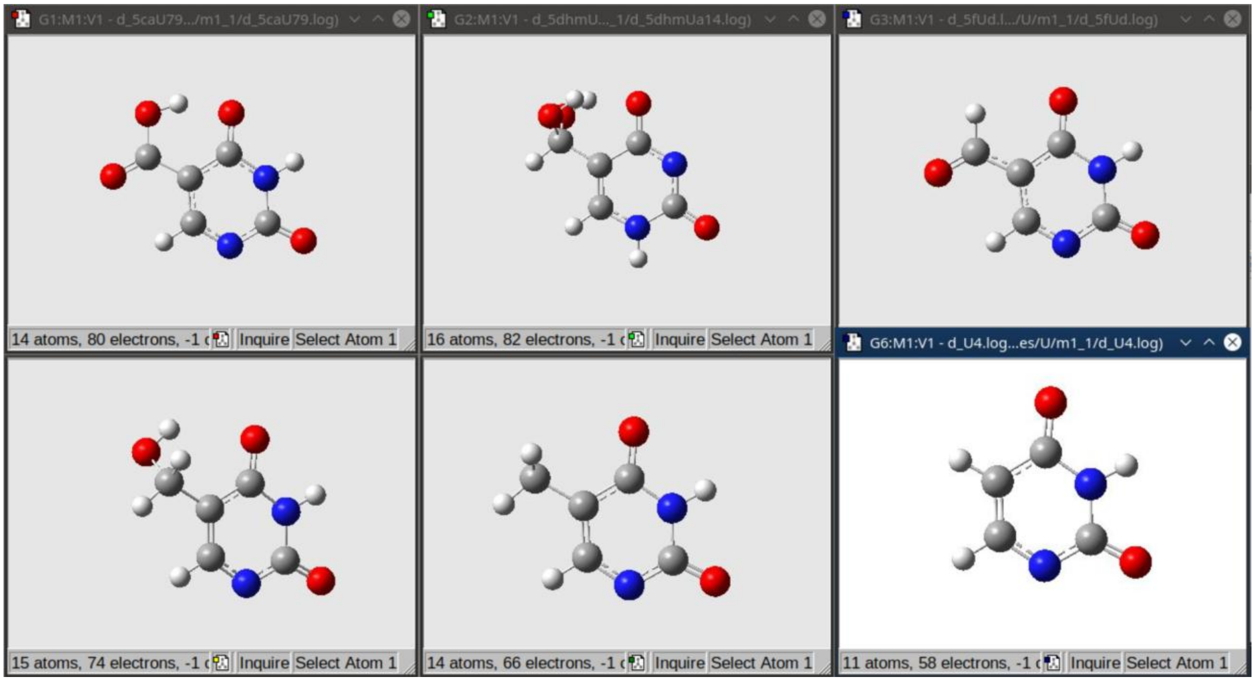

Figure S 11. d_U derivatives: charge multiplicity “-1 1”

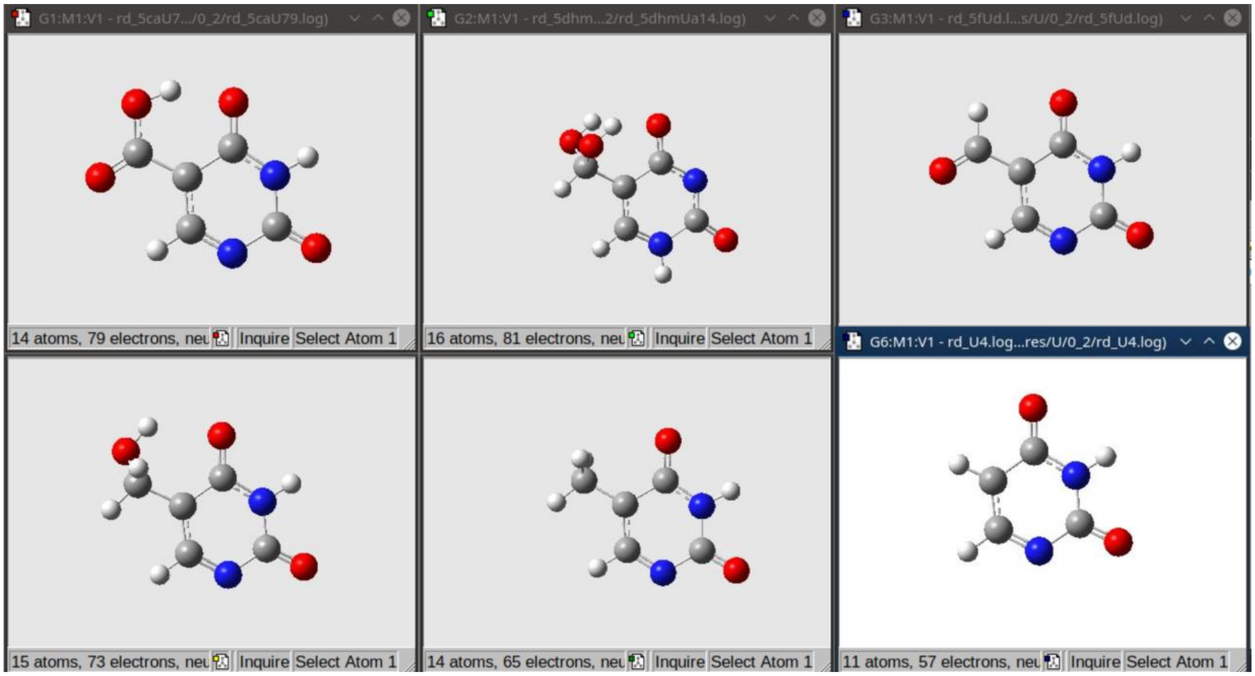

Figure S 12. rd_U derivatives: charge multiplicity “0 2”

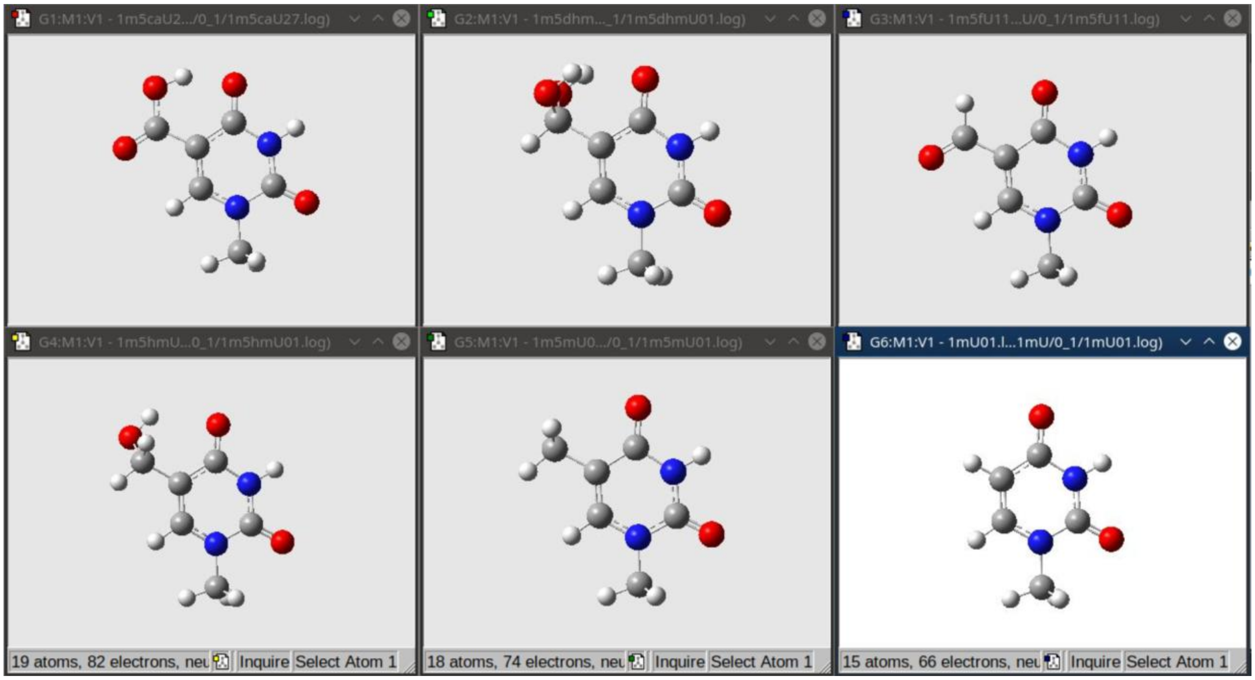

Figure S 13. 1mU derivatives: charge multiplicity “0 1”

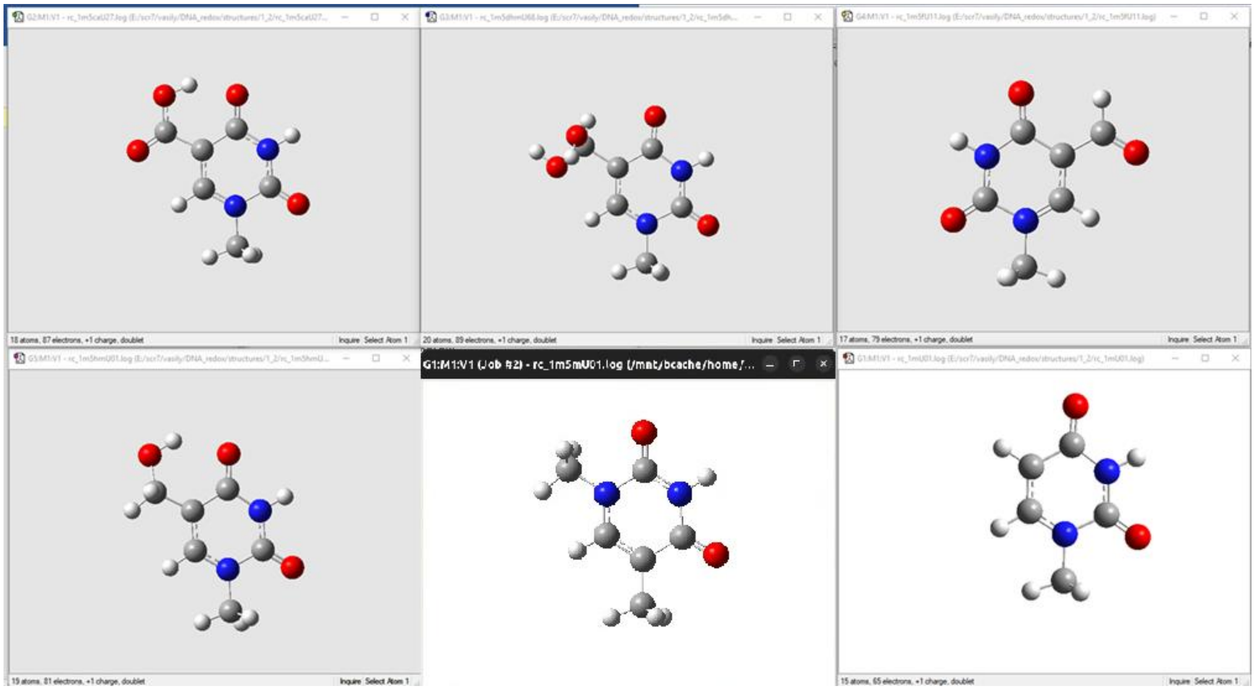

Figure S 14. rc_1mU derivatives: charge multiplicity “1 2”

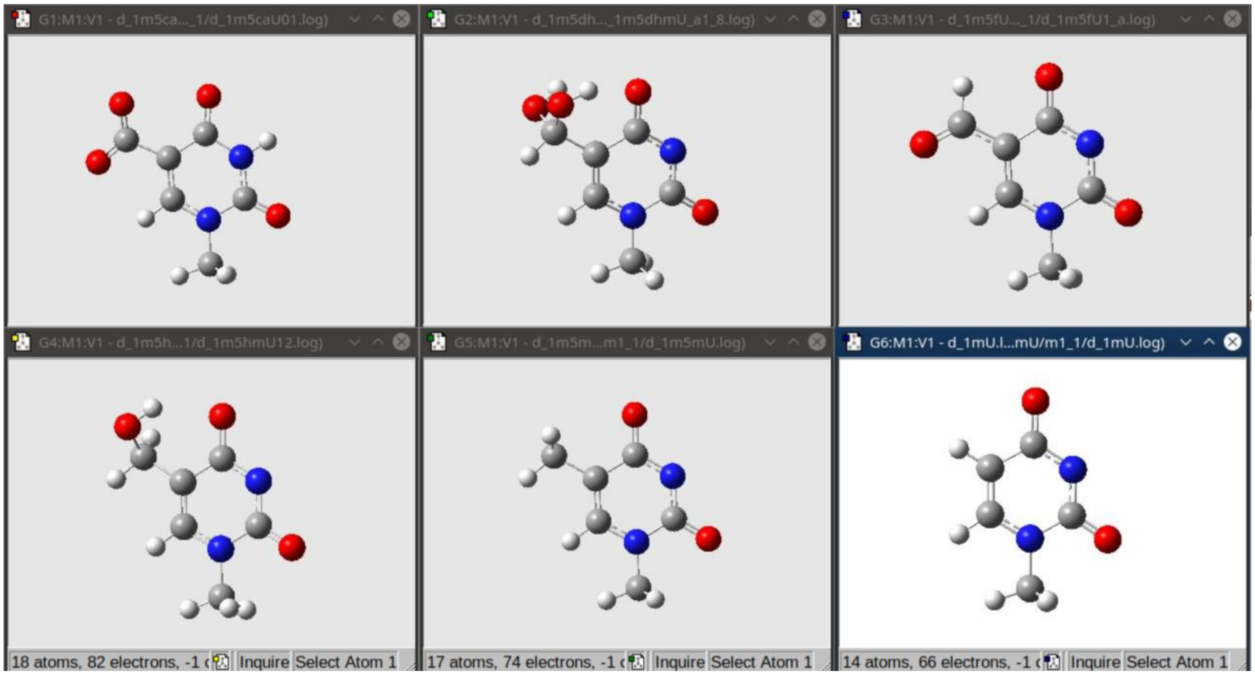

Figure S 15. d_1mU derivatives: charge multiplicity “-1 1”

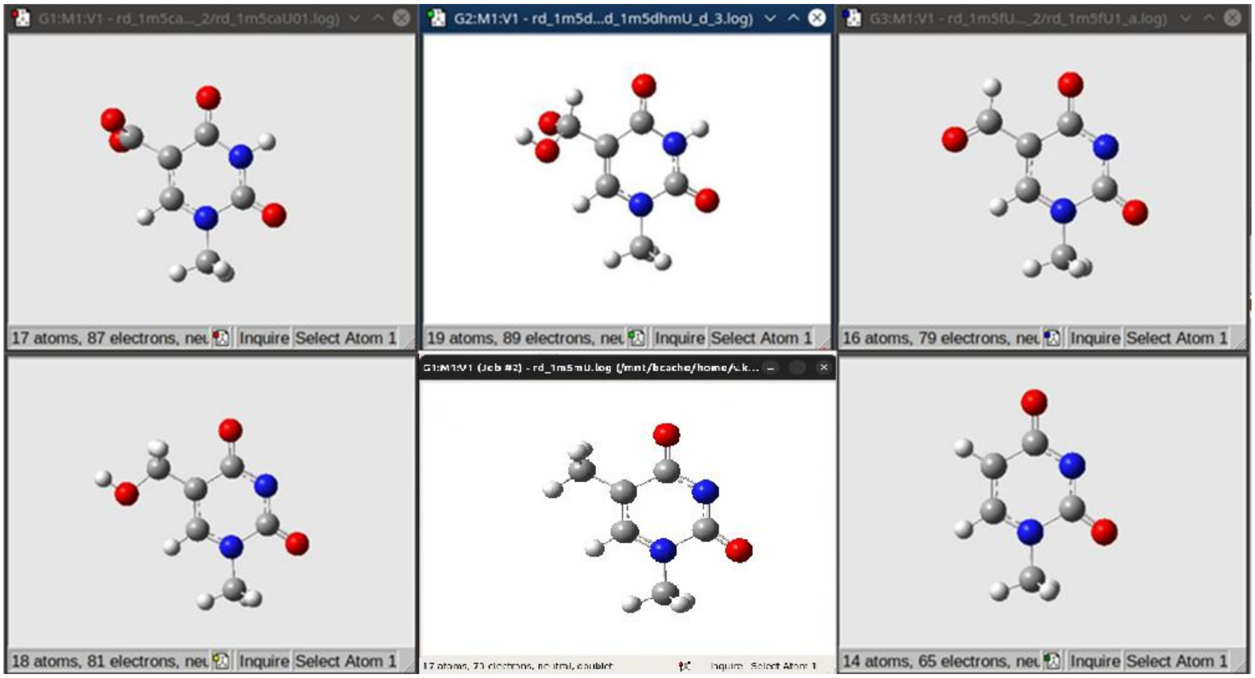

Figure S 16. rd_1mU derivatives: charge multiplicity “0 2”

3 Energies

Table S1. Energies calculated at the (U)B3LYP-D3 and DLPNO-CCSD(T) level of theory for the systems **without** the addition of explicit water. (Quasi-Harmonic Approximation)

№ conf	Molecule				(U)B3LYP-D3/6-31+G(d,p)//		DLPNO-CCSD(T)/BASIS//			
		SMD/(U)B3LYP-D3/6-31+G(d,p)			SMD/(U)B3LYP-D3/6-31+G(d,p)		SMD/(U)B3LYP-D3/6-31+G(d,p)			
		SMD/DFT			DFT		cc-pVTZ	cc-pVQZ	CBS	
		solution			gas		gas	gas	gas	solution (+Esolv)
		1atm	1atm	1atm	1atm	1atm	1atm	1atm	1atm	1atm
		Etot sol	Gcorr	G298 sol	Etot	Esolv	Etot	Etot	Etot	G298 sol
1	A_01	-467.3899123	0.0800592	-467.3098530	-467.3616811	-0.0282311	-466.5390752	-466.6769037	-464.6761458	-464.7043769
2	A_02	-467.3883455	0.0801974	-467.3081480	-467.3473911	-0.0409544	-466.5251331	-466.6632326	-464.6610841	-464.7020385
3	A_05	-467.3830126	0.0793106	-467.3037020	-467.3485521	-0.0344606	-466.5244688	-466.6626401	-464.6588989	-464.6933594
4	A_04	-467.3809574	0.0791013	-467.3018560	-467.3301701	-0.0507873	-466.5073375	-466.6456168	-464.6397434	-464.6905307
5	A_03	-467.3580735	0.0815815	-467.2764920	-467.2902417	-0.0678318	-466.4688446	-466.6064839	-464.5952610	-464.6630928
1	5mU01	-454.2047404	0.0816993	-454.1230410	-454.1790946	-0.0256458	-453.4135415	-453.5503929	-451.6648480	-451.6904938
2	5mU02	-454.1889777	0.0819867	-454.1069910	-454.1572580	-0.0317197	-453.3930873	-453.5300824	-451.6419214	-451.6736412
3	5mU03	-454.1869883	0.0818033	-454.1051850	-454.1448884	-0.0420999	-453.3819577	-453.5192477	-451.6286374	-451.6707373
4	5mU04	-454.1844523	0.0815282	-454.1029240	-454.1615856	-0.0228667	-453.3980131	-453.5346271	-451.6477318	-451.6705985
5	5mU05	-454.1825585	0.0811774	-454.1013810	-454.1474781	-0.0350804	-453.3847371	-453.5218201	-451.6333625	-451.6684428
6	5mU06	-454.1809402	0.0805152	-454.1004250	-454.1481491	-0.0327911	-453.3854530	-453.5222683	-451.6339908	-451.6667819
7	5mU08	-454.1790994	0.0814923	-454.0976070	-454.1299897	-0.0491096	-453.3688973	-453.5062977	-451.6158109	-451.6649206
8	5mU07	-454.1792129	0.0816449	-454.0975680	-454.1407659	-0.0384470	-453.3758400	-453.5127665	-451.6236227	-451.6620697
9	5mU11	-454.1770708	0.0804978	-454.0965730	-454.1480759	-0.0289949	-453.3951942	-453.5315981	-451.6443237	-451.6733186
10	5mU10	-454.1772341	0.0812160	-454.0960180	-454.1556398	-0.0215943	-453.3709722	-453.5079105	-451.6187475	-451.6403418
11	5mU09	-454.1775778	0.0816497	-454.0959280	-454.1576115	-0.0199663	-453.3968713	-453.5332718	-451.6462806	-451.6662469
12	5mU13	-454.1759775	0.0804004	-454.0955770	-454.1407658	-0.0352117	-453.3888006	-453.5253979	-451.6365550	-451.6717667
13	5mU12	-454.1763612	0.0815541	-454.0948070	-454.1359625	-0.0403987	-453.3889595	-453.5255319	-451.6366248	-451.6770236
14	5mU14	-454.1209095	0.0798925	-454.0410170	-454.0571420	-0.0637675	-453.2931953	-453.4306364	-451.5318827	-451.5956502
15	5mU15	-414.7912057	0.0406587	-414.7505470	-414.7782235	-0.0129822	-414.1026812	-414.2262768	-412.5441001	-412.5570823
1	G_01	-542.6457200	0.0831570	-542.5625630	-542.5993598	-0.0463603	-541.6784584	-541.8408826	-539.5780261	-539.6243864
2	G_02	-542.6446524	0.0832054	-542.5614470	-542.6010772	-0.0435752	-541.6793502	-541.8420689	-539.5792469	-539.6228221
3	G_19	-542.6395669	0.0828359	-542.5567310	-542.5890823	-0.0504847	-541.6671451	-541.8304034	-539.5662738	-539.6167584
4	G_07	-542.6329132	0.0831641	-542.5497490	-542.5995290	-0.0333841	-541.6808925	-541.8429159	-539.5814928	-539.6148770
5	G_06	-542.6309714	0.0818203	-542.5491510	-542.5595076	-0.0714638	-541.6371533	-541.8000114	-539.5331233	-539.6045871
6	G_12	-542.6320481	0.0829650	-542.5490830	-542.5986269	-0.0334211	#N/A	#N/A	#N/A	#N/A
7	G_08	-542.6312103	0.0829303	-542.5482800	-542.5948930	-0.0363174	#N/A	#N/A	#N/A	#N/A
8	G_13	-542.6291977	0.0827567	-542.5464410	-542.5817918	-0.0474060	#N/A	#N/A	#N/A	#N/A
9	G_11	-542.6236608	0.0824287	-542.5412320	-542.5756497	-0.0480111	#N/A	#N/A	#N/A	#N/A
10	G_18	-542.6206134	0.0852273	-542.5353860	-542.5439834	-0.0766300	#N/A	#N/A	#N/A	#N/A
11	G_17	-542.6188218	0.0846148	-542.5342070	-542.5334661	-0.0853558	#N/A	#N/A	#N/A	#N/A
12	G_04	-542.6168303	0.0828413	-542.5339890	-542.5664027	-0.0504276	#N/A	#N/A	#N/A	#N/A
13	G_03	-542.6138726	0.0831115	-542.5307610	-542.5484958	-0.0653768	#N/A	#N/A	#N/A	#N/A
14	G_05	-542.6067063	0.0848373	-542.5218690	-542.4972171	-0.1094892	#N/A	#N/A	#N/A	#N/A
15	G_10	-542.6051325	0.0845964	-542.5205360	-542.5261539	-0.0789786	#N/A	#N/A	#N/A	#N/A
16	G_15	-542.6051846	0.0847295	-542.5204550	-542.5321919	-0.0729927	#N/A	#N/A	#N/A	#N/A
17	G_16	-542.5990382	0.0841551	-542.5148830	-542.4813131	-0.1177250	#N/A	#N/A	#N/A	#N/A

18	G_09	-542.5577919	0.0819459	-542.4758460	-542.4819639	-0.0758281	#N/A	#N/A	#N/A	#N/A
1	C01	-395.0059909	0.0675438	-394.9384470	-394.9676379	-0.0383530	-394.2903188	-394.4100191	-392.7529425	-392.7912955
2	C02	-395.0001376	0.0669506	-394.9331870	-394.9558406	-0.0442970	-394.2779882	-394.3974415	-392.7403931	-392.7846901
3	C03	-394.9928364	0.0664724	-394.9263640	-394.9684989	-0.0243376	-394.2954721	-394.4144039	-392.7584001	-392.7827377
4	C04	-394.9927211	0.0668181	-394.9259030	-394.9672378	-0.0254834	-394.2943688	-394.4132642	-392.7572539	-392.7827373
5	C05	-394.9690172	0.0683061	-394.9007110	-394.8824527	-0.0865644	-394.2064143	-394.3259053	-392.6550467	-392.7416111
1	U114	-414.8799057	0.0560827	-414.8238230	-414.8531491	-0.0267566	-414.1683693	-414.2937248	-412.6132253	-412.6399820
2	U02	-414.8647348	0.0558468	-414.8088880	-414.8327863	-0.0319486	-414.1496872	-414.2751563	-412.5925489	-412.6244975
3	U03	-414.8631895	0.0559865	-414.8072030	-414.8211208	-0.0420687	-414.1390329	-414.2647161	-412.5803981	-412.6224668
4	U04	-414.8593768	0.0558987	-414.8034780	-414.8352118	-0.0241650	-414.1522962	-414.2774198	-412.5962496	-412.6204146
5	U05	-414.8573193	0.0552472	-414.8020720	-414.8203611	-0.0369582	-414.1384293	-414.2640219	-412.5804820	-412.6174402
6	U134	-414.8560663	0.0554503	-414.8006160	-414.8217981	-0.0342682	-414.1397851	-414.2651056	-412.5826222	-412.6168904
7	U07	-414.8557639	0.0559269	-414.7998370	-414.8177419	-0.0380220	-414.1341861	-414.2594540	-412.5769826	-412.6150047
8	U09	-414.8534727	0.0553777	-414.7980950	-414.8126988	-0.0407739	-414.1291645	-414.2545300	-412.5715435	-412.6123174
9	U08	-414.8537143	0.0556223	-414.7980920	-414.8025450	-0.0511693	-414.1222368	-414.2481416	-412.5624583	-412.6136276
10	U10	-414.8529310	0.0552149	-414.7977160	-414.8324955	-0.0204354	-414.1532017	-414.2780568	-412.5967076	-412.6171431
11	U11	-414.8525952	0.0554451	-414.7971500	-414.8306611	-0.0219341	-414.1517282	-414.2765060	-412.5950294	-412.6169635
12	U126	-414.8521422	0.0557432	-414.7963990	-414.8237881	-0.0283542	-414.1456185	-414.2705434	-412.5881355	-412.6164897
13	U13	-414.8519537	0.0560646	-414.7958890	-414.8237428	-0.0282109	-414.1456786	-414.2705507	-412.5882441	-412.6164550
14	U156	-414.7925275	0.0419675	-414.7505600	-414.7780857	-0.0144418	-414.1019472	-414.2259807	-412.5433117	-412.5577535
15	U123	-414.7960420	0.0553749	-414.7406670	-414.7306001	-0.0654419	-414.0473893	-414.1733430	-412.4799902	-412.5454321
1	rG_166	-1038.9455454	0.2120780	-1038.7334674	-1038.8810922	-0.0644532	-1037.1772376	-1037.4918121	-1033.1848481	-1033.2493013
2	rG_154	-1038.9454530	0.2121458	-1038.7333071	-1038.8817540	-0.0636990	#N/A	#N/A	#N/A	#N/A
3	rG_153	-1038.9447999	0.2124501	-1038.7323497	-1038.8819145	-0.0628853	#N/A	#N/A	#N/A	#N/A
4	rG_159	-1038.9432208	0.2124259	-1038.7307949	-1038.8747657	-0.0684552	#N/A	#N/A	#N/A	#N/A
5	rG_174	-1038.9430918	0.2128318	-1038.7302600	-1038.8738996	-0.0691923	#N/A	#N/A	#N/A	#N/A
6	rG_158	-1038.9412311	0.2113741	-1038.7298569	-1038.8775897	-0.0636413	#N/A	#N/A	#N/A	#N/A
7	rG_156	-1038.9407199	0.2101775	-1038.7305424	-1038.8767813	-0.0639386	#N/A	#N/A	#N/A	#N/A
8	rG_161	-1038.9407161	0.2104601	-1038.7302559	-1038.8791533	-0.0615627	#N/A	#N/A	#N/A	#N/A
9	rG_181	-1038.9406995	0.2103252	-1038.7303743	-1038.8694248	-0.0712747	#N/A	#N/A	#N/A	#N/A
10	rG_72	-1038.9405822	0.2122523	-1038.7283298	-1038.8734976	-0.0670846	#N/A	#N/A	#N/A	#N/A
11	rG_51	-1038.9405714	0.2105669	-1038.7300044	-1038.8685961	-0.0719753	#N/A	#N/A	#N/A	#N/A
12	rG_183	-1038.9404730	0.2097834	-1038.7306896	-1038.8709750	-0.0694980	#N/A	#N/A	#N/A	#N/A
13	rG_191	-1038.9404312	0.2097320	-1038.7306992	-1038.8693015	-0.0711297	#N/A	#N/A	#N/A	#N/A
14	rG_170	-1038.9404002	0.2107033	-1038.7296969	-1038.8705329	-0.0698673	#N/A	#N/A	#N/A	#N/A
15	rG_198	-1038.9403731	0.2122380	-1038.7281351	-1038.8730104	-0.0673627	#N/A	#N/A	#N/A	#N/A
16	rG_187	-1038.9401485	0.2123710	-1038.7277774	#N/A	#N/A	#N/A	#N/A	#N/A	#N/A
17	rG_197	-1038.9400709	0.2121046	-1038.7279663	#N/A	#N/A	#N/A	#N/A	#N/A	#N/A
18	rG_189	-1038.9398815	0.2103780	-1038.7295035	#N/A	#N/A	#N/A	#N/A	#N/A	#N/A
19	rG_135	-1038.9398344	0.2108692	-1038.7289651	#N/A	#N/A	#N/A	#N/A	#N/A	#N/A
20	rG_53	-1038.9397243	0.2102947	-1038.7294296	#N/A	#N/A	#N/A	#N/A	#N/A	#N/A
1	1mG_01	-581.9543271	0.1099731	-581.8443540	-581.9134097	-0.0409174	-580.9127672	-581.0862295	-578.6153249	-578.6562423
2	1mG_02	-581.9535660	0.1098849	-581.8436810	-581.9155642	-0.0380018	-580.9139394	-581.0877095	-578.6169567	-578.6549585
3	1mG_06	-581.9398704	0.1086763	-581.8311940	-581.8750091	-0.0648612	-580.8728954	-581.0469090	-578.5722317	-578.6370930
4	1mG_03	-581.9252357	0.1096157	-581.8156200	-581.8800854	-0.0451503	-580.8809031	-581.0544326	-578.5823232	-578.6274735
5	1mG_04	-581.9200834	0.1100834	-581.8100000	-581.8607165	-0.0593669	-580.8631786	-581.0364973	-578.5618782	-578.6212451
6	1mG_05	-581.9162690	0.1112809	-581.8049880	-581.8137551	-0.1025139	#N/A	#N/A	#N/A	#N/A
1	X01	-562.5210176	0.0714676	-562.4495500	-562.4867208	-0.0342969	-561.5560694	-561.7247855	-559.4395790	-559.4738759

2	X02	-562.5169521	0.0713011	-562.4456510	-562.4709015	-0.0460506	-561.5416780	-561.7103111	-559.4239414	-559.4699920
3	X03	-562.5102952	0.0715381	-562.4387570	-562.4473408	-0.0629544	-561.5127959	-561.6823429	-559.3904419	-559.4533963
4	X18	-562.5055249	0.0706939	-562.4348310	-562.4652909	-0.0402340	-561.5383191	-561.7066156	-559.4187433	-559.4589773
5	X15	-562.5054462	0.0711162	-562.4343300	-562.4684996	-0.0369466	-561.5404953	-561.7091497	-559.4215004	-559.4584470
6	X19	-562.5024200	0.0709550	-562.4314650	-562.4534734	-0.0489466	-561.5272740	-561.6957913	-559.4064978	-559.4554445
7	X11	-562.5020524	0.0712224	-562.4308300	-562.4590425	-0.0430099	-561.5297435	-561.6984863	-559.4104830	-559.4534929
8	X14	-562.5021181	0.0716540	-562.4304640	-562.4541150	-0.0480031	#N/A	#N/A	#N/A	#N/A
9	X09	-562.4998187	0.0710777	-562.4287410	-562.4579724	-0.0418463	#N/A	#N/A	#N/A	#N/A
10	X12	-562.4994312	0.0707491	-562.4286820	-562.4418652	-0.0575660	#N/A	#N/A	#N/A	#N/A
11	X22	-562.4988509	0.0710858	-562.4277650	-562.4499155	-0.0489354	#N/A	#N/A	#N/A	#N/A
12	X21	-562.4976686	0.0710226	-562.4266460	-562.4455482	-0.0521204	#N/A	#N/A	#N/A	#N/A
13	X20	-562.4979568	0.0719057	-562.4260510	-562.4523682	-0.0455885	#N/A	#N/A	#N/A	#N/A
14	X04	-562.4968247	0.0708517	-562.4259730	-562.4058558	-0.0909689	#N/A	#N/A	#N/A	#N/A
15	X10	-562.4961934	0.0705083	-562.4256850	-562.4413838	-0.0548096	#N/A	#N/A	#N/A	#N/A
16	X17	-562.4958031	0.0716191	-562.4241840	-562.4417798	-0.0540233	#N/A	#N/A	#N/A	#N/A
17	X28	-562.4951044	0.0710294	-562.4240750	-562.4600001	-0.0351043	#N/A	#N/A	#N/A	#N/A
18	X27	-562.4951258	0.0711387	-562.4239870	-562.4597247	-0.0354011	#N/A	#N/A	#N/A	#N/A
19	X13	-562.4930267	0.0693696	-562.4236570	-562.4267089	-0.0663177	#N/A	#N/A	#N/A	#N/A
20	X25	-562.4943716	0.0707665	-562.4236050	-562.4578688	-0.0365028	#N/A	#N/A	#N/A	#N/A
1	Bixanthine_22	-1123.7831791	0.1449525	-1123.6382265	#N/A	#N/A	#N/A	#N/A	#N/A	#N/A
2	Bixanthine_19	-1123.7816364	0.1431613	-1123.6384751	#N/A	#N/A	#N/A	#N/A	#N/A	#N/A
3	bixanthine_13	-1123.7802957	0.1435244	-1123.6367712	#N/A	#N/A	#N/A	#N/A	#N/A	#N/A
4	bixanthine_14cc	-1123.7767940	0.1433245	-1123.6334695	#N/A	#N/A	#N/A	#N/A	#N/A	#N/A
5	Bixanthine_17	-1123.7711613	0.1438038	-1123.6273575	#N/A	#N/A	#N/A	#N/A	#N/A	#N/A
6	Bixanthine_16	-1123.7700176	0.1448681	-1123.6251494	#N/A	#N/A	#N/A	#N/A	#N/A	#N/A
7	Bixanthine_15	-1123.7689704	0.1437968	-1123.6251736	#N/A	#N/A	#N/A	#N/A	#N/A	#N/A
8	Bixanthine_23	-1123.7672944	0.1434434	-1123.6238510	#N/A	#N/A	#N/A	#N/A	#N/A	#N/A
9	Bixanthine_25	-1123.7665869	0.1439595	-1123.6226274	#N/A	#N/A	#N/A	#N/A	#N/A	#N/A
10	bixanthine_2	-1123.7655460	0.1432670	-1123.6222789	#N/A	#N/A	#N/A	#N/A	#N/A	#N/A
11	Bixanthine_18	-1123.7642242	0.1430784	-1123.6211458	#N/A	#N/A	#N/A	#N/A	#N/A	#N/A
12	bixanthine_3	-1123.7416544	0.1437395	-1123.5979149	#N/A	#N/A	#N/A	#N/A	#N/A	#N/A
13	bixanthine_8	-1123.7349464	0.1422398	-1123.5927065	#N/A	#N/A	#N/A	#N/A	#N/A	#N/A
14	bixanthine_11	-1123.7155596	0.1423330	-1123.5732265	#N/A	#N/A	#N/A	#N/A	#N/A	#N/A
15	bixanthine_10	-1123.7133812	0.1431924	-1123.5701888	#N/A	#N/A	#N/A	#N/A	#N/A	#N/A
16	bixanthine_12	-1123.7101343	0.1414657	-1123.5686686	#N/A	#N/A	#N/A	#N/A	#N/A	#N/A
17	bixanthine_9	-1123.6952038	0.1427292	-1123.5524745	#N/A	#N/A	#N/A	#N/A	#N/A	#N/A
18	bixanthine_5	-1123.5508947	0.1408694	-1123.4100252	#N/A	#N/A	#N/A	#N/A	#N/A	#N/A
1	hX_a	-487.2624213	0.0689912	-487.1934300	-487.2241692	-0.0382520	-486.3931507	-486.5370652	-484.5086324	-484.5468844
2	hX_b	-487.2624568	0.0691257	-487.1933310	-487.2263161	-0.0361407	-486.3947261	-486.5389601	-484.5109187	-484.5470594
3	hX_h	-487.2491730	0.0685059	-487.1806670	-487.2195502	-0.0296227	-486.3911928	-486.5348603	-484.5089405	-484.5385632
4	hX_c	-487.2483327	0.0680586	-487.1802740	-487.2155447	-0.0327880	-486.3864551	-486.5303339	-484.5039316	-484.5367196
5	hX_g	-487.2482880	0.0682209	-487.1800670	-487.2176478	-0.0306401	-486.3895346	-486.5334101	-484.5069504	-484.5375905
6	hX_d	-487.2463003	0.0677632	-487.1785370	-487.2014998	-0.0448005	-486.3736074	-486.5177817	-484.4898316	-484.5346322
7	hX_e	-487.2357506	0.0682346	-487.1675160	-487.1942188	-0.0415318	-486.3647287	-486.5088403	-484.4792060	-484.5207378
8	hX_f	-487.2330378	0.0679678	-487.1650700	-487.1773381	-0.0556997	#N/A	#N/A	#N/A	#N/A
1	rc_A_01	-467.1738013	0.0795613	-467.0942400	-467.0653279	-0.1084734	-466.2408215	-466.3735131	-464.4091088	-464.5175821
2	rc_A_02	-467.1692604	0.0798164	-467.0894440	-467.0425092	-0.1267512	-466.2194648	-466.3523885	-464.3750590	-464.5018102
3	rc_A_05	-467.1680298	0.0788298	-467.0892000	-467.0548288	-0.1132010	-466.2325319	-466.3652782	-464.4049718	-464.5181728

4	rc_A_04	-467.1664992	0.0782851	-467.0882140	-467.0373210	-0.1291782	-466.2155296	-466.3483357	-464.3811076	-464.5102858
5	rc_A_03	-467.1319601	0.0782920	-467.0536680	-466.9937506	-0.1382095	#N/A	#N/A	#N/A	#N/A
1	rc_5mU01	-453.9736640	0.0797930	-453.8938710	-453.8567892	-0.1168748	-453.0902060	-453.2222362	-451.3657722	-451.4826470
2	rc_5mU04	-453.9593482	0.0797862	-453.8795620	-453.8521125	-0.1072357	-453.0874352	-453.2191376	-451.3627111	-451.4699468
3	rc_5mU02	-453.9588596	0.0797416	-453.8791180	-453.8439214	-0.1149382	-453.0787085	-453.2106994	-451.3367548	-451.4516931
4	rc_5mU06	-453.9565708	0.0788787	-453.8776920	-453.8395031	-0.1170677	-453.0758589	-453.2076474	-451.3501453	-451.4672130
5	rc_5mU07	-453.9558795	0.0792925	-453.8765870	-453.8334085	-0.1224710	-453.0713040	-453.2031561	-451.3318115	-451.4542825
6	rc_5mU10	-453.9555261	0.0797400	-453.8757860	-453.8317345	-0.1237915	-453.0693760	-453.2012047	-451.3313558	-451.4551473
7	rc_5mU03	-453.9557672	0.0802722	-453.8754950	-453.8294427	-0.1263245	-453.0662502	-453.1984615	-451.3210103	-451.4473348
8	rc_5mU05	-453.9503383	0.0793252	-453.8710130	-453.8318834	-0.1184548	-453.0674254	-453.1993763	-451.3281242	-451.4465790
9	rc_5mU08	-453.9458487	0.0795536	-453.8662950	-453.8128052	-0.1330435	-453.0493514	-453.1814587	-451.3077595	-451.4408030
10	rc_5mU09	-453.9452320	0.0802700	-453.8649620	-453.8391912	-0.1060409	-453.0752555	-453.2069979	-451.3594655	-451.4655064
11	rc_5mU13	-453.9425810	0.0792070	-453.8633740	-453.8261729	-0.1164081	-453.0638909	-453.1958152	-451.3444480	-451.4608561
12	rc_5mU12	-453.9426128	0.0799828	-453.8626300	-453.8255944	-0.1170184	-453.0632331	-453.1951813	-451.3441980	-451.4612164
13	rc_5mU14	-453.9029559	0.0783919	-453.8245640	-453.7709880	-0.1319679	-453.0089469	-453.1405402	-451.2624329	-451.3944008
14	rc_5mU15	-414.5391660	0.0403999	-414.4987660	-414.4355250	-0.1036410	-413.7568031	-413.8752442	-412.2221006	-412.3257416
1	rc_G_01	-542.4407598	0.0829448	-542.3578150	-542.3190785	-0.1216813	-541.3987468	-541.5554778	-539.3242767	-539.4459580
2	rc_G_02	-542.4376706	0.0829345	-542.3547360	-542.3163372	-0.1213333	-541.3952254	-541.5520336	-539.3228694	-539.4442028
3	rc_G_07	-542.4276466	0.0832215	-542.3444250	-542.3183119	-0.1093347	-541.3979907	-541.5547201	-539.3285988	-539.4379335
4	rc_G_12	-542.4261841	0.0831140	-542.3430700	-542.3154173	-0.1107668	-541.3954546	-541.5523180	-539.3236552	-539.4344220
5	rc_G_06	-542.4243805	0.0817885	-542.3425920	-542.2836339	-0.1407466	-541.3660060	-541.5228084	-539.2925429	-539.4332895
6	rc_G_08	-542.4245081	0.0832371	-542.3412710	-542.3115565	-0.1129517	#N/A	#N/A	#N/A	#N/A
7	rc_G_19	-542.4215213	0.0824872	-542.3390340	-542.2919099	-0.1296114	#N/A	#N/A	#N/A	#N/A
8	rc_G_13	-542.4217502	0.0828501	-542.3389000	-542.2963594	-0.1253908	#N/A	#N/A	#N/A	#N/A
9	rc_G_11	-542.4146031	0.0825070	-542.3320960	-542.2909911	-0.1236120	#N/A	#N/A	#N/A	#N/A
10	rc_G_14	-542.4133079	0.0817089	-542.3315990	-542.2905582	-0.1227497	#N/A	#N/A	#N/A	#N/A
11	rc_G_04	-542.4121086	0.0828496	-542.3292590	-542.2837294	-0.1283792	#N/A	#N/A	#N/A	#N/A
12	rc_G_03	-542.4098969	0.0829648	-542.3269320	-542.2686608	-0.1412361	#N/A	#N/A	#N/A	#N/A
13	rc_G_17	-542.4081787	0.0834257	-542.3247530	-542.2680828	-0.1400959	#N/A	#N/A	#N/A	#N/A
14	rc_G_18	-542.4069294	0.0830594	-542.3238700	-542.2713199	-0.1356095	#N/A	#N/A	#N/A	#N/A
15	rc_G_05	-542.3969981	0.0820470	-542.3149510	-542.2318262	-0.1651719	#N/A	#N/A	#N/A	#N/A
16	rc_G_16	-542.3905957	0.0819976	-542.3085980	-542.2220309	-0.1685648	#N/A	#N/A	#N/A	#N/A
17	rc_G_10	-542.3887325	0.0821644	-542.3065680	-542.2496078	-0.1391247	#N/A	#N/A	#N/A	#N/A
18	rc_G_15	-542.3879681	0.0820861	-542.3058820	-542.2514588	-0.1365093	#N/A	#N/A	#N/A	#N/A
19	rc_G_09	-542.3603862	0.0823091	-542.2780770	-542.2145226	-0.1458635	#N/A	#N/A	#N/A	#N/A
1	rc_C01	-394.7821290	0.0668469	-394.7152820	-394.6554173	-0.1267117	-393.9816302	-394.0954174	-392.4573178	-392.5840295
2	rc_C02	-394.7746201	0.0668320	-394.7077880	-394.6539377	-0.1206823	-393.9773494	-394.0910553	-392.4440005	-392.5646828
3	rc_C04	-394.7689079	0.0660648	-394.7028430	-394.6565268	-0.1123811	-393.9827517	-394.0965694	-392.4704610	-392.5828421
4	rc_C03	-394.7693593	0.0667493	-394.7026100	-394.6559977	-0.1133616	-393.9824755	-394.0962107	-392.4708132	-392.5841748
5	rc_C05	-394.7512459	0.0667118	-394.6845340	-394.6052403	-0.1460055	-393.9362897	-394.0491055	-392.4000870	-392.5460925
1	rc_U01	-414.6366283	0.0542853	-414.5823430	-414.5136351	-0.1229933	-413.8304884	-413.9505348	-412.2945907	-412.4175840
2	rc_U04	-414.6236957	0.0542157	-414.5694800	-414.5113735	-0.1123223	-413.8300315	-413.9497693	-412.2952038	-412.4075261
3	rc_U02	-414.6229257	0.0540776	-414.5688480	-414.5038996	-0.1190261	-413.8225362	-413.9425651	-412.2722247	-412.3912508
4	rc_U03	-414.6208267	0.0530976	-414.5677290	-414.4926336	-0.1281931	-413.8126767	-413.9328582	-412.2610864	-412.3892795
5	rc_U07	-414.6219442	0.0542611	-414.5676830	-414.4967341	-0.1252101	-413.8172145	-413.9370633	-412.2711457	-412.3963558
6	rc_U34	-414.6214636	0.0539546	-414.5675090	-414.4988544	-0.1226093	-413.8187228	-413.9385447	-412.2830215	-412.4056308
7	rc_U09	-414.6210897	0.0540556	-414.5670340	-414.4937907	-0.1272990	-413.8144800	-413.9343372	-412.2694728	-412.3967717
8	rc_U05	-414.6144998	0.0539138	-414.5605860	-414.4923983	-0.1221015	-413.8107401	-413.9305611	-412.2675791	-412.3896806

9	rc_U08	-414.6098533	0.0536373	-414.5562160	-414.4741810	-0.1356723	-413.7930796	-413.9130442	-412.2503946	-412.3860669
10	rc_U10	-414.6084403	0.0533292	-414.5551110	-414.4973976	-0.1110427	-413.8171355	-413.9369796	-412.2930109	-412.4040536
11	rc_U11	-414.6086820	0.0541930	-414.5544890	-414.4963373	-0.1123447	-413.8164540	-413.9362577	-412.2916357	-412.4039804
12	rc_U26	-414.6067004	0.0537354	-414.5529650	-414.4859204	-0.1207801	-413.8066843	-413.9266977	-412.2795549	-412.4003350
13	rc_U13	-414.6071165	0.0545494	-414.5525670	-414.4867518	-0.1203646	-413.8077118	-413.9276538	-412.2802333	-412.4005979
14	rc_U14	-414.5694815	0.0534564	-414.5160250	-414.4332462	-0.1362353	-413.7544047	-413.8740815	-412.2033369	-412.3395722
15	rc_U15	-414.5387858	0.0411267	-414.4976590	-414.4295268	-0.1092590	-413.7506082	-413.8698083	-412.2174611	-412.3267202
1	rc_rG_166	-1038.7371552	0.2122385	-1038.5249166	-1038.6025830	-0.1345721	-1036.8975495	-1037.2064542	-1032.9355553	-1033.0701274
2	rc_rG_154	-1038.7368594	0.2118933	-1038.5249661	-1038.6008158	-0.1360437	#N/A	#N/A	#N/A	#N/A
3	rc_rG_153	-1038.7362272	0.2124484	-1038.5237787	-1038.5964203	-0.1398068	#N/A	#N/A	#N/A	#N/A
4	rc_rG_21	-1038.7347629	0.2110675	-1038.5236953	-1038.5970284	-0.1377345	#N/A	#N/A	#N/A	#N/A
5	rc_rG_159	-1038.7346987	0.2120981	-1038.5226006	-1038.5961395	-0.1385593	#N/A	#N/A	#N/A	#N/A
6	rc_rG_181	-1038.7346760	0.2117445	-1038.5229314	-1038.5920362	-0.1426398	#N/A	#N/A	#N/A	#N/A
7	rc_rG_183	-1038.7345962	0.2110350	-1038.5235612	-1038.5947926	-0.1398037	#N/A	#N/A	#N/A	#N/A
8	rc_rG_135	-1038.7345432	0.2111303	-1038.5234128	-1038.5918055	-0.1427377	#N/A	#N/A	#N/A	#N/A
9	rc_rG_34	-1038.7344926	0.2101822	-1038.5243104	-1038.5886257	-0.1458669	#N/A	#N/A	#N/A	#N/A
10	rc_rG_174	-1038.7344696	0.2115446	-1038.5229249	-1038.5904974	-0.1439722	#N/A	#N/A	#N/A	#N/A
11	rc_rG_45	-1038.7344420	0.2110216	-1038.5234204	-1038.5895841	-0.1448580	#N/A	#N/A	#N/A	#N/A
12	rc_rG_51	-1038.7343792	0.2107164	-1038.5236627	-1038.5950054	-0.1393738	#N/A	#N/A	#N/A	#N/A
13	rc_rG_170	-1038.7343659	0.2097977	-1038.5245682	-1038.5911799	-0.1431860	#N/A	#N/A	#N/A	#N/A
14	rc_rG_22	-1038.7343635	0.2097752	-1038.5245882	-1038.5986405	-0.1357230	#N/A	#N/A	#N/A	#N/A
15	rc_rG_191	-1038.7341081	0.2100527	-1038.5240554	-1038.5939199	-0.1401883	#N/A	#N/A	#N/A	#N/A
16	rc_rG_152	-1038.7340807	0.2102551	-1038.5238255	#N/A	#N/A	#N/A	#N/A	#N/A	#N/A
17	rc_rG_103	-1038.7339413	0.2101329	-1038.5238084	#N/A	#N/A	#N/A	#N/A	#N/A	#N/A
18	rc_rG_111	-1038.7338929	0.2112826	-1038.5226103	#N/A	#N/A	#N/A	#N/A	#N/A	#N/A
19	rc_rG_171	-1038.7338894	0.2100119	-1038.5238774	#N/A	#N/A	#N/A	#N/A	#N/A	#N/A
20	rc_rG_188	-1038.7337440	0.2103121	-1038.5234318	#N/A	#N/A	#N/A	#N/A	#N/A	#N/A
1	rc_1mG_01	-581.7517571	0.1099450	-581.6418120	-581.6395407	-0.1122163	-580.6385744	-580.8067273	-578.3684590	-578.4806753
2	rc_1mG_02	-581.7489372	0.1097621	-581.6391750	-581.6372902	-0.1116469	-580.6356446	-580.8038493	-578.3672642	-578.4789112
3	rc_1mG_06	-581.7349912	0.1085872	-581.6264040	-581.6044976	-0.1304937	-580.6061140	-580.7743786	-578.3377893	-578.4682829
4	rc_1mG_03	-581.7225057	0.1085136	-581.6139920	-581.6038556	-0.1186501	-580.6028382	-580.7710746	-578.3356136	-578.4542637
5	rc_1mG_04	-581.7172237	0.1103496	-581.6068740	-581.5856082	-0.1316155	-580.5854967	-580.7536352	-578.3185471	-578.4501626
6	rc_1mG_05	-581.7090024	0.1091434	-581.5998590	-581.5532916	-0.1557108	#N/A	#N/A	#N/A	#N/A
1	rc_X03	-562.2948008	0.0707997	-562.2240010	-562.1598864	-0.1349144	-561.2315551	-561.3946924	-559.1258990	-559.2608134
2	rc_X01	-562.2940092	0.0705841	-562.2234250	-562.1722571	-0.1217521	-561.2439229	-561.4068225	-559.1540714	-559.2758234
3	rc_X02	-562.2931129	0.0704668	-562.2226460	-562.1602180	-0.1328949	-561.2331728	-561.3961720	-559.1387878	-559.2716826
4	rc_X18	-562.2861914	0.0699154	-562.2162760	-562.1670854	-0.1191060	-561.2405650	-561.4032957	-559.1471449	-559.2662509
5	rc_X19	-562.2836620	0.0696420	-562.2140200	-562.1560258	-0.1276362	-561.2302351	-561.3930045	-559.1357254	-559.2633616
6	rc_X15	-562.2828401	0.0695131	-562.2133270	-562.1643691	-0.1184710	#N/A	#N/A	#N/A	#N/A
7	rc_X20	-562.2823534	0.0716034	-562.2107500	-562.1601656	-0.1221878	#N/A	#N/A	#N/A	#N/A
8	rc_X14	-562.2801067	0.0698167	-562.2102900	-562.1505948	-0.1295120	#N/A	#N/A	#N/A	#N/A
9	rc_X17	-562.2811207	0.0710517	-562.2100690	-562.1516134	-0.1295073	#N/A	#N/A	#N/A	#N/A
10	rc_X13	-562.2757376	0.0691786	-562.2065590	-562.1340507	-0.1416869	#N/A	#N/A	#N/A	#N/A
11	rc_X11	-562.2767458	0.0702408	-562.2065050	-562.1545234	-0.1222224	#N/A	#N/A	#N/A	#N/A
12	rc_X22	-562.2759929	0.0702229	-562.2057700	-562.1464409	-0.1295520	#N/A	#N/A	#N/A	#N/A
13	rc_X16	-562.2760075	0.0708245	-562.2051830	-562.1377042	-0.1383034	#N/A	#N/A	#N/A	#N/A
14	rc_X21	-562.2741159	0.0701789	-562.2039370	-562.1400816	-0.1340343	#N/A	#N/A	#N/A	#N/A
15	rc_X12	-562.2732824	0.0695894	-562.2036930	-562.1356434	-0.1376391	#N/A	#N/A	#N/A	#N/A

16	rc_X05	-562.2728939	0.0706638	-562.2022300	-562.1513198	-0.1215741	#N/A	#N/A	#N/A	#N/A
17	rc_X09	-562.2717279	0.0697618	-562.2019660	-562.1484526	-0.1232753	#N/A	#N/A	#N/A	#N/A
18	rc_X27	-562.2711224	0.0704493	-562.2006730	-562.1560998	-0.1150225	#N/A	#N/A	#N/A	#N/A
19	rc_X06	-562.2713029	0.0706458	-562.2006570	-562.1402823	-0.1310205	#N/A	#N/A	#N/A	#N/A
20	rc_X28	-562.2707648	0.0705607	-562.2002040	-562.1553620	-0.1154027	#N/A	#N/A	#N/A	#N/A
1	rc_bixanthine_13	-1123.5482379	0.1424585	-1123.4057794	#N/A	#N/A	#N/A	#N/A	#N/A	#N/A
2	rc_bixanthine_23	-1123.5379148	0.1416979	-1123.3962169	#N/A	#N/A	#N/A	#N/A	#N/A	#N/A
3	rc_bixanthine_25	-1123.5325295	0.1404221	-1123.3921073	#N/A	#N/A	#N/A	#N/A	#N/A	#N/A
4	rc_bixanthine_2	-1123.5307933	0.1418017	-1123.3889915	#N/A	#N/A	#N/A	#N/A	#N/A	#N/A
5	rc_bixanthine_3	-1123.5172306	0.1424365	-1123.3747940	#N/A	#N/A	#N/A	#N/A	#N/A	#N/A
6	rc_bixanthine_14cc	-1123.5058672	0.1412697	-1123.3645974	#N/A	#N/A	#N/A	#N/A	#N/A	#N/A
7	rc_bixanthine_8	-1123.5035533	0.1408636	-1123.3626897	#N/A	#N/A	#N/A	#N/A	#N/A	#N/A
8	rc_bixanthine_16	-1123.5034194	0.1404420	-1123.3629773	#N/A	#N/A	#N/A	#N/A	#N/A	#N/A
9	rc_bixanthine_17	-1123.5025076	0.1401752	-1123.3623324	#N/A	#N/A	#N/A	#N/A	#N/A	#N/A
10	rc_bixanthine_15	-1123.5012579	0.1399982	-1123.3612596	#N/A	#N/A	#N/A	#N/A	#N/A	#N/A
11	rc_bixanthine_18	-1123.4981471	0.1396625	-1123.3584845	#N/A	#N/A	#N/A	#N/A	#N/A	#N/A
12	rc_bixanthine_10	-1123.4850068	0.1417556	-1123.3432511	#N/A	#N/A	#N/A	#N/A	#N/A	#N/A
13	rc_bixanthine_11	-1123.4808188	0.1411699	-1123.3396489	#N/A	#N/A	#N/A	#N/A	#N/A	#N/A
14	rc_bixanthine_9	-1123.4740442	0.1415907	-1123.3324534	#N/A	#N/A	#N/A	#N/A	#N/A	#N/A
15	rc_bixanthine_12	-1123.4674173	0.1398867	-1123.3275305	#N/A	#N/A	#N/A	#N/A	#N/A	#N/A
16	rc_bixanthine_5	-1123.4338164	0.1442177	-1123.2895986	#N/A	#N/A	#N/A	#N/A	#N/A	#N/A
1	rc_hX_a	-487.0348379	0.0673019	-486.9675360	-486.9169382	-0.1178998	-486.0857799	-486.2244839	-484.2265996	-484.3444994
2	rc_hX_b	-487.0316179	0.0677699	-486.9638480	-486.9117414	-0.1198765	-486.0803754	-486.2191052	-484.2242101	-484.3440866
3	rc_hX_h	-487.0131897	0.0667857	-486.9464040	-486.9003939	-0.1127959	-486.0703901	-486.2091729	-484.2246779	-484.3374738
4	rc_hX_g	-487.0115789	0.0666999	-486.9448790	-486.8948801	-0.1166989	-486.0654185	-486.2043547	-484.2186644	-484.3353632
5	rc_hX_c	-487.0082538	0.0657708	-486.9424830	-486.8886393	-0.1196145	-486.0594164	-486.1982191	-484.2105215	-484.3301360
6	rc_hX_e	-487.0056923	0.0664213	-486.9392710	-486.8809530	-0.1247393	#N/A	#N/A	#N/A	#N/A
7	rc_hX_d	-487.0054743	0.0664233	-486.9390510	-486.8709302	-0.1345441	#N/A	#N/A	#N/A	#N/A
8	rc_hX_f	-487.0038562	0.0656952	-486.9381610	-486.8685505	-0.1353058	#N/A	#N/A	#N/A	#N/A
1	rc_caffeine	-680.2409969	0.1496159	-680.0913810	-680.1452242	-0.0957727	-678.9724279	-679.1694875	-676.2995722	-676.3953449
1	rc_PhOH	-307.2937355	0.0751005	-307.2186350	-307.1966551	-0.0970804	-306.6254007	-306.7117012	-305.3908398	-305.4879202
1	rc_1mInd	-402.9825693	0.1244263	-402.8581430	-402.9014650	-0.0811043	-402.1074859	-402.2192774	-400.3806431	-400.4617474
1	rc_Ind	-363.6650931	0.0985081	-363.5665850	-363.5781214	-0.0869717	-362.8645182	-362.9649563	-361.3316147	-361.4185864
1	rc_tAn	-669.5802754	0.0964483	-669.4838270	-669.4997106	-0.0805647	-668.4903191	-668.5840740	-667.0977601	-667.1783249
1	rc_4mAnil	-326.7857608	0.1124787	-326.6732820	-326.6967971	-0.0889637	-326.0508490	-326.1427494	-324.6367075	-324.7256712
1	rc_An	-346.5975728	0.1008988	-346.4966740	-346.5131085	-0.0844643	-345.8577825	-345.9552251	-344.4311360	-344.5156003
1	rc_Napht	-385.7239079	0.1145878	-385.6093200	-385.6435342	-0.0803736	-384.8587908	-384.9639033	-383.2110389	-383.2914125
1	d_A	-466.9159901	0.0678541	-466.8481360	-466.8121046	-0.1038855	-465.9828722	-466.1254579	-464.1092147	-464.2131002
1	d_5mU4	-453.7333786	0.0686916	-453.6646870	-453.6332324	-0.1001463	-452.8569872	-452.9989575	-451.1024901	-451.2026364
2	d_5mU1	-453.7306761	0.0688970	-453.6617790	-453.6115417	-0.1191344	-452.8367360	-452.9798481	-451.0800468	-451.1991812
3	d_5mU3	-453.7164863	0.0682612	-453.6482250	-453.6113031	-0.1051832	-452.8397426	-452.9816231	-451.0818623	-451.1870455
4	d_5mU2	-453.7164586	0.0687885	-453.6476700	-453.6089933	-0.1074652	#N/A	#N/A	#N/A	#N/A
5	d_5mU5	-453.7131727	0.0681156	-453.6450570	-453.5994269	-0.1137458	#N/A	#N/A	#N/A	#N/A
6	d_5mU6	-453.7115585	0.0682264	-453.6433320	-453.5877491	-0.1238094	#N/A	#N/A	#N/A	#N/A
1	d_G14_02	-542.1717008	0.0704208	-542.1012800	-542.0519171	-0.1197837	-541.1214373	-541.2900108	-539.0127427	-539.1325264
2	d_G14_17	-542.1717040	0.0704670	-542.1012370	-542.0462350	-0.1254690	-541.1164264	-541.2845602	-539.0085759	-539.1340449
3	d_G16_03	-542.1719813	0.0708913	-542.1010900	-542.0493115	-0.1226698	-541.1208691	-541.2885321	-539.0104198	-539.1330896
4	d_G14_16	-542.1671952	0.0701822	-542.0970130	-542.0366268	-0.1305684	-541.1075740	-541.2756640	-538.9979442	-539.1285126

5	d_G14_06	-542.1593587	0.0700466	-542.0893120	-542.0531910	-0.1061676	-541.1246712	-541.2920233	-539.0175544	-539.1237221
6	d_G15_06	-542.1594213	0.0704963	-542.0889250	-542.0535719	-0.1058494	#N/A	#N/A	#N/A	#N/A
7	d_G13_10	-542.1584412	0.0702581	-542.0881830	-542.0480748	-0.1103664	#N/A	#N/A	#N/A	#N/A
8	d_G16_13	-542.1580474	0.0699764	-542.0880710	-542.0512415	-0.1068059	#N/A	#N/A	#N/A	#N/A
9	d_G14_19	-542.1514032	0.0701961	-542.0812070	-542.0346684	-0.1167348	#N/A	#N/A	#N/A	#N/A
10	d_G13_19	-542.1487875	0.0702335	-542.0785540	-542.0213491	-0.1274384	#N/A	#N/A	#N/A	#N/A
11	d_G16_17	-542.1443493	0.0723442	-542.0720050	-541.9729824	-0.1713669	#N/A	#N/A	#N/A	#N/A
12	d_G15_11	-542.1396472	0.0702842	-542.0693630	-542.0388532	-0.1007941	#N/A	#N/A	#N/A	#N/A
13	d_G14_11	-542.1376414	0.0700563	-542.0675850	-542.0299739	-0.1076675	#N/A	#N/A	#N/A	#N/A
14	d_G13_07	-542.1314278	0.0696438	-542.0617840	-542.0154778	-0.1159500	#N/A	#N/A	#N/A	#N/A
15	d_G14_07	-542.1314916	0.0702846	-542.0612070	-542.0148670	-0.1166246	#N/A	#N/A	#N/A	#N/A
16	d_G13_12	-542.1301380	0.0696880	-542.0604500	-542.0112467	-0.1188913	#N/A	#N/A	#N/A	#N/A
17	d_G13_08	-542.1275238	0.0695497	-542.0579740	-542.0041041	-0.1234197	#N/A	#N/A	#N/A	#N/A
18	d_G14_08	-542.1272351	0.0694891	-542.0577460	-542.0014517	-0.1257835	#N/A	#N/A	#N/A	#N/A
19	d_G13_13	-542.1250305	0.0694754	-542.0555550	-541.9879397	-0.1370907	#N/A	#N/A	#N/A	#N/A
20	d_G14_13	-542.1247708	0.0693617	-542.0554090	-541.9866935	-0.1380773	#N/A	#N/A	#N/A	#N/A
1	d_C56	-394.5257074	0.0549713	-394.4707360	-394.4025034	-0.1232040	-393.7164862	-393.8412510	-392.1707716	-392.2939756
2	d_C46	-394.5173848	0.0552317	-394.4621530	-394.4157529	-0.1016318	-393.7300211	-393.8545537	-392.1858791	-392.2875109
3	d_C45	-394.5158890	0.0550640	-394.4608250	-394.4124774	-0.1034116	-393.7271729	-393.8516898	-392.1824811	-392.2858927
4	d_C15	-394.5127191	0.0549100	-394.4578090	-394.4006206	-0.1120985	-393.7155656	-393.8408730	-392.1687481	-392.2808466
5	d_C16	-394.5114774	0.0547723	-394.4567050	-394.3906995	-0.1207779	-393.7065058	-393.8322320	-392.1585783	-392.2793561
6	d_C35	-394.4961751	0.0547540	-394.4414210	-394.3965447	-0.0996304	-393.7153657	-393.8394010	-392.1674254	-392.2670558
7	d_C25	-394.4959636	0.0547136	-394.4412500	-394.3954641	-0.1004995	-393.7143079	-393.8384695	-392.1661011	-392.2666007
8	d_C36	-394.4956621	0.0549160	-394.4407460	-394.3911243	-0.1045378	#N/A	#N/A	#N/A	#N/A
9	d_C26	-394.4953432	0.0546781	-394.4406650	-394.3885695	-0.1067737	#N/A	#N/A	#N/A	#N/A
1	d_U4	-414.4103597	0.0431767	-414.3671830	-414.3105298	-0.0998299	-413.6142037	-413.7449778	-412.0544424	-412.1542723
2	d_U1	-414.4070022	0.0430492	-414.3639530	-414.2868952	-0.1201071	-413.5916816	-413.7236745	-412.0289336	-412.1490406
3	d_U3	-414.3929288	0.0427988	-414.3501300	-414.2866781	-0.1062508	-413.5947840	-413.7255425	-412.0314562	-412.1377070
4	d_U2	-414.3927665	0.0426854	-414.3500810	-414.2842230	-0.1085435	-413.5925332	-413.7233757	-412.0287987	-412.1373422
5	d_U5	-414.3906033	0.0426403	-414.3479630	-414.2779879	-0.1126154	-413.5852160	-413.7157861	-412.0204691	-412.1330845
6	d_U6	-414.3891872	0.0427362	-414.3464510	-414.2670169	-0.1221703	-413.5753372	-413.7061075	-412.0095053	-412.1316757
1	d_1mG43	-581.4795067	0.0975686	-581.3819380	-581.3606212	-0.1188855	-580.3533457	-580.5315622	-578.0454646	-578.1643501
2	d_1mG62	-581.4669421	0.0964930	-581.3704490	-581.3639667	-0.1029754	-580.3564982	-580.5346478	-578.0519151	-578.1548905
3	d_1mG61	-581.4663745	0.0964995	-581.3698750	-581.3629536	-0.1034209	-580.3555811	-580.5336896	-578.0515420	-578.1549629
4	d_1mG11	-581.4645608	0.0967088	-581.3678520	-581.3596307	-0.1049301	-580.3500479	-580.5282430	-578.0456863	-578.1506164
5	d_1mG12	-581.4625255	0.0966114	-581.3659140	-581.3498035	-0.1127220	-580.3411676	-580.5196262	-578.0348862	-578.1476081
6	d_1mG21	-581.4616079	0.0965128	-581.3650950	-581.3561890	-0.1054189	#N/A	#N/A	#N/A	#N/A
7	d_1mG22	-581.4593999	0.0964969	-581.3629030	-581.3440147	-0.1153853	#N/A	#N/A	#N/A	#N/A
8	d_1mG31	-581.4360496	0.0962106	-581.3398390	-581.3318519	-0.1041978	#N/A	#N/A	#N/A	#N/A
9	d_1mG41	-581.4316539	0.0961428	-581.3355110	-581.3163842	-0.1152697	#N/A	#N/A	#N/A	#N/A
10	d_1mG42	-581.4284998	0.0956807	-581.3328190	-581.2992295	-0.1292703	#N/A	#N/A	#N/A	#N/A
1	d_X11	-562.0559995	0.0588765	-561.9971230	-561.9427795	-0.1132200	-561.0025714	-561.1765972	-558.8796808	-558.9929008
2	d_X16	-562.0540303	0.0585482	-561.9954820	-561.9534672	-0.1005630	-561.0148449	-561.1883768	-558.8922519	-558.9928150
3	d_X05	-562.0534011	0.0585251	-561.9948760	-561.9372896	-0.1161115	-560.9956784	-561.1704432	-558.8719735	-558.9880850
4	d_X02	-562.0485823	0.0582812	-561.9903010	-561.9250247	-0.1235576	-560.9838901	-561.1593457	-558.8581492	-558.9817068
5	d_X08	-562.0435994	0.0579544	-561.9856450	-561.9048306	-0.1387688	-560.9648822	-561.1401800	-558.8390015	-558.9777703
6	d_X04	-562.0384317	0.0582627	-561.9801690	-561.9214329	-0.1169988	#N/A	#N/A	#N/A	#N/A
7	d_X03	-562.0382712	0.0582342	-561.9800370	-561.9198950	-0.1183762	#N/A	#N/A	#N/A	#N/A

8	d_X10	-562.0380328	0.0583348	-561.9796980	-561.9170495	-0.1209834	#N/A	#N/A	#N/A	#N/A
9	d_X09	-562.0375198	0.0583567	-561.9791630	-561.9130574	-0.1244623	#N/A	#N/A	#N/A	#N/A
10	d_X18	-562.0357292	0.0582371	-561.9774920	-561.9312875	-0.1044417	#N/A	#N/A	#N/A	#N/A
11	d_X17	-562.0350207	0.0580207	-561.9770000	-561.9312310	-0.1037897	#N/A	#N/A	#N/A	#N/A
12	d_X20	-562.0350777	0.0582226	-561.9768550	-561.9227791	-0.1122985	#N/A	#N/A	#N/A	#N/A
13	d_X13	-562.0322889	0.0582279	-561.9740610	-561.9075872	-0.1247017	#N/A	#N/A	#N/A	#N/A
14	d_X23	-562.0317159	0.0585719	-561.9731440	-561.9087473	-0.1229686	#N/A	#N/A	#N/A	#N/A
15	d_X12	-562.0310217	0.0582036	-561.9728180	-561.9032606	-0.1277611	#N/A	#N/A	#N/A	#N/A
16	d_X15	-562.0297454	0.0578574	-561.9718880	-561.9120105	-0.1177349	#N/A	#N/A	#N/A	#N/A
17	d_X07	-562.0290951	0.0577711	-561.9713240	-561.8956696	-0.1334256	#N/A	#N/A	#N/A	#N/A
18	d_X01	-562.0276825	0.0583844	-561.9692980	-561.8595411	-0.1681414	#N/A	#N/A	#N/A	#N/A
19	d_X06	-562.0266333	0.0576362	-561.9689970	-561.8796199	-0.1470133	#N/A	#N/A	#N/A	#N/A
20	d_X14	-562.0261945	0.0573484	-561.9688460	-561.8960647	-0.1301297	#N/A	#N/A	#N/A	#N/A
1	d_hX12_e	-486.7928392	0.0562121	-486.7366270	-486.6819000	-0.1109392	-485.8428370	-485.9917244	-483.9489489	-484.0598880
2	d_hX13_b	-486.7908291	0.0560631	-486.7347660	-486.6766012	-0.1142279	-485.8350972	-485.9851088	-483.9440591	-484.0582870
3	d_hX13_a	-486.7901879	0.0558858	-486.7343020	-486.6706243	-0.1195636	-485.8299179	-485.9793793	-483.9392194	-484.0587830
4	d_hX13_s	-486.7858947	0.0555707	-486.7303240	-486.6679661	-0.1179287	-485.8282139	-485.9775605	-483.9343812	-484.0523098
5	d_hX12_f	-486.7786618	0.0554447	-486.7232170	-486.6759730	-0.1026888	-485.8403410	-485.9886819	-483.9474418	-484.0501305
6	d_hX11_g	-486.7782645	0.0554515	-486.7228130	-486.6784247	-0.0998398	#N/A	#N/A	#N/A	#N/A
1	d_PhOH	-307.0336943	0.0622102	-306.9714840	-306.9330421	-0.1006522	-306.3515751	-306.4470803	-305.0760297	-305.1766819
1	d_Ind	-363.3703232	0.0859862	-363.2843370	-363.2833345	-0.0869888	-362.5689868	-362.6766245	-360.9970721	-361.0840608
1	rd_A	-466.7274178	0.0664587	-466.6609590	-466.6936178	-0.0338000	-465.8679623	-466.0033316	-464.0243434	-464.0581434
1	rd_5mU4	-453.5408903	0.0669392	-453.4739510	-453.5154635	-0.0254268	-452.7475640	-452.8818913	-451.0124199	-451.0378467
2	rd_5mU1	-453.5240783	0.0669953	-453.4570830	-453.4730817	-0.0509966	-452.6984516	-452.8344834	-450.9540176	-451.0050142
3	rd_5mU5	-453.5210265	0.0668285	-453.4541980	-453.4909669	-0.0300596	-452.7253054	-452.8592882	-450.9768894	-451.0069491
4	rd_5mU3	-453.5196954	0.0666424	-453.4530530	-453.4929165	-0.0267790	-452.7272487	-452.8613830	-450.9808869	-451.0076659
5	rd_5mU2	-453.5192492	0.0670442	-453.4522050	-453.4904099	-0.0288393	-452.7247011	-452.8589307	-450.9762139	-451.0050532
6	rd_5mU6	-453.5184110	0.0666179	-453.4517930	-453.4782854	-0.0401256	#N/A	#N/A	#N/A	#N/A
1	rd_G16_06	-541.9932421	0.0699140	-541.9233280	-541.9423834	-0.0508587	-541.0185443	-541.1785142	-538.9412242	-538.9920829
2	rd_G15_01	-541.9899580	0.0697490	-541.9202090	-541.9400263	-0.0499317	-541.0146540	-541.1748123	-538.9224193	-538.9723510
3	rd_G15_13	-541.9888673	0.0702072	-541.9186600	-541.9438138	-0.0450534	-541.0178073	-541.1781415	-538.9264722	-538.9715256
4	rd_G14_16	-541.9815617	0.0692017	-541.9123600	-541.9253498	-0.0562119	-541.0023741	-541.1625955	-538.9206581	-538.9768700
5	rd_G14_03	-541.9790317	0.0702356	-541.9087960	-541.9394969	-0.0395348	-541.0166954	-541.1762883	-538.9415531	-538.9810879
6	rd_G15_06	-541.9785955	0.0703125	-541.9082830	-541.9382157	-0.0403798	#N/A	#N/A	#N/A	#N/A
7	rd_G15_09	-541.9780845	0.0698144	-541.9082700	-541.9403789	-0.0377056	#N/A	#N/A	#N/A	#N/A
8	rd_G14_06	-541.9778348	0.0698397	-541.9079950	-541.9360787	-0.0417561	#N/A	#N/A	#N/A	#N/A
9	rd_G14_19	-541.9695364	0.0697053	-541.8998310	-541.9251678	-0.0443686	#N/A	#N/A	#N/A	#N/A
10	rd_G13_07	-541.9691442	0.0697051	-541.8994390	-541.9300065	-0.0391377	#N/A	#N/A	#N/A	#N/A
11	rd_G14_07	-541.9693680	0.0700509	-541.8993170	-541.9302880	-0.0390800	#N/A	#N/A	#N/A	#N/A
12	rd_G13_12	-541.9677751	0.0695901	-541.8981850	-541.9262328	-0.0415423	#N/A	#N/A	#N/A	#N/A
13	rd_G14_12	-541.9682171	0.0702790	-541.8979380	-541.9279368	-0.0402803	#N/A	#N/A	#N/A	#N/A
14	rd_G13_08	-541.9671488	0.0696277	-541.8975210	-541.9256371	-0.0415117	#N/A	#N/A	#N/A	#N/A
15	rd_G14_08	-541.9672013	0.0698262	-541.8973750	-541.9236473	-0.0435539	#N/A	#N/A	#N/A	#N/A
16	rd_G13_19	-541.9661126	0.0692935	-541.8968190	-541.9111452	-0.0549674	#N/A	#N/A	#N/A	#N/A
17	rd_G13_13	-541.9645519	0.0693109	-541.8952410	-541.9096534	-0.0548985	#N/A	#N/A	#N/A	#N/A
18	rd_G14_13	-541.9646494	0.0695333	-541.8951160	-541.9091705	-0.0554789	#N/A	#N/A	#N/A	#N/A
19	rd_G15_11	-541.9643834	0.0697773	-541.8946060	-541.9305042	-0.0338792	#N/A	#N/A	#N/A	#N/A
20	rd_G14_11	-541.9618190	0.0694700	-541.8923490	-541.9198089	-0.0420101	#N/A	#N/A	#N/A	#N/A

1	rd_C	-394.3345963	0.0535293	-394.2810670	-394.2962187	-0.0383777	-393.6191405	-393.7355220	-392.0806179	-392.1189955
2	rd_C46	-394.3342561	0.0537431	-394.2805130	-394.3039170	-0.0303392	-393.6277156	-393.7441643	-392.0957316	-392.1260708
3	rd_C45	-394.3334694	0.0539554	-394.2795140	-394.3026643	-0.0308051	-393.6264765	-393.7428201	-392.0955577	-392.1263628
4	rd_C15	-394.3243184	0.0537303	-394.2705880	-394.2913748	-0.0329435	-393.6125116	-393.7286056	-392.0890373	-392.1219808
5	rd_C16	-394.3230828	0.0534497	-394.2696330	-394.2826053	-0.0404775	-393.6047803	-393.7209684	-392.0779860	-392.1184635
6	rd_C35	-394.3207799	0.0536549	-394.2671250	-394.2985980	-0.0221819	-393.6249742	-393.7408151	-392.0952142	-392.1173961
7	rd_C25	-394.3205001	0.0534081	-394.2670920	-394.2985192	-0.0219810	-393.6247083	-393.7406039	-392.0956031	-392.1175841
8	rd_C36	-394.3200042	0.0537031	-394.2663010	-394.2920365	-0.0279677	#N/A	#N/A	#N/A	#N/A
9	rd_C26	-394.3196568	0.0535887	-394.2660680	-394.2905055	-0.0291512	#N/A	#N/A	#N/A	#N/A
1	rd_U4	-414.2081492	0.0412522	-414.1668970	-414.1834247	-0.0247246	-413.4977262	-413.6202877	-411.9548544	-411.9795790
2	rd_U1	-414.1898590	0.0412769	-414.1485820	-414.1380876	-0.0517713	-413.4487717	-413.5685071	-411.8949577	-411.9467290
3	rd_U5	-414.1890463	0.0410053	-414.1480410	-414.1606407	-0.0284057	-413.4784631	-413.6007144	-411.9219915	-411.9503972
4	rd_U3	-414.1875587	0.0409797	-414.1465790	-414.1618249	-0.0257338	-413.4787724	-413.6011399	-411.9245956	-411.9503294
5	rd_U6	-414.1871005	0.0406495	-414.1464510	-414.1495611	-0.0375394	-413.4682226	-413.5905827	-411.9105210	-411.9480604
6	rd_U2	-414.1868573	0.0408383	-414.1460190	-414.1595111	-0.0273462	-413.4762861	-413.5987746	-411.9210050	-411.9483512
1	rd_X11	-561.8665609	0.0582428	-561.8083180	-561.8254361	-0.0411248	-560.8934298	-561.0593328	-558.7892751	-558.8303999
2	rd_X05	-561.8629284	0.0577924	-561.8051360	-561.8217048	-0.0412236	-560.8897262	-561.0557720	-558.7814657	-558.8226894
3	rd_X16	-561.8605254	0.0573814	-561.8031440	-561.8264712	-0.0340542	-560.8958580	-561.0615651	-558.8017107	-558.8357649
4	rd_X02	-561.8451960	0.0574559	-561.7877400	-561.7880656	-0.0571304	-560.8512981	-561.0186302	-558.7491144	-558.8062448
5	rd_X20	-561.8444259	0.0566858	-561.7877400	-561.8049912	-0.0394346	-560.8763677	-561.0421601	-558.7785369	-558.8179715
6	rd_X10	-561.8443501	0.0576360	-561.7867140	-561.7994874	-0.0448626	#N/A	#N/A	#N/A	#N/A
7	rd_X13	-561.8441821	0.0576570	-561.7865250	-561.7997237	-0.0444584	#N/A	#N/A	#N/A	#N/A
8	rd_X09	-561.8432933	0.0574012	-561.7858920	-561.7949971	-0.0482962	#N/A	#N/A	#N/A	#N/A
9	rd_X08	-561.8422253	0.0570633	-561.7851620	-561.7672901	-0.0749352	#N/A	#N/A	#N/A	#N/A
10	rd_X04	-561.8426341	0.0576470	-561.7849870	-561.8002351	-0.0423989	#N/A	#N/A	#N/A	#N/A
11	rd_X12	-561.8425846	0.0578156	-561.7847690	-561.7949580	-0.0476266	#N/A	#N/A	#N/A	#N/A
12	rd_X18	-561.8417407	0.0571436	-561.7845970	-561.8059731	-0.0357676	#N/A	#N/A	#N/A	#N/A
13	rd_X03	-561.8419192	0.0574041	-561.7845150	-561.7982510	-0.0436682	#N/A	#N/A	#N/A	#N/A
14	rd_X23	-561.8412812	0.0567732	-561.7845080	-561.7905939	-0.0506873	#N/A	#N/A	#N/A	#N/A
15	rd_X17	-561.8403473	0.0568552	-561.7834920	-561.8034545	-0.0368928	#N/A	#N/A	#N/A	#N/A
16	rd_X07	-561.8407419	0.0573929	-561.7833490	-561.7922848	-0.0484572	#N/A	#N/A	#N/A	#N/A
17	rd_X27	-561.8394721	0.0583320	-561.7811400	-561.7962990	-0.0431731	#N/A	#N/A	#N/A	#N/A
18	rd_X06	-561.8376539	0.0571499	-561.7805040	-561.7753775	-0.0622764	#N/A	#N/A	#N/A	#N/A
19	rd_X01	-561.8370159	0.0572779	-561.7797380	-561.7523975	-0.0846184	#N/A	#N/A	#N/A	#N/A
20	rd_X26	-561.8375935	0.0579664	-561.7796270	-561.7838799	-0.0537136	#N/A	#N/A	#N/A	#N/A
1	rd_hX12_e	-486.5952690	0.0543189	-486.5409500	-486.5583317	-0.0369373	-485.7258465	-485.8672575	-483.8587273	-483.8956645
2	rd_hX13_a	-486.5921629	0.0543898	-486.5377730	-486.5536610	-0.0385018	-485.7203924	-485.8616329	-483.8387639	-483.8772658
3	rd_hX13_b	-486.5906946	0.0548495	-486.5358450	-486.5544358	-0.0362588	-485.7214099	-485.8628363	-483.8380378	-483.8742967
4	rd_hX13_s	-486.5878648	0.0539987	-486.5338660	-486.5465644	-0.0413004	-485.7146399	-485.8560001	-483.8412734	-483.8825738
5	rd_hX12_f	-486.5748861	0.0536381	-486.5212480	-486.5439811	-0.0309050	-485.7134723	-485.8545934	-483.8475070	-483.8784121
6	rd_hX11_g	-486.5738018	0.0534667	-486.5203350	-486.5424628	-0.0313390	#N/A	#N/A	#N/A	#N/A
1	rd_1mG63	-581.3029092	0.0964691	-581.2064400	-581.2573926	-0.0455166	-580.2532386	-580.4243025	-577.9803603	-578.0258769
2	rd_1mG11	-581.2961314	0.0967374	-581.1993940	-581.2614689	-0.0346625	-580.2579997	-580.4287286	-577.9678542	-578.0025168
3	rd_1mG21	-581.2943738	0.0965818	-581.1977920	-581.2623343	-0.0320396	-580.2579080	-580.4287821	-577.9684285	-578.0004681
4	rd_1mG12	-581.2942870	0.0966950	-581.1975920	-581.2531250	-0.0411620	-580.2506025	-580.4213984	-577.9596900	-578.0008520
5	rd_1mG22	-581.2922442	0.0963731	-581.1958710	-581.2513347	-0.0409095	-580.2481411	-580.4190784	-577.9577521	-577.9986616
6	rd_1mG62	-581.2881403	0.0965093	-581.1916310	-581.2524533	-0.0356870	#N/A	#N/A	#N/A	#N/A
7	rd_1mG61	-581.2870460	0.0962640	-581.1907820	-581.2494664	-0.0375797	#N/A	#N/A	#N/A	#N/A

8	rd_1mG31	-581.2708293	0.0961213	-581.1747080	-581.2371967	-0.0336326	#N/A	#N/A	#N/A	#N/A
9	rd_1mG32	-581.2679355	0.0958595	-581.1720760	-581.2226418	-0.0452937	#N/A	#N/A	#N/A	#N/A
10	rd_1mG41	-581.2673112	0.0960462	-581.1712650	-581.2233140	-0.0439973	#N/A	#N/A	#N/A	#N/A
11	rd_1mG42	-581.2630611	0.0961521	-581.1669090	-581.2052463	-0.0578148	#N/A	#N/A	#N/A	#N/A
1	rd_PhOH	-306.8660782	0.0626451	-306.8034330	-306.8559956	-0.0100825	-306.2833793	-306.3714287	-305.0277441	-305.0378266
1	rd_Ind	-363.2089511	0.0851831	-363.1237680	-363.1986358	-0.0103154	-362.5640077	-362.6718786	-360.9930370	-361.0033523
1	h2o	-76.4480650	0.0033390	-76.4447259	#N/A	#N/A	#N/A	#N/A	#N/A	#N/A
1	p_A24	-467.8429121	0.0931370	-467.7497750	-467.7333745	-0.1095375	-466.9136421	-467.0487456	-465.0584338	-465.1679713
2	p_A35	-467.8426938	0.0937427	-467.7489510	-467.7331608	-0.1095330	-466.9121801	-467.0475739	-465.0589506	-465.1684835
3	p_A25	-467.8409367	0.0936806	-467.7472560	-467.7148798	-0.1260569	-466.8950229	-467.0304787	-465.0403914	-465.1664483
4	p_A34	-467.8388061	0.0932330	-467.7455730	-467.7309053	-0.1079008	-466.9106507	-467.0459044	-465.0560878	-465.1639886
5	p_A45	-467.8375547	0.0930357	-467.7445190	-467.7195546	-0.1180001	-466.8986472	-467.0341377	-465.0429646	-465.1609647
6	p_A23	-467.8242061	0.0928740	-467.7313320	-467.6936443	-0.1305618	#N/A	#N/A	#N/A	#N/A
7	p_A14	-467.8202430	0.0939589	-467.7262840	-467.6971865	-0.1230565	#N/A	#N/A	#N/A	#N/A
8	p_A15	-467.8178993	0.0941472	-467.7237520	-467.6710957	-0.1468036	#N/A	#N/A	#N/A	#N/A
9	p_A13	-467.8082943	0.0946842	-467.7136100	-467.6766624	-0.1316319	#N/A	#N/A	#N/A	#N/A
10	p_A12	-467.7986390	0.0945889	-467.7040500	-467.6359749	-0.1626641	#N/A	#N/A	#N/A	#N/A
1	p_5mU146	-454.6328147	0.0950166	-454.5377980	-454.5203833	-0.1124313	-453.7588399	-453.8929758	-452.0157071	-452.1281384
2	p_5mU145	-454.6311296	0.0953105	-454.5358190	-454.5157496	-0.1153799	-453.7543493	-453.8884425	-452.0112974	-452.1266773
3	p_5mU124	-454.6254110	0.0939339	-454.5314770	-454.5053313	-0.1200796	-453.7470754	-453.8811131	-452.0026223	-452.1227019
4	p_5mU134	-454.6254588	0.0940187	-454.5314400	-454.5073874	-0.1180714	-453.7485342	-453.8825590	-452.0041121	-452.1221835
5	p_5mU135	-454.6255261	0.0941281	-454.5313980	-454.5251018	-0.1004243	-453.7665619	-453.9005670	-452.0250294	-452.1254537
6	p_5mU136	-454.6241788	0.0937767	-454.5304020	-454.5172020	-0.1069768	-453.7595809	-453.8937915	-452.0167280	-452.1237048
7	p_5mU125	-454.6226300	0.0941349	-454.5284950	-454.5116699	-0.1109601	-453.7543477	-453.8885112	-452.0115956	-452.1225556
8	p_5mU126	-454.6211458	0.0939247	-454.5272210	-454.5018477	-0.1192981	#N/A	#N/A	#N/A	#N/A
9	p_5mU235	-454.5699995	0.0932964	-454.4767030	-454.4491971	-0.1208024	#N/A	#N/A	#N/A	#N/A
10	p_5mU236	-454.5691780	0.0927789	-454.4763990	-454.4441480	-0.1250300	#N/A	#N/A	#N/A	#N/A
11	p_5mU234	-454.5639853	0.0945432	-454.4694420	-454.4166425	-0.1473427	#N/A	#N/A	#N/A	#N/A
12	p_5mU123	-454.5618789	0.0935928	-454.4682860	-454.4031525	-0.1587264	#N/A	#N/A	#N/A	#N/A
1	p_G3	-543.0945827	0.0952867	-542.9992960	-542.9784992	-0.1160836	-542.0592008	-542.2187791	-539.9660646	-540.0821481
2	p_G2	-543.0924479	0.0958029	-542.9966450	-542.9692919	-0.1231560	-542.0518675	-542.2113167	-539.9593805	-540.0825365
3	p_G1	-543.0873007	0.0956407	-542.9916600	-542.9475609	-0.1397398	-542.0315758	-542.1910381	-539.9366047	-540.0763445
4	p_G4	-543.0817475	0.0953755	-542.9863720	-542.9411016	-0.1406459	-542.0226177	-542.1830400	-539.9257174	-540.0663633
5	p_G6	-543.0789784	0.0945114	-542.9844670	-542.9566096	-0.1223689	-542.0399188	-542.1996995	-539.9451246	-540.0674935
6	p_G5	-543.0789863	0.0955602	-542.9834260	-542.9604551	-0.1185312	#N/A	#N/A	#N/A	#N/A
7	p_G7	-543.0760620	0.0940979	-542.9819640	-542.9506763	-0.1253857	#N/A	#N/A	#N/A	#N/A
1	p_C14	-395.4597642	0.0815132	-395.3782510	-395.3456216	-0.1141426	-394.6718327	-394.7881117	-393.1433971	-393.2575397
2	p_C13	-395.4473667	0.0810766	-395.3662900	-395.3470245	-0.1003422	-394.6760174	-394.7922337	-393.1485650	-393.2489072
3	p_C12	-395.4442925	0.0815705	-395.3627220	-395.3328907	-0.1114018	-394.6630886	-394.7795249	-393.1342036	-393.2456054
4	p_C24	-395.4404063	0.0799873	-395.3604190	-395.3329768	-0.1074295	-394.6610975	-394.7773458	-393.1338368	-393.2412664
5	p_C34	-395.4367556	0.0796145	-395.3571410	-395.3152278	-0.1215277	-394.6447430	-394.7611785	-393.1159043	-393.2374320
6	p_C15	-395.4316662	0.0821901	-395.3494760	-395.2906898	-0.1409764	-394.6195752	-394.7354317	-393.0778900	-393.2188665
7	p_C25	-395.4233011	0.0819590	-395.3413420	-395.2951827	-0.1281183	-394.6278592	-394.7435276	-393.0898227	-393.2179411
8	p_C35	-395.4229105	0.0816074	-395.3413030	-395.2909498	-0.1319607	-394.6241412	-394.7398038	-393.0858285	-393.2177892
9	p_C45	-395.4186044	0.0816014	-395.3370030	-395.2542241	-0.1643804	-394.5856240	-394.7017604	-393.0416946	-393.2060749
10	p_C23	-395.3886697	0.0802247	-395.3084450	-395.2694097	-0.1192601	-394.5994367	-394.7157286	-393.0634568	-393.1827169
1	p_U146	-415.3079988	0.0695017	-415.2384970	-415.1918106	-0.1161882	-414.5117001	-414.6340647	-412.9634719	-413.0796601
2	p_U145	-415.3058070	0.0688209	-415.2369860	-415.1869664	-0.1188406	-414.5070624	-414.6294207	-412.9584916	-413.0773322

3	p_U134	-415.2988622	0.0682932	-415.2305690	-415.1751971	-0.1236651	-414.4981059	-414.6203950	-412.9471557	-413.0708209
4	p_U135	-415.2994697	0.0691707	-415.2302990	-415.1943537	-0.1051160	#N/A	#N/A	#N/A	#N/A
5	p_U124	-415.2986425	0.0688954	-415.2297470	-415.1729901	-0.1256524	#N/A	#N/A	#N/A	#N/A
6	p_U136	-415.2988028	0.0696908	-415.2291120	-415.1872408	-0.1115621	#N/A	#N/A	#N/A	#N/A
7	p_U125	-415.2967033	0.0698803	-415.2268230	-415.1807044	-0.1159989	#N/A	#N/A	#N/A	#N/A
8	p_U126	-415.2958622	0.0701381	-415.2257240	-415.1717171	-0.1241451	#N/A	#N/A	#N/A	#N/A
9	p_U246	-415.2912066	0.0687646	-415.2224420	-415.1824226	-0.1087840	#N/A	#N/A	#N/A	#N/A
10	p_U245	-415.2886241	0.0682150	-415.2204090	-415.1752002	-0.1134239	#N/A	#N/A	#N/A	#N/A
11	p_U346	-415.2877639	0.0685809	-415.2191830	-415.1665888	-0.1211751	#N/A	#N/A	#N/A	#N/A
12	p_U345	-415.2849981	0.0669931	-415.2180050	-415.1574896	-0.1275085	#N/A	#N/A	#N/A	#N/A
13	p_U156	-415.2514342	0.0668602	-415.1845740	-415.1029769	-0.1484573	#N/A	#N/A	#N/A	#N/A
14	p_U235	-415.2439710	0.0682039	-415.1757670	-415.1187867	-0.1251843	#N/A	#N/A	#N/A	#N/A
15	p_U236	-415.2437003	0.0686773	-415.1750230	-415.1144973	-0.1292030	#N/A	#N/A	#N/A	#N/A
16	p_U256	-415.2417091	0.0678971	-415.1738120	-415.1048024	-0.1369068	#N/A	#N/A	#N/A	#N/A
17	p_U234	-415.2370344	0.0687734	-415.1682610	-415.0846368	-0.1523976	#N/A	#N/A	#N/A	#N/A
18	p_U123	-415.2354013	0.0674382	-415.1679630	-415.0724266	-0.1629746	#N/A	#N/A	#N/A	#N/A
19	p_U456	-415.2311547	0.0673877	-415.1637670	-415.0564374	-0.1747173	#N/A	#N/A	#N/A	#N/A
1	p_1mG23	-582.4043847	0.1224207	-582.2819640	-582.2971341	-0.1072506	-581.2975338	-581.4684986	-579.0075345	-579.1147851
2	p_1mG22	-582.4026272	0.1228312	-582.2797960	-582.2893756	-0.1132516	-581.2915631	-581.4623247	-579.0027944	-579.1160460
3	p_1mG12	-582.3972003	0.1227782	-582.2744220	-582.2678165	-0.1293838	-581.2714733	-581.4423741	-578.9801752	-579.1095590
4	p_1mG17	-582.3887902	0.1216692	-582.2671210	-582.2878813	-0.1009089	-581.2917841	-581.4623835	-579.0042003	-579.1051092
5	p_1mG26	-582.3855120	0.1222620	-582.2632500	-582.2678213	-0.1176908	-581.2710591	-581.4420902	-578.9839173	-579.1016081
6	p_1mG16	-582.3833272	0.1224182	-582.2609090	-582.2705042	-0.1128230	#N/A	#N/A	#N/A	#N/A
7	p_1mG25	-582.3816784	0.1215004	-582.2601780	-582.2650064	-0.1166720	#N/A	#N/A	#N/A	#N/A
8	p_1mG21	-582.3816074	0.1243604	-582.2572470	-582.2401198	-0.1414876	#N/A	#N/A	#N/A	#N/A
9	p_1mG11	-582.3802605	0.1242654	-582.2559950	-582.2325416	-0.1477188	#N/A	#N/A	#N/A	#N/A
10	p_1mG15	-582.3514927	0.1223976	-582.2290950	-582.2271823	-0.1243104	#N/A	#N/A	#N/A	#N/A
11	p_1mG24	-582.3510422	0.1234032	-582.2276390	-582.2260491	-0.1249931	#N/A	#N/A	#N/A	#N/A
12	p_1mG27	-582.3504887	0.1228787	-582.2276100	-582.2262661	-0.1242226	#N/A	#N/A	#N/A	#N/A
13	p_1mG13	-582.3487909	0.1230039	-582.2257870	-582.2212113	-0.1275796	#N/A	#N/A	#N/A	#N/A
1	p_X1	-562.9593578	0.0841267	-562.8752310	-562.8270976	-0.1322601	-561.9007919	-562.0666168	-559.7868166	-559.9190767
2	p_X4	-562.9451693	0.0829512	-562.8622180	-562.8184084	-0.1267609	-561.8937664	-562.0594072	-559.7844842	-559.9112451
3	p_X5	-562.9443164	0.0844514	-562.8598650	-562.8201682	-0.1241483	-561.8954730	-562.0610157	-559.7861553	-559.9103036
4	p_X2	-562.9414555	0.0834214	-562.8580340	-562.8166279	-0.1248275	-561.8935963	-562.0589940	-559.7806133	-559.9054409
5	p_X3	-562.9417122	0.0847492	-562.8569630	-562.8174183	-0.1242940	-561.8941829	-562.0595859	-559.7812975	-559.9055915
1	p_hX3	-487.7074682	0.0823001	-487.6251680	-487.5903398	-0.1171284	-486.7615303	-486.9029430	-484.8854947	-485.0026231
2	p_hX2	-487.7033465	0.0821755	-487.6211710	-487.5845847	-0.1187619	-486.7562998	-486.8976837	-484.8783117	-484.9970735
3	p_hX1	-487.6975570	0.0818090	-487.6157480	-487.5637938	-0.1337632	-486.7366365	-486.8780364	-484.8560744	-484.9898377
4	p_hX4	-487.6938919	0.0817868	-487.6121050	-487.5551602	-0.1387317	-486.7273609	-486.8692427	-484.8480581	-484.9867898
1	p_PhOH	-307.9124692	0.0875012	-307.8249680	-307.7924279	-0.1200413	-307.2279466	-307.3162536	-305.9658507	-306.0858920
1	p_1mInd	-403.5974626	0.1394885	-403.4579740	-403.5080056	-0.0894570	-402.7226288	-402.8356664	-400.9775296	-401.0669866
1	p_Ind	-364.2824360	0.1129509	-364.1694850	-364.1844891	-0.0979469	-363.4801671	-363.5818767	-361.9285809	-362.0265278
1	p_tAn	-670.1951820	0.1072820	-670.0879000	-670.1113724	-0.0838096	-669.1085928	-669.2047579	-667.7026699	-667.7864795
1	caffeine	-680.4568848	0.1500887	-680.3067960	-680.4343425	-0.0225423	-679.2623549	-679.4643288	-676.5632230	-676.5857653
1	p_caffeine1	-680.8998831	0.1628646	-680.7370185	-680.7915754	-0.1083077	-679.6220107	-679.8218681	-676.9266799	-677.0349877
2	p_caffeine2	-680.8841039	0.1626296	-680.7214742	-680.7815284	-0.1025754	#N/A	#N/A	#N/A	#N/A
3	p_caffeine3	-680.8827046	0.1613331	-680.7213714	-680.7839233	-0.0987813	#N/A	#N/A	#N/A	#N/A
4	p_caffeine5	-680.8801142	0.1620843	-680.7180298	-680.7814017	-0.0987125	#N/A	#N/A	#N/A	#N/A

5	p_caffeine7	-680.8554333	0.1626274	-680.6928058	-680.7544711	-0.1009622	#N/A	#N/A	#N/A	#N/A
6	p_caffeine6	-680.8542153	0.1623300	-680.6918852	-680.7552329	-0.0989824	#N/A	#N/A	#N/A	#N/A
7	p_caffeine8	-680.8465465	0.1642282	-680.6823182	-680.7317299	-0.1148165	#N/A	#N/A	#N/A	#N/A
1	p_caffeine1_aaw	-757.3594689	0.1809352	-757.1785336	#N/A	#N/A	#N/A	#N/A	#N/A	#N/A
2	p_caffeine1_abl	-757.3554855	0.1823694	-757.1731161	#N/A	#N/A	#N/A	#N/A	#N/A	#N/A
3	p_caffeine1_abf	-757.3547182	0.1823599	-757.1723583	#N/A	#N/A	#N/A	#N/A	#N/A	#N/A
4	p_caffeine1_aap	-757.3548981	0.1832832	-757.1716149	#N/A	#N/A	#N/A	#N/A	#N/A	#N/A
5	p_caffeine1_aau	-757.3546959	0.1824068	-757.1722891	#N/A	#N/A	#N/A	#N/A	#N/A	#N/A
6	p_caffeine1_aaq	-757.3546002	0.1824720	-757.1721281	#N/A	#N/A	#N/A	#N/A	#N/A	#N/A
7	p_caffeine1_aax	-757.3547928	0.1835924	-757.1712004	#N/A	#N/A	#N/A	#N/A	#N/A	#N/A
8	p_caffeine1_abj	-757.3549101	0.1830982	-757.1718119	#N/A	#N/A	#N/A	#N/A	#N/A	#N/A
9	p_caffeine1_aae	-757.3543414	0.1833491	-757.1709922	#N/A	#N/A	#N/A	#N/A	#N/A	#N/A
10	p_caffeine1_abe	-757.3535477	0.1819680	-757.1715796	#N/A	#N/A	#N/A	#N/A	#N/A	#N/A
11	p_caffeine1_abq	-757.3531516	0.1810871	-757.1720644	#N/A	#N/A	#N/A	#N/A	#N/A	#N/A
12	p_caffeine1_abt	-757.3530163	0.1822842	-757.1707320	#N/A	#N/A	#N/A	#N/A	#N/A	#N/A
13	p_caffeine1_aah	-757.3528383	0.1819266	-757.1709117	#N/A	#N/A	#N/A	#N/A	#N/A	#N/A
14	p_caffeine1_aak	-757.3511821	0.1785955	-757.1725866	#N/A	#N/A	#N/A	#N/A	#N/A	#N/A
15	p_caffeine1_aan	-757.3513746	0.1810665	-757.1703080	#N/A	#N/A	#N/A	#N/A	#N/A	#N/A
16	p_caffeine1_aav	-757.3509070	0.1801771	-757.1707298	#N/A	#N/A	#N/A	#N/A	#N/A	#N/A
17	p_caffeine1_abn	-757.3506515	0.1795908	-757.1710607	#N/A	#N/A	#N/A	#N/A	#N/A	#N/A
18	p_caffeine1_abo	-757.3504705	0.1799614	-757.1705090	#N/A	#N/A	#N/A	#N/A	#N/A	#N/A
19	p_caffeine1_aal	-757.3501451	0.1803088	-757.1698362	#N/A	#N/A	#N/A	#N/A	#N/A	#N/A
20	p_caffeine1_abh	-757.2598121	0.1826324	-757.0771797	#N/A	#N/A	#N/A	#N/A	#N/A	#N/A
1	oxocaffeine	-755.7072053	0.1511730	-755.5560323	#N/A	#N/A	#N/A	#N/A	#N/A	#N/A
1	dihydroxyoxocaffeine_cis2_5	-907.3825417	0.1809720	-907.2015696	#N/A	#N/A	#N/A	#N/A	#N/A	#N/A
2	dihydroxyoxocaffeine_cis2_10	-907.3809558	0.1801890	-907.2007668	#N/A	#N/A	#N/A	#N/A	#N/A	#N/A
3	dihydroxyoxocaffeine_cis1_9	-907.3799197	0.1822279	-907.1976918	#N/A	#N/A	#N/A	#N/A	#N/A	#N/A
4	dihydroxyoxocaffeine_cis1_7	-907.3796171	0.1797161	-907.1999010	#N/A	#N/A	#N/A	#N/A	#N/A	#N/A
5	dihydroxyoxocaffeine_cis2_11	-907.3793933	0.1802273	-907.1991660	#N/A	#N/A	#N/A	#N/A	#N/A	#N/A
6	dihydroxyoxocaffeine_cis2_1	-907.3787755	0.1823551	-907.1964203	#N/A	#N/A	#N/A	#N/A	#N/A	#N/A
7	dihydroxyoxocaffeine_cis1_4	-907.3787247	0.1819356	-907.1967890	#N/A	#N/A	#N/A	#N/A	#N/A	#N/A
8	dihydroxyoxocaffeine_cis1_1	-907.3786136	0.1814414	-907.1971721	#N/A	#N/A	#N/A	#N/A	#N/A	#N/A
9	dihydroxyoxocaffeine_cis2_2	-907.3775824	0.1822452	-907.1953372	#N/A	#N/A	#N/A	#N/A	#N/A	#N/A
10	dihydroxyoxocaffeine_cis1_14	-907.3775120	0.1823313	-907.1951806	#N/A	#N/A	#N/A	#N/A	#N/A	#N/A
11	dihydroxyoxocaffeine_cis2_14	-907.3770218	0.1821993	-907.1948224	#N/A	#N/A	#N/A	#N/A	#N/A	#N/A
12	dihydroxyoxocaffeine_cis1_13	-907.3769974	0.1823397	-907.1946577	#N/A	#N/A	#N/A	#N/A	#N/A	#N/A
13	dihydroxyoxocaffeine_trans1_10	-907.3664373	0.1812683	-907.1851689	#N/A	#N/A	#N/A	#N/A	#N/A	#N/A
14	dihydroxyoxocaffeine_trans2_5	-907.3661272	0.1814156	-907.1847116	#N/A	#N/A	#N/A	#N/A	#N/A	#N/A
15	dihydroxyoxocaffeine_trans2_14	-907.3654143	0.1816203	-907.1837939	#N/A	#N/A	#N/A	#N/A	#N/A	#N/A
16	dihydroxyoxocaffeine_trans2_13	-907.3649455	0.1812608	-907.1836847	#N/A	#N/A	#N/A	#N/A	#N/A	#N/A
17	dihydroxyoxocaffeine_trans1_9	-907.3648073	0.1814611	-907.1833462	#N/A	#N/A	#N/A	#N/A	#N/A	#N/A
18	dihydroxyoxocaffeine_trans1_6	-907.3647570	0.1812846	-907.1834723	#N/A	#N/A	#N/A	#N/A	#N/A	#N/A
19	dihydroxyoxocaffeine_trans2_10	-907.3643552	0.1797780	-907.1845771	#N/A	#N/A	#N/A	#N/A	#N/A	#N/A
20	dihydroxyoxocaffeine_trans1_5	-907.3643546	0.1798181	-907.1845365	#N/A	#N/A	#N/A	#N/A	#N/A	#N/A
1	PhOH	-307.5101919	0.0752499	-307.4349420	-307.5004289	-0.0097630	-306.9328416	-307.0230454	-305.6665014	-305.6762644
1	1mInd	-403.1754066	0.1250746	-403.0503320	-403.1698609	-0.0055457	-402.3802379	-402.4955955	-400.6269089	-400.6324546
1	Ind	-363.8623284	0.0991264	-363.7632020	-363.8542039	-0.0081246	-363.1456391	-363.2498549	-361.5852441	-361.5933686
1	tAn	-669.7867258	0.0973737	-669.6893520	-669.7827985	-0.0039273	-668.7784883	-668.8753832	-667.3661385	-667.3700658

Table S2. Energies calculated at the (U)B3LYP-D3 and DLPNO-CCSD(T) level of theory for the systems **with** the addition of explicit water. (Quasi-Harmonic Approximation)

№ conf	Molecule				(U)B3LYP-D3/6-31+G(d,p)//		DLPNO-CCSD(T)/BASIS//			
		SMD/(U)B3LYP-D3/6-31+G(d,p)			SMD/(U)B3LYP-D3/6-31+G(d,p)		SMD/(U)B3LYP-D3/6-31+G(d,p)			
		SMD/DFT			DFT		cc-pVTZ	cc-pVQZ	CBS	
		solution			gas		gas	gas	gas	solution (+Esolv)
		1atm	1atm	1atm	1atm	1atm	1atm	1atm	1atm	1atm
		Etot sol	Gcorr	G298 sol	Etot	Esolv	Etot	Etot	Etot	G298 sol
1	A_01_aaf	-543.8465943	0.0983802	-543.7482140	-543.8104856	-0.0361087	-542.8835486	-543.0484304	-540.7390861	-540.7751948
2	A_01_aae	-543.8466073	0.0985042	-543.7481030	-543.8102082	-0.0363991	-542.8834797	-543.0483143	-540.7389406	-540.7753397
3	A_01_abd	-543.8454389	0.0979199	-543.7475190	-543.8101936	-0.0352453	-542.8834823	-543.0484695	-540.7396429	-540.7748882
4	A_01_aas	-543.8473177	0.1000266	-543.7472910	-543.8155239	-0.0317938	-542.8894146	-543.0535089	-540.7424461	-540.7742399
5	A_01_abg	-543.8445057	0.0972277	-543.7472780	-543.8061379	-0.0383678	-542.8814119	-543.0455832	-540.7376138	-540.7759816
6	A_01_abx	-543.8454501	0.0982690	-543.7471810	-543.8105356	-0.0349145	-542.8834805	-543.0485329	-540.7398033	-540.7747177
7	A_01_aax	-543.8454545	0.0983975	-543.7470570	-543.8101105	-0.0353440	-542.8833066	-543.0483001	-540.7395094	-540.7748535
8	A_01_aan	-543.8445083	0.0975542	-543.7469540	-543.8061694	-0.0383389	#N/A	#N/A	#N/A	#N/A
9	A_01_aab	-543.8445215	0.0977395	-543.7467820	-543.8084022	-0.0361193	#N/A	#N/A	#N/A	#N/A
10	A_01_abh	-543.8444620	0.0977330	-543.7467290	-543.8066020	-0.0378601	#N/A	#N/A	#N/A	#N/A
11	A_01_abb	-543.8445266	0.0978225	-543.7467040	-543.8084188	-0.0361078	#N/A	#N/A	#N/A	#N/A
12	A_02_abl	-543.8449760	0.0985440	-543.7464320	-543.7961434	-0.0488326	-542.8693689	-543.0346618	-540.7239804	-540.7728130
13	A_01_aav	-543.8439841	0.0978520	-543.7461320	-543.8021579	-0.0418262	#N/A	#N/A	#N/A	#N/A
14	A_02_aac	-543.8442633	0.0981373	-543.7461260	-543.7938759	-0.0503875	#N/A	#N/A	#N/A	#N/A
15	A_02_abt	-543.8449979	0.0990539	-543.7459440	-543.7955798	-0.0494181	-542.8693527	-543.0344779	-540.7237244	-540.7731425
16	A_02_aba	-543.8453343	0.0994103	-543.7459240	-543.7992759	-0.0460584	-542.8737169	-543.0378178	-540.7272485	-540.7733070
17	A_02_aax	-543.8442493	0.0984352	-543.7458140	-543.7931477	-0.0511015	#N/A	#N/A	#N/A	#N/A
18	A_01_aah	-543.8423316	0.0966736	-543.7456580	-543.8035130	-0.0388186	#N/A	#N/A	#N/A	#N/A
19	A_02_abu	-543.8435858	0.0981118	-543.7454740	-543.7935010	-0.0500848	#N/A	#N/A	#N/A	#N/A
20	A_01_abp	-543.8425809	0.0971268	-543.7454540	-543.8067149	-0.0358660	#N/A	#N/A	#N/A	#N/A
1	5mUadm	-530.6599072	0.0990372	-530.5608700	-530.6253904	-0.0345169	-529.7560489	-529.9202148	-527.7284340	-527.7629508
2	5mU_aaz	-530.6599452	0.0992472	-530.5606980	-530.6277464	-0.0321988	-529.7591293	-529.9222413	-527.7302387	-527.7624375
3	5mU_aad	-530.6600553	0.0995453	-530.5605100	-530.6246534	-0.0354019	-529.7567691	-529.9204437	-527.7280828	-527.7634847
4	5mU_adb	-530.6599531	0.0994791	-530.5604740	-530.6255966	-0.0343565	-529.7565829	-529.9206161	-527.7287117	-527.7630682
5	5mU_aaa	-530.6597380	0.0993260	-530.5604120	-530.6242211	-0.0355169	-529.7560415	-529.9192645	-527.7265994	-527.7621163
6	5mUadq	-530.6597010	0.0995010	-530.5602000	-530.6228444	-0.0368566	#N/A	#N/A	#N/A	#N/A
7	5mU_abq	-530.6597477	0.0995566	-530.5601910	-530.6249008	-0.0348469	#N/A	#N/A	#N/A	#N/A
8	5mU_adj	-530.6602148	0.1002387	-530.5599760	-530.6256905	-0.0345243	-529.7557020	-529.9199401	-527.7278336	-527.7623579
9	5mUacp	-530.6587593	0.0989193	-530.5598400	-530.6235608	-0.0351985	#N/A	#N/A	#N/A	#N/A
10	5mU_aae	-530.6597229	0.1000809	-530.5596420	-530.6232005	-0.0365224	#N/A	#N/A	#N/A	#N/A
11	5mUacq	-530.6599457	0.1004397	-530.5595060	-530.6277326	-0.0322131	#N/A	#N/A	#N/A	#N/A
12	5mU_abn	-530.6590480	0.0995830	-530.5594650	-530.6256244	-0.0334236	#N/A	#N/A	#N/A	#N/A
13	5mU_aam	-530.6590500	0.0996870	-530.5593630	-530.6254835	-0.0335665	#N/A	#N/A	#N/A	#N/A
14	5mU_aau	-530.6600182	0.1006582	-530.5593600	-530.6262948	-0.0337234	-529.7580054	-529.9217728	-527.7294416	-527.7631650
15	5mUacx	-530.6603178	0.1010077	-530.5593100	-530.6252781	-0.0350397	#N/A	#N/A	#N/A	#N/A
16	5mU_abo	-530.6599978	0.1007388	-530.5592590	-530.6272046	-0.0327933	-529.7583495	-529.9222504	-527.7301538	-527.7629471
17	5mUaac	-530.6603183	0.1010773	-530.5592410	-530.6251923	-0.0351261	#N/A	#N/A	#N/A	#N/A
18	5mUaag	-530.6599919	0.1008538	-530.5591380	-530.6259607	-0.0340312	#N/A	#N/A	#N/A	#N/A
19	5mUabo	-530.6599455	0.1009164	-530.5590290	-530.6276072	-0.0323383	#N/A	#N/A	#N/A	#N/A
20	5mU_act	-530.6589435	0.1000054	-530.5589380	-530.6251427	-0.0338008	#N/A	#N/A	#N/A	#N/A
1	G01_abt	-619.1013834	0.1013493	-619.0000340	-619.0473072	-0.0540762	-618.0228377	-618.2122455	-615.6408777	-615.6949540

2	G02_abw	-619.1005261	0.1006030	-618.9999230	-619.0509198	-0.0496063	-618.0245270	-618.2144169	-615.6437176	-615.6933240
3	G01_aaa	-619.0986974	0.0987943	-618.9999030	-619.0395879	-0.0591095	-618.0162674	-618.2056376	-615.6368911	-615.6960006
4	G02_abt	-619.1030414	0.1033794	-618.9996620	-619.0586175	-0.0444240	-618.0337557	-618.2227024	-615.6513352	-615.6957592
5	G01_aak	-619.1005003	0.1009243	-618.9995760	-619.0460425	-0.0544578	-618.0229080	-618.2113043	-615.6422097	-615.6966676
6	G02_aag	-619.1030431	0.1035711	-618.9994720	-619.0585257	-0.0445174	-618.0337529	-618.2226981	-615.6512392	-615.6957567
7	G01_abg	-619.1019692	0.1025581	-618.9994110	-619.0443505	-0.0576186	-618.0211450	-618.2103189	-615.6398177	-615.6974363
8	G01_aae	-619.1013560	0.1020389	-618.9993170	-619.0472643	-0.0540917	-618.0227955	-618.2121997	-615.6408690	-615.6949607
9	G01_aat	-619.1027418	0.1034278	-618.9993140	-619.0495275	-0.0532144	-618.0241276	-618.2134128	-615.6411763	-615.6943907
10	G01_abr	-619.1013349	0.1020209	-618.9993140	-619.0507293	-0.0506056	-618.0264334	-618.2150532	-615.6454095	-615.6960151
11	G01_abj	-619.1003077	0.1010596	-618.9992480	-619.0439991	-0.0563085	#N/A	#N/A	#N/A	#N/A
12	G01_abn	-619.1017302	0.1025372	-618.9991930	-619.0506209	-0.0511093	-618.0249657	-618.2147654	-615.6459384	-615.6970477
13	G01_aay	-619.0986443	0.0995613	-618.9990830	-619.0414623	-0.0571820	#N/A	#N/A	#N/A	#N/A
14	G01_aal	-619.1010791	0.1020541	-618.9990250	-619.0492835	-0.0517956	#N/A	#N/A	#N/A	#N/A
15	G02_aap	-619.1005714	0.1019323	-618.9986390	-619.0497745	-0.0507969	#N/A	#N/A	#N/A	#N/A
16	G02_aaq	-619.1005441	0.1019470	-618.9985970	-619.0504893	-0.0500547	#N/A	#N/A	#N/A	#N/A
17	G01_aba	-619.0987745	0.1001935	-618.9985810	-619.0434039	-0.0553706	#N/A	#N/A	#N/A	#N/A
18	G01_aaz	-619.1019465	0.1035924	-618.9983540	-619.0424534	-0.0594931	-618.0190907	-618.2084022	-615.6378654	-615.6973584
19	G01_aaf	-619.0940244	0.0956824	-618.9983420	-619.0287153	-0.0653091	#N/A	#N/A	#N/A	#N/A
20	G01_abi	-619.1005116	0.1022495	-618.9982620	-619.0455310	-0.0549806	#N/A	#N/A	#N/A	#N/A
1	C68	-471.4627920	0.0847599	-471.3780320	-471.4191061	-0.0436859	-470.6364206	-470.7832459	-468.8185173	-468.8622032
2	C16	-471.4628362	0.0857212	-471.3771150	-471.4196293	-0.0432069	-470.6369867	-470.7838727	-468.8191891	-468.8623960
3	Caap	-471.4619817	0.0849567	-471.3770250	-471.4154858	-0.0464959	-470.6337342	-470.7808371	-468.8176536	-468.8641495
4	C67	-471.4622126	0.0852025	-471.3770100	-471.4156935	-0.0465191	-470.6343412	-470.7811742	-468.8178258	-468.8643448
5	C18	-471.4598650	0.0828990	-471.3769660	-471.4093494	-0.0505156	-470.6302157	-470.7762044	-468.8118426	-468.8623582
6	Cabf	-471.4627860	0.0860730	-471.3767130	-471.4192133	-0.0435727	-470.6365037	-470.7833640	-468.8186276	-468.8622004
7	Cabb	-471.4627532	0.0861001	-471.3766530	-471.4188884	-0.0438647	-470.6369346	-470.7836222	-468.8186877	-468.8625524
8	C04	-471.4627850	0.0861700	-471.3766150	-471.4193679	-0.0434171	-470.6370695	-470.7838708	-468.8190659	-468.8624830
9	C78	-471.4627794	0.0862654	-471.3765140	-471.4110691	-0.0517103	-470.6304451	-470.7770969	-468.8108995	-468.8626098
10	C62	-471.4622100	0.0859440	-471.3762660	-471.4158669	-0.0463431	#N/A	#N/A	#N/A	#N/A
11	Caba	-471.4598658	0.0836137	-471.3762520	-471.4096443	-0.0502215	#N/A	#N/A	#N/A	#N/A
12	Cabk	-471.4627085	0.0864674	-471.3762410	-471.4179237	-0.0447848	#N/A	#N/A	#N/A	#N/A
13	Cack	-471.4626812	0.0865161	-471.3761650	-471.4177372	-0.0449440	#N/A	#N/A	#N/A	#N/A
14	C08	-471.4626789	0.0866159	-471.3760630	-471.4186930	-0.0439860	-470.6383688	-470.7848469	-468.8208972	-468.8648832
15	C10	-471.4622152	0.0862471	-471.3759680	-471.4155165	-0.0466987	-470.6342248	-470.7810776	-468.8177174	-468.8644161
16	C52	-471.4619904	0.0860744	-471.3759160	-471.4150857	-0.0469047	#N/A	#N/A	#N/A	#N/A
17	Cabc	-471.4619787	0.0861206	-471.3758580	-471.4152547	-0.0467240	#N/A	#N/A	#N/A	#N/A
18	Cacr	-471.4627991	0.0869670	-471.3758320	-471.4107838	-0.0520153	-470.6307532	-470.7775677	-468.8128414	-468.8648566
19	Cadt	-471.4628015	0.0871024	-471.3756990	-471.4121532	-0.0506483	-470.6316533	-470.7781704	-468.8119531	-468.8626015
20	C58	-471.4627998	0.0871597	-471.3756400	-471.4111956	-0.0516042	-470.6312109	-470.7779519	-468.8131967	-468.8648009
1	Uacb	-491.3354328	0.0738727	-491.2615600	-491.3000514	-0.0353813	-490.5112626	-490.6638566	-488.6769522	-488.7123335
2	Uaac	-491.3352650	0.0737700	-491.2614950	-491.2983628	-0.0369022	-490.5113631	-490.6634994	-488.6760886	-488.7129909
3	U114_abc	-491.3356642	0.0742262	-491.2614380	-491.2994727	-0.0361915	-490.5118652	-490.6640480	-488.6767131	-488.7129045
4	U114_abu	-491.3350633	0.0736893	-491.2613740	-491.2975928	-0.0374705	-490.5104551	-490.6620694	-488.6743364	-488.7118069
5	Uacu	-491.3350961	0.0737970	-491.2612990	-491.2990521	-0.0360440	-490.5104041	-490.6631155	-488.6763999	-488.7124439
6	U114_aaf	-491.3356736	0.0744505	-491.2612230	-491.2988574	-0.0368162	-490.5114817	-490.6636143	-488.6761482	-488.7129643
7	U114_acw	-491.3353409	0.0741488	-491.2611920	-491.3021635	-0.0331774	#N/A	#N/A	#N/A	#N/A
8	U114_abt	-491.3351421	0.0740180	-491.2611240	-491.3000442	-0.0350979	#N/A	#N/A	#N/A	#N/A
9	Uadn	-491.3358217	0.0747266	-491.2610950	-491.2955675	-0.0402542	#N/A	#N/A	#N/A	#N/A

10	Uabj	-491.3351118	0.0740308	-491.2610810	-491.2988599	-0.0362519	#N/A	#N/A	#N/A	#N/A
11	Uabg	-491.3353620	0.0742979	-491.2610640	-491.3021891	-0.0331729	#N/A	#N/A	#N/A	#N/A
12	U14_adk	-491.3353408	0.0743347	-491.2610060	-491.3022250	-0.0331158	#N/A	#N/A	#N/A	#N/A
13	U14_abl	-491.3353258	0.0745067	-491.2608190	-491.3020098	-0.0333159	#N/A	#N/A	#N/A	#N/A
14	Uabw	-491.3354589	0.0746698	-491.2607890	-491.3003045	-0.0351544	#N/A	#N/A	#N/A	#N/A
15	Uaab	-491.3354309	0.0746758	-491.2607550	-491.2992792	-0.0361517	#N/A	#N/A	#N/A	#N/A
16	U14_acu	-491.3350853	0.0744013	-491.2606840	-491.2979507	-0.0371346	#N/A	#N/A	#N/A	#N/A
17	Uabe	-491.3353413	0.0748033	-491.2605380	-491.2976887	-0.0376526	#N/A	#N/A	#N/A	#N/A
18	U14_adh	-491.3350604	0.0745884	-491.2604720	-491.2974194	-0.0376410	#N/A	#N/A	#N/A	#N/A
19	U14_abs	-491.3354817	0.0750517	-491.2604300	-491.3004288	-0.0350529	#N/A	#N/A	#N/A	#N/A
20	Uabz	-491.3355279	0.0751089	-491.2604190	-491.3000399	-0.0354880	#N/A	#N/A	#N/A	#N/A
1	1mG_02_aae	-658.4117681	0.1285111	-658.2832570	-658.3729865	-0.0387816	-657.2684013	-657.4683934	-654.6886728	-654.7274544
2	1mG_01_abo	-658.4101375	0.1274375	-658.2827000	-658.3579715	-0.0521660	-657.2532750	-657.4539164	-654.6743314	-654.7264974
3	1mG_02_abh	-658.4118152	0.1296752	-658.2821400	-658.3728830	-0.0389322	-657.2682174	-657.4682103	-654.6885459	-654.7274781
4	1mG_01_aam	-658.4098514	0.1277833	-658.2820680	-658.3610145	-0.0488368	-657.2559012	-657.4567280	-654.6796724	-654.7285093
5	1mG_01_aat	-658.4111790	0.1291380	-658.2820410	-658.3640121	-0.0471669	-657.2585068	-657.4589392	-654.6786626	-654.7258296
6	1mG_01_abm	-658.4098873	0.1279223	-658.2819650	-658.3610403	-0.0488470	-657.2560204	-657.4568607	-654.6796746	-654.7285216
7	1mG_01_abs	-658.4104304	0.1288704	-658.2815600	-658.3557470	-0.0546834	-657.2526079	-657.4528352	-654.6741359	-654.7288193
8	1mG_01_aah	-658.4088797	0.1273916	-658.2814880	-658.3591342	-0.0497454	#N/A	#N/A	#N/A	#N/A
9	1mG_01_aan	-658.4087942	0.1273241	-658.2814700	-658.3581411	-0.0506531	#N/A	#N/A	#N/A	#N/A
10	1mG_01_aaz	-658.4101269	0.1289058	-658.2812210	-658.3620182	-0.0481087	-657.2571319	-657.4578641	-654.6804804	-654.7285891
11	1mG_01_abv	-658.4096906	0.1288635	-658.2808270	-658.3610411	-0.0486495	#N/A	#N/A	#N/A	#N/A
12	1mG_02_aaq	-658.4089008	0.1280937	-658.2808070	-658.3622591	-0.0466417	#N/A	#N/A	#N/A	#N/A
13	1mG_02_abu	-658.4089703	0.1283972	-658.2805730	-658.3631900	-0.0457803	#N/A	#N/A	#N/A	#N/A
14	1mG_02_abf	-658.4085386	0.1281096	-658.2804290	-658.3636913	-0.0448473	#N/A	#N/A	#N/A	#N/A
15	1mG_01_aba	-658.4107498	0.1303237	-658.2804260	-658.3562285	-0.0545213	-657.2530465	-657.4532823	-654.6744920	-654.7290133
16	1mG_01_aab	-658.4112624	0.1310514	-658.2802110	-658.3640488	-0.0472137	-657.2585106	-657.4589752	-654.6786370	-654.7258506
17	1mG_02_aaj	-658.4098604	0.1296593	-658.2802010	-658.3594829	-0.0503774	#N/A	#N/A	#N/A	#N/A
18	1mG_01_abg	-658.4095049	0.1293288	-658.2801760	-658.3578911	-0.0516138	#N/A	#N/A	#N/A	#N/A
19	1mG_02_abv	-658.4078555	0.1279684	-658.2798870	-658.3541901	-0.0536654	#N/A	#N/A	#N/A	#N/A
20	1mG_01_abx	-658.4086707	0.1288046	-658.2798660	-658.3545673	-0.0541034	#N/A	#N/A	#N/A	#N/A
1	X01_abd	-638.9792472	0.0919481	-638.8872990	-638.9449273	-0.0343199	-637.9110381	-638.1059289	-635.5123407	-635.5466606
2	X01_abk	-638.9755166	0.0894485	-638.8860680	-638.9327099	-0.0428067	-637.8988820	-638.0945978	-635.5011971	-635.5440038
3	X01_abx	-638.9753895	0.0893755	-638.8860140	-638.9313569	-0.0440326	-637.8985217	-638.0934713	-635.5011535	-635.5451862
4	X01_aas	-638.9764296	0.0904645	-638.8859650	-638.9329788	-0.0434508	-637.8997856	-638.0951642	-635.5031735	-635.5466243
5	X01_aao	-638.9761150	0.0903189	-638.8857960	-638.9356713	-0.0404437	-637.9030863	-638.0981106	-635.5051906	-635.5456343
6	X01_abl	-638.9748887	0.0894257	-638.8854630	-638.9238040	-0.0510848	#N/A	#N/A	#N/A	#N/A
7	X01_aaa	-638.9756715	0.0903774	-638.8852940	-638.9332795	-0.0423920	-637.9001063	-638.0952479	-635.5029554	-635.5453474
8	X01_aal	-638.9765944	0.0913653	-638.8852290	-638.9289959	-0.0475985	-637.8962813	-638.0917879	-635.4991276	-635.5467261
9	X02_aaq	-638.9730106	0.0882885	-638.8847220	-638.9199457	-0.0530649	#N/A	#N/A	#N/A	#N/A
10	X01_abt	-638.9752229	0.0905229	-638.8847000	-638.9363520	-0.0388709	-637.9034012	-638.0984991	-635.5044595	-635.5433304
11	X01_abe	-638.9749551	0.0904821	-638.8844730	-638.9241265	-0.0508286	#N/A	#N/A	#N/A	#N/A
12	X01_aab	-638.9766682	0.0922021	-638.8844660	-638.9289548	-0.0477134	-637.8959203	-638.0914208	-635.4988703	-635.5465837
13	X01_aax	-638.9766637	0.0922267	-638.8844370	-638.9286245	-0.0480392	-637.8954888	-638.0910325	-635.4985680	-635.5466072
14	X01_abq	-638.9720696	0.0878036	-638.8842660	-638.9204001	-0.0516695	#N/A	#N/A	#N/A	#N/A
15	X01_abr	-638.9726610	0.0884189	-638.8842420	-638.9254284	-0.0472326	#N/A	#N/A	#N/A	#N/A
16	X01_abc	-638.9725802	0.0884042	-638.8841760	-638.9193788	-0.0532014	#N/A	#N/A	#N/A	#N/A
17	X01_aaf	-638.9745831	0.0904411	-638.8841420	-638.9246806	-0.0499025	#N/A	#N/A	#N/A	#N/A

18	X02_aan	-638.9730761	0.0891341	-638.8839420	-638.9170460	-0.0560302	#N/A	#N/A	#N/A	#N/A
19	X02_aas	-638.9730300	0.0891820	-638.8838480	-638.9169811	-0.0560490	#N/A	#N/A	#N/A	#N/A
20	X01_abg	-638.9735979	0.0899898	-638.8836080	-638.9242224	-0.0493755	#N/A	#N/A	#N/A	#N/A
1	hX_a_aaw	-563.7172853	0.0855462	-563.6317390	-563.6689227	-0.0483626	-562.7338365	-562.9052446	-560.5707410	-560.6191036
2	hX_b_aam	-563.7207265	0.0896034	-563.6311230	-563.6840422	-0.0366843	-562.7495600	-562.9199336	-560.5831290	-560.6198133
3	hX_a_aad	-563.7174964	0.0866824	-563.6308140	-563.6694998	-0.0479967	-562.7364008	-562.9066038	-560.5708722	-560.6188689
4	hX_b_aad	-563.7171382	0.0863342	-563.6308040	-563.6704119	-0.0467263	-562.7366055	-562.9072014	-560.5718369	-560.6185632
5	hX_a_aab	-563.7174563	0.0867572	-563.6306990	-563.6694568	-0.0479995	-562.7363670	-562.9065866	-560.5708275	-560.6188270
6	hX_b_abk	-563.7172751	0.0866850	-563.6305900	-563.6737697	-0.0435054	#N/A	#N/A	#N/A	#N/A
7	hX_a_abw	-563.7174264	0.0868804	-563.6305460	-563.6696698	-0.0477567	#N/A	#N/A	#N/A	#N/A
8	hX_a_aba	-563.7182885	0.0877995	-563.6304890	-563.6713762	-0.0469123	-562.7377442	-562.9085832	-560.5732632	-560.6201755
9	hX_b_abr	-563.7175577	0.0870957	-563.6304620	-563.6708642	-0.0466935	-562.7375358	-562.9079894	-560.5721825	-560.6188760
10	hX_b_aap	-563.7171396	0.0867265	-563.6304130	-563.6729082	-0.0442314	#N/A	#N/A	#N/A	#N/A
11	hX_a_abr	-563.7189126	0.0885205	-563.6303920	-563.6734480	-0.0454645	-562.7382103	-562.9090603	-560.5713574	-560.6168220
12	hX_a_aau	-563.7172378	0.0868627	-563.6303750	-563.6720313	-0.0452065	#N/A	#N/A	#N/A	#N/A
13	hX_b_abn	-563.7172176	0.0868655	-563.6303520	-563.6714066	-0.0458110	#N/A	#N/A	#N/A	#N/A
14	hX_b_abf	-563.7171015	0.0867605	-563.6303410	-563.6726466	-0.0444550	#N/A	#N/A	#N/A	#N/A
15	hX_a_aan	-563.7175819	0.0872448	-563.6303370	-563.6702556	-0.0473263	-562.7354040	-562.9066579	-560.5719230	-560.6192493
16	hX_a_aaq	-563.7168099	0.0865038	-563.6303060	-563.6700156	-0.0467943	#N/A	#N/A	#N/A	#N/A
17	hX_a_aae	-563.7171008	0.0868037	-563.6302970	-563.6683978	-0.0487029	#N/A	#N/A	#N/A	#N/A
18	hX_a_abt	-563.7172912	0.0870782	-563.6302130	-563.6686375	-0.0486537	#N/A	#N/A	#N/A	#N/A
19	hX_a_aam	-563.7176562	0.0874582	-563.6301980	-563.6710288	-0.0466274	-562.7360799	-562.9072750	-560.5724288	-560.6190562
20	hX_b_abt	-563.7171997	0.0870696	-563.6301300	-563.6710102	-0.0461895	#N/A	#N/A	#N/A	#N/A
1	rc_A_01_abg	-543.6313273	0.0980933	-543.5332340	-543.5239738	-0.1073535	-542.5970754	-542.7557544	-540.4849080	-540.5922615
2	rc_A_01_abf	-543.6313523	0.0984732	-543.5328790	-543.5228904	-0.1084619	-542.5960970	-542.7547733	-540.4834818	-540.5919437
3	rc_A_01_abv	-543.6314573	0.0986752	-543.5327820	-543.5247408	-0.1067164	-542.5978507	-542.7564539	-540.4858185	-540.5925349
4	rc_A_01_aaf	-543.6299202	0.0973161	-543.5326040	-543.5219606	-0.1079595	-542.5954772	-542.7542741	-540.4739440	-540.5819035
5	rc_A_01_aay	-543.6313028	0.0987518	-543.5325510	-543.5233713	-0.1079315	-542.5964509	-542.7551865	-540.4841496	-540.5920812
6	rc_A_01_aah	-543.6301798	0.0982648	-543.5319150	-543.5223488	-0.1078310	-542.5960710	-542.7547199	-540.4752328	-540.5830638
7	rc_A_01_aaj	-543.6313078	0.0995828	-543.5317250	-543.5226550	-0.1086528	-542.5963631	-542.7550991	-540.4658209	-540.5744737
8	rc_A_01_abs	-543.6299703	0.0983152	-543.5316550	-543.5222941	-0.1076761	-542.5962088	-542.7548282	-540.4723555	-540.5800317
9	rc_A_01_abx	-543.6268269	0.0978469	-543.5289800	-543.5068316	-0.1199953	#N/A	#N/A	#N/A	#N/A
10	rc_A_01_abb	-543.6262918	0.0977827	-543.5285090	-543.5073216	-0.1189702	#N/A	#N/A	#N/A	#N/A
11	rc_A_01_abm	-543.6256467	0.0975867	-543.5280600	-543.4967091	-0.1289376	#N/A	#N/A	#N/A	#N/A
12	rc_A_01_aad	-543.6256596	0.0976856	-543.5279740	-543.4974415	-0.1282181	#N/A	#N/A	#N/A	#N/A
13	rc_A_01_abh	-543.6253284	0.0975133	-543.5278150	-543.4940428	-0.1312855	#N/A	#N/A	#N/A	#N/A
14	rc_A_01_abk	-543.6279348	0.1003168	-543.5276180	-543.4964890	-0.1314458	-542.5709167	-542.7301437	-540.4590942	-540.5905401
15	rc_A_01_aap	-543.6278815	0.1006005	-543.5272810	-543.4970119	-0.1308696	#N/A	#N/A	#N/A	#N/A
16	rc_A_01_abr	-543.6281377	0.1008756	-543.5272620	-543.4955070	-0.1326306	-542.5702315	-542.7293819	-540.4560551	-540.5886857
17	rc_A_01_abp	-543.6270568	0.1002407	-543.5268160	-543.4966500	-0.1304068	#N/A	#N/A	#N/A	#N/A
18	rc_A_01_aax	-543.6239661	0.0975341	-543.5264320	-543.4963369	-0.1276292	#N/A	#N/A	#N/A	#N/A
19	rc_A_01_abj	-543.6252391	0.0993710	-543.5258680	-543.4907991	-0.1344399	#N/A	#N/A	#N/A	#N/A
20	rc_A_01_aau	-543.6248292	0.0993661	-543.5254630	-543.4949634	-0.1298658	#N/A	#N/A	#N/A	#N/A
1	rc_5mUaaq	-530.4337928	0.0989538	-530.3348390	-530.3211002	-0.1126926	-529.4521192	-529.6098917	-527.4442160	-527.5569086
2	rc_5mUaat	-530.4337742	0.0990042	-530.3347700	-530.3205026	-0.1132717	-529.4515590	-529.6093724	-527.4433709	-527.5566425
3	rc_5mUaau	-530.4337413	0.0989773	-530.3347640	-530.3218730	-0.1118683	-529.4528712	-529.6105299	-527.4452626	-527.5571309
4	rc_5mUach	-530.4302712	0.0979832	-530.3322880	-530.3136990	-0.1165722	-529.4447297	-529.6025992	-527.4384573	-527.5550296
5	rc_5mUadt	-530.4303479	0.0984488	-530.3318990	-530.3141838	-0.1161641	-529.4447616	-529.6028844	-527.4384006	-527.5545647

6	rc_5mUaak	-530.4304359	0.0987009	-530.3317350	-530.3143976	-0.1160383	-529.4451621	-529.6031956	-527.4392107	-527.5552491
7	rc_5mUabn	-530.4303092	0.0986881	-530.3316210	-530.3149432	-0.1153660	-529.4454761	-529.6035309	-527.4389397	-527.5543057
8	rc_5mUadp	-530.4303720	0.0988569	-530.3315150	-530.3141014	-0.1162705	-529.4447531	-529.6028071	-527.4381061	-527.5543766
9	rc_5mUaab	-530.4270035	0.0965265	-530.3304770	-530.2974000	-0.1296036	#N/A	#N/A	#N/A	#N/A
10	rc_5mUaca	-530.4277829	0.0979269	-530.3298560	-530.2944943	-0.1332886	#N/A	#N/A	#N/A	#N/A
11	rc_5mUado	-530.4274235	0.0977635	-530.3296600	-530.2976719	-0.1297516	#N/A	#N/A	#N/A	#N/A
12	rc_5mUaai	-530.4229497	0.0936767	-530.3292730	-530.3014933	-0.1214564	#N/A	#N/A	#N/A	#N/A
13	rc_5mUacv	-530.4277091	0.0985810	-530.3291280	-530.2986647	-0.1290443	#N/A	#N/A	#N/A	#N/A
14	rc_5mUaba	-530.4255675	0.0966815	-530.3288860	-530.3000006	-0.1255669	#N/A	#N/A	#N/A	#N/A
15	rc_5mUacy	-530.4277720	0.0988969	-530.3288750	-530.2958445	-0.1319275	#N/A	#N/A	#N/A	#N/A
16	rc_5mUacn	-530.4286774	0.1000374	-530.3286400	-530.3051050	-0.1235724	#N/A	#N/A	#N/A	#N/A
17	rc_5mUabc	-530.4238702	0.0952711	-530.3285990	-530.2875345	-0.1363356	#N/A	#N/A	#N/A	#N/A
18	rc_5mUack	-530.4277214	0.0991884	-530.3285330	-530.2971158	-0.1306056	#N/A	#N/A	#N/A	#N/A
19	rc_5mUacm	-530.4285633	0.1001202	-530.3284430	-530.3025260	-0.1260372	#N/A	#N/A	#N/A	#N/A
20	rc_5mUacu	-530.4286077	0.1003166	-530.3282910	-530.3036200	-0.1249877	#N/A	#N/A	#N/A	#N/A
1	rc_G01_aan	-618.8981285	0.1010974	-618.7970310	-618.7762702	-0.1218583	-617.7535271	-617.9362637	-615.3978982	-615.5197565
2	rc_G01_aag	-618.8984357	0.1014507	-618.7969850	-618.7769386	-0.1214971	-617.7537861	-617.9366232	-615.3975652	-615.5190624
3	rc_G01_abm	-618.8982042	0.1019852	-618.7962190	-618.7767522	-0.1214520	-617.7539581	-617.9366691	-615.3982777	-615.5197298
4	rc_G01_aai	-618.8986173	0.1028092	-618.7958080	-618.7828262	-0.1157911	-617.7593476	-617.9418945	-615.4034929	-615.5192840
5	rc_G01_aaj	-618.8966603	0.1009462	-618.7957140	-618.7746282	-0.1220320	-617.7519574	-617.9348305	-615.3961780	-615.5182100
6	rc_G01_aah	-618.8967821	0.1011340	-618.7956480	-618.7743527	-0.1224294	-617.7517756	-617.9346058	-615.3957413	-615.5181708
7	rc_G02_abr	-618.8953659	0.1013759	-618.7939900	-618.7738018	-0.1215641	#N/A	#N/A	#N/A	#N/A
8	rc_G02_aaw	-618.8961395	0.1021884	-618.7939510	-618.7827452	-0.1133943	-617.7584851	-617.9410230	-615.4035289	-615.5169232
9	rc_G01_aat	-618.8933663	0.0998282	-618.7935380	-618.7635563	-0.1298100	#N/A	#N/A	#N/A	#N/A
10	rc_G02_aal	-618.8960123	0.1025932	-618.7934190	-618.7758524	-0.1201599	-617.7522988	-617.9350254	-615.3994375	-615.5195974
11	rc_G02_aak	-618.8956548	0.1023087	-618.7933460	-618.7791379	-0.1165168	-617.7550311	-617.9376966	-615.4009021	-615.5174189
12	rc_G01_aba	-618.8950862	0.1017731	-618.7933130	-618.7518251	-0.1432610	#N/A	#N/A	#N/A	#N/A
13	rc_G02_aag	-618.8955649	0.1024388	-618.7931260	-618.7773808	-0.1181841	#N/A	#N/A	#N/A	#N/A
14	rc_G01_aaf	-618.8933046	0.1003005	-618.7930040	-618.7653483	-0.1279563	#N/A	#N/A	#N/A	#N/A
15	rc_G01_abs	-618.8921905	0.0992365	-618.7929540	-618.7621421	-0.1300484	#N/A	#N/A	#N/A	#N/A
16	rc_G02_aaa	-618.8935042	0.1007691	-618.7927350	-618.7717231	-0.1217810	#N/A	#N/A	#N/A	#N/A
17	rc_G02_abg	-618.8964664	0.1037873	-618.7926790	-618.7758031	-0.1206633	-617.7517971	-617.9347409	-615.3987565	-615.5194198
18	rc_G02_aaf	-618.8934941	0.1009351	-618.7925590	-618.7716285	-0.1218656	#N/A	#N/A	#N/A	#N/A
19	rc_G02_aay	-618.8950083	0.1026272	-618.7923810	-618.7702169	-0.1247914	#N/A	#N/A	#N/A	#N/A
20	rc_G01_aac	-618.8921911	0.0998720	-618.7923190	-618.7633064	-0.1288847	#N/A	#N/A	#N/A	#N/A
1	rc_Caar	-471.2397707	0.0848467	-471.1549240	-471.1110224	-0.1287483	-470.3357942	-470.4753360	-468.5298565	-468.6586048
2	rc_Caab	-471.2406526	0.0860346	-471.1546180	-471.1241402	-0.1165124	-470.3470031	-470.4864358	-468.5409932	-468.6575056
3	rc_Cadd	-471.2398340	0.0852240	-471.1546100	-471.1169295	-0.1229045	-470.3402783	-470.4800610	-468.5342155	-468.6571200
4	rc_Cacu	-471.2406525	0.0860435	-471.1546090	-471.1241403	-0.1165122	-470.3470373	-470.4864490	-468.5409915	-468.6575038
5	rc_Caaw	-471.2395330	0.0852630	-471.1542700	-471.1126402	-0.1268928	-470.3363718	-470.4763293	-468.5312565	-468.6581493
6	rc_Cacq	-471.2404400	0.0863929	-471.1540470	-471.1084423	-0.1319977	-470.3332434	-470.4726906	-468.5248859	-468.6568835
7	rc_Cact	-471.2403194	0.0862884	-471.1540310	-471.1210018	-0.1193176	-470.3439949	-470.4835998	-468.5383286	-468.6576462
8	rc_Caao	-471.2392709	0.0855789	-471.1536920	-471.1165947	-0.1226762	-470.3402144	-470.4798939	-468.5351239	-468.6578001
9	rc_Caay	-471.2392616	0.0857816	-471.1534800	-471.1179766	-0.1212851	-470.3414974	-470.4811409	-468.5366201	-468.6579052
10	rc_Caad	-471.2397295	0.0862594	-471.1534700	-471.1112551	-0.1284743	-470.3357769	-470.4754219	-468.5301147	-468.6585890
11	rc_Cacy	-471.2397136	0.0864466	-471.1532670	-471.1108603	-0.1288533	-470.3350638	-470.4748894	-468.5296393	-468.6584927
12	rc_Cabi	-471.2402702	0.0871511	-471.1531190	-471.1109811	-0.1292891	-470.3348257	-470.4746282	-468.5278516	-468.6571407
13	rc_Cace	-471.2395767	0.0865587	-471.1530180	-471.1205162	-0.1190606	-470.3437589	-470.4833828	-468.5386782	-468.6577388

14	rc_Cabh	-471.2403191	0.0877950	-471.1525240	-471.1096834	-0.1306357	-470.3339504	-470.4735968	-468.5263788	-468.6570145
15	rc_Cabc	-471.2394700	0.0869510	-471.1525190	-471.1128688	-0.1266012	-470.3366101	-470.4764544	-468.5309556	-468.6575568
16	rc_Cabm	-471.2363981	0.0842761	-471.1521220	-471.0995668	-0.1368313	-470.3215691	-470.4628128	-468.5206841	-468.6575154
17	rc_Cacz	-471.2341352	0.0829641	-471.1511710	-471.1000950	-0.1340402	-470.3228367	-470.4638506	-468.5228455	-468.6568857
18	rc_Cadq	-471.2364613	0.0854572	-471.1510040	-471.0999589	-0.1365024	-470.3226242	-470.4636461	-468.5211627	-468.6576651
19	rc_Cack	-471.2364907	0.0856496	-471.1508410	-471.0995809	-0.1369098	-470.3230312	-470.4637742	-468.5208469	-468.6577567
20	rc_Cadh	-471.2352820	0.0848869	-471.1503950	-471.0939922	-0.1412898	#N/A	#N/A	#N/A	#N/A
1	rc_U14_ack	-491.0974078	0.0726418	-491.0247660	-490.9804415	-0.1169664	-490.1946658	-490.3403243	-488.3756134	-488.4925798
2	rc_U14_aar	-491.0974138	0.0730237	-491.0243900	-490.9785248	-0.1188890	-490.1928475	-490.3386877	-488.3729863	-488.4918752
3	rc_U14_abi	-491.0974635	0.0732155	-491.0242480	-490.9802490	-0.1172145	-490.1945035	-490.3401698	-488.3753121	-488.4925266
4	rc_Uabn	-491.0972515	0.0732144	-491.0240370	-490.9768557	-0.1203958	-490.1914360	-490.3372533	-488.3710953	-488.4914911
5	rc_U14_aak	-491.0974985	0.0735474	-491.0239510	-490.9795252	-0.1179733	-490.1937812	-490.3395744	-488.3741885	-488.4921617
6	rc_U14_abc	-491.0971587	0.0733307	-491.0238280	-490.9756369	-0.1215218	-490.1906079	-490.3363035	-488.3698859	-488.4914078
7	rc_U14_abz	-491.0971982	0.0735652	-491.0236330	-490.9757008	-0.1214974	-490.1905658	-490.3363035	-488.3699208	-488.4914182
8	rc_U14_aal	-491.0941263	0.0731833	-491.0209430	-490.9722377	-0.1218886	#N/A	#N/A	#N/A	#N/A
9	rc_Uacq	-491.0941560	0.0736209	-491.0205350	-490.9724811	-0.1216748	-490.1865786	-490.3325838	-488.3686134	-488.4902882
10	rc_Uadu	-491.0941379	0.0737999	-491.0203380	-490.9726842	-0.1214537	#N/A	#N/A	#N/A	#N/A
11	rc_Uade	-491.0938275	0.0735475	-491.0202800	-490.9718305	-0.1219970	#N/A	#N/A	#N/A	#N/A
12	rc_Uadd	-491.0942083	0.0743272	-491.0198810	-490.9729115	-0.1212968	-490.1868268	-490.3329141	-488.3688734	-488.4901702
13	rc_Uaah	-491.0937796	0.0750935	-491.0186860	-490.9674862	-0.1262934	#N/A	#N/A	#N/A	#N/A
14	rc_Uaav	-491.0940481	0.0753670	-491.0186810	-490.9642795	-0.1297686	#N/A	#N/A	#N/A	#N/A
15	rc_Uaaa	-491.0940291	0.0754161	-491.0186130	-490.9665121	-0.1275170	#N/A	#N/A	#N/A	#N/A
16	rc_U14_abb	-491.0941454	0.0758093	-491.0183360	-490.9604559	-0.1336895	#N/A	#N/A	#N/A	#N/A
17	rc_U14_aba	-491.0941456	0.0758515	-491.0182940	-490.9595797	-0.1345659	#N/A	#N/A	#N/A	#N/A
18	rc_U14_abe	-491.0939698	0.0758117	-491.0181580	-490.9670353	-0.1269345	#N/A	#N/A	#N/A	#N/A
19	rc_Uadp	-491.0941013	0.0761093	-491.0179920	-490.9646803	-0.1294210	#N/A	#N/A	#N/A	#N/A
20	rc_Uaad	-491.0940579	0.0761989	-491.0178590	-490.9654548	-0.1286032	#N/A	#N/A	#N/A	#N/A
1	rc_1mG_01_abo	-658.2089620	0.1288950	-658.0800670	-658.0963309	-0.1126311	-656.9929441	-657.1870554	-654.4417885	-654.5544196
2	rc_1mG_01_abe	-658.2090080	0.1291589	-658.0798490	-658.0965446	-0.1124634	-656.9931488	-657.1872422	-654.4419284	-654.5543918
3	rc_1mG_01_abg	-658.2089392	0.1291241	-658.0798150	-658.0959376	-0.1130016	-656.9925567	-657.1866994	-654.4414451	-654.5544467
4	rc_1mG_01_aae	-658.2079917	0.1283916	-658.0796000	-658.0932464	-0.1147453	-656.9900314	-657.1841306	-654.4376924	-654.5524376
5	rc_1mG_01_abq	-658.2090062	0.1294881	-658.0795180	-658.0958669	-0.1131393	-656.9925076	-657.1866207	-654.4416602	-654.5547995
6	rc_1mG_01_aal	-658.2073635	0.1280245	-658.0793390	-658.0935363	-0.1138273	-656.9904973	-657.1847275	-654.4387163	-654.5525436
7	rc_1mG_01_abs	-658.2079851	0.1287110	-658.0792740	-658.0934776	-0.1145075	-656.9901224	-657.1842991	-654.4378305	-654.5523380
8	rc_1mG_01_abi	-658.2079311	0.1292020	-658.0787290	-658.0942312	-0.1136998	-656.9907440	-657.1849245	-654.4383319	-654.5520317
9	rc_1mG_02_aae	-658.2067621	0.1282841	-658.0784780	-658.0937709	-0.1129912	#N/A	#N/A	#N/A	#N/A
10	rc_1mG_01_abt	-658.2049940	0.1269069	-658.0780870	-658.0759398	-0.1290541	#N/A	#N/A	#N/A	#N/A
11	rc_1mG_02_abb	-658.2067765	0.1286904	-658.0780860	-658.0936810	-0.1130955	#N/A	#N/A	#N/A	#N/A
12	rc_1mG_01_aai	-658.2079979	0.1302119	-658.0777860	-658.0918884	-0.1161096	-656.9891592	-657.1830651	-654.4363446	-654.5524542
13	rc_1mG_01_aab	-658.2051971	0.1277601	-658.0774370	-658.0733142	-0.1318830	#N/A	#N/A	#N/A	#N/A
14	rc_1mG_02_aam	-658.2075026	0.1302775	-658.0772250	-658.0958503	-0.1116523	-656.9914282	-657.1857386	-654.4422924	-654.5539446
15	rc_1mG_02_abg	-658.2053032	0.1280942	-658.0772090	-658.0913992	-0.1139041	#N/A	#N/A	#N/A	#N/A
16	rc_1mG_01_abw	-658.2061975	0.1292025	-658.0769950	-658.0723000	-0.1338975	#N/A	#N/A	#N/A	#N/A
17	rc_1mG_01_aah	-658.2029602	0.1259761	-658.0769840	-658.0821190	-0.1208411	#N/A	#N/A	#N/A	#N/A
18	rc_1mG_02_aaq	-658.2046305	0.1279175	-658.0767130	-658.0929725	-0.1116580	#N/A	#N/A	#N/A	#N/A
19	rc_1mG_01_abc	-658.2046408	0.1279728	-658.0766680	-658.0699600	-0.1346808	#N/A	#N/A	#N/A	#N/A
20	rc_1mG_02_abm	-658.2048378	0.1281917	-658.0766460	-658.0934195	-0.1114183	#N/A	#N/A	#N/A	#N/A
1	rc_X03_abq	-638.7558832	0.0900412	-638.6658420	-638.6269244	-0.1289588	-637.5957713	-637.7846490	-635.2071099	-635.3360688

2	rc_X03_aax	-638.7547073	0.0893973	-638.6653100	-638.6244772	-0.1302301	-637.5936758	-637.7826240	-635.2056994	-635.3359295
3	rc_X03_abj	-638.7546423	0.0893973	-638.6652450	-638.6218323	-0.1328100	-637.5912682	-637.7802520	-635.2027885	-635.3355986
4	rc_X03_aap	-638.7545940	0.0893939	-638.6652000	-638.6221736	-0.1324203	-637.5915183	-637.7805547	-635.2032072	-635.3356275
5	rc_X03_abu	-638.7545856	0.0894136	-638.6651720	-638.6226567	-0.1319289	-637.5919200	-637.7809640	-635.2037073	-635.3356362
6	rc_X03_aau	-638.7546518	0.0894988	-638.6651530	-638.6237926	-0.1308592	-637.5930210	-637.7820189	-635.2049572	-635.3358164
7	rc_X01_aam	-638.7531459	0.0881838	-638.6649620	-638.6345330	-0.1186129	#N/A	#N/A	#N/A	#N/A
8	rc_X03_abh	-638.7545965	0.0897314	-638.6648650	-638.6225042	-0.1320923	-637.5918261	-637.7808598	-635.2035509	-635.3356432
9	rc_X02_aas	-638.7537372	0.0890911	-638.6646460	-638.6185662	-0.1351710	-637.5888976	-637.7778473	-635.2102944	-635.3454653
10	rc_X01_aaa	-638.7532360	0.0886920	-638.6645440	-638.6330045	-0.1202315	#N/A	#N/A	#N/A	#N/A
11	rc_X01_aax	-638.7539183	0.0894992	-638.6644190	-638.6358491	-0.1180692	-637.6047696	-637.7936606	-635.2333221	-635.3513913
12	rc_X01_aba	-638.7531933	0.0888773	-638.6643160	-638.6343817	-0.1188117	#N/A	#N/A	#N/A	#N/A
13	rc_X01_abh	-638.7533037	0.0890367	-638.6642670	-638.6332286	-0.1200751	#N/A	#N/A	#N/A	#N/A
14	rc_X02_abh	-638.7528987	0.0889437	-638.6639550	-638.6207001	-0.1321986	#N/A	#N/A	#N/A	#N/A
15	rc_X02_aat	-638.7529011	0.0890450	-638.6638560	-638.6189370	-0.1339641	#N/A	#N/A	#N/A	#N/A
16	rc_X01_abf	-638.7539430	0.0903210	-638.6636220	-638.6359743	-0.1179687	-637.6051649	-637.7938867	-635.2338550	-635.3518237
17	rc_X02_abl	-638.7530681	0.0896240	-638.6634440	-638.6249306	-0.1281375	#N/A	#N/A	#N/A	#N/A
18	rc_X01_abv	-638.7509753	0.0885353	-638.6624400	-638.6295831	-0.1213922	#N/A	#N/A	#N/A	#N/A
19	rc_X03_aal	-638.7505520	0.0884679	-638.6620840	-638.6134354	-0.1371166	#N/A	#N/A	#N/A	#N/A
20	rc_X03_abk	-638.7507438	0.0886798	-638.6620640	-638.6148105	-0.1359334	#N/A	#N/A	#N/A	#N/A
1	rc_hX_a_aal	-563.4934143	0.0857403	-563.4076740	-563.3764094	-0.1170049	-562.4431082	-562.6076792	-560.3018500	-560.4188549
2	rc_hX_a_aaz	-563.4933230	0.0858879	-563.4074350	-563.3766366	-0.1166864	-562.4433463	-562.6079348	-560.3022469	-560.4189333
3	rc_hX_a_abq	-563.4934401	0.0861310	-563.4073090	-563.3773812	-0.1160589	-562.4441015	-562.6085870	-560.3030516	-560.4191105
4	rc_hX_a_abe	-563.4934186	0.0862856	-563.4071330	-563.3771942	-0.1162245	-562.4439004	-562.6084219	-560.3028816	-560.4191061
5	rc_hX_a_abs	-563.4928961	0.0859971	-563.4068990	-563.3772401	-0.1156561	-562.4435281	-562.6082176	-560.3014531	-560.4171091
6	rc_hX_a_aae	-563.4933060	0.0864270	-563.4068790	-563.3761301	-0.1171759	-562.4428280	-562.6074534	-560.3016449	-560.4188208
7	rc_hX_a_aaa	-563.4933395	0.0865155	-563.4068240	-563.3759777	-0.1173618	-562.4426835	-562.6073093	-560.3014538	-560.4188157
8	rc_hX_b_aab	-563.4909696	0.0854216	-563.4055480	-563.3732852	-0.1176845	-562.4395555	-562.6042406	-560.3015632	-560.4192477
9	rc_hX_b_aas	-563.4903895	0.0859235	-563.4044660	-563.3719694	-0.1184201	-562.4385819	-562.6031926	-560.2990857	-560.4175058
10	rc_hX_b_aad	-563.4909011	0.0869871	-563.4039140	-563.3729076	-0.1179935	-562.4391503	-562.6038513	-560.3013566	-560.4193502
11	rc_hX_b_abm	-563.4896983	0.0861313	-563.4035670	-563.3705305	-0.1191678	#N/A	#N/A	#N/A	#N/A
12	rc_hX_b_abs	-563.4897694	0.0864423	-563.4033270	-563.3721512	-0.1176182	#N/A	#N/A	#N/A	#N/A
13	rc_hX_b_abl	-563.4897278	0.0864827	-563.4032450	-563.3703775	-0.1193502	#N/A	#N/A	#N/A	#N/A
14	rc_hX_b_aak	-563.4897187	0.0866266	-563.4030920	-563.3708784	-0.1188403	#N/A	#N/A	#N/A	#N/A
15	rc_hX_b_aae	-563.4897180	0.0866549	-563.4030630	-563.3702920	-0.1194259	#N/A	#N/A	#N/A	#N/A
16	rc_hX_b_abf	-563.4898431	0.0869091	-563.4029340	-563.3734269	-0.1164163	#N/A	#N/A	#N/A	#N/A
17	rc_hX_a_aay	-563.4877880	0.0850489	-563.4027390	-563.3541500	-0.1336379	#N/A	#N/A	#N/A	#N/A
18	rc_hX_a_aac	-563.4889995	0.0865244	-563.4024750	-563.3645697	-0.1244298	#N/A	#N/A	#N/A	#N/A
19	rc_hX_a_abk	-563.4886060	0.0863460	-563.4022600	-563.3604335	-0.1281725	#N/A	#N/A	#N/A	#N/A
20	rc_hX_a_aat	-563.4880688	0.0858447	-563.4022240	-563.3556990	-0.1323697	#N/A	#N/A	#N/A	#N/A
1	rc_caffeine_abi	-756.6946627	0.1671937	-756.5274690	-756.5795166	-0.1151462	#N/A	#N/A	#N/A	#N/A
2	rc_caffeine_aad	-756.6947723	0.1677972	-756.5269750	-756.5786872	-0.1160851	#N/A	#N/A	#N/A	#N/A
3	rc_caffeine_abl	-756.6943193	0.1674743	-756.5268450	-756.5784907	-0.1158287	#N/A	#N/A	#N/A	#N/A
4	rc_caffeine_abp	-756.6949197	0.1682766	-756.5266430	-756.5805123	-0.1144074	-755.3054648	-755.5290890	-752.3527726	-752.4671800
5	rc_caffeine_abe	-756.6942071	0.1677270	-756.5264800	-756.5890815	-0.1051256	#N/A	#N/A	#N/A	#N/A
6	rc_caffeine_aal	-756.6920566	0.1656705	-756.5263860	-756.5825929	-0.1094637	#N/A	#N/A	#N/A	#N/A
7	rc_caffeine_aat	-756.6915451	0.1651761	-756.5263690	-756.5769447	-0.1146004	#N/A	#N/A	#N/A	#N/A
8	rc_caffeine_abx	-756.6932426	0.1668836	-756.5263590	-756.5851503	-0.1080923	#N/A	#N/A	#N/A	#N/A
9	rc_caffeine_aar	-756.6948570	0.1687120	-756.5261450	-756.5806369	-0.1142201	-755.3058534	-755.5296476	-752.3534635	-752.4676836

10	rc_caffeine_abt	-756.6919115	0.1660145	-756.5258970	-756.5835328	-0.1083787	#N/A	#N/A	#N/A	#N/A
11	rc_caffeine_aag	-756.6919030	0.1661750	-756.5257280	-756.5798293	-0.1120737	#N/A	#N/A	#N/A	#N/A
12	rc_caffeine_aau	-756.6942593	0.1685512	-756.5257080	-756.5786726	-0.1155866	#N/A	#N/A	#N/A	#N/A
13	rc_caffeine_aam	-756.6941057	0.1684627	-756.5256430	-756.5738378	-0.1202679	#N/A	#N/A	#N/A	#N/A
14	rc_caffeine_aay	-756.6952829	0.1699069	-756.5253760	-756.5768230	-0.1184600	-755.3023785	-755.5256982	-752.3477553	-752.4662153
15	rc_caffeine_abd	-756.6944592	0.1691202	-756.5253390	-756.5848323	-0.1096269	#N/A	#N/A	#N/A	#N/A
16	rc_caffeine_aai	-756.6953260	0.1700399	-756.5252860	-756.5763859	-0.1189400	-755.3023964	-755.5255561	-752.3462261	-752.4651662
17	rc_caffeine_abo	-756.6918051	0.1665680	-756.5252370	-756.5879035	-0.1039015	#N/A	#N/A	#N/A	#N/A
18	rc_caffeine_abr	-756.6939099	0.1686728	-756.5252370	-756.5737186	-0.1201913	#N/A	#N/A	#N/A	#N/A
19	rc_caffeine_aax	-756.6936705	0.1685294	-756.5251410	-756.5737435	-0.1199270	#N/A	#N/A	#N/A	#N/A
20	rc_caffeine_aaa	-756.6948348	0.1697287	-756.5251060	-756.5782195	-0.1166153	-755.3033898	-755.5274730	-752.3506244	-752.4672397
1	rc_PhOH_abz	-383.7572039	0.0936898	-383.6635140	-383.6585215	-0.0986824	-382.9853506	-383.0967873	-381.4620134	-381.5606957
2	rc_PhOH_aby	-383.7572028	0.0938287	-383.6633740	-383.6611925	-0.0960102	-382.9871908	-383.0989284	-381.4648114	-381.5608217
3	rc_PhOH_adv	-383.7569122	0.0935951	-383.6633170	-383.6654063	-0.0915059	-382.9911613	-383.1027105	-381.4694203	-381.5609262
4	rc_PhOH_abw	-383.7572248	0.0941408	-383.6630840	-383.6592693	-0.0979555	-382.9856394	-383.0972652	-381.4626568	-381.5606123
5	rc_PhOH_adm	-383.7572428	0.0941627	-383.6630800	-383.6596496	-0.0975932	-382.9858016	-383.0975301	-381.4629827	-381.5605759
6	rc_PhOH_ado	-383.7443634	0.0902903	-383.6540730	-383.6316696	-0.1126938	#N/A	#N/A	#N/A	#N/A
7	rc_PhOH_aav	-383.7469886	0.0930795	-383.6539090	-383.6266549	-0.1203337	-382.9538651	-383.0670898	-381.4383208	-381.5586545
8	rc_PhOH_adh	-383.7447969	0.0914138	-383.6533830	-383.6287800	-0.1160168	#N/A	#N/A	#N/A	#N/A
9	rc_PhOH_ade	-383.7448029	0.0916568	-383.6531460	-383.6239689	-0.1208340	#N/A	#N/A	#N/A	#N/A
10	rc_PhOH_adc	-383.7461935	0.0930864	-383.6531070	-383.6253318	-0.1208617	-382.9528214	-383.0658600	-381.4390769	-381.5599386
11	rc_PhOH_ach	-383.7458415	0.0927554	-383.6530860	-383.6329292	-0.1129122	#N/A	#N/A	#N/A	#N/A
12	rc_PhOH_adn	-383.7454054	0.0924814	-383.6529240	-383.6247901	-0.1206153	#N/A	#N/A	#N/A	#N/A
13	rc_PhOH_abq	-383.7451353	0.0922383	-383.6528970	-383.6243440	-0.1207913	#N/A	#N/A	#N/A	#N/A
14	rc_PhOH_abc	-383.7443217	0.0914706	-383.6528510	-383.6324989	-0.1118228	#N/A	#N/A	#N/A	#N/A
15	rc_PhOH_abi	-383.7458677	0.0930216	-383.6528460	-383.6333101	-0.1125575	#N/A	#N/A	#N/A	#N/A
16	rc_PhOH_acs	-383.7461303	0.0934413	-383.6526890	-383.6294633	-0.1166670	#N/A	#N/A	#N/A	#N/A
17	rc_PhOH_abk	-383.7461007	0.0934647	-383.6526360	-383.6341393	-0.1119614	#N/A	#N/A	#N/A	#N/A
18	rc_PhOH_abn	-383.7461836	0.0936655	-383.6525180	-383.6253049	-0.1208787	-382.9521573	-383.0654733	-381.4387895	-381.5596682
19	rc_PhOH_acq	-383.7452010	0.0927840	-383.6524170	-383.6246350	-0.1205660	#N/A	#N/A	#N/A	#N/A
20	rc_PhOH_aar	-383.7444495	0.0921785	-383.6522710	-383.6335155	-0.1109341	#N/A	#N/A	#N/A	#N/A
1	rc_1mInd_aaf	-479.4360076	0.1430985	-479.2929090	-479.3329618	-0.1030458	-478.4371200	-478.5756344	-476.4296335	-476.5326793
2	rc_1mInd_abq	-479.4362987	0.1435237	-479.2927750	-479.3335423	-0.1027565	-478.4376369	-478.5764247	-476.4303186	-476.5330751
3	rc_1mInd_aau	-479.4353380	0.1425870	-479.2927510	-479.3391495	-0.0961885	-478.4425172	-478.5816909	-476.4372945	-476.5334830
4	rc_1mInd_abk	-479.4338145	0.1414564	-479.2923580	-479.3294980	-0.1043165	#N/A	#N/A	#N/A	#N/A
5	rc_1mInd_aah	-479.4332071	0.1408930	-479.2923140	-479.3349409	-0.0982661	#N/A	#N/A	#N/A	#N/A
6	rc_1mInd_abn	-479.4339210	0.1417180	-479.2922030	-479.3406459	-0.0932751	-478.4441181	-478.5829745	-476.4387462	-476.5320213
7	rc_1mInd_aae	-479.4366703	0.1445512	-479.2921190	-479.3341012	-0.1025691	-478.4378697	-478.5765464	-476.4299738	-476.5325430
8	rc_1mInd_aaw	-479.4366568	0.1445517	-479.2921050	-479.3338776	-0.1027792	-478.4376093	-478.5762850	-476.4297039	-476.5324831
9	rc_1mInd_aam	-479.4356531	0.1436281	-479.2920250	-479.3331820	-0.1024711	-478.4372971	-478.5759146	-476.4298644	-476.5323355
10	rc_1mInd_aal	-479.4337303	0.1423672	-479.2913630	-479.3384693	-0.0952610	#N/A	#N/A	#N/A	#N/A
11	rc_1mInd_abx	-479.4331751	0.1420730	-479.2911020	-479.3364682	-0.0967069	#N/A	#N/A	#N/A	#N/A
12	rc_1mInd_abj	-479.4332561	0.1423790	-479.2908770	-479.3320108	-0.1012453	#N/A	#N/A	#N/A	#N/A
13	rc_1mInd_aac	-479.4341779	0.1435958	-479.2905820	-479.3318394	-0.1023385	-478.4380845	-478.5758447	-476.4310268	-476.5333652
14	rc_1mInd_abw	-479.4341486	0.1436405	-479.2905080	-479.3312138	-0.1029348	-478.4374481	-478.5752480	-476.4305079	-476.5334427
15	rc_1mInd_aaz	-479.4332598	0.1428827	-479.2903770	-479.3279638	-0.1052960	#N/A	#N/A	#N/A	#N/A
16	rc_1mInd_abr	-479.4338948	0.1439177	-479.2899770	-479.3296867	-0.1042081	#N/A	#N/A	#N/A	#N/A
17	rc_1mInd_aba	-479.4336827	0.1440917	-479.2895910	-479.3311575	-0.1025252	#N/A	#N/A	#N/A	#N/A

18	rc_1mInd_abb	-479.4341927	0.1447586	-479.2894340	-479.3315043	-0.1026884	-478.4375889	-478.5754869	-476.4309211	-476.5336094
1	rc_Ind_abk	-440.1218563	0.1164862	-440.0053700	-440.0360744	-0.0857819	-439.2208009	-439.3470919	-437.4055465	-437.4913284
2	rc_Ind_abm	-440.1219514	0.1166943	-440.0052570	-440.0335401	-0.0884113	-439.2187121	-439.3449985	-437.4033937	-437.4918050
3	rc_Ind_abe	-440.1218387	0.1167037	-440.0051350	-440.0355051	-0.0863336	-439.2202437	-439.3465803	-437.4050238	-437.4913574
4	rc_Ind_aak	-440.1219231	0.1173861	-440.0045370	-440.0335755	-0.0883476	-439.2185747	-439.3449482	-437.4032820	-437.4916296
5	rc_Ind_abh	-440.1219019	0.1173709	-440.0045310	-440.0366906	-0.0852113	-439.2213642	-439.3476195	-437.4060832	-437.4912945
6	rc_Ind_aav	-440.1219330	0.1176250	-440.0043080	-440.0340736	-0.0878595	-439.2193646	-439.3455719	-437.4041167	-437.4919761
7	rc_Ind_abp	-440.1158694	0.1139374	-440.0019320	-440.0125652	-0.1033042	#N/A	#N/A	#N/A	#N/A
8	rc_Ind_abd	-440.1158952	0.1140971	-440.0017980	-440.0126670	-0.1032282	#N/A	#N/A	#N/A	#N/A
9	rc_Ind_abf	-440.1178023	0.1162292	-440.0015730	-440.0161606	-0.1016416	#N/A	#N/A	#N/A	#N/A
10	rc_Ind_aag	-440.1177910	0.1169110	-440.0008800	-440.0172532	-0.1005379	#N/A	#N/A	#N/A	#N/A
11	rc_Ind_aao	-440.1191411	0.1182740	-440.0008670	-440.0096107	-0.1095304	#N/A	#N/A	#N/A	#N/A
12	rc_Ind_aba	-440.1187214	0.1182094	-440.0005120	-440.0097392	-0.1089823	#N/A	#N/A	#N/A	#N/A
13	rc_Ind_aau	-440.1158554	0.1154234	-440.0004320	-440.0142786	-0.1015769	#N/A	#N/A	#N/A	#N/A
14	rc_Ind_aaj	-440.1158478	0.1154587	-440.0003890	-440.0135264	-0.1023214	#N/A	#N/A	#N/A	#N/A
15	rc_Ind_aam	-440.1156977	0.1156597	-440.0000380	-440.0126179	-0.1030798	#N/A	#N/A	#N/A	#N/A
16	rc_Ind_aaf	-440.1160794	0.1163963	-439.9996830	-440.0174619	-0.0986175	#N/A	#N/A	#N/A	#N/A
17	rc_Ind_abg	-440.1164474	0.1169654	-439.9994820	-440.0066559	-0.1097915	#N/A	#N/A	#N/A	#N/A
18	rc_Ind_abo	-440.1158857	0.1167376	-439.9991480	-440.0124578	-0.1034279	#N/A	#N/A	#N/A	#N/A
19	rc_Ind_abi	-440.1166993	0.1180343	-439.9986650	-440.0073962	-0.1093031	#N/A	#N/A	#N/A	#N/A
20	rc_Ind_abb	-440.1171293	0.1189152	-439.9982140	-440.0077272	-0.1094021	#N/A	#N/A	#N/A	#N/A
1	rc_tAn_abp	-746.0303859	0.1113558	-745.9190300	-745.9371597	-0.0932262	#N/A	#N/A	#N/A	#N/A
2	rc_tAn_abs	-746.0299646	0.1110636	-745.9189010	-745.9451322	-0.0848324	#N/A	#N/A	#N/A	#N/A
3	rc_tAn_aal	-746.0321225	0.1133725	-745.9187500	-745.9446606	-0.0874619	#N/A	#N/A	#N/A	#N/A
4	rc_tAn_aav	-746.0320106	0.1134416	-745.9185690	-745.9384739	-0.0935368	#N/A	#N/A	#N/A	#N/A
5	rc_tAn_aan	-746.0305279	0.1120728	-745.9184550	-745.9308248	-0.0997030	#N/A	#N/A	#N/A	#N/A
6	rc_tAn_abc	-746.0322245	0.1138435	-745.9183810	-745.9418797	-0.0903448	#N/A	#N/A	#N/A	#N/A
7	rc_tAn_abj	-746.0339192	0.1159221	-745.9179970	-745.9304186	-0.1035006	-744.8190429	-744.9398836	-743.1456257	-743.2491263
8	rc_tAn_aaz	-746.0316037	0.1137086	-745.9178950	-745.9286031	-0.1030006	#N/A	#N/A	#N/A	#N/A
9	rc_tAn_aab	-746.0303548	0.1126508	-745.9177040	-745.9406368	-0.0897180	#N/A	#N/A	#N/A	#N/A
10	rc_tAn_aaq	-746.0338669	0.1163569	-745.9175100	-745.9314572	-0.1024097	-744.8200664	-744.9409284	-743.1469500	-743.2493597
11	rc_tAn_abn	-746.0290062	0.1117002	-745.9173060	-745.9306061	-0.0984001	#N/A	#N/A	#N/A	#N/A
12	rc_tAn_aac	-746.0311207	0.1138207	-745.9173000	-745.9431230	-0.0879977	#N/A	#N/A	#N/A	#N/A
13	rc_tAn_aae	-746.0335358	0.1165077	-745.9170280	-745.9297146	-0.1038212	-744.8183539	-744.9391371	-743.1449127	-743.2487339
14	rc_tAn_aax	-746.0315508	0.1145398	-745.9170110	-745.9359521	-0.0955987	#N/A	#N/A	#N/A	#N/A
15	rc_tAn_aau	-746.0310369	0.1144528	-745.9165840	-745.9374944	-0.0935425	#N/A	#N/A	#N/A	#N/A
16	rc_tAn_abh	-746.0309822	0.1150222	-745.9159600	-745.9323696	-0.0986126	#N/A	#N/A	#N/A	#N/A
17	rc_tAn_abb	-746.0313657	0.1158606	-745.9155050	-745.9270614	-0.1043043	#N/A	#N/A	#N/A	#N/A
18	rc_tAn_abo	-746.0153972	0.1203362	-745.8950610	-745.9179147	-0.0974825	#N/A	#N/A	#N/A	#N/A
1	empty	0.0000000	#VALUE!	0.0000000	0.0000000	0.0000000	#VALUE!			
1	empty	0.0000000	#VALUE!	0.0000000	0.0000000	0.0000000	#VALUE!			
1	d_A_aao	-543.3755129	0.0872898	-543.2882230	-543.2752786	-0.1002343	-542.3423084	-542.5109842	-540.1846829	-540.2849171
2	d_A_aat	-543.3731072	0.0850192	-543.2880880	-543.2711863	-0.1019210	-542.3366178	-542.5062773	-540.1817943	-540.2837152
3	d_A_abl	-543.3737500	0.0858120	-543.2879380	-543.2712893	-0.1024607	-542.3371332	-542.5066948	-540.1822827	-540.2847434
4	d_A_abd	-543.3731511	0.0854000	-543.2877510	-543.2704256	-0.1027255	-542.3361124	-542.5057619	-540.1812044	-540.2839299
5	d_A_aaf	-543.3731503	0.0854182	-543.2877320	-543.2704259	-0.1027244	-542.3361145	-542.5057786	-540.1812142	-540.2839386
6	d_A_aap	-543.3737839	0.0860639	-543.2877200	-543.2708745	-0.1029094	-542.3368649	-542.5063998	-540.1819682	-540.2848776
7	d_A_aar	-543.3737756	0.0861565	-543.2876190	-543.2705513	-0.1032242	-542.3366389	-542.5061665	-540.1816902	-540.2849144

8	d_A_aan	-543.3737787	0.0863876	-543.2873910	-543.2713450	-0.1024337	-542.3371797	-542.5067428	-540.1823333	-540.2847669
9	d_A_aaa	-543.3739765	0.0867135	-543.2872630	-543.2669296	-0.1070469	#N/A	#N/A	#N/A	#N/A
10	d_A_abe	-543.3752906	0.0882056	-543.2870850	-543.2689090	-0.1063816	-542.3352926	-542.5046384	-540.1792179	-540.2855996
11	d_A_abj	-543.3740201	0.0873891	-543.2866310	-543.2670198	-0.1070003	-542.3334886	-542.5029542	-540.1772606	-540.2842609
12	d_A_aah	-543.3703874	0.0855044	-543.2848830	-543.2617835	-0.1086039	#N/A	#N/A	#N/A	#N/A
13	d_A_abm	-543.3704557	0.0860666	-543.2843890	-543.2620917	-0.1083640	#N/A	#N/A	#N/A	#N/A
14	d_A_aae	-543.3704564	0.0861673	-543.2842890	-543.2607754	-0.1096810	#N/A	#N/A	#N/A	#N/A
15	d_A_aaw	-543.3709100	0.0866409	-543.2842690	-543.2585743	-0.1123356	#N/A	#N/A	#N/A	#N/A
16	d_A_abp	-543.3700300	0.0859239	-543.2841060	-543.2605199	-0.1095101	#N/A	#N/A	#N/A	#N/A
17	d_A_abx	-543.3714111	0.0875251	-543.2838860	-543.2649346	-0.1064765	#N/A	#N/A	#N/A	#N/A
18	d_A_aax	-543.3704607	0.0866296	-543.2838310	-543.2606450	-0.1098156	#N/A	#N/A	#N/A	#N/A
19	d_A_abw	-543.3704035	0.0867575	-543.2836460	-543.2617665	-0.1086371	#N/A	#N/A	#N/A	#N/A
20	d_A_abc	-543.3702080	0.0869449	-543.2832630	-543.2617015	-0.1085065	#N/A	#N/A	#N/A	#N/A
1	d_5mU4_aai	-530.1912491	0.0855110	-530.1057380	-530.0944284	-0.0968207	-529.2128773	-529.3818945	-527.1772050	-527.2740257
2	d_5mU4_abw	-530.1906787	0.0866556	-530.1040230	-530.0907074	-0.0999712	-529.2121008	-529.3805970	-527.1765076	-527.2764788
3	d_5mU4_aan	-530.1912279	0.0876309	-530.1035970	-530.0948541	-0.0963738	-529.2134385	-529.3823024	-527.1776121	-527.2739859
4	d_5mU4_abd	-530.1905488	0.0880388	-530.1025100	-530.0882114	-0.1023375	-529.2091404	-529.3776219	-527.1732564	-527.2755939
5	d_5mU4_abv	-530.1902355	0.0878014	-530.1024340	-530.0892440	-0.1009914	-529.2100653	-529.3786244	-527.1747571	-527.2757485
6	d_5mU4_aae	-530.1902574	0.0882783	-530.1019790	-530.0929672	-0.0972902	-529.2130001	-529.3817222	-527.1783214	-527.2756116
7	d_5mU4_abi	-530.1905714	0.0887223	-530.1018490	-530.0875290	-0.1030424	-529.2081450	-529.3767865	-527.1724696	-527.2755120
8	d_5mU4_aaw	-530.1899260	0.0882789	-530.1016470	-530.0897676	-0.1001584	-529.2089375	-529.3781512	-527.1740216	-527.2741800
9	d_5mU1_aac	-530.1882375	0.0867675	-530.1014700	-530.0733911	-0.1148465	#N/A	#N/A	#N/A	#N/A
10	d_5mU4_abj	-530.1907572	0.0893631	-530.1013940	-530.0890897	-0.1016674	-529.2098085	-529.3783174	-527.1735332	-527.2752007
11	d_5mU1_abo	-530.1877071	0.0865170	-530.1011900	-530.0675957	-0.1201114	#N/A	#N/A	#N/A	#N/A
12	d_5mU1_aae	-530.1888120	0.0877500	-530.1010620	-530.0721615	-0.1166506	-529.1927639	-529.3623649	-527.1542988	-527.2709494
13	d_5mU4_abg	-530.1872568	0.0864068	-530.1008500	-530.0792696	-0.1079872	#N/A	#N/A	#N/A	#N/A
14	d_5mU1_abb	-530.1887021	0.0878571	-530.1008450	-530.0722998	-0.1164023	#N/A	#N/A	#N/A	#N/A
15	d_5mU1_aap	-530.1880432	0.0874442	-530.1005990	-530.0719861	-0.1160571	#N/A	#N/A	#N/A	#N/A
16	d_5mU4_aaq	-530.1878402	0.0876191	-530.1002210	-530.0822530	-0.1055872	#N/A	#N/A	#N/A	#N/A
17	d_5mU1_aaj	-530.1876130	0.0874699	-530.1001430	-530.0729591	-0.1146538	#N/A	#N/A	#N/A	#N/A
18	d_5mU4_aaa	-530.1860422	0.0859371	-530.1001050	-530.0721659	-0.1138763	#N/A	#N/A	#N/A	#N/A
19	d_5mU1_aar	-530.1878158	0.0878648	-530.0999510	-530.0713394	-0.1164764	#N/A	#N/A	#N/A	#N/A
20	d_5mU4_aab	-530.1860251	0.0861330	-530.0998920	-530.0717373	-0.1142878	#N/A	#N/A	#N/A	#N/A
1	d_G14_02_aaa	-618.6319908	0.0906137	-618.5413770	-618.5195684	-0.1124223	#N/A	#N/A	#N/A	#N/A
2	d_G14_17_aad	-618.6286739	0.0878719	-618.5408020	-618.5026818	-0.1259921	#N/A	#N/A	#N/A	#N/A
3	d_G16_03_aag	-618.6297728	0.0890588	-618.5407140	-618.5095115	-0.1202613	-617.4761668	-617.6707249	-615.0844806	-615.2047419
4	d_G14_17_abv	-618.6295437	0.0889427	-618.5406010	-618.5053267	-0.1242170	#N/A	#N/A	#N/A	#N/A
5	d_G14_17_aaj	-618.6309598	0.0903647	-618.5405950	-618.5103468	-0.1206130	-617.4751111	-617.6699450	-615.0855186	-615.2061316
6	d_G14_17_abe	-618.6295223	0.0890052	-618.5405170	-618.5028688	-0.1266535	#N/A	#N/A	#N/A	#N/A
7	d_G16_03_aap	-618.6298272	0.0893192	-618.5405080	-618.5083323	-0.1214949	-617.4753466	-617.6699210	-615.0835788	-615.2050737
8	d_G16_03_abx	-618.6298621	0.0895140	-618.5403480	-618.5084383	-0.1214238	#N/A	#N/A	#N/A	#N/A
9	d_G14_02_abx	-618.6306815	0.0903735	-618.5403080	-618.5148191	-0.1158624	-617.4790240	-617.6743244	-615.0887414	-615.2046038
10	d_G14_17_aah	-618.6287528	0.0884518	-618.5403010	-618.5025953	-0.1261576	#N/A	#N/A	#N/A	#N/A
11	d_G14_17_abw	-618.6295778	0.0893648	-618.5402130	-618.5039304	-0.1256475	-617.4701776	-617.6650549	-615.0798223	-615.2054698
12	d_G14_17_abm	-618.6295734	0.0895514	-618.5400220	-618.5079036	-0.1216699	-617.4743910	-617.6690624	-615.0861263	-615.2077961
13	d_G16_03_abi	-618.6289728	0.0889537	-618.5400190	-618.5062545	-0.1227183	#N/A	#N/A	#N/A	#N/A
14	d_G14_17_aat	-618.6286416	0.0890015	-618.5396400	-618.5033800	-0.1252616	#N/A	#N/A	#N/A	#N/A
15	d_G14_17_abf	-618.6280570	0.0884290	-618.5396280	-618.5007262	-0.1273308	#N/A	#N/A	#N/A	#N/A

16	d_G16_03_aau	-618.6290108	0.0893987	-618.5396120	-618.5088181	-0.1201927	#N/A	#N/A	#N/A	#N/A
17	d_G14_17_aaf	-618.6289385	0.0893304	-618.5396080	-618.5052182	-0.1237203	#N/A	#N/A	#N/A	#N/A
18	d_G16_03_abp	-618.6290024	0.0894393	-618.5395630	-618.5070772	-0.1219251	#N/A	#N/A	#N/A	#N/A
19	d_G14_17_aae	-618.6286828	0.0892038	-618.5394790	-618.5026761	-0.1260067	#N/A	#N/A	#N/A	#N/A
20	d_G14_02_abq	-618.6281863	0.0887473	-618.5394390	-618.5062538	-0.1219325	#N/A	#N/A	#N/A	#N/A
1	d_C56_057	-470.9847234	0.0723803	-470.9123430	-470.8667424	-0.1179809	-470.0748454	-470.2265326	-468.2478308	-468.3658117
2	d_C56_044	-470.9847416	0.0727426	-470.9119990	-470.8573099	-0.1274317	-470.0679538	-470.2193226	-468.2388694	-468.3663011
3	d_C56_037	-470.9837362	0.0721702	-470.9115660	-470.8624352	-0.1213010	-470.0723622	-470.2240462	-468.2465833	-468.3678843
4	d_C56_072	-470.9845624	0.0731204	-470.9114420	-470.8629806	-0.1215819	-470.0729904	-470.2242138	-468.2449408	-468.3665227
5	d_C56_058	-470.9841339	0.0729549	-470.9111790	-470.8663460	-0.1177880	-470.0765949	-470.2278555	-468.2503442	-468.3681322
6	d_C_aac	-470.9842648	0.0731667	-470.9110980	-470.8594123	-0.1248525	-470.0689494	-470.2205719	-468.2405987	-468.3654511
7	d_C_aaw	-470.9841173	0.0732313	-470.9108860	-470.8659354	-0.1181819	-470.0762453	-470.2275315	-468.2499829	-468.3681648
8	d_C56_022	-470.9848502	0.0740202	-470.9108300	-470.8675376	-0.1173126	-470.0757728	-470.2274008	-468.2486893	-468.3660019
9	d_C56_059	-470.9840634	0.0733254	-470.9107380	-470.8663489	-0.1177145	#N/A	#N/A	#N/A	#N/A
10	d_C56_047	-470.9848267	0.0743496	-470.9104770	-470.8673514	-0.1174753	#N/A	#N/A	#N/A	#N/A
11	d_C_aab	-470.9838967	0.0736596	-470.9102370	-470.8634461	-0.1204506	-470.0732864	-470.2249803	-468.2475890	-468.3680395
12	d_C56_016	-470.9847330	0.0746370	-470.9100960	-470.8571650	-0.1275680	#N/A	#N/A	#N/A	#N/A
13	d_C56_032	-470.9847668	0.0747988	-470.9099680	-470.8570330	-0.1277338	#N/A	#N/A	#N/A	#N/A
14	d_C_abu	-470.9830417	0.0731247	-470.9099170	-470.8604405	-0.1226012	-470.0707520	-470.2225659	-468.2458374	-468.3684386
15	d_C56_082	-470.9797286	0.0700715	-470.9096570	-470.8527356	-0.1269929	#N/A	#N/A	#N/A	#N/A
16	d_C56_079	-470.9794016	0.0706025	-470.9087990	-470.8490824	-0.1303192	#N/A	#N/A	#N/A	#N/A
17	d_C56_089	-470.9791545	0.0712275	-470.9079270	-470.8466493	-0.1325052	#N/A	#N/A	#N/A	#N/A
18	d_C56_023	-470.9803226	0.0725166	-470.9078060	-470.8549082	-0.1254145	#N/A	#N/A	#N/A	#N/A
19	d_C56_014	-470.9799180	0.0724389	-470.9074790	-470.8531150	-0.1268030	#N/A	#N/A	#N/A	#N/A
20	d_C56_078	-470.9798376	0.0723976	-470.9074400	-470.8525132	-0.1273244	#N/A	#N/A	#N/A	#N/A
1	d_U4_aba_b	-490.8678169	0.0602799	-490.8075370	-490.7665507	-0.1012663	#N/A	#N/A	#N/A	#N/A
2	d_U4_abs	-490.8681378	0.0606907	-490.8074470	-490.7717289	-0.0964089	-489.9703809	-490.1280403	-488.1293574	-488.2257663
3	d_U4_aay	-490.8674121	0.0607851	-490.8066270	-490.7663589	-0.1010532	-489.9674974	-490.1247546	-488.1269081	-488.2279612
4	d_U4_adq	-490.8678282	0.0613282	-490.8065000	-490.7661337	-0.1016945	-489.9658965	-490.1235474	-488.1238970	-488.2255915
5	d_U4_acf	-490.8677709	0.0612789	-490.8064920	-490.7665845	-0.1011864	-489.9671819	-490.1245034	-488.1261290	-488.2273153
6	d_U4_aac	-490.8679092	0.0616042	-490.8063050	-490.7670705	-0.1008387	-489.9669909	-490.1245810	-488.1251196	-488.2259584
7	d_U4_abx	-490.8681348	0.0619267	-490.8062080	-490.7716010	-0.0965337	-489.9703572	-490.1279847	-488.1292844	-488.2258182
8	d_U4_abp	-490.8679791	0.0617881	-490.8061910	-490.7682625	-0.0997166	-489.9679681	-490.1255446	-488.1263832	-488.2260999
9	d_U4_abt	-490.8672207	0.0611196	-490.8061010	-490.7668915	-0.1003291	#N/A	#N/A	#N/A	#N/A
10	d_U4_act	-490.8672481	0.0612601	-490.8059880	-490.7666919	-0.1005562	#N/A	#N/A	#N/A	#N/A
11	d_U4_aam	-490.8673929	0.0614779	-490.8059150	-490.7672077	-0.1001853	#N/A	#N/A	#N/A	#N/A
12	d_U4_aaq	-490.8672364	0.0613293	-490.8059070	-490.7667447	-0.1004917	#N/A	#N/A	#N/A	#N/A
13	d_U4_aci	-490.8672245	0.0614154	-490.8058090	-490.7657475	-0.1014770	#N/A	#N/A	#N/A	#N/A
14	d_U4_abk	-490.8668084	0.0610403	-490.8057680	-490.7662720	-0.1005363	#N/A	#N/A	#N/A	#N/A
15	d_U4_abz	-490.8672697	0.0616307	-490.8056390	-490.7666886	-0.1005811	#N/A	#N/A	#N/A	#N/A
16	d_U4_abq_b	-490.8671269	0.0615879	-490.8055390	-490.7700638	-0.0970631	#N/A	#N/A	#N/A	#N/A
17	d_U4_aap	-490.8679030	0.0627980	-490.8051050	-490.7656954	-0.1022076	-489.9669289	-490.1242459	-488.1258099	-488.2280176
18	d_U4_acm	-490.8676802	0.0626192	-490.8050610	-490.7650878	-0.1025925	#N/A	#N/A	#N/A	#N/A
19	d_U4_acu	-490.8679057	0.0629697	-490.8049360	-490.7658602	-0.1020455	#N/A	#N/A	#N/A	#N/A
20	d_U4_abv	-490.8671445	0.0622415	-490.8049030	-490.7699110	-0.0972336	#N/A	#N/A	#N/A	#N/A
1	d_1mG43_abd	-657.9374572	0.1155972	-657.8218600	-657.8199544	-0.1175028	-656.7079273	-656.9131115	-654.1187179	-654.2362207
2	d_1mG43_aar	-657.9382720	0.1167700	-657.8215020	-657.8232595	-0.1150125	-656.7105842	-656.9156076	-654.1210557	-654.2360682
3	d_1mG43_aay	-657.9367948	0.1153987	-657.8213960	-657.8188752	-0.1179196	-656.7067354	-656.9118172	-654.1174047	-654.2353243

4	d_1mG43_abn	-657.9387820	0.1174420	-657.8213400	-657.8258925	-0.1128896	-656.7136945	-656.9183243	-654.1221837	-654.2350733
5	d_1mG43_aai	-657.9374907	0.1164606	-657.8210300	-657.8212534	-0.1162373	-656.7088747	-656.9140038	-654.1197079	-654.2359452
6	d_1mG43_aas	-657.9375388	0.1165268	-657.8210120	-657.8164381	-0.1211007	-656.7046455	-656.9098638	-654.1146415	-654.2357422
7	d_1mG43_aax	-657.9362503	0.1158373	-657.8204130	-657.8171249	-0.1191255	#N/A	#N/A	#N/A	#N/A
8	d_1mG43_aal	-657.9363623	0.1160403	-657.8203220	-657.8171178	-0.1192446	-656.7057822	-656.9110034	-654.1180744	-654.2373190
9	d_1mG43_abk	-657.9363271	0.1161881	-657.8201390	-657.8171659	-0.1191612	-656.7057929	-656.9110276	-654.1181407	-654.2373019
10	d_1mG43_abc	-657.9342187	0.1141667	-657.8200520	-657.8085216	-0.1256971	#N/A	#N/A	#N/A	#N/A
11	d_1mG43_aad	-657.9357597	0.1161247	-657.8196350	-657.8192622	-0.1164975	#N/A	#N/A	#N/A	#N/A
12	d_1mG43_aah	-657.9355165	0.1160904	-657.8194260	-657.8116914	-0.1238251	#N/A	#N/A	#N/A	#N/A
13	d_1mG43_abe	-657.9369340	0.1183070	-657.8186270	-657.8127526	-0.1241814	-656.7028573	-656.9075713	-654.1133217	-654.2375031
14	d_1mG43_aac	-657.9365261	0.1179140	-657.8186120	-657.8119557	-0.1245703	-656.7022110	-656.9069038	-654.1127515	-654.2373219
15	d_1mG43_aba	-657.9354647	0.1170116	-657.8184530	-657.8129695	-0.1224951	#N/A	#N/A	#N/A	#N/A
16	d_1mG43_abp	-657.9353165	0.1169705	-657.8183460	-657.8123874	-0.1229292	#N/A	#N/A	#N/A	#N/A
17	d_1mG43_abu	-657.9356876	0.1173626	-657.8183250	-657.8101552	-0.1255324	#N/A	#N/A	#N/A	#N/A
18	d_1mG43_abf	-657.9338265	0.1156505	-657.8181760	-657.8082918	-0.1255347	#N/A	#N/A	#N/A	#N/A
19	d_1mG43_abr	-657.9342503	0.1163423	-657.8179080	-657.8096393	-0.1246110	#N/A	#N/A	#N/A	#N/A
20	d_1mG43_aao	-657.9338025	0.1163055	-657.8174970	-657.8103729	-0.1234296	#N/A	#N/A	#N/A	#N/A
1	d_X11_abv	-638.5127356	0.0772196	-638.4355160	-638.4036541	-0.1090815	-637.3584896	-637.5595020	-634.9546859	-635.0637674
2	d_X11_aay	-638.5124833	0.0770912	-638.4353920	-638.3988807	-0.1136026	-637.3550876	-637.5560952	-634.9522843	-635.0658869
3	d_X11_abc	-638.5128715	0.0775115	-638.4353600	-638.4036277	-0.1092438	-637.3586951	-637.5596510	-634.9547649	-635.0640087
4	d_X05_aan	-638.5107700	0.0760659	-638.4347040	-638.3970004	-0.1137696	#N/A	#N/A	#N/A	#N/A
5	d_X16_aav	-638.5111577	0.0766776	-638.4344800	-638.4086870	-0.1024707	-637.3662824	-637.5667280	-634.9614818	-635.0639524
6	d_X11_abs	-638.5134129	0.0789978	-638.4344150	-638.3977701	-0.1156427	-637.3551160	-637.5558331	-634.9508370	-635.0664797
7	d_X16_aaa	-638.5109699	0.0767849	-638.4341850	-638.4108283	-0.1001416	#N/A	#N/A	#N/A	#N/A
8	d_X11_aac	-638.5137300	0.0796639	-638.4340660	-638.4047733	-0.1089567	-637.3600847	-637.5605219	-634.9534756	-635.0624323
9	d_X11_aab	-638.5109995	0.0769415	-638.4340580	-638.3945383	-0.1164612	#N/A	#N/A	#N/A	#N/A
10	d_X11_aax	-638.5129379	0.0792488	-638.4336890	-638.3965955	-0.1163423	-637.3537752	-637.5542918	-634.9496836	-635.0660260
11	d_X11_aal	-638.5099232	0.0763442	-638.4335790	-638.3806184	-0.1293049	#N/A	#N/A	#N/A	#N/A
12	d_X11_abj	-638.5099196	0.0763775	-638.4335420	-638.3796952	-0.1302244	#N/A	#N/A	#N/A	#N/A
13	d_X05_aav	-638.5123171	0.0789860	-638.4333310	-638.3999314	-0.1123857	-637.3556287	-637.5563261	-634.9489074	-635.0612931
14	d_X05_abi	-638.5094409	0.0761368	-638.4333040	-638.3946738	-0.1147671	#N/A	#N/A	#N/A	#N/A
15	d_X11_aah	-638.5103146	0.0770275	-638.4332870	-638.3925184	-0.1177962	#N/A	#N/A	#N/A	#N/A
16	d_X16_aba	-638.5074958	0.0742528	-638.4332430	-638.3994190	-0.1080769	#N/A	#N/A	#N/A	#N/A
17	d_X16_aau	-638.5125936	0.0793936	-638.4332000	-638.4164430	-0.0961507	-637.3733876	-637.5733189	-634.9670509	-635.0632015
18	d_X05_abv	-638.5104382	0.0772802	-638.4331580	-638.3941437	-0.1162945	#N/A	#N/A	#N/A	#N/A
19	d_X16_aas	-638.5102144	0.0771793	-638.4330350	-638.4043266	-0.1058878	#N/A	#N/A	#N/A	#N/A
20	d_X16_abl	-638.5081776	0.0754296	-638.4327480	-638.3935109	-0.1146667	#N/A	#N/A	#N/A	#N/A
1	d_hX12_e_aad	-563.2500304	0.0722313	-563.1777990	-563.1415167	-0.1085136	-562.1976700	-562.3734687	-560.0226194	-560.1311330
2	d_hX12_e_aax	-563.2511048	0.0745888	-563.1765160	-563.1437614	-0.1073435	-562.1996898	-562.3753356	-560.0244284	-560.1317719
3	d_hX12_e_aba	-563.2487226	0.0727096	-563.1760130	-563.1363057	-0.1124169	#N/A	#N/A	#N/A	#N/A
4	d_hX12_e_aao	-563.2501217	0.0742727	-563.1758490	-563.1395595	-0.1105622	-562.1962042	-562.3720280	-560.0210879	-560.1316500
5	d_hX12_e_aah	-563.2516448	0.0761077	-563.1755370	-563.1463073	-0.1053375	-562.2027878	-562.3780848	-560.0254943	-560.1308317
6	d_hX12_e_aae	-563.2500282	0.0745012	-563.1755270	-563.1416632	-0.1083650	-562.1978604	-562.3736264	-560.0227877	-560.1311527
7	d_hX12_e_abe	-563.2501591	0.0746931	-563.1754660	-563.1399898	-0.1101693	-562.1966119	-562.3724410	-560.0215159	-560.1316853
8	d_hX12_e_abm	-563.2501609	0.0747548	-563.1754060	-563.1368986	-0.1132623	-562.1937538	-562.3695579	-560.0179612	-560.1312235
9	d_hX12_e_aav	-563.2501567	0.0748167	-563.1753400	-563.1402246	-0.1099321	-562.1967480	-562.3726015	-560.0216906	-560.1316227
10	d_hX12_e_aaf	-563.2501292	0.0748922	-563.1752370	-563.1405705	-0.1095588	-562.1969525	-562.3728127	-560.0219127	-560.1314715
11	d_hX13_b_abm	-563.2510327	0.0761387	-563.1748940	-563.1441795	-0.1068532	-562.2006808	-562.3763853	-560.0257067	-560.1325598

12	d_hX13_b_aaj	-563.2479800	0.0734340	-563.1745460	-563.1336340	-0.1143461	#N/A	#N/A	#N/A	#N/A
13	d_hX13_b_abc	-563.2484161	0.0739100	-563.1745060	-563.1330392	-0.1153769	#N/A	#N/A	#N/A	#N/A
14	d_hX12_e_aal	-563.2495201	0.0751461	-563.1743740	-563.1390526	-0.1104675	#N/A	#N/A	#N/A	#N/A
15	d_hX12_e_aam	-563.2495897	0.0753416	-563.1742480	-563.1392014	-0.1103883	#N/A	#N/A	#N/A	#N/A
16	d_hX12_e_aar	-563.2487384	0.0744953	-563.1742430	-563.1377964	-0.1109420	#N/A	#N/A	#N/A	#N/A
17	d_hX13_b_abq	-563.2477272	0.0736221	-563.1741050	-563.1354101	-0.1123171	#N/A	#N/A	#N/A	#N/A
18	d_hX13_b_aau	-563.2477566	0.0737375	-563.1740190	-563.1347624	-0.1129942	#N/A	#N/A	#N/A	#N/A
19	d_hX12_e_abb	-563.2465644	0.0726923	-563.1738720	-563.1317476	-0.1148168	#N/A	#N/A	#N/A	#N/A
20	d_hX13_b_aal	-563.2477513	0.0739102	-563.1738410	-563.1351774	-0.1125739	#N/A	#N/A	#N/A	#N/A
1	d_PhOH_abc	-383.4940586	0.0808485	-383.4132100	-383.3973128	-0.0967458	-382.7118264	-382.8342675	-381.1551506	-381.2518964
2	d_PhOH_aao	-383.4940596	0.0809275	-383.4131320	-383.3974888	-0.0965707	-382.7124343	-382.8347173	-381.1555398	-381.2521105
3	d_PhOH_aba	-383.4940563	0.0810172	-383.4130390	-383.3974047	-0.0966516	-382.7120963	-382.8344877	-381.1553186	-381.2519703
4	d_PhOH_abv	-383.4945702	0.0817982	-383.4127720	-383.3967134	-0.0978569	-382.7122573	-382.8341736	-381.1543441	-381.2522009
5	d_PhOH_aah	-383.4881961	0.0792411	-383.4089550	-383.3866879	-0.1015082	-382.7021117	-382.8244078	-381.1457131	-381.2472213
6	d_PhOH_abq	-383.4881035	0.0791955	-383.4089080	-383.3864091	-0.1016944	-382.7018598	-382.8241702	-381.1456254	-381.2473198
7	d_PhOH_abw	-383.4882095	0.0796394	-383.4085700	-383.3853165	-0.1028930	-382.7004747	-382.8229756	-381.1441569	-381.2470499
8	d_PhOH_aaw	-383.4881829	0.0796908	-383.4084920	-383.3854611	-0.1027218	-382.7008495	-382.8230571	-381.1441376	-381.2468593
9	d_PhOH_abh	-383.4882475	0.0803705	-383.4078770	-383.3852761	-0.1029714	-382.7006852	-382.8229104	-381.1438734	-381.2468448
10	d_PhOH_abm	-383.4882307	0.0804637	-383.4077670	-383.3868101	-0.1014206	-382.7021807	-382.8244872	-381.1458622	-381.2472828
1	d_Ind_abi	-439.8308564	0.1029043	-439.7279520	-439.7448797	-0.0859767	-438.9245307	-439.0595786	-437.0702266	-437.1562032
2	d_Ind_abq	-439.8312266	0.1044206	-439.7268060	-439.7452484	-0.0859782	-438.9255330	-439.0601634	-437.0703557	-437.1563339
3	d_Ind_aam	-439.8262869	0.1034978	-439.7227890	-439.7371272	-0.0891597	#N/A	#N/A	#N/A	#N/A
4	d_Ind_aan	-439.8250317	0.1022966	-439.7227350	-439.7350682	-0.0899635	#N/A	#N/A	#N/A	#N/A
5	d_Ind_abv	-439.8259927	0.1033587	-439.7226340	-439.7359437	-0.0900490	#N/A	#N/A	#N/A	#N/A
6	d_Ind_abd	-439.8263013	0.1038532	-439.7224480	-439.7371734	-0.0891279	-438.9194230	-439.0539218	-437.0660527	-437.1551806
7	d_Ind_aai	-439.8259798	0.1035558	-439.7224240	-439.7351013	-0.0908785	#N/A	#N/A	#N/A	#N/A
8	d_Ind_abe	-439.8263002	0.1039662	-439.7223340	-439.7371363	-0.0891640	#N/A	#N/A	#N/A	#N/A
9	d_Ind_abw	-439.8250244	0.1028063	-439.7222180	-439.7349617	-0.0900627	#N/A	#N/A	#N/A	#N/A
10	d_Ind_aap	-439.8252278	0.1038597	-439.7213680	-439.7364834	-0.0887444	#N/A	#N/A	#N/A	#N/A
11	d_Ind_abr	-439.8253483	0.1040383	-439.7213100	-439.7365111	-0.0888373	#N/A	#N/A	#N/A	#N/A
12	d_Ind_abl	-439.8190672	0.0987741	-439.7202930	-439.7125049	-0.1065622	#N/A	#N/A	#N/A	#N/A
13	d_Ind_aae	-439.8219240	0.1040940	-439.7178300	-439.7279590	-0.0939650	#N/A	#N/A	#N/A	#N/A
1	rd_A_aba	-543.1821410	0.0848140	-543.0973270	-543.1383284	-0.0438126	-542.2084165	-542.3711533	-540.0859467	-540.1297593
2	rd_A_aaw	-543.1852362	0.0883371	-543.0968990	-543.1510892	-0.0341470	-542.2219764	-542.3833438	-540.0957590	-540.1299060
3	rd_A_abn	-543.1814914	0.0850014	-543.0964900	-543.1369254	-0.0445660	-542.2089481	-542.3706341	-540.0855645	-540.1301305
4	rd_A_abp	-543.1820623	0.0859982	-543.0960640	-543.1410787	-0.0409836	-542.2127774	-542.3743666	-540.0900002	-540.1309838
5	rd_A_aao	-543.1785013	0.0824742	-543.0960270	-543.1311546	-0.0473467	-542.2029025	-542.3655592	-540.0818414	-540.1291881
6	rd_A_abk	-543.1810781	0.0852781	-543.0958000	-543.1306328	-0.0504453	-542.2028886	-542.3648577	-540.0792422	-540.1296875
7	rd_A_aah	-543.1784464	0.0829073	-543.0955390	-543.1286914	-0.0497550	#N/A	#N/A	#N/A	#N/A
8	rd_A_aal	-543.1784440	0.0829159	-543.0955280	-543.1290141	-0.0494299	#N/A	#N/A	#N/A	#N/A
9	rd_A_aas	-543.1793358	0.0842677	-543.0950680	-543.1299030	-0.0494328	-542.2021001	-542.3643524	-540.0800431	-540.1294759
10	rd_A_aam	-543.1814710	0.0865620	-543.0949090	-543.1306201	-0.0508509	-542.2032337	-542.3650934	-540.0791815	-540.1300324
11	rd_A_abl	-543.1817195	0.0869335	-543.0947860	-543.1311677	-0.0505518	-542.2036961	-542.3656081	-540.0797222	-540.1302740
12	rd_A_aac	-543.1794297	0.0854147	-543.0940150	-543.1302807	-0.0491490	-542.2024059	-542.3646682	-540.0805054	-540.1296544
13	rd_A_aag	-543.1782161	0.0844091	-543.0938070	-543.1271920	-0.0510242	#N/A	#N/A	#N/A	#N/A
1	rd_5mU4_aab	-529.9955530	0.0848279	-529.9107250	-529.9620405	-0.0335125	-529.0916703	-529.2522391	-527.0765738	-527.1100863
2	rd_5mU4_abe	-529.9956074	0.0849114	-529.9106960	-529.9625769	-0.0330305	-529.0921033	-529.2527223	-527.0770477	-527.1100782
3	rd_5mU4_abj	-529.9955720	0.0852120	-529.9103600	-529.9658731	-0.0296989	-529.0954006	-529.2560553	-527.0805050	-527.1102039

4	rd_5mU4_aao	-529.9953713	0.0851983	-529.9101730	-529.9614536	-0.0339177	-529.0891584	-529.2509758	-527.0744695	-527.1083872
5	rd_5mU4_aay	-529.9949777	0.0851436	-529.9098340	-529.9612429	-0.0337348	-529.0886088	-529.2501577	-527.0740839	-527.1078188
6	rd_5mU4_aaw	-529.9949953	0.0851973	-529.9097980	-529.9591380	-0.0358573	-529.0873686	-529.2490097	-527.0746696	-527.1105269
7	rd_5mU4_abx	-529.9953397	0.0855817	-529.9097580	-529.9612778	-0.0340620	-529.0890775	-529.2508581	-527.0743459	-527.1084078
8	rd_5mU4_abr	-529.9959357	0.0866307	-529.9093050	-529.9552113	-0.0407245	-529.0846041	-529.2460338	-527.0707353	-527.1114598
9	rd_5mU4_aap	-529.9950481	0.0859661	-529.9090820	-529.9592750	-0.0357732	-529.0874509	-529.2490887	-527.0747207	-527.1104938
10	rd_5mU4_aar	-529.9959698	0.0868898	-529.9090800	-529.9557039	-0.0402659	-529.0851338	-529.2464998	-527.0693595	-527.1096253
11	rd_5mU4_abl	-529.9950713	0.0860883	-529.9089830	-529.9593384	-0.0357330	-529.0875058	-529.2491364	-527.0747490	-527.1104819
12	rd_5mU4_abc	-529.9949059	0.0860678	-529.9088380	-529.9590803	-0.0358255	#N/A	#N/A	#N/A	#N/A
13	rd_5mU4_abi	-529.9936616	0.0854235	-529.9082380	-529.9494067	-0.0442549	#N/A	#N/A	#N/A	#N/A
14	rd_5mU4_aaz	-529.9911248	0.0829688	-529.9081560	-529.9497663	-0.0413585	#N/A	#N/A	#N/A	#N/A
15	rd_5mU4_aam	-529.9911868	0.0834088	-529.9077780	-529.9512394	-0.0399474	#N/A	#N/A	#N/A	#N/A
16	rd_5mU4_aag	-529.9946267	0.0872126	-529.9074140	-529.9523414	-0.0422852	#N/A	#N/A	#N/A	#N/A
1	rd_G16_03_abv	-618.4479924	0.0879864	-618.3600060	-618.3915729	-0.0564195	-617.3648586	-617.5510776	-615.0076124	-615.0640319
2	rd_G16_03_aba	-618.4479167	0.0881437	-618.3597730	-618.3903628	-0.0575539	-617.3637358	-617.5500208	-615.0064469	-615.0640008
3	rd_G16_03_aaw	-618.4497966	0.0900296	-618.3597670	-618.3877952	-0.0620014	-617.3613917	-617.5476720	-615.0029544	-615.0649558
4	rd_G16_03_aan	-618.4493502	0.0896192	-618.3597310	-618.3899654	-0.0593848	-617.3641108	-617.5498555	-615.0059519	-615.0653367
5	rd_G16_03_aag	-618.4480767	0.0884097	-618.3596670	-618.3888001	-0.0592766	-617.3605348	-617.5478038	-615.0026973	-615.0619739
6	rd_G16_03_aah	-618.4477388	0.0881397	-618.3595990	-618.3868140	-0.0609248	#N/A	#N/A	#N/A	#N/A
7	rd_G16_03_aat	-618.4481571	0.0886600	-618.3594970	-618.3909767	-0.0571803	-617.3637278	-617.5505251	-615.0089252	-615.0661055
8	rd_G16_03_abx	-618.4481128	0.0886188	-618.3594940	-618.3885412	-0.0595716	-617.3604991	-617.5477372	-615.0024883	-615.0620599
9	rd_G16_03_aab	-618.4481213	0.0886902	-618.3594310	-618.3885525	-0.0595688	-617.3605306	-617.5477615	-615.0025233	-615.0620921
10	rd_G16_03_abm	-618.4481785	0.0890354	-618.3591430	-618.3915236	-0.0566549	-617.3644143	-617.5511249	-615.0094649	-615.0661198
11	rd_G16_03_aai	-618.4463356	0.0873325	-618.3590030	-618.3834388	-0.0628968	#N/A	#N/A	#N/A	#N/A
12	rd_G14_02_aaa	-618.4479111	0.0890880	-618.3588230	-618.4042622	-0.0436489	#N/A	#N/A	#N/A	#N/A
13	rd_G16_03_abl	-618.4491395	0.0904245	-618.3587150	-618.3905689	-0.0585706	-617.3620589	-617.5488455	-615.0037010	-615.0622716
14	rd_G16_03_abf	-618.4474938	0.0891308	-618.3583630	-618.3916101	-0.0558837	#N/A	#N/A	#N/A	#N/A
15	rd_G14_17_aal	-618.4465733	0.0883662	-618.3582070	-618.3916089	-0.0549644	#N/A	#N/A	#N/A	#N/A
16	rd_G14_17_aao	-618.4459652	0.0880342	-618.3579310	-618.3867282	-0.0592371	#N/A	#N/A	#N/A	#N/A
17	rd_G16_03_aax	-618.4449867	0.0871487	-618.3578380	-618.3831576	-0.0618291	#N/A	#N/A	#N/A	#N/A
18	rd_G14_17_abb	-618.4465445	0.0887614	-618.3577830	-618.3911187	-0.0554258	#N/A	#N/A	#N/A	#N/A
19	rd_G14_17_abh	-618.4460085	0.0882764	-618.3577320	-618.3878149	-0.0581936	#N/A	#N/A	#N/A	#N/A
20	rd_G14_17_aat	-618.4447463	0.0871682	-618.3575780	-618.3858845	-0.0588617	#N/A	#N/A	#N/A	#N/A
1	rd_C_aau	-470.7898725	0.0709655	-470.7189070	-470.7407023	-0.0491703	-469.9588619	-470.1025492	-468.1419903	-468.1911606
2	rd_C_aba	-470.7898734	0.0709924	-470.7188810	-470.7406358	-0.0492377	-469.9587358	-470.1024561	-468.1419102	-468.1911479
3	rd_C56_050	-470.7907394	0.0720713	-470.7186680	-470.7454758	-0.0452636	-469.9624547	-470.1061149	-468.1447789	-468.1900424
4	rd_C_aap	-470.7896122	0.0711161	-470.7184960	-470.7411366	-0.0484756	-469.9592483	-470.1029391	-468.1419564	-468.1904320
5	rd_C56_013	-470.7897422	0.0713721	-470.7183700	-470.7413809	-0.0483612	-469.9590277	-470.1029223	-468.1426992	-468.1910604
6	rd_C46_049	-470.7902496	0.0719285	-470.7183210	-470.7493496	-0.0409000	-469.9685731	-470.1124014	-468.1573959	-468.1982959
7	rd_C56_033	-470.7906511	0.0729730	-470.7176780	-470.7372252	-0.0534258	#N/A	#N/A	#N/A	#N/A
8	rd_C56_053	-470.7898594	0.0721813	-470.7176780	-470.7405798	-0.0492796	-469.9588455	-470.1025248	-468.1419915	-468.1912711
9	rd_C56_097	-470.7882596	0.0706495	-470.7176100	-470.7430772	-0.0451824	#N/A	#N/A	#N/A	#N/A
10	rd_C46_024	-470.7900610	0.0724970	-470.7175640	-470.7499828	-0.0400783	#N/A	#N/A	#N/A	#N/A
11	rd_C46_013	-470.7906445	0.0730934	-470.7175510	-470.7454720	-0.0451725	#N/A	#N/A	#N/A	#N/A
12	rd_C56_017	-470.7854291	0.0680100	-470.7174190	-470.7338177	-0.0516114	#N/A	#N/A	#N/A	#N/A
13	rd_C46_041	-470.7890001	0.0715820	-470.7174180	-470.7484159	-0.0405842	#N/A	#N/A	#N/A	#N/A
14	rd_C46_058	-470.7906396	0.0732445	-470.7173950	-470.7459434	-0.0446962	#N/A	#N/A	#N/A	#N/A
15	rd_C_aaw	-470.7880883	0.0707243	-470.7173640	-470.7403176	-0.0477708	#N/A	#N/A	#N/A	#N/A

16	rd_C_aal	-470.7882290	0.0708730	-470.7173560	-470.7427940	-0.0454350	-469.9622523	-470.1050611	-468.1447031	-468.1901382
17	rd_C56_065	-470.7896255	0.0723735	-470.7172520	-470.7409799	-0.0486456	#N/A	#N/A	#N/A	#N/A
18	rd_C_abw	-470.7881811	0.0709571	-470.7172240	-470.7421038	-0.0460773	#N/A	#N/A	#N/A	#N/A
19	rd_C46_011	-470.7899767	0.0728446	-470.7171320	-470.7513774	-0.0385992	#N/A	#N/A	#N/A	#N/A
20	rd_C56_019	-470.7887939	0.0716628	-470.7171310	-470.7426317	-0.0461622	#N/A	#N/A	#N/A	#N/A
1	rd_U4_aan	-490.6591416	0.0549946	-490.6041470	-490.6209365	-0.0382052	-489.8324700	-489.9823507	-488.0121801	-488.0503853
2	rd_U4_adc	-490.6629192	0.0588171	-490.6041020	-490.6269493	-0.0359699	-489.8390698	-489.9885829	-488.0157252	-488.0516951
3	rd_U4_abj	-490.6628659	0.0595808	-490.6032850	-490.6282806	-0.0345853	-489.8389348	-489.9888663	-488.0164358	-488.0510211
4	rd_U4_abs	-490.6620087	0.0587616	-490.6032470	-490.6284724	-0.0335362	-489.8383507	-489.9881028	-488.0160356	-488.0495718
5	rd_U4_abp	-490.6632460	0.0602840	-490.6029620	-490.6299583	-0.0332878	-489.8421180	-489.9909081	-488.0180755	-488.0513632
6	rd_U4_aai	-490.6634367	0.0604817	-490.6029550	-490.6307728	-0.0326639	-489.8423789	-489.9912987	-488.0194376	-488.0521015
7	rd_U4_abi	-490.6630245	0.0600805	-490.6029440	-490.6287378	-0.0342868	-489.8405439	-489.9898960	-488.0187746	-488.0530614
8	rd_U4_aaq	-490.6630783	0.0602293	-490.6028490	-490.6334795	-0.0295989	-489.8452683	-489.9941295	-488.0222673	-488.0518661
9	rd_U4_aam	-490.6628791	0.0600850	-490.6027940	-490.6292092	-0.0336699	-489.8394148	-489.9893394	-488.0188430	-488.0525129
10	rd_U4_abc	-490.6629562	0.0601682	-490.6027880	-490.6294641	-0.0334921	-489.8402207	-489.9899292	-488.0192375	-488.0527296
11	rd_U4_abt	-490.6627606	0.0601116	-490.6026490	-490.6287659	-0.0339947	-489.8397717	-489.9895375	-488.0176411	-488.0516358
12	rd_U4_aak	-490.6620769	0.0595059	-490.6025710	-490.6259689	-0.0361080	#N/A	#N/A	#N/A	#N/A
13	rd_U4_adt	-490.6631925	0.0606715	-490.6025210	-490.6224820	-0.0407105	-489.8346293	-489.9842358	-488.0110351	-488.0517456
14	rd_U4_aah	-490.6620996	0.0597096	-490.6023900	-490.6263883	-0.0357113	-489.8367554	-489.9867750	-488.0166142	-488.0523255
15	rd_U4_aap	-490.6632780	0.0609980	-490.6022800	-490.6223733	-0.0409047	-489.8341382	-489.9838140	-488.0120180	-488.0529227
16	rd_U4_abv	-490.6623387	0.0601406	-490.6021980	-490.6265491	-0.0357896	#N/A	#N/A	#N/A	#N/A
17	rd_U4_aay	-490.6628021	0.0606981	-490.6021040	-490.6290922	-0.0337099	-489.8394977	-489.9894772	-488.0177374	-488.0514473
18	rd_U4_adq	-490.6617320	0.0600340	-490.6016980	-490.6242123	-0.0375197	#N/A	#N/A	#N/A	#N/A
19	rd_U4_aaa	-490.6591218	0.0574818	-490.6016400	-490.6185248	-0.0405970	#N/A	#N/A	#N/A	#N/A
20	rd_U4_ads	-490.6632675	0.0616334	-490.6016340	-490.6230924	-0.0401751	-489.8350429	-489.9846381	-488.0127883	-488.0529633
1	rd_X11_abg	-638.3230606	0.0756286	-638.2474320	-638.2759561	-0.0471046	-637.2411275	-637.4332873	-634.8571402	-634.9042448
2	rd_X11_abc	-638.3231126	0.0759346	-638.2471780	-638.2761444	-0.0469682	-637.2413197	-637.4334458	-634.8573846	-634.9043528
3	rd_X11_aba	-638.3229557	0.0758466	-638.2471090	-638.2733638	-0.0495919	-637.2387774	-637.4310007	-634.8540556	-634.9036476
4	rd_X11_aah	-638.3230204	0.0761363	-638.2468840	-638.2754129	-0.0476075	-637.2406218	-637.4328192	-634.8564673	-634.9040748
5	rd_X11_abs	-638.3211430	0.0750300	-638.2461130	-638.2704764	-0.0506666	-637.2360594	-637.4282027	-634.8505205	-634.9011871
6	rd_X11_abb	-638.3212712	0.0757101	-638.2455610	-638.2717710	-0.0495002	-637.2371091	-637.4293413	-634.8519500	-634.9014501
7	rd_X11_aas	-638.3212516	0.0760315	-638.2452200	-638.2714829	-0.0497687	-637.2369476	-637.4291554	-634.8524618	-634.9022305
8	rd_X11_aat	-638.3212461	0.0769811	-638.2442650	-638.2708777	-0.0503684	-637.2361624	-637.4288407	-634.8514490	-634.9018174
9	rd_X11_aao	-638.3212530	0.0771869	-638.2440660	-638.2743243	-0.0469287	-637.2401574	-637.4324051	-634.8561082	-634.9030369
10	rd_X11_aaz	-638.3203396	0.0762755	-638.2440640	-638.2693578	-0.0509818	-637.2335421	-637.4267561	-634.8496863	-634.9006681
11	rd_X11_abl	-638.3202238	0.0762127	-638.2440110	-638.2690854	-0.0511384	#N/A	#N/A	#N/A	#N/A
12	rd_X11_abv	-638.3187189	0.0750608	-638.2436580	-638.2659877	-0.0527312	#N/A	#N/A	#N/A	#N/A
13	rd_X05_abu	-638.3204799	0.0768688	-638.2436110	-638.2749777	-0.0455022	#N/A	#N/A	#N/A	#N/A
14	rd_X11_abw	-638.3199447	0.0768647	-638.2430800	-638.2622090	-0.0577358	#N/A	#N/A	#N/A	#N/A
15	rd_X05_abk	-638.3214047	0.0783547	-638.2430500	-638.2801395	-0.0412652	-637.2449097	-637.4371204	-634.8533845	-634.8946498
16	rd_X11_abp	-638.3202595	0.0772565	-638.2430030	-638.2619123	-0.0583473	#N/A	#N/A	#N/A	#N/A
17	rd_X11_aan	-638.3204150	0.0776670	-638.2427480	-638.2628930	-0.0575220	#N/A	#N/A	#N/A	#N/A
18	rd_X16_aae	-638.3174217	0.0750016	-638.2424200	-638.2753768	-0.0420448	#N/A	#N/A	#N/A	#N/A
19	rd_X05_abt	-638.3175601	0.0751571	-638.2424030	-638.2664809	-0.0510792	#N/A	#N/A	#N/A	#N/A
20	rd_X11_aaf	-638.3182096	0.0759696	-638.2422400	-638.2593138	-0.0588958	#N/A	#N/A	#N/A	#N/A
1	rd_hX12_e_aab	-563.0515496	0.0718795	-562.9796700	-563.0081744	-0.0433752	-562.0728345	-562.2404050	-559.9263407	-559.9697158
2	rd_hX12_e_abn	-563.0514912	0.0724432	-562.9790480	-563.0080103	-0.0434810	-562.0727411	-562.2403179	-559.9260750	-559.9695560
3	rd_hX12_e_aad	-563.0514459	0.0727539	-562.9786920	-563.0049070	-0.0465389	-562.0698537	-562.2375598	-559.9219366	-559.9684755

4	rd_hX12_e_abi	-563.0514530	0.0728930	-562.9785600	-563.0068654	-0.0445876	-562.0715925	-562.2392751	-559.9245439	-559.9691315
5	rd_hX12_e_abs	-563.0514226	0.0730116	-562.9784110	-563.0036946	-0.0477280	-562.0690890	-562.2366091	-559.9203629	-559.9680910
6	rd_hX12_e_abg	-563.0514338	0.0732797	-562.9781540	-563.0057812	-0.0456525	-562.0706363	-562.2383223	-559.9231120	-559.9687645
7	rd_hX12_e_aag	-563.0494198	0.0713857	-562.9780340	-563.0026104	-0.0468094	-562.0660830	-562.2348297	-559.9194137	-559.9662231
8	rd_hX12_e_aao	-563.0494207	0.0719287	-562.9774920	-563.0033949	-0.0460259	-562.0666892	-562.2354546	-559.9202271	-559.9662530
9	rd_hX12_e_abb	-563.0501299	0.0733209	-562.9768090	-563.0050816	-0.0450483	-562.0682839	-562.2366089	-559.9210768	-559.9661252
10	rd_hX13_a_aan	-563.0486037	0.0718447	-562.9767590	-563.0029373	-0.0456664	#N/A	#N/A	#N/A	#N/A
11	rd_hX12_e_aar	-563.0486878	0.0719428	-562.9767450	-562.9995438	-0.0491440	#N/A	#N/A	#N/A	#N/A
12	rd_hX12_e_aau	-563.0491488	0.0724977	-562.9766510	-563.0035133	-0.0456355	#N/A	#N/A	#N/A	#N/A
13	rd_hX12_e_aan	-563.0493342	0.0727911	-562.9765430	-563.0012190	-0.0481152	#N/A	#N/A	#N/A	#N/A
14	rd_hX13_a_aal	-563.0465476	0.0700085	-562.9765390	-562.9981161	-0.0484314	#N/A	#N/A	#N/A	#N/A
15	rd_hX13_a_abn	-563.0485739	0.0724559	-562.9761180	-563.0024992	-0.0460747	#N/A	#N/A	#N/A	#N/A
16	rd_hX12_e_abw	-563.0477537	0.0717076	-562.9760460	-562.9932507	-0.0545030	#N/A	#N/A	#N/A	#N/A
17	rd_hX13_a_abd	-563.0486283	0.0726573	-562.9759710	-563.0006262	-0.0480022	#N/A	#N/A	#N/A	#N/A
18	rd_hX13_a_aam	-563.0485857	0.0726206	-562.9759650	-563.0005983	-0.0479874	#N/A	#N/A	#N/A	#N/A
19	rd_hX13_a_aaj	-563.0486279	0.0727229	-562.9759050	-563.0006235	-0.0480044	#N/A	#N/A	#N/A	#N/A
20	rd_hX12_e_abe	-563.0486888	0.0727857	-562.9759030	-563.0016268	-0.0470620	#N/A	#N/A	#N/A	#N/A
1	rd_1mG63_abv	-657.7576256	0.1136565	-657.6439690	-657.7066848	-0.0509408	-656.5997326	-656.7969474	-654.0467796	-654.0977204
2	rd_1mG63_abn	-657.7555901	0.1119310	-657.6436590	-657.7012081	-0.0543820	-656.5933038	-656.7918247	-654.0446076	-654.0989895
3	rd_1mG63_abw	-657.7549434	0.1114963	-657.6434470	-657.6989745	-0.0559688	-656.5917920	-656.7898562	-654.0416524	-654.0976212
4	rd_1mG63_aam	-657.7574096	0.1140665	-657.6433430	-657.7047575	-0.0526521	-656.5980317	-656.7954433	-654.0448782	-654.0975303
5	rd_1mG63_aav	-657.7579976	0.1150375	-657.6429600	-657.7036447	-0.0543528	-656.5967051	-656.7939727	-654.0427167	-654.0970695
6	rd_1mG63_abu	-657.7567041	0.1141351	-657.6425690	-657.6986482	-0.0580560	#N/A	#N/A	#N/A	#N/A
7	rd_1mG63_abc	-657.7580536	0.1155245	-657.6425290	-657.6972110	-0.0608425	-656.5910103	-656.7887772	-654.0390243	-654.0998669
8	rd_1mG63_aag	-657.7587531	0.1166210	-657.6421320	-657.7061733	-0.0525798	-656.5971734	-656.7951445	-654.0431799	-654.0957597
9	rd_1mG63_aas	-657.7581260	0.1161610	-657.6419650	-657.7026686	-0.0554574	-656.5963898	-656.7933555	-654.0419742	-654.0974316
10	rd_1mG63_abi	-657.7538582	0.1119642	-657.6418940	-657.6937984	-0.0600598	#N/A	#N/A	#N/A	#N/A
11	rd_1mG63_aab	-657.7539508	0.1120948	-657.6418560	-657.6955716	-0.0583792	#N/A	#N/A	#N/A	#N/A
12	rd_1mG63_aae	-657.7573673	0.1158053	-657.6415620	-657.7031785	-0.0541888	-656.5949475	-656.7931961	-654.0443694	-654.0985582
13	rd_1mG63_abt	-657.7570366	0.1155415	-657.6414950	-657.6964511	-0.0605855	#N/A	#N/A	#N/A	#N/A
14	rd_1mG63_aax	-657.7539923	0.1128303	-657.6411620	-657.6954314	-0.0585609	#N/A	#N/A	#N/A	#N/A
15	rd_1mG63_aah	-657.7557273	0.1149643	-657.6407630	-657.6992042	-0.0565231	-656.5925212	-656.7903500	-654.0399761	-654.0964992
16	rd_1mG63_aap	-657.7536301	0.1130760	-657.6405540	-657.6898522	-0.0637778	#N/A	#N/A	#N/A	#N/A
17	rd_1mG63_abj	-657.7571687	0.1166596	-657.6405090	-657.6942587	-0.0629100	-656.5883421	-656.7858724	-654.0339331	-654.0968431
18	rd_1mG63_abk	-657.7561306	0.1163926	-657.6397380	-657.6982255	-0.0579051	-656.5920080	-656.7895766	-654.0382958	-654.0962010
19	rd_1mG63_aak	-657.7574482	0.1179191	-657.6395290	-657.6968942	-0.0605539	-656.5906124	-656.7881278	-654.0372089	-654.0977628
20	rd_1mG63_aaq	-657.7538058	0.1149977	-657.6388080	-657.6902569	-0.0635489	#N/A	#N/A	#N/A	#N/A
1	rd_PhOH_aas	-383.3216024	0.0800563	-383.2415460	-383.3024771	-0.0191253	-382.6249856	-382.7403182	-381.0900554	-381.1091807
2	rd_PhOH_aaq	-383.3216084	0.0801714	-383.2414370	-383.3027044	-0.0189040	-382.6249540	-382.7403786	-381.0901435	-381.1090475
3	rd_PhOH_aao	-383.3151810	0.0745850	-383.2405960	-383.2919281	-0.0232529	-382.6163804	-382.7317876	-381.0841683	-381.1074212
4	rd_PhOH_aal	-383.3220353	0.0816172	-383.2404180	-383.2984179	-0.0236173	-382.6217841	-382.7368827	-381.0855569	-381.1091742
5	rd_PhOH_abk	-383.3195936	0.0792656	-383.2403280	-383.2943279	-0.0252657	-382.6193150	-382.7342779	-381.0835660	-381.1088318
6	rd_PhOH_abh	-383.3150767	0.0754066	-383.2396700	-383.2923466	-0.0227300	-382.6169279	-382.7321010	-381.0848479	-381.1075780
7	rd_PhOH_aav	-383.3195853	0.0801293	-383.2394560	-383.2944443	-0.0251410	-382.6193987	-382.7343813	-381.0837524	-381.1088935
8	rd_PhOH_abn	-383.3169819	0.0784218	-383.2385600	-383.2931687	-0.0238132	-382.6184110	-382.7335116	-381.0849992	-381.1088124
9	rd_PhOH_abd	-383.3158140	0.0774080	-383.2384060	-383.2914978	-0.0243162	-382.6166567	-382.7317234	-381.0840526	-381.1083688
10	rd_PhOH_aan	-383.3168641	0.0785821	-383.2382820	-383.2916915	-0.0251726	-382.6172729	-382.7324059	-381.0839214	-381.1090941
11	rd_PhOH_aab	-383.3176360	0.0798489	-383.2377870	-383.2911820	-0.0264540	-382.6176421	-382.7319842	-381.0825173	-381.1089713

12	rd_PhOH_aak	-383.3171721	0.0795050	-383.2376670	-383.2892792	-0.0278928	-382.6150475	-382.7300332	-381.0808380	-381.1087308
13	rd_PhOH_aae	-383.3169036	0.0792946	-383.2376090	-383.2913153	-0.0255883	#N/A	#N/A	#N/A	#N/A
14	rd_PhOH_aaf	-383.3172341	0.0804310	-383.2368030	-383.2894484	-0.0277857	-382.6151734	-382.7301823	-381.0809580	-381.1087437
15	rd_PhOH_abl	-383.3172886	0.0805035	-383.2367850	-383.2896185	-0.0276701	-382.6153876	-382.7303019	-381.0810812	-381.1087513
1	rd_Ind_aas	-439.6650629	0.1033958	-439.5616670	-439.6464754	-0.0185875	#N/A	#N/A	#N/A	#N/A
2	rd_Ind_aav	-439.6650816	0.1039245	-439.5611570	-439.6463841	-0.0186975	#N/A	#N/A	#N/A	#N/A
3	rd_Ind_abp	-439.6654323	0.1048343	-439.5605980	-439.6437315	-0.0217008	#N/A	#N/A	#N/A	#N/A
4	rd_Ind_abs	-439.6582008	0.0983537	-439.5598470	-439.6364480	-0.0217528	-438.8238724	-438.9526240	-437.0031910	-437.0249438
5	rd_Ind_abm	-439.6630943	0.1037283	-439.5593660	-439.6391306	-0.0239637	-438.8255074	-438.9542100	-437.0008634	-437.0248271
6	rd_Ind_aai	-439.6629740	0.1037539	-439.5592200	-439.6388917	-0.0240822	-438.8253512	-438.9540357	-437.0002674	-437.0243497
7	rd_Ind_abo	-439.6630951	0.1040711	-439.5590240	-439.6389451	-0.0241500	-438.8253654	-438.9540847	-437.0005939	-437.0247439
8	rd_Ind_aba	-439.6585452	0.0998811	-439.5586640	#N/A	#N/A	-438.8209105	-438.9496644	-436.9993226	#N/A
9	rd_Ind_abi	-439.6602721	0.1026781	-439.5575940	#N/A	#N/A	-438.8215118	-438.9502033	-436.9985256	#N/A
10	rd_Ind_aak	-439.6596976	0.1021925	-439.5575050	#N/A	#N/A	-438.8217607	-438.9507780	-437.0000087	#N/A
11	rd_Ind_abh	-439.6595756	0.1020825	-439.5574930	#N/A	#N/A	#N/A	#N/A	#N/A	#N/A
12	rd_Ind_aaf	-439.6596637	0.1021916	-439.5574720	#N/A	#N/A	#N/A	#N/A	#N/A	#N/A
13	rd_Ind_abt	-439.6592194	0.1022054	-439.5570140	#N/A	#N/A	#N/A	#N/A	#N/A	#N/A
14	rd_Ind_abf	-439.6597838	0.1027848	-439.5569990	#N/A	#N/A	-438.8217274	-438.9507375	-436.9998309	#N/A
15	rd_Ind_aaj	-439.6600050	0.1030110	-439.5569940	#N/A	#N/A	-438.8199878	-438.9488245	-436.9973908	#N/A
16	rd_Ind_aax	-439.6605083	0.1039703	-439.5565380	#N/A	#N/A	-438.8229942	-438.9509827	-436.9993198	#N/A
17	rd_Ind_aaq	-439.6593775	0.1029365	-439.5564410	#N/A	#N/A	#N/A	#N/A	#N/A	#N/A
18	rd_Ind_aaz	-439.6605339	0.1042358	-439.5562980	#N/A	#N/A	-438.8227462	-438.9507445	-436.9991282	#N/A
1	h2o	-76.4480650	0.0033390	-76.4447259	#N/A	#N/A	#N/A	#N/A	#N/A	#N/A
1	p_A24_abq	-544.3006439	0.1106728	-544.1899710	-544.1934986	-0.1071453	-543.2711930	-543.4321898	-541.1339283	-541.2410736
2	p_A24_abl	-544.3007148	0.1111948	-544.1895200	-544.1949836	-0.1057312	-543.2726292	-543.4335449	-541.1353317	-541.2410628
3	p_A25_aaq	-544.2986945	0.1100924	-544.1886020	-544.1774656	-0.1212288	#N/A	#N/A	#N/A	#N/A
4	p_A24_aad	-544.3006393	0.1123292	-544.1883100	-544.1915691	-0.1090702	-543.2693472	-543.4304383	-541.1320849	-541.2411551
5	p_A24_aaz	-544.3009267	0.1126666	-544.1882600	-544.1876082	-0.1133184	-543.2664073	-543.4271949	-541.1284329	-541.2417513
6	p_A35_aao	-544.2995439	0.1116179	-544.1879260	-544.1918203	-0.1077236	#N/A	#N/A	#N/A	#N/A
7	p_A35_aal	-544.2994496	0.1118065	-544.1876430	-544.1907508	-0.1086987	#N/A	#N/A	#N/A	#N/A
8	p_A35_aat	-544.3014406	0.1139115	-544.1875290	-544.1989107	-0.1025299	-543.2748377	-543.4357488	-541.1381159	-541.2406457
9	p_A24_aax	-544.2989665	0.1119274	-544.1870390	-544.1889130	-0.1100535	#N/A	#N/A	#N/A	#N/A
10	p_A24_abt	-544.2988705	0.1119734	-544.1868970	-544.1879263	-0.1109442	#N/A	#N/A	#N/A	#N/A
11	p_A35_aay	-544.2994866	0.1126316	-544.1868550	-544.1895776	-0.1099091	#N/A	#N/A	#N/A	#N/A
12	p_A24_aav	-544.2989138	0.1121378	-544.1867760	-544.1882185	-0.1106954	#N/A	#N/A	#N/A	#N/A
13	p_A35_abo	-544.3002858	0.1135468	-544.1867390	-544.1868391	-0.1134467	-543.2641735	-543.4252498	-541.1281453	-541.2415921
14	p_A24_aat	-544.2988421	0.1121780	-544.1866640	-544.1880093	-0.1108328	#N/A	#N/A	#N/A	#N/A
15	p_A35_abu	-544.2994434	0.1129083	-544.1865350	-544.1895724	-0.1098709	#N/A	#N/A	#N/A	#N/A
16	p_A35_aap	-544.3001273	0.1135963	-544.1865310	-544.1861593	-0.1139680	-543.2629904	-543.4243761	-541.1274173	-541.2413853
17	p_A25_aad	-544.2986660	0.1121929	-544.1864730	-544.1711202	-0.1275458	#N/A	#N/A	#N/A	#N/A
18	p_A35_abe	-544.3004622	0.1141532	-544.1863090	-544.1857872	-0.1146750	-543.2635447	-543.4244619	-541.1269891	-541.2416642
19	p_A25_aba	-544.3001739	0.1141929	-544.1859810	-544.1816868	-0.1184871	-543.2586998	-543.4196466	-541.1203982	-541.2388853
20	p_A25_aay	-544.2987640	0.1129279	-544.1858360	-544.1691704	-0.1295936	#N/A	#N/A	#N/A	#N/A
1	p_5mU145_aah	-531.0954337	0.1146757	-530.9807580	-530.9882224	-0.1072113	-530.1234639	-530.2829265	-528.0965414	-528.2037527
2	p_5mU146_abd	-531.0948587	0.1149887	-530.9798700	-530.9826673	-0.1121915	-530.1179495	-530.2777408	-528.0903737	-528.2025652
3	p_5mU146_aal	-531.0948632	0.1151751	-530.9796880	-530.9846347	-0.1102285	-530.1195042	-530.2793193	-528.0918317	-528.2020602
4	p_5mU146_aak	-531.0901525	0.1129544	-530.9771980	-530.9781553	-0.1119972	-530.1145080	-530.2746497	-528.0887570	-528.2007542
5	p_5mU146_abb	-531.0896283	0.1124992	-530.9771290	-530.9778680	-0.1117603	-530.1138275	-530.2740502	-528.0886613	-528.2004216

6	p_5mU146_aaf	-531.0898569	0.1127648	-530.9770920	-530.9810259	-0.1088310	-530.1167442	-530.2767828	-528.0921658	-528.2009967
7	p_5mU146_abe	-531.0897683	0.1128633	-530.9769050	-530.9793895	-0.1103789	-530.1152185	-530.2753986	-528.0902880	-528.2006669
8	p_5mU146_aam	-531.0901461	0.1137130	-530.9764330	-530.9798176	-0.1103285	#N/A	#N/A	#N/A	#N/A
9	p_5mU145_aat	-531.0879972	0.1117742	-530.9762230	-530.9746652	-0.1133320	-530.1104273	-530.2706046	-528.0857213	-528.1990533
10	p_5mU146_aar	-531.0899541	0.1144211	-530.9755330	-530.9762728	-0.1136813	-530.1131413	-530.2730944	-528.0869981	-528.2006794
11	p_5mU145_abv	-531.0878810	0.1127310	-530.9751500	-530.9717563	-0.1161247	-530.1081464	-530.2682055	-528.0827210	-528.1988457
12	p_5mU146_abk	-531.0819195	0.1084234	-530.9734960	-530.9655316	-0.1163879	#N/A	#N/A	#N/A	#N/A
13	p_5mU146_abj	-531.0830778	0.1097728	-530.9733050	-530.9518886	-0.1311892	#N/A	#N/A	#N/A	#N/A
14	p_5mU146_aay	-531.0847078	0.1114578	-530.9732500	-530.9607835	-0.1239243	#N/A	#N/A	#N/A	#N/A
15	p_5mU146_aah	-531.0873315	0.1143204	-530.9730110	-530.9546584	-0.1326730	-530.0916518	-530.2527350	-528.0682006	-528.2008736
16	p_5mU146_abl	-531.0846071	0.1116971	-530.9729100	-530.9598458	-0.1247613	#N/A	#N/A	#N/A	#N/A
17	p_5mU146_aae	-531.0871294	0.1142403	-530.9728890	-530.9603664	-0.1267630	-530.0962065	-530.2576128	-528.0737977	-528.2005606
18	p_5mU146_abr	-531.0847592	0.1119241	-530.9728350	-530.9623306	-0.1224286	#N/A	#N/A	#N/A	#N/A
19	p_5mU146_abw	-531.0855408	0.1128457	-530.9726950	-530.9486494	-0.1368914	#N/A	#N/A	#N/A	#N/A
20	p_5mU146_abf	-531.0864581	0.1140341	-530.9724240	-530.9487428	-0.1377153	-530.0861763	-530.2469488	-528.0613714	-528.1990867
1	p_G3_aac	-619.5541364	0.1151783	-619.4389580	-619.4417696	-0.1123668	-618.4194562	-618.6051680	-616.0433390	-616.1557058
2	p_G3_aal	-619.5528735	0.1149404	-619.4379330	-619.4402843	-0.1125892	-618.4185574	-618.6040605	-616.0438321	-616.1564213
3	p_G3_abu	-619.5517873	0.1142403	-619.4375470	-619.4353451	-0.1164423	#N/A	#N/A	#N/A	#N/A
4	p_G3_aab	-619.5519961	0.1150091	-619.4369870	-619.4372206	-0.1147755	-618.4158023	-618.6013484	-616.0412827	-616.1560582
5	p_G3_abo	-619.5519094	0.1149643	-619.4369450	-619.4366496	-0.1152598	-618.4152755	-618.6008667	-616.0406354	-616.1558952
6	p_G3_aaf	-619.5512910	0.1146320	-619.4366590	-619.4403921	-0.1108990	-618.4179844	-618.6034095	-616.0430430	-616.1539420
7	p_G2_aar	-619.5507638	0.1145317	-619.4362320	-619.4364219	-0.1143419	-618.4157718	-618.6009276	-616.0411372	-616.1554791
8	p_G3_abb	-619.5510693	0.1152243	-619.4358450	-619.4278711	-0.1231983	-618.4074054	-618.5927847	-616.0313258	-616.1545240
9	p_G3_aas	-619.5509872	0.1153132	-619.4356740	-619.4305633	-0.1204239	-618.4088197	-618.5946790	-616.0340051	-616.1544290
10	p_G2_aai	-619.5498604	0.1142034	-619.4356570	-619.4303147	-0.1195457	#N/A	#N/A	#N/A	#N/A
11	p_G3_abh	-619.5498479	0.1144469	-619.4354010	-619.4213337	-0.1285142	#N/A	#N/A	#N/A	#N/A
12	p_G3_abn	-619.5498429	0.1145618	-619.4352810	-619.4222947	-0.1275482	#N/A	#N/A	#N/A	#N/A
13	p_G3_aat	-619.5484753	0.1132313	-619.4352440	-619.4289870	-0.1194884	#N/A	#N/A	#N/A	#N/A
14	p_G2_abt	-619.5502818	0.1150767	-619.4352050	-619.4358142	-0.1144676	#N/A	#N/A	#N/A	#N/A
15	p_G2_aat	-619.5506047	0.1156367	-619.4349680	-619.4226280	-0.1279767	-618.4037637	-618.5889825	-616.0279426	-616.1559193
16	p_G2_abh	-619.5499614	0.1150743	-619.4348870	-619.4212680	-0.1286934	#N/A	#N/A	#N/A	#N/A
17	p_G2_aae	-619.5503952	0.1155611	-619.4348340	-619.4232604	-0.1271348	#N/A	#N/A	#N/A	#N/A
18	p_G2_aag	-619.5482460	0.1135380	-619.4347080	-619.4236837	-0.1245624	#N/A	#N/A	#N/A	#N/A
19	p_G3_aba	-619.5501303	0.1154312	-619.4346990	-619.4333715	-0.1167588	#N/A	#N/A	#N/A	#N/A
20	p_G2_aam	-619.5499567	0.1153197	-619.4346370	-619.4321301	-0.1178266	#N/A	#N/A	#N/A	#N/A
1	p_C14_abd	-471.9160831	0.0983311	-471.8177520	-471.8037266	-0.1123565	-471.0271567	-471.1695531	-469.2176916	-469.3300481
2	p_C14_aaq	-471.9175941	0.1002901	-471.8173040	-471.8122406	-0.1053535	-471.0354183	-471.1774150	-469.2250201	-469.3303736
3	p_C14_aan	-471.9158348	0.0991668	-471.8166680	-471.8007420	-0.1150928	-471.0247159	-471.1670260	-469.2144833	-469.3295761
4	p_C96	-471.9174324	0.1008904	-471.8165420	-471.7975479	-0.1198845	-471.0225780	-471.1646037	-469.2108806	-469.3307651
5	p_C12	-471.9172331	0.1007890	-471.8164440	-471.8073707	-0.1098624	-471.0307917	-471.1730421	-469.2208515	-469.3307139
6	p_C14_aao	-471.9158506	0.0994315	-471.8164190	-471.8007519	-0.1150986	-471.0246300	-471.1669931	-469.2144764	-469.3295750
7	p_C14_abe	-471.9161033	0.0996853	-471.8164180	-471.8038658	-0.1122376	-471.0273146	-471.1697015	-469.2178370	-469.3300746
8	p_C14_abo	-471.9172968	0.1011788	-471.8161180	-471.8046837	-0.1126131	-471.0282164	-471.1706132	-469.2182357	-469.3308488
9	p_C03	-471.9157398	0.0997977	-471.8159420	-471.8080149	-0.1077248	#N/A	#N/A	#N/A	#N/A
10	p_C02	-471.9145446	0.0988626	-471.8156820	-471.7902456	-0.1242990	#N/A	#N/A	#N/A	#N/A
11	p_C07	-471.9153526	0.0998956	-471.8154570	-471.7981653	-0.1171873	#N/A	#N/A	#N/A	#N/A
12	p_C13	-471.9150323	0.0997403	-471.8152920	-471.8048896	-0.1101428	#N/A	#N/A	#N/A	#N/A
13	p_C45	-471.9157130	0.1006379	-471.8150750	-471.8009312	-0.1147818	#N/A	#N/A	#N/A	#N/A

14	p_C43	-471.9153636	0.1003835	-471.8149800	-471.7987038	-0.1166598	#N/A	#N/A	#N/A	#N/A
15	p_C67	-471.9161126	0.1011406	-471.8149720	-471.7998643	-0.1162483	#N/A	#N/A	#N/A	#N/A
16	p_C49	-471.9146584	0.0998743	-471.8147840	-471.7904026	-0.1242557	#N/A	#N/A	#N/A	#N/A
17	p_C62	-471.9152720	0.1005399	-471.8147320	-471.8004559	-0.1148160	#N/A	#N/A	#N/A	#N/A
18	p_C42	-471.9146214	0.0999653	-471.8146560	-471.7905538	-0.1240675	#N/A	#N/A	#N/A	#N/A
19	p_C14_aam	-471.9153587	0.1007587	-471.8146000	-471.7989865	-0.1163722	-471.0231625	-471.1655675	-469.2131134	-469.3294855
20	p_C84	-471.9123933	0.0978332	-471.8145600	-471.7832549	-0.1291384	#N/A	#N/A	#N/A	#N/A
1	p_U146_aba	-491.7717905	0.0887164	-491.6830740	-491.6540428	-0.1177477	-490.8714011	-491.0192782	-489.0382570	-489.1560047
2	p_U145_aao	-491.7704495	0.0896694	-491.6807800	-491.6599074	-0.1105421	-490.8764298	-491.0242037	-489.0439154	-489.1544574
3	p_U145_aap	-491.7705600	0.0899260	-491.6806340	-491.6493082	-0.1212518	-490.8671839	-491.0149955	-489.0336431	-489.1548949
4	p_U145_aar	-491.7701464	0.0895244	-491.6806220	-491.6507946	-0.1193519	-490.8676579	-491.0157790	-489.0350051	-489.1543570
5	p_U146_aat	-491.7656904	0.0873564	-491.6783340	-491.6524245	-0.1132659	-490.8700554	-491.0183248	-489.0393859	-489.1526518
6	p_U146_abk	-491.7656394	0.0877834	-491.6778560	#N/A	#N/A	#N/A	#N/A	#N/A	#N/A
7	p_U146_aas	-491.7655121	0.0877760	-491.6777360	-491.6488752	-0.1166368	-490.8668536	-491.0150870	-489.0357618	-489.1523986
8	p_U146_aab	-491.7656540	0.0880369	-491.6776170	-491.6501675	-0.1154865	-490.8678211	-491.0161813	-489.0369577	-489.1524442
9	p_U146_abo	-491.7652038	0.0877157	-491.6774880	-491.6512732	-0.1139306	-490.8685258	-491.0169246	-489.0386282	-489.1525588
10	p_U146_abg	-491.7649734	0.0875134	-491.6774600	-491.6482513	-0.1167222	-490.8660755	-491.0144030	-489.0354083	-489.1521305
11	p_U145_abm	-491.7628350	0.0869049	-491.6759300	-491.6477683	-0.1150667	-490.8650979	-491.0133933	-489.0351814	-489.1502481
12	p_U145_abn	-491.7626542	0.0869121	-491.6757420	-491.6445416	-0.1181126	#N/A	#N/A	#N/A	#N/A
13	p_U145_aaa	-491.7626488	0.0873117	-491.6753370	-491.6438067	-0.1188421	#N/A	#N/A	#N/A	#N/A
14	p_U146_aaj	-491.7624824	0.0877543	-491.6747280	-491.6333764	-0.1291060	#N/A	#N/A	#N/A	#N/A
15	p_U146_aad	-491.7626070	0.0892220	-491.6733850	-491.6223116	-0.1402954	#N/A	#N/A	#N/A	#N/A
16	p_U146_abr	-491.7607392	0.0874072	-491.6733320	-491.6241204	-0.1366188	#N/A	#N/A	#N/A	#N/A
17	p_U146_aal	-491.7626770	0.0901020	-491.6725750	-491.6243401	-0.1383369	#N/A	#N/A	#N/A	#N/A
18	p_U145_abx	-491.7602525	0.0878335	-491.6724190	-491.6282181	-0.1320344	#N/A	#N/A	#N/A	#N/A
19	p_U145_abf	-491.7604160	0.0882169	-491.6721990	-491.6307757	-0.1296403	#N/A	#N/A	#N/A	#N/A
20	p_U146_abq	-491.7590210	0.0869739	-491.6720470	-491.6196039	-0.1394170	#N/A	#N/A	#N/A	#N/A
1	p_1mG22_abt	-658.8613002	0.1406122	-658.7206880	-658.7554629	-0.1058373	-657.6545894	-657.8510668	-655.0836646	-655.1895019
2	p_1mG23_abu	-658.8638366	0.1431826	-658.7206540	-658.7597856	-0.1040511	-657.6573207	-657.8543340	-655.0841790	-655.1882300
3	p_1mG23_abx	-658.8614449	0.1408749	-658.7205700	-658.7534626	-0.1079823	-657.6517768	-657.8487573	-655.0801890	-655.1881713
4	p_1mG23_aak	-658.8569559	0.1376638	-658.7192920	-658.7372680	-0.1196879	#N/A	#N/A	#N/A	#N/A
5	p_1mG22_abr	-658.8606710	0.1419429	-658.7187280	-658.7427127	-0.1179583	-657.6430858	-657.8397222	-655.0713872	-655.1893454
6	p_1mG22_abo	-658.8608887	0.1424236	-658.7184650	-658.7427941	-0.1180946	-657.6434968	-657.8399797	-655.0714537	-655.1895483
7	p_1mG22_aao	-658.8595076	0.1410986	-658.7184090	-658.7434544	-0.1160533	-657.6441576	-657.8406395	-655.0732856	-655.1893389
8	p_1mG23_aau	-658.8580205	0.1398075	-658.7182130	-658.7471282	-0.1108924	#N/A	#N/A	#N/A	#N/A
9	p_1mG23_abm	-658.8593898	0.1414328	-658.7179570	-658.7383412	-0.1210486	-657.6351196	-657.8333870	-655.0659173	-655.1869658
10	p_1mG22_abg	-658.8606309	0.1429279	-658.7177030	-658.7454254	-0.1152055	-657.6445733	-657.8415963	-655.0730881	-655.1882936
11	p_1mG22_abv	-658.8583113	0.1406093	-658.7177020	-658.7429348	-0.1153765	#N/A	#N/A	#N/A	#N/A
12	p_1mG23_abr	-658.8599749	0.1423249	-658.7176500	-658.7325182	-0.1274567	-657.6309520	-657.8287198	-655.0601298	-655.1875865
13	p_1mG22_aar	-658.8587984	0.1411753	-658.7176230	-658.7453980	-0.1134004	#N/A	#N/A	#N/A	#N/A
14	p_1mG22_abx	-658.8585664	0.1409744	-658.7175920	-658.7428102	-0.1157562	#N/A	#N/A	#N/A	#N/A
15	p_1mG23_abd	-658.8593974	0.1419324	-658.7174650	-658.7375814	-0.1218160	-657.6345808	-657.8328305	-655.0651839	-655.1869999
16	p_1mG23_aap	-658.8588010	0.1415120	-658.7172890	-658.7465500	-0.1122511	#N/A	#N/A	#N/A	#N/A
17	p_1mG23_aal	-658.8578995	0.1406195	-658.7172800	-658.7474724	-0.1104271	#N/A	#N/A	#N/A	#N/A
18	p_1mG23_abn	-658.8553328	0.1380767	-658.7172560	-658.7477307	-0.1076021	#N/A	#N/A	#N/A	#N/A
19	p_1mG23_aas	-658.8586772	0.1414722	-658.7172050	-658.7478937	-0.1107835	#N/A	#N/A	#N/A	#N/A
20	p_1mG23_aax	-658.8550014	0.1378624	-658.7171390	-658.7331263	-0.1218751	#N/A	#N/A	#N/A	#N/A
1	p_X1_aae	-639.4185745	0.1027775	-639.3157970	-639.2892294	-0.1293451	-638.2603490	-638.4521165	-635.8643806	-635.9937257

2	p_X1_abs	-639.4187009	0.1032229	-639.3154780	-639.2874142	-0.1312868	-638.2589282	-638.4506281	-635.8628390	-635.9941257
3	p_X1_abi	-639.4187322	0.1034092	-639.3153230	-639.2865755	-0.1321567	-638.2580581	-638.4498068	-635.8619172	-635.9940740
4	p_X1_aab	-639.4182920	0.1030190	-639.3152730	-639.2916882	-0.1266038	-638.2628681	-638.4544472	-635.8673237	-635.9939274
5	p_X1_aar	-639.4178867	0.1027657	-639.3151210	-639.2864606	-0.1314261	-638.2584140	-638.4500667	-635.8618238	-635.9932499
6	p_X1_aal	-639.4174381	0.1026881	-639.3147500	-639.2944177	-0.1230204	-638.2647396	-638.4562758	-635.8693261	-635.9923464
7	p_X1_abn	-639.4170970	0.1028329	-639.3142640	-639.2881847	-0.1289123	-638.2586304	-638.4504100	-635.8634028	-635.9923151
8	p_X1_aak	-639.4193430	0.1051379	-639.3142050	-639.2933034	-0.1260395	-638.2639863	-638.4557963	-635.8668771	-635.9929166
9	p_X1_aap	-639.4168657	0.1028706	-639.3139950	-639.2751467	-0.1417190	-638.2469260	-638.4387367	-635.8498292	-635.9915482
10	p_X1_aad	-639.4168651	0.1034960	-639.3133690	-639.2760116	-0.1408534	-638.2474778	-638.4393899	-635.8507815	-635.9916349
11	p_X1_abh	-639.4168162	0.1042911	-639.3125250	-639.2771209	-0.1396953	#N/A	#N/A	#N/A	#N/A
12	p_X1_abr	-639.4149860	0.1028220	-639.3121640	-639.2810175	-0.1339685	#N/A	#N/A	#N/A	#N/A
13	p_X1_aag	-639.4147629	0.1029129	-639.3118500	-639.2794549	-0.1353080	#N/A	#N/A	#N/A	#N/A
14	p_X1_aba	-639.4141348	0.1033298	-639.3108050	-639.2683486	-0.1457862	#N/A	#N/A	#N/A	#N/A
15	p_X1_aan	-639.4142711	0.1035320	-639.3107390	-639.2666396	-0.1476315	#N/A	#N/A	#N/A	#N/A
16	p_X1_abw	-639.4144532	0.1037332	-639.3107200	-639.2630123	-0.1514409	#N/A	#N/A	#N/A	#N/A
17	p_X1_aaj	-639.4144110	0.1039259	-639.3104850	-639.2586939	-0.1557171	#N/A	#N/A	#N/A	#N/A
18	p_X1_abv	-639.4102488	0.0999967	-639.3102520	-639.2765048	-0.1337440	#N/A	#N/A	#N/A	#N/A
19	p_X1_aat	-639.4144576	0.1044316	-639.3100260	-639.2617226	-0.1527351	#N/A	#N/A	#N/A	#N/A
20	p_X1_aaq	-639.4145038	0.1046388	-639.3098650	-639.2598115	-0.1546923	#N/A	#N/A	#N/A	#N/A
1	p_hX3_aar	-564.1653774	0.0998044	-564.0655730	-564.0489603	-0.1164172	-563.1181698	-563.2855076	-560.9596600	-561.0760772
2	p_hX3_aam	-564.1654432	0.1000172	-564.0654260	-564.0512679	-0.1141753	-563.1202648	-563.2875777	-560.9621087	-561.0762840
3	p_hX3_aan	-564.1653544	0.1004223	-564.0649320	-564.0491216	-0.1162328	-563.1182798	-563.2856350	-560.9598052	-561.0760380
4	p_hX3_abx	-564.1670824	0.1027593	-564.0643230	-564.0547985	-0.1122839	-563.1233408	-563.2907517	-560.9637605	-561.0760444
5	p_hX3_abs	-564.1634062	0.1003751	-564.0630310	-564.0468107	-0.1165955	-563.1153182	-563.2827853	-560.9581260	-561.0747215
6	p_hX3_abn	-564.1633854	0.1004303	-564.0629550	-564.0465547	-0.1168307	-563.1150082	-563.2825605	-560.9577792	-561.0746099
7	p_hX3_abk	-564.1632688	0.1005368	-564.0627320	-564.0440013	-0.1192676	-563.1128563	-563.2803906	-560.9549902	-561.0742578
8	p_hX3_aac	-564.1632755	0.1008365	-564.0624390	-564.0437015	-0.1195740	-563.1126943	-563.2801750	-560.9546797	-561.0742537
9	p_hX3_abu	-564.1626941	0.1008100	-564.0618840	-564.0452914	-0.1174027	-563.1138810	-563.2815262	-560.9563816	-561.0737843
10	p_hX3_abg	-564.1609682	0.0994522	-564.0615160	-564.0219809	-0.1389874	#N/A	#N/A	#N/A	#N/A
11	p_hX2_aag	-564.1619057	0.1004747	-564.0614310	-564.0453625	-0.1165432	#N/A	#N/A	#N/A	#N/A
12	p_hX3_abe	-564.1624293	0.1012193	-564.0612100	-564.0314993	-0.1309300	#N/A	#N/A	#N/A	#N/A
13	p_hX3_aaz	-564.1586670	0.0975529	-564.0611140	-564.0415737	-0.1170933	#N/A	#N/A	#N/A	#N/A
14	p_hX2_abd	-564.1619250	0.1008610	-564.0610640	-564.0435273	-0.1183977	#N/A	#N/A	#N/A	#N/A
15	p_hX2_aan	-564.1619139	0.1009688	-564.0609450	-564.0438868	-0.1180271	#N/A	#N/A	#N/A	#N/A
16	p_hX2_aau	-564.1621242	0.1013521	-564.0607720	-564.0474374	-0.1146867	#N/A	#N/A	#N/A	#N/A
17	p_hX2_abp	-564.1608448	0.1005077	-564.0603370	-564.0446572	-0.1161876	#N/A	#N/A	#N/A	#N/A
18	p_hX3_aah	-564.1595847	0.0992696	-564.0603150	-564.0293191	-0.1302656	#N/A	#N/A	#N/A	#N/A
19	p_hX3_abd	-564.1591046	0.0989676	-564.0601370	-564.0190220	-0.1400826	#N/A	#N/A	#N/A	#N/A
20	p_hX2_abw	-564.1598245	0.0998365	-564.0599880	-564.0406766	-0.1191480	#N/A	#N/A	#N/A	#N/A
1	p_PhOH_aat	-384.3898145	0.1071614	-384.2826530	-384.2649421	-0.1248724	-383.5981495	-383.7117437	-382.0506502	-382.1755226
2	p_PhOH_aap	-384.3902781	0.1076471	-384.2826310	-384.2644705	-0.1258077	-383.5976491	-383.7112401	-382.0495724	-382.1753801
3	p_PhOH_aae	-384.3901306	0.1079105	-384.2822200	-384.2657892	-0.1243413	-383.5987289	-383.7122447	-382.0511739	-382.1755153
4	p_PhOH_abx	-384.3903131	0.1082160	-384.2820970	-384.2645407	-0.1257723	-383.5976262	-383.7112348	-382.0499062	-382.1756785
5	p_PhOH_aaz	-384.3900574	0.1083423	-384.2817150	-384.2641511	-0.1259063	-383.5973677	-383.7110262	-382.0495783	-382.1754846
6	p_PhOH_abg	-384.3898306	0.1084435	-384.2813870	-384.2644772	-0.1253534	-383.5976099	-383.7112214	-382.0501262	-382.1754795
7	p_PhOH_abs	-384.3902102	0.1089241	-384.2812860	-384.2651480	-0.1250622	-383.5981793	-383.7117357	-382.0501329	-382.1751951
8	p_PhOH_abt	-384.3904025	0.1094355	-384.2809670	-384.2651454	-0.1252572	-383.5982319	-383.7117750	-382.0503748	-382.1756320
9	p_PhOH_aag	-384.3659998	0.1055128	-384.2604870	-384.2249840	-0.1410158	-383.5586882	-383.6739871	-382.0159954	-382.1570112

10	p_PhOH_abv	-384.3624275	0.1036284	-384.2587990	-384.2325248	-0.1299027	#N/A	#N/A	#N/A	#N/A
11	p_PhOH_abo	-384.3633693	0.1060653	-384.2573040	-384.2232893	-0.1400800	#N/A	#N/A	#N/A	#N/A
12	p_PhOH_aac	-384.3640321	0.1068061	-384.2572260	-384.2226998	-0.1413323	#N/A	#N/A	#N/A	#N/A
13	p_PhOH_abn	-384.3618176	0.1051165	-384.2567010	-384.2361326	-0.1256850	#N/A	#N/A	#N/A	#N/A
14	p_PhOH_abm	-384.3640869	0.1075169	-384.2565700	-384.2230577	-0.1410292	-383.5584856	-383.6728697	-382.0163961	-382.1574252
15	p_PhOH_abk	-384.3633495	0.1071025	-384.2562470	-384.2171211	-0.1462284	#N/A	#N/A	#N/A	#N/A
1	p_1mInd_abs	-480.0569403	0.1573723	-479.8995680	-479.9676210	-0.0893193	-479.0798261	-479.2185424	-477.0505393	-477.1398586
2	p_1mInd_abb	-480.0569430	0.1581269	-479.8988160	-479.9677906	-0.0891524	-479.0799370	-479.2186651	-477.0506922	-477.1398446
3	p_1mInd_abu	-480.0569231	0.1582120	-479.8987110	-479.9680906	-0.0888325	-479.0802581	-479.2189761	-477.0511515	-477.1399840
4	p_1mInd_aar	-480.0569444	0.1583853	-479.8985590	-479.9656057	-0.0913387	-479.0780982	-479.2167967	-477.0482586	-477.1395972
5	p_1mInd_aaw	-480.0568862	0.1584551	-479.8984310	-479.9662259	-0.0906602	-479.0784788	-479.2172079	-477.0489394	-477.1395996
6	p_1mInd_aba	-480.0569413	0.1587113	-479.8982300	-479.9675071	-0.0894343	-479.0797719	-479.2184866	-477.0505354	-477.1399697
7	p_1mInd_abc	-480.0520713	0.1592682	-479.8928030	-479.9400918	-0.1119795	-479.0523039	-479.1922844	-477.0254438	-477.1374233
8	p_1mInd_abq	-480.0486658	0.1559657	-479.8927000	-479.9352846	-0.1133812	#N/A	#N/A	#N/A	#N/A
9	p_1mInd_abj	-480.0503651	0.1577700	-479.8925950	-479.9383262	-0.1120388	-479.0510995	-479.1911710	-477.0257251	-477.1377639
10	p_1mInd_aaf	-480.0522731	0.1598341	-479.8924390	-479.9413113	-0.1109618	-479.0538794	-479.1935851	-477.0268722	-477.1378340
11	p_1mInd_aat	-480.0504956	0.1580946	-479.8924010	-479.9374867	-0.1130089	-479.0510437	-479.1905048	-477.0247856	-477.1377945
12	p_1mInd_aaz	-480.0480698	0.1558197	-479.8922500	-479.9440480	-0.1040218	#N/A	#N/A	#N/A	#N/A
13	p_1mInd_aau	-480.0490620	0.1570579	-479.8920040	-479.9450734	-0.1039886	#N/A	#N/A	#N/A	#N/A
14	p_1mInd_abf	-480.0501193	0.1582543	-479.8918650	-479.9373560	-0.1127633	#N/A	#N/A	#N/A	#N/A
15	p_1mInd_abt	-480.0481001	0.1564600	-479.8916400	-479.9468904	-0.1012096	#N/A	#N/A	#N/A	#N/A
16	p_1mInd_abg	-480.0486109	0.1571998	-479.8914110	-479.9378472	-0.1107637	#N/A	#N/A	#N/A	#N/A
17	p_1mInd_aad	-480.0489036	0.1575036	-479.8914000	-479.9386646	-0.1102390	#N/A	#N/A	#N/A	#N/A
18	p_1mInd_aas	-480.0481519	0.1567518	-479.8914000	-479.9433041	-0.1048478	#N/A	#N/A	#N/A	#N/A
19	p_1mInd_abm	-480.0485793	0.1574262	-479.8911530	-479.9376519	-0.1109273	#N/A	#N/A	#N/A	#N/A
20	p_1mInd_abo	-480.0486620	0.1576300	-479.8910320	-479.9379480	-0.1107140	#N/A	#N/A	#N/A	#N/A
1	p_Ind_abc	-440.7420431	0.1297871	-440.6122560	-440.6453111	-0.0967320	-439.8386454	-439.9660559	-438.0028994	-438.0996315
2	p_Ind_aav	-440.7419701	0.1301860	-440.6117840	-440.6461414	-0.0958287	-439.8393407	-439.9667572	-438.0038249	-438.0996536
3	p_Ind_aac	-440.7419709	0.1305849	-440.6113860	-440.6461012	-0.0958697	-439.8392916	-439.9667233	-438.0037865	-438.0996562
4	p_Ind_aai	-440.7420112	0.1310692	-440.6109420	-440.6459507	-0.0960606	-439.8391685	-439.9666083	-438.0036138	-438.0996744
5	p_Ind_aab	-440.7418162	0.1308822	-440.6109340	-440.6449658	-0.0968504	-439.8381924	-439.9656979	-438.0026334	-438.0994838
6	p_Ind_abq	-440.7420139	0.1311098	-440.6109040	-440.6458706	-0.0961433	-439.8391406	-439.9665543	-438.0036812	-438.0998245
7	p_Ind_abp	-440.7418084	0.1310103	-440.6107980	-440.6450887	-0.0967197	-439.8383190	-439.9658122	-438.0027885	-438.0995081
8	p_Ind_abk	-440.7419362	0.1312932	-440.6106430	-440.6460257	-0.0959105	-439.8392348	-439.9666351	-438.0037601	-438.0996705
9	p_Ind_abe	-440.7419667	0.1316496	-440.6103170	-440.6447116	-0.0972551	-439.8381784	-439.9656030	-438.0023014	-438.0995565
10	p_Ind_abg	-440.7332826	0.1276826	-440.6056000	-440.6223978	-0.1108848	#N/A	#N/A	#N/A	#N/A
11	p_Ind_aaz	-440.7371498	0.1317968	-440.6053530	-440.6223949	-0.1147549	-439.8163056	-439.9444785	-437.9831468	-438.0979017
12	p_Ind_abw	-440.7330935	0.1287445	-440.6043490	-440.6226525	-0.1104410	#N/A	#N/A	#N/A	#N/A
13	p_Ind_aap	-440.7332737	0.1299076	-440.6033660	-440.6228731	-0.1104005	#N/A	#N/A	#N/A	#N/A
14	p_Ind_aao	-440.7338369	0.1305868	-440.6032500	-440.6148749	-0.1189620	#N/A	#N/A	#N/A	#N/A
15	p_Ind_aan	-440.7330465	0.1300265	-440.6030200	-440.6201171	-0.1129294	#N/A	#N/A	#N/A	#N/A
16	p_Ind_aau	-440.7331993	0.1302592	-440.6029400	-440.6206295	-0.1125698	#N/A	#N/A	#N/A	#N/A
17	p_Ind_aaj	-440.7331123	0.1301853	-440.6029270	-440.6205952	-0.1125171	#N/A	#N/A	#N/A	#N/A
18	p_Ind_abb	-440.7337686	0.1309446	-440.6028240	-440.6129760	-0.1207926	#N/A	#N/A	#N/A	#N/A
19	p_Ind_abf	-440.7329739	0.1301538	-440.6028200	-440.6199535	-0.1130204	#N/A	#N/A	#N/A	#N/A
20	p_Ind_aba	-440.7326013	0.1299003	-440.6027010	-440.6147538	-0.1178476	#N/A	#N/A	#N/A	#N/A
1	p_tAn_aba	-746.6605450	0.1288830	-746.5316620	-746.5471477	-0.1133973	-745.4412743	-745.5623451	-743.7474086	-743.8608059
2	p_tAn_abj	-746.6606857	0.1299547	-746.5307310	-746.5477630	-0.1129227	-745.4420120	-745.5631891	-743.7480409	-743.8609636

3	p_tAn_abx	-746.6606382	0.1299861	-746.5306520	-746.5482168	-0.1124214	-745.4425542	-745.5636895	-743.7487017	-743.8611231
4	p_tAn_aab	-746.6607544	0.1302984	-746.5304560	-746.5472762	-0.1134783	-745.4416473	-745.5627802	-743.7476363	-743.8611146
5	p_tAn_abo	-746.6606969	0.1308798	-746.5298170	-746.5467233	-0.1139736	-745.4411185	-745.5622139	-743.7470818	-743.8610554
6	p_tAn_aat	-746.6458973	0.1223622	-746.5235350	-746.5501612	-0.0957361	#N/A	#N/A	#N/A	#N/A
7	p_tAn_aah	-746.6501137	0.1266957	-746.5234180	-746.5429250	-0.1071887	-745.4380184	-745.5611668	-743.7507422	-743.8579309
8	p_tAn_abt	-746.6502318	0.1269808	-746.5232510	-746.5445836	-0.1056482	-745.4396639	-745.5626041	-743.7524292	-743.8580774
9	p_tAn_abm	-746.6487383	0.1256102	-746.5231280	-746.5544080	-0.0943302	-745.4487530	-745.5719332	-743.7636223	-743.8579525
10	p_tAn_abu	-746.6490668	0.1263348	-746.5227320	-746.5437628	-0.1053040	-745.4386987	-745.5618912	-743.7521321	-743.8574361
11	p_tAn_aai	-746.6457778	0.1232198	-746.5225580	-746.5460435	-0.0997344	#N/A	#N/A	#N/A	#N/A
12	p_tAn_abr	-746.6460475	0.1235804	-746.5224670	-746.5471524	-0.0988951	#N/A	#N/A	#N/A	#N/A
13	p_tAn_aaq	-746.6456110	0.1234709	-746.5221400	-746.5465712	-0.0990398	#N/A	#N/A	#N/A	#N/A
14	p_tAn_abs	-746.6461067	0.1243026	-746.5218040	-746.5459192	-0.1001874	#N/A	#N/A	#N/A	#N/A
15	p_tAn_aan	-746.6483707	0.1268116	-746.5215590	-746.5548157	-0.0935550	-745.4505573	-745.5727994	-743.7628926	-743.8564477
16	p_tAn_aae	-746.6458862	0.1251322	-746.5207540	-746.5463117	-0.0995745	#N/A	#N/A	#N/A	#N/A
1	caffeine_aaw	-756.9119341	0.1672771	-756.7446570	-756.8784489	-0.0334853	-755.6040731	-755.8327990	-752.6242655	-752.6577508
2	caffeine_aar	-756.9127709	0.1689299	-756.7438410	-756.8775673	-0.0352036	-755.6029625	-755.8317729	-752.6229295	-752.6581331
3	caffeine_aac	-756.9123439	0.1686489	-756.7436950	-756.8825876	-0.0297563	-755.6061677	-755.8354404	-752.6273442	-752.6571005
4	caffeine_aaa	-756.9128105	0.1694544	-756.7433560	-756.8775358	-0.0352747	-755.6029438	-755.8316798	-752.6224226	-752.6576974
5	caffeine_abl	-756.9123199	0.1690329	-756.7432870	-756.8802036	-0.0321164	-755.6039481	-755.8331825	-752.6230559	-752.6551723
6	caffeine_abp	-756.9124445	0.1694765	-756.7429680	-756.8774267	-0.0350178	-755.6027386	-755.8316236	-752.6227005	-752.6577183
7	caffeine_abh	-756.9126814	0.1697544	-756.7429270	-756.8770832	-0.0355983	-755.6024542	-755.8312606	-752.6222996	-752.6578978
8	caffeine_aau	-756.9130625	0.1702784	-756.7427840	-756.8789267	-0.0341358	-755.6036575	-755.8324081	-752.6220652	-752.6562009
9	caffeine_aao	-756.9111898	0.1686607	-756.7425290	-756.8738506	-0.0373392	-755.5996355	-755.8281319	-752.6189513	-752.6562905
10	caffeine_aab	-756.9109715	0.1691024	-756.7418690	-756.8719375	-0.0390340	-755.5977626	-755.8261718	-752.6168945	-752.6559285
11	caffeine_abm	-756.9115381	0.1697151	-756.7418230	-756.8734854	-0.0380527	-755.5992555	-755.8276032	-752.6187238	-752.6567765
12	caffeine_aba	-756.9116015	0.1699294	-756.7416720	-756.8722988	-0.0393027	-755.5980749	-755.8262657	-752.6170473	-752.6563499
13	caffeine_abr	-756.9098183	0.1682002	-756.7416180	-756.8686532	-0.0411650	-755.5948359	-755.8236550	-752.6159915	-752.6571565
14	caffeine_aay	-756.9116445	0.1704545	-756.7411900	-756.8723736	-0.0392710	-755.5982069	-755.8263521	-752.6170109	-752.6562819
15	caffeine_aag	-756.9077046	0.1667995	-756.7409050	-756.8696577	-0.0380469	-755.5959867	-755.8250610	-752.6192704	-752.6573173
16	caffeine_abx	-756.9088570	0.1683129	-756.7405440	-756.8684991	-0.0403578	-755.5943852	-755.8235351	-752.6168596	-752.6572174
17	caffeine_aal	-756.9077356	0.1675776	-756.7401580	-756.8707252	-0.0370104	-755.5964931	-755.8257570	-752.6200865	-752.6570969
18	caffeine_abj	-756.9116972	0.1719931	-756.7397040	-756.8729421	-0.0387550	-755.5987323	-755.8269446	-752.6179535	-752.6567085
1	p_caffeine1_aaw	-757.3594689	0.1809352	-757.1785336	-757.2502407	-0.1092281	-755.9786446	-756.2041516	-753.0002094	-753.1094375
2	p_caffeine1_abl	-757.3554855	0.1823694	-757.1731161	-757.2271793	-0.1283062	-755.9559103	-756.1826177	-752.9794060	-753.1077122
3	p_caffeine1_abf	-757.3547182	0.1823599	-757.1723583	-757.2311830	-0.1235353	#N/A	#N/A	#N/A	#N/A
4	p_caffeine1_aap	-757.3548981	0.1832832	-757.1716149	-757.2283773	-0.1265208	#N/A	#N/A	#N/A	#N/A
5	p_caffeine1_aau	-757.3546959	0.1824068	-757.1722891	-757.2279282	-0.1267677	#N/A	#N/A	#N/A	#N/A
6	p_caffeine1_aaq	-757.3546002	0.1824720	-757.1721281	-757.2359878	-0.1186123	#N/A	#N/A	#N/A	#N/A
7	p_caffeine1_aax	-757.3547928	0.1835924	-757.1712004	-757.2262735	-0.1285194	#N/A	#N/A	#N/A	#N/A
8	p_caffeine1_abj	-757.3549101	0.1830982	-757.1718119	-757.2270341	-0.1278761	-755.9557800	-756.1818418	-752.9785974	-753.1064735
9	p_caffeine1_aae	-757.3543414	0.1833491	-757.1709922	-757.2251536	-0.1291877	#N/A	#N/A	#N/A	#N/A
10	p_caffeine1_abe	-757.3535477	0.1819680	-757.1715796	-757.2245983	-0.1289494	#N/A	#N/A	#N/A	#N/A
11	p_caffeine1_abq	-757.3531516	0.1810871	-757.1720644	-757.2348988	-0.1182528	#N/A	#N/A	#N/A	#N/A
12	p_caffeine1_abt	-757.3530163	0.1822842	-757.1707320	-757.2203972	-0.1326190	#N/A	#N/A	#N/A	#N/A
13	p_caffeine1_aah	-757.3528383	0.1819266	-757.1709117	-757.2191621	-0.1336762	#N/A	#N/A	#N/A	#N/A
14	p_caffeine1_aak	-757.3511821	0.1785955	-757.1725866	-757.2219020	-0.1292801	#N/A	#N/A	#N/A	#N/A
15	p_caffeine1_aan	-757.3513746	0.1810665	-757.1703080	-757.2210525	-0.1303221	#N/A	#N/A	#N/A	#N/A
16	p_caffeine1_aav	-757.3509070	0.1801771	-757.1707298	-757.2315025	-0.1194045	#N/A	#N/A	#N/A	#N/A

17	p_caffeine1_abn	-757.3506515	0.1795908	-757.1710607	-757.2296826	-0.1209690	#N/A	#N/A	#N/A	#N/A
18	p_caffeine1_abo	-757.3504705	0.1799614	-757.1705090	-757.2240626	-0.1264078	#N/A	#N/A	#N/A	#N/A
19	p_caffeine1_aal	-757.3501451	0.1803088	-757.1698362	-757.2239140	-0.1262311	#N/A	#N/A	#N/A	#N/A
20	p_caffeine1_abh	-757.2598121	0.1826324	-757.0771797	-757.1518106	-0.1080016	#N/A	#N/A	#N/A	#N/A
1	oxocaffeine_abo	-832.1640057	0.1708347	-831.9931709	#N/A	#N/A	#N/A	#N/A	#N/A	#N/A
2	oxocaffeine_abn	-832.1638298	0.1727162	-831.9911136	#N/A	#N/A	#N/A	#N/A	#N/A	#N/A
3	oxocaffeine_aay	-832.1636997	0.1732151	-831.9904846	#N/A	#N/A	#N/A	#N/A	#N/A	#N/A
4	oxocaffeine_abc	-832.1635698	0.1712588	-831.9923109	#N/A	#N/A	#N/A	#N/A	#N/A	#N/A
5	oxocaffeine_aar	-832.1635218	0.1702453	-831.9932765	#N/A	#N/A	#N/A	#N/A	#N/A	#N/A
6	oxocaffeine_aae	-832.1635155	0.1726630	-831.9908525	#N/A	#N/A	#N/A	#N/A	#N/A	#N/A
7	oxocaffeine_aau	-832.1635106	0.1708225	-831.9926881	#N/A	#N/A	#N/A	#N/A	#N/A	#N/A
8	oxocaffeine_aaj	-832.1633769	0.1714517	-831.9919251	#N/A	#N/A	#N/A	#N/A	#N/A	#N/A
9	oxocaffeine_abq	-832.1632325	0.1719967	-831.9912357	#N/A	#N/A	#N/A	#N/A	#N/A	#N/A
10	oxocaffeine_aac	-832.1630314	0.1716688	-831.9913626	#N/A	#N/A	#N/A	#N/A	#N/A	#N/A
11	oxocaffeine_aaa	-832.1628944	0.1725308	-831.9903636	#N/A	#N/A	#N/A	#N/A	#N/A	#N/A
12	oxocaffeine_aaf	-832.1625285	0.1715919	-831.9909366	#N/A	#N/A	#N/A	#N/A	#N/A	#N/A
13	oxocaffeine_abj	-832.1625205	0.1712058	-831.9913147	#N/A	#N/A	#N/A	#N/A	#N/A	#N/A
14	oxocaffeine_abw	-832.1625037	0.1704740	-831.9920296	#N/A	#N/A	#N/A	#N/A	#N/A	#N/A
15	oxocaffeine_abh	-832.1624186	0.1713802	-831.9910384	#N/A	#N/A	#N/A	#N/A	#N/A	#N/A
16	oxocaffeine_aaw	-832.1623039	0.1726838	-831.9896200	#N/A	#N/A	#N/A	#N/A	#N/A	#N/A
17	oxocaffeine_abl	-832.1621885	0.1731255	-831.9890630	#N/A	#N/A	#N/A	#N/A	#N/A	#N/A
18	oxocaffeine_abd	-832.1621299	0.1744889	-831.9876409	#N/A	#N/A	#N/A	#N/A	#N/A	#N/A
19	oxocaffeine_abr	-832.1620689	0.1705958	-831.9914730	#N/A	#N/A	#N/A	#N/A	#N/A	#N/A
20	oxocaffeine_abf	-832.1620477	0.1720577	-831.9899899	#N/A	#N/A	#N/A	#N/A	#N/A	#N/A
1	PhOH_adf	-383.9643174	0.0931493	-383.8711680	-383.9422541	-0.0220633	-383.2709300	-383.3884651	-381.7250142	-381.7470775
2	PhOH_adu	-383.9648079	0.0936728	-383.8711350	-383.9427377	-0.0220702	-383.2719990	-383.3891008	-381.7253029	-381.7473731
3	PhOH_ads	-383.9643320	0.0932039	-383.8711280	-383.9429033	-0.0214287	-383.2716303	-383.3890968	-381.7257035	-381.7471322
4	PhOH_aal	-383.9643128	0.0934458	-383.8708670	-383.9426552	-0.0216576	-383.2713222	-383.3888185	-381.7254055	-381.7470631
5	PhOH_acu	-383.9632504	0.0926243	-383.8706260	-383.9398679	-0.0233825	-383.2698442	-383.3867905	-381.7232941	-381.7466766
6	PhOH_aae	-383.9639657	0.0934937	-383.8704720	-383.9417831	-0.0221826	-383.2712792	-383.3883974	-381.7243752	-381.7465578
7	PhOH_acy	-383.9615439	0.0917829	-383.8697610	-383.9366401	-0.0249039	-383.2677642	-383.3845420	-381.7224421	-381.7473459
8	PhOH_acq	-383.9623999	0.0944119	-383.8679880	-383.9346815	-0.0277184	-383.2662958	-383.3830150	-381.7203932	-381.7481117
9	PhOH_acv	-383.9615304	0.0936363	-383.8678940	-383.9356121	-0.0259182	-383.2667173	-383.3835693	-381.7214597	-381.7473779
10	PhOH_aaa	-383.9617536	0.0938865	-383.8678670	-383.9370144	-0.0247391	-383.2683933	-383.3848315	-381.7225341	-381.7472732
11	PhOH_adp	-383.9614491	0.0939240	-383.8675250	-383.9355487	-0.0259004	#N/A	#N/A	#N/A	#N/A
1	1mInd_abv	-479.6301485	0.1428014	-479.4873470	-479.6141014	-0.0160471	-478.7211460	-478.8632795	-476.6866701	-476.7027172
2	1mInd_aal	-479.6301399	0.1430888	-479.4870510	-479.6140210	-0.0161188	-478.7210739	-478.8632008	-476.6863721	-476.7024909
3	1mInd_abq	-479.6298564	0.1433564	-479.4865000	-479.6123463	-0.0175101	-478.7195985	-478.8618271	-476.6848854	-476.7023955
4	1mInd_aag	-479.6297528	0.1432808	-479.4864720	-479.6103049	-0.0194479	-478.7177589	-478.8598688	-476.6827814	-476.7022293
5	1mInd_aau	-479.6301577	0.1437147	-479.4864430	-479.6138877	-0.0162700	-478.7209460	-478.8631324	-476.6864223	-476.7026923
6	1mInd_abx	-479.6298556	0.1434315	-479.4864240	-479.6123446	-0.0175109	-478.7195802	-478.8618133	-476.6848805	-476.7023915
7	1mInd_aai	-479.6293944	0.1431034	-479.4862910	-479.6107530	-0.0186414	#N/A	#N/A	#N/A	#N/A
8	1mInd_aaw	-479.6300293	0.1437553	-479.4862740	-479.6140387	-0.0159906	-478.7212268	-478.8631896	-476.6862167	-476.7022073
9	1mInd_abg	-479.6301580	0.1439030	-479.4862550	-479.6139493	-0.0162088	-478.7210478	-478.8631816	-476.6862664	-476.7024751
10	1mInd_abj	-479.6301171	0.1439400	-479.4861770	-479.6139765	-0.0161406	-478.7210337	-478.8631538	-476.6863356	-476.7024762
11	1mInd_aam	-479.6295027	0.1436997	-479.4858030	-479.6106712	-0.0188315	-478.7179693	-478.8604882	-476.6835989	-476.7024303
12	1mInd_aak	-479.6293744	0.1436413	-479.4857330	-479.6101920	-0.0191824	#N/A	#N/A	#N/A	#N/A
13	1mInd_abf	-479.6292568	0.1435877	-479.4856690	-479.6104347	-0.0188221	#N/A	#N/A	#N/A	#N/A

14	1mInd_abk	-479.6267286	0.1416895	-479.4850390	-479.6041142	-0.0226144	#N/A	#N/A	#N/A	#N/A
15	1mInd_abr	-479.6268459	0.1424638	-479.4843820	-479.6056764	-0.0211695	#N/A	#N/A	#N/A	#N/A
16	1mInd_aav	-479.6249381	0.1406481	-479.4842900	-479.6032726	-0.0216655	#N/A	#N/A	#N/A	#N/A
17	1mInd_abw	-479.6267993	0.1432203	-479.4835790	-479.6061256	-0.0206737	#N/A	#N/A	#N/A	#N/A
18	1mInd_aac	-479.6270793	0.1435713	-479.4835080	-479.6073287	-0.0197506	#N/A	#N/A	#N/A	#N/A
19	1mInd_abo	-479.6243602	0.1409191	-479.4834410	-479.6068307	-0.0175295	#N/A	#N/A	#N/A	#N/A
20	1mInd_abt	-479.6270389	0.1445919	-479.4824470	-479.6074251	-0.0196138	#N/A	#N/A	#N/A	#N/A
1	Ind_aaz	-440.3159339	0.1150418	-440.2008920	-440.2944685	-0.0214653	-439.4827348	-439.6142452	-437.6418342	-437.6632995
2	Ind_aaj	-440.3167721	0.1164090	-440.2003630	-440.2974325	-0.0193396	-439.4857908	-439.6168382	-437.6443199	-437.6636595
3	Ind_abc	-440.3166005	0.1166015	-440.1999990	-440.2964790	-0.0201215	-439.4848527	-439.6159478	-437.6432557	-437.6633772
4	Ind_abf	-440.3165884	0.1167284	-440.1998600	-440.2966386	-0.0199498	-439.4850145	-439.6161067	-437.6434215	-437.6633713
5	Ind_aaf	-440.3167305	0.1170375	-440.1996930	-440.2974279	-0.0193026	-439.4858239	-439.6168197	-437.6442793	-437.6635819
6	Ind_abu	-440.3167213	0.1171293	-440.1995920	-440.2972493	-0.0194721	#N/A	#N/A	#N/A	#N/A
7	Ind_aav	-440.3158313	0.1165782	-440.1992530	-440.2978811	-0.0179502	#N/A	#N/A	#N/A	#N/A
8	Ind_abq	-440.3157529	0.1165739	-440.1991790	-440.2982363	-0.0175166	#N/A	#N/A	#N/A	#N/A
9	Ind_aae	-440.3167741	0.1176170	-440.1991570	-440.2977062	-0.0190679	#N/A	#N/A	#N/A	#N/A
10	Ind_aaa	-440.3168219	0.1177549	-440.1990670	-440.2976526	-0.0191693	-439.4859795	-439.6169539	-437.6443828	-437.6635522
11	Ind_aak	-440.3159906	0.1169636	-440.1990270	-440.2975682	-0.0184224	#N/A	#N/A	#N/A	#N/A
12	Ind_abw	-440.3135361	0.1164100	-440.1971260	-440.2907708	-0.0227653	#N/A	#N/A	#N/A	#N/A
13	Ind_aaq	-440.3134647	0.1168597	-440.1966050	-440.2891926	-0.0242721	#N/A	#N/A	#N/A	#N/A
14	Ind_abb	-440.3141503	0.1176292	-440.1965210	-440.2918109	-0.0223394	#N/A	#N/A	#N/A	#N/A
15	Ind_abk	-440.3143159	0.1181459	-440.1961700	-440.2910153	-0.0233006	#N/A	#N/A	#N/A	#N/A
16	Ind_aad	-440.3143544	0.1184654	-440.1958890	-440.2897878	-0.0245666	#N/A	#N/A	#N/A	#N/A
17	Ind_abh	-440.3142258	0.1186188	-440.1956070	-440.2904481	-0.0237777	#N/A	#N/A	#N/A	#N/A
1	tAn_aal	-746.2398443	0.1127632	-746.1270810	-746.2234324	-0.0164119	-745.1158418	-745.2401237	-743.4236784	-743.4400903
2	tAn_aaw	-746.2395257	0.1133716	-746.1261540	-746.2234618	-0.0160638	-745.1157464	-745.2402462	-743.4233020	-743.4393658
3	tAn_aau	-746.2394989	0.1136118	-746.1258870	-746.2240449	-0.0154539	-745.1162068	-745.2407074	-743.4239822	-743.4394361
4	tAn_abj	-746.2406631	0.1152541	-746.1254090	-746.2229805	-0.0176826	-745.1154038	-745.2391605	-743.4221647	-743.4398473
5	tAn_aac	-746.2403339	0.1150038	-746.1253300	-746.2256249	-0.0147090	-745.1179330	-745.2420108	-743.4246547	-743.4393637
6	tAn_aba	-746.2404674	0.1151443	-746.1253230	-746.2232629	-0.0172045	-745.1159404	-745.2398004	-743.4226340	-743.4398385
7	tAn_abh	-746.2361897	0.1122546	-746.1239350	-746.2160679	-0.0201218	#N/A	#N/A	#N/A	#N/A
8	tAn_aan	-746.2373596	0.1135005	-746.1238590	-746.2177518	-0.0196077	#N/A	#N/A	#N/A	#N/A
9	tAn_abp	-746.2374049	0.1137299	-746.1236750	-746.2178939	-0.0195110	#N/A	#N/A	#N/A	#N/A
10	tAn_aaf	-746.2378252	0.1146842	-746.1231410	-746.2175765	-0.0202487	#N/A	#N/A	#N/A	#N/A
11	tAn_abo	-746.2189119	0.1160058	-746.1029060	-746.2070394	-0.0118724	#N/A	#N/A	#N/A	#N/A

Table S3. Energies calculated at the (U)B3LYP-D3 and DLPNO-CCSD(T) level of theory for the systems **without** the addition of explicit water. (Quasi-Harmonic Approximation)

№ conf	Molecule				(U)B3LYP-D3/6-31+G(d,p)//		DLPNO-CCSD(T)/BASIS//			
		SMD/(U)B3LYP-D3/6-31+G(d,p)			SMD/(U)B3LYP-D3/6-31+G(d,p)		SMD/(U)B3LYP-D3/6-31+G(d,p)			
		SMD/DFT			DFT		cc-pVTZ	cc-pVQZ	CBS	
		solution			gas		gas	gas	gas	solution (+Esolv)
		1atm	1atm	1atm	1atm	1atm	1atm	1atm	1atm	1atm
		Etot sol	Gcorr	G298 sol	Etot	Esolv	Etot	Etot	Etot	G298 sol
1	C01	-395.0059909	0.0675438	-394.9384470	-394.9676379	-0.0383530	-394.2903188	-394.4100191	-394.4837083	-394.5220613
2	C02	-395.0001376	0.0669506	-394.9331870	-394.9558406	-0.0442970	-394.2779882	-394.3974415	-394.4709977	-394.5152947
3	C03	-394.9928364	0.0664724	-394.9263640	-394.9684989	-0.0243376	-394.2954721	-394.4144039	-394.4877368	-394.5120744
4	C04	-394.9927211	0.0668181	-394.9259030	-394.9672378	-0.0254834	-394.2943688	-394.4132642	-394.4865761	-394.5120595
5	C05	-394.9690172	0.0683061	-394.9007110	-394.8824527	-0.0865644	-394.2064143	-394.3259053	-394.3993157	-394.4858801
1	5mC01	-434.3300622	0.0940462	-434.2360160	-434.2913682	-0.0386940	-433.5332950	-433.6646249	-433.7456263	-433.7843203
2	5mC02	-434.3237760	0.0935780	-434.2301980	-434.2788726	-0.0449035	-433.5198884	-433.6510228	-433.7319069	-433.7768104
3	5mC04	-434.3171853	0.0924243	-434.2247610	-434.2917379	-0.0254475	-433.5379433	-433.6684837	-433.7491004	-433.7745479
4	5mC03	-434.3174433	0.0932403	-434.2242030	-434.2930911	-0.0243522	-433.5391942	-433.6697574	-433.7503897	-433.7747419
5	5mC05	-434.2926342	0.0937601	-434.1988740	-434.2042607	-0.0883735	-433.4469178	-433.5778083	-433.6584260	-433.7467995
1	5hmC01	-509.5560732	0.0978611	-509.4582120	-509.5125791	-0.0434940	-508.6573709	-508.8130134	-508.9089065	-508.9524005
2	5hmC02	-509.5550421	0.0979430	-509.4570990	-509.5097618	-0.0452802	-508.6548295	-508.8105981	-508.9065226	-508.9518028
3	5hmC03	-509.5545226	0.0986125	-509.4559100	-509.5047658	-0.0497568	-508.6496696	-508.8056801	-508.9016943	-508.9514511
4	5hmC04	-509.5533831	0.0982910	-509.4550920	-509.5053001	-0.0480830	-508.6512185	-508.8068711	-508.9027587	-508.9508417
5	5hmC05	-509.5522843	0.0975332	-509.4547510	-509.5058714	-0.0464129	-508.6510781	-508.8071802	-508.9033007	-508.9497136
6	5hmC07	-509.5503577	0.0976426	-509.4527150	-509.5018409	-0.0485167	-508.6456531	-508.8011903	-508.8970205	-508.9455372
7	5hmC06	-509.5504203	0.0980183	-509.4524020	-509.5002603	-0.0501600	-508.6440116	-508.7997644	-508.8956894	-508.9458494
8	5hmC08	-509.5493774	0.0976264	-509.4517510	-509.4986484	-0.0507291	-508.6428417	-508.7984698	-508.8942987	-508.9450278
9	5hmC09	-509.5493233	0.0984032	-509.4509200	-509.4921002	-0.0572231	-508.6366286	-508.7925012	-508.8884342	-508.9456573
10	5hmC10	-509.5465022	0.0976121	-509.4488900	-509.4910931	-0.0554091	-508.6361551	-508.7916567	-508.8874509	-508.9428600
11	5hmC11	-509.5452797	0.0967536	-509.4485260	-509.4900497	-0.0552300	-508.6347998	-508.7907881	-508.8868223	-508.9420523
12	5hmC12	-509.5435133	0.0974502	-509.4460630	-509.5144204	-0.0290929	-508.6633672	-508.8182584	-508.9137922	-508.9428851
13	5hmC13	-509.5433389	0.0973608	-509.4459780	-509.5133322	-0.0300067	-508.6623831	-508.8172511	-508.9127710	-508.9427777
14	5hmC14	-509.5425122	0.0973532	-509.4451590	-509.5114408	-0.0310714	-508.6607310	-508.8157031	-508.9112333	-508.9423047
15	5hmC15	-509.5423394	0.0973723	-509.4449670	-509.5102974	-0.0320420	-508.6596696	-508.8146590	-508.9102038	-508.9422458
16	5hmC16	-509.5420030	0.0980600	-509.4439430	-509.5057445	-0.0362585	-508.6549253	-508.8102481	-508.9059347	-508.9421932
17	5hmC17	-509.5418792	0.0979392	-509.4439400	-509.5042951	-0.0375841	-508.6537572	-508.8089907	-508.9046184	-508.9422025
18	5hmC18	-509.5406051	0.0971261	-509.4434790	-509.5062975	-0.0343076	-508.6561245	-508.8111141	-508.9066839	-508.9409915
19	5hmC22	-509.5394605	0.0961684	-509.4432920	-509.5057605	-0.0337000	-508.6552935	-508.8106994	-508.9064782	-508.9401782
20	5hmC21	-509.5404209	0.0971618	-509.4432590	-509.5047821	-0.0356388	-508.6547730	-508.8096908	-508.9052116	-508.9408504
1	5fC01	-508.3478985	0.0759575	-508.2719410	-508.3102196	-0.0376789	-507.4572734	-507.6101790	-507.7042893	-507.7419682
2	5fC02	-508.3453676	0.0756005	-508.2697670	-508.3050771	-0.0402905	-507.4519952	-507.6046792	-507.6987147	-507.7390052
3	5fC03	-508.3409901	0.0749441	-508.2660460	-508.3001815	-0.0408086	-507.4471420	-507.5999928	-507.6941193	-507.7349279
4	5fC04	-508.3386803	0.0746982	-508.2639820	-508.2892637	-0.0494166	-507.4372154	-507.5897032	-507.6836229	-507.7330395
5	5fC06	-508.3360775	0.0751445	-508.2609330	-508.3107124	-0.0253651	-507.4616318	-507.6138797	-507.7077014	-507.7330665
6	5fC05	-508.3362394	0.0758734	-508.2603660	-508.3117181	-0.0245213	-507.4625036	-507.6147791	-507.7086096	-507.7331309
7	5fC07	-508.3292360	0.0740240	-508.2552120	-508.2991874	-0.0300486	-507.4507472	-507.6028348	-507.6965784	-507.7266270
8	5fC08	-508.3291945	0.0744015	-508.2547930	-508.2974751	-0.0317195	-507.4492797	-507.6013076	-507.6950173	-507.7267368
9	5fC09	-508.3166565	0.0756805	-508.2409760	-508.2569092	-0.0597474	-507.4076387	-507.5604182	-507.6543331	-507.7140805
10	5fC10	-508.3131003	0.0753683	-508.2377320	-508.2483178	-0.0647825	-507.3968583	-507.5490968	-507.6428393	-507.7076218
11	5fC11	-508.3119112	0.0747291	-508.2371820	-508.2501963	-0.0617149	-507.4017399	-507.5545059	-507.6484069	-507.7101218

12	5fC12	-508.3098125	0.0748615	-508.2349510	-508.2513249	-0.0584876	-507.4025393	-507.5551152	-507.6489337	-507.7074213
13	5fC13	-508.3093880	0.0763860	-508.2330020	-508.2548597	-0.0545283	-507.4070881	-507.5592280	-507.6528499	-507.7073782
14	5fC14	-508.3069461	0.0752431	-508.2317030	-508.2215437	-0.0854024	-507.3708520	-507.5232220	-507.6169105	-507.7023129
1	5dhmC05	-584.7932991	0.1005640	-584.6927350	-584.7388527	-0.0544464	-583.7884446	-583.9684860	-584.0793251	-584.1337715
2	5dhmC01	-584.7938842	0.1016512	-584.6922330	-584.7475134	-0.0463708	-583.7965342	-583.9762531	-584.0870097	-584.1333805
3	5dhmC03	-584.7936810	0.1014780	-584.6922030	-584.7445973	-0.0490837	-583.7932341	-583.9730700	-584.0838119	-584.1328956
4	5dhmC02	-584.7938760	0.1018989	-584.6919770	-584.7437736	-0.0501024	-583.7936302	-583.9731797	-584.0838087	-584.1339111
5	5dhmC07	-584.7929032	0.1010672	-584.6918360	-584.7399796	-0.0529236	-583.7899536	-583.9698493	-584.0806534	-584.1335770
6	5dhmC04	-584.7934534	0.1022573	-584.6911960	-584.7411638	-0.0522896	-583.7913813	-583.9708853	-584.0815015	-584.1337911
7	5dhmC08	-584.7922636	0.1012515	-584.6910120	-584.7412745	-0.0509891	-583.7904163	-583.9704082	-584.0812270	-584.1322161
8	5dhmC09	-584.7916831	0.1007451	-584.6909380	-584.7387017	-0.0529814	-583.7887892	-583.9686495	-584.0794262	-584.1324076
9	5dhmC11	-584.7915463	0.1011652	-584.6903810	-584.7402103	-0.0513360	-583.7903241	-583.9700675	-584.0808111	-584.1321471
10	5dhmC12	-584.7902927	0.1002247	-584.6900680	-584.7378233	-0.0524695	-583.7875494	-583.9675046	-584.0783255	-584.1307950
11	5dhmC10	-584.7916034	0.1016344	-584.6899690	-584.7401112	-0.0514923	-583.7892973	-583.9690473	-584.0797437	-584.1312360
12	5dhmC15	-584.7880338	0.1004868	-584.6875470	-584.7300748	-0.0579591	-583.7787774	-583.9586191	-584.0693384	-584.1272975
13	5dhmC13	-584.7885868	0.1015788	-584.6870080	-584.7338488	-0.0547380	-583.7817639	-583.9615328	-584.0722472	-584.1269852
14	5dhmC16	-584.7880337	0.1014907	-584.6865430	-584.7349776	-0.0530561	-583.7836016	-583.9631611	-584.0738205	-584.1268766
15	5dhmC14	-584.7881894	0.1016843	-584.6865050	-584.7330251	-0.0551642	-583.7820480	-583.9614409	-584.0719911	-584.1271553
16	5dhmC19	-584.7869097	0.1008676	-584.6860420	-584.7327428	-0.0541669	-583.7806760	-583.9605929	-584.0713853	-584.1255522
17	5dhmC17	-584.7877815	0.1018604	-584.6859210	-584.7305010	-0.0572805	-583.7799424	-583.9592212	-584.0697165	-584.1269970
18	5dhmC18	-584.7870742	0.1011601	-584.6859140	-584.7274584	-0.0596157	-583.7770273	-583.9567411	-584.0674290	-584.1270447
19	5dhmC20	-584.7864453	0.1010832	-584.6853620	-584.7288538	-0.0575915	-583.7774750	-583.9571028	-584.0677410	-584.1253325
20	5dhmC22	-584.7849062	0.0996631	-584.6852430	-584.7288932	-0.0560130	-583.7777101	-583.9574792	-584.0682046	-584.1242176
1	5caC01	-583.6071366	0.0794666	-583.5276700	-583.5390733	-0.0680633	-582.5865929	-582.7655531	-582.8755521	-582.9436154
2	5caC02	-583.6056710	0.0785660	-583.5271050	-583.5672080	-0.0384630	-582.6190103	-582.7963335	-582.9055338	-582.9439968
3	5caC03	-583.6033571	0.0788311	-583.5245260	-583.5631625	-0.0401946	-582.6143728	-582.7916936	-582.9009125	-582.9411071
4	5caC04	-583.5997835	0.0790494	-583.5207340	-583.5512109	-0.0485726	-582.6049617	-582.7824126	-582.8915573	-582.9401299
5	5caC05	-583.5952259	0.0788058	-583.5164200	-583.5472741	-0.0479518	-582.6008482	-582.7779826	-582.8870131	-582.9349649
6	5caC06	-583.5931258	0.0790047	-583.5141210	-583.5288993	-0.0642265	-582.5790478	-582.7578743	-582.8677447	-582.9319712
7	5caC07	-583.5898491	0.0791931	-583.5106560	-583.5149963	-0.0748528	-582.5663204	-582.7453846	-582.8553092	-582.9301620
8	5caC08	-583.5839277	0.0795096	-583.5044180	-583.5140063	-0.0699214	-582.5636299	-582.7415276	-582.8509494	-582.9208708
1	1mC01	-434.3178347	0.0934406	-434.2243940	-434.2832880	-0.0345466	-433.5255087	-433.6563146	-433.7370028	-433.7715494
1	1m5mC01	-473.6415541	0.1200140	-473.5215400	-473.6068310	-0.0347231	-472.7684126	-472.9108546	-472.9988614	-473.0335845
1	1m5hmC01	-548.8679919	0.1243578	-548.7436340	-548.8280737	-0.0399182	-547.8924824	-548.0592803	-548.1621970	-548.2021152
2	1m5hmC02	-548.8669072	0.1240331	-548.7428740	-548.8255240	-0.0413832	-547.8902686	-548.0571091	-548.1600093	-548.2013925
3	1m5hmC03	-548.8664182	0.1244622	-548.7419560	-548.8208357	-0.0455825	-547.8853747	-548.0524592	-548.1554476	-548.2010301
4	1m5hmC04	-548.8652194	0.1237874	-548.7414320	-548.8206862	-0.0445333	-547.8862126	-548.0530259	-548.1559437	-548.2004770
5	1m5hmC05	-548.8641306	0.1230535	-548.7410770	-548.8212985	-0.0428321	-547.8861558	-548.0533842	-548.1565150	-548.1993471
1	1m5fC01	-547.6604171	0.1015000	-547.5589170	-547.6270462	-0.0333709	-546.6934959	-546.8575842	-546.9587366	-546.9921075
2	1m5fC02	-547.6540750	0.1008180	-547.5532570	-547.6173002	-0.0367749	-546.6835939	-546.8475991	-546.9487436	-546.9855185
1	1m5dhmC05	-624.1052120	0.1268619	-623.9783500	-624.0544679	-0.0507441	-623.0237115	-623.2148470	-623.3326748	-623.3834189
2	1m5dhmC01	-624.1059202	0.1280611	-623.9778590	-624.0632767	-0.0426435	-623.0319969	-623.2227915	-623.3405261	-623.3831696
3	1m5dhmC03	-624.1055727	0.1277847	-623.9777880	-624.0607696	-0.0448031	-623.0291414	-623.2200579	-623.3377846	-623.3825877
4	1m5dhmC06	-624.1049582	0.1272602	-623.9776980	-624.0557231	-0.0492351	-623.0253588	-623.2163366	-623.3341211	-623.3833562
5	1m5dhmC02	-624.1058664	0.1283723	-623.9774940	-624.0599903	-0.0458761	-623.0294514	-623.2201025	-623.3377348	-623.3836109
6	1m5dhmC07	-624.1041634	0.1270134	-623.9771500	-624.0571926	-0.0469708	-623.0258243	-623.2169649	-623.3348164	-623.3817872
7	1m5dhmC04	-624.1054927	0.1288006	-623.9766920	-624.0572920	-0.0482007	-623.0271199	-623.2176936	-623.3352892	-623.3834899
8	1m5dhmC10	-624.1022009	0.1258068	-623.9763940	-624.0537062	-0.0484947	-623.0230020	-623.2140556	-623.3318661	-623.3803608

9	1m5dhmC09	-624.1035155	0.1276775	-623.9758380	-624.0564217	-0.0470939	-623.0253312	-623.2161464	-623.3338158	-623.3809097
10	1m5dhmC08	-624.1035611	0.1278330	-623.9757280	-624.0560959	-0.0474651	-623.0258566	-623.2166667	-623.3343848	-623.3818499
1	1m5caC01	-622.9205641	0.1058711	-622.8146930	-622.8559549	-0.0646093	-621.8222151	-622.0125423	-622.1296607	-622.1942700
2	1m5caC02	-622.9185761	0.1051180	-622.8134580	-622.8838506	-0.0347255	-621.8550633	-622.0435364	-622.1597605	-622.1944860
3	1m5caC03	-622.9125981	0.1056990	-622.8068990	-622.8680191	-0.0445790	-621.8411196	-622.0297450	-622.1459181	-622.1904971
4	1m5caC04	-622.9080912	0.1056122	-622.8024790	-622.8649313	-0.0431599	-621.8379455	-622.0261384	-622.1421386	-622.1852985
5	1m5caC05	-622.9063551	0.1057060	-622.8006490	-622.8452733	-0.0610818	-621.8141788	-622.0042728	-622.1212252	-622.1823070
6	1m5caC06	-622.9018833	0.1055732	-622.7963100	-622.8311957	-0.0706876	-621.8016713	-621.9919094	-622.1088722	-622.1795598
7	1m5caC07	-622.8981329	0.1054118	-622.7927210	-622.8322421	-0.0658907	-621.8011539	-621.9904120	-622.1069591	-622.1728498
1	U114	-414.8799057	0.0560827	-414.8238230	-414.8531491	-0.0267566	-414.1683693	-414.2937248	-414.3709673	-414.3977239
2	U02	-414.8647348	0.0558468	-414.8088880	-414.8327863	-0.0319486	-414.1496872	-414.2751563	-414.3523725	-414.3843211
3	U03	-414.8631895	0.0559865	-414.8072030	-414.8211208	-0.0420687	-414.1390329	-414.2647161	-414.3419577	-414.3840264
4	U04	-414.8593768	0.0558987	-414.8034780	-414.8352118	-0.0241650	-414.1522962	-414.2774198	-414.3544873	-414.3786523
5	U05	-414.8573193	0.0552472	-414.8020720	-414.8203611	-0.0369582	-414.1384293	-414.2640219	-414.3412473	-414.3782055
6	U134	-414.8560663	0.0554503	-414.8006160	-414.8217981	-0.0342682	-414.1397851	-414.2651056	-414.3422154	-414.3764836
7	U07	-414.8557639	0.0559269	-414.7998370	-414.8177419	-0.0380220	-414.1341861	-414.2594540	-414.3365299	-414.3745519
8	U09	-414.8534727	0.0553777	-414.7980950	-414.8126988	-0.0407739	-414.1291645	-414.2545300	-414.3316282	-414.3724021
9	U08	-414.8537143	0.0556223	-414.7980920	-414.8025450	-0.0511693	-414.1222368	-414.2481416	-414.3254323	-414.3766016
10	U10	-414.8529310	0.0552149	-414.7977160	-414.8324955	-0.0204354	-414.1532017	-414.2780568	-414.3549826	-414.3754180
11	U11	-414.8525952	0.0554451	-414.7971500	-414.8306611	-0.0219341	-414.1517282	-414.2765060	-414.3533741	-414.3753082
12	U126	-414.8521422	0.0557432	-414.7963990	-414.8237881	-0.0283542	-414.1456185	-414.2705434	-414.3474439	-414.3757981
13	U13	-414.8519537	0.0560646	-414.7958890	-414.8237428	-0.0282109	-414.1456786	-414.2705507	-414.3474246	-414.3756355
14	U156	-414.7925275	0.0419675	-414.7505600	-414.7780857	-0.0144418	-414.1019472	-414.2259807	-414.3020133	-414.3164551
15	U123	-414.7960420	0.0553749	-414.7406670	#N/A	#N/A	#N/A	#N/A	#N/A	#N/A
1	5mU01	-454.2047404	0.0816993	-454.1230410	-454.1790946	-0.0256458	-453.4135415	-453.5503929	-453.6349019	-453.6605477
2	5mU02	-454.1889777	0.0819867	-454.1069910	-454.1572580	-0.0317197	-453.3930873	-453.5300824	-453.6145648	-453.6462845
3	5mU03	-454.1869883	0.0818033	-454.1051850	-454.1448884	-0.0420999	-453.3819577	-453.5192477	-453.6037986	-453.6458985
4	5mU04	-454.1844523	0.0815282	-454.1029240	-454.1615856	-0.0228667	-453.3980131	-453.5346271	-453.6189602	-453.6418269
5	5mU05	-454.1825585	0.0811774	-454.1013810	-454.1474781	-0.0350804	-453.3847371	-453.5218201	-453.6063172	-453.6413976
6	5mU06	-454.1809402	0.0805152	-454.1004250	-454.1481491	-0.0327911	-453.3854530	-453.5222683	-453.6066419	-453.6394330
7	5mU08	-454.1790994	0.0814923	-454.0976070	-454.1299897	-0.0491096	-453.3688973	-453.5062977	-453.5908657	-453.6399753
8	5mU07	-454.1792129	0.0816449	-454.0975680	-454.1407659	-0.0384470	-453.3758400	-453.5127665	-453.5971678	-453.6356148
9	5mU11	-454.1770708	0.0804978	-454.0965730	-454.1556398	-0.0214310	-453.3951942	-453.5315981	-453.6157916	-453.6372226
10	5mU10	-454.1772341	0.0812160	-454.0960180	-454.1359625	-0.0412716	-453.3709722	-453.5079105	-453.5922897	-453.6335613
11	5mU09	-454.1775778	0.0816497	-454.0959280	-454.1576115	-0.0199663	-453.3968713	-453.5332718	-453.6174697	-453.6374360
12	5mU13	-454.1759775	0.0804004	-454.0955770	-454.1480759	-0.0279016	-453.3888006	-453.5253979	-453.6096459	-453.6375475
13	5mU12	-454.1763612	0.0815541	-454.0948070	-454.1482177	-0.0281435	-453.3889595	-453.5255319	-453.6097543	-453.6378978
14	5mU14	-454.1209095	0.0798925	-454.0410170	-454.0571420	-0.0637675	-453.2931953	-453.4306364	-453.5152105	-453.5789780
15	5mU15	-414.7912057	0.0406587	-414.7505470	-414.7782235	-0.0129822	-414.1026812	-414.2262768	-414.3021227	-414.3151049
1	5hmU01	-529.4306252	0.0864182	-529.3442070	-529.3984812	-0.0321440	-528.5356003	-528.6971885	-528.7967625	-528.8289065
2	5hmU02	-529.4143450	0.0857070	-529.3286380	-529.3746060	-0.0397391	-528.5131508	-528.6748548	-528.7743707	-528.8141098
3	5hmU03	-529.4110921	0.0859940	-529.3250980	-529.3576380	-0.0534541	-528.4978248	-528.6597554	-528.7592972	-528.8127513
4	5hmU04	-529.4102889	0.0855908	-529.3246980	-529.3807978	-0.0294911	-528.5198028	-528.6811779	-528.7805883	-528.8100794
5	5hmU05	-529.4087533	0.0855022	-529.3232510	-529.3686181	-0.0401352	-528.5085771	-528.6703483	-528.7698906	-528.8100258
6	5hmU06	-529.4068949	0.0852448	-529.3216500	-529.3671410	-0.0397539	-528.5070067	-528.6685836	-528.7680353	-528.8077892
7	5hmU07	-529.4051781	0.0855770	-529.3196010	-529.3512491	-0.0539290	-528.4927481	-528.6548417	-528.7544619	-528.8083909
8	5hmU08	-529.4042659	0.0864158	-529.3178500	-529.3530196	-0.0512463	-528.4918763	-528.6535342	-528.7529789	-528.8042252
9	5hmU10	-529.4027195	0.0852275	-529.3174920	-529.3741430	-0.0285766	-528.5162938	-528.6774475	-528.7766918	-528.8052684

10	5hmU09	-529.4029693	0.0857402	-529.3172290	-529.3519429	-0.0510264	-528.4899754	-528.6517549	-528.7512380	-528.8022644
11	5hmU11	-529.4023899	0.0852118	-529.3171780	-529.3720150	-0.0303748	-528.5145937	-528.6757456	-528.7749802	-528.8053550
12	5hmU13	-529.4002471	0.0859940	-529.3142530	-529.3599809	-0.0402662	-528.5042918	-528.6655619	-528.7648093	-528.8050755
13	5hmU12	-529.4003871	0.0863831	-529.3140040	-529.3600751	-0.0403121	-528.5041432	-528.6654789	-528.7647614	-528.8050735
14	5hmU14	-529.3476839	0.0837098	-529.2639740	-529.2790921	-0.0685918	-528.4180258	-528.5801421	-528.6797614	-528.7483532
15	5hmU15	-529.3395182	0.0853762	-529.2541420	-529.2414258	-0.0980924	-528.3788476	-528.5409543	-528.6404581	-528.7385505
1	5fU02	-528.2155251	0.0627811	-528.1527440	-528.1858671	-0.0296580	-527.3264932	-527.4848534	-527.5824925	-527.6121505
2	5fU01	-528.2157292	0.0632662	-528.1524630	-528.1760465	-0.0396827	-527.3182244	-527.4765238	-527.5740302	-527.6137129
3	5fU03	-528.2052229	0.0637358	-528.1414870	-528.1657976	-0.0394253	-527.3085646	-527.4672444	-527.5648061	-527.6042314
4	5fU05	-528.1993784	0.0623034	-528.1370750	-528.1653561	-0.0340223	-527.3071575	-527.4657286	-527.5633674	-527.5973897
5	5fU04	-528.1997309	0.0632918	-528.1364390	-528.1590235	-0.0407073	-527.3021194	-527.4606040	-527.5580970	-527.5988043
6	5fU06	-528.1952822	0.0623092	-528.1329730	-528.1516006	-0.0436817	-527.2939623	-527.4527949	-527.5504636	-527.5941453
7	5fU08	-528.1922871	0.0622821	-528.1300050	-528.1530926	-0.0391945	-527.2969715	-527.4555818	-527.5532212	-527.5924157
8	5fU07	-528.1923696	0.0629016	-528.1294680	-528.1417692	-0.0506004	-527.2870749	-527.4455307	-527.5429831	-527.5935835
9	5fU09	-528.1885282	0.0631451	-528.1253830	-528.1235623	-0.0649659	-527.2706189	-527.4293606	-527.5268610	-527.5918269
10	5fU10	-528.1818146	0.0638676	-528.1179470	-528.1032427	-0.0785719	-527.2417474	-527.4020500	-527.5001547	-527.5787266
11	5fU11	-528.1810402	0.0632021	-528.1178380	-528.1120164	-0.0690237	-527.2490296	-527.4092532	-527.5075071	-527.5765308
12	5fU12	-528.1785366	0.0631755	-528.1153610	-528.1062007	-0.0723359	-527.2438664	-527.4039875	-527.5021575	-527.5744934
1	5dhmU01	-604.6695338	0.0900287	-604.5795050	-604.6314025	-0.0381313	-603.6727754	-603.8584592	-603.9728622	-604.0109935
2	5dhmU02	-604.6691740	0.0901009	-604.5790730	-604.6305565	-0.0386174	-603.6724994	-603.8581079	-603.9724512	-604.0110686
3	5dhmU03	-604.6678553	0.0893992	-604.5784560	-604.6279949	-0.0398604	-603.6698287	-603.8554706	-603.9698953	-604.0097557
4	5dhmU05	-604.6671786	0.0898156	-604.5773630	-604.6243970	-0.0427816	-603.6670903	-603.8527150	-603.9670591	-604.0098407
5	5dhmU06	-604.6670277	0.0896806	-604.5773470	-604.6243146	-0.0427130	-603.6666084	-603.8524902	-603.9669981	-604.0097111
6	5dhmU04	-604.6674254	0.0900793	-604.5773460	-604.6251791	-0.0422462	-603.6675846	-603.8531896	-603.9675518	-604.0097980
7	5dhmU12	-604.6655599	0.0887539	-604.5768060	-604.6133317	-0.0522282	-603.6573796	-603.8431709	-603.9575352	-604.0097634
8	5dhmU09	-604.6663197	0.0895156	-604.5768040	-604.6307503	-0.0355694	-603.6721696	-603.8580395	-603.9725745	-604.0081439
9	5dhmU10	-604.6658570	0.0892760	-604.5765810	-604.6233482	-0.0425089	-603.6659078	-603.8516173	-603.9660464	-604.0085553
10	5dhmU08	-604.6664708	0.0900358	-604.5764350	-604.6283896	-0.0380812	-603.6707303	-603.8563557	-603.9707422	-604.0088234
11	5dhmU07	-604.6667684	0.0904314	-604.5763370	-604.6190100	-0.0477585	-603.6623180	-603.8478006	-603.9621021	-604.0098606
12	5dhmU11	-604.6657422	0.0897622	-604.5759800	-604.6212694	-0.0444728	-603.6643246	-603.8497683	-603.9640737	-604.0085465
13	5dhmU13	-604.6644203	0.0891283	-604.5752920	-604.6141213	-0.0502990	-603.6577448	-603.8435044	-603.9578705	-604.0081695
14	5dhmU14	-604.6622508	0.0882597	-604.5739910	-604.6086879	-0.0535629	-603.6532236	-603.8388328	-603.9531226	-604.0066855
15	5dhmU16	-604.6530716	0.0899436	-604.5631280	-604.6012916	-0.0517800	-603.6448127	-603.8304337	-603.9446537	-603.9964337
16	5dhmU22	-604.6518472	0.0890872	-604.5627600	-604.6071143	-0.0447330	-603.6503359	-603.8361841	-603.9506120	-603.9953450
17	5dhmU15	-604.6530909	0.0903908	-604.5627000	-604.6016471	-0.0514438	-603.6459947	-603.8313926	-603.9455327	-603.9969765
18	5dhmU23	-604.6517891	0.0891290	-604.5626600	-604.5954782	-0.0563109	-603.6399266	-603.8256776	-603.9399259	-603.9962368
19	5dhmU18	-604.6524193	0.0898093	-604.5626100	-604.6053314	-0.0470880	-603.6480664	-603.8338428	-603.9481795	-603.9952675
20	5dhmU20	-604.6523075	0.0897424	-604.5625650	-604.6064837	-0.0458238	-603.6495006	-603.8353538	-603.9497090	-603.9955328
1	5caU01	-603.4800197	0.0671247	-603.4128950	-603.4455879	-0.0344319	-602.4920673	-602.6748288	-602.7874184	-602.8218503
2	5caU02	-603.4742817	0.0665946	-603.4076870	-603.4346881	-0.0395935	-602.4808307	-602.6636241	-602.7762493	-602.8158428
3	5caU03	-603.4739973	0.0668362	-603.4071610	-603.4379600	-0.0360373	-602.4837048	-602.6664545	-602.7790764	-602.8151137
4	5caU04	-603.4690300	0.0673359	-603.4016940	-603.4185105	-0.0505195	-602.4668989	-602.6496938	-602.7621935	-602.8127130
5	5caU05	-603.4641481	0.0665561	-603.3975920	-603.4233210	-0.0408271	-602.4708176	-602.6538119	-602.7663871	-602.8072142
6	5caU06	-603.4604947	0.0663077	-603.3941870	-603.4180620	-0.0424327	-602.4646283	-602.6478261	-602.7605296	-602.8029623
7	5caU07	-603.4597943	0.0663993	-603.3933950	-603.4168078	-0.0429865	-602.4659732	-602.6489247	-602.7614956	-602.8044821
8	5caU08	-603.4592985	0.0667544	-603.3925440	-603.4180435	-0.0412550	-602.4650611	-602.6483094	-602.7610511	-602.8023061
9	5caU09	-603.4588088	0.0666247	-603.3921840	-603.4072524	-0.0515564	-602.4564695	-602.6397092	-602.7522812	-602.8038376
10	5caU10	-603.4584160	0.0663479	-603.3920680	-603.4174349	-0.0409811	-602.4647087	-602.6476282	-602.7602064	-602.8011875

11	5caU11	-603.4561694	0.0668324	-603.3893370	-603.3997027	-0.0564667	-602.4503489	-602.6335845	-602.7462097	-602.8026764
12	5caU12	-603.4535066	0.0649386	-603.3885680	-603.3973326	-0.0561740	-602.4433984	-602.6269129	-602.7397121	-602.7958861
13	5caU13	-603.4530796	0.0671105	-603.3859690	-603.4010977	-0.0519819	-602.4505848	-602.6335762	-602.7460628	-602.7980447
14	5caU14	-603.4511076	0.0663185	-603.3847890	-603.4003416	-0.0507660	-602.4495109	-602.6325063	-602.7451036	-602.7958696
15	5caU16	-603.4503991	0.0659010	-603.3844980	-603.3809789	-0.0694202	-602.4283668	-602.6121959	-602.7250796	-602.7944998
16	5caU15	-603.4506527	0.0664186	-603.3842340	-603.4039243	-0.0467283	-602.4527205	-602.6356892	-602.7482781	-602.7950064
17	5caU17	-603.4494559	0.0667798	-603.3826760	-603.3971317	-0.0523242	-602.4455176	-602.6287506	-602.7413661	-602.7936903
18	5caU18	-603.4472172	0.0662791	-603.3809380	-603.3823320	-0.0648851	-602.4330926	-602.6163967	-602.7290565	-602.7939416
19	5caU19	-603.4467608	0.0662007	-603.3805600	-603.3861719	-0.0605889	-602.4364963	-602.6198127	-602.7324901	-602.7930790
20	5caU20	-603.4464898	0.0662757	-603.3802140	-603.3865696	-0.0599202	-602.4377249	-602.6207734	-602.7332856	-602.7932058
1	1mU01	-454.1922460	0.0818060	-454.1104400	-454.1681269	-0.0241192	-453.4023996	-453.5389708	-453.6232624	-453.6473816
2	1mU02	-454.1773230	0.0816799	-454.0956430	-454.1488893	-0.0284337	-453.3851183	-453.5217757	-453.6060326	-453.6344663
3	1mU03	-454.1759272	0.0819201	-454.0940070	-454.1375658	-0.0383614	-453.3748230	-453.5116479	-453.5959029	-453.6342643
4	1mU05	-454.1693266	0.0815796	-454.0877470	-454.1344186	-0.0349080	-453.3712850	-453.5080188	-453.5922767	-453.6271847
5	1mU04	-454.1646479	0.0813038	-454.0833440	-454.1162461	-0.0484018	-453.3549270	-453.4918527	-453.5761212	-453.6245230
1	1m5mU01	-493.5164338	0.1077168	-493.4087170	-493.4938405	-0.0225934	-492.6474890	-492.7955225	-492.8870587	-492.9096521
2	1m5mU04	-493.5012164	0.1078854	-493.3933310	-493.4730859	-0.0281306	-492.6283833	-492.7765383	-492.8680417	-492.8961723
3	1m5mU05	-493.4992940	0.1078129	-493.3914810	-493.4610144	-0.0382796	-492.6175043	-492.7659437	-492.8575120	-492.8957916
4	1m5mU03	-493.4941307	0.1072957	-493.3868350	-493.4613944	-0.0327364	-492.6175922	-492.7657728	-492.8572731	-492.8900095
5	1m5mU02	-493.4896180	0.1080959	-493.3815220	-493.4435680	-0.0460499	-492.6015241	-492.7499085	-492.8414276	-492.8874775
1	1m5hmU01	-568.7428575	0.1128914	-568.6299660	-568.7137047	-0.0291528	-567.7699343	-567.9427348	-568.0493532	-568.0785060
2	1m5hmU03	-568.7271068	0.1125097	-568.6145970	-568.6908176	-0.0362892	-567.7487545	-567.9216578	-568.0282118	-568.0645010
3	1m5hmU02	-568.7239407	0.1128756	-568.6110650	-568.6745336	-0.0494071	-567.7340524	-567.9071433	-568.0137033	-568.0631104
4	1m5hmU04	-568.7207785	0.1120855	-568.6086930	-568.6828494	-0.0379291	-567.7415835	-567.9145094	-568.0210844	-568.0590135
5	1m5hmU05	-568.7161013	0.1120942	-568.6040070	-568.6652122	-0.0508890	-567.7257045	-567.8988392	-568.0054417	-568.0563307
1	1m5fU11	-567.5288307	0.0891436	-567.4396870	-567.5021303	-0.0267004	-566.5615618	-566.7311530	-566.8358432	-566.8625436
2	1m5fU12	-567.5288635	0.0896455	-567.4392180	-567.4920740	-0.0367895	-566.5531221	-566.7226708	-566.8272384	-566.8640279
3	1m5fU10	-567.5185140	0.0895540	-567.4289600	-567.4834140	-0.0351000	-566.5453616	-566.7152524	-566.8198642	-566.8549642
4	1m5fU08	-567.5131483	0.0888482	-567.4243000	-567.4827844	-0.0303638	-566.5437601	-566.7135357	-566.8182123	-566.8485761
5	1m5fU09	-567.5132227	0.0892127	-567.4240100	-567.4762217	-0.0370010	-566.5385159	-566.7082058	-566.8127372	-566.8497382
6	1m5fU07	-567.5092293	0.0890453	-567.4201840	-567.4696952	-0.0395341	-566.5313429	-566.7013452	-566.8060356	-566.8455697
7	1m5fU05	-567.5050788	0.0884647	-567.4166140	-567.4682126	-0.0368661	-566.5306898	-566.7004607	-566.8051371	-566.8420032
8	1m5fU06	-567.5050207	0.0888687	-567.4161520	-567.4566108	-0.0484099	-566.5205369	-566.6901556	-566.7946449	-566.8430548
9	1m5fU04	-567.4999401	0.0892720	-567.4106680	-567.4380148	-0.0619253	-566.5039058	-566.6737192	-566.7782186	-566.8401439
10	1m5fU03	-567.4954899	0.0896229	-567.4058670	-567.4223817	-0.0731082	-566.4811240	-566.6524648	-566.7575618	-566.8306700
11	1m5fU02	-567.4951808	0.0893367	-567.4058440	-567.4312250	-0.0639557	-566.4885035	-566.6597539	-566.7649859	-566.8289416
1	1m5dhmU01	-643.9818636	0.1155746	-643.8662890	-643.9469663	-0.0348974	-642.9073601	-643.1042806	-643.2257398	-643.2606372
2	1m5dhmU69	-643.9814163	0.1160943	-643.8653220	-643.9459477	-0.0354687	-642.9069407	-643.1038008	-643.2252128	-643.2606815
3	1m5dhmU68	-643.9800459	0.1155968	-643.8644490	-643.9430407	-0.0370051	-642.9040589	-643.1009472	-643.2224447	-643.2594498
4	1m5dhmU66	-643.9794218	0.1155847	-643.8638370	-643.9398170	-0.0396047	-642.9015999	-643.0984537	-643.2198514	-643.2594561
5	1m5dhmU65	-643.9792464	0.1159293	-643.8633170	-643.9394433	-0.0398031	-642.9009329	-643.0979990	-643.2195369	-643.2593400
6	1m5dhmU62	-643.9785886	0.1152806	-643.8633080	-643.9460317	-0.0325569	-642.9066811	-643.1037767	-643.2253654	-643.2579223
7	1m5dhmU67	-643.9796318	0.1164097	-643.8632220	-643.9403068	-0.0393250	-642.9019562	-643.0987310	-643.2201219	-643.2594469
8	1m5dhmU64	-643.9789470	0.1164090	-643.8625380	-643.9345563	-0.0443907	-642.8971151	-643.0937384	-643.2150527	-643.2594434
9	1m5dhmU61	-643.9780886	0.1157216	-643.8623670	-643.9382414	-0.0398473	-642.9000220	-643.0969153	-643.2183754	-643.2582227
10	1m5dhmU59	-643.9778950	0.1155660	-643.8623290	-643.9282760	-0.0496191	-642.8914933	-643.0884835	-643.2098888	-643.2595079
11	1m5dhmU63	-643.9788226	0.1167005	-643.8621220	-643.9442021	-0.0346205	-642.9056711	-643.1024769	-643.2238927	-643.2585132
12	1m5dhmU58	-643.9767682	0.1155102	-643.8612580	-643.9290197	-0.0477485	-642.8918130	-643.0887835	-643.2101934	-643.2579419

13	1m5dhmU57	-643.9746267	0.1153117	-643.8593150	-643.9239015	-0.0507252	-642.8875236	-643.0843282	-643.2056546	-643.2563798
14	1m5dhmU53	-643.9652235	0.1157295	-643.8494940	-643.9220088	-0.0432147	-642.8841172	-643.0810732	-643.2024311	-643.2456458
15	1m5dhmU51	-643.9650890	0.1156049	-643.8494840	-643.9229943	-0.0420947	-642.8854152	-643.0824658	-643.2038628	-643.2459575
16	1m5dhmU55	-643.9657750	0.1163190	-643.8494560	-643.9179937	-0.0477813	-642.8808692	-643.0776657	-643.1989158	-643.2466971
17	1m5dhmU56	-643.9659168	0.1167587	-643.8491580	-643.9180180	-0.0478987	-642.8818456	-643.0783491	-643.1994776	-643.2473763
18	1m5dhmU49	-643.9645284	0.1155683	-643.8489600	-643.9233056	-0.0412228	-642.8859720	-643.0830055	-643.2044702	-643.2456930
19	1m5dhmU54	-643.9655559	0.1166868	-643.8488690	-643.9192796	-0.0462763	-642.8827512	-643.0792669	-643.2004135	-643.2466898
20	1m5dhmU48	-643.9644885	0.1156324	-643.8488560	-643.9118024	-0.0526861	-642.8757318	-643.0726101	-643.1938595	-643.2465456
1	1m5caU27	-642.7933141	0.0933941	-642.6999200	-642.7622122	-0.0311019	-641.7275333	-641.9215621	-642.0412248	-642.0723267
2	1m5caU26	-642.7873303	0.0932263	-642.6941040	-642.7504977	-0.0368326	-641.7155909	-641.9096273	-642.0293132	-642.0661458
3	1m5caU25	-642.7871432	0.0936401	-642.6935030	-642.7539658	-0.0331773	-641.7186819	-641.9126650	-642.0323387	-642.0655160
4	1m5caU01	-642.7818993	0.0937402	-642.6881590	-642.7350866	-0.0468127	-641.7023026	-641.8963049	-642.0158487	-642.0626614
5	1m5caU24	-642.7777166	0.0927606	-642.6849560	-642.7406403	-0.0370763	-641.7073564	-641.9015628	-642.0211943	-642.0582706
6	1m5caU23	-642.7740452	0.0928802	-642.6811650	-642.7355168	-0.0385284	-641.7013407	-641.8957853	-642.0155651	-642.0540935
7	1m5caU22	-642.7728275	0.0926494	-642.6801780	-642.7326098	-0.0402176	-641.7004468	-641.8946290	-642.0142795	-642.0544971
8	1m5caU21	-642.7727389	0.0929848	-642.6797540	-642.7356633	-0.0370756	-641.7019723	-641.8964357	-642.0162196	-642.0532952
9	1m5caU20	-642.7723958	0.0929308	-642.6794650	-642.7249235	-0.0474723	-641.6933371	-641.8877984	-642.0074334	-642.0549057
10	1m5caU19	-642.7718997	0.0931736	-642.6787260	-642.7343986	-0.0375010	-641.7009320	-641.8950516	-642.0146685	-642.0521695
11	1m5caU17	-642.7673011	0.0913980	-642.6759030	-642.7137939	-0.0535072	-641.6785123	-641.8734355	-641.9933975	-642.0469047
12	1m5caU18	-642.7679820	0.0927239	-642.6752580	-642.7152441	-0.0527379	-641.6849471	-641.8792846	-641.9989218	-642.0516597
13	1m5caU16	-642.7664008	0.0934198	-642.6729810	-642.7187390	-0.0476619	-641.6872347	-641.8814153	-642.0009393	-642.0486012
14	1m5caU13	-642.7628135	0.0914244	-642.6713890	-642.6973616	-0.0654518	-641.6637630	-641.8588856	-641.9788711	-642.0443229
15	1m5caU15	-642.7637739	0.0928918	-642.6708820	-642.7150994	-0.0486745	-641.6830016	-641.8771413	-641.9967675	-642.0454420
16	1m5caU14	-642.7634531	0.0931801	-642.6702730	-642.7190263	-0.0444268	-641.6865280	-641.8806341	-642.0002561	-642.0446829
17	1m5caU12	-642.7630213	0.0939103	-642.6691110	-642.7151756	-0.0478458	-641.6827593	-641.8772121	-641.9968877	-642.0447335
18	1m5caU08	-642.7601579	0.0939398	-642.6662180	-642.6733425	-0.0868153	-641.6327972	-641.8294634	-641.9501866	-642.0370019
19	1m5caU09	-642.7589780	0.0931779	-642.6658000	-642.7009444	-0.0580335	-641.6707752	-641.8649461	-641.9844801	-642.0425136
20	1m5caU11	-642.7587677	0.0930116	-642.6657560	-642.6967611	-0.0620066	-641.6667711	-641.8610725	-641.9806883	-642.0426949
1	rc_C01	-394.7821290	0.0668469	-394.7152820	-394.6554173	-0.1267117	-393.9816302	-394.0954174	-394.1656043	-394.2923160
2	rc_C02	-394.7746201	0.0668320	-394.7077880	-394.6539377	-0.1206823	-393.9773494	-394.0910553	-394.1611655	-394.2818478
3	rc_C04	-394.7689079	0.0660648	-394.7028430	-394.6565268	-0.1123811	-393.9827517	-394.0965694	-394.1667241	-394.2791052
4	rc_C03	-394.7693593	0.0667493	-394.7026100	-394.6559977	-0.1133616	-393.9824755	-394.0962107	-394.1663383	-394.2796999
5	rc_C05	-394.7512459	0.0667118	-394.6845340	-394.6052403	-0.1460055	-393.9362897	-394.0491055	-394.1188046	-394.2648101
1	rc_5mC01	-434.1145867	0.0925767	-434.0220100	-433.9903660	-0.1242207	-433.2343538	-433.3601380	-433.4378341	-433.5620548
2	rc_5mC02	-434.1019923	0.0925613	-434.0094310	-433.9922461	-0.1097462	-433.2361761	-433.3618452	-433.4394452	-433.5491914
3	rc_5mC03	-434.1018030	0.0931429	-434.0086600	-433.9929055	-0.1088975	-433.2366512	-433.3623906	-433.4400159	-433.5489134
4	rc_5mC04	-434.0841946	0.0926146	-433.9915800	-433.9380003	-0.1461943	-433.1857993	-433.3105471	-433.3877178	-433.5339121
1	rc_5hmC02	-509.3348747	0.0963136	-509.2385610	-509.2106642	-0.1242104	-508.3575499	-508.5073726	-508.5998177	-508.7240281
2	rc_5hmC01	-509.3354197	0.0974617	-509.2379580	-509.2078754	-0.1275443	-508.3555265	-508.5051440	-508.5975091	-508.7250534
3	rc_5hmC03	-509.3340561	0.0976611	-509.2363950	-509.1982496	-0.1358065	-508.3468133	-508.4966140	-508.5890299	-508.7248364
4	rc_5hmC04	-509.3332813	0.0970952	-509.2361860	-509.2016055	-0.1316758	-508.3491852	-508.4993567	-508.5919465	-508.7236223
5	rc_5hmC05	-509.3316967	0.0981557	-509.2335410	-509.1933127	-0.1383840	-508.3412831	-508.4913390	-508.5838189	-508.7222029
6	rc_5hmC08	-509.3281735	0.0964264	-509.2317470	-509.2056265	-0.1225470	-508.3485958	-508.4988002	-508.5914277	-508.7139747
7	rc_5hmC09	-509.3279788	0.0972177	-509.2307610	-509.2061100	-0.1218688	-508.3502486	-508.5006767	-508.5934280	-508.7152968
8	rc_5hmC06	-509.3284919	0.0978179	-509.2306740	-509.2007682	-0.1277237	-508.3461995	-508.4962201	-508.5887602	-508.7164839
9	rc_5hmC07	-509.3282469	0.0976178	-509.2306290	-509.2032219	-0.1250250	-508.3474798	-508.4973506	-508.5898326	-508.7148576
10	rc_5hmC10	-509.3246989	0.0972648	-509.2274340	-509.1953856	-0.1293133	-508.3396124	-508.4898578	-508.5824356	-508.7117489
11	rc_5hmC12	-509.3231646	0.0961275	-509.2270370	-509.2133101	-0.1098545	-508.3598897	-508.5098083	-508.6022904	-508.7121449

12	rc_5hmC14	-509.3230012	0.0969171	-509.2260840	-509.2136838	-0.1093174	-508.3601284	-508.5101126	-508.6026065	-508.7119239
13	rc_5hmC11	-509.3232265	0.0973145	-509.2259120	-509.2090947	-0.1141319	-508.3569794	-508.5065887	-508.5989323	-508.7130642
14	rc_5hmC13	-509.3230182	0.0978452	-509.2251730	-509.2094645	-0.1135537	-508.3572305	-508.5069000	-508.5992551	-508.7128088
15	rc_5hmC15	-509.3218544	0.0977963	-509.2240580	-509.2004567	-0.1213977	-508.3489231	-508.4986663	-508.5910191	-508.7124168
16	rc_5hmC17	-509.3211640	0.0973020	-509.2238620	-509.2041947	-0.1169694	-508.3515351	-508.5016467	-508.5941813	-508.7111507
17	rc_5hmC16	-509.3214704	0.0976464	-509.2238240	-509.2012153	-0.1202551	-508.3493990	-508.4991962	-508.5915709	-508.7118260
18	rc_5hmC18	-509.3207259	0.0969239	-509.2238020	-509.2053096	-0.1154163	-508.3522858	-508.5025087	-508.5951037	-508.7105200
19	rc_5hmC19	-509.3190435	0.0972054	-509.2218380	-509.1959174	-0.1231261	-508.3434788	-508.4934579	-508.5858720	-508.7089981
20	rc_5hmC20	-509.3186592	0.0972992	-509.2213600	-509.1968535	-0.1218058	-508.3440098	-508.4940478	-508.5864803	-508.7082861
1	rc_5fC01	-508.1073697	0.0734177	-508.0339520	-507.9828144	-0.1245553	-507.1364140	-507.2833945	-507.3740091	-507.4985644
2	rc_5fC02	-508.1026052	0.0737152	-508.0288900	-507.9685695	-0.1340358	-507.1225286	-507.2692820	-507.3597607	-507.4937965
3	rc_5fC03	-508.0975606	0.0732836	-508.0242770	-507.9786905	-0.1188701	-507.1286320	-507.2757545	-507.3664680	-507.4853381
4	rc_5fC04	-508.0947365	0.0734784	-508.0212580	-507.9824708	-0.1122657	-507.1364186	-507.2833058	-507.3738543	-507.4861200
5	rc_5fC06	-508.0942331	0.0735050	-508.0207280	-507.9709360	-0.1232971	-507.1216091	-507.2687281	-507.3593964	-507.4826935
6	rc_5fC05	-508.0944568	0.0740038	-508.0204530	-507.9826747	-0.1117821	-507.1365449	-507.2834618	-507.3739997	-507.4857818
7	rc_5fC08	-508.0898983	0.0735563	-508.0163420	-507.9715634	-0.1183349	-507.1251043	-507.2718818	-507.3623299	-507.4806648
8	rc_5fC07	-508.0903968	0.0741137	-508.0162830	-507.9705101	-0.1198867	-507.1242989	-507.2710046	-507.3614309	-507.4813176
9	rc_5fC09	-508.0770551	0.0728741	-508.0041810	-507.9403828	-0.1366723	-507.0979192	-507.2439141	-507.3340090	-507.4706813
10	rc_5fC10	-508.0730471	0.0735311	-507.9995160	-507.9187699	-0.1542773	-507.0763499	-507.2221729	-507.3121794	-507.4664567
11	rc_5fC11	-508.0718088	0.0727428	-507.9990660	-507.9437310	-0.1280778	-507.0861391	-507.2333379	-507.3237991	-507.4518769
12	rc_5fC12	-508.0665627	0.0743086	-507.9922540	-507.9339379	-0.1326248	-507.0769458	-507.2240605	-507.3144816	-507.4471064
1	rc_5dhmC03	-584.5686548	0.1006917	-584.4679630	-584.4392327	-0.1294221	-583.4914923	-583.6649672	-583.7720203	-583.9014424
2	rc_5dhmC06	-584.5680234	0.1002354	-584.4677880	-584.4366797	-0.1313437	-583.4894729	-583.6630827	-583.7701661	-583.9015098
3	rc_5dhmC04	-584.5682502	0.1005921	-584.4676580	-584.4400497	-0.1282005	-583.4913713	-583.6651599	-583.7723052	-583.9005057
4	rc_5dhmC02	-584.5689459	0.1014618	-584.4674840	-584.4377765	-0.1311694	-583.4902701	-583.6636607	-583.7706684	-583.9018378
5	rc_5dhmC01	-584.5694948	0.1023388	-584.4671560	-584.4374046	-0.1320903	-583.4885775	-583.6623335	-583.7694793	-583.9015696
6	rc_5dhmC05	-584.5681434	0.1017074	-584.4664360	-584.4292384	-0.1389050	-583.4824697	-583.6558224	-583.7627945	-583.9016995
7	rc_5dhmC08	-584.5675021	0.1011670	-584.4663350	-584.4369275	-0.1305746	-583.4889667	-583.6626836	-583.7697999	-583.9003745
8	rc_5dhmC07	-584.5679474	0.1017354	-584.4662120	-584.4308036	-0.1371438	-583.4825413	-583.6562749	-583.7633953	-583.9005391
9	rc_5dhmC12	-584.5613940	0.1005160	-584.4608780	-584.4391151	-0.1222789	-583.4878747	-583.6616085	-583.7688073	-583.8910862
10	rc_5dhmC13	-584.5606782	0.1000841	-584.4605940	-584.4344410	-0.1262372	-583.4824091	-583.6563980	-583.7636280	-583.8898652
11	rc_5dhmC16	-584.5606397	0.1002046	-584.4604350	-584.4336743	-0.1269654	-583.4814015	-583.6554113	-583.7626529	-583.8896183
12	rc_5dhmC15	-584.5606471	0.1002270	-584.4604200	-584.4358356	-0.1248114	-583.4853302	-583.6591413	-583.7663316	-583.8911430
13	rc_5dhmC18	-584.5603925	0.1003425	-584.4600500	-584.4346581	-0.1257344	-583.4828094	-583.6568524	-583.7641294	-583.8898638
14	rc_5dhmC11	-584.5614334	0.1014024	-584.4600310	-584.4355988	-0.1258346	-583.4847961	-583.6584125	-583.7655379	-583.8913725
15	rc_5dhmC10	-584.5618784	0.1018504	-584.4600280	-584.4355504	-0.1263280	-583.4829816	-583.6569792	-583.7642440	-583.8905720
16	rc_5dhmC14	-584.5606516	0.1012806	-584.4593710	-584.4273658	-0.1332858	-583.4774617	-583.6509609	-583.7580012	-583.8912870
17	rc_5dhmC19	-584.5597049	0.1005739	-584.4591310	-584.4318355	-0.1278695	-583.4801293	-583.6540464	-583.7612510	-583.8891205
18	rc_5dhmC17	-584.5604142	0.1015231	-584.4588910	-584.4290316	-0.1313826	-583.4768716	-583.6508921	-583.7581628	-583.8895454
19	rc_5dhmC09	-584.5618997	0.1033477	-584.4585520	-584.4085902	-0.1533095	-583.4606863	-583.6345933	-583.7417140	-583.8950235
20	rc_5dhmC26	-584.5560105	0.1000674	-584.4559430	-584.4382474	-0.1177631	-583.4911406	-583.6647507	-583.7718216	-583.8895847
1	rc_5caC01	-583.3617865	0.0772845	-583.2845020	-583.2323349	-0.1294517	-582.2819735	-582.4525693	-582.5576390	-582.6870907
2	rc_5caC02	-583.3568764	0.0770244	-583.2798520	-583.2407140	-0.1161624	-582.2928569	-582.4640668	-582.5696897	-582.6858521
3	rc_5caC03	-583.3564558	0.0776788	-583.2787770	-583.2364066	-0.1200492	-582.2895311	-582.4608185	-582.5664161	-582.6864653
4	rc_5caC05	-583.3512619	0.0775888	-583.2736730	-583.2168703	-0.1343915	-582.2698803	-582.4415154	-582.5471500	-582.6815415
5	rc_5caC04	-583.3517638	0.0784547	-583.2733090	-583.2174742	-0.1342896	-582.2694678	-582.4410487	-582.5465915	-582.6808811
6	rc_5caC06	-583.3491276	0.0771526	-583.2719750	-583.2296319	-0.1194958	-582.2821305	-582.4527675	-582.5578422	-582.6773380
7	rc_5caC07	-583.3480481	0.0775870	-583.2704610	-583.2251354	-0.1229127	-582.2576360	-582.4283832	-582.5336162	-582.6565289

8	rc_5caC08	-583.3343902	0.0772502	-583.2571400	-583.1897837	-0.1446065	-582.2393610	-582.4094597	-582.5141839	-582.6587904
1	rc_1mC01	-434.0932452	0.0937581	-433.9994870	-433.9798871	-0.1133581	-433.2218429	-433.3472987	-433.4246891	-433.5380472
1	rc_1m5mC01	-473.4257287	0.1192256	-473.3065030	-473.3156619	-0.1100667	-472.4755805	-472.6130069	-472.6979060	-472.8079727
1	rc_1m5hmC03	-548.6454897	0.1221897	-548.5233000	-548.5310705	-0.1144192	-547.5943536	-547.7561835	-547.8560354	-547.9704547
2	rc_1m5hmC01	-548.6461866	0.1229056	-548.5232810	-548.5259278	-0.1202588	-547.5903987	-547.7518590	-547.8515146	-547.9717734
3	rc_1m5hmC02	-548.6458446	0.1233845	-548.5224600	-548.5290306	-0.1168140	-547.5923831	-547.7536583	-547.8531951	-547.9700091
4	rc_1m5hmC04	-548.6454057	0.1232127	-548.5221930	-548.5310338	-0.1143719	-547.5934194	-547.7549676	-547.8546078	-547.9689797
1	rc_1m5fC01	-547.4166824	0.0993303	-547.3173520	-547.3037457	-0.1129367	-546.3725651	-546.5311146	-546.6288551	-546.7417918
2	rc_1m5fC02	-547.4135959	0.0998428	-547.3137530	-547.2975454	-0.1160505	-546.3670096	-546.5255723	-546.6233388	-546.7393893
1	rc_1m5dhmC07	-623.8786624	0.1261184	-623.7525440	-623.7592235	-0.1194389	-622.7262905	-622.9117295	-623.0260590	-623.1454979
2	rc_1m5dhmC03	-623.8797920	0.1273279	-623.7524640	-623.7642204	-0.1155716	-622.7319217	-622.9171009	-623.0314015	-623.1469731
3	rc_1m5dhmC01	-623.8798714	0.1276843	-623.7521870	-623.7607942	-0.1190772	-622.7275155	-622.9129302	-623.0272860	-623.1463632
4	rc_1m5dhmC05	-623.8790452	0.1269091	-623.7521360	-623.7610123	-0.1180329	-622.7294576	-622.9147272	-623.0290252	-623.1470581
5	rc_1m5dhmC02	-623.8798441	0.1278310	-623.7520130	-623.7610829	-0.1187612	-622.7292532	-622.9143229	-623.0285440	-623.1473052
6	rc_1m5dhmC06	-623.8788889	0.1272748	-623.7516140	-623.7599256	-0.1189633	-622.7274087	-622.9128485	-623.0271894	-623.1461527
7	rc_1m5dhmC09	-623.8778398	0.1266697	-623.7511700	-623.7575410	-0.1202988	-622.7250107	-622.9103549	-623.0246286	-623.1449274
8	rc_1m5dhmC08	-623.8783452	0.1274912	-623.7508540	-623.7542176	-0.1241276	-622.7213197	-622.9067211	-623.0210550	-623.1451826
9	rc_1m5dhmC04	-623.8790532	0.1284671	-623.7505860	-623.7524635	-0.1265897	-622.7213127	-622.9063214	-623.0204961	-623.1470858
1	rc_1m5caC01	-622.6780305	0.1037864	-622.5742440	-622.5545475	-0.1234830	-621.5211823	-621.7033040	-621.8155828	-621.9390658
2	rc_1m5caC02	-622.6763822	0.1035492	-622.5728330	-622.5656858	-0.1106964	-621.5379511	-621.7207209	-621.8334314	-621.9441278
3	rc_1m5caC03	-622.6705708	0.1042508	-622.5663200	-622.5436729	-0.1268979	-621.5160130	-621.6990259	-621.8116992	-621.9385971
4	rc_1m5caC04	-622.6702332	0.1039842	-622.5662490	-622.5435178	-0.1267154	-621.5145540	-621.6975651	-621.8102182	-621.9369336
5	rc_1m5caC05	-622.6644361	0.1039291	-622.5605070	-622.5514394	-0.1129967	-621.5215418	-621.7034798	-621.8156389	-621.9286356
6	rc_1m5caC06	-622.6593189	0.1040688	-622.5552500	-622.5367572	-0.1225616	-621.5086536	-621.6907370	-621.8028954	-621.9254570
7	rc_1m5caC07	-622.6513307	0.1039227	-622.5474080	-622.5132269	-0.1381038	-621.4798154	-621.6614725	-621.7734203	-621.9115241
1	rc_U01	-414.6366283	0.0542853	-414.5823430	-414.5136351	-0.1229933	-413.8304884	-413.9505348	-414.0244272	-414.1474205
2	rc_U04	-414.6236957	0.0542157	-414.5694800	-414.5113735	-0.1123223	-413.8300315	-413.9497693	-414.0235208	-414.1358431
3	rc_U02	-414.6229257	0.0540776	-414.5688480	-414.5038996	-0.1190261	-413.8225362	-413.9425651	-414.0164448	-414.1354709
4	rc_U03	-414.6208267	0.0530976	-414.5677290	-414.4926336	-0.1281931	-413.8126767	-413.9328582	-414.0067912	-414.1349843
5	rc_U07	-414.6219442	0.0542611	-414.5676830	-414.4967341	-0.1252101	-413.8172145	-413.9370633	-414.0108268	-414.1360369
6	rc_U34	-414.6214636	0.0539546	-414.5675090	-414.4988544	-0.1226093	-413.8187228	-413.9385447	-414.0122797	-414.1348890
7	rc_U09	-414.6210897	0.0540556	-414.5670340	-414.4937907	-0.1272990	-413.8144800	-413.9343372	-414.0080917	-414.1353907
8	rc_U05	-414.6144998	0.0539138	-414.5605860	-414.4923983	-0.1221015	-413.8107401	-413.9305611	-414.0043734	-414.1264749
9	rc_U08	-414.6098533	0.0536373	-414.5562160	-414.4741810	-0.1356723	-413.7930796	-413.9130442	-413.9868591	-414.1225314
10	rc_U10	-414.6084403	0.0533292	-414.5551110	-414.4973976	-0.1110427	-413.8171355	-413.9369796	-414.0106957	-414.1217384
11	rc_U11	-414.6086820	0.0541930	-414.5544890	-414.4963373	-0.1123447	-413.8164540	-413.9362577	-414.0099576	-414.1223023
12	rc_U26	-414.6067004	0.0537354	-414.5529650	-414.4859204	-0.1207801	-413.8066843	-413.9266977	-414.0004168	-414.1211969
13	rc_U13	-414.6071165	0.0545494	-414.5525670	-414.4867518	-0.1203646	-413.8077118	-413.9276538	-414.0013516	-414.1217162
14	rc_U14	-414.5694815	0.0534564	-414.5160250	-414.4332462	-0.1362353	-413.7544047	-413.8740815	-413.9478016	-414.0840369
15	rc_U15	-414.5387858	0.0411267	-414.4976590	-414.4295268	-0.1092590	-413.7506082	-413.8698083	-413.9427783	-414.0520373
1	rc_5mU01	-453.9736640	0.0797930	-453.8938710	-453.8567892	-0.1168748	-453.0902060	-453.2222362	-453.3036754	-453.4205502
2	rc_5mU04	-453.9593482	0.0797862	-453.8795620	-453.8521125	-0.1072357	-453.0874352	-453.2191376	-453.3004151	-453.4076508
3	rc_5mU02	-453.9588596	0.0797416	-453.8791180	-453.8439214	-0.1149382	-453.0787085	-453.2106994	-453.2920902	-453.4070284
4	rc_5mU06	-453.9565708	0.0788787	-453.8776920	-453.8395031	-0.1170677	-453.0758589	-453.2076474	-453.2889081	-453.4059758
5	rc_5mU07	-453.9558795	0.0792925	-453.8765870	-453.8334085	-0.1224710	-453.0713040	-453.2031561	-453.2844350	-453.4069060
6	rc_5mU10	-453.9555261	0.0797400	-453.8757860	-453.8317345	-0.1237915	-453.0693760	-453.2012047	-453.2824775	-453.4062690
7	rc_5mU03	-453.9557672	0.0802722	-453.8754950	-453.8294427	-0.1263245	-453.0662502	-453.1984615	-453.2799008	-453.4062253
8	rc_5mU05	-453.9503383	0.0793252	-453.8710130	-453.8318834	-0.1184548	-453.0674254	-453.1993763	-453.2807558	-453.3992106

9	rc_5mU08	-453.9458487	0.0795536	-453.8662950	-453.8128052	-0.1330435	-453.0493514	-453.1814587	-453.2628264	-453.3958699
10	rc_5mU09	-453.9452320	0.0802700	-453.8649620	-453.8391912	-0.1060409	-453.0752555	-453.2069979	-453.2882002	-453.3942411
11	rc_5mU13	-453.9425810	0.0792070	-453.8633740	-453.8261729	-0.1164081	-453.0638909	-453.1958152	-453.2770326	-453.3934407
12	rc_5mU12	-453.9426128	0.0799828	-453.8626300	-453.8255944	-0.1170184	-453.0632331	-453.1951813	-453.2763938	-453.3934122
13	rc_5mU14	-453.9029559	0.0783919	-453.8245640	-453.7709880	-0.1319679	-453.0089469	-453.1405402	-453.2217457	-453.3537136
14	rc_5mU15	-414.5391660	0.0403999	-414.4987660	-414.4355250	-0.1036410	-413.7568031	-413.8752442	-413.9478473	-414.0514883
1	rc_5hmU01	-529.1924436	0.0835396	-529.1089040	-529.0679016	-0.1245420	-528.1989294	-528.3553171	-528.4516035	-528.5761455
2	rc_5hmU04	-529.1780224	0.0837473	-529.0942750	-529.0618413	-0.1161811	-528.1959605	-528.3520683	-528.4482224	-528.5644035
3	rc_5hmU06	-529.1753650	0.0830800	-529.0922850	-529.0486700	-0.1266950	-528.1843335	-528.3405167	-528.4366517	-528.5633467
4	rc_5hmU02	-529.1757859	0.0839218	-529.0918640	-529.0529644	-0.1228215	-528.1913337	-528.3474990	-528.4436488	-528.5664703
5	rc_5hmU09	-529.1731969	0.0845288	-529.0886680	-529.0390224	-0.1341745	-528.1800176	-528.3360991	-528.4321676	-528.5663421
6	rc_5hmU05	-529.1714567	0.0828856	-529.0885710	-529.0459976	-0.1254591	-528.1768718	-528.3333078	-528.4295713	-528.5550304
7	rc_5hmU08	-529.1726217	0.0863566	-529.0862650	-529.0357615	-0.1368602	-528.1777423	-528.3338538	-528.4299200	-528.5667802
8	rc_5hmU03	-529.1717444	0.0858243	-529.0859200	-529.0340441	-0.1377003	-528.1743077	-528.3306957	-528.4268789	-528.5645792
9	rc_5hmU07	-529.1670092	0.0821511	-529.0848580	-529.0273877	-0.1396215	-528.1588182	-528.3154485	-528.4117182	-528.5513397
10	rc_5hmU11	-529.1622895	0.0852235	-529.0770660	-529.0464628	-0.1158267	-528.1844297	-528.3403613	-528.4363161	-528.5521428
11	rc_5hmU10	-529.1621777	0.0851897	-529.0769880	-529.0482936	-0.1138841	-528.1856251	-528.3415514	-528.4374999	-528.5513840
12	rc_5hmU13	-529.1588285	0.0855644	-529.0732640	-529.0299829	-0.1288456	-528.1705283	-528.3266208	-528.4225774	-528.5514230
13	rc_5hmU12	-529.1585793	0.0865592	-529.0720200	-529.0296186	-0.1289607	-528.1697460	-528.3258532	-528.4218011	-528.5507618
14	rc_5hmU14	-529.1229983	0.0825372	-529.0404610	-528.9814741	-0.1415242	-528.1181893	-528.2743594	-528.3705231	-528.5120473
1	rc_5fU01	-527.9656808	0.0625367	-527.9031440	-527.8482714	-0.1174093	-526.9769066	-527.1298893	-527.2239844	-527.3413937
2	rc_5fU02	-527.9593020	0.0609879	-527.8983140	-527.8361929	-0.1231091	-526.9807992	-527.1337882	-527.2280181	-527.3511272
3	rc_5fU03	-527.9464882	0.0609272	-527.8855610	-527.8194973	-0.1269909	-526.9661087	-527.1192120	-527.2134190	-527.3404099
4	rc_5fU09	-527.9467195	0.0619404	-527.8847790	-527.8164030	-0.1303164	-526.9511383	-527.1039728	-527.1981161	-527.3284325
5	rc_5fU05	-527.9447110	0.0601609	-527.8845500	-527.8239272	-0.1207838	-526.9693445	-527.1224464	-527.2167106	-527.3374944
6	rc_5fU04	-527.9427795	0.0607305	-527.8820490	-527.8156626	-0.1271169	-526.9617816	-527.1146444	-527.2087295	-527.3358464
7	rc_5fU06	-527.9399046	0.0609866	-527.8789180	-527.8085855	-0.1313191	-526.9561523	-527.1093869	-527.2036583	-527.3349774
8	rc_5fU08	-527.9373877	0.0603647	-527.8770230	-527.8171333	-0.1202544	-526.9589983	-527.1119870	-527.2062134	-527.3264678
9	rc_5fU07	-527.9338823	0.0617902	-527.8720920	-527.8188997	-0.1149826	-526.9472919	-527.0999298	-527.1939491	-527.3089317
10	rc_5fU10	-527.9311399	0.0611228	-527.8700170	-527.7974192	-0.1337207	-526.9404481	-527.0930193	-527.1868744	-527.3205951
11	rc_5fU11	-527.9296948	0.0603427	-527.8693520	-527.8017676	-0.1279272	-526.9431875	-527.0958192	-527.1898093	-527.3177365
12	rc_5fU12	-527.9266626	0.0597176	-527.8669450	-527.7911763	-0.1354864	-526.9334331	-527.0860146	-527.1799069	-527.3153933
1	rc_5dhmU09	-604.4258634	0.0881474	-604.3377160	-604.3028281	-0.1230354	-603.3419679	-603.5221667	-603.6331308	-603.7561662
2	rc_5dhmU10	-604.4255564	0.0878704	-604.3376860	-604.3021374	-0.1234190	-603.3412736	-603.5215025	-603.6324736	-603.7558926
3	rc_5dhmU03	-604.4267426	0.0891035	-604.3376390	-604.2992901	-0.1274524	-603.3388700	-603.5189021	-603.6297956	-603.7572480
4	rc_5dhmU01	-604.4269654	0.0900423	-604.3369230	-604.2966584	-0.1303069	-603.3346392	-603.5146531	-603.6254580	-603.7557649
5	rc_5dhmU02	-604.4259911	0.0894010	-604.3365900	-604.2978079	-0.1281832	-603.3365550	-603.5165689	-603.6273894	-603.7555726
6	rc_5dhmU13	-604.4229461	0.0870790	-604.3358670	-604.2871075	-0.1358386	-603.3279225	-603.5079703	-603.6187560	-603.7545946
7	rc_5dhmU12	-604.4230040	0.0879219	-604.3350820	-604.2877891	-0.1352149	-603.3289270	-603.5090072	-603.6198088	-603.7550237
8	rc_5dhmU07	-604.4253786	0.0906045	-604.3347740	-604.2834616	-0.1419170	-603.3240896	-603.5041101	-603.6149323	-603.7568493
9	rc_5dhmU25	-604.4114174	0.0881894	-604.3232280	-604.2791475	-0.1322699	-603.3251944	-603.5048938	-603.6155498	-603.7478197
10	rc_5dhmU33	-604.4106329	0.0874649	-604.3231680	-604.2871587	-0.1234742	-603.3284831	-603.5087642	-603.6197434	-603.7432176
11	rc_5dhmU15	-604.4118961	0.0887600	-604.3231360	-604.2796450	-0.1322511	-603.3252484	-603.5048969	-603.6155774	-603.7478285
12	rc_5dhmU34	-604.4118125	0.0887055	-604.3231070	-604.2859788	-0.1258337	-603.3280938	-603.5082029	-603.6190989	-603.7449326
13	rc_5dhmU16	-604.4118775	0.0888145	-604.3230630	-604.2795896	-0.1322879	-603.3237475	-603.5036337	-603.6143776	-603.7466655
14	rc_5dhmU17	-604.4114890	0.0884659	-604.3230230	-604.2819493	-0.1295397	-603.3273173	-603.5069090	-603.6175602	-603.7470999
15	rc_5dhmU32	-604.4102964	0.0873664	-604.3229300	-604.2867039	-0.1235925	-603.3279040	-603.5082148	-603.6191970	-603.7427895
16	rc_5dhmU23	-604.4102785	0.0878074	-604.3224710	-604.2773506	-0.1329279	-603.3210597	-603.5008230	-603.6114364	-603.7443643

17	rc_5dhmU30	-604.4107961	0.0885331	-604.3222630	-604.2833487	-0.1274474	-603.3248939	-603.5049913	-603.6158263	-603.7432737
18	rc_5dhmU18	-604.4115928	0.0893758	-604.3222170	-604.2794036	-0.1321892	-603.3205549	-603.5005851	-603.6113971	-603.7435863
19	rc_5dhmU26	-604.4112772	0.0890931	-604.3221840	-604.2751227	-0.1361545	-603.3196626	-603.4996816	-603.6104962	-603.7466507
20	rc_5dhmU31	-604.4084179	0.0869499	-604.3214680	-604.2770881	-0.1313298	-603.3204267	-603.5006557	-603.6114928	-603.7428226
1	rc_5caU03	-603.2185219	0.0652648	-603.1532570	-603.0941240	-0.1243979	-602.1388629	-602.3160461	-602.4250660	-602.5494639
2	rc_5caU02	-603.2171435	0.0648945	-603.1522490	-603.0897550	-0.1273885	-602.1350576	-602.3122090	-602.4212173	-602.5486058
3	rc_5caU04	-603.2143253	0.0653193	-603.1490060	-603.0765922	-0.1377331	-602.1203590	-602.2976110	-602.4065045	-602.5442376
4	rc_5caU21	-603.2067313	0.0655383	-603.1411930	-603.0717945	-0.1349369	-602.0928454	-602.2692874	-602.3780067	-602.5129436
5	rc_5caU06	-603.2045551	0.0654210	-603.1391340	-603.0786609	-0.1258942	-602.1281934	-602.3055577	-602.4147039	-602.5405981
6	rc_5caU10	-603.2032835	0.0647035	-603.1385800	-603.0790494	-0.1242341	-602.1267340	-602.3039550	-602.4129760	-602.5372101
7	rc_5caU08	-603.2023562	0.0651732	-603.1371830	-603.0718803	-0.1304759	-602.1216891	-602.2993147	-602.4084943	-602.5389702
8	rc_5caU24	-603.2020481	0.0649551	-603.1370930	-603.0592896	-0.1427585	-602.0817038	-602.2580822	-602.3667672	-602.5095257
9	rc_5caU13	-603.2001610	0.0645999	-603.1355610	-603.0640747	-0.1360863	-602.1103720	-602.2878376	-602.3968366	-602.5329229
10	rc_5caU23	-603.1999958	0.0654438	-603.1345520	-603.0783567	-0.1216391	-602.1018774	-602.2783009	-602.3870261	-602.5086652
11	rc_5caU09	-603.1992450	0.0647490	-603.1344960	-603.0597739	-0.1394711	-602.1105096	-602.2878075	-602.3967899	-602.5362610
12	rc_5caU12	-603.1988084	0.0653743	-603.1334340	-603.0789131	-0.1198953	-602.1075023	-602.2841038	-602.3928220	-602.5127173
13	rc_5caU07	-603.1982170	0.0655000	-603.1327170	-603.0668088	-0.1314082	-602.1157723	-602.2933185	-602.4024513	-602.5338595
14	rc_5caU15	-603.1971831	0.0648191	-603.1323640	-603.0711058	-0.1260773	-602.1165953	-602.2938128	-602.4028511	-602.5289284
15	rc_5caU17	-603.1979627	0.0662027	-603.1317600	-603.0550392	-0.1429235	-602.1034723	-602.2811631	-602.3902887	-602.5332122
16	rc_5caU25	-603.1969502	0.0657331	-603.1312170	-603.0646315	-0.1323186	-599.8140279	-599.8999754	0.0000000	-0.1323186
17	rc_5caU14	-603.1955659	0.0643528	-603.1312130	-603.0663337	-0.1292321	-602.1134256	-602.2905901	-602.3995920	-602.5288241
18	rc_5caU20	-603.1952077	0.0648327	-603.1303750	-603.0635725	-0.1316352	-602.1073757	-602.2848226	-602.3938737	-602.5255089
19	rc_5caU16	-603.1956403	0.0656803	-603.1299600	-603.0630081	-0.1326322	-602.0930915	-602.2698963	-602.3786392	-602.5112714
20	rc_5caU19	-603.1925389	0.0641469	-603.1283920	-603.0531506	-0.1393883	-602.0987089	-602.2760931	-602.3851354	-602.5245237
1	rc_1mU01	-453.9563015	0.0813415	-453.8749600	-453.8434593	-0.1128422	-453.0788660	-453.2103423	-453.2914081	-453.4042503
2	rc_1mU02	-453.9414665	0.0810324	-453.8604340	-453.8311435	-0.1103229	-453.0682744	-453.1997564	-453.2807925	-453.3911154
3	rc_1mU03	-453.9395726	0.0805985	-453.8589740	-453.8194148	-0.1201577	-453.0572367	-453.1888671	-453.2699261	-453.3900838
4	rc_1mU05	-453.9329807	0.0802596	-453.8527210	-453.8196692	-0.1133115	-453.0565899	-453.1878715	-453.2688868	-453.3821983
5	rc_1mU04	-453.9268945	0.0803184	-453.8465760	-453.8003489	-0.1265456	-453.0383829	-453.1697969	-453.2508008	-453.3773464
1	rc_1m5mU01	-493.2914110	0.1049030	-493.1865080	-493.1843294	-0.1070816	-492.3368114	-492.4802841	-492.5689073	-492.6759889
2	rc_1m5mU04	-493.2763001	0.1059370	-493.1703630	-493.1701628	-0.1061372	-492.3243727	-492.4677641	-492.5562877	-492.6624249
3	rc_1m5mU05	-493.2732554	0.1059573	-493.1672980	-493.1557403	-0.1175150	-492.3112623	-492.4548801	-492.5434432	-492.6609582
4	rc_1m5mU03	-493.2672668	0.1053837	-493.1618830	-493.1577824	-0.1094844	-492.3120753	-492.4553427	-492.5438648	-492.6533492
5	rc_1m5mU02	-493.2611213	0.1056963	-493.1554250	-493.1380302	-0.1230911	-492.2934710	-492.4369177	-492.5254439	-492.6485350
1	rc_1m5hmU01	-568.5100011	0.1110831	-568.3989180	-568.3937408	-0.1162603	-567.4468334	-567.6145477	-567.7179416	-567.8342019
2	rc_1m5hmU03	-568.4941500	0.1105530	-568.3835970	-568.3796159	-0.1145341	-567.4368840	-567.6044775	-567.7077743	-567.8223084
3	rc_1m5hmU04	-568.4878905	0.1099314	-568.3779590	-568.3699924	-0.1178981	-567.4211803	-567.5889347	-567.6923327	-567.8102308
4	rc_1m5hmU02	-568.4898844	0.1123234	-568.3775610	-568.3608964	-0.1289880	-567.4201031	-567.5878934	-567.6911976	-567.8201856
5	rc_1m5hmU05	-568.4819710	0.1102419	-568.3717290	-568.3507014	-0.1312696	-567.4026240	-567.5706521	-567.6741165	-567.8053861
1	rc_1m5fU11	-567.2792665	0.0861455	-567.1931210	-567.1661323	-0.1131342	-566.2294287	-566.3938274	-566.4952068	-566.6083410
2	rc_1m5fU12	-567.2764800	0.0872500	-567.1892300	-567.1539478	-0.1225323	-566.2178107	-566.3819757	-566.4832053	-566.6057376
3	rc_1m5fU10	-567.2651182	0.0869661	-567.1781520	-567.1461614	-0.1189568	-566.2108578	-566.3754600	-566.4768042	-566.5957610
4	rc_1m5fU08	-567.2639274	0.0862244	-567.1777030	-567.1519707	-0.1119567	-566.2167167	-566.3812521	-566.4826619	-566.5946186
5	rc_1m5fU06	-567.2645790	0.0877709	-567.1768080	-567.1556591	-0.1089199	-566.2094063	-566.3737062	-566.4749510	-566.5838709
6	rc_1m5fU09	-567.2616993	0.0864723	-567.1752270	-567.1433091	-0.1183902	-566.2082488	-566.3725783	-566.4738241	-566.5922143
7	rc_1m5fU07	-567.2587582	0.0864842	-567.1722740	-567.1361699	-0.1225884	-566.2017326	-566.3663916	-566.4677747	-566.5903631
8	rc_1m5fU04	-567.2594032	0.0887351	-567.1706680	-567.1378802	-0.1215230	-566.1931998	-566.3576869	-566.4589176	-566.5804406
9	rc_1m5fU05	-567.2564837	0.0863747	-567.1701090	-567.1401488	-0.1163349	-566.2054121	-566.3697874	-566.4711685	-566.5875034

10	rc_1m5fU03	-567.2464433	0.0870413	-567.1594020	-567.1216586	-0.1247847	-563.8207375	-563.9077181	0.0000000	-0.1247847
1	rc_1m5dhmU68	-643.7452280	0.1151250	-643.6301030	-643.6267520	-0.1184760	-642.5863302	-642.7777960	-642.8958719	-643.0143479
2	rc_1m5dhmU62	-643.7439604	0.1143454	-643.6296150	-643.6301097	-0.1138507	-642.5892930	-642.7809523	-642.8991141	-643.0129648
3	rc_1m5dhmU01	-643.7460133	0.1167453	-643.6292680	-643.6244284	-0.1215849	-642.5823088	-642.7737882	-642.8917871	-643.0133720
4	rc_1m5dhmU69	-643.7447493	0.1157942	-643.6289550	-643.6253696	-0.1193797	-642.5840324	-642.7754851	-642.8934789	-643.0128586
5	rc_1m5dhmU63	-643.7437076	0.1156976	-643.6280100	-643.6235478	-0.1201598	-642.5841917	-642.7756232	-642.8936685	-643.0138283
6	rc_1m5dhmU57	-643.7419541	0.1146111	-643.6273430	-643.6152657	-0.1266884	-642.5761225	-642.7676584	-642.8856527	-643.0123411
7	rc_1m5dhmU64	-643.7438992	0.1168101	-643.6270890	-643.6117348	-0.1321644	-642.5722675	-642.7637061	-642.8817074	-643.0138718
8	rc_1m5dhmU50	-643.7297337	0.1138186	-643.6159150	-643.6057508	-0.1239829	-642.5697524	-642.7608685	-642.8786461	-643.0026290
9	rc_1m5dhmU56	-643.7301945	0.1148485	-643.6153460	-643.6058941	-0.1243004	-642.5695170	-642.7605112	-642.8782587	-643.0025591
10	rc_1m5dhmU37	-643.7298280	0.1146100	-643.6152180	-643.6125746	-0.1172535	-642.5740417	-642.7655633	-642.8836127	-643.0008662
11	rc_1m5dhmU54	-643.7299601	0.1148390	-643.6151210	-643.6079838	-0.1219762	-642.5714088	-642.7624465	-642.8802331	-643.0022094
12	rc_1m5dhmU55	-643.7301277	0.1152146	-643.6149130	-643.6055075	-0.1246202	-642.5677535	-642.7591114	-642.8770188	-643.0016390
13	rc_1m5dhmU45	-643.7293423	0.1144972	-643.6148450	-643.5995804	-0.1297619	-642.5622413	-642.7536391	-642.8715441	-643.0013060
14	rc_1m5dhmU38	-643.7284535	0.1136734	-643.6147800	-643.6136170	-0.1148365	-642.5751187	-642.7667831	-642.8849076	-642.9997441
15	rc_1m5dhmU44	-643.7282359	0.1135149	-643.6147210	-643.6128923	-0.1153436	-642.5753579	-642.7670005	-642.8851069	-643.0004505
16	rc_1m5dhmU42	-643.7284491	0.1138340	-643.6146150	-643.6036053	-0.1248438	-642.5660229	-642.7573904	-642.8752610	-643.0001048
17	rc_1m5dhmU53	-643.7300376	0.1155216	-643.6145160	-643.6063208	-0.1237168	-642.5665515	-642.7580437	-642.8760301	-642.9997469
18	rc_1m5dhmU35	-643.7285937	0.1142726	-643.6143210	-643.6015495	-0.1270442	-642.5648471	-642.7561322	-642.8739621	-643.0010063
19	rc_1m5dhmU41	-643.7292248	0.1151077	-643.6141170	-643.6099495	-0.1192753	-642.5706764	-642.7622352	-642.8802431	-642.9995184
20	rc_1m5dhmU52	-643.7291282	0.1158692	-643.6132590	-643.5966989	-0.1324293	-642.5610860	-642.7520506	-642.8697729	-643.0022022
1	rc_1m5caU27	-642.5393329	0.0914289	-642.4479040	-642.4189638	-0.1203692	-641.3862127	-641.5751125	-641.6913827	-641.8117519
2	rc_1m5caU25	-642.5379191	0.0917611	-642.4461580	-642.4223277	-0.1155915	-641.3871546	-641.5758235	-641.6920572	-641.8076487
3	rc_1m5caU26	-642.5370450	0.0916620	-642.4453830	-642.4181116	-0.1189335	-641.3835575	-641.5721998	-641.6884441	-641.8073776
4	rc_1m5caU01	-642.5338564	0.0910523	-642.4428040	-642.4044123	-0.1294441	-641.3684575	-641.5572470	-641.6733833	-641.8028274
5	rc_1m5caU24	-642.5257282	0.0908611	-642.4348670	-642.4094921	-0.1162361	-641.3791130	-641.5678041	-641.6840264	-641.8002625
6	rc_1m5caU23	-642.5234254	0.0912064	-642.4322190	-642.4059802	-0.1174452	-641.3745991	-641.5633298	-641.6795389	-641.7969841
7	rc_1m5caU19	-642.5222776	0.0907586	-642.4315190	-642.4062133	-0.1160643	-641.3731851	-641.5618672	-641.6780639	-641.7941282
8	rc_1m5caU08	-642.5220581	0.0910240	-642.4310340	-642.3962451	-0.1258130	-641.3355752	-638.8818345	0.0000000	-0.1258130
9	rc_1m5caU21	-642.5212516	0.0909765	-642.4302750	-642.4001872	-0.1210643	-641.3686602	-641.5577690	-641.6741323	-641.7951966
10	rc_1m5caU16	-642.5189649	0.0905838	-642.4283810	-642.3914369	-0.1275279	-641.3571623	-641.5460509	-641.6621801	-641.7897080
11	rc_1m5caU20	-642.5182738	0.0909828	-642.4272910	-642.3860400	-0.1322338	-641.3570501	-641.5459860	-641.6621794	-641.7944132
12	rc_1m5caU22	-642.5175741	0.0917580	-642.4258160	-642.3947490	-0.1228250	-641.3624080	-641.5513336	-641.6676262	-641.7904512
13	rc_1m5caU05	-642.5162923	0.0908593	-642.4254330	-642.3813605	-0.1349319	-638.8197731	-638.9119395	0.0000000	-0.1349319
14	rc_1m5caU14	-642.5155293	0.0907672	-642.4247620	-642.3973222	-0.1182070	-641.3630575	-641.5516946	-641.6679261	-641.7861331
15	rc_1m5caU12	-642.5164264	0.0921544	-642.4242720	-642.3824359	-0.1339905	-641.3494948	-641.5386118	-641.6548665	-641.7888570
16	rc_1m5caU06	-642.5149859	0.0911939	-642.4237920	-642.4017701	-0.1132158	-638.8471278	-638.9389343	0.0000000	-0.1132158
17	rc_1m5caU15	-642.5144829	0.0908639	-642.4236190	-642.3929302	-0.1215528	-641.3600285	-641.5485526	-641.6647246	-641.7862774
18	rc_1m5caU09	-642.5116944	0.0907064	-642.4209880	-642.3791194	-0.1325750	-641.3446787	-641.5334422	-641.6495565	-641.7821315
19	rc_1m5caU18	-642.5113795	0.0921755	-642.4192040	-642.3752144	-0.1361652	-641.3443726	-641.5334599	-641.6497484	-641.7859136
20	rc_1m5caU04	-642.5104846	0.0915196	-642.4189650	-642.3875173	-0.1229674	-641.3309171	-641.5188425	-641.6346941	-641.7576615
1	d_C56	-394.5257074	0.0549713	-394.4707360	-394.4025034	-0.1232040	-393.7164862	-393.8412510	-393.9173990	-394.0406030
2	d_C46	-394.5173848	0.0552317	-394.4621530	-394.4157529	-0.1016318	-393.7300211	-393.8545537	-393.9306202	-394.0322521
3	d_C45	-394.5158890	0.0550640	-394.4608250	-394.4124774	-0.1034116	-393.7271729	-393.8516898	-393.9277371	-394.0311487
4	d_C15	-394.5127191	0.0549100	-394.4578090	-394.4006206	-0.1120985	-393.7155656	-393.8408730	-393.9172229	-394.0293214
5	d_C16	-394.5114774	0.0547723	-394.4567050	-394.3906995	-0.1207779	-393.7065058	-393.8322320	-393.9086909	-394.0294688
6	d_C35	-394.4961751	0.0547540	-394.4414210	-394.3965447	-0.0996304	-393.7153657	-393.8394010	-393.9151207	-394.0147511
7	d_C25	-394.4959636	0.0547136	-394.4412500	-394.3954641	-0.1004995	-393.7143079	-393.8384695	-393.9142445	-394.0147440

8	d_C36	-394.4956621	0.0549160	-394.4407460	-394.3911243	-0.1045378	#N/A	#N/A	#N/A	#N/A
9	d_C26	-394.4953432	0.0546781	-394.4406650	-394.3885695	-0.1067737	#N/A	#N/A	#N/A	#N/A
1	d_5mC	-433.8484169	0.0808439	-433.7675730	-433.7240837	-0.1243332	-432.9577044	-433.0937891	-433.1771183	-433.3014515
1	d_5hmC	-509.0771628	0.0850418	-508.9921210	-508.9544115	-0.1227513	-508.0896512	-508.2503525	-508.3487483	-508.4714996
1	d_5fC	-507.8785034	0.0622794	-507.8162240	-507.7720372	-0.1064662	-506.9090605	-507.0669739	-507.1636547	-507.2701209
1	d_5dhmC	-584.3154627	0.0887126	-584.2267500	-584.1862858	-0.1291769	-583.2261744	-583.4112029	-583.5245932	-583.6537701
1	d_5caC01	-583.1515033	0.0661582	-583.0853450	-583.0370353	-0.1144679	-582.0763008	-582.2601990	-582.3727882	-582.4872561
2	d_5caC02	-583.1476222	0.0659382	-583.0816840	-583.0261510	-0.1214712	-582.0644170	-582.2484291	-582.3610456	-582.4825168
3	d_5caC03	-583.1382095	0.0652714	-583.0729380	-583.0277079	-0.1105016	-582.0706086	-582.2539578	-582.3662504	-582.4767520
4	d_5caC04	-583.1381692	0.0653962	-583.0727730	-583.0263969	-0.1117723	-582.0694573	-582.2527995	-582.3650878	-582.4768601
5	d_5caC05	-583.1350581	0.0645630	-583.0704950	-583.0232460	-0.1118121	-582.0654828	-582.2478910	-582.3596893	-582.4715014
6	d_5caC06	-583.1307971	0.0647610	-583.0660360	-583.0174060	-0.1133911	-582.0606809	-582.2432118	-582.3550021	-582.4683932
7	d_5caC07	-583.1266485	0.0660244	-583.0606240	-583.0141961	-0.1124524	-582.0597876	-582.2414395	-582.3528535	-582.4653059
8	d_5caC08	-583.1214617	0.0663806	-583.0550810	-582.9858841	-0.1355776	-582.0264038	-582.2095277	-582.3216255	-582.4572031
1	d_1mC_2	-433.8228800	0.0807019	-433.7421780	-433.7130141	-0.1098659	-432.9486473	-433.0845704	-433.1677027	-433.2775686
2	d_1mC_1	-433.8217340	0.0805100	-433.7412240	-433.7033408	-0.1183932	-432.9398725	-433.0761372	-433.1593325	-433.2777257
1	d_1m5mC_2	-473.1453598	0.1067747	-473.0385850	-473.0374126	-0.1079472	-472.1934029	-472.3405115	-472.4308015	-472.5387487
2	d_1m5mC_1	-473.1448199	0.1072338	-473.0375860	-473.0262464	-0.1185735	-472.1832627	-472.3309539	-472.4213949	-472.5399684
1	d_1m5hmC_3	-548.3749287	0.1120577	-548.2628710	-548.2532341	-0.1216947	-547.3018431	-547.4760838	-547.5826858	-547.7043805
2	d_1m5hmC_1	-548.3725901	0.1112530	-548.2613370	-548.2554324	-0.1171577	-547.3141015	-547.4860594	-547.5914722	-547.7086299
3	d_1m5hmC_2	-548.3724961	0.1118201	-548.2606760	-548.2478486	-0.1246475	-547.3065347	-547.4789362	-547.5844667	-547.7091142
1	d_1m5fC_2	-547.1698323	0.0890092	-547.0808230	-547.0544137	-0.1154186	-546.1142112	-546.2835929	-546.3871960	-546.5026146
2	d_1m5fC_3	-547.1668291	0.0882431	-547.0785860	-547.0659211	-0.1009080	-546.1249498	-546.2936856	-546.3972075	-546.4981155
3	d_1m5fC_1	-547.1669027	0.0887187	-547.0781840	-547.0525769	-0.1143259	-546.1133163	-546.2820981	-546.3854887	-546.4998146
4	d_1m5fC_4	-547.1647936	0.0887415	-547.0760520	-547.0542242	-0.1105694	-546.1133007	-546.2825746	-546.3862028	-546.4967722
1	d_1m5dhmC_3	-623.6232514	0.1139963	-623.5092550	-623.5006325	-0.1226189	-622.4520697	-622.6502583	-622.7717577	-622.8943766
2	d_1m5dhmC_4	-623.6221310	0.1137620	-623.5083690	-623.5030040	-0.1191270	-622.4556632	-622.6541738	-622.7758174	-622.8949444
3	d_1m5dhmC_1	-623.6117815	0.1145895	-623.4971920	-623.4927323	-0.1190493	-622.4549302	-622.6512181	-622.7716464	-622.8906957
4	d_1m5dhmC_2	-623.6109414	0.1143753	-623.4965660	-623.4827735	-0.1281678	-622.4452842	-622.6420104	-622.7625523	-622.8907201
1	d_1m5caC_0	-622.4635555	0.0925765	-622.3709790	-622.3504700	-0.1130855	-621.3096738	-621.5044970	-621.6239917	-621.7370772
2	d_1m5caC_2	-622.4483256	0.0934765	-622.3548490	-622.3366081	-0.1117175	-621.2986498	-621.4928121	-621.6119868	-621.7237043
3	d_1m5caC_1	-622.4400254	0.0926454	-622.3473800	-622.3075230	-0.1325025	-621.2695242	-621.4645436	-621.5839857	-621.7164882
1	d_U4	-414.4103597	0.0431767	-414.3671830	-414.3105298	-0.0998299	-413.6142037	-413.7449778	-413.8249576	-413.9247875
2	d_U1	-414.4070022	0.0430492	-414.3639530	-414.2868952	-0.1201071	-413.5916816	-413.7236745	-413.8040965	-413.9242036
3	d_U3	-414.3929288	0.0427988	-414.3501300	-414.2866781	-0.1062508	-413.5947840	-413.7255425	-413.8053992	-413.9116500
4	d_U2	-414.3927665	0.0426854	-414.3500810	-414.2842230	-0.1085435	-413.5925332	-413.7233757	-413.8032540	-413.9117975
5	d_U5	-414.3906033	0.0426403	-414.3479630	-414.2779879	-0.1126154	-413.5852160	-413.7157861	-413.7955390	-413.9081544
6	d_U6	-414.3891872	0.0427362	-414.3464510	-414.2670169	-0.1221703	-413.5753372	-413.7061075	-413.7859022	-413.9080725
1	d_5mU4	-453.7333786	0.0686916	-453.6646870	-453.6332322	-0.1001465	-452.8569870	-452.9989572	-453.0860763	-453.1862228
2	d_5mU1	-453.7306761	0.0688970	-453.6617790	-453.6115419	-0.1191342	-452.8367362	-452.9798489	-453.0673952	-453.1865294
3	d_5mU3	-453.7164863	0.0682612	-453.6482250	-453.6113027	-0.1051836	-452.8397420	-452.9816223	-453.0685953	-453.1737789
4	d_5mU2	-453.7164586	0.0687885	-453.6476700	-453.6089931	-0.1074654	-452.8376636	-452.9796174	-453.0666070	-453.1740724
5	d_5mU5	-453.7131727	0.0681156	-453.6450570	-453.5994270	-0.1137457	-452.8259317	-452.9677755	-453.0546934	-453.1684391
6	d_5mU6	-453.7115585	0.0682264	-453.6433320	-453.5877491	-0.1238093	-452.8156087	-452.9576182	-453.0445534	-453.1683627
1	d_5hmU214	-528.9617342	0.0730452	-528.8886890	-528.8617623	-0.0999719	-527.9873778	-528.1542366	-528.2565003	-528.3564722
2	d_5hmU114	-528.9591369	0.0732199	-528.8859170	-528.8434442	-0.1156927	-527.9709728	-528.1386537	-528.2412118	-528.3569045
3	d_5hmU134	-528.9452643	0.0728723	-528.8723920	-528.8428548	-0.1024095	-527.9738520	-528.1403958	-528.2424191	-528.3448286
4	d_5hmU125	-528.9451748	0.0728748	-528.8723000	-528.8409240	-0.1042508	-527.9721230	-528.1387098	-528.2407362	-528.3449870

5	d_5hmU156	-528.9410246	0.0724446	-528.8685800	-528.8255185	-0.1155061	-527.9538859	-528.1207346	-528.2228463	-528.3383524
6	d_5hmU246	-528.9384274	0.0730613	-528.8653660	-528.8102106	-0.1282167	-527.9403213	-528.1072953	-528.2094123	-528.3376290
1	d_5fUd	-527.7561824	0.0496363	-527.7065460	-527.6659975	-0.0901849	-526.7948614	-526.9582995	-527.0585860	-527.1487709
2	d_5fUda	-527.7564488	0.0501898	-527.7062590	-527.6573188	-0.0991300	-526.7872172	-526.9507469	-527.0509653	-527.1500953
3	d_5fUa	-527.7484747	0.0495186	-527.6989560	-527.6393358	-0.1091389	-526.7682190	-526.9329416	-527.0337127	-527.1428516
4	d_5fUaa	-527.7485408	0.0504127	-527.6981280	-527.6255643	-0.1229765	-526.7560536	-526.9207592	-527.0213659	-527.1443424
5	d_5fUfa	-527.7414586	0.0503256	-527.6911330	-527.6402340	-0.1012246	-526.7730369	-526.9364703	-527.0365438	-527.1377684
6	d_5fUc	-527.7364824	0.0491423	-527.6873400	-527.6394850	-0.0969974	-526.7718848	-526.9353025	-527.0354694	-527.1324668
7	d_5fUca	-527.7368910	0.0498389	-527.6870520	-527.6293809	-0.1075101	-526.7627759	-526.9262691	-527.0263470	-527.1338571
8	d_5fUb	-527.7362091	0.0492170	-527.6869920	-527.6376020	-0.0986071	-526.7701583	-526.9336754	-527.0338711	-527.1324782
9	d_5fUba	-527.7366325	0.0501825	-527.6864500	-527.6267433	-0.1098892	-526.7604673	-526.9240621	-527.0241663	-527.1340555
10	d_5fUe	-527.7352576	0.0490866	-527.6861710	-527.6342765	-0.1009811	-526.7659670	-526.9294295	-527.0295679	-527.1305490
11	d_5fUea	-527.7356115	0.0497664	-527.6858450	-527.6298482	-0.1057632	-526.7623854	-526.9257632	-527.0257694	-527.1315326
12	d_5fUga	-527.7319085	0.0492224	-527.6826860	-527.6230339	-0.1088746	-526.7551767	-526.9189920	-527.0191803	-527.1280549
13	d_5fUf	-527.7316934	0.0493623	-527.6823310	-527.6229867	-0.1087067	-526.7553843	-526.9190682	-527.0192379	-527.1279446
14	d_5fUg	-527.7195337	0.0498996	-527.6696340	-527.5885561	-0.1309775	-526.7212939	-526.8858752	-526.9864255	-527.1174030
15	d_5fUha	-527.7196119	0.0504958	-527.6691160	-527.5795116	-0.1401003	-526.7128520	-526.8776399	-526.9781419	-527.1182422
16	d_5fUh	-527.7182831	0.0506440	-527.6676390	-527.5917646	-0.1265184	-526.7243988	-526.8887961	-526.9892753	-527.1157937
1	d_5dhmU_52	-604.2023989	0.0768329	-604.1255660	-604.1018591	-0.1005398	-603.1317678	-603.3227748	-603.4399400	-603.5404798
2	d_5dhmU_50	-604.2023704	0.0770443	-604.1253260	-604.0979356	-0.1044348	-603.1284963	-603.3194180	-603.4365117	-603.5409465
3	d_5dhmU_56	-604.2005439	0.0767458	-604.1237980	-604.0966692	-0.1038746	-603.1277011	-603.3186527	-603.4357701	-603.5396447
4	d_5dhmU_27	-604.2005350	0.0768209	-604.1237140	-604.0857455	-0.1147895	-603.1172821	-603.3090779	-603.4265299	-603.5413194
5	d_5dhmU_53	-604.1997202	0.0760862	-604.1236340	-604.0896552	-0.1100650	-603.1197259	-603.3109059	-603.4281672	-603.5382322
6	d_5dhmUa11	-604.1999195	0.0771564	-604.1227630	-604.0784930	-0.1214265	-603.1106310	-603.3023898	-603.4197827	-603.5412092
7	d_5dhmU_30	-604.1982655	0.0765104	-604.1217550	-604.0759884	-0.1222771	#N/A	#N/A	#N/A	#N/A
8	d_5dhmU_48	-604.1981955	0.0766335	-604.1215620	-604.0936448	-0.1045507	-603.1245251	-603.3156619	-603.4328830	-603.5374337
9	d_5dhmUa28	-604.1973882	0.0772321	-604.1201560	-604.0813317	-0.1160565	-603.1139844	-603.3056339	-603.4230136	-603.5390701
10	d_5dhmU_29	-604.1963201	0.0762100	-604.1201100	-604.0670693	-0.1292507	#N/A	#N/A	#N/A	#N/A
11	d_5dhmU_24	-604.1964908	0.0766807	-604.1198100	-604.0775448	-0.1189460	#N/A	#N/A	#N/A	#N/A
12	d_5dhmUa21	-604.1953538	0.0762218	-604.1191320	-604.0592880	-0.1360658	#N/A	#N/A	#N/A	#N/A
13	d_5dhmUa27	-604.1958195	0.0768324	-604.1189870	-604.0634130	-0.1324065	-603.0951546	-603.2874761	-603.4051144	-603.5375209
14	d_5dhmUa29	-604.1953595	0.0769825	-604.1183770	-604.0623246	-0.1330349	-603.0947663	-603.2869107	-603.4044776	-603.5375125
15	d_5dhmUa13	-604.1935392	0.0756551	-604.1178840	-604.0443294	-0.1492098	-603.0784399	-603.2707984	-603.3883670	-603.5375768
16	d_5dhmUa26	-604.1942824	0.0764544	-604.1178280	-604.0642196	-0.1300629	-603.0966060	-603.2886501	-603.4061913	-603.5362542
17	d_5dhmUa24	-604.1941477	0.0764016	-604.1177460	-604.0613486	-0.1327991	-603.0932991	-603.2857289	-603.4034380	-603.5362371
18	d_5dhmUa17	-604.1923278	0.0760947	-604.1162330	-604.0482684	-0.1440594	-603.0814738	-603.2738938	-603.3915018	-603.5355612
19	d_5dhmUb15_b	-604.1871213	0.0766443	-604.1104770	#N/A	#N/A	#N/A	#N/A	#N/A	#N/A
20	d_5dhmU_13	-604.1867786	0.0766415	-604.1101370	-604.0784650	-0.1083136	#N/A	#N/A	#N/A	#N/A
1	d_5caU79	-603.0216517	0.0539167	-602.9677350	-602.9321451	-0.0895067	-601.9678257	-602.1555942	-602.2708782	-602.3603849
2	d_5caU78	-603.0190423	0.0541802	-602.9648620	-602.8909116	-0.1281307	-601.9227986	-602.1126942	-602.2289037	-602.3570344
3	d_5caU108	-603.0182345	0.0536874	-602.9645470	-602.9147763	-0.1034582	-601.9508993	-602.1394353	-602.2549631	-602.3584213
4	d_5caU106	-603.0172402	0.0536871	-602.9635530	-602.9110559	-0.1061842	-601.9463688	-602.1351747	-602.2508283	-602.3570125
5	d_5caU76	-603.0139792	0.0536421	-602.9603370	-602.9112282	-0.1027509	-601.9457645	-602.1338527	-602.2492427	-602.3519936
6	d_5caU303	-603.0136508	0.0537427	-602.9599080	-602.9140832	-0.0995676	-601.9486242	-602.1365910	-602.2519537	-602.3515213
7	d_5caU304	-603.0093470	0.0539870	-602.9553600	-602.9025504	-0.1067966	-601.9383203	-602.1263012	-602.2415510	-602.3483476
8	d_5caU307	-603.0061360	0.0531949	-602.9529410	-602.9125644	-0.0935716	-601.9521935	-602.1397131	-602.2547738	-602.3483454
9	d_5caU311	-603.0059918	0.0531937	-602.9527980	-602.9111818	-0.0948100	-601.9509235	-602.1385014	-602.2535736	-602.3483836
10	d_5caU105	-603.0062104	0.0535934	-602.9526170	-602.8805524	-0.1256580	-601.9154842	-602.1048342	-602.2206771	-602.3463351

11	d_5caU316	-603.0050964	0.0531063	-602.9519900	-602.9044472	-0.1006492	-601.9436792	-602.1319007	-602.2472646	-602.3479138
12	d_5caU312	-603.0049359	0.0530759	-602.9518600	-602.9047872	-0.1001488	-601.9440852	-602.1321818	-602.2474691	-602.3476179
13	d_5caU09	-603.0056705	0.0539315	-602.9517390	-602.8850709	-0.1205996	-601.9196029	-602.1088291	-602.2246418	-602.3452414
14	d_5caU208	-603.0036900	0.0540190	-602.9496710	-602.8783379	-0.1253521	-601.9116666	-602.1015998	-602.2177585	-602.3431106
15	d_5caU59	-603.0016450	0.0539469	-602.9476980	-602.8706489	-0.1309961	-601.9077467	-602.0969958	-602.2126975	-602.3436936
16	d_5caU305	-602.9992101	0.0533570	-602.9458530	-602.8912340	-0.1079761	-601.9292404	-602.1171325	-602.2322837	-602.3402598
17	d_5caU306	-602.9955578	0.0528987	-602.9426590	-602.8844450	-0.1111128	-601.9214893	-602.1096969	-602.2250329	-602.3361457
18	d_5caU309	-602.9953936	0.0531425	-602.9422510	-602.8855019	-0.1098917	-601.9245133	-602.1125448	-602.2276993	-602.3375910
19	d_5caU308	-602.9952491	0.0530650	-602.9421840	-602.8901187	-0.1051304	-601.9275350	-602.1157086	-602.2310415	-602.3361719
20	d_5caU223	-602.9954721	0.0534560	-602.9420160	-602.8492199	-0.1462522	-601.8828537	-602.0733366	-602.1896887	-602.3359409
1	d_1mU	-453.7180261	0.0686320	-453.6493940	-453.5994985	-0.1185276	-452.8247818	-452.9674328	-453.0546493	-453.1731769
1	d_1m5mU	-493.0412050	0.0948969	-492.9463080	-492.9240053	-0.1171997	-492.0698103	-492.2235791	-492.3179148	-492.4351145
1	d_1m5hmU12	-568.2700719	0.0998229	-568.1702490	-568.1561083	-0.1139636	-567.2041115	-567.3825064	-567.4918813	-567.6058449
2	d_1m5hmU11	-568.2683187	0.0992377	-568.1690810	-568.1423478	-0.1259710	-567.1906916	-567.3693197	-567.4787850	-567.6047560
3	d_1m5hmU5	-568.2672849	0.0990849	-568.1682000	-568.1389923	-0.1282926	-567.1872432	-567.3661088	-567.4756550	-567.6039476
4	d_1m5hmU7	-568.2670228	0.0989838	-568.1680390	-568.1450883	-0.1219345	-567.1937506	-567.3722413	-567.4816956	-567.6036301
5	d_1m5hmU6	-568.2660342	0.0983922	-568.1676420	-568.1433178	-0.1227164	-567.1915741	-567.3703933	-567.4800213	-567.6027377
1	d_1m5fU1_a	-567.0607870	0.0761790	-566.9846080	-566.9527385	-0.1080485	-566.0018576	-566.1773298	-566.2849356	-566.3929841
2	d_1m5fU2	-567.0605829	0.0765219	-566.9840610	-566.9390673	-0.1215156	-565.9898363	-566.1652945	-566.2727408	-566.3942564
1	d_1m5dhmU_a1_8	-643.5116889	0.1037718	-643.4079170	-643.3975965	-0.1140924	-642.3498403	-642.5523422	-642.6765900	-642.7906824
2	d_1m5dhmU_f2_12	-643.5110407	0.1032527	-643.4077880	-643.3916590	-0.1193817	-642.3442779	-642.5467453	-642.6709539	-642.7903356
3	d_1m5dhmU_a1_10	-643.5093677	0.1032376	-643.4061300	-643.3889607	-0.1204069	-642.3422813	-642.5447093	-642.6688924	-642.7892993
4	d_1m5dhmU_f2_3	-643.5076864	0.1026023	-643.4050840	-643.3905536	-0.1171328	-642.3434058	-642.5460932	-642.6704444	-642.7875772
5	d_1m5dhmU_a_12	-643.5073027	0.1022986	-643.4050040	-643.3793674	-0.1279352	-642.3313996	-642.5343917	-642.6588977	-642.7868329
6	d_1m5dhmU_a_6	-643.5086869	0.1037018	-643.4049850	-643.3944773	-0.1142096	-642.3475725	-642.5499393	-642.6741467	-642.7883563
7	d_1m5dhmU_f1_7	-643.5073028	0.1023197	-643.4049830	-643.3793735	-0.1279293	-642.3314052	-642.5343969	-642.6589026	-642.7868319
8	d_1m5dhmU_f1_2	-643.5076929	0.1028259	-643.4048670	-643.3905701	-0.1171228	-642.3434408	-642.5461232	-642.6704723	-642.7875951
9	d_1m5dhmU_f1_15	-643.5063517	0.1024886	-643.4038630	-643.3715971	-0.1347546	-642.3243266	-642.5275240	-642.6520674	-642.7868220
10	d_1m5dhmU_f1_6	-643.5068359	0.1033178	-643.4035180	-643.3757278	-0.1311081	#N/A	#N/A	#N/A	#N/A
11	d_1m5dhmU_a_10	-643.5063522	0.1031881	-643.4031640	-643.3753999	-0.1309523	-642.3285910	-642.5313292	-642.6556483	-642.7866006
12	d_1m5dhmU_a_11	-643.5053103	0.1026163	-643.4026940	-643.3764168	-0.1288935	-642.3294453	-642.5321742	-642.6565228	-642.7854163
13	d_1m5dhmU_f1_4	-643.5045526	0.1019996	-643.4025530	-643.3570670	-0.1474857	#N/A	#N/A	#N/A	#N/A
14	d_1m5dhmU_a_3	-643.5051875	0.1027025	-643.4024850	-643.3732842	-0.1319033	-642.3260006	-642.5290775	-642.6535718	-642.7854751
15	d_1m5dhmU_f_3	-643.5045527	0.1021747	-643.4023780	-643.3570691	-0.1474836	-642.3118337	-642.5148427	-642.6392012	-642.7866848
16	d_1m5dhmU_a1_7	-643.5033775	0.1023915	-643.4009860	-643.3616224	-0.1417551	-642.3154308	-642.5184849	-642.6428714	-642.7846265
17	d_1m5dhmU_d2_12	-643.4969055	0.1015795	-643.3953260	#N/A	#N/A	#N/A	#N/A	#N/A	#N/A
18	d_1m5dhmU_d_3	-643.4964259	0.1013719	-643.3950540	-643.3683830	-0.1280429	-642.3108045	-642.5159162	-642.6415047	-642.7695476
19	d_1m5dhmU_d1_6	-643.4969490	0.1020939	-643.3948550	-643.3688423	-0.1281067	-642.3117572	-642.5164667	-642.6418893	-642.7699960
20	d_1m5dhmU_d_8	-643.4969229	0.1023299	-643.3945930	-643.3638250	-0.1330979	-642.3044772	-642.5096391	-642.6350562	-642.7681541
1	d_1m5caU01	-642.3314915	0.0807375	-642.2507540	-642.2041783	-0.1273133	-641.1554491	-641.3564506	-641.4796539	-641.6069672
2	d_1m5caU02	-642.3315678	0.0810628	-642.2505050	-642.2040951	-0.1274727	-641.1556707	-641.3565320	-641.4796742	-641.6071469
3	d_1m5caU06	-642.3298439	0.0795789	-642.2502650	-642.2248588	-0.1049851	-641.1799708	-641.3797820	-641.5023830	-641.6073681
4	d_1m5caU08	-642.3181688	0.0804988	-642.2376700	-642.1937044	-0.1244645	-641.1490091	-641.3490579	-641.4717088	-641.5961733
5	d_1m5caU04	-642.3177014	0.0804684	-642.2372330	-642.1983734	-0.1193280	-641.1532899	-641.3532665	-641.4759184	-641.5952464
6	d_1m5caU05	-642.3167610	0.0804980	-642.2362630	-642.1925266	-0.1242344	-641.1456066	-641.3465568	-641.4696534	-641.5938878
7	d_1m5caU07	-642.3133657	0.0807037	-642.2326620	-642.1842060	-0.1291597	-641.1416171	-641.3415280	-641.4640248	-641.5931845
8	d_1m5caU13	-642.3075297	0.0801156	-642.2274140	-642.1618940	-0.1456357	-641.1145658	-641.3160880	-641.4394196	-641.5850553
9	d_1m5caU15	-642.3028103	0.0806383	-642.2221720	-642.1448553	-0.1579551	-641.0994923	-641.3011088	-641.4244043	-641.5823594

1	rd_C	-394.3345963	0.0535293	-394.2810670	-394.2962187	-0.0383777	-393.6191405	-393.7355220	-393.8072538	-393.8456315
2	rd_C46	-394.3342561	0.0537431	-394.2805130	-394.3039170	-0.0303392	-393.6277156	-393.7441643	-393.8158449	-393.8461841
3	rd_C45	-394.3334694	0.0539554	-394.2795140	-394.3026643	-0.0308051	-393.6264765	-393.7428201	-393.8144621	-393.8452672
4	rd_C15	-394.3243184	0.0537303	-394.2705880	-394.2913748	-0.0329435	-393.6125116	-393.7286056	-393.8000598	-393.8330033
5	rd_C16	-394.3230828	0.0534497	-394.2696330	-394.2826053	-0.0404775	-393.6047803	-393.7209684	-393.7924024	-393.8328799
6	rd_C35	-394.3207799	0.0536549	-394.2671250	-394.2985980	-0.0221819	-393.6249742	-393.7408151	-393.8122995	-393.8344814
7	rd_C25	-394.3205001	0.0534081	-394.2670920	-394.2985192	-0.0219810	-393.6247083	-393.7406039	-393.8121241	-393.8341051
8	rd_C36	-394.3200042	0.0537031	-394.2663010	-394.2920365	-0.0279677	#N/A	#N/A	#N/A	#N/A
9	rd_C26	-394.3196568	0.0535887	-394.2660680	-394.2905055	-0.0291512	#N/A	#N/A	#N/A	#N/A
1	rd_5mC	-433.6652509	0.0794588	-433.5857920	-433.6250079	-0.0402430	-432.8662447	-432.9944203	-433.0735453	-433.1137883
1	rd_5hmC	-508.8866035	0.0838515	-508.8027520	-508.8425933	-0.0440102	-507.9865936	-508.1388445	-508.2327569	-508.2767671
1	rd_5fC	-507.6645116	0.0605446	-507.6039670	-507.6316276	-0.0328840	-506.7796434	-506.9292007	-507.0213528	-507.0542368
1	rd_5dhmC	-584.1218203	0.0873692	-584.0344510	-584.0747776	-0.0470427	-583.1232785	-583.2997324	-583.4085529	-583.4555956
1	rd_5caC01	-582.9305150	0.0649349	-582.8655800	-582.8762747	-0.0542403	-581.9118078	-582.0877638	-582.1956980	-582.2499383
2	rd_5caC02	-582.9262310	0.0644530	-582.8617780	-582.8819830	-0.0442480	#N/A	#N/A	#N/A	#N/A
3	rd_5caC03	-582.9235424	0.0640853	-582.8594570	-582.8879640	-0.0355784	-581.9400214	-582.1139516	-582.2211451	-582.2567235
4	rd_5caC04	-582.9183968	0.0645247	-582.8538720	-582.8753454	-0.0430514	-581.9285489	-582.1026072	-582.2097415	-582.2527929
5	rd_5caC05	-582.9179586	0.0642746	-582.8536840	-582.8793622	-0.0385964	-581.9128334	-582.0873558	-582.1941781	-582.2327745
6	rd_5caC06	-582.9169040	0.0647270	-582.8521770	-582.8675572	-0.0493468	-581.9202406	-582.0944538	-582.2016291	-582.2509759
7	rd_5caC07	-582.9026798	0.0649587	-582.8377210	-582.8379791	-0.0647007	-581.8862046	-582.0611238	-582.1687162	-582.2334169
1	rd_1mC_2	-433.6393500	0.0800930	-433.5592570	-433.6088301	-0.0305199	-432.8485348	-432.9759239	-433.0544565	-433.0849764
2	rd_1mC_1	-433.6380565	0.0793884	-433.5586680	-433.5998967	-0.0381598	-432.8406777	-432.9682248	-433.0467672	-433.0849270
1	rd_1m5mC_2	-472.9641977	0.1052267	-472.8589710	-472.9325967	-0.0316010	-472.0895842	-472.2286225	-472.3144395	-472.3460405
2	rd_1m5mC_1	-472.9627989	0.1057949	-472.8570040	-472.9223634	-0.0404356	-472.0811183	-472.2202885	-472.3061099	-472.3465455
1	rd_1m5hmC_3	-548.1908591	0.1092210	-548.0816380	-548.1509395	-0.0399196	-547.2100920	-547.3738404	-547.4748544	-547.5147740
2	rd_1m5hmC_1	-548.1875543	0.1098493	-548.0777050	-548.1464636	-0.0410907	-547.2082727	-547.3719481	-547.4728133	-547.5139040
3	rd_1m5hmC_2	-548.1867053	0.1104682	-548.0762370	-548.1407468	-0.0459584	-547.2034298	-547.3671383	-547.4680005	-547.5139589
1	rd_1m5fC_2	-546.9723032	0.0863921	-546.8859110	-546.9357077	-0.0365955	-546.0022362	-546.1627983	-546.2616855	-546.2982810
2	rd_1m5fC_3	-546.9715407	0.0864186	-546.8851220	-546.9418383	-0.0297024	-546.0070940	-546.1675814	-546.2664996	-546.2962020
3	rd_1m5fC_1	-546.9691486	0.0859885	-546.8831600	-546.9320462	-0.0371023	-545.9989259	-546.1592891	-546.2580945	-546.2951968
4	rd_1m5fC_4	-546.9687454	0.0870833	-546.8816620	-546.9307749	-0.0379705	-545.9966967	-546.1572544	-546.2561133	-546.2940838
1	rd_1m5dhmC_4	-623.4321750	0.1144509	-623.3177240	-623.3879403	-0.0442347	-622.3451192	-622.5333532	-622.6493938	-622.6936285
2	rd_1m5dhmC_3	-623.4259695	0.1125725	-623.3133970	-623.3844747	-0.0414948	-622.3468951	-622.5346086	-622.6503869	-622.6918817
3	rd_1m5dhmC_1	-623.4254261	0.1140221	-623.3114040	-623.3827412	-0.0426849	-622.3481173	-622.5358837	-622.6516085	-622.6942934
4	rd_1m5dhmC_2	-623.4237956	0.1136426	-623.3101530	-623.3766889	-0.0471068	-622.3433940	-622.5310287	-622.6466820	-622.6937888
1	rd_1m5caC_3	-622.2424436	0.0909895	-622.1514540	-622.2006849	-0.0417587	-621.1514909	-621.3380440	-621.4523936	-621.4941523
2	rd_1m5caC_2	-622.2351633	0.0916093	-622.1435540	-622.1909794	-0.0441839	-621.1433376	-621.3296678	-621.4440683	-621.4882522
3	rd_1m5caC_1	-622.2336130	0.0901629	-622.1434500	-622.1802567	-0.0533562	-621.1333892	-621.3186031	-621.4318701	-621.4852263
1	rd_U4	-414.2081492	0.0412522	-414.1668970	-414.1834247	-0.0247246	-413.4977262	-413.6202877	-413.6956849	-413.7204095
2	rd_U1	-414.1898590	0.0412769	-414.1485820	-414.1380876	-0.0517713	-413.4487717	-413.5685071	-413.6453652	-413.6971365
3	rd_U5	-414.1890463	0.0410053	-414.1480410	-414.1606407	-0.0284057	-413.4784631	-413.6007144	-413.6760062	-413.7044119
4	rd_U3	-414.1875587	0.0409797	-414.1465790	-414.1618249	-0.0257338	-413.4787724	-413.6011399	-413.6764690	-413.7022028
5	rd_U6	-414.1871005	0.0406495	-414.1464510	-414.1495611	-0.0375394	-413.4682226	-413.5905827	-413.6658782	-413.7034176
6	rd_U2	-414.1868573	0.0408383	-414.1460190	-414.1595111	-0.0273462	-413.4762861	-413.5987746	-413.6741662	-413.7015124
1	rd_5mU4	-453.5408903	0.0669392	-453.4739510	-453.5154634	-0.0254268	-452.7475646	-452.8818902	-452.9647165	-452.9901433
2	rd_5mU1	-453.5240783	0.0669953	-453.4570830	-453.4730816	-0.0509967	-452.6984515	-452.8344830	-452.9173811	-452.9683778
3	rd_5mU5	-453.5210265	0.0668285	-453.4541980	-453.4909669	-0.0300597	-452.7253053	-452.8592702	-452.9419242	-452.9719839
4	rd_5mU3	-453.5196954	0.0666424	-453.4530530	-453.4929165	-0.0267790	-452.7271964	-452.8613531	-452.9440777	-452.9708567

5	rd_5mU2	-453.5192492	0.0670442	-453.4522050	-453.4904100	-0.0288393	-452.7246464	-452.8588989	-452.9416598	-452.9704991
6	rd_5mU6	-453.5184110	0.0666189	-453.4517920	-453.4782854	-0.0401256	-452.7145622	-452.8487412	-452.9314497	-452.9715753
1	rd_5hmU214	-528.7610347	0.0714937	-528.6895410	-528.7308182	-0.0302165	-527.8660840	-528.0248738	-528.1226304	-528.1528469
2	rd_5hmU114	-528.7454694	0.0716313	-528.6738380	-528.6910669	-0.0544025	-527.8198049	-527.9800371	-528.0782741	-528.1326766
3	rd_5hmU156	-528.7408829	0.0709218	-528.6699610	-528.7045571	-0.0363258	-527.8428096	-528.0013088	-528.0989256	-528.1352514
4	rd_5hmU134	-528.7409460	0.0715849	-528.6693610	-528.7087841	-0.0321619	-527.8453134	-528.0040442	-528.1017385	-528.1339004
5	rd_5hmU125	-528.7404616	0.0717485	-528.6687130	-528.7064683	-0.0339933	-527.8428903	-528.0016881	-528.0993938	-528.1333871
6	rd_5hmU246	-528.7368921	0.0726530	-528.6642390	-528.6866069	-0.0502852	-527.8265662	-527.9852157	-528.0828298	-528.1331150
1	rd_5fUd	-527.5356847	0.0476636	-527.4880210	-527.5112599	-0.0244248	-526.6526428	-526.8082244	-526.9040265	-526.9284513
2	rd_5fUda	-527.5336429	0.0479338	-527.4857090	#N/A	#N/A	#N/A	#N/A	#N/A	#N/A
3	rd_5fUfa	-527.5185336	0.0480865	-527.4704470	-527.4867049	-0.0318287	-526.6309344	-526.7863363	-526.8819800	-526.9138087
4	rd_5fUe	-527.5157406	0.0471736	-527.4685670	-527.4869190	-0.0288216	-526.6299707	-526.7853054	-526.8809938	-526.9098154
5	rd_5fUa	-527.5160016	0.0474945	-527.4685070	-527.4693980	-0.0466036	-526.6050154	-526.7619103	-526.8579489	-526.9045525
6	rd_5fUc	-527.5152726	0.0476456	-527.4676270	-527.4896445	-0.0256281	-526.6327025	-526.7881875	-526.8839173	-526.9095454
7	rd_5fUb	-527.5145018	0.0473797	-527.4671220	-527.4876527	-0.0268491	-526.6309588	-526.7860943	-526.8815395	-526.9083886
8	rd_5fUea	-527.5146408	0.0476737	-527.4669670	#N/A	#N/A	#N/A	#N/A	#N/A	#N/A
9	rd_5fUca	-527.5128392	0.0476372	-527.4652020	#N/A	#N/A	#N/A	#N/A	#N/A	#N/A
10	rd_5fUba	-527.5121763	0.0478222	-527.4643540	-527.4776033	-0.0345730	-526.6213236	-526.7767219	-526.8723788	-526.9069518
11	rd_5fUf	-527.5110533	0.0479132	-527.4631400	-527.4725501	-0.0385032	#N/A	#N/A	#N/A	#N/A
12	rd_5fUg	-527.4958516	0.0469795	-527.4488720	-527.4544713	-0.0413803	#N/A	#N/A	#N/A	#N/A
13	rd_5fUha	-527.4961385	0.0474805	-527.4486580	-527.4478497	-0.0482888	#N/A	#N/A	#N/A	#N/A
1	rd_5dhmU_52	-603.9986375	0.0764134	-603.9222240	-603.9634218	-0.0352157	-603.0021577	-603.1850083	-603.2975567	-603.3327724
2	rd_5dhmU_53	-603.9970720	0.0755410	-603.9215310	-603.9589903	-0.0380818	-602.9983562	-603.1812521	-603.2938461	-603.3319279
3	rd_5dhmU_50	-603.9975267	0.0762166	-603.9213100	-603.9612487	-0.0362780	-603.0008034	-603.1836071	-603.2961140	-603.3323920
4	rd_5dhmUa14	-603.9821917	0.0767527	-603.9054390	-603.9280695	-0.0541223	-602.9607006	-603.1447618	-603.2575458	-603.3116681
5	rd_5dhmU_30	-603.9803354	0.0760334	-603.9043020	-603.9214050	-0.0589304	#N/A	#N/A	#N/A	#N/A
6	rd_5dhmUa26	-603.9794721	0.0756900	-603.9037820	-603.9166541	-0.0628180	-602.9498215	-603.1341153	-603.2469133	-603.3097313
7	rd_5dhmU_24	-603.9782765	0.0748095	-603.9034670	-603.9221574	-0.0561192	-602.9550324	-603.1396283	-603.2525945	-603.3087137
8	rd_5dhmU_6	-603.9778165	0.0756765	-603.9021400	-603.9304217	-0.0473948	#N/A	#N/A	#N/A	#N/A
9	rd_5dhmU_66	-603.9780173	0.0759303	-603.9020870	-603.9349607	-0.0430566	#N/A	#N/A	#N/A	#N/A
10	rd_5dhmU_68	-603.9778413	0.0757562	-603.9020850	-603.9299925	-0.0478488	#N/A	#N/A	#N/A	#N/A
11	rd_5dhmU_59	-603.9770912	0.0751772	-603.9019140	-603.9348532	-0.0422381	#N/A	#N/A	#N/A	#N/A
12	rd_5dhmUa28	-603.9776842	0.0758672	-603.9018170	-603.9217830	-0.0559012	-602.9565975	-603.1406958	-603.2535988	-603.3095000
13	rd_5dhmU_70	-603.9774471	0.0757271	-603.9017200	-603.9305071	-0.0469400	#N/A	#N/A	#N/A	#N/A
14	rd_5dhmU_63	-603.9771828	0.0755917	-603.9015910	-603.9351498	-0.0420330	#N/A	#N/A	#N/A	#N/A
15	rd_5dhmU_15	-603.9772162	0.0756571	-603.9015590	-603.9394442	-0.0377720	#N/A	#N/A	#N/A	#N/A
16	rd_5dhmU_72	-603.9760949	0.0747489	-603.9013460	-603.9255983	-0.0504966	#N/A	#N/A	#N/A	#N/A
17	rd_5dhmUa29	-603.9780709	0.0767998	-603.9012710	-603.9072860	-0.0707849	#N/A	#N/A	#N/A	#N/A
18	rd_5dhmU_58	-603.9757019	0.0744939	-603.9012080	-603.9303781	-0.0453239	#N/A	#N/A	#N/A	#N/A
19	rd_5dhmU_17	-603.9765683	0.0753803	-603.9011880	-603.9370077	-0.0395606	#N/A	#N/A	#N/A	#N/A
20	rd_5dhmUb14	-603.9765193	0.0755693	-603.9009500	-603.9370626	-0.0394567	#N/A	#N/A	#N/A	#N/A
1	rd_5caU79	-602.7977801	0.0523550	-602.7454250	-602.7692473	-0.0285327	-601.8153355	-601.9953530	-602.1060877	-602.1346204
2	rd_5caU73	-602.7941571	0.0522360	-602.7419210	-602.7623044	-0.0318527	-601.8072790	-601.9873097	-602.0980822	-602.1299349
3	rd_5caU76	-602.7938287	0.0521877	-602.7416410	-602.7599256	-0.0339031	-601.8053702	-601.9853174	-602.0960476	-602.1299507
4	rd_5caU78	-602.7928964	0.0521893	-602.7407070	-602.7510841	-0.0418123	-601.7854067	-601.9651868	-602.0757330	-602.1175453
5	rd_5caU72	-602.7925801	0.0529310	-602.7396490	-602.7570689	-0.0355112	#N/A	#N/A	#N/A	#N/A
6	rd_5caU70	-602.7905720	0.0519349	-602.7386370	-602.7495538	-0.0410182	-601.7952750	-601.9754137	-602.0861215	-602.1271397
7	rd_5caU49	-602.7784418	0.0521268	-602.7263150	-602.7487313	-0.0297105	-601.7962974	-601.9762871	-602.0869548	-602.1166653

8	rd_5caU12	-602.7775914	0.0518093	-602.7257820	-602.7342429	-0.0433485	-601.7677294	-601.9477885	-602.0583895	-602.1017380
9	rd_5caU38	-602.7770873	0.0519913	-602.7250960	-602.7360166	-0.0410707	-601.7622354	-601.9437889	-602.0550685	-602.0961392
10	rd_5caU67	-602.7758801	0.0519871	-602.7238930	-602.7385682	-0.0373120	-601.7861674	-601.9659709	-602.0766631	-602.1139751
11	rd_5caU09	-602.7752348	0.0521948	-602.7230400	-602.7215638	-0.0536710	#N/A	#N/A	#N/A	#N/A
12	rd_5caU61	-602.7748450	0.0519990	-602.7228460	-602.7388471	-0.0359979	-601.7872009	-601.9671217	-602.0778316	-602.1138295
13	rd_5caU55	-602.7746953	0.0519033	-602.7227920	-602.7388740	-0.0358214	-601.7870040	-601.9666839	-602.0772814	-602.1131028
14	rd_5caU63	-602.7750129	0.0523899	-602.7226230	-602.7394443	-0.0355686	#N/A	#N/A	#N/A	#N/A
15	rd_5caU18	-602.7755411	0.0531780	-602.7223630	-602.7282450	-0.0472961	-601.7630287	-601.9431297	-602.0538960	-602.1011921
16	rd_5caU40	-602.7742874	0.0519333	-602.7223540	-602.7409576	-0.0333298	-601.7877850	-601.9676913	-602.0783949	-602.1117247
17	rd_5caU44	-602.7738620	0.0517240	-602.7221380	-602.7169407	-0.0569213	#N/A	#N/A	#N/A	#N/A
18	rd_5caU56	-602.7746784	0.0526563	-602.7220220	-602.7332413	-0.0414371	-601.7456057	-601.9254347	-602.0360236	-602.0774607
19	rd_5caU43	-602.7737452	0.0517391	-602.7220060	-602.7385317	-0.0352135	-601.7860421	-601.9658721	-602.0765494	-602.1117629
20	rd_5caU13	-602.7735727	0.0515977	-602.7219750	-602.7193438	-0.0542289	-601.7530445	-601.9329978	-602.0435005	-602.0977294
1	rd_1mU	-453.5076903	0.0679743	-453.4397160	-453.4624111	-0.0452793	-452.6908696	-452.8257052	-452.9085860	-452.9538653
1	rd_1m5mU	-492.8403015	0.0927874	-492.7475140	-492.7933084	-0.0469931	-491.9399360	-492.0868968	-492.1769802	-492.2239733
1	rd_1m5hmU6	-568.0624021	0.0967901	-567.9656120	-568.0117841	-0.0506180	-567.0612383	-567.2328749	-567.3381283	-567.3887463
2	rd_1m5hmU8	-568.0629787	0.0980737	-567.9649050	-568.0081825	-0.0547962	-567.0585778	-567.2298913	-567.3349527	-567.3897489
3	rd_1m5hmU14	-568.0617337	0.0980986	-567.9636350	-568.0116951	-0.0500385	-567.0610308	-567.2323812	-567.3375739	-567.3876124
4	rd_1m5hmU11	-568.0589463	0.0978502	-567.9610960	-568.0007190	-0.0582273	-567.0511803	-567.2223356	-567.3274768	-567.3857041
1	rd_1m5fU1_a	-566.8346224	0.0740224	-566.7606000	-566.7936935	-0.0409289	-565.8479303	-566.0160739	-566.1193054	-566.1602343
2	rd_1m5fU2	-566.8314545	0.0739474	-566.7575070	-566.7868300	-0.0446245	-565.8348862	-566.0025032	-566.1055829	-566.1502074
1	rd_1m5dhmU_d_3	-643.3053919	0.1013838	-643.2040080	-643.2709764	-0.0344154	-642.2233107	-642.4171722	-642.5367365	-642.5711519
2	rd_1m5dhmU_d_14	-643.3043779	0.1008999	-643.2034780	-643.2680404	-0.0363375	-642.2210133	-642.4148461	-642.5344071	-642.5707446
3	rd_1m5dhmU_d_6	-643.3053839	0.1023878	-643.2029960	-643.2680111	-0.0373728	-642.2205002	-642.4142417	-642.5337268	-642.5710996
4	rd_1m5dhmU_d_8	-643.3057415	0.1030344	-643.2027070	-643.2709792	-0.0347623	-642.2247708	-642.4182834	-642.5376479	-642.5724102
5	rd_1m5dhmU_d1_1	-643.3023385	0.1005364	-643.2018020	-643.2627608	-0.0395776	-642.2174002	-642.4111580	-642.5306368	-642.5702144
6	rd_1m5dhmU_d2_10	-643.3012034	0.1003944	-643.2008090	-643.2567050	-0.0444984	-642.2123651	-642.4061059	-642.5255399	-642.5700383
7	rd_1m5dhmU_d1_7	-643.3011850	0.1005659	-643.2006190	-643.2708539	-0.0303310	-642.2246898	-642.4185513	-642.5381007	-642.5684317
8	rd_1m5dhmU_a1_8	-643.2995662	0.1030421	-643.1965240	#N/A	#N/A	#N/A	#N/A	#N/A	#N/A
9	rd_1m5dhmU_a1_10	-643.2976098	0.1020117	-643.1955980	#N/A	#N/A	#N/A	#N/A	#N/A	#N/A
10	rd_1m5dhmU_a_11	-643.2966235	0.1020365	-643.1945870	#N/A	#N/A	#N/A	#N/A	#N/A	#N/A
11	rd_1m5dhmU_f1_2	-643.2953479	0.1009799	-643.1943680	-643.2434790	-0.0518690	#N/A	#N/A	#N/A	#N/A
12	rd_1m5dhmU_f2_3	-643.2953433	0.1012612	-643.1940820	-643.2437755	-0.0515678	#N/A	#N/A	#N/A	#N/A
13	rd_1m5dhmU_f1_15	-643.2949959	0.1014028	-643.1935930	-643.2373845	-0.0576113	#N/A	#N/A	#N/A	#N/A
14	rd_1m5dhmU_a_6	-643.2952647	0.1021976	-643.1930670	#N/A	#N/A	#N/A	#N/A	#N/A	#N/A
15	rd_1m5dhmU_f1_6	-643.2953790	0.1029900	-643.1923890	-643.2362708	-0.0591082	#N/A	#N/A	#N/A	#N/A
16	rd_1m5dhmU_a_10	-643.2952721	0.1034820	-643.1917900	#N/A	#N/A	#N/A	#N/A	#N/A	#N/A
17	rd_1m5dhmU_f1_4	-643.2928410	0.1014220	-643.1914190	-643.2216528	-0.0711883	#N/A	#N/A	#N/A	#N/A
18	rd_1m5dhmU_e1_11	-643.2893861	0.1013581	-643.1880280	-643.2510580	-0.0383281	#N/A	#N/A	#N/A	#N/A
19	rd_1m5dhmU_e2_7	-643.2884537	0.1009967	-643.1874570	-643.2481678	-0.0402859	#N/A	#N/A	#N/A	#N/A
20	rd_1m5dhmU_e_7	-643.2894566	0.1025836	-643.1868730	-643.2486819	-0.0407747	#N/A	#N/A	#N/A	#N/A
1	rd_1m5caU01	-642.1087407	0.0781896	-642.0305510	-642.0681205	-0.0406202	-641.0152696	-641.2092625	-641.3289513	-641.3695715
2	rd_1m5caU02	-642.1061679	0.0788068	-642.0273610	-642.0734951	-0.0326728	#N/A	#N/A	#N/A	#N/A
3	rd_1m5caU10	-642.0951089	0.0774119	-642.0176970	-642.0507294	-0.0443795	-641.0062247	-641.1989736	-641.3173261	-641.3617056
4	rd_1m5caU14	-642.0935614	0.0775123	-642.0160490	-642.0523768	-0.0411846	-641.0027856	-641.1940660	-641.3116926	-641.3528772
5	rd_1m5caU04	-642.0930781	0.0784691	-642.0146090	-642.0429650	-0.0501132	-641.0009069	-641.1933480	-641.3115652	-641.3616784
6	rd_1m5caU09	-642.0921906	0.0782436	-642.0139470	-642.0393680	-0.0528226	-640.9975333	-641.1897589	-641.3083342	-641.3611568
7	rd_1m5caU12	-642.0894922	0.0778932	-642.0115990	-642.0292352	-0.0602571	-640.9883295	-641.1809713	-641.2992332	-641.3594903

8	rd_1m5caU03	-642.0895300	0.0779649	-642.0115650	-642.0284769	-0.0610531	-640.9876264	-641.1801479	-641.2984481	-641.3595012
9	rd_1m5caU08	-642.0883119	0.0785489	-642.0097630	-642.0505590	-0.0377529	-640.9819369	-641.1730148	-641.2906323	-641.3283852
10	rd_1m5caU13	-642.0890044	0.0794693	-642.0095350	-642.0440802	-0.0449242	#N/A	#N/A	#N/A	#N/A
11	rd_1m5caU06	-642.0874173	0.0783362	-642.0090810	-642.0541880	-0.0332293	-640.9535830	-641.1475391	-641.2665761	-641.2998054
12	rd_1m5caU07	-642.0843133	0.0789982	-642.0053150	-642.0360510	-0.0482622	-640.9627734	-641.1540792	-641.2716729	-641.3199351
13	rd_1m5caU15	-642.0842127	0.0793046	-642.0049080	-642.0266844	-0.0575283	-640.9809506	-641.1724324	-641.2902567	-641.3477850
14	rd_1m5caU17	-642.0800725	0.0781635	-642.0019090	-642.0165202	-0.0635523	-640.9732142	-641.1645185	-641.2820600	-641.3456123

Table S4. Energies calculated at the (U)B3LYP-D3 and DLPNO-CCSD(T) level of theory for the systems **with** the addition of explicit water. (Quasi-Harmonic Approximation)

№ conf	Molecule				(U)B3LYP-D3/6-31+G(d,p)//		DLPNO-CCSD(T)/BASIS//			
		SMD/(U)B3LYP-D3/6-31+G(d,p)			SMD/(U)B3LYP-D3/6-31+G(d,p)		SMD/(U)B3LYP-D3/6-31+G(d,p)			
		SMD/DFT			DFT		cc-pVTZ	cc-pVQZ	CBS	
		solution			gas		gas	gas	gas	solution (+Esolv)
		1atm	1atm	1atm	1atm	1atm	1atm	1atm	1atm	1atm
		Etot sol	Gcorr	G298 sol	Etot	Esolv	Etot	Etot	Etot	G298 sol
1	C68	-471.4627920	0.0847599	-471.3780320	-471.4191061	-0.0436859	-470.6364206	-470.7832459	-470.8733697	-470.9170556
2	C16	-471.4628362	0.0857212	-471.3771150	-471.4196293	-0.0432069	-470.6369867	-470.7838727	-470.8740274	-470.9172343
3	Caap	-471.4619817	0.0849567	-471.3770250	-471.4154858	-0.0464959	-470.6337342	-470.7808371	-470.8710517	-470.9175476
4	C67	-471.4622126	0.0852025	-471.3770100	-471.4156935	-0.0465191	-470.6343412	-470.7811742	-470.8712842	-470.9178033
5	C18	-471.4598650	0.0828990	-471.3769660	-471.4093494	-0.0505156	-470.6302157	-470.7762044	-470.8658984	-470.9164140
6	Cabf	-471.4627860	0.0860730	-471.3767130	-471.4192133	-0.0435727	-470.6365037	-470.7833640	-470.8735068	-470.9170795
7	Cabb	-471.4627532	0.0861001	-471.3766530	-471.4188884	-0.0438647	-470.6369346	-470.7836222	-470.8736840	-470.9175487
8	C04	-471.4627850	0.0861700	-471.3766150	-471.4193679	-0.0434171	-470.6370695	-470.7838708	-470.8739866	-470.9174037
9	C78	-471.4627794	0.0862654	-471.3765140	-471.4110691	-0.0517103	-470.6304451	-470.7770969	-470.8671166	-470.9188269
10	C62	-471.4622100	0.0859440	-471.3762660	-471.4158669	-0.0463431	#N/A	#N/A	#N/A	#N/A
11	Caba	-471.4598658	0.0836137	-471.3762520	-471.4096443	-0.0502215	#N/A	#N/A	#N/A	#N/A
12	Cabk	-471.4627085	0.0864674	-471.3762410	-471.4179237	-0.0447848	#N/A	#N/A	#N/A	#N/A
13	Cack	-471.4626812	0.0865161	-471.3761650	-471.4177372	-0.0449440	#N/A	#N/A	#N/A	#N/A
14	C08	-471.4626789	0.0866159	-471.3760630	-471.4186930	-0.0439860	-470.6383688	-470.7848469	-470.8747929	-470.9187789
15	C10	-471.4622152	0.0862471	-471.3759680	-471.4155165	-0.0466987	-470.6342248	-470.7810776	-470.8711963	-470.9178950
16	C52	-471.4619904	0.0860744	-471.3759160	-471.4150857	-0.0469047	#N/A	#N/A	#N/A	#N/A
17	Cabc	-471.4619787	0.0861206	-471.3758580	-471.4152547	-0.0467240	#N/A	#N/A	#N/A	#N/A
18	Cacr	-471.4627991	0.0869670	-471.3758320	-471.4107838	-0.0520153	-470.6307532	-470.7775677	-470.8676365	-470.9196518
19	Cadt	-471.4628015	0.0871024	-471.3756990	-471.4121532	-0.0506483	-470.6316533	-470.7781704	-470.8681321	-470.9187804
20	C58	-471.4627998	0.0871597	-471.3756400	-471.4111956	-0.0516042	-470.6312109	-470.7779519	-470.8679850	-470.9195892
1	5mCaat	-510.7862609	0.1108989	-510.6753620	-510.7399613	-0.0462997	-509.8777861	-510.0362036	-510.1336040	-510.1799037
2	5mCacl	-510.7869838	0.1124767	-510.6745070	-510.7433014	-0.0436824	-509.8797527	-510.0382508	-510.1357092	-510.1793916
3	5mCabg	-510.7867342	0.1122601	-510.6744740	-510.7421517	-0.0445825	-509.8809022	-510.0391717	-510.1365072	-510.1810897
4	5mCaaw	-510.7861052	0.1117702	-510.6743350	-510.7394952	-0.0466101	-509.8773213	-510.0359128	-510.1333893	-510.1799994
5	5mCadq	-510.7867135	0.1125085	-510.6742050	-510.7429442	-0.0437694	#N/A	#N/A	#N/A	#N/A
6	5mCabr	-510.7868299	0.1127748	-510.6740550	-510.7372527	-0.0495771	-509.8765826	-510.0348474	-510.1321602	-510.1817373
7	5mCabn	-510.7862494	0.1122113	-510.6740380	-510.7397916	-0.0464578	#N/A	#N/A	#N/A	#N/A
8	5mCabm	-510.7867840	0.1130609	-510.6737230	-510.7338591	-0.0529248	#N/A	#N/A	#N/A	#N/A
9	5mCaaj	-510.7839009	0.1103209	-510.6735800	-510.7325112	-0.0513897	#N/A	#N/A	#N/A	#N/A
10	5mCacf	-510.7860482	0.1126171	-510.6734310	-510.7396058	-0.0464424	#N/A	#N/A	#N/A	#N/A
11	5mCaao	-510.7868024	0.1134334	-510.6733690	-510.7339870	-0.0528154	-509.8724265	-510.0307463	-510.1280722	-510.1808876
12	5mCaba	-510.7839788	0.1106227	-510.6733560	-510.7351808	-0.0487980	#N/A	#N/A	#N/A	#N/A
13	5mCacw	-510.7860230	0.1126969	-510.6733260	-510.7395656	-0.0464574	#N/A	#N/A	#N/A	#N/A
14	5mCaan	-510.7868537	0.1136006	-510.6732530	-510.7354774	-0.0513763	#N/A	#N/A	#N/A	#N/A
15	5mCabx	-510.7829411	0.1102251	-510.6727160	-510.7285709	-0.0543702	#N/A	#N/A	#N/A	#N/A
16	5mCacs	-510.7836075	0.1110635	-510.6725440	-510.7287651	-0.0548424	#N/A	#N/A	#N/A	#N/A
17	5mCabz	-510.7840749	0.1116068	-510.6724680	-510.7317220	-0.0523529	#N/A	#N/A	#N/A	#N/A
18	5mCacm	-510.7842172	0.1118141	-510.6724030	-510.7390781	-0.0451390	#N/A	#N/A	#N/A	#N/A
19	5mCaak	-510.7841177	0.1117336	-510.6723840	-510.7314424	-0.0526753	#N/A	#N/A	#N/A	#N/A
20	5mCadk	-510.7841326	0.1118405	-510.6722920	-510.7381733	-0.0459593	#N/A	#N/A	#N/A	#N/A
1	5hmCaai	-586.0126891	0.1157400	-585.8969490	-585.9625867	-0.0501024	-585.0045488	-585.1870539	-585.2992435	-585.3493459

2	5hmCaab	-586.0102422	0.1142191	-585.8960230	-585.9585631	-0.0516791	-585.0006725	-585.1828454	-585.2948795	-585.3465586
3	5hmCadr	-586.0128295	0.1169794	-585.8958500	-585.9637934	-0.0490361	-585.0031624	-585.1860754	-585.2984702	-585.3475063
4	5hmCact	-586.0111653	0.1153512	-585.8958140	-585.9539713	-0.0571939	-584.9955165	-585.1779621	-585.2900635	-585.3472574
5	5hmCada	-586.0122589	0.1165049	-585.8957540	-585.9600192	-0.0522398	#N/A	#N/A	#N/A	#N/A
6	5hmCabv	-586.0123014	0.1165953	-585.8957060	-585.9602689	-0.0520325	#N/A	#N/A	#N/A	#N/A
7	5hmCaay	-586.0111604	0.1154903	-585.8956700	-585.9544356	-0.0567248	#N/A	#N/A	#N/A	#N/A
8	5hmCadc	-586.0123485	0.1167335	-585.8956150	-585.9578760	-0.0544725	#N/A	#N/A	#N/A	#N/A
9	5hmCaax	-586.0100454	0.1144794	-585.8955660	-585.9551321	-0.0549133	#N/A	#N/A	#N/A	#N/A
10	5hmCadq	-586.0110621	0.1155390	-585.8955230	-585.9550481	-0.0560140	#N/A	#N/A	#N/A	#N/A
11	5hmCabx	-586.0121164	0.1165954	-585.8955210	-585.9564251	-0.0556913	#N/A	#N/A	#N/A	#N/A
12	5hmCaap	-586.0111654	0.1156454	-585.8955200	-585.9539992	-0.0571663	#N/A	#N/A	#N/A	#N/A
13	5hmCace	-586.0120506	0.1166055	-585.8954450	-585.9601591	-0.0518915	#N/A	#N/A	#N/A	#N/A
14	5hmCacj	-586.0124067	0.1169717	-585.8954350	-585.9596797	-0.0527270	#N/A	#N/A	#N/A	#N/A
15	5hmCacg	-586.0111870	0.1157970	-585.8953900	-585.9535610	-0.0576260	#N/A	#N/A	#N/A	#N/A
16	5hmCacf	-586.0111949	0.1158088	-585.8953860	-585.9536008	-0.0575941	#N/A	#N/A	#N/A	#N/A
17	5hmCabk	-586.0111598	0.1158077	-585.8953520	-585.9542450	-0.0569148	#N/A	#N/A	#N/A	#N/A
18	5hmCads	-586.0100061	0.1147361	-585.8952700	-585.9540291	-0.0559770	#N/A	#N/A	#N/A	#N/A
19	5hmCaao	-586.0100851	0.1148411	-585.8952440	-585.9559132	-0.0541719	#N/A	#N/A	#N/A	#N/A
20	5hmCaae	-586.0127476	0.1175655	-585.8951820	-585.9634356	-0.0493120	-585.0034498	-585.1861821	-585.2984951	-585.3478071
1	5fCabv	-584.8029862	0.0921112	-584.7108750	-584.7558914	-0.0470948	-583.7993476	-583.9795843	-584.0901743	-584.1372691
2	5fCaak	-584.8026713	0.0923383	-584.7103330	-584.7528141	-0.0498572	-583.7965035	-583.9766013	-584.0871068	-584.1369640
3	5fCadf	-584.8039329	0.0936068	-584.7103260	-584.7595903	-0.0443426	-583.8027727	-583.9826050	-584.0930550	-584.1373976
4	5fCaaw	-584.8027480	0.0924889	-584.7102590	-584.7540945	-0.0486534	-583.7990906	-583.9782181	-584.0883113	-584.1369647
5	5fCacr	-584.8026191	0.0924311	-584.7101880	-584.7517222	-0.0508969	#N/A	#N/A	#N/A	#N/A
6	5fCadg	-584.8029319	0.0927448	-584.7101870	-584.7597334	-0.0431985	-583.8038535	-583.9830862	-584.0932659	-584.1364644
7	5fCadj	-584.8025653	0.0925962	-584.7099690	-584.7544571	-0.0481082	#N/A	#N/A	#N/A	#N/A
8	5fCacu	-584.8025525	0.0926595	-584.7098930	-584.7546214	-0.0479311	#N/A	#N/A	#N/A	#N/A
9	5fCaci	-584.8030016	0.0933786	-584.7096230	-584.7596927	-0.0433089	#N/A	#N/A	#N/A	#N/A
10	5fCabu	-584.8037256	0.0941265	-584.7095990	-584.7590968	-0.0446288	-583.8034219	-583.9833465	-584.0938260	-584.1384548
11	5fCabt	-584.8029681	0.0934510	-584.7095170	-584.7592400	-0.0437280	#N/A	#N/A	#N/A	#N/A
12	5fCaba	-584.8028486	0.0934486	-584.7094000	-584.7571301	-0.0457185	#N/A	#N/A	#N/A	#N/A
13	5fCaca	-584.8027643	0.0934652	-584.7092990	-584.7544055	-0.0483588	#N/A	#N/A	#N/A	#N/A
14	5fCaar	-584.8030372	0.0938471	-584.7091900	-584.7559977	-0.0470394	#N/A	#N/A	#N/A	#N/A
15	5fCaah	-584.8029117	0.0937667	-584.7091450	-584.7588208	-0.0440909	#N/A	#N/A	#N/A	#N/A
16	5fCabr	-584.8033768	0.0944147	-584.7089620	-584.7566645	-0.0467122	#N/A	#N/A	#N/A	#N/A
17	5fCadk	-584.8026814	0.0937653	-584.7089160	-584.7532039	-0.0494775	#N/A	#N/A	#N/A	#N/A
18	5fCaaf	-584.8035837	0.0948257	-584.7087580	-584.7635989	-0.0399848	-583.8083328	-583.9877056	-584.0979636	-584.1379484
19	5fCacq	-584.8033469	0.0946098	-584.7087370	-584.7565481	-0.0467988	#N/A	#N/A	#N/A	#N/A
20	5fCads	-584.8007297	0.0920376	-584.7086920	-584.7541636	-0.0465661	#N/A	#N/A	#N/A	#N/A
1	5dhmCaaa	-661.2510826	0.1186725	-661.1324100	-661.1955332	-0.0555494	-660.1421187	-660.3479350	-660.4746925	-660.5302419
2	5dhmCaai	-661.2505665	0.1193514	-661.1312150	-661.1980403	-0.0525262	-660.1429093	-660.3494219	-660.4764518	-660.5289780
3	5dhmCact	-661.2506461	0.1194620	-661.1311840	-661.1990566	-0.0515895	-660.1427075	-660.3495868	-660.4767924	-660.5283819
4	5dhmCabk	-661.2504767	0.1193827	-661.1310940	-661.1978045	-0.0526722	-660.1438571	-660.3503950	-660.4774146	-660.5300868
5	5dhmCado	-661.2510207	0.1200416	-661.1309790	-661.1954483	-0.0555724	#N/A	#N/A	#N/A	#N/A
6	5dhmCaco	-661.2506036	0.1198716	-661.1307320	-661.1994189	-0.0511848	#N/A	#N/A	#N/A	#N/A
7	5dhmCaaj	-661.2480746	0.1173826	-661.1306920	-661.1898843	-0.0581903	#N/A	#N/A	#N/A	#N/A
8	5dhmCaao	-661.2504672	0.1198342	-661.1306330	-661.1980113	-0.0524560	#N/A	#N/A	#N/A	#N/A
9	5dhmCaat	-661.2504687	0.1198657	-661.1306030	-661.1971481	-0.0533206	#N/A	#N/A	#N/A	#N/A

10	5dhmCacf	-661.2510552	0.1204592	-661.1305960	-661.1954445	-0.0556107	-660.1420469	-660.3478393	-660.4745720	-660.5301827
11	5dhmCaas	-661.2479160	0.1174569	-661.1304590	-661.1876310	-0.0602849	#N/A	#N/A	#N/A	#N/A
12	5dhmCaax	-661.2489348	0.1185677	-661.1303670	-661.1890472	-0.0598876	#N/A	#N/A	#N/A	#N/A
13	5dhmCadg	-661.2510232	0.1207282	-661.1302950	-661.1956168	-0.0554065	-660.1420537	-660.3479498	-660.4747412	-660.5301477
14	5dhmCabw	-661.2499865	0.1197015	-661.1302850	-661.1950582	-0.0549283	#N/A	#N/A	#N/A	#N/A
15	5dhmCaab	-661.2500049	0.1197519	-661.1302530	-661.1950581	-0.0549469	#N/A	#N/A	#N/A	#N/A
16	5dhmCads	-661.2496969	0.1194918	-661.1302050	-661.1887516	-0.0609453	#N/A	#N/A	#N/A	#N/A
17	5dhmCace	-661.2500080	0.1200790	-661.1299290	-661.1952746	-0.0547335	#N/A	#N/A	#N/A	#N/A
18	5dhmCaaz	-661.2480928	0.1181898	-661.1299030	-661.1908989	-0.0571939	#N/A	#N/A	#N/A	#N/A
19	5dhmCaba	-661.2510843	0.1212322	-661.1298520	-661.1952663	-0.0558180	#N/A	#N/A	#N/A	#N/A
20	5dhmCacb	-661.2498551	0.1202101	-661.1296450	-661.1937848	-0.0560704	#N/A	#N/A	#N/A	#N/A
1	5caCacg	-660.0645097	0.0976477	-659.9668620	-659.9937638	-0.0707460	-658.9381189	-659.1430471	-659.2689905	-659.3397365
2	5caCaaa	-660.0642990	0.0974690	-659.9668300	-659.9888454	-0.0754536	-658.9335424	-659.1388013	-659.2648647	-659.3403183
3	5caCaay	-660.0642325	0.0974284	-659.9668040	-659.9910263	-0.0732061	-658.9357023	-659.1408653	-659.2668945	-659.3401006
4	5caCadk	-660.0642333	0.0974342	-659.9667990	-659.9903178	-0.0739155	-658.9349037	-659.1401074	-659.2661536	-659.3400691
5	5caCaap	-660.0645254	0.0979483	-659.9665770	-659.9938989	-0.0706264	-658.9382506	-659.1431631	-659.2690975	-659.3397239
6	5caCaae	-660.0637361	0.0971820	-659.9665540	-659.9882861	-0.0754500	#N/A	#N/A	#N/A	#N/A
7	5caCadn	-660.0632630	0.0967859	-659.9664770	-659.9893581	-0.0739049	#N/A	#N/A	#N/A	#N/A
8	5caCabn	-660.0631582	0.0966871	-659.9664710	-659.9880029	-0.0751553	#N/A	#N/A	#N/A	#N/A
9	5caCadu	-660.0643587	0.0979456	-659.9664130	-659.9868265	-0.0775322	-658.9316316	-659.1368982	-659.2629477	-659.3404799
10	5caCabf	-660.0641677	0.0979047	-659.9662630	-659.9902312	-0.0739365	#N/A	#N/A	#N/A	#N/A
11	5caCaas	-660.0641136	0.0979836	-659.9661300	-659.9908547	-0.0732590	#N/A	#N/A	#N/A	#N/A
12	5caCact	-660.0631390	0.0970659	-659.9660730	-659.9876169	-0.0755220	#N/A	#N/A	#N/A	#N/A
13	5caCacj	-660.0644403	0.0983823	-659.9660580	-659.9849290	-0.0795113	#N/A	#N/A	#N/A	#N/A
14	5caCaaw	-660.0632691	0.0972220	-659.9660470	-659.9892703	-0.0739988	#N/A	#N/A	#N/A	#N/A
15	5caCadp	-660.0631316	0.0970876	-659.9660440	-659.9872640	-0.0758677	#N/A	#N/A	#N/A	#N/A
16	5caCabz	-660.0640715	0.0980644	-659.9660070	-659.9906151	-0.0734564	#N/A	#N/A	#N/A	#N/A
17	5caCado	-660.0628387	0.0968756	-659.9659630	-659.9902796	-0.0725590	#N/A	#N/A	#N/A	#N/A
18	5caCabb	-660.0626507	0.0969656	-659.9656850	-659.9854029	-0.0772478	#N/A	#N/A	#N/A	#N/A
19	5caCack	-660.0636305	0.0980335	-659.9655970	-659.9870614	-0.0765691	#N/A	#N/A	#N/A	#N/A
20	5caCacq	-660.0636520	0.0981339	-659.9655180	-659.9862658	-0.0773862	#N/A	#N/A	#N/A	#N/A
1	1mCabu	-510.7741720	0.1103319	-510.6638400	-510.7318180	-0.0423540	-509.8697866	-510.0278079	-510.1249659	-510.1673199
2	1mCacx	-510.7746889	0.1115808	-510.6631080	-510.7351610	-0.0395279	-509.8718266	-510.0298266	-510.1269851	-510.1665130
3	1mCabw	-510.7741718	0.1113608	-510.6628110	-510.7316429	-0.0425289	-509.8696932	-510.0277114	-510.1248660	-510.1673949
4	1mCabs	-510.7738889	0.1115909	-510.6622980	-510.7307690	-0.0431199	-509.8686116	-510.0268282	-510.1240481	-510.1671680
5	1mCaaa	-510.7746878	0.1124028	-510.6622850	-510.7354807	-0.0392071	-509.8721197	-510.0301537	-510.1273277	-510.1665348
6	1mCabq	-510.7744479	0.1122749	-510.6621730	-510.7318811	-0.0425668	#N/A	#N/A	#N/A	#N/A
7	1mCaby	-510.7746754	0.1125263	-510.6621490	-510.7350482	-0.0396272	-509.8717811	-510.0297893	-510.1269523	-510.1665795
8	1mCacg	-510.7745702	0.1127632	-510.6618070	-510.7333159	-0.0412543	-509.8714297	-510.0290208	-510.1259811	-510.1672354
9	1mCadu	-510.7745810	0.1127940	-510.6617870	-510.7341802	-0.0404008	#N/A	#N/A	#N/A	#N/A
10	1mCact	-510.7741185	0.1127394	-510.6613790	-510.7273090	-0.0468095	#N/A	#N/A	#N/A	#N/A
11	1mCaac	-510.7742102	0.1129982	-510.6612120	-510.7273845	-0.0468257	#N/A	#N/A	#N/A	#N/A
12	1mCaae	-510.7741072	0.1132932	-510.6608140	-510.7268038	-0.0473034	#N/A	#N/A	#N/A	#N/A
13	1mCacd	-510.7741260	0.1133909	-510.6607350	-510.7269482	-0.0471778	#N/A	#N/A	#N/A	#N/A
14	1mCadf	-510.7737966	0.1130895	-510.6607070	-510.7328267	-0.0409698	#N/A	#N/A	#N/A	#N/A
15	1mCadv	-510.7688479	0.1084899	-510.6603580	-510.7174913	-0.0513566	#N/A	#N/A	#N/A	#N/A
16	1mCabx	-510.7704287	0.1102507	-510.6601780	-510.7204640	-0.0499648	#N/A	#N/A	#N/A	#N/A
17	1mCaaj	-510.7747655	0.1146694	-510.6600960	-510.7264800	-0.0482855	-509.8651412	-510.0229304	-510.1199484	-510.1682339

18	1mCabz	-510.7688437	0.1087646	-510.6600790	-510.7212843	-0.0475594	#N/A	#N/A	#N/A	#N/A
19	1mCadl	-510.7693031	0.1092300	-510.6600730	-510.7203149	-0.0489881	#N/A	#N/A	#N/A	#N/A
20	1mCadr	-510.7711022	0.1111941	-510.6599080	-510.7255012	-0.0456010	#N/A	#N/A	#N/A	#N/A
1	1m5mCaaq	-550.0990586	0.1372456	-549.9618130	-550.0592683	-0.0397903	-549.1152324	-549.2848374	-549.3892891	-549.4290794
2	1m5mCabe	-550.0979061	0.1367440	-549.9611620	-550.0552671	-0.0426390	-549.1123413	-549.2821673	-549.3866910	-549.4293300
3	1m5mCaaa	-550.0981809	0.1381368	-549.9600440	-550.0556413	-0.0425396	-549.1129201	-549.2825288	-549.3869636	-549.4295032
4	1m5mCabl	-550.0978979	0.1380738	-549.9598240	-550.0549403	-0.0429576	-549.1122078	-549.2819444	-549.3864170	-549.4293746
5	1m5mCaai	-550.0978297	0.1383416	-549.9594880	-550.0568379	-0.0409918	#N/A	#N/A	#N/A	#N/A
6	1m5mCacy	-550.0982182	0.1387501	-549.9594680	-550.0513160	-0.0469022	-549.1100689	-549.2794823	-549.3838139	-549.4307161
7	1m5mCaca	-550.0976699	0.1382288	-549.9594410	-550.0553590	-0.0423108	#N/A	#N/A	#N/A	#N/A
8	1m5mCadr	-550.0982191	0.1389700	-549.9592490	-550.0510760	-0.0471431	-549.1098162	-549.2792613	-549.3836067	-549.4307498
9	1m5mCabp	-550.0949740	0.1359350	-549.9590390	-550.0492618	-0.0457122	#N/A	#N/A	#N/A	#N/A
10	1m5mCadg	-550.0950760	0.1362920	-549.9587840	-550.0444657	-0.0506103	#N/A	#N/A	#N/A	#N/A
11	1m5mCaan	-550.0945136	0.1358286	-549.9586850	-550.0450114	-0.0495022	#N/A	#N/A	#N/A	#N/A
12	1m5mCaae	-550.0983642	0.1400141	-549.9583500	-550.0514405	-0.0469236	#N/A	#N/A	#N/A	#N/A
13	1m5mCaap	-550.0989194	0.1406694	-549.9582500	-550.0507208	-0.0481986	-549.1087962	-549.2781525	-549.3824513	-549.4306499
14	1m5mCabq	-550.0953702	0.1373802	-549.9579900	-550.0454124	-0.0499578	#N/A	#N/A	#N/A	#N/A
15	1m5mCacb	-550.0942071	0.1362320	-549.9579750	-550.0533551	-0.0408520	#N/A	#N/A	#N/A	#N/A
16	1m5mCadm	-550.0945089	0.1365638	-549.9579450	-550.0451531	-0.0493558	#N/A	#N/A	#N/A	#N/A
17	1m5mCabk	-550.0941718	0.1362657	-549.9579060	-550.0529738	-0.0411980	#N/A	#N/A	#N/A	#N/A
18	1m5mCaaw	-550.0954046	0.1376735	-549.9577310	-550.0457765	-0.0496280	#N/A	#N/A	#N/A	#N/A
19	1m5mCaba	-550.0958620	0.1382159	-549.9576460	-550.0480809	-0.0477811	#N/A	#N/A	#N/A	#N/A
20	1m5mCacj	-550.0957282	0.1383522	-549.9573760	-550.0471901	-0.0485381	#N/A	#N/A	#N/A	#N/A
1	1m5hmCacd	-625.3238187	0.1417886	-625.1820300	-625.2778775	-0.0459412	-624.2379480	-624.4320271	-624.5514461	-624.5973873
2	1m5hmCaas	-625.3249316	0.1429035	-625.1820280	-625.2797531	-0.0451785	-624.2389290	-624.4328837	-624.5522514	-624.5974299
3	1m5hmCacv	-625.3240563	0.1421982	-625.1818580	-625.2722012	-0.0518551	-624.2339819	-624.4277034	-624.5469234	-624.5987785
4	1m5hmCadl	-625.3236435	0.1423144	-625.1813290	-625.2763248	-0.0473187	-624.2374439	-624.4311100	-624.5503306	-624.5976493
5	1m5hmCacn	-625.3239721	0.1427240	-625.1812480	-625.2781015	-0.0458706	#N/A	#N/A	#N/A	#N/A
6	1m5hmCacc	-625.3240209	0.1427858	-625.1812350	-625.2716131	-0.0524078	#N/A	#N/A	#N/A	#N/A
7	1m5hmCacx	-625.3242359	0.1432998	-625.1809360	-625.2721400	-0.0520959	#N/A	#N/A	#N/A	#N/A
8	1m5hmCacq	-625.3242412	0.1433142	-625.1809270	-625.2717900	-0.0524512	#N/A	#N/A	#N/A	#N/A
9	1m5hmCabl	-625.3231781	0.1422671	-625.1809110	-625.2700763	-0.0531018	#N/A	#N/A	#N/A	#N/A
10	1m5hmCabd	-625.3238813	0.1429752	-625.1809060	-625.2784997	-0.0453815	#N/A	#N/A	#N/A	#N/A
11	1m5hmCabp	-625.3238748	0.1431048	-625.1807700	-625.2783998	-0.0454750	#N/A	#N/A	#N/A	#N/A
12	1m5hmCaci	-625.3249832	0.1443211	-625.1806620	-625.2708184	-0.0541648	#N/A	#N/A	#N/A	#N/A
13	1m5hmCabi	-625.3242451	0.1435891	-625.1806560	-625.2723127	-0.0519324	#N/A	#N/A	#N/A	#N/A
14	1m5hmCabw	-625.3242471	0.1436561	-625.1805910	-625.2766304	-0.0476167	#N/A	#N/A	#N/A	#N/A
15	1m5hmCacu	-625.3245094	0.1439424	-625.1805670	-625.2722583	-0.0522512	-624.2345257	-624.4281734	-624.5473683	-624.5996195
16	1m5hmCaac	-625.3244257	0.1438966	-625.1805290	-625.2736309	-0.0507947	#N/A	#N/A	#N/A	#N/A
17	1m5hmCace	-625.3232286	0.1427036	-625.1805250	-625.2701010	-0.0531277	#N/A	#N/A	#N/A	#N/A
18	1m5hmCaan	-625.3221283	0.1416093	-625.1805190	-625.2685715	-0.0535568	-624.2305666	-624.4239278	-624.5429627	-624.5965195
19	1m5hmCabz	-625.3243405	0.1438875	-625.1804530	-625.2765438	-0.0477968	#N/A	#N/A	#N/A	#N/A
20	1m5hmCabx	-625.3244547	0.1440347	-625.1804200	-625.2718376	-0.0526172	#N/A	#N/A	#N/A	#N/A
1	1m5fCaav	-624.1168279	0.1200718	-623.9967560	-624.0767589	-0.0400689	-623.0383287	-623.2295403	-623.3471150	-623.3871840
2	1m5fCabo	-624.1140144	0.1175893	-623.9964250	-624.0638173	-0.0501971	-623.0280490	-623.2187530	-623.3360404	-623.3862375
3	1m5fCadj	-624.1130370	0.1167840	-623.9962530	-624.0654886	-0.0475484	-623.0292629	-623.2205114	-623.3380230	-623.3855714
4	1m5fCacr	-624.1159108	0.1198038	-623.9961070	-624.0714869	-0.0444239	-623.0338056	-623.2252922	-623.3429465	-623.3873704
5	1m5fCace	-624.1160910	0.1200179	-623.9960730	-624.0737104	-0.0423806	-623.0363587	-623.2277225	-623.3453467	-623.3877273

6	1m5fCabm	-624.1167768	0.1207238	-623.9960530	-624.0737444	-0.0430324	-623.0372151	-623.2279823	-623.3453402	-623.3883726
7	1m5fCabb	-624.1158005	0.1198334	-623.9959670	-624.0751907	-0.0406098	#N/A	#N/A	#N/A	#N/A
8	1m5fCaaq	-624.1158923	0.1200672	-623.9958250	-624.0689274	-0.0469649	#N/A	#N/A	#N/A	#N/A
9	1m5fCadc	-624.1159010	0.1202150	-623.9956860	-624.0702242	-0.0456768	#N/A	#N/A	#N/A	#N/A
10	1m5fCaac	-624.1157951	0.1201160	-623.9956790	-624.0750568	-0.0407383	#N/A	#N/A	#N/A	#N/A
11	1m5fCacl	-624.1156279	0.1199968	-623.9956310	-624.0747624	-0.0408655	#N/A	#N/A	#N/A	#N/A
12	1m5fCaas	-624.1159069	0.1204189	-623.9954880	-624.0713487	-0.0445583	#N/A	#N/A	#N/A	#N/A
13	1m5fCaad	-624.1135806	0.1181385	-623.9954420	-624.0728982	-0.0406823	#N/A	#N/A	#N/A	#N/A
14	1m5fCaco	-624.1158982	0.1204631	-623.9954350	-624.0711853	-0.0447129	#N/A	#N/A	#N/A	#N/A
15	1m5fCaau	-624.1134717	0.1180887	-623.9953830	-624.0613169	-0.0521548	#N/A	#N/A	#N/A	#N/A
16	1m5fCadt	-624.1160941	0.1207650	-623.9953290	-624.0738256	-0.0422685	-623.0364936	-623.2278365	-623.3454503	-623.3877188
17	1m5fCaae	-624.1126621	0.1173871	-623.9952750	-624.0631233	-0.0495389	#N/A	#N/A	#N/A	#N/A
18	1m5fCabk	-624.1141362	0.1188692	-623.9952670	-624.0694036	-0.0447326	#N/A	#N/A	#N/A	#N/A
19	1m5fCaam	-624.1161872	0.1210271	-623.9951600	-624.0688845	-0.0473027	-623.0331252	-623.2242085	-623.3416773	-623.3889800
20	1m5fCabx	-624.1113308	0.1163827	-623.9949480	-624.0632083	-0.0481224	#N/A	#N/A	#N/A	#N/A
1	1m5dhmCacv	-700.5619380	0.1447449	-700.4171930	-700.5075038	-0.0544342	-699.3729111	-699.5903995	-699.7243516	-699.7787858
2	1m5dhmCadr	-700.5628304	0.1456924	-700.4171380	-700.5152155	-0.0476149	-699.3783299	-699.5963685	-699.7305968	-699.7782117
3	1m5dhmCadg	-700.5631847	0.1461166	-700.4170680	-700.5105280	-0.0526567	-699.3770909	-699.5937405	-699.7273700	-699.7800267
4	1m5dhmCacu	-700.5628243	0.1457662	-700.4170580	-700.5152121	-0.0476122	-699.3783501	-699.5963828	-699.7306086	-699.7782208
5	1m5dhmCaaf	-700.5630552	0.1462532	-700.4168020	-700.5111432	-0.0519121	-699.3772593	-699.5942630	-699.7280563	-699.7799684
6	1m5dhmCabt	-700.5620804	0.1454004	-700.4166800	-700.5071592	-0.0549212	#N/A	#N/A	#N/A	#N/A
7	1m5dhmCacb	-700.5616106	0.1451506	-700.4164600	-700.5104255	-0.0511852	#N/A	#N/A	#N/A	#N/A
8	1m5dhmCabn	-700.5615408	0.1451108	-700.4164300	-700.5083704	-0.0531704	#N/A	#N/A	#N/A	#N/A
9	1m5dhmCaad	-700.5622153	0.1458293	-700.4163860	-700.5113264	-0.0508889	#N/A	#N/A	#N/A	#N/A
10	1m5dhmCaaw	-700.5615269	0.1453198	-700.4162070	-700.5121240	-0.0494029	#N/A	#N/A	#N/A	#N/A
11	1m5dhmCadq	-700.5617016	0.1455905	-700.4161110	-700.5122124	-0.0494891	#N/A	#N/A	#N/A	#N/A
12	1m5dhmCach	-700.5622562	0.1461951	-700.4160610	-700.5115026	-0.0507536	#N/A	#N/A	#N/A	#N/A
13	1m5dhmCaco	-700.5616945	0.1456805	-700.4160140	-700.5087220	-0.0529726	#N/A	#N/A	#N/A	#N/A
14	1m5dhmCadi	-700.5615979	0.1456358	-700.4159620	-700.5101864	-0.0514115	#N/A	#N/A	#N/A	#N/A
15	1m5dhmCabx	-700.5615840	0.1456310	-700.4159530	-700.5104325	-0.0511516	#N/A	#N/A	#N/A	#N/A
16	1m5dhmCabi	-700.5618088	0.1458788	-700.4159300	-700.5097402	-0.0520686	#N/A	#N/A	#N/A	#N/A
17	1m5dhmCabb	-700.5628138	0.1471898	-700.4156240	-700.5073908	-0.0554230	#N/A	#N/A	#N/A	#N/A
18	1m5dhmCads	-700.5618064	0.1462464	-700.4155600	-700.5097467	-0.0520597	#N/A	#N/A	#N/A	#N/A
19	1m5dhmCaaj	-700.5620797	0.1465426	-700.4155370	-700.5069973	-0.0550824	#N/A	#N/A	#N/A	#N/A
20	1m5dhmCaah	-700.5628252	0.1474292	-700.4153960	-700.5076172	-0.0552080	-699.3740919	-699.5908874	-699.7245484	-699.7797564
1	1m5caCacv	-699.3778748	0.1231127	-699.2547620	-699.3106582	-0.0672166	-698.1740091	-698.3902273	-698.5232550	-698.5904716
2	1m5caCade	-699.3778044	0.1238954	-699.2539090	-699.3017954	-0.0760090	-698.1666051	-698.3827183	-698.5156448	-698.5916538
3	1m5caCaap	-699.3776058	0.1239688	-699.2536370	-699.3069365	-0.0706693	-698.1705104	-698.3870144	-698.5201480	-698.5908173
4	1m5caCacc	-699.3776991	0.1241720	-699.2535270	-699.3099646	-0.0677344	-698.1732216	-698.3895711	-698.5226643	-698.5903987
5	1m5caCadf	-699.3776730	0.1241589	-699.2535140	-699.3047408	-0.0729322	-698.1684393	-698.3850188	-698.5181718	-698.5911040
6	1m5caCabk	-699.3773767	0.1239836	-699.2533930	-699.3079284	-0.0694482	-698.1704592	-698.3873951	-698.5207391	-698.5901873
7	1m5caCabd	-699.3775442	0.1244052	-699.2531390	-699.3082545	-0.0692897	#N/A	#N/A	#N/A	#N/A
8	1m5caCaba	-699.3761493	0.1230693	-699.2530800	-699.3077076	-0.0684417	#N/A	#N/A	#N/A	#N/A
9	1m5caCaag	-699.3760493	0.1229992	-699.2530500	-699.3064067	-0.0696426	#N/A	#N/A	#N/A	#N/A
10	1m5caCaax	-699.3748727	0.1221507	-699.2527220	-699.3001863	-0.0746864	#N/A	#N/A	#N/A	#N/A
11	1m5caCaao	-699.3762716	0.1237175	-699.2525540	-699.3007633	-0.0755083	#N/A	#N/A	#N/A	#N/A
12	1m5caCaaz	-699.3761940	0.1238969	-699.2522970	-699.3006156	-0.0755784	#N/A	#N/A	#N/A	#N/A
13	1m5caCaas	-699.3759633	0.1240062	-699.2519570	-699.3007883	-0.0751750	#N/A	#N/A	#N/A	#N/A

14	1m5caCaai	-699.3759203	0.1240762	-699.2518440	-699.3017084	-0.0742118	#N/A	#N/A	#N/A	#N/A
15	1m5caCabj	-699.3772941	0.1263600	-699.2509340	-699.3109010	-0.0663931	#N/A	#N/A	#N/A	#N/A
16	1m5caCabu	-699.3772670	0.1263629	-699.2509040	-699.3105783	-0.0666887	#N/A	#N/A	#N/A	#N/A
17	1m5caCada	-699.3744546	0.1236355	-699.2508190	-699.2938169	-0.0806377	#N/A	#N/A	#N/A	#N/A
18	1m5caCabp	-699.3747202	0.1239651	-699.2507550	-699.2952244	-0.0794958	#N/A	#N/A	#N/A	#N/A
19	1m5caCadm	-699.3778688	0.1271287	-699.2507400	-699.3018243	-0.0760444	-698.1665374	-698.3833856	-698.5166522	-698.5926966
20	1m5caCadi	-699.3754639	0.1247328	-699.2507310	-699.2956323	-0.0798316	#N/A	#N/A	#N/A	#N/A
1	Uacb	-491.3354328	0.0738727	-491.2615600	-491.3000514	-0.0353813	-490.5112626	-490.6638566	-490.7575579	-490.7929392
2	Uaac	-491.3352650	0.0737700	-491.2614950	-491.2983628	-0.0369022	-490.5113631	-490.6634994	-490.7569733	-490.7938755
3	U114_abc	-491.3356642	0.0742262	-491.2614380	-491.2994727	-0.0361915	-490.5118652	-490.6640480	-490.7575468	-490.7937383
4	U114_abu	-491.3350633	0.0736893	-491.2613740	-491.2975928	-0.0374705	-490.5104551	-490.6620694	-490.7553103	-490.7927808
5	Uacu	-491.3350961	0.0737970	-491.2612990	-491.2990521	-0.0360440	-490.5104041	-490.6631155	-490.7568498	-490.7928938
6	U114_aaf	-491.3356736	0.0744505	-491.2612230	-491.2988574	-0.0368162	-490.5114817	-490.6636143	-490.7570885	-490.7939047
7	U114_acw	-491.3353409	0.0741488	-491.2611920	-491.3021635	-0.0331774	#N/A	#N/A	#N/A	#N/A
8	U114_abt	-491.3351421	0.0740180	-491.2611240	-491.3000442	-0.0350979	#N/A	#N/A	#N/A	#N/A
9	Uadn	-491.3358217	0.0747266	-491.2610950	-491.2955675	-0.0402542	#N/A	#N/A	#N/A	#N/A
10	Uabj	-491.3351118	0.0740308	-491.2610810	-491.2988599	-0.0362519	#N/A	#N/A	#N/A	#N/A
11	Uabg	-491.3353620	0.0742979	-491.2610640	-491.3021891	-0.0331729	#N/A	#N/A	#N/A	#N/A
12	U14_adk	-491.3353408	0.0743347	-491.2610060	-491.3022250	-0.0331158	#N/A	#N/A	#N/A	#N/A
13	U14_abl	-491.3353258	0.0745067	-491.2608190	-491.3020098	-0.0333159	#N/A	#N/A	#N/A	#N/A
14	Uabw	-491.3354589	0.0746698	-491.2607890	-491.3003045	-0.0351544	#N/A	#N/A	#N/A	#N/A
15	Uaab	-491.3354309	0.0746758	-491.2607550	-491.2992792	-0.0361517	#N/A	#N/A	#N/A	#N/A
16	U14_acu	-491.3350853	0.0744013	-491.2606840	-491.2979507	-0.0371346	#N/A	#N/A	#N/A	#N/A
17	Uabe	-491.3353413	0.0748033	-491.2605380	-491.2976887	-0.0376526	#N/A	#N/A	#N/A	#N/A
18	U14_adh	-491.3350604	0.0745884	-491.2604720	-491.2974194	-0.0376410	#N/A	#N/A	#N/A	#N/A
19	U14_abs	-491.3354817	0.0750517	-491.2604300	-491.3004288	-0.0350529	#N/A	#N/A	#N/A	#N/A
20	Uabz	-491.3355279	0.0751089	-491.2604190	-491.3000399	-0.0354880	#N/A	#N/A	#N/A	#N/A
1	5mUadr	-530.6575251	0.0965081	-530.5610170	-530.6160330	-0.0414921	-529.7482681	-529.9119876	-530.0127286	-530.0542207
2	5mUadm	-530.6599072	0.0990372	-530.5608700	-530.6253904	-0.0345169	-529.7561029	-529.9202567	-530.0212309	-530.0557478
3	5mU_aaz	-530.6599452	0.0992472	-530.5606980	-530.6277464	-0.0321988	-529.7591293	-529.9222413	-530.0227815	-530.0549803
4	5mUabf	-530.6600553	0.0995453	-530.5605100	-530.6246533	-0.0354020	#N/A	#N/A	#N/A	#N/A
5	5mU_adb	-530.6599531	0.0994791	-530.5604740	-530.6255966	-0.0343565	-529.7565829	-529.9206161	-530.0215410	-530.0558975
6	5mU_aaa	-530.6597380	0.0993260	-530.5604120	-530.6242211	-0.0355169	-529.7560415	-529.9192645	-530.0198315	-530.0553484
7	5mUadq	-530.6597010	0.0995010	-530.5602000	-530.6228444	-0.0368566	#N/A	#N/A	#N/A	#N/A
8	5mU_abq	-530.6597477	0.0995566	-530.5601910	-530.6249008	-0.0348469	#N/A	#N/A	#N/A	#N/A
9	5mU_adj	-530.6602148	0.1002387	-530.5599760	-530.6256905	-0.0345243	-529.7557020	-529.9199401	-530.0209520	-530.0554763
10	5mUabq	-530.6597996	0.0999195	-530.5598800	-530.6256156	-0.0341840	#N/A	#N/A	#N/A	#N/A
11	5mUacp	-530.6587593	0.0989193	-530.5598400	-530.6235608	-0.0351985	#N/A	#N/A	#N/A	#N/A
12	5mU_aae	-530.6597229	0.1000809	-530.5596420	-530.6232005	-0.0365224	#N/A	#N/A	#N/A	#N/A
13	5mUacq	-530.6599457	0.1004397	-530.5595060	-530.6277326	-0.0322131	#N/A	#N/A	#N/A	#N/A
14	5mU_abn	-530.6590480	0.0995830	-530.5594650	-530.6256244	-0.0334236	#N/A	#N/A	#N/A	#N/A
15	5mU_aam	-530.6590500	0.0996870	-530.5593630	-530.6254835	-0.0335665	#N/A	#N/A	#N/A	#N/A
16	5mU_aau	-530.6600182	0.1006582	-530.5593600	-530.6262948	-0.0337234	-529.7580054	-529.9217728	-530.0225735	-530.0562969
17	5mUacy	-530.6603178	0.1010077	-530.5593100	-530.6252780	-0.0350398	#N/A	#N/A	#N/A	#N/A
18	5mU_abo	-530.6599978	0.1007388	-530.5592590	-530.6272046	-0.0327933	-529.7583495	-529.9222504	-530.0231152	-530.0559085
19	5mUaac	-530.6603183	0.1010773	-530.5592410	-530.6251923	-0.0351261	-529.7565953	-529.9203721	-530.0211771	-530.0563032
20	5mUaag	-530.6599919	0.1008538	-530.5591380	-530.6259607	-0.0340312	#N/A	#N/A	#N/A	#N/A
1	5hmUabr	-605.8856799	0.1035299	-605.7821500	-605.8440003	-0.0416796	-604.8785235	-605.0664414	-605.1820492	-605.2237288

2	5hmUacj	-605.8857167	0.1036466	-605.7820700	-605.8449094	-0.0408073	-604.8792398	-605.0672069	-605.1828401	-605.2236474
3	5hmUaay	-605.8856753	0.1036153	-605.7820600	-605.8439870	-0.0416883	-604.8785223	-605.0664493	-605.1820578	-605.2237461
4	5hmUaas	-605.8861940	0.1041740	-605.7820200	-605.8445171	-0.0416770	-604.8787959	-605.0671688	-605.1829831	-605.2246601
5	5hmUabo	-605.8857441	0.1039211	-605.7818230	-605.8412566	-0.0444875	#N/A	#N/A	#N/A	#N/A
6	5hmUabv	-605.8878833	0.1061333	-605.7817500	-605.8430230	-0.0448604	-604.8770423	-605.0649200	-605.1805318	-605.2253922
7	5hmUado	-605.8858235	0.1041105	-605.7817130	-605.8446943	-0.0411292	#N/A	#N/A	#N/A	#N/A
8	5hmUacz	-605.8858967	0.1043437	-605.7815530	-605.8474966	-0.0384002	#N/A	#N/A	#N/A	#N/A
9	5hmUacu	-605.8858272	0.1043892	-605.7814380	-605.8453336	-0.0404936	#N/A	#N/A	#N/A	#N/A
10	5hmUaau	-605.8852469	0.1038178	-605.7814290	-605.8453700	-0.0398769	#N/A	#N/A	#N/A	#N/A
11	5hmUaai	-605.8889704	0.1076334	-605.7813370	-605.8499883	-0.0389821	-604.8837091	-605.0715949	-605.1872381	-605.2262202
12	5hmUaaf	-605.8866932	0.1053942	-605.7812990	-605.8471639	-0.0395293	#N/A	#N/A	#N/A	#N/A
13	5hmUaca	-605.8889919	0.1076938	-605.7812980	-605.8498972	-0.0390947	#N/A	#N/A	#N/A	#N/A
14	5hmUaan	-605.8879440	0.1066520	-605.7812920	-605.8422052	-0.0457388	-604.8765393	-605.0642129	-605.1797445	-605.2254833
15	5hmUact	-605.8866469	0.1054208	-605.7812260	-605.8401958	-0.0464511	#N/A	#N/A	#N/A	#N/A
16	5hmUaaw	-605.8857540	0.1045320	-605.7812220	-605.8435813	-0.0421728	#N/A	#N/A	#N/A	#N/A
17	5hmUabw	-605.8856945	0.1046604	-605.7810340	-605.8429908	-0.0427036	#N/A	#N/A	#N/A	#N/A
18	5hmUadu	-605.8858583	0.1048742	-605.7809840	-605.8438642	-0.0419941	#N/A	#N/A	#N/A	#N/A
19	5hmUabk	-605.8864138	0.1055298	-605.7808840	-605.8403892	-0.0460246	#N/A	#N/A	#N/A	#N/A
20	5hmUadv	-605.8857353	0.1048662	-605.7808690	-605.8422542	-0.0434811	#N/A	#N/A	#N/A	#N/A
1	5fUacj	-604.6704459	0.0785329	-604.5919130	-604.6227028	-0.0477431	-603.6620750	-603.8473766	-603.9612148	-604.0089579
2	5fUaak	-604.6705415	0.0795804	-604.5909610	-604.6214780	-0.0490635	-603.6591879	-603.8449499	-603.9589629	-604.0080264
3	5fUaae	-604.6718023	0.0808453	-604.5909570	-604.6228260	-0.0489763	-603.6621644	-603.8467598	-603.9602894	-604.0092657
4	5fUaat	-604.6719269	0.0811379	-604.5907890	-604.6254658	-0.0464611	-603.6644831	-603.8490665	-603.9626097	-604.0090708
5	5fUabr	-604.6705476	0.0798046	-604.5907430	-604.6215990	-0.0489486	#N/A	#N/A	#N/A	#N/A
6	5fUaad	-604.6720081	0.0813890	-604.5906190	-604.6263397	-0.0456684	-603.6652269	-603.8497472	-603.9632726	-604.0089410
7	5fUaam	-604.6705568	0.0799567	-604.5906000	-604.6174709	-0.0530859	#N/A	#N/A	#N/A	#N/A
8	5fUaaz	-604.6719746	0.0814296	-604.5905450	-604.6259592	-0.0460154	-603.6649106	-603.8494558	-603.9629874	-604.0090028
9	5fUaab	-604.6718248	0.0816897	-604.5901350	-604.6217300	-0.0500948	#N/A	#N/A	#N/A	#N/A
10	5fUacp	-604.6718650	0.0817380	-604.5901270	-604.6243541	-0.0475110	-603.6634574	-603.8480777	-603.9616265	-604.0091375
11	5fUaan	-604.6706003	0.0805762	-604.5900240	-604.6200150	-0.0505852	#N/A	#N/A	#N/A	#N/A
12	5fUacl	-604.6704568	0.0804707	-604.5899860	-604.6218873	-0.0485695	#N/A	#N/A	#N/A	#N/A
13	5fUaas	-604.6708872	0.0809342	-604.5899530	-604.6225364	-0.0483508	#N/A	#N/A	#N/A	#N/A
14	5fUaag	-604.6706039	0.0807849	-604.5898190	-604.6194768	-0.0511271	#N/A	#N/A	#N/A	#N/A
15	5fUace	-604.6705836	0.0809336	-604.5896500	-604.6205659	-0.0500177	#N/A	#N/A	#N/A	#N/A
16	5fUacf	-604.6711139	0.0815178	-604.5895960	-604.6227709	-0.0483430	-603.6605268	-603.8459909	-603.9599684	-604.0083114
17	5fUabe	-604.6707284	0.0811724	-604.5895560	-604.6155160	-0.0552124	#N/A	#N/A	#N/A	#N/A
18	5fUaay	-604.6705558	0.0811308	-604.5894250	-604.6203163	-0.0502395	#N/A	#N/A	#N/A	#N/A
19	5fUacu	-604.6711148	0.0816978	-604.5894170	-604.6222948	-0.0488200	#N/A	#N/A	#N/A	#N/A
20	5fUacy	-604.6706546	0.0814395	-604.5892150	-604.6228997	-0.0477549	#N/A	#N/A	#N/A	#N/A
1	5dhmUabh	-681.1241311	0.1058810	-681.0182500	-681.0773363	-0.0467948	-680.0163240	-680.2282242	-680.3586280	-680.4054228
2	5dhmUabp	-681.1247221	0.1066130	-681.0181090	-681.0775116	-0.0472104	-680.0162404	-680.2282433	-680.3586859	-680.4058963
3	5dhmUabs	-681.1248977	0.1067926	-681.0181050	-681.0809610	-0.0439367	-680.0193411	-680.2312904	-680.3617274	-680.4056641
4	5dhmUaax	-681.1247189	0.1066678	-681.0180510	-681.0774291	-0.0472898	-680.0162431	-680.2282342	-680.3586668	-680.4059566
5	5dhmUabz	-681.1243247	0.1063176	-681.0180070	-681.0790269	-0.0452978	-680.0177898	-680.2298557	-680.3603371	-680.4056349
6	5dhmUacs	-681.1247521	0.1068581	-681.0178940	-681.0767342	-0.0480179	#N/A	#N/A	#N/A	#N/A
7	5dhmUaaq	-681.1248858	0.1071868	-681.0176990	-681.0758868	-0.0489990	#N/A	#N/A	#N/A	#N/A
8	5dhmUacb	-681.1258928	0.1085348	-681.0173580	-681.0740223	-0.0518705	-680.0126791	-680.2247987	-680.3552650	-680.4071355
9	5dhmUact	-681.1247613	0.1074543	-681.0173070	-681.0732373	-0.0515241	#N/A	#N/A	#N/A	#N/A

10	5dhmUabc	-681.1248549	0.1075739	-681.0172810	-681.0766315	-0.0482234	#N/A	#N/A	#N/A	#N/A
11	5dhmUacv	-681.1246305	0.1073775	-681.0172530	-681.0784119	-0.0462186	#N/A	#N/A	#N/A	#N/A
12	5dhmUadg	-681.1246148	0.1075268	-681.0170880	-681.0845600	-0.0400549	#N/A	#N/A	#N/A	#N/A
13	5dhmUaau	-681.1248601	0.1080170	-681.0168430	-681.0793019	-0.0455582	#N/A	#N/A	#N/A	#N/A
14	5dhmUaaz	-681.1244453	0.1076073	-681.0168380	-681.0777590	-0.0466863	#N/A	#N/A	#N/A	#N/A
15	5dhmUaba	-681.1278282	0.1110111	-681.0168170	-681.0814898	-0.0463383	-680.0198830	-680.2316294	-680.3620069	-680.4083452
16	5dhmUacf	-681.1243154	0.1075384	-681.0167770	-681.0784524	-0.0458630	#N/A	#N/A	#N/A	#N/A
17	5dhmUacc	-681.1244978	0.1078867	-681.0166110	-681.0765291	-0.0479687	#N/A	#N/A	#N/A	#N/A
18	5dhmUaap	-681.1247861	0.1082901	-681.0164960	-681.0772817	-0.0475044	#N/A	#N/A	#N/A	#N/A
19	5dhmUaco	-681.1245437	0.1081126	-681.0164310	-681.0789592	-0.0455845	#N/A	#N/A	#N/A	#N/A
20	5dhmUaaj	-681.1277612	0.1114872	-681.0162740	-681.0800020	-0.0477592	-680.0185816	-680.2303896	-680.3607885	-680.4085477
1	5caUach	-679.9361832	0.0844492	-679.8517340	-679.8940653	-0.0421179	-678.8375733	-679.0466412	-679.1752767	-679.2173946
2	5caUacv	-679.9348340	0.0831769	-679.8516570	-679.8895704	-0.0452636	-678.8338777	-679.0434614	-679.1722940	-679.2175576
3	5caUads	-679.9363570	0.0851429	-679.8512140	-679.8963050	-0.0400519	-678.8396654	-679.0486203	-679.1772261	-679.2172780
4	5caUabb	-679.9363419	0.0853269	-679.8510150	-679.8962487	-0.0400933	-678.8394439	-679.0484139	-679.1770376	-679.2171309
5	5caUadm	-679.9361578	0.0851567	-679.8510010	-679.8934642	-0.0426936	-678.8371227	-679.0461467	-679.1747615	-679.2174551
6	5caUacu	-679.9363590	0.0854529	-679.8509060	-679.8961644	-0.0401945	-678.8395130	-679.0485129	-679.1771334	-679.2173279
7	5caUacn	-679.9347646	0.0839415	-679.8508230	-679.8913166	-0.0434479	#N/A	#N/A	#N/A	#N/A
8	5caUaco	-679.9361687	0.0856577	-679.8505110	-679.8927509	-0.0434178	-678.8366957	-679.0456033	-679.1741629	-679.2175807
9	5caUabh	-679.9355122	0.0854102	-679.8501020	-679.8926084	-0.0429038	#N/A	#N/A	#N/A	#N/A
10	5caUaaz	-679.9357134	0.0858983	-679.8498150	-679.8942149	-0.0414984	-678.8379001	-679.0470338	-679.1757073	-679.2172057
11	5caUabl	-679.9347210	0.0849830	-679.8497380	-679.8906089	-0.0441121	#N/A	#N/A	#N/A	#N/A
12	5caUaae	-679.9348359	0.0851238	-679.8497120	-679.8894252	-0.0454107	#N/A	#N/A	#N/A	#N/A
13	5caUaah	-679.9348234	0.0855973	-679.8492260	-679.8904861	-0.0443373	#N/A	#N/A	#N/A	#N/A
14	5caUabu	-679.9346877	0.0854826	-679.8492050	-679.8901296	-0.0445581	#N/A	#N/A	#N/A	#N/A
15	5caUaar	-679.9348588	0.0858028	-679.8490560	-679.8905838	-0.0442750	#N/A	#N/A	#N/A	#N/A
16	5caUacx	-679.9332375	0.0842414	-679.8489960	-679.8886459	-0.0445915	#N/A	#N/A	#N/A	#N/A
17	5caUabg	-679.9322988	0.0833117	-679.8489870	-679.8845826	-0.0477162	#N/A	#N/A	#N/A	#N/A
18	5caUace	-679.9353173	0.0864553	-679.8488620	-679.8965769	-0.0387404	#N/A	#N/A	#N/A	#N/A
19	5caUadc	-679.9345947	0.0858677	-679.8487270	-679.8892401	-0.0453546	#N/A	#N/A	#N/A	#N/A
20	5caUabo	-679.9351048	0.0864067	-679.8486980	-679.8842465	-0.0508583	#N/A	#N/A	#N/A	#N/A
1	1mUacm	-530.6479953	0.1001262	-530.5478690	-530.6145506	-0.0334447	-529.7459842	-529.9093914	-530.0099444	-530.0433891
2	1mUadf	-530.6478524	0.1005643	-530.5472880	-530.6152562	-0.0325961	-529.7450520	-529.9090106	-530.0098122	-530.0424083
3	1mUack	-530.6478768	0.1007737	-530.5471030	-530.6154008	-0.0324760	-529.7453804	-529.9092691	-530.0100413	-530.0425173
4	1mUaab	-530.6482271	0.1014610	-530.5467660	-530.6107914	-0.0374357	-529.7418318	-529.9054758	-530.0061369	-530.0435726
5	1mUaba	-530.6482506	0.1015046	-530.5467460	-530.6102935	-0.0379571	#N/A	#N/A	#N/A	#N/A
6	1mUacz	-530.6455017	0.0990637	-530.5464380	-530.6071826	-0.0383191	#N/A	#N/A	#N/A	#N/A
7	1mUado	-530.6429102	0.0966152	-530.5462950	-530.6054627	-0.0374475	#N/A	#N/A	#N/A	#N/A
8	1mUach	-530.6475503	0.1015382	-530.5460120	-530.6135615	-0.0339888	#N/A	#N/A	#N/A	#N/A
9	1mUaan	-530.6479254	0.1019913	-530.5459340	-530.6156890	-0.0322364	-529.7452201	-529.9092563	-530.0101146	-530.0423510
10	1mUacq	-530.6465049	0.1005798	-530.5459250	-530.6139470	-0.0325579	#N/A	#N/A	#N/A	#N/A
11	1mUabw	-530.6473064	0.1014384	-530.5458680	-530.6091393	-0.0381672	#N/A	#N/A	#N/A	#N/A
12	1mUaas	-530.6475118	0.1016518	-530.5458600	-530.6095943	-0.0379175	#N/A	#N/A	#N/A	#N/A
13	1mUaae	-530.6482720	0.1024990	-530.5457730	-530.6097767	-0.0384954	#N/A	#N/A	#N/A	#N/A
14	1mUacf	-530.6425345	0.0968265	-530.5457080	-530.6010006	-0.0415339	#N/A	#N/A	#N/A	#N/A
15	1mUact	-530.6469453	0.1012422	-530.5457030	-530.6143155	-0.0326297	#N/A	#N/A	#N/A	#N/A
16	1mUabd	-530.6451421	0.0995041	-530.5456380	-530.6045018	-0.0406403	#N/A	#N/A	#N/A	#N/A
17	1mUabp	-530.6475307	0.1019536	-530.5455770	-530.6096364	-0.0378943	#N/A	#N/A	#N/A	#N/A

18	1mUabi	-530.6473166	0.1019206	-530.5453960	-530.6094564	-0.0378602	#N/A	#N/A	#N/A	#N/A
19	1mUacw	-530.6461788	0.1007957	-530.5453830	-530.6063831	-0.0397956	#N/A	#N/A	#N/A	#N/A
20	1mUabo	-530.6452537	0.0998757	-530.5453780	-530.6037700	-0.0414838	#N/A	#N/A	#N/A	#N/A
1	1m5mUacu	-569.9721272	0.1247181	-569.8474090	-569.9396405	-0.0324867	-568.9908293	-569.1657088	-569.2735082	-569.3059949
2	1m5mUaal	-569.9720101	0.1254030	-569.8466070	-569.9403191	-0.0316910	-568.9903789	-569.1656387	-569.2736083	-569.3052993
3	1m5mUaar	-569.9723988	0.1259108	-569.8464880	-569.9354337	-0.0369651	-568.9863038	-569.1614347	-569.2693320	-569.3062971
4	1m5mUadc	-569.9728126	0.1266455	-569.8461670	-569.9357345	-0.0370781	-568.9862509	-569.1614352	-569.2693650	-569.3064431
5	1m5mUacs	-569.9727332	0.1270612	-569.8456720	-569.9374423	-0.0352910	-568.9883193	-569.1632537	-569.2710828	-569.3063738
6	1m5mUaay	-569.9723163	0.1267773	-569.8455390	-569.9367326	-0.0355837	-568.9880585	-569.1629451	-569.2707431	-569.3063268
7	1m5mUacl	-569.9722494	0.1268973	-569.8453520	-569.9412475	-0.0310019	#N/A	#N/A	#N/A	#N/A
8	1m5mUadd	-569.9704938	0.1254568	-569.8450370	-569.9324197	-0.0380742	#N/A	#N/A	#N/A	#N/A
9	1m5mUade	-569.9692915	0.1244505	-569.8448410	-569.9309988	-0.0382928	#N/A	#N/A	#N/A	#N/A
10	1m5mUadf	-569.9722537	0.1277166	-569.8445370	-569.9358016	-0.0364521	#N/A	#N/A	#N/A	#N/A
11	1m5mUabg	-569.9717295	0.1272015	-569.8445280	-569.9409596	-0.0307699	#N/A	#N/A	#N/A	#N/A
12	1m5mUabq	-569.9706273	0.1261322	-569.8444950	-569.9321551	-0.0384722	#N/A	#N/A	#N/A	#N/A
13	1m5mUabw	-569.9703723	0.1259123	-569.8444600	-569.9335196	-0.0368527	#N/A	#N/A	#N/A	#N/A
14	1m5mUadj	-569.9704073	0.1260762	-569.8443310	-569.9336728	-0.0367345	#N/A	#N/A	#N/A	#N/A
15	1m5mUadu	-569.9722892	0.1279771	-569.8443120	-569.9357012	-0.0365880	#N/A	#N/A	#N/A	#N/A
16	1m5mUabm	-569.9712268	0.1269427	-569.8442840	-569.9397804	-0.0314463	#N/A	#N/A	#N/A	#N/A
17	1m5mUaci	-569.9717489	0.1275338	-569.8442150	-569.9410984	-0.0306504	#N/A	#N/A	#N/A	#N/A
18	1m5mUaca	-569.9701935	0.1259944	-569.8441990	-569.9320037	-0.0381897	#N/A	#N/A	#N/A	#N/A
19	1m5mUacv	-569.9692267	0.1251736	-569.8440530	-569.9317406	-0.0374861	#N/A	#N/A	#N/A	#N/A
20	1m5mUaco	-569.9729439	0.1289018	-569.8440420	-569.9374879	-0.0354560	-568.9883334	-569.1632315	-569.2710379	-569.3064939
1	1m5hmUabv	-645.1999672	0.1313142	-645.0686530	-645.1573784	-0.0425889	-644.1102937	-644.3095335	-644.4322542	-644.4748431
2	1m5hmUacg	-645.2014891	0.1332830	-645.0682060	-645.1655246	-0.0359645	-644.1186257	-644.3175685	-644.4401863	-644.4761508
3	1m5hmUacb	-645.2001211	0.1321371	-645.0679840	-645.1584768	-0.0416444	-644.1114154	-644.3105878	-644.4332873	-644.4749317
4	1m5hmUaac	-645.1983325	0.1305945	-645.0677380	-645.1600393	-0.0382932	-644.1130304	-644.3127979	-644.4357316	-644.4740248
5	1m5hmUadd	-645.1981399	0.1304519	-645.0676880	-645.1589928	-0.0391471	#N/A	#N/A	#N/A	#N/A
6	1m5hmUadr	-645.2015010	0.1338540	-645.0676470	-645.1655382	-0.0359628	-644.1182880	-644.3174160	-644.4401195	-644.4760823
7	1m5hmUacq	-645.1983656	0.1308575	-645.0675080	-645.1546471	-0.0437185	#N/A	#N/A	#N/A	#N/A
8	1m5hmUada	-645.1960167	0.1287157	-645.0673010	-645.1494369	-0.0465798	#N/A	#N/A	#N/A	#N/A
9	1m5hmUaaz	-645.1953467	0.1280987	-645.0672480	-645.1532860	-0.0420607	#N/A	#N/A	#N/A	#N/A
10	1m5hmUabp	-645.1964382	0.1291941	-645.0672440	-645.1526960	-0.0437422	#N/A	#N/A	#N/A	#N/A
11	1m5hmUaap	-645.2001346	0.1329385	-645.0671960	-645.1583555	-0.0417791	#N/A	#N/A	#N/A	#N/A
12	1m5hmUaco	-645.1981363	0.1309752	-645.0671610	-645.1587472	-0.0393891	#N/A	#N/A	#N/A	#N/A
13	1m5hmUadf	-645.1989417	0.1317847	-645.0671570	-645.1626009	-0.0363408	#N/A	#N/A	#N/A	#N/A
14	1m5hmUack	-645.1988501	0.1317171	-645.0671330	-645.1600574	-0.0387927	#N/A	#N/A	#N/A	#N/A
15	1m5hmUact	-645.2001536	0.1330446	-645.0671090	-645.1577613	-0.0423923	-644.1110445	-644.3099508	-644.4325356	-644.4749279
16	1m5hmUadk	-645.1982371	0.1311911	-645.0670460	-645.1606754	-0.0375617	#N/A	#N/A	#N/A	#N/A
17	1m5hmUabd	-645.1975771	0.1305370	-645.0670400	-645.1597770	-0.0378000	#N/A	#N/A	#N/A	#N/A
18	1m5hmUadl	-645.2001760	0.1331630	-645.0670130	-645.1581119	-0.0420641	-644.1114127	-644.3102809	-644.4328447	-644.4749088
19	1m5hmUaaq	-645.1973439	0.1304159	-645.0669280	-645.1603845	-0.0369595	#N/A	#N/A	#N/A	#N/A
20	1m5hmUacf	-645.1983773	0.1314783	-645.0668990	-645.1603686	-0.0380087	#N/A	#N/A	#N/A	#N/A
1	1m5fUadq	-643.9836474	0.1063704	-643.8772770	-643.9383622	-0.0452852	-642.8970104	-643.0928891	-643.2135120	-643.2587972
2	1m5fUadi	-643.9833863	0.1067683	-643.8766180	-643.9399811	-0.0434052	-642.8990771	-643.0950406	-643.2157049	-643.2591101
3	1m5fUaax	-643.9834670	0.1071609	-643.8763060	-643.9413877	-0.0420793	-642.9003796	-643.0961724	-643.2167725	-643.2588518
4	1m5fUabe	-643.9839857	0.1079036	-643.8760820	-643.9386433	-0.0453424	-642.8963467	-643.0929883	-643.2139163	-643.2592587
5	1m5fUacy	-643.9839229	0.1078878	-643.8760350	-643.9372817	-0.0466412	#N/A	#N/A	#N/A	#N/A

6	1m5fUaal	-643.9809057	0.1049186	-643.8759870	-643.9331626	-0.0477431	#N/A	#N/A	#N/A	#N/A
7	1m5fUadg	-643.9841044	0.1081243	-643.8759800	-643.9381149	-0.0459895	-642.8959739	-643.0925775	-643.2134908	-643.2594803
8	1m5fUada	-643.9839971	0.1080470	-643.8759500	-643.9381026	-0.0458945	#N/A	#N/A	#N/A	#N/A
9	1m5fUaat	-643.9826743	0.1068342	-643.8758400	-643.9360555	-0.0466187	#N/A	#N/A	#N/A	#N/A
10	1m5fUaaq	-643.9843410	0.1085099	-643.8758310	-643.9393795	-0.0449615	-642.8957637	-643.0925532	-643.2136241	-643.2585856
11	1m5fUaco	-643.9839514	0.1083334	-643.8756180	-643.9389476	-0.0450038	-642.8955976	-643.0925836	-643.2136649	-643.2586687
12	1m5fUaas	-643.9839402	0.1084231	-643.8755170	-643.9356139	-0.0483263	#N/A	#N/A	#N/A	#N/A
13	1m5fUaay	-643.9839577	0.1084467	-643.8755110	-643.9364182	-0.0475395	-642.8939145	-643.0907235	-643.2117029	-643.2592424
14	1m5fUacg	-643.9839460	0.1085060	-643.8754400	-643.9357838	-0.0481623	#N/A	#N/A	#N/A	#N/A
15	1m5fUabc	-643.9827574	0.1073214	-643.8754360	-643.9353112	-0.0474462	#N/A	#N/A	#N/A	#N/A
16	1m5fUabx	-643.9833619	0.1080419	-643.8753200	-643.9360584	-0.0473035	#N/A	#N/A	#N/A	#N/A
17	1m5fUadm	-643.9833810	0.1080769	-643.8753040	-643.9373201	-0.0460609	#N/A	#N/A	#N/A	#N/A
18	1m5fUacl	-643.9833710	0.1081470	-643.8752240	-643.9376968	-0.0456742	#N/A	#N/A	#N/A	#N/A
19	1m5fUaad	-643.9842232	0.1092882	-643.8749350	-643.9391955	-0.0450278	-642.8953222	-643.0921672	-643.2132703	-643.2582981
20	1m5fUabb	-643.9809482	0.1060231	-643.8749250	-643.9306352	-0.0503130	#N/A	#N/A	#N/A	#N/A
1	1m5dhmUadr	-720.4363808	0.1323927	-720.3039880	-720.3941395	-0.0422413	-719.2522367	-719.4754489	-719.6129487	-719.6551900
2	1m5dhmUaae	-720.4402451	0.1366111	-720.3036340	-720.3974578	-0.0427873	-719.2548260	-719.4777944	-719.6152239	-719.6580112
3	1m5dhmUacv	-720.4383549	0.1348988	-720.3034560	-720.3898887	-0.0484662	-719.2474567	-719.4708660	-719.6084153	-719.6568815
4	1m5dhmUada	-720.4383448	0.1350717	-720.3032730	-720.3904725	-0.0478723	-719.2479831	-719.4710593	-719.6084791	-719.6563514
5	1m5dhmUacj	-720.4365060	0.1332350	-720.3032710	-720.3871595	-0.0493465	#N/A	#N/A	#N/A	#N/A
6	1m5dhmUabf	-720.4373381	0.1342640	-720.3030740	-720.3951017	-0.0422364	#N/A	#N/A	#N/A	#N/A
7	1m5dhmUabr	-720.4372933	0.1342312	-720.3030620	-720.3947664	-0.0425269	#N/A	#N/A	#N/A	#N/A
8	1m5dhmUabe	-720.4354370	0.1324469	-720.3029900	-720.3842069	-0.0512301	#N/A	#N/A	#N/A	#N/A
9	1m5dhmUaab	-720.4402300	0.1372439	-720.3029860	-720.3958160	-0.0444140	-719.2534276	-719.4764521	-719.6139002	-719.6583142
10	1m5dhmUaah	-720.4382859	0.1353938	-720.3028920	-720.3893824	-0.0489035	#N/A	#N/A	#N/A	#N/A
11	1m5dhmUabm	-720.4383108	0.1354208	-720.3028900	-720.3904007	-0.0479101	#N/A	#N/A	#N/A	#N/A
12	1m5dhmUadb	-720.4372210	0.1343600	-720.3028610	-720.3948409	-0.0423802	#N/A	#N/A	#N/A	#N/A
13	1m5dhmUabd	-720.4374930	0.1346390	-720.3028540	-720.3922390	-0.0452540	#N/A	#N/A	#N/A	#N/A
14	1m5dhmUack	-720.4383930	0.1358020	-720.3025910	-720.3903757	-0.0480174	-719.2476978	-719.4710705	-719.6086138	-719.6566312
15	1m5dhmUaao	-720.4366103	0.1340593	-720.3025510	-720.3871827	-0.0494276	#N/A	#N/A	#N/A	#N/A
16	1m5dhmUaax	-720.4367236	0.1343026	-720.3024210	-720.3929508	-0.0437728	#N/A	#N/A	#N/A	#N/A
17	1m5dhmUaco	-720.4364319	0.1340888	-720.3023430	-720.3939626	-0.0424693	#N/A	#N/A	#N/A	#N/A
18	1m5dhmUaav	-720.4384242	0.1360971	-720.3023270	-720.3905850	-0.0478392	#N/A	#N/A	#N/A	#N/A
19	1m5dhmUacs	-720.4383845	0.1360794	-720.3023050	-720.3902579	-0.0481266	-719.2477145	-719.4709616	-719.6084487	-719.6565753
20	1m5dhmUacp	-720.4365491	0.1342980	-720.3022510	-720.3871396	-0.0494094	#N/A	#N/A	#N/A	#N/A
1	1m5caUaal	-719.2488074	0.1087493	-719.1400580	-719.2105218	-0.0382855	-718.0732257	-718.2935882	-718.4293204	-718.4676059
2	1m5caUacr	-719.2486516	0.1099486	-719.1387030	-719.2094485	-0.0392032	-718.0724446	-718.2925600	-718.4281750	-718.4673782
3	1m5caUaaz	-719.2480448	0.1101067	-719.1379380	-719.2071098	-0.0409350	-718.0701515	-718.2909870	-718.4268892	-718.4678242
4	1m5caUacz	-719.2481498	0.1114628	-719.1366870	-719.2076521	-0.0404977	-718.0689498	-718.2899909	-718.4260064	-718.4665041
5	1m5caUabj	-719.2485900	0.1123080	-719.1362820	-719.2013279	-0.0472622	-718.0637989	-718.2849610	-718.4209927	-718.4682549
6	1m5caUabd	-719.2462354	0.1100683	-719.1361670	-719.2027756	-0.0434598	-718.0645449	-718.2857715	-718.4218100	-718.4652698
7	1m5caUabh	-719.2477174	0.1115574	-719.1361600	-719.2057393	-0.0419782	#N/A	#N/A	#N/A	#N/A
8	1m5caUaae	-719.2476686	0.1115306	-719.1361380	-719.2071078	-0.0405608	#N/A	#N/A	#N/A	#N/A
9	1m5caUacc	-719.2464266	0.1102956	-719.1361310	-719.2053302	-0.0410965	#N/A	#N/A	#N/A	#N/A
10	1m5caUaax	-719.2476396	0.1115576	-719.1360820	-719.2014806	-0.0461591	#N/A	#N/A	#N/A	#N/A
11	1m5caUadp	-719.2480901	0.1120470	-719.1360430	-719.2110559	-0.0370341	#N/A	#N/A	#N/A	#N/A
12	1m5caUabr	-719.2438470	0.1078579	-719.1359890	-719.1958542	-0.0479928	#N/A	#N/A	#N/A	#N/A
13	1m5caUadv	-719.2464164	0.1105014	-719.1359150	-719.2054547	-0.0409617	#N/A	#N/A	#N/A	#N/A

14	1m5caUaai	-719.2476657	0.1117567	-719.1359090	-719.2075280	-0.0401377	#N/A	#N/A	#N/A	#N/A
15	1m5caUaci	-719.2486225	0.1127185	-719.1359040	-719.2018814	-0.0467411	#N/A	#N/A	#N/A	#N/A
16	1m5caUaao	-719.2487549	0.1128879	-719.1358670	-719.2029833	-0.0457717	-718.0663650	-718.2872492	-718.4231622	-718.4689339
17	1m5caUabs	-719.2464545	0.1107955	-719.1356590	-719.2047464	-0.0417081	#N/A	#N/A	#N/A	#N/A
18	1m5caUaah	-719.2461678	0.1105827	-719.1355850	-719.2032248	-0.0429429	#N/A	#N/A	#N/A	#N/A
19	1m5caUacj	-719.2481569	0.1127138	-719.1354430	-719.2111372	-0.0370197	#N/A	#N/A	#N/A	#N/A
20	1m5caUaag	-719.2483127	0.1128896	-719.1354230	-719.2020869	-0.0462258	#N/A	#N/A	#N/A	#N/A
1	rc_C	-471.4584875	0.0853284	-471.3731590	#N/A	#N/A	#N/A	#N/A	#N/A	#N/A
2	rc_Caar	-471.2397707	0.0848467	-471.1549240	-471.1110224	-0.1287483	-470.3357942	-470.4753360	-470.5613236	-470.6900719
3	rc_Caab	-471.2406526	0.0860346	-471.1546180	-471.1241402	-0.1165124	-470.3470031	-470.4864358	-470.5724311	-470.6889435
4	rc_Cadd	-471.2398340	0.0852240	-471.1546100	-471.1169295	-0.1229045	-470.3402783	-470.4800610	-470.5661760	-470.6890806
5	rc_Cacu	-471.2406525	0.0860435	-471.1546090	-471.1241403	-0.1165122	-470.3470373	-470.4864490	-470.5724283	-470.6889405
6	rc_Caaw	-471.2395330	0.0852630	-471.1542700	-471.1126402	-0.1268928	-470.3363718	-470.4763293	-470.5625072	-470.6894000
7	rc_Cacq	-471.2404400	0.0863929	-471.1540470	-471.1084423	-0.1319977	-470.3332434	-470.4726906	-470.5586253	-470.6906230
8	rc_Cact	-471.2403194	0.0862884	-471.1540310	-471.1210018	-0.1193176	-470.3439949	-470.4835998	-470.5696616	-470.6889792
9	rc_Caao	-471.2392709	0.0855789	-471.1536920	-471.1165947	-0.1226762	-470.3402144	-470.4798939	-470.5659595	-470.6886357
10	rc_Caay	-471.2392616	0.0857816	-471.1534800	-471.1179766	-0.1212851	-470.3414974	-470.4811409	-470.5671948	-470.6884799
11	rc_Caad	-471.2397295	0.0862594	-471.1534700	-471.1112551	-0.1284743	-470.3357769	-470.4754219	-470.5614505	-470.6899248
12	rc_Cacy	-471.2397136	0.0864466	-471.1532670	-471.1108603	-0.1288533	-470.3350638	-470.4748894	-470.5609944	-470.6898477
13	rc_Cabi	-471.2402702	0.0871511	-471.1531190	-471.1109811	-0.1292891	-470.3348257	-470.4746282	-470.5607311	-470.6900202
14	rc_Cace	-471.2395767	0.0865587	-471.1530180	-471.1205162	-0.1190606	-470.3437589	-470.4833828	-470.5694391	-470.6884997
15	rc_Cabh	-471.2403191	0.0877950	-471.1525240	-471.1096834	-0.1306357	-470.3339504	-470.4735968	-470.5596245	-470.6902602
16	rc_Cabc	-471.2394700	0.0869510	-471.1525190	-471.1128688	-0.1266012	-470.3366101	-470.4764544	-470.5625833	-470.6891845
17	rc_Cabm	-471.2363981	0.0842761	-471.1521220	-471.0995668	-0.1368313	-470.3215691	-470.4628128	-470.5495032	-470.6863345
18	rc_Cacz	-471.2341352	0.0829641	-471.1511710	-471.1000950	-0.1340402	-470.3228367	-470.4638506	-470.5504375	-470.6844777
19	rc_Cadq	-471.2364613	0.0854572	-471.1510040	-471.0999589	-0.1365024	-470.3226242	-470.4636461	-470.5502438	-470.6867462
20	rc_Cack	-471.2364907	0.0856496	-471.1508410	-471.0995809	-0.1369098	-470.3230312	-470.4637742	-470.5502398	-470.6871496
1	rc_5mCaaf	-510.5729467	0.1119807	-510.4609660	-510.4585594	-0.1143874	-509.5991944	-509.7505947	-509.8440764	-509.9584638
2	rc_5mCaco	-510.5724480	0.1115709	-510.4608770	-510.4561753	-0.1162727	-509.5969330	-509.7484249	-509.8419314	-509.9582041
3	rc_5mCadd	-510.5724516	0.1120305	-510.4604210	-510.4558123	-0.1166393	-509.5966128	-509.7481105	-509.8416175	-509.9582568
4	rc_5mCabj	-510.5722892	0.1120032	-510.4602860	-510.4452154	-0.1270738	-509.5868044	-509.7385849	-509.8321771	-509.9592509
5	rc_5mCacc	-510.5721706	0.1119575	-510.4602130	-510.4454587	-0.1267119	#N/A	#N/A	#N/A	#N/A
6	rc_5mCaau	-510.5724242	0.1123742	-510.4600500	-510.4428719	-0.1295523	-509.5855341	-509.7369462	-509.8303676	-509.9599199
7	rc_5mCaaw	-510.5712631	0.1117591	-510.4595040	-510.4466949	-0.1245682	#N/A	#N/A	#N/A	#N/A
8	rc_5mCaam	-510.5702822	0.1108982	-510.4593840	-510.4497339	-0.1205483	#N/A	#N/A	#N/A	#N/A
9	rc_5mCabg	-510.5712851	0.1123500	-510.4589350	-510.4463700	-0.1249150	#N/A	#N/A	#N/A	#N/A
10	rc_5mCads	-510.5712292	0.1126512	-510.4585780	-510.4476326	-0.1235966	#N/A	#N/A	#N/A	#N/A
11	rc_5mCabe	-510.5712041	0.1129251	-510.4582790	-510.4435217	-0.1276824	#N/A	#N/A	#N/A	#N/A
12	rc_5mCada	-510.5712168	0.1130107	-510.4582060	-510.4445631	-0.1266537	#N/A	#N/A	#N/A	#N/A
13	rc_5mCaai	-510.5690249	0.1108839	-510.4581410	-510.4343345	-0.1346905	#N/A	#N/A	#N/A	#N/A
14	rc_5mCacp	-510.5719209	0.1137988	-510.4581220	-510.4476889	-0.1242320	#N/A	#N/A	#N/A	#N/A
15	rc_5mCadc	-510.5689428	0.1110468	-510.4578960	-510.4345802	-0.1343626	#N/A	#N/A	#N/A	#N/A
16	rc_5mCabz	-510.5690707	0.1114347	-510.4576360	-510.4361620	-0.1329087	#N/A	#N/A	#N/A	#N/A
17	rc_5mCadt	-510.5689889	0.1113979	-510.4575910	-510.4348519	-0.1341370	#N/A	#N/A	#N/A	#N/A
18	rc_5mCadb	-510.5691254	0.1119184	-510.4572070	-510.4203909	-0.1487346	#N/A	#N/A	#N/A	#N/A
19	rc_5mCadp	-510.5678074	0.1111583	-510.4566490	-510.4291501	-0.1386573	#N/A	#N/A	#N/A	#N/A
20	rc_5mCaan	-510.5649586	0.1092766	-510.4556820	-510.4289401	-0.1360186	#N/A	#N/A	#N/A	#N/A
1	rc_5hmCacg	-585.7926794	0.1148893	-585.6777900	-585.6637854	-0.1288940	-584.7086483	-584.8841209	-584.9923290	-585.1212230

2	rc_5hmCaaz	-585.7938117	0.1162607	-585.6775510	-585.6756078	-0.1182039	-584.7198340	-584.8950697	-585.0032154	-585.1214193
3	rc_5hmCacm	-585.7925485	0.1151394	-585.6774090	-585.6652920	-0.1272564	-584.7096411	-584.8852399	-584.9935064	-585.1207628
4	rc_5hmCabj	-585.7932390	0.1158390	-585.6774000	-585.6724008	-0.1208383	-584.7167938	-584.8921488	-585.0003238	-585.1211621
5	rc_5hmCaab	-585.7925098	0.1155178	-585.6769920	-585.6662442	-0.1262656	#N/A	#N/A	#N/A	#N/A
6	rc_5hmCabp	-585.7926732	0.1156881	-585.6769850	-585.6653732	-0.1273000	#N/A	#N/A	#N/A	#N/A
7	rc_5hmCack	-585.7864263	0.1101632	-585.6762630	-585.6520564	-0.1343699	#N/A	#N/A	#N/A	#N/A
8	rc_5hmCade	-585.7925842	0.1164822	-585.6761020	-585.6633727	-0.1292115	#N/A	#N/A	#N/A	#N/A
9	rc_5hmCacj	-585.7932270	0.1171550	-585.6760720	-585.6623576	-0.1308694	-584.7077292	-584.8832067	-584.9913932	-585.1222626
10	rc_5hmCacc	-585.7930175	0.1170584	-585.6759590	-585.6529034	-0.1401141	-584.6986050	-584.8738423	-584.9819269	-585.1220410
11	rc_5hmCabk	-585.7928039	0.1172898	-585.6755140	-585.6629309	-0.1298729	#N/A	#N/A	#N/A	#N/A
12	rc_5hmCaby	-585.7928388	0.1174198	-585.6754190	-585.6630397	-0.1297991	#N/A	#N/A	#N/A	#N/A
13	rc_5hmCaax	-585.7932085	0.1183404	-585.6748680	-585.6544318	-0.1387767	-584.7001670	-584.8755092	-584.9836439	-585.1224206
14	rc_5hmCadf	-585.7895876	0.1148125	-585.6747750	-585.6472405	-0.1423471	#N/A	#N/A	#N/A	#N/A
15	rc_5hmCabl	-585.7911712	0.1169272	-585.6742440	-585.6632054	-0.1279658	#N/A	#N/A	#N/A	#N/A
16	rc_5hmCadd	-585.7914153	0.1172383	-585.6741770	-585.6620569	-0.1293584	#N/A	#N/A	#N/A	#N/A
17	rc_5hmCadu	-585.7895468	0.1156907	-585.6738560	-585.6411341	-0.1484127	#N/A	#N/A	#N/A	#N/A
18	rc_5hmCacu	-585.7895460	0.1159200	-585.6736260	-585.6414808	-0.1480652	#N/A	#N/A	#N/A	#N/A
19	rc_5hmCaau	-585.7894937	0.1159176	-585.6735760	-585.6421343	-0.1473594	#N/A	#N/A	#N/A	#N/A
20	rc_5hmCach	-585.7888719	0.1153998	-585.6734720	-585.6462659	-0.1426060	#N/A	#N/A	#N/A	#N/A
1	rc_5fCaba	-584.5658067	0.0916796	-584.4741270	-584.4430485	-0.1227582	-583.4936289	-583.6665939	-583.7731218	-583.8958800
2	rc_5fCaca	-584.5658909	0.0923698	-584.4735210	-584.4449482	-0.1209426	-583.4953838	-583.6682538	-583.7747471	-583.8956897
3	rc_5fCaah	-584.5659183	0.0923983	-584.4735200	-584.4410293	-0.1248890	-583.4916669	-583.6646536	-583.7711807	-583.8960697
4	rc_5fCadh	-584.5659423	0.0931133	-584.4728290	-584.4392564	-0.1266859	-583.4901309	-583.6630746	-583.7695732	-583.8962591
5	rc_5fCaab	-584.5660825	0.0934274	-584.4726550	-584.4496115	-0.1164710	-583.4997820	-583.6724213	-583.7788273	-583.8952983
6	rc_5fCabw	-584.5659559	0.0934129	-584.4725430	-584.4384292	-0.1275268	-583.4895601	-583.6624073	-583.7688582	-583.8963850
7	rc_5fCact	-584.5593501	0.0889820	-584.4703680	-584.4271101	-0.1322399	#N/A	#N/A	#N/A	#N/A
8	rc_5fCacz	-584.5620981	0.0919760	-584.4701220	-584.4263221	-0.1357760	#N/A	#N/A	#N/A	#N/A
9	rc_5fCaam	-584.5613170	0.0917679	-584.4695490	-584.4270348	-0.1342822	#N/A	#N/A	#N/A	#N/A
10	rc_5fCabq	-584.5623811	0.0929891	-584.4693920	-584.4282366	-0.1341445	#N/A	#N/A	#N/A	#N/A
11	rc_5fCaad	-584.5603625	0.0909795	-584.4693830	-584.4236312	-0.1367313	#N/A	#N/A	#N/A	#N/A
12	rc_5fCaau	-584.5612710	0.0920000	-584.4692710	-584.4368440	-0.1244270	#N/A	#N/A	#N/A	#N/A
13	rc_5fCabk	-584.5611143	0.0920332	-584.4690810	-584.4376291	-0.1234852	#N/A	#N/A	#N/A	#N/A
14	rc_5fCacc	-584.5612891	0.0923000	-584.4689890	-584.4287654	-0.1325237	#N/A	#N/A	#N/A	#N/A
15	rc_5fCabs	-584.5623913	0.0935133	-584.4688780	-584.4268637	-0.1355276	#N/A	#N/A	#N/A	#N/A
16	rc_5fCaao	-584.5604146	0.0915715	-584.4688430	-584.4220113	-0.1384033	#N/A	#N/A	#N/A	#N/A
17	rc_5fCadq	-584.5595562	0.0907902	-584.4687660	-584.4195998	-0.1399565	#N/A	#N/A	#N/A	#N/A
18	rc_5fCadf	-584.5623287	0.0936096	-584.4687190	-584.4303391	-0.1319896	#N/A	#N/A	#N/A	#N/A
19	rc_5fCaak	-584.5622954	0.0935783	-584.4687170	-584.4247590	-0.1375363	#N/A	#N/A	#N/A	#N/A
20	rc_5fCabn	-584.5623861	0.0936901	-584.4686960	-584.4315690	-0.1308171	#N/A	#N/A	#N/A	#N/A
1	rc_5dhmCadu	-661.0272438	0.1189218	-660.9083220	-660.8985901	-0.1286537	-659.8468684	-660.0465418	-660.1695833	-660.2982370
2	rc_5dhmCabw	-661.0279931	0.1200001	-660.9079930	-660.9051733	-0.1228198	-659.8531059	-660.0525530	-660.1755322	-660.2983520
3	rc_5dhmCabj	-661.0268896	0.1190235	-660.9078660	-660.8962116	-0.1306780	-659.8443366	-660.0441090	-660.1671637	-660.2978417
4	rc_5dhmCabf	-661.0275015	0.1198435	-660.9076580	-660.9026615	-0.1248401	-659.8507346	-660.0502334	-660.1732093	-660.2980494
5	rc_5dhmCaad	-661.0274363	0.1203542	-660.9070820	-660.8908197	-0.1366166	-659.8402027	-660.0397584	-660.1627070	-660.2993236
6	rc_5dhmCabc	-661.0268062	0.1197841	-660.9070220	-660.8950517	-0.1317545	#N/A	#N/A	#N/A	#N/A
7	rc_5dhmCadf	-661.0270979	0.1202079	-660.9068900	-660.8946535	-0.1324444	#N/A	#N/A	#N/A	#N/A
8	rc_5dhmCaal	-661.0274691	0.1206740	-660.9067950	-660.8841128	-0.1433563	-659.8329514	-660.0324051	-660.1553359	-660.2986922
9	rc_5dhmCabg	-661.0271646	0.1205975	-660.9065670	-660.8935470	-0.1336176	#N/A	#N/A	#N/A	#N/A

10	rc_5dhmCadd	-661.0271008	0.1205408	-660.9065600	-660.8950570	-0.1320438	#N/A	#N/A	#N/A	#N/A
11	rc_5dhmCach	-661.0273991	0.1209510	-660.9064480	-660.8944187	-0.1329804	-659.8429705	-660.0427882	-660.1658705	-660.2988509
12	rc_5dhmCadt	-661.0271688	0.1209248	-660.9062440	-660.8923415	-0.1348273	#N/A	#N/A	#N/A	#N/A
13	rc_5dhmCacc	-661.0268581	0.1206171	-660.9062410	-660.9000904	-0.1267678	#N/A	#N/A	#N/A	#N/A
14	rc_5dhmCabx	-661.0265328	0.1207878	-660.9057450	-660.8982023	-0.1283305	#N/A	#N/A	#N/A	#N/A
15	rc_5dhmCacs	-661.0272209	0.1215129	-660.9057080	-660.8928178	-0.1344032	#N/A	#N/A	#N/A	#N/A
16	rc_5dhmCaaw	-661.0226879	0.1175689	-660.9051190	-660.8748730	-0.1478149	#N/A	#N/A	#N/A	#N/A
17	rc_5dhmCacy	-661.0226869	0.1178798	-660.9048070	-660.8749548	-0.1477320	#N/A	#N/A	#N/A	#N/A
18	rc_5dhmCadr	-661.0270085	0.1222464	-660.9047620	-660.8926586	-0.1343499	#N/A	#N/A	#N/A	#N/A
19	rc_5dhmCaam	-661.0236361	0.1197850	-660.9038510	-660.8840908	-0.1395453	#N/A	#N/A	#N/A	#N/A
20	rc_5dhmCacu	-661.0242334	0.1204523	-660.9037810	-660.8810486	-0.1431847	#N/A	#N/A	#N/A	#N/A
1	rc_5caCacr	-659.8212092	0.0954252	-659.7257840	-659.6974772	-0.1237321	-658.6428306	-658.8392905	-658.9602845	-659.0840166
2	rc_5caCabd	-659.8211173	0.0955072	-659.7256100	-659.6931252	-0.1279921	-658.6387377	-658.8354582	-658.9565546	-659.0845467
3	rc_5caCaay	-659.8206898	0.0954847	-659.7252050	-659.6909662	-0.1297236	-658.6360308	-658.8326901	-658.9537110	-659.0834346
4	rc_5caCaaw	-659.8211654	0.0959613	-659.7252040	-659.6933111	-0.1278543	-658.6388913	-658.8355581	-658.9566293	-659.0844836
5	rc_5caCaaj	-659.8207643	0.0957352	-659.7250290	-659.6917774	-0.1289869	#N/A	#N/A	#N/A	#N/A
6	rc_5caCacf	-659.8215053	0.0965222	-659.7249830	-659.7001190	-0.1213863	-658.6453310	-658.8416849	-658.9626362	-659.0840225
7	rc_5caCaao	-659.8205725	0.0956765	-659.7248960	-659.6883426	-0.1322299	#N/A	#N/A	#N/A	#N/A
8	rc_5caCaap	-659.8212793	0.0968562	-659.7244230	-659.6863610	-0.1349183	-658.6332138	-658.8295856	-658.9504788	-659.0853971
9	rc_5caCacq	-659.8212622	0.0970891	-659.7241730	-659.6867837	-0.1344785	-658.6331732	-658.8297478	-658.9507367	-659.0852152
10	rc_5caCadf	-659.8202055	0.0961774	-659.7240280	-659.6897567	-0.1304488	#N/A	#N/A	#N/A	#N/A
11	rc_5caCaat	-659.8199789	0.0974719	-659.7225070	-659.6856362	-0.1343427	#N/A	#N/A	#N/A	#N/A
12	rc_5caCabh	-659.8200931	0.0976651	-659.7224280	-659.6901203	-0.1299728	#N/A	#N/A	#N/A	#N/A
13	rc_5caCabn	-659.8186524	0.0964984	-659.7221540	-659.6965078	-0.1221446	#N/A	#N/A	#N/A	#N/A
14	rc_5caCaby	-659.8185838	0.0968077	-659.7217760	-659.6957827	-0.1228011	#N/A	#N/A	#N/A	#N/A
15	rc_5caCaar	-659.8138868	0.0927607	-659.7211260	-659.6837818	-0.1301050	#N/A	#N/A	#N/A	#N/A
16	rc_5caCabo	-659.8159230	0.0952819	-659.7206410	-659.6678965	-0.1480265	#N/A	#N/A	#N/A	#N/A
17	rc_5caCadk	-659.8155696	0.0953105	-659.7202590	-659.6738178	-0.1417518	#N/A	#N/A	#N/A	#N/A
18	rc_5caCado	-659.8156928	0.0955347	-659.7201580	-659.6662803	-0.1494124	#N/A	#N/A	#N/A	#N/A
19	rc_5caCacg	-659.8157098	0.0956258	-659.7200840	-659.6659859	-0.1497239	#N/A	#N/A	#N/A	#N/A
20	rc_5caCabv	-659.8153677	0.0953687	-659.7199990	-659.6614172	-0.1539506	#N/A	#N/A	#N/A	#N/A
1	rc_1mCaar	-510.5487883	0.1113232	-510.4374650	-510.4376595	-0.1111288	-509.5767024	-509.7281633	-509.8214334	-509.9325622
2	rc_1mCaad	-510.5487896	0.1121425	-510.4366470	-510.4379460	-0.1108436	-509.5769538	-509.7284120	-509.8216824	-509.9325260
3	rc_1mCaby	-510.5479259	0.1115089	-510.4364170	-510.4323259	-0.1156001	-509.5718030	-509.7234992	-509.8168171	-509.9324172
4	rc_1mCacv	-510.5467993	0.1104892	-510.4363100	-510.4209027	-0.1258965	#N/A	#N/A	#N/A	#N/A
5	rc_1mCaau	-510.5481094	0.1118153	-510.4362940	-510.4338499	-0.1142595	#N/A	#N/A	#N/A	#N/A
6	rc_1mCadf	-510.5473127	0.1110287	-510.4362840	-510.4220299	-0.1252828	#N/A	#N/A	#N/A	#N/A
7	rc_1mCadp	-510.5488477	0.1125647	-510.4362830	-510.4305879	-0.1182598	-509.5709038	-509.7221379	-509.8152636	-509.9335234
8	rc_1mCacm	-510.5482182	0.1120231	-510.4361950	-510.4340643	-0.1141539	#N/A	#N/A	#N/A	#N/A
9	rc_1mCadu	-510.5479233	0.1118612	-510.4360620	-510.4325071	-0.1154162	#N/A	#N/A	#N/A	#N/A
10	rc_1mCaac	-510.5489534	0.1128943	-510.4360590	-510.4297537	-0.1191996	-509.5695960	-509.7210168	-509.8142225	-509.9334221
11	rc_1mCabe	-510.5489215	0.1130255	-510.4358960	-510.4301781	-0.1187434	-509.5696848	-509.7212670	-509.8145528	-509.9332962
12	rc_1mCabo	-510.5441997	0.1083147	-510.4358850	-510.4192014	-0.1249983	#N/A	#N/A	#N/A	#N/A
13	rc_1mCaaz	-510.5473307	0.1117947	-510.4355360	-510.4219214	-0.1254093	#N/A	#N/A	#N/A	#N/A
14	rc_1mCabz	-510.5467766	0.1113666	-510.4354100	-510.4214874	-0.1252893	#N/A	#N/A	#N/A	#N/A
15	rc_1mCacb	-510.5469487	0.1118147	-510.4351340	-510.4211136	-0.1258351	#N/A	#N/A	#N/A	#N/A
16	rc_1mCabp	-510.5445546	0.1096076	-510.4349470	-510.4211530	-0.1234016	#N/A	#N/A	#N/A	#N/A
17	rc_1mCaaa	-510.5436061	0.1089421	-510.4346640	-510.4137489	-0.1298572	#N/A	#N/A	#N/A	#N/A

18	rc_1mCaca	-510.5470422	0.1124621	-510.4345800	-510.4122567	-0.1347855	#N/A	#N/A	#N/A	#N/A
19	rc_1mCaao	-510.5469534	0.1125074	-510.4344460	-510.4202340	-0.1267194	#N/A	#N/A	#N/A	#N/A
20	rc_1mCadv	-510.5467185	0.1124314	-510.4342870	-510.4086250	-0.1380935	#N/A	#N/A	#N/A	#N/A
1	rc_1m5mCaaj	-549.8804769	0.1364509	-549.7440260	-549.7693591	-0.1111178	-548.8266508	-548.9903007	-549.0911231	-549.2022409
2	rc_1m5mCacs	-549.8803430	0.1363390	-549.7440040	-549.7678706	-0.1124724	-548.8252975	-548.9890112	-549.0898579	-549.2023303
3	rc_1m5mCacf	-549.8804801	0.1366261	-549.7438540	-549.7694345	-0.1110456	-548.8267273	-548.9903631	-549.0911797	-549.2022253
4	rc_1m5mCadt	-549.8805870	0.1367769	-549.7438100	-549.7580813	-0.1225057	#N/A	#N/A	#N/A	#N/A
5	rc_1m5mCacd	-549.8804357	0.1367447	-549.7436910	-549.7690570	-0.1113788	#N/A	#N/A	#N/A	#N/A
6	rc_1m5mCaab	-549.8805033	0.1370503	-549.7434530	-549.7693721	-0.1111312	#N/A	#N/A	#N/A	#N/A
7	rc_1m5mCabs	-549.8800358	0.1366808	-549.7433550	-549.7532265	-0.1268093	#N/A	#N/A	#N/A	#N/A
8	rc_1m5mCace	-549.8802147	0.1370006	-549.7432140	-549.7481700	-0.1320447	#N/A	#N/A	#N/A	#N/A
9	rc_1m5mCacg	-549.8807056	0.1376656	-549.7430400	-549.7483181	-0.1323875	-548.8051926	-548.9698196	-549.0710939	-549.2034814
10	rc_1m5mCaba	-549.8806416	0.1379855	-549.7426560	-549.7581365	-0.1225051	-548.8132194	-548.9780036	-549.0793685	-549.2018736
11	rc_1m5mCadh	-549.8766226	0.1341036	-549.7425190	-549.7537790	-0.1228436	#N/A	#N/A	#N/A	#N/A
12	rc_1m5mCaay	-549.8806090	0.1383889	-549.7422200	-549.7496029	-0.1310060	#N/A	#N/A	#N/A	#N/A
13	rc_1m5mCabh	-549.8766978	0.1344868	-549.7422110	-549.7547972	-0.1219007	#N/A	#N/A	#N/A	#N/A
14	rc_1m5mCabx	-549.8772622	0.1352751	-549.7419870	-549.7446391	-0.1326230	#N/A	#N/A	#N/A	#N/A
15	rc_1m5mCacl	-549.8811356	0.1391805	-549.7419550	-549.7631644	-0.1179712	-548.8215784	-548.9846717	-549.0852614	-549.2032326
16	rc_1m5mCaan	-549.8801355	0.1382994	-549.7418360	-549.7617924	-0.1183431	#N/A	#N/A	#N/A	#N/A
17	rc_1m5mCabb	-549.8813072	0.1397231	-549.7415840	-549.7673844	-0.1139228	-548.8245779	-548.9880462	-549.0888255	-549.2027483
18	rc_1m5mCadj	-549.8787250	0.1372399	-549.7414850	-549.7492555	-0.1294695	#N/A	#N/A	#N/A	#N/A
19	rc_1m5mCacq	-549.8765587	0.1350906	-549.7414680	-549.7589833	-0.1175754	#N/A	#N/A	#N/A	#N/A
20	rc_1m5mCade	-549.8797725	0.1383475	-549.7414250	-549.7559074	-0.1238651	#N/A	#N/A	#N/A	#N/A
1	rc_1m5hmCads	-625.1037120	0.1422009	-624.9615110	-624.9716848	-0.1320272	-623.9342032	-624.1214725	-624.2369315	-624.3689587
2	rc_1m5hmCacs	-625.1036653	0.1423243	-624.9613410	-624.9774539	-0.1262114	-623.9386374	-624.1261216	-624.2416824	-624.3678938
3	rc_1m5hmCaby	-625.1001909	0.1388598	-624.9613310	-624.9608253	-0.1393656	-623.9225368	-624.1109074	-624.2267971	-624.3661627
4	rc_1m5hmCaaq	-625.1036113	0.1425353	-624.9610760	-624.9775914	-0.1260199	-623.9386926	-624.1261863	-624.2417602	-624.3677801
5	rc_1m5hmCabj	-625.1035434	0.1425974	-624.9609460	-624.9716688	-0.1318746	#N/A	#N/A	#N/A	#N/A
6	rc_1m5hmCaaj	-625.1030819	0.1421629	-624.9609190	-624.9820333	-0.1210486	-623.9432895	-624.1307667	-624.2463457	-624.3673943
7	rc_1m5hmCacu	-625.1037096	0.1428505	-624.9608590	-624.9735841	-0.1301255	-623.9355871	-624.1230095	-624.2385347	-624.3686602
8	rc_1m5hmCabo	-625.1006069	0.1401058	-624.9605010	-624.9799218	-0.1206850	#N/A	#N/A	#N/A	#N/A
9	rc_1m5hmCadi	-625.1037407	0.1433316	-624.9604090	-624.9704262	-0.1333144	-623.9331795	-624.1203670	-624.2357856	-624.3691000
10	rc_1m5hmCadk	-625.0999819	0.1397148	-624.9602670	-624.9665775	-0.1334044	#N/A	#N/A	#N/A	#N/A
11	rc_1m5hmCacg	-625.0997413	0.1396612	-624.9600800	-624.9667487	-0.1329926	#N/A	#N/A	#N/A	#N/A
12	rc_1m5hmCaav	-625.1002287	0.1401836	-624.9600450	-624.9605110	-0.1397176	-623.9220606	-624.1105170	-624.2264553	-624.3661729
13	rc_1m5hmCabg	-625.0997883	0.1400852	-624.9597030	-624.9620694	-0.1377188	#N/A	#N/A	#N/A	#N/A
14	rc_1m5hmCadl	-625.1037241	0.1441740	-624.9595500	-624.9757046	-0.1280194	-623.9374715	-624.1248323	-624.2403537	-624.3683731
15	rc_1m5hmCabc	-625.1037834	0.1444014	-624.9593820	-624.9691438	-0.1346397	-623.9319183	-624.1189836	-624.2343409	-624.3689806
16	rc_1m5hmCact	-625.1003733	0.1414573	-624.9589160	-624.9779793	-0.1223940	#N/A	#N/A	#N/A	#N/A
17	rc_1m5hmCade	-625.0994446	0.1406305	-624.9588140	-624.9576486	-0.1417960	#N/A	#N/A	#N/A	#N/A
18	rc_1m5hmCaal	-625.1005578	0.1418488	-624.9587090	-624.9584315	-0.1421263	#N/A	#N/A	#N/A	#N/A
19	rc_1m5hmCabk	-625.1016625	0.1429814	-624.9586810	-624.9708046	-0.1308579	#N/A	#N/A	#N/A	#N/A
20	rc_1m5hmCaao	-625.1005411	0.1418830	-624.9586580	-624.9585962	-0.1419449	#N/A	#N/A	#N/A	#N/A
1	rc_1m5fCaak	-623.8718266	0.1166376	-623.7551890	-623.7574276	-0.1143990	-622.7236547	-622.9084315	-623.0220978	-623.1364968
2	rc_1m5fCacx	-623.8717729	0.1166719	-623.7551010	-623.7570902	-0.1146827	-622.7232628	-622.9080686	-623.0217485	-623.1364312
3	rc_1m5fCaar	-623.8718557	0.1167656	-623.7550900	-623.7576626	-0.1141931	-622.7239437	-622.9086744	-623.0223268	-623.1365199
4	rc_1m5fCacn	-623.8716376	0.1167456	-623.7548920	-623.7568541	-0.1147835	#N/A	#N/A	#N/A	#N/A
5	rc_1m5fCacp	-623.8719134	0.1172103	-623.7547030	-623.7572374	-0.1146760	#N/A	#N/A	#N/A	#N/A

6	rc_1m5fCadq	-623.8718369	0.1171849	-623.7546520	-623.7577810	-0.1140560	#N/A	#N/A	#N/A	#N/A
7	rc_1m5fCaag	-623.8726922	0.1187532	-623.7539390	-623.7524108	-0.1202814	#N/A	#N/A	#N/A	#N/A
8	rc_1m5fCach	-623.8727935	0.1197944	-623.7529990	-623.7504819	-0.1223115	#N/A	#N/A	#N/A	#N/A
9	rc_1m5fCaad	-623.8708725	0.1178945	-623.7529780	-623.7443357	-0.1265368	#N/A	#N/A	#N/A	#N/A
10	rc_1m5fCacd	-623.8727397	0.1197637	-623.7529760	-623.7518328	-0.1209069	#N/A	#N/A	#N/A	#N/A
11	rc_1m5fCaau	-623.8699247	0.1173517	-623.7525730	-623.7539084	-0.1160164	#N/A	#N/A	#N/A	#N/A
12	rc_1m5fCadr	-623.8702810	0.1177509	-623.7525300	-623.7427221	-0.1275588	#N/A	#N/A	#N/A	#N/A
13	rc_1m5fCaco	-623.8709837	0.1184966	-623.7524870	-623.7465754	-0.1244082	#N/A	#N/A	#N/A	#N/A
14	rc_1m5fCaah	-623.8726104	0.1202343	-623.7523760	-623.7487067	-0.1239037	#N/A	#N/A	#N/A	#N/A
15	rc_1m5fCacg	-623.8729185	0.1205675	-623.7523510	-623.7494702	-0.1234483	-622.7146246	-622.8991092	-623.0127713	-623.1362196
16	rc_1m5fCabu	-623.8729438	0.1206218	-623.7523220	-623.7481389	-0.1248049	-622.7135297	-622.8982679	-623.0120294	-623.1368343
17	rc_1m5fCabd	-623.8694790	0.1172300	-623.7522490	-623.7495974	-0.1198816	#N/A	#N/A	#N/A	#N/A
18	rc_1m5fCabj	-623.8730457	0.1208336	-623.7522120	-623.7455133	-0.1275324	-622.7113118	-622.8958135	-623.0094314	-623.1369638
19	rc_1m5fCace	-623.8711556	0.1190096	-623.7521460	-623.7368556	-0.1343001	#N/A	#N/A	#N/A	#N/A
20	rc_1m5fCads	-623.8683323	0.1162353	-623.7520970	-623.7314363	-0.1368960	#N/A	#N/A	#N/A	#N/A
1	rc_1m5dhmCadu	-700.3378441	0.1455710	-700.1922730	-700.2159646	-0.1218794	-699.0788408	-699.2901730	-699.4204310	-699.5423104
2	rc_1m5dhmCacl	-700.3343935	0.1422215	-700.1921720	-700.2121540	-0.1222395	-699.0764698	-699.2881803	-699.4184720	-699.5407115
3	rc_1m5dhmCabd	-700.3374965	0.1454174	-700.1920790	-700.2170177	-0.1204788	-699.0806043	-699.2921340	-699.4224315	-699.5429103
4	rc_1m5dhmCabv	-700.3376335	0.1455934	-700.1920400	-700.2174994	-0.1201341	-699.0804058	-699.2917846	-699.4220732	-699.5422073
5	rc_1m5dhmCabn	-700.3381881	0.1462230	-700.1919650	-700.2109806	-0.1272074	-699.0747782	-699.2858522	-699.4159817	-699.5431891
6	rc_1m5dhmCaax	-700.3382059	0.1463948	-700.1918110	-700.2113368	-0.1268691	-699.0750632	-699.2861342	-699.4162645	-699.5431336
7	rc_1m5dhmCaal	-700.3375696	0.1458906	-700.1916790	-700.2180982	-0.1194715	#N/A	#N/A	#N/A	#N/A
8	rc_1m5dhmCaae	-700.3377379	0.1463879	-700.1913500	-700.2169068	-0.1208311	-699.0808662	-699.2922765	-699.4225209	-699.5433520
9	rc_1m5dhmCabb	-700.3343802	0.1438741	-700.1905060	-700.2121842	-0.1221959	#N/A	#N/A	#N/A	#N/A
10	rc_1m5dhmCaai	-700.3346820	0.1446930	-700.1899890	-700.2143641	-0.1203179	#N/A	#N/A	#N/A	#N/A
11	rc_1m5dhmCadi	-700.3363422	0.1466001	-700.1897420	-700.2190340	-0.1173082	#N/A	#N/A	#N/A	#N/A
12	rc_1m5dhmCaaj	-700.3327113	0.1438212	-700.1888900	-700.2051840	-0.1275273	#N/A	#N/A	#N/A	#N/A
13	rc_1m5dhmCach	-700.3336599	0.1448718	-700.1887880	-700.1988232	-0.1348366	#N/A	#N/A	#N/A	#N/A
14	rc_1m5dhmCaaq	-700.3337798	0.1453097	-700.1884700	-700.2020842	-0.1316956	#N/A	#N/A	#N/A	#N/A
15	rc_1m5dhmCade	-700.3351891	0.1467860	-700.1884030	-700.1941048	-0.1410843	#N/A	#N/A	#N/A	#N/A
16	rc_1m5dhmCaar	-700.3357544	0.1474463	-700.1883080	-700.1957106	-0.1400438	#N/A	#N/A	#N/A	#N/A
17	rc_1m5dhmCaba	-700.3335772	0.1452742	-700.1883030	-700.2015603	-0.1320169	#N/A	#N/A	#N/A	#N/A
18	rc_1m5dhmCabw	-700.3317914	0.1435064	-700.1882850	-700.2036667	-0.1281247	#N/A	#N/A	#N/A	#N/A
19	rc_1m5dhmCacf	-700.3358070	0.1475669	-700.1882400	-700.1964272	-0.1393797	#N/A	#N/A	#N/A	#N/A
20	rc_1m5dhmCabc	-700.3334521	0.1454710	-700.1879810	-700.1974954	-0.1359567	#N/A	#N/A	#N/A	#N/A
1	rc_1m5caCacq	-699.1378380	0.1211630	-699.0166750	-699.0220455	-0.1157925	-697.8843428	-698.0921242	-698.2202216	-698.3360141
2	rc_1m5caCaco	-699.1375679	0.1227968	-699.0147710	-699.0084150	-0.1291529	-697.8723879	-698.0802121	-698.2082666	-698.3374195
3	rc_1m5caCack	-699.1361931	0.1215600	-699.0146330	-699.0127324	-0.1234606	-697.8744541	-698.0824402	-698.2105759	-698.3340365
4	rc_1m5caCaar	-699.1361434	0.1229413	-699.0132020	-699.0126194	-0.1235240	-697.8743822	-698.0823348	-698.2104702	-698.3339942
5	rc_1m5caCabl	-699.1358390	0.1228480	-699.0129910	-699.0087742	-0.1270648	#N/A	#N/A	#N/A	#N/A
6	rc_1m5caCacc	-699.1358432	0.1228911	-699.0129520	-699.0175158	-0.1183274	#N/A	#N/A	#N/A	#N/A
7	rc_1m5caCacw	-699.1362235	0.1233275	-699.0128960	-699.0116195	-0.1246040	-697.8733633	-698.0810474	-698.2090487	-698.3336527
8	rc_1m5caCadt	-699.1360774	0.1234393	-699.0126380	-699.0111311	-0.1249463	#N/A	#N/A	#N/A	#N/A
9	rc_1m5caCabt	-699.1361043	0.1235243	-699.0125800	-699.0126523	-0.1234521	#N/A	#N/A	#N/A	#N/A
10	rc_1m5caCacy	-699.1317450	0.1199680	-699.0117770	-698.9979335	-0.1338116	#N/A	#N/A	#N/A	#N/A
11	rc_1m5caCacm	-699.1340730	0.1224460	-699.0116270	-698.9872718	-0.1468012	#N/A	#N/A	#N/A	#N/A
12	rc_1m5caCacj	-699.1314129	0.1198049	-699.0116080	-698.9931831	-0.1382298	#N/A	#N/A	#N/A	#N/A
13	rc_1m5caCaca	-699.1333889	0.1222229	-699.0111660	-699.0020670	-0.1313219	#N/A	#N/A	#N/A	#N/A

14	rc_1m5caCabj	-699.1340743	0.1230383	-699.0110360	-698.9872282	-0.1468461	#N/A	#N/A	#N/A	#N/A
15	rc_1m5caCadk	-699.1331555	0.1222184	-699.0109370	-698.9840380	-0.1491175	#N/A	#N/A	#N/A	#N/A
16	rc_1m5caCacr	-699.1282542	0.1175632	-699.0106910	-698.9860680	-0.1421863	#N/A	#N/A	#N/A	#N/A
17	rc_1m5caCabs	-699.1315824	0.1209084	-699.0106740	-698.9941617	-0.1374208	#N/A	#N/A	#N/A	#N/A
18	rc_1m5caCaaq	-699.1327309	0.1223518	-699.0103790	-698.9862164	-0.1465144	#N/A	#N/A	#N/A	#N/A
19	rc_1m5caCaaa	-699.1325625	0.1223594	-699.0102030	-698.9917022	-0.1408603	#N/A	#N/A	#N/A	#N/A
20	rc_1m5caCaas	-699.1315966	0.1214006	-699.0101960	-698.9873892	-0.1442074	#N/A	#N/A	#N/A	#N/A
1	rc_U14_ack	-491.0974078	0.0726418	-491.0247660	-490.9804415	-0.1169664	-490.1946658	-490.3403243	-490.4299642	-490.5469306
2	rc_U14_aar	-491.0974138	0.0730237	-491.0243900	-490.9785248	-0.1188890	-490.1928475	-490.3386877	-490.4283951	-490.5472841
3	rc_U14_abi	-491.0974635	0.0732155	-491.0242480	-490.9802490	-0.1172145	-490.1945035	-490.3401698	-490.4298124	-490.5470269
4	rc_Uabn	-491.0972515	0.0732144	-491.0240370	-490.9768557	-0.1203958	-490.1914360	-490.3372533	-490.4269325	-490.5473283
5	rc_U14_aak	-491.0974985	0.0735474	-491.0239510	-490.9795252	-0.1179733	-490.1937812	-490.3395744	-490.4292681	-490.5472414
6	rc_U14_abc	-491.0971587	0.0733307	-491.0238280	-490.9756369	-0.1215218	-490.1906079	-490.3363035	-490.4259161	-490.5474379
7	rc_U14_abz	-491.0971982	0.0735652	-491.0236330	-490.9757008	-0.1214974	-490.1905658	-490.3363035	-490.4259352	-490.5474326
8	rc_U14_aal	-491.0941263	0.0731833	-491.0209430	-490.9722377	-0.1218886	#N/A	#N/A	#N/A	#N/A
9	rc_Uacq	-491.0941560	0.0736209	-491.0205350	-490.9724811	-0.1216748	-490.1865786	-490.3325838	-490.4223378	-490.5440126
10	rc_Uadu	-491.0941379	0.0737999	-491.0203380	-490.9726842	-0.1214537	#N/A	#N/A	#N/A	#N/A
11	rc_Uade	-491.0938275	0.0735475	-491.0202800	-490.9718305	-0.1219970	#N/A	#N/A	#N/A	#N/A
12	rc_Uadd	-491.0942083	0.0743272	-491.0198810	-490.9729115	-0.1212968	-490.1868268	-490.3329141	-490.4227080	-490.5440048
13	rc_Uaah	-491.0937796	0.0750935	-491.0186860	-490.9674862	-0.1262934	#N/A	#N/A	#N/A	#N/A
14	rc_Uaav	-491.0940481	0.0753670	-491.0186810	-490.9642795	-0.1297686	#N/A	#N/A	#N/A	#N/A
15	rc_Uaaa	-491.0940291	0.0754161	-491.0186130	-490.9665121	-0.1275170	#N/A	#N/A	#N/A	#N/A
16	rc_U14_abb	-491.0941454	0.0758093	-491.0183360	-490.9604559	-0.1336895	#N/A	#N/A	#N/A	#N/A
17	rc_U14_aba	-491.0941456	0.0758515	-491.0182940	-490.9595797	-0.1345659	#N/A	#N/A	#N/A	#N/A
18	rc_U14_abe	-491.0939698	0.0758117	-491.0181580	-490.9670353	-0.1269345	#N/A	#N/A	#N/A	#N/A
19	rc_Uadp	-491.0941013	0.0761093	-491.0179920	-490.9646803	-0.1294210	#N/A	#N/A	#N/A	#N/A
20	rc_Uaad	-491.0940579	0.0761989	-491.0178590	-490.9654548	-0.1286032	#N/A	#N/A	#N/A	#N/A
1	rc_5mUaaq	-530.4337928	0.0989538	-530.3348390	-530.3211002	-0.1126926	-529.4521187	-529.6098921	-529.7071199	-529.8198125
2	rc_5mUaat	-530.4337742	0.0990042	-530.3347700	-530.3205026	-0.1132717	-529.4515594	-529.6093709	-529.7066063	-529.8198780
3	rc_5mUaau	-530.4337413	0.0989773	-530.3347640	-530.3218730	-0.1118683	-529.4528680	-529.6105300	-529.7077132	-529.8195815
4	rc_5mUach	-530.4302712	0.0979832	-530.3322880	-530.3136990	-0.1165722	-529.4447297	-529.6025992	-529.6998434	-529.8164156
5	rc_5mUadt	-530.4303479	0.0984488	-530.3318990	-530.3141838	-0.1161641	-529.4447616	-529.6028844	-529.7002431	-529.8164072
6	rc_5mUaak	-530.4304359	0.0987009	-530.3317350	-530.3143976	-0.1160383	-529.4451619	-529.6031952	-529.7005222	-529.8165605
7	rc_5mUabn	-530.4303092	0.0986881	-530.3316210	-530.3149432	-0.1153660	-529.4454761	-529.6035309	-529.7008635	-529.8162295
8	rc_5mUadp	-530.4303720	0.0988569	-530.3315150	-530.3141014	-0.1162705	-529.4447531	-529.6028071	-529.7001350	-529.8164055
9	rc_5mUaab	-530.4270035	0.0965265	-530.3304770	-530.2974000	-0.1296036	#N/A	#N/A	#N/A	#N/A
10	rc_5mUaca	-530.4277829	0.0979269	-530.3298560	-530.2944943	-0.1332886	#N/A	#N/A	#N/A	#N/A
11	rc_5mUado	-530.4274235	0.0977635	-530.3296600	-530.2976719	-0.1297516	#N/A	#N/A	#N/A	#N/A
12	rc_5mUaai	-530.4229497	0.0936767	-530.3292730	-530.3014933	-0.1214564	#N/A	#N/A	#N/A	#N/A
13	rc_5mUacv	-530.4277091	0.0985810	-530.3291280	-530.2986647	-0.1290443	#N/A	#N/A	#N/A	#N/A
14	rc_5mUaba	-530.4255675	0.0966815	-530.3288860	-530.3000006	-0.1255669	#N/A	#N/A	#N/A	#N/A
15	rc_5mUacy	-530.4277720	0.0988969	-530.3288750	-530.2958445	-0.1319275	#N/A	#N/A	#N/A	#N/A
16	rc_5mUacn	-530.4286774	0.1000374	-530.3286400	-530.3051050	-0.1235724	#N/A	#N/A	#N/A	#N/A
17	rc_5mUabc	-530.4238702	0.0952711	-530.3285990	-530.2875345	-0.1363356	#N/A	#N/A	#N/A	#N/A
18	rc_5mUack	-530.4277214	0.0991884	-530.3285330	-530.2971158	-0.1306056	#N/A	#N/A	#N/A	#N/A
19	rc_5mUacm	-530.4285633	0.1001202	-530.3284430	-530.3025260	-0.1260372	#N/A	#N/A	#N/A	#N/A
20	rc_5mUacu	-530.4286077	0.1003166	-530.3282910	-530.3036200	-0.1249877	#N/A	#N/A	#N/A	#N/A
1	rc_5hmUaas	-605.6515615	0.1003704	-605.5511910	-605.5266348	-0.1249266	-604.5582465	-604.7402214	-604.8521809	-604.9771075

2	rc_5hmUaav	-605.6518287	0.1013416	-605.5504870	-605.5320911	-0.1197376	-604.5626895	-604.7446755	-604.8566915	-604.9764291
3	rc_5hmUabf	-605.6497197	0.1011076	-605.5486120	-605.5260206	-0.1236990	-604.5544206	-604.7369039	-604.8491432	-604.9728422
4	rc_5hmUaab	-605.6497288	0.1019428	-605.5477860	-605.5263018	-0.1234270	-604.5546872	-604.7371967	-604.8494400	-604.9728670
5	rc_5hmUacj	-605.6496737	0.1019487	-605.5477250	-605.5259008	-0.1237729	-604.5546396	-604.7370620	-604.8492573	-604.9730302
6	rc_5hmUaai	-605.6465904	0.1006764	-605.5459140	-605.5084114	-0.1381790	#N/A	#N/A	#N/A	#N/A
7	rc_5hmUabt	-605.6460631	0.1009001	-605.5451630	-605.5110834	-0.1349797	#N/A	#N/A	#N/A	#N/A
8	rc_5hmUaci	-605.6461370	0.1018140	-605.5443230	-605.5119042	-0.1342328	#N/A	#N/A	#N/A	#N/A
9	rc_5hmUabn	-605.6463524	0.1020643	-605.5442880	-605.5048168	-0.1415355	#N/A	#N/A	#N/A	#N/A
10	rc_5hmUaay	-605.6465330	0.1022909	-605.5442420	-605.5078928	-0.1386401	#N/A	#N/A	#N/A	#N/A
11	rc_5hmUaap	-605.6465545	0.1023164	-605.5442380	-605.5071990	-0.1393554	#N/A	#N/A	#N/A	#N/A
12	rc_5hmUacn	-605.6461984	0.1022043	-605.5439940	-605.5003829	-0.1458155	#N/A	#N/A	#N/A	#N/A
13	rc_5hmUace	-605.6479189	0.1039748	-605.5439440	-605.5051666	-0.1427522	#N/A	#N/A	#N/A	#N/A
14	rc_5hmUaam	-605.6463168	0.1023928	-605.5439240	-605.5009529	-0.1453639	#N/A	#N/A	#N/A	#N/A
15	rc_5hmUacw	-605.6463214	0.1024233	-605.5438980	-605.5028607	-0.1434607	#N/A	#N/A	#N/A	#N/A
16	rc_5hmUabb	-605.6465467	0.1026536	-605.5438930	-605.5081272	-0.1384195	#N/A	#N/A	#N/A	#N/A
17	rc_5hmUadi	-605.6461741	0.1023221	-605.5438520	-605.5127331	-0.1334411	#N/A	#N/A	#N/A	#N/A
18	rc_5hmUadf	-605.6461973	0.1023602	-605.5438370	-605.5003369	-0.1458603	#N/A	#N/A	#N/A	#N/A
19	rc_5hmUabe	-605.6478214	0.1040923	-605.5437290	-605.5197969	-0.1280245	#N/A	#N/A	#N/A	#N/A
20	rc_5hmUabo	-605.6464018	0.1028077	-605.5435940	-605.5069554	-0.1394464	#N/A	#N/A	#N/A	#N/A
1	rc_5fUabg	-604.4234998	0.0802377	-604.3432620	-604.3075560	-0.1159438	-603.3325645	-603.5116126	-603.6216404	-603.7375842
2	rc_5fUacf	-604.4234314	0.0802354	-604.3431960	-604.3081083	-0.1153232	-603.3328824	-603.5118346	-603.6218093	-603.7371325
3	rc_5fUaab	-604.4241051	0.0809170	-604.3431880	-604.3107166	-0.1133885	-603.3363875	-603.5151600	-603.6250676	-603.7384561
4	rc_5fUaaw	-604.4234313	0.0802942	-604.3431370	-604.3081128	-0.1153185	#N/A	#N/A	#N/A	#N/A
5	rc_5fUadb	-604.4239356	0.0809435	-604.3429920	-604.3067387	-0.1171969	-603.3328277	-603.5116781	-603.6215903	-603.7387872
6	rc_5fUabq	-604.4240950	0.0812029	-604.3428920	-604.3103044	-0.1137906	-603.3359457	-603.5147823	-603.6247169	-603.7385075
7	rc_5fUaai	-604.4235044	0.0806924	-604.3428120	-604.3071609	-0.1163436	-603.3322908	-603.5112992	-603.6213138	-603.7376574
8	rc_5fUaay	-604.4234791	0.0807700	-604.3427090	-604.3082667	-0.1152124	#N/A	#N/A	#N/A	#N/A
9	rc_5fUacv	-604.4239427	0.0814927	-604.3424500	-604.3058802	-0.1180626	-603.3322511	-603.5110061	-603.6208700	-603.7389326
10	rc_5fUaae	-604.4191914	0.0801603	-604.3390310	-604.2894863	-0.1297051	#N/A	#N/A	#N/A	#N/A
11	rc_5fUace	-604.4192869	0.0807119	-604.3385750	-604.2901102	-0.1291768	#N/A	#N/A	#N/A	#N/A
12	rc_5fUadk	-604.4182703	0.0802032	-604.3380670	-604.2859037	-0.1323666	#N/A	#N/A	#N/A	#N/A
13	rc_5fUabf	-604.4191003	0.0814182	-604.3376820	-604.2818705	-0.1372298	#N/A	#N/A	#N/A	#N/A
14	rc_5fUado	-604.4194098	0.0817347	-604.3376750	-604.2773553	-0.1420545	#N/A	#N/A	#N/A	#N/A
15	rc_5fUadc	-604.4194206	0.0818305	-604.3375900	-604.2914400	-0.1279805	#N/A	#N/A	#N/A	#N/A
16	rc_5fUabu	-604.4189839	0.0817409	-604.3372430	-604.2818242	-0.1371597	#N/A	#N/A	#N/A	#N/A
17	rc_5fUaaj	-604.4197272	0.0824912	-604.3372360	-604.2798540	-0.1398732	#N/A	#N/A	#N/A	#N/A
18	rc_5fUacg	-604.4178611	0.0808400	-604.3370210	-604.2822540	-0.1356071	#N/A	#N/A	#N/A	#N/A
19	rc_5fUabm	-604.4191230	0.0821499	-604.3369730	-604.2822428	-0.1368802	#N/A	#N/A	#N/A	#N/A
20	rc_5fUaas	-604.4172734	0.0803124	-604.3369610	-604.2801672	-0.1371062	#N/A	#N/A	#N/A	#N/A
1	rc_5dhmUadn	-680.8871468	0.1070128	-680.7801340	-680.7604647	-0.1266821	-679.6967962	-679.9026594	-680.0293165	-680.1559986
2	rc_5dhmUada	-680.8871079	0.1072358	-680.7798720	-680.7597127	-0.1273952	-679.6961543	-679.9020254	-680.0286776	-680.1560728
3	rc_5dhmUacw	-680.8870039	0.1072858	-680.7797180	-680.7584570	-0.1285469	-679.6950940	-679.9009576	-680.0276553	-680.1562022
4	rc_5dhmUaci	-680.8871841	0.1076290	-680.7795550	-680.7607635	-0.1264206	-679.6970971	-679.9029061	-680.0295405	-680.1559611
5	rc_5dhmUact	-680.8869344	0.1076404	-680.7792940	-680.7571934	-0.1297410	#N/A	#N/A	#N/A	#N/A
6	rc_5dhmUaan	-680.8869776	0.1076935	-680.7792840	-680.7578557	-0.1291219	-679.6946996	-679.9004758	-680.0270655	-680.1561874
7	rc_5dhmUabq	-680.8872734	0.1088354	-680.7784380	-680.7608218	-0.1264516	-679.6971435	-679.9029392	-680.0295692	-680.1560208
8	rc_5dhmUadi	-680.8843569	0.1071688	-680.7771880	-680.7545546	-0.1298023	#N/A	#N/A	#N/A	#N/A
9	rc_5dhmUadc	-680.8844595	0.1073375	-680.7771220	-680.7559252	-0.1285344	#N/A	#N/A	#N/A	#N/A

10	rc_5dhmUadq	-680.8865360	0.1094849	-680.7770510	-680.7555580	-0.1309779	#N/A	#N/A	#N/A	#N/A
11	rc_5dhmUaag	-680.8844926	0.1077356	-680.7767570	-680.7553820	-0.1291106	#N/A	#N/A	#N/A	#N/A
12	rc_5dhmUabk	-680.8845089	0.1078028	-680.7767060	-680.7554788	-0.1290300	#N/A	#N/A	#N/A	#N/A
13	rc_5dhmUaat	-680.8844907	0.1079567	-680.7765340	-680.7550675	-0.1294232	#N/A	#N/A	#N/A	#N/A
14	rc_5dhmUaca	-680.8845051	0.1081491	-680.7763560	-680.7556003	-0.1289048	#N/A	#N/A	#N/A	#N/A

4 XYZ data

1m5caC/1m5caC01.xyz
6 -1.903992 0.564797 0.004192
6 0.470067 1.170349 0.004931
6 0.826533 -0.215890 0.000659
6 -0.205509 -1.117358 -0.005551
1 0.000649 -2.180491 -0.009802
7 -1.518150 -0.767668 -0.009960
7 -0.861318 1.474213 0.009056
8 -3.079115 0.923756 0.010946
7 1.350139 2.152447 0.008533
1 1.065819 3.125408 0.012213
1 2.332653 1.865622 -0.004840
6 2.261258 -0.702323 0.000330
8 3.172139 0.185370 -0.050579
8 2.450163 -1.949372 0.050308
1 -1.147245 2.451820 0.016330
6 -2.590121 -1.778905 -0.018798
1 -3.175458 -1.700359 0.898750
1 -3.236591 -1.614389 -0.881783
1 -2.129434 -2.762606 -0.084263
1m5caC/1m5caC02.xyz
6 1.909582 0.606430 0.004516
6 -0.343899 1.241148 -0.002394
6 -0.779693 -0.138777 -0.001757
6 0.218023 -1.091726 -0.005474
1 -0.007807 -2.150588 -0.007133
7 1.517077 -0.759809 -0.008471
7 0.956642 1.563306 0.002089

8 3.130959 0.857790 0.016998
7 -1.224165 2.249144 -0.006451
1 -0.882510 3.200918 -0.004913
1 -2.217496 2.054112 -0.008637
6 -2.187062 -0.533526 0.001589
8 -3.137555 0.252748 -0.005803
8 -2.380902 -1.872662 0.015467
1 -3.339389 -2.047697 0.017572
6 2.569773 -1.785855 -0.010486
1 3.222651 -1.636933 -0.871582
1 3.158539 -1.711373 0.905482
1 2.098777 -2.766086 -0.070228
1m5caC/1m5caC03.xyz
6 -1.904035 0.617950 -0.026375
6 0.357281 1.227367 -0.016811
6 0.784615 -0.160687 0.004403
6 -0.229844 -1.095299 0.049143
1 -0.042282 -2.160681 0.109450
7 -1.525303 -0.749698 0.044823
7 -0.939578 1.561837 -0.043437
8 -3.121858 0.881074 -0.064727
7 1.242159 2.230247 -0.022480
1 0.902805 3.182761 -0.038113
1 2.232919 2.029893 0.034737
6 2.203120 -0.543509 0.011224
8 3.119633 0.263096 0.173167
8 2.558566 -1.841506 -0.157684
1 1.831815 -2.424303 -0.428872
6 -2.589372 -1.763706 0.078784
1 -3.246731 -1.576162 0.928778
1 -3.170062 -1.715448 -0.844118
1 -2.130731 -2.746771 0.177538
1m5caC/1m5caC04.xyz
6 1.891910 0.628493 0.043492
6 -0.374592 1.192360 -0.115554
6 -0.770993 -0.192444 -0.040840

6 0.252674 -1.114568 -0.021100
1 0.046646 -2.178550 -0.003826
7 1.542614 -0.745835 0.004838
7 0.908331 1.554424 -0.052308
8 3.098098 0.923795 0.142764
7 -1.281581 2.184812 -0.253379
1 -0.920584 3.117550 -0.411975
1 -2.207699 1.994800 -0.613238
6 -2.147081 -0.725074 0.010246
8 -2.457218 -1.829537 -0.421708
8 -3.122423 0.028916 0.576977
1 -2.780159 0.785735 1.083709
6 2.624786 -1.739866 0.043192
1 3.295488 -1.577838 -0.802038
1 3.186973 -1.639739 0.973118
1 2.185910 -2.734569 -0.020690
1m5caC/1m5caC05.xyz
6 -1.772129 0.553923 0.005260
6 0.435595 1.183378 0.005559
6 0.847742 -0.197099 0.000328
6 -0.155111 -1.126058 -0.008819
1 0.056002 -2.187268 -0.015430
7 -1.473526 -0.776052 -0.013374
7 -0.883521 1.514251 0.013708
8 -3.073434 0.820416 0.014081
7 1.311382 2.180020 0.007284
1 0.989785 3.139421 0.011961
1 2.299923 1.932252 -0.009236
6 2.293898 -0.660045 0.001385
8 3.190996 0.239084 -0.058485
8 2.503038 -1.904752 0.061773
1 -3.209073 1.785174 0.026433
6 -2.546665 -1.790716 -0.024024
1 -3.124732 -1.721065 0.898405
1 -3.196195 -1.623080 -0.883552
1 -2.080845 -2.771257 -0.098993

1m5caC/1m5caC06.xyz
6 -1.769289 0.584631 0.005631
6 0.447908 1.186363 0.006973
6 0.848852 -0.197579 0.001050
6 -0.162604 -1.114627 -0.006916
1 0.037369 -2.177936 -0.013593
7 -1.480510 -0.752272 -0.010315
7 -0.865462 1.530174 0.014175
8 -3.039639 0.983397 0.013148
7 1.333660 2.174509 0.010672
1 1.020855 3.136812 0.015969
1 2.319841 1.918214 -0.007113
6 2.290938 -0.674844 0.000301
8 3.195665 0.215812 -0.065291
8 2.488019 -1.921193 0.064638
1 -3.681832 0.252662 0.009872
6 -2.549271 -1.772791 -0.025843
1 -3.144422 -1.698815 0.887466
1 -3.176316 -1.635293 -0.909723
1 -2.080885 -2.753569 -0.071731
1m5caC/1m5caC07.xyz
6 1.876811 0.664093 -0.000720
6 -0.389199 1.088626 0.006110
6 -0.768783 -0.267187 0.002857
6 0.295722 -1.149504 0.000725
1 0.138308 -2.221313 -0.001602
7 1.567040 -0.716400 -0.001419
7 0.831071 1.553555 0.005767
8 3.061746 1.021428 -0.005226
7 -1.460388 2.086798 0.017510
1 -1.424776 2.646969 0.877314
1 -2.373140 1.514269 -0.027063
6 -2.196043 -0.768218 -0.003798
8 -3.117345 0.123159 -0.051470
8 -2.385604 -2.007508 0.035511
1 -1.378035 2.725526 -0.781394

6 2.696351 -1.661664 -0.008530
1 3.301780 -1.492942 -0.900367
1 3.309362 -1.496970 0.878839
1 2.302904 -2.676724 -0.009120
1m5caU/1m5caU27.xyz
6 1.908301 -0.583920 0.000953
6 -0.483586 -1.198881 -0.001211
6 -0.804773 0.210159 -0.000389
6 0.222927 1.119346 -0.001424
1 0.020661 2.184252 -0.001275
7 1.526126 0.760679 -0.002912
7 0.867684 -1.489284 -0.000961
8 3.086654 -0.929722 0.005083
8 -1.321331 -2.125363 -0.001951
1 1.133843 -2.471810 0.000073
6 -2.202107 0.687784 0.001060
8 -2.513052 1.876382 0.002946
8 -3.158303 -0.256810 0.000397
1 -2.728100 -1.156179 -0.000840
6 2.605089 1.765164 -0.001646
1 3.199821 1.660856 0.906931
1 3.240182 1.616308 -0.875688
1 2.150077 2.753002 -0.037943
1m5caU/1m5caU26.xyz
6 -1.944769 0.505605 0.000680
6 0.410422 1.315566 0.018398
6 0.833200 -0.077436 0.000343
6 -0.132797 -1.056642 -0.010907
1 0.136246 -2.104810 -0.022694
7 -1.461152 -0.803684 -0.016430
7 -0.977131 1.480228 0.015163
8 -3.148833 0.758503 0.002416
8 1.129164 2.320763 0.042774
1 -1.316762 2.439125 0.031867
6 2.257754 -0.440749 -0.014208
8 2.465223 -1.775736 0.102626

8 3.190962 0.348311 -0.120728
1 3.424602 -1.942382 0.067705
6 -2.455139 -1.891687 -0.026777
1 -3.049774 -1.851277 0.887319
1 -3.106837 -1.779283 -0.894072
1 -1.923649 -2.839861 -0.083126
1m5caU/1m5caU25.xyz
6 1.937120 0.544224 0.001591
6 -0.448077 1.269684 -0.025259
6 -0.818879 -0.137781 -0.013104
6 0.183027 -1.079311 -0.001484
1 -0.060361 -2.135023 0.003435
7 1.500719 -0.781937 0.013375
7 0.934213 1.482377 -0.018040
8 3.131336 0.840260 0.008065
8 -1.197727 2.252294 -0.048905
1 1.238265 2.453110 -0.030725
6 -2.202601 -0.644135 -0.007302
8 -2.489647 -1.836197 -0.109965
8 -3.147989 0.303456 0.134720
1 -4.019078 -0.135011 0.133845
6 2.531573 -1.834406 0.034259
1 3.124585 -1.784533 -0.880381
1 3.178992 -1.690603 0.900054
1 2.032299 -2.799170 0.102903
1m5caU/1m5caU01.xyz
6 1.935433 0.513770 -0.024428
6 -0.426627 1.293670 -0.078858
6 -0.831467 -0.103635 -0.004103
6 0.140823 -1.067932 0.073090
1 -0.106281 -2.119301 0.167759
7 1.469063 -0.795742 0.076409
7 0.955819 1.476463 -0.097760
8 3.136183 0.782734 -0.045133
8 -1.168978 2.279537 -0.147989
1 1.285081 2.436272 -0.171740

6 -2.266585 -0.452330 0.067226
8 -2.666936 -1.636925 -0.459040
8 -3.112182 0.259464 0.590971
1 -1.989093 -2.061580 -1.011690
6 2.477289 -1.868106 0.155366
1 3.068922 -1.881113 -0.761602
1 3.130674 -1.692860 1.010837
1 1.958640 -2.817568 0.275677
1m5caU/1m5caU24.xyz
6 -1.905227 -0.623495 0.002159
6 0.339085 -1.209515 -0.001740
6 0.765500 0.160634 -0.000779
6 -0.239771 1.107124 -0.002654
1 -0.026474 2.169282 -0.003483
7 -1.530052 0.747968 -0.003999
7 -0.926536 -1.571675 -0.000356
8 -3.114021 -0.899750 0.009100
8 1.232305 -2.201887 -0.004100
1 2.139019 -1.800587 -0.004053
6 2.180628 0.515837 0.001305
8 2.430686 1.832797 0.005590
8 3.086131 -0.329584 -0.000221
1 3.395099 1.979052 0.007813
6 -2.605500 1.752281 -0.004480
1 -3.240324 1.602080 -0.878661
1 -3.205086 1.643361 0.900497
1 -2.155221 2.742964 -0.037442
1m5caU/1m5caU23.xyz
6 1.899643 -0.644287 -0.003191
6 -0.363424 -1.182518 -0.016796
6 -0.760924 0.197059 -0.009760
6 0.272681 1.114113 -0.001887
1 0.073416 2.179476 0.004737
7 1.555086 0.733222 0.005451
7 0.897051 -1.565021 -0.017742
8 3.101252 -0.951513 0.003512

8 -1.248749 -2.186918 -0.029212
1 -3.964323 0.125598 0.080419
6 -2.135397 0.714578 -0.004685
8 -2.437037 1.900069 -0.073676
8 -3.069608 -0.261494 0.094000
1 -2.163360 -1.837808 -0.012487
6 2.649686 1.716147 0.028444
1 3.262184 1.555242 0.916786
1 3.267103 1.592273 -0.862376
1 2.219571 2.716119 0.049216
1m5caU/1m5caU22.xyz
6 1.768966 -0.580429 0.000724
6 -0.454726 -1.203745 -0.000472
6 -0.817444 0.204359 0.000264
6 0.184493 1.134856 -0.001021
1 -0.014556 2.199655 -0.001453
7 1.490248 0.763113 -0.002277
7 0.877059 -1.534659 0.000984
8 3.070295 -0.849497 0.003327
8 -1.328761 -2.115799 -0.002161
1 3.201818 -1.814989 0.004081
6 -2.230175 0.653708 0.001075
8 -2.568189 1.836040 0.003760
8 -3.149735 -0.319708 -0.001435
1 -2.661852 -1.205221 -0.002587
6 2.581476 1.759154 -0.002290
1 3.179690 1.643755 0.902171
1 3.207277 1.612129 -0.882946
1 2.132047 2.749780 -0.027816
1m5caU/1m5caU21.xyz
6 -1.889599 0.639442 -0.004514
6 0.370337 1.157201 0.012939
6 0.776481 -0.211186 0.007152
6 -0.260755 -1.123482 0.007279
1 -0.067157 -2.189574 0.006881
7 -1.543599 -0.736184 0.005531

7 -0.879179 1.558618 0.008394
8 -3.087374 0.952880 -0.023214
8 1.338409 2.087129 0.026121
1 0.954001 2.982572 0.017727
6 2.161646 -0.726771 -0.003099
8 2.420474 -1.926761 0.020077
8 3.181595 0.153483 -0.043555
1 2.862808 1.079341 -0.054688
6 -2.640405 -1.717798 -0.002034
1 -3.282597 -1.548174 0.863163
1 -3.226258 -1.601637 -0.915165
1 -2.212409 -2.717844 0.042827
1m5caU/1m5caU20.xyz
6 -1.901717 -0.634525 -0.000016
6 0.350074 -1.196174 -0.000040
6 0.770220 0.183069 0.000018
6 -0.253061 1.111868 0.000066
1 -0.079196 2.182328 0.000115
7 -1.539314 0.739979 0.000056
7 -0.912282 -1.569086 -0.000056
8 -3.107068 -0.922991 -0.000063
8 1.246374 -2.181947 -0.000094
1 2.151261 -1.766430 -0.000086
6 2.196750 0.522225 0.000022
8 2.618046 1.796445 0.000031
8 3.070935 -0.355830 -0.000016
1 1.911271 2.462428 0.000025
6 -2.625484 1.733318 0.000084
1 -3.241115 1.595870 -0.889882
1 -3.241252 1.595665 0.889922
1 -2.186772 2.729793 0.000236
1m5caU/1m5caU19.xyz
6 -1.914726 0.577722 0.004948
6 0.318891 1.231752 0.009663
6 0.796571 -0.117426 -0.001774
6 -0.189303 -1.086899 -0.010735

1 0.051400 -2.141977 -0.019822
7 -1.493287 -0.774118 -0.015358
7 -0.959639 1.550511 0.015089
8 -3.130165 0.822847 0.013129
8 1.208487 2.228805 0.024755
1 0.727095 3.076167 0.035168
6 2.218722 -0.491387 -0.010209
8 2.407349 -1.829942 0.086921
8 3.160919 0.288811 -0.094735
1 3.364948 -2.008582 0.062441
6 -2.530266 -1.818447 -0.028363
1 -3.119550 -1.757612 0.888074
1 -3.182499 -1.668961 -0.889618
1 -2.042964 -2.789843 -0.096100
1m5caU/1m5caU17.xyz
6 -1.762279 -0.580011 0.000081
6 0.441387 -1.141469 -0.000010
6 0.822468 0.236045 -0.000033
6 -0.192278 1.153246 0.000017
1 -0.009313 2.220619 0.000009
7 -1.494278 0.758539 0.000078
7 -0.844801 -1.524461 0.000044
8 -3.051108 -0.876016 0.000134
8 1.356797 -2.077054 -0.000068
1 -3.171821 -1.843471 0.000122
6 2.275427 0.635691 -0.000111
8 2.584513 1.846197 -0.000130
8 3.106712 -0.351436 -0.000161
1 2.267097 -1.535077 -0.000111
6 -2.601458 1.739653 0.000135
1 -3.210130 1.594962 -0.892805
1 -3.210045 1.594960 0.893133
1 -2.167159 2.736998 0.000114
1m5caU/1m5caU18.xyz
6 1.766875 -0.610345 0.000198
6 -0.466408 -1.206388 -0.000060

6 -0.818443 0.204150 0.000040
6 0.191396 1.123148 -0.000017
1 0.002719 2.189734 -0.000080
7 1.496621 0.740073 -0.000053
7 0.859566 -1.549570 0.000171
8 3.036992 -1.011632 0.000484
8 -1.350029 -2.110052 -0.000293
1 3.684324 -0.285214 0.000684
6 -2.227830 0.666165 0.000024
8 -2.555136 1.851302 0.000310
8 -3.154581 -0.299656 -0.000374
1 -2.672880 -1.189316 -0.000441
6 2.581700 1.743884 -0.000310
1 3.190217 1.627960 0.899432
1 3.191029 1.626539 -0.899310
1 2.129568 2.733393 -0.001365
1m5caU/1m5caU16.xyz
6 1.906169 0.584893 -0.030082
6 -0.333038 1.212057 -0.067166
6 -0.794757 -0.141336 0.000736
6 0.197757 -1.096626 0.071486
1 -0.022665 -2.154287 0.163991
7 1.500534 -0.766461 0.068551
7 0.939483 1.546613 -0.095200
8 3.118568 0.844562 -0.053524
8 -1.244258 2.187124 -0.141779
1 -0.784442 3.044289 -0.200900
6 -2.227752 -0.501465 0.068331
8 -2.610129 -1.694476 -0.450873
8 -3.084654 0.202915 0.583291
1 -1.923489 -2.119346 -0.992408
6 2.550886 -1.794058 0.155064
1 3.147132 -1.781682 -0.758925
1 3.194833 -1.585098 1.010263
1 2.076712 -2.766737 0.277383
1m5caU/1m5caU13.xyz

6 1.760786 -0.607277 -0.000004
6 -0.450904 -1.145162 0.000003
6 -0.824297 0.234488 -0.000002
6 0.197428 1.141126 0.000001
1 0.023016 2.209778 0.000002
7 1.499599 0.736953 0.000004
7 0.829261 -1.538009 -0.000003
8 3.013982 -1.037561 -0.000014
8 -1.373401 -2.075652 0.000010
1 3.683262 -0.330391 -0.000033
6 -2.274381 0.647728 -0.000004
8 -2.569962 1.861893 -0.000015
8 -3.114226 -0.330555 0.000006
1 -2.277409 -1.533286 0.000011
6 2.599335 1.728158 0.000015
1 3.204751 1.599226 0.899728
1 3.204734 1.599268 -0.899715
1 2.160675 2.723437 0.000044
1m5caU/1m5caU15.xyz
6 1.808405 0.515719 0.005293
6 -0.377529 1.329929 -0.034572
6 -0.843916 -0.060115 -0.006374
6 0.093311 -1.062092 -0.001324
1 -0.176499 -2.109402 0.011346
7 1.425160 -0.802523 -0.003732
7 0.996528 1.530370 -0.012036
8 3.130872 0.679130 0.036950
8 -1.129879 2.321923 -0.081889
1 3.335130 1.631591 0.046552
6 -2.276769 -0.408195 0.024622
8 -2.501348 -1.741369 -0.095111
8 -3.202151 0.386513 0.149230
1 -3.461809 -1.897374 -0.044001
6 2.426679 -1.887295 0.010068
1 3.091583 -1.778465 -0.847081
1 3.002215 -1.842571 0.935702

1 1.896524 -2.835994 -0.051861
1m5caU/1m5caU14.xyz
6 -1.800204 0.548072 0.007355
6 0.414717 1.285056 -0.039322
6 0.830290 -0.120910 -0.013774
6 -0.142751 -1.087235 -0.006790
1 0.101767 -2.141942 0.001921
7 -1.463833 -0.783264 -0.002826
7 -0.952955 1.533244 -0.012517
8 -3.116304 0.757174 0.042395
8 1.198546 2.252140 -0.089961
1 -3.287877 1.715951 0.050914
6 2.225371 -0.610240 0.001928
8 2.527453 -1.798834 -0.102825
8 3.161735 0.343384 0.156688
1 4.035292 -0.090419 0.165162
6 -2.503286 -1.831414 0.014935
1 -3.070444 -1.770294 0.944768
1 -3.169926 -1.696129 -0.836999
1 -2.007556 -2.797921 -0.054739
1m5caU/1m5caU12.xyz
6 -1.895457 0.620669 0.039126
6 0.353927 1.184949 -0.133761
6 0.773798 -0.180240 -0.037841
6 -0.244003 -1.112494 -0.011744
1 -0.030841 -2.175179 0.010447
7 -1.534239 -0.748952 0.014037
7 -0.908037 1.550844 -0.086977
8 -3.095009 0.917577 0.149729
8 1.211086 2.205546 -0.323771
1 2.120228 1.915745 -0.508090
6 2.163357 -0.686858 0.008620
8 2.517074 -1.723336 -0.534378
8 3.083254 0.035691 0.693692
1 2.681979 0.653833 1.330029
6 -2.614142 -1.746705 0.064480

1 -3.166478 -1.643564 0.999802
1 -3.291484 -1.583718 -0.774972
1 -2.173598 -2.740109 -0.002093
1m5caU/1m5caU08.xyz
6 1.908106 0.532746 -0.001988
6 -0.449222 1.180527 -0.023422
6 -0.853327 -0.171497 -0.006822
6 0.168558 -1.101211 0.008632
1 -0.069456 -2.157598 0.016182
7 1.481151 -0.789551 0.021106
7 0.876217 1.462910 -0.027192
8 3.085986 0.865038 -0.002135
8 -1.308587 2.171743 -0.049245
1 1.181518 2.436458 -0.049217
6 -2.297250 -0.665453 0.005709
8 -2.452850 -1.910908 -0.155041
8 -3.210603 0.186805 0.181633
1 -0.895075 3.055771 -0.052968
6 2.528995 -1.828182 0.045871
1 3.119922 -1.764684 -0.868965
1 3.171106 -1.667082 0.912400
1 2.043676 -2.799387 0.115604
1m5caU/1m5caU09.xyz
6 1.798021 0.521516 -0.023466
6 -0.394564 1.308073 -0.088937
6 -0.841654 -0.085665 -0.003636
6 0.099717 -1.073976 0.072983
1 -0.153362 -2.123570 0.164421
7 1.431907 -0.795583 0.073390
7 0.973827 1.527085 -0.096825
8 3.118611 0.702250 -0.037291
8 -1.173879 2.276799 -0.177710
1 3.310354 1.653513 -0.123721
6 -2.283568 -0.420063 0.075370
8 -2.704200 -1.576265 -0.497065
8 -3.112602 0.274838 0.644678

1 -2.026720 -1.993961 -1.055648
6 2.446476 -1.865429 0.161883
1 3.041376 -1.882844 -0.752285
1 3.092230 -1.687479 1.022156
1 1.925987 -2.813896 0.283034
1m5caU/1m5caU11.xyz
6 -1.806778 0.548822 0.003462
6 0.391320 1.334272 -0.030670
6 0.845606 -0.059659 -0.003252
6 -0.101166 -1.049820 0.005266
1 0.157423 -2.099866 0.020106
7 -1.432860 -0.777240 0.003776
7 -0.978403 1.549857 -0.013079
8 -3.110187 0.842572 0.022152
8 1.155488 2.317877 -0.074688
1 -3.686665 0.060364 0.034127
6 2.275329 -0.424434 0.022657
8 2.483977 -1.758732 -0.108048
8 3.209142 0.359188 0.151153
1 3.442610 -1.926522 -0.059398
6 -2.429447 -1.866820 0.017180
1 -3.023026 -1.815953 0.933651
1 -3.070816 -1.792222 -0.864677
1 -1.897213 -2.815524 -0.011109
1m5dhmC/1m5dhmC05.xyz
6 1.947209 0.657288 0.000000
6 -0.356426 1.073017 0.000002
6 -0.660499 -0.333614 -0.000002
6 0.408375 -1.179863 -0.000001
1 0.281105 -2.256815 -0.000003
7 1.688728 -0.725229 0.000003
7 0.904936 1.522413 0.000002
8 3.140826 1.040509 -0.000005
6 -2.057768 -0.886785 -0.000004
1 -2.028036 -1.982023 -0.000013
7 -1.336537 1.993422 0.000010

1 -1.094986 2.974647 0.000005
1 -2.309123 1.723195 -0.000001
8 -2.727406 -0.430909 -1.172414
1 -3.561342 -0.919286 -1.256477
8 -2.727407 -0.430926 1.172411
1 -3.561332 -0.919319 1.256477
6 2.828101 -1.652021 0.000000
1 3.440944 -1.489126 -0.888716
1 3.440967 -1.489101 0.888696
1 2.449852 -2.673943 0.000020
1m5dhmC/1m5dhmC01.xyz
6 1.977188 0.574379 0.021924
6 -0.258396 1.248272 -0.117988
6 -0.725233 -0.112687 -0.127336
6 0.238824 -1.075511 -0.048554
1 -0.003189 -2.129796 -0.060863
7 1.558828 -0.765570 0.043610
7 1.042829 1.553510 -0.049854
8 3.206428 0.817473 0.077043
6 -2.190808 -0.448268 -0.291746
1 -2.562923 -0.082234 -1.255781
7 -1.129268 2.275538 -0.210438
1 -0.770673 3.216337 -0.113150
1 -2.112493 2.115584 -0.035347
8 -3.006109 0.219924 0.668059
1 -2.651117 0.035830 1.553652
8 -2.349312 -1.849792 -0.208979
1 -3.244855 -2.065734 -0.510524
6 2.580716 -1.818273 0.108990
1 3.213888 -1.779373 -0.780150
1 3.201214 -1.678064 0.995706
1 2.081614 -2.785220 0.162504
1m5dhmC/1m5dhmC03.xyz
6 -1.941666 0.661089 0.046006
6 0.360861 1.077843 -0.014018
6 0.665155 -0.325907 -0.106160

6 -0.406920 -1.169550 -0.135535
1 -0.282182 -2.244588 -0.206176
7 -1.685736 -0.716103 -0.069644
7 -0.897534 1.524855 0.061394
8 -3.132787 1.042582 0.125445
6 2.059310 -0.901267 -0.079658
1 2.025337 -1.963483 -0.337935
7 1.345542 1.999314 0.026710
1 1.089700 2.977543 0.017105
1 2.290730 1.741368 -0.225569
8 2.649439 -0.872933 1.216894
1 2.621782 0.032490 1.567287
8 2.862217 -0.191661 -1.013748
1 3.742795 -0.599172 -1.022479
6 -2.825537 -1.642432 -0.088832
1 -3.391159 -1.554864 0.840954
1 -3.482413 -1.408752 -0.928807
1 -2.448670 -2.659561 -0.193146
1m5dhmC/1m5dhmC06.xyz
6 1.981733 0.572075 0.020384
6 -0.252696 1.250553 -0.117668
6 -0.722153 -0.108925 -0.117740
6 0.239199 -1.073610 -0.038010
1 -0.006133 -2.127217 -0.044927
7 1.560392 -0.766631 0.046662
7 1.049473 1.552925 -0.054888
8 3.211604 0.812856 0.073690
6 -2.185059 -0.445545 -0.269877
1 -2.570086 -0.062581 -1.224434
7 -1.120574 2.280269 -0.218668
1 -0.757488 3.219597 -0.123178
1 -2.102069 2.127888 -0.029219
8 -2.895666 0.190178 0.793083
1 -3.834728 0.224796 0.552120
8 -2.350212 -1.849062 -0.227172
1 -3.216454 -2.063209 -0.604966

6 2.579709 -1.821637 0.114504
1 3.214850 -1.784334 -0.773256
1 3.198690 -1.682773 1.002550
1 2.078183 -2.787355 0.167199
1m5dhmC/1m5dhmC02.xyz
6 1.978808 0.581216 0.034425
6 -0.258788 1.249309 -0.105763
6 -0.723163 -0.113530 -0.118385
6 0.245682 -1.072373 -0.062694
1 0.011149 -2.128742 -0.097940
7 1.565565 -0.760110 0.025390
7 1.041487 1.557938 -0.032644
8 3.206458 0.827496 0.107990
6 -2.186069 -0.467365 -0.298556
1 -2.533299 -0.148111 -1.288508
7 -1.131550 2.274215 -0.204985
1 -0.775256 3.215794 -0.106562
1 -2.113140 2.110307 -0.023868
8 -2.924813 0.214187 0.708949
1 -3.864446 0.172807 0.469861
8 -2.438030 -1.856868 -0.259494
1 -2.209942 -2.188328 0.625123
6 2.590158 -1.810371 0.079312
1 3.265347 -1.714757 -0.773334
1 3.166568 -1.722449 1.002343
1 2.095826 -2.780664 0.048972
1m5dhmC/1m5dhmC07.xyz
6 -1.943579 0.661937 0.004115
6 0.360881 1.072983 -0.007686
6 0.662216 -0.334467 -0.026662
6 -0.409109 -1.178301 -0.028197
1 -0.283835 -2.255650 -0.037973
7 -1.688467 -0.721200 -0.015998
7 -0.899411 1.525028 0.005997
8 -3.136248 1.047713 0.020391
6 2.059355 -0.895693 -0.016185

1 2.013325 -1.991908 -0.022229
7 1.343311 1.990462 -0.003099
1 1.104462 2.972193 0.016092
1 2.315108 1.717824 -0.010929
8 2.813176 -0.465964 1.126606
1 2.419967 -0.864301 1.919496
8 2.755932 -0.422799 -1.149931
1 3.619572 -0.864369 -1.179842
6 -2.829922 -1.645510 -0.013571
1 -3.426844 -1.499291 0.888857
1 -3.457771 -1.463905 -0.888003
1 -2.453943 -2.667928 -0.041180
1m5dhmC/1m5dhmC04.xyz
6 1.970582 0.585497 0.037585
6 -0.268689 1.245698 -0.114376
6 -0.728859 -0.119354 -0.130707
6 0.244604 -1.074685 -0.076560
1 0.015208 -2.131942 -0.117913
7 1.562525 -0.757759 0.019657
7 1.030144 1.558794 -0.032853
8 3.196652 0.835651 0.121128
6 -2.196093 -0.473052 -0.306420
1 -2.548567 -0.156942 -1.292402
7 -1.143528 2.267591 -0.215718
1 -0.792426 3.211612 -0.123352
1 -2.129309 2.100168 -0.062929
8 -3.018486 0.229625 0.619120
1 -2.648691 0.121925 1.511238
8 -2.437137 -1.864091 -0.254163
1 -2.178281 -2.198567 0.620462
6 2.591123 -1.804082 0.075596
1 3.272403 -1.700784 -0.771244
1 3.160127 -1.719128 1.003507
1 2.101297 -2.776329 0.035643
1m5dhmC/1m5dhmC10.xyz
6 1.936509 0.667786 0.005402

6 -0.369150 1.071003 0.000174
6 -0.665978 -0.337783 -0.010083
6 0.408668 -1.178053 -0.013956
1 0.286529 -2.255994 -0.021556
7 1.686346 -0.716713 -0.007779
7 0.889401 1.527620 0.007481
8 3.127791 1.057631 0.014206
6 -2.064181 -0.907078 -0.006712
1 -2.002537 -2.003797 -0.025810
7 -1.355815 1.983652 0.004721
1 -1.121809 2.966784 0.004239
1 -2.325926 1.705709 -0.015814
8 -2.850924 -0.443874 -1.096717
1 -2.392564 -0.665849 -1.922004
8 -2.826567 -0.484461 1.116545
1 -2.395829 -0.817764 1.918742
6 2.831491 -1.636580 -0.009796
1 3.449482 -1.460620 -0.892403
1 3.437252 -1.479847 0.884886
1 2.459318 -2.660682 -0.023680
1m5dhmC/1m5dhmC09.xyz
6 1.935493 0.665937 0.051301
6 -0.368391 1.073924 -0.003246
6 -0.667918 -0.331365 -0.089118
6 0.407637 -1.170974 -0.125600
1 0.286462 -2.246419 -0.199180
7 1.684744 -0.712673 -0.064791
7 0.888304 1.525998 0.068650
8 3.125279 1.051776 0.127508
6 -2.063937 -0.912726 -0.071168
1 -2.018644 -1.970260 -0.349095
7 -1.357279 1.990783 0.032071
1 -1.107206 2.970421 0.015788
1 -2.301486 1.723518 -0.215368
8 -2.955578 -0.216718 -0.950904
1 -2.581559 -0.228182 -1.846997

8 -2.663135 -0.890224 1.208372
1 -2.608960 0.004179 1.582499
6 2.829141 -1.633123 -0.096533
1 3.476561 -1.395243 -0.942691
1 3.403310 -1.543363 0.827747
1 2.456452 -2.652116 -0.197834
1m5dhmC/1m5dhmC08.xyz
6 1.975950 0.570594 0.015130
6 -0.254907 1.259479 -0.119562
6 -0.731789 -0.098033 -0.112671
6 0.224427 -1.066088 -0.020120
1 -0.028110 -2.118605 -0.014664
7 1.547601 -0.765610 0.062385
7 1.048528 1.556469 -0.061098
8 3.207480 0.805224 0.053474
6 -2.202241 -0.433840 -0.281246
1 -2.571452 -0.039306 -1.235239
7 -1.120226 2.290977 -0.213630
1 -0.757305 3.231543 -0.131544
1 -2.106005 2.142572 -0.045648
8 -3.003468 0.184158 0.706814
1 -2.605957 0.022831 1.578012
8 -2.440878 -1.833450 -0.220592
1 -2.240965 -2.226671 -1.084393
6 2.561764 -1.825708 0.129102
1 3.182579 -1.805082 -0.769478
1 3.195957 -1.678327 1.004805
1 2.055635 -2.787692 0.203194
1m5dhmU/1m5dhmU01.xyz
6 1.954231 0.614596 0.000003
6 -0.478015 1.073135 -0.000009
6 -0.704565 -0.352921 -0.000006
6 0.368873 -1.186595 0.000007
1 0.249557 -2.264360 0.000016
7 1.665250 -0.738990 0.000008
7 0.856298 1.451490 -0.000018

8 3.110137 1.050809 0.000013
8 -1.379143 1.934797 -0.000047
1 1.061875 2.447533 -0.000029
6 -2.121993 -0.877766 -0.000005
8 -2.829309 -0.494314 1.171338
1 -2.801796 0.475259 1.240814
8 -2.829320 -0.494300 -1.171337
1 -2.801810 0.475273 -1.240798
1 -2.113650 -1.968521 -0.000010
6 2.803613 -1.672807 0.000042
1 3.413577 -1.512093 -0.890606
1 3.413492 -1.512141 0.890757
1 2.416128 -2.690227 -0.000006
1m5dhmU/1m5dhmU69.xyz
6 -1.964767 0.598511 0.051129
6 0.461839 1.101171 0.025022
6 0.710723 -0.316524 -0.091082
6 -0.351338 -1.164645 -0.135542
1 -0.213982 -2.236443 -0.226790
7 -1.653433 -0.744776 -0.068965
7 -0.881115 1.451126 0.084043
8 -3.127367 1.010249 0.121835
8 1.337573 1.987716 0.058611
1 -1.101838 2.440748 0.163705
6 2.127208 -0.836216 -0.137232
8 2.952552 -0.099339 -1.022003
1 2.854932 0.842860 -0.788199
8 2.640608 -0.796791 1.191317
1 3.510018 -1.228369 1.184170
1 2.137827 -1.863458 -0.513479
6 -2.773119 -1.699615 -0.107367
1 -3.335261 -1.647877 0.826867
1 -3.433580 -1.461454 -0.942799
1 -2.366472 -2.701216 -0.236662
1m5dhmU/1m5dhmU68.xyz
6 2.006731 0.444986 0.036995

6 -0.296659 1.346193 -0.169259
6 -0.781326 -0.016464 -0.153856
6 0.119133 -1.031236 -0.054894
1 -0.194634 -2.067057 -0.047547
7 1.471996 -0.830677 0.033865
7 1.086899 1.465704 -0.067311
8 3.221392 0.659001 0.123633
8 -0.998193 2.367564 -0.266052
1 1.471498 2.407057 -0.074512
6 -2.272434 -0.242221 -0.214197
8 -2.511966 -1.597420 -0.541747
1 -3.463475 -1.700498 -0.694405
8 -2.909822 0.111845 1.009175
1 -2.466598 -0.360977 1.733001
1 -2.725056 0.420253 -0.957957
6 2.413039 -1.960080 0.106198
1 3.117460 -1.905771 -0.725652
1 2.962214 -1.926930 1.049069
1 1.844134 -2.886257 0.046138
1m5dhmU/1m5dhmU66.xyz
6 1.954996 0.606881 0.054228
6 -0.473127 1.097503 0.014948
6 -0.714251 -0.321439 -0.108217
6 0.352158 -1.164411 -0.151977
1 0.221005 -2.236503 -0.250357
7 1.651892 -0.737265 -0.075068
7 0.866760 1.454302 0.086936
8 3.114845 1.024843 0.133109
8 -1.355288 1.977652 0.043124
1 1.082476 2.444761 0.170389
6 -2.130991 -0.848853 -0.139808
8 -2.732701 -0.782626 1.161800
1 -2.176248 -1.279422 1.782447
8 -2.967662 -0.125156 -1.010320
1 -2.857842 0.820757 -0.797064
1 -2.132152 -1.883954 -0.496082

6 2.778318 -1.683893 -0.121404
1 3.434871 -1.436383 -0.957296
1 3.342526 -1.633216 0.811602
1 2.378636 -2.687739 -0.255037
1m5dhmU/1m5dhmU65.xyz
6 2.009917 0.452651 0.035376
6 -0.299693 1.337313 -0.169664
6 -0.775090 -0.027871 -0.141939
6 0.131328 -1.036008 -0.039359
1 -0.176139 -2.073625 -0.023536
7 1.483342 -0.826307 0.042429
7 1.083345 1.466746 -0.073411
8 3.223495 0.674920 0.116853
8 -1.008268 2.353311 -0.273888
1 1.461735 2.410491 -0.088751
6 -2.260579 -0.263359 -0.205373
8 -2.502542 -1.621360 -0.523360
1 -3.439642 -1.713806 -0.751470
8 -2.807309 0.074645 1.068062
1 -3.752463 0.261287 0.954693
1 -2.712754 0.383605 -0.965813
6 2.431611 -1.949461 0.118098
1 3.132891 -1.895589 -0.716433
1 2.983687 -1.907893 1.058927
1 1.867911 -2.879252 0.065091
1m5dhmU/1m5dhmU62.xyz
6 1.997787 0.503497 0.034784
6 -0.346722 1.280039 -0.151277
6 -0.761347 -0.103527 -0.151754
6 0.189003 -1.071331 -0.068175
1 -0.068061 -2.122559 -0.076548
7 1.529745 -0.797517 0.028567
7 1.025314 1.476940 -0.063147
8 3.199663 0.780639 0.119480
8 -1.114347 2.259175 -0.218759
1 1.357311 2.438259 -0.059259

6 -2.240968 -0.386880 -0.272666
8 -2.459212 -1.770615 -0.180718
1 -3.364083 -1.953120 -0.476768
8 -2.977156 0.237186 0.788120
1 -2.879584 1.199459 0.679389
1 -2.615982 -0.000434 -1.230149
6 2.527016 -1.877377 0.104424
1 3.212955 -1.806860 -0.741667
1 3.090272 -1.798146 1.035960
1 2.001556 -2.830169 0.074101
1m5dhmU/1m5dhmU67.xyz
6 2.008312 0.468135 0.050352
6 -0.310931 1.325795 -0.161052
6 -0.772924 -0.046016 -0.141437
6 0.148678 -1.042036 -0.065189
1 -0.138466 -2.086090 -0.080092
7 1.498129 -0.816832 0.026109
7 1.069321 1.471785 -0.047273
8 3.218325 0.704252 0.146277
8 -1.028881 2.333507 -0.275915
1 1.435506 2.420500 -0.050721
6 -2.261156 -0.297385 -0.246513
8 -2.595502 -1.653156 -0.474065
1 -2.400631 -2.160696 0.331557
8 -2.855691 0.177740 0.952529
1 -3.813320 0.241046 0.807909
1 -2.664512 0.245439 -1.107974
6 2.459159 -1.929692 0.088415
1 3.162505 -1.856400 -0.742881
1 3.007495 -1.894921 1.031704
1 1.906437 -2.865109 0.020578
1m5dhmU/1m5dhmU64.xyz
6 1.999485 0.471179 0.052022
6 -0.321171 1.323476 -0.169229
6 -0.780709 -0.050188 -0.155416
6 0.144912 -1.043893 -0.082856

1 -0.137793 -2.088866 -0.107045
7 1.492953 -0.815158 0.017972
7 1.058056 1.472342 -0.046524
8 3.208096 0.710203 0.156755
8 -1.039728 2.330079 -0.285973
1 1.421332 2.422209 -0.044524
6 -2.272528 -0.303195 -0.253359
8 -2.594727 -1.666346 -0.454737
1 -2.376560 -2.169187 0.347492
8 -2.954162 0.227100 0.874089
1 -2.471446 -0.025653 1.677847
1 -2.681514 0.223852 -1.118460
6 2.456895 -1.925525 0.081303
1 3.164842 -1.846875 -0.745570
1 2.999663 -1.892954 1.027872
1 1.907278 -2.862212 0.006401
1m5dhmU/1m5dhmU61.xyz
6 2.010053 0.448181 0.034678
6 -0.294337 1.347804 -0.163703
6 -0.778389 -0.013739 -0.125462
6 0.121234 -1.027317 -0.025452
1 -0.198675 -2.061588 -0.008085
7 1.475039 -0.827327 0.050021
7 1.090137 1.468753 -0.074265
8 3.225542 0.662543 0.109273
8 -0.997693 2.367321 -0.270702
1 1.475688 2.409516 -0.096014
6 -2.267703 -0.254235 -0.200344
8 -2.587710 -1.614889 -0.469880
1 -2.454100 -1.784174 -1.416269
8 -2.822796 0.067435 1.058770
1 -3.784614 0.146250 0.955259
1 -2.705974 0.384848 -0.977324
6 2.415914 -1.956569 0.124583
1 3.114632 -1.909343 -0.712513
1 2.971644 -1.916825 1.063375

1 1.845783 -2.882697 0.075794
1m5dhmU/1m5dhmU59.xyz
6 -1.981691 0.578631 0.000864
6 0.443744 1.130060 -0.004135
6 0.716181 -0.292747 -0.001568
6 -0.332965 -1.158610 -0.002824
1 -0.175613 -2.231336 -0.001959
7 -1.643963 -0.763586 -0.005828
7 -0.914168 1.449517 -0.003084
8 -3.153733 0.971393 0.008592
8 1.283967 2.043762 -0.006952
1 -1.153540 2.437776 -0.001591
6 2.125679 -0.817234 0.003593
8 2.786640 -0.361636 -1.171032
1 3.606070 -0.870215 -1.273213
8 2.779708 -0.352152 1.178466
1 3.599929 -0.857826 1.288209
1 2.111203 -1.913125 0.007745
6 -2.742059 -1.743088 -0.000481
1 -3.323031 -1.646076 0.918623
1 -3.391549 -1.572050 -0.860426
1 -2.312539 -2.741671 -0.060285
1m5dhmU/1m5dhmU63.xyz
6 1.993320 0.515160 0.038892
6 -0.356735 1.275166 -0.131406
6 -0.763943 -0.110876 -0.149960
6 0.196284 -1.070783 -0.085241
1 -0.050396 -2.124952 -0.116397
7 1.534587 -0.789106 0.016205
7 1.012748 1.482293 -0.044314
8 3.192808 0.800239 0.125630
8 -1.132632 2.250205 -0.181929
1 1.336790 2.446280 -0.027140
6 -2.244165 -0.419919 -0.306646
8 -2.540650 -1.787960 -0.201027
1 -2.298464 -2.095774 0.688355

8 -3.018090 0.270402 0.676884
1 -2.782546 1.214696 0.618023
1 -2.576577 -0.123342 -1.308899
6 2.538997 -1.862932 0.082670
1 3.225372 -1.779730 -0.761840
1 3.100296 -1.788500 1.015773
1 2.020145 -2.818977 0.042572
1m5dhmU/1m5dhmU58.xyz
6 1.979864 0.581877 0.004122
6 -0.446359 1.131173 -0.000002
6 -0.717457 -0.291901 -0.026558
6 0.333045 -1.156420 -0.032108
1 0.176672 -2.229411 -0.046736
7 1.643972 -0.760402 -0.018837
7 0.911356 1.451910 0.008031
8 3.151506 0.976046 0.020370
8 -1.287636 2.043691 0.015544
1 1.150231 2.440201 0.022673
6 -2.128200 -0.822397 -0.023194
8 -2.864894 -0.400026 1.129614
1 -2.609659 -0.965331 1.875742
8 -2.805631 -0.327486 -1.157541
1 -3.663234 -0.777619 -1.214267
1 -2.100430 -1.919160 -0.041545
6 2.742837 -1.738955 -0.013073
1 3.396587 -1.563743 -0.868997
1 3.319164 -1.646008 0.909368
1 2.314224 -2.737537 -0.079610
1m5dhmU/1m5dhmU57.xyz
6 -1.973649 0.586912 -0.000684
6 0.454366 1.130329 0.003607
6 0.721961 -0.294247 0.001011
6 -0.331715 -1.155736 0.002185
1 -0.177463 -2.229359 0.001253
7 -1.641497 -0.756800 0.005303
7 -0.902856 1.454244 0.002885

8 -3.144187 0.984405 -0.007590
8 1.297021 2.041500 0.006218
1 -1.139636 2.443180 0.001755
6 2.134009 -0.835599 -0.003536
8 2.891173 -0.380370 -1.113821
1 2.490451 -0.736876 -1.921493
8 2.895808 -0.389363 1.107216
1 2.494861 -0.747748 1.913940
1 2.087995 -1.933644 -0.007826
6 -2.743367 -1.732117 0.000295
1 -3.390765 -1.560464 0.861722
1 -3.325713 -1.631417 -0.917534
1 -2.317401 -2.732415 0.057403
1m5dhmU/1m5dhmU53.xyz
6 -1.930307 0.667979 0.003218
6 0.359035 1.024310 0.001280
6 0.670783 -0.362828 -0.009484
6 -0.413544 -1.196569 -0.012608
1 -0.306912 -2.275604 -0.020034
7 -1.682934 -0.715125 -0.005694
7 -0.858908 1.517550 0.005978
8 -3.107214 1.076635 0.008458
8 1.404473 1.872997 0.006958
1 1.081612 2.792012 0.011119
6 2.084195 -0.903098 -0.011932
8 2.812680 -0.512005 -1.166600
1 2.813569 0.457496 -1.228689
8 2.789582 -0.577429 1.177708
1 2.782890 0.386212 1.299434
1 2.051176 -1.992971 -0.041766
6 -2.843203 -1.619558 -0.006329
1 -3.447285 -1.442724 0.885347
1 -3.452710 -1.435373 -0.892803
1 -2.487374 -2.649013 -0.011665
1m5dhmU/1m5dhmU51.xyz
6 -1.937888 0.657736 0.031626

6 0.350733 1.037113 0.016243
6 0.671473 -0.347112 -0.039174
6 -0.406093 -1.189389 -0.066667
1 -0.288239 -2.266164 -0.116027
7 -1.679310 -0.721864 -0.033849
7 -0.874476 1.515417 0.047787
8 -3.118391 1.054768 0.069549
8 1.379102 1.904258 0.024377
1 1.033562 2.814993 0.035174
6 2.082068 -0.881693 -0.064848
8 2.865192 -0.348892 -1.121751
1 2.886717 0.618761 -1.031165
8 2.674963 -0.605803 1.197453
1 3.539894 -1.045272 1.221665
1 2.059408 -1.960224 -0.246882
6 -2.830438 -1.637188 -0.062205
1 -3.423908 -1.508394 0.844654
1 -3.453369 -1.418851 -0.931402
1 -2.463615 -2.661183 -0.120451
1m5dhmU/1m5dhmU55.xyz
6 1.932011 0.672133 0.081308
6 -0.355875 1.064459 0.001820
6 -0.665314 -0.321310 -0.149560
6 0.412721 -1.163358 -0.194201
1 0.294859 -2.235328 -0.309405
7 1.681500 -0.701552 -0.089804
7 0.872268 1.528887 0.115716
8 3.112434 1.060610 0.187864
8 -1.326715 1.997001 0.034976
1 -2.182707 1.578157 -0.212372
6 -2.069050 -0.875837 -0.152725
8 -2.583721 -1.077038 1.153191
1 -2.485370 -0.255035 1.662347
8 -2.887947 0.039544 -0.879589
1 -3.811280 -0.251441 -0.807700
1 -2.092716 -1.862870 -0.621667

6 2.834650 -1.612098 -0.149053
1 3.471673 -1.345758 -0.994525
1 3.411280 -1.532119 0.774036
1 2.470628 -2.631830 -0.269176
1m5dhmU/1m5dhmU56.xyz
6 -1.970184 -0.618089 -0.035215
6 0.273213 -1.212147 0.063040
6 0.713759 0.146594 0.123192
6 -0.284069 1.083709 0.095274
1 -0.073779 2.144566 0.151874
7 -1.589433 0.733719 0.005236
7 -0.994381 -1.569764 -0.009339
8 -3.184165 -0.900203 -0.100334
8 1.152813 -2.229470 0.081386
1 2.059936 -1.856393 -0.019970
6 2.160206 0.553607 0.334503
8 2.976204 -0.364647 -0.394835
1 3.881404 -0.312745 -0.048175
8 2.416954 1.885210 -0.046786
1 2.156268 2.001482 -0.976495
1 2.424682 0.504020 1.395856
6 -2.648256 1.752118 -0.027958
1 -3.216770 1.663031 -0.955432
1 -3.320561 1.609464 0.819894
1 -2.186940 2.737021 0.028693
1m5dhmU/1m5dhmU49.xyz
6 -1.980156 0.524499 0.043081
6 0.213313 1.255306 -0.155013
6 0.747357 -0.062292 -0.151823
6 -0.180911 -1.063990 -0.064119
1 0.099021 -2.109134 -0.070394
7 -1.508172 -0.797835 0.026808
7 -1.070060 1.537389 -0.058367
8 -3.205917 0.731698 0.143342
8 1.086070 2.270902 -0.274339
1 0.598724 3.114248 -0.286426

6 2.235995 -0.309842 -0.220741
8 2.897020 0.102612 0.970546
1 2.460139 -0.321702 1.727505
8 2.446036 -1.685405 -0.473740
1 3.391335 -1.813258 -0.645342
1 2.690101 0.298543 -1.008491
6 -2.496152 -1.884805 0.091526
1 -3.057215 -1.819724 1.025520
1 -3.187626 -1.800918 -0.748835
1 -1.969205 -2.836645 0.043428
1m5dhmU/1m5dhmU54.xyz
6 1.972075 -0.608787 0.033207
6 -0.267612 -1.209146 -0.085587
6 -0.712499 0.147465 -0.121378
6 0.279743 1.089555 -0.074814
1 0.064771 2.149546 -0.114155
7 1.586492 0.741987 0.012094
7 1.000262 -1.564325 -0.013056
8 3.186952 -0.887530 0.098482
8 -1.144491 -2.229882 -0.131604
1 -2.056362 -1.872042 -0.033833
6 -2.166535 0.520164 -0.323439
8 -3.040389 -0.370339 0.378662
1 -2.866139 -0.286771 1.332553
8 -2.352033 1.854156 0.083631
1 -3.181632 2.176429 -0.301116
1 -2.453834 0.392093 -1.372799
6 2.641975 1.763830 0.051984
1 3.217029 1.665588 0.974376
1 3.309239 1.634288 -0.802034
1 2.176458 2.747508 0.010536
1m5dhmU/1m5dhmU48.xyz
6 -1.937179 0.665033 0.087007
6 0.348593 1.067737 0.011025
6 0.663801 -0.315886 -0.139007
6 -0.409124 -1.163096 -0.190958

1 -0.285876 -2.234107 -0.308913
7 -1.680318 -0.706524 -0.092598
7 -0.881782 1.526958 0.123825
8 -3.119467 1.046765 0.197142
8 1.315237 2.004234 0.041749
1 2.174219 1.587830 -0.196956
6 2.068992 -0.854334 -0.143082
8 2.876564 0.049312 -0.900039
1 3.795633 -0.262272 -0.887939
8 2.519729 -0.929136 1.200989
1 3.312644 -1.488293 1.230479
1 2.104286 -1.848907 -0.600072
6 -2.829625 -1.620813 -0.163553
1 -3.405495 -1.557658 0.761406
1 -3.468639 -1.343935 -1.004098
1 -2.461326 -2.636938 -0.299819
1m5fC/1m5fC01.xyz
6 -1.533273 0.762885 0.000275
6 0.798576 0.920435 0.000078
6 0.941495 -0.520507 -0.000145
6 -0.231056 -1.251083 -0.000160
1 -0.217335 -2.336411 -0.000327
7 -1.431810 -0.660921 0.000038
7 -0.403939 1.506738 0.000290
8 -2.676064 1.257965 0.000443
7 1.884170 1.700786 0.000067
1 1.777953 2.706739 0.000184
1 2.804824 1.276886 -0.000120
6 2.196554 -1.236700 -0.000365
1 2.108645 -2.336861 -0.000522
8 3.320500 -0.709109 -0.000381
6 -2.676208 -1.444119 -0.000056
1 -3.261253 -1.204740 0.889367
1 -3.261245 -1.204537 -0.889431
1 -2.422552 -2.503598 -0.000173
1m5fC/1m5fC02.xyz

6 1.595688 0.609943 0.002753
6 -0.664576 1.220376 -0.002067
6 -1.085401 -0.163219 -0.000084
6 -0.073590 -1.105729 -0.002915
1 -0.303842 -2.165369 -0.003261
7 1.219098 -0.764594 -0.005472
7 0.631598 1.556790 0.000165
8 2.812805 0.873588 0.011430
7 -1.544340 2.233746 -0.006527
1 -1.192999 3.182331 -0.006172
1 -2.543649 2.094848 -0.006826
6 -2.468506 -0.612414 0.003975
1 -3.248247 0.166006 0.005718
8 -2.815858 -1.800864 0.006048
6 2.285196 -1.776726 -0.005957
1 2.928342 -1.628308 -0.874467
1 2.880936 -1.682812 0.903524
1 1.826515 -2.763469 -0.049738
1m5fU/1m5fU11.xyz
6 -1.634038 0.508486 0.001753
6 0.722843 1.290911 -0.002386
6 1.127593 -0.103145 -0.000762
6 0.157385 -1.078495 -0.002606
1 0.432134 -2.127395 -0.001795
7 -1.165166 -0.811398 -0.005772
7 -0.659203 1.479043 -0.001671
8 -2.834923 0.770104 0.009757
8 1.476633 2.273244 -0.003880
1 -0.993117 2.440235 0.000694
6 2.540290 -0.457255 0.002518
1 3.240726 0.392239 0.002433
8 2.969972 -1.616210 0.005584
6 -2.173711 -1.885957 -0.003430
1 -2.760392 -1.834396 0.915187
1 -2.830635 -1.768041 -0.865939
1 -1.653773 -2.840546 -0.060691

1m5fU/1m5fU12.xyz
6 -1.579416 0.672237 0.000196
6 0.887967 0.994615 0.000057
6 1.020803 -0.455749 -0.000128
6 -0.122517 -1.224478 -0.000170
1 -0.058867 -2.308300 -0.000318
7 -1.371192 -0.712315 -0.000034
7 -0.436805 1.436671 0.000191
8 -2.707645 1.160028 0.000372
8 1.804878 1.822726 0.000101
1 -0.583781 2.443497 0.000340
6 2.296191 -1.155163 -0.000280
1 2.207097 -2.256514 -0.000429
8 3.413094 -0.630467 -0.000253
6 -2.570926 -1.569513 -0.000054
1 -3.165768 -1.364535 0.891167
1 -3.165931 -1.364259 -0.891101
1 -2.251998 -2.610359 -0.000250
1m5fU/1m5fU10.xyz
6 1.522368 -0.772005 0.000280
6 -0.790242 -0.891543 0.000093
6 -0.930350 0.538596 -0.000143
6 0.246794 1.264777 -0.000192
1 0.245230 2.350035 -0.000385
7 1.435157 0.652332 0.000007
7 0.371650 -1.505778 0.000306
8 2.648492 -1.286813 0.000464
8 -1.879387 -1.662673 0.000101
1 -2.676592 -1.070264 -0.000052
6 -2.212664 1.200264 -0.000347
1 -2.203925 2.301010 -0.000507
8 -3.294469 0.583853 -0.000339
6 2.694890 1.413579 -0.000123
1 3.272824 1.159823 0.889750
1 3.272795 1.159549 -0.889938
1 2.460149 2.477038 -0.000278

1m5fU/1m5fU08.xyz
6 -1.602147 0.591264 0.002598
6 0.636675 1.197798 -0.002063
6 1.087200 -0.157907 -0.000120
6 0.087067 -1.113887 -0.002544
1 0.321927 -2.172428 -0.002671
7 -1.207703 -0.775135 -0.004839
7 -0.630908 1.550954 -0.000682
8 -2.812145 0.854816 0.011046
8 1.568456 2.158638 -0.005880
1 1.131381 3.029535 -0.006075
6 2.494106 -0.538985 0.003402
1 3.219206 0.288983 0.004128
8 2.891925 -1.708963 0.005879
6 -2.270288 -1.793464 -0.005562
1 -2.865862 -1.698106 0.903627
1 -2.911454 -1.646062 -0.875490
1 -1.806491 -2.777491 -0.047500
1m5fU/1m5fU09.xyz
6 -1.542967 0.734882 0.000004
6 0.774229 0.927398 0.000001
6 0.969723 -0.492787 -0.000003
6 -0.192089 -1.244984 -0.000003
1 -0.158832 -2.329742 -0.000005
7 -1.405219 -0.678441 0.000001
7 -0.410709 1.498218 0.000004
8 -2.682547 1.219832 0.000008
8 1.856897 1.709070 0.000000
1 1.575412 2.642698 0.000002
6 2.239832 -1.203703 -0.000006
1 2.135602 -2.303729 -0.000009
8 3.362948 -0.693584 -0.000006
6 -2.636579 -1.485075 0.000000
1 -3.223543 -1.252469 0.889789
1 -3.223543 -1.252467 -0.889788
1 -2.364872 -2.539662 -0.000002

1m5fU/1m5fU07.xyz
6 -1.604274 0.601836 0.001729
6 0.635134 1.217119 -0.001560
6 1.085691 -0.143477 -0.000252
6 0.082053 -1.099610 -0.001845
1 0.320951 -2.157318 -0.001778
7 -1.210989 -0.765616 -0.003594
7 -0.635511 1.559315 -0.000600
8 -2.815132 0.863749 0.007869
8 1.477775 2.263373 -0.003725
1 2.411065 1.998128 -0.003818
6 2.482016 -0.562673 0.001914
1 3.244627 0.234817 0.001093
8 2.849286 -1.741185 0.004689
6 -2.273029 -1.784344 -0.003875
1 -2.873241 -1.682123 0.901430
1 -2.909536 -1.641301 -0.877955
1 -1.809354 -2.768700 -0.036941
1m5fU/1m5fU05.xyz
6 -1.499160 0.512887 0.002544
6 0.685562 1.309214 -0.005122
6 1.139838 -0.079359 -0.000801
6 0.203380 -1.081420 -0.005032
1 0.483489 -2.128217 -0.005145
7 -1.124295 -0.812614 -0.009363
7 -0.684928 1.527917 0.001958
8 -2.820317 0.679160 0.016548
8 1.467998 2.281972 -0.013573
1 -3.023906 1.631840 0.028401
6 2.564386 -0.403920 0.007454
1 3.246334 0.460217 0.011801
8 3.018910 -1.552712 0.010985
6 -2.135835 -1.888280 -0.009531
1 -2.716643 -1.844387 0.912783
1 -2.794081 -1.768113 -0.870228
1 -1.612379 -2.840560 -0.074533

1m5fU/1m5fU06.xyz
6 -1.446581 0.644485 0.000154
6 0.853043 1.026476 0.000057
6 1.043735 -0.426970 -0.000103
6 -0.068489 -1.233985 -0.000157
1 0.007966 -2.316531 -0.000294
7 -1.324345 -0.727229 -0.000046
7 -0.456991 1.488205 0.000196
8 -2.713019 1.055749 0.000268
8 1.790636 1.846856 0.000095
1 -2.731678 2.029977 0.000427
6 2.335870 -1.104192 -0.000229
1 2.267504 -2.207468 -0.000347
8 3.444318 -0.563777 -0.000190
6 -2.521801 -1.593071 -0.000059
1 -3.116336 -1.394816 0.892436
1 -3.116490 -1.394577 -0.892399
1 -2.191758 -2.630498 -0.000233
1m5fU/1m5fU04.xyz
6 1.444347 0.670791 0.000007
6 -0.862278 1.028198 -0.000004
6 -1.044990 -0.425690 0.000004
6 0.072297 -1.221912 -0.000004
1 0.004276 -2.304968 0.000003
7 1.327641 -0.706837 -0.000024
7 0.441824 1.499049 0.000024
8 2.665943 1.209199 0.000017
8 -1.806803 1.841408 -0.000028
1 3.390902 0.561679 -0.000004
6 -2.332027 -1.114317 0.000024
1 -2.254273 -2.217015 0.000082
8 -3.444005 -0.582300 -0.000006
6 2.514815 -1.586371 -0.000013
1 3.110259 -1.407252 -0.898378
1 3.110265 -1.407224 0.898342
1 2.178257 -2.621357 0.000007

1m5fU/1m5fU03.xyz
6 -1.568297 0.754631 0.000192
6 0.778574 1.052460 0.000169
6 0.956892 -0.420515 -0.000030
6 -0.197319 -1.226672 -0.000096
1 -0.124982 -2.310953 -0.000261
7 -1.401971 -0.698036 0.000011
7 -0.488708 1.545661 0.000255
8 -2.742276 1.159573 0.000270
8 1.768637 1.819777 0.000254
1 4.049413 -1.165017 -0.000593
6 2.159453 -1.104172 -0.000275
1 2.161350 -2.193608 -0.000438
8 3.326735 -0.510876 -0.000354
6 -2.617981 -1.527055 -0.000172
1 -3.208122 -1.303413 0.889267
1 -3.208005 -1.303180 -0.889631
1 -2.327595 -2.577049 -0.000290
1m5fU/1m5fU02.xyz
6 -1.644886 0.577765 0.010181
6 0.579896 1.369299 -0.009505
6 1.067527 -0.030055 -0.004344
6 0.117999 -1.068439 -0.012939
1 0.418997 -2.110792 -0.018489
7 -1.170839 -0.808367 -0.014192
7 -0.761982 1.584064 0.003512
8 -2.877695 0.719641 0.035626
8 1.389228 2.327078 -0.024174
1 3.857926 -1.543197 0.035442
6 2.422047 -0.300695 0.011642
1 3.143278 0.513322 0.021309
8 2.884862 -1.529692 0.018110
6 -2.176389 -1.881881 -0.018447
1 -2.766329 -1.826363 0.897440
1 -2.831920 -1.755465 -0.880438
1 -1.660532 -2.839565 -0.076523

1m5hmC/1m5hmC01.xyz
6 1.634725 0.707247 0.065486
6 -0.677844 0.990804 -0.119146
6 -0.901483 -0.424765 -0.237865
6 0.212787 -1.209374 -0.166931
1 0.152098 -2.290170 -0.233114
7 1.459217 -0.683338 -0.019214
7 0.545502 1.514131 0.021392
8 2.800435 1.155386 0.187131
6 -2.266828 -1.019239 -0.437115
1 -2.755273 -0.589205 -1.317175
1 -2.176840 -2.099897 -0.595344
7 -1.720152 1.846911 -0.171574
1 -1.550219 2.828171 0.004480
1 -2.664627 1.503682 -0.065582
8 -3.166364 -0.742460 0.658682
1 -2.776939 -1.110550 1.466882
6 2.646196 -1.545282 0.053304
1 3.319012 -1.324471 -0.777972
1 3.174786 -1.375286 0.993300
1 2.328139 -2.585956 -0.002603
1m5hmC/1m5hmC02.xyz
6 -1.641350 0.705241 0.062432
6 0.672024 0.985957 -0.114413
6 0.895500 -0.430729 -0.215794
6 -0.219239 -1.214353 -0.145209
1 -0.158900 -2.295592 -0.201658
7 -1.466086 -0.686169 -0.011595
7 -0.551537 1.511108 0.017103
8 -2.807404 1.154632 0.174883
6 2.256838 -1.021336 -0.419714
1 2.171266 -2.104362 -0.562973
1 2.728674 -0.595395 -1.315115
7 1.714967 1.840958 -0.177622
1 1.544995 2.823888 -0.011067
1 2.657688 1.496833 -0.057086

8 3.079205 -0.729824 0.734769
1 3.993177 -0.966799 0.516841
6 -2.654516 -1.546510 0.056238
1 -3.188743 -1.373336 0.992438
1 -3.321915 -1.326860 -0.779708
1 -2.337604 -2.587738 0.004682
1m5hmC/1m5hmC03.xyz
6 -1.648894 0.669774 0.098269
6 0.647979 1.060909 -0.123232
6 0.933025 -0.340977 -0.274463
6 -0.150654 -1.171252 -0.221727
1 -0.044396 -2.245511 -0.326838
7 -1.416291 -0.708524 -0.043144
7 -0.597031 1.522157 0.052268
8 -2.828731 1.063209 0.261922
6 2.315885 -0.905124 -0.456254
1 2.244778 -1.903825 -0.891943
1 2.908248 -0.286491 -1.141098
7 1.632750 1.981457 -0.176096
1 1.404521 2.951391 -0.006460
1 2.607791 1.721631 -0.211882
8 3.035885 -1.083249 0.786000
1 3.229207 -0.213811 1.167122
6 -2.563982 -1.624364 0.003142
1 -3.085170 -1.521555 0.956721
1 -3.257034 -1.394722 -0.808780
1 -2.201326 -2.646210 -0.105816
1m5hmC/1m5hmC04.xyz
6 1.713483 0.525410 0.007575
6 -0.496437 1.285651 -0.000751
6 -1.022637 -0.051584 -0.011463
6 -0.096089 -1.049754 -0.020730
1 -0.379919 -2.094500 -0.034323
7 1.244471 -0.792960 -0.016135
7 0.817241 1.541986 0.008939
8 2.954725 0.723497 0.026569

6 -2.513059 -0.315976 -0.014255
1 -2.978258 0.158071 0.861674
1 -2.969267 0.129806 -0.905402
7 -1.329641 2.346549 -0.002673
1 -0.943386 3.280670 0.005992
1 -2.333414 2.244200 -0.005871
8 -2.846170 -1.703388 -0.059142
1 -2.626151 -2.103973 0.795277
6 2.222377 -1.887202 -0.016476
1 2.886769 -1.792216 -0.877556
1 2.818326 -1.856868 0.898545
1 1.686524 -2.834349 -0.072064
1m5hmC/1m5hmC05.xyz
6 1.717153 0.520263 0.004314
6 -0.491530 1.284585 -0.003421
6 -1.019232 -0.051711 -0.005023
6 -0.094997 -1.052730 -0.009738
1 -0.379618 -2.096389 -0.012946
7 1.245847 -0.797166 -0.011133
7 0.822571 1.538114 0.001978
8 2.958763 0.716609 0.019529
6 -2.503197 -0.307524 -0.002919
1 -2.961554 0.168961 0.876074
1 -2.960670 0.141837 -0.896602
7 -1.322249 2.347331 -0.006620
1 -0.933596 3.280459 -0.003117
1 -2.326294 2.247515 -0.009932
8 -2.749153 -1.717411 0.018933
1 -3.708080 -1.850683 0.022186
6 2.221949 -1.893067 -0.008086
1 2.887963 -1.799992 -0.868072
1 2.816295 -1.862795 0.908005
1 1.684614 -2.839347 -0.063631
1m5hmU/1m5hmU01.xyz
6 -1.657847 0.647763 0.068670
6 0.784109 1.006746 -0.147965

6 0.949448 -0.427133 -0.228292
6 -0.157863 -1.212391 -0.148795
1 -0.087864 -2.293292 -0.195897
7 -1.430469 -0.713266 -0.013739
7 -0.529443 1.437997 -0.000751
8 -2.790000 1.129419 0.197152
8 1.711824 1.836242 -0.193517
1 -0.688459 2.440312 0.063254
6 2.334609 -0.984863 -0.398301
1 2.289752 -2.071799 -0.491571
1 2.793076 -0.579519 -1.308845
8 3.185804 -0.706482 0.732030
1 3.322637 0.253655 0.761016
6 -2.602890 -1.601283 0.052336
1 -3.135234 -1.445790 0.992348
1 -3.273416 -1.390351 -0.782900
1 -2.259527 -2.632797 -0.007207
1m5hmU/1m5hmU03.xyz
6 -1.634564 0.689346 0.083222
6 0.654995 0.984571 -0.134444
6 0.920264 -0.407215 -0.256546
6 -0.186914 -1.208827 -0.185748
1 -0.114920 -2.288078 -0.260592
7 -1.434845 -0.695912 -0.025512
7 -0.542747 1.509047 0.027277
8 -2.794026 1.127302 0.227074
8 1.717725 1.808709 -0.185878
6 2.309246 -0.954867 -0.433034
1 2.252452 -2.014141 -0.692235
1 2.824744 -0.428853 -1.244443
8 3.101231 -0.882942 0.771264
1 3.300183 0.049415 0.946404
6 -2.615570 -1.570462 0.039268
1 -3.140071 -1.413594 0.983364
1 -3.290432 -1.340145 -0.787399
1 -2.288916 -2.607128 -0.031942

1 1.415914 2.730744 -0.101498
1m5hmU/1m5hmU02.xyz
6 -1.643452 0.672471 0.113374
6 0.634347 1.046343 -0.140942
6 0.937762 -0.340059 -0.283435
6 -0.152278 -1.171119 -0.235489
1 -0.049015 -2.245497 -0.341651
7 -1.411682 -0.702308 -0.061313
7 -0.580021 1.522128 0.049254
8 -2.809376 1.070179 0.313013
8 1.590606 1.999994 -0.193671
6 2.330096 -0.896404 -0.428150
1 2.272209 -1.911160 -0.826547
1 2.925488 -0.301350 -1.132584
8 3.026634 -1.007948 0.830114
1 3.190406 -0.116788 1.174398
6 -2.567916 -1.610305 -0.019153
1 -3.032339 -1.571517 0.967943
1 -3.298200 -1.305739 -0.770492
1 -2.227829 -2.623933 -0.227637
1 2.466929 1.633879 -0.391895
1m5hmU/1m5hmU04.xyz
6 -1.515807 0.633803 0.044641
6 0.763729 1.010272 -0.145781
6 0.962894 -0.429128 -0.220996
6 -0.125554 -1.236441 -0.141460
1 -0.060173 -2.317435 -0.178944
7 -1.395062 -0.719888 -0.010610
7 -0.525328 1.486287 -0.017446
8 -2.779741 1.057622 0.170503
8 1.732606 1.812667 -0.186812
6 2.357125 -0.967796 -0.382830
1 2.341560 -2.059453 -0.415137
1 2.790567 -0.600425 -1.322044
8 3.213518 -0.602513 0.717475
1 3.260801 0.367929 0.721137

6 -2.581560 -1.592951 0.066039
1 -3.112308 -1.417484 1.002879
1 -3.245012 -1.388843 -0.775929
1 -2.246521 -2.627931 0.023715
1 -2.782198 2.030082 0.213718
1m5hmU/1m5hmU05.xyz
6 -1.514535 0.659661 0.037988
6 0.772384 1.013459 -0.132422
6 0.963958 -0.425746 -0.223140
6 -0.129136 -1.224014 -0.143681
1 -0.071053 -2.305180 -0.185221
7 -1.397675 -0.699841 -0.007568
7 -0.511982 1.497913 -0.015325
8 -2.735535 1.206923 0.150168
8 1.748777 1.808317 -0.154288
6 2.353326 -0.973483 -0.396247
1 2.329875 -2.064738 -0.436841
1 2.783159 -0.602235 -1.335699
8 3.217825 -0.621379 0.701469
1 3.265132 0.349125 0.714780
6 -2.572493 -1.587159 0.085947
1 -3.100104 -1.414238 1.027027
1 -3.239656 -1.418306 -0.763425
1 -2.228350 -2.619286 0.060843
1 -3.460966 0.561157 0.129325
1m5mC/1m5mC01.xyz
6 1.190875 -0.822824 0.000000
6 -1.143115 -0.727731 0.000000
6 -1.155488 0.710176 -0.000001
6 0.073135 1.301043 -0.000001
1 0.189551 2.378970 -0.000001
7 1.231111 0.575768 -0.000002
7 -0.012483 -1.445590 -0.000001
8 2.277349 -1.457232 0.000001
7 -2.306819 -1.409256 0.000000
1 -2.294041 -2.419728 0.000002

1 -3.197756 -0.935348 0.000002
6 -2.433939 1.501491 0.000001
1 -3.044174 1.278087 -0.883968
1 -3.044172 1.278083 0.883969
1 -2.221049 2.573782 0.000002
6 2.538591 1.241896 0.000001
1 3.105180 0.956734 0.889118
1 3.105190 0.956718 -0.889103
1 2.379466 2.319793 -0.000009
1m5mU/1m5mU01.xyz
6 1.235493 -0.748347 -0.000007
6 -1.245373 -0.790448 -0.000015
6 -1.240063 0.657321 -0.000003
6 -0.035203 1.287378 0.000029
1 0.037028 2.368571 0.000046
7 1.174792 0.627687 0.000035
7 0.013258 -1.386481 -0.000036
8 2.304654 -1.376112 -0.000017
8 -2.265203 -1.505207 -0.000070
6 -2.547954 1.393225 -0.000014
1 -3.144697 1.133885 -0.881710
1 -3.144706 1.133894 0.881679
1 -2.382835 2.473764 -0.000018
6 2.446693 1.366627 0.000087
1 3.025139 1.113576 0.890828
1 3.025174 1.113649 -0.890651
1 2.225301 2.432821 0.000125
1 0.046083 -2.402585 -0.000065
1m5mU/1m5mU04.xyz
6 1.192556 -0.810192 -0.000032
6 -1.124537 -0.723945 -0.000028
6 -1.178112 0.697512 -0.000013
6 0.048410 1.303676 0.000019
1 0.154928 2.382358 0.000043
7 1.208783 0.588403 0.000024
7 -0.017783 -1.441276 -0.000037

8 2.275361 -1.436837 0.000009
8 -2.307401 -1.370703 -0.000056
6 -2.475400 1.454589 -0.000011
1 -3.076393 1.209229 -0.882428
1 -3.076391 1.209223 0.882407
1 -2.288184 2.531629 -0.000006
6 2.512647 1.265866 0.000096
1 3.078055 0.981083 0.889641
1 3.078094 0.981209 -0.889464
1 2.344975 2.342051 0.000169
1 -2.149151 -2.331399 -0.000069
1m5mU/1m5mU05.xyz
6 1.203584 -0.810366 -0.000008
6 -1.114472 -0.749017 -0.000041
6 -1.177993 0.674876 -0.000015
6 0.044569 1.291497 0.000018
1 0.139437 2.371294 0.000038
7 1.209953 0.589419 0.000029
7 0.001336 -1.451629 -0.000040
8 2.293097 -1.426168 0.000001
8 -2.239529 -1.500848 -0.000055
6 -2.472873 1.440074 -0.000009
1 -3.076418 1.206817 -0.885633
1 -3.076417 1.206803 0.885611
1 -2.278585 2.515522 -0.000001
6 2.507696 1.278505 0.000092
1 3.075392 0.998184 0.889550
1 3.075438 0.998272 -0.889366
1 2.330823 2.353276 0.000140
1 -3.040305 -0.951980 -0.000054
1m5mU/1m5mU03.xyz
6 1.110669 -0.696862 -0.000005
6 -1.206476 -0.842186 -0.000033
6 -1.273600 0.614209 -0.000012
6 -0.102398 1.301779 0.000022
1 -0.055589 2.383876 0.000041

7 1.119409 0.659409 0.000031
7 0.037590 -1.444699 -0.000034
8 2.333464 -1.250319 -0.000001
8 -2.244931 -1.552379 -0.000049
6 -2.609186 1.297010 -0.000022
1 -3.194210 1.008353 -0.880585
1 -3.194225 1.008352 0.880530
1 -2.494890 2.384418 -0.000021
6 2.384511 1.414802 0.000084
1 2.964326 1.171268 0.891951
1 2.964367 1.171331 -0.891774
1 2.146281 2.477356 0.000115
1 2.235572 -2.218865 -0.000028
1m5mU/1m5mU02.xyz
6 1.103522 -0.722860 -0.000010
6 -1.217353 -0.839862 -0.000037
6 -1.273548 0.616701 -0.000007
6 -0.096464 1.291245 0.000027
1 -0.038953 2.372687 0.000053
7 1.122901 0.638049 0.000030
7 0.018515 -1.452640 -0.000041
8 2.266750 -1.399525 -0.000011
8 -2.262835 -1.540522 -0.000055
6 -2.602274 1.311934 -0.000009
1 -3.189592 1.027890 -0.880519
1 -3.189606 1.027867 0.880483
1 -2.478249 2.398240 0.000006
1 3.052244 -0.828879 0.000003
6 2.380412 1.407498 0.000085
1 2.962426 1.184620 0.897993
1 2.962469 1.184682 -0.897811
1 2.132256 2.467472 0.000114
1mC/1mC01.xyz
6 -0.618373 -0.878679 -0.000086
6 1.557292 -0.031274 -0.000018
6 1.084198 1.317546 0.000125

6 -0.264639 1.496404 0.000159
1 -0.719964 2.479776 0.000266
7 -1.119428 0.434303 0.000073
7 0.723423 -1.080043 -0.000108
8 -1.438307 -1.828979 -0.000190
7 2.881451 -0.279771 -0.000042
1 3.217655 -1.232741 -0.000136
1 3.551061 0.476345 0.000026
1 1.762980 2.160906 0.000204
6 -2.574022 0.627580 0.000071
1 -3.012666 0.170207 -0.889177
1 -3.012712 0.169903 0.889137
1 -2.784762 1.696555 0.000242
1mU/1mU01.xyz
6 0.631161 -0.856730 0.000151
6 -1.695877 0.007955 -0.000025
6 -1.144841 1.337066 -0.000297
6 0.203003 1.503628 -0.000306
1 0.657464 2.487221 -0.000491
7 1.084208 0.450423 -0.000113
7 -0.739614 -1.007651 0.000165
8 1.397391 -1.828181 0.000366
8 -2.906410 -0.283537 0.000046
1 -1.813929 2.186732 -0.000471
6 2.539609 0.662785 -0.000033
1 2.980885 0.213027 0.891300
1 2.981087 0.212316 -0.890901
1 2.731096 1.734401 -0.000429
1 -1.074952 -1.967592 0.000393
1mU/1mU02.xyz
6 0.601625 -0.880552 0.000000
6 -1.546777 -0.009031 0.000000
6 -1.093893 1.331029 0.000000
6 0.261749 1.499703 0.000000
1 0.725671 2.478734 0.000000
7 1.103237 0.431988 0.000000

7 -0.752515 -1.062535 0.000000
8 1.403560 -1.836745 0.000000
8 -2.879861 -0.206202 0.000000
1 -3.064539 -1.162572 -0.000001
1 -1.777798 2.168502 0.000000
6 2.561805 0.610067 0.000000
1 2.990307 0.144652 0.889534
1 2.990309 0.144642 -0.889527
1 2.784342 1.676142 -0.000005
1mU/1mU03.xyz
6 -0.625235 -0.877723 0.000011
6 1.544255 -0.064242 0.000004
6 1.121432 1.288318 -0.000008
6 -0.229995 1.492366 -0.000003
1 -0.666984 2.483948 -0.000010
7 -1.096485 0.447263 0.000005
7 0.721786 -1.095754 0.000005
8 -1.453451 -1.812021 0.000017
8 2.855910 -0.389093 -0.000027
1 3.410868 0.408112 -0.000045
1 1.822469 2.113215 -0.000022
6 -2.549996 0.661025 0.000007
1 -2.989852 0.206186 -0.889469
1 -2.989846 0.206209 0.889496
1 -2.746202 1.732218 -0.000006
1mU/1mU05.xyz
6 0.529201 -0.767467 0.000009
6 -1.688788 -0.079461 -0.000004
6 -1.215950 1.288899 -0.000008
6 0.117417 1.533695 -0.000013
1 0.539648 2.531079 -0.000019
7 1.026682 0.499616 -0.000014
7 -0.738477 -1.088428 0.000011
8 1.474614 -1.716423 0.000033
8 -2.911976 -0.370854 -0.000008
1 1.039123 -2.587052 0.000039

1 -1.928655 2.104015 -0.000010
6 2.479516 0.746101 -0.000011
1 2.930833 0.309440 0.892281
1 2.930863 0.309261 -0.892197
1 2.641273 1.822554 -0.000118
1mU/1mU04.xyz
6 -0.523480 -0.783188 -0.005601
6 1.695702 -0.089871 0.003165
6 1.227633 1.279534 -0.018169
6 -0.104131 1.524046 -0.016457
1 -0.526091 2.521272 -0.026333
7 -1.018009 0.490700 -0.002508
7 0.746116 -1.095899 0.000045
8 -1.382479 -1.815203 -0.010340
8 2.918983 -0.382478 0.021607
1 -2.314554 -1.549593 -0.069309
1 1.940706 2.094281 -0.029709
6 -2.466013 0.768806 0.027589
1 -2.945547 0.384975 -0.876499
1 -2.915788 0.323538 0.918380
1 -2.605764 1.847401 0.067406
5caC/5caC01.xyz
6 -2.212299 -0.161261 0.000002
6 -0.052340 0.991625 0.000006
6 0.638517 -0.268660 0.000003
6 -0.140740 -1.391766 -0.000004
1 0.294952 -2.382792 -0.000008
7 -1.497281 -1.340535 -0.000015
7 -1.419703 0.973368 0.000024
8 -3.438861 -0.110661 -0.000004
7 0.570694 2.150691 -0.000017
1 0.068801 3.031839 0.000016
1 1.594933 2.097338 -0.000006
6 2.150352 -0.382838 0.000002
8 2.813058 0.704104 -0.000007
8 2.642506 -1.544618 0.000010

1 -1.929116 1.855706 0.000003
1 -2.040101 -2.199960 0.000000
5caC/5caC02.xyz
6 -2.222801 -0.114282 0.000001
6 -0.184462 1.031092 -0.000002
6 0.575750 -0.206884 0.000000
6 -0.162513 -1.368306 0.000005
1 0.289245 -2.352066 0.000009
7 -1.502145 -1.324903 0.000009
7 -1.526261 1.042084 -0.000001
8 -3.468684 -0.176961 -0.000004
7 0.434368 2.215253 -0.000010
1 -0.120134 3.061085 -0.000001
1 1.446209 2.256878 0.000004
6 2.039115 -0.247770 0.000002
8 2.768111 0.746657 0.000018
8 2.549358 -1.498858 -0.000019
1 3.521625 -1.435910 -0.000002
1 -2.039486 -2.186831 0.000010
5caC/5caC03.xyz
6 -2.229278 -0.069295 0.000000
6 -0.149483 1.009663 -0.000002
6 0.567734 -0.254902 -0.000002
6 -0.214868 -1.387363 -0.000002
1 0.212976 -2.382453 -0.000005
7 -1.550970 -1.303184 -0.000006
7 -1.491802 1.061226 -0.000007
8 -3.476749 -0.089085 0.000010
7 0.487014 2.185957 0.000000
1 -0.062816 3.034817 0.000001
1 1.495678 2.236884 0.000004
6 2.023245 -0.461632 0.000001
8 2.562199 -1.566688 0.000002
8 2.749337 0.678123 0.000002
1 3.697893 0.452474 0.000010
1 -2.115815 -2.147341 0.000009

5caC/5caC04.xyz
6 2.218933 -0.103282 0.010936
6 0.170089 1.024106 0.042803
6 -0.581925 -0.221298 -0.025501
6 0.171432 -1.367575 -0.135187
1 -0.255058 -2.357320 -0.256602
7 1.511502 -1.311417 -0.133340
7 1.510369 1.043618 0.085922
8 3.464263 -0.155763 0.053447
7 -0.454536 2.204465 0.083786
1 0.099245 3.049537 0.130844
1 -1.461333 2.253064 -0.009247
6 -2.053000 -0.252894 -0.026582
8 -2.750387 0.726561 -0.284980
8 -2.694455 -1.410687 0.264440
1 -2.119051 -2.093794 0.646999
1 2.056308 -2.163379 -0.228637
5caC/5caC05.xyz
6 2.214235 -0.076696 0.056315
6 0.144657 0.993356 -0.135415
6 -0.571900 -0.262463 -0.045977
6 0.200750 -1.397129 -0.001883
1 -0.228455 -2.391490 0.031793
7 1.539725 -1.309923 0.033833
7 1.479190 1.053832 -0.064561
8 3.455941 -0.086946 0.168899
7 -0.506557 2.158551 -0.304726
1 0.045626 2.995911 -0.441561
1 -1.478166 2.192638 -0.582443
6 -2.036959 -0.441953 0.010820
8 -2.616172 -1.407903 -0.469574
8 -2.777084 0.499775 0.645418
1 -2.235489 1.107517 1.178528
1 2.103804 -2.151903 0.100760
5caC/5caC06.xyz
6 -2.077053 -0.099057 -0.000004

6 -0.065565 0.999043 0.000000
6 0.654387 -0.255065 0.000000
6 -0.109909 -1.386147 0.000007
1 0.314450 -2.381000 0.000024
7 -1.468955 -1.311777 0.000001
7 -1.429024 1.035516 -0.000005
8 -3.402746 -0.162513 0.000004
7 0.569606 2.160763 0.000005
1 0.047977 3.028045 0.000022
1 1.590006 2.129875 0.000002
6 2.169187 -0.370709 -0.000001
8 2.834933 0.712816 -0.000003
8 2.660386 -1.534597 -0.000004
1 -3.784731 0.733848 -0.000042
1 -2.035958 -2.156319 0.000000
5caC/5caC07.xyz
6 2.082758 -0.063919 0.000001
6 0.052942 1.005257 -0.000001
6 -0.654000 -0.256602 0.000000
6 0.124197 -1.376278 0.000001
1 -0.285551 -2.377336 0.000001
7 1.484900 -1.285603 0.000002
7 1.413068 1.058931 0.000000
8 3.409136 0.020323 0.000002
7 -0.597123 2.159417 0.000001
1 -0.085603 3.032640 0.000002
1 -1.616894 2.116529 0.000005
6 -2.167807 -0.390300 -0.000002
8 -2.845685 0.685456 -0.000006
8 -2.645031 -1.559888 0.000001
1 3.860556 -0.841823 0.000002
1 2.045673 -2.135305 0.000002
5caC/5caC08.xyz
6 -2.220516 -0.056191 0.000001
6 -0.125814 0.904170 -0.000001
6 0.581885 -0.319941 -0.000005

6 -0.231919 -1.434552 -0.000005
1 0.157423 -2.445196 -0.000003
7 -1.566713 -1.294805 -0.000008
7 -1.421965 1.064458 0.000003
8 -3.456828 -0.012427 0.000006
7 0.668444 2.134380 -0.000001
1 0.460434 2.700942 -0.830458
1 1.695401 1.805116 -0.000010
6 2.089657 -0.451153 0.000001
8 2.761795 0.640904 -0.000003
8 2.578425 -1.606130 0.000008
1 0.460456 2.700928 0.830471
1 -2.158963 -2.122790 0.000000
5caU/5caU01.xyz
6 -2.223737 -0.113491 -0.000006
6 -0.033264 1.027028 -0.000007
6 0.611584 -0.273223 -0.000002
6 -0.176146 -1.392507 0.000005
1 0.242926 -2.391726 0.000012
7 -1.525032 -1.312997 -0.000001
7 -1.418108 1.005768 -0.000021
8 -3.450170 -0.061859 0.000012
8 0.570030 2.118149 -0.000006
1 -1.897175 1.904014 -0.000011
6 2.083821 -0.412404 0.000001
8 2.658874 -1.497420 0.000018
8 2.795783 0.726716 0.000001
1 2.171321 1.502839 -0.000011
1 -2.084765 -2.161628 0.000015
5caU/5caU02.xyz
6 -2.223414 -0.202657 0.000001
6 -0.108771 1.114289 -0.000003
6 0.627538 -0.147737 -0.000003
6 -0.087134 -1.318893 -0.000006
1 0.392601 -2.289074 -0.000002
7 -1.437943 -1.345590 -0.000019

7 -1.501781 0.965925 -0.000001
8 -3.452452 -0.245925 0.000015
8 0.364872 2.254085 -0.000003
1 -2.045942 1.825667 0.000001
6 2.099125 -0.174124 0.000002
8 2.602483 -1.431943 0.000005
8 2.827693 0.811769 0.000002
1 3.574878 -1.372412 0.000032
1 -1.928299 -2.235672 0.000014
5caU/5caU03.xyz
6 2.234723 -0.148583 -0.000001
6 0.066428 1.082379 -0.000003
6 -0.616863 -0.208915 -0.000006
6 0.146869 -1.348206 -0.000005
1 -0.304837 -2.332732 -0.000002
7 1.496536 -1.323089 -0.000007
7 1.465032 0.988519 -0.000034
8 3.464491 -0.140948 0.000027
8 -0.447199 2.204697 0.000008
1 1.973103 1.869980 -0.000006
6 -2.080699 -0.390595 0.000001
8 -2.623320 -1.494342 0.000001
8 -2.792079 0.750903 0.000008
1 -3.739484 0.518417 0.000019
1 2.022352 -2.192644 0.000005
5caU/5caU04.xyz
6 2.219657 -0.188243 0.023924
6 0.092460 1.097515 -0.101396
6 -0.630363 -0.171108 -0.003294
6 0.095529 -1.327807 0.094883
1 -0.363522 -2.304668 0.197879
7 1.449328 -1.336898 0.108189
7 1.485004 0.968552 -0.086531
8 3.449044 -0.213449 0.046587
8 -0.402880 2.222175 -0.210539
1 2.019388 1.831542 -0.156853

6 -2.107892 -0.178568 0.067402
8 -2.770595 -1.288286 -0.343688
8 -2.769623 0.754810 0.499404
1 -2.210080 -1.925232 -0.817072
1 1.949975 -2.215956 0.201215
5caU/5caU05.xyz
6 -2.227928 -0.075168 -0.000001
6 -0.177387 1.005525 -0.000001
6 0.560666 -0.232111 -0.000001
6 -0.196512 -1.382574 0.000002
1 0.234235 -2.376249 0.000003
7 -1.532652 -1.299953 0.000001
7 -1.493293 1.073578 0.000001
8 -3.467609 -0.098804 -0.000003
8 0.466284 2.172774 0.000004
1 1.441298 1.992391 0.000009
6 2.021492 -0.248289 -0.000001
8 2.568248 -1.470502 0.000002
8 2.705262 0.783822 -0.000005
1 3.539818 -1.385794 -0.000002
1 -2.093207 -2.148331 0.000005
5caU/5caU06.xyz
6 2.232573 -0.041016 -0.000002
6 0.149297 0.989096 0.000002
6 -0.557710 -0.267583 0.000004
6 0.237845 -1.393164 0.000006
1 -0.174397 -2.395102 0.000010
7 1.570472 -1.281900 0.000005
7 1.464465 1.084729 -0.000006
8 3.472845 -0.029277 -0.000006
8 -0.490649 2.162547 0.000004
1 -3.664350 0.537611 -0.000001
6 -2.015664 -0.457766 -0.000001
8 -2.574802 -1.547183 -0.000008
8 -2.704062 0.707663 0.000000
1 -1.460819 2.027506 0.000006

1 2.150295 -2.117226 0.000012
5caU/5caU07.xyz
6 2.083873 -0.045743 0.000005
6 0.042925 1.029693 -0.000008
6 -0.619621 -0.271322 -0.000006
6 0.155532 -1.395041 -0.000012
1 -0.246490 -2.400367 -0.000022
7 1.505937 -1.281687 -0.000008
7 1.419218 1.076497 -0.000002
8 3.411418 -0.094165 0.000010
8 -0.616481 2.103953 -0.000005
1 3.780884 0.807409 0.000044
6 -2.097426 -0.403223 0.000002
8 -2.680302 -1.485487 0.000013
8 -2.786774 0.744250 0.000004
1 -2.121935 1.504431 0.000003
1 2.096864 -2.109725 -0.000016
5caU/5caU08.xyz
6 -2.221212 -0.064239 -0.000002
6 -0.145395 0.965726 0.000007
6 0.579040 -0.272179 0.000002
6 -0.211006 -1.401194 0.000013
1 0.204259 -2.401565 0.000019
7 -1.546513 -1.296283 0.000014
7 -1.454973 1.068288 0.000002
8 -3.459382 -0.055906 -0.000025
8 0.583120 2.091947 0.000010
1 0.011285 2.881843 0.000037
6 2.049398 -0.444951 -0.000004
8 2.581174 -1.551209 -0.000006
8 2.833022 0.649853 -0.000015
1 2.306701 1.475205 0.000002
1 -2.120268 -2.135976 0.000026
5caU/5caU09.xyz
6 2.230250 -0.062906 -0.000033
6 0.167049 0.995195 -0.000035

6 -0.567402 -0.253592 0.000031
6 0.211615 -1.390599 0.000080
1 -0.180155 -2.401285 0.000142
7 1.546412 -1.293254 0.000060
7 1.482399 1.075575 -0.000072
8 3.469662 -0.074962 -0.000077
8 -0.483251 2.154976 -0.000073
1 -1.460057 1.954127 -0.000051
6 -2.035813 -0.247893 0.000038
8 -2.746476 -1.384173 0.000035
8 -2.675928 0.812622 0.000036
1 -2.221916 -2.201078 0.000036
1 2.114189 -2.136942 0.000094
5caU/5caU10.xyz
6 2.216304 -0.144095 -0.000001
6 0.199780 1.015846 0.000002
6 -0.592597 -0.182599 0.000008
6 0.131805 -1.356776 0.000021
1 -0.333104 -2.334016 0.000032
7 1.471638 -1.333929 0.000030
7 1.518104 1.027213 -0.000003
8 3.454321 -0.207040 -0.000027
8 -0.427706 2.193591 -0.000003
1 0.241775 2.903377 -0.000021
6 -2.064408 -0.204931 -0.000001
8 -2.563552 -1.463596 -0.000042
8 -2.792866 0.780585 0.000025
1 -3.536244 -1.408126 -0.000029
1 1.992501 -2.207217 0.000031
5caU/5caU11.xyz
6 2.088728 -0.010958 0.000003
6 0.030000 1.034019 -0.000011
6 -0.618987 -0.273599 -0.000006
6 0.169861 -1.385797 -0.000007
1 -0.217573 -2.396906 -0.000008
7 1.521623 -1.256127 -0.000005

7 1.401953 1.098647 0.000000
8 3.414946 0.088858 0.000009
8 -0.644272 2.100404 0.000005
1 3.876184 -0.768070 -0.000005
6 -2.096149 -0.420458 -0.000001
8 -2.667989 -1.508538 0.000005
8 -2.794805 0.720618 0.000004
1 -2.134368 1.486666 0.000003
1 2.106958 -2.089311 -0.000003
5caU/5caU12.xyz
6 2.079693 -0.046568 0.000095
6 0.046002 0.966024 -0.000032
6 -0.628419 -0.301317 -0.000043
6 0.160985 -1.415681 0.000012
1 -0.225700 -2.426736 0.000011
7 1.511957 -1.279869 0.000079
7 1.387992 1.071868 0.000036
8 3.400952 -0.064069 0.000175
8 -0.650686 2.068679 -0.000076
1 3.756538 0.843618 0.000177
6 -2.133652 -0.369883 -0.000096
8 -2.703683 -1.480282 -0.000084
8 -2.724804 0.778274 -0.000092
1 -1.665859 1.724782 -0.000112
1 2.113494 -2.101922 0.000123
5caU/5caU13.xyz
6 2.213260 -0.128335 0.017444
6 0.184103 1.002432 -0.084804
6 -0.594668 -0.204160 0.002439
6 0.142715 -1.363678 0.094333
1 -0.300999 -2.347886 0.193338
7 1.484258 -1.323383 0.101546
7 1.501106 1.031323 -0.088937
8 3.451726 -0.173444 0.041526
8 -0.466471 2.162115 -0.192414
1 0.185424 2.884104 -0.263409

6 -2.072765 -0.210622 0.067669
8 -2.728762 -1.326142 -0.337096
8 -2.740072 0.723350 0.489196
1 -2.164524 -1.966906 -0.800994
1 2.015307 -2.185744 0.190618
5caU/5caU14.xyz
6 2.090677 -0.122961 -0.000001
6 0.123553 1.126597 0.000000
6 -0.633992 -0.135406 0.000001
6 0.065432 -1.313063 0.000003
1 -0.402134 -2.288355 0.000006
7 1.419253 -1.312162 0.000000
7 1.515263 1.040431 0.000000
8 3.414310 -0.274710 0.000000
8 -0.404058 2.252943 0.000000
1 3.845488 0.598781 -0.000004
6 -2.109704 -0.169585 0.000000
8 -2.607017 -1.431454 -0.000003
8 -2.848342 0.808195 0.000000
1 -3.579620 -1.376007 0.000003
1 1.939715 -2.185573 0.000003
5caU/5caU16.xyz
6 -2.084276 -0.014285 0.000166
6 -0.035480 0.971879 -0.000051
6 0.628601 -0.301090 -0.000052
6 -0.173116 -1.404834 0.000063
1 0.200546 -2.420800 0.000081
7 -1.525398 -1.254819 0.000176
7 -1.372682 1.092519 0.000052
8 -3.400422 0.115278 0.000271
8 0.671953 2.069997 -0.000131
1 -3.881911 -0.731404 0.000300
6 2.134035 -0.386900 -0.000151
8 2.688824 -1.505608 -0.000166
8 2.738715 0.752638 -0.000212
1 1.678063 1.723389 -0.000183

1 -2.121280 -2.082139 0.000269
5caU/5caU15.xyz
6 -2.097731 -0.075922 0.016482
6 -0.080397 1.090077 -0.063107
6 0.622409 -0.201583 -0.012558
6 -0.124720 -1.347604 0.019288
1 0.313048 -2.337288 0.044800
7 -1.477150 -1.292189 0.033412
7 -1.474502 1.061625 -0.032314
8 -3.426294 -0.172049 0.055562
8 0.491550 2.191833 -0.141629
1 -3.822602 0.717649 0.040114
6 2.091178 -0.379887 -0.001577
8 2.642277 -1.462937 -0.191531
8 2.792370 0.737493 0.256902
1 3.740968 0.510589 0.251684
1 -2.033514 -2.142205 0.070115
5caU/5caU17.xyz
6 -2.221049 -0.066735 0.047334
6 -0.157564 0.986266 -0.134655
6 0.569497 -0.248739 -0.014942
6 -0.205168 -1.385468 0.047373
1 0.223135 -2.379536 0.099898
7 -1.541952 -1.294958 0.074016
7 -1.471461 1.063506 -0.096910
8 -3.458389 -0.063268 0.139131
8 0.449171 2.168491 -0.328635
1 1.407594 2.097311 -0.474899
6 2.041182 -0.424913 0.002569
8 2.603520 -1.356162 -0.553651
8 2.795110 0.485233 0.665464
1 2.293084 1.002551 1.320080
1 -2.106614 -2.136979 0.150642
5caU/5caU18.xyz
6 2.096933 -0.086076 0.000191
6 0.109482 1.133970 0.004045

6 -0.634344 -0.136110 -0.002345
6 0.080083 -1.302713 -0.009546
1 -0.372516 -2.285076 -0.016695
7 1.436315 -1.284951 -0.009970
7 1.499169 1.066327 0.007074
8 3.431526 -0.095716 0.002999
8 -0.433663 2.253691 0.008045
1 3.817948 -0.988772 -0.001582
6 -2.110177 -0.188640 -0.002487
8 -2.591795 -1.455950 0.025347
8 -2.859826 0.780202 -0.023937
1 -3.565047 -1.412651 0.016521
1 1.949430 -2.163543 -0.016757
5caU/5caU19.xyz
6 2.103336 -0.040669 0.015465
6 0.067499 1.097021 -0.062641
6 -0.622545 -0.201387 -0.011610
6 0.137986 -1.336569 0.020166
1 -0.285573 -2.332532 0.045980
7 1.492137 -1.265616 0.033638
7 1.458722 1.085556 -0.033684
8 3.436851 0.006328 0.050305
8 -0.518759 2.192157 -0.140388
1 3.862337 -0.868165 0.080522
6 -2.089639 -0.397285 -0.001778
8 -2.626893 -1.486584 -0.194158
8 -2.804056 0.711075 0.258317
1 -3.749917 0.472739 0.253693
1 2.040174 -2.122077 0.069888
5caU/5caU20.xyz
6 -2.082628 -0.104993 -0.015376
6 -0.098545 1.102366 0.117181
6 0.632691 -0.165145 -0.026707
6 -0.077241 -1.323636 -0.154325
1 0.374298 -2.300829 -0.282268
7 -1.435921 -1.296450 -0.155780

7 -1.487192 1.043528 0.118997
8 -3.409543 -0.226669 -0.027292
8 0.468372 2.200936 0.259039
1 -3.819316 0.650310 0.083201
6 2.114816 -0.183521 -0.087794
8 2.755884 -1.218592 0.507738
8 2.783363 0.666814 -0.655836
1 2.161014 -1.757577 1.056904
1 -1.973374 -2.151795 -0.267417
5dhmC/5dhmC05.xyz
6 2.293472 0.039789 0.000003
6 0.116386 0.890061 -0.000011
6 -0.464375 -0.433214 -0.000010
6 0.415186 -1.472300 -0.000013
1 0.101317 -2.509878 -0.000013
7 1.753825 -1.246770 -0.000014
7 1.442676 1.092887 -0.000001
8 3.541447 0.166088 0.000021
6 -1.945077 -0.692205 0.000008
1 -2.138609 -1.770711 0.000018
7 -0.667964 1.979288 -0.000028
1 -0.245288 2.897703 0.000019
1 -1.674430 1.900071 0.000009
8 -2.507710 -0.109630 -1.172228
1 -3.423673 -0.418005 -1.257659
8 -2.507684 -0.109613 1.172251
1 -3.423634 -0.418014 1.257727
1 2.402581 -2.026562 -0.000013
5dhmC/5dhmC01.xyz
6 2.273210 -0.142063 0.056146
6 0.257529 1.021105 -0.141194
6 -0.521353 -0.194594 -0.129307
6 0.187203 -1.353434 -0.017367
1 -0.275154 -2.331271 -0.009270
7 1.540506 -1.327896 0.083279
7 1.595961 1.025186 -0.058508

8 3.522688 -0.206937 0.144480
6 -2.025736 -0.173057 -0.281956
1 -2.308949 0.278425 -1.240174
7 -0.349532 2.217552 -0.265455
1 0.216166 3.052729 -0.189131
1 -1.345490 2.299653 -0.110500
8 -2.650876 0.661792 0.690140
1 -2.353434 0.383955 1.572764
8 -2.507580 -1.498355 -0.203550
1 -3.439627 -1.491180 -0.469866
1 2.058957 -2.195951 0.164485
5dhmC/5dhmC03.xyz
6 -2.290814 0.030654 0.030323
6 -0.121679 0.905334 0.004400
6 0.471801 -0.405926 -0.135441
6 -0.403431 -1.447406 -0.215804
1 -0.081711 -2.476694 -0.328255
7 -1.742355 -1.239874 -0.147057
7 -1.447659 1.088499 0.089849
8 -3.537132 0.140070 0.117811
6 1.955221 -0.690177 -0.108179
1 2.136651 -1.714571 -0.446592
7 0.650928 2.004720 0.067176
1 0.208296 2.912876 0.112653
1 1.637308 1.945091 -0.150208
8 2.511680 -0.651303 1.201649
1 2.332735 0.212156 1.608289
8 2.612765 0.235602 -0.962452
1 3.559032 0.021261 -0.964623
1 -2.383800 -2.023363 -0.208893
5dhmC/5dhmC02.xyz
6 2.277269 -0.134294 0.059123
6 0.257489 1.024392 -0.124552
6 -0.518821 -0.194127 -0.119893
6 0.195429 -1.350426 -0.032628
1 -0.260370 -2.332144 -0.047099

7 1.549117 -1.322786 0.065769
7 1.595821 1.032016 -0.041490
8 3.526755 -0.195488 0.149344
6 -2.024376 -0.190453 -0.290140
1 -2.293466 0.221704 -1.270043
7 -0.353403 2.218748 -0.247598
1 0.208763 3.055884 -0.166689
1 -1.348926 2.293645 -0.085516
8 -2.569690 0.627450 0.739224
1 -3.497496 0.808553 0.519573
8 -2.595117 -1.482039 -0.272088
1 -2.437139 -1.882887 0.599365
1 2.070368 -2.190550 0.130326
5dhmC/5dhmC07.xyz
6 2.278164 -0.136347 0.055511
6 0.258397 1.020906 -0.136509
6 -0.516810 -0.196664 -0.116348
6 0.195166 -1.353154 -0.007516
1 -0.264973 -2.331985 0.003904
7 1.549005 -1.324114 0.084530
7 1.597128 1.028759 -0.058567
8 3.528117 -0.197414 0.140618
6 -2.018389 -0.187043 -0.265137
1 -2.307707 0.254307 -1.228459
7 -0.352075 2.215117 -0.269066
1 0.211564 3.051604 -0.191289
1 -1.345745 2.293551 -0.098269
8 -2.556905 0.619187 0.782945
1 -3.465495 0.861684 0.544414
8 -2.499920 -1.513751 -0.190660
1 -3.405590 -1.527025 -0.534923
1 2.070047 -2.190826 0.163122
5dhmC/5dhmC04.xyz
6 2.271453 -0.135562 0.059148
6 0.251835 1.022850 -0.128770
6 -0.525175 -0.196361 -0.127417

6 0.190485 -1.352715 -0.040681
1 -0.263690 -2.335090 -0.059563
7 1.543556 -1.324461 0.063414
7 1.589986 1.030568 -0.041879
8 3.520653 -0.196765 0.151842
6 -2.034137 -0.187137 -0.300074
1 -2.300396 0.219773 -1.279813
7 -0.358484 2.217155 -0.249214
1 0.203357 3.054992 -0.174209
1 -1.356982 2.292506 -0.106001
8 -2.663810 0.679025 0.638072
1 -2.341253 0.467081 1.529903
8 -2.598585 -1.481248 -0.267053
1 -2.424335 -1.884477 0.599702
1 2.065067 -2.192156 0.127605
5dhmC/5dhmC08.xyz
6 -2.290281 0.043305 0.013669
6 -0.113184 0.893727 -0.004702
6 0.466923 -0.427976 -0.081965
6 -0.414357 -1.465386 -0.121366
1 -0.101185 -2.501909 -0.177438
7 -1.752610 -1.240482 -0.083174
7 -1.438691 1.095609 0.043542
8 -3.537367 0.168319 0.066419
6 1.949023 -0.695161 -0.049213
1 2.130874 -1.771135 -0.164133
7 0.672987 1.981329 0.028893
1 0.249815 2.899228 0.055238
1 1.674081 1.903252 -0.079730
8 2.549182 -0.247633 1.174450
1 2.288638 -0.857582 1.882870
8 2.573460 0.029053 -1.088046
1 3.517269 -0.197083 -1.089316
1 -2.402240 -2.018933 -0.113449
5dhmC/5dhmC09.xyz
6 2.273816 -0.152983 0.056393

6 0.271355 1.030919 -0.145633
6 -0.521142 -0.174487 -0.105831
6 0.172443 -1.338951 0.021294
1 -0.306912 -2.308640 0.052562
7 1.527370 -1.329415 0.108054
7 1.610557 1.020970 -0.073885
8 3.523220 -0.231544 0.137491
6 -2.027462 -0.150158 -0.248362
1 -2.311067 0.348847 -1.185838
7 -0.323786 2.231183 -0.285511
1 0.249273 3.063723 -0.244388
1 -1.319655 2.332416 -0.147547
8 -2.551354 0.566410 0.853098
1 -3.482648 0.768309 0.669624
8 -2.594914 -1.452581 -0.230493
1 -2.593994 -1.806403 -1.133838
1 2.036354 -2.201738 0.200890
5dhmC/5dhmC11.xyz
6 2.267901 -0.152791 0.055894
6 0.264781 1.029467 -0.148736
6 -0.527742 -0.177319 -0.116598
6 0.168042 -1.341356 0.011064
1 -0.308096 -2.312952 0.037300
7 1.522353 -1.330090 0.104538
7 1.603533 1.020734 -0.072606
8 3.517025 -0.230352 0.139091
6 -2.037565 -0.147641 -0.265505
1 -2.315051 0.338857 -1.208356
7 -0.331417 2.229818 -0.281941
1 0.240756 3.062519 -0.231936
1 -1.327971 2.327399 -0.144918
8 -2.649744 0.630369 0.744314
1 -2.304102 0.350867 1.608154
8 -2.605346 -1.448658 -0.214463
1 -2.486874 -1.880845 -1.075178
1 2.032070 -2.202112 0.196750

5dhmC/5dhmC12.xyz
6 2.285798 0.047814 0.004611
6 0.106375 0.891662 0.001291
6 -0.471181 -0.433516 -0.017818
6 0.412790 -1.469863 -0.033582
1 0.101258 -2.508359 -0.051727
7 1.750577 -1.240635 -0.025210
7 1.431908 1.098703 0.014262
8 3.533265 0.177576 0.019330
6 -1.957399 -0.704264 -0.011063
1 -2.122769 -1.789677 -0.052379
7 -0.682380 1.977206 0.009916
1 -0.263545 2.897447 0.005495
1 -1.687166 1.892960 -0.038378
8 -2.637475 -0.065226 -1.083086
1 -2.282966 -0.406171 -1.919066
8 -2.606400 -0.155878 1.128630
1 -2.221535 -0.558225 1.922500
1 2.402578 -2.017669 -0.038853
5dhmC/5dhmC10.xyz
6 2.286708 0.034327 0.031854
6 0.115631 0.903001 0.013404
6 -0.475202 -0.409990 -0.120312
6 0.402648 -1.448824 -0.210443
1 0.082970 -2.478568 -0.326073
7 1.741273 -1.237419 -0.148608
7 1.441047 1.090301 0.094719
8 3.532788 0.146659 0.117000
6 -1.962227 -0.696605 -0.094864
1 -2.135151 -1.719658 -0.443315
7 -0.661886 1.999352 0.072019
1 -0.223978 2.910246 0.108581
1 -1.647444 1.931616 -0.148215
8 -2.712085 0.222583 -0.897318
1 -2.408447 0.154893 -1.817399
8 -2.520000 -0.648733 1.202055

1 -2.307109 0.198723 1.624918
1 2.385149 -2.018422 -0.217148
5dhmC/5dhmC15.xyz
6 -2.311837 -0.147612 0.005331
6 -0.081497 0.863030 0.000688
6 0.466054 -0.435205 -0.078904
6 -0.441942 -1.486858 -0.119212
1 -0.047887 -2.499265 -0.183555
7 -1.778005 -1.393063 -0.083831
7 -1.441047 0.949526 0.036839
8 -3.541187 0.083224 0.059484
6 1.945339 -0.687224 -0.056278
1 2.147231 -1.750724 -0.226726
7 0.630825 1.993619 0.051192
1 0.176159 2.897665 0.067560
1 1.634683 1.954383 -0.063579
8 2.448438 -0.311043 1.224409
1 3.358601 -0.637798 1.302915
8 2.564346 0.084095 -1.083589
1 3.500361 -0.166765 -1.131541
1 -1.881033 1.864930 0.093340
5dhmC/5dhmC13.xyz
6 -2.307599 -0.160167 0.010510
6 -0.089869 0.881388 0.008653
6 0.474629 -0.402568 -0.151781
6 -0.424422 -1.461995 -0.232530
1 -0.018743 -2.463308 -0.364533
7 -1.759674 -1.391052 -0.161102
7 -1.450054 0.944691 0.078373
8 -3.538160 0.049387 0.106845
6 1.957913 -0.675279 -0.146382
1 2.147943 -1.667154 -0.567010
7 0.602594 2.022801 0.108821
1 0.129333 2.914841 0.175915
1 1.598225 2.004250 -0.074253
8 2.517528 -0.742561 1.163799

1 2.328714 0.082547 1.640705
8 2.613070 0.322977 -0.918234
1 3.560931 0.116606 -0.931009
1 -1.899867 1.850433 0.187438
5dhmC/5dhmC16.xyz
6 2.265951 -0.328333 0.067308
6 0.217795 0.991994 -0.142313
6 -0.522792 -0.208224 -0.129982
6 0.216270 -1.383121 -0.022975
1 -0.318216 -2.327192 -0.023136
7 1.549340 -1.481032 0.079470
7 1.572175 0.881791 -0.050498
8 3.514007 -0.280577 0.157355
6 -2.026707 -0.176626 -0.284267
1 -2.310359 0.253498 -1.252470
7 -0.312907 2.218062 -0.263245
1 0.273610 3.039010 -0.173736
1 -1.308391 2.331609 -0.119466
8 -2.646702 0.690292 0.665902
1 -2.352328 0.431975 1.555611
8 -2.521827 -1.494204 -0.171673
1 -3.453459 -1.483401 -0.439095
1 2.141968 1.724532 -0.047104
5dhmC/5dhmC14.xyz
6 2.271027 -0.318446 0.066333
6 0.215501 0.994423 -0.122884
6 -0.520050 -0.209750 -0.120211
6 0.226969 -1.380443 -0.042157
1 -0.299934 -2.329631 -0.067175
7 1.560883 -1.474720 0.055666
7 1.570424 0.889656 -0.032489
8 3.518728 -0.265024 0.159175
6 -2.024878 -0.199938 -0.293562
1 -2.294700 0.176335 -1.287721
7 -0.320457 2.218288 -0.239897
1 0.261832 3.041631 -0.144690

1 -1.315968 2.322270 -0.087367
8 -2.567822 0.664276 0.701472
1 -3.491263 0.848755 0.466660
8 -2.607365 -1.484213 -0.225857
1 -2.431495 -1.862539 0.651829
1 2.135841 1.735211 -0.017925
5dhmC/5dhmC19.xyz
6 2.310171 -0.142347 0.003248
6 0.077737 0.863063 -0.004962
6 -0.467422 -0.436979 -0.084602
6 0.443369 -1.486237 -0.119951
1 0.052079 -2.500121 -0.180535
7 1.779641 -1.389178 -0.082117
7 1.436680 0.953225 0.034225
8 3.539083 0.092103 0.055800
6 -1.948790 -0.692707 -0.056889
1 -2.137374 -1.766272 -0.182175
7 -0.638700 1.990315 0.040834
1 -0.188888 2.896205 0.076575
1 -1.644030 1.947616 -0.056013
8 -2.570671 0.045574 -1.088764
1 -3.516107 -0.173725 -1.089779
8 -2.554929 -0.253234 1.169664
1 -2.281931 -0.858285 1.877590
1 1.874648 1.869746 0.089086
5dhmC/5dhmC17.xyz
6 2.265364 -0.319324 0.066247
6 0.209411 0.992366 -0.126798
6 -0.526530 -0.212876 -0.125741
6 0.222693 -1.383329 -0.049521
1 -0.302102 -2.333544 -0.079017
7 1.556166 -1.476324 0.052625
7 1.564059 0.888397 -0.032592
8 3.512629 -0.265118 0.161589
6 -2.034453 -0.197697 -0.303949
1 -2.300354 0.170556 -1.299122

7 -0.326636 2.215581 -0.241579
1 0.254485 3.040496 -0.154154
1 -1.325711 2.319584 -0.113400
8 -2.660881 0.714578 0.595208
1 -2.339382 0.539588 1.495483
8 -2.611318 -1.483522 -0.218535
1 -2.413421 -1.866891 0.651797
1 2.129015 1.734293 -0.018300
5dhmC/5dhmC18.xyz
6 2.272415 -0.320654 0.065494
6 0.217327 0.990955 -0.133996
6 -0.517753 -0.212171 -0.117113
6 0.226783 -1.383633 -0.016449
1 -0.303991 -2.329756 -0.016198
7 1.560862 -1.476304 0.077750
7 1.572624 0.886291 -0.047284
8 3.520694 -0.267643 0.150674
6 -2.018815 -0.196520 -0.270119
1 -2.307465 0.206966 -1.250374
7 -0.318025 2.215106 -0.260429
1 0.265758 3.037148 -0.162204
1 -1.311079 2.321723 -0.094685
8 -2.556855 0.659345 0.740965
1 -3.461530 0.899245 0.485239
8 -2.512471 -1.514116 -0.140618
1 -3.418981 -1.531243 -0.482509
1 2.138377 1.731713 -0.044595
5dhmC/5dhmC20.xyz
6 2.304115 -0.156228 0.010640
6 0.083148 0.877588 0.021754
6 -0.478096 -0.408360 -0.134341
6 0.424328 -1.464381 -0.225450
1 0.021227 -2.466473 -0.360630
7 1.759695 -1.388910 -0.161511
7 1.443286 0.946031 0.083743
8 3.534279 0.057216 0.101731

6 -1.965014 -0.682389 -0.136109
1 -2.146144 -1.670528 -0.570519
7 -0.614469 2.015972 0.122361
1 -0.145552 2.911119 0.178510
1 -1.610760 1.989669 -0.058006
8 -2.706536 0.309576 -0.858342
1 -2.399759 0.318004 -1.779719
8 -2.531595 -0.742555 1.158334
1 -2.309454 0.062066 1.654860
1 1.890781 1.853224 0.190604
5dhmC/5dhmC22.xyz
6 -2.304167 -0.139501 0.004373
6 -0.071201 0.865317 0.003677
6 0.473243 -0.434289 -0.089977
6 -0.438862 -1.482417 -0.141102
1 -0.047392 -2.495284 -0.218551
7 -1.774771 -1.385629 -0.100857
7 -1.429971 0.955630 0.044961
8 -3.532562 0.094550 0.064892
6 1.957792 -0.697420 -0.063633
1 2.134515 -1.762985 -0.264425
7 0.647408 1.990932 0.062424
1 0.197283 2.897321 0.081961
1 1.648609 1.945817 -0.073066
8 2.552394 -0.333450 1.178790
1 2.105918 -0.827988 1.883354
8 2.686429 0.099906 -0.988798
1 2.379205 -0.113275 -1.883489
1 -1.867715 1.871679 0.109413
5dhmU/5dhmU01.xyz
6 2.292199 -0.003260 -0.000002
6 -0.006210 0.912142 -0.000010
6 -0.515451 -0.444549 -0.000005
6 0.371357 -1.471851 -0.000006
1 0.065455 -2.511762 -0.000001
7 1.724156 -1.256739 -0.000020

7 1.379365 1.031414 -0.000034
8 3.513075 0.182112 0.000032
8 -0.722039 1.930066 0.000005
1 1.767238 1.971882 -0.000003
6 -2.010580 -0.671889 0.000007
1 2.364389 -2.044226 0.000030
8 -2.625531 -0.155818 1.172004
1 -2.403462 0.787793 1.245714
8 -2.625541 -0.155822 -1.171990
1 -2.403501 0.787796 -1.245683
1 -2.222370 -1.742069 0.000008
5dhmU/5dhmU02.xyz
6 -2.295499 -0.023235 0.023464
6 -0.009244 0.931625 0.035859
6 0.518376 -0.410718 -0.107171
6 -0.355509 -1.446326 -0.190288
1 -0.032909 -2.475064 -0.304156
7 -1.709461 -1.259697 -0.130281
7 -1.397804 1.020822 0.097204
8 -3.517989 0.138390 0.087035
8 0.678382 1.966937 0.090717
1 -1.799364 1.949968 0.197479
6 2.010762 -0.634900 -0.134127
1 -2.336877 -2.054549 -0.198873
8 2.681735 0.243911 -1.019808
1 2.406056 1.150598 -0.789663
8 2.487902 -0.479001 1.198730
1 3.428991 -0.716689 1.205297
1 2.231407 -1.642704 -0.498359
5dhmU/5dhmU03.xyz
6 2.258578 -0.255420 0.066045
6 0.208851 1.119537 -0.187220
6 -0.573837 -0.100971 -0.147042
6 0.074590 -1.286166 -0.008343
1 -0.443433 -2.235857 0.027350
7 1.437316 -1.359287 0.092867

7 1.589354 0.939219 -0.083331
8 3.487810 -0.329834 0.165787
8 -0.250728 2.266038 -0.306141
1 2.166843 1.775998 -0.109213
6 -2.077249 0.013250 -0.205041
1 1.891255 -2.261228 0.193304
8 -2.609115 -1.246500 -0.569101
1 -3.564493 -1.138798 -0.693021
8 -2.617690 0.460865 1.033712
1 -2.292509 -0.126541 1.736342
1 -2.372173 0.780966 -0.925965
5dhmU/5dhmU05.xyz
6 2.288192 -0.019633 0.022824
6 0.003192 0.935950 0.036313
6 -0.524235 -0.402437 -0.140530
6 0.349431 -1.437408 -0.234513
1 0.026956 -2.463561 -0.371222
7 1.702986 -1.253100 -0.156094
7 1.390523 1.024470 0.107430
8 3.510099 0.140438 0.098859
8 -0.687214 1.968686 0.109460
1 1.792252 1.951485 0.225482
6 -2.019149 -0.630482 -0.161661
1 2.330871 -2.046879 -0.232792
8 -2.571808 -0.515259 1.157948
1 -2.123771 -1.155629 1.733585
8 -2.705317 0.291250 -0.973688
1 -2.397116 1.182799 -0.723192
1 -2.234415 -1.624666 -0.566444
5dhmU/5dhmU06.xyz
6 2.267713 -0.239847 0.068602
6 0.202074 1.110202 -0.188438
6 -0.566850 -0.117865 -0.129005
6 0.094441 -1.294598 0.015775
1 -0.413293 -2.249300 0.061785
7 1.458448 -1.352326 0.108759

7 1.584956 0.946102 -0.087399
8 3.498202 -0.300399 0.162242
8 -0.270057 2.250059 -0.322562
1 2.153335 1.788541 -0.126605
6 -2.067331 -0.022683 -0.202477
1 1.922464 -2.248582 0.213240
8 -2.594763 -1.298041 -0.519871
1 -3.529813 -1.188146 -0.749665
8 -2.536264 0.428564 1.065736
1 -3.430915 0.785080 0.948958
1 -2.362827 0.703262 -0.968339
5dhmU/5dhmU04.xyz
6 2.271630 -0.230438 0.072702
6 0.195428 1.103784 -0.175533
6 -0.566082 -0.131376 -0.129944
6 0.107575 -1.303380 -0.011030
1 -0.384956 -2.267722 0.002423
7 1.472125 -1.350091 0.089603
7 1.578878 0.951749 -0.066123
8 3.502343 -0.281731 0.169964
8 -0.285271 2.239858 -0.308924
1 2.139776 1.799674 -0.094506
6 -2.071926 -0.039813 -0.238211
1 1.943800 -2.244293 0.175987
8 -2.698421 -1.277920 -0.514210
1 -2.626802 -1.846054 0.270476
8 -2.548849 0.513744 0.979301
1 -3.468139 0.793234 0.842822
1 -2.338875 0.609285 -1.078500
5dhmU/5dhmU12.xyz
6 -2.304363 -0.044368 -0.000002
6 -0.027724 0.954458 -0.000001
6 0.521935 -0.391540 -0.000007
6 -0.337092 -1.443392 -0.000004
1 0.001786 -2.472998 0.000001
7 -1.695216 -1.278847 -0.000014

7 -1.424850 1.014791 -0.000029
8 -3.531285 0.100560 0.000021
8 0.622435 2.009238 0.000019
1 -1.843219 1.941788 -0.000005
6 2.008029 -0.622941 -0.000004
1 -2.305403 -2.089656 0.000043
8 2.560006 -0.040948 -1.174707
1 3.460125 -0.383776 -1.289253
8 2.560010 -0.040969 1.174716
1 3.460133 -0.383792 1.289243
1 2.212990 -1.699520 -0.000010
5dhmU/5dhmU09.xyz
6 2.274836 -0.197852 0.067946
6 0.162153 1.071426 -0.175045
6 -0.560301 -0.184096 -0.148333
6 0.143857 -1.337530 -0.024813
1 -0.324325 -2.312840 -0.006511
7 1.509246 -1.341002 0.082232
7 1.547307 0.965027 -0.069317
8 3.506124 -0.211340 0.168156
8 -0.365967 2.192486 -0.278074
1 2.081643 1.830466 -0.081727
6 -2.065481 -0.124055 -0.265698
1 2.007620 -2.220111 0.174491
8 -2.582462 -1.427355 -0.192247
1 -3.514015 -1.399277 -0.458296
8 -2.639701 0.628289 0.811657
1 -2.373324 1.556273 0.695016
1 -2.347801 0.353311 -1.213656
5dhmU/5dhmU10.xyz
6 2.265220 -0.245462 0.070099
6 0.207227 1.116342 -0.185473
6 -0.568919 -0.106462 -0.115737
6 0.085324 -1.286372 0.029089
1 -0.432634 -2.235804 0.076804
7 1.449433 -1.352962 0.117550

7 1.589684 0.944610 -0.088524
8 3.495756 -0.313197 0.159537
8 -0.259471 2.258028 -0.324541
1 2.163666 1.783013 -0.133069
6 -2.074387 -0.014503 -0.199309
1 1.908864 -2.251664 0.221334
8 -2.681665 -1.276332 -0.452327
1 -2.612638 -1.469483 -1.400592
8 -2.548980 0.437450 1.052697
1 -3.474237 0.710191 0.945070
1 -2.358742 0.693361 -0.987675
5dhmU/5dhmU08.xyz
6 2.274389 -0.189644 0.066105
6 0.154416 1.069327 -0.152365
6 -0.563218 -0.189862 -0.146538
6 0.150681 -1.339867 -0.050382
1 -0.309132 -2.320156 -0.061398
7 1.515554 -1.337329 0.061103
7 1.538949 0.971071 -0.049809
8 3.505444 -0.196627 0.164953
8 -0.381612 2.189342 -0.235508
1 2.067548 1.840259 -0.047988
6 -2.074147 -0.148798 -0.298056
1 2.019251 -2.215210 0.135937
8 -2.672643 -1.415750 -0.213982
1 -2.498247 -1.792271 0.664974
8 -2.666819 0.679058 0.705714
1 -2.255799 1.559641 0.638913
1 -2.332825 0.236424 -1.291498
5dhmU/5dhmU07.xyz
6 2.262878 -0.238108 0.070712
6 0.193636 1.106155 -0.181445
6 -0.574565 -0.126082 -0.142933
6 0.094539 -1.301603 -0.025370
1 -0.400535 -2.264657 -0.017523
7 1.458319 -1.354095 0.083010

7 1.575881 0.947348 -0.069265
8 3.492878 -0.294948 0.172920
8 -0.281539 2.244724 -0.310869
1 2.140696 1.792805 -0.094289
6 -2.083944 -0.021154 -0.239242
1 1.925743 -2.250494 0.170110
8 -2.710985 -1.255028 -0.533243
1 -2.625132 -1.849540 0.229782
8 -2.629288 0.571137 0.930749
1 -2.250478 0.129714 1.708583
1 -2.353478 0.647069 -1.059672
5dhmU/5dhmU11.xyz
6 2.253338 -0.259712 0.066604
6 0.210882 1.125828 -0.186818
6 -0.578914 -0.089761 -0.132909
6 0.063000 -1.277420 0.011037
1 -0.464009 -2.222352 0.054222
7 1.425684 -1.358691 0.106452
7 1.591038 0.938241 -0.089642
8 3.482562 -0.340795 0.160986
8 -0.243683 2.273781 -0.311222
1 2.173928 1.771021 -0.124094
6 -2.087547 0.024208 -0.192336
1 1.875242 -2.262520 0.209697
8 -2.704724 -1.214930 -0.524058
1 -2.488944 -1.436715 -1.443943
8 -2.627050 0.442026 1.044374
1 -2.260449 -0.121302 1.745872
1 -2.374205 0.795505 -0.913534
5dhmU/5dhmU13.xyz
6 2.302619 -0.040092 0.001808
6 0.024064 0.954561 -0.008832
6 -0.523146 -0.392489 -0.030524
6 0.338431 -1.442606 -0.035094
1 0.001132 -2.472858 -0.047061
7 1.696379 -1.275696 -0.022414

7 1.421154 1.017540 0.008336
8 3.529300 0.107197 0.017198
8 -0.628005 2.007956 -0.003579
1 1.837962 1.945115 0.025206
6 -2.011739 -0.629074 -0.011017
1 2.308687 -2.084892 -0.027466
8 -2.637342 -0.039225 1.133296
1 -2.448619 -0.598393 1.903502
8 -2.586536 -0.034784 -1.153792
1 -3.518029 -0.303477 -1.195691
1 -2.204570 -1.709357 -0.002974
5dhmU/5dhmU14.xyz
6 2.297794 -0.036516 0.001935
6 0.017448 0.954731 0.002416
6 -0.527862 -0.393474 -0.006894
6 0.335864 -1.442241 -0.010893
1 -0.000429 -2.473079 -0.016982
7 1.693638 -1.273483 -0.006744
7 1.414763 1.019939 0.005774
8 3.524346 0.112872 0.005804
8 -0.635760 2.007250 0.007142
1 1.830520 1.948161 0.011889
6 -2.020740 -0.635041 -0.005010
1 2.307558 -2.081498 -0.010081
8 -2.662837 -0.047678 1.115347
1 -2.415313 -0.552797 1.904992
8 -2.673486 -0.025984 -1.107682
1 -2.312986 -0.403443 -1.924928
1 -2.201286 -1.718970 -0.012321
5dhmU/5dhmU16.xyz
6 2.283829 0.063020 0.046408
6 0.114172 0.882213 0.010545
6 -0.465487 -0.415929 -0.175415
6 0.427578 -1.444089 -0.282100
1 0.126973 -2.474281 -0.436041
7 1.756608 -1.207755 -0.188531

7 1.409328 1.107522 0.121837
8 3.518206 0.197673 0.163988
8 -0.662811 1.976329 0.081490
1 -1.586076 1.734918 -0.161053
6 -1.952554 -0.678482 -0.149799
1 2.415345 -1.976644 -0.267780
8 -2.472103 -0.769113 1.166505
1 -2.241583 0.037084 1.659965
8 -2.582745 0.383202 -0.865048
1 -3.544859 0.277489 -0.788643
1 -2.180960 -1.642054 -0.612901
5dhmU/5dhmU22.xyz
6 -2.257277 -0.187415 0.064886
6 -0.289615 1.014557 -0.169731
6 0.539953 -0.146002 -0.145949
6 -0.128739 -1.330756 -0.016234
1 0.366140 -2.292676 0.008781
7 -1.479847 -1.343821 0.085623
7 -1.605710 1.005129 -0.076074
8 -3.495858 -0.280384 0.171677
8 0.331166 2.196884 -0.303174
1 -0.332317 2.910385 -0.314123
6 2.044828 -0.039699 -0.216874
1 -1.969931 -2.228061 0.180405
8 2.593323 0.498271 0.980498
1 2.265904 -0.026609 1.729647
8 2.565873 -1.326450 -0.487837
1 3.514731 -1.229724 -0.660947
1 2.343435 0.666848 -0.996494
5dhmU/5dhmU15.xyz
6 2.288317 -0.071212 -0.045042
6 0.231474 0.997703 0.067396
6 -0.506162 -0.232159 0.127931
6 0.256351 -1.366138 0.092442
1 -0.164956 -2.362438 0.142842
7 1.603637 -1.284833 -0.002571

7 1.547435 1.073418 -0.012516
8 3.533852 -0.078176 -0.117163
8 -0.403446 2.180148 0.089948
1 -1.372138 2.016248 -0.005709
6 -2.008569 -0.305297 0.334462
1 2.160326 -2.133774 -0.029589
8 -2.594250 0.784688 -0.379759
1 -3.490525 0.932424 -0.037300
8 -2.556486 -1.537779 -0.070300
1 -2.338086 -1.685232 -1.006258
1 -2.257948 -0.215736 1.396676
5dhmU/5dhmU23.xyz
6 -2.288424 0.049635 0.046437
6 -0.125658 0.890055 0.035668
6 0.465612 -0.399203 -0.171334
6 -0.416558 -1.434852 -0.292378
1 -0.104845 -2.459773 -0.458821
7 -1.747691 -1.213845 -0.198030
7 -1.424347 1.100609 0.142686
8 -3.525117 0.171102 0.154451
8 0.638542 1.990488 0.126930
1 1.567950 1.758152 -0.101662
6 1.951109 -0.647093 -0.170937
1 -2.398166 -1.988489 -0.288532
8 2.572057 0.447871 -0.847419
1 3.535323 0.333644 -0.817690
8 2.391459 -0.723730 1.175437
1 3.283425 -1.106805 1.185649
1 2.188567 -1.581187 -0.691461
5dhmU/5dhmU18.xyz
6 -2.282005 0.054282 0.000001
6 -0.106966 0.846050 -0.000005
6 0.477470 -0.456569 -0.000001
6 -0.419101 -1.486821 -0.000003
1 -0.124450 -2.529910 -0.000003
7 -1.751471 -1.233819 -0.000008

7 -1.398446 1.098893 -0.000004
8 -3.517975 0.210273 0.000011
8 0.756666 1.876314 -0.000012
1 0.274822 2.723115 -0.000008
6 1.971936 -0.697751 0.000007
1 -2.412608 -2.004863 -0.000002
8 2.599398 -0.200694 -1.173158
1 2.400571 0.745567 -1.265996
8 2.599388 -0.200675 1.173169
1 2.400558 0.745587 1.265992
1 2.162704 -1.771908 0.000016
5dhmU/5dhmU20.xyz
6 -2.286325 0.042219 0.015609
6 -0.116145 0.855997 0.022751
6 0.477008 -0.440382 -0.055720
6 -0.411365 -1.476644 -0.107346
1 -0.106276 -2.514667 -0.173598
7 -1.745235 -1.238945 -0.074152
7 -1.411975 1.092459 0.054936
8 -3.523469 0.185382 0.053745
8 0.728246 1.899761 0.053765
1 0.227294 2.735039 0.079745
6 1.967460 -0.678030 -0.072669
1 -2.398977 -2.015076 -0.115790
8 2.636930 0.018118 -1.111796
1 2.457043 0.968301 -1.012591
8 2.478520 -0.310392 1.201515
1 3.412050 -0.574011 1.233989
1 2.163721 -1.736095 -0.270826
5fC/5fC01.xyz
6 1.939733 0.135111 -0.000021
6 -0.285200 0.838817 0.000003
6 -0.776073 -0.529155 0.000009
6 0.184431 -1.519038 0.000001
1 -0.066459 -2.574778 0.000005
7 1.484041 -1.201876 -0.000011

7 1.023680 1.128724 -0.000011
8 3.171430 0.325208 -0.000037
7 -1.150743 1.855532 0.000016
1 -0.803360 2.805923 0.000009
1 -2.146962 1.668030 0.000026
6 -2.169910 -0.918658 0.000022
1 -2.352117 -2.007125 0.000024
8 -3.131302 -0.135211 0.000027
1 2.191141 -1.931150 -0.000019
5fC/5fC02.xyz
6 1.970938 -0.043793 -0.000002
6 -0.323176 0.802029 0.000009
6 -0.774140 -0.549153 0.000000
6 0.222812 -1.543530 0.000009
1 -0.100113 -2.583042 0.000027
7 1.531090 -1.342534 0.000013
7 1.022721 0.990663 0.000026
8 3.173331 0.265670 -0.000031
7 -1.132454 1.851973 -0.000001
1 -0.770445 2.798342 0.000005
1 -2.134080 1.683259 -0.000021
6 -2.164604 -0.917606 -0.000007
1 -2.362161 -2.002973 -0.000019
8 -3.119530 -0.118391 -0.000011
1 1.395913 1.937783 0.000035
5fC/5fC03.xyz
6 1.900278 -0.212471 -0.000001
6 -0.049275 1.078961 0.000003
6 -0.897598 -0.098982 0.000001
6 -0.241214 -1.312428 0.000007
1 -0.777630 -2.254255 0.000008
7 1.094111 -1.370652 0.000013
7 1.289310 0.992091 0.000002
8 3.137027 -0.366480 -0.000014
7 -0.561983 2.316776 0.000003
1 0.071820 3.105290 0.000009

1 -1.554660 2.498843 0.000010
6 -2.355519 -0.084779 -0.000007
1 -2.853770 0.897988 -0.000015
8 -3.055265 -1.104604 -0.000006
1 1.570042 -2.268499 0.000005
5fC/5fC04.xyz
6 1.879039 -0.406782 -0.000001
6 -0.086976 1.057397 0.000008
6 -0.901120 -0.108627 0.000003
6 -0.220152 -1.344560 0.000011
1 -0.828353 -2.245479 0.000023
7 1.089540 -1.528673 0.000008
7 1.258099 0.848614 0.000037
8 3.119846 -0.445313 -0.000032
7 -0.516249 2.317832 -0.000028
1 0.140283 3.089135 0.000032
1 -1.502174 2.538498 0.000024
6 -2.348806 -0.070005 -0.000005
1 -2.830036 0.922166 -0.000010
8 -3.072034 -1.077548 -0.000009
1 1.886144 1.649626 0.000044
5fC/5fC06.xyz
6 1.824869 -0.026501 0.000001
6 -0.299548 0.812030 0.000002
6 -0.799133 -0.533040 -0.000012
6 0.165341 -1.545314 -0.000015
1 -0.154980 -2.584472 -0.000028
7 1.481996 -1.329325 -0.000010
7 1.034297 1.038975 0.000004
8 3.154414 0.185984 0.000011
7 -1.112406 1.871660 0.000003
1 -0.722245 2.805035 0.000038
1 -2.115842 1.730025 0.000016
6 -2.199713 -0.886070 -0.000010
1 -2.412353 -1.969129 -0.000014
8 -3.141863 -0.076086 0.000012

1 3.326912 1.143553 0.000030
5fC/5fC05.xyz
6 -1.820872 0.034974 0.000002
6 0.318026 0.820378 -0.000018
6 0.789902 -0.535792 -0.000016
6 -0.194893 -1.527124 -0.000025
1 0.099473 -2.573886 -0.000022
7 -1.505532 -1.276432 -0.000017
7 -1.009015 1.082213 0.000001
8 -3.132053 0.341055 0.000035
7 1.156506 1.860160 -0.000050
1 0.787948 2.802260 0.000081
1 2.156103 1.694358 0.000057
6 2.182651 -0.919874 0.000015
1 2.370657 -2.007545 0.000025
8 3.143228 -0.131970 0.000035
1 -3.646192 -0.484822 0.000025
5fC/5fC07.xyz
6 1.762018 -0.253838 0.000002
6 -0.099557 1.068407 0.000003
6 -0.910802 -0.114066 -0.000002
6 -0.215996 -1.328285 -0.000006
1 -0.779113 -2.256627 -0.000011
7 1.113325 -1.436316 -0.000008
7 1.250394 0.965892 0.000009
8 3.107651 -0.298263 -0.000002
7 -0.590664 2.316107 -0.000012
1 0.054916 3.094575 0.000016
1 -1.580491 2.511874 0.000007
6 -2.366601 -0.111638 0.000001
1 -2.873388 0.867758 -0.000003
8 -3.062517 -1.135728 0.000007
1 3.391253 -1.228910 0.000033
5fC/5fC08.xyz
6 -1.753833 -0.319887 0.000000
6 0.074884 1.055912 0.000001

6 0.914180 -0.105377 0.000002
6 0.249489 -1.337415 0.000009
1 0.838060 -2.249918 0.000007
7 -1.075486 -1.484197 0.000005
7 -1.272512 0.914557 0.000000
8 -3.091481 -0.468793 -0.000006
7 0.534673 2.315286 0.000001
1 -0.127284 3.079755 0.000008
1 1.519961 2.533422 0.000016
6 2.368649 -0.063642 -0.000005
1 2.848087 0.929384 -0.000013
8 3.092570 -1.068339 -0.000005
1 -3.514476 0.407343 -0.000014
5fC/5fC09.xyz
6 1.983833 0.037645 -0.000011
6 -0.205756 0.845882 0.000026
6 -0.714266 -0.521977 0.000019
6 0.296406 -1.544459 0.000045
1 -0.028063 -2.584343 0.000064
7 1.575162 -1.317099 0.000036
7 1.101794 1.086048 0.000010
8 3.215204 0.263447 -0.000071
7 -1.039691 1.894067 0.000046
1 -0.650372 2.828303 0.000018
1 -2.042861 1.776965 0.000012
6 -2.024719 -0.925871 -0.000019
1 -2.273158 -1.985826 -0.000028
8 -3.038285 -0.074368 -0.000055
1 -3.894739 -0.536155 -0.000065
5fC/5fC10.xyz
6 -1.975130 0.057240 0.000001
6 0.229297 0.720303 -0.000007
6 0.708690 -0.602299 -0.000006
6 -0.324411 -1.570229 -0.000011
1 -0.053776 -2.624872 -0.000018
7 -1.609502 -1.283660 -0.000010

7 -1.019127 1.077658 -0.000009
8 -3.186329 0.370980 0.000021
7 1.206719 1.826881 -0.000006
1 1.077500 2.418893 -0.829697
1 1.077516 2.418876 0.829698
6 2.097202 -0.989847 0.000009
1 2.288010 -2.075025 0.000022
8 3.058872 -0.196625 0.000012
1 2.169881 1.430119 -0.000012
5fC/5fC11.xyz
6 1.905392 -0.344383 0.006655
6 0.065541 1.092213 -0.010046
6 -0.835846 -0.055954 -0.007496
6 -0.183111 -1.338931 -0.047983
1 -0.803353 -2.231469 -0.078708
7 1.103966 -1.511516 -0.049402
7 1.384531 0.921935 -0.001133
8 3.144649 -0.506829 0.060536
7 -0.390817 2.353319 -0.024335
1 0.277674 3.112393 -0.018176
1 -1.373009 2.584964 -0.031425
6 -2.205214 0.040896 0.028342
1 -2.731319 0.991785 0.063117
8 -2.972369 -1.039694 0.028497
1 -3.912568 -0.794695 0.060185
5fC/5fC12.xyz
6 -1.911003 -0.333208 -0.000001
6 -0.055305 1.082923 0.000001
6 0.842402 -0.074068 0.000000
6 0.169160 -1.349600 0.000006
1 0.756686 -2.265699 0.000011
7 -1.118974 -1.508216 0.000007
7 -1.375616 0.925240 0.000000
8 -3.152455 -0.482745 -0.000009
7 0.403898 2.342857 0.000005
1 -0.266066 3.100696 0.000003

1 1.384784 2.579230 0.000005
6 2.213792 0.049534 -0.000006
1 2.706215 1.016871 -0.000013
8 3.120477 -0.915480 -0.000005
1 2.754772 -1.817940 0.000005
5fC/5fC13.xyz
6 -1.960360 0.032973 0.088836
6 0.209583 0.850724 -0.114310
6 0.721072 -0.512895 -0.135042
6 -0.293840 -1.525384 -0.267448
1 0.024320 -2.555420 -0.423922
7 -1.570723 -1.296701 -0.187708
7 -1.079957 1.090459 0.032803
8 -3.161497 0.244494 0.353975
7 1.051106 1.906697 -0.235791
1 0.611486 2.818356 -0.304180
1 1.896208 1.801411 -0.785589
6 2.012215 -0.946374 0.055223
1 2.237946 -2.006693 -0.034446
8 3.085687 -0.249479 0.386487
1 2.901505 0.684780 0.595759
5fC/5fC14.xyz
6 1.909059 -0.315589 0.000001
6 -0.019457 0.950175 0.000009
6 -0.859278 -0.175501 -0.000001
6 -0.139077 -1.397063 0.000016
1 -0.703336 -2.326359 0.000020
7 1.173306 -1.495414 0.000019
7 1.282732 0.930271 0.000015
8 3.159875 -0.359701 -0.000030
7 -0.590404 2.317901 0.000003
1 0.172582 3.005447 -0.000003
1 -1.175686 2.483254 0.829890
6 -2.309766 -0.151071 -0.000014
1 -2.799380 0.837102 -0.000032
8 -3.018225 -1.167510 -0.000009

1 -1.175690 2.483242 -0.829885
5fU/5fU02.xyz
6 -1.901203 -0.265259 0.000000
6 0.139234 1.149097 -0.000001
6 0.933381 -0.071809 0.000000
6 0.285719 -1.281599 0.000000
1 0.828267 -2.220282 0.000006
7 -1.060201 -1.372489 -0.000006
7 -1.243659 0.942657 0.000001
8 -3.125329 -0.373943 0.000002
8 0.584792 2.302370 -0.000003
1 -1.832726 1.772494 -0.000007
6 2.390601 0.000300 0.000006
1 2.815486 1.015699 0.000024
8 3.133391 -0.986046 -0.000004
1 -1.513238 -2.282525 0.000017
5fU/5fU01.xyz
6 1.950774 0.022353 -0.000002
6 -0.369030 0.918288 0.000004
6 -0.854623 -0.459860 0.000004
6 0.066633 -1.479929 0.000006
1 -0.232578 -2.522940 0.000008
7 1.396606 -1.251916 0.000007
7 1.025771 1.038552 0.000000
8 3.165876 0.208264 -0.000011
8 -1.061265 1.939153 0.000009
1 1.402315 1.983996 -0.000002
6 -2.265977 -0.821533 -0.000001
1 -2.454480 -1.910384 0.000004
8 -3.215884 -0.035422 -0.000013
1 2.051625 -2.028994 0.000001
5fU/5fU03.xyz
6 -1.932935 -0.168901 -0.000001
6 0.289872 -0.808769 0.000003
6 0.760695 0.554602 0.000005
6 -0.215189 1.530608 0.000002

1 0.011060 2.591804 0.000001
7 -1.503895 1.176100 -0.000003
7 -0.981847 -1.148685 -0.000004
8 -3.150520 -0.395794 0.000001
8 1.173418 -1.805782 0.000002
1 2.086061 -1.411561 0.000001
6 2.163484 0.897890 0.000001
1 2.412173 1.969943 0.000005
8 3.067766 0.043746 -0.000006
1 -2.229973 1.887962 -0.000006
5fU/5fU05.xyz
6 1.902740 -0.204366 -0.000001
6 -0.049588 1.044986 0.000009
6 -0.896904 -0.111546 0.000001
6 -0.235932 -1.323321 0.000021
1 -0.760759 -2.271582 0.000029
7 1.101304 -1.362439 0.000036
7 1.266807 1.004220 0.000014
8 3.134695 -0.332170 -0.000048
8 -0.650858 2.238702 0.000010
1 0.024405 2.942345 0.000033
6 -2.355133 -0.043249 -0.000025
1 -2.792823 0.966149 -0.000055
8 -3.086933 -1.037504 -0.000015
1 1.586074 -2.256626 0.000030
5fU/5fU04.xyz
6 1.938785 0.095360 -0.000001
6 -0.262445 0.835837 0.000003
6 -0.803291 -0.497695 0.000003
6 0.138438 -1.508525 0.000004
1 -0.134319 -2.558595 0.000006
7 1.446001 -1.221522 0.000001
7 1.025746 1.111046 0.000002
8 3.164070 0.276213 -0.000007
8 -1.126109 1.851412 0.000005
1 -0.634149 2.693337 0.000003

6 -2.212656 -0.870579 0.000001
1 -2.387029 -1.961587 0.000009
8 -3.172147 -0.097095 -0.000010
1 2.133777 -1.970447 0.000000
5fU/5fU06.xyz
6 -1.905208 -0.200375 0.001340
6 0.040026 1.070198 -0.001751
6 0.894098 -0.088007 -0.001471
6 0.234010 -1.303607 -0.005273
1 0.768128 -2.246759 -0.006470
7 -1.099325 -1.355413 -0.005488
7 -1.276471 1.008037 0.000173
8 -3.137017 -0.336109 0.007319
8 0.512690 2.325721 -0.004364
1 1.481271 2.381246 -0.014797
6 2.354773 -0.070900 0.009134
1 2.851500 0.912224 0.030745
8 3.049368 -1.090266 -0.000275
1 -1.576852 -2.253694 -0.005580
5fU/5fU08.xyz
6 1.772144 -0.172942 -0.000002
6 -0.131844 1.156647 0.000000
6 -0.940451 -0.066022 0.000002
6 -0.302961 -1.277845 0.000007
1 -0.829593 -2.224698 0.000013
7 1.048296 -1.335397 0.000005
7 1.253840 1.018598 -0.000008
8 3.086246 -0.385955 -0.000004
8 -0.635484 2.296614 0.000002
1 3.556435 0.467317 -0.000002
6 -2.402102 -0.001023 0.000010
1 -2.832763 1.011633 0.000044
8 -3.139821 -0.990758 -0.000017
1 1.534716 -2.228761 0.000019
5fU/5fU07.xyz
6 -1.806133 0.057858 -0.000008

6 0.349187 0.934737 0.000020
6 0.868893 -0.441058 0.000017
6 -0.029551 -1.478234 0.000029
1 0.267568 -2.521237 0.000040
7 -1.360318 -1.236157 0.000024
7 -1.034117 1.101634 -0.000009
8 -3.134759 0.146498 -0.000041
8 1.080209 1.940143 0.000047
1 -3.397967 1.084629 -0.000062
6 2.285428 -0.797294 -0.000008
1 2.479143 -1.885742 0.000016
8 3.234288 -0.010746 -0.000060
1 -2.032541 -1.999187 0.000030
5fU/5fU09.xyz
6 -1.807274 0.097315 0.007129
6 0.363195 0.938384 -0.017405
6 0.866501 -0.443482 -0.014160
6 -0.045801 -1.466067 -0.027338
1 0.234150 -2.513846 -0.037445
7 -1.374471 -1.203990 -0.025722
7 -1.015010 1.125859 0.009373
8 -3.122231 0.317604 0.039477
8 1.109958 1.932716 -0.042533
1 -3.646320 -0.502460 0.046891
6 2.277975 -0.820876 0.009499
1 2.456349 -1.911892 -0.008664
8 3.236852 -0.048430 0.052932
1 -2.042028 -1.971647 -0.031700
5fU/5fU10.xyz
6 1.959682 0.136407 0.000001
6 -0.256437 0.950351 -0.000001
6 -0.769756 -0.447619 0.000000
6 0.169549 -1.491382 -0.000001
1 -0.118470 -2.538424 -0.000002
7 1.455833 -1.212855 -0.000004
7 1.091019 1.153905 0.000001

8 3.199484 0.246748 0.000004
8 -1.049668 1.917304 -0.000003
1 -3.953169 -0.452893 0.000017
6 -2.100422 -0.836629 0.000001
1 -2.353926 -1.896496 -0.000003
8 -3.095397 0.010158 0.000001
1 2.146548 -1.959986 -0.000004
5fU/5fU11.xyz
6 -1.929350 -0.176772 0.000001
6 -0.005600 1.184626 -0.000002
6 0.852179 -0.030944 0.000004
6 0.225050 -1.285816 -0.000005
1 0.775652 -2.220703 -0.000007
7 -1.088502 -1.349957 -0.000017
7 -1.359655 1.034541 -0.000012
8 -3.152947 -0.400490 0.000022
8 0.516656 2.323258 -0.000009
1 3.954318 -0.706885 0.000049
6 2.231428 0.084755 0.000019
1 2.697738 1.066976 0.000043
8 3.014957 -0.964700 -0.000009
1 -1.562183 -2.251111 -0.000011
5fU/5fU12.xyz
6 1.934329 -0.162681 0.000002
6 -0.005623 1.173456 0.000005
6 -0.855934 -0.054172 0.000004
6 -0.207673 -1.299674 0.000005
1 -0.727856 -2.252439 0.000012
7 1.106321 -1.345256 0.000005
7 1.349677 1.040446 0.000025
8 3.159767 -0.372983 -0.000022
8 -0.539408 2.305779 -0.000013
1 -2.835862 -1.752654 0.000027
6 -2.235756 0.091228 0.000005
1 -2.656354 1.092429 0.000019
8 -3.162678 -0.833021 -0.000016

1 1.590582 -2.240803 0.000006
5hmC/5hmC01.xyz
6 2.014919 0.072368 0.072402
6 -0.172503 0.863142 -0.115844
6 -0.720945 -0.469717 -0.202706
6 0.181941 -1.485800 -0.108718
1 -0.101866 -2.531327 -0.150167
7 1.508877 -1.225614 0.030506
7 1.140457 1.105374 0.008670
8 3.255652 0.230091 0.179515
6 -2.183412 -0.733022 -0.427318
1 -2.523537 -0.249949 -1.349046
1 -2.349495 -1.811537 -0.528247
7 -0.993953 1.927678 -0.179881
1 -0.606440 2.853692 -0.056228
1 -1.993656 1.808331 -0.090802
8 -3.025640 -0.185972 0.608968
1 -2.747744 -0.567689 1.455563
1 2.174975 -1.988359 0.089118
5hmC/5hmC02.xyz
6 2.019666 0.070748 -0.072330
6 -0.167308 0.862531 0.114341
6 -0.716751 -0.470109 0.195325
6 0.185143 -1.486781 0.100426
1 -0.099710 -2.532068 0.137109
7 1.512403 -1.227037 -0.033792
7 1.146053 1.104205 -0.006691
8 3.260582 0.227494 -0.177517
6 -2.175765 -0.727467 0.417867
1 -2.345093 -1.802806 0.543026
1 -2.514954 -0.219203 1.330415
7 -0.987892 1.927532 0.182878
1 -0.599613 2.853749 0.063451
1 -1.987414 1.809500 0.089655
8 -2.926594 -0.226964 -0.712570
1 -3.866814 -0.249645 -0.479166

1 2.177833 -1.990208 -0.094325
5hmC/5hmC03.xyz
6 -2.007634 -0.007066 0.100343
6 0.123642 0.925940 -0.122009
6 0.751995 -0.367285 -0.264657
6 -0.093390 -1.436825 -0.213267
1 0.250896 -2.460225 -0.312566
7 -1.428890 -1.267872 -0.037604
7 -1.198566 1.077728 0.053543
8 -3.250087 0.068239 0.261667
6 2.231749 -0.572297 -0.452073
1 2.404696 -1.569219 -0.862408
1 2.645534 0.154155 -1.162136
7 0.854114 2.055125 -0.175774
1 0.393507 2.946892 -0.055012
1 1.860591 2.044353 -0.251696
8 2.983779 -0.534037 0.782961
1 2.996851 0.372952 1.123581
1 -2.046392 -2.072193 -0.007959
5hmC/5hmC04.xyz
6 1.977109 -0.269463 0.009088
6 0.077839 1.087665 -0.001766
6 -0.818323 -0.041997 -0.030803
6 -0.221286 -1.264285 -0.053804
1 -0.777055 -2.192799 -0.083580
7 1.136471 -1.375105 -0.036983
7 1.413373 0.962899 0.020848
8 3.219922 -0.457078 0.037831
6 -2.321263 0.135736 -0.031860
1 -2.622858 0.755673 0.824339
1 -2.637244 0.658620 -0.941570
7 -0.415185 2.340857 0.001656
1 0.225383 3.122756 0.028952
1 -1.406059 2.533091 -0.011388
8 -3.036822 -1.099717 -0.020263
1 -2.924724 -1.517893 0.847708

1 1.570679 -2.291593 -0.048780
5hmC/5hmC05.xyz
6 -1.980437 -0.267418 -0.000001
6 -0.077388 1.084632 -0.000001
6 0.815176 -0.047787 0.000002
6 0.216144 -1.269858 -0.000001
1 0.767807 -2.200406 -0.000001
7 -1.142295 -1.375537 -0.000004
7 -1.413254 0.963287 -0.000004
8 -3.224227 -0.450931 0.000006
6 2.309866 0.131453 0.000005
1 2.616165 0.705456 -0.886924
1 2.616163 0.705444 0.886943
7 0.417998 2.336854 0.000000
1 -0.221835 3.119776 -0.000006
1 1.409168 2.527900 -0.000002
8 2.943389 -1.151088 -0.000002
1 3.900910 -1.009446 -0.000002
1 -1.578981 -2.290933 -0.000001
5hmC/5hmC07.xyz
6 2.036453 -0.119154 0.078825
6 -0.203599 0.838125 -0.125725
6 -0.725496 -0.469350 -0.213237
6 0.202082 -1.501144 -0.121874
1 -0.166657 -2.523885 -0.173544
7 1.530713 -1.376690 0.015346
7 1.145248 0.960984 0.017763
8 3.257581 0.137846 0.196928
6 -2.190522 -0.719292 -0.425065
1 -2.546112 -0.217702 -1.331261
1 -2.360756 -1.795227 -0.545153
7 -0.946915 1.950211 -0.190055
1 -0.526612 2.863989 -0.076145
1 -1.954038 1.878300 -0.137484
8 -3.019619 -0.190688 0.634325
1 -2.726665 -0.586523 1.469356

1 1.560310 1.887140 0.085301
5hmC/5hmC06.xyz
6 -2.027351 -0.156847 0.085944
6 0.182644 0.869782 -0.127035
6 0.740326 -0.420627 -0.248607
6 -0.158758 -1.479655 -0.172339
1 0.238437 -2.489901 -0.253717
7 -1.488196 -1.398017 -0.015106
7 -1.168092 0.948617 0.032821
8 -3.252760 0.064066 0.228671
6 2.212092 -0.636976 -0.449530
1 2.387389 -1.685200 -0.716595
1 2.597440 -0.014674 -1.263724
7 0.885411 2.008183 -0.169305
1 0.433428 2.907283 -0.062178
1 1.894909 1.981799 -0.195460
8 3.007061 -0.275512 0.703134
1 2.725717 -0.833800 1.444326
1 -1.609312 1.860515 0.123437
5hmC/5hmC08.xyz
6 2.041294 -0.121100 0.079393
6 -0.198124 0.837471 -0.123645
6 -0.721275 -0.469667 -0.205046
6 0.205289 -1.502306 -0.112748
1 -0.164498 -2.524731 -0.160530
7 1.534114 -1.378435 0.019894
7 1.151199 0.959536 0.015909
8 3.262741 0.134793 0.195044
6 -2.182639 -0.714526 -0.415326
1 -2.536308 -0.191577 -1.314348
1 -2.355829 -1.787401 -0.555579
7 -0.939698 1.950548 -0.193258
1 -0.517948 2.864145 -0.083513
1 -1.946786 1.880679 -0.137146
8 -2.921167 -0.230317 0.732808
1 -3.864948 -0.263219 0.514880

1 1.567154 1.885523 0.079836
5hmC/5hmC09.xyz
6 2.019378 -0.200001 0.092219
6 -0.155404 0.900942 -0.128047
6 -0.754894 -0.368673 -0.264783
6 0.113164 -1.456726 -0.203614
1 -0.315524 -2.451973 -0.307735
7 1.442184 -1.422112 -0.036737
7 1.196729 0.931744 0.041659
8 3.248618 -0.020831 0.254512
6 -2.232877 -0.555559 -0.459054
1 -2.639238 0.187131 -1.157192
1 -2.415187 -1.543853 -0.885941
7 -0.807319 2.070855 -0.167923
1 -0.318040 2.946996 -0.037054
1 -1.816061 2.096201 -0.218677
8 -2.994059 -0.530050 0.773836
1 -2.956212 0.362005 1.151112
1 1.666436 1.827231 0.149383
5hmC/5hmC10.xyz
6 -1.950638 -0.460359 0.005204
6 -0.042134 1.066519 -0.003090
6 0.820228 -0.046161 -0.036843
6 0.202652 -1.289003 -0.059540
1 0.831378 -2.173900 -0.094989
7 -1.120860 -1.529370 -0.038486
7 -1.382316 0.819274 0.016173
8 -3.203311 -0.544436 0.037978
6 2.322695 0.147348 -0.045344
1 2.636239 0.649822 -0.967591
1 2.621999 0.790797 0.794713
7 0.368179 2.342815 0.007067
1 -0.296191 3.105114 0.042857
1 1.351731 2.572680 -0.005598
8 3.052476 -1.079560 -0.004686
1 2.924193 -1.490984 0.864085

1 -2.034496 1.599332 0.044582
5hmC/5hmC11.xyz
6 1.955744 -0.456933 -0.000076
6 0.041555 1.062446 0.000059
6 -0.816171 -0.053912 0.000395
6 -0.195466 -1.296027 0.000622
1 -0.819683 -2.183744 0.000958
7 1.129640 -1.529347 0.000384
7 1.382509 0.820498 -0.000166
8 3.209284 -0.535268 -0.000442
6 -2.310416 0.142860 0.000578
1 -2.613070 0.718964 0.887846
1 -2.612885 0.721340 -0.885212
7 -0.374607 2.336844 0.000030
1 0.286679 3.102612 -0.000571
1 -1.359411 2.562009 -0.000019
8 -2.960722 -1.131718 -0.001229
1 -3.915875 -0.975529 -0.000352
1 2.031487 1.603667 -0.000486
5hmC/5hmC12.xyz
6 -1.892623 -0.019231 0.060996
6 0.205045 0.847130 -0.122831
6 0.732394 -0.474315 -0.205575
6 -0.193907 -1.493717 -0.111717
1 0.135820 -2.528662 -0.153699
7 -1.526550 -1.300712 0.021167
7 -1.120555 1.062697 0.006850
8 -3.219373 0.252024 0.180793
6 2.195178 -0.743702 -0.418623
1 2.362034 -1.823673 -0.504656
1 2.543166 -0.272067 -1.343619
7 1.007377 1.934227 -0.201066
1 0.601907 2.843217 -0.021228
1 2.000217 1.825505 -0.044168
8 3.033961 -0.185942 0.617277
1 2.745934 -0.553967 1.466500

1 -3.704212 -0.589483 0.214148
5hmC/5hmC13.xyz
6 1.894116 -0.080565 0.062166
6 -0.186359 0.837517 -0.128060
6 -0.740924 -0.471643 -0.208135
6 0.164303 -1.510824 -0.112933
1 -0.190202 -2.537626 -0.154675
7 1.500577 -1.352126 0.021095
7 1.144872 1.019401 0.003839
8 3.236407 0.093946 0.188781
6 -2.209766 -0.710082 -0.415738
1 -2.552387 -0.229117 -1.337932
1 -2.399277 -1.786180 -0.503026
7 -0.961771 1.943554 -0.210909
1 -0.534932 2.842805 -0.031634
1 -1.956493 1.859194 -0.050890
8 -3.031461 -0.137009 0.625614
1 -2.747541 -0.513492 1.472536
1 3.427292 1.046692 0.208487
5hmC/5hmC14.xyz
6 1.897513 -0.021010 0.061245
6 -0.199524 0.846566 -0.120494
6 -0.728041 -0.474507 -0.197026
6 0.197120 -1.494730 -0.103189
1 -0.133651 -2.529350 -0.141828
7 1.530013 -1.302310 0.025314
7 1.126588 1.061454 0.004888
8 3.224585 0.249114 0.178444
6 -2.187579 -0.737050 -0.409809
1 -2.532709 -0.238081 -1.325575
1 -2.358127 -1.813895 -0.522037
7 -1.000855 1.934232 -0.203568
1 -0.593863 2.843462 -0.028510
1 -1.993025 1.827767 -0.040616
8 -2.935529 -0.225323 0.719269
1 -3.876890 -0.256728 0.491776

1 3.708659 -0.592736 0.214280
5hmC/5hmC15.xyz
6 -1.898938 -0.082150 0.062778
6 0.180945 0.836991 -0.125687
6 0.736561 -0.471776 -0.199673
6 -0.167668 -1.511692 -0.104911
1 0.187737 -2.538173 -0.143841
7 -1.504138 -1.353548 0.024913
7 -1.150748 1.018225 0.002392
8 -3.241402 0.091317 0.187344
6 2.201940 -0.704014 -0.407007
1 2.394497 -1.777036 -0.520005
1 2.541587 -0.196501 -1.320151
7 0.955140 1.943663 -0.213935
1 0.526771 2.843015 -0.038895
1 1.949331 1.861834 -0.049081
8 2.933577 -0.179161 0.726880
1 3.877034 -0.196874 0.506634
1 -3.433184 1.043939 0.204959
5hmC/5hmC16.xyz
6 1.880162 -0.089705 0.076286
6 -0.157047 0.915432 -0.124074
6 -0.764556 -0.366949 -0.266241
6 0.102411 -1.442700 -0.216493
1 -0.288058 -2.451567 -0.325565
7 1.438976 -1.342895 -0.044397
7 1.178015 1.037813 0.045873
8 3.215805 0.091851 0.250817
6 -2.243152 -0.573095 -0.455141
1 -2.660293 0.165952 -1.150775
1 -2.418263 -1.563781 -0.880068
7 -0.864103 2.066286 -0.178551
1 -0.391452 2.940610 0.003955
1 -1.873338 2.066021 -0.177883
8 -2.993911 -0.554883 0.783273
1 -2.970161 0.338879 1.157122

1 3.649289 -0.778183 0.253998
5hmC/5hmC17.xyz
6 1.878889 -0.152040 0.074657
6 -0.137335 0.903462 -0.130375
6 -0.771325 -0.365021 -0.267299
6 0.072757 -1.459848 -0.213676
1 -0.342415 -2.459234 -0.319423
7 1.411022 -1.394230 -0.042504
7 1.201068 0.992201 0.039044
8 3.223986 -0.066482 0.249663
6 -2.254503 -0.537397 -0.451197
1 -2.657199 0.209703 -1.146690
1 -2.454095 -1.524699 -0.873253
7 -0.816421 2.070723 -0.191102
1 -0.323774 2.932923 -0.003491
1 -1.825419 2.092573 -0.176934
8 -3.000310 -0.499964 0.790072
1 -2.955784 0.393616 1.162480
1 3.468699 0.870888 0.328701
5hmC/5hmC18.xyz
6 1.839377 -0.301135 0.004227
6 0.029795 1.081045 -0.002971
6 -0.829791 -0.055007 -0.034526
6 -0.195526 -1.278823 -0.063568
1 -0.778441 -2.193084 -0.098709
7 1.151482 -1.438650 -0.046494
7 1.372088 0.943320 0.022841
8 3.198503 -0.386191 0.043444
6 -2.334102 0.104879 -0.031064
1 -2.639197 0.727027 0.823130
1 -2.660286 0.622229 -0.940778
7 -0.453079 2.343338 -0.003350
1 0.191732 3.119916 0.045104
1 -1.442752 2.539406 -0.000356
8 -3.044238 -1.135035 -0.011242
1 -2.902666 -1.561612 0.848069

1 3.445540 -1.325889 0.022357
5hmC/5hmC22.xyz
6 1.843755 -0.299584 0.000157
6 0.030167 1.076767 -0.000567
6 -0.825673 -0.061782 0.001252
6 -0.189238 -1.285636 0.002639
1 -0.768106 -2.202086 0.004410
7 1.158524 -1.439588 0.001278
7 1.372497 0.943524 0.000851
8 3.203738 -0.379199 -0.000662
6 -2.322237 0.101102 0.000387
1 -2.634931 0.672444 0.887504
1 -2.633272 0.675879 -0.884891
7 -0.457280 2.338731 -0.024493
1 0.186775 3.112679 0.065061
1 -1.442465 2.528219 0.085736
8 -2.952339 -1.184393 -0.002084
1 -3.909980 -1.043987 -0.002137
1 3.453956 -1.318276 -0.000383
5hmC/5hmC21.xyz
6 1.829538 -0.365486 0.002605
6 0.052788 1.066533 -0.004567
6 -0.831899 -0.048599 -0.036143
6 -0.226485 -1.287544 -0.066395
1 -0.833117 -2.186196 -0.103339
7 1.114713 -1.484320 -0.049356
7 1.391942 0.892275 0.020974
8 3.178065 -0.553045 0.042732
6 -2.331596 0.146996 -0.030992
1 -2.620704 0.778632 0.821793
1 -2.648024 0.668518 -0.941777
7 -0.401180 2.339365 -0.002727
1 0.258590 3.103434 0.039412
1 -1.386619 2.556064 -0.007363
8 -3.071024 -1.075922 -0.005752
1 -2.932617 -1.506180 0.852319

1 3.613764 0.314833 0.073843
5hmU/5hmU01.xyz
6 -2.016330 0.000958 0.084014
6 0.276713 0.908048 -0.154201
6 0.778993 -0.451240 -0.222373
6 -0.110548 -1.471955 -0.121031
1 0.188057 -2.513451 -0.153081
7 -1.456864 -1.253711 0.021873
7 -1.104093 1.030928 -0.003728
8 -3.231477 0.190966 0.210894
8 0.985395 1.927427 -0.213367
1 -1.485438 1.972392 0.045586
6 2.255836 -0.663190 -0.400607
1 2.468439 -1.729064 -0.504839
1 2.598602 -0.151355 -1.308176
8 3.022054 -0.203162 0.730521
1 2.936953 0.762658 0.765607
1 -2.095679 -2.039278 0.088700
5hmU/5hmU02.xyz
6 -2.004317 0.046668 0.093795
6 0.154943 0.850496 -0.138624
6 0.741717 -0.446985 -0.240994
6 -0.147999 -1.480691 -0.155907
1 0.147665 -2.521877 -0.215167
7 -1.473702 -1.237813 0.002832
7 -1.130834 1.095390 0.021882
8 -3.234809 0.194742 0.236906
8 1.000967 1.891652 -0.213068
1 0.501572 2.725080 -0.139780
6 2.220832 -0.650004 -0.420777
1 2.418534 -1.702242 -0.635408
1 2.584538 -0.051945 -1.264160
8 2.980568 -0.334035 0.763546
1 2.942604 0.624795 0.902094
1 -2.128024 -2.012619 0.055389
5hmU/5hmU03.xyz

6 -2.006062 0.006836 0.104083
6 0.116961 0.911073 -0.132266
6 0.753318 -0.363283 -0.280242
6 -0.099679 -1.433130 -0.220874
1 0.238528 -2.458902 -0.314833
7 -1.428789 -1.253389 -0.040832
7 -1.178075 1.088469 0.049355
8 -3.239122 0.098370 0.274053
8 0.816657 2.063158 -0.170056
1 1.761463 1.919179 -0.335653
6 2.238269 -0.560341 -0.442840
1 2.423960 -1.562012 -0.835106
1 2.659265 0.157119 -1.159369
8 2.957141 -0.490316 0.806034
1 2.920767 0.418374 1.141962
1 -2.050184 -2.055950 -0.004060
5hmU/5hmU04.xyz
6 -1.894064 -0.091123 0.067082
6 0.300771 0.893581 -0.154914
6 0.790035 -0.460448 -0.216714
6 -0.116183 -1.483497 -0.115877
1 0.226623 -2.513588 -0.149240
7 -1.470510 -1.327846 0.021041
7 -1.084135 0.994212 -0.008313
8 -3.189571 0.212946 0.198500
8 0.996137 1.927444 -0.213144
1 -1.492916 1.925606 0.038423
6 2.266618 -0.677240 -0.392524
1 2.480291 -1.745357 -0.470169
1 2.609780 -0.188353 -1.313094
8 3.036679 -0.189880 0.726110
1 2.938148 0.775174 0.746698
1 -3.708428 -0.609762 0.244224
5hmU/5hmU05.xyz
6 -1.870572 0.060584 0.057979
6 0.277686 0.894660 -0.148469

6 0.783433 -0.474158 -0.195421
6 -0.105648 -1.492804 -0.088761
1 0.168018 -2.540885 -0.101562
7 -1.447139 -1.225402 0.035631
7 -1.085452 1.100869 -0.021724
8 -3.197750 0.185848 0.177509
8 1.055959 1.878466 -0.213034
1 -3.428846 1.131737 0.189240
6 2.259995 -0.697181 -0.370957
1 2.487895 -1.765315 -0.360570
1 2.582093 -0.285491 -1.336450
8 3.032141 -0.100554 0.688512
1 2.851226 0.853837 0.654826
1 -2.124416 -1.978829 0.107043
5hmU/5hmU06.xyz
6 -1.898491 -0.129535 0.067230
6 0.291370 0.884319 -0.162379
6 0.798772 -0.460344 -0.219299
6 -0.096955 -1.493657 -0.117498
1 0.260053 -2.519127 -0.148992
7 -1.451143 -1.358215 0.020490
7 -1.097704 0.966024 -0.016059
8 -3.219315 0.043081 0.207912
8 0.967891 1.930414 -0.223205
1 -1.499845 1.901114 0.028210
6 2.279436 -0.656596 -0.385647
1 2.509938 -1.721823 -0.453306
1 2.621307 -0.169253 -1.307507
8 3.034636 -0.148985 0.734419
1 2.921501 0.814604 0.747064
1 -3.481521 0.978614 0.256061
5hmU/5hmU07.xyz
6 1.872534 0.100971 -0.057348
6 -0.290594 0.901031 0.140885
6 -0.780992 -0.473490 0.191456
6 0.121216 -1.478615 0.085364

1 -0.134913 -2.531151 0.098298
7 1.460230 -1.191899 -0.037477
7 1.068189 1.126731 0.017816
8 3.185492 0.350445 -0.165231
8 -1.083464 1.874130 0.201688
1 3.720693 -0.461531 -0.197062
6 -2.252609 -0.721425 0.374280
1 -2.461213 -1.793627 0.361017
1 -2.575312 -0.320460 1.344125
8 -3.042123 -0.134634 -0.677117
1 -2.877536 0.822606 -0.641150
1 2.132776 -1.950025 -0.110145
5hmU/5hmU08.xyz
6 -2.017370 -0.179273 0.096882
6 0.155782 0.880215 -0.140775
6 0.762175 -0.364042 -0.280190
6 -0.119228 -1.456377 -0.212075
1 0.304544 -2.453607 -0.316039
7 -1.437453 -1.407444 -0.040010
7 -1.183016 0.943562 0.039561
8 -3.240005 -0.002435 0.268496
8 0.743473 2.077426 -0.162767
1 1.704187 2.014095 -0.294798
6 2.245646 -0.546200 -0.448576
1 2.440264 -1.541896 -0.851753
1 2.658302 0.184249 -1.156494
8 2.970395 -0.480089 0.798988
1 2.901913 0.422068 1.147698
1 -1.618870 1.857109 0.145193
5hmU/5hmU10.xyz
6 -1.881431 -0.044267 0.073938
6 0.186083 0.838608 -0.141386
6 0.755710 -0.446371 -0.240384
6 -0.155473 -1.487073 -0.156972
1 0.188799 -2.515589 -0.222548
7 -1.484839 -1.313571 -0.003018

7 -1.117852 1.051425 0.016441
8 -3.200979 0.208270 0.228000
8 1.006694 1.905154 -0.206912
1 0.485767 2.725096 -0.138995
6 2.234196 -0.657674 -0.418192
1 2.429111 -1.712063 -0.625801
1 2.602700 -0.066539 -1.264905
8 2.995352 -0.338338 0.765579
1 2.951571 0.620077 0.904878
1 -3.682155 -0.635991 0.258044
5hmU/5hmU09.xyz
6 -2.023699 -0.152774 0.093235
6 0.179670 0.844334 -0.145868
6 0.748621 -0.419929 -0.256359
6 -0.154844 -1.486462 -0.165700
1 0.241427 -2.497502 -0.237749
7 -1.475118 -1.395767 -0.002210
7 -1.158711 0.953203 0.017648
8 -3.242801 0.064470 0.244976
8 0.942339 1.934691 -0.192736
1 0.442934 2.764892 -0.098549
6 2.229357 -0.607213 -0.438223
1 2.427639 -1.640850 -0.728986
1 2.603072 0.049779 -1.231868
8 2.986092 -0.384338 0.771091
1 2.948136 0.561193 0.980775
1 -1.586074 1.874109 0.099152
5hmU/5hmU11.xyz
6 -1.881202 -0.105420 0.074091
6 0.166584 0.832206 -0.148820
6 0.764755 -0.438607 -0.245531
6 -0.123151 -1.500707 -0.161964
1 0.247569 -2.520000 -0.229107
7 -1.455321 -1.363696 -0.005550
7 -1.143899 1.009775 0.010643
8 -3.215724 0.041044 0.238165

8 0.959128 1.919198 -0.216244
1 0.417672 2.725978 -0.153133
6 2.248268 -0.617710 -0.415777
1 2.465406 -1.665306 -0.635122
1 2.611750 -0.008647 -1.251731
8 2.994063 -0.299812 0.778222
1 2.930624 0.655470 0.930175
1 -3.429748 0.987944 0.290132
5hmU/5hmU13.xyz
6 -1.878965 -0.133504 0.076218
6 0.132678 0.888009 -0.140686
6 0.773179 -0.361041 -0.279830
6 -0.081254 -1.456404 -0.220226
1 0.327368 -2.458446 -0.322395
7 -1.412150 -1.376133 -0.045117
7 -1.184089 1.004852 0.033160
8 -3.215630 -0.039289 0.260645
8 0.779123 2.074352 -0.170138
1 1.731526 1.967038 -0.319889
6 2.260739 -0.534164 -0.439363
1 2.465752 -1.533217 -0.829361
1 2.674428 0.189631 -1.154085
8 2.973613 -0.451718 0.813353
1 2.898945 0.451658 1.156589
1 -3.459460 0.898168 0.345285
5hmU/5hmU12.xyz
6 -1.880202 -0.074369 0.078388
6 0.151310 0.898374 -0.133431
6 0.766676 -0.364392 -0.277435
6 -0.110211 -1.440667 -0.220430
1 0.274078 -2.451982 -0.324677
7 -1.439514 -1.327457 -0.045078
7 -1.160889 1.047036 0.041607
8 -3.206234 0.121417 0.259284
8 0.823797 2.070922 -0.159664
1 1.773047 1.946357 -0.314512

6 2.249566 -0.571279 -0.443692
1 2.429765 -1.576574 -0.829910
1 2.674707 0.139674 -1.164395
8 2.971669 -0.498631 0.804467
1 2.930493 0.410431 1.138623
1 -3.655955 -0.740622 0.266072
5hmU/5hmU14.xyz
6 1.751135 -0.032517 -0.056351
6 -0.308903 0.881911 0.142165
6 -0.797301 -0.482918 0.187291
6 0.108655 -1.505793 0.076755
1 -0.212212 -2.542906 0.093783
7 1.459732 -1.302828 -0.049693
7 1.058849 1.066354 0.016098
8 3.186750 0.211655 -0.224622
8 -1.082093 1.880115 0.204497
1 3.494295 1.056944 0.170787
6 -2.271650 -0.712126 0.370595
1 -2.501505 -1.779661 0.336351
1 -2.588565 -0.325146 1.348869
8 -3.056968 -0.087983 -0.666197
1 -2.841008 0.859820 -0.630015
1 3.745797 -0.535375 0.083242
5hmU/5hmU15.xyz
6 -2.027548 -0.085152 0.098129
6 0.076103 0.773556 -0.133061
6 0.718718 -0.445133 -0.275945
6 -0.210129 -1.499532 -0.202260
1 0.162154 -2.518580 -0.293585
7 -1.519702 -1.362188 -0.028860
7 -1.182939 1.017894 0.045078
8 -3.264778 0.099720 0.263285
8 0.893017 1.989006 -0.110149
1 1.586004 2.051580 -0.804189
6 2.197723 -0.662182 -0.429597
1 2.375416 -1.694877 -0.736594

1 2.617744 -0.007098 -1.203066
8 2.926902 -0.483745 0.803761
1 2.873101 0.451249 1.054248
1 0.333738 2.798587 -0.129114
5mC/5mC01.xyz
6 1.655255 0.134404 -0.000125
6 -0.580878 0.798610 0.000120
6 -1.068311 -0.560117 0.000038
6 -0.102501 -1.519990 -0.000113
1 -0.324186 -2.580974 -0.000181
7 1.219489 -1.184282 -0.000190
7 0.723150 1.117696 0.000041
8 2.892953 0.361357 -0.000225
7 -1.458930 1.819076 0.000286
1 -1.118157 2.770883 0.000318
1 -2.455819 1.661574 0.000335
1 1.927605 -1.909865 -0.000310
6 -2.535921 -0.884203 0.000121
1 -3.038312 -0.472815 0.884227
1 -3.038447 -0.472664 -0.883837
1 -2.688135 -1.966646 0.000041
5mC/5mC02.xyz
6 1.689326 -0.053147 -0.000047
6 -0.609867 0.771044 0.000032
6 -1.071980 -0.560033 0.000012
6 -0.080906 -1.533489 -0.000024
1 -0.391641 -2.576395 -0.000032
7 1.249732 -1.333375 -0.000056
7 0.738296 0.973819 0.000026
8 2.903253 0.272779 -0.000049
7 -1.415753 1.841954 -0.000069
1 -1.044353 2.783136 0.000507
1 -2.418687 1.723236 0.000483
1 1.102320 1.923439 0.000055
6 -2.542003 -0.877397 0.000026
1 -3.046289 -0.467196 0.884101

1 -3.046293 -0.467241 -0.884066
1 -2.694432 -1.959866 0.000056
5mC/5mC04.xyz
6 -1.541502 -0.042272 0.000289
6 0.583170 0.788231 -0.000847
6 1.097792 -0.539722 0.000345
6 0.143419 -1.537555 0.000748
1 0.456387 -2.578522 0.001533
7 -1.195976 -1.325285 -0.000078
7 -0.746600 1.025995 0.001243
8 -2.885541 0.189112 0.000697
7 1.401928 1.864104 -0.020083
1 1.008073 2.791690 0.054227
1 2.401648 1.759280 0.067076
1 -3.034719 1.149234 -0.000458
6 2.574495 -0.819871 0.000119
1 3.065048 -0.395014 -0.884795
1 3.066894 -0.388876 0.881301
1 2.761282 -1.897257 0.004037
5mC/5mC03.xyz
6 1.536834 0.032426 0.000072
6 -0.610918 0.784913 -0.000987
6 -1.082317 -0.560062 0.000515
6 -0.096802 -1.525844 0.001670
1 -0.370629 -2.577687 0.003076
7 1.233642 -1.262572 0.000907
7 0.708483 1.073035 0.000283
8 2.858428 0.368734 0.000247
7 -1.468787 1.830688 -0.022505
1 -1.106666 2.770260 0.063282
1 -2.462358 1.689073 0.082662
1 3.378533 -0.452032 0.000843
6 -2.548844 -0.888464 -0.001027
1 -3.056094 -0.472346 0.878819
1 -3.051304 -0.481611 -0.887613
1 -2.699993 -1.971396 0.004699

5mC/5mC05.xyz
6 -1.694880 0.067934 0.000395
6 0.521116 0.679043 0.002176
6 1.000513 -0.624873 0.000742
6 -0.041771 -1.563736 -0.001160
1 0.206797 -2.623864 -0.002774
7 -1.338472 -1.262852 -0.001710
7 -0.737052 1.065002 0.002636
8 -2.923745 0.394195 -0.000078
7 1.499221 1.791261 -0.001942
1 1.011059 2.693734 0.029085
1 2.083840 1.769301 -0.847885
1 2.134011 1.736835 0.805269
6 2.458718 -0.992500 0.001117
1 2.977143 -0.597561 -0.881154
1 2.975083 -0.601390 0.886469
1 2.573970 -2.079692 -0.000885
5mU/5mU01.xyz
6 1.659856 0.018316 -0.000029
6 -0.662973 0.885503 0.000007
6 -1.154294 -0.481622 0.000025
6 -0.236331 -1.482322 0.000020
1 -0.515030 -2.529452 0.000032
7 1.118476 -1.240992 -0.000004
1 1.772042 -2.016704 -0.000011
7 0.725426 1.030693 -0.000026
8 2.880340 0.231581 -0.000061
8 -1.382490 1.899460 0.000008
1 1.091990 1.979112 -0.000047
6 -2.635357 -0.719720 0.000055
1 -3.105184 -0.269810 0.881834
1 -3.105244 -0.269660 -0.881614
1 -2.854092 -1.790648 -0.000024
5mU/5mU02.xyz
6 1.650315 0.108450 0.000014
6 -0.562975 0.790230 0.000025

6 -1.093056 -0.535652 -0.000029
6 -0.140967 -1.515533 -0.000060
1 -0.379168 -2.572732 -0.000101
7 1.182616 -1.198448 -0.000042
1 1.877983 -1.937887 -0.000066
7 0.719090 1.108123 0.000046
8 2.882304 0.323365 0.000031
8 -1.464288 1.788880 0.000056
1 -0.997410 2.643483 0.000083
6 -2.569449 -0.808409 -0.000037
1 -3.051611 -0.373950 0.882412
1 -3.051623 -0.373854 -0.882433
1 -2.757457 -1.885248 -0.000093
5mU/5mU03.xyz
6 1.658151 0.124499 -0.000266
6 -0.556591 0.802748 0.000126
6 -1.079270 -0.529240 0.000144
6 -0.122430 -1.505883 0.000011
1 -0.358826 -2.563559 0.000029
7 1.197868 -1.185883 -0.000162
1 1.896027 -1.922792 -0.000270
7 0.725328 1.119568 -0.000088
8 2.890063 0.342703 -0.000507
8 -1.384126 1.869435 0.000344
1 -2.317566 1.601408 0.000616
6 -2.552467 -0.828153 0.000276
1 -3.047061 -0.411907 0.886254
1 -3.047223 -0.411813 -0.885568
1 -2.719583 -1.908069 0.000236
5mU/5mU04.xyz
6 1.536127 -0.079237 -0.000018
6 -0.684991 0.876081 0.000021
6 -1.167786 -0.483919 0.000000
6 -0.237149 -1.490593 -0.000027
1 -0.565522 -2.525773 -0.000042
7 1.125195 -1.317908 -0.000035

1 3.363576 -0.577338 -0.000029
7 0.707123 0.993738 0.000007
8 2.837667 0.241845 -0.000019
8 -1.388434 1.907060 0.000048
1 1.105046 1.930704 0.000025
6 -2.648626 -0.723515 0.000014
1 -3.120770 -0.273959 0.881238
1 -3.120803 -0.273868 -0.881144
1 -2.867073 -1.794730 -0.000035
5mU/5mU05.xyz
6 1.512328 0.050422 -0.000013
6 -0.642973 0.903942 -0.000002
6 -1.169059 -0.460892 -0.000004
6 -0.275648 -1.482836 -0.000004
1 -0.555315 -2.529113 -0.000003
7 1.077631 -1.228601 -0.000008
1 1.749712 -1.989518 -0.000005
7 0.730438 1.095675 0.000001
8 2.848751 0.166390 0.000011
8 -1.405215 1.901397 0.000014
1 3.085942 1.110396 0.000026
6 -2.652258 -0.680215 -0.000003
1 -3.114130 -0.219474 0.880551
1 -3.114130 -0.219484 -0.880563
1 -2.891193 -1.747153 0.000003
5mU/5mU06.xyz
6 1.540266 -0.129881 -0.000225
6 -0.666433 0.877995 0.000057
6 -1.180941 -0.467465 0.000227
6 -0.271139 -1.494155 0.000183
1 -0.624522 -2.521276 0.000310
7 1.093332 -1.356390 -0.000035
1 3.123438 0.991558 -0.000622
7 0.730755 0.961446 -0.000184
8 2.870789 0.052658 -0.000493
8 -1.340325 1.928532 0.000104

1 1.130971 1.898038 -0.000330
6 -2.667318 -0.670215 0.000411
1 -3.127453 -0.207835 0.881428
1 -3.127655 -0.207858 -0.880514
1 -2.913717 -1.735210 0.000450
5mU/5mU08.xyz
6 1.512039 0.098931 -0.000007
6 -0.663777 0.900255 0.000034
6 -1.163766 -0.474604 -0.000003
6 -0.248922 -1.475449 -0.000047
1 -0.501634 -2.528598 -0.000080
7 1.099505 -1.190260 -0.000054
1 1.772962 -1.950466 -0.000078
7 0.703309 1.123488 0.000027
8 2.832012 0.350222 -0.000013
8 -1.449413 1.880020 0.000071
1 3.364554 -0.463600 -0.000032
6 -2.641297 -0.728084 0.000005
1 -3.113820 -0.278782 0.880742
1 -3.113830 -0.278783 -0.880727
1 -2.854393 -1.800596 0.000005
5mU/5mU07.xyz
6 1.685915 -0.061910 -0.000116
6 -0.592141 0.773452 0.000033
6 -1.093743 -0.522598 0.000094
6 -0.107706 -1.520438 0.000059
1 -0.437787 -2.557303 0.000109
7 1.214014 -1.339503 -0.000040
1 1.098207 1.929137 -0.000113
7 0.746911 0.973960 -0.000065
8 2.901537 0.232850 -0.000226
8 -1.303063 1.904954 0.000054
1 -2.259019 1.728980 0.000147
6 -2.568223 -0.822574 0.000166
1 -3.065337 -0.408258 0.885437
1 -3.065413 -0.408270 -0.885068

1 -2.729528 -1.903513 0.000181
5mU/5mU11.xyz
6 1.533178 -0.061098 -0.000022
6 -0.567904 0.781383 0.000008
6 -1.122934 -0.512543 0.000010
6 -0.181083 -1.530979 -0.000008
1 -0.509869 -2.566628 -0.000010
7 1.155531 -1.334717 -0.000025
1 3.049415 1.102111 -0.000057
7 0.743943 1.018591 -0.000004
8 2.874063 0.146212 -0.000052
8 -1.410231 1.836419 0.000050
1 -0.899732 2.665088 0.000053
6 -2.605751 -0.747952 0.000032
1 -3.078205 -0.301112 0.881986
1 -3.078244 -0.301023 -0.881856
1 -2.823379 -1.819468 -0.000015
5mU/5mU10.xyz
6 1.680887 -0.072811 -0.000099
6 -0.598668 0.768823 0.000004
6 -1.097434 -0.528116 0.000108
6 -0.115898 -1.526919 0.000117
1 -0.446318 -2.563550 0.000182
7 1.208046 -1.347256 0.000012
1 1.110302 1.917742 -0.000172
7 0.741523 0.968617 -0.000089
8 2.895818 0.225170 -0.000216
8 -1.426723 1.815786 -0.000008
1 -0.965081 2.672989 -0.000061
6 -2.573159 -0.813160 0.000173
1 -3.063568 -0.389189 0.883384
1 -3.063603 -0.389371 -0.883105
1 -2.745846 -1.892698 0.000286
5mU/5mU09.xyz
6 1.529496 0.009761 0.000004
6 -0.593784 0.778685 0.000125

6 -1.108941 -0.533071 -0.000128
6 -0.136734 -1.520927 -0.000313
1 -0.428325 -2.567530 -0.000518
7 1.192539 -1.276066 -0.000251
1 3.362550 -0.497476 -0.000027
7 0.707822 1.063332 0.000190
8 2.848166 0.327249 0.000106
8 -1.471234 1.804724 0.000344
1 -0.985456 2.648014 0.000483
6 -2.583525 -0.815672 -0.000140
1 -3.069896 -0.384644 0.882097
1 -3.070039 -0.383911 -0.881936
1 -2.765887 -1.893755 -0.000561
5mU/5mU13.xyz
6 1.541568 -0.044960 -0.000100
6 -0.561944 0.791718 -0.000079
6 -1.109829 -0.507530 0.000179
6 -0.161856 -1.522480 0.000255
1 -0.488243 -2.558972 0.000419
7 1.171749 -1.322510 0.000114
1 3.052402 1.123917 -0.000450
7 0.749775 1.029580 -0.000195
8 2.881942 0.167094 -0.000264
8 -1.321604 1.912616 -0.000201
1 -2.268810 1.700784 -0.000268
6 -2.590095 -0.770867 0.000327
1 -3.075209 -0.340833 0.885214
1 -3.075353 -0.340982 -0.884555
1 -2.785218 -1.846366 0.000426
5mU/5mU12.xyz
6 1.537576 0.025869 -0.000003
6 -0.587666 0.789492 -0.000018
6 -1.095541 -0.527202 -0.000006
6 -0.117917 -1.511842 -0.000002
1 -0.407380 -2.559119 0.000007
7 1.208198 -1.263559 0.000001

1 3.372272 -0.476707 0.000016
7 0.713784 1.074555 -0.000009
8 2.855854 0.346706 0.000008
8 -1.387400 1.882677 0.000007
1 -2.326120 1.635223 0.000034
6 -2.566783 -0.835351 0.000004
1 -3.064772 -0.420437 0.884944
1 -3.064779 -0.420458 -0.884942
1 -2.728742 -1.916332 0.000017
5mU/5mU14.xyz
6 -1.389170 -0.046872 -0.010260
6 0.672602 0.895491 -0.002164
6 1.186139 -0.463893 0.002592
6 0.279747 -1.494078 -0.000456
1 0.612091 -2.527712 0.004920
7 -1.083069 -1.311478 -0.009995
7 -0.705145 1.059235 -0.009464
8 -2.838990 0.187173 -0.068722
8 1.425095 1.910475 -0.000008
1 -3.367481 -0.550957 0.306071
1 -3.110899 1.041358 0.332613
6 2.669831 -0.685868 0.011001
1 3.138918 -0.239100 -0.873749
1 3.132926 -0.214521 0.885924
1 2.908213 -1.753228 0.025993
5mU/5mU15.xyz
6 1.888512 -1.028624 0.000687
6 0.369243 1.643449 -0.001585
6 -0.705815 0.717408 -0.004689
6 -0.517540 -0.620559 -0.004293
1 -1.373399 -1.283972 -0.006943
7 0.697901 -1.272886 -0.000789
7 1.259160 2.400783 0.000991
8 3.063242 -0.959064 0.002379
8 -4.015269 -0.438267 -0.013700
1 -1.716250 1.111711 -0.008179

1 -4.475394 -0.382444 0.836553
1 -4.724572 -0.431972 -0.672995
C/C01.xyz
6 -1.174133 -0.500042 -0.000098
6 1.143889 -0.255561 -0.000011
6 1.052312 1.176124 0.000137
6 -0.197626 1.709537 0.000175
1 -0.396017 2.774682 0.000286
7 -1.285047 0.890024 0.000076
7 0.064278 -1.053643 -0.000129
8 -2.228486 -1.181984 -0.000199
7 2.354170 -0.842512 0.000014
1 2.428808 -1.850503 -0.000083
1 3.199126 -0.288554 0.000109
1 1.936039 1.801066 0.000226
1 -2.220529 1.281760 0.000103
C/C02.xyz
6 -1.271476 -0.358249 0.000000
6 1.166736 -0.199809 -0.000005
6 1.037851 1.197270 0.000019
6 -0.249845 1.711034 0.000006
1 -0.379733 2.790674 0.000021
7 -1.386772 0.993936 -0.000006
7 0.012050 -0.924009 0.000005
8 -2.250541 -1.141406 0.000010
7 2.336665 -0.850744 -0.000213
1 2.383489 -1.861214 0.000668
1 3.200665 -0.325932 0.000582
1 1.914450 1.831944 0.000036
1 0.052254 -1.939986 -0.000002
C/C03.xyz
6 -1.114514 -0.319235 -0.000191
6 1.160182 -0.266801 -0.002027
6 1.107825 1.149785 0.000671
6 -0.144589 1.720977 0.001292
1 -0.259547 2.800920 0.003474

7 -1.293908 1.001912 0.000119
7 0.027369 -1.003330 -0.000700
8 -2.227672 -1.102109 0.001327
7 2.335549 -0.933342 -0.026423
1 2.340029 -1.938119 0.082982
1 3.205105 -0.432236 0.087872
1 2.010868 1.748811 0.001655
1 -3.011569 -0.527539 0.003957
C/C04.xyz
6 1.135366 -0.270270 0.000015
6 -1.145196 -0.279173 -0.001725
6 -1.126398 1.137238 0.000299
6 0.112843 1.738250 0.001175
1 0.197031 2.821078 0.002996
7 1.282375 1.053288 0.000502
7 0.009005 -0.983008 -0.000346
8 2.303217 -0.968141 0.001194
7 -2.301559 -0.976960 -0.023925
1 -2.281648 -1.982692 0.073564
1 -3.185064 -0.498687 0.080224
1 -2.043734 1.714159 0.000720
1 2.099234 -1.918237 0.000747
C/C05.xyz
6 1.247336 -0.452048 0.000088
6 -1.029743 -0.144515 -0.014020
6 -0.940088 1.235875 -0.011931
6 0.380170 1.705006 0.004105
1 0.568286 2.776416 0.011397
7 1.449917 0.915386 0.010738
7 -0.026529 -0.994193 -0.008241
8 2.245881 -1.232458 -0.001016
7 -2.366147 -0.782993 0.008870
1 -2.368626 -1.636605 -0.562496
1 -3.096383 -0.152001 -0.340095
1 -1.801437 1.891584 -0.019093
1 -2.615621 -1.053037 0.969398

U/U114.xyz
6 -1.205819 -0.394749 -0.000003
6 1.275870 -0.333498 0.000022
6 1.198840 1.107646 0.000028
6 -0.016623 1.709028 0.000002
1 -0.143423 2.784827 -0.000006
7 -1.178967 0.981129 -0.000020
1 -2.077467 1.452753 -0.000044
7 0.039008 -0.986882 0.000004
8 -2.256778 -1.046195 -0.000054
8 2.322087 -1.004601 0.000034
1 0.049663 -2.003672 -0.000008
1 2.114860 1.682166 0.000042
U/U02.xyz
6 1.167081 -0.488976 -0.000001
6 -1.136606 -0.253606 -0.000003
6 -1.086652 1.165182 0.000002
6 0.163468 1.711505 0.000003
1 0.354306 2.777850 0.000006
7 1.253622 0.901934 0.000002
1 2.185531 1.305334 0.000003
7 -0.080814 -1.047687 -0.000003
8 2.215586 -1.165585 -0.000002
8 -2.358420 -0.815601 0.000001
1 -2.266357 -1.785690 0.000001
1 -1.984214 1.767633 0.000005
U/U03.xyz
6 1.185697 -0.471569 -0.000012
6 -1.122316 -0.298983 0.000017
6 -1.106402 1.122708 0.000016
6 0.129641 1.701917 0.000007
1 0.290985 2.773151 0.000008
7 1.239149 0.922117 -0.000006
1 2.160662 1.348993 -0.000014
7 -0.044708 -1.062173 0.000000
8 2.252281 -1.120582 -0.000025

8 -2.286267 -0.979781 0.000006
1 -3.044679 -0.372644 0.000006
1 -2.015886 1.709358 0.000022
U/U04.xyz
6 1.135600 -0.210879 -0.000002
6 -1.281469 -0.364377 -0.000002
6 -1.255202 1.069476 0.000000
6 -0.050440 1.718063 0.000000
1 -0.015268 2.802911 0.000000
7 1.172242 1.097461 -0.000001
1 3.027568 -0.371821 0.000002
7 -0.005833 -0.943282 -0.000001
8 2.247311 -0.954451 0.000001
8 -2.288728 -1.099688 0.000003
1 0.057374 -1.959257 0.000001
1 -2.194136 1.608332 0.000002
U/U05.xyz
6 -1.069044 -0.322290 0.000000
6 1.247727 -0.424825 -0.000001
6 1.276803 1.026978 0.000000
6 0.110640 1.716275 0.000002
1 0.036855 2.796402 0.000003
7 -1.081409 1.033164 0.000002
1 -1.968520 1.527093 0.000003
7 0.009371 -1.056774 -0.000001
8 -2.293183 -0.865376 0.000000
8 2.295852 -1.114577 -0.000002
1 -2.211866 -1.835803 -0.000001
1 2.229698 1.540369 0.000000
U/U134.xyz
6 -1.151300 -0.167555 0.000002
6 1.269289 -0.385824 0.000027
6 1.283348 1.045581 0.000000
6 0.094364 1.724819 0.000000
1 0.090140 2.810352 -0.000012
7 -1.144388 1.141681 0.000003

1 -2.277230 -1.750187 -0.000011
7 -0.025392 -0.928291 0.000008
8 -2.343195 -0.779322 -0.000009
8 2.251078 -1.154511 -0.000017
1 -0.098223 -1.944220 0.000000
1 2.236504 1.558860 -0.000017
U/U07.xyz
6 -1.270776 -0.334843 -0.000001
6 1.153272 -0.233453 0.000000
6 1.096413 1.149363 0.000000
6 -0.186526 1.708205 0.000001
1 -0.279848 2.791391 0.000002
7 -1.333929 1.029126 0.000000
1 0.035124 -1.951003 -0.000001
7 -0.003727 -0.934236 -0.000001
8 -2.274859 -1.075765 0.000001
8 2.250278 -0.992442 0.000000
1 3.054164 -0.444340 0.000001
1 1.996506 1.749742 0.000001
U/U09.xyz
6 1.261995 -0.350367 -0.000003
6 -1.165009 -0.201925 0.000002
6 -1.074482 1.179504 0.000003
6 0.217648 1.711882 0.000001
1 0.334578 2.792663 0.000003
7 1.352609 1.009189 -0.000001
1 -0.062761 -1.943177 -0.000002
7 -0.020523 -0.925918 -0.000001
8 2.247584 -1.115501 -0.000005
8 -2.347348 -0.818606 0.000003
1 -2.276616 -1.790132 0.000001
1 -1.962600 1.796043 0.000006
U/U08.xyz
6 -1.058620 -0.356100 0.000001
6 1.260767 -0.410893 0.000003
6 1.266476 1.041250 0.000002

6 0.088645 1.707950 -0.000001
1 -0.006815 2.786351 0.000002
7 -1.092928 1.002419 -0.000009
1 -1.979198 1.499111 0.000035
7 0.037152 -1.064441 0.000000
8 -2.219736 -1.027616 -0.000001
8 2.322204 -1.081551 -0.000001
1 -2.996792 -0.443166 0.000015
1 2.209885 1.571945 0.000005
U/U10.xyz
6 1.106295 -0.306095 0.000004
6 -1.151981 -0.271845 0.000015
6 -1.147356 1.130437 -0.000004
6 0.101984 1.722472 -0.000005
1 0.202139 2.803420 -0.000016
7 1.254311 1.018021 -0.000001
1 3.003664 -0.495952 0.000006
7 -0.035959 -1.001494 0.000010
8 2.221190 -1.073296 0.000005
8 -2.337932 -0.914169 -0.000015
1 -2.188110 -1.876086 -0.000021
1 -2.065872 1.702830 -0.000017
U/U11.xyz
6 1.126084 -0.256579 -0.000006
6 -1.137242 -0.287126 -0.000002
6 -1.168429 1.113397 0.000001
6 0.066069 1.738030 0.000004
1 0.133067 2.821605 0.000009
7 1.238897 1.069911 0.000002
1 2.125153 -1.884801 0.000008
7 0.001389 -0.982955 -0.000004
8 2.300485 -0.928762 0.000006
8 -2.304101 -0.962904 -0.000004
1 -2.128785 -1.920394 -0.000007
1 -2.101403 1.661903 0.000002
U/U126.xyz

6 1.119408 -0.295515 0.000016
6 -1.137165 -0.311505 0.000014
6 -1.160592 1.091755 0.000001
6 0.076272 1.711623 0.000000
1 0.151076 2.794714 -0.000014
7 1.242060 1.032793 0.000004
1 3.020812 -0.443785 -0.000027
7 -0.005225 -1.015228 0.000014
8 2.251377 -1.038328 -0.000014
8 -2.261169 -1.062471 -0.000014
1 -3.049188 -0.494621 -0.000033
1 -2.089746 1.648975 -0.000013
U/U13.xyz
6 -1.138484 -0.245499 0.000003
6 1.121603 -0.326206 -0.000003
6 1.180336 1.074424 0.000000
6 -0.040767 1.726749 0.000002
1 -0.082418 2.811710 0.000004
7 -1.226278 1.084501 0.000003
1 -2.170063 -1.852028 -0.000006
7 -0.031981 -0.995918 -0.000001
8 -2.327323 -0.892772 -0.000002
8 2.224022 -1.107636 -0.000001
1 3.027144 -0.560896 0.000001
1 2.123427 1.607598 0.000001
U/U156.xyz
6 -2.350061 -0.045250 -0.073657
6 0.653687 -0.632713 0.136913
6 0.651431 0.786371 0.142289
6 -0.482297 1.516243 0.054996
1 -0.428567 2.597993 0.060541
7 -1.766088 1.021156 -0.047105
1 4.911522 -0.169583 -0.155721
7 0.655764 -1.801171 0.127856
8 -3.057988 -0.984245 -0.110481
8 3.975830 0.078621 -0.163113

1 3.499943 -0.759000 -0.061389
1 1.610070 1.287781 0.216817
U/U123.xyz
6 -0.989040 -0.149781 -0.017695
6 1.259288 -0.453524 -0.000039
6 1.333575 0.987791 0.014273
6 0.179664 1.724021 0.006084
1 0.199600 2.808889 0.016216
7 -1.069143 1.152055 -0.012305
1 -3.026448 -0.231466 0.292928
7 -0.016269 -1.012032 -0.019636
8 -2.306591 -0.794813 -0.067284
8 2.273342 -1.204788 0.004864
1 -2.316151 -1.677662 0.364435
1 2.305958 1.465844 0.033627
rd_1m5caC/rd_1m5caC_3.xyz
6 -1.844328 0.659912 0.025346
6 0.417167 1.207438 0.073347
6 0.805180 -0.194483 -0.044930
6 -0.232437 -1.137682 -0.101180
1 -0.017419 -2.198782 -0.164245
7 -1.496333 -0.755734 -0.083116
7 -0.861203 1.578662 0.103687
8 -3.046958 0.924060 0.040143
7 1.364559 2.137036 0.163794
1 1.102208 3.112669 0.246587
1 2.343515 1.897466 0.057123
6 2.224595 -0.675647 -0.037617
8 3.052418 -0.158777 -0.822961
8 2.430903 -1.573503 0.828560
6 -2.598766 -1.726091 -0.138913
1 -3.180551 -1.651166 0.781385
1 -3.238570 -1.490646 -0.990222
1 -2.177721 -2.724215 -0.243441
rd_1m5caC/rd_1m5caC_2.xyz
6 -1.890437 0.573821 0.038728

6 0.478848 1.248983 0.068020
6 0.853543 -0.141532 -0.032778
6 -0.159344 -1.107183 -0.095741
1 0.086259 -2.161152 -0.161321
7 -1.449444 -0.785539 -0.091119
7 -0.893589 1.493664 0.126484
8 -3.083302 0.835758 0.069469
7 1.271083 2.276391 0.116309
1 2.244052 1.968060 0.044010
6 2.290140 -0.607904 -0.015289
8 3.109002 -0.043935 -0.780736
8 2.504681 -1.538825 0.810435
1 -1.193417 2.462014 0.221099
6 -2.505908 -1.806828 -0.185659
1 -3.094331 -1.790544 0.733476
1 -3.149313 -1.572688 -1.034853
1 -2.033700 -2.777419 -0.321160
rd_1m5caC/rd_1m5caC_1.xyz
6 -1.888795 0.546845 0.003068
6 0.480244 1.237346 -0.006373
6 0.853102 -0.157081 -0.000593
6 -0.148564 -1.130454 -0.000636
1 0.096932 -2.186908 0.001573
7 -1.444321 -0.813507 -0.003606
7 -0.896583 1.481447 -0.004040
8 -3.082365 0.808065 0.012830
7 1.381917 2.165475 -0.013807
1 0.959635 3.098855 -0.018433
6 2.299764 -0.575606 0.004605
8 2.821375 -0.754453 -1.124311
8 2.822261 -0.724904 1.137309
1 -1.215043 2.448809 -0.002563
6 -2.493402 -1.846715 -0.003999
1 -3.080708 -1.755748 0.911253
1 -3.139082 -1.693363 -0.869573
1 -2.013092 -2.821219 -0.055132

rd_1m5caU/rd_1m5caU01.xyz
6 -1.876204 0.553200 -0.003006
6 0.493199 1.253540 0.028464
6 0.854363 -0.163250 -0.042934
6 -0.151557 -1.130457 -0.038904
1 0.089819 -2.188030 -0.045773
7 -1.444764 -0.806122 -0.041641
7 -0.874993 1.491376 0.031833
8 -3.063368 0.829674 0.003060
8 1.303207 2.176855 0.079478
6 2.292626 -0.564473 -0.040987
8 2.627988 -0.930797 1.127932
8 2.999658 -0.519713 -1.060348
1 -1.179188 2.463157 0.073009
6 -2.503498 -1.831674 -0.062256
1 -3.083444 -1.759268 0.859216
1 -3.152178 -1.654217 -0.920577
1 -2.030163 -2.807894 -0.140461
rd_1m5caU/rd_1m5caU02.xyz
6 1.867011 -0.598226 0.000011
6 -0.524270 -1.148197 -0.000006
6 -0.806399 0.253041 -0.000002
6 0.225100 1.154317 0.000004
1 0.040016 2.223265 0.000005
7 1.516751 0.758911 0.000008
7 0.807335 -1.489336 0.000000
8 3.034770 -0.970606 0.000009
8 -1.421145 -2.024664 -0.000015
6 -2.219870 0.629770 -0.000007
8 -2.673217 1.786522 -0.000003
8 -3.070115 -0.375526 -0.000012
1 1.055817 -2.476855 -0.000004
6 2.625339 1.732011 0.000013
1 3.236020 1.585494 0.891776
1 3.236042 1.585478 -0.891731
1 2.199694 2.733478 -0.000001

rd_1m5caU/rd_1m5caU10.xyz
6 -1.834351 -0.699659 0.017000
6 0.423085 -1.192384 0.003714
6 0.778158 0.224095 -0.003356
6 -0.278442 1.153502 -0.018629
1 -0.082355 2.220183 -0.035864
7 -1.527748 0.736181 -0.016365
7 -0.823728 -1.607998 0.019139
8 -3.020957 -0.997791 0.039050
8 1.393802 -2.068149 -0.008755
6 2.221672 0.682336 0.005561
8 2.438426 1.909051 0.080985
8 3.085313 -0.259722 -0.059928
1 2.275934 -1.504751 -0.035096
6 -2.661018 1.673461 -0.040858
1 -3.273871 1.458960 -0.917744
1 -3.257096 1.525375 0.861072
1 -2.269585 2.687725 -0.083182
rd_1m5caU/rd_1m5caU14.xyz
6 -1.842228 0.624607 -0.010726
6 0.403443 1.180982 0.044427
6 0.809597 -0.200009 -0.036192
6 -0.217134 -1.152040 -0.031354
1 -0.001648 -2.215121 -0.039432
7 -1.487636 -0.774460 -0.030310
7 -0.847780 1.562862 0.039144
8 -3.035260 0.909470 -0.032391
8 1.384885 2.069908 0.117322
6 2.243667 -0.620158 -0.055665
8 2.586138 -1.037189 1.093094
8 2.941919 -0.542348 -1.080323
1 1.021911 2.974598 0.160952
6 -2.587255 -1.753823 -0.046365
1 -3.200880 -1.610000 0.843853
1 -3.194770 -1.590497 -0.937324
1 -2.158692 -2.753890 -0.056251

rd_1m5caU/rd_1m5caU04.xyz
6 -1.878052 -0.667772 0.052707
6 0.378318 -1.280127 -0.182359
6 0.773400 0.141675 -0.022508
6 -0.224960 1.130693 -0.005083
1 0.019915 2.188131 0.000887
7 -1.491686 0.773761 0.021893
7 -0.934143 -1.598497 -0.088368
8 -3.086183 -0.870084 0.190206
8 1.263017 -2.134444 -0.400226
1 3.866345 0.223943 0.843671
6 2.191577 0.589452 0.029028
8 2.568888 1.535422 -0.647300
8 2.951666 -0.119940 0.855992
6 -2.570319 1.766118 0.043124
1 -3.240025 1.570316 -0.796092
1 -3.128764 1.662457 0.975196
1 -2.135553 2.760441 -0.037168
rd_1m5caU/rd_1m5caU09.xyz
6 1.897382 0.634267 0.020736
6 -0.342490 1.322225 -0.151886
6 -0.786359 -0.087855 -0.002235
6 0.179097 -1.107459 0.047140
1 -0.095407 -2.155853 0.097240
7 1.458894 -0.794133 0.034840
7 0.982401 1.594853 -0.101120
8 3.115850 0.794393 0.114940
8 -1.200792 2.210939 -0.333975
6 -2.228859 -0.436549 0.122615
8 -2.559058 -1.498498 -0.617928
8 -2.989151 0.177791 0.850720
1 -3.500163 -1.721017 -0.477766
6 2.499176 -1.825831 0.055858
1 3.164322 -1.664681 -0.794157
1 3.073642 -1.729209 0.979440
1 2.026065 -2.804073 -0.004218

rd_1m5caU/rd_1m5caU12.xyz
6 1.879830 0.650339 -0.017116
6 -0.377982 1.282773 -0.049109
6 -0.774102 -0.144281 0.017200
6 0.209492 -1.139691 0.058171
1 -0.035821 -2.196271 0.103422
7 1.481421 -0.786486 0.034950
7 0.936982 1.594436 -0.059936
8 3.097446 0.842001 -0.019490
8 -1.282663 2.144390 -0.108443
6 -2.221777 -0.496530 0.115003
8 -2.854363 -0.922303 -0.976865
8 -2.795122 -0.396514 1.184446
1 -2.278051 -0.946168 -1.763907
6 2.553404 -1.785591 0.049615
1 3.136297 -1.686181 -0.868293
1 3.203106 -1.592868 0.904930
1 2.110074 -2.776874 0.118987
rd_1m5caU/rd_1m5caU03.xyz
6 1.880687 0.649335 -0.015051
6 -0.375328 1.286068 -0.086091
6 -0.777091 -0.138103 0.017051
6 0.204720 -1.136183 0.068626
1 -0.043357 -2.191637 0.124786
7 1.477533 -0.786785 0.046146
7 0.940857 1.593887 -0.088296
8 3.098553 0.837866 0.001677
8 -1.274803 2.148994 -0.184412
6 -2.225316 -0.482704 0.128479
8 -2.820504 -1.064268 -0.912867
8 -2.831770 -0.242456 1.156241
1 -2.216932 -1.179782 -1.670433
6 2.547277 -1.787809 0.073596
1 3.134551 -1.695280 -0.842340
1 3.193471 -1.589875 0.930395
1 2.102026 -2.777847 0.147858

rd_1m5caU/rd_1m5caU08.xyz
6 1.869304 -0.648905 0.000157
6 -0.383848 -1.171645 0.000124
6 -0.761439 0.208647 -0.000070
6 0.253315 1.139898 -0.000149
1 0.058185 2.206638 -0.000286
7 1.529927 0.738728 -0.000055
7 0.874559 -1.585219 0.000220
8 3.070173 -0.950385 0.000280
8 -1.344186 -2.064922 0.000233
6 -2.179823 0.466072 -0.000156
8 -2.573651 1.737117 -0.000314
8 -2.985765 -0.486353 -0.000083
1 -3.548605 1.787176 -0.000361
6 2.637143 1.708670 -0.000091
1 3.249899 1.559194 0.889923
1 3.250303 1.558649 -0.889733
1 2.218333 2.713699 -0.000508
rd_1m5caU/rd_1m5caU13.xyz
6 1.726156 -0.592741 0.000153
6 -0.493533 -1.133157 0.000111
6 -0.822646 0.258870 -0.000079
6 0.188468 1.174657 -0.000146
1 0.013739 2.244360 -0.000284
7 1.482124 0.757373 -0.000037
7 0.807023 -1.531062 0.000224
8 3.014897 -0.897697 0.000272
8 -1.430749 -1.999665 0.000183
6 -2.259944 0.589992 -0.000188
8 -2.723244 1.743664 -0.000330
8 -3.042485 -0.459753 -0.000128
1 3.126116 -1.866195 0.000439
6 2.606709 1.717978 -0.000057
1 3.213198 1.564357 0.892889
1 3.213498 1.563964 -0.892730
1 2.190801 2.723344 -0.000362

rd_1m5caU/rd_1m5caU06.xyz
6 1.869663 0.634216 -0.000019
6 -0.384477 1.193571 -0.000076
6 -0.785562 -0.183406 0.000001
6 0.233988 -1.120420 0.000048
1 0.025832 -2.185251 0.000096
7 1.514077 -0.747418 0.000036
7 0.874018 1.566656 -0.000076
8 3.070358 0.927611 -0.000008
8 -1.272514 2.195600 -0.000137
6 -2.160520 -0.612714 0.000036
8 -2.551029 -1.840865 0.000103
8 -3.175560 0.149110 0.000005
1 -2.192176 1.866162 -0.000133
6 2.605355 -1.735566 0.000072
1 3.219481 -1.594051 -0.890136
1 3.219533 -1.593930 0.890225
1 2.169945 -2.733323 0.000156
rd_1m5caU/rd_1m5caU07.xyz
6 -1.863213 -0.659967 -0.000019
6 0.395860 -1.159959 -0.000042
6 0.767064 0.225456 0.000009
6 -0.262310 1.142836 0.000050
1 -0.098457 2.214868 0.000093
7 -1.534651 0.730535 0.000040
7 -0.858718 -1.583729 -0.000055
8 -3.061136 -0.971104 -0.000019
8 1.358213 -2.049333 -0.000108
6 2.193336 0.472117 0.000012
8 2.736592 1.687210 0.000057
8 2.971425 -0.505495 -0.000042
1 2.084761 2.410412 0.000101
6 -2.649498 1.692685 0.000080
1 -3.260920 1.538327 -0.889916
1 -3.260967 1.538199 0.890021
1 -2.239021 2.701335 0.000166

rd_1m5caU/rd_1m5caU15.xyz
6 1.724704 -0.620324 0.000219
6 -0.503265 -1.134967 0.000057
6 -0.823820 0.258699 -0.000072
6 0.194519 1.163419 -0.000127
1 0.028897 2.234419 -0.000265
7 1.487989 0.735951 -0.000019
7 0.790487 -1.543722 0.000228
8 2.976742 -1.060494 0.000447
8 -1.448832 -1.994203 0.000037
1 3.652805 -0.360345 0.000723
6 -2.259335 0.601020 -0.000170
8 -2.712538 1.758499 -0.000250
8 -3.047803 -0.443579 -0.000166
6 2.604959 1.707009 -0.000199
1 3.208444 1.569619 0.899558
1 3.208460 1.569268 -0.899890
1 2.184944 2.710521 -0.000391
rd_1m5caU/rd_1m5caU17.xyz
6 -1.737857 0.589594 -0.009671
6 0.479900 1.260054 0.054960
6 0.871221 -0.152571 -0.036835
6 -0.120938 -1.125387 -0.027176
1 0.106673 -2.185374 -0.026903
7 -1.415830 -0.778593 -0.036421
7 -0.869981 1.551260 0.042033
8 -3.018050 0.924276 -0.028818
8 1.328013 2.161865 0.133146
6 2.311320 -0.542649 -0.068135
8 2.622015 -0.958920 1.097320
8 3.037854 -0.446283 -1.065489
1 -3.635225 0.171943 -0.046511
6 -2.478661 -1.806734 -0.057560
1 -3.087785 -1.712567 0.844899
1 -3.087285 -1.678944 -0.955952
1 -2.004263 -2.785076 -0.077590

rd_1m5dhmC/rd_1m5dhmC_4.xyz
6 1.931927 0.496866 -0.035943
6 -0.245701 1.349263 -0.090737
6 -0.808660 0.019652 -0.106780
6 0.082608 -1.028745 0.023584
1 -0.259493 -2.054628 0.078708
7 1.407121 -0.824554 0.052873
7 1.076515 1.542949 -0.083052
8 3.169405 0.629399 -0.055747
6 -2.291226 -0.273776 -0.113963
1 -2.888737 0.530145 -0.566816
7 -1.030380 2.435129 -0.106418
1 -0.602569 3.352025 -0.089267
1 -2.036709 2.377212 -0.052601
8 -2.471209 -0.350998 1.251476
8 -2.546050 -1.488087 -0.802216
1 -3.504213 -1.637812 -0.787995
6 2.354955 -1.946667 0.123953
1 2.970152 -1.965070 -0.777242
1 2.997913 -1.824389 0.996755
1 1.790284 -2.874218 0.205849
rd_1m5dhmC/rd_1m5dhmC_3.xyz
6 1.931007 0.603688 0.011071
6 -0.323433 1.221501 -0.116671
6 -0.745446 -0.153337 -0.103219
6 0.240519 -1.095362 -0.014512
1 0.021169 -2.155836 -0.003983
7 1.548471 -0.750424 0.062771
7 0.970436 1.557181 -0.063323
8 3.151988 0.876728 0.044880
6 -2.210762 -0.539554 -0.251597
1 -2.567014 -0.118819 -1.223439
7 -1.222131 2.220606 -0.208406
1 -0.891120 3.174339 -0.144753
1 -2.204715 2.042479 -0.050076
8 -3.044404 0.052987 0.732965

1 -2.646853 -0.087521 1.607777
8 -2.398026 -1.873688 -0.420601
6 2.600792 -1.772624 0.135003
1 3.214435 -1.736429 -0.767819
1 3.233309 -1.592067 1.005427
1 2.130830 -2.751779 0.221172
rd_1m5dhmC/rd_1m5dhmC_1.xyz
6 1.987753 0.498320 0.037686
6 -0.192254 1.350705 -0.153723
6 -0.772114 0.039861 -0.146477
6 0.106340 -1.020818 -0.061149
1 -0.229844 -2.049883 -0.058937
7 1.433636 -0.816949 0.013855
7 1.142907 1.547621 -0.058388
8 3.225347 0.610024 0.141623
6 -2.268729 -0.180369 -0.159851
1 -2.766956 0.600710 -0.735978
7 -1.017337 2.411897 -0.271621
1 -0.438382 3.260105 -0.254145
8 -2.815270 -0.075665 1.147953
1 -2.344183 -0.695657 1.729582
8 -2.517442 -1.455400 -0.721315
1 -3.477532 -1.554504 -0.813948
6 2.370814 -1.944955 0.073095
1 3.072270 -1.870389 -0.760106
1 2.929368 -1.901229 1.010356
1 1.808866 -2.875263 0.012682
rd_1m5dhmC/rd_1m5dhmC_2.xyz
6 1.981463 0.581188 0.037955
6 -0.251724 1.297619 -0.141074
6 -0.743995 -0.054861 -0.150877
6 0.204784 -1.049193 -0.078645
1 -0.055943 -2.098787 -0.096206
7 1.518357 -0.758628 0.018092
7 1.066306 1.576123 -0.048613
8 3.209090 0.783949 0.131033

6 -2.220582 -0.384031 -0.279905
1 -2.605273 -0.028827 -1.242286
7 -1.054829 2.386683 -0.229092
1 -2.035409 2.090916 -0.256362
8 -3.004672 0.296831 0.689343
1 -2.642139 0.109569 1.571258
8 -2.374314 -1.783620 -0.179726
1 -3.275388 -2.004597 -0.461141
6 2.522335 -1.827518 0.088877
1 3.205434 -1.735941 -0.757983
1 3.091509 -1.728541 1.015258
1 2.013853 -2.789552 0.061572
rd_1m5dhmU/rd_1m5dhmU_d_3.xyz
6 1.957577 0.409509 0.021320
6 -0.313244 1.405048 0.089889
6 -0.840939 0.052809 0.087861
6 0.024542 -1.009087 -0.013748
1 -0.340735 -2.027789 -0.060389
7 1.370474 -0.857827 -0.033089
7 1.076695 1.466396 0.073665
8 3.177518 0.563486 0.018817
8 -0.986002 2.442884 0.114867
6 -2.333093 -0.167907 0.068945
1 -2.867998 0.728125 0.413174
8 -2.683689 -1.290732 0.856792
1 -3.651596 -1.353860 0.867135
6 2.281024 -2.014869 -0.077380
1 2.920482 -1.938883 -0.957940
1 2.897142 -2.029219 0.823129
1 1.683164 -2.922638 -0.130810
8 -2.498642 -0.360631 -1.290187
1 1.500668 2.391203 0.098038
rd_1m5dhmU/rd_1m5dhmU_d_14.xyz
6 -1.954823 0.414035 -0.022966
6 0.319169 1.401488 -0.096123
6 0.841515 0.048026 -0.091742

6 -0.026103 -1.010231 0.017093
1 0.339209 -2.029089 0.064955
7 -1.371648 -0.854959 0.039497
7 -1.070937 1.468187 -0.084223
8 -3.174464 0.571562 -0.020756
8 0.997208 2.436082 -0.123128
6 2.344547 -0.181171 -0.079259
1 2.871001 0.686702 -0.501627
8 2.752952 -1.377457 -0.719579
1 2.888551 -1.192203 -1.662636
6 -2.285142 -2.009516 0.088813
1 -2.925819 -1.926925 0.967860
1 -2.899997 -2.027854 -0.812520
1 -1.689157 -2.918137 0.148501
8 2.495769 -0.287055 1.279289
1 -1.492398 2.394066 -0.112966
rd_1m5dhmU/rd_1m5dhmU_d_6.xyz
6 -1.960057 0.415414 -0.020301
6 0.316125 1.395173 -0.093239
6 0.839161 0.039094 -0.097683
6 -0.034783 -1.015353 0.008275
1 0.315067 -2.040256 0.052721
7 -1.379903 -0.855255 0.033653
7 -1.073023 1.466518 -0.075818
8 -3.179031 0.576071 -0.014864
8 0.995815 2.428323 -0.115588
6 2.347666 -0.162956 -0.094859
1 2.844229 0.729606 -0.494294
8 2.834094 -1.253309 -0.849734
1 2.599168 -2.084209 -0.404607
6 -2.297607 -2.006951 0.077408
1 -2.940610 -1.923714 0.954629
1 -2.909623 -2.020492 -0.825947
1 -1.705592 -2.918138 0.136759
8 2.498595 -0.301379 1.267254
1 -1.490965 2.394197 -0.098255

rd_1m5dhmU/rd_1m5dhmU_d_8.xyz
6 1.885745 0.644188 0.024910
6 -0.552687 1.083242 0.024002
6 -0.756060 -0.346887 -0.117899
6 0.337184 -1.178836 -0.130555
1 0.219026 -2.254877 -0.198735
7 1.610906 -0.723656 -0.070907
7 0.781470 1.466283 0.055743
8 3.034739 1.076743 0.076690
8 -1.449871 1.938466 0.088112
6 -2.164865 -0.916503 -0.139942
1 -2.191157 -1.913475 -0.595147
8 -3.109655 -0.133944 -0.834472
1 -2.983665 0.795011 -0.559358
6 2.767779 -1.636056 -0.123412
1 3.377765 -1.402769 -0.997454
1 3.364103 -1.515283 0.781976
1 2.397851 -2.657528 -0.191398
8 -2.351540 -0.957064 1.233606
1 0.977471 2.462027 0.132150
rd_1m5dhmU/rd_1m5dhmU_d1_1.xyz
6 -1.929265 0.590936 0.070050
6 0.491371 1.124812 0.002429
6 0.743052 -0.290879 -0.140665
6 -0.306542 -1.155932 -0.180662
1 -0.156453 -2.223945 -0.291478
7 -1.607273 -0.749167 -0.083486
7 -0.854067 1.455076 0.106093
8 -3.093290 0.987471 0.167151
8 1.357832 2.014028 0.035826
6 2.178919 -0.762058 -0.182348
1 2.208302 -1.737089 -0.723107
8 2.640889 -0.936772 1.148587
1 3.564369 -1.235705 1.109066
6 -2.719757 -1.713120 -0.116926
1 -3.269642 -1.673805 0.824953

1 -3.391818 -1.470999 -0.941851
1 -2.306018 -2.709605 -0.261315
8 2.959479 0.048681 -0.951252
1 -1.085314 2.439975 0.211728
rd_1m5dhmU/rd_1m5dhmU_d2_10.xyz
6 -1.921105 0.597609 0.066997
6 0.501569 1.126436 -0.000522
6 0.748094 -0.289567 -0.156153
6 -0.303518 -1.151512 -0.199987
1 -0.157003 -2.219371 -0.317821
7 -1.603505 -0.741951 -0.094380
7 -0.843275 1.459121 0.104155
8 -3.083649 0.997404 0.170736
8 1.370911 2.011639 0.041585
6 2.189034 -0.770373 -0.185323
1 2.197681 -1.760250 -0.701232
8 2.742034 -0.906674 1.120921
1 2.167795 -1.492184 1.640854
6 -2.717386 -1.704507 -0.119568
1 -3.261711 -1.662251 0.825437
1 -3.393953 -1.464090 -0.941263
1 -2.305718 -2.701967 -0.263419
8 2.967265 0.021067 -0.953172
1 -1.072243 2.443917 0.215797
rd_1m5dhmU/rd_1m5dhmU_d1_7.xyz
6 1.951470 0.548343 0.024234
6 -0.423533 1.231713 -0.127497
6 -0.776551 -0.167523 -0.109806
6 0.209841 -1.101770 -0.039167
1 -0.010601 -2.162011 -0.041010
7 1.534840 -0.773877 0.038083
7 0.939207 1.482584 -0.059275
8 3.141110 0.870660 0.082610
8 -1.233868 2.175804 -0.190228
6 -2.253674 -0.535441 -0.227980
1 -2.558690 -0.253910 -1.280434

8 -3.066596 0.183877 0.682230
1 -2.842434 1.129560 0.585936
6 2.577980 -1.810872 0.106272
1 3.209731 -1.755987 -0.782316
1 3.188336 -1.660897 0.997905
1 2.091123 -2.783310 0.154076
8 -2.508370 -1.845057 -0.221538
1 1.232803 2.456641 -0.066743
rd_1m5dhmU/rd_1m5dhmU_a1_8.xyz
6 -1.882243 0.742557 0.017539
6 0.428938 1.116225 0.003637
6 0.678339 -0.343081 -0.041437
6 -0.419659 -1.211370 -0.050670
1 -0.295170 -2.289514 -0.083244
7 -1.648099 -0.724267 -0.020606
7 -0.838425 1.577647 0.025440
8 -3.069565 1.076146 0.039621
8 1.426112 1.881498 0.022415
6 2.090715 -0.880995 -0.049682
1 2.083577 -1.964778 -0.169101
8 2.703639 -0.624205 1.199178
1 2.710062 0.336991 1.353107
6 -2.826374 -1.598481 -0.033778
1 -3.418398 -1.407437 0.862998
1 -3.430015 -1.365516 -0.912849
1 -2.498154 -2.636224 -0.060837
8 2.820948 -0.359664 -1.138102
1 2.786398 0.613491 -1.082466
rd_1m5dhmU/rd_1m5dhmU_a1_10.xyz
6 -1.893156 0.724532 0.074873
6 0.411262 1.148519 0.006544
6 0.686233 -0.302645 -0.109627
6 -0.397961 -1.188330 -0.157631
1 -0.253572 -2.259480 -0.262271
7 -1.634216 -0.729725 -0.076894
7 -0.865615 1.577310 0.104226

8 -3.085522 1.030946 0.160488
8 1.383679 1.944549 0.017200
6 2.099102 -0.838060 -0.121540
1 2.103804 -1.875235 -0.466636
8 2.553642 -0.755548 1.218335
1 3.238967 -1.429921 1.351698
6 -2.795174 -1.625069 -0.132705
1 -3.375013 -1.508182 0.784243
1 -3.417001 -1.344170 -0.984881
1 -2.445442 -2.650875 -0.236922
8 2.919231 -0.120586 -1.014409
1 2.816997 0.826205 -0.788953
rd_1m5dhmU/rd_1m5dhmU_a_11.xyz
6 -1.964954 0.560431 0.027797
6 0.215599 1.409139 -0.163245
6 0.762110 0.031740 -0.152393
6 -0.129394 -1.045694 -0.052673
1 0.214661 -2.073474 -0.043504
7 -1.429219 -0.824631 0.018048
7 -1.123721 1.591186 -0.066091
8 -3.194298 0.637736 0.125571
8 1.010272 2.371193 -0.264602
6 2.252844 -0.190335 -0.166739
1 2.744920 0.591969 -0.747161
8 2.506242 -1.470385 -0.697034
1 3.455091 -1.539303 -0.886496
6 -2.392389 -1.928877 0.080652
1 -2.963284 -1.849792 1.007512
1 -3.076046 -1.846059 -0.766032
1 -1.848066 -2.870740 0.045134
8 2.746803 -0.048047 1.156740
1 2.328254 -0.720898 1.721053
rd_1m5dhmU/rd_1m5dhmU_f1_2.xyz
6 -1.953144 -0.610778 -0.016096
6 0.263623 -1.351893 0.170973
6 0.742820 0.049637 0.164722

6 -0.194491 1.082576 0.040369
1 0.099670 2.125340 0.021822
7 -1.482135 0.796984 -0.044167
7 -1.064128 -1.600883 0.087472
8 -3.178271 -0.747989 -0.096173
8 1.111034 -2.271004 0.256898
6 2.225688 0.310734 0.240652
1 2.664640 -0.280788 1.051670
8 2.441064 1.681875 0.425917
1 3.371798 1.814271 0.663689
6 -2.495194 1.853972 -0.136452
1 -3.098597 1.692238 -1.030747
1 -3.140124 1.799847 0.742882
1 -1.991419 2.817690 -0.182926
8 2.805104 -0.076520 -1.007591
1 2.950627 -1.037686 -0.986938
rd_1m5dhmU/rd_1m5dhmU_f2_3.xyz
6 1.952246 -0.614697 -0.013221
6 -0.267420 -1.347300 0.166698
6 -0.741010 0.056147 0.163795
6 0.199858 1.085665 0.039557
1 -0.090448 2.129501 0.020496
7 1.486395 0.794828 -0.045534
7 1.059320 -1.601734 0.087594
8 3.177099 -0.756282 -0.087962
8 -1.119759 -2.262645 0.246145
6 -2.223107 0.319995 0.247672
1 -2.652555 -0.251729 1.078288
8 -2.817937 -0.097084 -0.983456
1 -2.928137 -1.062851 -0.949366
6 2.503595 1.847497 -0.141174
1 3.145767 1.796484 0.740396
1 3.109037 1.677976 -1.032639
1 2.003709 2.812889 -0.194996
8 -2.437704 1.694793 0.402317
1 -3.365949 1.831969 0.647076

rd_1m5dhmU/rd_1m5dhmU_f1_15.xyz
6 1.959776 0.590279 0.043638
6 -0.239160 1.377342 -0.192857
6 -0.750189 -0.013557 -0.152271
6 0.169713 -1.066169 -0.057762
1 -0.145108 -2.102884 -0.056081
7 1.462821 -0.809066 0.023260
7 1.092703 1.596604 -0.075525
8 3.184014 0.702443 0.167788
8 -1.055625 2.315338 -0.336153
6 -2.235094 -0.262205 -0.174456
1 -2.706209 0.420903 -0.887832
8 -2.709691 0.010097 1.136097
1 -3.559686 0.472165 1.069005
6 2.454340 -1.887583 0.088454
1 3.140555 -1.782532 -0.753889
1 3.017387 -1.798381 1.019041
1 1.935009 -2.843132 0.044736
8 -2.460937 -1.604339 -0.541577
1 -3.389010 -1.695861 -0.806850
rd_1m5dhmU/rd_1m5dhmU_a_6.xyz
6 -1.943644 -0.659996 -0.035553
6 0.304195 -1.309562 0.072778
6 0.730089 0.110804 0.114453
6 -0.260600 1.102046 0.078963
1 -0.012031 2.156673 0.117465
7 -1.535178 0.764821 -0.003658
7 -1.008379 -1.614285 0.003409
8 -3.161403 -0.848799 -0.098647
8 1.189805 -2.202987 0.105262
6 2.187786 0.488682 0.322308
1 2.383199 0.379487 1.401740
8 2.456708 1.832647 0.024816
1 2.351572 1.971987 -0.932118
6 -2.594947 1.777736 -0.042286
1 -3.188296 1.634875 -0.946719

1 -3.238061 1.645516 0.829836
1 -2.136851 2.764836 -0.035355
8 3.062193 -0.332565 -0.427143
1 2.729667 -1.251749 -0.331393
rd_1m5dhmU/rd_1m5dhmU_f1_6.xyz
6 -1.964710 0.591602 0.057119
6 0.234697 1.373739 -0.187795
6 0.746140 -0.018616 -0.146033
6 -0.179106 -1.067902 -0.077526
1 0.125741 -2.108169 -0.099323
7 -1.471510 -0.808380 0.012683
7 -1.095675 1.596302 -0.057740
8 -3.187558 0.705216 0.193236
8 1.051040 2.310362 -0.338989
6 2.239311 -0.258962 -0.192327
1 2.660379 0.402662 -0.956559
8 2.585802 -1.567459 -0.588917
1 2.453387 -2.172878 0.160220
6 -2.465678 -1.884725 0.068726
1 -3.021911 -1.807019 1.004430
1 -3.157274 -1.765487 -0.767350
1 -1.950076 -2.841372 0.007656
8 2.740531 0.060398 1.088902
1 3.677604 0.297856 0.999485
rd_1m5dhmU/rd_1m5dhmU_a_10.xyz
6 -1.962565 0.574983 0.047012
6 0.226817 1.389101 -0.181920
6 0.758035 0.003019 -0.158810
6 -0.153237 -1.058953 -0.080873
1 0.161801 -2.095969 -0.100968
7 -1.449217 -0.817374 0.006650
7 -1.107224 1.592421 -0.059252
8 -3.187876 0.671207 0.174576
8 1.032227 2.338450 -0.310497
6 2.257178 -0.212696 -0.179178
1 2.706550 0.529443 -0.840187

8 2.635745 -1.472000 -0.687747
1 2.463207 -2.164493 -0.028081
6 -2.429206 -1.907016 0.061216
1 -2.986094 -1.837089 0.997248
1 -3.122597 -1.796255 -0.774373
1 -1.901371 -2.856936 -0.000434
8 2.778985 0.035969 1.112347
1 2.318808 -0.523677 1.760895
rd_1m5dhmU/rd_1m5dhmU_f1_4.xyz
6 -1.917028 0.707473 -0.000005
6 0.387788 1.172240 0.000009
6 0.691416 -0.280195 -0.000013
6 -0.377403 -1.186011 -0.000021
1 -0.212235 -2.259015 -0.000034
7 -1.624068 -0.749350 0.000001
7 -0.907181 1.576965 0.000006
8 -3.119673 0.991115 -0.000020
8 1.332541 1.992247 0.000036
6 2.103546 -0.809955 -0.000018
1 2.087645 -1.904058 -0.000079
8 2.734230 -0.338787 1.175381
1 3.384859 -0.998834 1.461213
6 -2.762977 -1.672550 0.000012
1 -3.369764 -1.485209 0.887780
1 -3.369729 -1.485274 -0.887794
1 -2.389894 -2.695249 0.000056
8 2.734308 -0.338669 -1.175331
1 3.384569 -0.998928 -1.461503
rd_1m5dhmU/rd_1m5dhmU_e1_11.xyz
6 1.934229 0.483351 0.020907
6 -0.230098 1.320014 0.090997
6 -0.810262 0.015905 0.095433
6 0.076597 -1.038066 -0.006379
1 -0.263218 -2.065350 -0.048748
7 1.403548 -0.830706 -0.029240
7 1.065826 1.536934 0.071259

8 3.164454 0.629747 0.013096
8 -1.069883 2.359785 0.126180
6 -2.301182 -0.225267 0.075917
1 -2.860195 0.642312 0.450276
8 -2.620872 -1.381192 0.828467
1 -3.586157 -1.477031 0.827667
6 2.354921 -1.952572 -0.079561
1 2.986443 -1.851208 -0.963258
1 2.978817 -1.939151 0.815607
1 1.792204 -2.883378 -0.127941
8 -2.462788 -0.381353 -1.288901
1 -0.566025 3.194120 0.137655
rd_1m5dhmU/rd_1m5dhmU_e2_7.xyz
6 -1.932050 0.487819 -0.026326
6 0.234697 1.317608 -0.091205
6 0.810304 0.011973 -0.093418
6 -0.078513 -1.038507 0.013817
1 0.261412 -2.065878 0.058741
7 -1.405094 -0.827461 0.036625
7 -1.060778 1.539150 -0.079922
8 -3.162088 0.637278 -0.026568
8 1.079744 2.353358 -0.124556
6 2.312310 -0.238481 -0.082330
1 2.859777 0.607707 -0.519423
8 2.691000 -1.452433 -0.707427
1 2.838033 -1.281261 -1.651466
6 -2.359218 -1.946734 0.092914
1 -2.996278 -1.834185 0.971243
1 -2.977555 -1.942623 -0.806221
1 -1.798607 -2.877945 0.155681
8 2.462416 -0.330719 1.277643
1 0.580559 3.190431 -0.138926
rd_1m5dhmU/rd_1m5dhmU_e_7.xyz
6 1.937418 0.487901 0.024602
6 -0.230764 1.312527 0.085865
6 -0.807711 0.005044 0.097068

6 0.086461 -1.042566 -0.006003
1 -0.239453 -2.075224 -0.046863
7 1.412555 -0.828392 -0.031211
7 1.063752 1.537017 0.071070
8 3.166845 0.639623 0.024582
8 -1.076088 2.347854 0.112298
6 -2.315488 -0.218356 0.096486
1 -2.834358 0.657006 0.503119
8 -2.775710 -1.323013 0.847445
1 -2.529035 -2.146757 0.395510
6 2.369625 -1.945577 -0.083032
1 3.009277 -1.831627 -0.959233
1 2.984690 -1.938593 0.818327
1 1.812147 -2.878541 -0.147067
8 -2.464716 -0.355171 -1.266106
1 -0.577302 3.185184 0.121523
rd_1m5fC/rd_1m5fC_2.xyz
6 1.544747 0.742181 -0.000259
6 -0.798461 0.978868 -0.000091
6 -0.982028 -0.455296 0.000144
6 0.165008 -1.226444 0.000149
1 0.123290 -2.311212 0.000314
7 1.384433 -0.668299 -0.000055
7 0.424699 1.530421 -0.000291
8 2.690005 1.211854 -0.000385
7 -1.827661 1.865512 -0.000146
1 -2.710188 1.336561 0.000051
6 -2.262309 -1.154761 0.000403
1 -2.186533 -2.255362 0.000572
8 -3.371798 -0.612559 0.000400
6 2.606391 -1.486476 0.000028
1 3.195171 -1.253786 -0.889143
1 3.195138 -1.253653 0.889187
1 2.327095 -2.538773 0.000100
rd_1m5fC/rd_1m5fC_3.xyz
6 1.603962 0.582475 0.006888

6 -0.644377 1.257578 -0.005763
6 -1.109143 -0.101450 -0.000204
6 -0.136132 -1.088644 -0.005923
1 -0.394678 -2.141429 -0.007015
7 1.164023 -0.777136 -0.010542
7 0.663558 1.568187 -0.003094
8 2.822236 0.804082 0.028071
7 -1.563532 2.256000 -0.014901
1 -1.054304 3.148280 -0.020308
6 -2.527664 -0.468165 0.008365
1 -3.244215 0.365111 0.009755
8 -2.924924 -1.635967 0.014437
6 2.198769 -1.821170 -0.015363
1 2.856600 -1.666536 -0.871945
1 2.783693 -1.750201 0.903686
1 1.713577 -2.793244 -0.082483
rd_1m5fC/rd_1m5fC_1.xyz
6 -1.546990 0.720112 -0.029046
6 0.795854 0.986436 0.022572
6 1.006454 -0.439985 0.023647
6 -0.134158 -1.223960 0.034036
1 -0.077959 -2.307713 0.052643
7 -1.362849 -0.686656 0.016455
7 -0.437191 1.519435 -0.018378
8 -2.698714 1.171867 -0.075490
7 1.853854 1.831470 0.107047
1 1.480360 2.785957 0.026810
6 2.284474 -1.154152 0.002022
1 2.176747 -2.250284 0.090633
8 3.404279 -0.657856 -0.110161
6 -2.571672 -1.524523 0.027838
1 -3.173773 -1.272871 0.902913
1 -3.154089 -1.328746 -0.874257
1 -2.276282 -2.571751 0.064183
rd_1m5fC/rd_1m5fC_4.xyz
6 1.607952 0.591283 0.006707

6 -0.642575 1.278992 -0.005781
6 -1.106540 -0.087258 -0.000414
6 -0.130095 -1.070888 -0.006008
1 -0.390020 -2.123370 -0.007035
7 1.171107 -0.764402 -0.010526
7 0.667473 1.578017 -0.003132
8 2.825449 0.818285 0.027588
7 -1.457076 2.368553 -0.015120
1 -2.441287 2.082791 -0.011820
6 -2.514036 -0.493399 0.007431
1 -3.268816 0.308828 0.006317
8 -2.881816 -1.670108 0.015355
6 2.204025 -1.810355 -0.015172
1 2.860977 -1.658140 -0.872880
1 2.790117 -1.739249 0.903102
1 1.717061 -2.781696 -0.080366
rd_1m5fU/rd_1m5fU1_a.xyz
6 1.589526 0.644504 0.040851
6 -0.652176 1.323293 -0.030175
6 -1.095001 -0.090201 -0.000358
6 -0.119452 -1.110193 -0.037195
1 -0.396829 -2.159268 -0.062147
7 1.153208 -0.790566 -0.037173
7 0.669743 1.608274 0.016795
8 2.808642 0.798643 0.117382
8 -1.526509 2.216687 -0.102349
6 2.201837 -1.813912 -0.075323
1 2.866880 -1.602956 -0.914254
1 2.774015 -1.762802 0.853323
1 1.735436 -2.790823 -0.189148
6 -2.523019 -0.447650 0.057110
1 -3.233437 0.387924 0.096679
8 -2.896760 -1.619215 0.063560
rd_1m5fU/rd_1m5fU2.xyz
6 1.496242 0.785992 0.127910
6 -0.827363 1.025762 -0.150309

6 -0.984903 -0.451418 -0.064522
6 0.175675 -1.251225 -0.116643
1 0.115567 -2.333048 -0.203638
7 1.363385 -0.692172 -0.056167
7 0.410333 1.550277 0.029658
8 2.647111 1.173256 0.337126
8 -1.825949 1.739688 -0.369911
6 2.598122 -1.476474 -0.145824
1 3.209372 -1.061138 -0.949545
1 3.143260 -1.387313 0.795705
1 2.346212 -2.515484 -0.350299
6 -2.279706 -1.143490 0.063323
1 -2.249722 -2.226050 -0.146287
8 -3.327302 -0.590268 0.377286
rd_1m5hmC/rd_1m5hmC_3.xyz
6 1.597270 0.711239 0.057350
6 -0.721171 0.980176 -0.095618
6 -0.935377 -0.439013 -0.178997
6 0.185457 -1.216120 -0.130509
1 0.129512 -2.297996 -0.185201
7 1.430054 -0.682202 -0.015102
7 0.502563 1.510174 0.018594
8 2.761206 1.166855 0.158390
6 -2.293547 -1.062884 -0.333618
1 -2.815460 -0.691019 -1.244866
1 -2.225143 -2.150473 -0.511582
7 -1.761134 1.836049 -0.149617
1 -1.586478 2.825998 -0.041718
1 -2.717207 1.514688 -0.111673
8 -3.197320 -0.820252 0.692414
6 2.624303 -1.535532 0.040007
1 3.282354 -1.311012 -0.801933
1 3.166200 -1.359773 0.971279
1 2.313145 -2.578577 -0.009545
rd_1m5hmC/rd_1m5hmC_1.xyz
6 1.639485 0.665573 0.101896

6 -0.655676 1.112183 -0.126075
6 -0.978195 -0.275725 -0.295780
6 0.084741 -1.155225 -0.244480
1 -0.052128 -2.225076 -0.358598
7 1.345305 -0.721828 -0.057103
7 0.613579 1.543950 0.062675
8 2.830823 0.994553 0.269940
6 -2.383421 -0.787395 -0.467539
1 -2.959372 -0.115333 -1.103962
1 -2.354647 -1.776487 -0.938250
7 -1.663473 2.008437 -0.168902
1 -1.257268 2.937393 -0.006260
8 -3.094482 -0.850522 0.783771
1 -2.656737 -1.505815 1.349017
6 2.471712 -1.662351 -0.008177
1 3.174496 -1.423660 -0.808919
1 2.982258 -1.563695 0.951652
1 2.092917 -2.675853 -0.130117
rd_1m5hmC/rd_1m5hmC_2.xyz
6 1.637772 0.701670 0.082873
6 -0.673899 1.065337 -0.131187
6 -0.944757 -0.341661 -0.260575
6 0.147293 -1.178588 -0.192930
1 0.048715 -2.255823 -0.270056
7 1.396109 -0.694188 -0.033959
7 0.578984 1.542973 0.034657
8 2.816201 1.086262 0.227299
6 -2.329484 -0.908441 -0.436905
1 -2.843283 -0.425606 -1.272836
1 -2.257377 -1.978423 -0.660166
7 -1.647766 2.005465 -0.162597
1 -2.558540 1.556452 -0.297637
8 -3.165492 -0.683050 0.712715
1 -2.777141 -1.152781 1.466973
6 2.556472 -1.592497 0.026265
1 3.238884 -1.353941 -0.792135

1 3.077823 -1.447942 0.974435
1 2.213562 -2.622315 -0.060648
rd_1m5hmU/rd_1m5hmU6.xyz
6 1.699461 0.561981 -0.008596
6 -0.494751 1.395801 0.007695
6 -1.030974 0.012270 0.003555
6 -0.123424 -1.055028 0.010819
1 -0.454834 -2.086345 0.016706
7 1.179389 -0.821175 0.011058
7 0.845787 1.588806 -0.003346
8 2.933009 0.655595 -0.028903
8 -1.303808 2.351874 0.019137
6 -2.496480 -0.194765 -0.007316
1 -2.921178 0.317303 0.871670
1 -2.908145 0.326001 -0.887410
8 -2.819979 -1.576502 -0.016806
1 -3.784818 -1.658781 -0.027658
6 2.153379 -1.918116 0.016276
1 2.752663 -1.863273 -0.894469
1 2.807486 -1.805492 0.882187
1 1.615547 -2.863419 0.062970
rd_1m5hmU/rd_1m5hmU8.xyz
6 1.698224 0.564500 -0.003927
6 -0.495060 1.398196 0.010432
6 -1.033869 0.015941 -0.004120
6 -0.127221 -1.051512 -0.000199
1 -0.460973 -2.082774 -0.004407
7 1.175682 -0.819481 0.006792
7 0.845610 1.591877 0.004363
8 2.931777 0.655494 -0.020389
8 -1.304945 2.353840 0.024384
6 -2.509610 -0.194755 -0.022381
1 -2.943244 0.313972 0.851960
1 -2.917618 0.318277 -0.902792
8 -2.912986 -1.552892 -0.095898
1 -2.785587 -1.970356 0.769774

6 2.148628 -1.917105 0.007289
1 2.746712 -1.859796 -0.904177
1 2.804184 -1.807076 0.872420
1 1.610166 -2.862144 0.051809
rd_1m5hmU/rd_1m5hmU14.xyz
6 1.579858 0.795086 0.022624
6 -0.755291 0.988018 -0.043146
6 -0.899039 -0.484412 -0.027598
6 0.274370 -1.254444 -0.027581
1 0.238338 -2.339650 -0.040206
7 1.460336 -0.674362 -0.001065
7 0.472648 1.546501 0.000698
8 2.737143 1.227726 0.061070
8 -1.799009 1.694672 -0.103319
6 -2.210863 -1.186130 -0.116089
1 -2.293600 -1.537868 -1.165723
1 -2.181649 -2.099306 0.493453
8 -3.353115 -0.435943 0.245237
1 -3.128369 0.508669 0.091679
6 2.701186 -1.458248 0.015441
1 3.292649 -1.205714 -0.866312
1 3.270280 -1.204465 0.911128
1 2.449985 -2.517505 0.012733
rd_1m5hmU/rd_1m5hmU11.xyz
6 1.606487 0.721953 0.096251
6 -0.689937 1.166493 -0.116765
6 -0.967996 -0.277224 -0.310770
6 0.109828 -1.173625 -0.246711
1 -0.037575 -2.243703 -0.357339
7 1.340463 -0.731174 -0.058555
7 0.588225 1.582147 0.064032
8 2.797482 1.015148 0.248594
8 -1.648518 1.972215 -0.136572
6 -2.370179 -0.780797 -0.484562
1 -2.946880 -0.079783 -1.088570
1 -2.369840 -1.763597 -0.965503

8 -3.007075 -0.835403 0.804700
1 -2.632062 -1.580181 1.300969
6 2.486565 -1.644762 0.007114
1 3.181049 -1.399965 -0.798510
1 2.991983 -1.509622 0.964818
1 2.128788 -2.667863 -0.095328
rd_1m5mC/rd_1m5mC_2.xyz
6 1.174103 -0.830248 0.000003
6 -1.175877 -0.797162 0.000000
6 -1.227817 0.644424 0.000000
6 -0.001141 1.292435 0.000000
1 0.074237 2.373941 -0.000002
7 1.152479 0.608002 0.000001
7 0.003536 -1.480782 0.000001
8 2.295760 -1.383485 0.000001
7 -2.338027 -1.456085 -0.000001
1 -2.131262 -2.461197 -0.000001
6 -2.508102 1.417560 -0.000002
1 -3.111368 1.172658 -0.880354
1 -3.111370 1.172658 0.880350
1 -2.310467 2.492397 -0.000001
6 2.442281 1.306815 -0.000001
1 3.008051 1.018049 0.888078
1 3.008051 1.018046 -0.888079
1 2.261447 2.380427 -0.000002
rd_1m5mC/rd_1m5mC_1.xyz
6 1.190895 -0.826294 0.000001
6 -1.159246 -0.825557 0.000001
6 -1.225695 0.617993 0.000000
6 -0.007897 1.277872 -0.000001
1 0.056079 2.360045 -0.000002
7 1.154896 0.606809 0.000000
7 0.026921 -1.491356 0.000001
8 2.317271 -1.371791 0.000001
7 -2.259669 -1.592752 -0.000001
1 -3.096228 -1.002462 -0.000002

6 -2.511145 1.388191 -0.000001
1 -3.114611 1.150582 -0.883728
1 -3.114611 1.150583 0.883725
1 -2.315574 2.463065 -0.000002
6 2.436034 1.321349 -0.000001
1 3.005613 1.039689 0.887984
1 3.005612 1.039688 -0.887985
1 2.242836 2.392913 -0.000002
rd_1m5mU/rd_1m5mU.xyz
6 1.147414 -0.857175 -0.000006
6 -1.200989 -0.844616 -0.000002
6 -1.213985 0.641659 0.000001
6 0.024310 1.303469 -0.000001
1 0.087594 2.386726 0.000001
7 1.156948 0.620037 -0.000005
7 -0.017869 -1.508361 -0.000005
8 2.266839 -1.385893 -0.000008
8 -2.296896 -1.451129 -0.000001
6 -2.490185 1.385534 0.000003
1 -3.087606 1.098887 -0.875634
1 -3.087607 1.098881 0.875636
1 -2.333300 2.465642 0.000006
6 2.459091 1.296053 -0.000008
1 3.016040 0.995152 0.888925
1 3.016040 0.995146 -0.888939
1 2.293260 2.371789 -0.000011
rd_1mC/rd_1mC_2.xyz
6 0.560352 -0.895392 0.000095
6 -1.615228 -0.025157 0.000009
6 -1.139116 1.317635 -0.000121
6 0.224117 1.505736 -0.000157
1 0.680489 2.488502 -0.000263
7 1.056279 0.445353 -0.000070
7 -0.778258 -1.088759 0.000103
8 1.393434 -1.823579 0.000221
7 -2.954469 -0.210718 0.000023

1 -3.132766 -1.222754 0.000101
1 -1.821559 2.156907 -0.000192
6 2.512410 0.623648 -0.000085
1 2.933733 0.148983 0.888288
1 2.933754 0.148669 -0.888279
1 2.738800 1.688375 -0.000263
rd_1mC/rd_1mC_1.xyz
6 0.594267 -0.890418 0.000079
6 -1.609875 -0.098379 0.000016
6 -1.176834 1.261307 -0.000114
6 0.177901 1.495254 -0.000142
1 0.600699 2.493156 -0.000239
7 1.045437 0.462638 -0.000062
7 -0.737719 -1.130767 0.000095
8 1.457017 -1.792049 0.000189
7 -2.925446 -0.425860 0.000048
1 -3.475945 0.440185 -0.000042
1 -1.883418 2.081934 -0.000187
6 2.494259 0.690984 -0.000072
1 2.932546 0.231984 0.888387
1 2.932595 0.231601 -0.888306
1 2.683192 1.762967 -0.000290
rd_1mU/rd_1mU.xyz
6 -0.529236 -0.924350 -0.000006
6 1.660119 -0.092564 -0.000008
6 1.149057 1.285162 -0.000004
6 -0.231388 1.506618 -0.000004
1 -0.658081 2.503912 -0.000002
7 -1.054759 0.470465 -0.000004
7 0.788530 -1.131921 -0.000001
8 -1.400450 -1.800078 0.000014
8 2.903546 -0.266150 0.000005
1 1.841125 2.118530 -0.000001
6 -2.509045 0.643216 0.000002
1 -2.738225 1.706983 0.000004
1 -2.921516 0.161045 -0.888219

1 -2.921510 0.161043 0.888224
rd_5caC/rd_5caC01.xyz
6 2.181652 -0.226956 0.004335
6 0.085243 0.997208 0.041436
6 -0.606365 -0.270516 -0.081671
6 0.204902 -1.426587 -0.232430
1 -0.293700 -2.380161 -0.375996
7 1.518334 -1.435535 -0.199677
7 1.434548 0.950758 0.107447
8 3.405017 -0.169091 0.084226
7 -0.557946 2.139636 0.088653
1 -0.068108 3.024327 0.193568
1 -1.572640 2.112992 -0.035580
6 -2.109016 -0.385836 0.010387
8 -2.792580 0.588654 -0.412373
8 -2.529450 -1.454471 0.525824
1 1.967508 1.814211 0.209285
rd_5caC/rd_5caC02.xyz
6 -2.172419 -0.038607 0.017765
6 -0.102935 1.016605 -0.093936
6 0.606259 -0.251254 0.062818
6 -0.179968 -1.408424 0.145261
1 0.252067 -2.399689 0.223404
7 -1.495128 -1.297241 0.141434
7 -1.435089 1.084796 -0.103617
8 -3.406932 -0.074648 0.029670
7 0.599679 2.138705 -0.224635
1 0.112903 3.021829 -0.326840
1 1.609812 2.144086 -0.151558
6 2.094101 -0.377139 0.060607
8 2.804170 0.253242 0.867900
8 2.471767 -1.149803 -0.873314
1 -2.083295 -2.127458 0.213583
rd_5caC/rd_5caC03.xyz
6 2.200832 -0.192504 0.005689
6 0.232634 1.034341 -0.002392

6 -0.537890 -0.210323 -0.013163
6 0.214121 -1.411661 -0.041854
1 -0.305551 -2.363423 -0.068209
7 1.526113 -1.442981 -0.040495
7 1.561175 1.009097 0.015901
8 3.447923 -0.246324 0.035918
7 -0.389858 2.213172 -0.007562
1 0.163686 3.061593 -0.000689
1 -1.402355 2.258419 -0.020827
6 -2.024414 -0.244227 0.004139
8 -2.729981 0.758553 -0.018093
8 -2.513111 -1.488924 0.049837
1 -3.488135 -1.451809 0.059015
rd_5caC/rd_5caC04.xyz
6 2.196857 -0.180652 -0.001436
6 0.216348 1.026029 0.063375
6 -0.542764 -0.224126 -0.033393
6 0.223145 -1.407069 -0.181593
1 -0.276473 -2.362358 -0.322853
7 1.535274 -1.426921 -0.169194
7 1.543764 1.010136 0.094451
8 3.443254 -0.225125 0.045715
7 -0.415286 2.197936 0.140814
1 0.135038 3.045994 0.206601
1 -1.420607 2.256793 0.035202
6 -2.033561 -0.254771 -0.035135
8 -2.698280 0.698923 -0.418780
8 -2.665150 -1.362926 0.374242
1 -2.082956 -2.051924 0.738230
rd_5caC/rd_5caC05.xyz
6 -2.051704 -0.108620 -0.015691
6 -0.077249 0.995847 0.097072
6 0.625360 -0.261610 -0.054998
6 -0.190618 -1.414648 -0.175718
1 0.275183 -2.389552 -0.280279
7 -1.505342 -1.362053 -0.169900

7 -1.426495 1.042112 0.127792
8 -3.376522 -0.052089 -0.005670
7 0.591534 2.133997 0.207640
1 0.085534 3.007732 0.317889
1 1.603800 2.158708 0.131960
6 2.115796 -0.383729 -0.038319
8 2.789126 0.281474 -0.861751
8 2.542316 -1.166119 0.857130
1 -3.751249 -0.944847 -0.120036
rd_5caC/rd_5caC06.xyz
6 2.193194 -0.156310 0.055823
6 0.193347 1.002009 -0.147097
6 -0.531372 -0.255831 -0.010408
6 0.248437 -1.436534 0.023347
1 -0.255589 -2.399112 0.038796
7 1.559350 -1.426461 0.043806
7 1.517526 1.022957 -0.082656
8 3.433965 -0.160345 0.184538
7 -0.469892 2.145361 -0.351365
1 0.053551 3.008833 -0.439371
1 -1.480392 2.192813 -0.379988
6 -2.015329 -0.397730 0.002095
8 -2.571304 -1.068270 -0.852032
8 -2.727287 0.208677 0.958053
1 -2.179100 0.630344 1.645035
rd_5caC/rd_5caC07.xyz
6 2.188480 -0.150631 0.000299
6 0.152460 0.914428 0.036193
6 -0.543963 -0.323015 -0.029823
6 0.293239 -1.470445 -0.136627
1 -0.170149 -2.447592 -0.228131
7 1.603205 -1.411413 -0.126759
7 1.443346 1.035813 0.051523
8 3.425001 -0.066204 0.064721
7 -0.630558 2.149651 0.068099
1 -0.542182 2.614742 0.981013

1 -1.639581 1.864934 -0.101822
6 -2.056085 -0.452849 0.005082
8 -2.718864 0.600149 -0.269812
8 -2.513233 -1.578661 0.305732
1 -0.308054 2.802376 -0.656969
rd_5caU/rd_5caU79.xyz
6 2.205315 -0.186399 0.000001
6 0.089231 1.039235 0.000001
6 -0.575853 -0.271091 -0.000003
6 0.229029 -1.435579 -0.000004
1 -0.260012 -2.404638 -0.000005
7 1.543564 -1.425021 -0.000001
7 1.456259 0.983921 0.000006
8 3.431769 -0.137612 0.000005
8 -0.528573 2.114987 0.000008
1 1.967576 1.865565 0.000011
6 -2.063585 -0.399819 -0.000004
8 -2.603515 -1.498035 -0.000021
8 -2.779340 0.725252 0.000007
1 -2.173873 1.511959 0.000021
rd_5caU/rd_5caU73.xyz
6 2.209236 -0.208904 -0.045578
6 0.112618 1.066978 0.125092
6 -0.575450 -0.227484 -0.005997
6 0.205382 -1.407681 -0.093093
1 -0.306810 -2.363371 -0.155766
7 1.518407 -1.429287 -0.124698
7 1.488754 0.965779 0.084961
8 3.437983 -0.188942 -0.086233
8 -0.450746 2.151794 0.273642
1 2.021036 1.830049 0.169071
6 -2.056656 -0.362089 0.006105
8 -2.596216 -1.324663 0.533608
8 -2.700028 0.629487 -0.607704
1 -3.663073 0.471550 -0.560816
rd_5caU/rd_5caU76.xyz

6 2.197970 -0.259965 -0.033598
6 0.149654 1.092852 0.120580
6 -0.584719 -0.176448 -0.010508
6 0.150484 -1.383572 -0.116000
1 -0.391722 -2.320466 -0.200427
7 1.463945 -1.454664 -0.123390
7 1.520717 0.940355 0.094033
8 3.426905 -0.284096 -0.065359
8 -0.375053 2.197610 0.260417
1 2.084920 1.783693 0.181963
6 -2.070258 -0.178395 -0.078640
8 -2.600073 -1.251181 0.519291
8 -2.710954 0.688958 -0.649630
1 -3.571210 -1.240234 0.415196
rd_5caU/rd_5caU78.xyz
6 2.172620 -0.118754 0.026257
6 0.009617 1.076258 -0.052762
6 -0.657230 -0.225270 0.075101
6 0.109855 -1.385710 0.098753
1 -0.334625 -2.374348 0.132229
7 1.441582 -1.320170 0.098603
7 1.400191 1.014734 -0.054769
8 3.393430 -0.120902 0.030695
8 -0.580323 2.149818 -0.145009
6 -2.146308 -0.302699 0.060922
8 -2.478146 -0.698475 -1.108251
8 -2.883718 -0.014636 1.008712
1 1.996476 -2.174621 0.143927
1 1.904467 1.897621 -0.121798
rd_5caU/rd_5caU72.xyz
6 2.193180 -0.088701 -0.000002
6 -0.011945 0.989628 -0.000001
6 -0.616914 -0.312987 -0.000004
6 0.176682 -1.425497 -0.000004
1 -0.225329 -2.432650 -0.000005
7 1.522099 -1.307463 0.000010

7 1.365835 1.020743 -0.000007
8 3.415902 -0.009193 0.000002
8 -0.682059 2.045909 0.000009
6 -2.079962 -0.354513 -0.000001
8 -2.786868 -1.375539 0.000002
8 -2.683352 0.814325 -0.000003
1 2.106211 -2.139996 -0.000012
1 1.828345 1.928089 -0.000024
rd_5caU/rd_5caU70.xyz
6 2.195205 -0.237075 0.032196
6 0.122531 1.071340 -0.143269
6 -0.583263 -0.208745 0.012424
6 0.170252 -1.397808 0.154855
1 -0.354901 -2.342948 0.265919
7 1.485445 -1.441526 0.159281
7 1.495330 0.948915 -0.119606
8 3.424799 -0.237428 0.052380
8 -0.436984 2.157418 -0.299184
1 2.043779 1.800275 -0.227983
6 -2.071307 -0.204890 0.115248
8 -2.777508 -0.967179 -0.723189
8 -2.625593 0.454740 0.977114
1 -2.232517 -1.406385 -1.401353
rd_5caU/rd_5caU49.xyz
6 2.079383 -0.112892 0.000003
6 0.107049 1.033060 -0.000009
6 -0.594790 -0.260514 -0.000003
6 0.201314 -1.429571 -0.000011
1 -0.269848 -2.406716 -0.000024
7 1.515137 -1.383509 -0.000008
7 1.475068 1.048823 -0.000003
8 3.402848 -0.179294 0.000016
8 -0.547476 2.102247 -0.000017
1 3.790142 0.715771 0.000025
6 -2.083603 -0.385509 0.000002
8 -2.626866 -1.483169 0.000000

8 -2.795010 0.741043 0.000021
1 -2.175817 1.519690 0.000016
rd_5caU/rd_5caU12.xyz
6 2.164523 -0.057398 0.027725
6 0.103658 0.992595 -0.060830
6 -0.613193 -0.256236 0.063962
6 0.166164 -1.415071 0.083056
1 -0.263297 -2.410259 0.108874
7 1.486888 -1.306064 0.084076
7 1.410964 1.082622 -0.056048
8 3.393474 -0.065899 0.049476
8 -0.649020 2.078990 -0.162624
6 -2.103435 -0.341101 0.070471
8 -2.463369 -0.774892 -1.074390
8 -2.819513 -0.025241 1.027963
1 2.070987 -2.141853 0.111386
1 -0.091529 2.875811 -0.246158
rd_5caU/rd_5caU38.xyz
6 2.170972 0.001875 0.034005
6 0.092570 1.007872 0.034787
6 -0.585856 -0.274896 -0.092485
6 0.229676 -1.415586 -0.215546
1 -0.178393 -2.412330 -0.341356
7 1.536378 -1.266596 -0.173537
7 1.399864 1.124568 0.104433
8 3.395866 0.001047 0.119745
8 -0.631378 2.107480 0.063573
6 -2.075998 -0.388843 0.001560
8 -2.501201 -1.487224 0.444220
8 -2.754175 0.635713 -0.303683
1 -1.590965 1.843960 -0.082387
1 2.154583 -2.076087 -0.257304
rd_5caU/rd_5caU67.xyz
6 -2.204507 -0.127547 -0.002797
6 -0.185309 0.999069 0.023241
6 0.515703 -0.270162 -0.013894

6 -0.296102 -1.433395 -0.051429
1 0.186057 -2.405319 -0.083693
7 -1.606550 -1.399446 -0.052546
7 -1.493244 1.056074 0.033208
8 -3.448622 -0.085300 0.007643
8 0.451181 2.168677 0.052157
1 3.630001 0.549615 -0.090453
6 1.991934 -0.453028 0.005835
8 2.522814 -1.542963 0.137792
8 2.665918 0.700184 -0.131942
1 1.421849 2.044892 0.018580
rd_5caU/rd_5caU09.xyz
6 -2.200882 0.004830 0.063921
6 -0.130784 1.082521 -0.187016
6 0.560953 -0.224039 -0.007250
6 -0.210319 -1.404129 0.018212
1 0.227769 -2.396781 0.023686
7 -1.514566 -1.284991 0.052767
7 -1.482802 1.120162 -0.077326
8 -3.428924 -0.070977 0.192391
8 0.553767 2.096481 -0.428434
6 2.040842 -0.353121 0.035836
8 2.599227 -1.180082 -0.670732
8 2.637993 0.487633 0.868958
1 -2.108834 -2.115951 0.076253
1 3.607292 0.365719 0.832300
rd_5caU/rd_5caU61.xyz
6 -2.195188 -0.148334 -0.000001
6 -0.185063 0.978033 0.000003
6 0.535837 -0.274155 0.000000
6 -0.270836 -1.441909 -0.000003
1 0.214260 -2.412428 -0.000004
7 -1.582412 -1.413257 -0.000003
7 -1.486408 1.043606 0.000002
8 -3.436636 -0.111257 -0.000001
8 0.551562 2.092289 0.000005

1 -0.004772 2.894081 0.000006
6 2.023309 -0.439515 0.000000
8 2.525928 -1.554741 -0.000002
8 2.801817 0.646686 -0.000001
1 2.282520 1.477365 0.000000
rd_5caU/rd_5caU55.xyz
6 -2.184485 -0.215676 -0.029947
6 -0.230753 1.008395 0.104163
6 0.543348 -0.205785 -0.009496
6 -0.198013 -1.411554 -0.100599
1 0.332968 -2.355326 -0.174796
7 -1.510505 -1.448365 -0.101524
7 -1.537319 1.001534 0.088583
8 -3.426608 -0.239114 -0.072802
8 0.436391 2.149985 0.246834
1 -0.193554 2.889611 0.336827
6 2.028799 -0.214579 -0.082700
8 2.552080 -1.300094 0.498825
8 2.678014 0.654524 -0.642056
1 3.522968 -1.293682 0.393619
rd_5caU/rd_5caU63.xyz
6 -2.192916 -0.089648 0.000000
6 -0.139099 0.991449 -0.000016
6 0.596826 -0.241378 0.000016
6 -0.161426 -1.394648 0.000048
1 0.276685 -2.386396 0.000076
7 -1.494444 -1.314816 0.000044
7 -1.451703 1.058786 -0.000030
8 -3.428648 -0.109485 -0.000010
8 0.567212 2.121696 -0.000052
6 2.040704 -0.336854 0.000008
8 2.703251 -1.425116 0.000051
8 2.854849 0.648043 -0.000052
1 -2.054556 -2.164071 0.000065
1 -0.036939 2.888051 -0.000077
rd_5caU/rd_5caU18.xyz

6 -2.051221 -0.021784 -0.000001
6 0.004524 0.969028 0.000001
6 0.629801 -0.323875 0.000001
6 -0.159405 -1.434437 0.000000
1 0.217181 -2.450242 -0.000003
7 -1.506771 -1.272467 0.000004
7 -1.354340 1.088922 0.000001
8 -3.374984 -0.027713 0.000000
8 0.734938 2.011790 0.000002
6 2.105093 -0.336656 -0.000001
8 2.804872 -1.363002 -0.000001
8 2.645339 0.856137 -0.000001
1 -2.125381 -2.080916 -0.000004
1 -3.718090 0.884627 -0.000021
rd_5caU/rd_5caU40.xyz
6 -2.083828 -0.129528 0.041573
6 -0.124559 1.047662 -0.161788
6 0.589567 -0.227496 -0.006426
6 -0.184036 -1.408495 0.070746
1 0.306389 -2.375703 0.120871
7 -1.499156 -1.387328 0.098600
7 -1.501244 1.030697 -0.105858
8 -3.408069 -0.208895 0.138311
8 0.492544 2.111720 -0.356294
1 -3.802952 0.681569 0.101101
6 2.071350 -0.339333 0.007604
8 2.643801 -1.224373 -0.612769
8 2.677234 0.582411 0.755128
1 3.644324 0.446791 0.723576
rd_5caU/rd_5caU44.xyz
6 2.201972 -0.044104 0.040170
6 0.169957 1.107659 -0.170647
6 -0.571404 -0.175492 -0.011452
6 0.158074 -1.382436 0.051037
1 -0.312987 -2.358717 0.096952
7 1.466079 -1.308538 0.062513

7 1.524486 1.095617 -0.094191
8 3.428391 -0.169645 0.141787
8 -0.480679 2.150360 -0.382480
6 -2.052459 -0.195146 0.133869
8 -2.629520 -1.109844 -0.645356
8 -2.629820 0.537311 0.918322
1 -3.595257 -1.119149 -0.493680
1 2.030484 -2.160021 0.102430
rd_5caU/rd_5caU56.xyz
6 2.205796 -0.023030 0.000007
6 0.124409 0.979178 -0.000002
6 -0.558749 -0.285020 0.000001
6 0.221170 -1.416241 0.000011
1 -0.181141 -2.422870 0.000017
7 1.551974 -1.276551 0.000016
7 1.443677 1.111698 -0.000002
8 3.444264 -0.011544 0.000014
8 -0.614811 2.060697 -0.000018
6 -2.000474 -0.214578 -0.000006
8 -2.667590 -1.364577 -0.000007
8 -2.569350 0.894694 -0.000012
1 -3.629629 -1.198908 -0.000013
1 2.148209 -2.100260 0.000024
rd_5caU/rd_5caU43.xyz
6 2.078551 -0.170305 -0.027667
6 0.157973 1.071910 0.148326
6 -0.597987 -0.179498 -0.007943
6 0.135311 -1.384473 -0.098698
1 -0.383296 -2.335116 -0.172114
7 1.452439 -1.408027 -0.099120
7 1.533409 1.008918 0.109806
8 3.400939 -0.291552 -0.103218
8 -0.424470 2.156696 0.333181
1 3.821370 0.586874 -0.057487
6 -2.080747 -0.172292 -0.109660
8 -2.642756 -1.164893 0.589494

8 -2.694436 0.636617 -0.786663
1 -3.611834 -1.145005 0.466305
rd_5caU/rd_5caU13.xyz
6 -2.049507 -0.225152 0.019416
6 0.002796 1.052000 -0.027360
6 0.678388 -0.233273 0.041409
6 -0.102538 -1.406819 0.043474
1 0.393015 -2.372842 0.051601
7 -1.429234 -1.420238 0.053274
7 -1.400129 0.950874 -0.028131
8 -3.357166 -0.272472 0.029269
8 0.544629 2.153756 -0.074977
1 -3.797349 0.598760 0.008373
6 2.168629 -0.290695 0.039171
8 2.591894 -0.484747 -1.141400
8 2.839037 -0.164570 1.078985
1 -1.923889 1.827530 -0.067653
rd_5dhmC/rd_5dhmC.xyz
6 2.250218 -0.214572 0.051685
6 0.302568 1.027539 -0.124279
6 -0.486342 -0.199079 -0.114206
6 0.243103 -1.400103 0.023488
1 -0.291492 -2.343068 0.052817
7 1.555180 -1.447628 0.118769
7 1.629331 0.995014 -0.061368
8 3.497965 -0.277819 0.111354
6 -1.988597 -0.177092 -0.293408
1 -2.226493 0.261697 -1.271131
7 -0.307930 2.214958 -0.225301
1 0.255332 3.056915 -0.219069
1 -1.311597 2.297080 -0.123883
8 -2.613112 0.682913 0.648728
1 -2.380024 0.390606 1.546578
8 -2.473790 -1.495684 -0.205580
1 -3.395996 -1.495080 -0.505705
rd_5dhmU/rd_5dhmU_52.xyz

6 -2.274293 -0.072467 -0.000002
6 -0.037098 0.926451 0.000011
6 0.486953 -0.442445 0.000004
6 -0.435527 -1.511514 0.000006
1 -0.054434 -2.529880 0.000005
7 -1.746502 -1.369685 0.000005
7 -1.409404 1.012636 0.000003
8 -3.492470 0.109826 -0.000009
8 0.687006 1.931013 -0.000001
6 1.982031 -0.670085 -0.000001
8 2.569593 -0.154822 1.177867
1 2.389631 0.799581 1.234969
8 2.569585 -0.154828 -1.177877
1 2.389629 0.799575 -1.234979
1 2.192639 -1.740031 0.000001
1 -1.828237 1.940945 -0.000002
rd_5dhmU/rd_5dhmU_53.xyz
6 2.214399 -0.354575 0.048253
6 0.282924 1.148073 -0.152535
6 -0.550282 -0.055180 -0.128138
6 0.086705 -1.312404 -0.002934
1 -0.526119 -2.208338 0.025681
7 1.389833 -1.486708 0.078848
7 1.637636 0.898834 -0.075508
8 3.438784 -0.468768 0.131065
8 -0.161861 2.297755 -0.237123
6 -2.050616 0.091548 -0.137058
8 -2.516405 0.180653 1.202201
1 -2.299572 -0.645216 1.669497
8 -2.593390 -1.014782 -0.819559
1 -3.528176 -0.829604 -1.001717
1 -2.339822 1.034581 -0.605187
1 2.265601 1.700062 -0.089847
rd_5dhmU/rd_5dhmU_50.xyz
6 -2.274326 -0.091633 0.017447
6 -0.051907 0.946467 0.019460

6 0.490843 -0.406871 -0.124123
6 -0.416876 -1.484798 -0.216136
1 -0.019975 -2.489460 -0.341950
7 -1.728274 -1.370602 -0.149698
7 -1.426035 1.002673 0.088771
8 -3.493661 0.064135 0.098204
8 0.646099 1.966988 0.073396
6 1.984609 -0.631420 -0.113350
8 2.655262 0.210215 -1.026603
1 2.441052 1.132791 -0.796972
8 2.402410 -0.419863 1.222871
1 3.236456 -0.896080 1.363644
1 2.205110 -1.653195 -0.432869
1 -1.857411 1.919185 0.191907
rd_5dhmU/rd_5dhmUa14.xyz
6 -2.242648 0.139063 0.005994
6 -0.051972 0.950720 0.000278
6 0.478904 -0.437153 -0.033181
6 -0.439062 -1.502680 -0.046743
1 -0.137576 -2.545105 -0.070035
7 -1.723739 -1.219293 -0.029424
7 -1.386776 1.166550 0.016520
8 -3.478183 0.218160 0.023550
8 0.781807 1.889258 0.013976
6 1.969895 -0.683936 -0.027177
1 -2.418813 -1.967325 -0.039953
8 2.576776 -0.079761 -1.148181
1 2.372864 0.873782 -1.125998
8 2.515074 -0.245179 1.200690
1 2.320453 0.703522 1.305727
1 2.182180 -1.751578 -0.094715
rd_5dhmU/rd_5dhmU_30.xyz
6 -2.245863 0.111883 0.047379
6 -0.072416 0.979711 0.016086
6 0.485720 -0.388413 -0.147116
6 -0.412248 -1.465222 -0.257589

1 -0.088779 -2.490189 -0.410207
7 -1.700840 -1.220254 -0.170220
7 -1.411163 1.152904 0.121608
8 -3.480102 0.154734 0.139902
8 0.728638 1.944690 0.063145
6 1.975387 -0.636400 -0.126133
8 2.658054 0.253317 -0.977427
1 2.368978 1.155047 -0.728665
8 2.361361 -0.496710 1.229675
1 3.160001 -1.027449 1.380883
1 2.195241 -1.643357 -0.490897
1 -2.378510 -1.980211 -0.248962
rd_5dhmU/rd_5dhmUa26.xyz
6 2.233616 -0.147789 0.038414
6 0.309484 1.175402 -0.157753
6 -0.543566 -0.040365 -0.133543
6 0.082382 -1.296012 -0.002527
1 -0.465444 -2.231010 0.033403
7 1.393564 -1.335861 0.071661
7 1.659987 1.051578 -0.077431
8 3.448929 -0.377119 0.125659
8 -0.247706 2.291383 -0.253779
6 -2.045751 0.074626 -0.149306
1 1.878843 -2.230324 0.156712
8 -2.565704 -1.069864 -0.781808
1 -3.493951 -0.900974 -1.007680
8 -2.493612 0.203336 1.192574
1 -2.294416 -0.618441 1.675529
1 -2.352129 0.993669 -0.650451
rd_5dhmU/rd_5dhmU_24.xyz
6 2.237550 -0.112115 0.044074
6 0.274921 1.140587 -0.200119
6 -0.536295 -0.102532 -0.161506
6 0.123260 -1.330302 0.031861
1 -0.395870 -2.279219 0.102501
7 1.434839 -1.323468 0.122115

7 1.628010 1.064856 -0.117348
8 3.459078 -0.299343 0.142839
8 -0.324881 2.233844 -0.309268
6 -2.036884 -0.010729 -0.214724
8 -2.558703 -1.262483 -0.561042
1 -3.497199 -1.154290 -0.781752
8 -2.458330 0.312738 1.114020
1 -2.454906 1.279754 1.211930
1 -2.352691 0.772603 -0.909041
1 1.948114 -2.196052 0.253088
rd_5dhmU/rd_5dhmU_6.xyz
6 -2.259162 -0.032576 -0.029811
6 -0.158185 0.924733 -0.025951
6 0.438911 -0.371799 0.212553
6 -0.464764 -1.455110 0.322704
1 -0.070028 -2.451812 0.505148
7 -1.767481 -1.327934 0.215597
7 -1.451685 1.083770 -0.141210
8 -3.492755 0.098501 -0.144750
8 0.595503 2.021113 -0.144206
6 1.927418 -0.637918 0.225875
8 2.364250 -0.992076 -1.071824
1 2.139694 -0.280274 -1.695997
8 2.574908 0.522349 0.713300
1 3.535758 0.381443 0.702412
1 2.153519 -1.503009 0.855094
1 1.534661 1.799717 0.060256
rd_5dhmU/rd_5dhmU_66.xyz
6 2.260839 -0.023220 -0.000003
6 0.136850 0.863856 0.000035
6 -0.441953 -0.457033 0.000018
6 0.487352 -1.522852 0.000048
1 0.118031 -2.545328 0.000074
7 1.791504 -1.348528 0.000050
7 1.421923 1.081155 0.000020
8 3.491853 0.156006 -0.000076

8 -0.732480 1.880254 0.000047
6 -1.935555 -0.703469 -0.000027
8 -2.536165 -0.205064 1.178805
1 -2.381304 0.752218 1.249654
8 -2.536089 -0.205047 -1.178892
1 -2.381258 0.752244 -1.249697
1 -2.125464 -1.777383 -0.000042
1 -0.266139 2.736964 0.000037
rd_5dhmU/rd_5dhmU_68.xyz
6 -2.262575 -0.148507 0.040188
6 -0.268635 1.010010 -0.042452
6 0.465151 -0.237830 -0.118627
6 -0.323539 -1.412192 -0.095810
1 0.169823 -2.377564 -0.147584
7 -1.635583 -1.405653 -0.012599
7 -1.574324 1.049045 0.031991
8 -3.507071 -0.133400 0.100216
8 0.368624 2.182218 -0.054345
6 1.963750 -0.323852 -0.330740
8 2.568258 0.799876 0.297454
1 3.446065 0.940531 -0.093463
8 2.515593 -1.534528 0.121495
1 2.347308 -1.624328 1.074837
1 2.156442 -0.298310 -1.411415
1 1.341558 2.016814 0.007969
rd_5dhmU/rd_5dhmU_59.xyz
6 -2.212497 -0.290190 0.058251
6 -0.342332 1.035632 -0.166829
6 0.509142 -0.126053 -0.147025
6 -0.154550 -1.366990 0.000855
1 0.428716 -2.281274 0.032111
7 -1.461590 -1.478956 0.103919
7 -1.643875 0.964829 -0.084562
8 -3.449703 -0.384338 0.155136
8 0.265145 2.218759 -0.283244
6 2.012065 0.006821 -0.190496

8 2.486690 0.379887 1.095293
1 2.225689 -0.303156 1.736756
8 2.550382 -1.220346 -0.624189
1 3.485774 -1.080778 -0.839452
1 2.306394 0.824191 -0.853224
1 -0.399406 2.932901 -0.284206
rd_5dhmU/rd_5dhmUa28.xyz
6 -2.258623 -0.043353 -0.047947
6 -0.226590 1.102836 0.108971
6 0.518692 -0.184677 0.119645
6 -0.220037 -1.379527 0.038462
1 0.242141 -2.360136 0.046801
7 -1.528732 -1.301950 -0.057484
7 -1.577812 1.103282 0.038473
8 -3.488731 -0.160699 -0.124982
8 0.435285 2.168886 0.164519
6 2.024004 -0.198218 0.312407
1 -2.092743 -2.150880 -0.125526
8 2.597059 -1.462543 0.125582
1 2.539334 -1.701195 -0.815476
8 2.661679 0.727095 -0.550053
1 2.185316 1.576977 -0.449945
1 2.204746 0.061635 1.367470
rd_5dhmU/rd_5dhmU_70.xyz
6 -2.261066 -0.161182 -0.043482
6 -0.274343 1.003141 0.073019
6 0.465019 -0.241619 0.108413
6 -0.313625 -1.419749 0.031978
1 0.183711 -2.383457 0.044520
7 -1.626252 -1.415166 -0.053812
7 -1.580152 1.039954 0.008896
8 -3.506285 -0.151110 -0.089087
8 0.359008 2.178573 0.115531
6 1.961514 -0.279120 0.327722
8 2.618598 0.818012 -0.307202
1 2.552933 0.703878 -1.272250

8 2.457189 -1.514250 -0.114079
1 3.330446 -1.651974 0.285554
1 2.153198 -0.129989 1.399119
1 1.331476 2.029405 0.030269
rd_5dhmU/rd_5dhmU_63.xyz
6 -2.262592 -0.036056 0.012618
6 -0.145678 0.873511 0.013863
6 0.442189 -0.441377 -0.055632
6 -0.477127 -1.513320 -0.117954
1 -0.097766 -2.529592 -0.190443
7 -1.782687 -1.353935 -0.085204
7 -1.434510 1.074542 0.047709
8 -3.494845 0.128709 0.066758
8 0.706072 1.902333 0.036819
6 1.934293 -0.681134 -0.054509
8 2.417019 -0.263836 1.206488
1 3.270731 -0.697321 1.365618
8 2.572930 -0.014214 -1.125400
1 2.437226 0.944048 -1.025691
1 2.132184 -1.743858 -0.217243
1 0.222089 2.748797 0.062593
rd_5dhmU/rd_5dhmU_15.xyz
6 -2.140805 -0.036903 0.013644
6 -0.070885 0.939833 0.024092
6 0.510004 -0.399269 -0.124968
6 -0.386076 -1.485132 -0.222547
1 -0.003693 -2.494243 -0.351676
7 -1.694748 -1.337614 -0.159445
7 -1.438861 1.063694 0.104319
8 -3.470255 0.017180 0.087859
8 0.666137 1.948842 0.074801
6 2.004882 -0.612917 -0.114799
8 2.671646 0.240274 -1.019736
1 2.418018 1.155872 -0.799765
8 2.417932 -0.407063 1.225147
1 3.253078 -0.881435 1.365388

1 2.236405 -1.631266 -0.438161
1 -3.754938 0.940977 0.213009
rd_5dhmU/rd_5dhmU_72.xyz
6 -2.262542 -0.039552 0.031423
6 -0.164522 0.922466 0.039949
6 0.435322 -0.371743 -0.199976
6 -0.463854 -1.455045 -0.330737
1 -0.066175 -2.448457 -0.524096
7 -1.767803 -1.330743 -0.229486
7 -1.458567 1.077974 0.156130
8 -3.496749 0.086436 0.146727
8 0.587497 2.019253 0.162195
6 1.924485 -0.621161 -0.207526
8 2.551860 0.525211 -0.758193
1 3.513789 0.393390 -0.767752
8 2.307649 -0.832258 1.137941
1 3.038719 -1.470219 1.158050
1 2.157456 -1.505945 -0.809225
1 1.525406 1.801699 -0.051644
rd_5dhmU/rd_5dhmUa29.xyz
6 2.240933 -0.126656 0.053757
6 0.294376 1.153795 -0.182697
6 -0.539239 -0.079365 -0.144795
6 0.113633 -1.320186 -0.023457
1 -0.406461 -2.271777 -0.006812
7 1.425100 -1.331695 0.067649
7 1.645293 1.060024 -0.075759
8 3.458639 -0.331200 0.165484
8 -0.283558 2.256093 -0.304097
6 -2.046420 0.042298 -0.180575
1 1.928419 -2.216256 0.150733
8 -2.681500 -1.088550 -0.732573
1 -2.692576 -1.818652 -0.090562
8 -2.515684 0.365025 1.113876
1 -2.213951 -0.306198 1.749650
1 -2.311047 0.884316 -0.821151

rd_5dhmU/rd_5dhmU_58.xyz
6 2.226506 -0.259287 0.068346
6 0.326671 1.019889 -0.172670
6 -0.498075 -0.160485 -0.129703
6 0.194241 -1.384671 0.013818
1 -0.367413 -2.311980 0.046233
7 1.504089 -1.464710 0.119358
7 1.629655 0.980888 -0.086163
8 3.465515 -0.324773 0.167966
8 -0.308699 2.184893 -0.318106
6 -1.999877 -0.056923 -0.195624
8 -2.432949 0.406135 1.074258
1 -3.264978 0.890537 0.955203
8 -2.525659 -1.329785 -0.501333
1 -3.438524 -1.215806 -0.807790
1 -2.285448 0.666370 -0.968006
1 0.337693 2.914734 -0.345289
rd_5dhmU/rd_5dhmU_17.xyz
6 -2.101863 -0.251541 0.034929
6 -0.292152 1.141979 -0.150579
6 0.564678 -0.046655 -0.121039
6 -0.069747 -1.302076 0.018754
1 0.521516 -2.210913 0.062877
7 -1.378836 -1.431585 0.096402
7 -1.658928 0.973506 -0.075249
8 -3.413824 -0.481920 0.108497
8 0.203102 2.284275 -0.243083
6 2.065589 0.091078 -0.144924
8 2.546165 0.190390 1.188838
1 2.324246 -0.627179 1.667997
8 2.593772 -1.028619 -0.819418
1 3.526750 -0.851791 -1.017495
1 2.359098 1.025260 -0.626955
1 -3.900014 0.361453 0.063991
rd_5dhmU/rd_5dhmUb14.xyz
6 2.140437 0.015642 0.019594

6 0.056482 0.951700 0.034232
6 -0.504535 -0.395059 -0.125852
6 0.407709 -1.467037 -0.219281
1 0.044115 -2.482897 -0.349480
7 1.713056 -1.293845 -0.149827
7 1.420127 1.102316 0.113622
8 3.462250 0.170266 0.092690
8 -0.699727 1.946908 0.095319
6 -1.996063 -0.631754 -0.127801
1 3.906063 -0.692780 0.008905
8 -2.418817 -0.452551 1.213030
1 -3.250992 -0.935729 1.340411
8 -2.670723 0.225257 -1.022982
1 -2.429562 1.140672 -0.787992
1 -2.209950 -1.648561 -0.468223
rd_5fC/rd_5fC.xyz
6 1.934364 0.056055 -0.000022
6 -0.243425 0.849888 0.000003
6 -0.739174 -0.527548 0.000006
6 0.245230 -1.549009 -0.000006
1 -0.074402 -2.588703 -0.000004
7 1.534676 -1.311103 -0.000016
7 1.060521 1.102316 -0.000010
8 3.165286 0.261546 -0.000029
7 -1.108237 1.864037 0.000011
1 -0.757170 2.814603 0.000006
1 -2.106477 1.687080 0.000018
6 -2.152028 -0.924242 0.000019
1 -2.336819 -2.009998 0.000030
8 -3.090743 -0.126620 0.000036
rd_5fU/rd_5fUd.xyz
6 -1.872233 -0.336430 -0.000002
6 0.083097 1.146854 -0.000002
6 0.899706 -0.074671 0.000000
6 0.240784 -1.335099 0.000000
1 0.847837 -2.236009 0.000001

7 -1.061644 -1.488740 -0.000001
7 -1.274996 0.913547 -0.000001
8 -3.096198 -0.442030 0.000000
8 0.552293 2.287277 0.000002
6 2.366541 0.024492 0.000001
1 2.792538 1.036651 0.000000
8 3.081579 -0.976331 0.000001
1 -1.892647 1.723502 0.000000
rd_5fU/rd_5fUda.xyz
6 -1.940313 -0.045267 0.017031
6 0.322948 0.922364 -0.028892
6 0.818775 -0.468496 -0.027593
6 -0.134393 -1.522418 -0.039106
1 0.227286 -2.547906 -0.061065
7 -1.437547 -1.359049 -0.017254
7 -1.055132 1.017303 0.003487
8 -3.153326 0.150436 0.055637
8 1.032577 1.926368 -0.058491
6 2.247873 -0.823674 -0.007930
1 2.454943 -1.903621 -0.087536
8 3.162555 -0.012362 0.096846
1 -1.457249 1.953147 0.011965
rd_5fU/rd_5fUfa.xyz
6 -1.923327 -0.077469 0.000003
6 0.257476 -0.823339 0.000000
6 0.721072 0.550178 0.000000
6 -0.279036 1.556177 0.000002
1 0.020163 2.601452 0.000003
7 -1.562932 1.286035 0.000004
7 -1.015996 -1.121186 0.000001
8 -3.139875 -0.337988 -0.000003
8 1.123593 -1.831947 0.000002
6 2.140317 0.908581 -0.000003
1 2.382798 1.980753 -0.000002
8 3.029667 0.051324 -0.000004
1 2.043439 -1.472033 0.000002

rd_5fU/rd_5fUe.xyz
6 1.866403 -0.292550 0.000000
6 -0.004971 1.053290 -0.000002
6 -0.856973 -0.113106 -0.000002
6 -0.189627 -1.369716 -0.000002
1 -0.784229 -2.278407 -0.000001
7 1.114919 -1.488663 0.000000
7 1.297522 0.971312 -0.000001
8 3.103054 -0.395152 0.000000
8 -0.611242 2.238493 0.000000
6 -2.327519 -0.026988 -0.000004
1 -2.769841 0.978176 0.000007
8 -3.030738 -1.035386 0.000008
1 0.054514 2.952464 0.000001
rd_5fU/rd_5fUa.xyz
6 -1.893856 -0.123555 0.000006
6 0.054614 1.172317 -0.000003
6 0.889260 -0.057017 0.000004
6 0.240786 -1.319438 0.000009
1 0.784449 -2.258750 0.000013
7 -1.063642 -1.336660 0.000007
7 -1.298466 1.068850 -0.000005
8 -3.108820 -0.349049 -0.000013
8 0.639855 2.277668 -0.000002
6 2.361256 0.005273 0.000005
1 2.810716 1.005622 -0.000012
8 3.043545 -1.017613 -0.000004
1 -1.569402 -2.225730 0.000008
rd_5fU/rd_5fUc.xyz
6 -1.757014 -0.236474 -0.000004
6 0.075542 1.135416 0.000001
6 0.916970 -0.067678 -0.000001
6 0.260387 -1.324755 -0.000002
1 0.843454 -2.240511 -0.000004
7 -1.048807 -1.434347 -0.000003
7 -1.293733 0.985643 -0.000001

8 -3.068022 -0.454760 0.000002
8 0.592712 2.270715 0.000001
6 2.386063 0.028155 0.000000
1 2.814893 1.039060 0.000007
8 3.099697 -0.973425 0.000005
1 -3.547359 0.394148 0.000006
rd_5fU/rd_5fUb.xyz
6 -1.764912 -0.174951 0.000006
6 0.099145 1.144590 0.000006
6 0.912900 -0.077395 0.000003
6 0.227829 -1.318445 0.000003
1 0.786838 -2.249101 0.000001
7 -1.084255 -1.390819 0.000002
7 -1.272552 1.034258 0.000003
8 -3.090111 -0.284020 -0.000008
8 0.647507 2.266236 -0.000003
6 2.384047 -0.013543 0.000000
1 2.833706 0.988280 0.000000
8 3.076347 -1.029813 -0.000004
1 -3.346893 -1.224018 -0.000013
rd_5fU/rd_5fUea.xyz
6 -1.924857 0.015951 0.007700
6 0.232686 0.845672 -0.013280
6 0.758831 -0.504404 -0.012865
6 -0.212636 -1.542521 -0.022795
1 0.124242 -2.576165 -0.035674
7 -1.506787 -1.328997 -0.016049
7 -1.051549 1.089182 -0.000657
8 -3.147962 0.233527 0.036211
8 1.103289 1.848992 -0.028667
6 2.182229 -0.883897 0.001993
1 2.365166 -1.970801 -0.026391
8 3.116594 -0.089959 0.047522
1 0.626057 2.700380 -0.026041
rd_5fU/rd_5fUca.xyz
6 -1.804163 -0.005884 0.025463

6 0.297490 0.919021 -0.063137
6 0.843950 -0.450367 -0.053316
6 -0.088782 -1.516569 -0.082965
1 0.264986 -2.543363 -0.130694
7 -1.391383 -1.330632 -0.051467
7 -1.072015 1.075235 0.006770
8 -3.128075 0.084522 0.116300
8 1.037924 1.915744 -0.135326
6 2.279385 -0.783750 -0.007276
1 2.507396 -1.849280 -0.180187
8 3.177214 0.022793 0.211292
1 -3.392385 1.021249 0.173021
rd_5fU/rd_5fUba.xyz
6 -1.802527 0.049601 -0.028216
6 0.315297 0.925076 0.056963
6 0.836405 -0.453882 0.052917
6 -0.115904 -1.501861 0.078480
1 0.214286 -2.536490 0.125239
7 -1.413699 -1.284048 0.042405
7 -1.048167 1.114259 -0.014340
8 -3.115792 0.246121 -0.109298
8 1.078528 1.905932 0.128631
6 2.265674 -0.814223 0.019574
1 2.472245 -1.880423 0.214771
8 3.179551 -0.029001 -0.209015
1 -3.585426 -0.607240 -0.097320
rd_5fU/rd_5fUf.xyz
6 -1.865695 -0.292678 0.002490
6 -0.001837 1.077320 -0.005919
6 0.854895 -0.089798 -0.002129
6 0.188624 -1.350957 -0.017217
1 0.791827 -2.253800 -0.025634
7 -1.112622 -1.483991 -0.018939
7 -1.305042 0.970920 0.000688
8 -3.103858 -0.399003 0.023283
8 0.461993 2.330530 -0.013415

6 2.328941 -0.053712 0.023463
1 2.827816 0.924970 0.067190
8 2.997421 -1.084134 0.003404
1 1.429996 2.400143 -0.024116
rd_5fU/rd_5fUg.xyz
6 -1.961340 -0.301215 0.003188
6 -0.009972 1.166067 -0.053779
6 0.852471 -0.035826 -0.027875
6 0.179121 -1.291776 -0.080566
1 0.762129 -2.210871 -0.057750
7 -1.114201 -1.423592 -0.140957
7 -1.338522 0.877372 -0.299226
8 -3.149407 -0.418481 0.284175
8 0.393769 2.325810 0.083082
6 2.218934 0.104420 0.054183
1 2.670215 1.093776 0.086774
8 3.019151 -0.937818 0.078811
1 3.953318 -0.665469 0.112814
rd_5fU/rd_5fUha.xyz
6 -2.023577 0.029640 0.014535
6 0.257216 0.923167 -0.074886
6 0.768192 -0.467284 -0.040007
6 -0.221503 -1.492550 -0.065996
1 0.093398 -2.535021 -0.030846
7 -1.504336 -1.275547 -0.113520
7 -1.106070 0.993590 -0.288052
8 -3.203491 0.237405 0.282638
8 0.958390 1.933639 0.027131
6 2.097083 -0.824889 0.045930
1 2.369932 -1.879637 0.051836
8 3.070931 0.050405 0.111431
1 3.938402 -0.391739 0.142957
rd_5hmC/rd_5hmC.xyz
6 2.017523 0.046394 0.043563
6 -0.156419 0.843873 -0.064852
6 -0.666605 -0.522192 -0.101812

6 0.311870 -1.537526 -0.000311
1 -0.011343 -2.576086 0.015106
7 1.603623 -1.308705 0.083024
7 1.147670 1.096305 -0.005182
8 3.251988 0.251664 0.069234
6 -2.105805 -0.881403 -0.333603
1 -2.321482 -0.765368 -1.404024
1 -2.263757 -1.935311 -0.077329
7 -1.016154 1.867528 -0.121135
1 -0.661198 2.815624 -0.091029
1 -2.012960 1.694350 -0.049640
8 -3.042056 -0.041534 0.349349
1 -2.958061 -0.195017 1.303396
rd_5hmU/rd_5hmU214.xyz
6 -2.011171 -0.026655 0.069079
6 0.258468 0.884459 -0.121939
6 0.732707 -0.502734 -0.142617
6 -0.234322 -1.531908 -0.069492
1 0.105447 -2.564510 -0.092195
7 -1.534542 -1.341811 0.033538
7 -1.107285 1.022156 0.000469
8 -3.219264 0.201151 0.162381
8 1.005901 1.866950 -0.214271
1 -1.487815 1.966349 0.019829
6 2.193963 -0.788149 -0.305437
1 2.386019 -1.847406 -0.120491
1 2.466686 -0.576152 -1.351919
8 3.021973 -0.048838 0.594073
1 2.915705 0.895125 0.391717
rd_5hmU/rd_5hmU114.xyz
6 1.981919 0.197334 -0.030529
6 -0.251026 0.890485 0.048537
6 -0.716265 -0.517513 0.022959
6 0.266495 -1.523782 0.047378
1 0.024061 -2.581831 0.071915
7 1.536763 -1.176773 0.027378

7 1.068702 1.176359 -0.006289
8 3.210343 0.351938 -0.096179
8 -1.118424 1.803009 0.127773
6 -2.145362 -0.921779 0.050122
1 -2.300596 -1.756046 -0.651017
1 -2.315951 -1.365782 1.054703
8 -3.101124 0.082659 -0.208576
1 -2.652179 0.947714 -0.066138
1 2.265480 -1.890454 0.027966
rd_5hmU/rd_5hmU156.xyz
6 -1.990108 0.003691 0.084727
6 0.144139 0.841090 -0.123637
6 0.689826 -0.491460 -0.205179
6 -0.262098 -1.533740 -0.116280
1 0.080848 -2.564271 -0.164390
7 -1.554592 -1.331627 0.025035
7 -1.130319 1.088185 0.012957
8 -3.211915 0.208518 0.207819
8 1.025761 1.840850 -0.217770
6 2.157685 -0.742408 -0.385104
1 2.343992 -1.817034 -0.433745
1 2.488422 -0.290355 -1.329561
8 2.924098 -0.238797 0.717624
1 2.972914 0.727187 0.646613
1 0.567995 2.700967 -0.173416
rd_5hmU/rd_5hmU134.xyz
6 1.878138 0.031387 -0.028165
6 -0.252377 0.862697 0.067806
6 -0.748403 -0.516896 0.046321
6 0.229070 -1.542928 0.040077
1 -0.083336 -2.583931 0.054934
7 1.523166 -1.303698 -0.000220
7 1.101673 1.085111 0.005245
8 3.201163 0.189026 -0.101116
8 -1.050955 1.826746 0.154425
6 -2.196031 -0.864235 0.135919

1 -2.386363 -1.793491 -0.413429
1 -2.388700 -1.096711 1.202626
8 -3.107965 0.116062 -0.331048
1 -2.733666 0.990398 -0.106704
1 3.417868 1.139019 -0.122429
rd_5hmU/rd_5hmU125.xyz
6 1.874747 0.087967 -0.028126
6 -0.269148 0.871720 0.055977
6 -0.740440 -0.517672 0.041954
6 0.255136 -1.525066 0.025494
1 -0.033910 -2.572855 0.032417
7 1.543150 -1.255102 -0.015087
7 1.078757 1.125625 -0.001043
8 3.183867 0.343022 -0.076565
8 -1.089613 1.818734 0.134512
6 -2.179846 -0.891529 0.152244
1 -2.358974 -1.831559 -0.382188
1 -2.354252 -1.113493 1.224528
8 -3.116295 0.065337 -0.314524
1 -2.753800 0.949228 -0.108161
1 3.681212 -0.494248 -0.086319
rd_5hmU/rd_5hmU246.xyz
6 -1.972592 -0.065429 0.105256
6 0.098254 0.924876 -0.145847
6 0.718979 -0.372170 -0.303374
6 -0.172932 -1.473527 -0.250903
1 0.231901 -2.475378 -0.373515
7 -1.468171 -1.365710 -0.061910
7 -1.186177 1.070191 0.049634
8 -3.195769 0.056045 0.309783
8 0.798865 2.067694 -0.192420
1 1.737383 1.939087 -0.403594
6 2.201502 -0.596411 -0.428570
1 2.383258 -1.616607 -0.770260
1 2.656293 0.089107 -1.153624
8 2.827228 -0.477684 0.860823

1 2.889733 0.459939 1.102053
rd_5mC/rd_5mC.xyz
6 1.644903 0.068838 -0.000134
6 -0.547106 0.815217 0.000127
6 -1.034526 -0.558243 0.000044
6 -0.026225 -1.551141 -0.000109
1 -0.325629 -2.596907 -0.000159
7 1.262766 -1.293668 -0.000193
7 0.751114 1.101598 0.000034
8 2.875321 0.303442 -0.000260
7 -1.430050 1.822423 0.000299
1 -1.094806 2.778019 0.000338
1 -2.427607 1.660796 0.000371
6 -2.479325 -0.899384 0.000104
1 -2.980610 -0.476222 0.881234
1 -2.980699 -0.476124 -0.880929
1 -2.626368 -1.981287 0.000051
rd_5mU/rd_5mU4.xyz
6 -1.643277 -0.047145 0.000168
6 0.622359 0.900502 -0.000476
6 1.122954 -0.479938 -0.001023
6 0.162025 -1.521152 -0.000798
1 0.518648 -2.548359 -0.001089
7 -1.145338 -1.352614 -0.000178
7 -0.753310 1.014530 -0.000221
8 -2.859562 0.164966 0.000805
8 1.353296 1.897879 -0.000215
1 -1.149781 1.952062 0.000091
6 2.581486 -0.725230 0.000640
1 3.050207 -0.239141 -0.865033
1 3.041088 -0.270096 0.888293
1 2.807215 -1.792849 -0.015263
rd_5mU/rd_5mU1.xyz
6 -1.613208 0.161733 0.000186
6 0.601886 0.927310 -0.000526
6 1.111396 -0.475064 -0.000971

6 0.156546 -1.511677 -0.000736
1 0.430950 -2.561448 -0.001004
7 -1.124146 -1.202722 -0.000257
7 -0.735900 1.168021 -0.000082
8 -2.850223 0.269944 0.000782
8 1.437510 1.858316 -0.000283
1 -1.832102 -1.937356 -0.000186
6 2.559222 -0.752391 0.000622
1 3.032681 -0.267733 -0.863835
1 3.023864 -0.293434 0.884238
1 2.771591 -1.822660 -0.012286
rd_5mU/rd_5mU5.xyz
6 1.635734 0.039349 -0.000001
6 -0.535546 0.807482 0.000000
6 -1.051654 -0.540648 0.000000
6 -0.055684 -1.550310 0.000000
1 -0.368596 -2.591557 0.000000
7 1.236284 -1.307142 -0.000001
7 0.738899 1.095214 -0.000001
8 2.857755 0.282225 0.000000
8 -1.448694 1.784135 0.000000
1 -1.003967 2.651879 0.000000
6 -2.503857 -0.830446 0.000001
1 -2.983829 -0.377287 0.877418
1 -2.983830 -0.377286 -0.877415
1 -2.692507 -1.905698 0.000001
rd_5mU/rd_5mU3.xyz
6 1.502953 -0.013556 0.000165
6 -0.595313 0.910788 -0.000996
6 -1.151959 -0.452268 -0.001482
6 -0.216133 -1.515917 -0.001695
1 -0.572751 -2.542563 -0.002581
7 1.089608 -1.333049 -0.000961
7 0.774893 1.072652 -0.000146
8 2.836057 0.084443 0.001687
8 -1.350655 1.904508 -0.000969

1 3.092535 1.024381 0.002540
6 -2.614885 -0.671233 0.001843
1 -3.063534 -0.212228 0.892912
1 -3.076722 -0.170965 -0.859034
1 -2.862225 -1.734332 -0.018841
rd_5mU/rd_5mU2.xyz
6 -1.500096 0.055623 0.000251
6 0.623639 0.906606 -0.000881
6 1.139098 -0.473180 -0.001896
6 0.171589 -1.507533 -0.001299
1 0.492792 -2.545739 -0.001876
7 -1.126826 -1.277756 -0.000184
7 -0.738734 1.116861 -0.000086
8 -2.819866 0.268312 0.001069
8 1.413060 1.874138 -0.000364
1 -3.288800 -0.584933 0.001033
6 2.594455 -0.736957 0.000986
1 3.072724 -0.243802 -0.854894
1 3.055449 -0.299189 0.896560
1 2.809099 -1.807032 -0.027545
rd_5mU/rd_5mU6.xyz
6 1.642326 0.051198 0.000868
6 -0.531401 0.821099 -0.001856
6 -1.040738 -0.532530 0.000466
6 -0.040308 -1.539837 -0.006815
1 -0.353233 -2.581107 -0.011801
7 1.250244 -1.296814 -0.008193
7 0.744778 1.103228 0.000302
8 2.864456 0.298205 0.008930
8 -1.357677 1.878804 -0.005131
1 -2.294809 1.622858 -0.014161
6 -2.488769 -0.853985 0.009726
1 -2.977306 -0.423513 0.895228
1 -2.989640 -0.421500 -0.867982
1 -2.651060 -1.933373 0.009231
rd_C/rd_C56.xyz

6 1.187270 -0.450952 -0.000004
6 -1.114179 -0.268014 -0.000001
6 -1.000718 1.174326 0.000002
6 0.297706 1.709797 -0.000001
1 0.436855 2.787487 0.000012
7 1.380656 0.956180 -0.000004
7 -0.040890 -1.052121 0.000001
8 2.227046 -1.146546 0.000003
7 -2.337276 -0.812899 0.000006
1 -2.444530 -1.820159 -0.000020
1 -3.166344 -0.232460 -0.000025
1 -1.880252 1.808436 0.000009
rd_C/rd_C46.xyz
6 1.231765 -0.343199 0.000002
6 -1.216923 -0.329361 -0.000002
6 -1.149884 1.096699 0.000003
6 0.117645 1.711504 0.000000
1 0.177128 2.796623 0.000005
7 1.265690 1.057431 -0.000002
7 0.011638 -0.976578 -0.000001
8 2.283503 -0.999866 -0.000006
7 -2.302022 -1.059536 0.000003
1 0.016588 -1.993292 -0.000001
1 -3.121453 -0.447258 0.000010
1 -2.063048 1.679786 0.000008
rd_C/rd_C45.xyz
6 1.216003 -0.366148 0.000002
6 -1.236430 -0.279349 0.000000
6 -1.120940 1.143152 -0.000002
6 0.163824 1.719409 -0.000002
1 0.256269 2.802091 -0.000004
7 1.292371 1.031302 0.000000
7 -0.025063 -0.965400 0.000002
8 2.245399 -1.056077 0.000002
7 -2.408680 -0.858007 -0.000001
1 -0.036913 -1.982450 0.000003

1 -2.310526 -1.879058 0.000000
1 -2.017154 1.750375 -0.000004
rd_C/rd_C15.xyz
6 1.142966 -0.483968 0.000009
6 -1.190488 -0.303111 -0.000008
6 -1.149425 1.123198 -0.000006
6 0.093082 1.709783 -0.000004
1 0.259982 2.780231 -0.000005
7 1.186723 0.922457 0.000002
7 -0.081576 -1.075219 0.000001
8 2.219791 -1.113276 0.000007
7 -2.415975 -0.889956 -0.000003
1 2.112116 1.346485 0.000004
1 -2.274637 -1.908196 0.000002
1 -2.056804 1.711299 -0.000009
rd_C/rd_C16.xyz
6 1.167848 -0.459752 0.000005
6 -1.169646 -0.361485 -0.000011
6 -1.173819 1.068378 -0.000007
6 0.046900 1.695636 -0.000003
1 0.177596 2.771109 -0.000001
7 1.167298 0.945191 0.000002
7 -0.036113 -1.093038 -0.000001
8 2.263378 -1.056588 0.000010
7 -2.327564 -1.078333 0.000000
1 2.078178 1.399239 0.000006
1 -3.117777 -0.423006 0.000001
1 -2.098060 1.631955 -0.000007
rd_C/rd_C35.xyz
6 -1.089251 -0.263366 -0.000024
6 1.197766 -0.357994 0.000002
6 1.231264 1.062287 0.000004
6 0.007398 1.733070 0.000005
1 -0.028885 2.818083 0.000009
7 -1.164735 1.094416 -0.000004
7 -0.010315 -1.016678 -0.000010

8 -2.300006 -0.859539 0.000014
7 2.349172 -1.036879 0.000002
1 -2.181718 -1.825221 0.000027
1 2.134345 -2.040957 -0.000003
1 2.174384 1.594412 0.000009
rd_C/rd_C25.xyz
6 -1.069825 -0.316797 0.000009
6 1.214105 -0.335982 -0.000001
6 1.206608 1.085102 -0.000008
6 -0.035486 1.718403 -0.000006
1 -0.109657 2.801396 -0.000012
7 -1.184839 1.038124 0.000001
7 0.031501 -1.036492 0.000006
8 -2.224482 -1.016858 0.000008
7 2.388883 -0.977042 -0.000007
1 -2.976297 -0.399566 0.000008
1 2.206505 -1.987457 -0.000004
1 2.134082 1.644005 -0.000015
rd_C/rd_C36.xyz
6 -1.105762 -0.248882 -0.000009
6 1.175939 -0.407783 0.000001
6 1.245624 1.013192 -0.000003
6 0.041268 1.717310 -0.000003
1 0.034760 2.803056 -0.000005
7 -1.147463 1.110092 -0.000003
7 -0.048301 -1.030866 -0.000001
8 -2.331715 -0.815368 0.000014
7 2.254879 -1.200781 -0.000001
1 -2.233883 -1.783387 0.000021
1 3.094935 -0.612764 -0.000005
1 2.201692 1.523906 -0.000005
rd_C/rd_C26.xyz
6 -1.087277 -0.302836 -0.000001
6 1.193450 -0.387268 -0.000009
6 1.223123 1.035531 -0.000008
6 -0.000364 1.703121 -0.000005

1 -0.043828 2.787924 -0.000002
7 -1.167866 1.054293 0.000000
7 -0.006794 -1.052248 0.000000
8 -2.260897 -0.972349 0.000017
7 2.298771 -1.146108 -0.000001
1 -2.994951 -0.334056 0.000023
1 3.119363 -0.531384 0.000001
1 2.164229 1.573464 -0.000008
rd_U/rd_U4.xyz
6 1.212803 -0.343197 0.000001
6 -1.239994 -0.346644 0.000007
6 -1.153915 1.106866 0.000005
6 0.113881 1.719520 0.000002
1 0.176310 2.804041 0.000001
7 1.257860 1.058811 -0.000001
1 -0.006577 -2.003869 -0.000004
7 -0.015218 -0.985845 0.000001
8 2.255871 -1.000175 -0.000011
8 -2.305105 -0.976471 -0.000001
1 -2.071002 1.682970 0.000004
rd_U/rd_U1.xyz
6 -1.116504 -0.510498 -0.000021
6 1.218234 -0.392831 0.000028
6 1.157322 1.079581 0.000028
6 -0.097193 1.712337 0.000001
1 -0.225843 2.789239 -0.000001
7 -1.168653 0.948723 -0.000023
1 -2.101514 1.365182 -0.000043
7 0.069942 -1.121131 0.000004
8 -2.228178 -1.058852 -0.000039
8 2.350106 -0.933494 0.000028
1 2.071756 1.659664 0.000049
rd_U/rd_U5.xyz
6 1.176829 -0.437200 -0.000002
6 -1.115879 -0.258142 0.000004
6 -1.017949 1.171960 0.000001

6 0.284035 1.709210 -0.000012
1 0.422609 2.786396 -0.000016
7 1.367300 0.956609 -0.000014
1 -2.312013 -1.740284 0.000018
7 -0.069873 -1.043514 0.000006
8 2.196246 -1.152806 -0.000002
8 -2.352541 -0.765593 0.000015
1 -1.904447 1.794441 0.000006
rd_U/rd_U3.xyz
6 -1.081881 -0.262272 0.000005
6 1.198091 -0.441389 -0.000009
6 1.263961 1.011894 -0.000001
6 0.053805 1.727995 -0.000003
1 0.054143 2.813732 0.000001
7 -1.122295 1.123048 -0.000002
1 -2.252236 -1.757998 -0.000002
7 -0.039425 -1.052723 0.000001
8 -2.310337 -0.785109 -0.000001
8 2.247621 -1.123818 0.000007
1 2.228003 1.506042 0.000003
rd_U/rd_U6.xyz
6 1.192276 -0.418228 -0.000004
6 -1.105599 -0.298172 0.000003
6 -1.036519 1.134918 0.000005
6 0.253129 1.703330 0.000004
1 0.363580 2.783783 0.000006
7 1.354030 0.978505 0.000000
1 -3.032405 -0.320146 0.000003
7 -0.037128 -1.053494 -0.000002
8 2.227984 -1.112547 -0.000007
8 -2.282711 -0.939594 0.000001
1 -1.931394 1.747320 0.000008
rd_U/rd_U2.xyz
6 -1.062154 -0.320216 -0.000002
6 1.218069 -0.415601 -0.000007
6 1.236682 1.039367 -0.000003

6 0.003757 1.714649 0.000003
1 -0.036483 2.799587 0.000008
7 -1.147636 1.064155 0.000006
1 -2.965449 -0.304086 0.000011
7 0.006165 -1.073179 -0.000004
8 -2.238535 -0.953070 0.000005
8 2.292354 -1.060325 -0.000002
1 2.183562 1.565627 -0.000004
p_5caC/p_5caC148.xyz
6 -2.238879 -0.204006 0.000000
6 -0.118540 1.023723 -0.000004
6 0.603296 -0.221091 -0.000002
6 -0.137106 -1.379159 0.000001
1 0.325363 -2.357984 0.000005
7 -1.483094 -1.366679 0.000000
7 -1.481855 0.956126 -0.000021
8 -3.460878 -0.198246 0.000012
7 0.456831 2.205530 0.000004
1 -0.088907 3.061749 0.000003
1 1.473586 2.246232 0.000012
6 2.075873 -0.251010 0.000001
8 2.778849 0.757672 0.000009
8 2.582549 -1.492658 -0.000006
1 -2.021158 1.821445 -0.000007
1 -2.000177 -2.243265 0.000009
1 3.556088 -1.437904 0.000003
p_5caC/p_5caC147.xyz
6 -2.247312 -0.159964 0.000004
6 -0.084659 1.001275 -0.000005
6 0.595623 -0.267188 -0.000006
6 -0.187041 -1.397837 -0.000017
1 0.253892 -2.387309 -0.000024
7 -1.530531 -1.346376 -0.000023
7 -1.450819 0.972447 0.000002
8 -3.468495 -0.112046 0.000027
7 0.504689 2.177654 -0.000012

1 -0.036431 3.036781 -0.000013
1 1.517547 2.231277 -0.000007
6 2.061534 -0.463159 0.000006
8 2.760998 0.686105 0.000003
8 2.594205 -1.564489 0.000019
1 -1.963131 1.853946 0.000016
1 -2.074620 -2.206434 -0.000028
1 3.716836 0.490343 0.000020
p_5caC/p_5caC149.xyz
6 -2.242107 -0.193706 0.004246
6 -0.108430 1.012671 0.009438
6 0.610994 -0.241232 -0.008756
6 -0.150578 -1.385517 -0.043350
1 0.274239 -2.381144 -0.079367
7 -1.496084 -1.361095 -0.042488
7 -1.472338 0.956156 0.030365
8 -3.463449 -0.177447 0.019464
7 0.466741 2.193309 0.008564
1 -0.080090 3.048899 0.022988
1 1.483935 2.230211 -0.017362
6 2.091811 -0.251448 -0.002950
8 2.750629 0.783497 -0.065888
8 2.765354 -1.411951 0.067601
1 -2.001975 1.827292 0.050637
1 -2.019083 -2.233888 -0.069631
1 2.224331 -2.207347 0.196466
p_5caC/p_5caC145.xyz
6 -2.236040 -0.167139 0.049989
6 -0.081631 0.989788 -0.120359
6 0.599123 -0.268039 -0.016095
6 -0.171592 -1.401627 0.057834
1 0.272809 -2.388578 0.110450
7 -1.517047 -1.350447 0.095992
7 -1.444460 0.966395 -0.064515
8 -3.456205 -0.118113 0.098426
7 0.513678 2.154904 -0.291251

1 1.516225 2.223660 -0.422577
1 2.312420 0.999331 1.290438
6 2.076417 -0.439889 -0.010278
8 2.822849 0.467334 0.654379
8 2.628673 -1.368295 -0.574978
1 -1.959811 1.842585 -0.144966
1 -2.058623 -2.208310 0.175698
1 -0.028409 3.009376 -0.379798
p_5caC/p_5caC183.xyz
6 2.105854 -0.135086 0.000002
6 0.130088 1.028669 -0.000003
6 -0.618332 -0.209656 -0.000004
6 0.108229 -1.372717 -0.000005
1 -0.341371 -2.356540 -0.000006
7 1.458870 -1.334975 -0.000004
7 1.491025 1.016603 0.000004
8 3.422198 -0.241933 0.000004
7 -0.455546 2.213622 -0.000006
1 0.113195 3.052055 -0.000001
1 -1.468643 2.274141 -0.000003
6 -2.094449 -0.240574 0.000001
8 -2.798187 0.766186 0.000004
8 -2.601227 -1.483536 0.000000
1 -3.574742 -1.429342 0.000031
1 2.001928 -2.196448 -0.000006
1 3.838572 0.639827 0.000011
p_5caC/p_5caC173.xyz
6 -2.112790 -0.096267 0.000001
6 -0.096770 1.006148 -0.000001
6 0.611038 -0.256589 0.000002
6 -0.157618 -1.392859 0.000002
1 0.269853 -2.387169 0.000003
7 -1.504875 -1.315925 0.000002
7 -1.459008 1.032869 0.000001
8 -3.432045 -0.161486 0.000000
7 0.504404 2.184705 -0.000005

1 -0.060802 3.025503 -0.000012
1 1.514142 2.255223 -0.000008
6 2.080956 -0.451266 0.000000
8 2.615611 -1.552144 -0.000009
8 2.779618 0.697238 0.000010
1 3.735142 0.499504 0.000005
1 -2.073851 -2.160486 -0.000003
1 -3.822506 0.732015 -0.000001
p_5caC/p_5caC182.xyz
6 2.112168 -0.100186 0.000008
6 0.117760 1.036118 0.000000
6 -0.618290 -0.210144 -0.000003
6 0.122144 -1.362216 -0.000001
1 -0.312789 -2.352670 -0.000003
7 1.474858 -1.308372 0.000005
7 1.476160 1.041191 0.000006
8 3.434133 -0.060833 0.000013
7 -0.483192 2.214198 -0.000003
1 0.075533 3.059263 -0.000001
1 -1.496767 2.262598 -0.000008
6 -2.094595 -0.259323 -0.000009
8 -2.810101 0.738766 -0.000011
8 -2.585159 -1.508545 -0.000007
1 -3.559319 -1.467594 -0.000012
1 2.010517 -2.175488 0.000006
1 3.861919 -0.935838 0.000019
p_5caC/p_5caC482.xyz
6 -2.105217 -0.268636 0.000004
6 -0.089914 1.010448 -0.000008
6 0.614293 -0.232394 -0.000021
6 -0.146969 -1.393091 -0.000024
1 0.350325 -2.355773 -0.000036
7 -1.494153 -1.435741 -0.000014
7 -1.456451 0.923604 0.000003
8 -3.417485 -0.154738 0.000023
7 0.466476 2.205337 -0.000026

1 -0.093733 3.051540 0.000068
1 1.481146 2.262087 0.000009
6 2.087365 -0.260246 0.000002
8 2.790163 0.749396 0.000011
8 2.599259 -1.500584 0.000016
1 3.572463 -1.440120 0.000063
1 -2.017790 1.776521 0.000013
1 -3.836355 -1.035744 0.000022
p_5caC/p_5caC172.xyz
6 -2.118516 -0.063644 0.000053
6 -0.084939 1.012116 0.000000
6 0.610799 -0.257671 -0.000013
6 -0.170626 -1.383105 0.000011
1 0.243320 -2.383220 0.000007
7 -1.519710 -1.291681 0.000046
7 -1.443798 1.054204 0.000035
8 -3.438319 0.021788 0.000072
7 0.529830 2.184529 -0.000020
1 -0.027144 3.030759 -0.000007
1 1.540057 2.244911 -0.000041
6 2.079055 -0.468464 -0.000051
8 2.601705 -1.574880 -0.000079
8 2.789180 0.672677 -0.000053
1 3.742694 0.465482 -0.000081
1 -2.081577 -2.141992 0.000061
1 -3.896762 -0.837374 0.000103
p_5caC/p_5caC472.xyz
6 -2.116021 -0.230419 0.000000
6 -0.056709 0.986903 0.000003
6 0.606386 -0.278213 0.000007
6 -0.196206 -1.411455 0.000015
1 0.280443 -2.385346 0.000020
7 -1.542499 -1.416591 0.000008
7 -1.427014 0.938105 0.000003
8 -3.423769 -0.073841 -0.000015
7 0.513869 2.176820 -0.000002

1 -0.040939 3.026548 -0.000004
1 1.524859 2.245489 0.000002
6 2.072668 -0.470814 -0.000002
8 2.771314 0.680789 0.000005
8 2.611017 -1.569848 -0.000018
1 3.727166 0.485263 -0.000008
1 -1.961329 1.808245 0.000001
1 -3.869903 -0.941356 -0.000001
p_5caC/p_5caC483.xyz
6 -2.106820 -0.302751 -0.000005
6 -0.098343 1.000083 -0.000005
6 0.619939 -0.231282 0.000003
6 -0.130394 -1.400420 0.000010
1 0.379000 -2.356864 0.000028
7 -1.474204 -1.460289 -0.000001
7 -1.466972 0.897214 -0.000006
8 -3.424758 -0.335634 0.000002
7 0.440324 2.203476 -0.000001
1 -0.131987 3.041702 -0.000040
1 1.454119 2.274900 -0.000009
6 2.092671 -0.241533 0.000004
8 2.783538 0.776460 0.000015
8 2.619719 -1.475632 -0.000018
1 3.592116 -1.402896 -0.000004
1 -2.019718 1.756837 0.000034
1 -3.857877 0.537375 0.000005
p_5caC/p_5caC473.xyz
6 2.118098 -0.264034 0.000000
6 0.065745 0.977406 0.000000
6 -0.612167 -0.276758 0.000003
6 0.179450 -1.418752 0.000005
1 -0.309014 -2.386807 0.000008
7 1.522759 -1.440710 0.000005
7 1.438978 0.913303 -0.000003
8 3.436176 -0.255749 -0.000002
7 -0.488970 2.175075 0.000002

1 0.075376 3.018622 -0.000015
1 -1.499177 2.255655 -0.000005
6 -2.080113 -0.452617 -0.000001
8 -2.765828 0.706918 0.000007
8 -2.630756 -1.545634 -0.000010
1 -3.723835 0.522438 -0.000001
1 1.964498 1.789603 -0.000004
1 3.839975 0.631053 -0.000005
p_5caC/p_5caC185.xyz
6 2.247925 -0.121163 0.000001
6 0.196205 0.935795 0.000000
6 -0.558745 -0.265320 -0.000001
6 0.207952 -1.419728 -0.000006
1 -0.220115 -2.414418 -0.000010
7 1.540147 -1.334937 -0.000003
7 1.491511 1.033346 0.000002
8 3.477769 -0.126461 0.000004
7 -0.512135 2.222925 -0.000001
1 -0.253254 2.772951 -0.829938
1 -1.539976 2.057251 0.000006
6 -2.032742 -0.310273 0.000000
8 -2.743982 0.695789 -0.000004
8 -2.525140 -1.551012 0.000009
1 -3.499968 -1.512251 -0.000001
1 2.097153 -2.188209 -0.000010
1 -0.253245 2.772957 0.829929
p_5caC/p_5caC175.xyz
6 2.252539 -0.076694 -0.000002
6 0.161074 0.911318 0.000007
6 -0.551416 -0.315265 0.000001
6 0.259284 -1.438018 -0.000002
1 -0.143470 -2.443829 -0.000006
7 1.588147 -1.312487 -0.000001
7 1.453889 1.049917 0.000004
8 3.482002 -0.035240 -0.000007
7 -0.558601 2.193679 0.000010

1 0.124228 2.961392 0.000068
1 -1.164110 2.283632 -0.827796
6 -2.016298 -0.519221 -0.000002
8 -2.539222 -1.624920 0.000019
8 -2.714088 0.627886 -0.000026
1 -3.669499 0.430203 -0.000018
1 2.172349 -2.147259 -0.000009
1 -1.164182 2.283577 0.827767
p_5caC/p_5caC487.xyz
6 2.248582 -0.291519 0.000003
6 0.172024 1.015396 -0.000002
6 -0.544438 -0.225812 -0.000011
6 0.240030 -1.415467 -0.000017
1 -0.283575 -2.366377 -0.000026
7 1.548797 -1.479664 -0.000011
7 1.525687 0.909300 0.000004
8 3.480697 -0.237914 0.000012
7 -0.359881 2.225863 -0.000005
1 0.232956 3.049772 0.000016
1 -1.363507 2.351868 0.000002
6 -1.954311 -0.327739 0.000000
8 -2.682903 0.757780 -0.000010
8 -2.489547 -1.518792 0.000023
1 -3.464715 -1.530535 0.000001
1 2.088637 1.758308 0.000018
1 -3.649306 0.610719 0.000036
p_5caC/p_5caC479.xyz
6 2.253908 -0.277908 0.008836
6 0.162582 1.003889 -0.009458
6 -0.546827 -0.247990 -0.015598
6 0.258337 -1.423570 -0.046931
1 -0.231239 -2.393353 -0.089010
7 1.566221 -1.472919 -0.039298
7 1.516850 0.912779 0.006107
8 3.483812 -0.212959 0.048398
7 -0.377219 2.209671 -0.017938

1 0.210016 3.037760 -0.014755
1 -1.381798 2.328072 -0.034666
6 -1.958681 -0.332327 0.008056
8 -2.663212 0.768920 0.001794
8 -2.673526 -1.425808 0.036748
1 -3.625119 0.598237 0.041990
1 2.068468 1.769381 0.019111
1 -2.173795 -2.260611 0.070286
p_5caC/p_5caC179.xyz
6 -2.259033 -0.091099 -0.006001
6 -0.202994 1.030074 0.038612
6 0.544832 -0.233051 0.011680
6 -0.228049 -1.391131 -0.034262
1 0.188495 -2.390361 -0.092153
7 -1.548810 -1.323526 -0.047172
7 -1.540923 1.047638 0.041693
8 -3.496282 -0.145937 -0.020066
7 0.400771 2.216880 0.072881
1 -0.181055 3.044962 0.104556
1 1.404970 2.319629 0.067256
6 1.967065 -0.336427 -0.006271
8 2.673443 -1.424724 0.118492
8 2.668108 0.745104 -0.184050
1 3.632454 0.582349 -0.147455
1 -2.101027 -2.176740 -0.101262
1 2.185818 -2.232524 0.359679
p_5caC/p_5caC142.xyz
6 2.105257 -0.150092 0.000009
6 -0.001758 0.994179 -0.000013
6 -0.681052 -0.275265 -0.000016
6 0.093200 -1.392172 -0.000029
1 -0.322574 -2.390948 -0.000040
7 1.466797 -1.323341 -0.000024
7 1.376869 0.977007 0.000036
8 3.393431 -0.007306 0.000021
7 -0.620490 2.144142 -0.000036

1 -0.124040 3.030531 -0.000003
1 -1.648292 2.081567 -0.000021
6 -2.204602 -0.389981 0.000008
8 -2.852228 0.701504 0.000008
8 -2.683728 -1.551115 0.000032
1 1.894705 1.859617 0.000051
1 2.016210 -2.184129 -0.000057
1 3.895684 -0.845964 -0.000009
p_5caC/p_5caC143.xyz
6 -2.104394 -0.166686 -0.000003
6 -0.003918 0.990440 -0.000002
6 0.682869 -0.272841 0.000000
6 -0.084980 -1.395632 0.000002
1 0.338328 -2.391210 0.000005
7 -1.454830 -1.333627 0.000002
7 -1.386859 0.967311 -0.000007
8 -3.399211 -0.203532 0.000003
7 0.606565 2.144728 0.000003
1 0.105540 3.028594 -0.000004
1 1.635062 2.087885 0.000003
6 2.206497 -0.377794 0.000000
8 2.847867 0.717601 0.000005
8 2.692940 -1.535989 -0.000006
1 -1.895595 1.855790 -0.000010
1 -2.009986 -2.189924 0.000008
1 -3.836694 0.670414 0.000004
p_5caC/p_5caC485.xyz
6 2.248914 -0.285089 0.000003
6 0.148405 0.905038 -0.000005
6 -0.556671 -0.275943 -0.000016
6 0.247211 -1.457536 -0.000020
1 -0.255056 -2.419780 -0.000031
7 1.557445 -1.478920 -0.000011
7 1.485423 0.903299 0.000003
8 3.475962 -0.203978 0.000015
7 -0.501674 2.202304 0.000000

1 -0.244054 2.752049 -0.833777
1 -1.536062 2.038934 -0.000014
6 -2.032888 -0.306412 -0.000001
8 -2.733029 0.708893 -0.000009
8 -2.534625 -1.539723 0.000025
1 -3.509576 -1.494417 0.000033
1 2.004188 1.782536 0.000019
1 -0.244086 2.752017 0.833806
p_5dhmC/p_5dhmC141.xyz
6 -2.317947 -0.042119 0.002874
6 -0.048393 0.876708 -0.001389
6 0.494305 -0.448909 -0.017445
6 -0.389324 -1.485829 -0.024768
1 -0.066313 -2.520111 -0.035909
7 -1.735492 -1.289231 -0.016439
7 -1.407532 1.004471 0.009026
8 -3.531032 0.143928 0.013106
6 1.980391 -0.698403 -0.009400
1 2.178539 -1.775025 -0.039707
7 0.688711 1.971505 0.004318
1 0.264102 2.892971 0.015316
1 1.699216 1.903257 -0.015152
8 2.507568 -0.143338 1.186912
1 3.421478 -0.452049 1.293076
8 2.536132 -0.070254 -1.155647
1 3.458892 -0.356734 -1.246409
1 -2.366957 -2.085415 -0.023686
1 -1.824304 1.934513 0.019933
p_5dhmC/p_5dhmC14a2.xyz
6 -2.289047 -0.227973 0.059594
6 -0.190291 1.017401 -0.132644
6 0.551071 -0.207424 -0.127875
6 -0.164623 -1.364206 -0.027293
1 0.308344 -2.336862 -0.027964
7 -1.521258 -1.370099 0.073244
7 -1.550989 0.940768 -0.051031

8 -3.514040 -0.227309 0.140177
6 2.059648 -0.192878 -0.290817
1 2.326305 0.202358 -1.277755
7 0.367174 2.212300 -0.220749
1 -0.193918 3.057998 -0.217886
1 1.376011 2.299378 -0.161162
8 2.529210 -1.511642 -0.138603
1 3.449885 -1.536906 -0.441768
8 2.680054 0.699858 0.623581
1 2.426832 0.449392 1.528577
1 -2.020226 -2.252298 0.148179
1 -2.100061 1.799375 -0.047497
p_5dhmC/p_5dhmC14a4.xyz
6 -2.294002 -0.220597 0.058832
6 -0.190177 1.020320 -0.111793
6 0.548610 -0.207126 -0.113632
6 -0.173167 -1.360927 -0.038719
1 0.295547 -2.336227 -0.061782
7 -1.530178 -1.365135 0.057829
7 -1.551341 0.946946 -0.032677
8 -3.519259 -0.216603 0.135280
6 2.057717 -0.213375 -0.300487
1 2.304249 0.136883 -1.309467
7 0.369831 2.213698 -0.196730
1 -0.188588 3.061129 -0.193052
1 1.379036 2.294514 -0.133342
8 2.616774 -1.502976 -0.211915
1 2.497786 -1.837157 0.693391
8 2.603424 0.667653 0.667701
1 3.522179 0.857996 0.419049
1 -2.031927 -2.247165 0.114226
1 -2.097862 1.807115 -0.021706
p_5dhmC/p_5dhmC14a1.xyz
6 -2.317355 -0.044649 0.011689
6 -0.049878 0.880717 0.001989
6 0.495341 -0.441850 -0.071558

6 -0.386606 -1.479854 -0.107172
1 -0.061036 -2.512148 -0.159674
7 -1.732768 -1.288045 -0.071476
7 -1.409159 1.003230 0.045057
8 -3.530505 0.137235 0.051301
6 1.981203 -0.694621 -0.050402
1 2.179613 -1.760189 -0.208026
7 0.684632 1.977130 0.031200
1 0.257733 2.896172 0.081164
1 1.692843 1.911261 -0.050298
8 2.470101 -0.295645 1.221448
1 3.372406 -0.637767 1.323774
8 2.573623 0.067848 -1.092049
1 3.508085 -0.183749 -1.163063
1 -2.362668 -2.084831 -0.104085
1 -1.827896 1.931084 0.093859
p_5dhmC/p_5dhmC14a3.xyz
6 -2.289336 -0.221345 0.059397
6 -0.185499 1.018032 -0.117077
6 0.553807 -0.209949 -0.120015
6 -0.169339 -1.363501 -0.043048
1 0.296764 -2.339996 -0.068019
7 -1.525978 -1.366322 0.059195
7 -1.546314 0.945808 -0.035056
8 -3.514370 -0.216775 0.137844
6 2.066603 -0.207779 -0.309524
1 2.308250 0.144025 -1.316154
7 0.376025 2.210757 -0.201447
1 -0.181454 3.058855 -0.197495
1 1.386283 2.290265 -0.147340
8 2.621235 -1.498655 -0.215573
1 2.474636 -1.851930 0.677943
8 2.696602 0.713873 0.565514
1 2.424984 0.528465 1.480444
1 -2.028344 -2.247970 0.116944
1 -2.092408 1.806306 -0.025849

p_5dhmC/p_5dhmC145.xyz
6 2.315750 -0.040045 0.011756
6 0.046936 0.881279 -0.005394
6 -0.496289 -0.441847 -0.089005
6 0.388103 -1.477885 -0.125192
1 0.064773 -2.510777 -0.183211
7 1.734212 -1.283874 -0.081138
7 1.405543 1.006359 0.045922
8 3.528491 0.144133 0.059495
6 -1.984943 -0.699846 -0.054081
1 -2.170719 -1.772736 -0.183325
7 -0.690479 1.975289 0.026330
1 -0.266502 2.895031 0.087162
1 -1.698024 1.908114 -0.059948
8 -2.589597 0.049138 -1.083199
1 -3.538082 -0.157709 -1.091154
8 -2.562569 -0.258886 1.174983
1 -2.352909 -0.906943 1.866534
1 2.366053 -2.079117 -0.113183
1 1.822534 1.934700 0.100594
p_5dhmC/p_5dhmC142.xyz
6 -2.316512 -0.060306 0.025011
6 -0.062170 0.902899 0.009344
6 0.502929 -0.404419 -0.152696
6 -0.369035 -1.450281 -0.234241
1 -0.029745 -2.471997 -0.360111
7 -1.716096 -1.286009 -0.153262
7 -1.422740 0.997506 0.094484
8 -3.530585 0.099012 0.111353
6 1.994284 -0.680901 -0.145336
1 2.176707 -1.667953 -0.579239
7 0.651890 2.012178 0.080571
1 0.209960 2.917822 0.195181
1 1.656072 1.959347 -0.062120
8 2.533966 -0.762136 1.163828
1 2.398064 0.078455 1.632899

8 2.634685 0.328642 -0.905259
1 3.583859 0.128545 -0.933514
1 -2.333841 -2.090081 -0.222445
1 -1.853960 1.914039 0.204922
p_5dhmC/p_5dhmC146.xyz
6 -2.311415 -0.037602 0.012554
6 -0.041873 0.882234 -0.000039
6 0.500764 -0.441254 -0.081738
6 -0.384835 -1.476338 -0.124392
1 -0.061522 -2.509292 -0.185200
7 -1.731030 -1.281761 -0.084168
7 -1.400486 1.008295 0.049980
8 -3.524105 0.147446 0.059510
6 1.994869 -0.704014 -0.049770
1 2.168275 -1.775088 -0.216531
7 0.696744 1.975402 0.029850
1 0.273862 2.895967 0.085831
1 1.703494 1.905627 -0.063540
8 2.567185 -0.290072 1.178380
1 2.225150 -0.859627 1.885156
8 2.699807 0.071568 -1.001347
1 2.426925 -0.201521 -1.891342
1 -2.363788 -2.076110 -0.120947
1 -1.817137 1.936808 0.104910
p_5dhmC/p_5dhmC147.xyz
6 2.313956 -0.057022 0.025234
6 0.057512 0.899936 0.020880
6 -0.505375 -0.408118 -0.138921
6 0.368833 -1.451328 -0.231066
1 0.031511 -2.473276 -0.361870
7 1.716132 -1.283760 -0.155856
7 1.417904 0.998631 0.101975
8 3.528053 0.105100 0.106982
6 -2.002254 -0.683960 -0.139697
1 -2.177715 -1.661532 -0.597711
7 -0.661424 2.006098 0.092138

1 -0.223723 2.914659 0.199793
1 -1.664751 1.945404 -0.056548
8 -2.731862 0.326550 -0.831698
1 -2.511038 0.287142 -1.776889
8 -2.541014 -0.773941 1.159666
1 -2.383336 0.051198 1.648660
1 2.336218 -2.085337 -0.232985
1 1.847115 1.916232 0.211582
p_5dhmC/p_5dhmC148.xyz
6 2.327346 -0.113879 0.001587
6 0.101090 0.931494 0.003018
6 -0.522353 -0.362597 -0.009056
6 0.324268 -1.437827 -0.020795
1 -0.046963 -2.455955 -0.034993
7 1.677208 -1.324849 -0.016574
7 1.468398 0.972160 0.011348
8 3.548169 0.011807 0.008500
6 -2.019869 -0.679607 -0.013429
1 -2.102534 -1.765639 -0.058217
7 -0.517441 2.101513 0.003613
1 0.007795 2.969807 0.017920
1 -1.525695 2.178561 0.010463
8 -2.696273 -0.226240 -1.172346
1 -2.819898 0.735005 -1.159670
8 -2.687032 -0.321331 1.181981
1 -2.684492 0.637688 1.327718
1 2.258208 -2.158976 -0.027827
1 1.934621 1.878337 0.022864
p_5dhmC/p_5dhmC13a4.xyz
6 -2.161128 -0.154114 0.045542
6 -0.204247 1.023026 -0.133720
6 0.562114 -0.196569 -0.117548
6 -0.140866 -1.357799 -0.011176
1 0.319462 -2.335441 -0.000949
7 -1.501739 -1.335555 0.081606
7 -1.561602 1.004653 -0.062765

8 -3.480468 -0.263458 0.130197
6 2.067379 -0.188125 -0.268223
1 2.342569 0.224959 -1.247902
7 0.370055 2.218091 -0.233699
1 -0.206640 3.050488 -0.228861
1 1.375093 2.315991 -0.164977
8 2.544436 -1.510945 -0.156296
1 3.436781 -1.545622 -0.532812
8 2.594720 0.650272 0.752978
1 3.499997 0.897476 0.506781
1 -2.028603 -2.201945 0.161050
1 -3.894667 0.618306 0.107399
p_5dhmC/p_5dhmC13a5.xyz
6 -2.156638 -0.159476 0.046823
6 -0.204194 1.023040 -0.141150
6 0.565823 -0.194309 -0.133447
6 -0.133296 -1.357853 -0.022132
1 0.329022 -2.334602 -0.013979
7 -1.493687 -1.339059 0.080345
7 -1.560973 1.001158 -0.064241
8 -3.475220 -0.272585 0.137617
6 2.074677 -0.171361 -0.284179
1 2.345525 0.260544 -1.254787
7 0.367482 2.220051 -0.233954
1 -0.211412 3.050952 -0.226868
1 1.372897 2.321789 -0.177192
8 2.551566 -1.493363 -0.177461
1 3.479991 -1.498894 -0.457008
8 2.686617 0.688004 0.667099
1 2.422161 0.407226 1.559585
1 -2.017749 -2.206734 0.164521
1 -3.892123 0.607985 0.117155
p_5dhmC/p_5dhmC13a1.xyz
6 -2.176929 -0.003599 0.006827
6 -0.064373 0.884415 0.000029
6 0.512710 -0.434802 -0.059378

6 -0.353480 -1.483129 -0.095306
1 -0.036853 -2.517630 -0.140767
7 -1.701259 -1.268701 -0.068793
7 -1.411613 1.058434 0.038755
8 -3.499742 0.081875 0.046994
6 2.000960 -0.679346 -0.038395
1 2.208421 -1.747141 -0.167463
7 0.683065 1.981674 0.022167
1 0.240047 2.891521 0.059587
1 1.690999 1.926010 -0.047223
8 2.492400 -0.243411 1.220427
1 3.392664 -0.587466 1.333324
8 2.583288 0.057697 -1.103491
1 3.514625 -0.202325 -1.182679
1 -2.352227 -2.049866 -0.097623
1 -3.769915 1.016512 0.103830
p_5dhmC/p_5dhmC13a2.xyz
6 -2.176536 -0.014908 0.019355
6 -0.075674 0.905526 0.007579
6 0.520331 -0.399090 -0.146762
6 -0.337233 -1.453133 -0.236609
1 -0.008446 -2.477598 -0.360640
7 -1.685907 -1.263687 -0.161698
7 -1.424098 1.053487 0.100827
8 -3.498664 0.046447 0.105580
6 2.013346 -0.669438 -0.130142
1 2.203921 -1.667253 -0.535426
7 0.651928 2.015833 0.067172
1 0.192590 2.911964 0.171891
1 1.654465 1.976086 -0.075965
8 2.546373 -0.712526 1.183976
1 2.401827 0.138632 1.630105
8 2.652745 0.320396 -0.915575
1 3.599650 0.111324 -0.951724
1 -2.326248 -2.051122 -0.230477
1 -3.780258 0.970263 0.235751

p_5dhmC/p_5dhmC13a6.xyz
6 -2.155896 -0.153739 0.048440
6 -0.199550 1.024225 -0.128032
6 0.568711 -0.196225 -0.129962
6 -0.137129 -1.357273 -0.042169
1 0.318236 -2.337958 -0.058557
7 -1.497302 -1.335926 0.063054
7 -1.556310 1.006275 -0.048248
8 -3.474497 -0.263778 0.140411
6 2.082475 -0.185467 -0.302299
1 2.332893 0.202645 -1.293606
7 0.376148 2.219146 -0.217031
1 -0.199242 3.052470 -0.207237
1 1.382845 2.313574 -0.162283
8 2.644159 -1.476951 -0.247372
1 2.494631 -1.858009 0.634181
8 2.700562 0.704298 0.613238
1 2.412835 0.488380 1.516241
1 -2.024272 -2.203249 0.131963
1 -3.889138 0.617997 0.128800
p_5dhmC/p_5dhmC133.xyz
6 -2.175001 0.000303 0.006626
6 -0.061504 0.885580 -0.008201
6 0.514117 -0.433656 -0.084865
6 -0.354303 -1.480329 -0.124396
1 -0.039374 -2.515059 -0.179784
7 -1.701937 -1.264486 -0.086523
7 -1.408005 1.061249 0.043524
8 -3.497478 0.087583 0.059836
6 2.004666 -0.683908 -0.046360
1 2.200542 -1.756689 -0.161717
7 0.688388 1.980786 0.018802
1 0.247967 2.891020 0.073397
1 1.695508 1.923959 -0.059644
8 2.574448 -0.225263 1.180447
1 2.377300 -0.874115 1.874855

8 2.606451 0.055356 -1.083813
1 3.553029 -0.159174 -1.098951
1 -2.354444 -2.044249 -0.117531
1 -3.764867 1.022124 0.130173
p_5dhmC/p_5dhmC138.xyz
6 2.171885 0.003543 0.003869
6 0.056282 0.883289 0.000362
6 -0.517620 -0.438731 -0.026194
6 0.353052 -1.484488 -0.046666
1 0.039553 -2.520981 -0.070571
7 1.700672 -1.264900 -0.035189
7 1.403039 1.063714 0.020616
8 3.495023 0.094728 0.023923
6 -2.014095 -0.691060 -0.014393
1 -2.194428 -1.772700 -0.067793
7 -0.695627 1.977158 0.007413
1 -0.257128 2.889873 0.023129
1 -1.704134 1.917082 -0.035181
8 -2.674671 -0.021635 -1.073523
1 -2.360086 -0.382659 -1.917440
8 -2.628641 -0.141579 1.137572
1 -2.328892 -0.637590 1.915352
1 2.354996 -2.043616 -0.053229
1 3.762810 1.031351 0.050214
p_5dhmC/p_5dhmC12a2.xyz
6 2.163513 -0.122299 0.044051
6 0.191379 1.031202 -0.136350
6 -0.565161 -0.195261 -0.128243
6 0.148850 -1.347624 -0.013244
1 -0.298027 -2.331582 -0.002276
7 1.511624 -1.310862 0.088649
7 1.545998 1.027768 -0.065113
8 3.489573 -0.091923 0.115541
6 -2.073154 -0.190267 -0.286647
1 -2.342457 0.240077 -1.258425
7 -0.397604 2.220689 -0.226151

1 0.170552 3.058920 -0.218109
1 -1.403815 2.308591 -0.160463
8 -2.700152 0.661050 0.661849
1 -2.437544 0.382594 1.555606
8 -2.535710 -1.517623 -0.184882
1 -3.461317 -1.533614 -0.473269
1 2.030391 -2.182705 0.173313
1 3.899843 -0.971987 0.184464
p_5dhmC/p_5dhmC12a1.xyz
6 2.167813 -0.117217 0.042890
6 0.191535 1.031132 -0.129510
6 -0.561576 -0.197372 -0.112783
6 0.156132 -1.347525 -0.003406
1 -0.288855 -2.332295 0.009320
7 1.519335 -1.307540 0.088535
7 1.546620 1.031023 -0.063520
8 3.494092 -0.083009 0.109462
6 -2.066076 -0.206637 -0.269833
1 -2.340194 0.205055 -1.250410
7 -0.399889 2.218665 -0.227203
1 0.166040 3.058369 -0.221569
1 -1.405636 2.302778 -0.150992
8 -2.607576 0.624336 0.749860
1 -3.513362 0.864262 0.498429
8 -2.529074 -1.534710 -0.162673
1 -3.415206 -1.580503 -0.552427
1 2.040730 -2.178244 0.168308
1 3.907515 -0.961678 0.177308
p_5dhmC/p_5dhmC12a3.xyz
6 2.167480 -0.115984 0.046247
6 0.191739 1.034325 -0.118376
6 -0.562902 -0.194496 -0.117292
6 0.156329 -1.344730 -0.027847
1 -0.285839 -2.331576 -0.039079
7 1.519286 -1.306896 0.070992
7 1.546505 1.033740 -0.046447

8 3.493302 -0.082437 0.119394
6 -2.071787 -0.208758 -0.295306
1 -2.324367 0.179631 -1.289149
7 -0.399625 2.222245 -0.206868
1 0.165828 3.062246 -0.196581
1 -1.406262 2.304540 -0.139172
8 -2.619472 0.627573 0.710491
1 -3.543655 0.812598 0.478938
8 -2.624435 -1.504779 -0.255799
1 -2.496654 -1.875235 0.634067
1 2.040471 -2.178866 0.137714
1 3.907010 -0.961961 0.172283
p_5fC/p_5fC_14a.xyz
6 -1.972758 0.054723 -0.000002
6 0.350532 0.817774 -0.000001
6 0.798881 -0.550066 -0.000001
6 -0.163802 -1.534534 -0.000001
1 0.095640 -2.587976 0.000001
7 -1.476134 -1.242420 -0.000001
7 -0.995431 1.038206 -0.000001
8 -3.167260 0.309529 0.000003
7 1.173141 1.841564 -0.000001
1 0.831580 2.797782 0.000001
1 2.174232 1.655117 0.000000
6 2.206454 -0.927196 0.000000
1 2.402248 -2.011521 0.000001
8 3.140172 -0.120366 0.000002
1 -2.168325 -1.988470 0.000001
1 -1.345544 1.996119 0.000001
p_5fC/p_5fC_14.xyz
6 1.908344 -0.312764 -0.000001
6 -0.109063 1.083542 0.000002
6 -0.925694 -0.099129 0.000002
6 -0.274553 -1.313493 0.000001
1 -0.828035 -2.245472 -0.000004
7 1.064120 -1.413565 0.000007

7 1.244951 0.903862 -0.000002
8 3.126499 -0.403116 -0.000003
7 -0.564212 2.321146 0.000007
1 0.073548 3.111250 -0.000014
1 -1.556751 2.520719 -0.000004
6 -2.394961 -0.079028 -0.000005
1 -2.889885 0.904235 -0.000016
8 -3.077308 -1.101542 0.000000
1 1.511977 -2.327820 -0.000008
1 1.857173 1.719486 -0.000003
p_5fC/p_5fC_13a.xyz
6 1.828288 0.075153 0.000000
6 -0.333579 0.829020 -0.000001
6 -0.824761 -0.531993 -0.000001
6 0.115410 -1.533447 -0.000001
1 -0.142096 -2.586492 -0.000001
7 1.431187 -1.231264 0.000000
7 1.002066 1.087066 0.000000
8 3.139167 0.229321 0.000001
7 -1.155266 1.863390 -0.000001
1 -0.780652 2.805229 0.000000
1 -2.158919 1.706851 -0.000001
6 -2.239232 -0.898212 -0.000001
1 -2.446429 -1.980930 0.000001
8 -3.164249 -0.083388 0.000002
1 2.135779 -1.966471 0.000000
1 3.370326 1.176872 0.000002
p_5fC/p_5fC_12a.xyz
6 1.829665 0.110834 0.000000
6 -0.345007 0.832590 0.000002
6 -0.822182 -0.533796 0.000000
6 0.129600 -1.521553 0.000002
1 -0.111403 -2.578564 0.000002
7 1.443232 -1.201920 0.000004
7 0.985147 1.108561 0.000000
8 3.119338 0.401188 -0.000004

7 -1.181376 1.855908 0.000002
1 -0.819600 2.802665 0.000002
1 -2.182648 1.685408 0.000000
6 -2.232283 -0.919246 -0.000003
1 -2.424558 -2.004853 -0.000006
8 -3.168260 -0.117567 -0.000002
1 2.142255 -1.942869 0.000004
1 3.699552 -0.381561 -0.000008
p_5fC/p_5fC_24a.xyz
6 -1.853791 -0.049411 0.000000
6 0.370995 0.803576 0.000000
6 0.813835 -0.555183 -0.000001
6 -0.161939 -1.544356 -0.000001
1 0.141261 -2.587285 -0.000001
7 -1.488678 -1.316994 -0.000001
7 -0.984854 0.994794 0.000000
8 -3.119793 0.311168 0.000001
7 1.170610 1.849496 -0.000002
1 0.807375 2.797049 0.000000
1 2.174397 1.685279 -0.000001
6 2.223222 -0.924698 0.000000
1 2.424808 -2.008165 0.000000
8 3.154093 -0.113316 0.000002
1 -3.688430 -0.482170 0.000001
1 -1.367301 1.941842 0.000000
p_5fC/p_5fC_13.xyz
6 -1.783039 -0.223612 0.000002
6 0.103250 1.083759 0.000004
6 0.940150 -0.093765 0.000004
6 0.296984 -1.307798 0.000008
1 0.832142 -2.249299 0.000010
7 -1.049591 -1.374508 0.000007
7 -1.253369 0.969878 -0.000002
8 -3.087373 -0.424402 -0.000009
7 0.580765 2.320227 0.000004
1 -0.067286 3.099349 -0.000010

1 1.571582 2.522268 -0.000008
6 2.413708 -0.073634 0.000001
1 2.907643 0.910228 0.000014
8 3.096871 -1.094896 -0.000013
1 -1.528077 -2.274048 0.000010
1 -3.562936 0.427005 -0.000020
p_5fC/p_5fC_34a.xyz
6 1.860210 -0.089424 0.000000
6 -0.361022 0.795867 0.000000
6 -0.824406 -0.553091 0.000000
6 0.139694 -1.554948 0.000000
1 -0.180019 -2.593048 0.000000
7 1.468433 -1.350344 0.000000
7 0.999813 0.965392 0.000000
8 3.158080 0.139818 0.000000
7 -1.140251 1.857503 0.000000
1 -0.758984 2.798032 0.000000
1 -2.147105 1.713423 0.000000
6 -2.239611 -0.898879 -0.000001
1 -2.460916 -1.978373 -0.000001
8 -3.155352 -0.070254 0.000000
1 3.404612 1.083053 -0.000003
1 1.375437 1.915390 0.000001
p_5fC/p_5fC_12.xyz
6 -1.791235 -0.186751 0.000009
6 0.120134 1.088457 -0.000004
6 0.941428 -0.100330 -0.000006
6 0.279239 -1.302125 -0.000005
1 0.798373 -2.252597 -0.000008
7 -1.070803 -1.349945 -0.000001
7 -1.235109 0.995023 0.000006
8 -3.111778 -0.234790 0.000018
7 0.614304 2.319001 -0.000010
1 -0.025389 3.104970 -0.000008
1 1.607159 2.509661 -0.000018
6 2.416029 -0.103898 -0.000007

1 2.925778 0.871899 -0.000006
8 3.082421 -1.135891 -0.000003
1 -1.544434 -2.252540 -0.000001
1 -3.478945 -1.136620 0.000032
p_5fC/p_5fC_46a.xyz
6 -2.011416 -0.049218 0.000003
6 0.273122 0.830541 0.000001
6 0.737555 -0.537001 0.000000
6 -0.270687 -1.557680 0.000001
1 0.065102 -2.592497 -0.000003
7 -1.559802 -1.358911 0.000000
7 -1.069887 0.994671 0.000002
8 -3.206061 0.239703 -0.000003
7 1.055884 1.889581 -0.000001
1 0.662207 2.825776 0.000000
1 2.064065 1.788549 -0.000002
6 2.060344 -0.933216 -0.000002
1 2.313061 -1.992147 0.000000
8 3.046024 -0.071811 0.000000
1 -1.459275 1.936726 0.000001
1 3.918268 -0.507480 0.000001
p_5fC/p_5fC_24.xyz
6 -1.771111 -0.358537 0.000000
6 0.146025 1.070277 -0.000001
6 0.939370 -0.115590 0.000001
6 0.260956 -1.330498 0.000002
1 0.841272 -2.247220 0.000000
7 -1.075527 -1.479413 0.000003
7 -1.211169 0.877191 -0.000005
8 -3.087202 -0.338113 0.000003
7 0.585817 2.317310 -0.000008
1 -0.061070 3.098929 0.000031
1 1.575609 2.527102 0.000022
6 2.408797 -0.100051 0.000004
1 2.908090 0.881514 0.000013
8 3.089880 -1.123994 -0.000004

1 -3.444922 -1.245742 -0.000020
1 -1.838469 1.683058 -0.000002
p_5fC/p_5fC_16a.xyz
6 1.981575 0.135422 -0.000003
6 -0.236639 0.864538 -0.000002
6 -0.738798 -0.514877 -0.000002
6 0.232990 -1.530753 -0.000002
1 -0.032713 -2.583275 -0.000001
7 1.510325 -1.219427 -0.000002
7 1.071895 1.130802 0.000000
8 3.205700 0.298952 0.000006
7 -1.076561 1.895309 -0.000003
1 -0.695524 2.834265 0.000001
1 -2.080237 1.773401 0.000001
6 -2.062979 -0.933557 -0.000001
1 -2.303611 -1.995893 0.000002
8 -3.053271 -0.088633 0.000004
1 2.219005 -1.950907 -0.000001
1 -3.922865 -0.531565 0.000008
p_5fC/p_5fC_46.xyz
6 -1.905592 -0.444073 -0.000002
6 -0.000032 1.096135 0.000004
6 0.862592 -0.061407 0.000003
6 0.213901 -1.344076 0.000015
1 0.847975 -2.226247 0.000019
7 -1.073270 -1.552825 0.000013
7 -1.328683 0.835533 -0.000004
8 -3.130931 -0.534918 -0.000013
7 0.394646 2.354498 0.000000
1 -0.283289 3.109613 0.000031
1 1.374336 2.609878 0.000020
6 2.242718 0.042037 -0.000003
1 2.759457 0.998808 -0.000003
8 2.995640 -1.027732 -0.000016
1 -1.988236 1.612614 -0.000013
1 3.941706 -0.795608 0.000008

p_5fC/p_5fC_15a_a.xyz
6 -1.971286 0.152908 0.000001
6 0.257609 0.737922 0.000000
6 0.731850 -0.600676 0.000006
6 -0.268267 -1.562387 -0.000004
1 -0.059689 -2.627260 -0.000005
7 -1.549103 -1.189030 -0.000015
7 -0.981118 1.119638 -0.000003
8 -3.170862 0.417818 0.000015
7 1.244671 1.827514 -0.000007
1 1.118907 2.421332 -0.830957
1 1.118933 2.421319 0.830957
6 2.134856 -1.002977 0.000009
1 2.317536 -2.088652 0.000016
8 3.079680 -0.207252 0.000003
1 -2.282810 -1.895870 -0.000026
1 2.206857 1.429011 -0.000022
p_5fC/p_5fC_34.xyz
6 -1.769993 -0.393811 0.000000
6 0.134384 1.063097 0.000001
6 0.943757 -0.108623 0.000002
6 0.280549 -1.332842 0.000003
1 0.873621 -2.241434 -0.000001
7 -1.050744 -1.500931 0.000007
7 -1.223412 0.851417 -0.000009
8 -3.080388 -0.525476 0.000000
7 0.556749 2.316254 0.000004
1 -0.098398 3.090783 0.000004
1 1.544308 2.537317 0.000011
6 2.412011 -0.071370 0.000001
1 2.896438 0.917588 0.000007
8 3.107953 -1.085454 -0.000006
1 -3.571521 0.316251 -0.000005
1 -1.847358 1.661046 -0.000018
p_5fC/p_5fC_16.xyz
6 1.934350 -0.245860 0.000000

6 0.035489 1.115688 0.000001
6 -0.858597 -0.048649 0.000001
6 -0.232908 -1.308976 0.000002
1 -0.797293 -2.234832 0.000000
7 1.077562 -1.396634 0.000001
7 1.364186 0.975329 0.000003
8 3.150448 -0.459883 -0.000004
7 -0.437926 2.359811 -0.000013
1 0.220216 3.129681 0.000033
1 -1.424198 2.579942 0.000051
6 -2.248996 0.026542 -0.000004
1 -2.782223 0.974417 -0.000014
8 -2.978202 -1.052524 0.000005
1 1.533317 -2.308204 0.000002
1 -3.930580 -0.843753 -0.000029
p_5fC/p_5fC_47.xyz
6 -1.913866 -0.430945 -0.000004
6 0.010608 1.084389 0.000006
6 0.868774 -0.082792 0.000001
6 0.196432 -1.356601 0.000021
1 0.793864 -2.264517 0.000041
7 -1.092694 -1.548027 0.000016
7 -1.320596 0.838862 0.000017
8 -3.139525 -0.508480 -0.000034
7 0.408538 2.341460 0.000003
1 -0.268733 3.097316 0.000015
1 1.387091 2.599564 0.000030
6 2.250678 0.048867 -0.000024
1 2.731006 1.022828 -0.000067
8 3.149571 -0.900425 -0.000010
1 -1.969699 1.624874 0.000038
1 2.803605 -1.812400 0.000036
p_5fC/p_5fC_36a.xyz
6 -1.866178 -0.023359 -0.000008
6 0.251938 0.835244 -0.000003
6 0.763484 -0.522466 -0.000002

6 -0.216492 -1.557555 -0.000006
1 0.113365 -2.593351 -0.000008
7 -1.511341 -1.341648 -0.000008
7 -1.083189 1.042084 -0.000004
8 -3.183037 0.160774 0.000015
7 1.034709 1.904237 -0.000007
1 0.611698 2.825678 -0.000008
1 2.043467 1.827526 -0.000007
6 2.095499 -0.904384 0.000004
1 2.364554 -1.959699 0.000003
8 3.065460 -0.030568 0.000010
1 -3.388046 1.113532 0.000028
1 3.944818 -0.452914 0.000012
p_5fC/p_5fC_17.xyz
6 -1.942153 -0.231080 -0.000005
6 -0.024068 1.102963 0.000004
6 0.863591 -0.073666 0.000011
6 0.212046 -1.323580 0.000003
1 0.737558 -2.272909 0.000004
7 -1.099060 -1.391505 -0.000020
7 -1.354211 0.980018 -0.000019
8 -3.160234 -0.429951 0.000019
7 0.454947 2.344693 0.000025
1 -0.203171 3.114634 -0.000085
1 1.440019 2.568384 -0.000094
6 2.256649 0.030826 0.000016
1 2.748435 0.998830 0.000020
8 3.140400 -0.924897 -0.000008
1 -1.565555 -2.297755 -0.000007
1 2.793264 -1.837616 -0.000001
p_5fC/p_5fC_15.xyz
6 1.933290 -0.220468 0.000001
6 -0.041869 0.976180 0.000001
6 -0.880626 -0.166277 0.000002
6 -0.189344 -1.370270 -0.000004
1 -0.698077 -2.327389 -0.000008

7 1.143453 -1.383673 -0.000013
7 1.257310 0.984274 0.000000
8 3.159322 -0.306599 0.000011
7 -0.639460 2.323572 -0.000003
1 0.105013 3.032491 0.000002
1 -1.230439 2.472875 -0.831253
6 -2.351182 -0.162800 0.000001
1 -2.853099 0.817146 -0.000010
8 -3.019802 -1.193618 0.000008
1 1.640147 -2.273657 -0.000009
1 -1.230454 2.472877 0.831235
p_5fC/p_5fC_26a.xyz
6 1.862500 0.035475 -0.000008
6 -0.269698 0.843960 0.000000
6 -0.754464 -0.524678 -0.000001
6 0.244812 -1.539849 0.000000
1 -0.061450 -2.582903 -0.000001
7 1.533915 -1.291381 -0.000003
7 1.059457 1.083892 -0.000003
8 3.162063 0.317019 0.000007
7 -1.077059 1.895125 -0.000001
1 -0.673970 2.825350 0.000000
1 -2.083896 1.796513 0.000001
6 -2.078490 -0.934123 0.000000
1 -2.326249 -1.994790 0.000001
8 -3.066295 -0.080871 0.000003
1 3.674651 -0.511486 0.000013
1 -3.937376 -0.520035 0.000005
p_5hmC/p_5hmC14_4.xyz
6 -2.047161 0.002683 -0.070283
6 0.243280 0.838652 0.118452
6 0.744170 -0.501442 0.183287
6 -0.173822 -1.505399 0.092711
1 0.110670 -2.550591 0.124918
7 -1.509144 -1.262155 -0.027885
7 -1.104400 1.018134 0.001351

8 -3.250351 0.230215 -0.170748
6 2.210011 -0.778988 0.398254
1 2.528660 -0.379561 1.366089
1 2.374426 -1.861459 0.404941
7 1.024861 1.902526 0.172261
1 0.644794 2.841914 0.125956
1 2.031494 1.784998 0.144577
8 3.049248 -0.132970 -0.571890
1 2.830953 -0.482279 -1.449767
1 -2.165325 -2.035913 -0.085689
1 -1.484949 1.962358 -0.044540
p_5hmC/p_5hmC14_1.xyz
6 -2.049063 -0.005126 -0.072994
6 0.236155 0.844322 0.119116
6 0.743974 -0.493024 0.186599
6 -0.167878 -1.502653 0.095845
1 0.122901 -2.545937 0.128469
7 -1.503852 -1.267081 -0.025952
7 -1.112353 1.015758 -0.001133
8 -3.253155 0.215009 -0.176294
6 2.205822 -0.758110 0.398342
1 2.534144 -0.297736 1.339533
1 2.371933 -1.838135 0.467709
7 1.010152 1.913485 0.175759
1 0.623126 2.850245 0.133546
1 2.018054 1.807413 0.161121
8 2.943989 -0.192780 -0.702026
1 3.885310 -0.214149 -0.472791
1 -2.155303 -2.044750 -0.084912
1 -1.498521 1.957621 -0.048280
p_5hmC/p_5hmC14_10.xyz
6 1.981376 -0.368230 0.006489
6 0.019382 1.094498 -0.001893
6 -0.848840 -0.040023 -0.029830
6 -0.255037 -1.266137 -0.046378
1 -0.829986 -2.182786 -0.072358

7 1.100452 -1.420020 -0.028248
7 1.366509 0.875085 0.016427
8 3.204301 -0.496365 0.028180
6 -2.353839 0.140956 -0.037501
1 -2.652754 0.763872 0.816549
1 -2.656952 0.662324 -0.951864
7 -0.414249 2.345231 0.005877
1 0.232328 3.126428 0.029448
1 -1.404605 2.555578 -0.007628
8 -3.057969 -1.094567 -0.025927
1 -2.995603 -1.484990 0.860010
1 1.506168 -2.351540 -0.037898
1 2.003513 1.670111 0.042003
p_5hmC/p_5hmC14_11.xyz
6 2.037177 -0.083639 0.096359
6 -0.190818 0.910155 -0.127346
6 -0.776002 -0.390672 -0.254593
6 0.079756 -1.453246 -0.193002
1 -0.269897 -2.475496 -0.279814
7 1.422335 -1.307710 -0.025425
7 1.162377 0.990654 0.040329
8 3.248042 0.062622 0.241537
6 -2.256881 -0.608912 -0.443233
1 -2.660505 0.073858 -1.199728
1 -2.409807 -1.628373 -0.802183
7 -0.878454 2.039463 -0.167034
1 -0.418518 2.940109 -0.086945
1 -1.883409 2.034286 -0.290200
8 -3.004205 -0.494564 0.781752
1 -3.240567 0.432726 0.930895
1 2.025055 -2.125253 0.009917
1 1.603747 1.904712 0.127542
p_5hmC/p_5hmC13_9.xyz
6 -1.904199 0.034186 0.054556
6 0.228635 0.844799 -0.118037
6 0.764539 -0.491166 -0.183105

6 -0.134999 -1.508762 -0.086609
1 0.143523 -2.554903 -0.110942
7 -1.471293 -1.246768 0.033357
7 -1.106203 1.071277 -0.010293
8 -3.220988 0.167717 0.158437
6 2.232523 -0.757718 -0.397141
1 2.411634 -1.838154 -0.397486
1 2.542550 -0.360163 -1.368764
7 1.021645 1.911101 -0.170025
1 0.621354 2.839156 -0.107882
1 2.027670 1.805622 -0.129841
8 3.067765 -0.097124 0.566957
1 2.853222 -0.441563 1.447544
1 -2.145856 -2.005366 0.097645
1 -3.456351 1.113310 0.177309
p_5hmC/p_5hmC13_14.xyz
6 -1.906296 0.027743 0.057208
6 0.221847 0.850109 -0.118675
6 0.764140 -0.483068 -0.185527
6 -0.129748 -1.505815 -0.091246
1 0.154703 -2.550164 -0.117382
7 -1.466838 -1.251157 0.029621
7 -1.113992 1.069003 -0.006976
8 -3.223261 0.153926 0.165050
6 2.228500 -0.737342 -0.396221
1 2.408999 -1.815697 -0.459858
1 2.547492 -0.277578 -1.341154
7 1.006886 1.921884 -0.175222
1 0.599460 2.847178 -0.118402
1 2.014349 1.828693 -0.150403
8 2.963140 -0.158503 0.699262
1 3.903607 -0.168310 0.466337
1 -2.137113 -2.013596 0.093705
1 -3.463587 1.098214 0.187454
p_5hmC/p_5hmC13_8.xyz
6 -1.896063 -0.034388 -0.078915

6 0.179895 0.912589 0.130601
6 0.794287 -0.384478 0.256765
6 -0.047259 -1.455600 0.201585
1 0.291267 -2.480778 0.287124
7 -1.391426 -1.284272 0.035293
7 -1.162274 1.049494 -0.037180
8 -3.212816 0.013725 -0.238410
6 2.278375 -0.594604 0.434137
1 2.681714 0.086770 1.192031
1 2.446254 -1.615626 0.782630
7 0.884910 2.040290 0.177774
1 0.409778 2.930962 0.099346
1 1.888297 2.043777 0.302259
8 3.010884 -0.462151 -0.798356
1 3.243666 0.467170 -0.938745
1 -2.017841 -2.084949 -0.002141
1 -3.501571 0.940378 -0.324636
p_5hmC/p_5hmC13_10.xyz
6 1.859616 -0.270696 0.005466
6 0.023590 1.095109 -0.002293
6 -0.861867 -0.038152 -0.029028
6 -0.272347 -1.264449 -0.050500
1 -0.826977 -2.192423 -0.077840
7 1.090754 -1.379557 -0.034086
7 1.372794 0.946346 0.019606
8 3.166827 -0.506521 0.030802
6 -2.364428 0.140016 -0.030015
1 -2.658573 0.758483 0.829305
1 -2.669089 0.671186 -0.938547
7 -0.441414 2.343702 0.001820
1 0.209586 3.118971 0.026200
1 -1.431128 2.550275 -0.012274
8 -3.075515 -1.093019 -0.025836
1 -3.004338 -1.494539 0.854420
1 1.532284 -2.295970 -0.047206
1 3.655409 0.335931 0.053049

p_5hmC/p_5hmC12_4.xyz
6 -1.905890 0.072844 -0.055307
6 0.239835 0.852423 0.113973
6 0.762234 -0.489440 0.183655
6 -0.148993 -1.494388 0.087449
1 0.112981 -2.544792 0.113160
7 -1.482953 -1.214166 -0.033604
7 -1.090477 1.097070 0.006875
8 -3.206530 0.332002 -0.152354
6 2.225711 -0.776840 0.401742
1 2.539829 -0.379253 1.371973
1 2.387707 -1.860028 0.408479
7 1.048632 1.907582 0.162524
1 0.662272 2.841370 0.099492
1 2.053088 1.787211 0.125375
8 3.072769 -0.135668 -0.564720
1 2.857374 -0.485896 -1.442813
1 -2.152661 -1.977505 -0.098904
1 -3.754294 -0.472771 -0.179801
p_5hmC/p_5hmC12_14.xyz
6 -1.908479 0.066806 0.057268
6 0.233231 0.857239 -0.114092
6 0.761593 -0.482131 -0.184240
6 -0.144432 -1.491860 -0.089838
1 0.123133 -2.540681 -0.116250
7 -1.479249 -1.218359 0.031083
7 -1.098221 1.094900 -0.004701
8 -3.209920 0.319343 0.157427
6 2.221244 -0.758411 -0.399777
1 2.384376 -1.839579 -0.463672
1 2.543489 -0.304608 -1.346446
7 1.034991 1.917399 -0.166604
1 0.642265 2.848889 -0.109108
1 2.040881 1.807897 -0.141784
8 2.969165 -0.191692 0.692613
1 3.909183 -0.227340 0.460341

1 -2.144995 -1.985212 0.096265
1 -3.753883 -0.488007 0.185964
p_5hmC/p_5hmC12_10.xyz
6 1.868313 -0.232526 0.006378
6 0.006534 1.101195 -0.005320
6 -0.863782 -0.044228 -0.033203
6 -0.254478 -1.259306 -0.065336
1 -0.793129 -2.196526 -0.097555
7 1.112938 -1.354329 -0.052488
7 1.354746 0.973407 0.023328
8 3.195222 -0.319186 0.051418
6 -2.368791 0.111674 -0.024947
1 -2.664974 0.729282 0.834356
1 -2.686698 0.636122 -0.932879
7 -0.474984 2.343970 -0.004596
1 0.167747 3.126142 0.020591
1 -1.466815 2.538973 -0.022799
8 -3.061977 -1.131121 -0.011508
1 -2.977674 -1.528457 0.869425
1 1.552698 -2.271974 -0.070517
1 3.527206 -1.233299 0.050962
p_5hmC/p_5hmC24_12.xyz
6 1.925674 -0.093853 -0.059048
6 -0.264825 0.823771 0.120717
6 -0.757154 -0.508865 0.182929
6 0.175250 -1.517567 0.093455
1 -0.154178 -2.551328 0.125201
7 1.519943 -1.337049 -0.021281
7 1.088311 0.977441 -0.003163
8 3.205887 0.242522 -0.164818
6 -2.223935 -0.785550 0.389080
1 -2.392412 -1.867571 0.378531
1 -2.544674 -0.400177 1.362249
7 -1.028771 1.906247 0.183031
1 -0.631625 2.837383 0.129802
1 -2.035902 1.804522 0.145054

8 -3.060510 -0.123260 -0.573879
1 -2.839260 -0.459547 -1.456001
1 3.752775 -0.563289 -0.199685
1 1.495813 1.911826 -0.048485
p_5hmC/p_5hmC12_11.xyz
6 1.901042 0.002512 0.079372
6 -0.192121 0.917303 -0.122478
6 -0.792307 -0.386462 -0.253012
6 0.062516 -1.444946 -0.193470
1 -0.260415 -2.475279 -0.277917
7 1.406119 -1.255896 -0.024570
7 1.146315 1.072155 0.039607
8 3.208569 0.189826 0.228890
6 -2.272225 -0.615207 -0.443132
1 -2.676437 0.063987 -1.202648
1 -2.424393 -1.637128 -0.796235
7 -0.913125 2.035801 -0.162078
1 -0.449867 2.932419 -0.080993
1 -1.916885 2.025342 -0.281955
8 -3.017596 -0.495442 0.782863
1 -3.266713 0.429882 0.921891
1 2.028595 -2.060467 0.012840
1 3.721742 -0.637446 0.236608
p_5hmC/p_5hmC241.xyz
6 -1.927719 -0.099162 -0.060525
6 0.258697 0.828002 0.120047
6 0.756131 -0.502729 0.182386
6 -0.171741 -1.515725 0.093909
1 0.162250 -2.547840 0.126741
7 -1.516648 -1.340734 -0.020884
7 -1.095155 0.975645 -0.004064
8 -3.208981 0.231326 -0.168069
6 2.218699 -0.769001 0.387550
1 2.545362 -0.329755 1.339661
1 2.388597 -1.849990 0.431097
7 1.016426 1.914537 0.185161

1 0.613643 2.843607 0.138340
1 2.024596 1.822238 0.156869
8 2.957675 -0.176566 -0.698654
1 3.898610 -0.202147 -0.468672
1 -3.752531 -0.576820 -0.202545
1 -1.506842 1.908195 -0.049388
p_5hmC/p_5hmC244.xyz
6 -1.905198 -0.180366 0.075009
6 0.207945 0.907859 -0.129275
6 0.795375 -0.379580 -0.265472
6 -0.064344 -1.456657 -0.211139
1 0.338130 -2.459777 -0.314360
7 -1.411289 -1.387328 -0.041049
7 -1.149660 0.949491 0.037355
8 -3.199915 0.057784 0.239648
6 2.278998 -0.576134 -0.451797
1 2.449518 -1.571680 -0.865130
1 2.686197 0.153434 -1.162166
7 0.866744 2.060362 -0.162015
1 0.386986 2.947371 -0.059992
1 1.874422 2.075251 -0.252824
8 3.014429 -0.536780 0.786569
1 3.049226 0.373933 1.116864
1 -3.694010 -0.782384 0.247034
1 -1.623793 1.847414 0.136844
p_5hmC/p_5hmC24_5.xyz
6 -1.844408 -0.402827 0.001632
6 0.016962 1.084371 -0.003837
6 0.861690 -0.056296 -0.032449
6 0.239624 -1.283359 -0.054661
1 0.839999 -2.184959 -0.084860
7 -1.109038 -1.482201 -0.037079
7 -1.330779 0.856704 0.013547
8 -3.173189 -0.428077 0.035145
6 2.366544 0.115254 -0.032607
1 2.677087 0.639247 -0.943367

1 2.664038 0.736629 0.823792
7 0.445641 2.342136 0.005540
1 -0.203742 3.119819 0.032174
1 1.434950 2.553445 -0.007017
8 3.071890 -1.120468 -0.020889
1 2.980602 -1.527511 0.854928
1 -3.485401 -1.350713 0.027118
1 -1.980387 1.643071 0.040667
p_5hmC/p_5hmC24_8.xyz
6 -1.911437 -0.168188 -0.075034
6 0.213803 0.896857 0.126748
6 0.788627 -0.397421 0.252473
6 -0.082109 -1.465000 0.191947
1 0.310524 -2.473010 0.284635
7 -1.429298 -1.380762 0.028641
7 -1.143991 0.953646 -0.035145
8 -3.205102 0.083876 -0.228314
6 2.268964 -0.612637 0.444170
1 2.670782 0.072334 1.200023
1 2.425023 -1.631909 0.802831
7 0.885738 2.041488 0.159966
1 0.413326 2.935011 0.082929
1 1.890406 2.046351 0.279729
8 3.018788 -0.497419 -0.780701
1 3.242509 0.432082 -0.935554
1 -3.706380 -0.752008 -0.236722
1 -1.609904 1.857236 -0.121804
p_5hmC/p_5hmC246.xyz
6 -1.847923 -0.400819 -0.000001
6 0.017929 1.080748 0.000002
6 0.858607 -0.063117 0.000001
6 0.234205 -1.290024 0.000014
1 0.830078 -2.194167 0.000029
7 -1.115511 -1.482618 0.000008
7 -1.330119 0.857036 0.000006
8 -3.177227 -0.420414 -0.000022

6 2.353917 0.111389 -0.000009
1 2.655016 0.687225 -0.887479
1 2.655033 0.687241 0.887444
7 0.450277 2.337221 0.000003
1 -0.197223 3.116908 0.000011
1 1.440242 2.545942 0.000003
8 2.979328 -1.170265 -0.000003
1 3.937607 -1.032564 -0.000023
1 -3.493452 -1.341677 0.000026
1 -1.977049 1.645989 0.000028
p_5hmC/p_5hmC34_9.xyz
6 -1.931250 -0.129357 0.058281
6 0.257091 0.813191 -0.126247
6 0.765583 -0.510593 -0.183261
6 -0.158086 -1.528721 -0.094004
1 0.184136 -2.558555 -0.123229
7 -1.502896 -1.365913 0.020258
7 -1.100091 0.950388 -0.003150
8 -3.240299 0.073933 0.171180
6 2.237021 -0.768287 -0.380648
1 2.422237 -1.847272 -0.355266
1 2.556060 -0.390389 -1.357317
7 1.004583 1.907560 -0.192625
1 0.593950 2.832832 -0.138710
1 2.012815 1.820126 -0.147568
8 3.059278 -0.080485 0.576894
1 2.838761 -0.407529 1.462679
1 -3.493774 1.012905 0.219950
1 -1.499339 1.888660 0.038765
p_5hmC/p_5hmC3410.xyz
6 1.932532 -0.135277 0.060422
6 -0.250901 0.818507 -0.127162
6 -0.765134 -0.503061 -0.185840
6 0.153443 -1.526044 -0.098848
1 -0.193811 -2.553942 -0.131465
7 1.498334 -1.369813 0.017146

7 1.106862 0.948561 -0.001743
8 3.241942 0.060582 0.178062
6 -2.232668 -0.749500 -0.380023
1 -2.560126 -0.310100 -1.331756
1 -2.419231 -1.827967 -0.416093
7 -0.990573 1.917844 -0.196896
1 -0.573298 2.840432 -0.148157
1 -1.999979 1.842114 -0.166334
8 -2.953889 -0.139731 0.708832
1 -3.896154 -0.143528 0.483074
1 3.500999 0.997691 0.232968
1 1.511186 1.884591 0.041775
p_5mC/p_5mC14.xyz
6 1.692414 0.050860 -0.000120
6 -0.638195 0.785650 0.000132
6 -1.096080 -0.570242 0.000047
6 -0.128946 -1.531839 -0.000113
1 -0.366753 -2.588767 -0.000190
7 1.204108 -1.231224 -0.000190
7 0.705517 1.025031 0.000052
8 2.890929 0.329817 -0.000250
7 -1.462235 1.820310 0.000281
1 -1.117615 2.774330 0.000323
1 -2.464353 1.677686 0.000343
1 1.892266 -1.978449 -0.000327
6 -2.564409 -0.885008 0.000113
1 -3.058716 -0.469419 0.885897
1 -3.058822 -0.469279 -0.885547
1 -2.718039 -1.965953 0.000037
1 1.044170 1.985970 0.000106
p_5mC/p_5mC13.xyz
6 1.545439 0.065416 -0.000096
6 -0.618738 0.803443 0.000123
6 -1.124056 -0.545470 0.000022
6 -0.183553 -1.531125 -0.000127
1 -0.424921 -2.586390 -0.000214

7 1.151677 -1.224920 -0.000181
7 0.711601 1.076605 0.000057
8 2.863730 0.240629 -0.000172
7 -1.446324 1.846993 0.000270
1 -1.072055 2.787679 0.000317
1 -2.449885 1.726487 0.000317
1 1.851562 -1.962443 -0.000302
6 -2.596652 -0.834213 0.000081
1 -3.081691 -0.405199 0.884845
1 -3.081806 -0.404986 -0.884516
1 -2.775976 -1.911590 -0.000037
1 3.071611 1.192360 -0.000077
p_5mC/p_5mC12.xyz
6 1.544757 0.108600 -0.000058
6 -0.636358 0.799554 0.000085
6 -1.118095 -0.558597 0.000009
6 -0.159232 -1.524346 -0.000178
1 -0.376685 -2.584845 -0.000277
7 1.171528 -1.191134 -0.000248
7 0.686074 1.099590 0.000076
8 2.837969 0.428008 -0.000043
7 -1.485269 1.826522 0.000198
1 -1.129048 2.774181 0.000239
1 -2.486199 1.685884 0.000219
1 1.872782 -1.927881 -0.000395
6 -2.584525 -0.875591 0.000128
1 -3.077295 -0.455896 0.885002
1 -3.077480 -0.455756 -0.884576
1 -2.743255 -1.956149 0.000061
1 3.427806 -0.346166 -0.000019
p_5mC/p_5mC24.xyz
6 1.572272 -0.055427 -0.000137
6 -0.656753 0.775901 0.000128
6 -1.112364 -0.570598 0.000042
6 -0.135411 -1.541515 -0.000112
1 -0.424883 -2.587387 -0.000179

7 1.208504 -1.309800 -0.000200
7 0.694545 0.983680 0.000023
8 2.847379 0.325207 -0.000214
7 -1.461322 1.832543 0.000296
1 -1.096952 2.778290 0.000332
1 -2.464959 1.707813 0.000360
1 3.418121 -0.464638 -0.000300
6 -2.581089 -0.880117 0.000112
1 -3.074913 -0.461544 0.885171
1 -3.075027 -0.461390 -0.884810
1 -2.739196 -1.960714 0.000029
1 1.066759 1.933490 0.000087
p_5mC/p_5mC34.xyz
6 1.577701 -0.104580 -0.000131
6 -0.640429 0.778112 0.000134
6 -1.127531 -0.554452 0.000046
6 -0.170185 -1.546216 -0.000095
1 -0.485259 -2.584828 -0.000154
7 1.177120 -1.348778 -0.000186
7 0.717540 0.952839 0.000037
8 2.888795 0.133279 -0.000255
7 -1.415640 1.857237 0.000293
1 -1.025300 2.792596 0.000346
1 -2.422379 1.761486 0.000357
1 3.119310 1.078916 -0.000338
6 -2.603037 -0.829797 0.000104
1 -3.086862 -0.398894 0.884875
1 -3.086960 -0.398712 -0.884524
1 -2.787275 -1.906253 0.000003
1 1.092103 1.901976 0.000116
p_5mC/p_5mC15.xyz
6 1.692931 0.171287 -0.000002
6 -0.549560 0.691939 0.000002
6 -1.016751 -0.637955 -0.000003
6 0.003728 -1.563189 0.000011
1 -0.169760 -2.632977 0.000018

7 1.293677 -1.162152 0.000019
7 0.687055 1.111844 0.000005
8 2.898131 0.466616 -0.000023
7 -1.555155 1.769756 0.000010
1 -1.096149 2.689053 0.000014
1 -2.164651 1.709863 0.828523
1 2.033764 -1.860071 0.000019
6 -2.472555 -1.007556 -0.000019
1 -2.981468 -0.609218 0.885061
1 -2.981445 -0.609227 -0.885116
1 -2.586468 -2.093532 -0.000015
1 -2.164655 1.709881 -0.828500
p_5mC/p_5mC25.xyz
6 1.576420 0.061804 -0.000001
6 -0.577524 0.682157 -0.000003
6 -1.038380 -0.629666 -0.000001
6 -0.000998 -1.569577 -0.000005
1 -0.233750 -2.630442 -0.000007
7 1.297543 -1.243442 -0.000005
7 0.689566 1.062682 -0.000002
8 2.865982 0.448971 0.000005
7 -1.548958 1.785451 -0.000002
1 -1.068735 2.693873 -0.000021
1 -2.160039 1.743582 0.828059
1 3.435598 -0.339845 0.000027
6 -2.492417 -1.001784 0.000007
1 -3.002086 -0.602516 0.884471
1 -3.002109 -0.602472 -0.884423
1 -2.606329 -2.087950 -0.000019
1 -2.160070 1.743561 -0.828039
p_5mC/p_5mC35.xyz
6 1.581796 -0.006147 0.000756
6 -0.555639 0.685278 0.004125
6 -1.055705 -0.610637 0.001151
6 -0.045331 -1.582300 -0.001482
1 -0.313184 -2.634850 -0.004150

7 1.262573 -1.301565 -0.002067
7 0.725553 1.021374 0.004253
8 2.900771 0.264543 -0.000881
7 -1.486793 1.822170 -0.002784
1 -0.976276 2.711617 0.061536
1 -2.148633 1.770960 0.784391
1 3.038468 1.227252 -0.000510
6 -2.520028 -0.942501 0.000996
1 -3.013825 -0.547481 0.896551
1 -3.023316 -0.510801 -0.871719
1 -2.663870 -2.024861 -0.019876
1 -2.045423 1.835812 -0.868271
p_5mC/p_5mC45.xyz
6 1.726615 0.002473 -0.001053
6 -0.589433 0.668533 0.013701
6 -1.028720 -0.623467 0.005954
6 0.028826 -1.579548 0.001322
1 -0.242929 -2.632866 -0.000791
7 1.318294 -1.311010 -0.001865
7 0.727236 0.979146 0.006818
8 2.917294 0.351636 -0.006114
7 -1.509916 1.799150 -0.005761
1 -1.087185 2.658797 0.372945
1 -2.350467 1.598134 0.554226
1 1.033245 1.951629 0.014703
6 -2.479749 -1.016428 -0.003571
1 -2.995190 -0.656468 0.894318
1 -2.997214 -0.606359 -0.877401
1 -2.571358 -2.104120 -0.032138
1 -1.821794 1.997774 -0.969407
p_5mC/p_5mC23.xyz
6 1.430769 -0.037085 -0.009257
6 -0.645829 0.794430 -0.002231
6 -1.141524 -0.547943 0.003026
6 -0.189735 -1.544136 -0.002226
1 -0.486230 -2.587646 0.001623

7 1.157071 -1.308800 -0.011542
7 0.690511 1.037770 -0.008132
8 2.849291 0.255326 -0.064558
7 -1.463734 1.856708 -0.001072
1 -1.078532 2.791645 -0.014758
1 -2.467389 1.746849 -0.006171
1 3.415599 -0.477163 0.265934
6 -2.614942 -0.833518 0.013411
1 -3.096550 -0.396878 0.896950
1 -3.106530 -0.408532 -0.870336
1 -2.798870 -1.910469 0.021595
1 3.104810 1.109359 0.350519
p_C/p_C14.xyz
6 -1.237072 -0.453919 0.000000
6 1.195595 -0.208961 0.000001
6 1.055896 1.206139 0.000003
6 -0.201779 1.725094 0.000001
1 -0.396102 2.790587 0.000003
7 -1.303500 0.921061 -0.000007
7 0.055612 -0.960375 0.000011
8 -2.225265 -1.183587 -0.000002
7 2.368050 -0.820667 -0.000015
1 2.442579 -1.832161 0.000032
1 3.220308 -0.272440 0.000020
1 1.931926 1.839933 0.000005
1 -2.234029 1.329510 0.000016
1 0.120469 -1.976986 -0.000006
p_C/p_C13.xyz
6 -1.103579 -0.373145 -0.000009
6 1.180050 -0.282905 0.000000
6 1.145001 1.147156 0.000007
6 -0.074712 1.744732 0.000001
1 -0.226422 2.816179 0.000003
7 -1.207056 0.976069 -0.000013
7 0.037382 -1.019460 0.000000
8 -2.269523 -1.008052 0.000009

7 2.333291 -0.945768 0.000031
1 2.338962 -1.958075 -0.000096
1 3.215546 -0.449933 -0.000118
1 2.056551 1.730763 0.000022
1 -2.128822 1.406290 0.000002
1 -2.125508 -1.971713 0.000000
p_C/p_C12.xyz
6 -1.095549 -0.397300 -0.000084
6 1.188567 -0.277644 0.000005
6 1.141452 1.152469 0.000162
6 -0.083446 1.736212 0.000176
1 -0.248444 2.805745 0.000284
7 -1.209718 0.954717 0.000044
7 0.056051 -1.025169 -0.000113
8 -2.189552 -1.152888 -0.000178
7 2.349346 -0.928452 -0.000019
1 2.364700 -1.940634 -0.000111
1 3.226580 -0.423917 0.000102
1 2.046808 1.745686 0.000267
1 -2.129038 1.391677 0.000053
1 -3.020082 -0.645551 -0.000117
p_C/p_C24.xyz
6 -1.178679 -0.261500 -0.000003
6 1.202085 -0.236199 -0.000001
6 1.119745 1.174422 0.000001
6 -0.129402 1.744040 0.000002
1 -0.234310 2.823519 0.000000
7 -1.298108 1.041774 0.000003
7 0.015614 -0.916611 -0.000018
8 -2.222334 -1.082756 0.000010
7 2.337222 -0.922909 -0.000008
1 2.352639 -1.935843 0.000050
1 3.219780 -0.426343 0.000037
1 2.023744 1.769559 0.000005
1 -3.053004 -0.573070 -0.000020
1 0.004229 -1.936133 0.000025

p_C/p_C34.xyz
6 -1.193346 -0.229754 0.000001
6 1.194872 -0.246697 0.000002
6 1.139471 1.163000 0.000000
6 -0.101064 1.753519 0.000000
1 -0.185023 2.834908 -0.000002
7 -1.281666 1.076337 -0.000007
7 -0.006786 -0.905136 0.000018
8 -2.324172 -0.928180 -0.000002
7 2.314311 -0.959660 0.000024
1 2.306352 -1.972747 -0.000133
1 3.207630 -0.482673 -0.000099
1 2.053766 1.742106 -0.000006
1 -2.192184 -1.892328 -0.000070
1 -0.017785 -1.925021 0.000060
p_C/p_C15.xyz
6 1.210262 -0.544283 -0.000557
6 -1.056694 -0.152129 -0.012518
6 -0.949817 1.244709 -0.006971
6 0.341630 1.717323 0.002645
1 0.594338 2.770699 0.006519
7 1.368795 0.844654 0.005392
7 -0.081906 -1.021325 -0.008885
8 2.204725 -1.282002 0.001566
7 -2.403272 -0.751042 0.006350
1 -2.399729 -1.668671 -0.457544
1 -3.096585 -0.148557 -0.455333
1 -1.807801 1.903212 -0.011761
1 2.325386 1.191564 0.010480
1 -2.711018 -0.891971 0.979514
p_C/p_C25.xyz
6 1.154394 -0.360849 -0.000206
6 -1.077846 -0.160079 -0.005913
6 -1.012234 1.220446 -0.004131
6 0.285472 1.737020 0.001633
1 0.452766 2.809238 0.004124

7 1.374523 0.959545 0.003864
7 -0.034229 -0.974031 -0.004544
8 2.210293 -1.190720 0.000042
7 -2.379422 -0.844195 0.003808
1 -2.418901 -1.561202 -0.732772
1 -3.156478 -0.188285 -0.142046
1 -1.890889 1.853236 -0.007118
1 3.035420 -0.675069 0.003255
1 -2.529081 -1.320625 0.904027
p_C/p_C35.xyz
6 1.172183 -0.323845 -0.000095
6 -1.067755 -0.164823 -0.005045
6 -1.024420 1.215042 -0.003479
6 0.265978 1.753518 0.001393
1 0.411961 2.828873 0.003515
7 1.369675 0.999326 0.003273
7 -0.006264 -0.958039 -0.003815
8 2.291306 -1.066070 0.000483
7 -2.355505 -0.873530 0.003218
1 -2.388981 -1.576266 -0.747537
1 -3.145732 -0.229401 -0.123348
1 -1.913112 1.833690 -0.005999
1 2.062009 -2.011467 -0.001095
1 -2.487848 -1.370526 0.895211
p_C/p_C45.xyz
6 1.300401 -0.423403 0.000000
6 -1.086431 -0.092930 0.000005
6 -0.939797 1.257528 0.000005
6 0.403901 1.719384 -0.000003
1 0.583279 2.791294 -0.000001
7 1.469461 0.945114 -0.000004
7 -0.013150 -0.913125 0.000007
8 2.242335 -1.226470 -0.000001
7 -2.387562 -0.747492 -0.000007
1 -2.507224 -1.344622 -0.833231
1 -3.144247 -0.050783 0.000008

1 -1.786010 1.931775 0.000007
1 -0.126931 -1.926231 0.000002
1 -2.507231 -1.344640 0.833207
p_C/p_C23.xyz
6 -1.039624 -0.193393 -0.011581
6 1.193461 -0.311499 -0.000548
6 1.207751 1.110211 0.008824
6 -0.000974 1.760888 0.006180
1 -0.055160 2.843650 0.014052
7 -1.198732 1.099978 -0.004153
7 0.005814 -0.975263 -0.015018
8 -2.284847 -0.928644 -0.068994
7 2.315586 -1.041027 0.003542
1 2.270762 -2.051180 -0.005788
1 3.220160 -0.589955 0.011884
1 2.144382 1.654660 0.019181
1 -3.062355 -0.405488 0.228262
1 -2.261379 -1.805590 0.376517
rc_1m5caC/rc_1m5caC01.xyz
6 -1.880826 0.571447 0.023580
6 0.481311 1.190234 0.055300
6 0.828394 -0.204948 -0.061395
6 -0.208272 -1.140844 -0.106443
1 0.013404 -2.200380 -0.172515
7 -1.490654 -0.786871 -0.085146
7 -0.842679 1.485321 0.093082
8 -3.048083 0.903601 0.053535
7 1.371261 2.151396 0.123621
1 1.089701 3.124942 0.214965
1 2.362870 1.941689 0.048087
6 2.241525 -0.677982 -0.049488
8 3.116458 -0.247657 -0.811104
8 2.387195 -1.530384 0.892220
1 -1.138040 2.460383 0.164123
6 -2.581979 -1.778962 -0.152926
1 -3.157074 -1.729120 0.772868

1 -3.223868 -1.540483 -1.001511
1 -2.137961 -2.764090 -0.275890
rc_1m5caC/rc_1m5caC02.xyz
6 -1.907710 0.647223 -0.022012
6 0.335060 1.265608 -0.003263
6 0.772906 -0.136016 0.009080
6 -0.236165 -1.124173 0.037401
1 0.008334 -2.179769 0.064614
7 -1.505076 -0.780626 0.031778
7 -0.955203 1.589440 -0.027853
8 -3.115709 0.851217 -0.055680
7 1.238095 2.235055 0.004144
1 0.924824 3.200877 -0.006488
1 2.230494 2.015252 0.030743
6 2.210651 -0.548799 -0.000279
8 3.131436 0.249369 0.091803
8 2.357806 -1.866470 -0.116533
1 3.307980 -2.093730 -0.116532
6 -2.574415 -1.783856 0.053181
1 -3.229389 -1.576026 0.901073
1 -3.149501 -1.694780 -0.871130
1 -2.127687 -2.771763 0.139868
rc_1m5caC/rc_1m5caC03.xyz
6 -1.901036 0.638096 -0.032730
6 0.344273 1.249715 -0.095116
6 0.772599 -0.153042 -0.015851
6 -0.236053 -1.132148 0.079014
1 0.006867 -2.187063 0.162765
7 -1.505458 -0.786184 0.073849
7 -0.944179 1.575800 -0.114822
8 -3.107650 0.850876 -0.036523
7 1.250515 2.214642 -0.179394
1 0.936786 3.178422 -0.239693
1 2.243960 2.027364 -0.107045
6 2.213352 -0.555573 0.075948
8 2.978573 0.100309 0.763582

8 2.635098 -1.643792 -0.561233
1 1.985289 -2.056360 -1.159053
6 -2.577928 -1.782782 0.159664
1 -3.210695 -1.542863 1.015975
1 -3.175191 -1.724949 -0.752888
1 -2.132564 -2.769093 0.270326
rc_1m5caC/rc_1m5caC04.xyz
6 1.890045 0.645297 0.044920
6 -0.356921 1.228299 -0.132064
6 -0.763810 -0.172091 -0.001773
6 0.254148 -1.146285 0.008869
1 0.014887 -2.204865 0.015195
7 1.518104 -0.784062 0.027399
7 0.923974 1.573457 -0.070240
8 3.088049 0.882405 0.149006
7 -1.273372 2.171029 -0.322309
1 -0.977027 3.137592 -0.416021
1 -2.266316 1.969827 -0.351734
6 -2.179348 -0.670072 0.004865
8 -2.521301 -1.425033 -0.888046
8 -3.023167 -0.279882 0.951090
1 -2.614695 0.253897 1.659110
6 2.606263 -1.769736 0.043951
1 3.258262 -1.578945 -0.810195
1 3.177538 -1.646030 0.965615
1 2.175497 -2.766835 -0.014928
rc_1m5caC/rc_1m5caC05.xyz
6 -1.751581 0.560366 0.013199
6 0.447359 1.195654 0.055463
6 0.852358 -0.188138 -0.056212
6 -0.154933 -1.147402 -0.096671
1 0.069273 -2.206337 -0.149252
7 -1.445222 -0.791716 -0.088663
7 -0.866854 1.517526 0.086873
8 -3.040996 0.797033 0.039372
7 1.328197 2.175954 0.122603

1 1.006740 3.136644 0.198326
1 2.326499 2.000437 0.069547
6 2.278482 -0.629129 -0.057854
8 3.130001 -0.285251 -0.879390
8 2.439450 -1.372821 0.976602
1 -3.214343 1.753609 0.132899
6 -2.533207 -1.794139 -0.144347
1 -3.108458 -1.739244 0.780503
1 -3.173284 -1.576541 -0.999237
1 -2.077771 -2.775886 -0.252615
rc_1m5caC/rc_1m5caC06.xyz
6 -1.751064 0.586632 0.011049
6 0.457978 1.197571 0.065314
6 0.853040 -0.188390 -0.052465
6 -0.161398 -1.136575 -0.087578
1 0.053413 -2.197480 -0.138393
7 -1.452640 -0.771934 -0.078049
7 -0.850994 1.529503 0.090214
8 -3.008659 0.967374 0.026706
7 1.346379 2.171112 0.143743
1 1.031223 3.133395 0.223876
1 2.343347 1.988587 0.094908
6 2.276154 -0.640168 -0.068629
8 3.125846 -0.294115 -0.889912
8 2.436849 -1.397126 0.957725
1 -3.660274 0.245096 -0.029658
6 -2.533040 -1.783614 -0.129544
1 -3.121107 -1.724256 0.789363
1 -3.155151 -1.602033 -1.008382
1 -2.072974 -2.765863 -0.208104
rc_1m5caC/rc_1m5caC07.xyz
6 -1.853179 0.660011 -0.017578
6 0.411402 1.104108 -0.066625
6 0.771175 -0.265816 0.041024
6 -0.303646 -1.170457 0.087629
1 -0.132145 -2.239509 0.150364

7 -1.555060 -0.738129 0.067616
7 -0.798091 1.556969 -0.079270
8 -3.018123 1.023572 -0.033809
7 1.470431 2.111232 -0.162959
1 1.951717 2.043972 -1.074075
1 2.182680 1.973251 0.571072
6 2.178144 -0.752835 0.062029
8 3.031886 -0.252741 0.814125
8 2.364276 -1.667060 -0.803486
1 1.073562 3.056473 -0.079265
6 -2.702944 -1.664503 0.128901
1 -3.304972 -1.419172 1.004576
1 -3.299845 -1.537478 -0.775029
1 -2.321996 -2.681273 0.197730
rc_1m5caU/rc_1m5caU27.xyz
6 1.906909 -0.615401 0.000573
6 -0.465042 -1.240646 -0.002431
6 -0.804624 0.191407 -0.001188
6 0.233077 1.146561 -0.001236
1 0.005593 2.207725 -0.001409
7 1.500872 0.782841 -0.000469
7 0.881248 -1.524467 -0.001710
8 3.081416 -0.899679 0.003087
8 -1.318495 -2.128510 -0.003811
1 1.155918 -2.507926 -0.001491
6 -2.220532 0.682079 0.000547
8 -2.451502 1.882003 -0.004203
8 -3.190145 -0.223015 0.007741
1 -2.818349 -1.139615 0.007552
6 2.592221 1.769831 0.001034
1 3.199634 1.610275 0.894287
1 3.205283 1.606731 -0.887646
1 2.154832 2.764810 -0.002339
rc_1m5caU/rc_1m5caU25.xyz
6 1.924927 0.576469 0.003096
6 -0.438289 1.275164 -0.108876

6 -0.807644 -0.146726 0.006170
6 0.200727 -1.130133 0.049842
1 -0.054702 -2.184213 0.083969
7 1.479723 -0.805128 0.067020
7 0.928688 1.508553 -0.091008
8 3.109119 0.827852 0.026407
8 -1.245366 2.187929 -0.223612
1 1.233954 2.479583 -0.161992
6 -2.223763 -0.612938 -0.010429
8 -2.516140 -1.608455 -0.654786
8 -3.052282 0.134690 0.698619
1 -3.962543 -0.217588 0.633043
6 2.540628 -1.823450 0.129390
1 3.132143 -1.755216 -0.786365
1 3.176883 -1.612112 0.990242
1 2.073233 -2.800876 0.220843
rc_1m5caU/rc_1m5caU26.xyz
6 1.936652 0.537650 -0.009667
6 -0.403772 1.311276 -0.108793
6 -0.818343 -0.099959 -0.003793
6 0.157149 -1.112556 0.073169
1 -0.127487 -2.157364 0.135995
7 1.447786 -0.829223 0.070545
7 0.969858 1.500001 -0.101148
8 3.128295 0.750572 0.001785
8 -1.181060 2.249591 -0.217637
1 1.305685 2.460446 -0.177094
6 -2.266518 -0.451307 0.082166
8 -2.572802 -1.515203 -0.651954
8 -3.025739 0.178938 0.794974
1 -3.514469 -1.749756 -0.531307
6 2.472835 -1.883228 0.126381
1 3.059552 -1.837382 -0.793964
1 3.123076 -1.690059 0.981361
1 1.972564 -2.843771 0.225108
rc_1m5caU/rc_1m5caU01.xyz

6 1.922563 0.549364 -0.020937
6 -0.429473 1.277717 -0.082891
6 -0.811465 -0.141622 -0.003721
6 0.175975 -1.138044 0.066879
1 -0.087379 -2.189461 0.125655
7 1.462243 -0.825749 0.057689
7 0.937735 1.497158 -0.091135
8 3.110155 0.786035 -0.025468
8 -1.238807 2.193326 -0.154809
1 1.254911 2.464958 -0.154385
6 -2.262232 -0.488159 0.109621
8 -2.861688 -1.108673 -0.893792
8 -2.837886 -0.204657 1.143248
1 -2.274400 -1.247219 -1.661515
6 2.511923 -1.856124 0.103351
1 3.084752 -1.802846 -0.825191
1 3.168995 -1.645099 0.948639
1 2.035337 -2.827239 0.213685
rc_1m5caU/rc_1m5caU24.xyz
6 -1.885348 -0.651084 0.011494
6 0.354902 -1.241668 -0.001719
6 0.755540 0.156020 -0.005501
6 -0.266641 1.130821 -0.018080
1 -0.036790 2.190483 -0.031671
7 -1.530150 0.765380 -0.015492
7 -0.907855 -1.597565 0.010998
8 -3.079583 -0.914641 0.033586
8 1.249374 -2.205504 -0.007157
1 2.160786 -1.815469 -0.018969
6 2.193763 0.542170 0.001192
8 2.388832 1.849622 0.050019
8 3.079435 -0.307579 -0.034344
1 3.345379 2.051712 0.061102
6 -2.626098 1.747739 -0.026066
1 -3.267561 1.542269 -0.884181
1 -3.202032 1.633367 0.894051

1 -2.194911 2.743761 -0.093629
rc_1m5caU/rc_1m5caU23.xyz
6 1.878015 -0.672464 -0.011581
6 -0.379293 -1.218591 -0.026645
6 -0.751466 0.188645 -0.007956
6 0.298078 1.136571 0.007220
1 0.079657 2.199294 0.022074
7 1.552816 0.749637 0.015568
7 0.877287 -1.594242 -0.033105
8 3.064845 -0.966203 -0.010592
8 -1.271763 -2.188669 -0.036108
1 -3.967542 0.155231 0.134238
6 -2.143447 0.732236 -0.007228
8 -2.372649 1.920358 -0.138308
8 -3.065388 -0.220045 0.148668
1 -2.190381 -1.847115 0.007878
6 2.668468 1.709331 0.055327
1 3.270088 1.504234 0.942306
1 3.278378 1.567431 -0.838225
1 2.256602 2.715257 0.090382
rc_1m5caU/rc_1m5caU19.xyz
6 1.894422 0.606510 -0.004770
6 -0.331103 1.246989 -0.095786
6 -0.775171 -0.131229 0.000324
6 0.222128 -1.128138 0.066120
1 -0.037004 -2.179910 0.123364
7 1.496520 -0.796492 0.059161
7 0.935898 1.573032 -0.094128
8 3.092164 0.842419 0.018958
8 -1.268516 2.163147 -0.217929
1 -0.875687 3.053984 -0.301295
6 -2.216279 -0.515278 0.085861
8 -2.479204 -1.623152 -0.599995
8 -3.009658 0.123360 0.752812
1 -3.417108 -1.874980 -0.487011
6 2.565855 -1.806453 0.104638

1 3.147725 -1.731398 -0.816013
1 3.208859 -1.595019 0.960034
1 2.108881 -2.789044 0.196611
rc_1m5caU/rc_1m5caU08.xyz
6 1.910974 0.543443 -0.002567
6 -0.442066 1.182049 0.001363
6 -0.815126 -0.181531 -0.000463
6 0.200221 -1.126673 0.000688
1 -0.031578 -2.186706 0.000393
7 1.495741 -0.796820 0.002625
7 0.871377 1.475069 -0.000357
8 3.079761 0.876867 -0.007670
8 -1.348290 2.118000 0.005151
1 1.167503 2.453379 -0.001213
6 -2.201806 -0.625506 -0.001665
8 -2.564678 -1.842289 -0.004070
8 -3.223528 0.133253 -0.000759
1 -1.001102 3.032348 0.007676
6 2.564711 -1.817946 0.005282
1 3.158213 -1.711425 -0.903154
1 3.194032 -1.666586 0.882432
1 2.095535 -2.798404 0.040940
rc_1m5caU/rc_1m5caU21.xyz
6 1.872650 0.670892 -0.001041
6 -0.378645 1.200189 -0.014780
6 -0.765564 -0.197710 -0.003213
6 0.287094 -1.143097 0.001478
1 0.073338 -2.206452 0.006100
7 1.541347 -0.751140 0.004162
7 0.865245 1.593663 -0.012001
8 3.056004 0.965704 0.005483
8 -1.365681 2.077450 -0.023189
1 -1.037680 2.997830 -0.019763
6 -2.166897 -0.739774 0.001261
8 -2.343989 -1.947126 -0.052821
8 -3.193330 0.101829 0.060886

1 -2.920292 1.042061 0.088056
6 2.658049 -1.710957 0.016309
1 3.270374 -1.540053 -0.870571
1 3.257095 -1.533439 0.911048
1 2.246869 -2.717725 0.017064
rc_1m5caU/rc_1m5caU16.xyz
6 1.883520 0.616362 -0.025860
6 -0.350111 1.212072 -0.077647
6 -0.766008 -0.174079 -0.004648
6 0.241930 -1.154015 0.063291
1 0.002315 -2.210863 0.125972
7 1.510436 -0.793901 0.050210
7 0.906348 1.568397 -0.096587
8 3.077037 0.871943 -0.026462
8 -1.323057 2.097366 -0.153483
1 -0.966661 3.005194 -0.208855
6 -2.211187 -0.546785 0.113493
8 -2.796895 -1.198000 -0.879321
8 -2.796792 -0.256941 1.140052
1 -2.207084 -1.339694 -1.644420
6 2.599952 -1.781223 0.106398
1 3.185563 -1.700833 -0.811204
1 3.234374 -1.551777 0.963794
1 2.163094 -2.772450 0.202907
rc_1m5caU/rc_1m5caU20.xyz
6 -1.886328 -0.657870 -0.000016
6 0.361532 -1.224230 -0.000045
6 0.758204 0.182746 0.000009
6 -0.283685 1.137569 0.000061
1 -0.099109 2.206515 0.000107
7 -1.544182 0.760001 0.000051
7 -0.899140 -1.589885 -0.000057
8 -3.078520 -0.933755 -0.000050
8 1.255283 -2.184985 -0.000080
1 2.165307 -1.780829 -0.000054
6 2.212543 0.547366 0.000010

8 2.616066 1.807378 0.000050
8 3.054829 -0.348350 -0.000048
1 1.923890 2.490329 0.000068
6 -2.649739 1.732360 0.000089
1 -3.257242 1.564193 -0.890699
1 -3.257358 1.563996 0.890759
1 -2.228667 2.735038 0.000229
rc_1m5caU/rc_1m5caU22.xyz
6 1.781493 -0.607103 -0.025470
6 -0.425121 -1.241234 -0.005640
6 -0.828081 0.172311 -0.010717
6 0.185256 1.146485 0.022777
1 -0.040928 2.206661 0.049443
7 1.457526 0.779664 0.032776
7 0.909470 -1.561272 -0.039604
8 3.073722 -0.797262 -0.066596
8 -1.291304 -2.135814 0.042198
1 3.284697 -1.749333 -0.133476
6 -2.254679 0.638053 -0.024579
8 -2.509792 1.828099 -0.137401
8 -3.205724 -0.279385 0.088868
1 -2.811695 -1.185631 0.135177
6 2.548444 1.771899 0.089349
1 3.171029 1.552373 0.958756
1 3.144138 1.678526 -0.820918
1 2.104710 2.761085 0.167946
rc_1m5caU/rc_1m5caU05.xyz
6 -1.807409 0.488564 -0.002357
6 0.512609 1.241954 0.004240
6 0.868567 -0.160340 0.001699
6 -0.101663 -1.120179 0.005059
1 0.138552 -2.177466 0.003552
7 -1.431433 -0.811364 0.009393
7 -0.886842 1.456661 -0.000966
8 -3.084851 0.716771 -0.014971
8 1.274883 2.200747 0.008775

1 -3.329255 1.662890 -0.037993
6 2.275038 -0.552633 -0.003771
8 2.708408 -1.736936 -0.018207
8 3.239762 0.290063 0.005721
1 -1.198900 2.430196 -0.003751
6 -2.473215 -1.869945 0.010366
1 -3.131398 -1.717990 0.865039
1 -3.035702 -1.817093 -0.921792
1 -1.964539 -2.827305 0.094008
rc_1m5caU/rc_1m5caU14.xyz
6 -1.796060 0.575922 0.004148
6 0.403361 1.266171 -0.132110
6 0.825771 -0.136170 -0.004045
6 -0.159950 -1.134307 0.041427
1 0.090469 -2.188964 0.073247
7 -1.443158 -0.799823 0.058935
7 -0.948907 1.545094 -0.098254
8 -3.094946 0.744353 0.059422
8 1.234534 2.173206 -0.276378
1 -3.320934 1.693534 0.012661
6 2.253640 -0.566744 -0.002537
8 2.595392 -1.516244 -0.690414
8 3.038177 0.160640 0.776034
1 3.959030 -0.164105 0.723219
6 -2.508382 -1.820291 0.115693
1 -3.130452 -1.627316 0.990893
1 -3.111090 -1.734884 -0.790759
1 -2.038104 -2.798288 0.181217
rc_1m5caU/rc_1m5caU12.xyz
6 -1.879979 0.636391 0.029284
6 0.360354 1.232876 -0.136836
6 0.768068 -0.156348 -0.004387
6 -0.256062 -1.130417 0.028744
1 -0.013745 -2.187905 0.051174
7 -1.520489 -0.773604 0.049992
7 -0.900316 1.578088 -0.081048

8 -3.069313 0.908483 0.099017
8 1.219092 2.223814 -0.305471
1 2.150335 1.954990 -0.393563
6 2.173273 -0.675292 0.005300
8 2.452885 -1.556949 -0.788969
8 3.072880 -0.172554 0.836542
1 2.707320 0.464427 1.479005
6 -2.612797 -1.760785 0.088618
1 -3.237166 -1.556348 0.958875
1 -3.206165 -1.652352 -0.821021
1 -2.176427 -2.755107 0.149635
rc_1m5caU/rc_1m5caU06.xyz
6 -1.777939 0.533502 -0.001440
6 0.405130 1.182949 0.001293
6 0.834815 -0.172931 0.000392
6 -0.149337 -1.137990 0.002041
1 0.080371 -2.197351 0.002180
7 -1.454466 -0.798287 0.003467
7 -0.887696 1.506946 -0.000819
8 -3.065328 0.778835 -0.006687
8 1.306453 2.140975 0.004096
1 -3.234250 1.740362 -0.009758
6 2.236794 -0.584688 -0.001501
8 2.631676 -1.789753 -0.006529
8 3.234365 0.204552 0.001387
1 0.879300 3.019326 0.003612
6 -2.526652 -1.822394 0.004413
1 -3.158823 -1.669407 0.878722
1 -3.112646 -1.720893 -0.909060
1 -2.053016 -2.800218 0.046445
rc_1m5caU/rc_1m5caU15.xyz
6 1.810049 0.544690 -0.012157
6 -0.369135 1.300633 -0.114908
6 -0.836087 -0.088327 0.001440
6 0.119040 -1.113130 0.071836
1 -0.158466 -2.159082 0.134334

7 1.413737 -0.818486 0.064897
7 0.990721 1.538938 -0.097114
8 3.114441 0.671199 0.007450
8 -1.172489 2.234188 -0.250836
1 3.369593 1.611511 -0.064268
6 -2.291064 -0.412308 0.094984
8 -2.631577 -1.436475 -0.682712
8 -3.031805 0.201118 0.841050
1 -3.579454 -1.646534 -0.565171
6 2.442391 -1.874968 0.123998
1 3.033891 -1.828542 -0.792792
1 3.085144 -1.686195 0.985460
1 1.938355 -2.834106 0.217176
rc_1m5caU/rc_1m5caU09.xyz
6 1.793817 0.556914 -0.019692
6 -0.397916 1.270042 -0.068678
6 -0.830289 -0.131773 0.012393
6 0.139478 -1.139963 0.076595
1 -0.115359 -2.192599 0.137745
7 1.428079 -0.814571 0.055617
7 0.954969 1.538284 -0.072544
8 3.095524 0.709857 -0.034943
8 -1.234390 2.180345 -0.151246
1 3.329106 1.655271 -0.113897
6 -2.287284 -0.455512 0.109488
8 -2.891333 -1.012535 -0.930850
8 -2.870960 -0.212233 1.148814
1 -2.293826 -1.132716 -1.693177
6 2.483677 -1.845553 0.094344
1 3.058720 -1.782624 -0.831651
1 3.135012 -1.644706 0.946435
1 2.005393 -2.817018 0.192132
rc_1m5caU/rc_1m5caU18.xyz
6 1.781864 -0.634435 0.003529
6 -0.436248 -1.240460 0.010539
6 -0.829643 0.172881 0.006836

6 0.192672 1.136240 0.001911
1 -0.024928 2.198629 -0.001570
7 1.464370 0.761068 -0.003056
7 0.892218 -1.573177 0.013669
8 3.045674 -0.975349 -0.000424
8 -1.311933 -2.130081 0.007072
1 3.685191 -0.239436 -0.005865
6 -2.251666 0.650269 0.001432
8 -2.497879 1.847206 0.033932
8 -3.209167 -0.265878 -0.041356
1 -2.817215 -1.174954 -0.044591
6 2.549247 1.764031 -0.016847
1 3.157955 1.631489 0.882280
1 3.150089 1.616082 -0.918902
1 2.101873 2.754605 -0.023836
rc_1m5caU/rc_1m5caU04.xyz
6 1.776392 0.561538 -0.000001
6 -0.415994 1.185715 0.000003
6 -0.835840 -0.173813 0.000003
6 0.156391 -1.127329 0.000003
1 -0.062602 -2.188944 0.000003
7 1.461653 -0.776427 0.000002
7 0.870996 1.521610 0.000001
8 3.031847 0.945040 -0.000003
8 -1.326165 2.136069 0.000003
1 3.681585 0.217836 -0.000011
6 -2.234711 -0.598166 0.000000
8 -2.619211 -1.806220 0.000002
8 -3.238011 0.183612 -0.000008
1 -0.904056 3.016925 0.000000
6 2.528464 -1.807501 0.000000
1 3.134446 -1.694137 -0.901249
1 3.134453 -1.694132 0.901243
1 2.051742 -2.784500 0.000002
rc_1m5dhmC/rc_1m5dhmC07.xyz
6 -1.918015 0.709494 0.053119

6 0.372807 1.111092 -0.009856
6 0.670080 -0.317623 -0.135304
6 -0.426894 -1.194034 -0.155064
1 -0.281074 -2.266245 -0.241651
7 -1.661856 -0.736540 -0.075652
7 -0.877627 1.558284 0.076833
8 -3.099022 1.038680 0.129340
6 2.063638 -0.901589 -0.106803
1 2.035332 -1.962435 -0.371987
7 1.369629 1.987292 0.024925
1 1.155592 2.974476 0.118806
1 2.333463 1.696124 -0.099305
8 2.524027 -0.743872 1.230176
1 2.522164 -1.607898 1.673371
8 2.875016 -0.169821 -0.985475
1 3.727416 -0.626736 -1.074289
6 -2.825035 -1.632425 -0.114441
1 -3.403621 -1.493332 0.800277
1 -3.444539 -1.366680 -0.973046
1 -2.475408 -2.659907 -0.197154
rc_1m5dhmC/rc_1m5dhmC03.xyz
6 1.969420 0.619954 -0.028247
6 -0.258509 1.283187 -0.074248
6 -0.722535 -0.106741 -0.100954
6 0.261536 -1.104220 0.012242
1 -0.006361 -2.152982 0.044191
7 1.540214 -0.785283 0.072901
7 1.037446 1.585965 -0.074810
8 3.183058 0.808480 -0.051809
6 -2.178012 -0.468446 -0.323652
1 -2.472331 -0.100401 -1.315078
7 -1.147517 2.269572 -0.076581
1 -0.820222 3.230127 -0.072175
1 -2.139463 2.077655 0.020839
8 -3.017036 0.213676 0.589604
1 -2.762859 -0.028112 1.497159

8 -2.306761 -1.863750 -0.240810
1 -3.145503 -2.117315 -0.657072
6 2.585273 -1.810280 0.185462
1 3.196146 -1.787471 -0.719156
1 3.212699 -1.579305 1.047650
1 2.109771 -2.781948 0.303582
rc_1m5dhmC/rc_1m5dhmC01.xyz
6 -1.927442 0.704503 0.044779
6 0.360453 1.135851 0.013762
6 0.677362 -0.283781 -0.163253
6 -0.411301 -1.174222 -0.181793
1 -0.251662 -2.243768 -0.277053
7 -1.650620 -0.737515 -0.082841
7 -0.897408 1.564012 0.095942
8 -3.113542 1.018924 0.098916
6 2.067566 -0.889516 -0.191949
1 2.032729 -1.862144 -0.689024
7 1.343775 2.024405 0.100009
1 1.114402 3.003240 0.236720
1 2.311529 1.744636 -0.027581
8 2.462827 -1.166295 1.139471
1 2.548798 -0.334737 1.637617
8 2.939252 -0.007871 -0.852851
1 3.788718 -0.456798 -0.992396
6 -2.798468 -1.653811 -0.098316
1 -3.367336 -1.514870 0.822496
1 -3.433711 -1.408673 -0.951409
1 -2.431001 -2.675415 -0.174791
rc_1m5dhmC/rc_1m5dhmC05.xyz
6 1.974125 0.633039 -0.005078
6 -0.261103 1.276070 -0.057031
6 -0.717480 -0.117560 -0.080058
6 0.280500 -1.106544 -0.013893
1 0.023599 -2.158376 -0.012397
7 1.556456 -0.777951 0.046045
7 1.033044 1.589317 -0.042073

8 3.186530 0.832695 -0.003536
6 -2.158073 -0.522269 -0.299711
1 -2.389567 -0.313311 -1.357077
7 -1.154209 2.257861 -0.072910
1 -0.831523 3.220023 -0.068092
1 -2.145437 2.051248 0.009629
8 -2.984078 0.267070 0.534164
1 -3.886697 0.242559 0.177832
8 -2.294900 -1.896938 -0.038154
1 -3.045359 -2.235580 -0.550403
6 2.610101 -1.797657 0.114898
1 3.213357 -1.735166 -0.793156
1 3.242340 -1.592280 0.979903
1 2.143426 -2.776794 0.201766
rc_1m5dhmC/rc_1m5dhmC02.xyz
6 1.973516 0.635890 0.012554
6 -0.261209 1.277873 -0.055940
6 -0.717957 -0.115513 -0.092322
6 0.280475 -1.104201 -0.039685
1 0.024026 -2.156829 -0.059272
7 1.556276 -0.776909 0.030559
7 1.032349 1.592288 -0.021820
8 3.185553 0.835644 0.036232
6 -2.162335 -0.530714 -0.323905
1 -2.375073 -0.343581 -1.387490
7 -1.155810 2.258297 -0.074671
1 -0.835737 3.221269 -0.060340
1 -2.147785 2.049364 -0.007759
8 -2.989579 0.261468 0.499977
1 -3.898498 0.199902 0.163769
8 -2.376184 -1.906034 -0.134389
1 -2.291101 -2.110960 0.813019
6 2.610199 -1.797028 0.083119
1 3.220701 -1.711353 -0.818161
1 3.235274 -1.611619 0.957788
1 2.144042 -2.778385 0.142484

rc_1m5dhmC/rc_1m5dhmC06.xyz
6 1.923166 0.708908 0.000005
6 -0.369397 1.095708 -0.000001
6 -0.662180 -0.338034 -0.000006
6 0.434861 -1.212268 -0.000003
1 0.291835 -2.288116 -0.000013
7 1.669321 -0.745670 0.000008
7 0.879414 1.554267 0.000020
8 3.104166 1.044329 -0.000010
6 -2.062856 -0.901443 0.000003
1 -2.027526 -1.994028 0.000000
7 -1.372758 1.965571 -0.000013
1 -1.165355 2.958846 0.000009
1 -2.341375 1.668370 -0.000021
8 -2.689457 -0.423078 -1.172292
1 -3.373691 -1.055479 -1.443722
8 -2.689451 -0.423077 1.172293
1 -3.373656 -1.055493 1.443763
6 2.835621 -1.637468 -0.000004
1 3.434684 -1.430593 -0.888730
1 3.434715 -1.430577 0.888697
1 2.491178 -2.669918 0.000011
rc_1m5dhmC/rc_1m5dhmC09.xyz
6 1.922601 0.701399 0.099741
6 -0.360660 1.122572 -0.029208
6 -0.670098 -0.304327 -0.128778
6 0.422445 -1.185161 -0.195625
1 0.270810 -2.252936 -0.321214
7 1.659509 -0.736573 -0.106168
7 0.889561 1.558972 0.105759
8 3.101985 1.013366 0.240419
6 -2.069387 -0.890340 -0.078256
1 -2.037053 -1.917152 -0.459747
7 -1.352788 2.003941 -0.055581
1 -1.140379 2.992555 0.026824
1 -2.303054 1.701754 -0.250625

8 -2.995994 -0.125604 -0.825805
1 -2.952753 -0.389475 -1.759799
8 -2.451886 -0.855030 1.273247
1 -3.133846 -1.531488 1.417271
6 2.819473 -1.632766 -0.190225
1 3.447938 -1.308985 -1.022164
1 3.387914 -1.558021 0.738273
1 2.467368 -2.650739 -0.345667
rc_1m5dhmC/rc_1m5dhmC08.xyz
6 1.922465 0.708624 0.065591
6 -0.364605 1.135388 0.001809
6 -0.678723 -0.285666 -0.164072
6 0.413549 -1.171251 -0.205077
1 0.258052 -2.239829 -0.318796
7 1.651468 -0.731483 -0.099927
7 0.890443 1.566442 0.108165
8 3.106753 1.023151 0.151421
6 -2.073615 -0.894319 -0.170371
1 -2.041128 -1.849222 -0.702098
7 -1.354095 2.019505 0.058518
1 -1.134936 3.001064 0.191795
1 -2.312846 1.725457 -0.108133
8 -3.042783 -0.039129 -0.737518
1 -3.024496 -0.129041 -1.704685
8 -2.439172 -1.191245 1.156113
1 -2.442997 -0.376687 1.687894
6 2.803542 -1.642032 -0.135290
1 3.438516 -1.373326 -0.981546
1 3.370430 -1.523215 0.789434
1 2.440624 -2.663131 -0.236835
rc_1m5dhmC/rc_1m5dhmC04.xyz
6 1.969766 0.622410 -0.018209
6 -0.258255 1.283865 -0.070881
6 -0.723992 -0.106660 -0.097212
6 0.262030 -1.102434 0.000873
1 -0.001362 -2.153366 0.016447

7 1.540842 -0.784149 0.067475
7 1.037374 1.588005 -0.064832
8 3.183424 0.811558 -0.031619
6 -2.183783 -0.470651 -0.334086
1 -2.453157 -0.099209 -1.329496
7 -1.147894 2.269603 -0.079102
1 -0.821767 3.230584 -0.077427
1 -2.140612 2.077882 0.009756
8 -3.023336 0.207511 0.577277
1 -2.748930 0.004273 1.488283
8 -2.405820 -1.856150 -0.365833
1 -2.311260 -2.225405 0.528574
6 2.585902 -1.810356 0.167863
1 3.194583 -1.778568 -0.738043
1 3.215470 -1.587681 1.030616
1 2.110637 -2.783118 0.277810
rc_1m5dhmU/rc_1m5dhmU68.xyz
6 1.996235 0.455822 0.025909
6 -0.271163 1.401945 -0.155830
6 -0.796116 0.027774 -0.153422
6 0.098389 -1.050316 -0.044962
1 -0.262069 -2.072601 -0.029134
7 1.408229 -0.865988 0.021658
7 1.109164 1.493229 -0.062902
8 3.199917 0.584164 0.104342
8 -0.978404 2.400336 -0.235198
1 1.518923 2.427028 -0.064528
6 -2.288727 -0.183650 -0.168341
8 -2.547416 -1.445268 -0.725152
1 -3.492801 -1.501934 -0.936941
8 -2.739417 -0.069491 1.171420
1 -2.369380 -0.803598 1.693676
1 -2.780944 0.620663 -0.717944
6 2.352605 -1.994134 0.083953
1 3.036610 -1.918377 -0.763058
1 2.917921 -1.923804 1.015093

1 1.785206 -2.920640 0.044406
rc_1m5dhmU/rc_1m5dhmU62.xyz
6 1.988836 0.479584 0.023701
6 -0.296905 1.374419 -0.180818
6 -0.790277 -0.010659 -0.173240
6 0.124549 -1.066886 -0.037452
1 -0.214048 -2.096114 -0.009180
7 1.430226 -0.853209 0.036124
7 1.080365 1.496992 -0.090366
8 3.188831 0.636845 0.109980
8 -1.028632 2.355599 -0.261760
1 1.469580 2.439490 -0.095153
6 -2.277987 -0.245106 -0.196394
8 -2.520490 -1.552143 -0.621331
1 -3.462778 -1.638191 -0.835700
8 -2.687970 -0.102064 1.164408
1 -2.964321 0.818612 1.313809
1 -2.778876 0.497190 -0.823556
6 2.397527 -1.961597 0.114532
1 3.077130 -1.886700 -0.736111
1 2.964123 -1.866580 1.042461
1 1.846678 -2.898614 0.090772
rc_1m5dhmU/rc_1m5dhmU01.xyz
6 1.939652 0.651965 0.000015
6 -0.476811 1.108060 -0.000025
6 -0.711044 -0.345356 -0.000010
6 0.391601 -1.214221 0.000002
1 0.249250 -2.290525 0.000009
7 1.638641 -0.763828 0.000011
7 0.852552 1.485402 -0.000005
8 3.092128 1.027806 0.000019
8 -1.387266 1.933143 -0.000028
1 1.060690 2.483986 -0.000007
6 -2.124491 -0.889338 -0.000006
8 -2.793918 -0.505941 1.178954
1 -2.868522 0.464299 1.211197

8 -2.793923 -0.505957 -1.178968
1 -2.868518 0.464283 -1.211226
1 -2.094626 -1.978806 0.000002
6 2.799860 -1.669996 0.000030
1 3.397996 -1.468777 -0.890695
1 3.397967 -1.468777 0.890775
1 2.438641 -2.695801 0.000025
rc_1m5dhmU/rc_1m5dhmU69.xyz
6 -1.946571 0.640533 0.034878
6 0.467350 1.123659 0.007236
6 0.714309 -0.325181 -0.062303
6 -0.379332 -1.204054 -0.095442
1 -0.225682 -2.276860 -0.160017
7 -1.630547 -0.768819 -0.046562
7 -0.868498 1.483512 0.049847
8 -3.103014 1.002310 0.082327
8 1.362786 1.963308 0.028945
1 -1.085844 2.478625 0.103825
6 2.128719 -0.857698 -0.071564
8 2.874776 -0.282474 -1.118752
1 2.941737 0.677110 -0.964085
8 2.661622 -0.573952 1.202945
1 3.320389 -1.251899 1.424878
1 2.113793 -1.932147 -0.266937
6 -2.780178 -1.688992 -0.065443
1 -3.348311 -1.554261 0.856818
1 -3.411813 -1.439565 -0.920062
1 -2.406092 -2.706997 -0.145305
rc_1m5dhmU/rc_1m5dhmU63.xyz
6 -2.002267 -0.493878 0.044589
6 0.292369 -1.362675 -0.170961
6 0.777513 0.028246 -0.137004
6 -0.154574 1.074781 -0.055002
1 0.163893 2.110988 -0.061366
7 -1.457538 0.846792 0.027420
7 -1.082961 -1.500638 -0.062185

8 -3.199636 -0.660709 0.142578
8 1.028122 -2.336284 -0.285573
1 -1.461313 -2.447650 -0.068820
6 2.271474 0.263952 -0.204529
8 2.601286 1.565558 -0.622202
1 2.519065 2.173645 0.132961
8 2.783893 -0.045596 1.071082
1 3.696263 -0.362388 0.969363
1 2.673733 -0.410035 -0.969878
6 -2.435600 1.945269 0.098177
1 -3.122862 1.847781 -0.744247
1 -2.992347 1.858777 1.032993
1 -1.895751 2.887883 0.053654
rc_1m5dhmU/rc_1m5dhmU57.xyz
6 1.955654 0.627285 0.000003
6 -0.457036 1.141779 -0.000010
6 -0.720358 -0.307350 0.000005
6 0.363095 -1.199850 0.000008
1 0.195743 -2.272324 0.000014
7 1.620361 -0.780553 -0.000001
7 0.888797 1.482310 -0.000020
8 3.118337 0.973665 0.000021
8 -1.330842 2.000499 -0.000016
1 1.118662 2.475901 -0.000014
6 -2.134169 -0.829777 0.000013
8 -2.750646 -0.340545 1.171620
1 -3.356859 -1.016905 1.513278
8 -2.750636 -0.340593 -1.171619
1 -3.356848 -1.016966 -1.513253
1 -2.123812 -1.922792 0.000034
6 2.756545 -1.716771 -0.000006
1 3.359865 -1.531642 -0.890689
1 3.359885 -1.531627 0.890660
1 2.367165 -2.732051 0.000007
rc_1m5dhmU/rc_1m5dhmU64.xyz
6 2.003438 0.460369 0.024438

6 -0.268922 1.392898 -0.143953
6 -0.792545 0.015679 -0.130472
6 0.111464 -1.055647 -0.035551
1 -0.232654 -2.084187 -0.024000
7 1.421045 -0.863985 0.026267
7 1.110795 1.492802 -0.057770
8 3.207088 0.594034 0.092838
8 -0.980955 2.388063 -0.221064
1 1.515287 2.428915 -0.065521
6 -2.294841 -0.176977 -0.168147
8 -2.680204 -1.353351 -0.834354
1 -2.536105 -2.128749 -0.266069
8 -2.790093 -0.087115 1.151131
1 -2.368531 -0.763000 1.710657
1 -2.736425 0.651820 -0.722374
6 2.372260 -1.986872 0.084590
1 3.048888 -1.908596 -0.768209
1 2.944623 -1.910829 1.010862
1 1.810231 -2.916847 0.051339
rc_1m5dhmU/rc_1m5dhmU50.xyz
6 -1.954098 -0.653832 -0.009920
6 0.283458 -1.246711 0.031452
6 0.700845 0.149356 0.089905
6 -0.321252 1.118358 0.057067
1 -0.088040 2.176356 0.084656
7 -1.587725 0.757255 -0.005691
7 -0.977312 -1.601034 0.000858
8 -3.150165 -0.911707 -0.030305
8 1.179239 -2.213465 0.016477
1 2.087565 -1.815552 -0.058776
6 2.131099 0.578228 0.308509
8 2.986081 -0.373642 -0.309796
1 3.849636 -0.355618 0.134030
8 2.312014 1.870365 -0.201988
1 3.025914 2.300846 0.293907
1 2.301364 0.565228 1.398028

6 -2.677066 1.745118 -0.049488
1 -3.272301 1.569027 -0.946661
1 -3.303744 1.609533 0.833464
1 -2.240403 2.741132 -0.065070
rc_1m5dhmU/rc_1m5dhmU56.xyz
6 -1.948980 -0.661604 -0.031034
6 0.291346 -1.245029 0.028423
6 0.702646 0.151518 0.116716
6 -0.325714 1.115050 0.103737
1 -0.098252 2.173647 0.161057
7 -1.589604 0.750748 0.022363
7 -0.967631 -1.603908 -0.027305
8 -3.142950 -0.923821 -0.084314
8 1.190750 -2.208060 0.008619
1 2.098889 -1.805058 -0.030706
6 2.133693 0.608494 0.332888
8 2.996212 -0.361017 -0.234485
1 3.861765 -0.308755 0.202709
8 2.370556 1.897785 -0.164978
1 2.246962 1.897293 -1.130261
1 2.290633 0.671250 1.419408
6 -2.682904 1.734655 -0.007608
1 -3.242293 1.606022 -0.935658
1 -3.341861 1.548283 0.841658
1 -2.252255 2.731833 0.048927
rc_1m5dhmU/rc_1m5dhmU37.xyz
6 -1.960537 0.533923 0.029166
6 0.206080 1.324755 -0.145197
6 0.755445 -0.015982 -0.152397
6 -0.166577 -1.075191 -0.049947
1 0.162643 -2.107319 -0.042906
7 -1.461914 -0.834178 0.022155
7 -1.075445 1.568464 -0.064574
8 -3.169966 0.687292 0.117856
8 1.082006 2.307830 -0.243094
1 0.635122 3.176399 -0.245017

6 2.244767 -0.262956 -0.177754
8 2.723844 -0.094653 1.145807
1 2.327005 -0.778860 1.713686
8 2.462359 -1.556651 -0.676144
1 3.403477 -1.648219 -0.894476
1 2.750303 0.496505 -0.777015
6 -2.451842 -1.922345 0.080618
1 -3.031200 -1.820126 0.999285
1 -3.115473 -1.830935 -0.780701
1 -1.920328 -2.871212 0.061749
rc_1m5dhmU/rc_1m5dhmU54.xyz
6 1.951302 -0.648924 0.014928
6 -0.283450 -1.245381 -0.060418
6 -0.701915 0.149369 -0.104932
6 0.315971 1.120865 -0.051066
1 0.081205 2.178595 -0.070466
7 1.583298 0.761314 0.017303
7 0.976701 -1.599345 -0.019698
8 3.147105 -0.905949 0.052020
8 -1.178973 -2.214769 -0.076311
1 -2.090366 -1.833049 0.008596
6 -2.146000 0.547116 -0.326645
8 -3.036760 -0.359012 0.312407
1 -2.970396 -0.237498 1.277037
8 -2.322543 1.868358 0.093966
1 -3.096926 2.236297 -0.360298
1 -2.349331 0.431198 -1.401584
6 2.670700 1.750853 0.071136
1 3.263512 1.571262 0.969183
1 3.300177 1.621110 -0.810774
1 2.231856 2.745882 0.090403
rc_1m5dhmU/rc_1m5dhmU55.xyz
6 1.896430 0.714024 0.080593
6 -0.384469 1.105371 -0.005241
6 -0.665436 -0.311621 -0.185000
6 0.440613 -1.181932 -0.212652

1 0.305856 -2.252583 -0.331874
7 1.668460 -0.714917 -0.099966
7 0.834728 1.565578 0.117963
8 3.059114 1.079209 0.187187
8 -1.366005 1.988376 0.044726
1 -2.228711 1.556004 -0.174785
6 -2.061225 -0.894013 -0.188945
8 -2.415860 -1.168784 1.151053
1 -2.433849 -0.337528 1.658857
8 -2.915237 0.047668 -0.797008
1 -3.827724 -0.286141 -0.779371
1 -2.082289 -1.854126 -0.709413
6 2.849361 -1.593020 -0.152462
1 3.464034 -1.297361 -1.004505
1 3.418976 -1.464789 0.768897
1 2.513642 -2.622711 -0.259199
rc_1m5dhmU/rc_1m5dhmU45.xyz
6 -1.902694 0.709443 0.094659
6 0.373176 1.127662 0.007700
6 0.671580 -0.284130 -0.176956
6 -0.426036 -1.165139 -0.232563
1 -0.279132 -2.231589 -0.375074
7 -1.658744 -0.713972 -0.114523
7 -0.851405 1.573274 0.139284
8 -3.069529 1.057487 0.213602
8 1.348889 2.015163 0.052908
1 2.210085 1.568596 -0.166597
6 2.074957 -0.861172 -0.209644
8 3.022781 0.117504 -0.601403
1 3.193918 0.046556 -1.555551
8 2.391275 -1.337613 1.075001
1 2.350802 -0.606497 1.717267
1 2.106903 -1.720836 -0.883495
6 -2.829441 -1.604133 -0.186559
1 -3.400376 -1.503290 0.737280
1 -3.446998 -1.296706 -1.032331

1 -2.480751 -2.626871 -0.315514
rc_1m5dhmU/rc_1m5dhmU38.xyz
6 -1.955029 0.560497 0.022502
6 0.228923 1.297214 -0.158384
6 0.745812 -0.056078 -0.161202
6 -0.198840 -1.091505 -0.037681
1 0.104713 -2.131308 -0.020774
7 -1.487958 -0.817636 0.041481
7 -1.045761 1.573826 -0.080724
8 -3.161290 0.743101 0.100656
8 1.131704 2.256481 -0.256750
1 0.709050 3.136942 -0.258692
6 2.229280 -0.325562 -0.203588
8 2.686469 -0.125849 1.132512
1 3.135307 0.734177 1.189802
8 2.425416 -1.657174 -0.581370
1 3.359968 -1.776821 -0.813132
1 2.728703 0.371345 -0.882202
6 -2.503589 -1.880825 0.119986
1 -3.109272 -1.723771 1.013081
1 -3.137338 -1.818542 -0.766468
1 -1.992831 -2.840263 0.162901
rc_1m5dhmU/rc_1m5dhmU44.xyz
6 -1.957274 0.566468 0.035813
6 0.229053 1.294260 -0.159886
6 0.741005 -0.061443 -0.144739
6 -0.210146 -1.093726 -0.044883
1 0.088550 -2.134875 -0.040844
7 -1.497970 -0.814792 0.035266
7 -1.044431 1.575692 -0.071301
8 -3.161706 0.754017 0.128726
8 1.131882 2.249677 -0.283326
1 0.710271 3.130459 -0.302789
6 2.222820 -0.337581 -0.189469
8 2.710339 -0.052911 1.110617
1 3.540211 0.443901 1.033864

8 2.405094 -1.687074 -0.535633
1 3.317671 -1.806227 -0.842124
1 2.695351 0.325058 -0.922218
6 -2.517868 -1.874073 0.104347
1 -3.113167 -1.731079 1.006846
1 -3.160891 -1.790050 -0.773613
1 -2.011601 -2.836594 0.122956
rc_1m5dhmU/rc_1m5dhmU42.xyz
6 -1.893725 0.710439 0.102196
6 0.385228 1.102768 -0.011505
6 0.665365 -0.316279 -0.178534
6 -0.440738 -1.184192 -0.220445
1 -0.308708 -2.253409 -0.354382
7 -1.668608 -0.714462 -0.111934
7 -0.831941 1.562526 0.129147
8 -3.054244 1.072168 0.240051
8 1.365431 1.988490 0.002444
1 2.224782 1.555197 -0.224600
6 2.063714 -0.885910 -0.154640
8 2.901104 0.013979 -0.836789
1 3.816606 -0.310794 -0.806193
8 2.421583 -0.934101 1.217028
1 2.451235 -1.860170 1.508108
1 2.092295 -1.884882 -0.598309
6 -2.852420 -1.585406 -0.198628
1 -3.418066 -1.497815 0.729990
1 -3.468351 -1.247068 -1.033628
1 -2.521479 -2.610322 -0.354012
rc_1m5dhmU/rc_1m5dhmU53.xyz
6 -1.904402 0.705390 0.000004
6 0.377387 1.062606 -0.000010
6 0.667022 -0.359232 -0.000005
6 -0.447185 -1.218093 -0.000004
1 -0.322486 -2.296249 -0.000006
7 -1.675853 -0.733523 -0.000002
7 -0.831725 1.551402 -0.000005

8 -3.064314 1.087881 0.000017
8 1.435628 1.855829 -0.000018
1 1.174548 2.796597 -0.000016
6 2.072687 -0.928526 0.000008
8 2.752126 -0.566960 -1.180054
1 2.850938 0.399856 -1.225826
8 2.752104 -0.566938 1.180077
1 2.850914 0.399879 1.225832
1 2.015948 -2.016897 0.000017
6 -2.862035 -1.606221 -0.000009
1 -3.454398 -1.389464 0.890417
1 -3.454376 -1.389478 -0.890453
1 -2.533242 -2.643441 0.000003
rc_1m5dhmU/rc_1m5dhmU35.xyz
6 -1.914633 0.693893 0.114896
6 0.354289 1.146364 0.009289
6 0.671372 -0.262110 -0.164869
6 -0.414192 -1.155486 -0.245782
1 -0.253034 -2.216872 -0.409104
7 -1.652814 -0.721814 -0.125704
7 -0.876049 1.572366 0.157961
8 -3.084789 1.021391 0.257406
8 1.315953 2.048018 0.040222
1 2.184293 1.606357 -0.167062
6 2.079753 -0.821741 -0.199891
8 3.025985 0.177637 -0.555433
1 3.218513 0.119510 -1.506609
8 2.350665 -1.277254 1.099015
1 3.050245 -1.950076 1.051179
1 2.117021 -1.638050 -0.932247
6 -2.811748 -1.624077 -0.222855
1 -3.374929 -1.569879 0.709780
1 -3.441460 -1.289012 -1.048864
1 -2.450157 -2.635238 -0.397286
rc_1m5dhmU/rc_1m5dhmU41.xyz
6 1.909735 0.695215 0.026629

6 -0.371109 1.074472 0.006742
6 -0.668790 -0.345564 -0.013169
6 0.437348 -1.213821 -0.038862
1 0.302284 -2.290125 -0.074258
7 1.670167 -0.741000 -0.017306
7 0.844926 1.549406 0.032332
8 3.073283 1.066963 0.053715
8 -1.415161 1.883028 -0.002729
1 -1.131183 2.817342 0.001100
6 -2.074527 -0.904443 -0.023528
8 -2.688202 -0.499658 1.177095
1 -3.362850 -1.154927 1.417820
8 -2.765296 -0.467725 -1.173495
1 -2.900614 0.494735 -1.121450
1 -2.028256 -1.992746 -0.101430
6 2.848058 -1.624198 -0.043393
1 3.440468 -1.388323 -0.929078
1 3.444287 -1.437910 0.851118
1 2.508945 -2.657718 -0.072205
rc_1m5dhmU/rc_1m5dhmU52.xyz
6 1.950041 -0.651255 0.014042
6 -0.284736 -1.246844 -0.051605
6 -0.703804 0.148175 -0.103106
6 0.314542 1.118640 -0.047919
1 0.079536 2.176987 -0.067424
7 1.582026 0.759377 0.020587
7 0.975182 -1.601638 -0.013928
8 3.145983 -0.908196 0.042238
8 -1.180493 -2.216146 -0.059834
1 -2.091753 -1.834643 0.018666
6 -2.151777 0.549225 -0.348450
8 -3.040050 -0.346517 0.304251
1 -2.964018 -0.234972 1.269269
8 -2.408647 1.893478 -0.061546
1 -2.281222 2.064733 0.887774
1 -2.338491 0.417277 -1.421656

6 2.670147 1.748129 0.072698
1 3.271414 1.560088 0.963311
1 3.291177 1.625835 -0.816344
1 2.232072 2.743152 0.104958
rc_1m5fC/rc_1m5fC01.xyz
6 -1.525740 0.801570 -0.021873
6 0.794787 0.946450 0.020713
6 0.935421 -0.515643 0.007329
6 -0.254773 -1.276446 0.021451
1 -0.222082 -2.362110 0.035346
7 -1.426435 -0.684827 0.015486
7 -0.399907 1.530203 -0.005807
8 -2.665242 1.248012 -0.062375
7 1.886967 1.695310 0.052595
1 1.798383 2.706663 0.058104
1 2.807624 1.264500 0.059409
6 2.212161 -1.261416 -0.035006
1 2.118984 -2.357524 -0.064688
8 3.306817 -0.708931 -0.037327
6 -2.679546 -1.444800 0.033941
1 -3.255246 -1.140596 0.910629
1 -3.248223 -1.198809 -0.865457
1 -2.450281 -2.507858 0.069022
rc_1m5fC/rc_1m5fC02.xyz
6 -1.588312 0.652839 0.043483
6 0.659266 1.247092 -0.024018
6 1.080809 -0.156179 0.025378
6 0.057448 -1.134376 -0.028483
1 0.304621 -2.190697 -0.051935
7 -1.204009 -0.779383 -0.036362
7 -0.624725 1.587487 0.020178
8 -2.790677 0.871841 0.117879
7 1.567579 2.211312 -0.117310
1 1.255351 3.177356 -0.148676
1 2.562746 2.033915 -0.170780
6 2.482091 -0.629257 0.131880

1 3.268770 0.106553 0.344380
8 2.750272 -1.816738 -0.010456
6 -2.288585 -1.766977 -0.084076
1 -2.862298 -1.696783 0.842255
1 -2.939529 -1.527988 -0.926660
1 -1.854649 -2.757940 -0.198507
rc_1m5fU/rc_1m5fU11.xyz
6 -1.629387 0.547277 0.003905
6 0.710532 1.312219 -0.003720
6 1.124993 -0.099020 -0.001128
6 0.141846 -1.115737 -0.002845
1 0.431137 -2.161678 -0.002113
7 -1.142889 -0.827970 -0.006042
7 -0.660413 1.512379 -0.000574
8 -2.820178 0.759212 0.014097
8 1.501947 2.248147 -0.007739
1 -0.992929 2.477116 0.002414
6 2.559043 -0.457371 0.002425
1 3.272594 0.376211 -0.001195
8 2.915245 -1.630425 0.009860
6 -2.174039 -1.878399 -0.008274
1 -2.760953 -1.784967 0.907971
1 -2.822256 -1.721997 -0.872187
1 -1.678519 -2.844831 -0.060491
rc_1m5fU/rc_1m5fU12.xyz
6 -1.570335 0.710932 0.000188
6 0.882443 1.007107 0.000102
6 1.014235 -0.466485 -0.000054
6 -0.155292 -1.262445 -0.000058
1 -0.082514 -2.347141 -0.000191
7 -1.362572 -0.733132 0.000049
7 -0.430878 1.462003 0.000162
8 -2.696369 1.153582 0.000285
8 1.822225 1.788478 0.000116
1 -0.568982 2.472810 0.000262
6 2.315349 -1.175607 -0.000376

1 2.242175 -2.274226 -0.000765
8 3.396180 -0.604631 -0.000251
6 -2.583312 -1.553610 -0.000077
1 -3.165029 -1.304372 0.890170
1 -3.164958 -1.304167 -0.890314
1 -2.301358 -2.603793 -0.000189
rc_1m5fU/rc_1m5fU10.xyz
6 -1.502200 -0.794886 -0.000314
6 0.808624 -0.915438 -0.000077
6 0.918954 0.535597 0.000168
6 -0.281324 1.283114 0.000224
1 -0.264367 2.369099 0.000437
7 -1.443109 0.669603 0.000016
7 -0.351864 -1.523633 -0.000316
8 -2.616472 -1.295623 -0.000483
8 1.887925 -1.668300 -0.000081
1 2.698212 -1.100484 0.000063
6 2.215429 1.237771 0.000331
1 2.189510 2.336130 0.000485
8 3.274258 0.611067 0.000303
6 -2.718650 1.402749 0.000125
1 -3.281060 1.116670 -0.890512
1 -3.281042 1.116414 0.890694
1 -2.507120 2.469788 0.000276
rc_1m5fU/rc_1m5fU08.xyz
6 -1.580777 0.633704 0.000003
6 0.653069 1.230745 -0.000002
6 1.075515 -0.157167 0.000000
6 0.055954 -1.140168 -0.000003
1 0.304192 -2.196765 -0.000005
7 -1.207324 -0.784612 -0.000003
7 -0.604424 1.588510 0.000000
8 -2.773725 0.885119 0.000009
8 1.618088 2.127323 -0.000006
1 1.250216 3.032409 -0.000006
6 2.495981 -0.574685 0.000004

1 3.250167 0.222380 0.000005
8 2.798277 -1.762451 0.000005
6 -2.297776 -1.773215 -0.000005
1 -2.907806 -1.616039 0.890877
1 -2.907818 -1.616021 -0.890876
1 -1.859624 -2.768454 -0.000019
rc_1m5fU/rc_1m5fU06.xyz
6 -1.375804 0.703783 -0.000008
6 0.902330 0.848432 0.000007
6 0.970802 -0.593742 0.000002
6 -0.197559 -1.321713 -0.000003
1 -0.213980 -2.405486 -0.000017
7 -1.379183 -0.679607 0.000000
7 -0.304664 1.465094 0.000005
8 -2.581688 1.224907 -0.000003
8 1.983358 1.513038 0.000011
1 -2.531815 2.199957 -0.000077
6 2.296606 -1.121973 -0.000007
1 2.474672 -2.207782 -0.000019
8 3.269655 -0.350645 -0.000004
6 -2.665362 -1.415965 0.000010
1 -3.228173 -1.147781 0.894173
1 -3.228184 -1.147791 -0.894148
1 -2.442261 -2.480859 0.000017
rc_1m5fU/rc_1m5fU09.xyz
6 1.518684 0.768964 -0.029924
6 -0.793969 0.945358 0.029123
6 -0.951060 -0.501275 0.015993
6 0.239059 -1.268658 0.031347
1 0.198201 -2.353853 0.052037
7 1.417981 -0.689772 0.017419
7 0.381426 1.520075 -0.011338
8 2.642080 1.242918 -0.079848
8 -1.890801 1.668960 0.072793
1 -1.671178 2.621343 0.079644
6 -2.233610 -1.242817 -0.036231

1 -2.129487 -2.339212 -0.055494
8 -3.328628 -0.700030 -0.061176
6 2.672161 -1.458240 0.038859
1 3.242251 -1.216090 -0.859859
1 3.242861 -1.160203 0.920345
1 2.432693 -2.518885 0.071612
rc_1m5fU/rc_1m5fU07.xyz
6 -1.580441 0.637217 0.000020
6 0.657021 1.249959 -0.000014
6 1.077250 -0.142349 -0.000005
6 0.053264 -1.124752 -0.000026
1 0.305006 -2.180484 -0.000046
7 -1.210529 -0.776631 -0.000024
7 -0.607512 1.591345 -0.000001
8 -2.774698 0.892225 0.000074
8 1.515372 2.254796 -0.000046
1 2.450030 1.987154 -0.000006
6 2.487082 -0.601526 0.000022
1 3.276203 0.164071 0.000018
8 2.758947 -1.795127 0.000047
6 -2.298682 -1.768626 -0.000041
1 -2.908567 -1.612987 0.891066
1 -2.908635 -1.612885 -0.891083
1 -1.857692 -2.762558 -0.000116
rc_1m5fU/rc_1m5fU04.xyz
6 -1.374059 0.724906 -0.000002
6 0.908168 0.848283 -0.000008
6 0.971267 -0.593330 -0.000007
6 -0.200528 -1.310888 -0.000015
1 -0.224051 -2.394410 -0.000013
7 -1.382934 -0.662096 -0.000017
7 -0.290969 1.471152 -0.000002
8 -2.515346 1.380659 0.000012
8 1.994594 1.507802 -0.000009
1 -3.317314 0.828909 -0.000006
6 2.295967 -1.128978 0.000000

1 2.469361 -2.215389 -0.000029
8 3.270219 -0.360424 0.000026
6 -2.657261 -1.418813 0.000012
1 -3.224017 -1.176705 0.901257
1 -3.224038 -1.176750 -0.901231
1 -2.419668 -2.480419 0.000040
rc_1m5fU/rc_1m5fU05.xyz
6 -1.505842 0.543881 0.004401
6 0.664773 1.315085 -0.005469
6 1.145537 -0.069296 0.000198
6 0.193752 -1.106807 -0.004484
1 0.485854 -2.150997 -0.005738
7 -1.098825 -0.821954 -0.007898
7 -0.695874 1.549819 0.001875
8 -2.810545 0.655414 0.019214
8 1.470239 2.260390 -0.016913
1 -3.075912 1.595529 0.030597
6 2.592168 -0.389231 0.007758
1 3.280334 0.465630 0.009394
8 2.984944 -1.549625 0.013033
6 -2.122414 -1.885570 -0.014041
1 -2.709825 -1.801818 0.902520
1 -2.769269 -1.736270 -0.879997
1 -1.613236 -2.844930 -0.067480
rc_1m5fU/rc_1m5fU03.xyz
6 1.588045 0.713163 -0.000175
6 -0.811832 1.014114 -0.000135
6 -0.961613 -0.440883 0.000068
6 0.198310 -1.225905 0.000146
1 0.136664 -2.310313 0.000310
7 1.415347 -0.694354 0.000006
7 0.481271 1.554160 -0.000133
8 2.717758 1.191844 -0.000327
8 -1.750950 1.823088 -0.000322
1 -4.071844 -1.107255 0.000506
6 -2.182587 -1.118401 0.000273

1 -2.199377 -2.208051 0.000455
8 -3.314497 -0.490741 0.000296
6 2.642118 -1.517669 0.000142
1 3.224577 -1.282877 -0.891735
1 3.224547 -1.282607 0.891970
1 2.355972 -2.567581 0.000295
rc_1m5hmC/rc_1m5hmC03.xyz
6 1.703516 0.566288 0.009135
6 -0.497573 1.317972 -0.006296
6 -1.019458 -0.049922 -0.002728
6 -0.068127 -1.085177 -0.010630
1 -0.373773 -2.123714 -0.015538
7 1.225559 -0.818900 -0.012555
7 0.808120 1.569967 0.004239
8 2.923663 0.718797 0.029877
6 -2.474009 -0.341583 0.008542
1 -2.919395 0.145093 0.893803
1 -2.932803 0.139930 -0.872897
7 -1.343092 2.343934 -0.016970
1 -0.973213 3.288480 -0.017827
1 -2.348107 2.226835 -0.031246
8 -2.709911 -1.736592 0.014611
1 -3.667896 -1.880635 0.038790
6 2.230451 -1.890430 -0.017797
1 2.888483 -1.748579 -0.876154
1 2.816486 -1.824836 0.900828
1 1.717294 -2.848113 -0.080012
rc_1m5hmC/rc_1m5hmC01.xyz
6 1.702218 0.569738 0.008041
6 -0.497778 1.320960 -0.000495
6 -1.022305 -0.046210 -0.006012
6 -0.071600 -1.081487 -0.013924
1 -0.380744 -2.119636 -0.023915
7 1.222121 -0.816705 -0.011727
7 0.807792 1.574028 0.008352
8 2.922431 0.719732 0.022865

6 -2.488522 -0.344709 -0.008633
1 -2.946984 0.132094 0.872511
1 -2.937778 0.133325 -0.890668
7 -1.345511 2.345043 -0.004473
1 -0.978439 3.290855 -0.002292
1 -2.350365 2.225038 -0.009467
8 -2.797576 -1.723028 -0.074705
1 -2.697137 -2.115881 0.806037
6 2.226504 -1.888493 -0.017160
1 2.879354 -1.751419 -0.880351
1 2.818381 -1.816861 0.897248
1 1.712954 -2.846500 -0.070355
rc_1m5hmC/rc_1m5hmC02.xyz
6 1.620326 0.780375 0.014998
6 -0.697701 0.962816 -0.052757
6 -0.871297 -0.492345 -0.093933
6 0.308454 -1.259616 -0.054651
1 0.266593 -2.344436 -0.065225
7 1.494327 -0.686289 0.005770
7 0.506704 1.530442 -0.006536
8 2.768184 1.219478 0.050155
6 -2.180801 -1.208857 -0.263040
1 -2.360419 -1.307452 -1.344143
1 -2.088572 -2.225053 0.139248
7 -1.775584 1.737415 -0.081142
1 -1.665954 2.745923 -0.075217
1 -2.701927 1.317240 -0.049998
8 -3.307634 -0.537211 0.282276
1 -3.275981 -0.609771 1.249695
6 2.732000 -1.472784 0.078025
1 3.352982 -1.233515 -0.786987
1 3.267138 -1.198806 0.988794
1 2.477717 -2.530765 0.085896
rc_1m5hmC/rc_1m5hmC04.xyz
6 -1.627224 0.776400 0.001955
6 0.693396 0.965023 -0.024034

6 0.871607 -0.488790 -0.036322
6 -0.308866 -1.256651 -0.013886
1 -0.264443 -2.341335 -0.012311
7 -1.496832 -0.686745 0.008657
7 -0.514540 1.527978 -0.008309
8 -2.776523 1.215461 0.011632
6 2.159263 -1.227164 -0.170861
1 2.080980 -2.181508 0.370643
1 2.262050 -1.486612 -1.241072
7 1.766573 1.747536 -0.042329
1 1.647398 2.754838 -0.054879
1 2.695333 1.333001 -0.008626
8 3.275917 -0.477928 0.276515
1 4.078799 -0.870757 -0.098096
6 -2.734305 -1.476980 0.051135
1 -3.291672 -1.207198 0.949719
1 -3.335226 -1.238522 -0.827952
1 -2.477987 -2.534587 0.063350
rc_1m5hmU/rc_1m5hmU01.xyz
6 -1.640738 0.715520 0.036441
6 0.806014 0.971296 -0.056835
6 0.927751 -0.491519 -0.034975
6 -0.249989 -1.260418 -0.040962
1 -0.198894 -2.344683 -0.067410
7 -1.455700 -0.711510 -0.001997
7 -0.488240 1.455407 0.013396
8 -2.757634 1.189840 0.082749
8 1.770647 1.733364 -0.136983
1 -0.609965 2.467810 0.016705
6 2.239989 -1.184700 -0.123448
1 2.198286 -2.129006 0.433405
1 2.332569 -1.482656 -1.192353
8 3.382757 -0.476048 0.296839
1 3.285200 0.461606 0.050261
6 -2.686756 -1.522135 0.005926
1 -3.253123 -1.289267 0.909026

1 -3.279485 -1.262669 -0.872994
1 -2.410797 -2.573932 -0.014172
rc_1m5hmU/rc_1m5hmU03.xyz
6 -1.590716 0.747399 0.074001
6 0.697379 0.987582 -0.137287
6 0.901736 -0.443564 -0.267211
6 -0.254950 -1.242928 -0.191479
1 -0.190515 -2.323985 -0.259529
7 -1.447759 -0.697254 -0.035943
7 -0.478524 1.537565 0.014358
8 -2.722913 1.184972 0.219808
8 1.791759 1.725192 -0.195074
6 2.269453 -1.042736 -0.416192
1 2.188401 -2.118755 -0.577171
1 2.784478 -0.592634 -1.271910
8 2.996980 -0.842783 0.801230
1 3.384194 0.046967 0.794383
6 -2.671585 -1.509081 0.058816
1 -3.135050 -1.326257 1.029853
1 -3.356932 -1.203347 -0.732977
1 -2.403640 -2.557902 -0.048739
1 1.578541 2.674669 -0.114414
rc_1m5hmU/rc_1m5hmU04.xyz
6 -1.506764 0.691337 -0.000002
6 0.779704 0.975210 0.000003
6 0.952936 -0.481106 -0.000001
6 -0.205667 -1.271206 0.000000
1 -0.155843 -2.355140 0.000002
7 -1.413484 -0.714441 0.000003
7 -0.492760 1.496439 0.000000
8 -2.754279 1.105940 -0.000010
8 1.774236 1.730274 0.000010
6 2.266162 -1.163318 -0.000006
1 2.288289 -1.851068 0.869508
1 2.288284 -1.851060 -0.869526
8 3.422763 -0.366703 -0.000004

1 3.150253 0.576707 0.000005
6 -2.647496 -1.529565 0.000007
1 -3.226351 -1.289960 0.892929
1 -3.226343 -1.289981 -0.892927
1 -2.363716 -2.579695 0.000021
1 -2.785882 2.082010 -0.000012
rc_1m5hmU/rc_1m5hmU02.xyz
6 -1.594510 0.720235 0.116980
6 0.679837 1.073519 -0.151475
6 0.938050 -0.347839 -0.321676
6 -0.191032 -1.192596 -0.264304
1 -0.082775 -2.266313 -0.381205
7 -1.403387 -0.707536 -0.079755
7 -0.519799 1.554627 0.051884
8 -2.735593 1.106425 0.331997
8 1.650487 1.975444 -0.203586
6 2.311875 -0.953129 -0.432417
1 2.239029 -1.964574 -0.833451
1 2.969361 -0.368141 -1.083545
8 2.832528 -1.057970 0.899682
1 3.178715 -0.194051 1.176996
6 -2.595289 -1.570837 -0.025818
1 -3.020126 -1.513223 0.977869
1 -3.322939 -1.205376 -0.751164
1 -2.299366 -2.591532 -0.260234
1 2.527438 1.606262 -0.402662
rc_1m5hmU/rc_1m5hmU05.xyz
6 -1.507073 0.713961 -0.000003
6 0.785983 0.976053 0.000006
6 0.954437 -0.479579 0.000004
6 -0.207949 -1.260115 0.000005
1 -0.165490 -2.344446 0.000006
7 -1.415616 -0.697116 0.000008
7 -0.480722 1.504460 -0.000001
8 -2.704108 1.265099 -0.000020
8 1.786245 1.725609 0.000014

6 2.263091 -1.168942 -0.000015
1 2.281641 -1.858239 0.868845
1 2.281630 -1.858189 -0.868917
8 3.423578 -0.379360 -0.000001
1 3.155969 0.565818 0.000003
6 -2.636864 -1.529237 0.000007
1 -3.218265 -1.315102 0.900204
1 -3.218254 -1.315111 -0.900199
1 -2.342223 -2.576074 0.000019
1 -3.456105 0.646301 0.000019
rc_1m5mC/rc_1m5mC01.xyz
6 1.155478 -0.874479 0.000120
6 -1.163564 -0.741952 -0.000053
6 -1.144546 0.724339 0.000115
6 0.131164 1.322503 0.000076
1 0.238000 2.402251 0.000101
7 1.230430 0.591274 -0.000043
7 -0.049858 -1.469818 0.000029
8 2.231478 -1.470045 0.000299
7 -2.335209 -1.369067 -0.000282
1 -2.355694 -2.383060 -0.000407
1 -3.215868 -0.869861 -0.000293
6 -2.374107 1.546021 0.000191
1 -2.988600 1.312175 -0.880932
1 -2.988737 1.311871 0.881132
1 -2.140219 2.611374 0.000374
6 2.566126 1.203411 -0.000310
1 3.103500 0.872777 0.889973
1 3.103493 0.871903 -0.890265
1 2.451462 2.285143 -0.000842
rc_1m5mU/rc_1m5mU01.xyz
6 1.150762 -0.855335 -0.000007
6 -1.156234 -0.734553 -0.000054
6 -1.154510 0.719563 -0.000016
6 0.120452 1.325111 0.000038
1 0.224493 2.405039 0.000070

7 1.221893 0.595445 0.000052
7 -0.071458 -1.462842 -0.000060
8 2.209187 -1.469019 -0.000016
8 -2.351182 -1.300079 -0.000054
6 -2.401529 1.503783 -0.000017
1 -3.009401 1.235174 -0.875589
1 -3.009373 1.235206 0.875587
1 -2.203003 2.575887 -0.000034
6 2.560691 1.208434 0.000104
1 3.095674 0.877647 0.891476
1 3.095695 0.877754 -0.891296
1 2.442682 2.289496 0.000166
1 -2.271646 -2.273654 -0.000052
rc_1m5mU/rc_1m5mU04.xyz
6 1.150762 -0.855335 -0.000007
6 -1.156234 -0.734553 -0.000054
6 -1.154510 0.719563 -0.000016
6 0.120452 1.325111 0.000038
1 0.224493 2.405039 0.000070
7 1.221893 0.595445 0.000052
7 -0.071458 -1.462842 -0.000060
8 2.209187 -1.469019 -0.000016
8 -2.351182 -1.300079 -0.000054
6 -2.401529 1.503783 -0.000017
1 -3.009401 1.235174 -0.875589
1 -3.009373 1.235206 0.875587
1 -2.203003 2.575887 -0.000034
6 2.560691 1.208434 0.000104
1 3.095674 0.877647 0.891476
1 3.095695 0.877754 -0.891296
1 2.442682 2.289496 0.000166
1 -2.271646 -2.273654 -0.000052
rc_1m5mU/rc_1m5mU05.xyz
6 1.159627 -0.853226 -0.000011
6 -1.152597 -0.757173 -0.000044
6 -1.157065 0.700016 -0.000011

6 0.115825 1.313739 0.000040
1 0.209507 2.394680 0.000066
7 1.223348 0.596415 0.000052
7 -0.055593 -1.468895 -0.000057
8 2.222889 -1.460601 -0.000012
8 -2.282269 -1.452250 -0.000062
6 -2.396036 1.501456 -0.000019
1 -3.008073 1.250613 -0.879736
1 -3.008038 1.250669 0.879739
1 -2.183324 2.570795 -0.000052
6 2.556708 1.221117 0.000099
1 3.094380 0.895019 0.891545
1 3.094398 0.895113 -0.891371
1 2.428566 2.301062 0.000153
1 -3.085439 -0.903358 -0.000043
rc_1m5mU/rc_1m5mU03.xyz
6 1.093397 -0.740681 -0.002524
6 -1.211758 -0.857497 0.000841
6 -1.282987 0.618662 -0.013501
6 -0.063190 1.320099 -0.008533
1 -0.028681 2.403486 -0.014450
7 1.098954 0.675410 0.002689
7 0.029182 -1.471238 -0.001063
8 2.314466 -1.229933 -0.010064
8 -2.249130 -1.533092 0.014801
6 -2.580373 1.317112 -0.007777
1 -3.249111 0.882004 -0.759378
1 -3.074129 1.152780 0.962474
1 -2.465013 2.389624 -0.171163
6 2.385214 1.400518 0.014505
1 2.944606 1.104204 0.903061
1 2.946776 1.132389 -0.881885
1 2.173561 2.467210 0.031154
1 2.290534 -2.205993 -0.017155
rc_1m5mU/rc_1m5mU02.xyz
6 -1.090619 -0.762422 -0.004368

6 1.219742 -0.853343 0.000822
6 1.283312 0.621284 -0.016542
6 0.057774 1.310387 -0.014720
1 0.015495 2.393444 -0.023256
7 -1.103430 0.658967 -0.000284
7 -0.013415 -1.474332 -0.001813
8 -2.246466 -1.396805 -0.010683
8 2.262435 -1.523050 0.017793
6 2.574736 1.329646 -0.005973
1 3.064488 1.168704 0.967014
1 3.250479 0.897374 -0.753019
1 2.453132 2.401169 -0.170743
1 -3.042017 -0.836629 -0.019422
6 -2.379808 1.401966 0.020493
1 -2.947288 1.166682 -0.883532
1 -2.938726 1.124509 0.917491
1 -2.156222 2.466039 0.044995
rc_1mC/rc_1mC01.xyz
6 -0.582510 0.919020 0.000102
6 1.563863 0.050553 -0.000202
6 1.067045 -1.313200 -0.000061
6 -0.319409 -1.506052 0.000078
1 -0.757222 -2.498100 0.000132
7 -1.126660 -0.458975 0.000132
7 0.749720 1.102998 -0.000115
8 -1.418177 1.817863 0.000288
7 2.880567 0.231987 -0.000419
1 3.264033 1.171947 -0.000518
1 3.518024 -0.556763 -0.000487
1 1.739236 -2.163458 -0.000086
6 -2.585734 -0.605866 0.000213
1 -2.986012 -0.114940 0.889236
1 -2.986100 -0.114906 -0.888753
1 -2.831463 -1.665476 0.000204
rc_1mU/rc_1mU01.xyz
6 0.597011 -0.892061 0.000005

6 -1.697387 -0.007930 -0.000003
6 -1.129983 1.337650 0.000003
6 0.260092 1.516990 0.000002
1 0.700810 2.507944 0.000003
7 1.083505 0.477028 0.000000
7 -0.763156 -1.034465 -0.000003
8 1.386002 -1.812578 0.000003
8 -2.905222 -0.232042 -0.000001
1 -1.799255 2.189002 0.000007
6 2.545135 0.637727 -0.000005
1 2.946976 0.150919 0.890742
1 2.946974 0.150886 -0.890734
1 2.775857 1.699800 -0.000022
1 -1.109247 -1.993779 -0.000004
rc_1mU/rc_1mU02.xyz
6 0.566176 -0.903944 -0.000024
6 -1.567657 -0.023711 0.000002
6 -1.064596 1.326635 -0.000004
6 0.328103 1.501345 -0.000006
1 0.777668 2.487951 -0.000003
7 1.125073 0.446466 -0.000008
7 -0.788430 -1.075613 -0.000002
8 1.361591 -1.831683 0.000019
8 -2.883712 -0.143101 0.000009
1 -3.147839 -1.083583 0.000012
1 -1.734262 2.177955 -0.000001
6 2.590494 0.572354 0.000004
1 2.981361 0.077422 0.890564
1 2.981374 0.077437 -0.890560
1 2.847050 1.629056 0.000015
rc_1mU/rc_1mU03.xyz
6 -0.585558 -0.899292 0.000016
6 1.568895 -0.065872 -0.000010
6 1.086527 1.294222 -0.000005
6 -0.303363 1.496444 -0.000001
1 -0.731641 2.492673 -0.000005

7 -1.120693 0.458457 0.000005
7 0.763066 -1.099166 0.000000
8 -1.399068 -1.813119 0.000034
8 2.866184 -0.333791 -0.000034
1 3.418756 0.467872 -0.000048
1 1.764998 2.140036 -0.000009
6 -2.583073 0.613099 0.000003
1 -2.983950 0.126663 -0.890695
1 -2.983951 0.126690 0.890714
1 -2.818327 1.674699 -0.000013
rc_1mU/rc_1mU05.xyz
6 -0.507963 0.803295 0.000020
6 1.680917 0.093726 -0.000018
6 1.222596 -1.287689 -0.000009
6 -0.151991 -1.541800 -0.000013
1 -0.560816 -2.545642 -0.000020
7 -1.011113 -0.523594 -0.000007
7 0.746431 1.114430 0.000010
8 -1.477592 1.689770 0.000059
8 2.899074 0.350361 -0.000027
1 -1.108575 2.593680 0.000071
1 1.939259 -2.099958 -0.000010
6 -2.469125 -0.741515 -0.000021
1 -2.890438 -0.274411 0.892012
1 -2.890433 -0.274317 -0.892007
1 -2.654678 -1.812356 -0.000076
rc_1mU/rc_1mU04.xyz
6 -0.506055 -0.818266 -0.010484
6 1.684089 -0.098233 0.004319
6 1.231197 1.281356 -0.036706
6 -0.141918 1.532186 -0.026695
1 -0.551121 2.535509 -0.032783
7 -1.005763 0.514968 -0.001554
7 0.752880 -1.116593 0.004030
8 -1.372313 -1.809201 -0.025419
8 2.903861 -0.356425 0.039442

1 -2.310271 -1.555784 -0.077557
1 1.946748 2.094206 -0.060972
6 -2.459649 0.765870 0.046765
1 -2.917130 0.390378 -0.872776
1 -2.874140 0.256965 0.920966
1 -2.622269 1.837637 0.130409
rc_5caC/rc_5caC01.xyz
6 2.187780 -0.134262 -0.007521
6 0.034212 1.014116 0.058751
6 -0.636605 -0.254903 -0.107263
6 0.146351 -1.400713 -0.214744
1 -0.293890 -2.384079 -0.336184
7 1.475818 -1.326643 -0.187752
7 1.392247 0.995701 0.108635
8 3.401686 -0.100079 0.036930
7 -0.610497 2.150683 0.156326
1 -0.116161 3.029966 0.287613
1 -1.623503 2.176281 0.075268
6 -2.115732 -0.374108 -0.049325
8 -2.911244 0.315245 -0.692655
8 -2.416466 -1.260485 0.833736
1 1.903363 1.872361 0.221678
1 2.039361 -2.170942 -0.292325
rc_5caC/rc_5caC02.xyz
6 -2.219637 -0.083951 0.010907
6 -0.195535 1.061039 -0.001463
6 0.568108 -0.195566 -0.014679
6 -0.181409 -1.401362 -0.043959
1 0.287740 -2.377975 -0.069663
7 -1.486265 -1.335694 -0.040583
7 -1.529973 1.064878 0.028907
8 -3.445496 -0.191652 0.034239
7 0.446334 2.218767 -0.014969
1 -0.089786 3.080518 -0.007589
1 1.461721 2.245179 -0.034422
6 2.063749 -0.255375 0.005634

8 2.766400 0.743678 -0.019462
8 2.513344 -1.505941 0.054535
1 3.490186 -1.507967 0.066480
1 -2.046177 -2.192788 -0.061433
rc_5caC/rc_5caC03.xyz
6 -2.222549 -0.030423 0.018915
6 -0.156104 1.034662 -0.080379
6 0.556710 -0.246007 0.026875
6 -0.234854 -1.420722 0.087254
1 0.207360 -2.409824 0.137708
7 -1.536080 -1.307274 0.096856
7 -1.488719 1.090074 -0.073087
8 -3.450817 -0.088076 0.042941
7 0.528923 2.164403 -0.189810
1 0.021464 3.039693 -0.271637
1 1.541795 2.182847 -0.152994
6 2.035142 -0.452794 0.002547
8 2.501252 -1.493235 -0.431604
8 2.732793 0.579242 0.462084
1 3.691722 0.406428 0.382029
1 -2.127104 -2.141318 0.148536
rc_5caC/rc_5caC05.xyz
6 -2.210423 -0.074372 0.014047
6 -0.172192 1.034714 -0.126139
6 0.568205 -0.227350 -0.002512
6 -0.190106 -1.413687 0.129635
1 0.273218 -2.390085 0.233272
7 -1.496538 -1.330542 0.127247
7 -1.503792 1.060731 -0.115492
8 -3.437270 -0.157084 0.047459
7 0.492012 2.172493 -0.276001
1 -0.030931 3.036776 -0.376156
1 1.503407 2.223019 -0.251272
6 2.059230 -0.281097 0.109812
8 2.597901 0.402243 0.964397
8 2.754814 -1.085189 -0.680372

1 2.228265 -1.522403 -1.375811
1 -2.067576 -2.175080 0.218753
rc_5caC/rc_5caC04.xyz
6 2.208006 -0.046296 0.057041
6 0.153062 1.028006 -0.138567
6 -0.561621 -0.243899 0.007556
6 0.213457 -1.424695 0.029555
1 -0.236040 -2.412152 0.035512
7 1.517887 -1.319616 0.063390
7 1.481595 1.079614 -0.066800
8 3.432459 -0.100975 0.155387
7 -0.526465 2.150106 -0.341477
1 -0.016397 3.023109 -0.433999
1 -1.538941 2.183388 -0.377490
6 -2.047233 -0.407270 0.012090
8 -2.530051 -1.079038 -0.884053
8 -2.794228 0.173380 0.935352
1 -2.282891 0.610837 1.642202
1 2.103669 -2.157916 0.088443
rc_5caC/rc_5caC06.xyz
6 2.056177 -0.075648 -0.021802
6 0.046525 1.010745 0.084430
6 -0.655670 -0.248175 -0.069780
6 0.112036 -1.401163 -0.145596
1 -0.315616 -2.393468 -0.223218
7 1.446314 -1.303792 -0.144754
7 1.402322 1.047352 0.102566
8 3.365647 -0.149694 -0.036686
7 -0.603829 2.151479 0.199349
1 -0.084789 3.017444 0.309765
1 -1.616933 2.196834 0.162512
6 -2.142923 -0.354018 -0.078791
8 -2.902443 0.195839 -0.873917
8 -2.462330 -1.080094 0.937171
1 3.771841 0.734439 0.047165
1 2.027993 -2.139379 -0.219661

rc_5caC/rc_5caC07.xyz
6 2.084349 -0.081313 0.000001
6 0.071121 1.024958 0.000001
6 -0.635312 -0.237428 0.000000
6 0.121310 -1.381757 0.000000
1 -0.306636 -2.377329 -0.000001
7 1.467288 -1.305007 0.000001
7 1.427044 1.048403 0.000002
8 3.401439 -0.017107 0.000001
7 -0.541130 2.197416 0.000002
1 0.016148 3.044564 0.000004
1 -1.550615 2.277866 0.000003
6 -2.086175 -0.363727 -0.000003
8 -2.915948 0.590965 -0.000015
8 -2.714738 -1.480299 0.000009
1 3.851033 -0.881412 0.000001
1 2.019874 -2.162239 0.000000
rc_5caC/rc_5caC08.xyz
6 -2.198413 -0.049666 -0.010723
6 -0.108109 0.922491 0.078767
6 0.580769 -0.316130 -0.062228
6 -0.236729 -1.448710 -0.165436
1 0.161956 -2.451906 -0.266376
7 -1.552666 -1.299128 -0.154839
7 -1.391483 1.077270 0.093359
8 -3.418640 0.002843 0.012098
7 0.684778 2.149997 0.191216
1 0.085707 2.977224 0.068106
1 1.440598 2.167606 -0.512042
6 2.060236 -0.436572 -0.064021
8 2.797443 0.293596 -0.741375
8 2.442373 -1.335284 0.762527
1 1.131097 2.208362 1.120540
1 -2.159684 -2.115979 -0.242538
rc_5caU/rc_5caU03.xyz
6 2.224938 -0.097882 0.040899

6 0.067807 1.082229 -0.135901
6 -0.605063 -0.222987 0.029846
6 0.171013 -1.401522 0.108492
1 -0.280941 -2.386171 0.174621
7 1.481901 -1.318090 0.129318
7 1.455559 1.024950 -0.101285
8 3.436583 -0.124178 0.086120
8 -0.531746 2.135308 -0.298936
1 1.958495 1.907433 -0.198507
6 -2.086081 -0.359264 0.003092
8 -2.569722 -1.228316 -0.707036
8 -2.737644 0.507133 0.752439
1 -3.703457 0.374524 0.667873
1 2.048224 -2.166817 0.220531
rc_5caU/rc_5caU02.xyz
6 -2.220463 -0.146181 -0.020309
6 -0.099162 1.099653 0.132761
6 0.612433 -0.189137 -0.010054
6 -0.128245 -1.386681 -0.134851
1 0.349841 -2.355843 -0.239083
7 -1.442215 -1.342689 -0.132835
7 -1.484012 0.999730 0.108583
8 -3.431204 -0.210719 -0.042426
8 0.468873 2.171380 0.284040
1 -2.013768 1.865511 0.213302
6 2.100575 -0.206705 -0.095707
8 2.660732 -1.058602 0.746570
8 2.665278 0.491641 -0.918220
1 3.631696 -1.076372 0.624695
1 -1.984442 -2.207871 -0.219894
rc_5caU/rc_5caU04.xyz
6 2.215043 -0.128033 0.013105
6 0.075584 1.084474 -0.130510
6 -0.613290 -0.218817 -0.017729
6 0.142067 -1.404654 0.093729
1 -0.319243 -2.383835 0.179939

7 1.456615 -1.338391 0.095896
7 1.461273 1.008764 -0.107276
8 3.426605 -0.171423 0.049689
8 -0.517001 2.146974 -0.251632
1 1.976812 1.886458 -0.179951
6 -2.099558 -0.235881 0.117608
8 -2.863467 -0.773442 -0.813377
8 -2.552186 0.239140 1.144552
1 -2.365975 -1.112068 -1.582278
1 2.012515 -2.195702 0.170882
rc_5caU/rc_5caU21.xyz
6 2.211923 -0.175608 -0.000002
6 0.073242 0.992186 0.000000
6 -0.621534 -0.245611 0.000004
6 0.137437 -1.400648 0.000014
1 -0.317349 -2.385429 0.000025
7 1.473120 -1.355962 0.000012
7 1.419527 0.977274 -0.000007
8 3.425699 -0.140013 -0.000010
8 -0.592252 2.108833 0.000002
1 1.935504 1.859951 -0.000012
6 -2.077063 -0.339389 -0.000001
8 -2.725402 -1.429373 -0.000026
8 -2.880629 0.647724 0.000014
1 -0.041471 2.915470 0.000001
1 2.011436 -2.222135 0.000018
rc_5caU/rc_5caU06.xyz
6 2.212524 -0.024758 -0.005421
6 0.142430 1.023828 0.050423
6 -0.545716 -0.255198 -0.023746
6 0.258360 -1.419771 -0.066532
1 -0.172217 -2.414705 -0.101236
7 1.561864 -1.296163 -0.070458
7 1.458012 1.105778 0.057243
8 3.441000 -0.027521 -0.011921
8 -0.502959 2.169178 0.112274

1 -3.665069 0.520870 -0.188337
6 -2.025704 -0.457225 -0.000493
8 -2.517980 -1.542275 0.247997
8 -2.700405 0.663024 -0.260055
1 -1.474420 2.049776 0.057271
1 2.163949 -2.123745 -0.106941
rc_5caU/rc_5caU10.xyz
6 2.200655 -0.104090 0.023460
6 0.184429 1.025472 -0.119536
6 -0.569218 -0.207956 0.003472
6 0.173517 -1.407209 0.108522
1 -0.300748 -2.379165 0.193888
7 1.483969 -1.340993 0.110325
7 1.496485 1.056491 -0.102483
8 3.424316 -0.164026 0.063930
8 -0.514697 2.128634 -0.273812
1 0.076223 2.899982 -0.377310
6 -2.058012 -0.235471 0.095961
8 -2.596320 -1.199986 -0.637237
8 -2.651665 0.542866 0.820394
1 -3.566550 -1.220228 -0.513591
1 2.046599 -2.193456 0.184651
rc_5caU/rc_5caU08.xyz
6 -2.207414 -0.044468 0.000003
6 -0.149417 1.010326 0.000000
6 0.565270 -0.252554 -0.000002
6 -0.232509 -1.424424 -0.000002
1 0.203827 -2.417106 -0.000004
7 -1.536852 -1.308753 0.000000
7 -1.457012 1.095839 0.000003
8 -3.432183 -0.058957 0.000005
8 0.599974 2.096874 -0.000002
1 0.075778 2.921925 -0.000001
6 2.056495 -0.445497 -0.000001
8 2.511672 -1.579048 -0.000001
8 2.851982 0.616663 -0.000001

1 2.362289 1.464419 -0.000001
1 -2.130956 -2.143386 -0.000001
rc_5caU/rc_5caU24.xyz
6 -2.093162 -0.145484 -0.000001
6 0.024488 1.064467 0.000005
6 0.674851 -0.233677 0.000007
6 -0.070602 -1.374383 0.000005
1 0.364265 -2.367480 0.000004
7 -1.431155 -1.319316 0.000000
7 -1.394496 0.990007 0.000006
8 -3.386830 -0.215718 -0.000009
8 0.569800 2.158875 0.000003
1 -3.842541 0.649162 0.000000
6 2.134972 -0.312946 -0.000004
8 2.815623 -1.373293 0.000004
8 2.890689 0.721773 -0.000012
1 -1.897445 1.880558 0.000004
1 -1.982251 -2.178045 -0.000007
rc_5caU/rc_5caU13.xyz
6 2.197613 -0.080071 0.007134
6 0.161274 1.009918 -0.113579
6 -0.568703 -0.238784 0.000920
6 0.193030 -1.420424 0.128631
1 -0.260752 -2.400973 0.232229
7 1.503027 -1.327643 0.122664
7 1.470501 1.068420 -0.114285
8 3.422086 -0.119707 0.022860
8 -0.572635 2.095081 -0.237966
1 -0.010664 2.889451 -0.327109
6 -2.059294 -0.269921 0.112444
8 -2.779129 -0.893705 -0.803309
8 -2.560510 0.260815 1.087324
1 -2.246914 -1.250318 -1.539597
1 2.081630 -2.167782 0.211242
rc_5caU/rc_5caU23.xyz
6 -2.077127 -0.111420 -0.000001

6 -0.086443 0.990605 0.000003
6 0.638002 -0.240090 0.000002
6 -0.107565 -1.396526 -0.000001
1 0.328353 -2.388609 -0.000001
7 -1.450932 -1.320999 -0.000004
7 -1.420968 1.031947 0.000002
8 -3.384163 -0.182832 -0.000002
8 0.589212 2.116786 0.000005
1 -3.792786 0.703990 0.000004
6 2.098494 -0.328408 0.000002
8 2.753756 -1.412679 0.000010
8 2.891259 0.666813 -0.000016
1 -0.009167 2.889585 0.000005
1 -2.015778 -2.171278 -0.000006
rc_5caU/rc_5caU09.xyz
6 2.214224 -0.052304 -0.000048
6 0.160337 1.021316 -0.000041
6 -0.553129 -0.251023 0.000041
6 0.240809 -1.421569 0.000104
1 -0.164062 -2.427808 0.000165
7 1.548097 -1.311851 0.000093
7 1.480246 1.088519 -0.000127
8 3.445895 -0.074179 -0.000093
8 -0.481298 2.162763 -0.000053
1 -1.462085 1.979993 0.000001
6 -2.052643 -0.259823 0.000036
8 -2.750361 -1.382476 0.000010
8 -2.653909 0.813453 0.000057
1 -2.250651 -2.215770 0.000000
1 2.138193 -2.149162 0.000148
rc_5caU/rc_5caU12.xyz
6 2.082213 -0.091575 0.000002
6 0.083640 0.999764 0.000002
6 -0.627796 -0.243569 -0.000004
6 0.128170 -1.394983 -0.000021
1 -0.301017 -2.390426 -0.000038

7 1.468236 -1.309279 -0.000019
7 1.418885 1.045058 0.000008
8 3.390280 -0.154633 0.000015
8 -0.495713 2.181313 -0.000008
1 3.792501 0.735060 0.000028
6 -2.084750 -0.334362 0.000005
8 -2.740355 -1.436034 0.000036
8 -2.874074 0.648822 -0.000021
1 -1.471752 2.132250 0.000040
1 2.040464 -2.154725 -0.000031
rc_5caU/rc_5caU07.xyz
6 2.095835 -0.015329 0.000006
6 0.072507 1.062273 0.000009
6 -0.626423 -0.235709 -0.000008
6 0.163134 -1.407805 -0.000014
1 -0.256703 -2.407542 -0.000023
7 1.471702 -1.278201 -0.000010
7 1.446491 1.103217 0.000022
8 3.397227 -0.135314 0.000003
8 -0.594930 2.115077 0.000010
1 3.839038 0.736810 0.000005
6 -2.117357 -0.391027 -0.000005
8 -2.611324 -1.510067 0.000013
8 -2.856657 0.707792 -0.000020
1 -2.280917 1.512457 -0.000020
1 2.070532 -2.111164 -0.000016
rc_5caU/rc_5caU15.xyz
6 -2.096774 -0.025833 0.035600
6 -0.077399 1.069799 -0.159925
6 0.618541 -0.218975 0.017429
6 -0.152936 -1.395217 0.083370
1 0.276208 -2.390292 0.128738
7 -1.466134 -1.279817 0.108910
7 -1.460092 1.088497 -0.110143
8 -3.399562 -0.147040 0.119992
8 0.567626 2.107882 -0.353173

1 -3.839822 0.723800 0.073537
6 2.101136 -0.342497 0.014714
8 2.611645 -1.153900 -0.742829
8 2.726769 0.472966 0.840955
1 3.695727 0.358154 0.767266
1 -2.055768 -2.115348 0.172408
rc_5caU/rc_5caU17.xyz
6 -2.200217 -0.062460 0.056428
6 -0.159234 1.033729 -0.151987
6 0.564697 -0.219676 0.007231
6 -0.208087 -1.404543 0.060453
1 0.250641 -2.387205 0.091257
7 -1.514485 -1.316252 0.092042
7 -1.470216 1.080557 -0.076248
8 -3.423403 -0.087646 0.137932
8 0.432074 2.196621 -0.353663
1 1.399503 2.170395 -0.454343
6 2.050958 -0.409735 0.017618
8 2.509355 -1.244512 -0.743947
8 2.821603 0.322898 0.804780
1 2.328963 0.904109 1.415040
1 -2.091923 -2.160210 0.138212
rc_5caU/rc_5caU25.xyz
6 2.083019 -0.081888 -0.000002
6 0.075922 0.996137 0.000005
6 -0.638083 -0.241557 0.000001
6 0.119309 -1.387657 -0.000001
1 -0.303511 -2.385416 -0.000001
7 1.465011 -1.298677 -0.000005
7 1.406791 1.051606 0.000001
8 3.390483 0.001304 -0.000004
8 -0.612582 2.115723 0.000013
1 3.858210 -0.854579 0.000008
6 -2.097929 -0.344979 0.000001
8 -2.741657 -1.435844 0.000020
8 -2.900167 0.642523 -0.000029

1 -0.021798 2.894309 0.000018
1 2.022441 -2.154793 -0.000012
rc_5caU/rc_5caU14.xyz
6 2.097222 -0.063520 0.010610
6 0.105977 1.085045 -0.138980
6 -0.625371 -0.186465 0.012873
6 0.116169 -1.379843 0.120143
1 -0.335427 -2.361708 0.214330
7 1.432862 -1.298361 0.111007
7 1.488022 1.068543 -0.114908
8 3.397947 -0.220828 0.059077
8 -0.514579 2.143097 -0.310087
1 3.859596 0.637985 -0.004430
6 -2.113276 -0.204313 0.114051
8 -2.688006 -0.974650 -0.796646
8 -2.670061 0.423092 0.996676
1 -3.658268 -0.990129 -0.669399
1 2.001176 -2.148533 0.182459
rc_5caU/rc_5caU20.xyz
6 -2.091120 -0.024705 0.023915
6 -0.072038 1.072362 -0.153659
6 0.625095 -0.213985 0.036775
6 -0.147198 -1.388638 0.139423
1 0.282776 -2.381089 0.221562
7 -1.460343 -1.273414 0.153727
7 -1.453621 1.087150 -0.139079
8 -3.395166 -0.144875 0.080325
8 0.578728 2.113547 -0.313404
1 -3.834625 0.723433 -0.008308
6 2.108438 -0.353414 0.000419
8 2.895050 0.420565 0.722447
8 2.552297 -1.206610 -0.754935
1 2.412931 1.057317 1.282064
1 -2.049673 -2.106548 0.245445
rc_5caU/rc_5caU16.xyz
6 -2.088008 -0.062122 0.000003

6 -0.073286 1.005052 0.000002
6 0.627944 -0.244932 -0.000001
6 -0.139645 -1.385907 -0.000016
1 0.276394 -2.386921 -0.000029
7 -1.481997 -1.286988 -0.000015
7 -1.404644 1.064381 0.000008
8 -3.395554 0.029559 0.000012
8 0.519765 2.181075 -0.000008
1 -3.868365 -0.823493 0.000005
6 2.084336 -0.350243 0.000003
8 2.727965 -1.458789 0.000026
8 2.883093 0.625051 -0.000015
1 1.494950 2.120848 0.000029
1 -2.046694 -2.138438 -0.000025
rc_5caU/rc_5caU19.xyz
6 2.104092 -0.000539 0.032535
6 0.067231 1.071061 -0.156334
6 -0.619683 -0.219801 0.021444
6 0.162795 -1.385714 0.088221
1 -0.254823 -2.385673 0.135323
7 1.478067 -1.260254 0.110829
7 1.445874 1.103346 -0.112683
8 3.414286 0.039671 0.100067
8 -0.588294 2.106134 -0.346049
1 3.861711 -0.821991 0.191715
6 -2.101293 -0.355796 0.013557
8 -2.601547 -1.169170 -0.748726
8 -2.737373 0.451720 0.839315
1 -3.704968 0.329632 0.759758
1 2.055071 -2.105720 0.172800
rc_5dhmC/rc_5dhmC03.xyz
6 2.274874 -0.281512 0.050864
6 0.228107 1.017637 -0.089625
6 -0.513504 -0.229897 -0.107166
6 0.247025 -1.423043 -0.038230
1 -0.281921 -2.368846 -0.056873

7 1.559788 -1.477869 0.046391
7 1.575043 0.924726 -0.020516
8 3.499476 -0.266828 0.117574
6 -2.018026 -0.254822 -0.310461
1 -2.210109 -0.043675 -1.372027
7 -0.353603 2.193220 -0.146307
1 0.192012 3.052490 -0.140469
1 -1.371033 2.245218 -0.106960
8 -2.679283 0.781188 0.392524
1 -2.556774 0.641648 1.347780
8 -2.485897 -1.527423 0.049994
1 -3.365511 -1.647475 -0.341128
1 2.139509 1.774421 -0.000328
rc_5dhmC/rc_5dhmC06.xyz
6 -2.277734 -0.279026 -0.046801
6 -0.229580 1.020272 0.081651
6 0.511964 -0.227103 0.095220
6 -0.250033 -1.420024 0.041600
1 0.279649 -2.365363 0.063652
7 -1.562715 -1.475228 -0.039060
7 -1.577175 0.926770 0.020859
8 -3.502472 -0.264134 -0.112280
6 2.011105 -0.267621 0.285874
1 2.208806 -0.080041 1.353835
7 0.351828 2.195775 0.136471
1 -0.193541 3.055162 0.135968
1 1.368906 2.243181 0.082652
8 2.596137 0.762350 -0.491316
1 3.490934 0.927474 -0.153306
8 2.481131 -1.540636 -0.078060
1 3.310321 -1.707851 0.395961
1 -2.141351 1.776595 0.001337
rc_5dhmC/rc_5dhmC04.xyz
6 2.289807 -0.127549 0.009827
6 0.095704 0.918563 0.013848
6 -0.478199 -0.397288 -0.190084

6 0.425065 -1.484905 -0.277363
1 0.013820 -2.479564 -0.429459
7 1.733830 -1.389728 -0.192972
7 1.444155 0.981326 0.096747
8 3.501971 0.031843 0.109362
6 -1.967279 -0.659792 -0.168481
1 -2.177460 -1.641512 -0.603647
7 -0.632827 2.006602 0.116927
1 -0.200597 2.915476 0.263356
1 -1.641800 1.944305 -0.009360
8 -2.619281 0.366610 -0.868355
1 -3.561966 0.145250 -0.940396
8 -2.364032 -0.636006 1.197926
1 -2.489402 -1.548362 1.506441
1 1.901456 1.883262 0.230200
rc_5dhmC/rc_5dhmC02.xyz
6 -2.276541 -0.280780 -0.048968
6 -0.232420 1.023081 0.079851
6 0.512255 -0.222462 0.095918
6 -0.245599 -1.416116 0.037422
1 0.286671 -2.360911 0.060095
7 -1.558189 -1.475377 -0.046959
7 -1.579632 0.927533 0.020009
8 -3.501406 -0.269436 -0.111245
6 2.015370 -0.256049 0.309520
1 2.190901 -0.029980 1.371808
7 0.349489 2.198456 0.131654
1 -0.195375 3.058208 0.132752
1 1.366012 2.249370 0.078899
8 2.597651 0.735413 -0.512207
1 3.500596 0.898353 -0.194290
8 2.575052 -1.525750 0.100448
1 2.507245 -1.752873 -0.842953
1 -2.146484 1.775698 0.002341
rc_5dhmC/rc_5dhmC01.xyz
6 -2.292364 -0.133785 0.010721

6 -0.102880 0.924242 0.010580
6 0.479195 -0.390077 -0.184334
6 -0.420729 -1.482696 -0.264925
1 -0.004984 -2.476536 -0.409229
7 -1.729493 -1.394742 -0.181124
7 -1.451913 0.978930 0.091736
8 -3.505260 0.020523 0.106356
6 1.970168 -0.667428 -0.185097
1 2.160498 -1.615291 -0.694695
7 0.615353 2.020168 0.109565
1 0.174185 2.925514 0.251560
1 1.625389 1.965995 -0.014791
8 2.407118 -0.864756 1.147274
1 2.314038 -0.038389 1.653859
8 2.628233 0.401117 -0.821531
1 3.566188 0.172092 -0.924478
1 -1.914009 1.879520 0.218085
rc_5dhmC/rc_5dhmC05.xyz
6 2.269687 -0.286936 0.055539
6 0.230203 1.021690 -0.096157
6 -0.518172 -0.222622 -0.111989
6 0.236928 -1.417798 -0.041886
1 -0.296630 -2.361797 -0.065900
7 1.549158 -1.480130 0.048725
7 1.576256 0.922869 -0.022456
8 3.493897 -0.277797 0.129301
6 -2.028579 -0.238493 -0.318698
1 -2.215409 0.024725 -1.366453
7 -0.346229 2.199528 -0.160402
1 0.203373 3.056343 -0.160035
1 -1.362580 2.261065 -0.123281
8 -2.669448 0.762670 0.447317
1 -2.485107 0.619950 1.391963
8 -2.585632 -1.512483 -0.133092
1 -2.494412 -1.779789 0.797565
1 2.145532 1.769472 -0.003983

rc_5dhmC/rc_5dhmC08.xyz
6 -2.292890 -0.147025 0.014036
6 -0.117532 0.936462 0.027339
6 0.479628 -0.364214 -0.193132
6 -0.406178 -1.463870 -0.318964
1 0.021927 -2.446615 -0.500647
7 -1.714966 -1.395525 -0.217474
7 -1.466974 0.974386 0.116338
8 -3.507022 -0.012857 0.123926
6 1.976184 -0.619878 -0.198279
1 2.175641 -1.483780 -0.843513
7 0.593904 2.034083 0.146722
1 0.150029 2.933293 0.315925
1 1.601497 1.978131 -0.007775
8 2.320382 -0.888328 1.138277
1 3.148978 -1.395101 1.146801
8 2.724749 0.495479 -0.645992
1 2.816368 0.453870 -1.611899
1 -1.938342 1.866395 0.266320
rc_5dhmC/rc_5dhmC07.xyz
6 2.287491 -0.138891 0.011515
6 0.107725 0.937916 0.015658
6 -0.485544 -0.365680 -0.205177
6 0.406752 -1.463720 -0.314806
1 -0.017196 -2.449667 -0.488893
7 1.714639 -1.390320 -0.213984
7 1.456994 0.980092 0.106350
8 3.500863 0.001080 0.123298
6 -1.981598 -0.638408 -0.210604
1 -2.179143 -1.494325 -0.861498
7 -0.606258 2.034853 0.130973
1 -0.164104 2.935958 0.294221
1 -1.614477 1.974322 -0.017098
8 -2.742012 0.481740 -0.616628
1 -2.853058 0.462264 -1.581114
8 -2.373774 -1.024877 1.085365

1 -2.144574 -0.326012 1.722269
1 1.925355 1.874243 0.252938
rc_5dhmC/rc_5dhmC12.xyz
6 2.268211 -0.109884 0.039705
6 0.267979 1.059522 -0.119248
6 -0.518725 -0.179740 -0.110783
6 0.203751 -1.385830 0.026894
1 -0.285013 -2.351409 0.066273
7 1.512899 -1.340830 0.105130
7 1.601087 1.050486 -0.072694
8 3.492924 -0.229770 0.102197
6 -2.022486 -0.189526 -0.297593
1 -2.250024 0.234230 -1.284693
7 -0.359213 2.226868 -0.198128
1 0.188060 3.081126 -0.219540
1 -1.368515 2.291112 -0.123635
8 -2.650541 0.673233 0.631251
1 -2.448770 0.374024 1.535204
8 -2.467178 -1.516582 -0.195767
1 -3.367134 -1.564489 -0.554857
1 2.053976 -2.202554 0.205795
rc_5dhmC/rc_5dhmC13.xyz
6 2.269787 0.077491 0.029967
6 0.111020 0.934871 -0.003285
6 -0.475619 -0.402352 -0.160121
6 0.428152 -1.482057 -0.235418
1 0.095236 -2.507960 -0.356138
7 1.717587 -1.246624 -0.160671
7 1.428183 1.122719 0.094742
8 3.497572 0.137586 0.114911
6 -1.957615 -0.688003 -0.101874
1 -2.156713 -1.727671 -0.376609
7 -0.693311 1.988967 0.049399
1 -0.292225 2.914851 0.155845
1 -1.694168 1.891293 -0.086977
8 -2.618227 0.206340 -0.959234

1 -3.543461 -0.074285 -1.052225
8 -2.319684 -0.457179 1.251468
1 -2.695758 -1.270012 1.625366
1 2.388246 -2.015323 -0.226329
rc_5dhmC/rc_5dhmC16.xyz
6 -2.269675 0.068574 0.029008
6 -0.118540 0.949488 0.016739
6 0.481059 -0.375734 -0.189129
6 -0.412589 -1.464147 -0.270697
1 -0.069133 -2.484179 -0.410540
7 -1.703679 -1.246456 -0.179341
7 -1.438411 1.120485 0.115137
8 -3.498518 0.115311 0.109813
6 1.963797 -0.668966 -0.165724
1 2.162284 -1.662766 -0.578096
7 0.672271 2.010820 0.109343
1 0.257638 2.926871 0.244083
1 1.674994 1.925935 -0.026093
8 2.320928 -0.629566 1.212179
1 2.456575 -1.537464 1.531335
8 2.643845 0.330274 -0.871303
1 3.577482 0.076531 -0.953971
1 -2.365463 -2.022314 -0.249390
rc_5dhmC/rc_5dhmC15.xyz
6 -2.280788 -0.088285 -0.032251
6 -0.262082 1.056063 0.083688
6 0.511638 -0.193287 0.076975
6 -0.231919 -1.391142 -0.013276
1 0.243014 -2.364209 -0.029254
7 -1.540606 -1.329716 -0.082907
7 -1.596175 1.063357 0.053093
8 -3.507571 -0.194470 -0.077172
6 2.006451 -0.251999 0.284073
1 2.188099 0.012243 1.339443
7 0.380034 2.215874 0.140696
1 -0.155413 3.077759 0.153715

1 1.391621 2.251164 0.058443
8 2.616202 0.704082 -0.559974
1 3.504470 0.890205 -0.215496
8 2.453821 -1.559324 0.028739
1 3.259098 -1.717176 0.545387
1 -2.093076 -2.187000 -0.156412
rc_5dhmC/rc_5dhmC18.xyz
6 2.274313 0.080367 -0.000007
6 0.109859 0.919268 -0.000004
6 -0.469388 -0.429397 -0.000003
6 0.437682 -1.508461 -0.000007
1 0.110113 -2.542779 -0.000009
7 1.727006 -1.258553 -0.000010
7 1.427833 1.123509 -0.000005
8 3.504190 0.154250 -0.000005
6 -1.955274 -0.695302 0.000007
1 -2.149754 -1.770979 0.000004
7 -0.700420 1.970502 -0.000003
1 -0.301437 2.903696 -0.000003
1 -1.708684 1.873168 -0.000006
8 -2.467387 -0.094797 -1.171127
1 -3.236341 -0.600774 -1.480014
8 -2.467366 -0.094806 1.171156
1 -3.236311 -0.600790 1.480056
1 2.402828 -2.025778 -0.000014
rc_5dhmC/rc_5dhmC11.xyz
6 -2.281627 -0.088557 -0.037873
6 -0.264810 1.057874 0.078401
6 0.511027 -0.190520 0.083508
6 -0.231620 -1.388684 0.006489
1 0.244206 -2.362195 0.009412
7 -1.539982 -1.330094 -0.072786
7 -1.598681 1.064814 0.040024
8 -3.507986 -0.196696 -0.087648
6 2.010582 -0.249649 0.309320
1 2.170384 0.019185 1.365468

7 0.377927 2.217672 0.128724
1 -0.156437 3.080352 0.133453
1 1.389973 2.253081 0.054198
8 2.622859 0.689210 -0.546133
1 3.519513 0.860156 -0.214417
8 2.541464 -1.541281 0.165721
1 2.537065 -1.781249 -0.777269
1 -2.091562 -2.188723 -0.137175
rc_5dhmC/rc_5dhmC10.xyz
6 -2.273415 0.060949 0.030617
6 -0.127833 0.957698 0.013079
6 0.482195 -0.364039 -0.188683
6 -0.406675 -1.458734 -0.266677
1 -0.057134 -2.477100 -0.403392
7 -1.698335 -1.250642 -0.174248
7 -1.449023 1.118222 0.112217
8 -3.502265 0.099100 0.112651
6 1.966995 -0.674368 -0.184485
1 2.144954 -1.630189 -0.683669
7 0.651043 2.028904 0.100791
1 0.225201 2.939923 0.235123
1 1.654872 1.954477 -0.035111
8 2.360060 -0.875469 1.162248
1 2.296283 -0.039537 1.658800
8 2.658673 0.372823 -0.811670
1 3.585106 0.108278 -0.932094
1 -2.354421 -2.031914 -0.239906
rc_5dhmC/rc_5dhmC14.xyz
6 2.272231 -0.103986 0.041333
6 0.267260 1.058917 -0.104185
6 -0.518449 -0.182900 -0.099533
6 0.211846 -1.386158 0.012568
1 -0.272095 -2.355368 0.033143
7 1.520741 -1.338349 0.092997
7 1.600627 1.054731 -0.058897
8 3.497322 -0.220974 0.101400

6 -2.026264 -0.204540 -0.313560
1 -2.211773 0.174717 -1.325750
7 -0.364226 2.224232 -0.174822
1 0.179243 3.081001 -0.193194
1 -1.374804 2.280714 -0.105476
8 -2.676275 0.689146 0.564481
1 -2.459707 0.464388 1.486056
8 -2.560382 -1.502321 -0.282851
1 -2.550583 -1.844545 0.627941
1 2.064655 -2.200020 0.178376
rc_5dhmC/rc_5dhmC19.xyz
6 2.276451 0.053979 0.045349
6 0.137196 0.961029 0.019567
6 -0.478314 -0.355572 -0.179692
6 0.404960 -1.447774 -0.316605
1 0.051657 -2.458237 -0.497063
7 1.697156 -1.247147 -0.213285
7 1.457902 1.114813 0.140596
8 3.503994 0.079637 0.149257
6 -1.968265 -0.647556 -0.155143
1 -2.146636 -1.581649 -0.701572
7 -0.645471 2.029173 0.101378
1 -0.227141 2.941178 0.251853
1 -1.642931 1.946301 -0.077656
8 -2.737079 0.394390 -0.719861
1 -2.788051 0.271631 -1.682315
8 -2.305707 -0.774929 1.202877
1 -3.087303 -1.347430 1.274257
1 2.351465 -2.027092 -0.307366
rc_5dhmC/rc_5dhmC17.xyz
6 2.271255 0.055698 0.037670
6 0.134413 0.971435 0.016009
6 -0.487283 -0.340280 -0.205590
6 0.394158 -1.438647 -0.318846
1 0.037466 -2.449830 -0.488603
7 1.686406 -1.244530 -0.209483

7 1.456225 1.119483 0.131659
8 3.499244 0.078264 0.137616
6 -1.977006 -0.648543 -0.200677
1 -2.156588 -1.525914 -0.828322
7 -0.641361 2.043981 0.112056
1 -0.215622 2.951867 0.266149
1 -1.642137 1.964416 -0.050635
8 -2.771852 0.436582 -0.619782
1 -2.904753 0.385455 -1.580291
8 -2.332711 -1.019143 1.111187
1 -2.134345 -0.292596 1.727726
1 2.336419 -2.029533 -0.289224
rc_5dhmC/rc_5dhmC09.xyz
6 -2.304040 -0.195598 0.002076
6 -0.149240 0.951351 -0.007853
6 0.497575 -0.354365 -0.016626
6 -0.376462 -1.477505 -0.029097
1 0.076359 -2.465114 -0.043980
7 -1.689214 -1.442626 -0.021923
7 -1.502818 0.946960 0.005946
8 -3.524578 -0.076157 0.019645
6 1.995001 -0.677619 -0.001541
1 2.070476 -1.764773 -0.021924
7 0.466511 2.113592 -0.012165
1 -0.063160 2.983050 -0.004169
1 1.477709 2.192437 -0.016555
8 2.619416 -0.294808 1.201781
1 2.658664 0.669245 1.310145
8 2.661239 -0.248913 -1.167215
1 2.837432 0.705450 -1.159077
1 -2.004457 1.835658 0.017116
rc_5dhmC/rc_5dhmC26.xyz
6 -2.159647 -0.191236 -0.034395
6 -0.235765 1.007682 0.057235
6 0.532989 -0.223558 0.109115
6 -0.236981 -1.415081 0.103225

1 0.267585 -2.372079 0.162395
7 -1.550307 -1.428259 0.024973
7 -1.587068 0.990058 -0.015943
8 -3.476670 -0.285124 -0.113333
6 2.031026 -0.298923 0.286690
1 2.226153 -0.265747 1.371295
7 0.358788 2.187611 0.083567
1 -0.204653 3.034564 0.055045
1 1.378414 2.237818 0.070056
8 2.645673 0.814282 -0.335021
1 3.525236 0.938354 0.056411
8 2.486839 -1.519506 -0.246129
1 3.304947 -1.769845 0.210945
1 -3.884053 0.600544 -0.149671
rc_5dhmU/rc_5dhmU09.xyz
6 2.234372 -0.254777 0.039766
6 0.235228 1.168874 -0.170615
6 -0.589453 -0.050819 -0.143157
6 0.041165 -1.300391 0.019731
1 -0.518169 -2.227602 0.079097
7 1.356769 -1.374943 0.088546
7 1.605725 0.954454 -0.094477
8 3.436391 -0.415907 0.118612
8 -0.234897 2.297540 -0.259154
1 2.209587 1.775899 -0.119512
6 -2.087410 0.061186 -0.157605
1 1.815611 -2.284247 0.182961
8 -2.618572 -1.065664 -0.784840
1 -3.551116 -0.895824 -0.993701
8 -2.426203 0.043607 1.230446
1 -2.536803 0.958855 1.543296
1 -2.413735 0.995288 -0.619862
rc_5dhmU/rc_5dhmU10.xyz
6 2.234585 -0.269540 -0.020157
6 0.253088 1.189920 0.105629
6 -0.589523 -0.016211 0.091385

6 0.025642 -1.279998 -0.027437
1 -0.547682 -2.199520 -0.076592
7 1.340223 -1.376937 -0.067435
7 1.621823 0.951937 0.064398
8 3.435513 -0.451497 -0.058367
8 -0.199618 2.328147 0.158606
1 2.237853 1.764239 0.089184
6 -2.088212 0.097060 0.101275
1 1.786620 -2.295359 -0.130814
8 -2.470503 -0.226928 -1.232800
1 -2.658469 0.592269 -1.722645
8 -2.596317 -0.861473 0.982723
1 -3.513761 -0.630471 1.201242
1 -2.404964 1.110475 0.355405
rc_5dhmU/rc_5dhmU03.xyz
6 2.238636 -0.259612 0.039135
6 0.246120 1.176413 -0.148536
6 -0.587575 -0.038615 -0.127031
6 0.039979 -1.295574 0.005223
1 -0.522457 -2.222075 0.045227
7 1.354422 -1.376804 0.070721
7 1.615818 0.953664 -0.075627
8 3.439245 -0.430337 0.113283
8 -0.216131 2.308233 -0.230651
1 2.223651 1.772430 -0.093153
6 -2.088751 0.086473 -0.151412
1 1.809337 -2.290300 0.147292
8 -2.608944 -1.041336 -0.800508
1 -3.526270 -0.857867 -1.058808
8 -2.502858 0.187671 1.201728
1 -2.367928 -0.668895 1.647532
1 -2.388967 1.020331 -0.628845
rc_5dhmU/rc_5dhmU01.xyz
6 2.281899 0.027123 -0.000005
6 0.004252 0.951267 -0.000001
6 -0.523510 -0.429485 0.000003

6 0.390652 -1.503372 0.000003
1 0.066464 -2.539324 0.000005
7 1.689289 -1.269913 -0.000001
7 1.385295 1.062649 -0.000005
8 3.490294 0.145256 -0.000007
8 -0.724959 1.938323 0.000001
1 1.782111 2.002452 -0.000007
6 -2.017692 -0.675874 0.000005
1 2.351827 -2.050212 -0.000001
8 -2.594077 -0.168315 1.180492
1 -2.471011 0.796552 1.223560
8 -2.594076 -0.168319 -1.180485
1 -2.471012 0.796548 -1.223555
1 -2.211531 -1.748678 0.000006
rc_5dhmU/rc_5dhmU02.xyz
6 -2.281399 0.016723 0.024968
6 -0.009115 0.960302 -0.002260
6 0.525509 -0.414218 -0.085929
6 -0.381340 -1.491159 -0.150049
1 -0.049728 -2.521407 -0.235136
7 -1.681167 -1.270660 -0.099333
7 -1.391542 1.055838 0.071070
8 -3.490021 0.123278 0.079600
8 0.705830 1.956489 0.008408
1 -1.793946 1.990550 0.139578
6 2.016573 -0.651791 -0.054050
1 -2.337883 -2.054498 -0.142137
8 2.654360 0.045652 -1.097284
1 2.524034 1.001649 -0.960252
8 2.445825 -0.240696 1.224455
1 3.190635 -0.799264 1.501678
1 2.226520 -1.710202 -0.223396
rc_5dhmU/rc_5dhmU13.xyz
6 2.282975 -0.000500 0.003176
6 0.018735 0.979100 -0.009034
6 -0.533611 -0.391530 -0.087722

6 0.359035 -1.482199 -0.102025
1 0.013067 -2.510153 -0.144576
7 1.662920 -1.281664 -0.068535
7 1.408373 1.050194 0.030832
8 3.494518 0.090202 0.036972
8 -0.668013 1.991357 0.017244
1 1.824622 1.979796 0.080864
6 -2.020546 -0.627534 -0.046243
1 2.306108 -2.077007 -0.095447
8 -2.432828 -0.139965 1.222030
1 -2.729024 -0.884751 1.770858
8 -2.624446 0.092474 -1.085006
1 -3.473125 -0.327302 -1.300848
1 -2.234069 -1.696848 -0.125761
rc_5dhmU/rc_5dhmU12.xyz
6 -2.286507 -0.001266 0.000007
6 -0.022353 0.975353 -0.000001
6 0.530769 -0.396998 -0.000003
6 -0.361432 -1.488127 -0.000001
1 -0.015860 -2.517013 -0.000002
7 -1.665086 -1.285066 0.000003
7 -1.411921 1.049772 0.000005
8 -3.498219 0.089311 0.000009
8 0.667354 1.986325 -0.000005
1 -1.828369 1.980732 0.000005
6 2.019762 -0.624091 -0.000004
1 -2.308164 -2.081198 0.000004
8 2.522546 -0.018096 -1.170453
1 3.231133 -0.572257 -1.535912
8 2.522548 -0.018095 1.170443
1 3.231130 -0.572259 1.535907
1 2.233911 -1.695738 -0.000003
rc_5dhmU/rc_5dhmU07.xyz
6 2.248311 -0.254078 0.043270
6 0.248371 1.169552 -0.144347
6 -0.585264 -0.048637 -0.117032

6 0.054879 -1.301667 -0.011489
1 -0.493392 -2.237807 0.010560
7 1.369291 -1.375644 0.060496
7 1.619024 0.955577 -0.067384
8 3.449096 -0.419931 0.123309
8 -0.218430 2.299591 -0.224827
1 2.221733 1.778281 -0.080586
6 -2.092801 0.095036 -0.137914
1 1.828282 -2.287474 0.134011
8 -2.717017 -0.906220 -0.900362
1 -2.717587 -1.752646 -0.421159
8 -2.551285 0.163623 1.195302
1 -2.349237 -0.664890 1.666201
1 -2.347889 1.047251 -0.603116
rc_5dhmU/rc_5dhmU25.xyz
6 -2.277145 -0.038924 0.038129
6 -0.226566 1.034376 -0.026548
6 0.492598 -0.232977 -0.115404
6 -0.296867 -1.404635 -0.123920
1 0.137933 -2.395007 -0.194260
7 -1.603470 -1.299151 -0.046237
7 -1.537002 1.101974 0.048428
8 -3.503200 -0.067864 0.098868
8 0.430698 2.174383 -0.017018
1 1.407997 1.986183 0.010222
6 1.985451 -0.358312 -0.297207
1 -2.192229 -2.136016 -0.050817
8 2.614063 0.815191 0.191081
1 3.447218 0.952413 -0.288254
8 2.425151 -1.513379 0.366814
1 3.221443 -1.845389 -0.077855
1 2.162421 -0.445756 -1.382636
rc_5dhmU/rc_5dhmU33.xyz
6 -2.224024 -0.207746 0.038515
6 -0.316229 1.080593 -0.157740
6 0.548822 -0.081912 -0.142753

6 -0.086653 -1.332288 0.017083
1 0.464500 -2.263889 0.073018
7 -1.398939 -1.375196 0.093208
7 -1.623211 1.011269 -0.095048
8 -3.436015 -0.371963 0.120285
8 0.295107 2.247145 -0.254077
1 -0.352980 2.978304 -0.262091
6 2.051132 0.023025 -0.161290
1 -1.882782 -2.269946 0.197000
8 2.407258 0.037529 1.221194
1 2.556531 0.956178 1.504500
8 2.564324 -1.125971 -0.766215
1 3.499306 -0.975208 -0.978349
1 2.386802 0.938105 -0.653573
rc_5dhmU/rc_5dhmU15.xyz
6 2.273441 -0.028103 -0.043846
6 0.216941 1.034046 0.019160
6 -0.493789 -0.235868 0.137043
6 0.303856 -1.402250 0.160472
1 -0.124757 -2.394137 0.251944
7 1.609028 -1.290751 0.072931
7 1.526691 1.108313 -0.066752
8 3.498836 -0.051201 -0.118395
8 -0.446984 2.169848 -0.001484
1 -1.423266 1.976358 -0.001535
6 -1.992518 -0.382027 0.326786
1 2.203676 -2.123344 0.090629
8 -2.622289 0.794494 -0.142501
1 -3.471720 0.905915 0.314517
8 -2.488195 -1.539639 -0.293413
1 -2.340398 -1.475474 -1.253209
1 -2.172100 -0.515057 1.403061
rc_5dhmU/rc_5dhmU34.xyz
6 -2.225731 -0.213893 0.037790
6 -0.324398 1.086862 -0.140520
6 0.547187 -0.071508 -0.133201

6 -0.084115 -1.328120 0.001892
1 0.470571 -2.258580 0.040447
7 -1.395886 -1.378512 0.076903
7 -1.631488 1.009430 -0.079257
8 -3.437039 -0.385901 0.115681
8 0.278739 2.257354 -0.227579
1 -0.374234 2.984287 -0.230425
6 2.052091 0.046789 -0.159812
1 -1.875871 -2.277466 0.164465
8 2.472704 0.190970 1.187116
1 2.312682 -0.643529 1.665080
8 2.562949 -1.107078 -0.770915
1 3.485735 -0.942850 -1.022602
1 2.363704 0.958184 -0.671790
rc_5dhmU/rc_5dhmU16.xyz
6 2.257171 0.072866 0.044544
6 0.115679 0.953194 0.019215
6 -0.474491 -0.356773 -0.229270
6 0.421203 -1.444608 -0.322189
1 0.081588 -2.461322 -0.492850
7 1.710385 -1.228151 -0.199737
7 1.411481 1.135318 0.138287
8 3.477341 0.152352 0.152446
8 -0.646742 2.022983 0.133041
1 -1.591286 1.790844 -0.055466
6 -1.961510 -0.640671 -0.244055
1 2.376769 -2.000992 -0.270303
8 -2.325771 -1.050605 1.057902
1 -2.144333 -0.332609 1.691426
8 -2.623691 0.530693 -0.658977
1 -3.584895 0.391217 -0.627214
1 -2.188306 -1.484737 -0.900212
rc_5dhmU/rc_5dhmU17.xyz
6 2.273239 -0.056411 -0.033866
6 0.235555 1.032529 0.068029
6 -0.494986 -0.228645 0.103266

6 0.276677 -1.408351 0.022217
1 -0.169774 -2.395594 0.024504
7 1.584535 -1.310698 -0.058683
7 1.547095 1.093372 0.023457
8 3.499931 -0.095484 -0.072902
8 -0.413448 2.179723 0.092662
1 -1.388689 2.018001 0.010569
6 -1.990808 -0.288242 0.329017
1 2.164661 -2.151379 -0.120691
8 -2.651388 0.807028 -0.292522
1 -2.623142 0.683488 -1.258872
8 -2.464047 -1.527281 -0.111243
1 -3.313265 -1.705461 0.323919
1 -2.157647 -0.144938 1.407208
rc_5dhmU/rc_5dhmU32.xyz
6 2.222048 -0.229439 0.018582
6 0.336086 1.102385 -0.099081
6 -0.550962 -0.042209 -0.092030
6 0.063145 -1.308711 0.032152
1 -0.506730 -2.229556 0.083734
7 1.374822 -1.380902 0.077453
7 1.642925 1.004029 -0.070385
8 3.432236 -0.420193 0.056228
8 -0.249623 2.284639 -0.155044
1 0.416232 2.999765 -0.166406
6 -2.054206 0.065186 -0.108005
1 1.842296 -2.287400 0.152770
8 -2.545689 -0.916382 -0.974608
1 -3.472720 -0.715015 -1.181540
8 -2.445721 -0.236766 1.228086
1 -2.644611 0.589265 1.701147
1 -2.384985 1.067398 -0.386180
rc_5dhmU/rc_5dhmU23.xyz
6 -2.257128 0.073168 0.044236
6 -0.113820 0.947628 0.033597
6 0.473680 -0.361285 -0.224582

6 -0.422583 -1.445684 -0.329307
1 -0.084837 -2.461746 -0.507385
7 -1.712330 -1.226618 -0.209719
7 -1.408810 1.132706 0.151254
8 -3.477612 0.154875 0.146820
8 0.651461 2.015182 0.153474
1 1.593315 1.786247 -0.046272
6 1.960564 -0.632306 -0.229335
1 -2.380461 -1.996628 -0.291749
8 2.608902 0.512955 -0.718760
1 3.571332 0.382005 -0.685167
8 2.293088 -0.838524 1.133865
1 2.499233 -1.778098 1.272630
1 2.198404 -1.519431 -0.823650
rc_5dhmU/rc_5dhmU30.xyz
6 2.263088 0.068909 0.021026
6 0.106843 0.897479 0.004137
6 -0.475578 -0.432868 -0.053752
6 0.438592 -1.506211 -0.110346
1 0.117247 -2.540558 -0.175851
7 1.729763 -1.254286 -0.073690
7 1.396187 1.122555 0.047910
8 3.482785 0.182324 0.069788
8 -0.755994 1.896121 0.015357
1 -0.303329 2.761324 0.042597
6 -1.966586 -0.690008 -0.039737
1 2.408597 -2.018726 -0.108619
8 -2.442549 -0.230586 1.203423
1 -3.201849 -0.774173 1.470050
8 -2.587866 -0.057708 -1.135483
1 -2.519072 0.908056 -1.031769
1 -2.152394 -1.758811 -0.168598
rc_5dhmU/rc_5dhmU18.xyz
6 -2.262156 0.079148 0.000000
6 -0.101438 0.890439 -0.000005
6 0.474259 -0.444182 0.000001

6 -0.447424 -1.513402 0.000003
1 -0.133619 -2.552038 0.000005
7 -1.736889 -1.250319 0.000001
7 -1.387796 1.128271 -0.000005
8 -3.481350 0.203626 0.000000
8 0.774324 1.879733 -0.000010
1 0.335386 2.752498 -0.000010
6 1.967269 -0.711102 0.000005
1 -2.421498 -2.010520 0.000002
8 2.556731 -0.219654 -1.181304
1 2.459216 0.747096 -1.235336
8 2.556725 -0.219646 1.181313
1 2.459208 0.747104 1.235341
1 2.139598 -1.787675 0.000009
rc_5dhmU/rc_5dhmU26.xyz
6 -2.262092 0.050999 0.045593
6 -0.140294 0.978030 0.029988
6 0.481097 -0.316393 -0.218224
6 -0.393165 -1.420938 -0.339085
1 -0.032323 -2.426903 -0.529095
7 -1.686708 -1.234246 -0.216042
7 -1.440700 1.131280 0.148717
8 -3.483748 0.099929 0.156402
8 0.599895 2.062005 0.145559
1 1.550421 1.835673 -0.032728
6 1.976335 -0.580994 -0.265511
1 -2.336589 -2.019640 -0.300589
8 2.701213 0.622756 -0.415002
1 3.136647 0.646341 -1.282128
8 2.341019 -1.239552 0.924978
1 2.139197 -0.669971 1.688683
1 2.196174 -1.270071 -1.084937
rc_5dhmU/rc_5dhmU31.xyz
6 -2.264082 0.052838 -0.000007
6 -0.115713 0.909477 0.000002
6 0.480873 -0.417610 0.000003

6 -0.419911 -1.503694 0.000001
1 -0.085758 -2.535832 0.000002
7 -1.714320 -1.267206 -0.000003
7 -1.410705 1.116036 -0.000003
8 -3.486450 0.152186 -0.000014
8 0.726605 1.923060 0.000004
1 0.250876 2.776181 0.000002
6 1.969389 -0.661616 0.000003
1 -2.383652 -2.040780 -0.000005
8 2.483829 -0.063737 -1.169676
1 3.180228 -0.631694 -1.536934
8 2.483827 -0.063746 1.169688
1 3.180221 -0.631709 1.536947
1 2.167430 -1.736449 0.000000
rc_5fC/rc_5fC01.xyz
6 1.966909 -0.024384 0.000004
6 -0.304012 0.831880 0.000003
6 -0.766413 -0.549557 -0.000001
6 0.221897 -1.568274 0.000010
1 -0.105053 -2.604731 0.000016
7 1.517707 -1.350342 0.000014
7 1.033106 1.015747 0.000005
8 3.162285 0.236190 -0.000004
7 -1.128705 1.847592 0.000006
1 -0.784856 2.806018 0.000010
1 -2.132973 1.668663 0.000002
6 -2.191002 -0.930044 -0.000013
1 -2.389912 -2.011731 -0.000011
8 -3.102401 -0.107408 -0.000022
1 1.414683 1.962812 0.000003
rc_5fC/rc_5fC02.xyz
6 1.876618 -0.393994 0.000003
6 -0.052864 1.087478 0.000004
6 -0.894795 -0.099286 -0.000002
6 -0.235787 -1.363168 -0.000007
1 -0.854493 -2.255157 -0.000012

7 1.063437 -1.532116 -0.000005
7 1.278806 0.870276 0.000012
8 3.096367 -0.481362 0.000000
7 -0.518786 2.314660 0.000002
1 0.115663 3.111399 0.000007
1 -1.514613 2.512245 -0.000002
6 -2.374736 -0.055511 -0.000003
1 -2.872900 0.923052 0.000001
8 -3.029972 -1.091023 -0.000006
1 1.920365 1.664684 0.000016
rc_5fC/rc_5fC03.xyz
6 1.931489 0.166883 0.013370
6 -0.284788 0.865064 -0.015577
6 -0.766774 -0.523431 -0.009181
6 0.218513 -1.547668 -0.036937
1 -0.045037 -2.601250 -0.057061
7 1.480544 -1.212648 -0.033224
7 1.019245 1.149992 0.012597
8 3.152404 0.318685 0.048923
7 -1.161668 1.855907 -0.044167
1 -0.826698 2.814048 -0.045013
1 -2.159065 1.663917 -0.050994
6 -2.184079 -0.942927 0.026664
1 -2.359805 -2.028876 0.034860
8 -3.111413 -0.140862 0.045053
1 2.209666 -1.930717 -0.050080
rc_5fC/rc_5fC04.xyz
6 1.833117 0.005193 0.000227
6 -0.279754 0.825110 -0.015024
6 -0.803846 -0.533753 -0.005934
6 0.168352 -1.567251 -0.025050
1 -0.152561 -2.604909 -0.039058
7 1.462080 -1.331298 -0.030323
7 1.050158 1.060439 -0.007859
8 3.141402 0.153681 0.040456
7 -1.097808 1.858462 -0.027466

1 -0.717649 2.803931 -0.032643
1 -2.108536 1.710454 -0.023418
6 -2.236574 -0.890028 0.018106
1 -2.455558 -1.968343 0.020174
8 -3.133337 -0.052370 0.037441
1 3.381009 1.099527 0.077363
rc_5fC/rc_5fC06.xyz
6 1.896807 -0.190278 0.000010
6 -0.028840 1.112052 0.000001
6 -0.892390 -0.076088 0.000001
6 -0.238925 -1.341094 -0.000001
1 -0.797261 -2.271266 -0.000004
7 1.063504 -1.382089 0.000001
7 1.300254 1.012244 0.000003
8 3.108368 -0.395547 -0.000004
7 -0.566458 2.325739 -0.000002
1 0.051201 3.131943 -0.000002
1 -1.565164 2.491945 -0.000002
6 -2.375497 -0.075125 0.000000
1 -2.901237 0.887959 -0.000002
8 -2.994624 -1.132940 -0.000005
1 1.554485 -2.280740 -0.000001
rc_5fC/rc_5fC05.xyz
6 1.830127 0.060873 0.000004
6 -0.296064 0.832708 -0.000003
6 -0.795092 -0.536066 0.000004
6 0.196359 -1.550931 0.000030
1 -0.100819 -2.595747 0.000048
7 1.483635 -1.283515 0.000031
7 1.027147 1.099807 -0.000007
8 3.123060 0.314252 -0.000013
7 -1.136951 1.848800 -0.000006
1 -0.775946 2.801858 -0.000010
1 -2.144266 1.679245 -0.000008
6 -2.220350 -0.922088 -0.000006
1 -2.415383 -2.004953 0.000008

8 -3.135921 -0.104921 -0.000029
1 3.632618 -0.517666 -0.000001
rc_5fC/rc_5fC08.xyz
6 -1.773031 -0.234444 -0.000005
6 0.067756 1.083899 -0.000001
6 0.916587 -0.098652 0.000004
6 0.229051 -1.345837 0.000006
1 0.802622 -2.266737 0.000011
7 -1.078748 -1.436926 0.000001
7 -1.277403 0.980870 -0.000006
8 -3.086214 -0.334026 -0.000008
7 0.574331 2.305257 0.000000
1 -0.061798 3.101706 -0.000003
1 1.573102 2.493122 0.000004
6 2.398690 -0.088881 0.000005
1 2.918705 0.878731 0.000002
8 3.031760 -1.138061 0.000005
1 -3.358585 -1.271033 -0.000005
rc_5fC/rc_5fC07.xyz
6 -1.765062 -0.293292 -0.000001
6 0.046003 1.072554 0.000002
6 0.919287 -0.091170 0.000003
6 0.259830 -1.353554 0.000000
1 0.855622 -2.260191 0.000002
7 -1.045010 -1.478608 -0.000001
7 -1.297285 0.934820 0.000000
8 -3.064085 -0.506096 -0.000004
7 0.526440 2.303700 0.000003
1 -0.124291 3.088103 0.000002
1 1.521338 2.512007 0.000005
6 2.399790 -0.044734 0.000006
1 2.895036 0.935521 -0.000003
8 3.058861 -1.077896 -0.000006
1 -3.554015 0.338292 -0.000005
rc_5fC/rc_5fC09.xyz
6 -1.958711 0.056506 -0.000001

6 0.235126 0.747368 -0.000001
6 0.690623 -0.602537 0.000000
6 -0.337106 -1.589429 -0.000002
1 -0.057750 -2.639803 -0.000003
7 -1.615330 -1.295825 -0.000003
7 -1.010532 1.091330 -0.000001
8 -3.157440 0.370459 0.000002
7 1.200794 1.852062 -0.000001
1 1.062364 2.443442 -0.831950
1 1.062370 2.443437 0.831952
6 2.108311 -1.018137 0.000003
1 2.290000 -2.102378 0.000004
8 3.034665 -0.211140 0.000004
1 2.171251 1.485157 -0.000004
rc_5fC/rc_5fC10.xyz
6 -1.891067 -0.322357 -0.000005
6 0.005476 0.985043 0.000003
6 0.840773 -0.168062 0.000003
6 0.143808 -1.415223 0.000000
1 0.724474 -2.332462 -0.000001
7 -1.161454 -1.513056 -0.000005
7 -1.286651 0.945282 -0.000001
8 -3.127632 -0.369955 -0.000009
7 0.588166 2.334544 0.000006
1 -0.161553 3.039572 0.000009
1 1.177667 2.490329 -0.832298
6 2.324922 -0.148057 0.000003
1 2.828329 0.828990 0.000003
8 2.968822 -1.189070 0.000003
1 1.177671 2.490324 0.832308
rc_5fC/rc_5fC11.xyz
6 -2.005278 -0.341998 0.017553
6 -0.042546 1.082819 -0.027668
6 0.869611 -0.061636 -0.014771
6 0.202725 -1.319303 -0.065698
1 0.773899 -2.247855 -0.073538

7 -1.081450 -1.420395 -0.111834
7 -1.356436 0.849311 -0.049585
8 -3.204207 -0.518064 0.130113
7 0.367191 2.342263 -0.024464
1 -0.311707 3.094676 -0.012186
1 1.347869 2.590574 0.001800
6 2.249780 0.036494 0.052789
1 2.758943 0.995954 0.116438
8 2.996256 -1.032042 0.044042
1 3.943715 -0.809009 0.102193
rc_5fC/rc_5fC12.xyz
6 -2.030204 0.006093 0.137721
6 0.198141 0.871762 -0.165470
6 0.771631 -0.482505 -0.188178
6 -0.217754 -1.495234 -0.333245
1 0.071982 -2.538467 -0.467796
7 -1.484720 -1.231546 -0.309959
7 -1.115625 1.005471 0.003556
8 -3.185888 0.120480 0.502598
7 0.928378 1.963726 -0.329779
1 0.483771 2.875655 -0.294407
1 1.904392 1.924358 -0.598639
6 2.072885 -0.912477 0.050076
1 2.311958 -1.958080 -0.134211
8 3.092805 -0.251836 0.513640
1 2.908135 0.657994 0.813003
rc_5fU/rc_5fU01.xyz
6 1.915921 0.144590 0.000002
6 -0.440488 0.787086 0.000004
6 -0.788479 -0.617040 0.000006
6 0.208773 -1.574275 -0.000002
1 0.002307 -2.639594 -0.000002
7 1.486677 -1.192753 -0.000011
7 0.892474 1.084690 0.000013
8 3.093974 0.445854 0.000000
8 -1.322995 1.671439 -0.000011

1 1.176044 2.064407 -0.000011
6 -2.184704 -0.851119 0.000003
1 -2.594181 -1.873258 0.000005
8 -2.987490 0.102939 0.000000
1 2.227728 -1.892415 0.000005
rc_5fU/rc_5fU02.xyz
6 -1.901718 -0.236064 0.000006
6 0.116063 1.172613 0.000009
6 0.931173 -0.056621 0.000005
6 0.279830 -1.319990 0.000006
1 0.836615 -2.252078 0.000003
7 -1.028020 -1.376290 0.000009
7 -1.257791 0.970516 0.000000
8 -3.101606 -0.402053 -0.000009
8 0.605570 2.295205 -0.000009
1 -1.852371 1.799936 -0.000011
6 2.406565 0.017494 0.000001
1 2.850033 1.020468 0.000023
8 3.080868 -1.006625 -0.000011
1 -1.503730 -2.284728 0.000006
rc_5fU/rc_5fU03.xyz
6 -1.915649 -0.180329 -0.000025
6 0.303371 -0.836473 0.000001
6 0.748870 0.544818 -0.000003
6 -0.247927 1.551003 -0.000003
1 -0.005219 2.609672 0.000002
7 -1.507698 1.191117 -0.000006
7 -0.974426 -1.163425 -0.000002
8 -3.125660 -0.396588 0.000019
8 1.169314 -1.824567 0.000002
1 2.092440 -1.467784 -0.000001
6 2.172343 0.924468 0.000000
1 2.404540 1.997853 0.000004
8 3.052600 0.065196 0.000007
1 -2.252981 1.893174 0.000002
rc_5fU/rc_5fU09.xyz

6 -1.770365 0.185574 0.000000
6 0.429648 0.788965 -0.000001
6 0.809726 -0.608955 0.000001
6 -0.175550 -1.564757 -0.000001
1 0.011560 -2.632658 -0.000001
7 -1.457680 -1.155903 -0.000001
7 -0.876795 1.149226 0.000000
8 -3.033454 0.549730 -0.000002
8 1.348230 1.664283 0.000000
1 -3.674934 -0.184645 0.000025
6 2.221255 -0.835376 0.000000
1 2.632805 -1.855269 -0.000003
8 2.996553 0.132910 0.000001
1 -2.207028 -1.848783 0.000002
rc_5fU/rc_5fU05.xyz
6 1.889413 -0.188121 0.000028
6 -0.040206 1.084776 -0.000006
6 -0.883600 -0.094916 -0.000007
6 -0.223566 -1.352203 -0.000001
1 -0.770108 -2.289526 -0.000007
7 1.083741 -1.378390 0.000010
7 1.270989 1.027863 0.000005
8 3.103788 -0.341469 0.000005
8 -0.669052 2.241003 -0.000013
1 -0.035715 2.985527 -0.000010
6 -2.363586 -0.040024 -0.000019
1 -2.825935 0.954407 -0.000005
8 -3.023455 -1.072460 -0.000001
1 1.587667 -2.270387 0.000009
rc_5fU/rc_5fU04.xyz
6 1.922722 0.125475 -0.000002
6 -0.276737 0.860220 0.000001
6 -0.784424 -0.501962 0.000002
6 0.186987 -1.538795 0.000002
1 -0.090866 -2.588652 0.000004
7 1.459233 -1.228583 0.000001

7 1.009567 1.135916 -0.000001
8 3.136501 0.300036 -0.000003
8 -1.160507 1.831615 0.000003
1 -0.715874 2.702072 0.000003
6 -2.212174 -0.902560 0.000001
1 -2.386310 -1.989859 0.000007
8 -3.136302 -0.102776 -0.000004
1 2.175664 -1.960159 0.000001
rc_5fU/rc_5fU06.xyz
6 -1.886639 -0.193117 0.002221
6 0.035853 1.108651 -0.003760
6 0.883315 -0.069550 0.000014
6 0.226299 -1.330861 -0.007958
1 0.783237 -2.261978 -0.011656
7 -1.079247 -1.375327 -0.007717
7 -1.278291 1.024142 0.001873
8 -3.103377 -0.349280 0.010499
8 0.510437 2.340222 -0.009533
1 1.480254 2.409760 -0.014732
6 2.366155 -0.065067 0.017290
1 2.884161 0.901678 0.050614
8 2.990172 -1.118243 -0.003434
1 -1.572640 -2.273090 -0.010409
rc_5fU/rc_5fU08.xyz
6 1.782926 -0.141228 -0.000002
6 -0.112659 1.159592 -0.000004
6 -0.945603 -0.050021 0.000004
6 -0.293205 -1.305732 0.000006
1 -0.825627 -2.250922 0.000008
7 1.021326 -1.326643 0.000006
7 1.265377 1.042231 -0.000007
8 3.066222 -0.404346 0.000000
8 -0.651184 2.279360 -0.000007
1 3.590406 0.420134 -0.000001
6 -2.424972 0.017074 0.000012
1 -2.871128 1.019007 0.000042

8 -3.097830 -1.006744 -0.000012
1 1.522835 -2.221613 0.000010
rc_5fU/rc_5fU07.xyz
6 -1.814905 0.096726 -0.000006
6 0.336210 0.933060 -0.000004
6 0.873642 -0.442328 0.000008
6 -0.059841 -1.507570 0.000018
1 0.232689 -2.553198 0.000031
7 -1.346255 -1.229054 0.000013
7 -1.036242 1.126345 -0.000012
8 -3.124815 0.133177 -0.000020
8 1.093361 1.913888 -0.000001
1 -3.453779 1.052603 -0.000033
6 2.314608 -0.806908 0.000005
1 2.513377 -1.890786 0.000009
8 3.222023 0.010291 -0.000001
1 -2.037630 -1.986387 0.000022
rc_5fU/rc_5fU10.xyz
6 -1.975170 0.085421 -0.021776
6 0.294325 0.925026 -0.019759
6 0.776826 -0.461710 -0.010379
6 -0.168087 -1.491263 -0.000645
1 0.117683 -2.538273 0.023988
7 -1.469251 -1.225119 -0.002168
7 -1.085574 1.138222 -0.215799
8 -3.174334 0.297839 0.107349
8 1.004377 1.924194 0.109252
1 3.957926 -0.384487 0.000250
6 2.124529 -0.835631 -0.000315
1 2.393548 -1.891966 -0.002212
8 3.076387 0.037208 0.004922
1 -2.151369 -1.981974 0.048798
rc_5fU/rc_5fU11.xyz
6 -1.932645 -0.247556 0.029249
6 0.022651 1.176320 0.025780
6 0.861658 -0.027809 0.019586

6 0.234489 -1.277162 0.002837
1 0.793486 -2.206165 -0.035103
7 -1.089618 -1.374642 0.005941
7 -1.349650 0.987064 0.301649
8 -3.136315 -0.350757 -0.151579
8 0.421947 2.325074 -0.155344
1 3.972844 -0.677057 -0.010974
6 2.253055 0.108655 0.003905
1 2.703678 1.099651 0.009764
8 3.028289 -0.925693 -0.010711
1 -1.541763 -2.287066 -0.063893
rc_5fU/rc_5fU12.xyz
6 1.943864 -0.227521 0.037197
6 -0.036283 1.155107 0.024270
6 -0.864560 -0.064888 0.020858
6 -0.206230 -1.299899 -0.015039
1 -0.725926 -2.250620 -0.070034
7 1.119929 -1.368754 -0.011084
7 1.327123 0.981509 0.345074
8 3.147519 -0.300681 -0.146957
8 -0.441921 2.292813 -0.193531
1 -2.919191 -1.709481 -0.028794
6 -2.254952 0.112769 0.020825
1 -2.641440 1.128931 0.048990
8 -3.195970 -0.772162 0.000464
1 1.589129 -2.271295 -0.096561
rc_5hmC/rc_5hmC02.xyz
6 -2.055352 -0.022155 -0.022574
6 0.223726 0.820177 0.037565
6 0.689917 -0.550211 0.034321
6 -0.312468 -1.557480 -0.010921
1 0.010469 -2.595269 -0.021799
7 -1.606744 -1.340456 -0.038570
7 -1.116246 1.010785 0.009448
8 -3.252333 0.250735 -0.039632
6 2.116797 -0.955965 0.175473

1 2.287105 -1.156574 1.248198
1 2.268234 -1.908369 -0.350959
7 1.043889 1.845600 0.074587
1 0.695373 2.800838 0.092351
1 2.047869 1.658274 0.038846
8 3.012139 0.040731 -0.300419
1 3.881007 -0.107968 0.102064
1 -1.490520 1.959641 0.010272
rc_5hmC/rc_5hmC01.xyz
6 -2.052003 -0.021541 -0.035718
6 0.224778 0.819317 0.055666
6 0.690940 -0.552405 0.060973
6 -0.309275 -1.558848 0.002215
1 0.012640 -2.596929 -0.002784
7 -1.604125 -1.340915 -0.045099
7 -1.113602 1.011786 0.008451
8 -3.248459 0.250702 -0.069730
6 2.130209 -0.944583 0.226322
1 2.308979 -1.081091 1.303048
1 2.290442 -1.918825 -0.250555
7 1.047346 1.841670 0.104857
1 0.702729 2.798540 0.114654
1 2.051326 1.653363 0.080861
8 3.072021 0.020098 -0.228656
1 3.118048 -0.021622 -1.197025
1 -1.487896 1.960729 0.004669
rc_5hmC/rc_5hmC03.xyz
6 -1.944242 -0.448895 0.001910
6 -0.074134 1.099789 0.000608
6 0.817775 -0.042104 -0.010179
6 0.209646 -1.321801 -0.024619
1 0.855739 -2.193036 -0.042707
7 -1.087924 -1.544086 -0.019714
7 -1.399516 0.838771 0.004796
8 -3.164532 -0.583889 0.017188
6 2.303285 0.146805 -0.011198

1 2.580950 0.738658 -0.894758
1 2.586300 0.744776 0.870058
7 0.358492 2.341574 0.005014
1 -0.292857 3.122607 0.010989
1 1.349829 2.559787 0.002842
8 3.036064 -1.061378 -0.071837
1 3.045090 -1.476325 0.804838
1 -2.068647 1.609095 0.016123
rc_5hmC/rc_5hmC04.xyz
6 1.946085 -0.445964 -0.000187
6 0.070119 1.097672 0.000000
6 -0.816478 -0.047798 0.000074
6 -0.204156 -1.326874 0.000008
1 -0.845261 -2.201126 0.000064
7 1.094190 -1.543820 -0.000121
7 1.396112 0.839455 -0.000095
8 3.167321 -0.575107 -0.000323
6 -2.290446 0.136838 0.000174
1 -2.571027 0.735341 0.884515
1 -2.571150 0.735125 -0.884277
7 -0.362460 2.339597 0.000017
1 0.289232 3.120183 -0.000049
1 -1.353595 2.558272 0.000090
8 -2.946655 -1.116772 0.000378
1 -3.901604 -0.954838 0.000341
1 2.062438 1.612194 -0.000151
rc_5hmC/rc_5hmC05.xyz
6 1.998719 -0.183154 0.091293
6 -0.141163 0.946979 -0.129489
6 -0.763115 -0.352144 -0.298502
6 0.097548 -1.478418 -0.221634
1 -0.348494 -2.464167 -0.325842
7 1.400008 -1.433338 -0.048731
7 1.198305 0.961675 0.045969
8 3.208409 -0.062500 0.255046
6 -2.245923 -0.541311 -0.472926

1 -2.687461 0.225869 -1.116955
1 -2.425660 -1.515009 -0.930499
7 -0.813432 2.076819 -0.151357
1 -0.342085 2.970381 -0.031441
1 -1.821139 2.090740 -0.271444
8 -2.867722 -0.572203 0.821429
1 -2.955951 0.331774 1.164711
1 1.684734 1.850240 0.166044
rc_5hmC/rc_5hmC08.xyz
6 2.025847 0.162132 -0.008745
6 -0.194041 0.856629 0.017021
6 -0.694041 -0.523478 0.012998
6 0.293493 -1.533709 -0.016006
1 0.037332 -2.588442 -0.026764
7 1.564535 -1.203226 -0.026807
7 1.110215 1.144419 0.009177
8 3.249317 0.321241 -0.014046
6 -2.107378 -0.969737 0.118241
1 -2.243682 -1.853982 -0.525628
1 -2.246161 -1.333420 1.154956
7 -1.064922 1.858913 0.040755
1 -0.719634 2.812787 0.059543
1 -2.063877 1.667175 0.022969
8 -3.042968 0.040799 -0.196204
1 -3.910898 -0.224682 0.143699
1 2.284044 -1.927522 -0.049704
rc_5hmC/rc_5hmC09.xyz
6 -1.971357 -0.249381 0.000000
6 -0.095408 1.121604 0.000001
6 0.814480 -0.030227 0.000001
6 0.209471 -1.304658 0.000000
1 0.789314 -2.219447 0.000001
7 -1.102488 -1.396135 -0.000001
7 -1.420590 0.978337 0.000000
8 -3.181547 -0.487738 -0.000004
6 2.287322 0.117786 0.000002

1 2.577111 0.716244 -0.883039
1 2.577110 0.716243 0.883044
7 0.405452 2.352509 0.000000
1 -0.232469 3.141297 -0.000001
1 1.399388 2.544803 0.000000
8 2.921757 -1.144662 0.000001
1 3.879982 -1.001537 0.000001
1 -1.555780 -2.312128 -0.000003
rc_5hmC/rc_5hmC06.xyz
6 1.968680 -0.251981 0.017751
6 0.099192 1.125309 -0.006254
6 -0.816600 -0.021826 -0.030413
6 -0.215802 -1.297485 -0.077740
1 -0.801991 -2.207613 -0.126056
7 1.094945 -1.394727 -0.061420
7 1.423420 0.978063 0.031722
8 3.175970 -0.498419 0.064475
6 -2.302644 0.126955 -0.013929
1 -2.585883 0.757206 0.844816
1 -2.604143 0.679472 -0.916668
7 -0.401819 2.355942 -0.017165
1 0.234624 3.145948 0.002137
1 -1.396018 2.546243 -0.040157
8 -3.004106 -1.100328 -0.009794
1 -2.979488 -1.480274 0.883188
1 1.545213 -2.311788 -0.093159
rc_5hmC/rc_5hmC07.xyz
6 -2.020762 0.166584 -0.030275
6 0.197536 0.854756 0.047496
6 0.693798 -0.528366 0.055915
6 -0.295089 -1.537757 0.006063
1 -0.041230 -2.593026 0.000004
7 -1.564021 -1.203031 -0.033823
7 -1.104421 1.147560 0.008867
8 -3.242808 0.326294 -0.068736
6 2.123667 -0.950847 0.214834

1 2.287249 -1.116853 1.291748
1 2.264982 -1.923103 -0.274939
7 1.075841 1.847950 0.102518
1 0.742815 2.806247 0.119588
1 2.072950 1.644670 0.082315
8 3.090611 -0.007059 -0.214368
1 3.130904 -0.021495 -1.183991
1 -2.286794 -1.923897 -0.077024
rc_5hmC/rc_5hmC10.xyz
6 -1.985483 0.002386 0.084492
6 0.111546 0.986640 -0.104599
6 0.768201 -0.312061 -0.299246
6 -0.065705 -1.448776 -0.195077
1 0.326644 -2.457977 -0.269200
7 -1.357305 -1.293645 -0.022588
7 -1.208932 1.099511 0.050684
8 -3.211416 -0.004405 0.210093
6 2.244431 -0.517626 -0.511173
1 2.412244 -1.462743 -1.028934
1 2.700621 0.284935 -1.096376
7 0.841820 2.097257 -0.101367
1 0.380939 2.991514 0.028802
1 1.849144 2.092843 -0.199001
8 2.837355 -0.641513 0.790589
1 2.930694 0.239669 1.189471
1 -1.974821 -2.106141 0.036295
rc_5hmC/rc_5hmC12.xyz
6 1.918382 0.007490 -0.009214
6 -0.203966 0.817418 0.008994
6 -0.722293 -0.535820 0.010128
6 0.266837 -1.558239 -0.029619
1 -0.049680 -2.597347 -0.050642
7 1.558630 -1.321502 -0.042103
7 1.128550 1.057376 0.001961
8 3.233329 0.176099 0.009207
6 -2.148460 -0.944338 0.109376

1 -2.317110 -1.789233 -0.577001
1 -2.298551 -1.345571 1.128020
7 -1.019386 1.860061 0.020235
1 -0.632259 2.800152 0.034549
1 -2.028842 1.707937 0.012766
8 -3.050418 0.116278 -0.155182
1 -3.927617 -0.131112 0.173728
1 3.453214 1.125551 0.047739
rc_5hmC/rc_5hmC14.xyz
6 -1.914651 0.063211 -0.007868
6 0.219878 0.826838 0.015230
6 0.714085 -0.536304 0.012253
6 -0.293188 -1.540567 -0.018194
1 0.000365 -2.586557 -0.033125
7 -1.578955 -1.273629 -0.028276
7 -1.106136 1.097328 0.006010
8 -3.213124 0.331939 -0.005640
6 2.132621 -0.972289 0.099730
1 2.281438 -1.383033 1.114947
1 2.279781 -1.816931 -0.592182
7 1.057406 1.852554 0.032019
1 0.688335 2.799843 0.049361
1 2.063632 1.680835 0.022929
8 3.053291 0.071360 -0.164823
1 3.930180 -0.201445 0.144174
1 -3.723735 -0.498208 -0.017568
rc_5hmC/rc_5hmC11.xyz
6 -1.914761 0.010224 -0.026856
6 0.204852 0.814917 0.057581
6 0.723376 -0.540586 0.061374
6 -0.264511 -1.561801 0.013711
1 0.049351 -2.601699 0.007665
7 -1.555974 -1.321971 -0.030491
7 -1.125436 1.059644 0.012119
8 -3.227324 0.178108 -0.079828
6 2.166814 -0.922854 0.193718

1 2.352951 -1.121876 1.259851
1 2.333890 -1.868091 -0.337804
7 1.026387 1.849448 0.110316
1 0.649636 2.793921 0.130719
1 2.035061 1.687498 0.101140
8 3.098333 0.072157 -0.212387
1 3.150066 0.070626 -1.181543
1 -3.448492 1.128256 -0.083084
rc_5hmC/rc_5hmC13.xyz
6 1.911626 0.066132 -0.026768
6 -0.220364 0.824905 0.046275
6 -0.714762 -0.540653 0.056498
6 0.290508 -1.543416 -0.003274
1 -0.000640 -2.589810 -0.018473
7 1.575885 -1.273303 -0.048258
7 1.103470 1.100014 0.004515
8 3.208719 0.333006 -0.049071
6 -2.148785 -0.948819 0.211514
1 -2.304148 -1.905375 -0.302882
1 -2.317451 -1.134770 1.283028
7 -1.064663 1.841873 0.092839
1 -0.707638 2.794092 0.109383
1 -2.069694 1.657912 0.085054
8 -3.104627 0.021802 -0.197064
1 -3.164942 0.007570 -1.165607
1 3.719588 -0.497064 -0.070565
rc_5hmC/rc_5hmC15.xyz
6 -1.834617 -0.342844 0.001541
6 -0.079778 1.092213 0.001266
6 0.840947 -0.032719 -0.011020
6 0.230310 -1.314012 -0.025136
1 0.855337 -2.199712 -0.043265
7 -1.072868 -1.492303 -0.020068
7 -1.418220 0.904937 0.009074
8 -3.131544 -0.600704 0.013651
6 2.324755 0.150510 -0.013089

1 2.603905 0.739526 -0.898107
1 2.609800 0.751751 0.865356
7 0.369387 2.336242 0.004743
1 -0.294033 3.107007 0.012058
1 1.359512 2.561638 0.001219
8 3.057631 -1.059081 -0.069534
1 3.060523 -1.473897 0.807212
1 -3.641536 0.230950 0.024980
rc_5hmC/rc_5hmC17.xyz
6 1.835950 -0.340777 -0.000004
6 0.076255 1.090456 0.000003
6 -0.839268 -0.038567 0.000004
6 -0.224847 -1.319144 0.000037
1 -0.845456 -2.207357 0.000070
7 1.078934 -1.492555 0.000036
7 1.414916 0.905543 -0.000003
8 3.134297 -0.593087 -0.000035
6 -2.311685 0.140438 -0.000005
1 -2.593654 0.739387 0.883660
1 -2.593656 0.739423 -0.883641
7 -0.372980 2.334464 0.000010
1 0.290881 3.104562 0.000019
1 -1.362821 2.560298 0.000045
8 -2.968915 -1.113462 -0.000028
1 -3.923789 -0.951156 -0.000099
1 3.640906 0.240633 -0.000054
rc_5hmC/rc_5hmC16.xyz
6 -1.842132 -0.282838 0.007451
6 -0.057847 1.108276 -0.009083
6 0.840317 -0.035603 -0.035152
6 0.201782 -1.301954 -0.098244
1 0.805815 -2.200060 -0.161234
7 -1.105418 -1.447227 -0.087130
7 -1.399078 0.953965 0.025481
8 -3.153704 -0.437916 0.083215
6 2.328854 0.108544 -0.011078

1 2.644309 0.647659 -0.915880
1 2.612662 0.740779 0.845351
7 0.416538 2.343179 -0.015444
1 -0.232998 3.125778 0.001583
1 1.410577 2.548850 -0.034593
8 3.026195 -1.123010 0.008349
1 2.951877 -1.517846 0.891744
1 -3.382312 -1.385731 0.056793
rc_5hmC/rc_5hmC18.xyz
6 1.845662 -0.281671 0.000000
6 0.055509 1.104173 -0.000001
6 -0.837268 -0.044033 0.000001
6 -0.195646 -1.311118 0.000001
1 -0.794756 -2.214008 -0.000001
7 1.112211 -1.451300 0.000000
7 1.397289 0.953302 -0.000006
8 3.160887 -0.427066 0.000002
6 -2.313524 0.101826 0.000002
1 -2.608752 0.694610 0.883627
1 -2.608752 0.694616 -0.883620
7 -0.419440 2.338981 0.000004
1 0.230709 3.120921 -0.000001
1 -1.413326 2.545493 -0.000003
8 -2.943076 -1.166088 -0.000003
1 -3.901368 -1.024543 0.000005
1 3.394943 -1.373797 0.000002
rc_5hmC/rc_5hmC19.xyz
6 1.865376 -0.125322 0.072916
6 -0.123208 0.944123 -0.132923
6 -0.794647 -0.331638 -0.306737
6 0.051102 -1.470502 -0.240959
1 -0.385625 -2.459020 -0.351711
7 1.352183 -1.400118 -0.068274
7 1.214981 1.017257 0.048021
8 3.175172 -0.132089 0.252385
6 -2.281028 -0.499926 -0.466430

1 -2.729539 0.291336 -1.074786
1 -2.484118 -1.457848 -0.947591
7 -0.798389 2.081303 -0.153749
1 -0.301635 2.960512 -0.030963
1 -1.806101 2.117013 -0.270601
8 -2.871105 -0.561803 0.842602
1 -2.925818 0.333018 1.216415
1 3.513303 0.776624 0.362153
rc_5hmC/rc_5hmC20.xyz
6 1.869502 -0.078011 0.071846
6 -0.133392 0.961028 -0.113482
6 -0.789382 -0.322315 -0.298205
6 0.066483 -1.452214 -0.209435
1 -0.358337 -2.447804 -0.302215
7 1.366070 -1.361332 -0.036144
7 1.204417 1.055062 0.053066
8 3.180338 0.017694 0.216224
6 -2.268962 -0.513075 -0.496263
1 -2.719405 0.289301 -1.088172
1 -2.441988 -1.458268 -1.013056
7 -0.826151 2.088884 -0.110526
1 -0.340368 2.972876 0.020924
1 -1.835639 2.111817 -0.214017
8 -2.884185 -0.630317 0.796569
1 -2.942121 0.248326 1.206226
1 3.592783 -0.866045 0.216419
rc_5hmU/rc_5hmU01.xyz
6 -2.023455 0.097047 0.016064
6 0.308327 0.878426 -0.026302
6 0.755786 -0.521277 -0.012665
6 -0.224600 -1.530229 -0.014901
1 0.035829 -2.584228 -0.023542
7 -1.511582 -1.223714 0.004247
7 -1.061710 1.075052 -0.004749
8 -3.222596 0.299691 0.040560
8 1.088185 1.833525 -0.057435

1 -1.394793 2.038975 -0.008545
6 2.179551 -0.913073 -0.056958
1 2.353138 -1.736011 0.657582
1 2.324878 -1.402638 -1.048843
8 3.157336 0.074896 0.134174
1 2.751099 0.959763 0.039959
1 -2.214167 -1.965482 0.017089
rc_5hmU/rc_5hmU04.xyz
6 -1.910099 -0.002580 0.019480
6 0.326278 0.862590 -0.035410
6 0.774643 -0.523068 -0.020030
6 -0.221575 -1.535491 -0.025168
1 0.083879 -2.578012 -0.040774
7 -1.520498 -1.297797 0.002448
7 -1.061137 1.032343 0.003051
8 -3.176800 0.295509 0.051543
8 1.072036 1.843954 -0.082618
1 -1.426616 1.986689 0.000993
6 2.204126 -0.908299 -0.074177
1 2.376784 -1.757365 0.603488
1 2.353185 -1.334323 -1.092114
8 3.174421 0.078113 0.188231
1 2.807282 0.959192 -0.011310
1 -3.720559 -0.517522 0.055811
rc_5hmU/rc_5hmU06.xyz
6 -1.916535 -0.041196 0.019798
6 0.316646 0.853581 -0.053013
6 0.784241 -0.522533 -0.028283
6 -0.200791 -1.546587 -0.048061
1 0.120768 -2.584100 -0.074607
7 -1.502533 -1.330757 -0.017948
7 -1.075958 1.002378 -0.007791
8 -3.206794 0.126895 0.087440
8 1.042313 1.848613 -0.112278
1 -1.435918 1.959357 -0.005972
6 2.221678 -0.884167 -0.062251

1 2.397890 -1.732875 0.613838
1 2.396017 -1.297819 -1.080720
8 3.168226 0.118216 0.228914
1 2.805974 0.990248 -0.012455
1 -3.496685 1.059464 0.138339
rc_5hmU/rc_5hmU02.xyz
6 -1.983367 0.102592 0.086258
6 0.182326 0.871112 -0.128101
6 0.726396 -0.473306 -0.230707
6 -0.211852 -1.523439 -0.131629
1 0.080605 -2.567373 -0.173979
7 -1.488713 -1.237376 0.009357
7 -1.093419 1.134457 0.010417
8 -3.193892 0.248215 0.216733
8 1.069630 1.844317 -0.215607
1 0.640944 2.720433 -0.163987
6 2.193976 -0.726666 -0.408681
1 2.375711 -1.796429 -0.524170
1 2.549741 -0.207374 -1.306230
8 2.888728 -0.293518 0.764548
1 3.051526 0.661348 0.702563
1 -2.184205 -1.984041 0.079152
rc_5hmU/rc_5hmU09.xyz
6 -2.012460 -0.100650 0.091097
6 0.193394 0.860156 -0.139456
6 0.731830 -0.475402 -0.238993
6 -0.204419 -1.536909 -0.143108
1 0.168852 -2.555347 -0.203011
7 -1.502969 -1.386837 0.009558
7 -1.120804 0.996959 0.012249
8 -3.206219 0.128773 0.228033
8 1.007901 1.867342 -0.218544
1 0.586400 2.748854 -0.149665
6 2.206296 -0.703550 -0.407692
1 2.390675 -1.770278 -0.544819
1 2.565972 -0.167166 -1.293434

8 2.900758 -0.293094 0.774482
1 3.085028 0.657734 0.721293
1 -1.537885 1.929319 0.074134
rc_5hmU/rc_5hmU05.xyz
6 -1.879026 0.130610 0.002115
6 0.295354 0.877296 -0.007231
6 0.775589 -0.513193 -0.004147
6 -0.191486 -1.530582 -0.005827
1 0.057163 -2.586729 -0.007543
7 -1.477321 -1.202143 -0.002661
7 -1.058800 1.132777 -0.001630
8 -3.189442 0.246685 0.011754
8 1.113518 1.819470 -0.014636
1 -3.451991 1.187043 0.016418
6 2.200920 -0.894042 -0.005878
1 2.374216 -1.590643 0.840309
1 2.374903 -1.541317 -0.892405
8 3.161833 0.125331 0.026097
1 2.699496 0.993163 0.013919
1 -2.196309 -1.928377 -0.000581
rc_5hmU/rc_5hmU08.xyz
6 -1.990440 -0.158776 0.099648
6 0.158361 0.922498 -0.148845
6 0.762753 -0.379845 -0.311376
6 -0.122888 -1.490011 -0.234008
1 0.301844 -2.484225 -0.344645
7 -1.422203 -1.414971 -0.051547
7 -1.157328 0.978595 0.042333
8 -3.190292 0.002446 0.280048
8 0.774166 2.066441 -0.181972
1 1.734043 2.009156 -0.345934
6 2.246930 -0.576492 -0.447002
1 2.443946 -1.583802 -0.814569
1 2.696018 0.141482 -1.141346
8 2.819978 -0.478846 0.864982
1 2.927735 0.457095 1.100550

1 -1.605972 1.890352 0.155477
rc_5hmU/rc_5hmU03.xyz
6 -1.973343 0.023870 0.093608
6 0.133547 0.961924 -0.125814
6 0.760427 -0.339407 -0.313855
6 -0.102498 -1.456127 -0.214841
1 0.264955 -2.474087 -0.294873
7 -1.391163 -1.274103 -0.034479
7 -1.159076 1.114184 0.042482
8 -3.189724 0.081606 0.249130
8 0.831447 2.087184 -0.136158
1 1.785306 1.968501 -0.277829
6 2.238360 -0.564069 -0.483121
1 2.418494 -1.556231 -0.897252
1 2.706714 0.179603 -1.134836
8 2.790347 -0.550182 0.842947
1 2.908625 0.367945 1.140825
1 -2.027936 -2.072306 0.024714
rc_5hmU/rc_5hmU07.xyz
6 1.880818 0.162466 -0.006989
6 -0.306034 0.879543 0.021345
6 -0.774301 -0.514405 0.010048
6 0.203004 -1.518550 0.031456
1 -0.031169 -2.577985 0.045347
7 1.487183 -1.175992 0.030605
7 1.042403 1.150889 -0.002472
8 3.172093 0.420838 -0.047864
8 -1.136305 1.812475 0.050097
1 3.743976 -0.368521 -0.066874
6 -2.194501 -0.911129 -0.017059
1 -2.344332 -1.598353 -0.875965
1 -2.377972 -1.574002 0.856077
8 -3.165362 0.097748 -0.054430
1 -2.712058 0.969817 -0.012762
1 2.197122 -1.911278 0.042018
rc_5hmU/rc_5hmU11.xyz

6 -1.845115 -0.109448 0.069453
6 0.149102 0.917986 -0.151640
6 0.796796 -0.368845 -0.322456
6 -0.069347 -1.497224 -0.236558
1 0.348364 -2.494720 -0.337380
7 -1.367132 -1.391826 -0.067342
7 -1.155956 1.035246 0.026480
8 -3.136558 -0.062743 0.269330
8 0.921008 1.975454 -0.195294
1 0.410300 2.803136 -0.086542
6 2.280651 -0.505026 -0.464362
1 2.540628 -1.475318 -0.887576
1 2.714673 0.295729 -1.065699
8 2.766316 -0.482249 0.891138
1 2.815458 0.440616 1.194130
1 -3.446457 0.858261 0.381091
rc_5hmU/rc_5hmU10.xyz
6 -1.848427 -0.052085 0.072547
6 0.168072 0.924794 -0.136003
6 0.786468 -0.375285 -0.316862
6 -0.100525 -1.485507 -0.212210
1 0.294849 -2.493372 -0.299220
7 -1.394906 -1.346967 -0.040325
7 -1.132826 1.075318 0.038297
8 -3.135116 0.110440 0.239317
8 0.967769 1.962992 -0.169727
1 0.477995 2.801784 -0.050330
6 2.264375 -0.541263 -0.486618
1 2.497908 -1.517596 -0.911475
1 2.702131 0.249715 -1.098084
8 2.774298 -0.524897 0.860126
1 2.848220 0.397167 1.161715
1 -3.602363 -0.748366 0.248733
rc_5hmU/rc_5hmU13.xyz
6 -1.852732 -0.109814 0.067966
6 0.136901 0.940948 -0.140283

6 0.793057 -0.346276 -0.319455
6 -0.069409 -1.481205 -0.227423
1 0.359270 -2.474749 -0.322643
7 -1.366473 -1.390662 -0.059817
7 -1.171529 1.036274 0.030105
8 -3.146861 -0.070318 0.254107
8 0.782974 2.084041 -0.154335
1 1.745030 2.002292 -0.280053
6 2.275080 -0.525014 -0.471665
1 2.494244 -1.510024 -0.884133
1 2.744901 0.236600 -1.100571
8 2.777490 -0.502921 0.876465
1 2.878264 0.418513 1.171565
1 -3.461896 0.849833 0.359079
rc_5hmU/rc_5hmU12.xyz
6 -1.855019 -0.056657 0.071111
6 0.154221 0.952379 -0.123656
6 0.785531 -0.346269 -0.314920
6 -0.094467 -1.466102 -0.206948
1 0.315573 -2.468459 -0.292117
7 -1.388734 -1.347916 -0.036298
7 -1.151220 1.075015 0.043941
8 -3.145261 0.093297 0.225558
8 0.823664 2.083352 -0.127020
1 1.783825 1.986142 -0.254685
6 2.260500 -0.555770 -0.494506
1 2.452968 -1.541932 -0.917230
1 2.735419 0.201918 -1.124158
8 2.782844 -0.554754 0.846862
1 2.902550 0.361407 1.153208
1 -3.605230 -0.769423 0.231794
rc_5hmU/rc_5hmU14.xyz
6 1.774035 0.026541 -0.021525
6 -0.317148 0.864264 0.030231
6 -0.799747 -0.513585 0.023247
6 0.178359 -1.539832 0.012751

1 -0.123573 -2.582833 0.021189
7 1.477609 -1.285824 -0.022391
7 1.056721 1.088248 -0.004322
8 3.180590 0.246896 -0.096407
8 -1.099479 1.836209 0.073235
1 3.499996 1.122475 0.222241
6 -2.235883 -0.882918 0.089459
1 -2.423624 -1.730802 -0.585072
1 -2.389901 -1.295932 1.109837
8 -3.189876 0.122037 -0.174990
1 -2.775278 0.994872 -0.029672
1 3.744490 -0.492721 0.228793
rc_5mC/rc_5mC01.xyz
6 1.678924 -0.018513 0.000002
6 -0.602075 0.805533 -0.000004
6 -1.065872 -0.569008 -0.000008
6 -0.054659 -1.566933 -0.000008
1 -0.369291 -2.606875 -0.000015
7 1.240269 -1.338693 -0.000002
7 0.733179 1.011141 0.000002
8 2.874094 0.263808 0.000005
7 -1.432401 1.823712 -0.000003
1 -1.092666 2.782476 0.000000
1 -2.436353 1.676570 -0.000020
1 1.101526 1.962503 0.000001
6 -2.508833 -0.898220 0.000009
1 -3.004359 -0.468573 0.881783
1 -3.004429 -0.468455 -0.881665
1 -2.659421 -1.978392 -0.000052
rc_5mC/rc_5mC02.xyz
6 -1.537722 -0.002545 -0.000026
6 0.574771 0.817394 0.000003
6 1.109811 -0.535970 0.000010
6 0.124792 -1.563027 0.000014
1 0.446954 -2.600111 0.000022
7 -1.169421 -1.331841 0.000000

7 -0.755928 1.055030 -0.000013
8 -2.851991 0.155926 -0.000014
7 1.387822 1.859423 0.000006
1 1.001598 2.800084 -0.000001
1 2.397090 1.754567 0.000013
1 -3.081402 1.104220 -0.000016
6 2.559417 -0.832401 0.000015
1 3.044450 -0.388334 -0.880592
1 3.044453 -0.388308 0.880606
1 2.739062 -1.908514 0.000030
rc_5mC/rc_5mC03.xyz
6 1.533378 0.067883 -0.000029
6 -0.601361 0.813247 0.000172
6 -1.093866 -0.556997 -0.000069
6 -0.077852 -1.552464 -0.000288
1 -0.363100 -2.600297 -0.000467
7 1.206639 -1.273615 -0.000266
7 0.719190 1.099353 0.000168
8 2.828397 0.342670 0.000003
7 -1.451310 1.826103 0.000402
1 -1.097275 2.779397 0.000530
1 -2.456244 1.684688 0.000437
1 3.344653 -0.484508 -0.000128
6 -2.532917 -0.898671 -0.000131
1 -3.031651 -0.470854 0.880741
1 -3.031586 -0.470729 -0.880982
1 -2.677902 -1.979932 -0.000216
rc_5mC/rc_5mC04.xyz
6 -1.679911 0.096383 -0.000001
6 0.533842 0.704659 0.000004
6 0.970606 -0.647481 0.000000
6 -0.098933 -1.592407 0.000014
1 0.145908 -2.651313 0.000025
7 -1.367623 -1.261615 0.000017
7 -0.699185 1.098763 0.000005
8 -2.871103 0.445568 -0.000022

7 1.545544 1.770236 0.000009
1 1.093250 2.694121 0.000012
1 2.154546 1.706942 -0.829862
1 2.154545 1.706932 0.829880
6 2.398167 -1.042133 -0.000019
1 2.910585 -0.629796 -0.880251
1 2.910638 -0.629719 0.880146
1 2.505581 -2.127523 0.000023
rc_5mU/rc_5mU01.xyz
6 -1.648993 0.063788 0.000420
6 0.661895 0.913517 -0.001201
6 1.152566 -0.485903 -0.002754
6 0.192429 -1.522847 -0.001533
1 0.480166 -2.568920 -0.001866
7 -1.100132 -1.247494 -0.000339
1 -1.786109 -2.005867 0.000076
7 -0.719021 1.067667 -0.000150
8 -2.854774 0.223591 0.001481
8 1.411680 1.882041 -0.000492
1 -1.083838 2.019812 0.000735
6 2.597263 -0.748755 0.000747
1 3.072697 -0.225117 -0.840530
1 3.046444 -0.309731 0.904626
1 2.818498 -1.815242 -0.041601
rc_5mU/rc_5mU04.xyz
6 -1.531867 -0.034647 0.000384
6 0.681640 0.897219 -0.000983
6 1.174910 -0.484967 -0.002731
6 0.202492 -1.523380 -0.001274
1 0.537108 -2.556394 -0.001672
7 -1.103086 -1.321484 0.000194
1 -3.325977 -0.607604 0.000594
7 -0.715983 1.023874 0.000292
8 -2.807416 0.221671 0.000704
8 1.395184 1.894311 -0.000387
1 -1.112961 1.965415 0.000726

6 2.624759 -0.739612 0.000341
1 3.100882 -0.218575 -0.841240
1 3.074615 -0.308710 0.906801
1 2.846063 -1.806400 -0.045575
rc_5mU/rc_5mU02.xyz
6 -1.632572 0.153829 -0.000046
6 0.576190 0.821997 -0.000024
6 1.075867 -0.547069 0.000025
6 0.079684 -1.550824 0.000072
1 0.324583 -2.607519 0.000123
7 -1.191088 -1.204699 0.000051
1 -1.921215 -1.920010 0.000088
7 -0.692836 1.144724 -0.000059
8 -2.842677 0.353533 -0.000030
8 1.507836 1.757407 -0.000040
1 1.101670 2.645640 -0.000062
6 2.513874 -0.856184 0.000034
1 2.993419 -0.392703 -0.874340
1 2.993430 -0.392622 0.874357
1 2.696050 -1.930973 0.000080
rc_5mU/rc_5mU06.xyz
6 1.537178 -0.077901 0.002029
6 -0.665435 0.900439 0.007598
6 -1.188243 -0.468047 0.001929
6 -0.236674 -1.524705 0.015596
1 -0.594024 -2.550143 0.025654
7 1.071906 -1.354031 0.015938
1 3.183886 0.935722 -0.046300
7 0.738754 0.997269 0.013477
8 2.834968 0.023626 -0.023089
8 -1.353025 1.915156 0.006734
1 1.139460 1.937725 0.009858
6 -2.643534 -0.691470 -0.021147
1 -3.121125 -0.159722 0.812647
1 -3.070358 -0.253088 -0.935087
1 -2.887757 -1.753314 0.022125

rc_5mU/rc_5mU07.xyz
6 -1.672403 -0.039451 -0.000011
6 0.587438 0.815182 -0.000110
6 1.081511 -0.543433 -0.000738
6 0.080138 -1.558878 0.000177
1 0.411220 -2.593486 0.000513
7 -1.215962 -1.348189 0.000614
1 -1.093039 1.965315 -0.000143
7 -0.729299 1.009655 -0.000209
8 -2.864287 0.242321 -0.000285
8 1.322415 1.888112 0.000435
1 2.283141 1.716712 0.000820
6 2.527412 -0.846513 -0.000524
1 3.016610 -0.388698 -0.872880
1 3.009335 -0.411970 0.887933
1 2.699957 -1.923040 -0.013047
rc_5mU/rc_5mU10.xyz
6 1.669187 -0.043044 0.000206
6 -0.592743 0.806116 -0.000496
6 -1.080565 -0.552118 -0.001349
6 -0.081214 -1.566816 -0.000876
1 -0.409106 -2.602362 -0.001178
7 1.215122 -1.351325 0.000043
1 1.098652 1.960781 -0.000030
7 0.722395 1.009769 -0.000228
8 2.859222 0.243752 0.000740
8 -1.456791 1.775751 -0.000183
1 -1.063447 2.671853 0.000118
6 -2.528391 -0.834189 0.000421
1 -2.996432 -0.396572 0.893594
1 -3.011212 -0.350806 -0.859788
1 -2.718164 -1.907725 -0.023312
rc_5mU/rc_5mU03.xyz
6 1.637903 0.161678 -0.007059
6 -0.573429 0.836682 0.006890
6 -1.067579 -0.537285 -0.001502

6 -0.067958 -1.539506 0.023974
1 -0.315106 -2.595666 0.039586
7 1.202057 -1.196806 0.026262
1 1.931744 -1.912507 0.042329
7 0.699403 1.150112 -0.008478
8 2.848608 0.364013 -0.028896
8 -1.410721 1.862898 0.023553
1 -2.348726 1.605945 0.053298
6 -2.501191 -0.879427 -0.036581
1 -3.012605 -0.456380 0.842056
1 -2.979510 -0.424313 -0.916857
1 -2.655588 -1.958366 -0.056484
rc_5mU/rc_5mU05.xyz
6 1.508935 0.095515 0.000552
6 -0.639695 0.926437 -0.001782
6 -1.180024 -0.456333 -0.003665
6 -0.240454 -1.512088 -0.002210
1 -0.527678 -2.557730 -0.003416
7 1.051975 -1.228835 -0.000353
1 1.745551 -1.980922 -0.000437
7 0.732573 1.126789 0.000121
8 2.823604 0.143616 0.001796
8 -1.414602 1.889941 -0.000877
1 3.136719 1.068094 0.002295
6 -2.629237 -0.701352 0.001304
1 -3.068380 -0.272032 0.914317
1 -3.102019 -0.160209 -0.829259
1 -2.865205 -1.764413 -0.054418
rc_5mU/rc_5mU08.xyz
6 1.511381 0.132592 0.000652
6 -0.655964 0.921783 -0.002518
6 -1.177657 -0.466805 -0.003095
6 -0.220757 -1.504055 -0.004170
1 -0.487045 -2.555322 -0.006625
7 1.068461 -1.199376 -0.003201
1 1.755569 -1.957580 -0.004988

7 0.710415 1.145566 0.000390
8 2.813510 0.333135 0.004617
8 -1.447303 1.873641 -0.003288
1 3.347251 -0.482888 0.006435
6 -2.621603 -0.740559 0.004591
1 -3.068778 -0.317390 0.916445
1 -3.106548 -0.213252 -0.827865
1 -2.834644 -1.808842 -0.047114
rc_5mU/rc_5mU09.xyz
6 1.522906 0.048531 0.000203
6 -0.590855 0.816972 -0.000541
6 -1.103979 -0.545516 -0.001716
6 -0.094089 -1.557189 -0.000709
1 -0.390448 -2.601732 -0.000819
7 1.189787 -1.285172 0.000351
1 3.333716 -0.486514 0.000432
7 0.699785 1.100130 0.000003
8 2.800113 0.332821 0.000340
8 -1.485082 1.774900 -0.000120
1 -1.061180 2.656680 0.000616
6 -2.542990 -0.841029 -0.000060
1 -3.012558 -0.401833 0.893188
1 -3.030717 -0.349025 -0.853924
1 -2.732020 -1.914670 -0.026798
rc_5mU/rc_5mU13.xyz
6 1.532994 -0.010910 0.003603
6 -0.561316 0.834692 0.007463
6 -1.110754 -0.517577 -0.006209
6 -0.127520 -1.558277 0.020155
1 -0.462035 -2.591268 0.033047
7 1.164120 -1.334951 0.029978
1 3.060385 1.109552 -0.064613
7 0.741572 1.062798 0.007251
8 2.830445 0.159596 -0.029573
8 -1.319864 1.906545 0.017949
1 -2.276985 1.723212 0.019307

6 -2.554533 -0.800330 -0.033058
1 -3.038163 -0.382522 0.864614
1 -3.028978 -0.297073 -0.889374
1 -2.751937 -1.871547 -0.082309
rc_5mU/rc_5mU12.xyz
6 1.528945 0.057759 -0.000001
6 -0.586958 0.831839 0.000000
6 -1.096223 -0.537042 0.000001
6 -0.082572 -1.547584 0.000001
1 -0.380772 -2.591678 0.000002
7 1.199991 -1.278076 0.000000
1 3.340803 -0.475694 -0.000002
7 0.706228 1.106588 -0.000001
8 2.806372 0.343086 -0.000003
8 -1.384250 1.876262 0.000001
1 -2.333108 1.653999 0.000003
6 -2.530751 -0.864661 0.000002
1 -3.024489 -0.419982 0.878283
1 -3.024489 -0.419985 -0.878281
1 -2.693100 -1.942884 0.000004
rc_5mU/rc_5mU14.xyz
6 -1.395563 -0.006392 -0.010054
6 0.667148 0.909311 -0.002645
6 1.204840 -0.457970 0.007522
6 0.260647 -1.516415 -0.007036
1 0.599157 -2.547629 -0.006954
7 -1.048211 -1.309163 -0.021706
7 -0.722491 1.082078 -0.006737
8 -2.814321 0.152617 -0.051039
8 1.408807 1.903118 -0.005848
1 -3.338460 -0.598446 0.312915
1 -3.159286 1.023229 0.254525
6 2.662785 -0.688771 0.019974
1 3.110189 -0.283668 -0.898670
1 3.126996 -0.137867 0.847386
1 2.901283 -1.750481 0.098423

rc_5mU/rc_5mU15.xyz
6 2.228142 -0.469889 0.001025
6 -0.281675 1.253907 0.002534
6 -0.828596 -0.034258 -0.007082
6 -0.040452 -1.212446 -0.008964
1 -0.560853 -2.165016 -0.014508
7 1.280318 -1.290069 -0.005024
7 0.164878 2.337860 0.009443
8 3.220150 0.114337 0.005736
8 -3.913460 -0.400696 -0.039230
1 -1.921578 -0.139867 -0.012610
1 -4.255183 -0.308348 0.862537
1 -4.296793 0.345680 -0.523471
rc_C/rc_C01.xyz
6 -1.251085 -0.400262 -0.000090
6 1.164225 -0.226786 0.000136
6 1.008657 1.204062 0.000099
6 -0.298932 1.728749 -0.000109
1 -0.432021 2.806173 -0.000174
7 -1.391789 0.988752 -0.000214
7 0.030820 -0.961888 0.000122
8 -2.230194 -1.138938 -0.000147
7 2.350676 -0.793646 0.000165
1 2.457167 -1.805885 0.000133
1 3.188083 -0.217470 0.000166
1 1.884446 1.842228 0.000180
1 0.078737 -1.980651 0.000142
rc_C/rc_C02.xyz
6 -1.143397 -0.556883 -0.000102
6 1.150702 -0.266119 -0.000004
6 1.018199 1.182685 0.000125
6 -0.281738 1.720136 0.000185
1 -0.479744 2.786368 0.000306
7 -1.297667 0.885930 0.000089
7 0.095641 -1.081761 -0.000129
8 -2.194219 -1.199380 -0.000215

7 2.378768 -0.773963 0.000011
1 2.512612 -1.780245 -0.000057
1 3.194763 -0.171829 0.000123
1 1.884719 1.833885 0.000197
1 -2.258395 1.236511 0.000126
rc_C/rc_C04.xyz
6 1.101281 -0.364017 -0.000002
6 -1.150970 -0.287417 0.000000
6 -1.106023 1.151988 0.000009
6 0.176551 1.737572 -0.000001
1 0.288055 2.816760 -0.000006
7 1.276843 1.009494 -0.000010
7 -0.027930 -1.038369 -0.000005
8 2.210432 -1.084561 0.000007
7 -2.323351 -0.901939 0.000000
1 -2.367925 -1.919024 -0.000005
1 -3.192818 -0.373329 -0.000013
1 -2.011982 1.747203 0.000025
1 2.997245 -0.508187 0.000013
rc_C/rc_C03.xyz
6 1.119866 -0.318222 0.000001
6 -1.137572 -0.300467 0.000009
6 -1.126277 1.138981 0.000018
6 0.142837 1.754018 -0.000002
1 0.225331 2.835790 -0.000016
7 1.263458 1.057279 -0.000019
7 0.006531 -1.020400 0.000004
8 2.287447 -0.938299 0.000006
7 -2.291618 -0.947154 -0.000016
1 -2.307342 -1.965028 -0.000028
1 -3.175459 -0.443127 0.000014
1 -2.047096 1.711054 0.000031
1 2.163272 -1.906231 0.000016
rc_C/rc_C05.xyz
6 1.228725 -0.479768 -0.000058
6 -1.040509 -0.154471 0.000171

6 -0.878790 1.246125 0.002218
6 0.461020 1.705185 -0.000361
1 0.660124 2.772420 -0.002145
7 1.493763 0.889203 -0.001532
7 -0.067987 -1.012631 -0.001233
8 2.182328 -1.276355 0.001461
7 -2.397901 -0.712091 -0.000781
1 -2.369067 -1.736913 -0.081170
1 -2.949374 -0.337373 -0.786155
1 -1.722163 1.927348 0.002744
1 -2.895941 -0.468433 0.868046
rc_U/rc_U01.xyz
6 -1.179898 -0.453026 -0.000022
6 1.276574 -0.340630 0.000022
6 1.172780 1.120420 0.000027
6 -0.097339 1.730381 0.000000
1 -0.228262 2.807202 0.000002
7 -1.179935 0.977920 -0.000023
1 -2.108228 1.410980 -0.000037
7 0.064245 -1.022930 0.000008
8 -2.233677 -1.056077 -0.000043
8 2.351107 -0.933466 0.000035
1 0.095012 -2.042378 0.000001
1 2.079163 1.712751 0.000046
rc_U/rc_U04.xyz
6 1.120303 -0.264019 -0.000005
6 -1.276158 -0.371575 -0.000015
6 -1.247572 1.077889 0.000000
6 0.002992 1.737960 -0.000004
1 0.040671 2.822517 0.000007
7 1.160241 1.098483 -0.000006
1 3.011451 -0.360661 0.000012
7 -0.008691 -0.978404 -0.000004
8 2.226058 -0.944606 0.000007
8 -2.296857 -1.055870 0.000015
1 0.041689 -1.999331 0.000004

1 -2.185665 1.619213 0.000013
rc_U/rc_U02.xyz
6 1.132242 -0.540134 0.000000
6 -1.156804 -0.254849 0.000001
6 -1.031235 1.181276 0.000003
6 0.271526 1.716061 0.000003
1 0.471440 2.781849 0.000004
7 1.291346 0.883151 0.000001
1 2.252943 1.233218 0.000001
7 -0.126058 -1.069146 -0.000001
8 2.158312 -1.213387 -0.000005
8 -2.392578 -0.718487 -0.000001
1 -2.398277 -1.695677 -0.000004
1 -1.903362 1.823445 0.000003
rc_U/rc_U03.xyz
6 1.148355 -0.521586 -0.000011
6 -1.149450 -0.291790 0.000002
6 -1.049283 1.146671 0.000005
6 0.241622 1.710121 0.000008
1 0.415897 2.780401 0.000017
7 1.280327 0.901945 0.000002
1 2.233440 1.274398 0.000006
7 -0.095261 -1.078737 -0.000010
8 2.189016 -1.175625 -0.000022
8 -2.325407 -0.897630 0.000016
1 -3.073061 -0.273188 0.000041
1 -1.928086 1.781479 0.000008
rc_U/rc_U07.xyz
6 -1.248750 -0.372476 0.000000
6 1.158762 -0.257089 0.000000
6 1.047185 1.173916 0.000000
6 -0.250501 1.729140 0.000001
1 -0.358680 2.809028 0.000001
7 -1.358747 1.012308 0.000001
1 0.077435 -1.987759 -0.000001
7 0.032663 -0.966204 0.000000

8 -2.232507 -1.101435 -0.000001
8 2.271844 -0.925257 0.000000
1 3.066361 -0.356118 0.000000
1 1.942616 1.784710 0.000001
rc_U/rc_U34.xyz
6 1.135498 -0.226432 0.000000
6 -1.265538 -0.393962 0.000001
6 -1.276686 1.053570 0.000001
6 -0.043230 1.744034 0.000002
1 -0.034815 2.829259 0.000002
7 1.131303 1.138692 0.000002
1 2.346922 -1.735394 -0.000004
7 0.021778 -0.967486 -0.000001
8 2.321319 -0.757197 -0.000001
8 -2.263452 -1.109953 -0.000003
1 0.081822 -1.988301 -0.000002
1 -2.228705 1.569927 -0.000001
rc_U/rc_U09.xyz
6 1.242813 -0.387426 -0.000004
6 -1.165668 -0.233743 0.000002
6 -1.027027 1.194421 0.000003
6 0.277988 1.729662 0.000002
1 0.404768 2.807487 0.000003
7 1.374488 0.994220 -0.000001
1 -0.101089 -1.985297 -0.000002
7 -0.052221 -0.963666 -0.000001
8 2.211800 -1.134214 -0.000004
8 -2.362210 -0.734986 0.000003
1 -2.409224 -1.713480 0.000001
1 -1.915683 1.813528 0.000006
rc_U/rc_U05.xyz
6 -1.057328 -0.368918 0.000000
6 1.237589 -0.433069 -0.000001
6 1.280685 1.024538 0.000000
6 0.066359 1.731452 0.000002
1 -0.005306 2.813045 0.000003

7 -1.058840 1.034386 0.000002
1 -1.964949 1.512610 0.000003
7 0.017083 -1.087671 -0.000001
8 -2.282620 -0.843627 0.000000
8 2.301113 -1.081096 -0.000002
1 -2.281212 -1.820455 -0.000001
1 2.231997 1.541562 0.000000
rc_U/rc_U08.xyz
6 -1.052941 -0.391820 0.000001
6 1.245060 -0.420305 -0.000004
6 1.275182 1.034942 -0.000003
6 0.053393 1.724136 0.000003
1 -0.032969 2.804606 0.000006
7 -1.067415 1.013715 0.000006
1 -1.966922 1.505111 0.000010
7 0.037300 -1.088986 -0.000002
8 -2.210767 -1.015793 0.000002
8 2.318057 -1.058454 -0.000006
1 -2.992101 -0.433692 0.000006
1 2.220310 1.563137 -0.000005
rc_U/rc_U10.xyz
6 -1.088397 -0.358991 -0.000001
6 1.155189 -0.294532 0.000003
6 1.113198 1.151383 0.000003
6 -0.177918 1.740777 0.000005
1 -0.286432 2.820188 0.000007
7 -1.272155 1.012351 0.000003
1 -2.979432 -0.543883 -0.000021
7 0.061708 -1.037584 0.000000
8 -2.168090 -1.090965 -0.000015
8 2.344442 -0.840178 0.000005
1 2.290407 -1.818467 0.000006
1 2.025335 1.736114 0.000003
rc_U/rc_U11.xyz
6 1.106990 -0.314199 0.000002
6 -1.141199 -0.309672 -0.000003

6 -1.133783 1.135777 -0.000003
6 0.142415 1.756095 0.000004
1 0.220835 2.838072 0.000007
7 1.257468 1.059294 0.000008
1 2.128732 -1.913214 -0.000003
7 -0.026319 -1.022302 -0.000002
8 2.250293 -0.942002 0.000002
8 -2.314852 -0.886280 -0.000005
1 -2.237411 -1.863002 -0.000004
1 -2.060258 1.697451 -0.000006
rc_U/rc_U26.xyz
6 -1.100162 -0.347497 0.000000
6 1.145322 -0.329804 0.000004
6 1.126423 1.119574 0.000002
6 -0.153750 1.733951 0.000000
1 -0.239097 2.815517 -0.000003
7 -1.261327 1.027804 -0.000002
1 -2.994238 -0.496453 -0.000007
7 0.034739 -1.047386 0.000002
8 -2.193903 -1.059418 -0.000004
8 2.269652 -1.003431 0.000001
1 3.060546 -0.431574 -0.000003
1 2.045919 1.695035 0.000002
rc_U/rc_U13.xyz
6 -1.118689 -0.301200 0.000002
6 1.129281 -0.346118 -0.000001
6 1.147808 1.101886 0.000001
6 -0.115641 1.748921 0.000003
1 -0.169624 2.832484 0.000003
7 -1.244464 1.076091 0.000002
1 -2.168167 -1.880436 -0.000007
7 -0.002216 -1.032009 0.000000
8 -2.274286 -0.907142 -0.000003
8 2.235095 -1.047780 -0.000001
1 3.038502 -0.493536 -0.000001
1 2.083016 1.651360 0.000000

rc_U/rc_U14.xyz
6 -0.989013 -0.191057 -0.001238
6 1.245742 -0.463309 0.001880
6 1.350626 0.981181 -0.000675
6 0.165447 1.737614 0.002484
1 0.191222 2.821814 0.005188
7 -1.031029 1.157335 -0.000076
1 -3.024420 -0.199301 0.299063
7 -0.029633 -1.040305 0.000783
8 -2.296126 -0.751704 -0.069173
8 2.254391 -1.195264 0.004803
1 -2.374651 -1.697349 0.195062
1 2.329555 1.444795 -0.004010
rc_U/rc_U15.xyz
6 -2.301419 0.042681 -0.072969
6 0.592379 -0.781151 0.126747
6 0.709235 0.613703 0.129827
6 -0.408330 1.483715 0.071072
1 -0.219903 2.552811 0.092466
7 -1.679827 1.130591 -0.009441
1 4.823711 0.072503 -0.383674
7 0.500798 -1.949674 0.125846
8 -3.041054 -0.836189 -0.140615
8 3.897523 0.166607 -0.117520
1 3.538395 -0.731846 -0.162272
1 1.708053 1.043080 0.175649
d_1m5caC/d_1m5caC_0.xyz
6 -1.865593 0.634620 -0.006784
6 0.408522 1.192708 -0.006044
6 0.810672 -0.195352 0.000394
6 -0.216271 -1.102882 0.010604
1 -0.019126 -2.167664 0.019139
7 -1.517291 -0.731762 0.015358
7 -0.883493 1.562222 -0.013330
8 -3.083871 0.932158 -0.018677
7 1.326934 2.169088 -0.012327

1 1.032771 3.135706 -0.011849
1 2.305849 1.895390 0.012016
6 2.236698 -0.687394 -0.001030
8 3.167231 0.182506 0.066499
8 2.429553 -1.940047 -0.069712
6 -2.594693 -1.728935 0.025945
1 -3.245624 -1.561609 0.885918
1 -3.185656 -1.650663 -0.888971
1 -2.150587 -2.721525 0.092453
d_1m5caC/d_1m5caC_2.xyz
6 -1.900852 0.553070 0.007748
6 0.487390 1.267351 0.007383
6 0.854846 -0.150750 0.000171
6 -0.145905 -1.074496 -0.012415
1 0.083457 -2.132417 -0.020190
7 -1.482215 -0.767134 -0.021958
7 -0.903031 1.485715 0.020291
8 -3.102501 0.862477 0.021534
7 1.242239 2.312967 0.009429
1 2.211378 1.989935 -0.004257
6 2.287629 -0.630792 0.002126
8 3.207396 0.245278 -0.083103
8 2.492225 -1.881683 0.088609
1 -1.211167 2.453087 0.037369
6 -2.511780 -1.815113 -0.033228
1 -3.091644 -1.784314 0.891850
1 -3.179767 -1.670596 -0.884123
1 -2.016139 -2.780731 -0.122009
d_1m5caC/d_1m5caC_1.xyz
6 -1.901679 0.501890 -0.023424
6 0.471829 1.277590 0.107143
6 0.867956 -0.124947 0.027358
6 -0.102910 -1.074168 -0.014423
1 0.150711 -2.126481 -0.038949
7 -1.452737 -0.801130 -0.048045
7 -0.924432 1.462846 0.039040

8 -3.108805 0.791376 -0.054145
7 1.286757 2.266716 0.265258
1 0.773064 3.148350 0.328677
6 2.311149 -0.586388 -0.039035
8 3.112667 0.070961 -0.768321
8 2.609701 -1.637156 0.611476
1 -1.275376 2.415047 0.091931
6 -2.452406 -1.876623 -0.088872
1 -3.055913 -1.864346 0.821641
1 -3.103092 -1.748833 -0.955818
1 -1.928652 -2.828313 -0.165808
d_1m5caU/d_1m5caU01.xyz
6 -1.903028 0.524738 -0.007993
6 0.475026 1.270685 0.069720
6 0.871574 -0.125852 0.022179
6 -0.119155 -1.067484 -0.010264
1 0.129381 -2.121114 -0.027889
7 -1.454230 -0.785271 -0.036173
7 -0.910291 1.473491 0.046896
8 -3.104047 0.817577 -0.027382
8 1.209644 2.268014 0.148442
6 2.314853 -0.587328 -0.018642
8 2.542915 -1.789720 0.324840
8 3.193667 0.234942 -0.415408
1 -1.225253 2.439550 0.085711
6 -2.465843 -1.853153 -0.075071
1 -3.072048 -1.821974 0.832381
1 -3.108004 -1.720409 -0.947086
1 -1.950416 -2.809733 -0.141693
d_1m5caU/d_1m5caU02.xyz
6 1.904583 -0.526590 -0.000299
6 -0.475264 -1.276236 0.028492
6 -0.874768 0.121497 0.006236
6 0.118963 1.062371 -0.011721
1 -0.132224 2.115231 -0.021752
7 1.453542 0.783352 -0.024890

7 0.911975 -1.475273 0.023355
8 3.106136 -0.817018 0.000984
8 -1.201864 -2.281706 0.061233
6 -2.316308 0.594989 -0.006143
8 -2.509646 1.841059 0.158059
8 -3.232344 -0.261911 -0.185678
1 1.226136 -2.442063 0.046865
6 2.463752 1.853074 -0.041643
1 3.052458 1.821018 0.877247
1 3.122711 1.722232 -0.901272
1 1.948295 2.809014 -0.116661
d_1m5caU/d_1m5caU06.xyz
6 1.856815 0.657738 -0.000023
6 -0.408982 1.155419 -0.000075
6 -0.789553 -0.226469 0.000010
6 0.246224 -1.125134 0.000055
1 0.069929 -2.194311 0.000106
7 1.533707 -0.718224 0.000037
7 0.846863 1.570612 -0.000065
8 3.060813 0.983450 0.000021
8 -1.358711 2.089515 -0.000153
6 -2.223080 -0.662294 0.000040
8 -2.499915 -1.888823 0.000182
8 -3.096353 0.286846 -0.000088
1 -2.240976 1.558060 -0.000157
6 2.639589 -1.686445 0.000056
1 3.255675 -1.541347 -0.889045
1 3.255767 -1.541209 0.889069
1 2.222877 -2.692704 0.000157
d_1m5caU/d_1m5caU08.xyz
6 1.906015 -0.587842 0.012055
6 -0.344801 -1.340515 -0.033216
6 -0.808693 0.055047 -0.006835
6 0.151765 1.042906 -0.006998
1 -0.108513 2.093624 0.001476
7 1.466441 0.764737 -0.009792

7 0.999867 -1.577455 -0.011299
8 3.146294 -0.783278 0.051267
8 -1.142882 -2.317922 -0.082023
6 -2.222068 0.430483 0.024629
8 -2.428197 1.777454 -0.080822
8 -3.181052 -0.333318 0.137322
1 -3.387013 1.937822 -0.026169
6 2.471494 1.834942 0.002532
1 3.052929 1.790097 0.925729
1 3.143461 1.719273 -0.849391
1 1.959404 2.794602 -0.062953
d_1m5caU/d_1m5caU04.xyz
6 -1.895148 -0.624630 0.013327
6 0.383319 -1.295085 -0.038490
6 0.793736 0.117150 -0.015478
6 -0.203239 1.067165 -0.012918
1 0.030777 2.125328 -0.008054
7 -1.506282 0.743524 -0.007032
7 -0.953159 -1.580495 -0.013756
8 -3.127721 -0.864100 0.053992
8 1.213931 -2.244861 -0.089437
1 3.993498 0.174803 0.159466
6 2.166069 0.633101 0.002869
8 2.455847 1.831561 -0.093685
8 3.139186 -0.294464 0.151963
6 -2.550636 1.775088 0.011714
1 -3.219575 1.638269 -0.839449
1 -3.128418 1.705272 0.935547
1 -2.074750 2.753307 -0.050817
d_1m5caU/d_1m5caU05.xyz
6 -1.871073 0.598300 -0.000486
6 0.382276 1.191503 -0.026604
6 0.836449 -0.162231 -0.004541
6 -0.177163 -1.093478 0.011522
1 0.045413 -2.152837 0.022719
7 -1.483490 -0.753427 0.020924

7 -0.892794 1.544944 -0.028934
8 -3.085863 0.879164 0.004625
8 1.280994 2.189147 -0.065633
6 2.274000 -0.646475 0.009001
8 2.452112 -1.892579 -0.168145
8 3.196696 0.198883 0.202582
1 0.794938 3.033090 -0.084308
6 -2.538960 -1.776547 0.042134
1 -3.180360 -1.622817 0.911311
1 -3.140135 -1.703394 -0.866052
1 -2.070554 -2.758013 0.098815
d_1m5caU/d_1m5caU07.xyz
6 -1.894246 -0.596320 -0.034939
6 0.364000 -1.320825 -0.075100
6 0.807588 0.079755 0.001183
6 -0.158354 1.052959 0.075364
1 0.080283 2.106173 0.177801
7 -1.473092 0.755225 0.072434
7 -0.975050 -1.575576 -0.099338
8 -3.132161 -0.808626 -0.063278
8 1.186950 -2.276433 -0.147602
6 2.230814 0.444036 0.066658
8 2.612394 1.645852 -0.461081
8 3.110471 -0.241896 0.577086
1 1.916571 2.053708 -1.002796
6 -2.491889 1.809107 0.166764
1 -3.094957 1.828533 -0.743289
1 -3.142892 1.622003 1.022405
1 -1.990717 2.768590 0.293632
d_1m5caU/d_1m5caU13.xyz
6 1.762495 -0.527033 0.006915
6 -0.442461 -1.273434 -0.115380
6 -0.877323 0.118656 -0.025698
6 0.080303 1.086666 0.016159
1 -0.166201 2.140311 0.047022
7 1.418817 0.790761 0.037701

7 0.922171 -1.521601 -0.066003
8 3.084131 -0.739431 0.059581
8 -1.228108 -2.238899 -0.261177
6 -2.332607 0.539206 0.042935
8 -2.646580 1.629449 -0.528921
8 -3.127713 -0.202970 0.693433
1 3.249627 -1.698202 0.029230
6 2.444475 1.848492 0.091855
1 3.081014 1.702219 0.965318
1 3.050256 1.823247 -0.815522
1 1.935272 2.807792 0.168025
d_1m5caU/d_1m5caU15.xyz
6 1.759931 -0.559711 -0.001897
6 -0.456472 -1.277750 -0.108093
6 -0.878578 0.118391 -0.023515
6 0.088313 1.074670 0.019778
1 -0.146786 2.130838 0.052056
7 1.426236 0.765268 0.040295
7 0.903738 -1.540117 -0.073335
8 3.059098 -0.898752 0.031802
8 -1.255006 -2.235441 -0.238423
1 3.655937 -0.132920 0.066201
6 -2.329693 0.555681 0.037440
8 -2.630843 1.638688 -0.554293
8 -3.132625 -0.166967 0.699658
6 2.447608 1.827028 0.111300
1 3.068811 1.691895 1.000539
1 3.063255 1.818414 -0.792052
1 1.937311 2.785641 0.184500
d_1m5dhmC/d_1m5dhmC_3.xyz
6 1.937429 0.601965 0.023870
6 -0.316181 1.207899 -0.120783
6 -0.752150 -0.162419 -0.131659
6 0.241185 -1.090573 -0.045240
1 0.019966 -2.150373 -0.055733
7 1.557363 -0.744704 0.051477

7 0.976976 1.555565 -0.052067
8 3.163039 0.881677 0.077620
6 -2.216010 -0.590950 -0.326412
1 -2.574201 -0.129002 -1.269878
7 -1.221645 2.208989 -0.212311
1 -0.896222 3.157297 -0.077667
1 -2.186011 1.993426 0.011862
8 -3.040787 0.164995 0.667444
1 -2.757417 -0.145621 1.542698
8 -2.421709 -1.917401 -0.259247
6 2.606245 -1.769021 0.111876
1 3.231033 -1.720214 -0.783213
1 3.233033 -1.611209 0.991426
1 2.133509 -2.748832 0.174359
d_1m5dhmC/d_1m5dhmC_4.xyz
6 1.953137 0.578802 0.019204
6 -0.285976 1.238437 -0.085906
6 -0.754879 -0.121906 -0.109786
6 0.215461 -1.075815 -0.050682
1 -0.013973 -2.132897 -0.068007
7 1.542939 -0.757685 0.032844
7 1.014584 1.556115 -0.029667
8 3.185713 0.830743 0.061584
6 -2.249311 -0.425848 -0.259961
1 -2.518633 -0.236085 -1.322299
7 -1.182101 2.247888 -0.151110
1 -0.852565 3.191021 0.007919
1 -2.143405 2.000996 0.083629
8 -3.045488 0.274218 0.593476
8 -2.378771 -1.880282 -0.080486
1 -3.330466 -2.054696 -0.132015
6 2.567606 -1.806670 0.083470
1 3.192938 -1.766282 -0.811601
1 3.198242 -1.670191 0.963996
1 2.072049 -2.775535 0.139277
d_1m5dhmC/d_1m5dhmC_1.xyz

6 1.974158 0.503464 0.028871
6 -0.228625 1.382500 -0.157465
6 -0.762385 0.020051 -0.148817
6 0.114389 -1.011258 -0.057369
1 -0.209009 -2.044797 -0.049383
7 1.466941 -0.809854 0.025333
7 1.139579 1.546159 -0.061318
8 3.234140 0.636532 0.121881
6 -2.249144 -0.212835 -0.186328
1 -2.740123 0.526730 -0.823242
7 -1.044811 2.407804 -0.256934
1 -0.478222 3.259036 -0.259994
8 -2.858022 -0.032048 1.096412
1 -2.336632 -0.532007 1.745552
8 -2.508776 -1.525487 -0.670893
1 -3.470638 -1.626296 -0.732197
6 2.397292 -1.938161 0.096004
1 3.101315 -1.906449 -0.739063
1 2.962103 -1.913424 1.031618
1 1.826394 -2.866096 0.048568
d_1m5dhmC/d_1m5dhmC_2.xyz
6 1.972430 0.557955 0.023991
6 -0.257016 1.365531 -0.157907
6 -0.743394 -0.020896 -0.149498
6 0.169103 -1.021007 -0.052119
1 -0.117501 -2.065061 -0.048518
7 1.510690 -0.773077 0.043169
7 1.105007 1.569795 -0.072066
8 3.228947 0.731989 0.106363
6 -2.219656 -0.310414 -0.244979
1 -2.676140 0.245634 -1.072008
7 -1.030508 2.426189 -0.244575
1 -2.008270 2.144722 -0.275449
8 -2.932637 0.142132 0.910239
1 -2.462808 -0.182086 1.696106
8 -2.422842 -1.700350 -0.438740

1 -3.367070 -1.835163 -0.609909
6 2.477342 -1.870806 0.114360
1 3.157376 -1.839373 -0.740621
1 3.064877 -1.805943 1.033438
1 1.932635 -2.815425 0.105278
d_1m5dhmU/d_1m5dhmU_a1_8.xyz
6 -1.899433 0.681915 0.072235
6 0.419450 1.105099 0.017095
6 0.687200 -0.317302 -0.125361
6 -0.379121 -1.158736 -0.173687
1 -0.265829 -2.232216 -0.284672
7 -1.659501 -0.694561 -0.080289
7 -0.858231 1.542710 0.113811
8 -3.098161 1.062422 0.162875
8 1.392020 1.940966 0.039656
6 2.102243 -0.844583 -0.167814
1 2.131582 -1.844804 -0.604703
8 2.644864 -1.009493 1.150447
1 2.531323 -0.174735 1.633533
6 -2.805056 -1.609817 -0.129608
1 -3.382586 -1.538365 0.794636
1 -3.453654 -1.360343 -0.972232
1 -2.435335 -2.628584 -0.247434
8 2.944177 -0.018923 -0.952371
1 2.683725 0.902768 -0.715797
d_1m5dhmU/d_1m5dhmU_f2_12.xyz
6 -1.909461 0.671250 0.084852
6 0.405677 1.119253 0.028439
6 0.686622 -0.299040 -0.127878
6 -0.371473 -1.150105 -0.186591
1 -0.246760 -2.220786 -0.311835
7 -1.655819 -0.700835 -0.088505
7 -0.877514 1.541791 0.134365
8 -3.112145 1.036583 0.184416
8 1.365718 1.968857 0.051513
6 2.101813 -0.815363 -0.186066

1 2.144186 -1.788000 -0.687141
8 2.563600 -0.964336 1.165632
1 3.448883 -1.360951 1.129343
6 -2.792384 -1.625928 -0.152225
1 -3.369832 -1.575677 0.773503
1 -3.443647 -1.367890 -0.990217
1 -2.412772 -2.638802 -0.287604
8 2.958483 0.047418 -0.910525
1 2.683260 0.956832 -0.638548
d_1m5dhmU/d_1m5dhmU_a1_10.xyz
6 -1.898820 0.679455 0.088268
6 0.416938 1.119695 0.022770
6 0.692092 -0.298178 -0.149647
6 -0.369512 -1.144874 -0.212600
1 -0.249782 -2.214829 -0.350019
7 -1.651795 -0.691444 -0.101971
7 -0.863156 1.546263 0.139992
8 -3.099147 1.048623 0.199790
8 1.382118 1.963490 0.046713
6 2.106517 -0.823842 -0.195735
1 2.139144 -1.797264 -0.697607
8 2.648210 -0.970600 1.137822
1 2.047030 -1.540833 1.642954
6 -2.792443 -1.611519 -0.166022
1 -3.365029 -1.565019 0.762952
1 -3.446876 -1.345499 -0.999016
1 -2.417717 -2.625100 -0.309531
8 2.978599 0.037652 -0.886123
1 2.691007 0.947071 -0.623699
d_1m5dhmU/d_1m5dhmU_f2_3.xyz
6 -1.949290 -0.605366 0.025796
6 0.309131 -1.270954 -0.104960
6 0.729841 0.122616 -0.135405
6 -0.237072 1.074753 -0.070346
1 -0.013253 2.133886 -0.094276
7 -1.558444 0.742599 0.026531

7 -1.009375 -1.575999 -0.031617
8 -3.184061 -0.858765 0.085019
8 1.183714 -2.206854 -0.140401
6 2.194295 0.456146 -0.304332
1 2.505924 0.277810 -1.344675
8 3.009358 -0.350727 0.553139
1 2.647335 -1.261098 0.442004
6 -2.594143 1.779705 0.076648
1 -3.228032 1.725457 -0.811768
1 -3.215862 1.646943 0.964137
1 -2.109365 2.755019 0.118286
8 2.415373 1.809344 0.023944
1 3.276330 2.070400 -0.336120
d_1m5dhmU/d_1m5dhmU_a_12.xyz
6 -1.969614 0.529220 0.030485
6 0.236050 1.361424 -0.169699
6 0.753328 -0.001613 -0.154816
6 -0.140940 -1.021133 -0.056959
1 0.161819 -2.060841 -0.047679
7 -1.483142 -0.789191 0.029691
7 -1.107571 1.561408 -0.071058
8 -3.218679 0.692295 0.128046
8 1.023316 2.355363 -0.276435
6 2.239865 -0.231289 -0.207091
1 2.709497 0.447480 -0.924750
8 2.492470 -1.580624 -0.569045
1 3.451205 -1.681726 -0.667813
6 -2.438777 -1.899127 0.104047
1 -3.003040 -1.852965 1.038365
1 -3.138156 -1.851106 -0.733501
1 -1.886665 -2.838049 0.061932
8 2.874574 0.080398 1.035445
1 2.387428 -0.372643 1.743118
d_1m5dhmU/d_1m5dhmU_a_6.xyz
6 1.940580 -0.618081 0.040475
6 -0.322345 -1.267770 -0.089348

6 -0.734907 0.128172 -0.146544
6 0.242726 1.071906 -0.107681
1 0.028853 2.132820 -0.159965
7 1.561002 0.732610 -0.004971
7 0.992600 -1.581612 -0.001897
8 3.171793 -0.879313 0.124940
8 -1.202103 -2.200340 -0.112014
6 -2.196878 0.497895 -0.331653
1 -2.476671 0.426094 -1.391358
8 -2.479895 1.833787 0.022409
1 -2.202228 1.973527 0.943441
6 2.604435 1.762016 0.035319
1 3.168137 1.693419 0.968386
1 3.291594 1.633816 -0.803875
1 2.131193 2.741579 -0.030641
8 -3.039170 -0.360239 0.435802
1 -2.622749 -1.254231 0.349584
d_1m5dhmU/d_1m5dhmU_f1_7.xyz
6 1.969620 0.529108 0.030453
6 -0.235964 1.361526 -0.169770
6 -0.753363 -0.001463 -0.155007
6 0.140813 -1.021064 -0.057144
1 -0.162030 -2.060748 -0.047817
7 1.483049 -0.789246 0.029439
7 1.107669 1.561384 -0.071123
8 3.218685 0.692087 0.128240
8 -1.023150 2.355548 -0.276374
6 -2.239931 -0.231056 -0.206880
1 -2.709824 0.448178 -0.923925
8 -2.874150 0.079841 1.036059
1 -2.386592 -0.373427 1.743313
6 2.438557 -1.899281 0.104085
1 3.138572 -1.850965 -0.732907
1 3.002159 -1.853565 1.038829
1 1.886434 -2.838158 0.061128
8 -2.492708 -1.580171 -0.569672

1 -3.451560 -1.681350 -0.667278
d_1m5dhmU/d_1m5dhmU_f1_2.xyz
6 1.948628 -0.606106 0.029399
6 -0.309886 -1.270267 -0.107839
6 -0.730007 0.123495 -0.137179
6 0.237907 1.075038 -0.076892
1 0.015133 2.134332 -0.103718
7 1.559208 0.742213 0.017536
7 1.008247 -1.576068 -0.030659
8 3.182639 -0.860061 0.101026
8 -1.184609 -2.205965 -0.147545
6 -2.194589 0.459171 -0.302244
1 -2.508029 0.289515 -1.343488
8 -2.414010 1.809920 0.037318
1 -3.277521 2.072854 -0.315172
6 2.595528 1.778528 0.069376
1 3.176410 1.687134 0.989715
1 3.268809 1.681145 -0.785222
1 2.113158 2.755441 0.041951
8 -3.009075 -0.353830 0.549939
1 -2.645190 -1.263106 0.434169
d_1m5dhmU/d_1m5dhmU_f1_15.xyz
6 -1.972979 0.538808 -0.031442
6 0.240050 1.349729 0.171239
6 0.745507 -0.016809 0.139715
6 -0.157137 -1.027574 0.040086
1 0.136457 -2.069794 0.023858
7 -1.497957 -0.783503 -0.038756
7 -1.101871 1.562760 0.075763
8 -3.220911 0.713301 -0.125944
8 1.035705 2.335701 0.291890
6 2.226857 -0.257577 0.200794
1 2.689304 0.392127 0.952297
8 2.783970 0.064473 -1.077918
1 3.731857 0.235365 -0.962865
6 -2.463128 -1.885142 -0.112159

1 -3.157008 -1.834887 0.729888
1 -3.032594 -1.830632 -1.042819
1 -1.918349 -2.828595 -0.077170
8 2.478085 -1.616417 0.528053
1 3.419324 -1.703447 0.739702
d_1m5dhmU/d_1m5dhmU_f1_6.xyz
6 -1.968855 0.551073 0.048486
6 0.249771 1.342208 -0.167221
6 0.745452 -0.029903 -0.144310
6 -0.169285 -1.031573 -0.071295
1 0.105161 -2.079796 -0.087401
7 -1.507604 -0.775560 0.018677
7 -1.088483 1.567798 -0.052584
8 -3.213323 0.736195 0.164908
8 1.052341 2.320228 -0.301465
6 2.230228 -0.280914 -0.232938
1 2.658380 0.289497 -1.063638
8 2.577797 -1.630340 -0.501235
1 2.317412 -2.173852 0.260530
6 -2.482520 -1.868872 0.084279
1 -3.036287 -1.828105 1.025177
1 -3.189544 -1.794111 -0.744840
1 -1.948536 -2.816993 0.020659
8 2.815748 0.145616 0.994898
1 3.776772 0.191996 0.868013
d_1m5dhmU/d_1m5dhmU_a_10.xyz
6 -1.958543 0.555069 0.049984
6 0.261755 1.339496 -0.174690
6 0.753816 -0.034990 -0.155871
6 -0.165543 -1.033990 -0.086667
1 0.104247 -2.083119 -0.111601
7 -1.502039 -0.773349 0.011097
7 -1.075063 1.568928 -0.051619
8 -3.201484 0.743722 0.174383
8 1.065916 2.314939 -0.313077
6 2.241135 -0.289033 -0.240980

1 2.673830 0.261506 -1.079278
8 2.574941 -1.648351 -0.478004
1 2.303635 -2.178513 0.289004
6 -2.480946 -1.863270 0.074862
1 -3.030354 -1.825713 1.018423
1 -3.191273 -1.780688 -0.750676
1 -1.951096 -2.813111 0.003083
8 2.915484 0.194821 0.918837
1 2.381823 -0.030143 1.697744
d_1m5dhmU/d_1m5dhmU_a_11.xyz
6 -1.965973 0.528744 0.025726
6 0.236768 1.369428 -0.167360
6 0.759800 0.008601 -0.141842
6 -0.130343 -1.014075 -0.042345
1 0.180597 -2.051848 -0.025973
7 -1.473797 -0.787871 0.038009
7 -1.108194 1.564477 -0.079612
8 -3.216084 0.686770 0.116009
8 1.021846 2.365393 -0.272297
6 2.250786 -0.222889 -0.190840
1 2.716732 0.466835 -0.901314
8 2.589583 -1.570154 -0.534481
1 2.297186 -1.742206 -1.443768
6 -2.425237 -1.901093 0.117675
1 -2.992462 -1.850325 1.049980
1 -3.122450 -1.862409 -0.722124
1 -1.869075 -2.838001 0.084592
8 2.873961 0.044678 1.052313
1 2.354139 -0.384090 1.751398
d_1m5dhmU/d_1m5dhmU_f1_4.xyz
6 -1.939066 0.648908 0.003426
6 0.377284 1.158359 -0.008379
6 0.689285 -0.268781 -0.002206
6 -0.351086 -1.144691 -0.004474
1 -0.199017 -2.218971 -0.003727
7 -1.648655 -0.727014 -0.008172

7 -0.931112 1.544099 -0.001729
8 -3.155257 0.988921 0.017471
8 1.295011 2.037005 -0.019685
6 2.092903 -0.801726 0.007477
1 2.073846 -1.898273 0.015235
8 2.762376 -0.346156 1.182965
1 3.578421 -0.860339 1.282994
6 -2.754938 -1.688954 -0.005957
1 -3.348889 -1.583054 0.904845
1 -3.401350 -1.520586 -0.869858
1 -2.343687 -2.697337 -0.054693
8 2.775671 -0.364312 -1.167302
1 3.590332 -0.883398 -1.252398
d_1m5dhmU/d_1m5dhmU_a_3.xyz
6 -1.972665 0.535912 0.030174
6 0.235975 1.359277 -0.166515
6 0.749305 -0.003779 -0.124312
6 -0.147231 -1.019498 -0.026187
1 0.157402 -2.058892 -0.007084
7 -1.489840 -0.783820 0.046089
7 -1.107567 1.565153 -0.079022
8 -3.221915 0.703204 0.119063
8 1.027862 2.348469 -0.287797
6 2.234070 -0.247060 -0.193815
1 2.683743 0.397732 -0.959342
8 2.566241 -1.609349 -0.477142
1 2.376289 -1.783962 -1.412658
6 -2.448894 -1.890707 0.119906
1 -3.021764 -1.836413 1.048521
1 -3.140294 -1.847555 -0.724566
1 -1.898449 -2.831096 0.090225
8 2.797988 0.053048 1.070280
1 3.760169 0.123012 0.964705
d_1m5dhmU/d_1m5dhmU_f_3.xyz
6 -1.939140 0.648885 0.004134
6 0.377211 1.158473 -0.009879

6 0.689314 -0.268600 -0.002581
6 -0.351059 -1.144554 -0.004963
1 -0.198876 -2.218815 -0.004126
7 -1.648646 -0.727022 -0.009534
7 -0.931232 1.544106 -0.001814
8 -3.155325 0.988799 0.020295
8 1.294845 2.037195 -0.023291
6 2.092901 -0.801728 0.008782
1 2.073628 -1.898267 0.018338
8 2.761474 -0.344345 1.184129
1 3.577500 -0.858262 1.285499
6 -2.754748 -1.689169 -0.007010
1 -3.344443 -1.588537 0.907205
1 -3.405186 -1.516177 -0.866920
1 -2.343480 -2.697157 -0.063389
8 2.776659 -0.366323 -1.165994
1 3.591904 -0.884821 -1.249182
d_1m5dhmU/d_1m5dhmU_a1_7.xyz
6 1.935639 0.652942 0.004932
6 -0.381743 1.159867 -0.008514
6 -0.691931 -0.268119 -0.025910
6 0.350652 -1.142390 -0.029310
1 0.199545 -2.216969 -0.039164
7 1.647436 -0.723604 -0.019994
7 0.926400 1.546773 0.007005
8 3.151238 0.994390 0.025260
8 -1.299951 2.037888 -0.006922
6 -2.094493 -0.810997 -0.015877
1 -2.058249 -1.908838 -0.023318
8 -2.799409 -0.347275 -1.149820
1 -3.643490 -0.823374 -1.193234
6 2.754867 -1.684297 -0.015973
1 3.407608 -1.509441 -0.873789
1 3.341658 -1.584206 0.900118
1 2.344900 -2.692691 -0.074936
8 -2.850782 -0.392975 1.134071

1 -2.455542 -0.804938 1.918440
d_1m5dhmU/d_1m5dhmU_d2_12.xyz
6 1.958807 -0.524502 -0.037484
6 -0.405166 -1.247717 0.154053
6 -0.797426 0.141548 0.151468
6 0.179034 1.080079 0.063031
1 -0.065280 2.135202 0.070234
7 1.518911 0.782105 -0.036872
7 0.965484 -1.475635 0.062359
8 3.157350 -0.830523 -0.119200
8 -1.183467 -2.221708 0.223411
6 -2.278493 0.526248 0.299160
1 -2.643634 0.042487 1.231487
8 -3.022491 -0.165383 -0.799151
1 -2.859159 -1.119600 -0.699062
6 2.534446 1.843524 -0.105822
1 3.210081 1.767695 0.748520
1 3.108426 1.752699 -1.029884
1 2.024666 2.805219 -0.085698
8 -2.525115 1.842557 0.242465
1 1.276716 -2.443622 0.059352
d_1m5dhmU/d_1m5dhmU_d_3.xyz
6 1.973644 0.474252 0.047893
6 -0.358046 1.304981 -0.170282
6 -0.808508 -0.067882 -0.158437
6 0.126612 -1.047798 -0.069100
1 -0.165629 -2.090686 -0.080638
7 1.477560 -0.812364 0.032705
7 1.024546 1.466543 -0.060153
8 3.183725 0.726342 0.145396
8 -1.077519 2.316475 -0.275462
6 -2.298366 -0.401765 -0.274731
1 -2.729103 0.277408 -1.039093
8 -2.862905 0.082121 1.022128
1 -3.816464 -0.083859 0.960310
6 2.445039 -1.917329 0.108291

1 3.149681 -1.852019 -0.722956
1 2.993642 -1.870847 1.051172
1 1.896998 -2.856326 0.051183
8 -2.581619 -1.698509 -0.500410
1 1.380431 2.418892 -0.062861
d_1m5dhmU/d_1m5dhmU_d1_6.xyz
6 1.967163 0.476366 0.050882
6 -0.364779 1.304563 -0.174044
6 -0.814635 -0.069416 -0.174969
6 0.122782 -1.047914 -0.088905
1 -0.165701 -2.091566 -0.110399
7 1.473414 -0.810275 0.025145
7 1.016703 1.467832 -0.054944
8 3.176584 0.730519 0.155728
8 -1.085473 2.315559 -0.274912
6 -2.310810 -0.397146 -0.282530
1 -2.747576 0.287533 -1.034963
8 -2.948346 0.117281 0.966382
1 -2.661489 -0.487598 1.669933
6 2.442140 -1.914217 0.098035
1 3.149228 -1.844279 -0.730810
1 2.988066 -1.871754 1.042674
1 1.895596 -2.853697 0.034858
8 -2.581641 -1.697830 -0.495158
1 1.370907 2.420804 -0.049831
d_1m5dhmU/d_1m5dhmU_d_8.xyz
6 1.931358 0.602721 0.022314
6 -0.504843 1.084589 0.039252
6 -0.750643 -0.339480 -0.025222
6 0.321699 -1.171141 -0.072850
1 0.197221 -2.247001 -0.131970
7 1.631120 -0.739408 -0.046874
7 0.840254 1.445415 0.053432
8 3.093933 1.033689 0.051570
8 -1.374953 1.977396 0.063266
6 -2.179608 -0.887253 0.016739

1 -2.138617 -1.941809 -0.319957
8 -2.942704 -0.255416 -1.085252
1 -2.978237 0.691273 -0.866528
6 2.753774 -1.688915 -0.077460
1 3.400089 -1.474569 -0.930600
1 3.334044 -1.610450 0.844202
1 2.350999 -2.696344 -0.170244
8 -2.770001 -0.718777 1.223213
1 1.054265 2.438594 0.090186
d_1m5fC/d_1m5fC_2.xyz
6 1.527950 0.760182 -0.000323
6 -0.835063 1.030583 -0.000054
6 -0.986563 -0.439116 0.000142
6 0.158381 -1.210699 0.000160
1 0.104419 -2.295901 0.000335
7 1.383961 -0.671869 -0.000051
7 0.451203 1.539017 -0.000310
8 2.713949 1.191512 -0.000547
7 -1.835511 1.871827 0.000029
1 -2.707432 1.344253 0.000248
6 -2.232353 -1.155877 0.000340
1 -2.133596 -2.257077 0.000475
8 -3.375344 -0.654892 0.000370
6 2.592585 -1.503921 0.000108
1 3.191913 -1.295473 -0.888209
1 3.191850 -1.295227 0.888414
1 2.296820 -2.553285 0.000242
d_1m5fC/d_1m5fC_3.xyz
6 1.587623 0.595012 0.008642
6 -0.681881 1.306522 -0.007749
6 -1.110282 -0.101245 -0.001252
6 -0.136727 -1.074641 -0.009496
1 -0.398467 -2.127386 -0.009896
7 1.172700 -0.778648 -0.017184
7 0.677450 1.568248 0.007214
8 2.832310 0.796043 0.032206

7 -1.568880 2.267292 -0.025604
1 -1.069285 3.158915 -0.024892
6 -2.500858 -0.489198 0.014527
1 -3.223141 0.341067 0.026826
8 -2.925454 -1.662673 0.016821
6 2.195818 -1.829215 -0.017668
1 2.862959 -1.699317 -0.871614
1 2.782424 -1.784633 0.902636
1 1.699612 -2.797268 -0.088278
d_1m5fC/d_1m5fC_1.xyz
6 1.534174 0.733528 -0.000308
6 -0.831002 1.042528 -0.000046
6 -1.012984 -0.420397 0.000146
6 0.124030 -1.204993 0.000161
1 0.053457 -2.288808 0.000333
7 1.362345 -0.691477 -0.000047
7 0.467339 1.527980 -0.000297
8 2.725115 1.149069 -0.000536
7 -1.854967 1.854047 0.000014
1 -1.486202 2.808589 -0.000147
6 -2.253999 -1.156503 0.000355
1 -2.115007 -2.254938 0.000492
8 -3.414216 -0.708735 0.000391
6 2.553995 -1.548378 0.000093
1 3.157514 -1.352383 -0.888270
1 3.157495 -1.352122 0.888414
1 2.237244 -2.591567 0.000243
d_1m5fC/d_1m5fC_4.xyz
6 1.590971 0.612244 0.011703
6 -0.678754 1.326643 -0.009669
6 -1.104633 -0.089152 -0.001316
6 -0.125199 -1.060373 -0.012205
1 -0.386714 -2.113385 -0.014155
7 1.180721 -0.764381 -0.020827
7 0.680269 1.580768 0.011301
8 2.836132 0.813973 0.040344

7 -1.481830 2.360530 -0.032771
1 -2.454715 2.059627 -0.045877
6 -2.485092 -0.511355 0.018272
1 -3.243313 0.289812 0.031747
8 -2.881998 -1.693655 0.023360
6 2.206090 -1.812798 -0.024369
1 2.872789 -1.677833 -0.877779
1 2.792182 -1.768592 0.896151
1 1.712279 -2.781843 -0.098130
d_1m5fU/d_1m5fU1_a.xyz
6 1.590301 0.596181 0.004101
6 -0.665994 1.306743 -0.004446
6 -1.102638 -0.092890 -0.000792
6 -0.132725 -1.072059 -0.004791
1 -0.393175 -2.125176 -0.004829
7 1.173679 -0.771575 -0.008949
7 0.670775 1.576748 0.000506
8 2.826528 0.806089 0.018183
8 -1.504595 2.254445 -0.011213
6 2.201299 -1.820371 -0.008863
1 2.859916 -1.691669 -0.869182
1 2.792620 -1.762194 0.907096
1 1.707127 -2.789859 -0.067281
6 -2.503219 -0.453255 0.005939
1 -3.208600 0.392328 0.007952
8 -2.945834 -1.616251 0.010336
d_1m5fU/d_1m5fU2.xyz
6 1.529589 0.737449 0.000190
6 -0.822983 1.035051 -0.000307
6 -1.003089 -0.424482 -0.000114
6 0.133198 -1.208920 -0.000108
1 0.070161 -2.293377 -0.000097
7 1.365308 -0.681941 -0.000131
7 0.447567 1.532991 -0.000046
8 2.707461 1.168095 0.000091
8 -1.807457 1.825802 0.000068

6 2.567397 -1.526635 0.000050
1 3.166863 -1.320213 -0.888367
1 3.166203 -1.320800 0.889056
1 2.261759 -2.572747 -0.000420
6 -2.265211 -1.129158 0.000009
1 -2.163919 -2.231241 0.000244
8 -3.403078 -0.633748 0.000154
d_1m5hmC/d_1m5hmC_3.xyz
6 1.590851 0.734253 0.021167
6 -0.732148 0.921354 -0.091514
6 -0.913838 -0.503718 -0.163951
6 0.227347 -1.240731 -0.090331
1 0.215768 -2.325250 -0.113213
7 1.463488 -0.657676 0.011384
7 0.471801 1.502157 -0.013681
8 2.746819 1.232640 0.075699
6 -2.301183 -1.111643 -0.329451
1 -2.620798 -0.891930 -1.372026
1 -2.175404 -2.212003 -0.290452
7 -1.835306 1.702982 -0.125992
1 -1.733983 2.688027 0.081932
1 -2.702851 1.220271 0.127296
8 -3.238413 -0.636089 0.602578
6 2.679240 -1.473271 0.099529
1 3.330305 -1.274036 -0.754715
1 3.221777 -1.241540 1.018644
1 2.396435 -2.525645 0.101656
d_1m5hmC/d_1m5hmC_1.xyz
6 1.627616 0.663980 0.095766
6 -0.694415 1.138998 -0.122365
6 -0.967940 -0.291276 -0.279200
6 0.086452 -1.145985 -0.230049
1 -0.040842 -2.217961 -0.342179
7 1.373709 -0.714576 -0.041224
7 0.617469 1.541445 0.048895
8 2.838145 1.013379 0.259188

6 -2.355861 -0.823980 -0.470388
1 -2.926129 -0.189865 -1.152405
1 -2.312211 -1.836280 -0.891450
7 -1.683263 2.004131 -0.156293
1 -1.282551 2.938107 -0.043593
8 -3.136722 -0.851184 0.754482
1 -2.652531 -1.395988 1.393405
6 2.492014 -1.658659 -0.002562
1 3.189182 -1.463200 -0.821651
1 3.031296 -1.576319 0.944089
1 2.099796 -2.671512 -0.102438
d_1m5hmC/d_1m5hmC_2.xyz
6 1.628880 0.695264 0.082922
6 -0.702305 1.115127 -0.121293
6 -0.941169 -0.328648 -0.250008
6 0.132474 -1.158809 -0.189048
1 0.028392 -2.235766 -0.275814
7 1.409498 -0.693213 -0.024974
7 0.599359 1.546521 0.032983
8 2.832811 1.077283 0.226100
6 -2.315360 -0.893903 -0.452728
1 -2.843834 -0.373635 -1.258416
1 -2.246361 -1.953798 -0.725921
7 -1.654527 2.022627 -0.150950
1 -2.559115 1.563579 -0.244394
8 -3.178112 -0.744317 0.705221
1 -2.735969 -1.169071 1.455987
6 2.551280 -1.609679 0.016819
1 3.237724 -1.405000 -0.808905
1 3.094892 -1.504880 0.958696
1 2.183570 -2.632803 -0.071204
d_1m5hmU/d_1m5hmU12.xyz
6 1.596138 0.727934 0.048812
6 -0.740357 1.000939 -0.135063
6 -0.914449 -0.442249 -0.217144
6 0.198153 -1.216657 -0.131656

1 0.156360 -2.300353 -0.166186
7 1.444538 -0.666659 -0.002443
7 0.506869 1.526224 -0.010765
8 2.769202 1.185987 0.155094
8 -1.764667 1.764317 -0.166802
6 -2.293212 -1.011152 -0.385538
1 -2.726013 -0.680983 -1.339784
1 -2.262629 -2.103659 -0.384654
8 -3.179403 -0.622416 0.689448
1 -3.160718 0.351143 0.695643
6 2.639614 -1.512173 0.080836
1 3.308356 -1.306848 -0.758393
1 3.175527 -1.321501 1.013280
1 2.332882 -2.557705 0.049138
d_1m5hmU/d_1m5hmU11.xyz
6 1.625845 0.667206 0.098860
6 -0.683714 1.136745 -0.125185
6 -0.964463 -0.286507 -0.282997
6 0.091021 -1.144596 -0.232876
1 -0.033742 -2.217029 -0.342815
7 1.371245 -0.707278 -0.046700
7 0.604106 1.546441 0.052798
8 2.826101 1.024190 0.267787
8 -1.624203 1.993980 -0.160843
6 -2.362884 -0.793230 -0.465420
1 -2.924352 -0.143843 -1.140802
1 -2.342704 -1.803747 -0.891642
8 -3.128800 -0.803163 0.766573
1 -2.659506 -1.371489 1.396220
6 2.496695 -1.646371 -0.004023
1 3.196207 -1.435944 -0.816449
1 3.025104 -1.561118 0.947908
1 2.111752 -2.660516 -0.113390
d_1m5hmU/d_1m5hmU5.xyz
6 -1.631069 0.669298 0.096158
6 0.681730 1.120951 -0.130338

6 0.954485 -0.306423 -0.260531
6 -0.106782 -1.156783 -0.204853
1 0.011505 -2.231349 -0.298241
7 -1.385430 -0.708204 -0.036851
7 -0.603613 1.540791 0.041385
8 -2.828908 1.036215 0.260672
8 1.626007 1.973322 -0.183404
6 2.347000 -0.816318 -0.446912
1 2.324010 -1.845394 -0.825824
1 2.885240 -0.191034 -1.168442
8 3.048022 -0.783132 0.824394
1 3.982021 -0.975236 0.651457
6 -2.518192 -1.638788 0.005217
1 -3.045184 -1.552697 0.957897
1 -3.216622 -1.419681 -0.805746
1 -2.141665 -2.655574 -0.108579
d_1m5hmU/d_1m5hmU7.xyz
6 -1.702834 0.515728 0.006426
6 0.501008 1.366285 0.002529
6 1.035687 0.011463 -0.011607
6 0.148074 -1.014650 -0.015746
1 0.456351 -2.053278 -0.029636
7 -1.205999 -0.792326 -0.004612
7 -0.845381 1.558778 0.011478
8 -2.959526 0.670495 0.011455
8 1.293502 2.365999 0.004422
6 2.532161 -0.178480 -0.021662
1 2.960347 0.289109 -0.914434
1 2.976171 0.327235 0.846257
8 2.946928 -1.550354 -0.061998
1 2.716529 -1.968179 0.781268
6 -2.152804 -1.910719 -0.012908
1 -2.798578 -1.866917 0.867146
1 -2.775331 -1.875154 -0.910377
1 -1.590815 -2.844856 -0.001513
d_1m5hmU/d_1m5hmU6.xyz

6 -1.706323 0.511457 0.007123
6 0.496366 1.365543 -0.005124
6 1.032349 0.011605 -0.009662
6 0.146709 -1.016744 -0.016407
1 0.455642 -2.054344 -0.022996
7 -1.207672 -0.795479 -0.015160
7 -0.850227 1.555577 0.006169
8 -2.963151 0.664722 0.027258
8 1.287026 2.366637 -0.010195
6 2.522760 -0.174034 -0.009273
1 2.955107 0.285845 -0.908949
1 2.958300 0.342559 0.857101
8 2.845292 -1.572868 0.034496
1 3.809607 -1.653278 0.028521
6 -2.152488 -1.915611 -0.011640
1 -2.753173 -1.904061 0.901386
1 -2.819744 -1.850434 -0.873979
1 -1.590018 -2.848203 -0.060725
d_1m5mC/d_1m5mC_2.xyz
6 1.166859 -0.811242 0.000003
6 -1.210907 -0.805112 -0.000001
6 -1.213885 0.658700 0.000000
6 -0.010507 1.282605 -0.000001
1 0.078927 2.363735 -0.000001
7 1.181529 0.589873 0.000000
7 0.000447 -1.470909 0.000001
8 2.292903 -1.407521 -0.000001
7 -2.357430 -1.452992 0.000000
1 -2.151311 -2.454548 0.000001
6 -2.503023 1.431394 -0.000001
1 -3.113400 1.191559 -0.879045
1 -3.113400 1.191559 0.879044
1 -2.312202 2.509615 -0.000001
6 2.461241 1.299509 0.000000
1 3.046186 1.044416 0.887803
1 3.046191 1.044409 -0.887797

1 2.265291 2.372499 -0.000004
d_1m5mC/d_1m5mC_1.xyz
6 1.181384 -0.811965 0.000002
6 -1.194075 -0.837530 -0.000002
6 -1.210134 0.630694 -0.000001
6 -0.013899 1.268798 -0.000001
1 0.062352 2.351078 -0.000001
7 1.183689 0.590613 -0.000001
7 0.024581 -1.484517 0.000000
8 2.315153 -1.394409 0.000001
7 -2.286419 -1.576062 0.000001
1 -3.106566 -0.971374 0.000003
6 -2.505300 1.396159 0.000000
1 -3.115401 1.160143 -0.881817
1 -3.115401 1.160142 0.881815
1 -2.320274 2.474821 0.000001
6 2.456147 1.313436 0.000001
1 3.043464 1.064096 0.887760
1 3.043470 1.064090 -0.887753
1 2.249428 2.384480 -0.000003
d_1m5mU/d_1m5mU.xyz
6 -1.177909 -0.799973 -0.000030
6 1.186700 -0.831468 -0.000049
6 1.223837 0.626714 0.000012
6 0.029439 1.273967 0.000054
1 -0.043689 2.356201 0.000102
7 -1.165681 0.598644 0.000026
7 -0.011856 -1.479607 -0.000077
8 -2.304007 -1.381321 -0.000052
8 2.272622 -1.501115 -0.000089
6 2.536699 1.356383 0.000029
1 3.137107 1.096510 0.880037
1 3.137095 1.096570 -0.880006
1 2.381776 2.439856 0.000066
6 -2.438992 1.324410 0.000125
1 -3.023081 1.071106 -0.887968

1 -3.022954 1.071082 0.888296
1 -2.231060 2.394719 0.000123
d_1mC/d_1mC_2.xyz
6 0.556203 -0.879444 0.000078
6 -1.662551 -0.027923 0.000014
6 -1.127459 1.324125 -0.000105
6 0.214223 1.499701 -0.000157
1 0.676870 2.480358 -0.000258
7 1.076625 0.428336 -0.000100
7 -0.769836 -1.083628 0.000108
8 1.394314 -1.834714 0.000201
7 -2.966698 -0.209337 0.000039
1 -3.148991 -1.215808 0.000124
1 -1.795212 2.177472 -0.000162
6 2.526598 0.624432 -0.000072
1 2.977287 0.174139 0.888221
1 2.977372 0.173768 -0.888129
1 2.735444 1.694844 -0.000279
d_1mC/d_1mC_1.xyz
6 0.587904 -0.877267 0.000062
6 -1.657236 -0.107606 0.000034
6 -1.166653 1.264770 -0.000110
6 0.168220 1.488279 -0.000160
1 0.595127 2.485333 -0.000263
7 1.065209 0.448374 -0.000102
7 -0.728244 -1.128839 0.000104
8 1.461601 -1.801076 0.000170
7 -2.938480 -0.417614 0.000077
1 -3.479703 0.447982 -0.000007
1 -1.861489 2.097560 -0.000176
6 2.507404 0.694197 -0.000052
1 2.973403 0.259630 0.888178
1 2.973531 0.259255 -0.888028
1 2.679096 1.771161 -0.000264
d_1mU/d_1mU.xyz
6 -0.568545 0.876249 0.000001

6 1.657589 0.078484 0.000000
6 1.166211 -1.284887 0.000003
6 -0.175277 -1.492243 0.000002
1 -0.616438 -2.482808 0.000002
7 -1.053413 -0.442862 -0.000001
7 0.762001 1.107217 0.000000
8 -1.419344 1.811191 0.000003
8 2.910327 0.320554 -0.000004
1 1.860357 -2.116237 0.000006
6 -2.501760 -0.665499 -0.000004
1 -2.691397 -1.738903 0.000001
1 -2.954911 -0.219557 0.888459
1 -2.954905 -0.219567 -0.888475
d_5caC/d_5caC01.xyz
6 -2.193435 -0.073798 0.000011
6 -0.117555 1.002001 -0.000017
6 0.611687 -0.252524 -0.000003
6 -0.165733 -1.378164 0.000007
1 0.258884 -2.374041 0.000012
7 -1.515170 -1.297567 0.000010
7 -1.462757 1.061939 -0.000008
8 -3.447677 -0.092727 -0.000003
7 0.546834 2.163297 0.000000
1 0.036195 3.035345 0.000020
1 1.563872 2.121172 0.000033
6 2.115669 -0.381569 -0.000002
8 2.806918 0.690429 -0.000050
8 2.607199 -1.551347 0.000045
1 -2.076618 -2.142677 0.000016
d_5caC/d_5caC02.xyz
6 -2.193297 -0.260355 0.000006
6 -0.076249 0.968576 -0.000003
6 0.612621 -0.271510 -0.000001
6 -0.196543 -1.409615 0.000002
1 0.300357 -2.375390 0.000003
7 -1.533102 -1.450174 0.000004

7 -1.439656 0.917606 0.000001
8 -3.439993 -0.158857 0.000003
7 0.518837 2.159620 -0.000011
1 -0.011589 3.021407 0.000002
1 1.538584 2.142713 -0.000007
6 2.110430 -0.376296 -0.000001
8 2.784491 0.710893 0.000007
8 2.627638 -1.535650 -0.000007
1 -1.968762 1.786026 0.000000
d_5caC/d_5caC03.xyz
6 2.060928 -0.138842 0.000002
6 0.077957 0.990046 -0.000001
6 -0.621703 -0.261090 -0.000001
6 0.185109 -1.388682 -0.000001
1 -0.276068 -2.370549 0.000000
7 1.532321 -1.368188 0.000001
7 1.433606 1.028788 0.000001
8 3.415654 -0.049075 0.000003
7 -0.564697 2.167576 -0.000004
1 -0.039271 3.030187 -0.000002
1 -1.580639 2.142855 -0.000007
6 -2.123337 -0.394224 -0.000001
8 -2.819388 0.676638 0.000010
8 -2.617729 -1.564435 -0.000009
1 3.785353 -0.948004 0.000003
d_5caC/d_5caC04.xyz
6 -2.057045 -0.204725 -0.000005
6 -0.097774 0.974312 -0.000003
6 0.627392 -0.260720 0.000003
6 -0.156154 -1.405766 0.000004
1 0.329310 -2.375807 0.000011
7 -1.503076 -1.420557 0.000000
7 -1.454436 0.977288 -0.000007
8 -3.414662 -0.209557 -0.000012
7 0.514340 2.167739 -0.000010
1 -0.033096 3.016622 -0.000013

1 1.530392 2.169752 -0.000011
6 2.131268 -0.361163 0.000009
8 2.803906 0.724579 0.000025
8 2.650598 -1.520480 -0.000002
1 -3.729266 0.710167 -0.000017
d_5caC/d_5caC05.xyz
6 -2.204247 -0.210474 0.000001
6 -0.209661 1.006726 -0.000002
6 0.550718 -0.212230 -0.000001
6 -0.219230 -1.390598 0.000002
1 0.291524 -2.349818 0.000003
7 -1.539705 -1.434797 0.000002
7 -1.549268 0.989169 -0.000001
8 -3.473619 -0.222284 0.000001
7 0.389083 2.215933 -0.000006
1 -0.183828 3.047782 0.000004
1 1.398294 2.276841 0.000002
6 1.994957 -0.240036 0.000000
8 2.739509 0.754238 0.000000
8 2.535796 -1.493097 0.000001
1 3.504532 -1.398129 0.000002
d_5caC/d_5caC06.xyz
6 2.202334 -0.198940 0.005343
6 0.197279 1.002140 0.000747
6 -0.560559 -0.223720 -0.013996
6 0.227057 -1.390396 -0.065319
1 -0.251186 -2.367160 -0.124577
7 1.546425 -1.425960 -0.064522
7 1.536712 0.993408 0.016565
8 3.469930 -0.203007 0.054346
7 -0.403273 2.209225 0.004211
1 0.170529 3.040504 0.007911
1 -1.412302 2.266815 -0.025589
6 -2.010780 -0.238738 0.002790
8 -2.727044 0.772224 -0.044538
8 -2.680653 -1.429735 0.075150

1 -2.095935 -2.194807 0.191428
d_5caC/d_5caC07.xyz
6 2.199170 -0.163794 0.061171
6 0.162486 0.957323 -0.116823
6 -0.545370 -0.280426 -0.087938
6 0.272392 -1.425232 -0.090092
1 -0.203687 -2.402575 -0.119940
7 1.592799 -1.410367 -0.031449
7 1.487464 1.009919 -0.030762
8 3.455515 -0.110253 0.209941
7 -0.511877 2.162817 -0.210319
1 0.106896 2.960927 -0.310150
1 -1.277212 2.190111 -0.877951
6 -1.989392 -0.455048 0.028308
8 -2.581082 -1.511568 -0.209484
8 -2.749622 0.592053 0.464768
1 -2.198902 1.356157 0.726195
d_5caC/d_5caC08.xyz
6 -2.208660 -0.150947 0.000000
6 -0.150547 0.880105 -0.000001
6 0.558385 -0.324565 -0.000002
6 -0.285645 -1.450827 -0.000002
1 0.163197 -2.440761 -0.000003
7 -1.609194 -1.399070 -0.000001
7 -1.455531 1.012678 0.000000
8 -3.469481 -0.066316 0.000009
7 0.620936 2.132753 -0.000001
1 0.404432 2.695570 -0.829129
1 1.648592 1.822455 0.000000
6 2.054306 -0.442550 0.000000
8 2.735301 0.651641 0.000002
8 2.569036 -1.593407 -0.000004
1 0.404429 2.695572 0.829125
d_5caU/d_5caU79.xyz
6 2.205607 -0.206265 -0.000002
6 0.054887 0.998674 0.000021

6 -0.583453 -0.284298 0.000002
6 0.240436 -1.415552 -0.000007
1 -0.236843 -2.392414 -0.000016
7 1.570936 -1.420141 -0.000009
7 1.430863 0.954266 0.000010
8 3.449166 -0.092311 -0.000007
8 -0.539405 2.114474 0.000013
1 1.927389 1.841276 0.000010
6 -2.039092 -0.411532 -0.000003
8 -2.647743 -1.486950 -0.000007
8 -2.746572 0.742417 -0.000006
1 -2.097018 1.505061 -0.000006
d_5caU/d_5caU78.xyz
6 -2.191803 -0.177497 -0.024598
6 -0.042756 1.083926 0.070839
6 0.671982 -0.185361 0.007786
6 -0.074404 -1.327206 -0.040661
1 0.389164 -2.304845 -0.073435
7 -1.437241 -1.330006 -0.062006
7 -1.442185 0.971295 0.044458
8 -3.426963 -0.182534 -0.049255
8 0.442429 2.220557 0.160705
6 2.184554 -0.299190 -0.020668
8 2.676660 -1.432471 0.276884
8 2.856638 0.721097 -0.355407
1 -1.944148 -2.208056 -0.111484
1 -1.964584 1.842649 0.088143
d_5caU/d_5caU108.xyz
6 -2.197464 -0.009991 -0.000001
6 -0.090253 1.022367 -0.000003
6 0.575086 -0.280769 0.000000
6 -0.218938 -1.395634 0.000006
1 0.177280 -2.404421 0.000010
7 -1.557963 -1.265524 0.000007
7 -1.439122 1.107812 -0.000003
8 -3.450502 -0.004997 0.000002

8 0.617697 2.091223 -0.000004
6 2.041877 -0.415655 -0.000002
8 2.636892 -1.498609 -0.000001
8 2.728581 0.735580 -0.000004
1 2.030428 1.489889 -0.000004
1 -2.151310 -2.088969 0.000011
d_5caU/d_5caU106.xyz
6 2.195724 -0.023410 -0.000006
6 0.102978 0.971582 -0.000013
6 -0.589688 -0.290751 -0.000002
6 0.209878 -1.401355 0.000005
1 -0.182839 -2.410956 0.000013
7 1.552679 -1.268188 0.000005
7 1.421949 1.097905 -0.000010
8 3.443030 0.002061 0.000001
8 -0.612555 2.091236 -0.000002
6 -2.086286 -0.379472 0.000003
8 -2.640210 -1.507465 0.000011
8 -2.713309 0.747550 0.000000
1 -1.595649 1.769697 0.000002
1 2.144809 -2.093381 0.000012
d_5caU/d_5caU76.xyz
6 -2.198057 -0.299047 -0.000002
6 -0.137825 1.091829 0.000006
6 0.599828 -0.149799 0.000009
6 -0.143705 -1.341478 0.000019
1 0.398900 -2.281992 0.000029
7 -1.468641 -1.458273 0.000014
7 -1.517663 0.911505 0.000015
8 -3.448779 -0.288337 -0.000029
8 0.313067 2.257363 0.000000
1 -2.083155 1.755861 0.000003
6 2.053549 -0.166964 -0.000004
8 2.585859 -1.425946 -0.000015
8 2.798674 0.817085 -0.000005
1 3.555080 -1.335055 -0.000020

d_5caU/d_5caU303.xyz
6 2.213208 -0.245128 -0.000026
6 0.094230 1.060829 0.000020
6 -0.589440 -0.210943 0.000010
6 0.205627 -1.368288 0.000006
1 -0.308237 -2.325846 0.000004
7 1.533068 -1.433886 -0.000002
7 1.481456 0.934836 -0.000006
8 3.462386 -0.182079 -0.000005
8 -0.396859 2.210022 0.000004
1 2.011332 1.801876 -0.000006
6 -2.035266 -0.386285 0.000008
8 -2.604521 -1.485684 -0.000004
8 -2.764596 0.758857 0.000000
1 -3.706195 0.507276 -0.000008
d_5caU/d_5caU304.xyz
6 2.194943 -0.285456 0.019804
6 0.122182 1.082361 -0.067470
6 -0.605891 -0.164728 0.013917
6 0.150061 -1.341775 0.118657
1 -0.369452 -2.291007 0.234730
7 1.477074 -1.444817 0.132528
7 1.502532 0.915822 -0.066121
8 3.445377 -0.263802 -0.003306
8 -0.342800 2.238250 -0.155348
1 2.059436 1.762870 -0.138046
6 -2.066770 -0.169950 0.045955
8 -2.725866 -1.323303 -0.290289
8 -2.772230 0.785046 0.369742
1 -2.150223 -1.981129 -0.713101
d_5caU/d_5caU307.xyz
6 2.065913 -0.145621 0.000006
6 0.072174 0.994947 0.000004
6 -0.590872 -0.287603 -0.000003
6 0.210692 -1.421259 -0.000005
1 -0.247483 -2.405953 -0.000011

7 1.550697 -1.386711 -0.000002
7 1.436663 1.021437 0.000008
8 3.421690 -0.122205 0.000007
8 -0.585683 2.096051 0.000006
1 3.715199 0.804786 0.000010
6 -2.056760 -0.397532 -0.000006
8 -2.676592 -1.467540 -0.000011
8 -2.729315 0.767267 -0.000004
1 -2.026917 1.511909 0.000000
d_5caU/d_5caU311.xyz
6 -2.067734 -0.080896 -0.000002
6 -0.052200 1.009736 -0.000004
6 0.585410 -0.286710 0.000000
6 -0.238112 -1.403242 0.000006
1 0.195878 -2.398845 0.000008
7 -1.577146 -1.334398 0.000005
7 -1.414902 1.071618 -0.000005
8 -3.418834 0.034198 0.000003
8 0.633138 2.094731 -0.000011
1 -3.806565 -0.857500 0.000009
6 2.049181 -0.426718 -0.000002
8 2.645901 -1.509933 0.000006
8 2.744842 0.723594 0.000002
1 2.055377 1.482064 -0.000001
d_5caU/d_5caU105.xyz
6 -2.206959 -0.106700 0.000007
6 -0.182386 1.126862 0.000002
6 0.591495 -0.130930 0.000001
6 -0.118073 -1.308551 0.000001
1 0.352307 -2.283510 -0.000001
7 -1.460849 -1.302541 0.000002
7 -1.550200 1.062458 0.000002
8 -3.459381 -0.216918 -0.000004
8 0.377246 2.256669 -0.000004
6 2.055357 -0.174575 -0.000001
8 2.556112 -1.444729 0.000002

8 2.818044 0.790627 -0.000004
1 3.527594 -1.380695 0.000000
1 -1.975333 -2.177051 0.000000
d_5caU/d_5caU316.xyz
6 -2.066946 -0.094663 0.000165
6 -0.067066 0.956823 -0.000039
6 0.600371 -0.296907 -0.000046
6 -0.227123 -1.410237 0.000058
1 0.203746 -2.406410 0.000059
7 -1.569505 -1.337006 0.000166
7 -1.399579 1.059884 0.000051
8 -3.411363 0.038648 0.000275
8 0.626770 2.094086 -0.000152
1 -3.816512 -0.845435 0.000333
6 2.097352 -0.388396 -0.000137
8 2.650689 -1.517998 -0.000142
8 2.729961 0.736295 -0.000197
1 1.608372 1.783720 -0.000191
d_5caU/d_5caU312.xyz
6 2.065059 -0.156084 0.000109
6 0.085091 0.941676 -0.000017
6 -0.605592 -0.297851 -0.000053
6 0.200917 -1.427716 -0.000018
1 -0.252789 -2.413692 -0.000034
7 1.544024 -1.386724 0.000067
7 1.420594 1.012757 0.000069
8 3.415867 -0.119978 0.000195
8 -0.582850 2.093689 -0.000032
1 3.708024 0.807502 0.000209
6 -2.103971 -0.358682 -0.000104
8 -2.680780 -1.476376 -0.000121
8 -2.712914 0.779193 -0.000111
1 -1.571179 1.803684 -0.000077
d_5caU/d_5caU09.xyz
6 -2.215308 -0.052693 0.000000
6 -0.138665 1.096879 -0.000001

6 0.580470 -0.193038 0.000002
6 -0.180078 -1.338019 0.000000
1 0.260591 -2.327712 0.000000
7 -1.520536 -1.279276 0.000000
7 -1.508716 1.086742 -0.000001
8 -3.471290 -0.109373 0.000001
8 0.462077 2.205284 -0.000003
6 2.035021 -0.391643 0.000000
8 2.576669 -1.502807 -0.000005
8 2.783266 0.733726 0.000006
1 -2.070440 -2.131912 0.000005
1 3.720192 0.463804 0.000003
d_5caU/d_5caU208.xyz
6 -2.183679 -0.118652 0.000003
6 -0.136209 0.991599 0.000003
6 0.637975 -0.215365 0.000000
6 -0.120616 -1.361503 0.000000
1 0.329687 -2.345538 -0.000002
7 -1.468298 -1.316559 0.000000
7 -1.459662 1.034323 0.000002
8 -3.429530 -0.150229 0.000003
8 0.494641 2.174829 -0.000002
1 -0.181370 2.877650 -0.000003
6 2.151326 -0.335353 -0.000001
8 2.622827 -1.515850 0.000000
8 2.844495 0.723553 -0.000005
1 -2.004838 -2.179237 -0.000001
d_5caU/d_5caU59.xyz
6 2.200059 -0.091513 0.025404
6 0.163515 1.108907 -0.118881
6 -0.593923 -0.155113 -0.008164
6 0.125525 -1.318905 0.076759
1 -0.325297 -2.300346 0.177739
7 1.471808 -1.294866 0.080668
7 1.529321 1.066256 -0.088889
8 3.452767 -0.179176 0.084969

8 -0.422132 2.216036 -0.262012
6 -2.062181 -0.178030 0.085000
8 -2.722902 -1.291225 -0.351312
8 -2.748323 0.724776 0.552525
1 -2.152638 -1.897441 -0.851766
1 1.996787 -2.157303 0.177519
d_5caU/d_5caU305.xyz
6 -2.213586 -0.170088 -0.000001
6 -0.204114 0.981008 0.000000
6 0.535584 -0.236584 -0.000001
6 -0.254221 -1.401971 -0.000001
1 0.235536 -2.372286 -0.000001
7 -1.574364 -1.406269 -0.000001
7 -1.519929 1.023263 0.000000
8 -3.475477 -0.143509 0.000001
8 0.425766 2.176193 0.000003
1 1.399134 1.999463 0.000005
6 1.979010 -0.242693 -0.000001
8 2.550865 -1.466784 0.000001
8 2.680174 0.788037 -0.000002
1 3.518712 -1.355665 0.000001
d_5caU/d_5caU306.xyz
6 2.220561 -0.135361 -0.000001
6 0.174502 0.963686 0.000000
6 -0.532375 -0.273527 0.000001
6 0.297424 -1.412491 0.000002
1 -0.173139 -2.392809 0.000003
7 1.616281 -1.387230 0.000001
7 1.490320 1.034733 -0.000001
8 3.481896 -0.071891 -0.000002
8 -0.450632 2.165955 0.000000
1 -3.633349 0.525779 -0.000001
6 -1.972933 -0.454314 0.000000
8 -2.558854 -1.538811 0.000000
8 -2.677824 0.716048 0.000000
1 -1.419478 2.036147 0.000001

d_5caU/d_5caU309.xyz
6 2.212214 -0.158190 -0.003259
6 0.192807 0.976441 0.003986
6 -0.544018 -0.248953 0.005769
6 0.262539 -1.403630 0.026750
1 -0.197141 -2.390562 0.049210
7 1.581383 -1.398334 0.026506
7 1.508194 1.027786 -0.001167
8 3.472740 -0.123948 -0.030666
8 -0.442824 2.165341 0.007846
1 -1.417248 1.973707 0.011950
6 -1.992496 -0.240553 -0.005015
8 -2.698928 -1.394782 -0.024153
8 -2.662902 0.809738 0.002329
1 -2.143629 -2.190791 -0.050761
d_5caU/d_5caU308.xyz
6 -2.206257 -0.159224 -0.000001
6 -0.166085 0.936741 0.000002
6 0.555739 -0.282701 0.000000
6 -0.269581 -1.424205 -0.000002
1 0.202804 -2.403222 -0.000004
7 -1.590452 -1.403305 -0.000003
7 -1.477209 1.016388 0.000002
8 -3.465948 -0.098780 -0.000001
8 0.541914 2.096955 0.000005
1 -0.066673 2.857408 0.000007
6 2.008343 -0.442334 0.000000
8 2.574450 -1.539538 -0.000002
8 2.793635 0.666648 -0.000001
1 2.252137 1.482302 0.000000
d_5caU/d_5caU223.xyz
6 -2.053878 -0.101047 0.028576
6 -0.054732 1.083896 -0.123940
6 0.674821 -0.184428 -0.016044
6 -0.053558 -1.332886 0.051771
1 0.394156 -2.316754 0.103868

7 -1.420176 -1.299890 0.075181
7 -1.447166 1.045520 -0.071313
8 -3.387193 -0.202352 0.094494
8 0.503213 2.191546 -0.282533
1 -3.781175 0.686943 0.052476
6 2.188286 -0.285856 0.043684
8 2.714519 -1.325484 -0.462553
8 2.821341 0.647306 0.621544
1 -1.962259 -2.155794 0.144686
d_5dhmC/d_5dhmC.xyz
6 2.253299 -0.242669 0.063474
6 0.284407 0.991574 -0.153485
6 -0.493890 -0.197366 -0.133114
6 0.238046 -1.371666 -0.009337
1 -0.284354 -2.323878 0.003495
7 1.571986 -1.438099 0.094459
7 1.619297 0.966674 -0.060499
8 3.531145 -0.258235 0.159706
6 -1.993314 -0.155546 -0.275234
1 -2.293531 0.335189 -1.209415
7 -0.301997 2.223438 -0.316955
1 0.293079 3.016997 -0.113511
1 -1.265016 2.319378 -0.020567
8 -2.613956 0.648553 0.737172
1 -2.268435 0.364087 1.599556
8 -2.503977 -1.476280 -0.241630
1 -3.443714 -1.434138 -0.474406
d_5dhmU/d_5dhmU_52.xyz
6 -2.284487 -0.101134 0.000000
6 -0.020896 0.889079 0.000003
6 0.484246 -0.446773 -0.000006
6 -0.432166 -1.480790 -0.000013
1 -0.061499 -2.504812 -0.000021
7 -1.776317 -1.358310 -0.000010
7 -1.400469 0.980086 0.000007
8 -3.520507 0.147106 0.000006

8 0.687493 1.934968 0.000011
6 1.972282 -0.671820 -0.000011
8 2.600340 -0.148067 1.170784
1 2.327548 0.780917 1.260521
8 2.600339 -0.148002 -1.170777
1 2.327542 0.780984 -1.260467
1 2.196763 -1.739897 -0.000040
1 -1.808040 1.910963 0.000015
d_5dhmU/d_5dhmU_50.xyz
6 -2.283833 -0.131049 0.016839
6 -0.044961 0.917274 0.047635
6 0.490835 -0.390088 -0.153404
6 -0.403849 -1.439471 -0.262101
1 -0.007835 -2.441080 -0.424308
7 -1.747155 -1.360891 -0.186038
7 -1.426005 0.965169 0.114353
8 -3.522687 0.075756 0.118584
8 0.625148 1.985909 0.147901
6 1.979052 -0.603199 -0.199256
8 2.663957 0.425501 -0.896758
1 2.265799 1.269286 -0.596802
8 2.454083 -0.689100 1.152491
1 3.399534 -0.906736 1.118815
1 2.220747 -1.533246 -0.723964
1 -1.853588 1.876492 0.252033
d_5dhmU/d_5dhmU_56.xyz
6 -2.276466 -0.121961 0.012963
6 -0.034548 0.918682 0.044885
6 0.496045 -0.389729 -0.168200
6 -0.402086 -1.435413 -0.282324
1 -0.010145 -2.437208 -0.453767
7 -1.745052 -1.352205 -0.200033
7 -1.414474 0.971221 0.118071
8 -3.514244 0.088958 0.118083
8 0.642534 1.981887 0.148874
6 1.985121 -0.608612 -0.206711

8 2.680935 0.409698 -0.889983
1 2.281482 1.255970 -0.598906
8 2.546847 -0.655390 1.126206
1 2.009554 -1.265681 1.655569
1 2.217867 -1.548420 -0.719311
1 -1.839060 1.883199 0.261044
d_5dhmU/d_5dhmU_27.xyz
6 2.253688 0.100051 0.000005
6 0.045581 0.911323 -0.000004
6 -0.475159 -0.452572 -0.000013
6 0.418205 -1.472598 -0.000025
1 0.136550 -2.520020 -0.000038
7 1.756348 -1.203343 -0.000021
7 1.385388 1.135192 0.000008
8 3.507308 0.249627 0.000023
8 -0.776355 1.887089 -0.000008
6 -1.964454 -0.680957 -0.000006
8 -2.579906 -0.148969 1.170923
1 -2.300674 0.781630 1.238634
8 -2.579929 -0.148908 -1.170890
1 -2.300672 0.781687 -1.238569
1 -2.193657 -1.748187 -0.000028
1 2.430193 -1.960242 -0.000030
d_5dhmU/d_5dhmU_53.xyz
6 2.227738 -0.364644 0.066444
6 0.248231 1.104768 -0.181207
6 -0.544433 -0.085426 -0.140116
6 0.115316 -1.293350 0.003575
1 -0.473692 -2.205959 0.039901
7 1.448754 -1.474946 0.104300
7 1.613820 0.876848 -0.086383
8 3.484913 -0.396573 0.163168
8 -0.176283 2.285172 -0.291848
6 -2.041452 0.044255 -0.181396
8 -2.596594 0.359394 1.100915
1 -2.217937 -0.256741 1.749356

8 -2.599593 -1.163818 -0.678012
1 -3.557715 -1.035436 -0.749605
1 -2.340697 0.886143 -0.812620
1 2.220069 1.691666 -0.110033
d_5dhmU/d_5dhmUa11.xyz
6 -2.259387 0.064081 0.046008
6 -0.073965 0.943786 0.047492
6 0.483946 -0.390580 -0.153092
6 -0.386405 -1.425394 -0.270348
1 -0.075881 -2.452203 -0.431363
7 -1.728371 -1.206864 -0.181324
7 -1.418223 1.115702 0.143154
8 -3.513490 0.168916 0.143005
8 0.704688 1.955180 0.119511
6 1.973919 -0.614504 -0.191327
1 -2.378882 -1.979608 -0.264648
8 2.654223 0.407112 -0.896813
1 2.216804 1.243301 -0.609129
8 2.434396 -0.680646 1.166212
1 3.382428 -0.887761 1.143672
1 2.214498 -1.554422 -0.699061
d_5dhmU/d_5dhmU_30.xyz
6 -2.251202 0.071421 0.045046
6 -0.064630 0.946102 0.045061
6 0.489498 -0.387758 -0.171373
6 -0.383441 -1.419500 -0.297501
1 -0.076209 -2.445182 -0.472274
7 -1.724706 -1.198064 -0.199934
7 -1.407188 1.120662 0.149873
8 -3.504334 0.178354 0.149533
8 0.718921 1.953333 0.120295
6 1.980643 -0.617231 -0.199801
8 2.672005 0.402763 -0.879550
1 2.225338 1.237609 -0.600197
8 2.523910 -0.654608 1.139603
1 2.029048 -1.317395 1.646977

1 2.213861 -1.562331 -0.702813
1 -2.378001 -1.967823 -0.288894
d_5dhmU/d_5dhmU_48.xyz
6 2.258591 -0.276506 0.063017
6 0.171446 1.030675 -0.138862
6 -0.527810 -0.214744 -0.141840
6 0.227258 -1.370166 -0.058558
1 -0.284447 -2.327865 -0.077043
7 1.571574 -1.444132 0.046262
7 1.545834 0.918277 -0.038963
8 3.513621 -0.210698 0.165285
8 -0.359479 2.177142 -0.208633
6 -2.029977 -0.191291 -0.292441
8 -2.557133 -1.458009 0.024528
1 -3.466973 -1.495606 -0.306330
8 -2.633569 0.763747 0.597117
1 -2.144268 1.599727 0.460426
1 -2.313005 0.078588 -1.320749
1 2.082257 1.780875 -0.021651
d_5dhmU/d_5dhmUa28.xyz
6 2.255104 -0.070325 0.054733
6 0.195978 1.061532 -0.105168
6 -0.525709 -0.208920 -0.153280
6 0.212024 -1.347632 -0.100451
1 -0.216559 -2.341636 -0.143304
7 1.569518 -1.282903 0.008207
7 1.550140 1.079762 -0.006296
8 3.512621 -0.107935 0.155260
8 -0.451643 2.164679 -0.143843
6 -2.034599 -0.234547 -0.325961
1 2.118020 -2.133897 0.053613
8 -2.608054 -1.475599 0.019860
1 -2.369299 -1.679357 0.939865
8 -2.655600 0.781056 0.460849
1 -2.064886 1.566707 0.361609
1 -2.300264 -0.088100 -1.381406

d_5dhmU/d_5dhmU_29.xyz
6 2.244347 -0.158393 0.057856
6 0.276580 1.124484 -0.187823
6 -0.535833 -0.091742 -0.148475
6 0.107267 -1.279901 -0.011412
1 -0.399935 -2.235979 0.025447
7 1.465943 -1.316345 0.086350
7 1.635756 1.035261 -0.085463
8 3.496226 -0.293935 0.169066
8 -0.274106 2.261130 -0.318990
6 -2.035927 0.015421 -0.197740
8 -2.577277 -1.236590 -0.592651
1 -3.537294 -1.128731 -0.671325
8 -2.582969 0.422848 1.058331
1 -2.210002 -0.152243 1.746782
1 -2.345213 0.800153 -0.893389
1 1.946942 -2.202445 0.185787
d_5dhmU/d_5dhmU_24.xyz
6 2.258928 -0.078826 0.052376
6 0.204443 1.058394 -0.128343
6 -0.521153 -0.209924 -0.141388
6 0.208584 -1.351426 -0.059597
1 -0.223966 -2.343683 -0.072725
7 1.567387 -1.288802 0.042113
7 1.560169 1.072762 -0.038095
8 3.517136 -0.120701 0.149158
8 -0.440057 2.161229 -0.190315
6 -2.024520 -0.206882 -0.295419
8 -2.536189 -1.474165 0.049274
1 -3.438426 -1.538583 -0.297839
8 -2.629737 0.766083 0.563693
1 -2.089568 1.578190 0.429429
1 -2.300364 0.029363 -1.334017
1 2.112515 -2.140593 0.106758
d_5dhmU/d_5dhmUa21.xyz
6 2.253710 -0.143992 0.059972

6 0.271706 1.115993 -0.189551
6 -0.527911 -0.107149 -0.128498
6 0.127449 -1.287138 0.015635
1 -0.369919 -2.247788 0.064606
7 1.487126 -1.309312 0.104089
7 1.632389 1.041767 -0.092750
8 3.507581 -0.265802 0.165898
8 -0.290961 2.245201 -0.336092
6 -2.025629 -0.016682 -0.193150
1 1.977364 -2.189722 0.208192
8 -2.561818 -1.282188 -0.554521
1 -3.501978 -1.163058 -0.756481
8 -2.504583 0.383654 1.094215
1 -3.406808 0.724616 0.989461
1 -2.332973 0.735663 -0.927591
d_5dhmU/d_5dhmUa27.xyz
6 2.255328 -0.135921 0.066708
6 0.265583 1.110954 -0.181053
6 -0.528301 -0.118540 -0.131965
6 0.138043 -1.294658 -0.013701
1 -0.343702 -2.265041 0.004481
7 1.497944 -1.307477 0.082625
7 1.625754 1.047013 -0.073672
8 3.509095 -0.249255 0.181889
8 -0.304593 2.235602 -0.330899
6 -2.031019 -0.027383 -0.222265
1 1.994408 -2.186131 0.170891
8 -2.669768 -1.250017 -0.556428
1 -2.550074 -1.875928 0.176225
8 -2.511333 0.457981 1.029056
1 -3.437188 0.724128 0.911403
1 -2.314351 0.665014 -1.020948
d_5dhmU/d_5dhmUa29.xyz
6 2.246202 -0.141396 0.064626
6 0.260957 1.112147 -0.185762
6 -0.537606 -0.115919 -0.144157

6 0.126346 -1.294738 -0.030620
1 -0.356546 -2.264647 -0.021571
7 1.485629 -1.311013 0.072462
7 1.620274 1.043756 -0.073223
8 3.499031 -0.258522 0.184260
8 -0.304970 2.238643 -0.334063
6 -2.043327 -0.015435 -0.226283
1 1.979656 -2.191338 0.158169
8 -2.681150 -1.239912 -0.556579
1 -2.551899 -1.873596 0.167153
8 -2.591929 0.529530 0.971897
1 -2.165729 0.094718 1.728089
1 -2.330087 0.679796 -1.017457
d_5dhmU/d_5dhmUa13.xyz
6 2.277199 0.045087 -0.000003
6 0.098700 0.970763 0.000010
6 -0.482574 -0.375273 -0.000011
6 0.371127 -1.431779 0.000000
1 0.040889 -2.464990 -0.000005
7 1.719054 -1.234396 0.000020
7 1.458373 1.114064 -0.000001
8 3.538419 0.133680 -0.000021
8 -0.639392 2.001548 0.000033
6 -1.963716 -0.620246 -0.000014
1 2.352392 -2.025329 0.000020
8 -2.537331 -0.049001 1.175042
1 -3.436458 -0.398982 1.272703
8 -2.537332 -0.048954 -1.175050
1 -3.436457 -0.398935 -1.272727
1 -2.161685 -1.698921 -0.000037
d_5dhmU/d_5dhmUa26.xyz
6 2.239373 -0.160698 0.057513
6 0.276990 1.130015 -0.187505
6 -0.541516 -0.081820 -0.132806
6 0.096270 -1.271701 0.012216
1 -0.418232 -2.223949 0.058876

7 1.455018 -1.314596 0.103777
7 1.636328 1.035046 -0.094029
8 3.491080 -0.302032 0.161560
8 -0.270275 2.267849 -0.322952
6 -2.046043 0.026256 -0.184592
1 1.932673 -2.202203 0.206021
8 -2.672595 -1.208426 -0.545978
1 -2.391038 -1.446819 -1.443443
8 -2.595795 0.405983 1.063805
1 -2.187326 -0.142073 1.753696
1 -2.345263 0.812583 -0.883821
d_5dhmU/d_5dhmUa24.xyz
6 2.250902 -0.148776 0.061308
6 0.276088 1.122009 -0.187124
6 -0.530927 -0.095558 -0.114728
6 0.117461 -1.278746 0.031100
1 -0.389489 -2.234501 0.083481
7 1.477266 -1.309399 0.114906
7 1.636713 1.040415 -0.096088
8 3.504333 -0.278010 0.163283
8 -0.281609 2.253436 -0.337322
6 -2.032652 -0.005030 -0.186332
1 1.963132 -2.192245 0.218993
8 -2.652119 -1.256388 -0.496753
1 -2.492553 -1.452599 -1.433357
8 -2.519370 0.381722 1.086185
1 -3.445664 0.653877 0.986753
1 -2.328392 0.738882 -0.936084
d_5dhmU/d_5dhmUa17.xyz
6 -2.274415 0.049624 0.003268
6 -0.094271 0.971215 -0.009158
6 0.484621 -0.375779 -0.034351
6 -0.371730 -1.430649 -0.044298
1 -0.043056 -2.464423 -0.060585
7 -1.719173 -1.230858 -0.031472
7 -1.453616 1.117074 0.011520

8 -3.535301 0.140362 0.026145
8 0.645667 2.000400 -0.004863
6 1.967180 -0.626402 -0.014468
1 -2.354600 -2.020108 -0.039119
8 2.569038 -0.032472 -1.146813
1 3.495750 -0.317800 -1.177803
8 2.610531 -0.060720 1.140296
1 2.371089 -0.600867 1.909684
1 2.152556 -1.708930 -0.016592
d_5dhmU/d_5dhmUb15_b.xyz
6 -2.130644 -0.013966 0.018239
6 -0.031001 0.922160 0.039251
6 0.496558 -0.405871 -0.150065
6 -0.403332 -1.439558 -0.254236
1 -0.053913 -2.458517 -0.406212
7 -1.748889 -1.278650 -0.174553
7 -1.385860 1.080028 0.123688
8 -3.475312 0.215486 0.121249
8 0.740508 1.944954 0.118329
6 1.987257 -0.640698 -0.182977
1 -3.936817 -0.634652 0.031032
8 2.661380 0.356549 -0.931988
1 2.229395 1.203618 -0.671547
8 2.536939 -0.730410 1.140845
1 2.261494 0.057465 1.637866
1 2.221942 -1.602594 -0.643833
d_5dhmU/d_5dhmU_13.xyz
6 -2.138080 -0.085350 0.015037
6 -0.063516 0.919186 0.054445
6 0.500333 -0.389939 -0.154254
6 -0.370920 -1.446979 -0.278763
1 0.011388 -2.451226 -0.449217
7 -1.720068 -1.333279 -0.200036
7 -1.425317 1.029530 0.141057
8 -3.496673 0.038994 0.113027
8 0.669434 1.968415 0.143780

6 1.993350 -0.586685 -0.206629
8 2.654384 0.474012 -0.873589
1 2.186478 1.290146 -0.572417
8 2.464435 -0.700159 1.146119
1 3.413846 -0.898770 1.109612
1 2.249958 -1.501993 -0.750673
1 -3.703620 0.976601 0.261841
d_5fC/d_5fC.xyz
6 1.940843 0.037852 -0.000020
6 -0.257254 0.820740 0.000005
6 -0.752460 -0.530726 0.000009
6 0.243914 -1.528184 0.000000
1 -0.068975 -2.572487 0.000004
7 1.542616 -1.301324 -0.000013
7 1.054374 1.082202 -0.000009
8 3.184099 0.284447 -0.000040
7 -1.112703 1.861894 0.000015
1 -0.750826 2.805503 0.000008
1 -2.109795 1.686328 0.000021
6 -2.128665 -0.908440 0.000020
1 -2.314005 -1.997792 0.000022
8 -3.112184 -0.130496 0.000029
d_5fU/d_5fUd.xyz
6 -1.869876 -0.362286 0.000001
6 0.111600 1.127184 0.000000
6 0.908703 -0.074970 -0.000002
6 0.232435 -1.306815 -0.000001
1 0.835040 -2.212725 0.000002
7 -1.082453 -1.489384 0.000001
7 -1.256201 0.886034 0.000001
8 -3.117619 -0.417578 -0.000001
8 0.529045 2.306299 -0.000001
6 2.344088 0.006780 0.000001
1 2.761482 1.026978 0.000006
8 3.123508 -0.967286 -0.000001
1 -1.867118 1.698360 -0.000001

d_5fU/d_5fUda.xyz
6 -1.943258 0.078144 0.000007
6 0.337557 -0.903534 -0.000016
6 0.830214 0.456021 -0.000015
6 -0.124338 1.488893 -0.000027
1 0.241557 2.515079 -0.000042
7 -1.445390 1.357092 -0.000020
7 -1.050133 -0.987958 -0.000014
8 -3.169006 -0.164760 0.000046
8 1.012678 -1.952827 -0.000026
6 2.223054 0.810896 0.000009
1 2.411190 1.901264 -0.000001
8 3.197335 0.034979 0.000045
1 -1.451510 -1.921941 0.000006
d_5fU/d_5fUa.xyz
6 -1.888938 -0.160905 -0.000003
6 0.074641 1.151528 0.000001
6 0.897082 -0.068810 0.000001
6 0.247723 -1.281102 0.000008
1 0.776315 -2.228116 0.000019
7 -1.091602 -1.330472 0.000000
7 -1.288008 1.040335 -0.000007
8 -3.133098 -0.330411 -0.000003
8 0.613875 2.294165 0.000006
6 2.344837 -0.011455 0.000001
1 2.779389 1.000017 0.000013
8 3.097356 -1.000470 -0.000010
1 -1.575562 -2.222752 0.000033
d_5fU/d_5fUaa.xyz
6 -1.917944 -0.109998 -0.010672
6 0.297015 -0.941670 0.015616
6 0.821389 0.437997 0.013398
6 -0.095809 1.466446 0.026353
1 0.197101 2.511837 0.037499
7 -1.411325 1.209850 0.023171
7 -1.056052 -1.138386 -0.007901

8 -3.167491 -0.234445 -0.040650
8 1.071511 -1.935337 0.040094
6 2.219032 0.815391 -0.008872
1 2.390881 1.908628 0.007523
8 3.199424 0.057567 -0.048909
1 -2.086002 1.968014 0.028861
d_5fU/d_5fUfa.xyz
6 -1.937277 -0.074462 0.000001
6 0.261323 -0.788935 0.000002
6 0.735990 0.558154 -0.000004
6 -0.277134 1.539072 0.000005
1 0.009064 2.590282 0.000009
7 -1.567434 1.271123 0.000010
7 -1.013941 -1.105239 0.000007
8 -3.164652 -0.358523 -0.000014
8 1.142812 -1.809535 0.000005
6 2.122019 0.889540 -0.000006
1 2.376791 1.961270 -0.000008
8 3.047053 0.037646 -0.000005
1 2.052540 -1.409664 0.000003
d_5fU/d_5fUc.xyz
6 -1.741549 -0.273025 0.000001
6 0.098251 1.131599 0.000000
6 0.915283 -0.070996 0.000000
6 0.257186 -1.302229 -0.000001
1 0.842063 -2.218515 -0.000005
7 -1.070697 -1.446989 -0.000002
7 -1.272589 0.960238 0.000001
8 -3.089233 -0.432170 0.000001
8 0.578537 2.300110 -0.000004
6 2.357505 0.007556 0.000004
1 2.779394 1.025477 0.000012
8 3.131564 -0.968413 -0.000001
1 -3.505455 0.446642 0.000004
d_5fU/d_5fUca.xyz
6 -1.797807 0.045300 0.000000

6 0.311532 -0.917788 0.000000
6 0.843488 0.439297 0.000001
6 -0.079083 1.489833 -0.000001
1 0.282452 2.516622 -0.000001
7 -1.405963 1.337661 -0.000001
7 -1.063969 -1.051141 -0.000003
8 -3.147213 -0.102766 0.000002
8 1.031162 -1.952342 0.000000
6 2.244644 0.784958 0.000001
1 2.440959 1.874426 0.000001
8 3.213887 0.005954 0.000000
1 -3.353217 -1.053046 0.000003
d_5fU/d_5fUb.xyz
6 -1.749599 -0.204859 0.000001
6 0.124736 1.143528 -0.000001
6 0.911646 -0.080045 -0.000001
6 0.223169 -1.293403 -0.000002
1 0.781353 -2.226069 0.000001
7 -1.108989 -1.397578 0.000000
7 -1.249774 1.013658 -0.000002
8 -3.105720 -0.266261 0.000002
8 0.639038 2.297917 -0.000001
6 2.356002 -0.038159 0.000004
1 2.803178 0.968973 0.000013
8 3.104313 -1.033625 -0.000001
1 -3.369970 -1.202085 -0.000002
d_5fU/d_5fUba.xyz
6 -1.795060 0.017279 -0.000001
6 0.332759 0.924975 0.000002
6 0.835349 -0.443913 0.000002
6 -0.109287 -1.473566 0.000003
1 0.227352 -2.508722 0.000004
7 -1.432106 -1.285769 0.000002
7 -1.037290 1.094484 -0.000001
8 -3.130567 0.260268 -0.000004
8 1.078620 1.941200 0.000004

6 2.229020 -0.819625 0.000000
1 2.402381 -1.913028 0.000003
8 3.214279 -0.061326 -0.000006
1 -3.599311 -0.591296 -0.000003
d_5fU/d_5fUe.xyz
6 1.877409 -0.301619 0.000001
6 -0.026444 1.018734 0.000000
6 -0.875856 -0.118719 -0.000005
6 -0.184901 -1.349968 0.000006
1 -0.766133 -2.269523 -0.000001
7 1.128209 -1.477300 0.000006
7 1.287667 0.948239 0.000003
8 3.134946 -0.381662 -0.000006
8 -0.598598 2.245754 0.000000
6 -2.312578 -0.034960 0.000002
1 -2.739487 0.980779 0.000015
8 -3.083768 -1.014656 -0.000005
1 0.108060 2.915873 -0.000002
d_5fU/d_5fUea.xyz
6 1.937147 -0.006359 0.000000
6 -0.234109 0.817970 -0.000001
6 -0.783159 -0.495820 -0.000001
6 0.191334 -1.517670 -0.000001
1 -0.146421 -2.553480 -0.000001
7 1.496775 -1.325066 0.000000
7 1.058793 1.061707 0.000000
8 3.176267 0.224795 0.000001
8 -1.077568 1.872485 0.000000
6 -2.174962 -0.859225 0.000000
1 -2.350977 -1.951414 -0.000003
8 -3.156487 -0.094080 0.000002
1 -0.546782 2.689425 -0.000007
d_5fU/d_5fUga.xyz
6 -1.927024 -0.119944 0.000001
6 0.297708 -0.822570 -0.000002
6 0.715137 0.578135 -0.000002

6 -0.319892 1.555251 -0.000004
1 -0.049624 2.611206 0.000001
7 -1.590366 1.256306 0.000000
7 -1.003192 -1.132701 -0.000001
8 -3.154380 -0.398264 0.000003
8 1.209254 -1.735356 -0.000001
6 2.054045 0.910480 0.000001
1 2.399250 1.943131 0.000006
8 2.996597 -0.006132 0.000003
1 2.473679 -0.909664 0.000001
d_5fU/d_5fUf.xyz
6 1.882352 -0.290821 -0.002598
6 -0.017974 1.040895 0.004599
6 -0.870125 -0.100804 0.003962
6 -0.176323 -1.333876 0.018711
1 -0.762224 -2.250334 0.028247
7 1.133745 -1.467251 0.018401
7 1.295115 0.956546 0.001575
8 3.139971 -0.373714 -0.024115
8 -0.476220 2.321007 0.010229
6 -2.309129 -0.065258 -0.019652
1 -2.791087 0.927476 -0.058661
8 -3.045960 -1.069395 -0.007737
1 -1.443838 2.373795 0.033446
d_5fU/d_5fUg.xyz
6 -1.900281 -0.281098 0.000001
6 -0.041108 1.164506 -0.000001
6 0.831863 -0.029445 -0.000001
6 0.179924 -1.308968 -0.000004
1 0.791327 -2.209696 -0.000007
7 -1.109834 -1.467795 -0.000004
7 -1.381829 0.975822 0.000000
8 -3.148494 -0.453269 0.000005
8 0.451912 2.329541 -0.000002
6 2.192183 0.101172 0.000002
1 2.661323 1.081318 0.000004

8 3.006409 -0.955584 0.000003
1 3.934888 -0.670312 0.000004
d_5fU/d_5fUha.xyz
6 -1.959747 -0.034941 -0.005248
6 0.222194 -0.933164 0.010699
6 0.747272 0.449884 0.007882
6 -0.230190 1.503393 0.018847
1 0.121261 2.535642 0.030540
7 -1.516046 1.315713 0.015995
7 -1.120920 -1.104106 0.000590
8 -3.208762 -0.208754 -0.029624
8 1.001396 -1.927661 0.022430
6 2.062909 0.828586 -0.006639
1 2.320974 1.887083 -0.005917
8 3.086541 -0.020122 -0.025220
1 3.928504 0.465772 -0.034648
d_5fU/d_5fUh.xyz
6 -1.902806 -0.270885 0.000001
6 -0.031512 1.157409 -0.000001
6 0.837420 -0.044913 -0.000004
6 0.169608 -1.317565 -0.000003
1 0.754753 -2.236981 -0.000005
7 -1.121155 -1.463755 0.000001
7 -1.373360 0.980149 0.000004
8 -3.151795 -0.433536 -0.000001
8 0.469016 2.318252 -0.000002
6 2.197749 0.111810 0.000001
1 2.630869 1.106839 0.000004
8 3.140934 -0.830054 0.000003
1 2.787980 -1.737045 -0.000007
d_5hmC/d_5hmC.xyz
6 2.008389 -0.041850 0.080906
6 -0.136738 0.852374 -0.127158
6 -0.699717 -0.448118 -0.229503
6 0.218601 -1.484741 -0.133724
1 -0.142103 -2.512050 -0.189490

7 1.541987 -1.335268 0.014584
7 1.177897 1.049592 0.025475
8 3.268831 0.155855 0.211259
6 -2.162531 -0.689929 -0.440725
1 -2.539001 -0.142509 -1.311507
1 -2.338378 -1.759106 -0.612280
7 -0.934363 1.966229 -0.226033
1 -0.512743 2.841658 0.058033
1 -1.910134 1.859704 0.019357
8 -2.991118 -0.225205 0.659443
1 -2.646007 -0.623188 1.473309
d_5hmU/d_5hmU214.xyz
6 -2.008828 -0.102639 0.084280
6 0.244439 0.890904 -0.155810
6 0.749696 -0.445418 -0.225281
6 -0.164250 -1.476471 -0.115311
1 0.204375 -2.500691 -0.153422
7 -1.502621 -1.357939 0.029554
7 -1.130559 0.978180 -0.004278
8 -3.240186 0.144129 0.216016
8 0.938642 1.942931 -0.211285
1 -1.534576 1.908507 0.053097
6 2.222001 -0.657899 -0.405399
1 2.439971 -1.723620 -0.508417
1 2.578285 -0.146166 -1.309069
8 3.005125 -0.198155 0.726297
1 2.857212 0.758173 0.797790
d_5hmU/d_5hmU114.xyz
6 -1.980891 0.121448 0.066902
6 0.235918 0.889514 -0.141577
6 0.730857 -0.485024 -0.190810
6 -0.180609 -1.483722 -0.078122
1 0.078005 -2.537090 -0.089216
7 -1.509127 -1.189831 0.048166
7 -1.097528 1.138440 -0.019441
8 -3.228884 0.295096 0.174208

8 1.065622 1.855622 -0.203896
6 2.200182 -0.731847 -0.372202
1 2.418195 -1.802291 -0.337756
1 2.530538 -0.347476 -1.346994
8 2.991822 -0.114545 0.667759
1 2.751643 0.828961 0.643570
1 -2.193022 -1.933959 0.119610
d_5hmU/d_5hmU134.xyz
6 -1.864692 -0.036695 0.057211
6 0.247842 0.864805 -0.149111
6 0.750167 -0.487540 -0.188870
6 -0.164107 -1.507156 -0.072649
1 0.168341 -2.543096 -0.081404
7 -1.505500 -1.318797 0.049268
7 -1.104902 1.051840 -0.026333
8 -3.216000 0.164044 0.175085
8 1.033695 1.872555 -0.212740
6 2.224097 -0.715530 -0.358888
1 2.460789 -1.780880 -0.294033
1 2.555873 -0.353448 -1.342452
8 3.003991 -0.056750 0.667142
1 2.734870 0.878668 0.626815
1 -3.380395 1.121360 0.168496
d_5hmU/d_5hmU125.xyz
6 -1.861011 0.026323 0.054602
6 0.268459 0.872953 -0.144736
6 0.741780 -0.490699 -0.186658
6 -0.194109 -1.489969 -0.074634
1 0.111841 -2.533948 -0.085053
7 -1.530767 -1.265554 0.044325
7 -1.077811 1.095891 -0.022704
8 -3.196055 0.318091 0.166942
8 1.080003 1.860790 -0.206846
6 2.210436 -0.750088 -0.358216
1 2.425199 -1.819847 -0.289076
1 2.547200 -0.399513 -1.344360

8 3.005476 -0.102230 0.662812
1 2.750012 0.837307 0.621059
1 -3.682920 -0.520675 0.220669
d_5hmU/d_5hmU156.xyz
6 -1.998788 -0.063085 0.092892
6 0.122653 0.835157 -0.140745
6 0.720828 -0.433271 -0.249576
6 -0.188697 -1.483760 -0.158501
1 0.180886 -2.506331 -0.229523
7 -1.509653 -1.346292 0.002204
7 -1.171625 1.039648 0.026131
8 -3.251958 0.122848 0.246400
8 0.939805 1.926377 -0.205144
6 2.195637 -0.625492 -0.432172
1 2.404492 -1.669909 -0.676744
1 2.572430 -0.000859 -1.251310
8 2.968691 -0.345991 0.764560
1 2.858562 0.594185 0.973360
1 0.390475 2.726264 -0.132044
d_5hmU/d_5hmU246.xyz
6 -2.000880 -0.093569 0.096345
6 0.085596 0.889857 -0.128182
6 0.726683 -0.357818 -0.275032
6 -0.150435 -1.441704 -0.209882
1 0.255716 -2.447592 -0.314523
7 -1.470845 -1.358576 -0.035597
7 -1.215756 1.036325 0.046844
8 -3.257927 0.041552 0.269721
8 0.770771 2.076545 -0.155904
1 1.718642 1.931298 -0.295719
6 2.204890 -0.543667 -0.454539
1 2.400035 -1.538250 -0.862543
1 2.623546 0.187823 -1.159623
8 2.957356 -0.491515 0.787153
1 2.851553 0.391230 1.173664
d_5mC/d_5mC.xyz

6 -1.656853 0.030597 -0.000219
6 0.551777 0.779920 0.004760
6 1.044502 -0.550474 0.000551
6 0.054926 -1.521036 -0.005817
1 0.348277 -2.570925 -0.012001
7 -1.268421 -1.285137 -0.005044
7 -0.757929 1.065784 0.000951
8 -2.914461 0.308400 0.000344
7 1.422853 1.843764 0.060259
1 1.041672 2.748353 -0.186426
1 2.378337 1.687420 -0.231483
6 2.514296 -0.874228 0.004077
1 3.031780 -0.454785 -0.869596
1 3.020467 -0.478914 0.894693
1 2.667744 -1.957904 -0.011213
d_5mU/d_5mU4.xyz
6 1.651869 -0.088827 0.000000
6 -0.630493 0.872022 0.000000
6 -1.128668 -0.469621 0.000000
6 -0.189198 -1.482117 0.000000
1 -0.542472 -2.512434 -0.000002
7 1.160266 -1.346708 -0.000001
7 0.753541 0.976790 -0.000001
8 2.889940 0.178490 0.000002
8 -1.331825 1.922129 0.000000
1 1.147308 1.913146 -0.000003
6 -2.611905 -0.712730 0.000001
1 -3.094763 -0.271374 0.880788
1 -3.094764 -0.271373 -0.880785
1 -2.826510 -1.785849 0.000001
d_5mU/d_5mU1.xyz
6 1.623424 0.110955 -0.000001
6 -0.602186 0.897336 0.000002
6 -1.116099 -0.473717 0.000001
6 -0.198627 -1.473889 0.000004
1 -0.461313 -2.526141 0.000006

7 1.142731 -1.192199 0.000002
7 0.743592 1.132528 0.000001
8 2.880571 0.277444 -0.000006
8 -1.413868 1.877754 0.000004
1 1.822017 -1.943525 0.000019
6 -2.596134 -0.726139 -0.000006
1 -3.075557 -0.280974 0.879990
1 -3.075540 -0.281022 -0.880035
1 -2.809763 -1.799503 0.000019
d_5mU/d_5mU3.xyz
6 1.504140 -0.059622 0.000000
6 -0.603882 0.888689 0.000003
6 -1.141826 -0.453105 0.000004
6 -0.235631 -1.487387 0.000013
1 -0.587164 -2.517225 -0.000003
7 1.118797 -1.331102 0.000002
7 0.761538 1.042856 -0.000001
8 2.868033 0.117126 -0.000008
8 -1.357532 1.921265 0.000005
1 3.044619 1.072031 -0.000001
6 -2.628513 -0.668878 -0.000010
1 -3.099875 -0.212841 0.879549
1 -3.099855 -0.212874 -0.879597
1 -2.869812 -1.736677 0.000006
d_5mU/d_5mU2.xyz
6 1.500919 0.014716 0.000001
6 -0.634294 0.884670 0.000000
6 -1.128997 -0.474468 0.000001
6 -0.190181 -1.478211 0.000000
1 -0.504501 -2.519878 -0.000002
7 1.157996 -1.270863 -0.000001
7 0.723194 1.089774 0.000005
8 2.847030 0.296070 -0.000002
8 -1.425120 1.889135 -0.000003
1 3.326494 -0.548367 0.000005
6 -2.608054 -0.737053 0.000001

1 -3.093190 -0.295868 0.879611
1 -3.093191 -0.295869 -0.879610
1 -2.815581 -1.811961 0.000002
d_5mU/d_5mU5.xyz
6 1.648766 0.002076 0.000000
6 -0.534425 0.773749 0.000000
6 -1.074486 -0.523574 0.000000
6 -0.099855 -1.516057 -0.000001
1 -0.413475 -2.559518 -0.000002
7 1.224542 -1.303005 -0.000002
7 0.759552 1.054529 -0.000001
8 2.903897 0.258502 0.000002
8 -1.412242 1.822734 -0.000001
1 -0.899772 2.649399 -0.000002
6 -2.552797 -0.799022 0.000001
1 -3.046742 -0.372841 0.881595
1 -3.046744 -0.372840 -0.881590
1 -2.738394 -1.877792 0.000001
d_5mU/d_5mU6.xyz
6 1.658391 0.022821 0.000000
6 -0.527361 0.784631 0.000002
6 -1.057909 -0.519367 0.000000
6 -0.076534 -1.506726 0.000003
1 -0.385531 -2.551605 0.000004
7 1.244582 -1.286781 0.000004
7 0.764977 1.068837 0.000004
8 2.912798 0.284893 -0.000007
8 -1.339869 1.891868 0.000003
1 -2.274111 1.630785 -0.000016
6 -2.532774 -0.821461 -0.000004
1 -3.038378 -0.411862 0.884286
1 -3.038371 -0.411872 -0.884302
1 -2.696838 -1.903311 0.000002
d_C/d_C56.xyz
6 1.211589 -0.403268 0.000417
6 -1.114249 -0.243910 -0.006677

6 -1.036966 1.166944 -0.001883
6 0.242449 1.695854 0.003701
1 0.378136 2.776424 0.009610
7 1.368477 0.964010 0.003857
7 -0.018472 -1.014793 -0.002757
8 2.260894 -1.143069 0.002606
7 -2.331890 -0.880029 -0.062013
1 -2.341795 -1.855669 0.207220
1 -3.144503 -0.346175 0.217519
1 -1.922733 1.791933 -0.002163
d_C/d_C46.xyz
6 -1.244764 -0.300853 0.000001
6 1.237547 -0.325931 0.000008
6 1.148775 1.109377 0.000000
6 -0.091712 1.696194 0.000001
1 -0.157789 2.782694 -0.000005
7 -1.291895 1.050469 0.000000
7 -0.012100 -0.945153 0.000006
8 -2.283722 -1.029041 -0.000006
7 2.300405 -1.087240 -0.000005
1 -0.032040 -1.959425 0.000009
1 3.128325 -0.490926 -0.000021
1 2.057335 1.700734 -0.000007
d_C/d_C45.xyz
6 -1.230072 -0.322256 0.000001
6 1.259186 -0.272643 -0.000001
6 1.122400 1.157722 -0.000004
6 -0.134269 1.706326 -0.000002
1 -0.233773 2.790061 0.000000
7 -1.316329 1.024599 0.000006
7 0.024632 -0.930851 -0.000008
8 -2.244489 -1.084065 0.000003
7 2.405636 -0.900952 0.000008
1 0.022479 -1.945820 -0.000030
1 2.254100 -1.912213 0.000010
1 2.012065 1.776030 -0.000004

d_C/d_C15.xyz
6 -1.129247 -0.478435 0.000001
6 1.237273 -0.319263 0.000003
6 1.135195 1.135869 0.000013
6 -0.090977 1.705725 0.000000
1 -0.259951 2.776524 0.000003
7 -1.215207 0.916652 -0.000014
7 0.071594 -1.070827 0.000012
8 -2.227392 -1.122007 0.000004
7 2.425728 -0.880743 -0.000014
1 -2.137819 1.332287 -0.000030
1 2.296953 -1.895665 -0.000017
1 2.031679 1.743960 0.000026
d_C/d_C16.xyz
6 -1.153513 -0.456248 0.000001
6 1.213679 -0.385793 0.000002
6 1.161821 1.075652 0.000014
6 -0.042734 1.690470 0.000001
1 -0.171986 2.766943 0.000003
7 -1.192390 0.942432 -0.000013
7 0.022824 -1.091612 0.000014
8 -2.275633 -1.058265 0.000003
7 2.341665 -1.063509 -0.000015
1 -2.100080 1.389649 -0.000036
1 3.118568 -0.401277 -0.000018
1 2.078352 1.655144 0.000030
d_C/d_C35.xyz
6 -1.089346 -0.238701 -0.000001
6 1.231953 -0.362952 0.000004
6 1.212054 1.082772 0.000008
6 0.006349 1.727200 -0.000002
1 -0.036791 2.813766 0.000000
7 -1.206364 1.089761 -0.000007
7 -0.005844 -1.000545 0.000005
8 -2.298690 -0.899271 -0.000001
7 2.366398 -1.034919 -0.000007

1 -2.112959 -1.852499 0.000024
1 2.149103 -2.033953 -0.000013
1 2.144778 1.636867 0.000016
d_C/d_C25.xyz
6 -1.065897 -0.298593 0.000000
6 1.252205 -0.340380 -0.000008
6 1.186710 1.104819 -0.000010
6 -0.038359 1.710398 0.000002
1 -0.120595 2.794631 0.000006
7 -1.226304 1.027842 0.000010
7 0.039159 -1.023680 -0.000013
8 -2.224999 -1.045077 -0.000006
7 2.409729 -0.972090 0.000014
1 -2.973355 -0.425910 0.000047
1 2.226193 -1.977806 0.000025
1 2.101701 1.687727 -0.000018
d_C/d_C36.xyz
6 -1.105115 -0.225028 -0.000003
6 1.208192 -0.417881 0.000000
6 1.227522 1.030733 0.000003
6 0.040750 1.710562 0.000000
1 0.029037 2.798175 0.000001
7 -1.187734 1.107631 -0.000001
7 -0.045719 -1.016887 -0.000003
8 -2.332656 -0.851706 -0.000004
7 2.275562 -1.194434 -0.000002
1 -2.171782 -1.809523 0.000052
1 3.105539 -0.599606 0.000000
1 2.175587 1.560112 0.000008
d_C/d_C26.xyz
6 -1.083010 -0.285262 -0.000001
6 1.229688 -0.397086 0.000004
6 1.204361 1.052152 -0.000008
6 -0.002738 1.694126 0.000003
1 -0.052063 2.780597 -0.000003
7 -1.208352 1.046631 0.000006

7 -0.001282 -1.041123 -0.000006
8 -2.263400 -0.998187 -0.000006
7 2.323283 -1.136760 0.000007
1 -2.993481 -0.357526 0.000039
1 3.132139 -0.513576 0.000002
1 2.135261 1.611187 -0.000022
d_U/d_U4.xyz
6 -1.234468 -0.292633 0.000000
6 1.244912 -0.327976 -0.000004
6 1.180733 1.095063 -0.000010
6 -0.062458 1.694911 0.000006
1 -0.117880 2.781801 0.000003
7 -1.258342 1.062691 0.000007
1 -0.018187 -1.960364 0.000009
7 -0.001008 -0.944804 -0.000010
8 -2.278222 -1.004056 -0.000001
8 2.288500 -1.037808 0.000010
1 2.096976 1.672080 -0.000021
d_U/d_U1.xyz
6 -1.143610 -0.447531 -0.000017
6 1.215618 -0.383038 0.000023
6 1.183934 1.070862 0.000026
6 -0.021417 1.691715 0.000002
1 -0.149160 2.768171 0.000000
7 -1.167644 0.947710 -0.000022
1 -2.073896 1.400625 -0.000040
7 0.043640 -1.087439 0.000001
8 -2.256442 -1.049842 -0.000045
8 2.328300 -1.002164 0.000036
1 2.109073 1.633308 0.000045
d_U/d_U3.xyz
6 1.094077 -0.213589 0.000002
6 -1.208798 -0.422374 -0.000001
6 -1.258433 1.013710 -0.000007
6 -0.075025 1.709675 0.000003
1 -0.073205 2.796932 -0.000001

7 1.150920 1.117864 0.000005
1 2.180119 -1.784111 -0.000015
7 0.033978 -1.015630 0.000000
8 2.323020 -0.823262 -0.000002
8 -2.259920 -1.148431 0.000004
1 -2.216930 1.520554 -0.000019
d_U/d_U2.xyz
6 1.072639 -0.275093 -0.000001
6 -1.230783 -0.398105 0.000001
6 -1.232082 1.039785 0.000012
6 -0.026398 1.694933 0.000011
1 0.015801 2.781283 -0.000022
7 1.175652 1.055566 -0.000019
1 2.982379 -0.339861 -0.000017
7 -0.012715 -1.038257 0.000009
8 2.250509 -0.979146 0.000007
8 -2.308824 -1.084751 -0.000012
1 -2.172476 1.579470 0.000012
d_U/d_U5.xyz
6 1.202219 -0.391515 0.000001
6 -1.108370 -0.243168 0.000011
6 -1.075158 1.156665 0.000005
6 0.204099 1.699473 -0.000001
1 0.329066 2.780729 -0.000007
7 1.333334 0.978834 -0.000002
1 -2.185872 -1.819949 0.000061
7 -0.030906 -1.011829 0.000002
8 2.251467 -1.119673 -0.000003
8 -2.329300 -0.857565 -0.000017
1 -1.974259 1.759363 0.000007
d_U/d_U6.xyz
6 1.220036 -0.373536 0.000000
6 -1.091370 -0.290719 0.000001
6 -1.094178 1.111426 0.000006
6 0.169963 1.690171 -0.000013
1 0.263378 2.774660 0.000014

7 1.317223 1.001159 0.000000
1 -3.017238 -0.412221 0.000009
7 0.006762 -1.027354 -0.000004
8 2.289367 -1.073123 0.000004
8 -2.256024 -1.014096 -0.000003
1 -2.007486 1.694626 0.000021

5 XYZ data (1 explicit H_2O)

1m5caC/1m5caCacv.xyz
6 -1.616737 0.821989 0.001689
6 0.108393 -0.904140 0.010492
6 1.128499 0.101405 0.000583
6 0.703473 1.403859 -0.001481
1 1.423430 2.212652 -0.007853
7 -0.603639 1.772827 0.005007
7 -1.191491 -0.489126 0.008770
8 -2.810338 1.122649 -0.005731
7 0.357144 -2.200326 0.020898
1 -0.399918 -2.877627 0.028402
1 1.345884 -2.460329 0.020244
6 2.608075 -0.217465 -0.005310
8 2.935404 -1.447817 0.004742
8 3.414475 0.755313 -0.019568
1 -1.926650 -1.216209 0.007837
6 -1.006321 3.189484 0.003787
1 -1.547807 3.418865 -0.915666
1 -1.648658 3.386235 0.863134
1 -0.108207 3.801035 0.067010
8 -2.583944 -2.925875 -0.010161
1 -3.066558 -3.125523 -0.826484
1 -3.202687 -3.127667 0.707826
1m5caC/1m5caCade.xyz
6 1.579621 0.814674 -0.006576
6 -0.125042 -0.928023 -0.003372
6 -1.156459 0.066225 -0.001318
6 -0.745719 1.373308 0.001384

1 -1.473948 2.174625 0.003576
7 0.556976 1.755560 0.002340
7 1.169879 -0.501996 -0.007215
8 2.769681 1.129163 -0.013085
7 -0.368958 -2.226146 -0.002053
1 0.380724 -2.908045 -0.001726
1 -1.357712 -2.489560 0.000475
6 -2.632781 -0.267706 0.000437
8 -2.947815 -1.501562 0.002651
8 -3.449262 0.696480 -0.000188
1 1.926220 -1.215766 -0.010236
6 0.945372 3.176318 0.009238
1 1.580154 3.376381 0.873479
1 1.490662 3.414090 -0.905784
1 0.040724 3.778681 0.068484
8 3.016191 -2.622773 -0.002721
1 2.871259 -3.103893 0.826586
1 2.736324 -3.237680 -0.698360
1m5caC/1m5caCaap.xyz
6 -1.565244 0.806734 0.003501
6 0.136138 -0.938637 0.001376
6 1.169171 0.054175 0.000869
6 0.760541 1.361875 -0.002612
1 1.489967 2.162095 -0.003567
7 -0.541615 1.746249 -0.006140
7 -1.157773 -0.510407 0.005713
8 -2.755275 1.122356 0.009578
7 0.379000 -2.237000 -0.002896
1 -0.369067 -2.920291 0.001573
1 1.367775 -2.500793 -0.002137
6 2.645090 -0.281826 0.003140
8 2.958422 -1.516097 -0.000005
8 3.462909 0.681204 0.008022
1 -1.918881 -1.219902 0.017245
6 -0.927868 3.167591 -0.015492
1 -1.558148 3.368377 -0.882875

1 -1.477131 3.406461 0.896846
1 -0.022057 3.768617 -0.070559
8 -3.009321 -2.616031 0.081073
1 -3.932660 -2.321469 0.068920
1 -2.897929 -3.125920 -0.736214
1m5caC/1m5caCacc.xyz
6 1.564935 0.808739 -0.008107
6 -0.134377 -0.938705 -0.008788
6 -1.168700 0.052655 -0.002339
6 -0.761661 1.360861 0.002536
1 -1.492034 2.160193 0.007557
7 0.539995 1.746961 0.002026
7 1.159264 -0.508842 -0.011786
8 2.754481 1.126290 -0.013529
7 -0.373964 -2.237609 -0.012226
1 0.378627 -2.916543 -0.017180
1 -1.361587 -2.504804 -0.009822
6 -2.644126 -0.285330 0.001950
8 -2.955749 -1.519970 -0.000640
8 -3.463270 0.676659 0.008057
1 1.920229 -1.218856 -0.012908
6 0.924280 3.168854 0.010640
1 1.557672 3.370104 0.875641
1 1.469710 3.408949 -0.903702
1 0.017827 3.768572 0.069468
8 2.969722 -2.635387 0.005540
1 3.659640 -2.573478 -0.672744
1 3.449273 -2.680892 0.846819
1m5caC/1m5caCadf.xyz
6 1.565775 0.807196 -0.008609
6 -0.135876 -0.938022 -0.008473
6 -1.168656 0.055062 -0.000774
6 -0.759835 1.362691 0.004152
1 -1.489172 2.162967 0.009307
7 0.542359 1.746869 0.003814
7 1.158029 -0.509965 -0.013801

8 2.755869 1.122402 -0.014806
7 -0.379828 -2.236190 -0.010057
1 0.366546 -2.920970 -0.018467
1 -1.369070 -2.498654 -0.008408
6 -2.644679 -0.280598 0.002150
8 -2.958352 -1.514818 0.000462
8 -3.462255 0.682599 0.006137
1 1.919591 -1.218662 -0.024342
6 0.928861 3.168151 0.013998
1 1.562188 3.367535 0.879472
1 1.475039 3.408223 -0.899888
1 0.023310 3.769194 0.073021
8 3.036146 -2.601770 -0.079677
1 3.888775 -2.342421 0.301845
1 2.694085 -3.286386 0.516188
1m5caC/1m5caCabk.xyz
6 -2.538744 0.159986 0.085960
6 -0.405334 1.358554 -0.008086
6 0.292354 0.111678 -0.100949
6 -0.471861 -1.026073 -0.102104
1 0.000705 -1.997751 -0.172662
7 -1.826744 -1.027589 -0.014399
7 -1.765826 1.308108 0.086885
8 -3.763199 0.203392 0.169849
7 0.195531 2.531517 -0.009730
1 -0.321848 3.399959 0.067138
1 1.216692 2.505308 -0.072929
6 1.798196 0.019146 -0.170266
8 2.454511 1.104915 -0.181435
8 2.305342 -1.142575 -0.204204
1 -2.293728 2.176116 0.160234
6 -2.609397 -2.277606 -0.021446
1 -3.222708 -2.324743 0.879328
1 -3.249653 -2.302455 -0.904833
1 -1.917204 -3.117427 -0.040931
8 4.994642 -1.034892 0.345471

1 4.031209 -1.050629 0.120200
1 5.214165 -0.093457 0.389065
1m5caC/1m5caCabd.xyz
6 -2.523266 -0.416642 0.045124
6 -0.221918 -1.251175 -0.031621
6 0.266207 0.094609 -0.056577
6 -0.672462 1.093170 -0.035543
1 -0.363642 2.130756 -0.053217
7 -2.010803 0.872629 0.009608
7 -1.575431 -1.424502 0.016290
8 -3.726309 -0.658440 0.097068
7 0.559592 -2.312460 -0.053288
1 0.184656 -3.253923 -0.033855
1 1.564766 -2.125253 -0.077575
6 1.735610 0.444865 -0.094250
8 2.560646 -0.531191 -0.086639
8 2.050157 1.662657 -0.127541
1 -1.958460 -2.368392 0.035610
6 -2.979577 1.983313 0.035071
1 -3.658434 1.892181 -0.813862
1 -3.548420 1.948626 0.965683
1 -2.427462 2.919037 -0.028276
8 5.164694 0.272043 0.206687
1 5.157422 1.239573 0.215885
1 4.215003 0.018350 0.091521
1m5caC/1m5caCaba.xyz
6 -2.291558 -0.690334 -0.132641
6 0.081900 -1.065221 0.341648
6 0.315056 0.346857 0.295832
6 -0.768613 1.143951 0.032364
1 -0.651144 2.219467 -0.017447
7 -2.023450 0.670673 -0.177063
7 -1.197371 -1.494282 0.133790
8 -3.411851 -1.159301 -0.315137
7 1.026266 -1.956412 0.568819
1 0.829269 -2.950472 0.586213

1 1.965975 -1.580346 0.717783
6 1.676369 0.968138 0.507378
8 2.643653 0.168607 0.753510
8 1.771931 2.219870 0.424362
1 -1.401109 -2.492029 0.162405
6 -3.159557 1.568634 -0.453458
1 -3.595174 1.315051 -1.420921
1 -3.911866 1.456352 0.328906
1 -2.792272 2.592794 -0.469279
8 4.144162 -0.536271 -1.483330
1 3.645984 -0.247138 -0.687904
1 4.967477 -0.028914 -1.460562
1m5caC/1m5caCaag.xyz
6 2.293985 0.684043 0.139966
6 -0.077429 1.073430 -0.332870
6 -0.316830 -0.337937 -0.299169
6 0.763269 -1.142137 -0.042681
1 0.641041 -2.217523 -0.002933
7 2.020141 -0.676247 0.170957
7 1.203422 1.495236 -0.119556
8 3.416128 1.146537 0.327369
7 -1.018039 1.970275 -0.552935
1 -0.816770 2.963579 -0.563919
1 -1.958523 1.599037 -0.709283
6 -1.680772 -0.951153 -0.517749
8 -2.642534 -0.146389 -0.767335
8 -1.783590 -2.202511 -0.435735
1 1.411558 2.492292 -0.139588
6 3.152641 -1.581557 0.437670
1 3.906855 -1.460811 -0.341579
1 3.586889 -1.342376 1.409468
1 2.782083 -2.604680 0.438470
8 -4.231543 0.521630 1.425536
1 -4.197176 -0.222605 2.042807
1 -3.671499 0.245976 0.667598
1m5caC/1m5caCaax.xyz

6 1.317561 0.592961 0.056049
6 -1.060549 1.169219 0.012503
6 -1.398351 -0.221721 -0.000270
6 -0.355372 -1.109124 0.018902
1 -0.546922 -2.174852 0.011745
7 0.953784 -0.742419 0.046389
7 0.267080 1.489176 0.040895
8 2.487171 0.977985 0.078690
7 -1.952296 2.139905 0.002281
1 -1.679878 3.116353 0.010096
1 -2.930728 1.840521 -0.034587
6 -2.827359 -0.725873 -0.033586
8 -3.745848 0.151306 -0.115425
8 -3.002364 -1.974410 0.020799
1 0.542270 2.469994 0.049109
6 2.037083 -1.742874 0.058448
1 2.630126 -1.650070 -0.852456
1 2.670343 -1.580563 0.931089
1 1.585427 -2.731580 0.108509
8 5.102599 -0.159762 -0.169966
1 4.185136 0.163151 -0.087396
1 5.653723 0.603937 0.051878
1m5caC/1m5caCaao.xyz
6 2.166960 0.723791 0.023939
6 -0.217769 0.975538 -0.481480
6 -0.405735 -0.432265 -0.299472
6 0.703676 -1.165090 0.034611
1 0.622300 -2.234241 0.187136
7 1.941829 -0.634306 0.198572
7 1.047436 1.461888 -0.316535
8 3.271437 1.243452 0.159848
7 -1.184448 1.811247 -0.807600
1 -1.019027 2.805229 -0.916249
1 -2.117765 1.405237 -0.892810
6 -1.742035 -1.123309 -0.455540
8 -2.762100 -0.377137 -0.648908

8 -1.762660 -2.378911 -0.377448
1 1.219914 2.457869 -0.443158
6 3.103443 -1.463697 0.567481
1 3.855741 -1.408738 -0.220986
1 3.528499 -1.100777 1.504211
1 2.766946 -2.491678 0.686423
8 -3.408576 0.905000 1.787118
1 -2.608076 1.430474 1.928780
1 -3.238284 0.435784 0.943483
1m5caC/1m5caCaaz.xyz
6 -2.215359 0.748310 0.069771
6 0.173167 0.992209 -0.419516
6 0.346751 -0.422195 -0.281564
6 -0.770703 -1.154509 0.025439
1 -0.699555 -2.228531 0.144988
7 -2.004402 -0.616999 0.200062
7 -1.087174 1.486386 -0.241592
8 -3.315420 1.273841 0.218371
7 1.152658 1.827319 -0.706522
1 0.998230 2.825521 -0.789248
1 2.077393 1.408126 -0.823130
6 1.677073 -1.119723 -0.453071
8 2.690502 -0.382698 -0.706427
8 1.700067 -2.372106 -0.334077
1 -1.249284 2.487694 -0.335582
6 -3.176663 -1.448449 0.528587
1 -3.613763 -1.108447 1.468271
1 -3.916564 -1.368144 -0.269475
1 -2.847781 -2.481219 0.625293
8 3.894277 0.746405 1.561370
1 3.385024 1.555732 1.709129
1 3.477730 0.334942 0.774348
1m5caC/1m5caCaas.xyz
6 -1.395044 1.292590 0.000092
6 -0.153101 -0.821917 -0.002535
6 1.081653 -0.094186 0.000179

6 0.994168 1.272468 0.001190
1 1.895726 1.872511 0.002472
7 -0.178659 1.959223 0.000276
7 -1.307797 -0.086227 -0.000742
8 -2.478629 1.874258 0.000515
7 -0.238274 -2.135095 -0.006703
1 -1.151030 -2.605971 -0.008821
1 0.656080 -2.629676 -0.007095
6 2.439873 -0.765563 0.001023
8 2.458757 -2.038383 -0.002308
8 3.456997 -0.016515 0.004834
1 -2.204238 -0.570474 -0.000513
6 -0.220356 3.433206 -0.000538
1 -0.744545 3.780709 -0.892244
1 -0.743375 3.781966 0.891348
1 0.801746 3.806895 -0.001614
8 -2.960703 -3.091511 -0.004078
1 -3.343243 -2.717769 0.804495
1 -3.388540 -2.605876 -0.726004
1m5caC/1m5caCaai.xyz
6 -0.951858 1.670614 -0.023019
6 0.813611 -0.031024 -0.039096
6 -0.181655 -1.062984 -0.008879
6 -1.489719 -0.658658 0.011543
1 -2.286909 -1.390981 0.035159
7 -1.882530 0.642755 0.007143
7 0.368405 1.263028 -0.047474
8 -1.266297 2.859374 -0.029838
7 2.109932 -0.253146 -0.059971
1 2.784662 0.523202 -0.055261
1 2.380935 -1.239111 -0.048949
6 0.158546 -2.538444 0.007212
8 1.393038 -2.847979 -0.008267
8 -0.802729 -3.358100 0.035244
1 1.047890 2.020079 -0.077651
6 -3.305845 1.022198 0.044059

1 -3.489330 1.662998 0.907523
1 -3.567969 1.558045 -0.869924
1 -3.900240 0.114054 0.125159
8 3.778998 2.091663 -0.022065
1 3.252422 2.817550 0.346041
1 4.525333 1.995833 0.588499
1m5caC/1m5caCabj.xyz
6 -2.219960 -0.203960 0.117415
6 -0.162082 -1.531273 0.176768
6 0.591540 -0.382688 -0.225840
6 -0.112970 0.772499 -0.446803
1 0.397299 1.671536 -0.767676
7 -1.457107 0.880571 -0.292740
7 -1.508508 -1.370597 0.337144
8 -3.436766 -0.146678 0.274043
7 0.375202 -2.714177 0.402336
1 -0.184687 -3.513512 0.676210
1 1.382114 -2.784550 0.236704
6 2.091568 -0.406679 -0.408682
8 2.671850 -1.532412 -0.343086
8 2.670182 0.702777 -0.616448
1 -2.076229 -2.165593 0.626274
6 -2.167812 2.150331 -0.532296
1 -2.890424 2.018310 -1.339104
1 -2.686711 2.452649 0.378497
1 -1.434770 2.905591 -0.808610
8 2.123841 2.785666 1.139266
1 2.940062 2.931052 1.637736
1 2.331683 2.049740 0.519215
1m5caC/1m5caCabu.xyz
6 2.219061 0.209978 0.118683
6 0.156549 1.530007 0.176559
6 -0.592516 0.379384 -0.228507
6 0.116198 -0.773152 -0.449633
1 -0.390694 -1.673375 -0.772287
7 1.460484 -0.876757 -0.293851

7 1.503303 1.373902 0.338986
8 3.435847 0.156673 0.276836
7 -0.385337 2.710680 0.402645
1 0.171284 3.511667 0.678386
1 -1.392235 2.777659 0.235511
6 -2.092400 0.398217 -0.412609
8 -2.676669 1.521912 -0.347320
8 -2.667146 -0.713210 -0.620708
1 2.067716 2.170618 0.629817
6 2.175740 -2.143896 -0.534154
1 2.898058 -2.008946 -1.340733
1 2.695399 -2.445118 0.376555
1 1.445427 -2.901573 -0.811162
8 -2.126081 -2.796988 1.134355
1 -2.326015 -2.056875 0.516796
1 -2.841485 -2.779165 1.785324
1m5caC/1m5caCada.xyz
6 1.919640 -0.615705 -0.237378
6 -0.450127 -1.233731 -0.209707
6 -0.811698 0.140850 -0.380493
6 0.215748 1.043631 -0.464873
1 0.004376 2.099395 -0.581127
7 1.529218 0.703484 -0.412045
7 0.882305 -1.529087 -0.170297
8 3.095561 -0.962111 -0.154500
7 -1.328127 -2.210093 -0.091785
1 -1.042875 -3.175149 0.029700
1 -2.311125 -1.924634 -0.125172
6 -2.247975 0.619959 -0.421163
8 -3.156275 -0.265480 -0.313166
8 -2.441397 1.859726 -0.559058
1 1.174035 -2.496683 -0.043027
6 2.598403 1.715041 -0.483331
1 3.159918 1.719126 0.452492
1 3.267760 1.478602 -1.311602
1 2.137376 2.687442 -0.645470

8 -0.119872 0.600442 2.950393
1 -0.758675 0.976957 2.326912
1 0.417351 0.003938 2.408502
1m5caC/1m5caCabp.xyz
6 1.414050 0.487506 -0.564926
6 -0.892501 1.168799 -0.117899
6 -1.277908 -0.206028 -0.010549
6 -0.292578 -1.139172 -0.193061
1 -0.523409 -2.194957 -0.127943
7 1.003244 -0.829912 -0.464049
7 0.421586 1.429562 -0.385927
8 2.581462 0.811009 -0.791967
7 -1.732052 2.174196 0.027638
1 -1.432064 3.139889 -0.042350
1 -2.702236 1.911529 0.225440
6 -2.696387 -0.648898 0.289366
8 -3.572221 0.265108 0.421957
8 -2.905409 -1.889610 0.386254
1 0.734688 2.396802 -0.453849
6 2.028516 -1.872238 -0.657870
1 2.473097 -1.763390 -1.648145
1 2.802181 -1.771624 0.104697
1 1.548594 -2.845202 -0.571309
8 4.156264 0.213620 1.619672
1 3.781887 -0.624551 1.926984
1 3.687898 0.393753 0.785145
1m5caC/1m5caCadm.xyz
6 2.200471 -0.272833 0.106689
6 0.100324 -1.530970 0.174205
6 -0.617042 -0.358946 -0.227696
6 0.124632 0.771458 -0.453435
1 -0.358783 1.685800 -0.773116
7 1.472084 0.835614 -0.303857
7 1.451967 -1.415360 0.330763
8 3.418790 -0.254401 0.260432
7 -0.476151 -2.694040 0.403582

1 0.057193 -3.511018 0.678658
1 -1.485323 -2.730423 0.240169
6 -2.118520 -0.333866 -0.401766
8 -2.733436 -1.441404 -0.340942
8 -2.663134 0.794665 -0.597415
1 1.992904 -2.228666 0.619949
6 2.221955 2.082695 -0.544948
1 2.752003 2.368534 0.364781
1 2.937754 1.929803 -1.353974
1 1.512427 2.861359 -0.818672
8 -2.097080 2.832156 1.208506
1 -2.281098 2.114436 0.560582
1 -1.134413 2.833333 1.305257
1m5caC/1m5caCadi.xyz
6 -1.313970 0.638803 -0.079427
6 1.074912 1.166950 -0.014000
6 1.384891 -0.230619 -0.000285
6 0.324698 -1.096617 -0.031323
1 0.495135 -2.165846 -0.024577
7 -0.976670 -0.704015 -0.072259
7 -0.245565 1.513784 -0.056144
8 -2.475144 1.048218 -0.107530
7 1.987036 2.118056 0.008420
1 1.736404 3.100281 0.000960
1 2.958363 1.796833 0.054548
6 2.803421 -0.762974 0.046771
8 3.738011 0.095961 0.139635
8 2.955069 -2.014628 -0.007689
1 -0.501654 2.499759 -0.063829
6 -2.078023 -1.684766 -0.093751
1 -2.659236 -1.600837 0.825678
1 -2.717024 -1.492955 -0.956163
1 -1.645615 -2.680548 -0.169234
8 -5.093150 -0.051752 0.208096
1 -4.173481 0.257635 0.101364
1 -5.020372 -1.016147 0.243117

1m5caU/1m5caUaal.xyz
6 1.577771 0.687300 0.002030
6 -0.214912 -0.991711 0.008997
6 -1.178435 0.087289 0.001436
6 -0.715913 1.379185 0.000765
1 -1.405566 2.215616 -0.005242
7 0.599634 1.687910 0.007182
7 1.107067 -0.604773 0.011265
8 2.778728 0.954397 -0.009106
8 -0.511745 -2.208178 0.012823
1 1.832992 -1.346822 0.018205
6 -2.632564 -0.169319 -0.004691
8 -3.481042 0.720451 -0.011600
8 -3.012152 -1.458894 -0.002923
1 -2.196867 -2.035869 0.002924
6 1.066757 3.084886 -0.003786
1 1.733310 3.251534 0.843135
1 1.601050 3.286950 -0.933663
1 0.199090 3.736962 0.074724
8 3.200823 -2.503981 0.088209
1 3.098545 -3.192119 -0.586746
1 3.977423 -1.994344 -0.190194
1m5caU/1m5caUacr.xyz
6 1.607502 0.735608 -0.005279
6 -0.170347 -0.959134 -0.008301
6 -1.144215 0.110258 -0.004785
6 -0.693588 1.406270 -0.003403
1 -1.390679 2.236453 0.003081
7 0.618953 1.726685 -0.009081
7 1.148989 -0.560840 -0.013061
8 2.805004 1.016834 0.003537
8 -0.455692 -2.178345 -0.006887
1 1.869764 -1.308144 -0.009959
6 -2.595828 -0.160191 0.001151
8 -3.452816 0.721371 0.004576
8 -2.962840 -1.453430 0.003085

1 -2.141689 -2.022045 0.000199
6 1.072404 3.127968 0.010797
1 1.609350 3.327208 0.939744
1 1.732888 3.308683 -0.837956
1 0.197894 3.771987 -0.057520
8 3.024390 -2.682046 0.042382
1 2.598903 -3.325002 0.630339
1 3.028047 -3.109802 -0.827555
1m5caU/1m5caUaaz.xyz
6 -1.489656 -0.440004 -0.020966
6 0.436535 1.100173 -0.014405
6 1.305708 -0.054128 -0.003394
6 0.740395 -1.303799 0.000282
1 1.359840 -2.193064 0.012117
7 -0.596908 -1.509411 -0.010389
7 -0.916151 0.810544 -0.024287
8 -2.710056 -0.611039 -0.026706
8 0.820413 2.287594 -0.015249
1 -1.562104 1.598330 -0.032055
6 2.777729 0.080015 0.009452
8 3.544087 -0.879836 0.017386
8 3.266720 1.331650 0.013049
1 2.510014 1.980017 0.004461
6 -1.175975 -2.866059 0.018185
1 -1.775695 -2.983089 0.922003
1 -1.805336 -3.010683 -0.860727
1 -0.359864 -3.586129 0.015252
8 -4.299070 1.760362 -0.061948
1 -3.806166 0.916964 -0.025021
1 -4.514460 1.962691 0.859536
1m5caU/1m5caUacz.xyz
6 -2.552580 0.241238 0.080296
6 -0.361131 1.372325 -0.016385
6 0.261251 0.070301 -0.099153
6 -0.538447 -1.045466 -0.095396
1 -0.105060 -2.037185 -0.155291

7 -1.885544 -0.984865 -0.014147
7 -1.739319 1.354908 0.070428
8 -3.774737 0.316282 0.166630
8 0.252957 2.460135 -0.014774
1 -2.215461 2.252267 0.135723
6 1.725943 -0.077395 -0.158380
8 2.295604 -1.169696 -0.207649
8 2.447990 1.050237 -0.149863
1 1.829451 1.832637 -0.103609
6 -2.722574 -2.198265 0.000101
1 -3.290730 -2.234296 0.930755
1 -3.408794 -2.176270 -0.847576
1 -2.072224 -3.068005 -0.070936
8 5.078723 -0.946137 0.328739
1 4.126268 -1.031829 0.116484
1 5.231520 0.009391 0.349319
1m5caU/1m5caUabj.xyz
6 -1.591307 1.129791 -0.011859
6 0.755173 0.365822 -0.017502
6 0.273412 -0.994508 -0.001986
6 -1.082048 -1.210601 0.008589
1 -1.480731 -2.218237 0.019399
7 -1.989479 -0.209503 0.007305
7 -0.226478 1.335488 -0.022433
8 -2.396553 2.056075 -0.019364
8 1.963322 0.694058 -0.027278
1 0.072786 2.308895 -0.039112
6 1.192893 -2.151195 0.002792
8 0.809679 -3.317931 0.017269
8 2.508357 -1.875912 -0.009446
1 2.638572 -0.889643 -0.020412
6 -3.439368 -0.478565 0.019157
1 -3.890132 0.002426 0.888050
1 -3.890457 -0.085945 -0.893387
1 -3.586980 -1.555341 0.073713
8 2.699410 3.463997 0.028766

1 2.470780 2.513956 0.011950
1 1.851616 3.915239 0.150970
1m5caU/1m5caUabd.xyz
6 2.463873 0.556082 0.165689
6 0.105043 1.214839 -0.147384
6 -0.238031 -0.188275 -0.199893
6 0.764411 -1.116863 -0.072624
1 0.545744 -2.177661 -0.110473
7 2.061551 -0.781554 0.100882
7 1.448378 1.480404 0.035925
8 3.637699 0.879806 0.322500
8 -0.710615 2.154998 -0.255619
1 1.730652 2.457673 0.074635
6 -1.628706 -0.638225 -0.400336
8 -1.968348 -1.816773 -0.434639
8 -2.550757 0.330074 -0.557086
1 -2.109761 1.223591 -0.483076
6 3.118377 -1.803673 0.212770
1 3.832885 -1.676587 -0.601866
1 3.630508 -1.697162 1.169849
1 2.652763 -2.785237 0.147216
8 -5.055996 -0.137201 0.849107
1 -4.970449 0.307809 1.704057
1 -4.207500 0.025084 0.398560
1m5caU/1m5caUabh.xyz
6 2.561366 0.174908 0.088921
6 0.415969 1.393309 0.002428
6 -0.256435 0.118327 -0.103829
6 0.498850 -1.027957 -0.108158
1 0.026490 -2.000399 -0.184887
7 1.846714 -1.023039 -0.014911
7 1.791971 1.319624 0.093675
8 3.785735 0.202080 0.171832
8 -0.154071 2.504741 0.017996
1 2.303167 2.196735 0.168267
6 -1.725224 0.029546 -0.181600

8 -2.336672 -1.038614 -0.250804
8 -2.401681 1.185069 -0.168088
1 -1.753671 1.941741 -0.099653
6 2.634945 -2.269939 -0.017681
1 3.280744 -2.291132 -0.896885
1 3.242597 -2.313757 0.886938
1 1.946867 -3.113082 -0.041424
8 -5.121988 -0.900087 0.303888
1 -5.300626 -1.690234 0.832841
1 -4.163766 -0.940634 0.104392
1m5caU/1m5caUaae.xyz
6 -1.321757 -0.610101 0.078577
6 1.073353 -1.199084 0.009709
6 1.377358 0.213811 0.002099
6 0.340047 1.110222 0.032554
1 0.529712 2.177215 0.026059
7 -0.959799 0.736622 0.071519
7 -0.274887 -1.503817 0.051968
8 -2.495338 -0.980052 0.108846
8 1.919744 -2.116016 -0.020645
1 -0.531847 -2.488902 0.056080
6 2.769069 0.708604 -0.041700
8 3.064680 1.900714 -0.044822
8 3.735733 -0.223923 -0.082577
1 3.318496 -1.128718 -0.067913
6 -2.048361 1.731892 0.093332
1 -2.639471 1.645713 -0.819288
1 -2.681317 1.556749 0.963587
1 -1.602076 2.722493 0.154768
8 -5.107998 0.162627 -0.184444
1 -5.632030 -0.634280 -0.347050
1 -4.191488 -0.158770 -0.088942
1m5caU/1m5caUacc.xyz
6 2.394364 -0.590902 0.205474
6 0.041736 -1.189792 -0.240340
6 -0.265802 0.221701 -0.292090

6 0.750792 1.124876 -0.106174
1 0.557586 2.190784 -0.140450
7 2.029782 0.756662 0.126506
7 1.365815 -1.489266 0.013981
8 3.548768 -0.945290 0.426187
8 -0.789073 -2.108951 -0.401978
1 1.623891 -2.473035 0.056423
6 -1.638836 0.704337 -0.535091
8 -1.953076 1.890106 -0.564124
8 -2.576035 -0.243533 -0.728837
1 -2.148325 -1.145187 -0.660832
6 3.104188 1.749195 0.313239
1 3.519629 1.651636 1.317487
1 3.887642 1.577131 -0.425924
1 2.680664 2.743083 0.180818
8 -4.647845 -0.119516 1.338195
1 -3.983525 -0.128252 0.626328
1 -4.887316 0.813042 1.437090
1m5caU/1m5caUaax.xyz
6 -2.054336 0.663475 -0.167387
6 0.361400 0.889984 -0.616020
6 0.518070 -0.502760 -0.268008
6 -0.592726 -1.213791 0.112771
1 -0.513312 -2.258571 0.390086
7 -1.830368 -0.674395 0.170137
7 -0.930080 1.370326 -0.538343
8 -3.172285 1.169890 -0.133545
8 1.291094 1.651749 -0.961004
1 -1.079790 2.347489 -0.781298
6 1.836039 -1.171624 -0.294797
8 2.004215 -2.346965 0.020672
8 2.883373 -0.428563 -0.690721
1 2.575278 0.494949 -0.895117
6 -3.005170 -1.463066 0.584373
1 -3.467285 -0.996305 1.455155
1 -3.723056 -1.500323 -0.236388

1 -2.673129 -2.468881 0.834054
8 2.207694 1.659510 2.076496
1 1.933291 0.730608 2.066929
1 2.178748 1.921241 1.143248
1m5caU/1m5caUadp.xyz
6 2.273276 -0.072399 0.099183
6 0.324178 -1.586686 0.151429
6 -0.517024 -0.451709 -0.155922
6 0.073141 0.776234 -0.328523
1 -0.517213 1.650086 -0.577098
7 1.403277 0.977799 -0.209723
7 1.671211 -1.301707 0.264565
8 3.484517 0.092249 0.213090
8 -0.082984 -2.756417 0.312845
1 2.294357 -2.076353 0.483724
6 -1.978137 -0.592728 -0.296229
8 -2.734904 0.349581 -0.537212
8 -2.482613 -1.825194 -0.149093
1 -1.740516 -2.462111 0.048091
6 2.005500 2.310018 -0.397690
1 2.516576 2.608976 0.518650
1 2.720180 2.271754 -1.221063
1 1.209843 3.015633 -0.627993
8 -2.563907 2.850450 0.845397
1 -2.624425 2.006858 0.356814
1 -3.426680 3.270781 0.721280
1m5caU/1m5caUabr.xyz
6 -0.107175 2.153982 -0.000872
6 1.741001 0.515053 -0.001306
6 0.752465 -0.539701 -0.000106
6 -0.575303 -0.194879 0.000173
1 -1.344596 -0.957528 -0.001651
7 -1.007864 1.085343 0.001980
7 1.225020 1.797338 -0.001690
8 -0.479468 3.324207 -0.002325
8 2.977237 0.338729 -0.002200

1 1.890562 2.567361 -0.003895
6 1.132916 -1.967325 0.001820
8 0.317540 -2.886362 0.005040
8 2.449484 -2.238873 0.000607
1 2.960385 -1.384711 -0.001235
6 -2.444881 1.416655 0.005260
1 -2.676957 2.018965 0.884513
1 -2.693462 1.975575 -0.898168
1 -3.010636 0.487264 0.033525
8 -4.171036 -2.162927 -0.123040
1 -3.511593 -1.748295 0.451902
1 -4.877987 -2.438300 0.478511
1m5caU/1m5caUadv.xyz
6 2.396903 -0.593797 0.200993
6 0.042053 -1.187045 -0.240624
6 -0.263089 0.225164 -0.286876
6 0.755434 1.125922 -0.099729
1 0.563886 2.192255 -0.129681
7 2.034372 0.754684 0.128456
7 1.366421 -1.489726 0.008365
8 3.551238 -0.951050 0.417412
8 -0.790869 -2.104188 -0.402917
1 1.622917 -2.474067 0.047114
6 -1.635536 0.711088 -0.526739
8 -1.947312 1.897523 -0.553672
8 -2.574917 -0.234567 -0.720874
1 -2.148852 -1.137251 -0.656029
6 3.110596 1.744733 0.317943
1 3.526483 1.642923 1.321602
1 3.893242 1.573896 -0.422347
1 2.688700 2.739798 0.189206
8 -4.648784 -0.125344 1.339534
1 -5.119348 0.714565 1.240262
1 -3.985591 -0.122200 0.626453
1m5caU/1m5caUaai.xyz
6 1.319105 0.629109 0.071005

6 -1.081145 1.198992 0.006720
6 -1.373819 -0.216285 0.002013
6 -0.329378 -1.104226 0.031864
1 -0.510946 -2.172625 0.028336
7 0.967510 -0.720532 0.067464
7 0.264871 1.514351 0.045070
8 2.489811 1.008584 0.097734
8 -1.935052 2.108914 -0.022620
1 0.514410 2.501314 0.046719
6 -2.761421 -0.722875 -0.036939
8 -3.046917 -1.917471 -0.038461
8 -3.736166 0.201358 -0.074318
1 -3.326454 1.109583 -0.062570
6 2.063289 -1.707745 0.089987
1 2.653780 -1.617700 -0.822571
1 2.694981 -1.527537 0.959991
1 1.624522 -2.701675 0.151807
8 5.082182 -0.195759 -0.192605
1 5.683311 0.560517 -0.142876
1 4.189077 0.184562 -0.092323
1m5caU/1m5caUaci.xyz
6 1.613253 -1.092212 0.000719
6 -0.750690 -0.382377 0.002900
6 -0.299877 0.988616 0.000779
6 1.050283 1.235624 -0.001790
1 1.425174 2.252229 -0.003697
7 1.980624 0.255875 -0.002409
7 0.253495 -1.329158 0.003480
8 2.440275 -1.999228 0.001168
8 -1.951099 -0.737961 0.004318
1 -0.024159 -2.309200 0.006446
6 -1.244494 2.124885 0.001410
8 -0.887276 3.299882 -0.000843
8 -2.553639 1.820421 0.004975
1 -2.661871 0.831445 0.005994
6 3.423907 0.558316 -0.005683

1 3.886279 0.135539 0.887316
1 3.883597 0.128435 -0.896653
1 3.546854 1.639742 -0.010369
8 -2.581830 -3.539562 -0.042547
1 -1.767262 -3.933365 0.301831
1 -2.413189 -2.577380 -0.024946
1m5caU/1m5caUaao.xyz
6 -2.236372 -0.229011 0.097753
6 -0.177709 -1.590991 0.161443
6 0.570840 -0.404008 -0.185520
6 -0.112224 0.771110 -0.380549
1 0.410166 1.677970 -0.660494
7 -1.451966 0.876497 -0.246835
7 -1.541890 -1.406327 0.280588
8 -3.454807 -0.152426 0.225470
8 0.319369 -2.721004 0.349815
1 -2.103860 -2.219829 0.522631
6 2.037914 -0.432191 -0.329121
8 2.715133 0.561637 -0.599014
8 2.640159 -1.614812 -0.144273
1 1.951860 -2.302794 0.074914
6 -2.154167 2.158778 -0.440163
1 -2.882102 2.056061 -1.246113
1 -2.665146 2.433905 0.483779
1 -1.417687 2.917795 -0.696583
8 2.315921 2.967706 0.948527
1 1.360167 3.004886 1.096400
1 2.447707 2.169895 0.401933
1m5caU/1m5caUabs.xyz
6 -2.372111 0.545347 0.232481
6 -0.044835 1.225821 -0.229290
6 0.300620 -0.173669 -0.336367
6 -0.687496 -1.111091 -0.168307
1 -0.466623 -2.169432 -0.244609
7 -1.971267 -0.787679 0.101268
7 -1.372745 1.478489 0.053906

8 -3.531890 0.858041 0.486161
8 0.757404 2.173348 -0.368709
1 -1.656378 2.452709 0.134478
6 1.683030 -0.609407 -0.611949
8 2.028706 -1.784679 -0.682819
8 2.594256 0.368821 -0.779840
1 2.144724 1.256026 -0.678182
6 -3.015360 -1.816067 0.263365
1 -3.429556 -1.759121 1.271049
1 -3.806808 -1.647374 -0.468126
1 -2.563006 -2.792894 0.102083
8 4.398423 -0.077852 1.526068
1 4.138803 -0.989843 1.721758
1 3.888666 0.147221 0.728835
1m5caU/1m5caUaah.xyz
6 2.502497 0.558778 0.104965
6 0.129354 1.212523 -0.082517
6 -0.214155 -0.191226 -0.108630
6 0.795448 -1.117947 -0.034298
1 0.577531 -2.179332 -0.060091
7 2.099748 -0.779743 0.066207
7 1.479813 1.481327 0.029068
8 3.682654 0.884501 0.197431
8 -0.691518 2.151631 -0.155710
1 1.761012 2.459476 0.050828
6 -1.612894 -0.643886 -0.233425
8 -1.952879 -1.822950 -0.235285
8 -2.540591 0.322203 -0.360970
1 -2.100697 1.217544 -0.313644
6 3.162043 -1.800450 0.119136
1 3.831357 -1.669942 -0.732370
1 3.724237 -1.695703 1.047863
1 2.693640 -2.781806 0.076315
8 -5.292205 -0.129297 0.454556
1 -4.360531 0.003054 0.202544
1 -5.400918 0.380169 1.270064

1m5caU/1m5caUacj.xyz
6 -2.263578 -0.128005 0.108297
6 -0.269912 -1.582712 0.164471
6 0.533189 -0.429375 -0.173829
6 -0.094664 0.777788 -0.360566
1 0.466182 1.664128 -0.632234
7 -1.428754 0.941991 -0.228918
7 -1.623694 -1.335892 0.288154
8 -3.477743 0.003480 0.232989
8 0.173715 -2.736685 0.341526
1 -2.221460 -2.124578 0.526787
6 1.995776 -0.528850 -0.329862
8 2.719248 0.432651 -0.597201
8 2.540653 -1.741471 -0.164071
1 1.821704 -2.397539 0.054266
6 -2.073985 2.252239 -0.429570
1 -2.800276 2.177847 -1.240219
1 -2.579559 2.551598 0.489699
1 -1.303974 2.977641 -0.684191
8 2.437102 2.866039 0.892968
1 3.331615 3.101119 1.177290
1 2.548153 2.048470 0.370620
1m5caU/1m5caUaag.xyz
6 1.492265 0.444720 -0.023244
6 -0.433191 -1.097501 -0.019964
6 -1.303598 0.055574 -0.005320
6 -0.739348 1.305837 -0.000154
1 -1.359863 2.194339 0.014007
7 0.597688 1.513118 -0.011910
7 0.918985 -0.806093 -0.030676
8 2.712208 0.616294 -0.026160
8 -0.815154 -2.285591 -0.023131
1 1.563968 -1.593917 -0.042983
6 -2.775358 -0.080621 0.010471
8 -3.543120 0.878067 0.021305
8 -3.262620 -1.332999 0.013745

1 -2.505180 -1.980421 0.002304
6 1.174642 2.870528 0.020253
1 1.772515 2.986852 0.925380
1 1.805278 3.017885 -0.857279
1 0.357425 3.589342 0.017104
8 4.362681 -1.733885 0.004318
1 3.785242 -2.381053 0.434301
1 3.839471 -0.908505 -0.007597
1m5dhmC/1m5dhmCacv.xyz
6 -2.309296 0.208849 -0.184125
6 -0.375481 1.518664 -0.063726
6 0.421344 0.399512 0.361014
6 -0.235473 -0.790710 0.485859
1 0.269217 -1.691995 0.807204
7 -1.561937 -0.910076 0.217947
7 -1.684538 1.403611 -0.316427
8 -3.533837 0.059191 -0.409289
6 1.889683 0.552463 0.685900
1 2.036869 1.258719 1.510400
7 0.171705 2.745679 -0.194955
1 -0.400374 3.486514 -0.578085
1 1.176104 2.851323 -0.237335
8 2.624143 1.114052 -0.395225
1 2.439797 0.593643 -1.195640
8 2.406024 -0.718471 1.043286
1 3.302846 -0.592098 1.390796
6 -2.255263 -2.196083 0.367434
1 -3.049247 -2.105755 1.111602
1 -2.693224 -2.494967 -0.586816
1 -1.535023 -2.946527 0.691494
8 2.425365 -2.330670 -1.323318
1 3.352802 -2.385398 -1.593799
1 2.436971 -1.796951 -0.503546
1m5dhmC/1m5dhmCadr.xyz
6 -1.643275 0.693850 0.017966
6 0.019808 -0.961067 -0.129671

6 1.068056 0.022374 -0.131533
6 0.667873 1.325402 -0.055998
1 1.378285 2.141357 -0.066666
7 -0.643052 1.676311 0.027616
7 -1.271357 -0.608704 -0.058586
8 -2.842550 1.045249 0.084179
6 2.520942 -0.371973 -0.280399
1 2.683695 -0.882489 -1.236809
7 0.293145 -2.275408 -0.229522
1 -0.468990 -2.938051 -0.169654
1 1.236687 -2.611243 -0.091794
8 2.917502 -1.333189 0.694333
1 2.683102 -0.996043 1.575033
8 3.317366 0.792075 -0.208631
1 4.208039 0.561778 -0.513308
6 -1.052417 3.084510 0.112349
1 -1.658126 3.349043 -0.756880
1 -1.638017 3.245190 1.019179
1 -0.159652 3.708268 0.138980
8 -3.342712 -2.510405 0.011010
1 -2.631355 -1.820248 -0.012105
1 -4.167586 -2.011376 0.094046
1m5dhmC/1m5dhmCadg.xyz
6 -2.111823 -0.567324 -0.071584
6 0.092950 -1.245142 -0.458388
6 0.558165 0.113412 -0.527085
6 -0.384541 1.078026 -0.325622
1 -0.138791 2.131261 -0.343408
7 -1.688984 0.771245 -0.093413
7 -1.194328 -1.548063 -0.254957
8 -3.327521 -0.807440 0.124539
6 2.012870 0.440018 -0.789149
1 2.327425 0.042649 -1.761541
7 0.950972 -2.274235 -0.630415
1 0.607766 -3.212925 -0.474577
1 1.946042 -2.112741 -0.548728

8 2.872132 -0.180960 0.155312
1 2.475012 -0.078931 1.051096
8 2.166158 1.848754 -0.777769
1 3.055602 2.049865 -1.105891
6 -2.682410 1.823821 0.152898
1 -3.476566 1.771105 -0.594370
1 -3.118529 1.697722 1.146140
1 -2.189011 2.793246 0.090695
8 1.456900 -0.038742 2.586948
1 0.930876 -0.839477 2.435208
1 0.853946 0.685838 2.358215
1m5dhmC/1m5dhmCacu.xyz
6 1.643448 0.695969 -0.019266
6 -0.018300 -0.960310 0.127977
6 -1.067318 0.022304 0.131230
6 -0.668229 1.325640 0.055295
1 -1.379283 2.141031 0.066455
7 0.642356 1.677585 -0.029186
7 1.272574 -0.606959 0.055994
8 2.842437 1.048600 -0.084028
6 -2.519695 -0.373196 0.281902
1 -2.680626 -0.884436 1.238234
7 -0.290498 -2.274904 0.227383
1 0.472225 -2.936896 0.167761
1 -1.233906 -2.611548 0.090779
8 -2.916982 -1.334097 -0.692833
1 -2.683951 -0.996297 -1.573644
8 -3.317087 0.790312 0.212090
1 -4.207070 0.559243 0.518198
6 1.050531 3.086164 -0.113601
1 1.654443 3.351626 0.756610
1 1.637583 3.247068 -1.019437
1 0.157225 3.709069 -0.142083
8 3.338098 -2.515245 -0.024773
1 2.629986 -1.822089 0.005785
1 4.168000 -2.022824 0.047126

1m5dhmC/1m5dhmCaaf.xyz
6 2.125768 -0.567084 0.038555
6 -0.070125 -1.258615 0.451999
6 -0.539294 0.097149 0.546845
6 0.397886 1.067791 0.346424
1 0.148727 2.119740 0.383675
7 1.699828 0.769837 0.091605
7 1.214685 -1.553587 0.222203
8 3.338420 -0.799910 -0.183031
6 -1.992114 0.415345 0.831372
1 -2.293427 0.000856 1.800893
7 -0.921067 -2.293189 0.623170
1 -0.576993 -3.228859 0.452092
1 -1.918273 -2.135383 0.565530
8 -2.861434 -0.192760 -0.111660
1 -2.490836 -0.053996 -1.014282
8 -2.148763 1.823638 0.844866
1 -3.038443 2.016539 1.177329
6 2.686204 1.829927 -0.151395
1 3.487825 1.771411 0.587498
1 3.113080 1.718045 -1.150255
1 2.189467 2.796455 -0.072538
8 -1.569202 0.119508 -2.590274
1 -1.640224 -0.707126 -3.091171
1 -0.657144 0.130014 -2.259617
1m5dhmC/1m5dhmCabt.xyz
6 -1.447327 -0.548297 -0.455486
6 0.769036 -1.248676 -0.228493
6 1.244572 0.105927 -0.151427
6 0.292661 1.079968 -0.234060
1 0.544717 2.131425 -0.200711
7 -1.027442 0.784963 -0.372685
7 -0.530603 -1.537523 -0.380346
8 -2.676203 -0.782210 -0.601219
6 2.718586 0.423055 -0.032684
1 3.267897 0.024459 -0.893590

7 1.627667 -2.287056 -0.178096
1 1.248145 -3.224292 -0.154567
1 2.574728 -2.147113 0.146584
8 3.314875 -0.225482 1.088153
1 2.794544 -0.010964 1.880661
8 2.878784 1.824320 0.043691
1 3.821793 2.021486 -0.064476
6 -2.034682 1.851297 -0.466287
1 -2.580813 1.764560 -1.407359
1 -2.738808 1.774589 0.363570
1 -1.525646 2.813344 -0.425189
8 -4.597079 -0.117707 1.300510
1 -4.613997 0.843882 1.405701
1 -3.900007 -0.285070 0.628808
1m5dhmC/1m5dhmCacb.xyz
6 -2.593215 0.143677 0.145421
6 -0.602760 1.371715 0.158432
6 0.195128 0.187523 -0.011057
6 -0.493837 -0.985637 -0.114858
1 0.012077 -1.933648 -0.239608
7 -1.851859 -1.031154 -0.057464
7 -1.937499 1.325410 0.240184
8 -3.842072 0.057670 0.228817
6 1.707370 0.239614 -0.017104
1 2.077869 0.657623 0.926950
7 -0.020567 2.584142 0.275641
1 -0.618961 3.399252 0.301158
1 0.942885 2.706593 -0.006157
8 2.198488 1.152100 -1.006436
1 1.802205 0.914466 -1.861089
8 2.222360 -1.053474 -0.214392
1 3.166280 -1.047205 0.072520
6 -2.574683 -2.303731 -0.181849
1 -3.120526 -2.517079 0.739711
1 -3.283330 -2.247565 -1.010263
1 -1.854435 -3.099012 -0.371610

8 4.812890 -0.984092 0.691934
1 5.236557 -1.854988 0.657365
1 5.347509 -0.423825 0.109205
1m5dhmC/1m5dhmCabn.xyz
6 2.587843 0.121759 -0.158726
6 0.612772 1.374774 -0.164450
6 -0.198325 0.202619 0.024747
6 0.477041 -0.978210 0.131697
1 -0.039397 -1.918570 0.270321
7 1.833511 -1.041808 0.060169
7 1.946081 1.310725 -0.257876
8 3.834788 0.019016 -0.252470
6 -1.710115 0.271717 0.046551
1 -2.087163 0.675723 -0.901181
7 0.045015 2.593352 -0.289054
1 0.654003 3.400072 -0.327953
1 -0.913705 2.730873 0.002045
8 -2.180406 1.207310 1.023703
1 -1.778541 0.980287 1.878662
8 -2.235242 -1.012297 0.273650
1 -3.178472 -1.009763 -0.014962
6 2.540470 -2.323237 0.184201
1 3.070397 -2.550776 -0.743299
1 3.261216 -2.270973 1.002359
1 1.812008 -3.106992 0.389408
8 -4.848144 -1.020171 -0.585989
1 -5.107647 -1.939881 -0.747990
1 -4.891037 -0.593289 -1.455345
1m5dhmC/1m5dhmCaad.xyz
6 -1.541540 -0.356814 -0.009996
6 0.278662 1.096655 0.139684
6 1.215107 0.005913 0.145927
6 0.678272 -1.245629 0.061432
1 1.294112 -2.135003 0.066024
7 -0.663434 -1.446510 -0.027153
7 -1.044071 0.896336 0.072771

8 -2.776788 -0.590260 -0.081322
6 2.701763 0.246198 0.275205
1 2.924635 0.788346 1.201682
7 0.707272 2.372185 0.230005
1 0.028734 3.116278 0.136885
1 1.679513 2.592165 0.062321
8 3.193363 1.104245 -0.752133
1 2.916698 0.744753 -1.611601
8 3.369929 -0.997951 0.266161
1 4.291671 -0.839914 0.521256
6 -1.223832 -2.800378 -0.134239
1 -1.889976 -2.994933 0.708430
1 -1.787497 -2.894102 -1.064469
1 -0.404503 -3.518579 -0.127777
8 -4.610226 1.445375 0.026182
1 -4.220536 2.251278 -0.340113
1 -3.892033 0.768679 -0.021184
1m5dhmC/1m5dhmCaaw.xyz
6 1.444475 -0.522613 -0.439822
6 -0.725036 -1.248364 0.030702
6 -1.209521 0.100708 0.139570
6 -0.288995 1.085747 -0.070423
1 -0.547941 2.134181 -0.008751
7 1.008553 0.806378 -0.365668
7 0.558280 -1.522204 -0.239784
8 2.657313 -0.740827 -0.700132
6 -2.647528 0.399826 0.501347
1 -2.891443 -0.015422 1.486231
7 -1.549515 -2.296890 0.228250
1 -1.190243 -3.228106 0.064997
1 -2.551279 -2.160904 0.216826
8 -3.563522 -0.243313 -0.381774
1 -3.324206 -0.011888 -1.294965
8 -2.834860 1.799798 0.501330
1 -3.695602 1.983603 0.907379
6 1.974950 1.881462 -0.630316

1 2.788036 1.839983 0.096198
1 2.386575 1.768074 -1.635166
1 1.462932 2.839788 -0.551058
8 4.594926 -0.014456 1.182246
1 5.408368 -0.418377 0.848669
1 3.912645 -0.232130 0.510343
1m5dhmC/1m5dhmCadq.xyz
6 -1.457711 0.508348 -0.481763
6 0.702787 1.245924 0.009865
6 1.190357 -0.100306 0.136829
6 0.275794 -1.090513 -0.075218
1 0.537590 -2.137432 -0.000873
7 -1.019053 -0.818243 -0.388497
7 -0.577667 1.512796 -0.280427
8 -2.668670 0.719707 -0.758124
6 2.625186 -0.390560 0.517792
1 2.854782 0.030561 1.503690
7 1.520683 2.299278 0.209893
1 1.160591 3.227080 0.029500
1 2.523042 2.166499 0.208833
8 3.549049 0.253035 -0.356869
1 3.323576 0.015176 -1.271976
8 2.819491 -1.789497 0.526560
1 3.676160 -1.966919 0.943886
6 -1.979221 -1.899303 -0.652025
1 -2.796602 -1.857020 0.069676
1 -2.384856 -1.794539 -1.660130
1 -1.463443 -2.854759 -0.563110
8 -4.515066 0.005309 1.213812
1 -3.865794 0.236617 0.514060
1 -4.654363 0.827945 1.703733
1m5dhmC/1m5dhmCach.xyz
6 -1.543306 0.362920 0.001751
6 0.273583 -1.094869 -0.146313
6 1.212661 -0.006406 -0.147894
6 0.678742 1.246400 -0.063736

1 1.296866 2.134220 -0.065211
7 -0.662730 1.450530 0.021101
7 -1.048823 -0.891360 -0.082832
8 -2.778120 0.598837 0.072391
6 2.699265 -0.250751 -0.268152
1 2.926339 -0.798184 -1.190485
7 0.699186 -2.371330 -0.237987
1 0.018480 -3.113849 -0.148214
1 1.670262 -2.594632 -0.068194
8 3.182206 -1.104475 0.766880
1 2.897402 -0.741398 1.622194
8 3.370432 0.991832 -0.261680
1 4.293355 0.830142 -0.510117
6 -1.220257 2.805529 0.128878
1 -1.885464 3.002074 -0.714050
1 -1.784234 2.899817 1.058870
1 -0.399370 3.521944 0.123332
8 -4.592468 -1.456308 0.042553
1 -3.880444 -0.771351 0.046992
1 -4.127148 -2.299692 -0.045460
1m5dhmC/1m5dhmCaco.xyz
6 2.584578 0.172025 -0.157766
6 0.574015 1.366463 -0.153905
6 -0.202263 0.169017 0.022340
6 0.506713 -0.992805 0.118525
1 0.017485 -1.949160 0.246724
7 1.864831 -1.015523 0.049144
7 1.908437 1.342781 -0.246970
8 3.833953 0.107701 -0.250017
6 -1.714696 0.198583 0.044966
1 -2.101905 0.607631 -0.896548
7 -0.030536 2.568562 -0.266645
1 0.553452 3.394023 -0.294453
1 -0.992147 2.673508 0.028576
8 -2.208105 1.107803 1.035804
1 -1.798629 0.880530 1.887057

8 -2.209929 -1.100847 0.251945
1 -3.157737 -1.100899 -0.020917
6 2.611154 -2.275139 0.169604
1 3.154117 -2.479845 -0.755571
1 3.324611 -2.205105 0.992810
1 1.906798 -3.083155 0.365707
8 -4.834404 -0.995613 -0.572741
1 -4.994113 -1.651645 -1.268047
1 -4.920187 -0.137843 -1.016553
1m5dhmC/1m5dhmCadi.xyz
6 2.596232 0.130268 -0.134571
6 0.614963 1.373075 -0.166419
6 -0.193073 0.195371 -0.000018
6 0.485882 -0.983952 0.100428
1 -0.028650 -1.927956 0.219645
7 1.843497 -1.041147 0.045770
7 1.949952 1.316459 -0.237469
8 3.846004 0.036763 -0.191926
6 -1.705005 0.259668 0.007427
1 -2.074001 0.679965 -0.936176
7 0.043422 2.589842 -0.291284
1 0.648895 3.399810 -0.313987
1 -0.920773 2.721438 -0.016357
8 -2.186913 1.176151 0.997183
1 -1.789516 0.936861 1.850852
8 -2.228708 -1.029716 0.206587
1 -3.176837 -1.016647 -0.065458
6 2.555726 -2.318998 0.178105
1 3.191735 -2.481735 -0.693766
1 3.177646 -2.308267 1.075980
1 1.823624 -3.122643 0.252175
8 -4.843340 -0.956717 -0.628901
1 -5.083397 -1.775134 -1.089278
1 -5.451530 -0.906196 0.123992
1m5dhmC/1m5dhmCabx.xyz
6 -2.588724 0.187579 0.140244

6 -0.567410 1.363691 0.148179
6 0.199703 0.157732 -0.009646
6 -0.518186 -0.999312 -0.095896
1 -0.035495 -1.961018 -0.207703
7 -1.876991 -1.010077 -0.035370
7 -1.902915 1.352937 0.227038
8 -3.840197 0.136955 0.208144
6 1.712288 0.174904 -0.017689
1 2.092991 0.592184 0.922682
7 0.047166 2.560959 0.256800
1 -0.528684 3.392410 0.272888
1 1.013333 2.654163 -0.027171
8 2.222827 1.068661 -1.014335
1 1.819796 0.834710 -1.866822
8 2.200210 -1.130485 -0.204343
1 3.146276 -1.128498 0.075089
6 -2.634281 -2.263252 -0.157275
1 -3.266993 -2.402675 0.720947
1 -3.263958 -2.232435 -1.049159
1 -1.931724 -3.092386 -0.237340
8 4.785687 -0.940805 0.699798
1 5.374136 -1.650681 0.401592
1 5.140946 -0.135170 0.294103
1m5dhmC/1m5dhmCabi.xyz
6 2.583408 0.203233 -0.150065
6 0.552481 1.361959 -0.143949
6 -0.203331 0.149054 0.015164
6 0.525233 -1.001067 0.105077
1 0.052508 -1.967351 0.221046
7 1.883996 -0.998547 0.044156
7 1.887455 1.363130 -0.231380
8 3.833946 0.161143 -0.237722
6 -1.715793 0.153242 0.025557
1 -2.101801 0.569285 -0.913325
7 -0.073926 2.553900 -0.245168
1 0.494139 3.390739 -0.259882

1 -1.038608 2.636463 0.047286
8 -2.233278 1.040480 1.024348
1 -1.830555 0.805859 1.876804
8 -2.191880 -1.156820 0.210037
1 -3.141621 -1.159116 -0.056623
6 2.653525 -2.244264 0.163281
1 3.215511 -2.427655 -0.754803
1 3.352240 -2.168518 0.998619
1 1.962921 -3.068475 0.340074
8 -4.823224 -0.996067 -0.573241
1 -4.877466 -1.101970 -1.535128
1 -5.027595 -0.061492 -0.415094
1m5dhmC/1m5dhmCabb.xyz
6 2.252467 0.361883 0.154669
6 0.214047 1.504909 0.047715
6 -0.486031 0.326306 -0.387689
6 0.268921 -0.803441 -0.519299
1 -0.158449 -1.740510 -0.849765
7 1.599365 -0.815689 -0.244568
7 1.528732 1.498592 0.296969
8 3.486714 0.316595 0.370785
6 -1.960878 0.354369 -0.722287
1 -2.155699 1.013880 -1.575209
7 -0.434279 2.680344 0.192015
1 0.073807 3.457779 0.592801
1 -1.444103 2.692441 0.243989
8 -2.741910 0.903728 0.332609
1 -2.540363 0.416895 1.150141
8 -2.377911 -0.965619 -1.026662
1 -3.276572 -0.921223 -1.388785
6 2.394435 -2.042699 -0.385839
1 3.145232 -1.913907 -1.168433
1 2.895610 -2.267665 0.557358
1 1.728620 -2.864556 -0.647430
8 -2.079338 -2.242690 1.551052
1 -2.268819 -1.928106 0.645840

1 -1.254184 -1.791833 1.782678
1m5dhmC/1m5dhmCads.xyz
6 2.584665 0.202322 -0.142879
6 0.553944 1.361888 -0.147160
6 -0.203029 0.149592 0.010712
6 0.524303 -1.001626 0.096352
1 0.049784 -1.967344 0.209030
7 1.883043 -1.001053 0.034177
7 1.889363 1.362335 -0.227925
8 3.836303 0.161519 -0.213539
6 -1.715553 0.154656 0.021828
1 -2.102174 0.568273 -0.917893
7 -0.071279 2.553962 -0.253819
1 0.497370 3.390416 -0.267908
1 -1.037198 2.637954 0.034055
8 -2.231837 1.044718 1.018599
1 -1.827370 0.813301 1.871115
8 -2.191835 -1.154792 0.210276
1 -3.142660 -1.157152 -0.052478
6 2.651187 -2.247701 0.154950
1 3.280514 -2.384186 -0.726172
1 3.285192 -2.209424 1.043425
1 1.956116 -3.082522 0.240864
8 -4.827725 -0.996984 -0.558351
1 -4.887759 -1.104080 -1.519756
1 -5.032058 -0.062435 -0.400009
1m5dhmC/1m5dhmCaaj.xyz
6 -1.457785 0.537315 -0.480725
6 0.708969 1.248290 0.022115
6 1.183885 -0.103985 0.133144
6 0.260459 -1.082863 -0.092103
1 0.512073 -2.133048 -0.029824
7 -1.031432 -0.794614 -0.403928
7 -0.568511 1.530763 -0.266954
8 -2.666965 0.762744 -0.753765
6 2.615645 -0.412058 0.512174

1 2.849230 -0.000767 1.501220
7 1.536569 2.290815 0.236665
1 1.185388 3.225212 0.074220
1 2.537565 2.148951 0.238997
8 3.546167 0.228555 -0.357629
1 3.318184 0.000271 -1.274554
8 2.795225 -1.813002 0.510643
1 3.647465 -2.003291 0.931298
6 -2.000905 -1.863747 -0.680921
1 -2.817892 -1.824546 0.041598
1 -2.406550 -1.742258 -1.687112
1 -1.493333 -2.824663 -0.604733
8 -4.474847 0.139839 1.281809
1 -3.839233 0.306034 0.551812
1 -4.507523 -0.823442 1.366040
1m5dhmC/1m5dhmCaah.xyz
6 -2.254587 0.356126 -0.155603
6 -0.220492 1.506879 -0.049427
6 0.483881 0.331235 0.386952
6 -0.266778 -0.801124 0.519866
1 0.164167 -1.736373 0.851005
7 -1.597197 -0.818621 0.244946
7 -1.535100 1.495391 -0.298623
8 -3.488551 0.306174 -0.372289
6 1.958949 0.364884 0.719506
1 2.153277 1.027942 1.569769
7 0.423555 2.684560 -0.194716
1 -0.087264 3.459946 -0.595990
1 1.433298 2.700442 -0.247027
8 2.736688 0.912299 -0.338757
1 2.535109 0.421394 -1.153895
8 2.380195 -0.952868 1.027962
1 3.279733 -0.904677 1.387517
6 -2.387438 -2.048769 0.386082
1 -3.140193 -1.922365 1.167167
1 -2.886037 -2.276718 -0.557787

1 -1.718666 -2.867573 0.649633
8 2.101864 -2.250515 -1.539631
1 2.274437 -1.919124 -0.636929
1 1.230559 -1.888305 -1.757254
1m5dhmU/1m5dhmUadr.xyz
6 -1.720754 0.487317 -0.009231
6 0.354966 -0.839284 -0.006257
6 1.101892 0.396361 0.011629
6 0.419233 1.571249 0.014113
1 0.932909 2.525861 0.027966
7 -0.949865 1.638235 -0.001444
7 -1.021231 -0.699215 -0.015730
8 -2.958097 0.520050 -0.010136
8 0.876072 -1.974918 -0.009648
1 -1.597329 -1.558256 -0.010480
6 2.612027 0.349014 0.028767
8 3.105923 -0.347857 1.164206
1 2.695496 -1.230002 1.177417
8 3.139868 -0.197303 -1.173557
1 2.749361 -1.077446 -1.307074
1 3.016479 1.359962 0.097354
6 -1.659872 2.927733 0.013825
1 -2.297958 3.007768 -0.868066
1 -2.273940 3.000576 0.913528
1 -0.922693 3.729010 0.006580
8 -2.762154 -2.961666 0.083054
1 -2.738315 -3.473253 -0.739644
1 -3.624176 -2.518146 0.074196
1m5dhmU/1m5dhmUaae.xyz
6 2.187376 0.778976 0.000811
6 -0.252374 0.834832 0.410747
6 -0.288799 -0.590699 0.200352
6 0.883392 -1.230592 -0.066972
1 0.917757 -2.304135 -0.215363
7 2.088712 -0.590125 -0.171761
7 0.996838 1.417982 0.280536

8 3.257724 1.389658 -0.080186
8 -1.235057 1.551865 0.708741
1 1.058373 2.422170 0.431311
6 -1.589649 -1.371101 0.290055
8 -2.361858 -0.971778 1.412201
1 -2.406670 0.002165 1.409644
8 -2.344475 -1.315056 -0.908578
1 -2.621819 -0.386136 -1.085782
1 -1.361221 -2.428086 0.440149
6 3.334453 -1.331438 -0.430889
1 3.796235 -0.969534 -1.350932
1 4.026045 -1.190673 0.401769
1 3.091432 -2.387633 -0.533025
8 -3.197085 1.338676 -1.284959
1 -2.607612 1.595741 -0.546698
1 -4.091759 1.364340 -0.914874
1m5dhmU/1m5dhmUacv.xyz
6 2.120089 -0.584228 -0.254430
6 -0.250871 -1.284602 -0.370500
6 -0.586904 -0.132922 0.431798
6 0.410046 0.701083 0.830838
1 0.206308 1.585861 1.423372
7 1.728066 0.495411 0.518411
7 1.100299 -1.424472 -0.652439
8 3.297534 -0.787075 -0.568167
8 -1.072880 -2.118859 -0.798012
1 1.380358 -2.222975 -1.216975
6 -2.034324 0.154001 0.758800
8 -2.746883 0.618587 -0.393195
1 -2.702963 -0.061545 -1.086899
8 -2.688318 -0.953881 1.344706
1 -2.533232 -1.728994 0.775824
1 -2.099418 0.969976 1.479492
6 2.774292 1.439972 0.941946
1 3.530652 0.915306 1.528056
1 3.242986 1.891620 0.065589

1 2.311964 2.214622 1.551572
8 -1.244173 2.804129 -1.492421
1 -1.761205 2.061519 -1.120028
1 -0.330220 2.615006 -1.235805
1m5dhmU/1m5dhmUada.xyz
6 -2.051191 -0.622043 -0.203542
6 0.363469 -1.157123 -0.288838
6 0.608963 0.017401 0.512238
6 -0.447097 0.783715 0.892151
1 -0.309792 1.686486 1.476787
7 -1.744594 0.485585 0.567708
7 -0.971575 -1.394467 -0.582275
8 -3.208203 -0.908301 -0.528951
8 1.249084 -1.927089 -0.708107
1 -1.187622 -2.208979 -1.151905
6 2.032747 0.417413 0.821672
8 2.725963 -0.565983 1.566426
1 2.655289 -1.412815 1.093001
8 2.747784 0.745928 -0.373823
1 2.820982 -0.060389 -0.914006
1 2.042785 1.325857 1.425260
6 -2.862570 1.338452 1.005493
1 -3.401363 1.716788 0.135054
1 -3.545189 0.761576 1.632013
1 -2.455799 2.171981 1.575478
8 0.851857 1.981544 -2.208001
1 0.066636 1.454733 -1.998772
1 1.529452 1.651247 -1.586340
1m5dhmU/1m5dhmUacj.xyz
6 1.465397 0.464527 -0.582146
6 -0.898803 1.057331 -0.170266
6 -1.180873 -0.350806 -0.019975
6 -0.163874 -1.240664 -0.162094
1 -0.326351 -2.307957 -0.062105
7 1.126044 -0.865809 -0.443223
7 0.426852 1.358810 -0.453464

8 2.626550 0.837920 -0.804880
8 -1.743191 1.966951 -0.065969
1 0.673969 2.341011 -0.549341
6 -2.592588 -0.795447 0.293012
8 -3.006661 -0.374768 1.585713
1 -2.917455 0.591302 1.640779
8 -3.512223 -0.373809 -0.702825
1 -3.440961 0.592355 -0.791059
1 -2.643221 -1.885228 0.306516
6 2.205791 -1.859995 -0.568211
1 2.696534 -1.749883 -1.536247
1 2.936824 -1.715161 0.228998
1 1.769793 -2.854316 -0.488669
8 4.297123 0.430614 1.531332
1 3.758700 0.510890 0.721660
1 4.318810 -0.518951 1.717393
1m5dhmU/1m5dhmUabf.xyz
6 -2.131644 -0.565148 -0.261898
6 0.229209 -1.298417 -0.375769
6 0.579352 -0.156640 0.434202
6 -0.406161 0.690825 0.832924
1 -0.191131 1.570838 1.428318
7 -1.726381 0.504424 0.518090
7 -1.123199 -1.418632 -0.660451
8 -3.310922 -0.748953 -0.580654
8 1.040537 -2.141462 -0.807337
1 -1.413271 -2.209858 -1.230093
6 2.029783 0.112934 0.761566
8 2.680069 -1.012488 1.317070
1 2.512485 -1.773063 0.731713
8 2.738719 0.599436 -0.384264
1 2.686114 -0.065450 -1.091987
1 2.104054 0.911906 1.500074
6 -2.759735 1.463723 0.939853
1 -3.208267 1.934763 0.063079
1 -3.532673 0.945926 1.510106

1 -2.290780 2.222250 1.564571
8 1.167986 2.793084 -1.366539
1 1.761491 3.395922 -1.836061
1 1.743116 2.065617 -1.054661
1m5dhmU/1m5dhmUabr.xyz
6 2.157614 -0.515045 -0.285874
6 -0.188728 -1.293703 -0.393401
6 -0.555943 -0.168920 0.432676
6 0.415406 0.691191 0.838422
1 0.187295 1.556508 1.450436
7 1.736883 0.535037 0.512078
7 1.163299 -1.382268 -0.690673
8 3.338235 -0.671289 -0.614056
8 -0.986312 -2.148729 -0.826953
1 1.465187 -2.160057 -1.272516
6 -2.008150 0.056348 0.782692
8 -2.753721 0.510652 -0.351926
1 -2.682324 -0.151152 -1.060785
8 -2.610510 -1.087399 1.355491
1 -2.433710 -1.843163 0.766870
1 -2.095915 0.856114 1.518733
6 2.755719 1.504879 0.944940
1 3.536871 0.991734 1.508277
1 3.196379 1.993154 0.073698
1 2.276122 2.248650 1.579268
8 -1.395687 2.820862 -1.358699
1 -1.890699 2.040949 -1.035463
1 -2.052026 3.356613 -1.825933
1m5dhmU/1m5dhmUabe.xyz
6 -1.857810 -0.944151 -0.133196
6 0.587579 -1.180462 -0.440270
6 0.762176 -0.007077 0.382743
6 -0.339878 0.606540 0.888755
1 -0.259782 1.497643 1.501380
7 -1.616383 0.161983 0.662416
7 -0.730835 -1.570912 -0.626134

8 -2.997494 -1.347266 -0.387800
8 1.516460 -1.824646 -0.964800
1 -0.899275 -2.387788 -1.208446
6 2.152641 0.531973 0.629161
8 2.989580 -0.415794 1.273976
1 2.995350 -1.231159 0.743938
8 2.747942 1.014060 -0.569095
1 2.739830 0.299887 -1.228406
1 2.103104 1.390895 1.300090
6 -2.782021 0.912211 1.154301
1 -3.313761 1.366148 0.314676
1 -3.452315 0.238261 1.689087
1 -2.430242 1.688912 1.831265
8 -0.623451 2.885238 -1.579989
1 -1.339237 2.711136 -0.951031
1 0.086444 2.281621 -1.313817
1m5dhmU/1m5dhmUaab.xyz
6 -2.180401 0.762592 0.025113
6 0.258017 0.831161 0.440843
6 0.306266 -0.590214 0.207132
6 -0.858375 -1.233123 -0.085273
1 -0.882825 -2.303667 -0.255896
7 -2.067412 -0.599779 -0.187801
7 -0.997010 1.405266 0.326073
8 -3.256370 1.364883 -0.043139
8 1.236438 1.551667 0.745172
1 -1.067761 2.405535 0.497666
6 1.609775 -1.366003 0.305199
8 2.373504 -1.309439 -0.887464
1 2.634612 -0.377672 -1.074041
8 2.371797 -0.963573 1.433053
1 2.411885 0.010785 1.431433
1 1.383597 -2.423537 0.454839
6 -3.303180 -1.342827 -0.485456
1 -4.002616 -1.243936 0.346751
1 -3.762178 -0.948670 -1.393525

1 -3.048070 -2.391458 -0.628296
8 3.197913 1.347626 -1.266510
1 2.573350 1.611509 -0.560581
1 2.832086 1.723867 -2.080501
1m5dhmU/1m5dhmUaah.xyz
6 2.135741 -0.568915 -0.252629
6 -0.225506 -1.302737 -0.361291
6 -0.579187 -0.138200 0.414501
6 0.405253 0.717824 0.797496
1 0.188029 1.610772 1.372924
7 1.726466 0.524829 0.490701
7 1.128205 -1.430258 -0.636601
8 3.317327 -0.765463 -0.554640
8 -1.035027 -2.157223 -0.772165
1 1.421350 -2.238284 -1.180555
6 -2.030346 0.130407 0.739050
8 -2.755863 0.553872 -0.419892
1 -2.684646 -0.131544 -1.105774
8 -2.661614 -0.974688 1.355100
1 -2.496920 -1.760401 0.803875
1 -2.108026 0.961801 1.440570
6 2.760758 1.486876 0.903984
1 3.507136 0.983267 1.520816
1 3.244573 1.911426 0.022497
1 2.284721 2.278972 1.479582
8 -1.341236 2.836506 -1.441198
1 -1.845436 2.063604 -1.116181
1 -0.482450 2.762813 -1.000775
1m5dhmU/1m5dhmUabm.xyz
6 2.048085 -0.631629 -0.197874
6 -0.367343 -1.159782 -0.299316
6 -0.613264 0.004047 0.517215
6 0.443972 0.757419 0.918993
1 0.306716 1.652224 1.515716
7 1.743110 0.454325 0.604935
7 0.968677 -1.399713 -0.585870

8 3.203078 -0.904307 -0.541313
8 -1.253518 -1.918959 -0.736730
1 1.184121 -2.201881 -1.173028
6 -2.037562 0.409765 0.816551
8 -2.733981 0.767051 -0.381740
1 -2.806939 -0.028004 -0.938339
8 -2.748326 -0.582036 1.533038
1 -2.673740 -1.421447 1.046966
1 -2.048169 1.306462 1.437453
6 2.852735 1.336426 1.004140
1 3.626107 0.751594 1.503608
1 3.276754 1.822666 0.123021
1 2.465153 2.088662 1.689323
8 -0.811042 2.032802 -2.169844
1 -0.026937 1.504157 -1.961190
1 -1.495007 1.689383 -1.562516
1m5dhmU/1m5dhmUadb.xyz
6 -2.191342 -0.483431 -0.254064
6 0.124654 -1.350524 -0.350352
6 0.546109 -0.189300 0.395777
6 -0.385802 0.734963 0.750687
1 -0.116215 1.628080 1.303093
7 -1.714892 0.615109 0.440800
7 -1.236161 -1.412554 -0.612992
8 -3.385004 -0.626272 -0.538392
8 0.883921 -2.257716 -0.745119
1 -1.576517 -2.219212 -1.130741
6 2.009570 0.001737 0.719753
8 2.587878 -1.142765 1.314589
1 2.374631 -1.910044 0.753088
8 2.750872 0.408060 -0.435736
1 2.617168 -0.244261 -1.143990
1 2.131146 0.816939 1.433731
6 -2.693639 1.642384 0.833236
1 -3.208767 2.014702 -0.053614
1 -3.421832 1.214824 1.525218

1 -2.161792 2.458997 1.318383
8 1.571337 2.881997 -1.237224
1 2.282664 3.537940 -1.249560
1 2.007547 2.042741 -0.985438
1m5dhmU/1m5dhmUabd.xyz
6 -1.888670 0.755971 0.056221
6 0.563524 0.461004 0.110983
6 0.359039 -0.961368 0.011998
6 -0.914644 -1.433708 -0.058251
1 -1.121200 -2.495303 -0.137674
7 -2.015630 -0.618817 -0.037191
7 -0.590991 1.223113 0.131461
8 -2.861063 1.516604 0.073215
8 1.686771 1.007835 0.174349
1 -0.486350 2.233423 0.196808
6 1.558127 -1.881723 -0.036270
8 2.347418 -1.657291 -1.196718
1 2.606974 -0.720581 -1.215766
8 2.344348 -1.796103 1.143168
1 2.600986 -0.867202 1.272866
1 1.225816 -2.918659 -0.098939
6 -3.380266 -1.166434 -0.112591
1 -3.946933 -0.865545 0.770214
1 -3.879217 -0.794309 -1.009166
1 -3.313948 -2.252138 -0.151882
8 1.868750 3.805453 -0.080590
1 1.855150 3.966314 -1.034729
1 1.832617 2.829491 0.008454
1m5dhmU/1m5dhmUack.xyz
6 2.123167 -0.553044 0.286178
6 -0.264417 -1.193789 0.393964
6 -0.565509 -0.050763 -0.434071
6 0.452584 0.757824 -0.830736
1 0.275071 1.635265 -1.442393
7 1.761819 0.531295 -0.494700
7 1.079109 -1.356124 0.698594

8 3.295266 -0.789917 0.596399
8 -1.112501 -1.997788 0.827120
1 1.334875 -2.152706 1.277136
6 -2.001205 0.229881 -0.814133
8 -2.548788 -0.775621 -1.647760
1 -2.438068 -1.638053 -1.213433
8 -2.817624 0.436139 0.340409
1 -2.761496 -0.358348 0.900427
1 -2.061829 1.159137 -1.381963
6 2.843081 1.401593 -0.985665
1 3.398688 1.812520 -0.141267
1 3.519910 0.827750 -1.621449
1 2.397812 2.212006 -1.560279
8 -1.396896 2.453858 1.795408
1 -0.505377 2.338894 1.436018
1 -1.927954 1.773764 1.334120
1m5dhmU/1m5dhmUaao.xyz
6 1.436585 -0.491562 -0.485088
6 -0.939683 -1.063047 -0.110669
6 -1.214075 0.348773 0.015987
6 -0.187474 1.229496 -0.111669
1 -0.344699 2.298934 -0.028258
7 1.106080 0.842532 -0.359412
7 0.389608 -1.377138 -0.360044
8 2.594789 -0.878624 -0.697111
8 -1.793295 -1.964999 -0.013029
1 0.631389 -2.361833 -0.443317
6 -2.632929 0.805707 0.270307
8 -3.495769 0.459237 -0.804047
1 -3.439010 -0.501322 -0.943942
8 -3.134514 0.319266 1.506745
1 -3.066850 -0.650848 1.503829
1 -2.666150 1.893769 0.341334
6 2.190960 1.831067 -0.488660
1 2.909009 1.704723 0.323153
1 2.697209 1.697741 -1.445748

1 1.755921 2.827741 -0.439275
8 4.663984 -0.336935 1.194308
1 4.501104 0.527782 1.597322
1 3.950381 -0.440618 0.534907
1m5dhmU/1m5dhmUaax.xyz
6 -1.390648 -0.570600 -0.087884
6 1.029064 -1.073948 -0.016533
6 1.280703 0.347743 -0.020178
6 0.223300 1.199729 -0.059315
1 0.361290 2.275045 -0.063041
7 -1.081325 0.774807 -0.098686
7 -0.312449 -1.427897 -0.053780
8 -2.553843 -0.995824 -0.110383
8 1.913123 -1.950963 0.017544
1 -0.537069 -2.419987 -0.047809
6 2.706208 0.848029 0.034506
8 3.337399 0.513196 1.263224
1 3.285220 -0.449562 1.386446
8 3.471263 0.399185 -1.074115
1 3.424328 -0.571808 -1.104061
1 2.717501 1.937604 -0.016289
6 -2.199019 1.733661 -0.131601
1 -2.833490 1.528680 -0.994843
1 -2.786297 1.649608 0.784385
1 -1.787546 2.738333 -0.211683
8 -5.177066 0.035355 0.222002
1 -5.665264 -0.787697 0.364753
1 -4.246912 -0.243855 0.109248
1m5dhmU/1m5dhmUaco.xyz
6 1.736863 -0.480042 -0.051945
6 -0.350875 0.827126 -0.059204
6 -1.086045 -0.414189 0.006091
6 -0.392087 -1.581936 0.039119
1 -0.896559 -2.539889 0.095178
7 0.977451 -1.637607 0.003651
7 1.026023 0.698944 -0.093990

8 2.974468 -0.500640 -0.062909
8 -0.881965 1.957953 -0.079096
1 1.591274 1.565836 -0.098002
6 -2.595988 -0.379477 0.051421
8 -3.072940 0.330614 1.186352
1 -2.672398 1.217233 1.175757
8 -3.152189 0.145200 -1.147299
1 -2.776609 1.029508 -1.295814
1 -2.990141 -1.392590 0.143439
6 1.698036 -2.919825 0.071198
1 2.357627 -3.018312 -0.792458
1 2.290867 -2.964107 0.987097
1 0.967897 -3.727583 0.067301
8 2.649894 3.042663 -0.016201
1 2.380313 3.582588 0.742254
1 3.525849 2.701702 0.220771
1m5dhmU/1m5dhmUaav.xyz
6 -2.114483 -0.562043 -0.287120
6 0.273401 -1.199432 -0.396497
6 0.572633 -0.061790 0.439022
6 -0.446592 0.741451 0.843156
1 -0.271190 1.613291 1.463318
7 -1.755402 0.515496 0.504140
7 -1.069064 -1.360265 -0.705668
8 -3.286077 -0.798198 -0.599800
8 1.123118 -2.001804 -0.829390
1 -1.323592 -2.154571 -1.287875
6 2.009331 0.216092 0.815662
8 2.587529 -0.841742 1.557820
1 2.483868 -1.664810 1.049845
8 2.802573 0.520295 -0.334239
1 2.776136 -0.244547 -0.935738
1 2.065321 1.101704 1.449836
6 -2.839224 1.375684 1.007027
1 -3.400793 1.789833 0.168227
1 -3.509530 0.792668 1.641470

1 -2.395901 2.184352 1.585589
8 1.314845 2.526426 -1.736728
1 1.864651 1.856033 -1.283133
1 0.415991 2.339847 -1.429641
1m5dhmU/1m5dhmUacs.xyz
6 2.096145 -0.580014 -0.257606
6 -0.308999 -1.154599 -0.349778
6 -0.575185 0.006079 0.465020
6 0.466577 0.792261 0.845000
1 0.314788 1.684715 1.441797
7 1.767897 0.525349 0.508321
7 1.028798 -1.360630 -0.653057
8 3.259799 -0.853421 -0.569636
8 -1.180544 -1.937760 -0.774628
1 1.259329 -2.170039 -1.224302
6 -2.002594 0.341353 0.833184
8 -2.821768 0.477242 -0.328129
1 -2.787517 -0.364028 -0.818697
8 -2.562099 -0.592243 1.740950
1 -2.464316 -1.487123 1.374838
1 -2.041449 1.308826 1.335280
6 2.873844 1.377566 0.976180
1 3.540931 0.798584 1.617586
1 3.433043 1.758833 0.120284
1 2.452786 2.209050 1.538687
8 -1.243996 2.133813 -2.093727
1 -0.361320 1.984727 -1.724639
1 -1.832996 1.606491 -1.518320
1m5dhmU/1m5dhmUacp.xyz
6 1.467908 -0.434736 -0.600164
6 -0.891378 -1.049965 -0.193281
6 -1.172994 0.349033 0.025251
6 -0.157887 1.246080 -0.080745
1 -0.320517 2.307213 0.071052
7 1.130384 0.886205 -0.386237
7 0.432234 -1.336432 -0.499652

8 2.625763 -0.793454 -0.860938
8 -1.734242 -1.964573 -0.122977
1 0.676801 -2.311982 -0.652958
6 -2.586326 0.775387 0.352524
8 -3.477124 0.519811 -0.725163
1 -3.422369 -0.423375 -0.953814
8 -3.053906 0.182903 1.555030
1 -2.975987 -0.782781 1.469296
1 -2.621154 1.853193 0.517359
6 2.202195 1.890152 -0.501585
1 2.989970 1.676054 0.221660
1 2.618428 1.868049 -1.510263
1 1.776845 2.872003 -0.301494
8 4.280522 -0.501109 1.515172
1 3.755685 -0.550225 0.694459
1 4.286762 0.439102 1.744937
1m5fC/1m5fCaav.xyz
6 1.412389 -0.377155 -0.096379
6 -0.612857 0.812579 -0.088957
6 -1.376348 -0.411132 0.014548
6 -0.650293 -1.585658 0.058818
1 -1.149174 -2.546051 0.140205
7 0.687904 -1.599405 0.001986
7 0.724778 0.788798 -0.147468
8 2.653261 -0.430298 -0.133148
7 -1.223859 1.998533 -0.126991
1 -0.671756 2.843523 -0.202134
1 -2.235791 2.042428 -0.089968
6 -2.818668 -0.489696 0.077061
1 -3.229378 -1.510793 0.157557
8 -3.586356 0.484700 0.047513
6 1.456031 -2.853149 0.069120
1 2.101734 -2.933057 -0.806163
1 2.068226 -2.859479 0.972668
1 0.758293 -3.689463 0.089943
8 2.375491 3.049443 0.213115

1 3.250190 2.661723 0.359201
1 1.785194 2.270192 0.060899
1m5fC/1m5fCabo.xyz
6 -1.720138 0.766494 0.097092
6 0.580170 1.071741 -0.176906
6 0.757192 -0.300006 -0.604626
6 -0.381696 -1.081976 -0.641074
1 -0.336072 -2.124416 -0.940377
7 -1.583372 -0.596339 -0.307149
7 -0.623425 1.555458 0.148672
8 -2.862149 1.166781 0.389362
7 1.633813 1.890725 -0.095680
1 1.499940 2.848285 0.201511
1 2.556101 1.548061 -0.339306
6 2.022352 -0.914078 -0.936922
1 1.966022 -1.979551 -1.219157
8 3.122794 -0.339877 -0.919605
6 -2.798544 -1.423786 -0.367134
1 -3.269256 -1.455943 0.616365
1 -3.498122 -0.995558 -1.086967
1 -2.521912 -2.431467 -0.676083
8 1.496792 -1.106016 2.635676
1 1.691026 -0.235502 2.257853
1 1.107650 -1.600253 1.899207
1m5fC/1m5fCadj.xyz
6 -1.590545 -0.854867 0.046500
6 0.737217 -1.058446 0.020312
6 0.889393 0.245775 -0.592191
6 -0.277980 0.941661 -0.848716
1 -0.255805 1.931386 -1.293752
7 -1.480570 0.434923 -0.556808
7 -0.467934 -1.559977 0.312098
8 -2.734477 -1.272697 0.304036
7 1.816177 -1.791398 0.312872
1 1.702804 -2.699449 0.743650
1 2.739855 -1.430639 0.103749

6 2.150194 0.870330 -0.925277
1 2.071516 1.875977 -1.372906
8 3.268339 0.363799 -0.742227
6 -2.717578 1.187634 -0.812443
1 -3.369322 0.608580 -1.468266
1 -3.232223 1.376950 0.131043
1 -2.458353 2.132204 -1.289260
8 0.332857 1.654938 2.626536
1 0.931448 1.351305 3.324373
1 0.718406 1.308013 1.808361
1m5fC/1m5fCacr.xyz
6 2.327596 0.432592 0.036361
6 0.124045 1.210866 -0.017293
6 -0.399236 -0.139619 -0.046927
6 0.536219 -1.159160 -0.042785
1 0.232315 -2.200604 -0.065458
7 1.849367 -0.913084 -0.009220
7 1.439246 1.450905 0.023942
8 3.559197 0.603535 0.086774
7 -0.708157 2.256753 -0.029189
1 -0.330508 3.194946 -0.002962
1 -1.709555 2.104171 -0.053455
6 -1.794098 -0.494915 -0.070537
1 -2.011306 -1.575341 -0.081895
8 -2.736937 0.318476 -0.074857
6 2.837158 -2.002348 -0.000212
1 3.507509 -1.893119 -0.853836
1 3.417645 -1.961447 0.923004
1 2.307040 -2.951921 -0.063647
8 -5.224140 -0.954083 0.027254
1 -4.366692 -0.475151 0.020041
1 -5.684689 -0.631476 0.814452
1m5fC/1m5fCace.xyz
6 -1.199914 0.252187 -0.161038
6 0.795375 -0.956512 -0.103459
6 1.573631 0.255077 0.038396

6 0.862972 1.438580 0.072249
1 1.368510 2.392800 0.180558
7 -0.472361 1.466599 -0.027644
7 -0.540069 -0.922901 -0.198627
8 -2.444124 0.339288 -0.240396
7 1.399601 -2.147102 -0.141654
1 0.843687 -2.985309 -0.250652
1 2.409813 -2.200428 -0.078525
6 3.014345 0.315698 0.146177
1 3.434405 1.330922 0.251356
8 3.771774 -0.666950 0.128331
6 -1.227671 2.728686 0.034057
1 -1.853130 2.824402 -0.854009
1 -1.859628 2.733857 0.923950
1 -0.520055 3.555675 0.078383
8 -4.124103 -1.789436 0.319706
1 -3.466766 -1.090495 0.101134
1 -3.607840 -2.603118 0.403866
1m5fC/1m5fCabm.xyz
6 1.493038 0.232307 -0.126931
6 -0.657939 -0.710331 -0.123761
6 -1.271928 0.590767 0.023267
6 -0.412614 1.670585 0.097841
1 -0.791292 2.681397 0.213103
7 0.916800 1.526464 0.028388
7 0.673401 -0.843768 -0.196975
8 2.730874 0.146682 -0.193696
7 -1.406289 -1.814127 -0.188448
1 -0.959104 -2.716852 -0.287400
1 -2.416195 -1.737255 -0.148742
6 -2.695095 0.834581 0.093043
1 -2.985290 1.893228 0.203877
8 -3.571319 -0.042143 0.032897
6 1.826372 2.679247 0.110942
1 2.486359 2.561775 0.971877
1 2.427306 2.737828 -0.797698

1 1.232951 3.585676 0.220127
8 1.735449 -3.441945 0.222088
1 1.409818 -2.522972 0.061325
1 1.457028 -3.646486 1.126065
1m5fC/1m5fCabb.xyz
6 -1.049200 0.496023 -0.490649
6 1.211704 0.938009 -0.115001
6 1.500309 -0.467437 0.069913
6 0.429686 -1.332154 -0.045897
1 0.557680 -2.402649 0.080445
7 -0.806767 -0.895883 -0.319804
7 -0.026861 1.368308 -0.389374
8 -2.222648 0.852495 -0.732585
7 2.185997 1.845966 -0.010489
1 1.971012 2.826747 -0.133197
1 3.131605 1.546889 0.198572
6 2.803354 -1.021554 0.365074
1 2.832812 -2.119618 0.470766
8 3.845198 -0.361458 0.500630
6 -1.945121 -1.823671 -0.423484
1 -2.641782 -1.643487 0.396513
1 -2.456029 -1.669621 -1.374232
1 -1.567553 -2.843785 -0.367809
8 -4.245639 0.440280 1.186602
1 -3.542885 0.549457 0.512871
1 -4.471115 1.341502 1.456832
1m5fC/1m5fCaaq.xyz
6 2.325342 0.422560 0.044017
6 0.128281 1.218511 -0.016533
6 -0.405315 -0.127614 -0.059242
6 0.522051 -1.154444 -0.058326
1 0.210297 -2.193346 -0.090358
7 1.836916 -0.918964 -0.015703
7 1.445040 1.447761 0.034281
8 3.557872 0.583536 0.103871
7 -0.695625 2.270960 -0.025631

1 -0.310971 3.205895 0.012318
1 -1.698170 2.126611 -0.053555
6 -1.802886 -0.471345 -0.087388
1 -2.029190 -1.550017 -0.106814
8 -2.738853 0.349797 -0.085939
6 2.816529 -2.015643 -0.009353
1 3.490557 -1.907176 -0.860199
1 3.394304 -1.983759 0.915896
1 2.279739 -2.960996 -0.079209
8 -5.255985 -0.854508 0.108088
1 -4.371159 -0.433366 0.040638
1 -5.078020 -1.764884 0.383451
1m5fC/1m5fCadc.xyz
6 2.327743 0.428849 0.023670
6 0.125904 1.212808 -0.016065
6 -0.401184 -0.136526 -0.033364
6 0.531550 -1.158563 -0.029953
1 0.224848 -2.199340 -0.043201
7 1.845615 -0.915808 -0.009070
7 1.442058 1.449466 0.012282
8 3.560179 0.596808 0.062104
7 -0.703552 2.260850 -0.027091
1 -0.323245 3.198145 -0.008038
1 -1.705596 2.110955 -0.039732
6 -1.797171 -0.487746 -0.042654
1 -2.018236 -1.567520 -0.045923
8 -2.737270 0.328792 -0.044004
6 2.830672 -2.007584 -0.000535
1 3.497100 -1.903153 -0.857784
1 3.415684 -1.964741 0.919760
1 2.297812 -2.955997 -0.057907
8 -5.251194 -0.871097 0.166889
1 -5.385939 -1.374136 -0.648639
1 -4.370096 -0.449222 0.062122
1m5fC/1m5fCaac.xyz
6 1.050763 -0.497731 -0.497238

6 -1.210411 -0.936560 -0.119262
6 -1.496024 0.468811 0.070670
6 -0.424320 1.332027 -0.046498
1 -0.550308 2.402460 0.082439
7 0.810652 0.894422 -0.324816
7 0.026976 -1.368381 -0.396557
8 2.223516 -0.855962 -0.740142
7 -2.186338 -1.842980 -0.016683
1 -1.973423 -2.823705 -0.143390
1 -3.131083 -1.542930 0.194754
6 -2.797095 1.024398 0.371838
1 -2.824175 2.122201 0.480852
8 -3.839719 0.365916 0.509241
6 1.949783 1.820942 -0.431464
1 2.649236 1.638665 0.385608
1 2.456817 1.667393 -1.384395
1 1.573567 2.841416 -0.373312
8 4.237455 -0.446590 1.191588
1 4.297210 -1.305033 1.633738
1 3.536953 -0.560123 0.516336
1m5fC/1m5fCacl.xyz
6 1.045873 0.499964 -0.482313
6 -1.215854 0.939419 -0.108408
6 -1.504764 -0.467148 0.067386
6 -0.433635 -1.331023 -0.050342
1 -0.561686 -2.402248 0.069452
7 0.803338 -0.892823 -0.318405
7 0.023146 1.371569 -0.377797
8 2.219522 0.857548 -0.721114
7 -2.190634 1.846493 -0.000285
1 -1.975553 2.828129 -0.115732
1 -3.136414 1.545754 0.205710
6 -2.808506 -1.023165 0.355382
1 -2.838002 -2.121812 0.454820
8 -3.850980 -0.364063 0.491190
6 1.942144 -1.819326 -0.426855

1 2.443592 -1.670917 -1.383691
1 2.646702 -1.631336 0.384364
1 1.566865 -2.839667 -0.360589
8 4.218297 0.477152 1.239916
1 3.543638 0.566715 0.535393
1 5.013647 0.891277 0.876654
1m5fC/1m5fCaas.xyz
6 -2.328531 0.433885 0.015195
6 -0.123512 1.209478 -0.009279
6 0.398615 -0.141806 -0.021873
6 -0.538004 -1.160327 -0.023454
1 -0.235048 -2.202233 -0.033608
7 -1.851243 -0.912588 -0.011550
7 -1.438948 1.451199 0.009008
8 -3.560597 0.606478 0.043518
7 0.709913 2.254428 -0.015167
1 0.332871 3.193135 -0.001396
1 1.711419 2.100714 -0.021175
6 1.793327 -0.498440 -0.024396
1 2.010088 -1.578998 -0.029035
8 2.736689 0.314367 -0.019544
6 -2.840221 -2.000828 -0.006911
1 -3.429079 -1.955605 0.910828
1 -3.502446 -1.894273 -0.867122
1 -2.310410 -2.951087 -0.061839
8 5.232733 -0.929749 0.138419
1 5.676986 -0.770971 -0.706472
1 4.366922 -0.472502 0.058939
1m5fC/1m5fCaad.xyz
6 1.159608 -1.283294 -0.003891
6 -0.732470 0.092346 -0.007466
6 0.085657 1.288752 0.000321
6 1.453993 1.096588 0.005150
1 2.139581 1.938531 0.010860
7 1.996816 -0.127051 0.003001
7 -0.181628 -1.129955 -0.009357

8 1.719388 -2.397125 -0.004684
7 -2.063795 0.181150 -0.013123
1 -2.635851 -0.666256 -0.013968
1 -2.494421 1.098169 -0.010064
6 -0.413145 2.644736 0.003059
1 0.363595 3.428968 0.009197
8 -1.612325 2.967443 -0.001195
6 3.453968 -0.326850 0.007701
1 3.744811 -0.888166 0.897116
1 3.751068 -0.882979 -0.882909
1 3.939238 0.648316 0.012318
8 -3.837691 -2.167873 -0.000875
1 -3.703362 -2.648241 0.829791
1 -3.565046 -2.790584 -0.691209
1m5fC/1m5fCaco.xyz
6 2.327977 0.429521 0.031918
6 0.125960 1.212475 -0.016582
6 -0.400216 -0.136922 -0.045681
6 0.533079 -1.158443 -0.043791
1 0.226987 -2.199268 -0.065416
7 1.846835 -0.915142 -0.013338
7 1.441759 1.449694 0.021560
8 3.560018 0.597883 0.080095
7 -0.704069 2.260104 -0.026267
1 -0.324474 3.197451 0.002061
1 -1.705938 2.109651 -0.044477
6 -1.795898 -0.489249 -0.061774
1 -2.015646 -1.569187 -0.073069
8 -2.737001 0.326178 -0.059681
6 2.832400 -2.006454 -0.005453
1 3.502568 -1.898087 -0.859316
1 3.413389 -1.967276 0.917534
1 2.300332 -2.954914 -0.069188
8 -5.226940 -0.920331 0.201259
1 -5.629715 -0.953429 -0.677869
1 -4.367593 -0.462955 0.070849

1m5fC/1m5fCaau.xyz
6 -1.549770 -1.085242 0.159536
6 0.780793 -1.258032 0.213006
6 0.936974 0.042410 -0.404011
6 -0.229177 0.710855 -0.723014
1 -0.205196 1.690423 -1.189517
7 -1.435102 0.193612 -0.464663
7 -0.427393 -1.774884 0.464441
8 -2.696086 -1.509468 0.396833
7 1.858453 -1.976594 0.544807
1 1.742601 -2.878475 0.987618
1 2.783750 -1.596857 0.381993
6 2.197002 0.693920 -0.680121
1 2.116527 1.692988 -1.142394
8 3.316963 0.218803 -0.433183
6 -2.666521 0.947946 -0.740201
1 -3.331360 0.350326 -1.364987
1 -3.169573 1.183752 0.199537
1 -2.402217 1.868279 -1.259807
8 0.083366 3.359673 1.355787
1 0.776732 2.740573 1.082185
1 -0.732731 2.990850 0.986612
1m5fC/1m5fCadt.xyz
6 1.201272 -0.255688 -0.156395
6 -0.791767 0.956572 -0.098678
6 -1.572634 -0.253938 0.037917
6 -0.864320 -1.438928 0.068649
1 -1.371846 -2.392605 0.172241
7 0.471129 -1.469129 -0.028953
7 0.543767 0.920705 -0.192075
8 2.445640 -0.345087 -0.232763
7 -1.393534 2.148472 -0.133744
1 -0.835655 2.986025 -0.237660
1 -2.403674 2.203642 -0.071232
6 -3.013749 -0.312188 0.142065
1 -3.435966 -1.326937 0.243145

8 -3.769251 0.671954 0.125337
6 1.223810 -2.732977 0.027970
1 1.849495 -2.826199 -0.860233
1 1.855347 -2.743198 0.918103
1 0.514394 -3.558614 0.068401
8 4.099252 1.796656 0.358238
1 3.714323 2.607229 -0.003159
1 3.463256 1.085019 0.118133
1m5fC/1m5fCaae.xyz
6 1.155302 1.394417 -0.107176
6 -1.141654 0.994874 -0.275935
6 -1.010187 -0.296479 0.364704
6 0.266897 -0.664620 0.741332
1 0.455996 -1.616955 1.227105
7 1.323163 0.130338 0.532753
7 -0.081859 1.783633 -0.488741
8 2.174647 2.086373 -0.288036
7 -2.342267 1.429219 -0.672994
1 -2.424364 2.334019 -1.117797
1 -3.160931 0.851825 -0.519522
6 -2.094959 -1.212605 0.632633
1 -1.801643 -2.152627 1.130752
8 -3.286787 -1.013138 0.347528
6 2.682394 -0.276100 0.917845
1 3.317020 -0.317060 0.031030
1 3.095889 0.446776 1.622982
1 2.633437 -1.259112 1.385011
8 1.415666 -2.996684 -1.348097
1 2.027505 -3.287877 -2.039584
1 1.988865 -2.650648 -0.648683
1m5fC/1m5fCabk.xyz
6 1.997205 -0.837727 0.091224
6 -0.295830 -0.813888 -0.364593
6 -0.345659 0.631078 -0.271015
6 0.855262 1.268094 -0.014370
1 0.912784 2.349462 0.058895

7 1.994228 0.589501 0.156772
7 0.843760 -1.488760 -0.176504
8 3.080247 -1.419936 0.283665
7 -1.398949 -1.511525 -0.651555
1 -1.346863 -2.518977 -0.724052
1 -2.278933 -1.033528 -0.798197
6 -1.523905 1.445276 -0.435786
1 -1.359578 2.532551 -0.349748
8 -2.669514 1.017133 -0.663296
6 3.269373 1.276564 0.413649
1 3.669644 0.963511 1.379013
1 3.982922 1.023917 -0.372068
1 3.088871 2.350811 0.418646
8 -4.347568 -0.437054 1.219946
1 -3.801081 0.064229 0.588715
1 -5.185049 0.045991 1.260629
1m5fC/1m5fCaam.xyz
6 1.039063 -0.528479 -0.460870
6 -1.228800 -0.937716 -0.090162
6 -1.503787 0.474574 0.059501
6 -0.422486 1.324913 -0.063865
1 -0.539935 2.399195 0.038038
7 0.811926 0.868924 -0.314740
7 0.006640 -1.387186 -0.347004
8 2.208403 -0.903213 -0.694055
7 -2.213187 -1.832817 0.029316
1 -2.006978 -2.818443 -0.067211
1 -3.156314 -1.518977 0.227934
6 -2.803838 1.048506 0.328113
1 -2.822943 2.148955 0.407568
8 -3.853770 0.402358 0.468263
6 1.960912 1.782328 -0.425343
1 2.646730 1.612085 0.405947
1 2.481212 1.603100 -1.366539
1 1.593961 2.807310 -0.394902
8 4.313531 -0.565646 1.131196

1 3.569110 -0.599689 0.494551
1 4.405814 0.371271 1.354138
1m5fC/1m5fCabx.xyz
6 0.496974 1.329327 -0.011224
6 -1.762672 0.732143 0.008322
6 -1.437642 -0.678709 -0.003110
6 -0.092780 -0.995287 -0.017558
1 0.241883 -2.027490 -0.026551
7 0.856165 -0.051439 -0.021330
7 -0.810753 1.672553 0.004425
8 1.421540 2.164068 -0.016789
7 -3.040041 1.126987 0.023585
1 -3.257480 2.114896 0.031198
1 -3.779235 0.433757 0.027571
6 -2.397968 -1.758828 -0.000362
1 -1.964324 -2.773605 -0.014018
8 -3.631294 -1.617973 0.015879
6 2.286035 -0.394461 -0.034578
1 2.755801 0.026601 -0.924346
1 2.765699 0.012914 0.857220
1 2.383445 -1.479208 -0.043793
8 5.665742 -0.987039 -0.060511
1 6.527189 -0.967273 0.380944
1 5.089829 -0.454854 0.507451
1m5fU/1m5fUadq.xyz
6 1.347673 -0.598090 -0.015264
6 -0.577511 0.958450 -0.014308
6 -1.454104 -0.205164 -0.004426
6 -0.882834 -1.459336 0.000575
1 -1.503071 -2.350226 0.012154
7 0.448983 -1.673203 -0.007587
7 0.780407 0.649787 -0.023775
8 2.566103 -0.777604 -0.014086
8 -0.931947 2.144432 -0.013607
1 1.445316 1.443606 -0.026786
6 -2.905889 -0.127398 0.007943

1 -3.414650 -1.108623 0.018395
8 -3.577570 0.908589 0.008382
6 1.023727 -3.029475 0.028082
1 1.659490 -3.179848 -0.845424
1 1.617706 -3.147579 0.935759
1 0.207044 -3.749228 0.021336
8 2.681401 2.776710 -0.061729
1 3.538665 2.325470 -0.028765
1 2.633511 3.279388 0.765563
1m5fU/1m5fUadi.xyz
6 -1.262541 -0.428354 0.024205
6 0.740430 1.041461 0.013482
6 1.555202 -0.165088 -0.002834
6 0.922762 -1.387888 -0.008177
1 1.497532 -2.308378 -0.023848
7 -0.418888 -1.539800 0.006518
7 -0.634550 0.791820 0.027985
8 -2.490247 -0.549476 0.035258
8 1.144827 2.208490 0.014231
1 -1.251937 1.604727 0.037414
6 3.011095 -0.162023 -0.016232
1 3.467914 -1.168207 -0.032017
8 3.731895 0.838830 -0.011491
6 -1.056780 -2.869361 -0.022469
1 -1.691620 -2.987049 0.856596
1 -1.661601 -2.960707 -0.925940
1 -0.273819 -3.625745 -0.020026
8 -3.512203 2.123371 0.057376
1 -3.772145 2.384295 -0.837757
1 -3.395445 1.154719 0.013212
1m5fU/1m5fUaax.xyz
6 -1.304367 0.447349 0.016555
6 0.700943 -1.013949 0.014520
6 1.514100 0.194468 -0.001213
6 0.878339 1.416096 -0.005707
1 1.450808 2.338121 -0.018317

7 -0.463106 1.563621 0.005378
7 -0.672490 -0.770926 0.027161
8 -2.531932 0.563920 0.016680
8 1.112031 -2.180112 0.016375
1 -1.300916 -1.583304 0.033878
6 2.969185 0.196472 -0.014845
1 3.422434 1.204378 -0.029558
8 3.695027 -0.801336 -0.011887
6 -1.105375 2.890581 -0.016137
1 -1.713572 2.984374 -0.917076
1 -1.738223 3.002182 0.865323
1 -0.325500 3.650221 -0.012471
8 -3.150321 -2.311352 0.067499
1 -3.370217 -2.725971 -0.779552
1 -3.391035 -1.373923 -0.038380
1m5fU/1m5fUabe.xyz
6 -1.255045 1.195062 -0.054584
6 0.881275 -0.072368 -0.072052
6 0.097869 -1.293455 0.008153
6 -1.275509 -1.192172 0.061001
1 -1.889715 -2.084903 0.123528
7 -1.943200 -0.020336 0.035270
7 0.115926 1.090363 -0.109533
8 -1.839859 2.275087 -0.081747
8 2.117552 0.007384 -0.107858
1 0.620600 1.973307 -0.159541
6 0.675707 -2.629786 0.027047
1 -0.068492 -3.444293 0.093559
8 1.877926 -2.899995 -0.028076
6 -3.415743 0.046524 0.084722
1 -3.724495 0.607167 0.968272
1 -3.784676 0.543847 -0.813289
1 -3.806007 -0.968200 0.134932
8 3.382292 2.518910 0.254426
1 2.975762 1.643977 0.085457
1 3.913336 2.704989 -0.532766

1m5fU/1m5fUacy.xyz
6 -2.350680 -0.344620 0.054582
6 -0.051706 -1.290150 -0.028276
6 0.447971 0.077401 -0.081557
6 -0.460474 1.115195 -0.070986
1 -0.123609 2.146100 -0.107920
7 -1.795595 0.939968 -0.012106
7 -1.443440 -1.376783 0.041156
8 -3.564282 -0.524210 0.120834
8 0.621955 -2.324968 -0.034791
1 -1.842078 -2.312054 0.085997
6 1.855358 0.421390 -0.121344
1 2.066208 1.504388 -0.141700
8 2.796206 -0.382997 -0.126878
6 -2.734114 2.076276 0.004444
1 -3.417700 1.994859 -0.841540
1 -3.301126 2.064495 0.936535
1 -2.159691 2.997706 -0.068563
8 5.277067 0.907243 0.103217
1 4.426916 0.420430 0.039591
1 5.738627 0.508276 0.854017
1m5fU/1m5fUaal.xyz
6 -1.667336 -0.849304 0.157050
6 0.778569 -1.293735 0.260632
6 0.980217 -0.035677 -0.443013
6 -0.124112 0.712027 -0.789933
1 -0.007883 1.654108 -1.315989
7 -1.393076 0.348633 -0.512503
7 -0.564243 -1.596460 0.496592
8 -2.816386 -1.200253 0.416681
8 1.654054 -2.076005 0.645611
1 -0.757015 -2.466976 0.986673
6 2.287066 0.497985 -0.793835
1 2.253187 1.467211 -1.323279
8 3.375689 -0.031045 -0.552291
6 -2.546969 1.174596 -0.913345

1 -3.177981 0.610216 -1.601526
1 -3.123960 1.448873 -0.029207
1 -2.173079 2.071943 -1.402726
8 0.366431 2.922673 1.535321
1 0.698554 3.442135 2.281717
1 1.106500 2.348959 1.287793
1m5fU/1m5fUadg.xyz
6 -1.270460 -1.183804 -0.069620
6 0.879437 0.059426 -0.111472
6 0.112439 1.287896 0.005311
6 -1.260907 1.201736 0.080478
1 -1.862965 2.100458 0.168739
7 -1.942661 0.038310 0.047664
7 0.100605 -1.094132 -0.147031
8 -1.868689 -2.256278 -0.100634
8 2.113657 -0.033173 -0.178766
1 0.594266 -1.981492 -0.222283
6 0.707240 2.616413 0.044068
1 -0.025327 3.438371 0.139918
8 1.911916 2.872751 -0.022241
6 -3.414515 -0.011539 0.123476
1 -3.806875 -0.490202 -0.774720
1 -3.714399 -0.581685 1.003981
1 -3.791664 1.006856 0.196319
8 3.390310 -2.541661 0.133174
1 2.961371 -1.666993 0.031867
1 3.643031 -2.588443 1.066039
1m5fU/1m5fUada.xyz
6 -1.254714 1.190697 -0.055354
6 0.884434 -0.071936 -0.075562
6 0.103865 -1.294675 0.007652
6 -1.269664 -1.196423 0.062689
1 -1.881691 -2.090487 0.127310
7 -1.940041 -0.026146 0.036585
7 0.116389 1.089050 -0.112440
8 -1.842018 2.269378 -0.082528

8 2.120490 0.010515 -0.114291
1 0.619051 1.973152 -0.162572
6 0.684694 -2.629703 0.027794
1 -0.057533 -3.445749 0.097204
8 1.887394 -2.897365 -0.029045
6 -3.412637 0.037463 0.087884
1 -3.721508 0.598672 0.971030
1 -3.783877 0.532714 -0.810325
1 -3.800678 -0.978025 0.140012
8 3.372157 2.526333 0.268284
1 3.701798 2.833823 -0.587841
1 2.969946 1.652146 0.086196
1m5fU/1m5fUaat.xyz
6 -1.096384 -0.445251 -0.585225
6 1.280471 -1.009816 -0.127611
6 1.519434 0.412109 0.073411
6 0.465615 1.286141 -0.074154
1 0.609962 2.352937 0.065333
7 -0.789912 0.903063 -0.392230
7 -0.043392 -1.314257 -0.456436
8 -2.240161 -0.821629 -0.850738
8 2.107346 -1.922440 -0.037021
1 -0.265867 -2.297601 -0.594810
6 2.814376 0.974957 0.429459
1 2.818342 2.075284 0.531318
8 3.854564 0.339007 0.617483
6 -1.892549 1.873452 -0.532908
1 -2.308856 1.803921 -1.538783
1 -2.667178 1.653427 0.202176
1 -1.493698 2.872302 -0.366433
8 -3.867296 -0.488465 1.577567
1 -3.428831 -0.548262 0.710073
1 -4.812148 -0.434989 1.375622
1m5fU/1m5fUaaq.xyz
6 -1.909224 0.856362 -0.012562
6 0.550068 0.684164 -0.319931

6 0.421344 -0.752662 -0.141774
6 -0.834012 -1.276961 0.080937
1 -0.970662 -2.345479 0.213976
7 -1.957026 -0.532706 0.148918
7 -0.654477 1.376569 -0.231051
8 -2.919470 1.553502 0.037043
8 1.592321 1.318242 -0.537571
1 -0.612584 2.385928 -0.354274
6 1.533923 -1.689019 -0.216220
1 1.250044 -2.743127 -0.044506
8 2.709586 -1.401493 -0.451074
6 -3.282480 -1.137682 0.373840
1 -3.724031 -0.724317 1.281569
1 -3.928987 -0.923396 -0.478355
1 -3.153470 -2.213117 0.481430
8 4.008482 0.967181 0.906444
1 4.230488 0.025753 0.867899
1 3.184650 1.026056 0.382709
1m5fU/1m5fUaco.xyz
6 1.265095 -1.199448 -0.057377
6 -0.872322 0.066322 -0.085105
6 -0.090515 1.287606 0.008136
6 1.282576 1.187320 0.068869
1 1.895515 2.080279 0.139704
7 1.951524 0.016217 0.041355
7 -0.105699 -1.095680 -0.120770
8 1.851020 -2.278817 -0.085116
8 -2.107974 -0.014081 -0.133375
1 -0.608247 -1.978900 -0.181731
6 -0.669820 2.623194 0.031959
1 0.073074 3.438087 0.107483
8 -1.872072 2.892405 -0.027556
6 3.423803 -0.049392 0.098335
1 3.728779 -0.612594 0.981564
1 3.797912 -0.543329 -0.799394
1 3.812881 0.965523 0.153841

8 -3.408834 -2.513516 0.183652
1 -2.990585 -1.636343 0.062603
1 -4.360128 -2.338018 0.202095
1m5fU/1m5fUaas.xyz
6 -2.347874 0.343551 -0.065713
6 -0.049942 1.290919 0.026598
6 0.449712 -0.075843 0.096910
6 -0.458208 -1.114102 0.088093
1 -0.120948 -2.144402 0.137198
7 -1.792769 -0.940206 0.016608
7 -1.441128 1.376158 -0.055437
8 -3.561037 0.521886 -0.142472
8 0.623346 2.325988 0.028978
1 -1.839716 2.310793 -0.112105
6 1.856598 -0.418950 0.152670
1 2.067869 -1.501673 0.184126
8 2.797131 0.385839 0.160078
6 -2.730626 -2.077082 0.003442
1 -3.417680 -1.990932 0.846166
1 -3.293994 -2.071268 -0.930876
1 -2.156070 -2.997754 0.084328
8 5.303183 -0.839900 -0.128897
1 4.425945 -0.410719 -0.027576
1 5.352928 -1.087173 -1.062957
1m5fU/1m5fUaay.xyz
6 -2.348291 0.340361 0.063465
6 -0.052660 1.293286 -0.027859
6 0.450596 -0.072381 -0.093694
6 -0.454695 -1.112930 -0.084091
1 -0.114897 -2.142542 -0.130153
7 -1.789835 -0.942153 -0.015431
7 -1.444145 1.375239 0.052039
8 -3.561991 0.515812 0.138349
8 0.618004 2.330067 -0.032097
1 -1.845169 2.308996 0.105910
6 1.858499 -0.412105 -0.144882

1 2.072385 -1.494372 -0.172671
8 2.797106 0.394931 -0.152030
6 -2.724969 -2.081221 -0.001129
1 -3.288705 -2.075593 0.932979
1 -3.411936 -1.997742 -0.844188
1 -2.148337 -3.000709 -0.080636
8 5.284131 -0.875848 0.116301
1 4.425411 -0.407249 0.033764
1 5.480233 -0.872137 1.063716
1m5fU/1m5fUacg.xyz
6 -2.347516 -0.338645 0.065513
6 -0.053195 -1.294654 -0.026672
6 0.451651 0.070219 -0.096689
6 -0.452339 1.111907 -0.088663
1 -0.111301 2.140971 -0.137994
7 -1.787569 0.942983 -0.017603
7 -1.444669 -1.374659 0.055550
8 -3.561324 -0.512353 0.142651
8 0.616180 -2.332266 -0.029465
1 -1.846746 -2.307779 0.112458
6 1.859885 0.408031 -0.150289
1 2.075139 1.489948 -0.182118
8 2.797482 -0.400186 -0.155096
6 -2.721307 2.083188 -0.004915
1 -3.408999 1.998891 -0.847308
1 -3.284415 2.080069 0.929575
1 -2.143705 3.001865 -0.086624
8 5.290685 0.855692 0.124506
1 4.425648 0.400144 0.034795
1 5.352797 1.090260 1.061109
1m5fU/1m5fUabc.xyz
6 1.068714 -0.476811 -0.501083
6 -1.321634 -1.015943 -0.083048
6 -1.557354 0.412663 0.069162
6 -0.494772 1.276413 -0.076621
1 -0.636261 2.347408 0.029302

7 0.765546 0.878156 -0.356536
7 0.007183 -1.335893 -0.375230
8 2.215169 -0.869915 -0.727420
8 -2.154479 -1.921994 0.017751
1 0.228559 -2.324114 -0.475598
6 -2.858783 0.993591 0.368736
1 -2.858845 2.096719 0.433232
8 -3.908329 0.369966 0.545545
6 1.877408 1.839618 -0.485291
1 2.588939 1.686679 0.327072
1 2.375746 1.690045 -1.443500
1 1.467513 2.846584 -0.434738
8 4.299883 -0.345465 1.209913
1 3.930254 -0.715914 2.024006
1 3.595577 -0.461180 0.545138
1m5fU/1m5fUabx.xyz
6 1.887312 0.885423 -0.030871
6 -0.565294 0.633235 -0.338088
6 -0.391236 -0.795939 -0.139638
6 0.878100 -1.278333 0.095223
1 1.045087 -2.340083 0.246965
7 1.977527 -0.498562 0.154282
7 0.618069 1.363752 -0.261795
8 2.875981 1.613663 0.011709
8 -1.628202 1.228908 -0.560791
1 0.545604 2.369444 -0.400175
6 -1.478962 -1.761742 -0.194726
1 -1.166666 -2.807103 -0.018840
8 -2.663927 -1.503584 -0.416598
6 3.318306 -1.054029 0.413182
1 3.973302 -0.831177 -0.430247
1 3.726547 -0.609732 1.321996
1 3.225877 -2.131500 0.537055
8 -4.085606 0.895372 0.867304
1 -4.130929 1.831768 1.106878
1 -3.263321 0.815486 0.345473

1m5fU/1m5fUadm.xyz
6 1.205985 0.386177 -0.042573
6 -0.807615 -1.071661 -0.026023
6 -1.613556 0.140191 0.008011
6 -0.973021 1.358625 0.015699
1 -1.541619 2.282615 0.043065
7 0.369515 1.502279 -0.012033
7 0.569608 -0.829286 -0.052073
8 2.433878 0.500446 -0.059455
8 -1.218048 -2.236046 -0.031917
1 1.176697 -1.646359 -0.078752
6 -3.069484 0.146390 0.034282
1 -3.519432 1.155270 0.065687
8 -3.796549 -0.849684 0.024496
6 1.015852 2.827832 0.022209
1 1.622501 2.911592 0.925194
1 1.649786 2.945522 -0.857433
1 0.237590 3.589004 0.024244
8 3.942943 -1.901511 0.115254
1 4.859195 -1.742330 -0.151696
1 3.494658 -1.037241 0.021774
1m5fU/1m5fUacl.xyz
6 -1.008023 -0.570745 -0.065227
6 1.437129 -1.015583 -0.002335
6 1.640087 0.426435 0.012539
6 0.536413 1.249357 -0.011912
1 0.652439 2.328747 -0.002673
7 -0.736944 0.799172 -0.052151
7 0.091991 -1.390816 -0.043018
8 -2.157390 -1.013404 -0.096685
8 2.309959 -1.889019 0.018062
1 -0.106050 -2.388884 -0.051097
6 2.948676 1.063280 0.052066
1 2.913951 2.167478 0.062979
8 4.038160 0.485069 0.071866
6 -1.890296 1.718528 -0.082764

1 -2.489421 1.520649 -0.972170
1 -2.496746 1.571984 0.811761
1 -1.514708 2.739731 -0.110138
8 -4.843421 -0.060063 0.154752
1 -5.306461 -0.904466 0.249838
1 -3.900700 -0.302027 0.079517
1m5fU/1m5fUaad.xyz
6 -1.927867 0.857306 0.016232
6 0.534239 0.705304 -0.307774
6 0.409990 -0.737693 -0.151534
6 -0.844868 -1.274972 0.044292
1 -0.977930 -2.347538 0.143850
7 -1.970665 -0.537921 0.131247
7 -0.676993 1.389768 -0.186006
8 -2.942621 1.546379 0.087232
8 1.568666 1.337946 -0.538185
1 -0.642864 2.401134 -0.292423
6 1.525961 -1.663028 -0.214361
1 1.240494 -2.727041 -0.146521
8 2.720378 -1.364604 -0.332170
6 -3.294622 -1.157083 0.322302
1 -3.749288 -0.773682 1.236600
1 -3.932994 -0.922446 -0.530742
1 -3.159490 -2.234335 0.399450
8 4.162074 0.791847 0.814295
1 3.580506 0.140869 0.372693
1 3.690201 1.628556 0.696082
1m5fU/1m5fUabb.xyz
6 -1.570997 0.828416 -0.019945
6 0.895983 1.109833 0.125672
6 1.026207 -0.195368 -0.506070
6 -0.119176 -0.884712 -0.840663
1 -0.058550 -1.863181 -1.306678
7 -1.366190 -0.414297 -0.629755
7 -0.427361 1.516486 0.307632
8 -2.698305 1.267826 0.197737

8 1.813559 1.848948 0.497471
1 -0.570082 2.421051 0.750966
6 2.302142 -0.831000 -0.795793
1 2.215204 -1.830081 -1.260090
8 3.417988 -0.354747 -0.570524
6 -2.567023 -1.172367 -1.024664
1 -3.180342 -1.369644 -0.144480
1 -3.141403 -0.592277 -1.748465
1 -2.248772 -2.112092 -1.471712
8 -0.006424 -1.851656 2.422873
1 0.024628 -0.917351 2.169918
1 -0.503176 -1.863530 3.253724
1m5hmC/1m5hmCacd.xyz
6 -1.134028 0.471505 -0.482721
6 1.085549 0.999626 0.004092
6 1.414697 -0.374662 0.267422
6 0.400370 -1.270796 0.096791
1 0.544342 -2.332581 0.264096
7 -0.851175 -0.883899 -0.274730
7 -0.147557 1.385571 -0.349701
8 -2.309805 0.795917 -0.800271
6 2.776284 -0.809035 0.734279
1 3.051465 -0.288391 1.657116
1 2.766234 -1.884987 0.941861
7 2.025233 1.956940 0.136176
1 1.795458 2.906423 -0.125381
1 3.001678 1.702562 0.193553
8 3.825918 -0.483704 -0.201339
1 3.637771 -0.940236 -1.035831
6 -1.933029 -1.865982 -0.435571
1 -2.701938 -1.708736 0.323054
1 -2.379901 -1.763044 -1.425594
1 -1.514364 -2.866120 -0.327600
8 -4.346907 0.355973 1.042172
1 -4.774782 1.218999 1.131201
1 -3.624181 0.496410 0.391067

1m5hmC/1m5hmCaas.xyz
6 1.491897 0.447898 0.015215
6 -0.478970 -0.814997 0.201678
6 -1.298552 0.364678 0.222342
6 -0.634049 1.551806 0.108516
1 -1.161804 2.499131 0.103852
7 0.721219 1.619235 0.004498
7 0.856543 -0.747857 0.113753
8 2.737306 0.538820 -0.074894
6 -2.793045 0.312192 0.375335
1 -3.070503 -0.230251 1.284699
1 -3.188553 1.331153 0.454216
7 -1.033019 -2.039105 0.290633
1 -0.440237 -2.852940 0.190001
1 -2.028544 -2.157401 0.164440
8 -3.445647 -0.401838 -0.696014
1 -3.247390 0.056042 -1.527330
6 1.413874 2.908015 -0.116555
1 2.096002 3.045203 0.725039
1 1.985274 2.937222 -1.046404
1 0.670880 3.704549 -0.118145
8 2.374115 -3.095149 -0.134368
1 3.165562 -2.867343 -0.642137
1 1.872983 -2.243500 -0.047395
1m5hmC/1m5hmCacv.xyz
6 -1.160627 -0.501973 -0.528214
6 1.108886 -0.972618 -0.263892
6 1.446418 0.421768 -0.173465
6 0.397773 1.292124 -0.242354
1 0.542073 2.364531 -0.172582
7 -0.884756 0.865873 -0.410342
7 -0.150018 -1.394778 -0.443063
8 -2.357652 -0.857339 -0.700048
6 2.861638 0.906393 -0.022781
1 3.489673 0.526460 -0.834723
1 2.880487 2.001309 -0.062957

7 2.073965 -1.909864 -0.186452
1 1.808673 -2.885578 -0.172954
1 2.998933 -1.663765 0.137068
8 3.491681 0.433670 1.186825
1 2.972027 0.753411 1.940207
6 -2.000320 1.820030 -0.480865
1 -2.535617 1.698246 -1.424114
1 -2.690647 1.647333 0.346821
1 -1.599927 2.831216 -0.415073
8 -4.050323 -0.548259 1.503183
1 -3.455763 -0.611263 0.724118
1 -4.276513 0.390572 1.562927
1m5hmC/1m5hmCadl.xyz
6 1.135950 -0.459788 -0.489567
6 -1.080986 -0.998005 -0.002033
6 -1.414732 0.373834 0.268066
6 -0.403150 1.274175 0.102828
1 -0.550660 2.334652 0.275181
7 0.849798 0.893022 -0.269660
7 0.153010 -1.378035 -0.359028
8 2.311403 -0.777596 -0.814684
6 -2.777502 0.801203 0.738036
1 -3.049459 0.276762 1.659682
1 -2.771389 1.876585 0.948702
7 -2.017271 -1.959204 0.126184
1 -1.784270 -2.906526 -0.140378
1 -2.994527 -1.708225 0.184700
8 -3.827196 0.474505 -0.197043
1 -3.641765 0.933723 -1.030669
6 1.928377 1.878254 -0.432584
1 2.726883 1.682211 0.284873
1 2.334406 1.817641 -1.444001
1 1.519645 2.874023 -0.264113
8 4.296206 -0.495037 1.120493
1 3.611738 -0.544195 0.417013
1 5.129587 -0.360153 0.647934

1m5hmC/1m5hmCacn.xyz
6 -1.076411 0.599782 -0.038312
6 1.217974 0.996910 0.124249
6 1.512133 -0.406109 0.229610
6 0.436833 -1.243150 0.167188
1 0.549106 -2.319981 0.226762
7 -0.836861 -0.777790 0.037488
7 -0.032461 1.458897 0.001421
8 -2.263370 1.003318 -0.150162
6 2.907736 -0.932913 0.412362
1 3.378690 -0.489595 1.295372
1 2.873574 -2.018590 0.557204
7 2.213882 1.904609 0.172186
1 1.991155 2.877422 0.008933
1 3.174354 1.612420 0.057170
8 3.782763 -0.594638 -0.684924
1 3.406226 -0.969676 -1.495983
6 -1.980256 -1.699471 -0.014533
1 -2.644630 -1.515060 0.831670
1 -2.532559 -1.556765 -0.944789
1 -1.607981 -2.722235 0.033892
8 -4.874396 0.076022 -0.052046
1 -5.345284 0.914653 -0.156737
1 -3.922606 0.319478 -0.077495
1m5hmC/1m5hmCacc.xyz
6 -1.175991 -0.480457 -0.558890
6 1.089650 -0.966802 -0.290763
6 1.433023 0.424004 -0.170922
6 0.389128 1.300706 -0.233107
1 0.537967 2.370985 -0.142933
7 -0.893752 0.883893 -0.419541
7 -0.170419 -1.379619 -0.483931
8 -2.374996 -0.825457 -0.739211
6 2.848579 0.899923 0.002587
1 3.483858 0.532216 -0.809396
1 2.871652 1.995371 -0.018246

7 2.049437 -1.910418 -0.227713
1 1.779042 -2.884789 -0.234421
1 2.975588 -1.675769 0.100709
8 3.464043 0.403588 1.210174
1 2.930072 0.700908 1.962771
6 -2.004462 1.843716 -0.485394
1 -2.527644 1.742720 -1.437892
1 -2.706610 1.656977 0.329223
1 -1.600926 2.851720 -0.394586
8 -3.937545 -0.609234 1.578600
1 -3.930049 0.325591 1.828216
1 -3.401373 -0.650639 0.757288
1m5hmC/1m5hmCacx.xyz
6 -1.134978 0.486414 -0.493005
6 1.085857 0.996906 0.006397
6 1.404289 -0.380863 0.264923
6 0.384177 -1.269038 0.087545
1 0.520038 -2.332422 0.251368
7 -0.863351 -0.871647 -0.286653
7 -0.142864 1.392860 -0.351839
8 -2.306590 0.819934 -0.816378
6 2.761256 -0.826629 0.734370
1 3.036978 -0.311360 1.660067
1 2.743385 -1.903267 0.937893
7 2.031189 1.947203 0.147591
1 1.808746 2.899799 -0.108811
1 3.005692 1.686692 0.209900
8 3.815858 -0.504756 -0.196793
1 3.626998 -0.956464 -1.033732
6 -1.951270 -1.845291 -0.455889
1 -2.728512 -1.675997 0.291639
1 -2.384883 -1.745125 -1.452168
1 -1.542799 -2.848253 -0.335968
8 -4.314628 0.532446 1.082693
1 -4.511171 -0.409043 1.187765
1 -3.602388 0.566538 0.406126

1m5hmC/1m5hmCacq.xyz
6 -1.134317 0.485207 -0.489922
6 1.086369 0.997471 0.008877
6 1.406771 -0.380456 0.264271
6 0.387510 -1.269425 0.086255
1 0.524632 -2.332917 0.248357
7 -0.860762 -0.872941 -0.286484
7 -0.143005 1.392509 -0.348055
8 -2.306456 0.818215 -0.811637
6 2.764859 -0.825697 0.730943
1 3.041381 -0.311929 1.657238
1 2.748343 -1.902738 0.932448
7 2.030447 1.948729 0.151890
1 1.806655 2.901651 -0.102123
1 3.005319 1.689526 0.213751
8 3.817774 -0.501018 -0.201126
1 3.627767 -0.950763 -1.038860
6 -1.948625 -1.847534 -0.450044
1 -2.711608 -1.693503 0.315576
1 -2.401866 -1.732859 -1.435749
1 -1.534878 -2.850918 -0.353838
8 -4.340233 0.531522 1.057130
1 -3.612484 0.563558 0.397019
1 -4.424106 -0.404338 1.287507
1m5hmC/1m5hmCabl.xyz
6 -2.376207 0.411051 -0.050624
6 -0.187135 1.211521 0.117274
6 0.354706 -0.118025 0.203501
6 -0.552174 -1.135140 0.128791
1 -0.248398 -2.175002 0.176031
7 -1.887057 -0.904585 0.005222
7 -1.499332 1.443512 -0.002239
8 -3.614407 0.584765 -0.153629
6 1.816746 -0.387672 0.406024
1 2.184861 0.108463 1.309197
1 1.986895 -1.464273 0.513316

7 0.632326 2.281311 0.178007
1 0.240454 3.203476 0.041092
1 1.630375 2.166126 0.070018
8 2.639231 0.143946 -0.662047
1 2.347440 -0.252745 -1.497949
6 -2.848360 -2.012681 -0.052415
1 -3.554926 -1.936124 0.776409
1 -3.399591 -1.980001 -0.994475
1 -2.301451 -2.952353 0.018026
8 5.203003 -0.768608 0.004500
1 4.998792 -1.523841 0.574278
1 4.325920 -0.430545 -0.278771
1m5hmC/1m5hmCabd.xyz
6 1.101896 0.582407 -0.291847
6 -1.192854 0.987027 -0.159317
6 -1.499993 -0.417048 -0.176328
6 -0.427113 -1.257924 -0.220446
1 -0.548119 -2.335445 -0.228286
7 0.853242 -0.796042 -0.275968
7 0.063300 1.445913 -0.225009
8 2.294114 0.981105 -0.365210
6 -2.909189 -0.940763 -0.188905
1 -3.457285 -0.544677 -1.049739
1 -2.892805 -2.033312 -0.270052
7 -2.187492 1.894992 -0.096895
1 -1.948767 2.873129 -0.002132
1 -3.119614 1.605073 0.165782
8 -3.679079 -0.530308 0.960442
1 -3.243593 -0.875966 1.754714
6 1.991312 -1.724780 -0.328243
1 2.625031 -1.485808 -1.183441
1 2.579351 -1.650088 0.588385
1 1.608542 -2.739795 -0.430453
8 4.636733 0.130461 0.856114
1 5.104822 0.965470 0.995230
1 3.790576 0.383792 0.424848

1m5hmC/1m5hmCabp.xyz
6 1.105197 0.575429 -0.309248
6 -1.187799 0.985560 -0.164974
6 -1.497840 -0.417974 -0.171880
6 -0.426977 -1.261350 -0.217714
1 -0.550217 -2.338638 -0.218396
7 0.853932 -0.802336 -0.284170
7 0.068918 1.441512 -0.240612
8 2.297866 0.971096 -0.392306
6 -2.908290 -0.938567 -0.174794
1 -3.458527 -0.547902 -1.036750
1 -2.894780 -2.031767 -0.247236
7 -2.180242 1.895767 -0.102075
1 -1.939449 2.874195 -0.016129
1 -3.111587 1.609048 0.166788
8 -3.673187 -0.517146 0.973951
1 -3.236682 -0.858784 1.769394
6 1.989794 -1.733772 -0.338294
1 2.621715 -1.497061 -1.195461
1 2.580600 -1.658954 0.576531
1 1.604477 -2.748032 -0.438400
8 4.623817 0.148963 0.874476
1 4.972508 0.985806 1.211952
1 3.781208 0.388214 0.428168
1m5hmC/1m5hmCaci.xyz
6 1.515148 -0.425386 0.004718
6 -0.481099 0.796135 -0.185272
6 -1.274203 -0.401229 -0.227382
6 -0.584737 -1.574708 -0.122219
1 -1.091324 -2.533415 -0.134891
7 0.770646 -1.613740 -0.007078
7 0.854619 0.757887 -0.083276
8 2.761465 -0.490621 0.102526
6 -2.768341 -0.380307 -0.390844
1 -3.051879 0.165960 -1.296067
1 -3.140685 -1.406808 -0.483336

7 -1.063689 2.007633 -0.268699
1 -0.494073 2.836496 -0.158375
1 -2.063182 2.100452 -0.153248
8 -3.443302 0.307195 0.684036
1 -3.238329 -0.154395 1.511642
6 1.489778 -2.888763 0.107955
1 2.173559 -3.009653 -0.734858
1 2.062381 -2.909418 1.037186
1 0.763097 -3.700323 0.107979
8 2.340670 3.141514 0.151713
1 1.833003 2.294189 0.066387
1 1.666463 3.835304 0.123009
1m5hmC/1m5hmCabi.xyz
6 1.121500 0.567456 -0.396908
6 -1.165051 0.983389 -0.184610
6 -1.477508 -0.419664 -0.171803
6 -0.410076 -1.265898 -0.242545
1 -0.535437 -2.342879 -0.228862
7 0.869102 -0.809730 -0.351036
7 0.089831 1.436137 -0.303125
8 2.312398 0.958341 -0.520871
6 -2.888323 -0.936485 -0.123589
1 -3.469536 -0.542497 -0.963329
1 -2.880952 -2.029573 -0.198697
7 -2.152827 1.896024 -0.095911
1 -1.909150 2.875241 -0.029403
1 -3.077728 1.613848 0.198296
8 -3.607408 -0.515118 1.054469
1 -3.140419 -0.859113 1.831215
6 2.001717 -1.744138 -0.422462
1 2.625025 -1.505011 -1.284978
1 2.602556 -1.674299 0.486529
1 1.612505 -2.756921 -0.522590
8 4.445191 0.373347 1.150409
1 4.568583 -0.585810 1.176026
1 3.666814 0.509481 0.565741

1m5hmC/1m5hmCabw.xyz
6 -2.045947 -0.847150 0.090001
6 0.279579 -0.860250 -0.193733
6 0.329384 0.575200 -0.306356
6 -0.870532 1.219326 -0.197107
1 -0.943327 2.299134 -0.266942
7 -2.040618 0.554208 -0.010074
7 -0.874009 -1.518049 -0.007670
8 -3.142888 -1.428711 0.273041
6 1.588781 1.360714 -0.536270
1 2.185375 0.927322 -1.344105
1 1.331053 2.387227 -0.817807
7 1.396488 -1.606129 -0.293721
1 1.317008 -2.603413 -0.144738
1 2.327759 -1.204120 -0.267449
8 2.475802 1.380441 0.608435
1 1.977136 1.662914 1.390390
6 -3.312617 1.276072 0.117473
1 -4.019374 0.922373 -0.635407
1 -3.737383 1.112296 1.110301
1 -3.125856 2.339369 -0.029641
8 4.283260 -0.685862 0.284576
1 3.764476 0.117704 0.503760
1 4.808826 -0.441424 -0.490571
1m5hmC/1m5hmCacu.xyz
6 1.072033 0.638906 -0.026448
6 -1.233553 0.992428 -0.124456
6 -1.503570 -0.415655 -0.225727
6 -0.411734 -1.232059 -0.188660
1 -0.505553 -2.310909 -0.244253
7 0.856100 -0.743470 -0.088779
7 0.010856 1.477728 -0.037352
8 2.253540 1.066633 0.046237
6 -2.892450 -0.969354 -0.377997
1 -3.389747 -0.536698 -1.251739
1 -2.840651 -2.054509 -0.521556

7 -2.248760 1.880142 -0.137968
1 -2.040506 2.855982 0.027007
1 -3.197634 1.566468 0.012743
8 -3.751494 -0.645526 0.736050
1 -3.355932 -1.017860 1.539224
6 2.015372 -1.646111 -0.049636
1 2.676859 -1.437641 -0.892358
1 2.564145 -1.508057 0.883513
1 1.660990 -2.674442 -0.113159
8 4.872937 0.199179 0.217682
1 3.915472 0.413096 0.157890
1 4.908730 -0.767447 0.209175
1m5hmC/1m5hmCaac.xyz
6 -1.837573 -0.672064 0.204460
6 0.412936 -1.204468 -0.125729
6 0.689885 0.072242 -0.725015
6 -0.361134 0.939693 -0.780947
1 -0.253076 1.941401 -1.181818
7 -1.604489 0.597613 -0.345110
7 -0.806777 -1.547811 0.302707
8 -2.997370 -0.951736 0.594749
6 2.058287 0.460920 -1.217315
1 2.395571 -0.217389 -2.008158
1 2.015080 1.473357 -1.637007
7 1.401199 -2.117162 0.000432
1 1.211261 -2.957976 0.529800
1 2.363649 -1.813289 -0.070646
8 3.063861 0.382240 -0.188359
1 2.711218 0.837751 0.604791
6 -2.713582 1.558472 -0.383608
1 -3.552624 1.137863 -0.940693
1 -3.042318 1.789683 0.632158
1 -2.370367 2.469380 -0.873360
8 1.667147 1.467177 2.055027
1 1.156574 0.648839 2.157557
1 1.073482 2.051699 1.558760

1m5hmC/1m5hmCace.xyz
6 2.374402 0.431621 0.051541
6 0.174901 1.202967 -0.114018
6 -0.348070 -0.132759 -0.217791
6 0.572483 -1.138037 -0.148289
1 0.283365 -2.181631 -0.205730
7 1.903194 -0.890261 -0.014654
7 1.483660 1.452441 0.009182
8 3.609972 0.620954 0.158076
6 -1.807488 -0.420154 -0.413832
1 -2.201182 0.117277 -1.281851
1 -1.958724 -1.492153 -0.578327
7 -0.659161 2.261705 -0.161017
1 -0.281079 3.188558 -0.017653
1 -1.656716 2.133203 -0.067510
8 -2.619435 0.033150 0.698190
1 -2.328533 -0.435777 1.496457
6 2.878487 -1.985955 0.046206
1 3.585573 -1.902675 -0.781530
1 3.427496 -1.944212 0.989172
1 2.343242 -2.932367 -0.022796
8 -5.224324 -0.752843 0.025548
1 -5.180097 -0.806596 -0.939786
1 -4.325181 -0.467001 0.297553
1m5hmC/1m5hmCaan.xyz
6 -1.451131 0.684563 -0.628258
6 0.817054 0.990609 -0.163182
6 1.029614 -0.421220 0.018193
6 -0.057368 -1.217615 -0.201573
1 -0.002128 -2.296114 -0.102562
7 -1.269206 -0.705938 -0.549314
7 -0.380255 1.501567 -0.470770
8 -2.603864 1.123262 -0.856535
6 2.353836 -0.996932 0.433896
1 2.705526 -0.529191 1.358698
1 2.245781 -2.071910 0.617293

7 1.839276 1.854552 0.004090
1 1.686847 2.833142 -0.200686
1 2.791643 1.518186 0.038845
8 3.401517 -0.751507 -0.528779
1 3.137277 -1.156249 -1.369330
6 -2.447575 -1.571050 -0.682990
1 -3.145883 -1.378118 0.135318
1 -2.946470 -1.374324 -1.633168
1 -2.123277 -2.610736 -0.650748
8 -1.365841 0.059860 2.851699
1 -2.040839 0.305017 2.201984
1 -0.558273 -0.054023 2.328720
1m5hmC/1m5hmCabz.xyz
6 1.277240 -0.296941 0.025206
6 -0.675830 0.966712 -0.136125
6 -1.497080 -0.209878 -0.224527
6 -0.837212 -1.401625 -0.144295
1 -1.363063 -2.348982 -0.187928
7 0.516324 -1.470389 -0.017853
7 0.657214 0.902945 -0.025922
8 2.528606 -0.406815 0.119559
6 -2.987197 -0.148970 -0.412769
1 -3.240788 0.419951 -1.312837
1 -3.383230 -1.164049 -0.530711
7 -1.236388 2.191809 -0.185510
1 -0.647772 2.998930 -0.026240
1 -2.233221 2.298216 -0.056986
8 -3.665477 0.536075 0.661666
1 -3.484853 0.056908 1.484857
6 1.209226 -2.761721 0.073558
1 1.891806 -2.880672 -0.770453
1 1.779699 -2.810202 1.002991
1 0.465926 -3.557855 0.057663
8 4.199673 1.759916 0.069099
1 3.523710 1.039371 0.083848
1 3.694440 2.578970 -0.028083

1m5hmC/1m5hmCabx.xyz
6 1.906404 -0.592897 -0.248257
6 -0.295507 -1.338324 -0.009979
6 -0.699425 -0.125508 0.646193
6 0.268769 0.826214 0.777755
1 0.064591 1.789385 1.232558
7 1.546390 0.622399 0.354363
7 0.960547 -1.549674 -0.419702
8 3.096245 -0.748921 -0.616047
6 -2.105692 0.110533 1.126204
1 -2.420899 -0.685722 1.808272
1 -2.147497 1.059017 1.675215
7 -1.193210 -2.328006 -0.211289
1 -0.914616 -3.124225 -0.769507
1 -2.181681 -2.128975 -0.139021
8 -3.067006 0.104682 0.052557
1 -2.786728 0.799181 -0.580583
6 2.563818 1.673617 0.480844
1 3.414431 1.303476 1.056022
1 2.910425 1.980378 -0.508395
1 2.121021 2.526719 0.993862
8 -1.839990 2.112947 -1.547026
1 -1.082892 1.554628 -1.783280
1 -1.516441 2.641647 -0.801187
1m5hmU/1m5hmUabv.xyz
6 -1.946955 0.459439 0.257322
6 0.316514 1.446098 0.029478
6 0.729481 0.240434 -0.654115
6 -0.182336 -0.756115 -0.819760
1 0.081551 -1.686935 -1.309192
7 -1.483999 -0.669275 -0.395068
7 -1.018202 1.465152 0.422691
8 -3.109011 0.564136 0.666721
8 1.050853 2.419408 0.277352
1 -1.349476 2.293470 0.910812
6 2.155690 0.104546 -1.101742

1 2.259366 -0.749030 -1.773660
1 2.480934 1.008452 -1.626698
8 3.057477 -0.151954 0.003686
1 3.098000 0.646495 0.553336
6 -2.421707 -1.791679 -0.556237
1 -2.720273 -2.169171 0.423885
1 -3.306122 -1.458182 -1.101325
1 -1.923050 -2.580864 -1.116400
8 1.954734 -2.253153 1.504578
1 1.009766 -2.209688 1.299960
1 2.348164 -1.509523 0.997550
1m5hmU/1m5hmUacg.xyz
6 1.995353 -0.805228 0.098124
6 -0.444749 -0.828401 -0.342441
6 -0.420855 0.615802 -0.372417
6 0.776805 1.237627 -0.179188
1 0.856856 2.318782 -0.201357
7 1.955070 0.575625 0.046059
7 0.785002 -1.431097 -0.111080
8 3.035570 -1.437520 0.314357
8 -1.445770 -1.555490 -0.514640
1 0.803279 -2.447141 -0.077300
6 -1.680535 1.406646 -0.612977
1 -1.413898 2.402578 -0.975025
1 -2.283877 0.912235 -1.382777
8 -2.467981 1.598453 0.576340
1 -3.007514 0.790516 0.713024
6 3.219434 1.300192 0.252210
1 3.606204 1.092243 1.251651
1 3.951138 0.984196 -0.493232
1 3.026035 2.366291 0.146872
8 -3.845696 -0.855856 0.655419
1 -4.545381 -0.800839 -0.011752
1 -3.055047 -1.187083 0.173375
1m5hmU/1m5hmUacb.xyz
6 1.924990 -0.512786 0.264767

6 -0.385195 -1.366601 -0.006172
6 -0.716970 -0.137929 -0.691556
6 0.250233 0.807408 -0.834628
1 0.047936 1.755560 -1.320097
7 1.538334 0.642035 -0.390253
7 0.938023 -1.465887 0.409174
8 3.071290 -0.683932 0.695985
8 -1.185185 -2.291249 0.228674
1 1.209918 -2.310387 0.906213
6 -2.125878 0.062447 -1.169757
1 -2.204723 0.993023 -1.734309
1 -2.432608 -0.767532 -1.815491
8 -3.063260 0.178058 -0.072329
1 -3.095180 -0.680498 0.379888
6 2.540910 1.709930 -0.534238
1 2.835283 2.079304 0.450192
1 3.419179 1.323942 -1.054113
1 2.099290 2.519118 -1.113192
8 -1.923678 2.059950 1.696988
1 -0.980912 1.973563 1.495023
1 -2.354550 1.403434 1.108398
1m5hmU/1m5hmUaac.xyz
6 -1.413873 1.113430 -0.014465
6 0.818134 0.079442 -0.267746
6 0.185703 -1.216993 -0.239308
6 -1.164675 -1.272345 -0.078004
1 -1.690443 -2.219405 -0.034140
7 -1.956474 -0.157343 0.034220
7 -0.042545 1.161419 -0.162376
8 -2.101340 2.137138 0.070539
8 2.049545 0.264834 -0.371826
1 0.367375 2.092630 -0.176060
6 1.043569 -2.443416 -0.379443
1 0.414515 -3.334741 -0.422165
1 1.626251 -2.387541 -1.307000
8 1.934311 -2.627472 0.739081

1 2.575633 -1.899545 0.723676
6 -3.413326 -0.261996 0.216068
1 -3.700377 0.199752 1.162568
1 -3.927386 0.242028 -0.604213
1 -3.684544 -1.316189 0.223701
8 3.056905 2.786568 0.349944
1 3.428652 3.205923 -0.438718
1 2.734887 1.911291 0.044926
1m5hmU/1m5hmUadd.xyz
6 1.279642 0.396344 0.001909
6 -0.727535 -1.036140 -0.202106
6 -1.532062 0.164615 -0.231058
6 -0.905465 1.367245 -0.131448
1 -1.461437 2.297783 -0.137650
7 0.457365 1.502036 -0.023945
7 0.643310 -0.819955 -0.092295
8 2.513300 0.490810 0.105577
8 -1.170496 -2.197021 -0.260949
1 1.245055 -1.640279 -0.065942
6 -3.023787 0.033053 -0.356689
1 -3.476152 1.020632 -0.467728
1 -3.273848 -0.563042 -1.242553
8 -3.628750 -0.560565 0.810104
1 -3.340695 -1.485747 0.851208
6 1.093962 2.825556 0.074398
1 1.649801 2.898728 1.010797
1 1.776452 2.970966 -0.765018
1 0.314310 3.584893 0.048720
8 4.030322 -1.883692 0.239838
1 4.270423 -2.113041 -0.669005
1 3.527825 -1.045750 0.164245
1m5hmU/1m5hmUadr.xyz
6 -1.978251 -0.807231 -0.096356
6 0.452215 -0.816170 0.393017
6 0.425398 0.628164 0.393781
6 -0.769352 1.243817 0.165318

1 -0.851858 2.325038 0.166363
7 -1.941183 0.574733 -0.072459
7 -0.770536 -1.426262 0.145669
8 -3.013486 -1.446083 -0.316914
8 1.451353 -1.536385 0.602501
1 -0.787014 -2.442768 0.134367
6 1.679934 1.424663 0.643112
1 1.407043 2.422769 0.994155
1 2.275040 0.937639 1.423880
8 2.480603 1.611773 -0.538308
1 3.003024 0.793826 -0.682933
6 -3.204983 1.291475 -0.308829
1 -3.593753 1.039361 -1.297020
1 -3.935939 1.010853 0.451520
1 -3.009678 2.361052 -0.253621
8 3.831912 -0.855608 -0.615972
1 3.809059 -1.273380 -1.488999
1 3.033287 -1.191571 -0.150903
1m5hmU/1m5hmUacq.xyz
6 -1.285112 0.391223 -0.092878
6 0.725686 -1.035641 0.121047
6 1.517382 0.167978 0.245477
6 0.884598 1.369487 0.177370
1 1.431559 2.302375 0.251414
7 -0.474574 1.499938 0.024503
7 -0.640958 -0.823736 -0.036340
8 -2.514561 0.481620 -0.239025
8 1.176517 -2.195010 0.139318
1 -1.231059 -1.646418 -0.136813
6 3.002867 0.039391 0.435540
1 3.444589 1.025447 0.593320
1 3.214837 -0.578975 1.316196
8 3.662253 -0.522382 -0.716903
1 3.375262 -1.445580 -0.795215
6 -1.116081 2.822402 -0.050076
1 -1.558072 2.965116 -1.038247

1 -1.893728 2.896453 0.711622
1 -0.358355 3.583756 0.127310
8 -4.075581 -1.862953 0.039628
1 -3.548821 -1.043896 -0.067209
1 -3.432547 -2.530913 0.317466
1m5hmU/1m5hmUada.xyz
6 -1.552607 -1.083745 -0.157524
6 0.912783 -1.333127 -0.130306
6 0.996874 -0.053680 0.538647
6 -0.156856 0.613811 0.806403
1 -0.149572 1.579370 1.298840
7 -1.402044 0.130181 0.486750
7 -0.379473 -1.762004 -0.414408
8 -2.657306 -1.534315 -0.483977
8 1.888142 -2.035506 -0.453721
1 -0.480935 -2.654559 -0.891124
6 2.352413 0.498988 0.874354
1 2.247492 1.406095 1.472752
1 2.926460 -0.234719 1.452711
8 3.097594 0.873702 -0.303071
1 3.293674 0.060826 -0.794789
6 -2.616167 0.924374 0.731003
1 -3.066885 1.215714 -0.220075
1 -3.332477 0.338249 1.308561
1 -2.339308 1.814429 1.294281
8 -0.481557 3.298357 -1.222785
1 -1.166879 3.113940 -0.563776
1 0.145427 2.565781 -1.130801
1m5hmU/1m5hmUaaz.xyz
6 -1.665773 -0.832850 0.121542
6 0.778340 -0.972570 0.501904
6 0.942787 0.156441 -0.387651
6 -0.166551 0.692787 -0.965280
1 -0.099563 1.542054 -1.635947
7 -1.436301 0.223868 -0.740955
7 -0.534529 -1.395101 0.676284

8 -2.800672 -1.251745 0.376718
8 1.707125 -1.559506 1.086796
1 -0.693204 -2.180295 1.302953
6 2.324644 0.708939 -0.602192
1 2.279852 1.582268 -1.256002
1 2.753054 1.019292 0.359347
8 3.207756 -0.235036 -1.238760
1 3.336869 -0.970346 -0.618836
6 -2.612219 0.899719 -1.311917
1 -3.244316 0.171046 -1.820884
1 -3.182460 1.386246 -0.517673
1 -2.266590 1.644363 -2.027085
8 -0.054260 2.512974 2.044192
1 0.423885 2.494804 2.885836
1 0.401329 1.860908 1.490991
1m5hmU/1m5hmUabp.xyz
6 1.749678 0.456162 -0.533892
6 -0.702172 0.783993 -0.661773
6 -0.868932 -0.502514 -0.021969
6 0.243836 -1.213125 0.305815
1 0.173318 -2.179685 0.791724
7 1.520575 -0.770333 0.062408
7 0.617111 1.167377 -0.871320
8 2.886856 0.889757 -0.752991
8 -1.633608 1.529658 -1.015854
1 0.777116 2.069028 -1.313371
6 -2.260820 -0.979821 0.280497
1 -2.220513 -1.904559 0.859421
1 -2.790972 -0.223660 0.872537
8 -3.017779 -1.277841 -0.909908
1 -3.161669 -0.441964 -1.380641
6 2.698151 -1.551890 0.473175
1 3.341158 -1.731056 -0.389875
1 3.258112 -1.008166 1.236676
1 2.358134 -2.503879 0.877230
8 -0.749236 1.445039 2.758592

1 -0.564921 0.571094 2.382731
1 -1.181881 1.923813 2.036133
1m5hmU/1m5hmUaap.xyz
6 1.948668 -0.486444 0.270496
6 -0.345823 -1.383090 0.005136
6 -0.704452 -0.157531 -0.672314
6 0.243746 0.807145 -0.814132
1 0.021139 1.753104 -1.295028
7 1.536613 0.664881 -0.375525
7 0.980212 -1.458143 0.416145
8 3.100764 -0.639296 0.692916
8 -1.126220 -2.325325 0.236107
1 1.271710 -2.301075 0.904701
6 -2.115284 0.011248 -1.157646
1 -2.214209 0.945106 -1.713575
1 -2.394668 -0.819788 -1.814490
8 -3.064834 0.090542 -0.068004
1 -3.063846 -0.766537 0.388095
6 2.518509 1.751724 -0.519302
1 2.812736 2.120044 0.465560
1 3.399895 1.384384 -1.047343
1 2.059124 2.556664 -1.090364
8 -2.061354 2.113653 1.613858
1 -1.122137 2.109903 1.379781
1 -2.446549 1.396126 1.065889
1m5hmU/1m5hmUaco.xyz
6 1.277410 0.401517 -0.001981
6 -0.716988 -1.048998 -0.211418
6 -1.532055 0.144940 -0.241361
6 -0.915587 1.352935 -0.140777
1 -1.479612 2.278567 -0.148379
7 0.445436 1.500071 -0.029569
7 0.651903 -0.820028 -0.098837
8 2.509911 0.506673 0.105101
8 -1.147554 -2.214125 -0.273177
1 1.260591 -1.635106 -0.070834

6 -3.023252 0.003457 -0.360790
1 -3.477843 0.982682 -0.523907
1 -3.270723 -0.639982 -1.213285
8 -3.628563 -0.526961 0.836609
1 -3.339515 -1.447599 0.932146
6 1.069688 2.829307 0.071340
1 1.621620 2.906959 1.009696
1 1.753676 2.981242 -0.765677
1 0.283207 3.581605 0.043591
8 4.040476 -1.859246 0.258654
1 3.534689 -1.024004 0.175974
1 4.283079 -2.094335 -0.648054
1m5hmU/1m5hmUadf.xyz
6 -1.975592 -0.428099 -0.293481
6 0.281666 -1.414712 -0.028229
6 0.680075 -0.216094 0.674328
6 -0.231019 0.782707 0.823221
1 0.023748 1.711580 1.320783
7 -1.524941 0.696562 0.373558
7 -1.042308 -1.431043 -0.452934
8 -3.130699 -0.532024 -0.722776
8 1.026280 -2.382887 -0.269257
1 -1.362129 -2.254206 -0.957105
6 2.093210 -0.104306 1.167589
1 2.214124 0.800844 1.764985
1 2.352898 -0.970839 1.785190
8 3.044775 0.004897 0.081688
1 3.032234 -0.831659 -0.410589
6 -2.471671 1.810893 0.538417
1 -2.763037 2.198991 -0.439578
1 -3.359326 1.463933 1.069983
1 -1.984378 2.595715 1.114458
8 2.089678 2.144942 -1.479297
1 2.852573 2.576928 -1.888323
1 2.463758 1.388330 -0.978099
1m5hmU/1m5hmUack.xyz

6 -2.003443 -0.855284 -0.084468
6 0.471722 -0.750249 -0.040751
6 0.373858 0.675857 0.164982
6 -0.867623 1.232147 0.229202
1 -0.999328 2.297596 0.381942
7 -2.029326 0.512618 0.114527
7 -0.743749 -1.411708 -0.158710
8 -3.030061 -1.536176 -0.188715
8 1.530628 -1.405518 -0.117807
1 -0.714118 -2.417487 -0.306636
6 1.623952 1.501659 0.287120
1 1.363553 2.514950 0.599879
1 2.291802 1.069367 1.040153
8 2.328248 1.635478 -0.964716
1 2.776819 0.796041 -1.148261
6 -3.347853 1.159316 0.205443
1 -3.900245 1.006227 -0.723463
1 -3.911169 0.734750 1.038237
1 -3.196555 2.224446 0.370984
8 4.207352 -0.825258 0.502013
1 3.262693 -0.987612 0.297365
1 4.645057 -1.673520 0.343716
1m5hmU/1m5hmUact.xyz
6 -1.844252 0.604415 0.180441
6 0.519375 1.229497 -0.223663
6 0.735476 -0.094619 -0.762273
6 -0.310204 -0.964197 -0.785845
1 -0.194130 -1.976959 -1.154616
7 -1.574280 -0.637468 -0.363961
7 -0.781826 1.483717 0.196563
8 -2.959482 0.915095 0.615696
8 1.392740 2.109276 -0.113282
1 -0.971062 2.397024 0.601271
6 2.119906 -0.484464 -1.192983
1 2.107791 -1.483807 -1.631523
1 2.508341 0.220224 -1.935632

8 3.037735 -0.543901 -0.073466
1 3.261693 0.367651 0.173017
6 -2.663306 -1.626531 -0.371236
1 -2.948840 -1.871972 0.653891
1 -3.525926 -1.220209 -0.901443
1 -2.310174 -2.522232 -0.879620
8 1.582476 -1.218855 2.276035
1 2.098877 -1.027252 1.464507
1 0.696463 -0.883737 2.075422
1m5hmU/1m5hmUadk.xyz
6 -1.419222 1.105391 -0.024942
6 0.818196 0.084492 -0.288997
6 0.194936 -1.215919 -0.238897
6 -1.153627 -1.278794 -0.064732
1 -1.672168 -2.228968 -0.005171
7 -1.952586 -0.168415 0.041824
7 -0.049699 1.161367 -0.186258
8 -2.112940 2.125262 0.055374
8 2.046690 0.278238 -0.408587
1 0.350961 2.095840 -0.220184
6 1.058848 -2.438676 -0.372880
1 0.434069 -3.333186 -0.411686
1 1.641561 -2.384311 -1.300417
8 1.949901 -2.614140 0.747014
1 2.591229 -1.886464 0.727322
6 -3.407203 -0.280850 0.236254
1 -3.689167 0.182290 1.183661
1 -3.930142 0.217924 -0.581585
1 -3.672488 -1.336522 0.249083
8 2.965657 2.835063 0.328676
1 2.720671 1.932660 0.032127
1 3.895433 2.940819 0.083253
1m5hmU/1m5hmUabd.xyz
6 -1.112966 0.541565 -0.315669
6 1.311351 1.023035 -0.184000
6 1.553609 -0.402487 -0.191858

6 0.485686 -1.241584 -0.249657
1 0.611973 -2.318271 -0.251207
7 -0.816106 -0.805236 -0.322106
7 -0.029430 1.387985 -0.253535
8 -2.273479 0.977056 -0.366624
8 2.193457 1.897765 -0.113795
1 -0.246858 2.381372 -0.242770
6 2.973067 -0.890248 -0.126060
1 2.999363 -1.976732 -0.229114
1 3.556703 -0.450051 -0.943676
8 3.603261 -0.585929 1.134717
1 3.683644 0.378983 1.194719
6 -1.939866 -1.755800 -0.380683
1 -2.512544 -1.714286 0.547519
1 -2.588228 -1.505903 -1.221399
1 -1.535639 -2.757570 -0.516618
8 -4.645385 0.089936 0.906673
1 -4.889149 0.868356 1.427002
1 -3.808646 0.337347 0.464821
1m5hmU/1m5hmUadl.xyz
6 -1.848026 0.608920 0.184348
6 0.518740 1.219545 -0.219728
6 0.726973 -0.105352 -0.758714
6 -0.322407 -0.970226 -0.778925
1 -0.211575 -1.984308 -1.145652
7 -1.584553 -0.635760 -0.356693
7 -0.780171 1.481795 0.200981
8 -2.961807 0.927408 0.617666
8 1.399425 2.092411 -0.107836
1 -0.964125 2.396193 0.605648
6 2.109389 -0.495777 -1.196391
1 2.104138 -1.511985 -1.594342
1 2.475981 0.183019 -1.974008
8 3.044782 -0.494947 -0.091113
1 3.218330 0.431868 0.140901
6 -2.678895 -1.619074 -0.361293

1 -2.965158 -1.860742 0.664523
1 -3.539638 -1.209315 -0.891900
1 -2.331049 -2.517801 -0.868006
8 1.625135 -1.240850 2.259366
1 2.139070 -1.025870 1.452119
1 0.732157 -0.923696 2.060258
1m5hmU/1m5hmUaaq.xyz
6 1.481707 0.552867 0.018224
6 -0.526782 -0.860814 -0.199496
6 -1.329677 0.341166 -0.231538
6 -0.700233 1.541420 -0.120788
1 -1.252918 2.474166 -0.125662
7 0.661339 1.667345 -0.003985
7 0.840905 -0.660261 -0.083941
8 2.713211 0.640798 0.126664
8 -0.985959 -2.019742 -0.259681
1 1.441351 -1.500532 -0.048317
6 -2.819374 0.209660 -0.372393
1 -3.277722 1.197936 -0.447367
1 -3.062218 -0.353073 -1.282282
8 -3.427606 -0.435239 0.766051
1 -3.101314 -1.348797 0.786822
6 1.302891 2.985811 0.107916
1 1.858363 3.048982 1.045527
1 1.988039 3.138257 -0.728337
1 0.527236 3.749641 0.089119
8 2.373034 -3.082974 0.014709
1 1.681602 -3.759935 0.075655
1 2.849239 -3.139644 0.856823
1m5hmU/1m5hmUacf.xyz
6 1.390242 -1.148574 0.109091
6 -0.818507 -0.055270 -0.094037
6 -0.143290 1.212768 -0.223312
6 1.216600 1.226955 -0.181936
1 1.774013 2.152166 -0.274218
7 1.977815 0.094662 -0.033301

7 0.010118 -1.154575 0.070824
8 2.046172 -2.185124 0.260526
8 -2.059347 -0.200862 -0.115388
1 -0.429865 -2.066132 0.173468
6 -0.970243 2.455119 -0.405829
1 -0.317952 3.323071 -0.520058
1 -1.586588 2.364900 -1.308838
8 -1.815818 2.726336 0.729401
1 -2.470824 2.012010 0.778877
6 3.448716 0.149992 -0.025689
1 3.829694 -0.228463 0.924553
1 3.843297 -0.456546 -0.842900
1 3.757190 1.185952 -0.156289
8 -3.187214 -2.764215 -0.140281
1 -4.109636 -2.667009 0.134974
1 -2.816315 -1.855568 -0.116020
1m5mC/1m5mCaaq.xyz
6 -0.599081 -1.065955 0.003522
6 0.058326 1.188530 0.004393
6 1.454149 0.849514 -0.001557
6 1.728050 -0.486451 -0.003577
1 2.746305 -0.857601 -0.008006
7 0.747186 -1.437876 -0.000648
7 -0.906362 0.256351 0.006250
8 -1.482370 -1.957951 0.005008
7 -0.331455 2.477037 0.009021
1 -1.317078 2.702554 0.010557
1 0.337335 3.232678 0.004039
6 2.533252 1.896153 -0.004747
1 2.453299 2.550507 -0.881513
1 2.475240 2.533728 0.886053
1 3.521112 1.428932 -0.021774
6 1.078417 -2.867794 -0.001729
1 0.667135 -3.347904 0.888706
1 0.662770 -3.347940 -0.890114
1 2.162680 -2.975459 -0.004416

8 -3.646092 0.866919 -0.015704
1 -4.087595 0.007035 0.031097
1 -2.677755 0.649160 -0.009254
1m5mC/1m5mCabe.xyz
6 0.905959 0.329475 0.000604
6 -0.939564 -1.095877 -0.000226
6 -1.869850 -0.000254 -0.007260
6 -1.306661 1.241076 -0.010815
1 -1.909460 2.141987 -0.014341
7 0.047462 1.427950 -0.009613
7 0.389305 -0.918716 0.002006
8 2.150927 0.544298 0.010152
7 -1.394781 -2.364128 0.004508
1 -0.737376 -3.131530 0.016986
1 -2.382275 -2.572582 0.011468
6 -3.357523 -0.213845 -0.006487
1 -3.680846 -0.782691 -0.886954
1 -3.680524 -0.771593 0.881251
1 -3.883874 0.744127 -0.012345
6 0.627351 2.776879 0.013227
1 1.220305 2.911339 0.920404
1 1.271209 2.922019 -0.856444
1 -0.182131 3.506109 -0.005740
8 3.905008 -1.558273 0.056171
1 4.626382 -1.267874 -0.519035
1 3.238926 -0.827978 0.021609
1m5mC/1m5mCaaa.xyz
6 -0.912299 0.344598 0.027710
6 0.921804 -1.095627 0.008844
6 1.860546 -0.007339 -0.005733
6 1.307340 1.238446 -0.002315
1 1.917228 2.134527 -0.014230
7 -0.045056 1.436171 0.017185
7 -0.405580 -0.907887 0.027769
8 -2.155217 0.569698 0.036743
7 1.367679 -2.367198 0.004112

1 0.705307 -3.130305 0.012107
1 2.353557 -2.582720 -0.011276
6 3.346374 -0.232354 -0.025458
1 3.676976 -0.798627 0.853980
1 3.653798 -0.797287 -0.914189
1 3.879775 0.721706 -0.031888
6 -0.614422 2.789382 -0.014693
1 -1.215995 2.918277 -0.917026
1 -1.247534 2.950170 0.859979
1 0.201129 3.512019 -0.012425
8 -3.966356 -1.471133 -0.081426
1 -3.575793 -2.276640 0.284685
1 -3.251224 -0.789884 -0.025841
1m5mC/1m5mCabl.xyz
6 -0.905057 0.323846 -0.060147
6 0.944951 -1.094966 -0.020763
6 1.870836 0.003665 0.018190
6 1.302958 1.242901 0.021514
1 1.902083 2.145666 0.053695
7 -0.051185 1.425415 -0.017804
7 -0.383861 -0.922265 -0.062535
8 -2.150253 0.534821 -0.095836
7 1.403807 -2.361869 -0.016555
1 0.749079 -3.130936 -0.050603
1 2.391591 -2.567747 0.005713
6 3.358895 -0.205030 0.053513
1 3.662978 -0.772759 0.941593
1 3.704430 -0.762123 -0.826054
1 3.881902 0.754597 0.071936
6 -0.636163 2.771650 0.028777
1 -1.260264 2.940239 -0.850805
1 -1.249998 2.879072 0.925787
1 0.171153 3.503281 0.048012
8 -3.932752 -1.538568 0.009144
1 -3.250538 -0.822634 -0.012246
1 -4.438222 -1.378028 0.818265

1m5mC/1m5mCaai.xyz
6 -0.758614 -0.467623 -0.480698
6 1.498940 -0.854975 -0.040191
6 1.765768 0.540407 0.175795
6 0.689804 1.365888 0.038287
1 0.773046 2.437489 0.179018
7 -0.552883 0.898734 -0.286463
7 0.281891 -1.320005 -0.357816
8 -1.921333 -0.868113 -0.771475
7 2.492450 -1.757599 0.076951
1 2.298141 -2.737651 -0.074968
1 3.435666 -1.482841 0.308606
6 3.136675 1.051027 0.520983
1 3.507203 0.607268 1.453169
1 3.860925 0.810480 -0.267139
1 3.122137 2.136592 0.647204
6 -1.695962 1.814237 -0.406780
1 -2.190437 1.665209 -1.367846
1 -2.411693 1.630680 0.397159
1 -1.330472 2.838499 -0.340785
8 -3.957262 -0.540697 1.077052
1 -4.176061 -1.449085 1.327564
1 -3.229567 -0.627833 0.420334
1m5mC/1m5mCacy.xyz
6 0.754035 -0.489846 -0.475437
6 -1.507634 -0.846764 -0.030614
6 -1.758613 0.553862 0.170491
6 -0.673103 1.365431 0.025562
1 -0.743934 2.439394 0.154470
7 0.564400 0.880597 -0.293405
7 -0.296018 -1.329073 -0.343117
8 1.911518 -0.906037 -0.764742
7 -2.511585 -1.736749 0.093631
1 -2.326252 -2.720947 -0.042313
1 -3.449377 -1.450111 0.332926
6 -3.124053 1.084398 0.507052

1 -3.850224 0.839941 -0.278042
1 -3.500663 0.659448 1.445510
1 -3.097691 2.171562 0.616486
6 1.716765 1.782023 -0.427665
1 2.438057 1.592866 0.369986
1 2.200806 1.624042 -1.392707
1 1.363956 2.810672 -0.361683
8 3.944093 -0.705106 1.098969
1 4.158857 0.228627 1.232583
1 3.219613 -0.704397 0.432878
1m5mC/1m5mCaca.xyz
6 0.698384 -0.574086 -0.156826
6 -1.614961 -0.838931 0.002430
6 -1.841265 0.579322 0.062084
6 -0.718674 1.349283 -0.001550
1 -0.767455 2.431742 0.030808
7 0.532985 0.810293 -0.112896
7 -0.390646 -1.373868 -0.104436
8 1.865385 -1.046763 -0.248604
7 -2.658280 -1.690466 0.051754
1 -2.490757 -2.686813 0.026536
1 -3.607710 -1.361581 0.145691
6 -3.218543 1.169231 0.183661
1 -3.852119 0.885843 -0.665963
1 -3.721520 0.825996 1.096020
1 -3.167080 2.260590 0.216187
6 1.721381 1.670354 -0.181640
1 2.348924 1.514880 0.698005
1 2.299272 1.436316 -1.077347
1 1.399379 2.710457 -0.218495
8 4.490053 -0.357812 0.268534
1 3.532177 -0.516022 0.110681
1 4.637039 -0.617552 1.188533
1m5mC/1m5mCadr.xyz
6 -0.751853 -0.492376 -0.468321
6 1.510980 -0.847732 -0.027424

6 1.762691 0.553704 0.167177
6 0.676844 1.364680 0.021861
1 0.747973 2.439133 0.146469
7 -0.561419 0.878767 -0.292527
7 0.298656 -1.331100 -0.335425
8 -1.909973 -0.909911 -0.752725
7 2.515173 -1.737286 0.098155
1 2.328872 -2.722295 -0.030436
1 3.452496 -1.450056 0.338606
6 3.128982 1.085758 0.497824
1 3.507737 0.666037 1.437782
1 3.853372 0.836951 -0.287524
1 3.102933 2.173516 0.601300
6 -1.714574 1.779742 -0.421860
1 -2.212176 1.610771 -1.377971
1 -2.424869 1.600972 0.388049
1 -1.360227 2.808809 -0.372934
8 -3.977219 -0.695013 1.069308
1 -4.109615 0.236726 1.293254
1 -3.234241 -0.696493 0.423785
1m5mC/1m5mCabp.xyz
6 -1.758880 -0.872206 0.000139
6 0.568155 -0.637390 -0.022545
6 0.489005 0.799907 -0.019021
6 -0.773258 1.314725 0.000595
1 -0.952906 2.383922 0.006720
7 -1.885303 0.520845 0.013179
7 -0.521229 -1.421315 -0.015930
8 -2.807381 -1.569258 0.005448
7 1.766516 -1.251092 -0.034957
1 1.790579 -2.261631 -0.028951
1 2.652762 -0.751432 -0.023243
6 1.711576 1.675061 -0.034484
1 2.346890 1.491292 0.839894
1 2.322572 1.493128 -0.926900
1 1.425636 2.730138 -0.029839

6 -3.232693 1.100643 0.034954
1 -3.789542 0.787854 -0.851074
1 -3.768333 0.767744 0.926602
1 -3.147168 2.186759 0.045842
8 4.582631 -0.241438 -0.021846
1 4.494281 0.702473 -0.221389
1 4.879899 -0.268184 0.899655
1m5mC/1m5mCadg.xyz
6 -1.508406 -0.722649 0.266304
6 0.759083 -1.092064 -0.161542
6 0.926769 0.201097 -0.768724
6 -0.195863 0.975780 -0.804369
1 -0.183947 1.972670 -1.230103
7 -1.392938 0.551099 -0.304409
7 -0.412826 -1.518341 0.325748
8 -2.628765 -1.086911 0.705647
7 1.808674 -1.933216 -0.070889
1 1.693669 -2.825597 0.389352
1 2.731336 -1.665051 -0.379232
6 2.247677 0.670912 -1.312422
1 3.028107 0.647971 -0.541576
1 2.588569 0.039964 -2.142417
1 2.167344 1.697468 -1.679080
6 -2.584780 1.406820 -0.361036
1 -3.361871 0.926555 -0.959776
1 -2.969397 1.578512 0.646343
1 -2.312721 2.359998 -0.814051
8 1.959846 1.599976 2.138524
1 2.790933 1.540977 1.644761
1 1.292077 1.285842 1.509984
1m5mC/1m5mCaan.xyz
6 1.443447 0.763163 0.265197
6 -0.841492 1.060794 -0.121148
6 -0.965019 -0.209714 -0.785658
6 0.187844 -0.935465 -0.871944
1 0.211868 -1.907523 -1.351317

7 1.371660 -0.487964 -0.361504
7 0.317759 1.510419 0.374684
8 2.554517 1.151574 0.706521
7 -1.922702 1.853472 0.016825
1 -1.836979 2.733283 0.506838
1 -2.835386 1.562782 -0.301024
6 -2.269967 -0.698440 -1.352000
1 -3.041571 -0.775587 -0.576121
1 -2.649859 -0.019896 -2.125748
1 -2.144836 -1.687035 -1.801240
6 2.602997 -1.276270 -0.496101
1 3.332174 -0.729895 -1.098635
1 3.031010 -1.470766 0.489184
1 2.365213 -2.222101 -0.982481
8 -1.645873 -1.638806 2.249397
1 -1.351777 -1.337123 1.375955
1 -1.662876 -2.604186 2.177134
1m5mC/1m5mCaae.xyz
6 0.695195 -0.605023 -0.204376
6 -1.618940 -0.823461 0.008042
6 -1.815436 0.599025 0.077047
6 -0.679453 1.346450 -0.014799
1 -0.704694 2.429651 0.019867
7 0.557924 0.782623 -0.160449
7 -0.408494 -1.382385 -0.129286
8 1.850240 -1.101594 -0.320078
7 -2.676894 -1.654824 0.079557
1 -2.529429 -2.654095 0.044174
1 -3.617409 -1.307804 0.194026
6 -3.177981 1.214943 0.234027
1 -3.837547 0.945242 -0.600158
1 -3.664466 0.879310 1.158039
1 -3.104956 2.305050 0.266991
6 1.758718 1.623287 -0.252974
1 2.357673 1.526497 0.655167
1 2.356173 1.320675 -1.113804

1 1.450131 2.661143 -0.374050
8 4.403910 -0.445382 0.498382
1 3.472814 -0.595423 0.218178
1 4.508133 0.516340 0.494582
1m5mC/1m5mCaap.xyz
6 -0.102326 -1.261319 0.030408
6 0.591683 0.981490 0.031265
6 -0.759249 1.468084 -0.009254
6 -1.724294 0.504807 -0.030692
1 -2.778531 0.755104 -0.058659
7 -1.430112 -0.829807 -0.017512
7 0.882936 -0.327833 0.049047
8 0.142577 -2.492015 0.056280
7 1.625596 1.843724 0.052477
1 2.572503 1.492621 0.101875
1 1.478929 2.842196 0.057915
6 -1.079273 2.936730 -0.023345
1 -0.681892 3.441327 0.865938
1 -0.648798 3.433220 -0.901662
1 -2.160743 3.093383 -0.044311
6 -2.495145 -1.839423 -0.027530
1 -2.362070 -2.511825 -0.877113
1 -2.468163 -2.421553 0.896459
1 -3.455667 -1.332038 -0.112213
8 3.573221 -1.143427 -0.045632
1 2.616230 -0.883341 -0.012990
1 4.024495 -0.370368 -0.413625
1m5mC/1m5mCabq.xyz
6 1.407238 0.830276 0.025521
6 -0.888671 0.872901 -0.406093
6 -0.932308 -0.537922 -0.687088
6 0.258984 -1.191154 -0.566372
1 0.344731 -2.257567 -0.742602
7 1.412060 -0.548595 -0.215754
7 0.239078 1.511157 -0.071213
8 2.490141 1.394506 0.325756

7 -2.017334 1.606523 -0.479045
1 -1.990308 2.589499 -0.245133
1 -2.909778 1.182529 -0.683610
6 -2.206845 -1.243847 -1.057483
1 -2.959303 -1.150651 -0.264434
1 -2.643999 -0.830611 -1.974719
1 -2.021960 -2.308535 -1.221640
6 2.672743 -1.285756 -0.070276
1 3.414497 -0.908058 -0.777178
1 3.056886 -1.169573 0.945378
1 2.485397 -2.340720 -0.268589
8 -1.601012 -0.700083 2.569117
1 -2.146463 -0.005703 2.170706
1 -1.046215 -1.006569 1.835667
1m5mC/1m5mCacb.xyz
6 0.839749 -1.233253 -0.014768
6 -0.764071 0.469106 -0.018281
6 0.238013 1.502905 -0.004613
6 1.528217 1.064643 0.007132
1 2.365285 1.753625 0.019145
7 1.848165 -0.264256 0.004869
7 -0.454711 -0.836639 -0.024689
8 1.173496 -2.447579 -0.022558
7 -2.070418 0.793800 -0.026617
1 -2.781986 0.063075 -0.023488
1 -2.365976 1.758505 -0.016541
6 -0.115054 2.964522 0.002100
1 -0.694565 3.242970 -0.886849
1 -0.716537 3.228399 0.880939
1 0.789937 3.577587 0.018616
6 3.246763 -0.704179 0.036615
1 3.417651 -1.345518 0.903534
1 3.489675 -1.263717 -0.869678
1 3.888630 0.173822 0.102781
8 -4.211382 -1.280777 -0.020311
1 -3.812172 -2.071837 -0.411970

1 -4.379800 -1.522857 0.902406
1m5mC/1m5mCadm.xyz
6 -1.418745 -0.788645 0.232435
6 0.873075 -1.025985 -0.155534
6 0.971840 0.268268 -0.777360
6 -0.197222 0.969604 -0.850047
1 -0.241114 1.955812 -1.298257
7 -1.374440 0.476186 -0.367789
7 -0.277446 -1.514429 0.323899
8 -2.520916 -1.208987 0.666777
7 1.970433 -1.799997 -0.040157
1 1.902457 -2.698167 0.418404
1 2.876101 -1.483971 -0.354054
6 2.268332 0.801758 -1.321982
1 3.035532 0.870005 -0.541051
1 2.664442 0.155815 -2.115240
1 2.124974 1.800877 -1.741398
6 -2.622554 1.240606 -0.482786
1 -3.346662 0.683519 -1.081268
1 -3.044495 1.420173 0.508146
1 -2.409295 2.193656 -0.966159
8 1.566753 1.512861 2.333062
1 1.290209 1.310526 1.425704
1 1.302977 2.434810 2.466438
1m5mC/1m5mCabk.xyz
6 -0.847631 -1.232786 -0.015810
6 0.762584 0.463514 -0.022909
6 -0.235605 1.501032 -0.003289
6 -1.527407 1.067603 0.010732
1 -2.361852 1.759662 0.026136
7 -1.852364 -0.260028 0.006317
7 0.448305 -0.841022 -0.028959
8 -1.186129 -2.445757 -0.023230
7 2.070040 0.783608 -0.037764
1 2.779801 0.051219 -0.038442
1 2.368799 1.747351 -0.028415

6 0.122742 2.961330 0.006126
1 0.730557 3.219962 0.882152
1 0.697800 3.240879 -0.885364
1 -0.779949 3.577530 0.030330
6 -3.252609 -0.694686 0.038416
1 -3.498009 -1.252239 -0.868446
1 -3.425568 -1.336429 0.904620
1 -3.891081 0.185687 0.105911
8 4.217601 -1.291915 -0.038289
1 3.757569 -2.136774 -0.154085
1 4.575848 -1.329428 0.861002
1m5mC/1m5mCaaw.xyz
6 1.391473 0.836545 -0.005369
6 -0.908460 0.858147 -0.415287
6 -0.945721 -0.556956 -0.675682
6 0.250623 -1.200944 -0.555953
1 0.341714 -2.269400 -0.716477
7 1.402680 -0.545827 -0.225778
7 0.218334 1.508697 -0.101922
8 2.473213 1.411705 0.277557
7 -2.043780 1.582028 -0.481900
1 -2.021112 2.568796 -0.264071
1 -2.934381 1.150112 -0.677652
6 -2.219551 -1.276521 -1.021648
1 -2.962321 -1.180253 -0.219844
1 -2.671639 -0.876694 -1.937598
1 -2.028498 -2.341523 -1.176582
6 2.668689 -1.273305 -0.077874
1 3.406161 -0.895536 -0.789104
1 3.054564 -1.147600 0.936056
1 2.488068 -2.330789 -0.268835
8 -1.490363 -0.648876 2.620519
1 -1.940566 0.128858 2.258964
1 -1.057744 -1.044697 1.848619
1m5mC/1m5mCaba.xyz
6 1.234855 0.843789 -0.270356

6 -1.097275 0.769124 -0.384923
6 -1.116539 -0.667130 -0.467550
6 0.109653 -1.264747 -0.472309
1 0.218748 -2.341596 -0.535104
7 1.270020 -0.550431 -0.391599
7 0.036451 1.476346 -0.298057
8 2.321283 1.465141 -0.149581
7 -2.256441 1.456939 -0.389399
1 -2.238473 2.466592 -0.349148
1 -3.147073 0.991518 -0.480973
6 -2.397955 -1.451365 -0.528758
1 -3.026276 -1.254749 0.348773
1 -2.987306 -1.195610 -1.417968
1 -2.189257 -2.523977 -0.559236
6 2.572293 -1.226666 -0.370814
1 3.204656 -0.841112 -1.172828
1 3.070624 -1.059785 0.586892
1 2.411609 -2.294442 -0.516576
8 -0.325393 -0.258789 2.897314
1 0.269887 0.395878 2.503322
1 -0.734676 -0.691550 2.132624
1m5mC/1m5mCacj.xyz
6 1.057858 0.961418 -0.297042
6 -1.260668 0.781170 -0.075855
6 -1.252180 -0.619211 -0.406970
6 -0.024455 -1.142349 -0.687939
1 0.104266 -2.187630 -0.945076
7 1.113891 -0.388261 -0.662542
7 -0.146790 1.523644 -0.033272
8 2.132785 1.611078 -0.231232
7 -2.424962 1.397561 0.209994
1 -2.424309 2.380808 0.443830
1 -3.306318 0.906187 0.187845
6 -2.506899 -1.446563 -0.440553
1 -3.012723 -1.446476 0.532652
1 -3.220560 -1.063468 -1.180516

1 -2.274809 -2.482788 -0.699927
6 2.431474 -1.004539 -0.856676
1 2.964269 -0.506024 -1.668668
1 3.019135 -0.919223 0.060940
1 2.293121 -2.056817 -1.103945
8 0.830086 -0.887304 2.731247
1 1.579715 -0.472374 2.279340
1 0.109490 -0.852553 2.084359
1m5mU/1m5mUacu.xyz
6 -0.890589 0.453513 -0.018326
6 0.924519 -1.231605 -0.009166
6 1.885923 -0.148723 0.006626
6 1.408989 1.124299 0.013673
1 2.076353 1.978185 0.024350
7 0.066038 1.438176 0.008466
7 -0.411398 -0.835610 -0.018846
8 -2.108674 0.706226 -0.041017
8 1.206318 -2.443676 -0.015545
6 3.350814 -0.474354 0.015717
1 3.612001 -1.079021 0.891373
1 3.629458 -1.052926 -0.872159
1 3.950153 0.439336 0.035069
6 -0.390632 2.836244 0.011140
1 -0.899021 3.067903 -0.927195
1 -1.077069 2.998720 0.843621
1 0.478734 3.481329 0.125051
1 -1.114505 -1.570636 -0.037161
8 -3.925218 -1.443895 -0.063057
1 -3.306074 -0.684059 -0.032822
1 -4.046067 -1.702274 0.861506
1m5mU/1m5mUaal.xyz
6 0.883204 0.429997 0.003867
6 -0.954893 -1.230254 -0.000727
6 -1.901337 -0.134171 -0.002059
6 -1.407136 1.132294 0.003040
1 -2.062918 1.995237 0.000549

7 -0.060122 1.427888 0.013214
7 0.386373 -0.852443 0.004458
8 2.104546 0.665941 -0.003430
8 -1.253420 -2.438326 -0.004426
6 -3.370620 -0.439384 -0.008504
1 -3.645014 -1.027189 -0.891591
1 -3.652571 -1.027154 0.872259
1 -3.957269 0.482729 -0.010954
6 0.415912 2.819373 -0.003239
1 1.101810 2.984998 0.828951
1 0.929919 3.029234 -0.943722
1 -0.444779 3.478104 0.097846
1 1.079786 -1.596580 -0.001404
8 3.949624 -1.460301 0.071451
1 4.648991 -1.243894 -0.561027
1 3.321503 -0.709244 0.022362
1m5mU/1m5mUaar.xyz
6 -0.893400 0.450097 0.024659
6 0.928126 -1.228531 0.013827
6 1.885329 -0.142087 -0.008626
6 1.403711 1.129228 -0.015340
1 2.067927 1.985522 -0.030412
7 0.059826 1.438130 -0.004730
7 -0.409404 -0.837569 0.027987
8 -2.111818 0.699385 0.047424
8 1.214372 -2.439557 0.022227
6 3.351499 -0.461734 -0.025142
1 3.637882 -1.036768 0.862550
1 3.610172 -1.067420 -0.900842
1 3.946746 0.454498 -0.050423
6 -0.402584 2.834341 -0.009874
1 -1.089997 2.992479 -0.842412
1 -0.911711 3.065468 0.928180
1 0.464093 3.482779 -0.124993
1 -1.109091 -1.575711 0.049846
8 -3.981093 -1.400108 -0.038601

1 -3.325524 -0.671412 -0.007719
1 -3.451224 -2.199001 -0.171999
1m5mU/1m5mUadc.xyz
6 0.887296 -1.207299 -0.009569
6 -0.915963 0.489557 -0.015100
6 0.090237 1.525365 0.000235
6 1.394155 1.138177 0.009628
1 2.198752 1.863974 0.020933
7 1.801642 -0.176888 0.006296
7 -0.434938 -0.812924 -0.018613
8 1.217077 -2.401252 -0.016301
8 -2.148344 0.696300 -0.025324
6 -0.339295 2.963201 0.005795
1 -0.926093 3.200993 -0.888528
1 -0.970974 3.179622 0.874415
1 0.529572 3.625481 0.035327
6 3.229021 -0.532030 0.016899
1 3.449173 -1.157923 0.883478
1 3.486658 -1.074374 -0.895160
1 3.811749 0.386065 0.073204
1 -1.119276 -1.565395 -0.032443
8 -3.983370 -1.407540 0.019435
1 -3.458944 -2.211891 0.140738
1 -3.323151 -0.679770 0.004460
1m5mU/1m5mUacs.xyz
6 -0.868902 -1.206980 -0.026851
6 0.921234 0.503838 -0.044811
6 -0.092704 1.531336 0.002941
6 -1.393518 1.133969 0.027434
1 -2.203070 1.853555 0.061201
7 -1.790969 -0.184067 0.012816
7 0.449866 -0.802335 -0.053034
8 -1.189564 -2.403377 -0.041362
8 2.151805 0.720045 -0.078475
6 0.325249 2.972551 0.025164
1 0.979359 3.176288 0.880144

1 0.884601 3.234225 -0.880036
1 -0.547750 3.626516 0.092983
6 -3.214962 -0.551370 0.042422
1 -3.485957 -1.073824 -0.877458
1 -3.412257 -1.200769 0.897030
1 -3.804027 0.359825 0.133130
1 1.138947 -1.549981 -0.087487
8 3.912810 -1.452207 0.031047
1 3.315207 -0.674054 -0.018710
1 3.463882 -2.062725 0.633253
1m5mU/1m5mUaay.xyz
6 0.900847 0.463284 -0.064689
6 -0.910212 -1.226244 -0.037767
6 -1.873363 -0.146438 0.020023
6 -1.399430 1.127742 0.032571
1 -2.068179 1.979537 0.074764
7 -0.057892 1.445093 -0.005786
7 0.424403 -0.827183 -0.075922
8 2.117270 0.720643 -0.106966
8 -1.189194 -2.438885 -0.056415
6 -3.336499 -0.476769 0.067412
1 -3.641962 -1.035452 -0.824355
1 -3.567504 -1.102930 0.936404
1 -3.937142 0.434232 0.127601
6 0.397349 2.843095 0.034881
1 1.080779 2.984271 0.873985
1 0.909420 3.099436 -0.894868
1 -0.473096 3.484254 0.161762
1 1.126719 -1.561929 -0.120604
8 3.899251 -1.453227 0.075326
1 3.312440 -0.671699 -0.003016
1 3.402178 -2.071363 0.630144
1m5mU/1m5mUacl.xyz
6 -1.715622 -0.868911 0.006744
6 0.740245 -0.554840 -0.049305
6 0.533529 0.875197 -0.033414

6 -0.749791 1.327734 0.002393
1 -0.971914 2.388532 0.015212
7 -1.851507 0.502305 0.022339
7 -0.414799 -1.324751 -0.031081
8 -2.683641 -1.641869 0.024220
8 1.849903 -1.127667 -0.078672
6 1.724284 1.788849 -0.055962
1 2.362724 1.621349 0.818120
1 2.337759 1.608888 -0.945384
1 1.407962 2.834719 -0.058379
6 -3.218681 1.044861 0.047614
1 -3.765330 0.707505 -0.835157
1 -3.735977 0.704023 0.946462
1 -3.159981 2.131901 0.048174
1 -0.300396 -2.335071 -0.040119
8 4.499145 -0.235125 0.106223
1 4.938998 -1.096261 0.142095
1 3.543263 -0.448504 0.047592
1m5mU/1m5mUadd.xyz
6 1.288800 -0.750902 -0.303758
6 -1.187169 -0.807439 -0.437149
6 -1.187298 0.641103 -0.465110
6 0.014681 1.277169 -0.430327
1 0.081286 2.358609 -0.446065
7 1.225403 0.624146 -0.368553
7 0.072804 -1.396691 -0.380254
8 2.356557 -1.370822 -0.197356
8 -2.203305 -1.525896 -0.459494
6 -2.498594 1.369775 -0.503880
1 -3.077287 1.095111 -1.392667
1 -3.109351 1.118036 0.370596
1 -2.339442 2.451086 -0.513963
6 2.488482 1.370391 -0.266178
1 2.938791 1.210148 0.716050
1 3.179866 1.035120 -1.040867
1 2.274948 2.429359 -0.403189

1 0.109214 -2.411720 -0.335799
8 -0.404379 0.089694 2.914388
1 -0.811614 0.554514 2.167753
1 0.161746 -0.576831 2.497901
1m5mU/1m5mUade.xyz
6 1.387662 -0.770581 0.058306
6 -1.059574 -0.988012 -0.281744
6 -1.088681 0.389556 -0.729873
6 0.088657 1.071761 -0.764794
1 0.130782 2.104609 -1.090681
7 1.298790 0.528990 -0.393899
7 0.197281 -1.465776 0.077799
8 2.456554 -1.284888 0.416327
8 -2.049965 -1.737436 -0.201651
6 -2.403526 1.003796 -1.112824
1 -2.880787 0.438192 -1.920544
1 -3.095656 1.002569 -0.263068
1 -2.265916 2.035713 -1.445384
6 2.543216 1.310912 -0.462673
1 2.974081 1.417363 0.534889
1 3.260162 0.807450 -1.113813
1 2.312192 2.295084 -0.866980
1 0.250875 -2.426502 0.406257
8 -1.121620 0.990921 2.657029
1 -1.126812 1.157471 1.702220
1 -1.117683 1.872185 3.057766
1m5mU/1m5mUadf.xyz
6 -0.702756 -0.586143 -0.081185
6 1.756870 -0.874829 0.012011
6 1.893671 0.566513 0.032783
6 0.757831 1.312179 -0.006065
1 0.793788 2.395338 0.003779
7 -0.511920 0.774914 -0.065616
7 0.446264 -1.341960 -0.050411
8 -1.825830 -1.117503 -0.122441
8 2.698425 -1.688044 0.044055

6 3.267497 1.167445 0.093132
1 3.800910 0.833146 0.989892
1 3.866529 0.862679 -0.772322
1 3.213031 2.258847 0.108538
6 -1.703599 1.638357 -0.107361
1 -2.301135 1.401439 -0.989099
1 -2.304817 1.489087 0.791402
1 -1.376723 2.675522 -0.157762
1 0.312167 -2.349854 -0.061257
8 -4.504887 -0.326215 0.196496
1 -4.550660 0.640152 0.205909
1 -3.552251 -0.524061 0.088339
1m5mU/1m5mUabg.xyz
6 -0.717613 -0.540527 -0.224633
6 1.721987 -0.898228 0.022275
6 1.894352 0.538023 0.085931
6 0.783458 1.315240 -0.012176
1 0.846710 2.396443 0.027529
7 -0.492303 0.813899 -0.168497
7 0.406853 -1.328331 -0.137979
8 -1.850308 -1.037395 -0.350933
8 2.637857 -1.737407 0.099849
6 3.274563 1.100304 0.261537
1 3.729840 0.736027 1.189304
1 3.930253 0.796151 -0.562034
1 3.245537 2.192323 0.295127
6 -1.655158 1.712352 -0.253714
1 -2.264812 1.443626 -1.117171
1 -2.254793 1.636448 0.655392
1 -1.293557 2.732714 -0.369116
1 0.247294 -2.331750 -0.178069
8 -4.422937 -0.210522 0.489083
1 -4.800082 -1.038834 0.816994
1 -3.514673 -0.442511 0.208070
1m5mU/1m5mUabq.xyz
6 1.296803 -0.778116 -0.228208

6 -1.176960 -0.855002 -0.373911
6 -1.182090 0.585889 -0.524437
6 0.016659 1.228975 -0.526243
1 0.080030 2.305818 -0.629675
7 1.228816 0.587307 -0.399602
7 0.083736 -1.432669 -0.256664
8 2.366544 -1.383446 -0.068621
8 -2.190761 -1.576835 -0.346254
6 -2.497057 1.300250 -0.636649
1 -3.064373 0.944919 -1.503912
1 -3.113863 1.119110 0.250959
1 -2.345225 2.377777 -0.738350
6 2.492765 1.338959 -0.373257
1 2.977650 1.222498 0.598233
1 3.158916 0.970317 -1.155423
1 2.273025 2.391013 -0.548039
1 0.123085 -2.439969 -0.124630
8 -0.459991 0.401146 2.889054
1 -0.879862 0.799994 2.112135
1 0.175690 -0.226600 2.515364
1m5mU/1m5mUabw.xyz
6 1.433697 -0.750933 -0.143307
6 -0.984716 -0.692336 -0.687697
6 -0.950076 0.749017 -0.552840
6 0.236903 1.327664 -0.227780
1 0.327003 2.400027 -0.099513
7 1.405643 0.622361 -0.038346
7 0.230822 -1.335531 -0.477066
8 2.459795 -1.420420 0.045477
8 -1.998466 -1.363142 -0.958652
6 -2.218178 1.528021 -0.741951
1 -2.627058 1.375892 -1.747014
1 -2.983385 1.203979 -0.027574
1 -2.040922 2.596729 -0.597469
6 2.659047 1.302910 0.321599
1 2.979176 0.996974 1.319881

1 3.437106 1.049830 -0.400570
1 2.483783 2.377447 0.306759
1 0.238972 -2.349047 -0.553447
8 -1.818476 -0.335694 2.606977
1 -1.174091 0.247919 2.179341
1 -2.298723 -0.739553 1.868927
1m5mU/1m5mUadj.xyz
6 -1.481132 0.744076 0.026040
6 0.933424 0.879150 -0.524877
6 0.948531 -0.561169 -0.677054
6 -0.212368 -1.235094 -0.455761
1 -0.263575 -2.314310 -0.542068
7 -1.396915 -0.625154 -0.105492
7 -0.301104 1.424436 -0.187278
8 -2.534287 1.329637 0.315499
8 1.919563 1.625715 -0.666734
6 2.237827 -1.241512 -1.033027
1 2.614630 -0.892260 -2.000800
1 3.009992 -1.023790 -0.286791
1 2.102304 -2.324736 -1.083784
6 -2.624794 -1.406023 0.108218
1 -3.018956 -1.208356 1.106577
1 -3.374645 -1.133712 -0.637419
1 -2.382170 -2.463303 0.014759
1 -0.349587 2.434104 -0.078592
8 1.974880 -0.350687 2.489128
1 2.478553 0.238130 1.907440
1 1.379420 -0.820630 1.885686
1m5mU/1m5mUadu.xyz
6 -0.906483 0.434877 0.171694
6 0.943698 -1.209806 0.101496
6 1.871246 -0.111323 -0.068527
6 1.363608 1.149286 -0.107636
1 2.004948 2.013882 -0.233286
7 0.019615 1.436074 0.007723
7 -0.395835 -0.841471 0.210025

8 -2.124925 0.663076 0.277783
8 1.256059 -2.413263 0.155336
6 3.336589 -0.410563 -0.192187
1 3.701474 -0.942983 0.693306
1 3.533131 -1.052240 -1.058415
1 3.912584 0.511185 -0.305061
6 -0.474186 2.820442 -0.049335
1 -1.168637 2.933655 -0.883972
1 -0.983864 3.074130 0.882206
1 0.377576 3.483240 -0.190608
1 -1.075921 -1.589848 0.321935
8 -3.871741 -1.467598 -0.332513
1 -3.288780 -0.714915 -0.099727
1 -3.260956 -2.173522 -0.588496
1m5mU/1m5mUabm.xyz
6 -0.520736 1.102581 -0.007581
6 0.044828 -1.300794 -0.009930
6 1.449000 -0.945111 0.003173
6 1.766486 0.377149 0.008272
1 2.797401 0.711608 0.017465
7 0.828612 1.386772 0.003834
7 -0.843887 -0.233797 -0.016494
8 -1.389744 1.990261 -0.010053
8 -0.394839 -2.468880 -0.015554
6 2.479098 -2.037011 0.010263
1 2.358299 -2.682666 0.887426
1 2.380144 -2.674428 -0.875635
1 3.489036 -1.619430 0.025035
6 1.226301 2.802370 0.007931
1 0.839951 3.299875 -0.883847
1 0.832480 3.297911 0.897549
1 2.314042 2.856381 0.012749
1 -1.852804 -0.450404 -0.028674
8 -3.682659 -0.710334 -0.082996
1 -3.925271 -1.399702 0.553384
1 -4.058280 0.106544 0.279219

1m5mU/1m5mUaci.xyz
6 0.717110 -0.542945 -0.222165
6 -1.723071 -0.897432 0.023557
6 -1.893914 0.539143 0.084037
6 -0.782032 1.314916 -0.014341
1 -0.844097 2.396265 0.023161
7 0.493293 0.811842 -0.168484
7 -0.408304 -1.329329 -0.134966
8 1.849398 -1.041199 -0.346692
8 -2.639891 -1.735462 0.102266
6 -3.273683 1.103390 0.256840
1 -3.928816 0.797933 -0.566692
1 -3.730344 0.741848 1.184991
1 -3.243514 2.195460 0.287755
6 1.657412 1.708584 -0.254310
1 2.258068 1.630687 0.654002
1 2.265519 1.439887 -1.118845
1 1.297319 2.729660 -0.367963
1 -0.249873 -2.333019 -0.172748
8 4.414157 -0.203199 0.508547
1 3.512028 -0.442930 0.214601
1 4.868539 -1.048442 0.631215
1m5mU/1m5mUaca.xyz
6 0.951039 -0.821105 -0.546329
6 -1.468749 -0.791579 -0.005614
6 -1.447004 0.651455 -0.126277
6 -0.269233 1.242902 -0.459880
1 -0.186444 2.318308 -0.566271
7 0.897399 0.547089 -0.698119
7 -0.250497 -1.422265 -0.240837
8 1.997522 -1.473011 -0.675918
8 -2.469192 -1.473370 0.285369
6 -2.711462 1.421625 0.117746
1 -3.499180 1.111122 -0.577829
1 -3.087403 1.245548 1.131813
1 -2.541857 2.493977 -0.007912

6 2.168113 1.252905 -0.926745
1 2.835277 1.103664 -0.074434
1 2.645948 0.873561 -1.831181
1 1.956559 2.314518 -1.045492
1 -0.223033 -2.432890 -0.135249
8 1.799737 0.277650 2.566356
1 2.341991 -0.186080 1.910854
1 1.269064 0.897131 2.044534
1m5mU/1m5mUacv.xyz
6 1.292310 0.779330 -0.240656
6 -1.182134 0.845970 -0.388696
6 -1.180746 -0.596696 -0.525016
6 0.021550 -1.234268 -0.527865
1 0.089010 -2.311235 -0.627604
7 1.231069 -0.586979 -0.409342
7 0.076085 1.427703 -0.266034
8 2.359391 1.390241 -0.086173
8 -2.198584 1.564176 -0.374127
6 -2.491800 -1.317271 -0.643490
1 -3.125175 -1.118552 0.228259
1 -3.044358 -0.982641 -1.528499
1 -2.335283 -2.396282 -0.719178
6 2.498024 -1.333433 -0.378173
1 3.166320 -0.960332 -1.156254
1 2.977493 -1.216971 0.596042
1 2.283378 -2.385964 -0.556452
1 0.111864 2.436251 -0.142752
8 -0.370680 -0.243011 2.866399
1 -0.722512 -0.628569 2.049743
1 -0.615062 -0.873808 3.558901
1m5mU/1m5mUaco.xyz
6 1.731462 0.847110 -0.004936
6 -0.732350 0.595287 0.029565
6 -0.561640 -0.839828 0.022250
6 0.710517 -1.324305 0.002152
1 0.906135 -2.390345 -0.003433

7 1.832516 -0.527073 -0.011931
7 0.442255 1.335523 0.018373
8 2.719025 1.594944 -0.017292
8 -1.826264 1.198162 0.045694
6 -1.772469 -1.727357 0.033806
1 -2.392406 -1.532408 0.915606
1 -2.396605 -1.548957 -0.848612
1 -1.477811 -2.779526 0.042462
6 3.185212 -1.104542 -0.028032
1 3.716889 -0.776970 -0.923439
1 3.735085 -0.782224 0.858392
1 3.097627 -2.189510 -0.029976
1 0.353201 2.348419 0.021393
8 -4.488096 0.374134 -0.067489
1 -3.526571 0.568063 -0.031644
1 -4.540652 -0.591807 -0.061976
1mC/1mCabu.xyz
6 0.484348 -0.390043 -0.000947
6 -1.113350 1.305887 0.001680
6 -2.184238 0.360137 0.002193
6 -1.842177 -0.956532 0.000306
1 -2.580210 -1.749747 0.000378
7 -0.535965 -1.348760 -0.001667
7 0.172356 0.925693 -0.000096
8 1.676695 -0.798606 -0.001392
7 -1.378527 2.625174 0.002749
1 -0.621562 3.295085 0.004027
1 -2.331737 2.959106 0.005358
1 -3.220697 0.672599 0.003788
6 -0.169594 -2.770563 -0.004011
1 0.422492 -3.003627 -0.891184
1 0.415389 -3.008844 0.886576
1 -1.082588 -3.365365 -0.009500
8 3.783672 0.958688 0.033322
1 3.481881 1.826310 -0.269443
1 2.979111 0.385762 0.012329

1mC/1mCacx.xyz
6 -0.397309 -0.811337 -0.103918
6 0.792252 1.210706 -0.053957
6 -0.412443 1.966891 0.071595
6 -1.579144 1.266417 0.103518
1 -2.542028 1.753995 0.200575
7 -1.597640 -0.094435 0.011009
7 0.776268 -0.127540 -0.142337
8 -0.439762 -2.061210 -0.170239
7 1.987262 1.826033 -0.083530
1 2.835095 1.284601 -0.185973
1 2.050957 2.832668 -0.032241
1 -0.400617 3.046970 0.142037
6 -2.856219 -0.849709 0.073361
1 -2.946389 -1.494433 -0.802413
1 -2.878502 -1.466129 0.974984
1 -3.687115 -0.145046 0.092879
8 3.077296 -1.687307 0.205608
1 2.257878 -1.147428 0.056930
1 2.766399 -2.603279 0.230693
1mC/1mCabw.xyz
6 0.484297 -0.388898 0.001030
6 -1.115099 1.305362 0.002933
6 -2.185055 0.358575 0.002453
6 -1.841683 -0.957749 -0.001221
1 -2.578937 -1.751693 -0.002468
7 -0.535055 -1.348643 -0.003303
7 0.170983 0.926467 0.001742
8 1.677078 -0.796438 0.003778
7 -1.381519 2.624405 0.004190
1 -0.625120 3.294954 0.007610
1 -2.335029 2.957469 0.007745
1 -3.221830 0.669995 0.004132
6 -0.167316 -2.770077 -0.008329
1 0.434779 -2.997852 -0.890030
1 0.408055 -3.012599 0.887452

1 -1.079608 -3.365670 -0.027291
8 3.777301 0.969829 0.056539
1 3.551116 1.731818 -0.494707
1 2.979821 0.387572 0.025411
1mC/1mCabs.xyz
6 -0.507389 0.286658 -0.167054
6 1.304562 -1.171261 -0.024267
6 2.218488 -0.090377 0.168816
6 1.694242 1.164923 0.177802
1 2.305348 2.049479 0.311965
7 0.356137 1.374117 0.016899
7 -0.012426 -0.971577 -0.182835
8 -1.735863 0.528896 -0.313573
7 1.756573 -2.438610 -0.051132
1 1.112726 -3.205043 -0.190365
1 2.739665 -2.639587 0.064152
1 3.280283 -0.258076 0.297378
6 -0.205589 2.731238 0.006335
1 -0.685050 2.929956 -0.954356
1 -0.944397 2.836865 0.803120
1 0.603657 3.443722 0.164008
8 -3.639003 -1.374917 0.209643
1 -4.212474 -0.949108 0.861867
1 -2.928691 -0.714638 0.023364
1mC/1mCaaa.xyz
6 0.418969 -0.818073 0.100138
6 -0.801136 1.185812 0.053992
6 0.391981 1.960834 -0.065430
6 1.569250 1.278460 -0.097671
1 2.524778 1.780998 -0.190310
7 1.608262 -0.082172 -0.010816
7 -0.765091 -0.152582 0.137453
8 0.481676 -2.067281 0.163759
7 -2.005533 1.782771 0.081945
1 -2.845226 1.228047 0.181024
1 -2.083848 2.788647 0.035416

1 0.363456 3.040870 -0.131470
6 2.878793 -0.816846 -0.076525
1 2.981907 -1.460611 0.798581
1 2.908510 -1.432673 -0.978336
1 3.697586 -0.098289 -0.098465
8 -3.111433 -1.644635 -0.200343
1 -2.262304 -1.150721 -0.058799
1 -2.857413 -2.578182 -0.212061
1mC/1mCabq.xyz
6 -0.459836 -0.833130 -0.067635
6 0.814795 1.136823 -0.038635
6 -0.358004 1.947428 0.035171
6 -1.554665 1.299545 0.057761
1 -2.497718 1.829694 0.117703
7 -1.629523 -0.060907 0.002904
7 0.743951 -0.202455 -0.090998
8 -0.560448 -2.080740 -0.107420
7 2.034827 1.701941 -0.057944
1 2.863135 1.122447 -0.090829
1 2.137614 2.705243 -0.002963
1 -0.300129 3.027496 0.076557
6 -2.920914 -0.758502 0.058206
1 -3.034677 -1.401759 -0.816226
1 -2.975190 -1.371691 0.960560
1 -3.718975 -0.016884 0.072171
8 3.128547 -1.648485 0.232096
1 2.269582 -1.175741 0.080939
1 3.538531 -1.708049 -0.642252
1mC/1mCaby.xyz
6 0.410243 -0.814433 -0.108559
6 -0.798746 1.195893 -0.055305
6 0.398340 1.963297 0.074387
6 1.571644 1.274122 0.106881
1 2.529607 1.770609 0.207133
7 1.603294 -0.086139 0.011168
7 -0.769979 -0.142044 -0.147870

8 0.465919 -2.063630 -0.177920
7 -1.999320 1.800324 -0.085217
1 -2.842370 1.251404 -0.187535
1 -2.072126 2.806031 -0.027632
1 0.375687 3.042989 0.147962
6 2.869138 -0.828846 0.077444
1 2.893606 -1.446923 0.977893
1 2.969728 -1.471055 -0.799047
1 3.692443 -0.115546 0.102311
8 -3.094835 -1.671357 0.185250
1 -2.796460 -2.550366 0.458164
1 -2.260459 -1.152436 0.046458
1mC/1mCacg.xyz
6 0.453228 -0.827277 -0.105103
6 -0.817854 1.144733 -0.058132
6 0.354624 1.949836 0.066819
6 1.548980 1.298220 0.101700
1 2.491362 1.824161 0.198479
7 1.622563 -0.060551 0.012862
7 -0.748083 -0.192731 -0.145706
8 0.551420 -2.074034 -0.171077
7 -2.036271 1.712501 -0.088882
1 -2.862788 1.137261 -0.182887
1 -2.138259 2.715777 -0.031882
1 0.298045 3.028575 0.135468
6 2.911171 -0.761720 0.086223
1 2.948487 -1.382230 0.984557
1 3.039535 -1.397856 -0.791189
1 3.710314 -0.021932 0.120636
8 -3.129387 -1.655019 0.110249
1 -3.165817 -1.933108 1.036226
1 -2.265502 -1.175505 0.020255
1mC/1mCadu.xyz
6 -0.446352 0.825340 0.106264
6 0.814374 -1.153157 0.056208
6 -0.362379 -1.951901 -0.069434

6 -1.553295 -1.293965 -0.102642
1 -2.498372 -1.814990 -0.199829
7 -1.619814 0.065067 -0.011623
7 0.751547 0.184426 0.145732
8 -0.537474 2.072614 0.173146
7 2.029746 -1.727381 0.086559
1 2.859752 -1.156572 0.176179
1 2.126856 -2.730738 0.022974
1 -0.311440 -3.030812 -0.139855
6 -2.904822 0.773114 -0.083293
1 -3.028355 1.410272 0.794048
1 -2.940359 1.393294 -0.981931
1 -3.707904 0.037512 -0.115926
8 3.133540 1.641861 -0.120951
1 2.267000 1.168352 -0.024777
1 3.048784 2.156514 -0.935739
1mC/1mCact.xyz
6 -0.310689 -0.405687 -0.451069
6 1.957399 -0.423965 0.088684
6 1.939446 0.966879 0.412906
6 0.753017 1.616704 0.268370
1 0.639242 2.672789 0.482632
7 -0.365501 0.963690 -0.161225
7 0.858641 -1.072701 -0.324821
8 -1.367318 -0.980239 -0.829726
7 3.100001 -1.127361 0.192541
1 3.111845 -2.111781 -0.036493
1 3.951393 -0.685823 0.509433
1 2.827205 1.485192 0.752495
6 -1.638831 1.675953 -0.333903
1 -2.010823 1.526232 -1.348849
1 -2.380067 1.306263 0.377109
1 -1.468303 2.738199 -0.161606
8 -3.545241 -1.203966 0.868714
1 -3.944603 -0.330813 0.986875
1 -2.779447 -1.051310 0.271101

1mC/1mCaac.xyz
6 -0.265876 -0.495303 0.194916
6 2.050849 -0.408947 -0.059748
6 2.032833 1.014794 -0.176528
6 0.818972 1.622379 -0.088086
1 0.699609 2.697095 -0.157388
7 -0.324756 0.900865 0.094348
7 0.930211 -1.123191 0.118885
8 -1.338291 -1.133633 0.364424
7 3.216669 -1.078351 -0.126529
1 3.228038 -2.084498 -0.031035
1 4.090026 -0.586629 -0.251647
1 2.939799 1.588066 -0.320501
6 -1.631176 1.565337 0.188554
1 -2.248330 1.311267 -0.675692
1 -2.140553 1.249837 1.100207
1 -1.471247 2.642776 0.213703
8 -3.991824 -0.936876 -0.376628
1 -4.202795 -0.024162 -0.617773
1 -3.042102 -0.914487 -0.123824
1mC/1mCaae.xyz
6 -0.308533 -0.409432 0.434974
6 1.963245 -0.430674 -0.089626
6 1.953101 0.963876 -0.398323
6 0.768370 1.616798 -0.254146
1 0.660128 2.675728 -0.457072
7 -0.355493 0.963645 0.161051
7 0.859308 -1.079539 0.309397
8 -1.369723 -0.984429 0.799204
7 3.103576 -1.137517 -0.195303
1 3.111811 -2.123233 0.028233
1 3.960009 -0.693913 -0.495173
1 2.845170 1.482443 -0.726029
6 -1.627023 1.679109 0.332351
1 -2.361653 1.327219 -0.394370
1 -2.011827 1.511076 1.339557

1 -1.449342 2.743617 0.182879
8 -3.620542 -1.177648 -0.802910
1 -2.819223 -1.027048 -0.253076
1 -3.759640 -0.343459 -1.272705
1mC/1mCacd.xyz
6 -0.306813 -0.412998 -0.432006
6 1.965694 -0.427264 0.089537
6 1.951815 0.967404 0.397534
6 0.765236 1.616957 0.253411
1 0.654271 2.675783 0.455359
7 -0.357134 0.960417 -0.160569
7 0.863075 -1.079634 -0.307417
8 -1.367025 -0.991781 -0.793263
7 3.108267 -1.130670 0.193891
1 3.119265 -2.116261 -0.030072
1 3.964089 -0.684210 0.491235
1 2.842622 1.488747 0.724250
6 -1.630451 1.672587 -0.332528
1 -2.015973 1.500626 -1.338785
1 -2.363389 1.321485 0.396275
1 -1.454867 2.737935 -0.186551
8 -3.609196 -1.177362 0.819442
1 -3.894738 -0.291174 1.081960
1 -2.813857 -1.030690 0.259863
1mC/1mCadf.xyz
6 -0.314978 -0.390010 -0.434734
6 1.957554 -0.448655 0.082467
6 1.967791 0.942844 0.404519
6 0.791788 1.613772 0.271508
1 0.698966 2.672020 0.485443
7 -0.342394 0.980166 -0.145618
7 0.843419 -1.078093 -0.319481
8 -1.385092 -0.945802 -0.803849
7 3.088453 -1.172122 0.176988
1 3.081774 -2.156537 -0.052282
1 3.950745 -0.743969 0.482544

1 2.868428 1.445282 0.734043
6 -1.604995 1.714175 -0.306072
1 -1.990321 1.567926 -1.316579
1 -2.344001 1.359006 0.414422
1 -1.414220 2.773796 -0.139176
8 -3.641619 -1.017748 0.805566
1 -2.835342 -0.958603 0.245381
1 -3.711648 -1.952940 1.043117
1mC/1mCadv.xyz
6 0.336145 1.029971 -0.006309
6 2.322808 -0.195858 0.001700
6 1.614811 -1.436880 0.005310
6 0.255842 -1.369306 0.003414
1 -0.368141 -2.255518 0.006521
7 -0.393797 -0.170673 -0.003193
7 1.692106 0.986140 -0.003790
8 -0.299488 2.112855 -0.010293
7 3.670186 -0.190623 -0.002907
1 4.172189 0.685907 0.031693
1 4.189660 -1.055501 0.042756
1 2.132118 -2.387839 0.009897
6 -1.860309 -0.097972 -0.003832
1 -2.208489 0.431585 0.885482
1 -2.207625 0.431553 -0.893073
1 -2.259200 -1.111955 -0.003235
8 -5.303600 -0.366307 0.004990
1 -4.756142 0.380290 0.289333
1 -4.664904 -1.044549 -0.259419
1mC/1mCabx.xyz
6 -0.907955 -0.829036 -0.480691
6 1.293798 -0.756421 0.292031
6 0.983651 0.286041 1.222073
6 -0.308771 0.717051 1.255093
1 -0.641028 1.498024 1.929117
7 -1.253335 0.183225 0.433778
7 0.369752 -1.284010 -0.521503

8 -1.805050 -1.279087 -1.232914
7 2.550191 -1.233744 0.214399
1 2.778183 -1.956976 -0.453952
1 3.282223 -0.857284 0.799532
1 1.733669 0.706378 1.879931
6 -2.640201 0.663824 0.454249
1 -2.908205 1.073390 -0.521800
1 -3.317191 -0.158778 0.692907
1 -2.730074 1.441468 1.212370
8 1.392157 2.394929 -1.413181
1 1.666703 3.276963 -1.123452
1 1.249472 1.903032 -0.589137
1mC/1mCaaj.xyz
6 0.465877 -0.830126 -0.083011
6 -0.823618 1.129892 -0.050009
6 0.342445 1.948121 0.045455
6 1.543541 1.308627 0.075526
1 2.482083 1.844790 0.150811
7 1.629073 -0.050686 0.008673
7 -0.741694 -0.207647 -0.117359
8 0.574624 -2.076815 -0.132007
7 -2.048014 1.686213 -0.069145
1 -2.870764 1.104763 -0.156263
1 -2.157132 2.689978 -0.040485
1 0.276466 3.027306 0.096322
6 2.924702 -0.739024 0.077793
1 2.974232 -1.351440 0.981027
1 3.052194 -1.381839 -0.794974
1 3.717267 0.008266 0.099642
8 -3.083694 -1.740475 0.143492
1 -2.244247 -1.222217 0.039562
1 -3.750784 -1.081390 0.382771
1mC/1mCabz.xyz
6 -1.655473 -0.067196 0.157673
6 0.137884 -1.541550 -0.089014
6 1.032104 -0.477029 -0.420033

6 0.513765 0.781010 -0.427331
1 1.112467 1.653955 -0.660008
7 -0.799071 1.007499 -0.137041
7 -1.156069 -1.328555 0.184168
8 -2.863720 0.180429 0.392211
7 0.592873 -2.808759 -0.044518
1 -0.030616 -3.565610 0.199697
1 1.562444 -3.015592 -0.237593
1 2.074640 -0.657358 -0.648753
6 -1.353532 2.367374 -0.134893
1 -2.133091 2.457519 -0.894410
1 -1.783763 2.592648 0.842935
1 -0.551212 3.072200 -0.351258
8 4.055403 1.077674 0.327916
1 3.177293 1.100908 0.735854
1 4.525750 1.829556 0.715838
1mC/1mCadl.xyz
6 0.591262 1.276125 -0.190191
6 1.710214 -0.764153 -0.010729
6 0.561977 -1.400952 0.553295
6 -0.548422 -0.632207 0.718711
1 -1.467295 -1.023205 1.138567
7 -0.556720 0.684118 0.362945
7 1.710333 0.528558 -0.363249
8 0.538680 2.489353 -0.509347
7 2.842029 -1.468813 -0.204207
1 3.654244 -1.022291 -0.607036
1 2.890898 -2.445881 0.046517
1 0.570779 -2.445328 0.838006
6 -1.760216 1.509672 0.517976
1 -1.538775 2.377538 1.142209
1 -2.106813 1.853897 -0.459034
1 -2.538566 0.911864 0.991962
8 -3.445049 -1.716680 -0.668123
1 -4.280050 -1.752482 -1.156732
1 -3.231861 -0.773460 -0.617495

1mC/1mCadr.xyz
6 -1.509780 0.755767 0.000553
6 0.822155 0.615742 -0.000114
6 0.783184 -0.815086 0.001616
6 -0.446100 -1.397490 0.001555
1 -0.581304 -2.472890 0.002393
7 -1.585356 -0.647162 0.000233
7 -0.295346 1.358127 -0.000045
8 -2.583364 1.408220 0.001026
7 2.006681 1.252400 -0.001487
1 2.028166 2.262749 -0.004749
1 2.877472 0.724229 -0.005286
1 1.688517 -1.408579 0.002952
6 -2.910576 -1.277109 -0.001489
1 -3.468808 -0.978968 0.888554
1 -3.468530 -0.973481 -0.889808
1 -2.781218 -2.358944 -0.004903
8 4.354915 -0.601572 -0.007101
1 4.081443 -1.177949 -0.736542
1 4.136699 -1.103840 0.792360
1mU/1mUacm.xyz
6 0.800927 0.910264 -0.014072
6 -0.920085 -0.872468 -0.014338
6 0.144198 -1.835199 0.002146
6 1.432701 -1.403266 0.014146
1 2.266503 -2.095151 0.025998
7 1.772718 -0.074101 0.012322
7 -0.501093 0.453558 -0.020374
8 1.073199 2.115963 -0.031160
8 -2.141132 -1.137514 -0.024742
1 -0.094537 -2.889832 0.006271
6 3.177793 0.360442 0.026785
1 3.421350 0.873798 -0.905739
1 3.344979 1.037731 0.865705
1 3.809032 -0.519637 0.136800
1 -1.219760 1.173406 -0.039142

8 -4.020063 0.946192 -0.063617
1 -3.372179 0.209451 -0.024397
1 -4.166004 1.198263 0.859013
1mU/1mUadf.xyz
6 -0.835772 -0.913191 -0.007640
6 0.917612 0.837948 -0.008167
6 -0.129072 1.820042 -0.004082
6 -1.425176 1.411934 0.003219
1 -2.246533 2.118533 0.006586
7 -1.789307 0.089187 0.007294
7 0.474666 -0.480368 -0.010202
8 -1.130729 -2.113584 -0.017676
8 2.142811 1.081181 -0.010628
1 0.129226 2.870030 -0.004843
6 -3.202315 -0.318991 0.017208
1 -3.447759 -0.843013 -0.908822
1 -3.389233 -0.979039 0.865660
1 -3.817856 0.574440 0.107333
1 1.180190 -1.212905 -0.019205
8 4.054815 -0.972463 0.027966
1 3.394326 -0.246144 0.011565
1 4.913289 -0.531169 -0.038438
1mU/1mUack.xyz
6 0.829435 -0.912790 -0.006077
6 -0.917868 0.844346 -0.011526
6 0.132140 1.822837 -0.004781
6 1.426845 1.410251 0.004840
1 2.250566 2.114110 0.009412
7 1.786426 0.086264 0.009708
7 -0.479457 -0.475451 -0.011319
8 1.120143 -2.114228 -0.014775
8 -2.142316 1.091709 -0.018521
1 -0.122499 2.873724 -0.006199
6 3.197969 -0.326886 0.020918
1 3.381057 -0.989985 0.867803
1 3.443182 -0.849194 -0.906164

1 3.816513 0.564087 0.114590
1 -1.187502 -1.205623 -0.019719
8 -4.042091 -0.972534 -0.023283
1 -4.837983 -0.609471 0.389991
1 -3.389126 -0.239464 -0.006050
1mU/1mUaab.xyz
6 -1.471880 -0.750630 0.000011
6 0.990747 -0.489472 -0.000022
6 0.793648 0.932641 -0.000024
6 -0.471663 1.429860 -0.000008
1 -0.665230 2.496026 -0.000009
7 -1.584993 0.628791 0.000014
7 -0.180785 -1.236785 -0.000026
8 -2.455834 -1.498916 0.000035
8 2.092250 -1.079852 -0.000025
1 1.654673 1.587244 -0.000037
6 -2.944174 1.192174 -0.000001
1 -3.480436 0.860807 -0.891130
1 -3.480442 0.860847 0.891138
1 -2.867105 2.277935 -0.000026
1 -0.090860 -2.249757 -0.000014
8 4.393738 0.475631 0.000041
1 3.556406 -0.040835 0.000009
1 4.112132 1.401356 0.000017
1mU/1mUaba.xyz
6 0.790352 -0.907602 0.005837
6 -0.922929 0.883359 0.009853
6 0.146200 1.841054 0.009664
6 1.432657 1.403052 0.001103
1 2.269727 2.091113 -0.000012
7 1.766657 0.072261 -0.007558
7 -0.509446 -0.444711 0.009292
8 1.056402 -2.114816 0.013309
8 -2.142621 1.153534 0.010941
1 -0.087365 2.896849 0.013474
6 3.169754 -0.368755 -0.020987

1 3.408054 -0.891084 0.907900
1 3.336558 -1.039601 -0.865114
1 3.805507 0.509217 -0.121251
1 -1.230722 -1.161882 0.013630
8 -4.043480 -0.917110 -0.057758
1 -3.577148 -1.686017 0.300381
1 -3.383697 -0.190959 -0.025897
1mU/1mUacz.xyz
6 0.925355 -0.762682 0.450047
6 -1.356402 -0.672691 -0.521651
6 -0.902188 0.411778 -1.351596
6 0.381667 0.844354 -1.245050
1 0.759875 1.662443 -1.846451
7 1.286589 0.286263 -0.377003
7 -0.376394 -1.197249 0.320354
8 1.713981 -1.281609 1.249473
8 -2.505452 -1.151119 -0.503685
1 -1.593653 0.875175 -2.041848
6 2.668536 0.780206 -0.275791
1 2.859996 1.139882 0.736841
1 3.367018 -0.024359 -0.512874
1 2.797948 1.596896 -0.984156
1 -0.646476 -1.964912 0.929972
8 -1.785628 1.972735 1.671395
1 -1.304384 2.013688 0.831440
1 -2.296699 1.152241 1.610404
1mU/1mUado.xyz
6 0.205872 0.913839 -0.001677
6 2.471667 -0.101777 0.002124
6 1.834773 -1.391775 -0.000179
6 0.478860 -1.469214 -0.002821
1 -0.039348 -2.420560 -0.004244
7 -0.331563 -0.360867 -0.003750
7 1.583392 0.973960 0.001197
8 -0.497252 1.932045 -0.002312
8 3.698418 0.111195 0.004873

1 2.446011 -2.283928 0.000572
6 -1.798195 -0.475596 -0.005134
1 -2.206350 0.004845 0.885912
1 -2.204763 0.003283 -0.897778
1 -2.062449 -1.531487 -0.004464
1 1.983809 1.908665 0.002623
8 -5.126624 -0.234091 0.013450
1 -4.460527 0.468462 -0.012065
1 -5.973383 0.233011 -0.034652
1mU/1mUach.xyz
6 -0.406889 0.490002 -0.084391
6 0.988491 -1.558058 -0.015700
6 2.140588 -0.702552 0.089542
6 1.976332 0.645193 0.093160
1 2.817098 1.324682 0.165732
7 0.743507 1.246314 0.000685
7 -0.226335 -0.873454 -0.095622
8 -1.533797 1.007952 -0.146588
8 0.995964 -2.801636 -0.039100
1 3.124344 -1.146146 0.160213
6 0.593296 2.710856 0.017364
1 0.078182 3.038703 -0.887048
1 0.018324 3.014783 0.894265
1 1.585203 3.157610 0.057333
1 -1.073814 -1.432547 -0.161734
8 -3.822134 -0.624290 0.043622
1 -3.031489 -0.048268 -0.018423
1 -4.009215 -0.687696 0.990898
1mU/1mUaan.xyz
6 1.470308 -0.761015 -0.003686
6 -0.986402 -0.447488 0.012146
6 -0.758756 0.969880 0.011959
6 0.516805 1.440072 0.004071
1 0.732581 2.501951 0.003535
7 1.613156 0.615732 -0.002806
7 0.169107 -1.219442 0.004496

8 2.437789 -1.530553 -0.010917
8 -2.100112 -1.014356 0.018496
1 -1.605317 1.642608 0.017232
6 2.984214 1.150084 -0.007396
1 3.516414 0.805461 0.881124
1 3.509749 0.808835 -0.901143
1 2.930795 2.237384 -0.005026
1 0.057577 -2.230263 0.003932
8 -4.377276 0.587218 0.005542
1 -3.564727 0.033023 0.007375
1 -5.093132 -0.020703 -0.226384
1mU/1mUacq.xyz
6 -0.378263 -0.837589 -0.065259
6 0.734548 1.368688 -0.035872
6 -0.570002 1.971524 0.043114
6 -1.672016 1.177961 0.066054
1 -2.672391 1.589863 0.129134
7 -1.601582 -0.191875 0.008541
7 0.728387 -0.021475 -0.094315
8 -0.285668 -2.073061 -0.103512
8 1.820887 1.982439 -0.053264
1 -0.655232 3.048871 0.085789
6 -2.807978 -1.030472 0.054691
1 -2.838360 -1.684069 -0.818682
1 -2.805715 -1.637724 0.962414
1 -3.682730 -0.381891 0.052538
1 1.640802 -0.504341 -0.110152
8 3.251847 -1.401045 0.010495
1 3.878652 -0.795954 0.435178
1 3.113081 -2.108636 0.658077
1mU/1mUabw.xyz
6 0.301865 -0.414661 0.418459
6 -2.114588 -0.463911 -0.140939
6 -2.027344 0.949579 -0.395588
6 -0.842008 1.588739 -0.224316
1 -0.735416 2.652794 -0.398693

7 0.302570 0.944894 0.182360
7 -0.909550 -1.046603 0.256841
8 1.320721 -1.037516 0.757249
8 -3.134634 -1.168439 -0.244878
1 -2.911423 1.485851 -0.712453
6 1.569240 1.675063 0.355392
1 2.003893 1.423228 1.323162
1 2.268977 1.410393 -0.439304
1 1.357278 2.742344 0.317285
1 -0.912338 -2.046713 0.441785
8 3.697096 -1.030236 -0.805876
1 3.904825 -0.122384 -1.068668
1 2.884608 -0.952877 -0.267536
1mU/1mUaas.xyz
6 0.265568 -0.493221 -0.136769
6 -2.207151 -0.431162 0.059270
6 -2.110381 1.002846 0.125405
6 -0.891102 1.595154 0.053826
1 -0.772860 2.671178 0.096302
7 0.278641 0.885164 -0.077424
7 -0.977842 -1.079808 -0.072304
8 1.297716 -1.173414 -0.241669
8 -3.255234 -1.099908 0.106774
1 -3.012556 1.590517 0.226253
6 1.583148 1.564024 -0.139453
1 2.169587 1.328647 0.750727
1 2.122689 1.240529 -1.030424
1 1.407802 2.637358 -0.187406
1 -0.988380 -2.095630 -0.116844
8 4.058197 -0.813594 0.258036
1 3.097071 -0.856506 0.081862
1 4.225135 0.115898 0.468837
1mU/1mUaae.xyz
6 -0.797177 -0.907786 -0.024806
6 0.922757 0.876695 -0.025499
6 -0.142824 1.838130 -0.002036

6 -1.430804 1.404934 0.016207
1 -2.265129 2.095994 0.036758
7 -1.769780 0.075345 0.011039
7 0.504322 -0.449773 -0.038906
8 -1.068000 -2.113823 -0.043715
8 2.143211 1.142764 -0.035675
1 0.094856 2.892982 0.006123
6 -3.173826 -0.361453 0.048527
1 -3.430618 -0.879239 -0.877951
1 -3.326662 -1.035608 0.892771
1 -3.804992 0.517949 0.164363
1 1.222323 -1.169680 -0.065183
8 4.068351 -0.903063 0.052496
1 3.559082 -1.719436 0.157911
1 3.392081 -0.192120 0.021069
1mU/1mUacf.xyz
6 -0.419969 1.059919 0.054535
6 -2.425740 -0.388962 -0.153515
6 -1.564162 -1.521892 0.056622
6 -0.233848 -1.324697 0.246861
1 0.448807 -2.150201 0.409828
7 0.342607 -0.078160 0.248749
7 -1.768202 0.841410 -0.133001
8 0.068753 2.196589 0.049867
8 -3.655921 -0.427217 -0.341306
1 -1.988383 -2.516621 0.059278
6 1.787066 0.101751 0.457389
1 2.219696 0.631034 -0.393978
1 1.963990 0.677147 1.368058
1 2.245318 -0.881710 0.551073
1 -2.332624 1.674462 -0.278630
8 5.409249 -0.367669 -0.439746
1 5.536864 -1.299947 -0.211786
1 4.448763 -0.247257 -0.435951
1mU/1mUact.xyz
6 0.316873 -0.372527 0.435542

6 -2.093782 -0.503753 -0.131734
6 -2.045421 0.905157 -0.418716
6 -0.878309 1.581277 -0.263366
1 -0.801170 2.643349 -0.463368
7 0.282953 0.978313 0.157970
7 -0.874129 -1.043241 0.282687
8 1.350261 -0.954535 0.802743
8 -3.093522 -1.238590 -0.221429
1 -2.943913 1.408341 -0.749032
6 1.528391 1.745326 0.327484
1 1.918107 1.591641 1.334835
1 2.271101 1.421547 -0.403524
1 1.304157 2.800307 0.179821
1 -0.850036 -2.038160 0.492704
8 3.692086 -0.905103 -0.812128
1 3.794403 -1.832211 -1.069547
1 2.888476 -0.887377 -0.255231
1mU/1mUabd.xyz
6 -0.700348 0.988636 0.412182
6 1.646978 0.506424 -0.236296
6 1.133948 -0.471228 -1.159382
6 -0.207907 -0.664805 -1.252931
1 -0.633279 -1.396101 -1.929920
7 -1.117136 0.043769 -0.508923
7 0.663039 1.179250 0.488467
8 -1.492917 1.624654 1.117783
8 2.847043 0.778521 -0.047152
1 1.825410 -1.047358 -1.758808
6 -2.562584 -0.202902 -0.619796
1 -2.956972 -0.536989 0.341674
1 -3.073259 0.713004 -0.922997
1 -2.724039 -0.975593 -1.369626
1 0.973181 1.872461 1.164416
8 0.312669 -2.793251 1.423307
1 0.814922 -2.011471 1.148809
1 -0.242168 -2.995244 0.655480

1mU/1mUabp.xyz
6 -0.265544 -0.493935 0.142098
6 2.206660 -0.429415 -0.060620
6 2.107763 1.004138 -0.132394
6 0.887916 1.595108 -0.059520
1 0.768369 2.670845 -0.105562
7 -0.280661 0.884210 0.077305
7 0.978473 -1.079077 0.076107
8 -1.296265 -1.175256 0.253670
8 3.255520 -1.096983 -0.107612
1 3.008827 1.592650 -0.238174
6 -1.585358 1.562341 0.145363
1 -2.176864 1.326286 -0.741142
1 -2.119739 1.239091 1.039605
1 -1.410428 2.635822 0.191599
1 0.990498 -2.094657 0.125453
8 -4.049526 -0.815520 -0.288456
1 -4.239461 0.132660 -0.322203
1 -3.092329 -0.865983 -0.093840
1mU/1mUabi.xyz
6 0.296117 -0.428690 0.400649
6 -2.125450 -0.454515 -0.139023
6 -2.030995 0.961641 -0.375758
6 -0.840094 1.590414 -0.205503
1 -0.727492 2.655883 -0.367095
7 0.303331 0.934436 0.185197
7 -0.921353 -1.049941 0.241802
8 1.313152 -1.064318 0.720643
8 -3.150715 -1.151199 -0.244609
1 -2.913912 1.507966 -0.678372
6 1.577565 1.654886 0.343611
1 2.046148 1.360498 1.282787
1 2.246529 1.424903 -0.488105
1 1.367050 2.723145 0.359651
1 -0.929036 -2.052498 0.412534
8 3.722149 -0.999711 -0.788776

1 4.001489 -0.078218 -0.884314
1 2.895839 -0.953728 -0.267999
1mU/1mUacw.xyz
6 0.655015 -0.117054 0.928069
6 -1.673234 -0.458289 0.138796
6 -1.125850 -0.872640 -1.126081
6 0.222214 -0.908052 -1.293012
1 0.674408 -1.216443 -2.228143
7 1.106534 -0.564308 -0.300782
7 -0.713544 -0.126163 1.094866
8 1.422924 0.254133 1.824272
8 -2.882331 -0.378264 0.424157
1 -1.796475 -1.148938 -1.928087
6 2.560163 -0.575275 -0.525719
1 2.954056 0.441830 -0.473648
1 3.045778 -1.191454 0.232646
1 2.751527 -0.992860 -1.512802
1 -1.048410 0.202806 1.996794
8 -0.164555 2.836888 -0.889908
1 -0.513311 1.974069 -1.159141
1 0.343357 2.650097 -0.086700
1mU/1mUabo.xyz
6 0.176007 1.275180 -0.249860
6 1.822640 -0.561562 0.031622
6 0.832706 -1.257606 0.810144
6 -0.380916 -0.683540 1.017000
1 -1.153398 -1.176701 1.594760
7 -0.717636 0.550008 0.517380
7 1.406486 0.683253 -0.441122
8 -0.104990 2.379064 -0.732595
8 2.967066 -0.971357 -0.236128
1 1.069142 -2.230445 1.218897
6 -2.060135 1.119327 0.709985
1 -1.983877 2.087277 1.208477
1 -2.549998 1.247341 -0.257530
1 -2.640710 0.435310 1.327266

1 2.065693 1.211266 -1.007625
8 -2.550818 -2.144784 -1.155960
1 -1.605050 -2.225746 -0.962623
1 -2.855628 -1.435306 -0.571315
5caC/5caCacg.xyz
6 1.504856 -1.376357 -0.001043
6 0.079342 0.602387 0.013627
6 -1.099234 -0.219421 -0.000064
6 -0.896071 -1.571812 -0.011046
1 -1.725367 -2.268021 -0.021047
7 0.344074 -2.123778 -0.011499
7 1.299031 -0.013639 0.015261
8 2.627886 -1.879241 -0.004536
7 0.043978 1.920293 0.028841
1 0.898557 2.468384 0.042273
1 -0.888814 2.339713 0.016313
6 -2.506322 0.342127 -0.003754
8 -2.627086 1.608458 -0.048463
8 -3.457402 -0.487151 0.035364
1 2.143285 0.588413 0.019141
1 0.453591 -3.134014 -0.021944
8 3.130149 2.082949 0.003694
1 3.614512 2.168907 -0.831560
1 3.810853 2.094818 0.693802
5caC/5caCaaa.xyz
6 -1.515692 1.264522 -0.013391
6 -0.014273 -0.652636 -0.014730
6 1.131511 0.215196 -0.000912
6 0.874752 1.558537 0.001835
1 1.674735 2.288240 0.009868
7 -0.386384 2.059110 -0.007066
7 -1.257104 -0.089539 -0.017860
8 -2.657872 1.723064 -0.015636
7 0.080082 -1.968302 -0.020308
1 -0.742368 -2.560867 -0.030693
1 1.031673 -2.345308 -0.030019

6 2.559565 -0.290440 0.008189
8 2.730295 -1.550232 -0.055409
8 3.476892 0.574198 0.076399
1 -2.094569 -0.712600 -0.021746
1 -0.537976 3.063888 -0.006064
8 -3.436437 -1.808643 -0.050931
1 -3.295691 -2.504179 0.609989
1 -4.210172 -1.316217 0.263971
5caC/5caCaay.xyz
6 1.519450 -1.234047 -0.011197
6 -0.002674 0.666661 -0.012960
6 -1.138899 -0.214306 -0.000662
6 -0.867503 -1.554629 0.002312
1 -1.659127 -2.293357 0.009532
7 0.399040 -2.041057 -0.005386
7 1.246697 0.117684 -0.013830
8 2.666313 -1.680667 -0.014531
7 -0.115171 1.980703 -0.019630
1 0.697565 2.586096 -0.030817
1 -1.072764 2.343099 -0.029921
6 -2.572693 0.275415 0.006857
8 -2.757420 1.533452 -0.053224
8 -3.480755 -0.599269 0.070389
1 2.081577 0.744364 -0.015623
1 0.562154 -3.044018 -0.005121
8 3.480208 1.771842 -0.042313
1 4.239042 1.167779 -0.039224
1 3.544736 2.257302 0.794422
5caC/5caCadk.xyz
6 -1.507351 -1.296381 0.008100
6 -0.029637 0.640018 -0.007286
6 1.126729 -0.213486 0.001076
6 0.886950 -1.559813 0.011397
1 1.696334 -2.279123 0.017595
7 -0.367933 -2.076169 0.017021
7 -1.265763 0.061021 -0.007305

8 -2.643373 -1.769525 0.012395
7 0.049207 1.956464 -0.019090
1 -0.779820 2.539677 -0.027142
1 0.996406 2.344394 -0.006986
6 2.548602 0.309703 -0.000264
8 2.703727 1.572014 0.053805
8 3.476652 -0.544373 -0.052326
1 -2.106796 0.676130 -0.019291
1 -0.506424 -3.082812 0.027000
8 -3.380873 1.877675 -0.093154
1 -4.245280 1.438921 -0.088423
1 -3.363807 2.407025 0.718981
5caC/5caCaap.xyz
6 -1.503117 -1.393671 -0.015091
6 -0.089080 0.594513 -0.024340
6 1.094027 -0.220491 -0.004306
6 0.899022 -1.574038 0.002197
1 1.732646 -2.265012 0.014118
7 -0.337720 -2.133785 -0.008743
7 -1.305275 -0.029560 -0.024565
8 -2.623130 -1.902987 -0.013875
7 -0.062721 1.912302 -0.039007
1 -0.923063 2.452429 -0.052278
1 0.867026 2.337976 -0.048136
6 2.497886 0.349083 0.009930
8 2.613268 1.614836 -0.058257
8 3.451956 -0.474152 0.087426
1 -2.151085 0.566937 -0.018000
1 -0.440961 -3.144730 -0.004264
8 -3.091568 2.122753 0.053010
1 -3.772210 2.185786 -0.634242
1 -3.568974 2.207937 0.892233
5caC/5caCaae.xyz
6 -2.824040 0.065187 0.049090
6 -0.605812 -0.970984 -0.029689
6 0.013525 0.326050 -0.047397

6 -0.824843 1.406137 -0.020459
1 -0.443089 2.419248 -0.030773
7 -2.174903 1.282135 0.023141
7 -1.971741 -1.024610 0.020961
8 -4.044853 -0.052419 0.094717
7 0.077938 -2.095731 -0.057881
1 -0.377430 -3.001462 -0.042592
1 1.096760 -1.993466 -0.087480
6 1.511786 0.530380 -0.081552
8 2.235473 -0.522715 -0.102153
8 1.942002 1.712011 -0.083916
1 -2.434208 -1.932256 0.036462
1 -2.762807 2.111156 0.043617
8 4.951376 -0.225423 0.085001
1 3.964553 -0.271758 0.033556
1 5.141486 0.103729 0.974513
5caC/5caCadn.xyz
6 1.560301 1.056799 -0.009295
6 -0.886501 1.133283 0.007580
6 -0.941410 -0.300862 -0.005147
6 0.260946 -0.956903 -0.020553
1 0.309575 -2.038881 -0.030595
7 1.452695 -0.314110 -0.024195
7 0.347012 1.725623 0.005505
8 2.636294 1.653811 -0.009842
7 -1.962466 1.894546 0.020101
1 -1.901909 2.906244 0.029004
1 -2.856628 1.393638 0.022325
6 -2.240060 -1.079796 -0.002859
8 -3.322408 -0.408010 0.012010
8 -2.158420 -2.339171 -0.016328
1 0.411914 2.742202 0.014909
1 2.340072 -0.856360 -0.026456
8 3.814268 -1.800857 -0.036596
1 4.035527 -2.054760 0.872475
1 4.523227 -1.195807 -0.303847

5caC/5caCabn.xyz
6 1.550587 1.106063 -0.017478
6 -0.897687 1.134966 0.007532
6 -0.924522 -0.299877 -0.005503
6 0.290587 -0.931885 -0.026350
1 0.360192 -2.012647 -0.036950
7 1.469905 -0.266583 -0.034958
7 0.323911 1.750417 0.001468
8 2.613527 1.725698 -0.018308
7 -1.987055 1.876903 0.023483
1 -1.945115 2.889498 0.032531
1 -2.872465 1.361005 0.028629
6 -2.207103 -1.104438 0.001434
8 -3.302382 -0.454423 0.020723
8 -2.100422 -2.362075 -0.013018
1 0.369925 2.767767 0.012465
1 2.363602 -0.798033 -0.038793
8 3.814448 -1.780308 -0.052599
1 3.601040 -2.702006 0.158633
1 4.402941 -1.490848 0.661346
5caC/5caCadu.xyz
6 -1.510849 1.284930 -0.010748
6 -0.024669 -0.644508 -0.005087
6 1.127865 0.214427 -0.000031
6 0.882021 1.559722 -0.002618
1 1.688000 2.282852 -0.000442
7 -0.375110 2.070201 -0.009919
7 -1.263374 -0.071554 -0.007825
8 -2.648895 1.753303 -0.013744
7 0.061217 -1.960726 -0.002616
1 -0.764093 -2.549193 -0.003119
1 1.010823 -2.343073 -0.013284
6 2.552125 -0.302048 0.005216
8 2.713474 -1.563261 -0.056277
8 3.475952 0.555935 0.067944
1 -2.104179 -0.688897 -0.011238

1 -0.518564 3.076179 -0.013349
8 -3.432303 -1.815343 -0.068142
1 -4.146257 -1.503785 0.509244
1 -3.149653 -2.659746 0.316069
5caC/5caCabf.xyz
6 -2.763872 -0.435761 -0.000004
6 -0.812142 1.044324 0.000003
6 0.065705 -0.092534 0.000010
6 -0.526549 -1.325597 0.000009
1 0.061296 -2.234686 0.000012
7 -1.872895 -1.488439 0.000007
7 -2.159888 0.809600 -0.000005
8 -3.982732 -0.579708 -0.000014
7 -0.380340 2.287762 0.000007
1 -1.017405 3.076274 -0.000017
1 0.637040 2.405525 0.000000
6 1.571783 0.030186 0.000008
8 2.069983 1.196437 0.000003
8 2.232939 -1.051102 0.000008
1 -2.802347 1.600454 -0.000013
1 -2.275131 -2.422048 -0.000004
8 4.937942 -0.637429 -0.000016
1 5.058624 0.322648 -0.000017
1 3.955174 -0.759918 -0.000025
5caC/5caCaas.xyz
6 -2.818270 0.118751 -0.029074
6 -0.631274 -0.982150 0.018141
6 0.027215 0.295384 0.029122
6 -0.778860 1.399945 0.013643
1 -0.366871 2.401176 0.020238
7 -2.132434 1.315811 -0.012445
7 -1.998315 -0.995955 -0.012496
8 -4.042486 0.038090 -0.057072
7 0.017644 -2.126974 0.035508
1 -0.464681 -3.018811 0.023873
1 1.039331 -2.056672 0.050683

6 1.531063 0.453036 0.048718
8 2.222725 -0.621665 0.054691
8 1.999144 1.620439 0.055821
1 -2.486082 -1.890464 -0.023263
1 -2.695866 2.161892 -0.023840
8 4.921582 -0.178266 -0.109027
1 3.942324 -0.308383 -0.051436
1 5.036609 0.782506 -0.108833
5caC/5caCact.xyz
6 1.548657 1.114508 0.013685
6 -0.899905 1.134828 -0.004922
6 -0.921583 -0.300119 0.003174
6 0.295871 -0.927795 0.017769
1 0.369322 -2.008318 0.024349
7 1.472971 -0.258519 0.024920
7 0.319509 1.754357 0.000353
8 2.609046 1.738378 0.014817
7 -1.991682 1.873330 -0.016147
1 -1.953144 2.886074 -0.021763
1 -2.875484 1.354734 -0.020205
6 -2.201201 -1.109312 -0.002479
8 -3.298865 -0.463236 -0.015303
8 -2.089867 -2.366628 0.006386
1 0.362112 2.771841 -0.006801
1 2.367942 -0.787739 0.028054
8 3.818290 -1.769251 0.050441
1 3.586961 -2.708956 0.106131
1 4.296829 -1.678583 -0.787729
5caC/5caCacj.xyz
6 1.505997 -1.327337 -0.011213
6 0.049504 0.625930 -0.004385
6 -1.115994 -0.214857 -0.001388
6 -0.891224 -1.563817 -0.003847
1 -1.708787 -2.273854 -0.001920
7 0.357740 -2.094221 -0.010776
7 1.279413 0.033013 -0.007889

8 2.636266 -1.813514 -0.014360
7 -0.014901 1.943283 0.000667
1 0.821436 2.516339 0.003107
1 -0.957807 2.341400 -0.010779
6 -2.532101 0.323489 0.003410
8 -2.674420 1.586865 -0.061647
8 -3.468698 -0.520271 0.069061
1 2.124324 0.641752 -0.006803
1 0.484773 -3.102444 -0.013434
8 3.407152 1.846681 0.017166
1 3.243275 2.507966 -0.672967
1 3.337537 2.335784 0.851561
5caC/5caCaaw.xyz
6 -1.555472 1.078685 0.001366
6 0.892219 1.133387 -0.002343
6 0.934067 -0.301160 0.001638
6 -0.274314 -0.946222 0.006081
1 -0.332471 -2.027718 0.009404
7 -1.460710 -0.293291 0.006592
7 -0.335865 1.736301 -0.002768
8 -2.625031 1.687045 0.000720
7 1.974443 1.885824 -0.005387
1 1.922456 2.898019 -0.007925
1 2.864640 1.378019 -0.005347
6 2.225462 -1.091838 0.001331
8 3.313842 -0.429794 -0.002458
8 2.132460 -2.350519 0.004885
1 -0.392008 2.753331 -0.005825
1 -2.350774 -0.831665 0.007320
8 -3.778624 -1.839637 0.018392
1 -4.214097 -1.791734 -0.846455
1 -4.415766 -1.453969 0.639032
5caC/5caCadp.xyz
6 -1.546963 -1.118058 0.010618
6 0.901768 -1.133877 -0.003170
6 0.920776 0.301125 0.002224

6 -0.297883 0.926573 0.012236
1 -0.373392 2.006963 0.016283
7 -1.473901 0.255292 0.017798
7 -0.316492 -1.755462 0.001384
8 -2.605794 -1.744385 0.011789
7 1.994719 -1.870681 -0.011504
1 1.957800 -2.883492 -0.015461
1 2.877740 -1.350799 -0.014990
6 2.198933 1.112595 -0.002079
8 3.297748 0.468447 -0.011750
8 2.085341 2.369733 0.004643
1 -0.357371 -2.773006 -0.003384
1 -2.369446 0.783148 0.019070
8 -3.833151 1.745030 0.024598
1 -4.086834 1.979483 -0.881112
1 -3.691712 2.592910 0.472643
5caC/5caCabz.xyz
6 2.818804 -0.110006 0.039952
6 0.626817 0.980289 -0.023266
6 -0.025102 -0.300587 -0.040908
6 0.786331 -1.401181 -0.022271
1 0.379412 -2.404447 -0.033704
7 2.139261 -1.310439 0.013551
7 1.993515 1.000649 0.019659
8 4.042312 -0.023189 0.079307
7 -0.027789 2.121884 -0.045229
1 0.449879 3.016095 -0.027680
1 -1.048987 2.046285 -0.067952
6 -1.528041 -0.465800 -0.067147
8 -2.224905 0.605441 -0.079050
8 -1.990307 -1.635499 -0.072240
1 2.476981 1.897379 0.036421
1 2.706786 -2.153742 0.027301
8 -4.924141 0.195038 0.125958
1 -5.052538 -0.738149 0.346777
1 -3.942972 0.301299 0.053022

5caC/5caCado.xyz
6 2.589597 -0.200863 0.226193
6 0.307166 -0.795151 -0.434474
6 -0.125830 0.555497 -0.204650
6 0.825455 1.438255 0.227717
1 0.585098 2.474757 0.428433
7 2.117465 1.078952 0.431501
7 1.625347 -1.089399 -0.217093
8 3.756676 -0.531280 0.413271
7 -0.496839 -1.753007 -0.844814
1 -0.169681 -2.701838 -0.988279
1 -1.476319 -1.487505 -0.977474
6 -1.553003 1.015946 -0.403344
8 -2.406073 0.129008 -0.748523
8 -1.803500 2.233456 -0.213580
1 1.952433 -2.042312 -0.368631
1 2.792411 1.764009 0.760137
8 -3.472938 -1.349647 1.379618
1 -4.141941 -0.790990 1.799680
1 -3.117442 -0.806341 0.644027
5caC/5caCabb.xyz
6 2.543605 0.175171 0.200871
6 0.271181 0.763450 -0.498086
6 -0.179200 -0.568923 -0.203878
6 0.759816 -1.441363 0.272974
1 0.507142 -2.464729 0.520446
7 2.054457 -1.086648 0.468317
7 1.591233 1.053460 -0.286246
8 3.714545 0.499235 0.374638
7 -0.517949 1.707713 -0.964864
1 -0.178117 2.644165 -1.153682
1 -1.502671 1.454122 -1.076873
6 -1.610980 -1.022340 -0.388110
8 -2.464696 -0.129022 -0.713697
8 -1.863334 -2.240196 -0.202839
1 1.932112 1.992036 -0.488913

1 2.720749 -1.764547 0.827871
8 -3.207860 1.471252 1.495375
1 -2.948136 0.908321 0.735987
1 -2.561045 1.262835 2.184272
5caC/5caCack.xyz
6 2.822951 -0.051056 0.064085
6 0.598032 0.967737 -0.044470
6 -0.011363 -0.334042 -0.057890
6 0.834616 -1.407602 -0.013254
1 0.460497 -2.423595 -0.018118
7 2.183192 -1.273007 0.043111
7 1.963042 1.032128 0.017646
8 4.042410 0.075992 0.120577
7 -0.093981 2.087015 -0.087133
1 0.354758 2.996097 -0.075468
1 -1.111753 1.976734 -0.123846
6 -1.507562 -0.550213 -0.105455
8 -2.239227 0.497267 -0.133003
8 -1.928103 -1.735173 -0.111559
1 2.418936 1.943119 0.029019
1 2.777066 -2.097208 0.078645
8 -4.948886 0.194565 0.139406
1 -5.129326 0.418096 1.063128
1 -3.965538 0.253652 0.049808
5caC/5caCacq.xyz
6 2.828000 -0.063125 -0.000001
6 0.607530 0.971728 0.000000
6 -0.011278 -0.325648 0.000001
6 0.828241 -1.405138 -0.000002
1 0.446966 -2.418485 -0.000002
7 2.179005 -1.280433 -0.000004
7 1.974465 1.026146 -0.000003
8 4.049542 0.055446 0.000000
7 -0.077854 2.095911 0.000000
1 0.376898 3.002072 0.000014
1 -1.096995 1.992466 0.000006

6 -1.509995 -0.530866 0.000005
8 -2.234800 0.521547 0.000006
8 -1.939561 -1.712794 0.000006
1 2.436645 1.934072 -0.000002
1 2.767505 -2.109289 -0.000005
8 -4.951311 0.088270 -0.000008
1 -5.333579 0.976836 -0.000020
1 -3.972703 0.229494 0.000003
5caU/5caUach.xyz
6 -1.565455 -1.097345 -0.014940
6 0.901477 -1.169734 0.008199
6 0.911389 0.279338 -0.005400
6 -0.291620 0.937971 -0.025999
1 -0.349956 2.020430 -0.037045
7 -1.469829 0.283358 -0.034055
7 -0.352276 -1.756499 0.003062
8 -2.644823 -1.687425 -0.014558
8 1.923215 -1.887616 0.023095
1 -0.391563 -2.773363 0.014849
6 2.172897 1.047402 0.001202
8 2.218245 2.275238 -0.004917
8 3.311178 0.332898 0.013671
1 3.085153 -0.637841 0.018809
1 -2.360516 0.821023 -0.039243
8 -3.807422 1.801170 -0.058489
1 -4.404373 1.501092 0.643976
1 -3.599280 2.720736 0.166824
5caU/5caUacv.xyz
6 -1.540772 0.835606 0.018464
6 0.257617 -0.850801 0.015210
6 1.227685 0.228171 -0.003264
6 0.770301 1.517866 -0.012874
1 1.442489 2.367911 -0.027565
7 -0.551618 1.803998 -0.003886
7 -1.071431 -0.457611 0.023205
8 -2.738937 1.120110 0.032606

8 0.543952 -2.063198 0.024603
1 -1.776660 -1.192659 0.039954
6 2.684096 -0.034731 -0.011456
8 3.529247 0.855683 -0.028129
8 3.063551 -1.322848 0.000427
1 2.255237 -1.904143 0.011159
1 -0.863314 2.771602 -0.010115
8 -4.573831 -1.089246 0.053301
1 -3.992519 -0.304479 0.025614
1 -4.689315 -1.343623 -0.873217
5caU/5caUads.xyz
6 -1.575463 -1.037929 -0.006997
6 0.888571 -1.166411 0.008040
6 0.932082 0.282084 -0.004851
6 -0.255585 0.968502 -0.019065
1 -0.289172 2.052079 -0.028877
7 -1.448252 0.340406 -0.021736
7 -0.378210 -1.724657 0.006017
8 -2.668865 -1.601867 -0.006891
8 1.893389 -1.907585 0.020012
1 -0.440252 -2.740400 0.015609
6 2.211139 1.021025 -0.002977
8 2.283985 2.247579 -0.010358
8 3.333729 0.281773 0.006583
1 3.088021 -0.683916 0.012993
1 -2.331964 0.889322 -0.025005
8 -3.826470 1.799616 -0.042277
1 -4.512460 1.169950 -0.312896
1 -4.059548 2.042976 0.866751
5caU/5caUabb.xyz
6 -1.570658 -1.064403 -0.000643
6 0.894886 -1.167341 0.002413
6 0.922868 0.281532 -0.001591
6 -0.272039 0.955307 -0.005607
1 -0.316864 2.038474 -0.008821
7 -1.458444 0.315278 -0.005794

7 -0.365962 -1.738563 0.002976
8 -2.657255 -1.641288 0.000165
8 1.907568 -1.897983 0.005281
1 -0.417679 -2.754924 0.005975
6 2.194075 1.033745 -0.001359
8 2.254523 2.260952 -0.003247
8 3.323849 0.305540 0.000991
1 3.086996 -0.662521 0.002726
1 -2.345367 0.860031 -0.006742
8 -3.781682 1.848708 -0.020267
1 -4.410585 1.449015 -0.640535
1 -4.216483 1.792452 0.844461
5caU/5caUadm.xyz
6 1.563682 -1.107637 0.012175
6 -0.903750 -1.169969 -0.005410
6 -0.907567 0.279149 0.003030
6 0.298180 0.932821 0.017721
1 0.360949 2.015050 0.024689
7 1.473825 0.273596 0.024693
7 0.347603 -1.761791 -0.000628
8 2.640305 -1.702577 0.012491
8 -1.928509 -1.883663 -0.015848
1 0.382737 -2.778849 -0.008376
6 -2.165872 1.052396 -0.002367
8 -2.206266 2.280408 0.001292
8 -3.306956 0.342370 -0.011030
1 -3.084526 -0.629253 -0.014016
1 2.365757 0.809166 0.028704
8 3.807958 1.794099 0.053790
1 4.289769 1.702023 -0.782381
1 3.575019 2.733580 0.106477
5caU/5caUacu.xyz
6 -1.575267 -1.037406 -0.007862
6 0.888762 -1.166440 0.007989
6 0.932679 0.282049 -0.005835
6 -0.254758 0.968749 -0.022708

1 -0.288125 2.052325 -0.034002
7 -1.447507 0.340854 -0.026188
7 -0.378181 -1.724393 0.006141
8 -2.668908 -1.600879 -0.005717
8 1.893383 -1.907835 0.020744
1 -0.440447 -2.740111 0.017066
6 2.211886 1.020742 -0.002036
8 2.284928 2.247291 -0.008596
8 3.334328 0.281309 0.008508
1 3.088483 -0.684332 0.014294
1 -2.330925 0.889676 -0.031575
8 -3.841090 1.779849 -0.060388
1 -3.957009 2.263038 0.772152
1 -4.533101 1.100127 -0.051308
5caU/5caUacn.xyz
6 -1.579865 0.632846 -0.032319
6 0.836383 1.125039 0.007919
6 1.095472 -0.302489 -0.008417
6 0.029805 -1.160022 -0.036552
1 0.157966 -2.235859 -0.050591
7 -1.245385 -0.711067 -0.048569
7 -0.501618 1.487208 -0.005788
8 -2.746553 1.027237 -0.041260
8 1.716577 2.006352 0.033651
1 -0.717097 2.482139 0.005371
6 2.472093 -0.844450 0.006116
8 2.722988 -2.046085 -0.006632
8 3.471209 0.052285 0.035396
1 3.087254 0.971150 0.039017
1 -2.019218 -1.371341 -0.069438
8 -4.717667 -1.084324 -0.028454
1 -4.117668 -0.314540 -0.007135
1 -4.857967 -1.313800 0.901190
5caU/5caUaco.xyz
6 1.561021 1.117702 0.006499
6 -0.906913 1.169156 -0.003105

6 -0.904262 -0.279972 0.002165
6 0.304357 -0.928334 0.009890
1 0.371965 -2.010281 0.013746
7 1.477329 -0.264212 0.013341
7 0.341805 1.766320 -0.000796
8 2.634413 1.718154 0.006948
8 -1.934833 1.878389 -0.009072
1 0.372535 2.783546 -0.005271
6 -2.159266 -1.058540 -0.000285
8 -2.194589 -2.286702 0.002559
8 -3.303181 -0.353105 -0.005507
1 -3.084412 0.619435 -0.007734
1 2.370351 -0.797438 0.014276
8 3.821247 -1.771473 0.001381
1 3.895677 -2.245807 -0.840811
1 3.785866 -2.466384 0.676513
5caU/5caUabh.xyz
6 1.543841 1.237211 -0.004203
6 0.010018 -0.679984 -0.005395
6 -1.110297 0.243937 0.000274
6 -0.844622 1.586811 0.002540
1 -1.632057 2.331540 0.006400
7 0.421438 2.056794 0.000100
7 1.268297 -0.110412 -0.008201
8 2.685260 1.695630 -0.004649
8 -0.107861 -1.924438 -0.008268
1 2.086670 -0.751174 -0.010250
6 -2.511431 -0.227943 0.003131
8 -3.479638 0.528521 0.007819
8 -2.697532 -1.558026 0.000423
1 -1.809054 -2.011493 -0.002983
1 0.595244 3.058093 0.002572
8 3.526313 -1.797601 -0.005051
1 3.431741 -2.489696 -0.677262
1 3.561915 -2.274827 0.837958
5caU/5caUaaz.xyz

6 -1.551281 1.195010 -0.005434
6 0.012554 -0.697258 -0.012888
6 1.117943 0.244664 0.000370
6 0.830654 1.583097 0.007542
1 1.606053 2.340306 0.016157
7 -0.442750 2.033119 0.004793
7 -1.253821 -0.147370 -0.017373
8 -2.700985 1.633071 -0.004463
8 0.149941 -1.939705 -0.020851
1 -2.065740 -0.796662 -0.027124
6 2.526567 -0.204289 0.005744
8 3.482217 0.567881 0.016783
8 2.734733 -1.531105 -0.001764
1 1.854131 -1.999174 -0.009782
1 -0.632716 3.031456 0.011532
8 -3.518780 -1.815724 -0.076519
1 -4.251673 -1.277410 0.260030
1 -3.429676 -2.541455 0.559759
5caU/5caUabl.xyz
6 -1.468963 -1.661858 -0.030033
6 -0.233211 0.471436 -0.105000
6 1.006323 -0.270924 -0.000844
6 0.941282 -1.636303 0.086453
1 1.830910 -2.249364 0.170164
7 -0.237641 -2.294461 0.073549
7 -1.388718 -0.287478 -0.123737
8 -2.530679 -2.276762 -0.038185
8 -0.304966 1.717255 -0.176338
1 -2.274661 0.211334 -0.188151
6 2.325162 0.399762 0.016970
8 3.388148 -0.209683 0.090456
8 2.324119 1.741303 -0.054966
1 1.387662 2.069373 -0.108172
1 -0.256893 -3.308240 0.144398
8 -2.839324 2.955230 0.292378
1 -1.959651 2.590027 0.074439

1 -3.064793 3.529019 -0.453403
5caU/5caUaae.xyz
6 -1.493261 1.638986 -0.030893
6 -0.227349 -0.476504 -0.119407
6 1.001006 0.281899 -0.000956
6 0.916511 1.645555 0.095480
1 1.797315 2.269886 0.188473
7 -0.271138 2.287762 0.080928
7 -1.393269 0.266604 -0.134593
8 -2.563683 2.238640 -0.037529
8 -0.281318 -1.722329 -0.205185
1 -2.271666 -0.243765 -0.211106
6 2.329388 -0.369596 0.021851
8 3.382489 0.254945 0.109507
8 2.349373 -1.710360 -0.060364
1 1.419303 -2.052805 -0.126866
1 -0.304457 3.300760 0.158375
8 -2.796518 -3.037289 0.127186
1 -1.921809 -2.615304 0.018643
1 -2.948350 -3.050227 1.082777
5caU/5caUaah.xyz
6 2.800868 -0.362235 0.007250
6 0.794877 1.077065 -0.002378
6 -0.027003 -0.117753 -0.008155
6 0.593129 -1.338828 -0.005698
1 0.035591 -2.267970 -0.009201
7 1.938670 -1.450606 0.001922
7 2.162820 0.860420 0.004352
8 4.021650 -0.487371 0.014190
8 0.350639 2.241996 -0.002822
1 2.763531 1.682385 0.008594
6 -1.500135 -0.040415 -0.013818
8 -2.223944 -1.037364 -0.017908
8 -2.043736 1.181960 -0.013688
1 -1.315596 1.864601 -0.009764
1 2.373985 -2.369408 0.003662

8 -5.020863 -0.610376 0.004882
1 -5.161513 0.321993 0.222449
1 -4.046811 -0.718061 -0.000093
5caU/5caUabu.xyz
6 1.462303 -1.690713 -0.030676
6 0.246496 0.452975 -0.118637
6 -0.999692 -0.276002 -0.002183
6 -0.947415 -1.641232 0.095313
1 -1.842269 -2.245345 0.187986
7 0.225247 -2.310516 0.081937
7 1.394881 -0.316158 -0.132675
8 2.517812 -2.316070 -0.038993
8 0.329661 1.697181 -0.203772
1 2.284657 0.173652 -0.209372
6 -2.311057 0.409370 0.019600
8 -3.379871 -0.186956 0.112958
8 -2.295190 1.749632 -0.071343
1 -1.354979 2.065523 -0.137197
1 0.234951 -3.323888 0.160050
8 2.814439 3.021342 0.283849
1 1.959742 2.593514 0.080294
1 2.778386 3.875837 -0.168693
5caU/5caUaar.xyz
6 2.801626 -0.352032 0.000009
6 0.787333 1.075713 0.000002
6 -0.027635 -0.123792 -0.000010
6 0.599502 -1.341278 -0.000018
1 0.047265 -2.273586 -0.000032
7 1.945647 -1.445351 -0.000010
7 2.156537 0.866959 0.000017
8 4.023102 -0.470177 0.000022
8 0.336411 2.238075 0.000001
1 2.752643 1.692300 0.000022
6 -1.501150 -0.054882 -0.000017
8 -2.219212 -1.056044 -0.000009
8 -2.051968 1.164251 -0.000033

1 -1.327798 1.851182 -0.000027
1 2.386119 -2.361695 -0.000026
8 -5.009980 -0.591715 0.000036
1 -5.119116 0.369894 0.000047
1 -4.039283 -0.726852 0.000034
5caU/5caUacx.xyz
6 -2.714205 -0.032118 0.287463
6 -0.495706 -0.969314 -0.257085
6 0.041384 0.378447 -0.253421
6 -0.805388 1.419887 0.017431
1 -0.465005 2.448438 0.037608
7 -2.115931 1.220833 0.276783
7 -1.849513 -1.071797 0.014406
8 -3.908320 -0.192548 0.521612
8 0.171888 -1.998020 -0.482686
1 -2.254646 -2.005692 0.019328
6 1.467419 0.646106 -0.527030
8 1.961025 1.768936 -0.532359
8 2.243017 -0.426797 -0.772771
1 1.687119 -1.254369 -0.725183
1 -2.721827 2.013083 0.473576
8 4.306167 -0.504271 1.337859
1 4.700550 0.378987 1.367788
1 3.660673 -0.460148 0.611176
5caU/5caUabg.xyz
6 2.081532 -0.070273 -0.600123
6 -0.098665 1.049802 -0.318994
6 -0.738141 -0.253169 -0.325030
6 0.049490 -1.365455 -0.460744
1 -0.363894 -2.367087 -0.462701
7 1.389967 -1.274256 -0.598166
7 1.275558 1.042361 -0.485070
8 3.303022 -0.009977 -0.705665
8 -0.700459 2.132861 -0.179480
1 1.750448 1.942791 -0.473263
6 -2.200726 -0.402750 -0.161680

8 -2.771472 -1.489951 -0.170016
8 -2.905678 0.728138 0.004220
1 -2.283961 1.506868 -0.014624
1 1.951895 -2.116896 -0.684707
8 1.143693 -0.221487 2.849203
1 0.332176 -0.345496 2.335536
1 0.840855 0.137485 3.695743
5caU/5caUace.xyz
6 1.590964 1.353657 0.001945
6 0.104772 -0.607255 0.004528
6 -1.037874 0.286254 -0.002107
6 -0.806547 1.635866 -0.004379
1 -1.614114 2.358715 -0.007687
7 0.446413 2.140898 -0.002370
7 1.347085 -0.000162 0.005249
8 2.720819 1.837696 0.002773
8 0.024165 -1.854772 0.009658
1 2.162608 -0.631281 0.011615
6 -2.426749 -0.221148 -0.003518
8 -3.413070 0.510917 -0.007742
8 -2.579939 -1.555543 -0.000122
1 -1.683050 -1.989442 0.003734
1 0.590917 3.146984 -0.003941
8 2.985664 -2.344634 0.087276
1 2.099696 -2.736234 0.000933
1 3.440950 -2.567456 -0.738363
5caU/5caUadc.xyz
6 2.795769 -0.405940 0.000001
6 0.825127 1.081406 0.000002
6 -0.025359 -0.093447 0.000000
6 0.565297 -1.329060 0.000003
1 -0.014552 -2.244431 -0.000001
7 1.907914 -1.473360 0.000011
7 2.187428 0.831570 0.000013
8 4.013369 -0.560006 -0.000013
8 0.409074 2.256578 -0.000005

1 2.807702 1.638772 0.000004
6 -1.496478 0.018519 -0.000004
8 -2.243362 -0.961248 -0.000010
8 -2.011137 1.253168 0.000002
1 -1.267813 1.918587 -0.000003
1 2.321284 -2.402237 -0.000003
8 -5.042174 -0.491866 0.000005
1 -5.432162 -1.377319 -0.000001
1 -4.074145 -0.642723 -0.000009
5caU/5caUabo.xyz
6 1.542194 0.831680 -0.018727
6 -0.259837 -0.851532 -0.016258
6 -1.227720 0.229303 0.002002
6 -0.767826 1.518166 0.011057
1 -1.438446 2.369447 0.025535
7 0.554480 1.801805 0.002026
7 1.070043 -0.460824 -0.024707
8 2.740396 1.114596 -0.030585
8 -0.548329 -2.063406 -0.025178
1 1.773254 -1.197596 -0.041084
6 -2.684636 -0.030409 0.011106
8 -3.527785 0.861911 0.027051
8 -3.067096 -1.317683 0.001138
1 -2.260319 -1.900954 -0.009587
1 0.868066 2.768802 0.008889
8 4.647028 -1.036307 0.026220
1 4.024783 -0.283624 0.001470
1 4.094236 -1.799078 0.249293
5dhmC/5dhmCaaa.xyz
6 -2.410445 0.124000 0.049603
6 -0.419832 -0.902120 -0.611405
6 0.333782 0.321793 -0.476042
6 -0.370478 1.409105 -0.054714
1 0.074686 2.384622 0.087585
7 -1.700045 1.312207 0.206473
7 -1.735412 -0.975950 -0.362485

8 -3.639960 0.121938 0.300540
6 1.814153 0.378101 -0.789386
1 1.991430 0.140582 -1.845429
7 0.188486 -2.030051 -1.025995
1 -0.344450 -2.889969 -1.031834
1 1.198215 -2.089780 -1.021104
8 2.547649 -0.597294 -0.066514
1 2.238533 -0.592534 0.870467
8 2.277879 1.686881 -0.508139
1 3.177674 1.760759 -0.860178
1 -2.217520 2.129538 0.511884
8 1.447155 -0.607902 2.500969
1 0.565122 -0.271187 2.277812
1 1.300244 -1.529735 2.761659
5dhmC/5dhmCaai.xyz
6 1.612423 -1.223088 0.078857
6 0.183083 0.630920 -0.098479
6 -0.994271 -0.202361 -0.122176
6 -0.776461 -1.544749 -0.032393
1 -1.575084 -2.274404 -0.050730
7 0.483839 -2.038253 0.078757
7 1.421226 0.117126 -0.008706
8 2.745476 -1.748038 0.161152
6 -2.375184 0.388908 -0.298291
1 -2.450219 0.905389 -1.262340
7 0.083267 1.967642 -0.192578
1 0.923530 2.526354 -0.114323
1 -0.813251 2.420428 -0.074798
8 -2.648097 1.407427 0.660397
1 -2.481978 1.049812 1.548718
8 -3.325490 -0.652737 -0.224977
1 -4.173318 -0.304757 -0.540874
1 0.633090 -3.039939 0.140483
8 3.618810 1.885009 0.011553
1 2.881290 1.223917 -0.012373
1 4.114495 1.736520 -0.806184

5dhmC/5dhmCact.xyz
6 1.623395 -1.168278 0.030700
6 0.153470 0.651749 -0.168116
6 -1.007390 -0.204369 -0.126810
6 -0.759831 -1.538420 0.002979
1 -1.544221 -2.282834 0.035225
7 0.512603 -2.004649 0.091825
7 1.402761 0.163529 -0.101692
8 2.769498 -1.665891 0.102666
6 -2.406129 0.352315 -0.276185
1 -2.526329 0.830963 -1.255100
7 0.026530 1.982738 -0.302527
1 0.858433 2.557834 -0.268655
1 -0.873443 2.425041 -0.171986
8 -2.667434 1.398796 0.654927
1 -2.468516 1.073893 1.549140
8 -3.330693 -0.705017 -0.131626
1 -4.198357 -0.381414 -0.418037
1 0.683551 -3.000141 0.188096
8 3.673579 1.819081 0.059890
1 4.410502 1.228281 0.270280
1 2.884436 1.223310 -0.004490
5dhmC/5dhmCabk.xyz
6 -1.642854 0.627478 -0.013852
6 0.618130 1.179623 0.126811
6 1.020682 -0.206993 0.124720
6 0.012325 -1.119649 0.045351
1 0.179177 -2.188424 0.049147
7 -1.281313 -0.714518 -0.035706
7 -0.668586 1.558853 0.069038
8 -2.865525 0.924638 -0.077444
6 2.470059 -0.611855 0.276490
1 2.864613 -0.269849 1.240421
7 1.538838 2.155685 0.217024
1 1.232838 3.118029 0.157389
1 2.517035 1.947395 0.066234

8 3.308365 0.022575 -0.684785
1 2.942856 -0.143014 -1.569718
8 2.556956 -2.018520 0.184595
1 3.443034 -2.281037 0.477086
1 -2.024688 -1.403259 -0.093275
8 -4.757598 -1.094720 -0.179135
1 -4.987970 -1.297367 0.738330
1 -4.097100 -0.366022 -0.121074
5dhmC/5dhmCado.xyz
6 2.410439 -0.134013 0.045154
6 0.428567 0.960906 -0.527989
6 -0.332597 -0.266199 -0.510375
6 0.364859 -1.392038 -0.191381
1 -0.086747 -2.373538 -0.141376
7 1.693650 -1.328080 0.081801
7 1.742935 1.003008 -0.265022
8 3.637832 -0.162760 0.304675
6 -1.813654 -0.285606 -0.824340
1 -1.991481 0.037040 -1.857365
7 -0.169484 2.127263 -0.840243
1 0.366612 2.980450 -0.749560
1 -1.178919 2.192884 -0.830505
8 -2.537688 0.635662 -0.024453
1 -2.228019 0.554969 0.908675
8 -2.288752 -1.608632 -0.647874
1 -3.191439 -1.644014 -0.998792
1 2.205899 -2.172803 0.312106
8 -1.421237 0.459780 2.533668
1 -0.506855 0.267200 2.272688
1 -1.725682 -0.348236 2.973853
5dhmC/5dhmCaco.xyz
6 -1.614210 -1.214662 -0.052838
6 -0.177306 0.631675 0.140398
6 0.998139 -0.204692 0.124189
6 0.775074 -1.544159 0.007757
1 1.572054 -2.275713 -0.004929

7 -0.488317 -2.032446 -0.090992
7 -1.418098 0.122520 0.063232
8 -2.750274 -1.734400 -0.128631
6 2.384197 0.378909 0.287069
1 2.479327 0.873948 1.260561
7 -0.072056 1.965839 0.260566
1 -0.911482 2.529035 0.215084
1 0.823291 2.419572 0.137944
8 2.642305 1.416869 -0.654617
1 2.461853 1.077227 -1.547251
8 3.329378 -0.663760 0.172947
1 4.185134 -0.323914 0.475904
1 -0.641122 -3.031995 -0.174927
8 -3.603781 1.900288 -0.061340
1 -2.880698 1.225930 -0.002155
1 -4.375439 1.480116 0.343797
5dhmC/5dhmCaaj.xyz
6 -1.599823 1.199569 -0.036536
6 0.730083 1.267579 0.116009
6 0.832977 -0.171519 0.124977
6 -0.347108 -0.850316 0.036811
1 -0.407861 -1.930766 0.046286
7 -1.526252 -0.189116 -0.059002
7 -0.445983 1.907930 0.040456
8 -2.731531 1.743174 -0.093652
6 2.162894 -0.869970 0.292159
1 2.617719 -0.605098 1.254009
7 1.837660 2.030215 0.214407
1 1.741385 3.033564 0.130267
1 2.749523 1.620582 0.063162
8 3.122944 -0.441415 -0.671354
1 2.731438 -0.527303 -1.556560
8 1.954035 -2.265322 0.218795
1 2.766600 -2.702353 0.515715
1 -2.402010 -0.727055 -0.101143
8 -3.832110 -1.914596 -0.118147

1 -3.515696 -2.784064 -0.406119
1 -4.119915 -2.047498 0.797714
5dhmC/5dhmCaao.xyz
6 1.644252 0.611543 -0.064639
6 -0.616620 1.179080 -0.107850
6 -1.027437 -0.205011 -0.134999
6 -0.022319 -1.124793 -0.116380
1 -0.195100 -2.192207 -0.146359
7 1.275683 -0.728430 -0.067205
7 0.673536 1.550152 -0.081803
8 2.870163 0.900798 -0.040952
6 -2.482973 -0.599263 -0.257442
1 -2.897281 -0.240731 -1.206953
7 -1.533203 2.162899 -0.134870
1 -1.218101 3.120602 -0.051682
1 -2.506884 1.953845 0.043041
8 -3.296615 0.025527 0.731001
1 -2.918354 -0.160905 1.606299
8 -2.576120 -2.006531 -0.184090
1 -3.466866 -2.260706 -0.469695
1 2.016891 -1.421722 -0.057094
8 4.776680 -1.085636 0.141028
1 5.009507 -1.165282 1.076645
1 4.101804 -0.367836 0.104909
5dhmC/5dhmCaat.xyz
6 1.645353 0.615922 -0.063736
6 -0.616351 1.180114 -0.106857
6 -1.025115 -0.204543 -0.136069
6 -0.018612 -1.122847 -0.119333
1 -0.189833 -2.190457 -0.151352
7 1.278812 -0.724646 -0.069458
7 0.673238 1.553060 -0.079805
8 2.870809 0.906993 -0.038181
6 -2.480118 -0.600807 -0.258550
1 -2.895227 -0.241956 -1.207590
7 -1.534369 2.162610 -0.132832

1 -1.220736 3.120641 -0.047960
1 -2.507788 1.951888 0.044526
8 -3.294430 0.021807 0.730714
1 -2.915780 -0.165017 1.605758
8 -2.571176 -2.008277 -0.186533
1 -3.461679 -2.263433 -0.472025
1 2.021087 -1.416872 -0.061554
8 4.778657 -1.078421 0.142591
1 4.100050 -0.364219 0.104199
1 4.844314 -1.311578 1.079220
5dhmC/5dhmCacf.xyz
6 2.420009 -0.141534 0.050983
6 0.448541 1.015035 -0.430309
6 -0.320513 -0.203339 -0.527953
6 0.365729 -1.357976 -0.300122
1 -0.093282 -2.336546 -0.340487
7 1.692702 -1.327949 -0.011913
7 1.762322 1.023649 -0.159792
8 3.647246 -0.202812 0.305318
6 -1.797046 -0.184749 -0.864974
1 -1.956793 0.232896 -1.866498
7 -0.141096 2.208014 -0.640680
1 0.400961 3.047876 -0.486038
1 -1.150049 2.280001 -0.628642
8 -2.529834 0.661747 0.005512
1 -2.251675 0.470376 0.932679
8 -2.279101 -1.516360 -0.817992
1 -3.180507 -1.514301 -1.173815
1 2.196126 -2.193208 0.151991
8 -1.504691 0.146030 2.547944
1 -0.616121 -0.139082 2.282394
1 -1.915438 -0.647476 2.923110
5dhmC/5dhmCaas.xyz
6 -2.062058 0.266416 -0.657870
6 -0.055887 1.083044 0.215553
6 0.675292 -0.094973 -0.192275

6 -0.044109 -1.039287 -0.863342
1 0.384562 -1.967516 -1.216694
7 -1.365156 -0.852695 -1.111611
7 -1.368161 1.234023 -0.012680
8 -3.295176 0.334720 -0.876404
6 2.135467 -0.278375 0.153845
1 2.274929 -0.276822 1.241281
7 0.567633 2.076271 0.876701
1 0.045734 2.917248 1.085284
1 1.577672 2.118700 0.897477
8 2.934299 0.811667 -0.298314
1 2.758959 0.950796 -1.244058
8 2.579873 -1.501870 -0.394111
1 3.451929 -1.696935 -0.018195
1 -1.897441 -1.572861 -1.588079
8 -1.511962 -1.507875 2.420161
1 -2.252657 -1.114679 1.935756
1 -0.732397 -1.275212 1.894237
5dhmC/5dhmCaax.xyz
6 2.414418 -0.568393 0.094468
6 0.829910 1.072445 -0.405147
6 -0.254511 0.276446 0.118747
6 0.092270 -0.935385 0.636524
1 -0.623582 -1.628187 1.058443
7 1.387437 -1.339485 0.636446
7 2.101070 0.646052 -0.418011
8 3.580564 -1.029588 0.108026
6 -1.684064 0.759367 0.049414
1 -1.972190 0.962661 -0.988380
7 0.587687 2.287510 -0.933269
1 1.375169 2.850635 -1.226654
1 -0.297045 2.749453 -0.773629
8 -1.871610 2.000434 0.714814
1 -1.518727 1.925893 1.617393
8 -2.527645 -0.242174 0.594250
1 -3.445729 0.045042 0.465710

1 1.634755 -2.246311 1.017879
8 -2.456987 -2.590955 -1.076921
1 -3.372553 -2.746697 -1.348013
1 -2.496174 -1.799648 -0.504299
5dhmC/5dhmCadg.xyz
6 2.403939 0.132213 -0.038958
6 0.419656 -0.927082 0.588435
6 -0.340654 0.296579 0.495437
6 0.357395 1.401704 0.112170
1 -0.094182 2.378281 0.001030
7 1.687480 1.321932 -0.150578
7 1.735532 -0.984601 0.336800
8 3.632465 0.145396 -0.294186
6 -1.823669 0.330818 0.797113
1 -2.010547 0.041312 1.838355
7 -0.181697 -2.072960 0.962831
1 0.355337 -2.929788 0.931202
1 -1.190903 -2.139253 0.954843
8 -2.543546 -0.613547 0.020423
1 -2.222273 -0.564902 -0.910630
8 -2.293316 1.648784 0.575362
1 -3.201661 1.694931 0.909936
1 2.200381 2.151865 -0.428303
8 -1.392909 -0.463588 -2.530593
1 -0.473378 -0.292917 -2.272818
1 -1.373521 -1.331873 -2.960225
5dhmC/5dhmCabw.xyz
6 -1.608692 -0.707281 -0.118347
6 0.112681 0.852233 0.084321
6 1.125202 -0.176412 0.113787
6 0.674234 -1.458484 0.014980
1 1.328534 -2.319573 0.039961
7 -0.653311 -1.716371 -0.110542
7 -1.196478 0.574598 -0.024414
8 -2.820155 -1.036523 -0.218707
6 2.585977 0.159751 0.314510

1 2.731502 0.674493 1.271539
7 0.450981 2.148566 0.192727
1 -0.276696 2.848195 0.130049
1 1.414603 2.440322 0.103201
8 3.060048 1.091892 -0.653275
1 2.853553 0.750023 -1.539128
8 3.335395 -1.036766 0.285721
1 4.228111 -0.834157 0.604554
1 -0.980431 -2.674909 -0.172097
8 -4.851066 0.760032 0.182768
1 -4.567949 1.642776 -0.093413
1 -4.069765 0.177366 0.023388
5dhmC/5dhmCaab.xyz
6 1.609134 -0.711466 0.114712
6 -0.110192 0.851176 -0.081667
6 -1.124164 -0.175946 -0.114340
6 -0.674917 -1.458999 -0.020526
1 -1.330416 -2.319086 -0.048465
7 0.652321 -1.719217 0.103567
7 1.198620 0.571257 0.024621
8 2.820145 -1.042415 0.214014
6 -2.584629 0.162993 -0.312682
1 -2.729950 0.682556 -1.267126
7 -0.446870 2.148454 -0.184780
1 0.281678 2.846826 -0.117991
1 -1.409917 2.440944 -0.091380
8 -3.057113 1.091015 0.659812
1 -2.851428 0.744116 1.543894
8 -3.335449 -1.032757 -0.289230
1 -4.228233 -0.827500 -0.606160
1 0.978010 -2.678442 0.161884
8 4.849313 0.773541 -0.114441
1 4.067974 0.179870 -0.003475
1 4.487227 1.605648 -0.449263
5dhmC/5dhmCads.xyz
6 -2.415112 -0.438937 -0.120060

6 -0.737497 1.094360 0.416879
6 0.298875 0.248487 -0.125367
6 -0.116302 -0.934134 -0.660444
1 0.559456 -1.658291 -1.095266
7 -1.431953 -1.264573 -0.663568
7 -2.032289 0.744455 0.417574
8 -3.607648 -0.824913 -0.156094
6 1.752731 0.654906 -0.065611
1 2.057334 0.850324 0.968962
7 -0.426563 2.280111 0.973321
1 -1.178256 2.876574 1.292973
1 0.489074 2.688973 0.849817
8 2.002890 1.877783 -0.742908
1 1.632768 1.818998 -1.639688
8 2.538467 -0.396454 -0.604431
1 3.470951 -0.156137 -0.485340
1 -1.732727 -2.146230 -1.065336
8 2.260235 -2.628032 1.195409
1 2.389283 -1.889900 0.567747
1 1.300025 -2.679414 1.306649
5dhmC/5dhmCace.xyz
6 1.610838 -0.733587 0.094789
6 -0.097328 0.841529 -0.100752
6 -1.119843 -0.177427 -0.115943
6 -0.680179 -1.463386 -0.016242
1 -1.342787 -2.318341 -0.031556
7 0.645931 -1.733654 0.098467
7 1.209864 0.551607 -0.002420
8 2.820179 -1.072786 0.187461
6 -2.579430 0.172017 -0.301505
1 -2.731512 0.683336 -1.259339
7 -0.423880 2.140615 -0.213105
1 0.311131 2.833303 -0.158629
1 -1.383640 2.442217 -0.114855
8 -3.032679 1.113493 0.667260
1 -2.813235 0.777261 1.552162

8 -3.339675 -1.017206 -0.258113
1 -4.233559 -0.808273 -0.569330
1 0.963971 -2.695124 0.161817
8 4.797796 0.810960 -0.104102
1 4.046760 0.177939 -0.001078
1 4.390149 1.687146 -0.061925
5dhmC/5dhmCaaz.xyz
6 1.604352 -1.163721 -0.013475
6 -0.725989 -1.263344 0.108389
6 -0.847495 0.174129 0.133930
6 0.325489 0.868820 0.076630
1 0.372008 1.949746 0.102167
7 1.514472 0.224008 -0.007488
7 0.458920 -1.887561 0.038455
8 2.743169 -1.692814 -0.071137
6 -2.189255 0.854205 0.284903
1 -2.655158 0.579105 1.238531
7 -1.824077 -2.042067 0.183949
1 -1.712367 -3.042866 0.089317
1 -2.739939 -1.643319 0.028211
8 -3.128899 0.417692 -0.694907
1 -2.725530 0.514114 -1.573637
8 -1.997754 2.252547 0.220241
1 -2.819923 2.678209 0.507141
1 2.386974 0.769546 -0.036514
8 3.868849 1.866530 -0.022401
1 3.600550 2.797488 -0.006221
1 4.362653 1.765143 -0.850039
5dhmC/5dhmCaba.xyz
6 2.410713 -0.149119 0.023996
6 0.442375 1.041644 -0.378308
6 -0.334690 -0.165442 -0.530401
6 0.345725 -1.334146 -0.369168
1 -0.120075 -2.306724 -0.454003
7 1.675796 -1.326577 -0.092923
7 1.758619 1.029214 -0.120449

8 3.640136 -0.229940 0.262697
6 -1.814791 -0.119727 -0.846038
1 -1.985470 0.357685 -1.818514
7 -0.142378 2.246754 -0.521415
1 0.407715 3.075210 -0.336538
1 -1.150621 2.324558 -0.498172
8 -2.530506 0.677627 0.083705
1 -2.237320 0.427485 0.991721
8 -2.304738 -1.448765 -0.871180
1 -3.208367 -1.420904 -1.220195
1 2.174839 -2.202107 0.021960
8 -1.451915 -0.074635 2.550793
1 -1.523796 -1.040527 2.503250
1 -0.520969 0.106021 2.345400
5dhmC/5dhmCacb.xyz
6 2.799348 -0.406985 -0.103164
6 0.960208 1.031440 -0.161590
6 0.024736 -0.048775 0.046573
6 0.572136 -1.288139 0.190556
1 -0.020741 -2.180033 0.343130
7 1.918101 -1.459847 0.136055
7 2.285619 0.839021 -0.240428
8 4.027180 -0.656775 -0.175093
6 -1.471690 0.176748 0.031686
1 -1.785114 0.578267 -0.940182
7 0.514107 2.293317 -0.316338
1 1.192481 3.040498 -0.382385
1 -0.436083 2.525850 -0.059174
8 -1.865446 1.194395 0.959102
1 -1.524568 0.955042 1.836946
8 -2.129692 -1.036878 0.295238
1 -3.062279 -0.941513 -0.013599
1 2.315507 -2.387699 0.236789
8 -4.672071 -0.713473 -0.680307
1 -4.626927 0.020385 -1.312441
1 -5.265254 -0.402121 0.020000

5dhmU/5dhmUabh.xyz
6 -1.724788 1.149357 0.009665
6 0.049985 -0.556572 0.031681
6 1.037599 0.504307 0.019830
6 0.606280 1.790737 0.010477
1 1.281816 2.638789 0.001936
7 -0.727343 2.100395 0.009619
7 -1.276979 -0.152278 0.024839
8 -2.925660 1.445583 -0.002161
8 0.335739 -1.770477 0.047204
1 -1.999941 -0.892433 0.019238
6 2.506304 0.147299 0.000984
1 -1.022911 3.070998 -0.000547
8 2.884476 -0.613103 1.139171
1 2.314803 -1.400665 1.180038
8 2.873152 -0.515347 -1.202662
1 2.313467 -1.303764 -1.303535
1 3.108974 1.055847 0.034130
8 -3.248512 -2.226751 -0.027132
1 -3.006280 -2.892307 0.634426
1 -3.165514 -2.683292 -0.878079
5dhmU/5dhmUabp.xyz
6 1.633313 1.086738 0.015219
6 -0.840603 1.068174 -0.008786
6 -0.796421 -0.377686 -0.000589
6 0.416807 -0.989115 0.013923
1 0.525628 -2.068171 0.019683
7 1.588179 -0.285322 0.022693
7 0.396088 1.702757 0.000317
8 2.691036 1.728495 0.020203
8 -1.890531 1.739289 -0.022934
1 0.399366 2.719871 -0.005168
6 -2.093802 -1.152293 -0.004048
1 2.488933 -0.794005 0.026539
8 -2.856353 -0.911870 1.171163
1 -3.003590 0.046033 1.248964

8 -2.860279 -0.896195 -1.172720
1 -3.003645 0.063513 -1.237053
1 -1.887128 -2.223626 -0.010696
8 3.975613 -1.790870 0.051958
1 4.454607 -1.700841 -0.785830
1 3.744311 -2.730510 0.106812
5dhmU/5dhmUabs.xyz
6 -1.640907 1.029371 -0.019623
6 0.832230 1.063374 0.015216
6 0.818802 -0.383288 0.016595
6 -0.381354 -1.020359 0.004255
1 -0.467730 -2.101466 0.005827
7 -1.566844 -0.341282 -0.012707
7 -0.417666 1.671871 -0.005961
8 -2.713052 1.647023 -0.035502
8 1.867508 1.756776 0.031674
1 -0.442643 2.688680 -0.006737
6 2.131306 -1.132495 0.016822
1 -2.461337 -0.861052 -0.014047
8 2.855472 -0.928987 -1.190300
1 2.986538 0.026131 -1.314240
8 2.925953 -0.812924 1.149328
1 3.043221 0.152540 1.171793
1 1.946213 -2.206349 0.072366
8 -3.983598 -1.802227 -0.026329
1 -4.211604 -2.044288 0.884146
1 -4.679819 -1.185237 -0.298988
5dhmU/5dhmUaax.xyz
6 -1.632769 -1.086293 0.017704
6 0.841110 -1.068570 -0.005339
6 0.797319 0.377274 -0.023156
6 -0.415916 0.988973 -0.023805
1 -0.524588 2.067977 -0.036914
7 -1.587417 0.285610 -0.005730
7 -0.395657 -1.702593 0.017184
8 -2.690598 -1.727613 0.035117

8 1.890841 -1.740198 -0.009774
1 -0.399026 -2.719649 0.029360
6 2.094638 1.152259 -0.024884
1 -2.488089 0.794220 -0.001768
8 2.819679 0.968881 1.184831
1 2.965665 0.016683 1.315122
8 2.897912 0.841904 -1.154030
1 3.033575 -0.121243 -1.171490
1 1.888820 2.221947 -0.086787
8 -3.978280 1.786248 0.053453
1 -3.754244 2.725705 0.135229
1 -4.463323 1.717613 -0.782821
5dhmU/5dhmUabz.xyz
6 -1.712305 1.092110 0.013435
6 0.088028 -0.587048 0.044406
6 1.058839 0.489293 0.019829
6 0.607497 1.768806 0.000707
1 1.270260 2.626673 -0.018177
7 -0.730788 2.058889 0.001747
7 -1.243961 -0.201919 0.040223
8 -2.918723 1.366315 0.001491
8 0.391987 -1.796369 0.067378
1 -1.964669 -0.944364 0.043992
6 2.533170 0.156084 -0.006157
1 -1.041023 3.024810 -0.016962
8 2.931744 -0.591880 1.133059
1 2.381792 -1.393199 1.176243
8 2.901857 -0.507393 -1.208848
1 2.356418 -1.307055 -1.299241
1 3.120892 1.074664 0.017641
8 -3.358599 -2.121421 0.041991
1 -4.112989 -1.596772 -0.267341
1 -3.224940 -2.793027 -0.643835
5dhmU/5dhmUacs.xyz
6 -1.631788 1.096733 -0.002827
6 0.842288 1.068903 0.006468

6 0.792898 -0.376532 -0.025517
6 -0.422688 -0.983059 -0.048615
1 -0.535497 -2.061472 -0.072221
7 -1.591733 -0.275226 -0.042002
7 -0.392096 1.707637 0.019506
8 -2.686649 1.742808 0.009948
8 1.894587 1.736340 0.021321
1 -0.391636 2.724514 0.042068
6 2.087218 -1.156395 -0.021328
1 -2.493166 -0.782333 -0.043094
8 2.893818 -0.853901 -1.150643
1 3.035759 0.108113 -1.172025
8 2.810707 -0.970686 1.188639
1 2.958469 -0.018159 1.315091
1 1.877408 -2.225495 -0.079916
8 -3.984013 -1.773727 -0.001087
1 -3.958319 -2.473116 -0.671773
1 -4.041387 -2.243490 0.844838
5dhmU/5dhmUaaq.xyz
6 -2.087803 -0.965036 -0.000467
6 -0.047096 0.426765 -0.006691
6 0.748404 -0.779383 -0.014606
6 0.107668 -1.976590 -0.013985
1 0.634883 -2.923715 -0.019733
7 -1.257683 -2.062885 -0.005314
7 -1.422138 0.245538 -0.001294
8 -3.318434 -1.050658 0.004237
8 0.438022 1.577607 -0.007241
1 -2.005920 1.079683 -0.002023
6 2.257343 -0.669754 -0.019144
1 -1.709231 -2.972213 -0.006042
8 2.734249 0.032055 -1.157145
1 2.304932 0.904608 -1.179603
8 2.749585 -0.094432 1.183166
1 2.329929 0.773633 1.306007
1 2.699685 -1.665609 -0.073536

8 -1.254165 3.818341 -0.080319
1 -1.508825 3.999582 0.835354
1 -0.651853 3.046139 -0.026406
5dhmU/5dhmUacb.xyz
6 -2.433512 0.056314 0.024458
6 -0.268600 -1.035910 -0.470544
6 0.367371 -0.131412 0.464683
6 -0.400079 0.785652 1.106784
1 0.007792 1.497658 1.815211
7 -1.749371 0.872943 0.896224
7 -1.641421 -0.871561 -0.620161
8 -3.650342 0.144205 -0.164367
8 0.329268 -1.916884 -1.114834
1 -2.116246 -1.492055 -1.271385
6 1.862509 -0.205440 0.672808
1 -2.297099 1.569832 1.390730
8 2.292870 -1.499976 1.044188
1 1.945823 -2.132097 0.390150
8 2.574095 0.259025 -0.478355
1 2.328112 -0.289513 -1.243144
1 2.158654 0.463463 1.481788
8 1.509634 2.862514 -1.006673
1 1.893943 1.975855 -0.852276
1 0.604234 2.790885 -0.672309
5dhmU/5dhmUact.xyz
6 -1.655776 0.811807 0.023391
6 0.287837 -0.713884 0.037224
6 1.158184 0.443712 0.018570
6 0.596785 1.678885 0.004392
1 1.183045 2.590479 -0.011675
7 -0.762424 1.853663 0.008174
7 -1.076374 -0.437027 0.038484
8 -2.882859 0.983396 0.024153
8 0.686061 -1.891797 0.052088
1 -1.711834 -1.232069 0.055190
6 2.656837 0.236528 -0.003699

1 -1.155536 2.789683 -0.004541
8 3.109941 -0.487084 1.130093
1 2.630837 -1.333173 1.161442
8 3.083325 -0.380582 -1.210927
1 2.606856 -1.221538 -1.313687
1 3.165593 1.200791 0.031865
8 -4.505748 -1.349772 -0.005270
1 -4.060447 -1.919642 -0.648418
1 -3.975906 -0.526552 0.002851
5dhmU/5dhmUabc.xyz
6 -2.100603 0.945815 0.006079
6 -0.045651 -0.424890 0.021533
6 0.737306 0.789120 -0.013069
6 0.084203 1.979273 -0.035522
1 0.601403 2.931488 -0.063898
7 -1.281963 2.051913 -0.023465
7 -1.422403 -0.257644 0.028622
8 -3.332037 1.018625 0.011970
8 0.451460 -1.570306 0.040977
1 -1.997817 -1.097373 0.045309
6 2.246985 0.695514 -0.027596
1 -1.742575 2.956499 -0.041799
8 2.754876 0.133837 1.174321
1 2.348703 -0.739882 1.302823
8 2.722196 -0.009491 -1.164619
1 2.301854 -0.886513 -1.177824
1 2.677809 1.695745 -0.093010
8 -1.210664 -3.828562 -0.120173
1 -0.616204 -3.055527 -0.016413
1 -1.262686 -4.226136 0.760354
5dhmU/5dhmUacv.xyz
6 -1.649512 0.565825 0.013353
6 0.782352 1.000726 -0.006227
6 1.006807 -0.429532 0.048684
6 -0.068545 -1.255516 0.090021
1 0.021649 -2.334844 0.133755

7 -1.350987 -0.772883 0.077131
7 -0.552107 1.395475 -0.015040
8 -2.809571 1.000356 -0.014062
8 1.687080 1.853049 -0.045252
1 -0.745394 2.393319 -0.058794
6 2.423546 -0.957812 0.036973
1 -2.137476 -1.415321 0.102526
8 3.068976 -0.707612 -1.204346
1 3.034581 0.246224 -1.387810
8 3.187689 -0.458802 1.123533
1 3.160036 0.513114 1.093735
1 2.412755 -2.042495 0.151079
8 -4.871664 -0.943425 -0.138500
1 -5.293198 -0.973408 0.731912
1 -4.199264 -0.235415 -0.068856
5dhmU/5dhmUadg.xyz
6 -1.685347 0.718416 0.017382
6 0.765932 1.029520 -0.004832
6 0.916905 -0.410592 -0.036905
6 -0.201536 -1.180820 -0.045169
1 -0.165967 -2.264233 -0.068725
7 -1.456260 -0.635951 -0.020827
7 -0.545772 1.492475 0.018061
8 -2.822527 1.209866 0.045989
8 1.713755 1.836223 0.002704
1 -0.685989 2.499705 0.042127
6 2.305462 -1.009514 -0.037792
1 -2.290037 -1.229491 -0.022218
8 3.078784 -0.563131 -1.140788
1 3.095067 0.409350 -1.124203
8 2.980891 -0.775913 1.191368
1 2.996606 0.180812 1.360753
1 2.240447 -2.093870 -0.136565
8 -4.314099 -1.275250 0.072399
1 -4.699413 -1.523893 -0.780395
1 -4.179408 -0.310465 0.019105

5dhmU/5dhmUaau.xyz
6 2.454080 0.105271 0.014415
6 0.294523 -0.966902 -0.542013
6 -0.336741 -0.154120 0.476616
6 0.425607 0.726663 1.172541
1 0.018352 1.377679 1.937519
7 1.770597 0.848307 0.950241
7 1.664094 -0.778255 -0.692714
8 3.668383 0.218303 -0.177589
8 -0.306779 -1.789707 -1.256231
1 2.136116 -1.336372 -1.400171
6 -1.824281 -0.279918 0.707944
1 2.316172 1.514421 1.487783
8 -2.565629 0.148923 -0.436058
1 -2.294714 -0.391514 -1.198770
8 -2.205457 -1.586257 1.096262
1 -1.876853 -2.214177 0.430109
1 -2.131806 0.381237 1.519089
8 -1.588913 2.800252 -0.921136
1 -1.970659 1.908850 -0.791493
1 -2.331415 3.351422 -1.205770
5dhmU/5dhmUaaz.xyz
6 1.648650 0.801495 -0.032690
6 -0.299212 -0.718655 -0.052429
6 -1.165640 0.440639 0.008412
6 -0.600761 1.673985 0.035940
1 -1.184051 2.586575 0.079409
7 0.758588 1.845358 0.009661
7 1.065923 -0.445349 -0.064343
8 2.876190 0.969923 -0.042658
8 -0.701325 -1.894630 -0.089935
1 1.699518 -1.241003 -0.104006
6 -2.664179 0.237096 0.047753
1 1.154601 2.779855 0.035300
8 -3.148142 -0.377389 -1.137783
1 -2.677866 -1.218992 -1.262432

8 -3.066318 -0.485384 1.202375
1 -2.585048 -1.330287 1.219197
1 -3.168154 1.202597 0.108380
8 4.446641 -1.383331 0.143835
1 3.966652 -0.536277 0.039631
1 5.253250 -1.283403 -0.381289
5dhmU/5dhmUaba.xyz
6 2.566105 0.262678 -0.103818
6 0.181890 0.646013 0.436509
6 -0.095203 -0.732927 0.097628
6 0.944488 -1.515394 -0.296756
1 0.820275 -2.561481 -0.553406
7 2.221457 -1.036997 -0.396698
7 1.504831 1.044626 0.303762
8 3.719199 0.693008 -0.193849
8 -0.664421 1.470938 0.843781
1 1.722415 2.010705 0.537493
6 -1.503614 -1.297366 0.175856
1 2.972425 -1.650772 -0.696896
8 -2.159067 -0.909982 1.373546
1 -2.053391 0.053482 1.474533
8 -2.278433 -0.986405 -0.969102
1 -2.401625 -0.011466 -1.038722
1 -1.446761 -2.387128 0.201266
8 -2.698193 1.795263 -1.070841
1 -2.049751 1.881157 -0.343526
1 -3.562266 1.931503 -0.654984
5dhmU/5dhmUacf.xyz
6 1.735139 1.153868 0.012839
6 -0.039213 -0.552352 0.044153
6 -1.027376 0.508122 0.052315
6 -0.595478 1.794655 0.051413
1 -1.271057 2.642698 0.060032
7 0.737897 2.104851 0.036366
7 1.287054 -0.147078 0.024450
8 2.936068 1.449294 -0.015099

8 -0.321812 -1.767355 0.057524
1 2.007974 -0.889486 0.010223
6 -2.497220 0.153719 0.035093
1 1.033109 3.075630 0.032730
8 -2.898207 -0.377623 -1.223457
1 -2.353460 -1.157439 -1.421471
8 -2.844486 -0.724217 1.093451
1 -2.247487 -1.492261 1.045766
1 -3.098192 1.053114 0.175859
8 3.146156 -2.314378 0.007460
1 3.627116 -2.347934 -0.833155
1 2.530484 -3.062579 -0.029592
5dhmU/5dhmUacc.xyz
6 2.479579 0.117539 -0.002384
6 0.266255 -0.647735 0.796998
6 -0.295523 -0.263567 -0.481499
6 0.526072 0.276375 -1.417269
1 0.174836 0.592948 -2.392772
7 1.862738 0.457026 -1.185665
7 1.630638 -0.422066 0.942593
8 3.686449 0.282344 0.198591
8 -0.389082 -1.141521 1.733030
1 2.054548 -0.682460 1.829815
6 -1.769751 -0.478521 -0.738223
1 2.453385 0.862542 -1.904409
8 -2.097394 -1.849778 -0.891237
1 -1.793900 -2.339029 -0.108322
8 -2.570457 0.124980 0.274636
1 -2.276708 -0.226662 1.135137
1 -2.059363 0.002936 -1.673015
8 -1.908823 2.894692 0.445150
1 -1.870427 3.089747 1.392157
1 -2.141339 1.944962 0.395819
5dhmU/5dhmUaap.xyz
6 2.107727 -0.933077 0.015034
6 0.043627 0.423534 0.061352

6 -0.731089 -0.794433 -0.014294
6 -0.069788 -1.978803 -0.072765
1 -0.580158 -2.933048 -0.136042
7 1.296925 -2.043152 -0.055257
7 1.421366 0.264616 0.074076
8 3.339615 -0.997513 0.025004
8 -0.460814 1.564590 0.109779
1 1.991824 1.106301 0.122454
6 -2.241082 -0.710480 -0.039737
1 1.763390 -2.943693 -0.101415
8 -2.763346 -0.169252 1.165009
1 -2.368526 0.708020 1.304985
8 -2.710770 0.007984 -1.170959
1 -2.295581 0.887428 -1.168430
1 -2.664333 -1.712540 -0.123935
8 1.208806 3.812976 -0.156837
1 0.596092 3.063885 -0.000196
1 0.964950 4.482672 0.497332
5dhmU/5dhmUaco.xyz
6 -1.646627 0.549260 -0.026981
6 0.782620 0.998724 0.015951
6 1.016437 -0.429154 -0.058287
6 -0.053505 -1.261004 -0.118124
1 0.043763 -2.338999 -0.177217
7 -1.339022 -0.786612 -0.106080
7 -0.554410 1.384996 0.019859
8 -2.809327 0.976252 -0.001800
8 1.681260 1.856443 0.074444
1 -0.754031 2.380991 0.075785
6 2.436254 -0.949566 -0.045849
1 -2.120459 -1.433894 -0.148795
8 3.208147 -0.416252 -1.110138
1 3.172642 0.554415 -1.053681
8 3.067630 -0.728943 1.208451
1 3.028837 0.219931 1.415036
1 2.433229 -2.030841 -0.188967

8 -4.881781 -0.956946 0.118387
1 -5.676648 -0.588639 -0.292419
1 -4.211823 -0.245644 0.058798
5dhmU/5dhmUaaj.xyz
6 2.554047 -0.242595 -0.097710
6 0.177411 -0.627628 0.472748
6 -0.114554 0.735723 0.087438
6 0.914974 1.513073 -0.342298
1 0.779283 2.548043 -0.635514
7 2.195410 1.042392 -0.434764
7 1.503635 -1.018216 0.348343
8 3.710066 -0.666154 -0.182270
8 -0.660513 -1.445882 0.909769
1 1.731846 -1.973609 0.613915
6 -1.526917 1.291566 0.157980
1 2.938845 1.651827 -0.761360
8 -2.309028 0.940483 -0.969877
1 -2.416065 -0.037834 -1.016399
8 -2.170531 0.938514 1.372409
1 -2.058032 -0.020541 1.503107
1 -1.477491 2.381989 0.148962
8 -2.705060 -1.837695 -0.986042
1 -2.016729 -1.919010 -0.296017
1 -2.324211 -2.255051 -1.772592
5fC/5fCabv.xyz
6 -1.273728 0.661999 -0.165717
6 0.591151 -0.733122 -0.110512
6 1.494948 0.392954 0.050633
6 0.909527 1.640276 0.096835
1 1.486647 2.551351 0.215719
7 -0.418946 1.770300 -0.006010
7 -0.737008 -0.572530 -0.213953
8 -2.500350 0.891799 -0.255433
7 1.075421 -1.975012 -0.160138
1 0.439459 -2.753830 -0.274203
1 2.074852 -2.129108 -0.086854

6 2.936106 0.298169 0.162348
1 3.461824 1.261094 0.281994
8 3.583884 -0.758162 0.132376
1 -0.850256 2.689764 0.029368
8 -4.274000 -1.188500 0.277689
1 -4.843426 -0.830410 0.972981
1 -3.629665 -0.470927 0.083127
5fC/5fCaak.xyz
6 -2.625599 -0.104608 0.067799
6 -0.539474 0.937480 -0.022487
6 0.154711 -0.338709 -0.093581
6 -0.640912 -1.466578 -0.092743
1 -0.230156 -2.469672 -0.145604
7 -1.970547 -1.355363 -0.022380
7 -1.874612 1.019722 0.060120
8 -3.868146 -0.111549 0.150451
7 0.160147 2.074293 -0.038046
1 -0.325080 2.960442 0.016800
1 1.172345 2.043225 -0.080824
6 1.585251 -0.510052 -0.143371
1 1.936049 -1.553801 -0.179534
8 2.413876 0.417543 -0.141034
1 -2.556732 -2.185414 -0.019520
8 5.072192 -0.435621 0.127641
1 5.111184 -0.893568 0.979041
1 4.140243 -0.139950 0.041607
5fC/5fCadf.xyz
6 -1.333194 1.132110 0.026987
6 0.263338 -0.585100 0.043285
6 1.359715 0.365258 -0.009019
6 1.010262 1.698669 -0.031071
1 1.748761 2.493291 -0.065152
7 -0.276615 2.066650 -0.010627
7 -1.017411 -0.184002 0.058420
8 -2.499507 1.563249 0.031134
7 0.509585 -1.894688 0.080427

1 -0.262009 -2.549340 0.117260
1 1.468764 -2.223500 0.068089
6 2.763935 0.014663 -0.041525
1 3.460204 0.869963 -0.077697
8 3.207848 -1.142830 -0.032538
1 -0.530762 3.050128 -0.028274
8 -3.061351 -2.153088 -0.002311
1 -2.387714 -1.430256 0.002651
1 -3.566417 -2.018246 -0.816624
5fC/5fCaaw.xyz
6 -1.125265 1.197140 -0.016078
6 1.179008 0.829523 0.006198
6 1.000609 -0.611966 -0.010320
6 -0.305545 -1.059685 -0.032862
1 -0.555898 -2.116142 -0.045801
7 -1.325692 -0.198464 -0.037360
7 0.140502 1.676348 0.002964
8 -2.137763 1.926876 -0.015889
7 2.411374 1.345747 0.026059
1 2.532976 2.349965 0.037771
1 3.213677 0.726289 0.029921
6 2.067890 -1.586260 -0.004057
1 1.739408 -2.639625 -0.019946
8 3.280431 -1.319829 0.017871
1 -2.295690 -0.553763 -0.043099
8 -3.906513 -1.395466 -0.008627
1 -4.152596 -1.564896 0.913286
1 -3.854579 -2.272409 -0.417887
5fC/5fCacr.xyz
6 2.624249 -0.115021 0.056835
6 0.544660 0.941117 -0.020236
6 -0.158994 -0.330455 -0.080331
6 0.628798 -1.463800 -0.081453
1 0.210506 -2.464143 -0.126430
7 1.959755 -1.361546 -0.022953
7 1.880911 1.014353 0.051366

8 3.867331 -0.130026 0.128886
7 -0.146974 2.082812 -0.034919
1 0.345127 2.965633 0.012120
1 -1.159632 2.058740 -0.069928
6 -1.591105 -0.491839 -0.116326
1 -1.949625 -1.533333 -0.146057
8 -2.413293 0.441416 -0.109045
1 2.540035 -2.195732 -0.021258
8 -5.074619 -0.408613 0.146694
1 -5.022180 -1.322767 0.459484
1 -4.141071 -0.119960 0.054400
5fC/5fCadg.xyz
6 1.161730 1.092815 0.011015
6 -1.157174 0.841565 -0.009185
6 -1.050954 -0.606839 0.012822
6 0.231210 -1.120660 0.038438
1 0.427389 -2.188456 0.055732
7 1.292310 -0.310547 0.040230
7 -0.077862 1.636089 -0.011462
8 2.210526 1.769986 0.008577
7 -2.362283 1.418895 -0.028357
1 -2.432791 2.427874 -0.038378
1 -3.195817 0.841919 -0.025745
6 -2.165184 -1.526629 0.009064
1 -1.888405 -2.594709 0.028403
8 -3.363088 -1.201428 -0.014999
1 2.249544 -0.701149 0.046396
8 3.921534 -1.399291 0.043608
1 4.106056 -1.810757 -0.814428
1 4.499316 -0.621431 0.074731
5fC/5fCadj.xyz
6 -2.630841 -0.097892 0.053504
6 -0.540141 0.936185 -0.022591
6 0.150163 -0.342875 -0.076803
6 -0.649400 -1.467884 -0.076573
1 -0.241781 -2.472804 -0.117175

7 -1.979346 -1.351467 -0.022025
7 -1.875773 1.023831 0.045705
8 -3.874130 -0.100141 0.124295
7 0.163940 2.070289 -0.039427
1 -0.318332 2.958640 0.005154
1 1.176425 2.035107 -0.070402
6 1.580660 -0.520368 -0.106010
1 1.926754 -1.565955 -0.131593
8 2.413486 0.403398 -0.097493
1 -2.568314 -2.179529 -0.019350
8 5.038545 -0.545715 0.213809
1 4.139556 -0.177272 0.075436
1 5.638107 0.099915 -0.185896
5fC/5fCacu.xyz
6 -2.627442 -0.112469 0.053608
6 -0.546709 0.941539 -0.022172
6 0.155947 -0.330724 -0.074585
6 -0.632747 -1.463430 -0.072742
1 -0.215117 -2.464297 -0.111787
7 -1.963785 -1.359848 -0.018720
7 -1.883089 1.016319 0.045338
8 -3.870737 -0.126151 0.122157
7 0.146214 2.082487 -0.039678
1 -0.345053 2.966045 0.002126
1 1.158902 2.057306 -0.072127
6 1.588009 -0.494805 -0.105283
1 1.943044 -1.537508 -0.129262
8 2.413040 0.436029 -0.099967
1 -2.544638 -2.193623 -0.014924
8 5.020288 -0.587498 0.164293
1 5.645793 0.150023 0.193948
1 4.138607 -0.170354 0.058622
5fC/5fCaci.xyz
6 1.136350 1.164733 0.001957
6 -1.173253 0.833893 0.001242
6 -1.017357 -0.610326 -0.000883

6 0.281785 -1.079077 -0.002193
1 0.515531 -2.139421 -0.004228
7 1.315064 -0.233619 -0.000926
7 -0.121591 1.664611 0.002883
8 2.160702 1.878153 0.003211
7 -2.397616 1.369144 0.001624
1 -2.504024 2.375104 0.002476
1 -3.209575 0.762340 0.000040
6 -2.099708 -1.567877 -0.002305
1 -1.786913 -2.626107 -0.004071
8 -3.308198 -1.282920 -0.001646
1 2.280724 -0.601619 -0.001865
8 3.850670 -1.508766 -0.026685
1 4.457033 -1.068350 -0.641095
1 4.283935 -1.442707 0.837727
5fC/5fCabu.xyz
6 1.238130 -0.598168 0.027470
6 -1.077770 -0.851070 -0.005865
6 -1.287795 0.586242 0.004582
6 -0.152445 1.366800 0.027014
1 -0.190156 2.451395 0.037280
7 1.060782 0.799872 0.037802
7 0.149697 -1.392519 0.006796
8 2.409492 -1.038171 0.039060
7 -2.126276 -1.675705 -0.028593
1 -1.973941 -2.676031 -0.036758
1 -3.065960 -1.295129 -0.037647
6 -2.578147 1.243712 -0.005713
1 -2.541021 2.346251 0.003981
8 -3.673648 0.664142 -0.024030
1 1.897090 1.377998 0.054739
8 4.606456 0.667416 0.041023
1 4.855720 0.788334 -0.885959
1 3.838609 0.053465 0.018966
5fC/5fCabt.xyz
6 1.136767 1.165079 -0.008307

6 -1.172788 0.834375 0.008132
6 -1.017218 -0.609735 -0.012122
6 0.281726 -1.078453 -0.033801
1 0.515326 -2.138736 -0.049752
7 1.314988 -0.233033 -0.033741
7 -0.121012 1.664971 0.010496
8 2.161360 1.878166 -0.005342
7 -2.397050 1.369587 0.025725
1 -2.503455 2.375473 0.037460
1 -3.209042 0.762823 0.023950
6 -2.099581 -1.567298 -0.010592
1 -1.786865 -2.625413 -0.028692
8 -3.307916 -1.282477 0.009810
1 2.280572 -0.600915 -0.038668
8 3.859642 -1.499374 -0.028633
1 4.562738 -0.897549 -0.316094
1 4.084116 -1.720682 0.887892
5fC/5fCaba.xyz
6 -1.129140 1.188046 -0.012542
6 1.176841 0.832320 0.009078
6 1.005921 -0.610019 -0.013055
6 -0.297832 -1.064455 -0.039616
1 -0.542888 -2.122109 -0.057819
7 -1.321992 -0.208082 -0.041251
7 0.133960 1.673993 0.009680
8 -2.146042 1.911935 -0.009715
7 2.406652 1.354496 0.030401
1 2.523603 2.359217 0.044271
1 3.211990 0.738939 0.030683
6 2.078024 -1.579106 -0.009361
1 1.754462 -2.633922 -0.029892
8 3.289194 -1.306924 0.015335
1 -2.290280 -0.567494 -0.049633
8 -3.871559 -1.477806 -0.057323
1 -4.564062 -0.913349 0.318434
1 -3.798806 -2.222481 0.558753

5fC/5fCaca.xyz
6 1.117598 1.223789 -0.007233
6 -1.182168 0.827744 0.002515
6 -0.986273 -0.611561 -0.002115
6 0.325315 -1.043119 -0.011874
1 0.589909 -2.096209 -0.016729
7 1.334635 -0.169275 -0.014869
7 -0.154066 1.687253 0.000012
8 2.121352 1.965569 -0.007386
7 -2.420796 1.329092 0.009745
1 -2.554449 2.331878 0.012977
1 -3.215465 0.699845 0.012199
6 -2.041836 -1.598514 0.001504
1 -1.701148 -2.648080 -0.003785
8 -3.257566 -1.346228 0.010129
1 2.307013 -0.516609 -0.019966
8 3.869601 -1.458670 -0.063974
1 3.647814 -2.399745 -0.132243
1 4.345007 -1.375981 0.776406
5fC/5fCaar.xyz
6 -1.266375 0.681484 -0.033815
6 0.583811 -0.734359 -0.019894
6 1.507290 0.386275 0.012980
6 0.939206 1.642649 0.021818
1 1.532626 2.550687 0.046035
7 -0.391935 1.785058 -0.001124
7 -0.746608 -0.561260 -0.042160
8 -2.493766 0.922878 -0.054457
7 1.051717 -1.983642 -0.029734
1 0.402031 -2.758951 -0.055003
1 2.052374 -2.146815 -0.015479
6 2.951528 0.276524 0.033574
1 3.495194 1.236395 0.058042
8 3.584380 -0.789163 0.025563
1 -0.811258 2.710831 0.005941
8 -4.327502 -1.167671 -0.022555

1 -4.812345 -1.061945 0.807943
1 -3.648497 -0.455086 -0.012722
5fC/5fCaah.xyz
6 1.157819 1.104741 -0.012922
6 -1.159606 0.840766 0.008829
6 -1.045427 -0.607091 -0.013267
6 0.239507 -1.113698 -0.041619
1 0.441769 -2.180375 -0.059731
7 1.295958 -0.297700 -0.044968
7 -0.084704 1.641338 0.010639
8 2.203222 1.787196 -0.009134
7 -2.368052 1.410989 0.028632
1 -2.444893 2.419501 0.038852
1 -3.197940 0.828801 0.027262
6 -2.154461 -1.533164 -0.007283
1 -1.871610 -2.599640 -0.026881
8 -3.354191 -1.214780 0.018990
1 2.255124 -0.683596 -0.052382
8 3.920927 -1.388449 -0.052655
1 4.506689 -0.654852 0.188981
1 4.021784 -2.033280 0.663739
5fC/5fCabr.xyz
6 -1.273120 0.702145 -0.004848
6 0.566399 -0.728195 -0.002933
6 1.498431 0.385747 0.001533
6 0.940166 1.646559 0.002217
1 1.540883 2.550100 0.005327
7 -0.390041 1.799091 -0.001179
7 -0.762901 -0.544968 -0.006019
8 -2.498722 0.952354 -0.006934
7 1.025632 -1.980757 -0.004228
1 0.370962 -2.752188 -0.007435
1 2.025352 -2.150498 -0.001990
6 2.941896 0.265246 0.004804
1 3.492795 1.221223 0.008153
8 3.566767 -0.805167 0.004093

1 -0.802256 2.728092 -0.000315
8 -4.350027 -1.115627 0.017079
1 -3.641545 -0.432122 0.005938
1 -3.881792 -1.959654 -0.048243
5fC/5fCadk.xyz
6 2.625334 -0.103403 0.070727
6 0.538641 0.937112 -0.024181
6 -0.154443 -0.339602 -0.096229
6 0.641995 -1.466900 -0.093213
1 0.232095 -2.470319 -0.146561
7 1.971390 -1.354685 -0.020132
7 1.873548 1.020372 0.061096
8 3.867733 -0.109403 0.155746
7 -0.161805 2.073396 -0.041391
1 0.322635 2.959927 0.014220
1 -1.173879 2.041579 -0.086550
6 -1.584718 -0.512069 -0.149369
1 -1.934662 -1.556107 -0.184707
8 -2.414065 0.414918 -0.150773
1 2.558201 -2.184286 -0.015847
8 -5.065939 -0.454906 0.130064
1 -4.139012 -0.145767 0.037301
1 -5.149993 -0.714305 1.058430
5fC/5fCaaf.xyz
6 1.254057 0.708514 -0.026165
6 -1.070542 0.855505 0.006377
6 -1.214624 -0.589993 -0.008311
6 -0.044310 -1.317317 -0.038734
1 -0.030208 -2.402324 -0.054523
7 1.141352 -0.696199 -0.049927
7 0.130789 1.452965 -0.002625
8 2.405791 1.200437 -0.030248
7 -2.156005 1.630880 0.028593
1 -2.051862 2.637379 0.038825
1 -3.076371 1.205510 0.037135
6 -2.473499 -1.305369 0.005073

1 -2.386641 -2.405345 -0.007703
8 -3.593613 -0.774642 0.029198
1 2.007532 -1.233102 -0.067673
8 4.277044 -0.869704 -0.034811
1 3.757637 -0.036448 -0.000534
1 4.546700 -1.035969 0.879648
5fC/5fCacq.xyz
6 -1.271761 0.698997 -0.010452
6 0.569276 -0.729273 -0.004575
6 1.500118 0.385636 0.002879
6 0.940491 1.645844 0.001954
1 1.540206 2.550045 0.006653
7 -0.389870 1.796912 -0.005166
7 -0.760207 -0.547492 -0.011065
8 -2.497621 0.947926 -0.014622
7 1.029766 -1.981367 -0.005495
1 0.375827 -2.753412 -0.010758
1 2.029637 -2.150122 -0.000735
6 2.943701 0.266640 0.009625
1 3.493622 1.223178 0.014966
8 3.569677 -0.803128 0.009701
1 -0.803094 2.725464 -0.005023
8 -4.357129 -1.111305 0.059642
1 -3.644533 -0.433241 0.020038
1 -3.959844 -1.913295 -0.307412
5fC/5fCads.xyz
6 1.136341 1.677473 -0.007130
6 -0.337319 -0.135441 -0.019066
6 0.759294 -1.091620 0.003794
6 2.032123 -0.561248 0.021423
1 2.921486 -1.182847 0.039949
7 2.214934 0.764348 0.015546
7 -0.122829 1.190956 -0.023565
8 1.412022 2.894271 -0.010859
7 -1.597420 -0.568403 -0.036249
1 -2.374125 0.096965 -0.044317

1 -1.772711 -1.566974 -0.029420
6 0.613811 -2.531067 0.012369
1 1.561078 -3.096923 0.031529
8 -0.465926 -3.141714 0.000825
1 3.152832 1.154719 0.029361
8 -3.911730 1.249315 -0.068314
1 -3.766377 1.954654 0.579826
1 -4.685401 0.768532 0.261392
5fU/5fUacj.xyz
6 1.259696 0.560578 -0.007726
6 -1.198894 0.914439 0.003555
6 -1.360492 -0.537961 -0.000119
6 -0.234443 -1.324600 -0.006582
1 -0.291970 -2.408515 -0.009811
7 1.011578 -0.803968 -0.009727
7 0.134781 1.343783 -0.000664
8 2.402393 1.024144 -0.012343
8 -2.101510 1.754216 0.009918
1 0.292479 2.349170 0.000917
6 -2.652853 -1.211960 0.002453
1 -2.585393 -2.314549 -0.000358
8 -3.756611 -0.663998 0.007239
1 1.825196 -1.414807 -0.015006
8 4.583593 -0.793743 -0.083817
1 4.971653 -0.822340 0.802103
1 3.862522 -0.135592 -0.022571
5fU/5fUaak.xyz
6 2.635199 -0.205904 0.020385
6 0.474237 1.026396 -0.010081
6 -0.210268 -0.264808 -0.034222
6 0.549544 -1.411455 -0.034686
1 0.097293 -2.397808 -0.051944
7 1.896450 -1.384475 -0.010870
7 1.870611 0.936315 0.018652
8 3.863140 -0.203326 0.047629
8 -0.058282 2.138693 -0.012541

1 2.383046 1.815442 0.036741
6 -1.653577 -0.417683 -0.044763
1 -2.001131 -1.464638 -0.060506
8 -2.480151 0.501811 -0.033692
1 2.427260 -2.251473 -0.008838
8 -5.091519 -0.551896 0.099972
1 -4.204575 -0.136790 0.039909
1 -5.707627 0.130857 -0.200598
5fU/5fUaae.xyz
6 -1.193323 1.149487 -0.018844
6 1.278955 0.876110 0.012431
6 1.071463 -0.568632 -0.013380
6 -0.221316 -1.041591 -0.044334
1 -0.438391 -2.105592 -0.063375
7 -1.296617 -0.232438 -0.049389
7 0.097685 1.625818 0.010213
8 -2.182311 1.882934 -0.017758
8 2.365582 1.462951 0.035290
1 0.200090 2.637916 0.027523
6 2.148489 -1.546361 -0.007797
1 1.802720 -2.595692 -0.033099
8 3.357410 -1.300069 0.022829
1 -2.247709 -0.648529 -0.053613
8 -3.740168 -1.599706 -0.043364
1 -3.524739 -2.520798 0.168787
1 -4.309153 -1.303918 0.683581
5fU/5fUaat.xyz
6 1.204577 1.102682 -0.018661
6 -1.272472 0.881114 0.016900
6 -1.095221 -0.567194 -0.017443
6 0.187287 -1.067486 -0.052757
1 0.380332 -2.135914 -0.076985
7 1.279443 -0.281032 -0.055636
7 -0.075920 1.605975 0.016723
8 2.208376 1.815874 -0.017726
8 -2.346659 1.490252 0.044698

1 -0.157101 2.619907 0.038334
6 -2.193158 -1.521272 -0.015685
1 -1.870666 -2.577805 -0.047135
8 -3.396249 -1.248338 0.018140
1 2.224970 -0.710560 -0.057467
8 3.749572 -1.607309 -0.028142
1 4.470199 -0.958884 -0.008236
1 3.821206 -2.082233 0.814003
5fU/5fUabr.xyz
6 2.632583 0.212193 -0.026544
6 0.476952 -1.029288 0.013192
6 -0.212782 0.258989 0.041346
6 0.542238 1.408765 0.039584
1 0.085844 2.393140 0.059759
7 1.889102 1.387619 0.009400
7 1.872804 -0.933276 -0.022321
8 3.860384 0.214763 -0.059725
8 -0.050982 -2.143772 0.017528
1 2.388963 -1.810155 -0.043278
6 -1.656649 0.405976 0.056940
1 -2.008348 1.451604 0.073735
8 -2.479649 -0.516763 0.048379
1 2.416124 2.256926 0.005513
8 -5.077932 0.571537 -0.087982
1 -4.202502 0.132231 -0.033363
1 -5.722035 -0.150074 -0.064630
5fU/5fUaad.xyz
6 -1.202309 -1.109975 -0.002436
6 1.274351 -0.879549 0.003933
6 1.091305 0.568485 -0.003994
6 -0.193465 1.064332 -0.011453
1 -0.390701 2.132209 -0.017126
7 -1.282816 0.273957 -0.011168
7 0.080439 -1.608867 0.005200
8 -2.203216 -1.827211 -0.001717
8 2.350946 -1.485046 0.009357

1 0.165560 -2.622688 0.009794
6 2.185607 1.526682 -0.004553
1 1.858636 2.582258 -0.011524
8 3.390166 1.258123 0.002206
1 -2.229511 0.701955 -0.010794
8 -3.716555 1.642892 -0.014604
1 -4.146667 1.583784 0.852154
1 -4.344340 1.236944 -0.631644
5fU/5fUaam.xyz
6 2.623503 0.230549 -0.021771
6 0.482164 -1.036009 0.007949
6 -0.222481 0.244211 0.035724
6 0.519263 1.402439 0.037706
1 0.051280 2.381301 0.057972
7 1.866267 1.397241 0.011730
7 1.877025 -0.923684 -0.021423
8 3.851303 0.247481 -0.049591
8 -0.032962 -2.156418 0.008261
1 2.403551 -1.794411 -0.041037
6 -1.667754 0.373912 0.050573
1 -2.033725 1.414961 0.069777
8 -2.478795 -0.559344 0.039719
1 2.382925 2.272764 0.010470
8 -5.114859 0.430129 -0.043141
1 -4.201857 0.071355 -0.020147
1 -5.010893 1.353740 -0.312252
5fU/5fUaaz.xyz
6 -1.203268 1.107189 -0.014870
6 1.273464 0.880283 0.014260
6 1.092916 -0.567738 -0.015105
6 -0.190824 -1.065274 -0.044658
1 -0.386315 -2.133315 -0.065295
7 -1.281340 -0.276500 -0.046513
7 0.078467 1.607730 0.014971
8 -2.205366 1.822764 -0.013779
8 2.349021 1.487201 0.037401

1 0.161917 2.621542 0.033220
6 2.188675 -1.524316 -0.014231
1 1.863566 -2.580168 -0.040920
8 3.392539 -1.254121 0.013967
1 -2.227447 -0.705070 -0.046876
8 -3.731199 -1.629867 -0.014789
1 -4.457790 -1.054442 -0.298753
1 -3.939562 -1.855841 0.904653
5fU/5fUaab.xyz
6 -1.189616 1.154817 0.008141
6 1.282055 0.872972 -0.004657
6 1.069220 -0.571261 0.005916
6 -0.225398 -1.039964 0.018602
1 -0.445744 -2.103356 0.026195
7 -1.298287 -0.227398 0.020790
7 0.103395 1.626656 -0.003861
8 -2.175469 1.892235 0.007712
8 2.370814 1.456206 -0.013936
1 0.209368 2.638511 -0.010853
6 2.142921 -1.552640 0.003207
1 1.793486 -2.600998 0.013579
8 3.352956 -1.310261 -0.009752
1 -2.250937 -0.639901 0.021113
8 -3.772457 -1.541016 -0.006168
1 -3.852601 -2.014047 -0.848548
1 -3.771168 -2.235857 0.669920
5fU/5fUacp.xyz
6 1.198801 1.125811 0.019978
6 -1.276014 0.878894 -0.016319
6 -1.084029 -0.567638 0.017452
6 0.203488 -1.054517 0.054072
1 0.407883 -2.120859 0.078578
7 1.287532 -0.256972 0.057834
7 -0.086848 1.615892 -0.015274
8 2.195447 1.848914 0.018260
8 -2.356316 1.477083 -0.044487

1 -0.178307 2.628946 -0.037239
6 -2.171622 -1.533589 0.013884
1 -1.837361 -2.586458 0.045668
8 -3.377637 -1.274128 -0.021723
1 2.235853 -0.679726 0.061604
8 3.743248 -1.609107 0.039630
1 4.451375 -1.053127 -0.320020
1 3.654082 -2.337076 -0.594355
5fU/5fUaan.xyz
6 2.631436 -0.208399 -0.043089
6 0.472607 1.026205 0.018648
6 -0.212400 -0.264274 0.063511
6 0.546355 -1.411628 0.060439
1 0.093618 -2.397383 0.093122
7 1.892676 -1.386113 0.011986
7 1.868030 0.934614 -0.037292
8 3.858686 -0.207027 -0.093267
8 -0.058869 2.138937 0.025208
1 2.380949 1.813055 -0.069521
6 -1.655532 -0.414804 0.093791
1 -2.005445 -1.460753 0.119577
8 -2.480121 0.506603 0.086469
1 2.422742 -2.253571 0.006848
8 -5.115362 -0.473135 -0.036319
1 -4.203083 -0.112301 -0.013605
1 -5.343197 -0.514187 -0.975801
5fU/5fUacl.xyz
6 -1.259518 0.557184 -0.023933
6 1.198452 0.914537 0.007161
6 1.362208 -0.537574 -0.005107
6 0.237410 -1.325705 -0.027660
1 0.296719 -2.409462 -0.039454
7 -1.009316 -0.806867 -0.037328
7 -0.135811 1.341931 -0.004607
8 -2.402814 1.019189 -0.030248
8 2.099705 1.755571 0.026917

1 -0.295071 2.347037 0.004358
6 2.655417 -1.209914 0.006849
1 2.589446 -2.312568 -0.001010
8 3.758360 -0.660595 0.024719
1 -1.821899 -1.418752 -0.055440
8 -4.605032 -0.775057 -0.049010
1 -3.866680 -0.134262 -0.012777
1 -4.852196 -0.921467 0.874986
5fU/5fUaas.xyz
6 -2.341093 0.394786 0.096853
6 0.096499 0.595051 -0.358166
6 0.217430 -0.838685 -0.098475
6 -0.917924 -1.545021 0.223090
1 -0.893325 -2.612770 0.415941
7 -2.128147 -0.960869 0.323532
7 -1.205003 1.095451 -0.229980
8 -3.455551 0.904608 0.183595
8 1.008052 1.360272 -0.678431
1 -1.330775 2.088877 -0.411993
6 1.462104 -1.583568 -0.182590
1 1.348955 -2.671637 -0.035456
8 2.584193 -1.113027 -0.396537
1 -2.946984 -1.513853 0.561889
8 3.601704 1.246436 0.844125
1 2.942689 1.948926 0.747431
1 3.202208 0.496702 0.361044
5fU/5fUaag.xyz
6 -2.630400 -0.209678 0.045866
6 -0.472672 1.026661 -0.018546
6 0.213089 -0.263217 -0.069421
6 -0.544873 -1.411091 -0.068322
1 -0.091538 -2.396416 -0.105519
7 -1.891083 -1.386708 -0.015771
7 -1.867824 0.933914 0.041689
8 -3.857495 -0.209260 0.099950
8 0.058007 2.139772 -0.023781

1 -2.381291 1.811873 0.077946
6 1.656292 -0.412412 -0.102008
1 2.007236 -1.457907 -0.133061
8 2.479978 0.509753 -0.090763
1 -2.420523 -2.254558 -0.011737
8 5.120219 -0.453460 0.048881
1 5.260912 -0.704072 0.972804
1 4.203261 -0.105384 0.018428
5fU/5fUace.xyz
6 -2.633499 -0.210126 -0.016122
6 -0.475261 1.027107 0.009745
6 0.212304 -0.262563 0.028979
6 -0.544908 -1.410926 0.029130
1 -0.090277 -2.396227 0.042810
7 -1.891890 -1.387056 0.009735
7 -1.871530 0.933828 -0.014066
8 -3.861511 -0.210392 -0.039110
8 0.054674 2.140614 0.012435
1 -2.386032 1.811810 -0.029002
6 1.655886 -0.412099 0.035373
1 2.006495 -1.458069 0.048343
8 2.480055 0.509623 0.023952
1 -2.420674 -2.255293 0.007548
8 5.103790 -0.496074 -0.149267
1 4.202821 -0.119396 -0.053717
1 5.528414 -0.348749 0.707626
5fU/5fUacf.xyz
6 2.327706 -0.381257 -0.074320
6 -0.110856 -0.586234 0.348630
6 -0.240016 0.838546 0.065477
6 0.889948 1.539638 -0.281938
1 0.858298 2.600246 -0.509881
7 2.105985 0.961559 -0.354406
7 1.190894 -1.080636 0.259287
8 3.445838 -0.887077 -0.126496
8 -1.027102 -1.362127 0.648116

1 1.318828 -2.070090 0.461259
6 -1.488500 1.584198 0.167042
1 -1.399085 2.649343 -0.112981
8 -2.573657 1.134266 0.537119
1 2.922326 1.512698 -0.605541
8 -3.420856 -1.384747 -0.902939
1 -2.618597 -1.321861 -0.348183
1 -3.823394 -0.508666 -0.814581
5fU/5fUabe.xyz
6 -1.256953 0.808608 -0.013392
6 0.613936 -0.826484 -0.012578
6 1.539638 0.303237 0.002434
6 1.022109 1.576055 0.006982
1 1.659965 2.453973 0.017728
7 -0.306482 1.817445 -0.001100
7 -0.738509 -0.460974 -0.019325
8 -2.465766 1.050826 -0.018532
8 0.913646 -2.022293 -0.019617
1 -1.417241 -1.219750 -0.029176
6 2.990459 0.159881 0.015400
1 3.540240 1.118173 0.025755
8 3.611526 -0.904561 0.016232
1 -0.655665 2.772168 0.003762
8 -4.292256 -1.173580 0.045200
1 -3.687263 -0.406900 0.016345
1 -3.717432 -1.943878 -0.070786
5fU/5fUaay.xyz
6 -2.634081 -0.186054 0.070703
6 -0.459508 1.016946 -0.032996
6 0.205120 -0.283307 -0.099034
6 -0.570196 -1.419483 -0.087682
1 -0.133218 -2.411761 -0.135753
7 -1.914825 -1.374129 -0.011107
7 -1.854313 0.945697 0.056287
8 -3.859782 -0.166710 0.149455
8 0.088130 2.121719 -0.047459

1 -2.353338 1.831255 0.106695
6 1.645415 -0.453715 -0.153028
1 1.980685 -1.504194 -0.182113
8 2.481765 0.456857 -0.160343
1 -2.457834 -2.233502 -0.000658
8 5.123792 -0.459436 0.139002
1 4.206555 -0.133500 0.014766
1 5.349381 -0.215034 1.047778
5fU/5fUacu.xyz
6 2.317017 -0.355244 0.079116
6 -0.111687 -0.590017 -0.383493
6 -0.267017 0.827713 -0.078991
6 0.847400 1.540389 0.294858
1 0.795666 2.596497 0.539467
7 2.071278 0.980252 0.374041
7 1.195596 -1.066217 -0.281139
8 3.441370 -0.845714 0.141517
8 -1.011612 -1.374861 -0.708894
1 1.340704 -2.050993 -0.494064
6 -1.526088 1.555627 -0.180958
1 -1.457126 2.615250 0.124131
8 -2.599617 1.097584 -0.574654
1 2.875217 1.539754 0.645987
8 -3.303891 -1.392380 0.992487
1 -3.633090 -0.481899 0.984670
1 -2.557255 -1.364696 0.362651
5fU/5fUacy.xyz
6 2.374957 -0.449266 0.067154
6 -0.092564 -0.596231 -0.217127
6 -0.153435 0.855581 -0.060113
6 1.021901 1.543662 0.131242
1 1.043566 2.622511 0.246060
7 2.218698 0.926609 0.194101
7 1.198155 -1.131606 -0.129222
8 3.476077 -0.991087 0.125775
8 -1.045446 -1.351104 -0.418606

1 1.280743 -2.140191 -0.235841
6 -1.381504 1.630984 -0.110934
1 -1.231876 2.721450 -0.027903
8 -2.526095 1.184189 -0.235524
1 3.066998 1.466660 0.341541
8 -3.906290 -1.181759 0.498239
1 -3.360249 -1.972877 0.386884
1 -3.319251 -0.452875 0.214696
5hmC/5hmCaai.xyz
6 -1.332610 0.605701 0.013753
6 0.975133 0.912009 0.100596
6 1.225430 -0.502535 0.239790
6 0.121883 -1.300957 0.223950
1 0.174398 -2.380327 0.309764
7 -1.122868 -0.765042 0.109922
7 -0.260340 1.426966 0.001697
8 -2.516190 1.032408 -0.065957
6 2.606724 -1.069508 0.412278
1 3.102044 -0.626280 1.281917
1 2.539231 -2.150897 0.576115
7 2.005603 1.776042 0.084320
1 1.819517 2.756907 -0.078014
1 2.953186 1.442482 -0.025795
8 3.477783 -0.777952 -0.700614
1 3.084303 -1.160232 -1.500173
1 -1.938073 -1.369820 0.108380
8 -4.598550 -0.787455 -0.091787
1 -4.706629 -1.056868 -1.014667
1 -3.868448 -0.124992 -0.104431
5hmC/5hmCaab.xyz
6 -1.260829 -1.092735 0.037971
6 1.063116 -0.932360 -0.094919
6 1.021479 0.500099 -0.257465
6 -0.223936 1.057181 -0.236101
1 -0.389912 2.124206 -0.339178
7 -1.333282 0.290001 -0.092689

7 -0.039520 -1.683750 0.038711
8 -2.331572 -1.740862 0.158199
6 2.256605 1.334189 -0.447697
1 2.848043 0.968259 -1.292961
1 1.968542 2.370057 -0.660070
7 2.248424 -1.574265 -0.097265
1 2.263942 -2.567499 0.092450
1 3.112667 -1.058755 -0.008457
8 3.154431 1.282588 0.682582
1 2.679508 1.615893 1.459405
1 -2.265176 0.724835 -0.071300
8 -3.955671 1.488088 0.009309
1 -3.943177 2.300862 0.536575
1 -4.479906 0.861469 0.530796
5hmC/5hmCadr.xyz
6 -1.410912 -1.095978 -0.050014
6 0.207422 0.589438 0.159189
6 1.288865 -0.364831 0.203184
6 0.926814 -1.673770 0.086352
1 1.646129 -2.485050 0.100540
7 -0.379079 -2.030538 -0.040909
7 -1.077298 0.216359 0.043088
8 -2.594555 -1.491780 -0.149575
6 2.722028 0.043730 0.401105
1 2.830006 0.638171 1.313784
1 3.345073 -0.851649 0.504056
7 0.458825 1.905253 0.254989
1 -0.306266 2.563762 0.186317
1 1.404022 2.256295 0.189914
8 3.220248 0.889744 -0.655677
1 3.154780 0.400157 -1.490101
1 -0.635538 -3.009418 -0.112360
8 -3.176663 2.086443 -0.079784
1 -2.457967 1.405082 -0.038702
1 -3.994929 1.578334 -0.172231
5hmC/5hmCact.xyz

6 -2.677837 -0.067196 -0.082732
6 -0.574089 0.920801 0.126031
6 0.090788 -0.356715 0.233562
6 -0.715891 -1.451890 0.149594
1 -0.339913 -2.467086 0.208835
7 -2.058629 -1.313986 -0.004022
7 -1.901766 1.042478 -0.019677
8 -3.924993 -0.026186 -0.215630
6 1.571523 -0.485503 0.437974
1 1.902161 0.090409 1.307829
1 1.833309 -1.535449 0.604607
7 0.145274 2.055952 0.189179
1 -0.322185 2.943959 0.065620
1 1.154159 2.036030 0.148206
8 2.333578 0.050511 -0.670838
1 2.113926 -0.460547 -1.465929
1 -2.652904 -2.134191 -0.059034
8 5.048611 -0.263299 -0.082515
1 5.098452 -0.258729 0.883761
1 4.094318 -0.150704 -0.286957
5hmC/5hmCada.xyz
6 -1.347392 -0.707989 -0.016606
6 0.479339 0.724169 0.175703
6 1.419705 -0.370581 0.199909
6 0.879326 -1.615642 0.082137
1 1.476856 -2.520086 0.077191
7 -0.465862 -1.781522 -0.027343
7 -0.847457 0.543360 0.079865
8 -2.580635 -0.950680 -0.107373
6 2.899181 -0.164663 0.367772
1 3.111131 0.387612 1.288802
1 3.398795 -1.137658 0.434223
7 0.924173 1.989899 0.267899
1 0.262532 2.750148 0.183537
1 1.908103 2.197359 0.167814
8 3.481079 0.632183 -0.685061

1 3.335985 0.172684 -1.526448
1 -0.860785 -2.712615 -0.108820
8 -4.421156 1.083469 -0.088696
1 -4.021307 1.882889 -0.458211
1 -3.702548 0.405964 -0.105482
5hmC/5hmCabv.xyz
6 -1.349295 -0.713303 -0.012724
6 0.475122 0.722500 0.175175
6 1.417445 -0.370575 0.200181
6 0.879120 -1.616818 0.085392
1 1.478251 -2.520209 0.081420
7 -0.465897 -1.785289 -0.022389
7 -0.851453 0.539137 0.081485
8 -2.582218 -0.958004 -0.102091
6 2.896781 -0.161536 0.365607
1 3.109061 0.392537 1.285483
1 3.398436 -1.133432 0.432649
7 0.917989 1.989164 0.264645
1 0.255098 2.748139 0.178613
1 1.901324 2.197891 0.161234
8 3.475445 0.634979 -0.689265
1 3.329574 0.174257 -1.529854
1 -0.859232 -2.717198 -0.102208
8 -4.405313 1.092177 -0.143876
1 -3.694432 0.406586 -0.123017
1 -3.940909 1.935515 -0.050443
5hmC/5hmCaay.xyz
6 -2.676571 -0.091070 -0.087228
6 -0.588097 0.927984 0.124551
6 0.094954 -0.339415 0.236984
6 -0.695374 -1.446543 0.153923
1 -0.304750 -2.455931 0.217926
7 -2.039494 -1.328505 -0.003589
7 -1.916949 1.030077 -0.025404
8 -3.923882 -0.068721 -0.222566
6 1.576153 -0.445902 0.449576

1 1.892163 0.130068 1.324786
1 1.853485 -1.492570 0.611803
7 0.115084 2.073264 0.188075
1 -0.364316 2.954329 0.060694
1 1.124246 2.066532 0.147107
8 2.337728 0.108118 -0.650790
1 2.117824 -0.390683 -1.453594
1 -2.621664 -2.157371 -0.058265
8 5.043417 -0.346167 -0.098453
1 5.171230 -0.094149 0.826881
1 4.096802 -0.160247 -0.283274
5hmC/5hmCadc.xyz
6 -2.244806 -0.190013 -0.142853
6 -0.294865 1.091680 -0.126883
6 0.414599 0.131562 0.684519
6 -0.282567 -0.992985 1.009811
1 0.140097 -1.797634 1.600832
7 -1.571404 -1.153387 0.605503
7 -1.570167 0.928035 -0.506372
8 -3.442090 -0.400941 -0.455761
6 1.827928 0.353905 1.154397
1 1.896102 1.277413 1.739157
1 2.123854 -0.477696 1.805424
7 0.326081 2.222706 -0.513091
1 -0.152352 2.851278 -1.144700
1 1.330943 2.299517 -0.433682
8 2.763575 0.511911 0.072938
1 2.706254 -0.294974 -0.484387
1 -2.086318 -1.988482 0.863121
8 2.311214 -1.823579 -1.412269
1 2.778113 -2.579594 -1.025931
1 1.388406 -1.935329 -1.135332
5hmC/5hmCaax.xyz
6 1.239695 -1.152264 -0.057635
6 -1.077010 -0.925842 0.105321
6 -0.993355 0.506772 0.249910

6 0.265813 1.029350 0.196734
1 0.462959 2.092872 0.279332
7 1.351476 0.230309 0.044863
7 0.002317 -1.709710 -0.032337
8 2.290351 -1.832138 -0.176927
6 -2.201634 1.377148 0.451061
1 -2.783802 1.043326 1.315770
1 -1.881086 2.408455 0.637215
7 -2.281013 -1.532577 0.128886
1 -2.328424 -2.525032 -0.059713
1 -3.128088 -0.990513 0.030926
8 -3.124932 1.328901 -0.658590
1 -2.657751 1.634160 -1.451554
1 2.291330 0.645570 0.002100
8 3.938984 1.490149 -0.140030
1 3.851505 2.440586 -0.306278
1 4.427624 1.428138 0.694354
5hmC/5hmCadq.xyz
6 -2.685479 -0.057261 -0.096933
6 -0.575714 0.914752 0.125246
6 0.078584 -0.367626 0.238843
6 -0.735205 -1.456688 0.145944
1 -0.367491 -2.474702 0.208431
7 -2.075636 -1.308651 -0.019273
7 -1.901366 1.046466 -0.028970
8 -3.931804 -0.006488 -0.234680
6 1.554036 -0.506722 0.474058
1 1.864595 0.050331 1.363393
1 1.808077 -1.560880 0.625226
7 0.152673 2.044128 0.191946
1 -0.307141 2.935127 0.061000
1 1.161256 2.013941 0.145639
8 2.345205 0.050417 -0.603188
1 2.142796 -0.440187 -1.415326
1 -2.675576 -2.124428 -0.078536
8 5.047387 -0.234870 0.056105

1 4.107282 -0.141931 -0.213494
1 5.532871 -0.362085 -0.771092
5hmC/5hmCabx.xyz
6 -2.251194 -0.255958 -0.149907
6 -0.359027 1.109574 -0.123100
6 0.398518 0.164700 0.662150
6 -0.247223 -0.993402 0.973678
1 0.214339 -1.789993 1.546320
7 -1.531593 -1.201428 0.577395
7 -1.629889 0.898605 -0.495510
8 -3.439691 -0.515583 -0.460441
6 1.809788 0.433090 1.112014
1 1.859364 1.361017 1.691428
1 2.141587 -0.385464 1.762294
7 0.208023 2.274615 -0.491008
1 -0.304290 2.895530 -1.103444
1 1.208061 2.399567 -0.414737
8 2.721259 0.611129 0.013265
1 2.707655 -0.221357 -0.508085
1 -2.007098 -2.063519 0.821393
8 2.429312 -1.854415 -1.284676
1 1.827698 -2.282796 -0.656318
1 3.234683 -2.392596 -1.269191
5hmC/5hmCaap.xyz
6 2.679080 -0.062719 0.083492
6 0.572510 0.919157 -0.125913
6 -0.088607 -0.360265 -0.233860
6 0.721176 -1.453075 -0.149104
1 0.348176 -2.469363 -0.208360
7 2.063442 -1.311301 0.005285
7 1.899770 1.044653 0.020156
8 3.926114 -0.017991 0.216450
6 -1.568654 -0.493342 -0.440814
1 -1.898957 0.080733 -1.312021
1 -1.827206 -1.544192 -0.606812
7 -0.150113 2.052265 -0.189065

1 0.314852 2.941491 -0.064779
1 -1.158903 2.029351 -0.147155
8 -2.334356 0.041999 0.665713
1 -2.115943 -0.468212 1.461670
1 2.660034 -2.129807 0.060575
8 -5.053843 -0.253805 0.087257
1 -5.113671 -0.247354 -0.878424
1 -4.096433 -0.152134 0.282512
5hmC/5hmCace.xyz
6 -1.342546 0.688921 0.011290
6 0.494567 -0.730381 -0.174570
6 1.427119 0.371049 -0.200033
6 0.877711 1.612468 -0.085664
1 1.468710 2.521194 -0.081752
7 -0.468813 1.768847 0.021521
7 -0.833639 -0.558892 -0.082062
8 -2.577781 0.922521 0.099411
6 2.908259 0.175462 -0.365296
1 3.125514 -0.378149 -1.284270
1 3.400771 1.151921 -0.434245
7 0.948582 -1.993243 -0.262077
1 0.292027 -2.757577 -0.174200
1 1.933621 -2.193160 -0.157580
8 3.494749 -0.613652 0.690844
1 3.344604 -0.152908 1.530661
1 -0.870423 2.697240 0.101039
8 -4.355840 -1.175791 0.126406
1 -3.687577 -0.448620 0.114744
1 -5.209841 -0.726658 0.196275
5hmC/5hmCacj.xyz
6 -2.239722 -0.183619 -0.140453
6 -0.286990 1.093817 -0.136823
6 0.417876 0.143741 0.690154
6 -0.282109 -0.975519 1.027660
1 0.137648 -1.773418 1.629816
7 -1.569940 -1.139056 0.621270

7 -1.561847 0.928114 -0.516987
8 -3.436811 -0.395884 -0.453201
6 1.831650 0.367106 1.157691
1 1.907557 1.306816 1.714926
1 2.117617 -0.447628 1.833964
7 0.337556 2.216925 -0.539301
1 -0.139223 2.839666 -1.177845
1 1.341298 2.298248 -0.452852
8 2.770908 0.481993 0.073985
1 2.705450 -0.343086 -0.455088
1 -2.086952 -1.970151 0.887578
8 2.309176 -1.934835 -1.265199
1 1.346842 -1.939576 -1.144805
1 2.438964 -1.896097 -2.224613
5hmC/5hmCacg.xyz
6 2.671341 -0.084531 0.074514
6 0.578117 0.926905 -0.127209
6 -0.102168 -0.343316 -0.224271
6 0.692536 -1.447453 -0.142983
1 0.304519 -2.458437 -0.196521
7 2.037715 -1.324412 0.000980
7 1.907901 1.033854 0.011272
8 3.919403 -0.057655 0.202109
6 -1.586321 -0.455784 -0.411808
1 -1.920997 0.124300 -1.277262
1 -1.861225 -1.502715 -0.576268
7 -0.128097 2.069988 -0.193040
1 0.350227 2.953504 -0.079487
1 -1.137147 2.062798 -0.152930
8 -2.330092 0.086441 0.706712
1 -2.105029 -0.427033 1.498757
1 2.622882 -2.151171 0.055334
8 -5.019236 -0.271371 0.037545
1 -4.975471 -0.549189 -0.888426
1 -4.082016 -0.142317 0.301931
5hmC/5hmCacf.xyz

6 2.672210 -0.091165 0.080436
6 0.583720 0.928138 -0.130346
6 -0.101138 -0.339561 -0.228140
6 0.688861 -1.446684 -0.141846
1 0.297110 -2.456212 -0.195766
7 2.033943 -1.328668 0.007177
7 1.913394 1.030085 0.012855
8 3.919988 -0.068933 0.211620
6 -1.584535 -0.446274 -0.424571
1 -1.910843 0.129892 -1.295775
1 -1.863260 -1.493006 -0.584117
7 -0.117852 2.073801 -0.200749
1 0.363506 2.955556 -0.086253
1 -1.127059 2.070212 -0.162872
8 -2.333154 0.106877 0.685290
1 -2.111916 -0.399102 1.483158
1 2.615654 -2.157617 0.065063
8 -5.027971 -0.244562 0.036183
1 -5.007885 -0.929855 -0.646874
1 -4.087311 -0.120180 0.290514
5hmC/5hmCabk.xyz
6 2.679822 -0.082980 0.088594
6 0.584881 0.921912 -0.125543
6 -0.090535 -0.350257 -0.230363
6 0.706380 -1.452040 -0.140736
1 0.322284 -2.464261 -0.198945
7 2.049976 -1.324688 0.015248
7 1.913228 1.033151 0.021584
8 3.927329 -0.052138 0.220895
6 -1.569726 -0.465779 -0.453434
1 -1.877433 0.086836 -1.346472
1 -1.843470 -1.516935 -0.590772
7 -0.126323 2.062125 -0.194517
1 0.347317 2.946464 -0.067897
1 -1.134903 2.046628 -0.139492
8 -2.343773 0.116841 0.622884

1 -2.131857 -0.356863 1.442604
1 2.637307 -2.149633 0.073586
8 -5.050513 -0.207264 0.012716
1 -5.204469 -1.162381 -0.001162
1 -4.102227 -0.108617 0.249273
5hmC/5hmCads.xyz
6 -1.230726 1.186904 -0.063686
6 1.081191 0.921921 0.111468
6 0.973857 -0.510570 0.240447
6 -0.292930 -1.012298 0.174656
1 -0.507761 -2.073247 0.245677
7 -1.364961 -0.194625 0.024665
7 0.015515 1.724305 -0.025688
8 -2.269480 1.884745 -0.182498
6 2.166375 -1.402117 0.442779
1 2.741885 -1.092063 1.320660
1 1.827367 -2.431224 0.607061
7 2.295338 1.507811 0.149465
1 2.361255 2.500599 -0.031847
1 3.132375 0.951274 0.045074
8 3.105053 -1.349251 -0.653527
1 2.644248 -1.634796 -1.457505
1 -2.309700 -0.598801 -0.018833
8 -3.927872 -1.510241 -0.064878
1 -3.917388 -2.199754 -0.745789
1 -4.041739 -1.989492 0.769642
5hmC/5hmCaao.xyz
6 1.244224 1.137202 0.058407
6 -1.073886 0.927730 -0.107139
6 -1.000650 -0.505617 -0.249859
6 0.254705 -1.037371 -0.194910
1 0.443937 -2.102429 -0.276127
7 1.345993 -0.246176 -0.042930
7 0.011169 1.703645 0.030788
8 2.299826 1.809322 0.178739
6 -2.215049 -1.367686 -0.449802

1 -2.797693 -1.027747 -1.311794
1 -1.901750 -2.400526 -0.639836
7 -2.272780 1.544191 -0.132691
1 -2.312039 2.537604 0.052694
1 -3.125296 1.010078 -0.039123
8 -3.134633 -1.316868 0.662866
1 -2.667047 -1.627792 1.453378
1 2.283603 -0.666473 0.001437
8 3.940387 -1.492197 0.152757
1 4.505578 -1.207349 -0.581102
1 3.879335 -2.454591 0.059218
5hmC/5hmCaae.xyz
6 -1.408946 1.127324 0.061872
6 0.188195 -0.577826 -0.147768
6 1.280367 0.363223 -0.206928
6 0.934732 1.676992 -0.095649
1 1.663119 2.479861 -0.120776
7 -0.365888 2.049373 0.039266
7 -1.091724 -0.189733 -0.022707
8 -2.586484 1.539862 0.164711
6 2.706813 -0.064115 -0.414259
1 2.800032 -0.664548 -1.324684
1 3.340103 0.822895 -0.527004
7 0.424605 -1.896796 -0.237497
1 -0.347308 -2.546575 -0.160779
1 1.366818 -2.257014 -0.179711
8 3.203197 -0.910723 0.642887
1 3.151191 -0.415889 1.475153
1 -0.611161 3.031418 0.105530
8 -3.122443 -2.137311 0.128534
1 -2.447747 -1.415162 0.059486
1 -3.845128 -1.863114 -0.453319
5hmU/5hmUabr.xyz
6 -1.281847 1.131674 0.054765
6 1.180313 0.926495 -0.134084
6 1.020963 -0.508394 -0.254783

6 -0.237376 -1.020594 -0.200688
1 -0.435699 -2.084412 -0.273884
7 -1.347567 -0.233938 -0.055891
7 -0.002985 1.649664 0.005018
8 -2.282612 1.848875 0.188493
8 2.273275 1.521902 -0.141949
1 0.075918 2.659609 0.092526
6 2.250666 -1.355090 -0.415827
1 1.968095 -2.396279 -0.583356
1 2.833193 -1.010727 -1.278973
8 3.085225 -1.345643 0.761579
1 3.428045 -0.444194 0.866983
1 -2.280699 -0.675261 -0.017464
8 -3.809887 -1.636915 -0.001173
1 -4.272161 -1.529985 0.843878
1 -3.587145 -2.579115 -0.045496
5hmU/5hmUacj.xyz
6 1.285411 1.115399 -0.050039
6 -1.178837 0.928628 0.128731
6 -1.030048 -0.506676 0.257866
6 0.224826 -1.028114 0.212106
1 0.414634 -2.092953 0.291996
7 1.341146 -0.250032 0.068178
7 0.010101 1.642694 -0.009451
8 2.292054 1.824580 -0.182865
8 -2.267611 1.531679 0.129161
1 -0.061319 2.652710 -0.102555
6 -2.266081 -1.344109 0.419105
1 -1.991210 -2.386313 0.593102
1 -2.848935 -0.991228 1.278583
8 -3.096828 -1.335253 -0.760998
1 -3.430817 -0.431410 -0.874029
1 2.272188 -0.695954 0.033185
8 3.817064 -1.637331 0.003015
1 4.383444 -1.304743 -0.709648
1 3.634220 -2.558916 -0.234838

5hmU/5hmUaay.xyz
6 1.281849 1.122029 -0.074433
6 -1.178387 0.926813 0.147254
6 -1.024404 -0.510031 0.250020
6 0.230285 -1.027956 0.171088
1 0.423744 -2.093535 0.229489
7 1.342178 -0.245262 0.018016
7 0.006614 1.645773 0.000811
8 2.284451 1.835818 -0.212614
8 -2.268101 1.527408 0.175881
1 -0.068328 2.657177 -0.072011
6 -2.255842 -1.352767 0.418052
1 -1.975750 -2.395945 0.577204
1 -2.828940 -1.010778 1.288383
8 -3.101036 -1.333841 -0.751630
1 -3.442267 -0.430885 -0.848409
1 2.273271 -0.689159 -0.034582
8 3.812091 -1.635913 -0.132337
1 3.630802 -2.572661 0.037868
1 4.405685 -1.364091 0.583983
5hmU/5hmUaas.xyz
6 1.717271 -1.137872 -0.076254
6 -0.158073 0.451223 0.190449
6 -1.076536 -0.667170 0.211468
6 -0.564381 -1.918754 0.081025
1 -1.185137 -2.807388 0.082013
7 0.780777 -2.144461 -0.053597
7 1.186339 0.129641 0.047248
8 2.929132 -1.344656 -0.198639
8 -0.505677 1.645638 0.284571
1 1.853203 0.898148 0.027856
6 -2.544409 -0.389372 0.378509
1 -3.096525 -1.329658 0.433038
1 -2.715330 0.166604 1.308510
8 -3.096641 0.345467 -0.731142
1 -2.700745 1.231083 -0.718688

1 1.131896 -3.092902 -0.139536
8 1.474189 3.629152 0.037989
1 0.754915 2.965186 0.102280
1 1.536662 3.839535 -0.904433
5hmU/5hmUabo.xyz
6 2.677421 -0.133209 -0.094752
6 0.472193 0.962320 0.167791
6 -0.142458 -0.349941 0.233318
6 0.656296 -1.442661 0.122487
1 0.272032 -2.455750 0.152606
7 2.014875 -1.336914 -0.028314
7 1.856518 0.969706 0.007212
8 3.902355 -0.045258 -0.234569
8 -0.148866 2.036789 0.236853
1 2.314408 1.876300 -0.046331
6 -1.629544 -0.433730 0.417517
1 -1.941778 -1.476541 0.493704
1 -1.929748 0.088945 1.333136
8 -2.352381 0.122591 -0.705209
1 -2.177160 1.077689 -0.721801
1 2.584358 -2.173113 -0.107210
8 -5.059961 -0.237501 -0.087781
1 -5.037174 -0.901182 0.616226
1 -4.117331 -0.095537 -0.325128
5hmU/5hmUabv.xyz
6 -2.219109 0.155844 -0.098590
6 -0.106664 -1.135220 -0.141898
6 0.490721 -0.135620 0.722302
6 -0.264582 0.929087 1.095941
1 0.116401 1.725051 1.725462
7 -1.571727 1.065012 0.705470
7 -1.437271 -0.910270 -0.489555
8 -3.399163 0.283015 -0.443247
8 0.486850 -2.136996 -0.577082
1 -1.882667 -1.594251 -1.096161
6 1.921587 -0.314652 1.141833

1 2.221836 0.497660 1.805614
1 2.047443 -1.265089 1.672255
8 2.829841 -0.278330 0.016599
1 2.653131 -1.064984 -0.525260
1 -2.114382 1.865429 1.012756
8 2.036913 1.949587 -1.516308
1 2.343600 1.167328 -1.008617
1 1.110374 2.050834 -1.254680
5hmU/5hmUado.xyz
6 1.343613 0.568755 -0.041832
6 -1.096225 0.923452 0.147125
6 -1.271355 -0.514503 0.226311
6 -0.167943 -1.302157 0.160749
1 -0.216827 -2.383940 0.207165
7 1.094585 -0.777982 0.038934
7 0.221816 1.361381 0.014903
8 2.485477 1.040218 -0.157452
8 -2.021309 1.751741 0.181996
1 0.377182 2.364730 -0.045397
6 -2.663315 -1.060610 0.379376
1 -2.626995 -2.148153 0.468528
1 -3.126993 -0.655167 1.287112
8 -3.497851 -0.770506 -0.758985
1 -3.631817 0.190192 -0.782714
1 1.900204 -1.394925 -0.005139
8 4.605945 -0.836017 -0.181758
1 4.761166 -1.074685 0.743029
1 3.902540 -0.154954 -0.150227
5hmU/5hmUacz.xyz
6 -1.294138 1.078550 0.055647
6 1.172150 0.932317 -0.128617
6 1.046276 -0.505101 -0.258961
6 -0.199736 -1.047280 -0.210191
1 -0.371864 -2.115006 -0.290672
7 -1.328226 -0.287632 -0.062850
7 -0.027902 1.626822 0.012787

8 -2.312166 1.771089 0.190454
8 2.250953 1.553015 -0.130929
1 0.027220 2.637926 0.105368
6 2.294996 -1.322551 -0.424637
1 2.036095 -2.368236 -0.602258
1 2.871083 -0.957340 -1.283513
8 3.127100 -1.305488 0.754502
1 3.443471 -0.396087 0.873311
1 -2.254034 -0.745052 -0.025933
8 -3.798243 -1.677153 0.022104
1 -4.514059 -1.087401 -0.259398
1 -4.003456 -1.890455 0.945036
5hmU/5hmUacu.xyz
6 -1.343522 0.569145 0.042767
6 1.096318 0.923617 -0.146683
6 1.271224 -0.514302 -0.226850
6 0.167709 -1.301835 -0.161424
1 0.216418 -2.383592 -0.208618
7 -1.094682 -0.777576 -0.038754
7 -0.221634 1.361655 -0.013853
8 -2.485312 1.040676 0.159042
8 2.021505 1.751810 -0.181281
1 -0.376822 2.364989 0.047130
6 2.663034 -1.060557 -0.380697
1 2.626454 -2.148010 -0.470852
1 3.126628 -0.654399 -1.288155
8 3.497880 -0.771697 0.757766
1 3.632270 0.188919 0.782255
1 -1.900475 -1.394390 0.004404
8 -4.596744 -0.845841 0.181257
1 -3.901232 -0.156850 0.148419
1 -4.836247 -1.001220 -0.743281
5hmU/5hmUaau.xyz
6 1.359496 1.222893 0.068225
6 -0.243852 -0.632912 -0.187904
6 -1.325671 0.334069 -0.218438

6 -1.013780 1.650107 -0.098304
1 -1.763209 2.433606 -0.106585
7 0.281447 2.078564 0.035168
7 1.037309 -0.108987 -0.047445
8 2.524677 1.623574 0.193751
8 -0.403206 -1.865411 -0.273814
1 1.822828 -0.779511 -0.021249
6 -2.732899 -0.166694 -0.376494
1 -3.421793 0.676369 -0.459469
1 -2.814860 -0.771917 -1.287655
8 -3.172617 -0.941979 0.757498
1 -2.632934 -1.747919 0.783763
1 0.485230 3.069018 0.116718
8 3.126673 -2.080987 -0.032220
1 2.625993 -2.911057 -0.035129
1 3.603463 -2.081978 0.811314
5hmU/5hmUaai.xyz
6 -2.382612 -0.308689 -0.173172
6 0.002572 -0.676354 0.391828
6 0.216421 0.756048 0.333408
6 -0.851555 1.545003 0.034801
1 -0.775114 2.625019 -0.020553
7 -2.097222 1.036039 -0.214868
7 -1.298449 -1.101827 0.135775
8 -3.508808 -0.767473 -0.390973
8 0.868446 -1.533074 0.658504
1 -1.474625 -2.102860 0.175687
6 1.581756 1.341430 0.584291
1 1.472644 2.385615 0.886761
1 2.073844 0.798770 1.399116
8 2.421618 1.338184 -0.584047
1 2.815557 0.442978 -0.668925
1 -2.870403 1.658198 -0.428532
8 3.378632 -1.295140 -0.487254
1 3.328599 -1.749904 -1.340409
1 2.520601 -1.481900 -0.046268

5hmU/5hmUaaf.xyz
6 2.237601 -0.306879 -0.115733
6 0.260915 1.184929 -0.150967
6 -0.442845 0.227665 0.680394
6 0.199445 -0.915349 1.033737
1 -0.266932 -1.686116 1.636271
7 1.491476 -1.170059 0.653352
7 1.571560 0.843545 -0.481358
8 3.403117 -0.541736 -0.453547
8 -0.227436 2.247023 -0.572834
1 2.093111 1.498757 -1.058594
6 -1.859653 0.526300 1.076160
1 -2.221474 -0.228930 1.775780
1 -1.924634 1.508749 1.555957
8 -2.761660 0.490924 -0.055082
1 -2.523673 1.222546 -0.647423
1 1.946939 -2.029742 0.942352
8 -2.319421 -2.044797 -1.189522
1 -3.164072 -2.366135 -1.534945
1 -2.510091 -1.144845 -0.847031
5hmU/5hmUaca.xyz
6 -2.396351 -0.310099 -0.163313
6 -0.001220 -0.688711 0.350855
6 0.209664 0.745258 0.326865
6 -0.864240 1.539979 0.067366
1 -0.789843 2.621169 0.038641
7 -2.113717 1.035799 -0.172866
7 -1.306349 -1.109194 0.107300
8 -3.525268 -0.765066 -0.374969
8 0.869848 -1.551303 0.578235
1 -1.480087 -2.111332 0.123238
6 1.578024 1.326437 0.570328
1 1.472384 2.365123 0.892519
1 2.080160 0.769705 1.369452
8 2.403683 1.342184 -0.607947
1 2.812722 0.454678 -0.700304

1 -2.890667 1.662197 -0.358793
8 3.373568 -1.291166 -0.573713
1 4.068855 -1.331104 0.099223
1 2.537015 -1.471051 -0.090465
5hmU/5hmUaan.xyz
6 -2.166714 0.072110 -0.047585
6 0.004621 -1.118648 -0.126830
6 0.550232 -0.125178 0.778409
6 -0.257651 0.886402 1.189224
1 0.085516 1.678957 1.844263
7 -1.570258 0.971805 0.804907
7 -1.335778 -0.943289 -0.470199
8 -3.347049 0.164260 -0.402530
8 0.641998 -2.075368 -0.597877
1 -1.745706 -1.621044 -1.107846
6 2.001227 -0.207518 1.153694
1 2.236139 0.552589 1.900811
1 2.239706 -1.192768 1.567464
8 2.866901 0.053378 0.021977
1 2.862516 -0.736846 -0.541040
1 -2.149329 1.736456 1.135302
8 1.515985 1.800054 -1.777862
1 2.011203 1.238309 -1.145201
1 0.589233 1.563132 -1.627846
5hmU/5hmUact.xyz
6 2.424872 -0.416656 0.081527
6 -0.034837 -0.653223 -0.092315
6 -0.139472 0.783149 -0.253957
6 1.007676 1.513269 -0.231046
1 1.013511 2.591327 -0.344658
7 2.239477 0.937792 -0.070395
7 1.257423 -1.149011 0.060850
8 3.538117 -0.933207 0.224761
8 -0.989350 -1.452686 -0.084301
1 1.357708 -2.154433 0.177245
6 -1.491333 1.419586 -0.414385

1 -1.374252 2.441633 -0.780014
1 -2.088466 0.857810 -1.140781
8 -2.211484 1.522367 0.830687
1 -2.509531 0.634570 1.078222
1 3.074351 1.514917 -0.063498
8 -3.794067 -1.368358 -0.208512
1 -4.082696 -1.108475 0.677766
1 -2.816091 -1.320490 -0.177496
5hmU/5hmUaaw.xyz
6 -2.686780 -0.112233 0.115051
6 -0.469398 0.954301 -0.164060
6 0.124798 -0.365664 -0.256170
6 -0.687976 -1.447929 -0.145305
1 -0.319146 -2.466035 -0.194018
7 -2.042528 -1.324485 0.027420
7 -1.850941 0.979913 0.017288
8 -3.908653 -0.008322 0.270012
8 0.166779 2.020189 -0.228381
1 -2.294776 1.892272 0.087062
6 1.606387 -0.468403 -0.472977
1 1.901968 -1.514313 -0.569581
1 1.893747 0.061695 -1.388471
8 2.361740 0.063061 0.640161
1 2.193739 1.018869 0.678546
1 -2.623256 -2.153184 0.103294
8 5.072771 -0.207335 0.003050
1 5.508859 -0.609189 0.767429
1 4.129864 -0.119290 0.264809
5hmU/5hmUabw.xyz
6 1.278308 1.136666 -0.072653
6 -1.180904 0.923622 0.146020
6 -1.016701 -0.511992 0.249474
6 0.241936 -1.020620 0.173491
1 0.443109 -2.084694 0.233203
7 1.348670 -0.230227 0.021653
7 -0.001023 1.650909 0.000362

8 2.275085 1.858351 -0.210864
8 -2.274982 1.516381 0.173278
1 -0.083141 2.661668 -0.073612
6 -2.242479 -1.363299 0.415746
1 -1.955453 -2.404981 0.572252
1 -2.817813 -1.027371 1.286983
8 -3.087915 -1.346844 -0.753776
1 -3.435862 -0.446086 -0.847284
1 2.282373 -0.669377 -0.026737
8 3.828175 -1.601298 -0.059491
1 3.803493 -2.290411 -0.740719
1 3.925902 -2.082513 0.776163
5hmU/5hmUadu.xyz
6 1.337571 -0.805385 0.020390
6 -0.549312 0.775261 0.264882
6 -1.463576 -0.349101 0.193022
6 -0.943411 -1.593385 0.037933
1 -1.560092 -2.482175 -0.029409
7 0.409084 -1.812858 -0.039777
7 0.805488 0.454047 0.170954
8 2.557977 -1.015475 -0.057153
8 -0.893633 1.961459 0.394059
1 1.470866 1.222660 0.217283
6 -2.939365 -0.080338 0.283446
1 -3.489098 -1.023590 0.289113
1 -3.166136 0.458007 1.211767
8 -3.431882 0.671870 -0.843080
1 -3.041013 1.558398 -0.795539
1 0.766958 -2.756116 -0.151068
8 4.252282 1.245943 -0.149206
1 3.691987 0.442429 -0.133289
1 4.295123 1.499381 -1.082094
5hmU/5hmUabk.xyz
6 1.673230 -1.189546 -0.073016
6 -0.149305 0.462246 0.185186
6 -1.103967 -0.625960 0.210393

6 -0.633083 -1.894335 0.086539
1 -1.282458 -2.762368 0.091566
7 0.704301 -2.164872 -0.044516
7 1.183885 0.095477 0.041511
8 2.877740 -1.436466 -0.193453
8 -0.457552 1.667276 0.275950
1 1.876368 0.840491 0.017332
6 -2.561586 -0.298639 0.377348
1 -3.144718 -1.219701 0.435932
1 -2.712374 0.266133 1.305488
8 -3.090050 0.450311 -0.734434
1 -2.661721 1.320796 -0.727537
1 1.023410 -3.125220 -0.123000
8 1.579740 3.608156 0.000094
1 0.847868 2.964717 0.107399
1 2.205559 3.164112 -0.590091
5hmU/5hmUadv.xyz
6 2.681925 0.132175 0.108618
6 0.477583 -0.962437 -0.165537
6 -0.134052 0.350028 -0.250726
6 0.665134 1.442495 -0.140776
1 0.282797 2.455815 -0.185194
7 2.021832 1.336202 0.025979
7 1.860172 -0.970452 0.009670
8 3.905466 0.043706 0.259823
8 -0.144732 -2.036403 -0.230845
1 2.316128 -1.877178 0.075482
6 -1.618208 0.434482 -0.457075
1 -1.927335 1.476928 -0.549420
1 -1.906065 -0.097239 -1.371412
8 -2.358822 -0.107945 0.660634
1 -2.184171 -1.062775 0.692316
1 2.591899 2.172187 0.102922
8 -5.078820 0.194353 0.065778
1 -5.237423 1.148697 0.081090
1 -4.128876 0.093172 0.294527

5mC/5mCaat.xyz
6 -0.938719 -0.730915 -0.008667
6 0.781755 0.838872 -0.004622
6 1.809819 -0.173631 0.004003
6 1.356807 -1.457684 0.006068
1 2.017764 -2.316606 0.012428
7 0.019722 -1.730930 -0.000027
7 -0.531938 0.556602 -0.010638
8 -2.156142 -1.067827 -0.014667
7 1.126372 2.138935 -0.007245
1 0.404783 2.846884 -0.012576
1 2.091165 2.435079 -0.003364
1 -0.301930 -2.692719 0.001946
6 3.272881 0.170214 0.010144
1 3.551477 0.753152 -0.876345
1 3.541521 0.764118 0.892408
1 3.878973 -0.739272 0.019155
8 -4.110034 0.864044 0.021924
1 -3.683494 1.731370 -0.015235
1 -3.365201 0.214857 0.007361
5mC/5mCacl.xyz
6 0.867331 1.178304 -0.000001
6 -0.425492 -0.778161 -0.000005
6 -1.667982 -0.046724 -0.000001
6 -1.542071 1.309399 0.000002
1 -2.395120 1.978083 0.000005
7 -0.314034 1.905511 -0.000006
7 0.775246 -0.175441 -0.000010
8 1.966606 1.784556 0.000004
7 -0.434298 -2.121857 0.000002
1 0.440733 -2.629214 -0.000012
1 -1.297361 -2.645564 -0.000025
1 -0.239886 2.917074 0.000014
6 -3.000264 -0.742741 0.000006
1 -3.118368 -1.380866 -0.884254
1 -3.118367 -1.380846 0.884282

1 -3.813141 -0.012214 -0.000003
8 3.244846 -1.508134 0.000008
1 3.901510 -0.797248 -0.000016
1 2.370853 -1.038536 -0.000002
5mC/5mCabg.xyz
6 0.930126 -0.574496 -0.032498
6 -1.393935 -0.714475 0.010019
6 -1.559136 0.719075 0.001127
6 -0.400810 1.433712 -0.029053
1 -0.374124 2.517160 -0.039982
7 0.811157 0.805919 -0.046859
7 -0.195275 -1.320550 -0.006930
8 2.084880 -1.088527 -0.045221
7 -2.479137 -1.508570 0.034892
1 -2.363586 -2.512934 0.043260
1 -3.414478 -1.130046 0.053822
1 1.664567 1.354540 -0.066439
6 -2.914828 1.367484 0.021256
1 -3.511192 1.076291 -0.852187
1 -3.481867 1.082126 0.915895
1 -2.818696 2.456217 0.016382
8 4.324949 0.510515 -0.034593
1 4.565824 0.628416 0.894913
1 3.529192 -0.073058 -0.019976
5mC/5mCaaw.xyz
6 0.952083 -0.651576 -0.232986
6 -0.836842 0.835164 -0.122336
6 -1.798179 -0.217188 0.102763
6 -1.279962 -1.475774 0.139322
1 -1.887234 -2.359048 0.299092
7 0.057467 -1.688567 -0.025427
7 0.479695 0.612659 -0.279657
8 2.175306 -0.936286 -0.372094
7 -1.252015 2.113241 -0.181952
1 -0.580813 2.853272 -0.335212
1 -2.224039 2.361781 -0.072694

1 0.430727 -2.631042 0.005837
6 -3.263846 0.061095 0.287529
1 -3.441228 0.705290 1.157390
1 -3.689668 0.567291 -0.587621
1 -3.813873 -0.871100 0.438799
8 4.098804 0.801303 0.525119
1 3.374938 0.233834 0.166457
1 4.002751 1.647921 0.067242
5mC/5mCadq.xyz
6 -0.929641 0.563637 -0.029402
6 1.393593 0.716820 0.007877
6 1.566866 -0.715778 0.002949
6 0.412524 -1.437133 -0.020207
1 0.391972 -2.520748 -0.026764
7 -0.803068 -0.816147 -0.036603
7 0.191604 1.316121 -0.008731
8 -2.087140 1.071235 -0.043504
7 2.474321 1.517129 0.028556
1 2.352755 2.520801 0.034809
1 3.411881 1.144159 0.046992
1 -1.653599 -1.369364 -0.050680
6 2.926566 -1.355798 0.020241
1 3.518446 -1.062168 -0.855419
1 3.494381 -1.065559 0.912804
1 2.837352 -2.445110 0.017143
8 -4.328280 -0.523822 -0.037677
1 -3.531741 0.058683 -0.019849
1 -4.757535 -0.390204 0.819094
5mC/5mCabr.xyz
6 0.931176 -0.591158 -0.020420
6 -1.394541 -0.712046 0.008545
6 -1.548167 0.722879 -0.002853
6 -0.384015 1.428008 -0.026417
1 -0.348430 2.511184 -0.038150
7 0.822839 0.790510 -0.035726
7 -0.200737 -1.327788 -0.000704

8 2.081549 -1.114301 -0.026371
7 -2.486505 -1.497022 0.028072
1 -2.380024 -2.502370 0.037130
1 -3.418665 -1.110338 0.039869
1 1.680015 1.333090 -0.052300
6 -2.898218 1.383167 0.008859
1 -3.492918 1.095039 -0.866739
1 -3.472264 1.105232 0.901386
1 -2.792230 2.470998 0.001636
8 4.342412 0.470022 -0.012870
1 4.103646 1.213615 0.558504
1 3.542588 -0.107229 -0.015182
5mC/5mCabn.xyz
6 0.940874 -0.715020 -0.059690
6 -0.792906 0.839513 -0.031190
6 -1.810760 -0.181581 0.027046
6 -1.346350 -1.461431 0.038040
1 -1.999062 -2.325592 0.080086
7 -0.007674 -1.722944 -0.004440
7 0.522807 0.568909 -0.072767
8 2.160307 -1.042519 -0.098252
7 -1.151155 2.135880 -0.047170
1 -0.438531 2.851698 -0.086559
1 -2.119064 2.421014 -0.024969
1 0.323187 -2.681582 0.007519
6 -3.275920 0.149804 0.072445
1 -3.521203 0.763561 0.947851
1 -3.588242 0.708265 -0.818489
1 -3.872973 -0.764209 0.123659
8 4.118893 0.851101 0.158480
1 3.372470 0.209934 0.061628
1 3.772349 1.687639 -0.181824
5mC/5mCabm.xyz
6 -0.780367 -1.299321 -0.023159
6 0.332612 0.764769 -0.031432
6 1.635829 0.148304 0.011415

6 1.631167 -1.213581 0.034455
1 2.539435 -1.804224 0.065491
7 0.461975 -1.916514 0.017596
7 -0.810326 0.057189 -0.049465
8 -1.818069 -2.005078 -0.033836
7 0.222922 2.104203 -0.055537
1 -0.692025 2.534182 -0.085997
1 1.036074 2.701924 -0.038962
1 0.479054 -2.930139 0.033747
6 2.901736 0.958679 0.028505
1 2.980109 1.597461 -0.859785
1 2.947040 1.612425 0.908228
1 3.775093 0.302007 0.050290
8 -3.306320 1.367742 0.072622
1 -3.087773 2.310672 0.079369
1 -2.429745 0.907133 0.030470
5mC/5mCaaj.xyz
6 0.710348 -1.191422 -0.004997
6 -1.518689 -0.501432 0.002346
6 -1.166981 0.896791 -0.002158
6 0.171942 1.152066 -0.007707
1 0.576885 2.157998 -0.011104
7 1.086951 0.143066 -0.009434
7 -0.612256 -1.491388 0.000787
8 1.608452 -2.075778 -0.006464
7 -2.816980 -0.863024 0.009483
1 -3.060926 -1.843816 0.009472
1 -3.559937 -0.180458 0.007396
1 2.089153 0.369184 -0.011334
6 -2.203232 1.985816 0.001223
1 -2.862674 1.916225 -0.872825
1 -2.838437 1.933291 0.894291
1 -1.725432 2.968816 -0.015037
8 3.865617 0.957726 -0.004142
1 3.986567 1.635469 -0.686137
1 4.037911 1.416218 0.832029

5mC/5mCacf.xyz
6 -0.937466 -0.670396 -0.077421
6 0.832302 0.842580 -0.039114
6 1.824867 -0.201969 0.036814
6 1.330280 -1.470328 0.052056
1 1.962167 -2.349051 0.107390
7 -0.013595 -1.700076 -0.003813
7 -0.489213 0.603447 -0.092713
8 -2.163256 -0.970459 -0.131361
7 1.223718 2.129326 -0.059612
1 0.531589 2.863477 -0.119389
1 2.199125 2.388055 -0.038850
1 -0.367746 -2.650397 0.011041
6 3.296817 0.095633 0.093299
1 3.628448 0.643288 -0.797421
1 3.549197 0.707577 0.967980
1 3.872371 -0.831566 0.153076
8 -4.109681 0.937456 0.148253
1 -4.926671 0.426050 0.229922
1 -3.392153 0.264591 0.050285
5mC/5mCaao.xyz
6 0.777874 1.301688 0.025535
6 -0.330815 -0.764511 0.034365
6 -1.635207 -0.150649 -0.012461
6 -1.633266 1.211158 -0.038419
1 -2.542623 1.799927 -0.072810
7 -0.465491 1.916409 -0.020394
7 0.810681 -0.054732 0.054684
8 1.814158 2.009563 0.037874
7 -0.218999 -2.103696 0.060548
1 0.696600 -2.532083 0.089865
1 -1.031034 -2.702775 0.039225
1 -0.484530 2.929959 -0.038788
6 -2.899559 -0.963435 -0.030184
1 -2.942339 -1.618949 -0.908724
1 -2.978197 -1.600659 0.859194

1 -3.774057 -0.308368 -0.054578
8 3.308612 -1.369088 -0.081718
1 2.441236 -0.893820 -0.021421
1 3.075298 -2.308417 -0.078098
5mC/5mCaba.xyz
6 -0.740808 1.142958 -0.011671
6 1.509388 0.527358 0.006345
6 1.203769 -0.881533 -0.003067
6 -0.125889 -1.181234 -0.019164
1 -0.496739 -2.200177 -0.028227
7 -1.073312 -0.203048 -0.025241
7 0.570880 1.486986 0.003446
8 -1.668793 1.996546 -0.013808
7 2.795280 0.931044 0.021218
1 3.007403 1.919304 0.021931
1 3.559978 0.272839 0.017212
1 -2.068966 -0.456682 -0.035074
6 2.276678 -1.934294 0.005696
1 2.936732 -1.842825 -0.865849
1 2.905793 -1.858168 0.901345
1 1.834098 -2.933625 -0.011153
8 -3.858889 -0.995011 -0.070395
1 -4.312330 -0.524882 0.645424
1 -3.933271 -1.932466 0.163224
5mC/5mCacw.xyz
6 -0.937197 0.667612 -0.076382
6 0.834569 -0.843024 -0.041340
6 1.825797 0.202717 0.035818
6 1.329553 1.470395 0.053118
1 1.960287 2.349864 0.109681
7 -0.014631 1.698406 -0.002161
7 -0.487339 -0.605628 -0.093513
8 -2.163369 0.966369 -0.128876
7 1.227770 -2.129186 -0.064382
1 0.536676 -2.864201 -0.125455
1 2.203584 -2.386516 -0.045169

1 -0.370121 2.648200 0.014584
6 3.298159 -0.093034 0.091385
1 3.551596 -0.705990 0.965053
1 3.630169 -0.638952 -0.800265
1 3.872523 0.834822 0.152391
8 -4.120579 -0.920183 0.204098
1 -4.903807 -0.517325 -0.195833
1 -3.395224 -0.262519 0.068032
5mC/5mCaan.xyz
6 0.930645 -0.580861 -0.005687
6 -1.394440 -0.714165 0.003264
6 -1.555638 0.719975 -0.001677
6 -0.395155 1.431529 -0.008680
1 -0.365256 2.514933 -0.012955
7 0.815196 0.800317 -0.010475
7 -0.197501 -1.323569 0.000775
8 2.083515 -1.098082 -0.007727
7 -2.482316 -1.504997 0.010185
1 -2.370261 -2.509781 0.014219
1 -3.416651 -1.123526 0.014241
1 1.669846 1.347124 -0.015517
6 -2.909698 1.372089 0.000337
1 -3.494949 1.082629 -0.881153
1 -3.489633 1.088256 0.887176
1 -2.810533 2.460563 -0.003327
8 4.372104 0.440880 -0.013354
1 3.549069 -0.102950 -0.008875
1 4.081473 1.326711 0.246098
5mC/5mCabx.xyz
6 1.898401 0.342990 0.088787
6 0.118786 -1.163770 0.163309
6 -0.737641 -0.315398 -0.630827
6 -0.179059 0.862377 -1.025455
1 -0.709467 1.595266 -1.622293
7 1.100561 1.183293 -0.677716
7 1.375516 -0.836990 0.501246

8 3.068777 0.709478 0.369091
7 -0.342741 -2.350241 0.599679
1 0.252065 -2.936677 1.169327
1 -1.283674 -2.659041 0.404771
1 1.504513 2.062959 -0.979975
6 -2.145521 -0.704857 -0.983608
1 -2.170157 -1.618777 -1.589840
1 -2.745568 -0.893077 -0.084322
1 -2.631035 0.091318 -1.553666
8 -2.577695 1.943112 1.342867
1 -1.674059 1.701592 1.090280
1 -3.134420 1.335234 0.834351
5mC/5mCacs.xyz
6 1.843756 -0.142602 0.020297
6 -0.331073 -0.823448 -0.477097
6 -0.798087 0.530151 -0.662577
6 0.143444 1.496670 -0.478942
1 -0.070600 2.554439 -0.580507
7 1.426751 1.171862 -0.150844
7 0.933510 -1.131673 -0.151605
8 3.044870 -0.360062 0.324292
7 -1.192233 -1.846781 -0.629981
1 -0.868353 -2.796224 -0.506214
1 -2.160305 -1.694568 -0.871068
1 2.116829 1.902545 -0.017097
6 -2.228199 0.840373 -1.002839
1 -2.520409 0.392433 -1.960420
1 -2.911298 0.453608 -0.236148
1 -2.379489 1.920517 -1.073852
8 -1.568663 0.146552 2.596536
1 -2.001206 -0.453720 1.971066
1 -0.970067 0.668316 2.041574
5mC/5mCabz.xyz
6 -1.702322 -0.322301 0.169357
6 0.522217 -0.937090 -0.172788
6 1.025940 0.101098 0.695117

6 0.073634 0.893487 1.261397
1 0.311497 1.714416 1.927846
7 -1.250472 0.688306 1.009687
7 -0.784536 -1.137027 -0.405691
8 -2.940131 -0.443504 -0.018122
7 1.387291 -1.764277 -0.787734
1 1.038339 -2.474743 -1.416309
1 2.385768 -1.658581 -0.684345
1 -1.947022 1.295210 1.427556
6 2.495511 0.298733 0.939836
1 2.951792 -0.593350 1.386228
1 3.031801 0.509113 0.006270
1 2.659595 1.138294 1.620167
8 0.334327 1.968099 -2.107870
1 -0.352015 1.285892 -2.061558
1 0.810814 1.874424 -1.269269
5mC/5mCacm.xyz
6 -0.778499 -1.081890 0.003673
6 1.494489 -0.557524 -0.006027
6 1.245465 0.862439 0.004292
6 -0.070783 1.215701 0.015410
1 -0.399340 2.249027 0.024536
7 -1.056466 0.276642 0.015407
7 0.518243 -1.478881 -0.006735
8 -1.740944 -1.896703 0.003316
7 2.763740 -1.010712 -0.016979
1 2.938706 -2.006242 -0.020832
1 3.552456 -0.381440 -0.012468
1 -2.042625 0.565107 0.022481
6 2.360602 1.870325 0.003705
1 3.007473 1.753485 0.882081
1 2.994356 1.766711 -0.885817
1 1.959109 2.886949 0.014607
8 -3.847429 1.037883 0.075070
1 -4.101880 1.497235 -0.739351
1 -4.287528 0.176079 0.019511

5mC/5mCaak.xyz
6 -1.718785 0.289953 0.156142
6 0.502240 0.945617 -0.124214
6 1.005815 -0.123082 0.705722
6 0.055597 -0.949088 1.225232
1 0.293698 -1.794188 1.860713
7 -1.266896 -0.748081 0.962746
7 -0.802947 1.138254 -0.371501
8 -2.955115 0.403642 -0.045167
7 1.367810 1.807785 -0.688306
1 1.022993 2.542943 -1.290095
1 2.365197 1.712400 -0.566828
1 -1.962492 -1.376749 1.348787
6 2.474726 -0.314326 0.959453
1 3.023542 -0.482540 0.024452
1 2.916002 0.564556 1.445507
1 2.640498 -1.177874 1.608549
8 0.460057 -1.838904 -2.186332
1 -0.312782 -1.268015 -2.062296
1 0.970489 -1.718591 -1.371379
5mC/5mCadk.xyz
6 -0.780200 -1.077386 -0.006296
6 1.494074 -0.559051 0.005384
6 1.248771 0.861586 -0.003302
6 -0.066467 1.218446 -0.018767
1 -0.392174 2.252667 -0.028481
7 -1.054540 0.281899 -0.022230
7 0.515444 -1.477808 0.005111
8 -1.744841 -1.889499 -0.003230
7 2.762119 -1.015545 0.016208
1 2.934480 -2.011539 0.017698
1 3.552471 -0.388331 0.011091
1 -2.040143 0.571562 -0.033280
6 2.366759 1.866271 0.003628
1 2.997332 1.757557 0.894814
1 3.016112 1.750724 -0.873078

1 1.968250 2.884087 -0.004772
8 -3.858896 1.004606 -0.082999
1 -4.272457 0.196664 0.257263
1 -4.072768 1.686734 0.571084
5mU/5mUadr.xyz
6 -1.916225 -0.377840 -0.090424
6 -0.018281 1.215365 -0.192683
6 0.748136 0.329900 0.667609
6 0.151719 -0.817763 1.083362
1 0.650518 -1.534597 1.724959
7 -1.130466 -1.158063 0.718390
1 -1.540283 -2.024120 1.052124
7 -1.308667 0.786870 -0.506871
8 -3.069556 -0.686949 -0.419810
8 0.394378 2.294660 -0.650586
1 -1.864347 1.392500 -1.105376
6 2.146355 0.719327 1.047228
1 2.756925 0.895129 0.154755
1 2.151646 1.647983 1.629012
1 2.617565 -0.066290 1.642798
8 2.732388 -1.780114 -1.392893
1 1.871249 -1.638386 -0.972413
1 3.302753 -1.108583 -0.990737
5mU/5mUadm.xyz
6 0.902577 0.789737 -0.004164
6 -0.859003 -0.949944 0.000650
6 -1.866043 0.097269 0.002837
6 -1.437209 1.385409 0.002675
1 -2.117365 2.229040 0.004575
7 -0.101133 1.718649 -0.000185
1 0.177497 2.694080 -0.001137
7 0.468157 -0.514008 -0.002529
8 2.106622 1.098016 -0.009144
8 -1.098767 -2.168760 0.001304
1 1.190876 -1.229673 -0.003210
6 -3.314653 -0.292212 0.004673

1 -3.559673 -0.891804 -0.879062
1 -3.555670 -0.898915 0.884586
1 -3.952974 0.594853 0.009518
8 4.048530 -0.963514 -0.041792
1 3.397860 -0.231697 -0.014107
1 4.845178 -0.605874 0.374873
5mU/5mUacz.xyz
6 -0.791737 -1.104932 0.002444
6 1.622044 -0.538805 0.000742
6 1.270091 0.868580 -0.001173
6 -0.052335 1.185088 -0.001626
1 -0.400937 2.211559 -0.003393
7 -1.047365 0.238897 0.000053
1 -2.036079 0.535852 -0.000270
7 0.548542 -1.429736 0.002438
8 -1.684223 -1.968050 0.004374
8 2.784546 -0.984809 0.000939
1 0.771869 -2.421336 0.003789
6 2.367524 1.892286 -0.003060
1 3.008683 1.781082 0.878611
1 3.008008 1.778439 -0.884886
1 1.953491 2.903768 -0.004458
8 -3.716318 1.201522 -0.029873
1 -4.134252 1.088460 0.837237
1 -4.244592 0.655458 -0.631556
5mU/5mUabf.xyz
6 -0.905952 -0.815613 0.018888
6 0.831337 0.947974 0.014595
6 1.852524 -0.085074 -0.011993
6 1.441381 -1.378898 -0.021355
1 2.132968 -2.212978 -0.040359
7 0.110091 -1.730498 -0.006786
1 -0.155520 -2.709577 -0.012855
7 -0.489565 0.493948 0.025881
8 -2.105864 -1.140512 0.034870
8 1.054316 2.169949 0.027998

1 -1.222296 1.199434 0.047067
6 3.295541 0.324199 -0.025096
1 3.514131 0.958661 -0.891088
1 3.546598 0.902355 0.871257
1 3.945464 -0.553544 -0.064187
8 -4.030151 0.935609 0.055939
1 -3.381541 0.202062 0.023509
1 -4.158872 1.193554 -0.867694
5mU/5mUacc.xyz
6 0.777737 1.126265 -0.005753
6 -1.627432 0.525114 0.003063
6 -1.255233 -0.877105 0.001465
6 0.071583 -1.174539 -0.004367
1 0.435730 -2.195667 -0.006509
7 1.052620 -0.213736 -0.007951
1 2.044948 -0.496249 -0.015017
7 -0.567161 1.431676 -0.000491
8 1.657423 2.002249 -0.008017
8 -2.796436 0.953579 0.007440
1 -0.804225 2.420140 0.000593
6 -2.338440 -1.915856 0.005879
1 -2.975375 -1.814061 0.891762
1 -2.986278 -1.810375 -0.871583
1 -1.910670 -2.921607 0.001103
8 3.762366 -1.082385 -0.079134
1 4.249080 -0.751148 0.690750
1 3.792468 -2.047425 0.003952
5mU/5mUadq.xyz
6 0.764784 1.147857 -0.004313
6 -1.631137 0.510386 0.002390
6 -1.237985 -0.886056 -0.001301
6 0.093144 -1.163444 -0.006285
1 0.473277 -2.178748 -0.009200
7 1.059905 -0.187906 -0.008131
1 2.055577 -0.458391 -0.010100
7 -0.584741 1.432780 0.000036

8 1.630108 2.037860 -0.004777
8 -2.806545 0.920798 0.007379
1 -0.835792 2.417840 0.002712
6 -2.306064 -1.940360 0.001873
1 -2.940788 -1.852700 0.890894
1 -2.959090 -1.839512 -0.872255
1 -1.863833 -2.939784 -0.010210
8 3.773643 -1.041420 -0.005572
1 3.960132 -1.500282 0.827497
1 3.910272 -1.710735 -0.693090
5mU/5mUacw.xyz
6 -0.782532 1.118085 0.003495
6 1.625936 0.530291 -0.002566
6 1.261452 -0.873960 -0.001376
6 -0.063717 -1.178709 0.003054
1 -0.421993 -2.201907 0.005010
7 -1.049999 -0.223405 0.005299
1 -2.041030 -0.510709 0.012303
7 0.560619 1.430949 -0.000451
8 -1.667298 1.989012 0.004977
8 2.792511 0.965403 -0.005407
1 0.792474 2.420626 -0.001088
6 2.350145 -1.906957 -0.004059
1 2.989152 -1.800487 -0.887883
1 2.994751 -1.799290 0.875528
1 1.927661 -2.914945 -0.002000
8 -3.755955 -1.095958 0.088318
1 -4.296890 -0.618820 -0.558862
1 -3.820242 -2.027419 -0.171329
5mU/5mUaaa.xyz
6 0.989987 1.518421 0.001926
6 -0.439079 -0.502818 0.020157
6 0.750235 -1.330312 -0.001763
6 1.953475 -0.699191 -0.019445
1 2.893809 -1.237373 -0.036475
7 2.067377 0.670769 -0.016657

1 2.985192 1.103088 -0.030567
7 -0.229001 0.873186 0.020074
8 1.095216 2.751684 0.002799
8 -1.604719 -0.947073 0.038058
1 -1.052001 1.470959 0.033755
6 0.596550 -2.822879 -0.004624
1 0.051347 -3.161138 0.883398
1 0.028965 -3.155733 -0.880670
1 1.573913 -3.311657 -0.019054
8 -3.842134 0.722801 -0.003324
1 -4.583290 0.157563 -0.262766
1 -3.060486 0.127981 0.010686
5mU/5mUabq.xyz
6 -0.786766 -1.111708 -0.004339
6 1.624300 -0.534587 0.004798
6 1.266031 0.871250 -0.001401
6 -0.057765 1.181813 -0.009839
1 -0.411361 2.206620 -0.015814
7 -1.048216 0.230926 -0.011455
1 -2.038115 0.522446 -0.019577
7 0.554946 -1.430505 0.002865
8 -1.675599 -1.978553 -0.004219
8 2.788870 -0.975025 0.011642
1 0.782614 -2.421129 0.007019
6 2.359031 1.899678 0.001314
1 2.992862 1.795499 0.889154
1 3.007794 1.784432 -0.874221
1 1.940728 2.909362 -0.008668
8 -3.749875 1.117636 -0.087866
1 -4.314210 0.493090 0.392589
1 -3.843576 1.955591 0.389999
5mU/5mUacp.xyz
6 -0.775231 1.249303 -0.024944
6 0.451755 -0.892735 -0.035853
6 1.705383 -0.158771 0.011208
6 1.640489 1.197277 0.035422

1 2.523370 1.824895 0.070387
7 0.444518 1.876578 0.015881
1 0.435982 2.890253 0.037970
7 -0.709290 -0.123352 -0.055923
8 -1.849982 1.870565 -0.033990
8 0.363286 -2.134866 -0.058843
1 -1.613682 -0.620834 -0.061466
6 2.995312 -0.924920 0.033460
1 3.048843 -1.577434 0.912280
1 3.085169 -1.566201 -0.850489
1 3.849891 -0.243826 0.055680
8 -3.273229 -1.437823 0.064329
1 -3.664380 -1.080565 0.876058
1 -3.838633 -1.102790 -0.647854
5mU/5mUadc.xyz
6 -0.773223 -1.132762 -0.010906
6 1.628961 -0.519629 0.005668
6 1.249900 0.880715 -0.001850
6 -0.078324 1.171586 -0.013773
1 -0.447683 2.190827 -0.020007
7 -1.054806 0.205989 -0.019152
1 -2.048156 0.484596 -0.025196
7 0.573238 -1.431350 -0.000366
8 -1.647906 -2.013585 -0.012776
8 2.800055 -0.942177 0.016974
1 0.814845 -2.418717 0.005216
6 2.328240 1.924501 0.005924
1 2.954394 1.834402 0.900793
1 2.987617 1.813515 -0.862157
1 1.896017 2.928164 -0.014190
8 -3.774522 1.043940 -0.027505
1 -3.878267 1.905740 -0.458552
1 -4.062128 1.187116 0.886796
5mU/5mUacq.xyz
6 -0.811759 1.069507 -0.000053
6 1.614384 0.559217 -0.004651

6 1.294964 -0.855977 0.003492
6 -0.019637 -1.203203 0.010023
1 -0.344256 -2.237509 0.016843
7 -1.035699 -0.280015 0.008475
1 -2.018596 -0.594793 0.012052
7 0.520570 1.425168 -0.005867
8 -1.725326 1.910383 -0.002144
8 2.766166 1.032180 -0.010498
1 0.721483 2.421504 -0.011356
6 2.415559 -1.854064 0.005102
1 3.049564 -1.732560 -0.880375
1 3.057373 -1.720788 0.883214
1 2.024744 -2.874695 0.013787
8 -3.736425 -1.168360 0.061370
1 -4.242786 -0.379354 0.308361
1 -4.038001 -1.384384 -0.834082
5mU/5mUabc.xyz
6 -0.802857 1.207525 -0.003761
6 0.486153 -0.898116 -0.010913
6 1.718066 -0.127165 0.003648
6 1.613494 1.226641 0.013365
1 2.478479 1.879550 0.024130
7 0.397936 1.870930 0.009874
1 0.359461 2.884255 0.018045
7 -0.696327 -0.162798 -0.015570
8 -1.896257 1.795439 -0.005729
8 0.432299 -2.142535 -0.019109
1 -1.587289 -0.682754 -0.023122
6 3.030896 -0.853919 0.007092
1 3.118058 -1.501567 0.886748
1 3.125973 -1.494956 -0.876522
1 3.864486 -0.147004 0.013566
8 -3.311394 -1.382622 -0.072057
1 -3.831037 -0.564408 -0.052700
1 -3.511092 -1.822096 0.768301
5mU/5mUabh.xyz

6 0.944974 0.539232 -0.025653
6 -1.520299 0.760559 0.011911
6 -1.631272 -0.688307 0.000875
6 -0.480979 -1.409286 -0.022820
1 -0.468636 -2.492790 -0.032977
7 0.762016 -0.816171 -0.035734
1 1.598866 -1.390905 -0.052791
7 -0.218911 1.268435 -0.005341
8 2.065892 1.077496 -0.035299
8 -2.480535 1.548336 0.034461
1 -0.115716 2.280072 0.001736
6 -2.996848 -1.309227 0.014911
1 -3.556130 -1.003919 0.906156
1 -3.579341 -0.992594 -0.857366
1 -2.926037 -2.399731 0.006894
8 4.320017 -0.599258 -0.050900
1 4.559042 -0.743736 0.875459
1 3.549768 0.007341 -0.021026
5mU/5mUacy.xyz
6 0.875753 -1.565071 -0.015769
6 -0.413890 0.547577 -0.070749
6 0.826442 1.292917 0.009596
6 1.982646 0.582360 0.073426
1 2.955270 1.055615 0.138211
7 2.005940 -0.792196 0.060364
1 2.892167 -1.283830 0.108419
7 -0.295486 -0.839060 -0.079264
8 0.896966 -2.802432 -0.027158
8 -1.546102 1.068739 -0.128906
1 -1.154868 -1.381069 -0.132619
6 0.775272 2.792023 0.020464
1 0.298402 3.173428 -0.889172
1 0.189656 3.157022 0.871458
1 1.782410 3.210807 0.087186
8 -3.862117 -0.504136 0.019952
1 -3.056755 0.055369 -0.027846

1 -4.066776 -0.564753 0.963752
5mU/5mUadb.xyz
6 -0.944302 -0.530542 -0.021692
6 1.520006 -0.763072 0.008923
6 1.637421 0.685320 0.002846
6 0.490322 1.411577 -0.013950
1 0.482937 2.495153 -0.019588
7 -0.755396 0.824080 -0.025565
1 -1.589612 1.402706 -0.036457
7 0.216347 -1.264997 -0.006528
8 -2.067513 -1.063896 -0.031966
8 2.476790 -1.555195 0.025903
1 0.108515 -2.276166 -0.003203
6 3.005943 1.299754 0.014376
1 3.566184 0.989959 0.903478
1 3.584390 0.981884 -0.860133
1 2.940451 2.390593 0.008596
8 -4.316406 0.616389 -0.054544
1 -4.768617 0.500989 0.793184
1 -3.550215 0.004686 -0.019390
5mU/5mUaac.xyz
6 0.885827 -1.561321 -0.015130
6 -0.416267 0.543718 -0.069099
6 0.819726 1.296382 0.009237
6 1.980196 0.592619 0.071227
1 2.950086 1.071664 0.134246
7 2.011551 -0.781745 0.058429
1 2.900729 -1.268151 0.105303
7 -0.289735 -0.842205 -0.077274
8 0.914360 -2.798546 -0.025543
8 -1.551637 1.058141 -0.125924
1 -1.145999 -1.389228 -0.129426
6 0.759705 2.795176 0.020353
1 0.277779 3.173710 -0.887794
1 0.174577 3.156669 0.873186
1 1.764554 3.219933 0.084093

8 -3.865152 -0.516650 0.016507
1 -4.073713 -0.578381 0.959383
1 -3.056413 0.038423 -0.026925
5mU/5mUaag.xyz
6 2.148547 -0.483981 0.050678
6 -0.320351 -0.473464 -0.144261
6 -0.308637 0.974112 -0.067685
6 0.898193 1.584688 0.065266
1 1.001899 2.661424 0.129728
7 2.078901 0.883264 0.128888
1 2.958923 1.379139 0.223416
7 0.921856 -1.098797 -0.082706
8 3.212716 -1.115065 0.095124
8 -1.342080 -1.178368 -0.261866
1 0.933191 -2.113939 -0.139727
6 -1.607259 1.722396 -0.135108
1 -2.160423 1.465393 -1.044766
1 -2.249446 1.473661 0.716464
1 -1.428949 2.800601 -0.129949
8 -4.056455 -0.677502 0.257733
1 -3.099146 -0.765999 0.064816
1 -4.337752 -1.566571 0.515473
5mU/5mUadh.xyz
6 -0.822101 -1.049233 -0.001181
6 1.610142 -0.569832 0.005338
6 1.308600 0.849310 -0.002968
6 -0.001371 1.213567 -0.010992
1 -0.312660 2.251972 -0.018272
7 -1.028838 0.303132 -0.010143
1 -2.008611 0.626837 -0.016148
7 0.505548 -1.421864 0.006092
8 -1.746634 -1.877972 0.000154
8 2.755932 -1.057120 0.011737
1 0.693765 -2.420688 0.011464
6 2.442428 1.832184 -0.003636
1 3.074765 1.700908 0.881631

1 3.082260 1.691093 -0.881981
1 2.065598 2.858051 -0.011065
8 -3.749822 1.133039 -0.079677
1 -4.225973 0.289186 -0.047067
1 -4.008100 1.594205 0.732710
5mU/5mUabd.xyz
6 2.152421 -0.477109 -0.047250
6 -0.317863 -0.481162 0.131009
6 -0.313178 0.967068 0.065412
6 0.891254 1.584937 -0.055323
1 0.989688 2.662631 -0.111436
7 2.075999 0.890264 -0.115994
1 2.953997 1.391410 -0.201132
7 0.928185 -1.099423 0.073720
8 3.220306 -1.102058 -0.089427
8 -1.336614 -1.192586 0.234877
1 0.944646 -2.114911 0.122665
6 -1.615891 1.708624 0.128226
1 -2.251487 1.460578 -0.728498
1 -2.173366 1.445177 1.033285
1 -1.442740 2.787691 0.128603
8 -4.049549 -0.661414 -0.262855
1 -3.094413 -0.774745 -0.071865
1 -4.409217 -1.559400 -0.268911
C/Cacj.xyz
6 -0.071043 -1.183921 -0.043022
6 -0.290126 1.149330 -0.032896
6 -1.719099 1.068998 0.035545
6 -2.262291 -0.176807 0.070219
1 -3.328098 -0.363190 0.125984
7 -1.458260 -1.275795 0.033759
7 0.486764 0.053925 -0.076341
8 0.609908 -2.234712 -0.074650
7 0.319953 2.345257 -0.059019
1 1.329724 2.398478 -0.083343
1 -0.214606 3.200517 0.004313

1 -2.335364 1.958088 0.064870
1 -1.861775 -2.206102 0.060991
8 3.288675 0.082371 0.101147
1 2.299254 0.074172 0.033145
1 3.538354 -0.852539 0.114194
C/Caap.xyz
6 -0.500677 -0.676644 -0.015595
6 1.206931 0.903214 -0.000500
6 2.221515 -0.110163 0.014193
6 1.797940 -1.401078 0.016427
1 2.466788 -2.253241 0.028293
7 0.464086 -1.677993 0.003229
7 -0.106026 0.616248 -0.016284
8 -1.711954 -1.025264 -0.032232
7 1.564331 2.198608 0.000716
1 0.858243 2.921823 -0.010222
1 2.538933 2.464714 0.009537
1 3.273833 0.143118 0.024656
1 0.141729 -2.639613 0.004739
8 -3.738521 0.842438 -0.022378
1 -4.486533 0.388560 0.390207
1 -3.000181 0.187242 -0.011100
C/Cabv.xyz
6 -0.497567 -0.708599 -0.001001
6 1.166387 0.917176 0.002317
6 2.207975 -0.068239 -0.000325
6 1.819862 -1.370331 -0.003481
1 2.511608 -2.204118 -0.005712
7 0.494058 -1.683396 -0.003672
7 -0.138041 0.594431 0.001219
8 -1.699224 -1.089241 -0.000974
7 1.487018 2.222006 0.006036
1 0.759657 2.924177 0.009871
1 2.453770 2.515390 0.008112
1 3.253037 0.213566 0.000062
1 0.197808 -2.653305 -0.005444

8 -3.777053 0.711616 0.035043
1 -3.456336 1.558827 -0.304043
1 -2.990519 0.115132 0.014464
C/Cabf.xyz
6 0.084933 -1.186070 -0.049896
6 0.276948 1.149485 -0.038059
6 1.706042 1.085974 0.042495
6 2.263237 -0.153431 0.082098
1 3.330594 -0.327448 0.147269
7 1.472498 -1.261741 0.038376
7 -0.487045 0.045209 -0.087776
8 -0.583294 -2.244819 -0.087061
7 -0.346485 2.338364 -0.069541
1 -1.356608 2.380094 -0.099565
1 0.177514 3.199589 0.000784
1 2.311677 1.982143 0.077145
1 1.886636 -2.187288 0.069256
8 -3.288351 0.062534 0.115935
1 -2.299865 0.054841 0.036552
1 -3.536527 -0.872216 0.150329
C/Cabb.xyz
6 -0.279115 1.199201 -0.045807
6 -0.099878 -1.136672 -0.032134
6 -1.521159 -1.300489 0.045719
6 -2.268184 -0.165733 0.078134
1 -3.349881 -0.162874 0.138373
7 -1.662121 1.053475 0.032704
7 0.481743 0.074504 -0.077057
8 0.210546 2.351839 -0.081184
7 0.700952 -2.213901 -0.062918
1 1.704940 -2.098392 -0.105819
1 0.313810 -3.146343 -0.027090
1 -1.974639 -2.282487 0.080375
1 -2.217588 1.901854 0.057036
8 3.296580 0.240493 0.028221
1 3.525874 0.837686 0.754130

1 2.306464 0.235511 0.002117
C/Caae.xyz
6 -0.264370 -1.199317 -0.045369
6 -0.113633 1.138384 -0.031800
6 -1.536922 1.284859 0.044253
6 -2.270216 0.141154 0.076554
1 -3.351842 0.124893 0.136007
7 -1.649094 -1.070518 0.031990
7 0.482756 -0.065521 -0.075973
8 0.239488 -2.345937 -0.080195
7 0.673949 2.225379 -0.060973
1 1.679358 2.122901 -0.102812
1 0.274982 3.152862 -0.026058
1 -2.002099 2.261405 0.078296
1 -2.194060 -1.925625 0.056560
8 3.298949 -0.201933 0.045741
1 3.534478 -0.960339 0.598507
1 2.309259 -0.208993 0.008000
C/Cabn.xyz
6 0.494943 -0.729381 -0.000015
6 -1.140201 0.925433 -0.000002
6 -2.198878 -0.041501 0.000019
6 -1.833493 -1.350272 0.000015
1 -2.539847 -2.171734 0.000029
7 -0.513437 -1.686728 -0.000006
7 0.157971 0.579492 -0.000027
8 1.690422 -1.129364 -0.000013
7 -1.436176 2.235954 -0.000005
1 -0.694995 2.923692 -0.000010
1 -2.397067 2.547922 0.000010
1 -3.238839 0.258505 0.000040
1 -0.233999 -2.661599 -0.000009
8 3.735052 0.714416 0.000031
1 3.343860 1.599038 -0.000048
1 2.964369 0.097058 0.000012
C/Caba.xyz

6 -0.192103 1.047595 0.022519
6 1.799464 -0.163362 -0.013324
6 1.104992 -1.416387 0.013214
6 -0.253783 -1.357885 0.038920
1 -0.890023 -2.235489 0.055413
7 -0.889715 -0.156193 0.044364
7 1.164765 1.019288 -0.008533
8 -0.847599 2.121450 0.029596
7 3.145328 -0.149736 -0.042276
1 3.641813 0.730024 -0.077190
1 3.671173 -1.011734 -0.064978
1 1.637194 -2.358955 0.008694
1 -1.916393 -0.130174 0.051256
8 -3.765465 -0.485286 0.027524
1 -4.082857 -0.289419 -0.866931
1 -3.850467 -1.446840 0.113913
C/Cabk.xyz
6 -0.308865 1.198437 -0.045317
6 -0.071528 -1.132539 -0.034613
6 -1.488075 -1.331923 0.046176
6 -2.262718 -0.215862 0.081021
1 -3.343995 -0.240414 0.143379
7 -1.687670 1.018151 0.035796
7 0.479540 0.092968 -0.079688
8 0.151674 2.362793 -0.080691
7 0.756319 -2.188983 -0.068670
1 1.756684 -2.047265 -0.118391
1 0.393477 -3.131281 -0.036349
1 -1.917440 -2.324679 0.080777
1 -2.264228 1.852395 0.061330
8 3.287601 0.316089 0.014360
1 3.513777 0.607150 0.908974
1 2.297317 0.289408 -0.004734
C/Cack.xyz
6 -0.486804 -0.521008 -0.031770
6 1.821677 -0.236204 0.016220

6 1.702096 1.193011 0.011725
6 0.443121 1.702659 -0.022062
1 0.223980 2.763547 -0.031387
7 -0.628780 0.862406 -0.044103
7 0.755270 -1.053959 -0.002204
8 -1.528856 -1.231688 -0.048789
7 3.040672 -0.801246 0.040339
1 3.134285 -1.807771 0.035525
1 3.875169 -0.231555 0.048257
1 2.573927 1.834196 0.030499
1 -1.570921 1.238449 -0.071720
8 -3.975449 0.040182 -0.036042
1 -4.139069 0.293236 0.883270
1 -3.113605 -0.439210 -0.018695
C/Caac.xyz
6 -0.487117 -0.515844 -0.046754
6 1.821920 -0.239739 0.021369
6 1.707667 1.189887 0.018329
6 0.450900 1.704264 -0.024688
1 0.235706 2.765966 -0.033669
7 -0.623925 0.868046 -0.056913
7 0.752712 -1.053526 -0.007607
8 -1.531576 -1.222605 -0.074403
7 3.038653 -0.809210 0.054812
1 3.128748 -1.816045 0.049714
1 3.875081 -0.242544 0.071860
1 2.581600 1.827900 0.045184
1 -1.564574 1.247504 -0.088876
8 -3.974832 0.055178 -0.011978
1 -4.222177 0.116801 0.921342
1 -3.115417 -0.428746 -0.016092
C/Cabo.xyz
6 0.497949 -0.704976 -0.004816
6 -1.170330 0.916140 0.002452
6 -2.209243 -0.072135 0.002380
6 -1.817609 -1.373147 -0.001495

1 -2.507041 -2.208849 -0.001759
7 -0.490936 -1.682509 -0.004815
7 0.135066 0.597084 -0.002620
8 1.700614 -1.082612 -0.007263
7 -1.494833 2.220019 0.007575
1 -0.769682 2.924442 0.011012
1 -2.462465 2.510457 0.013043
1 -3.255068 0.206823 0.005512
1 -0.191994 -2.651598 -0.006504
8 3.774839 0.718993 0.064659
1 2.990411 0.120496 0.026744
1 3.542539 1.469728 -0.499319
C/Cabc.xyz
6 -0.505162 -0.663586 0.068833
6 1.219823 0.896063 0.009398
6 2.220282 -0.128631 -0.063994
6 1.780726 -1.414138 -0.076366
1 2.437873 -2.273412 -0.134554
7 0.445172 -1.675491 -0.013771
7 -0.094941 0.624444 0.075816
8 -1.718630 -0.997985 0.137249
7 1.593129 2.186947 0.014743
1 0.897719 2.918356 0.070369
1 2.569992 2.441991 -0.023247
1 3.274362 0.112350 -0.113743
1 0.110288 -2.632834 -0.023962
8 -3.758672 0.835905 -0.046139
1 -3.000206 0.206448 0.011531
1 -4.369148 0.434188 -0.680022
C/Cacr.xyz
6 0.487881 -0.498947 -0.055516
6 -1.824555 -0.251416 0.023339
6 -1.728213 1.179519 0.026395
6 -0.478380 1.709574 -0.025212
1 -0.276404 2.773876 -0.032621
7 0.606782 0.886921 -0.068458

7 -0.745587 -1.051550 -0.012610
8 1.539851 -1.193005 -0.084737
7 -3.033911 -0.836353 0.058047
1 -3.110731 -1.844250 0.047609
1 -3.877676 -0.280839 0.080767
1 -2.609458 1.806883 0.062614
1 1.541438 1.279531 -0.109613
8 4.052871 -0.058661 0.103437
1 3.148433 -0.445898 0.039364
1 3.911236 0.898526 0.109400
C/Cadt.xyz
6 0.392670 1.192691 0.025489
6 -0.006534 -1.116401 0.028600
6 1.393850 -1.414712 -0.025212
6 2.244885 -0.355285 -0.054987
1 3.322643 -0.456533 -0.097652
7 1.756846 0.916182 -0.031153
7 -0.470824 0.144617 0.054922
8 0.012917 2.385796 0.046895
7 -0.907221 -2.111848 0.056228
1 -1.895870 -1.902658 0.101558
1 -0.610639 -3.077555 0.043845
1 1.753316 -2.435261 -0.042956
1 2.390892 1.707601 -0.053525
8 -3.255350 0.541753 -0.019104
1 -3.553231 -0.185921 -0.583551
1 -2.268481 0.449518 0.006627
C/Caau.xyz
6 -0.488155 -0.504045 0.018176
6 1.824540 -0.247901 -0.005904
6 1.722984 1.182698 -0.010420
6 0.470158 1.708025 0.002250
1 0.264007 2.771549 0.001101
7 -0.612677 0.881363 0.016573
7 0.747955 -1.051978 0.007394
8 -1.537866 -1.202296 0.029416

7 3.036388 -0.828530 -0.015234
1 3.116461 -1.836217 -0.011394
1 3.878448 -0.270078 -0.025863
1 2.602555 1.813354 -0.022300
1 -1.549158 1.271665 0.025612
8 -4.041764 -0.048732 -0.059722
1 -3.912308 0.870277 0.214161
1 -3.141798 -0.450969 -0.024616
C/Cace.xyz
6 0.277594 0.948339 0.011388
6 -1.818694 -0.071189 -0.004991
6 -1.241850 -1.383383 0.004485
6 0.116930 -1.451621 0.015445
1 0.667756 -2.385017 0.020855
7 0.861738 -0.314568 0.019839
7 -1.076460 1.047084 -0.000952
8 1.030252 1.956502 0.014971
7 -3.157940 0.067201 -0.019410
1 -3.572237 0.989159 -0.024186
1 -3.762090 -0.742247 -0.020854
1 -1.857528 -2.273673 0.000864
1 1.888768 -0.370214 0.019719
8 3.733717 -0.569665 -0.039173
1 4.097921 -0.198848 0.778776
1 4.030422 0.035246 -0.735864
C/Cadk.xyz
6 -0.348625 -1.195427 -0.029483
6 -0.034246 1.126591 -0.024989
6 -1.444643 1.373477 0.029026
6 -2.256087 0.283377 0.054215
1 -3.336887 0.344475 0.096266
7 -1.722048 -0.969309 0.025166
7 0.475680 -0.116402 -0.053416
8 0.074955 -2.373767 -0.053301
7 0.829449 2.154438 -0.049878
1 1.824873 1.981137 -0.095945

1 0.498217 3.108774 -0.035776
1 -1.841707 2.379923 0.050851
1 -2.326824 -1.783590 0.043257
8 3.263571 -0.506544 0.076811
1 3.614050 0.395264 0.100199
1 2.280114 -0.392694 0.027347
C/Caah.xyz
6 -0.892757 -0.810935 -0.451853
6 1.320702 -0.109604 -0.256037
6 1.229988 -0.029669 1.173746
6 0.041921 -0.382245 1.732627
1 -0.147113 -0.368629 2.799350
7 -0.989679 -0.781328 0.938868
7 0.293066 -0.494294 -1.028885
8 -1.909474 -1.135028 -1.113912
7 2.476106 0.207837 -0.866207
1 2.549252 0.152139 -1.872852
1 3.282228 0.496202 -0.330045
1 2.068554 0.292109 1.777771
1 -1.886071 -1.020891 1.347912
8 -1.352831 2.614961 -0.121271
1 -0.591077 2.155365 0.262265
1 -1.832911 1.923441 -0.600272
C/Cacl.xyz
6 -0.368977 -1.194569 -0.000015
6 -0.015157 1.121851 0.004113
6 -1.422224 1.392962 0.000541
6 -2.252700 0.317001 -0.004816
1 -3.333085 0.396922 -0.008534
7 -1.739505 -0.944724 -0.005910
7 0.474184 -0.129742 0.004553
8 0.034838 -2.379963 0.000660
7 0.865837 2.135137 0.007052
1 1.859502 1.946328 0.009666
1 0.549956 3.094764 0.008726
1 -1.802064 2.406255 0.001597

1 -2.358364 -1.748459 -0.009832
8 3.260338 -0.526081 0.027350
1 3.599042 0.326136 -0.281916
1 2.274336 -0.421762 0.017417
U/Uacb.xyz
6 -1.912028 -0.346673 0.050977
6 0.558668 -0.399483 -0.111701
6 0.554492 1.040815 -0.116691
6 -0.629124 1.699338 -0.034068
1 -0.705097 2.779903 -0.028588
7 -1.820028 1.026666 0.049565
1 -2.694377 1.538679 0.106337
7 -0.697881 -0.996572 -0.027557
8 -2.989354 -0.948093 0.116856
8 1.571992 -1.124248 -0.171760
1 -0.734035 -2.012885 -0.026601
1 1.495304 1.569331 -0.180741
8 4.059070 0.143197 0.103951
1 3.188709 -0.304506 0.022843
1 4.509142 -0.312024 0.829212
U/Uaac.xyz
6 0.429040 -0.808286 0.005235
6 -1.151272 1.100521 -0.000234
6 -2.234322 0.146544 -0.003292
6 -1.954254 -1.179923 -0.000737
1 -2.719726 -1.946631 -0.002851
7 -0.661985 -1.640562 0.004250
1 -0.472077 -2.637452 0.003651
7 0.129104 0.533502 0.006450
8 1.593144 -1.238291 0.005287
8 -1.271764 2.336581 -0.002709
1 0.922591 1.170860 0.007177
1 -3.252704 0.509821 -0.008245
8 3.666645 0.674269 -0.107879
1 3.843808 0.930112 0.808294
1 2.968929 -0.010901 -0.046352

U/U114_abc.xyz
6 -1.105180 1.064846 -0.014819
6 0.449177 -0.864016 -0.047470
6 -0.691897 -1.739429 0.017943
6 -1.936993 -1.201931 0.064578
1 -2.837107 -1.802342 0.116900
7 -2.134983 0.153885 0.047431
1 -3.075297 0.534052 0.083656
7 0.151543 0.498294 -0.058020
8 -1.288039 2.286653 -0.031531
8 1.639328 -1.234632 -0.091940
1 0.932492 1.148575 -0.109723
1 -0.534428 -2.808978 0.031756
8 3.677591 0.694102 -0.011812
1 2.980984 0.002581 -0.007786
1 3.895766 0.835051 0.920195
U/U114_abu.xyz
6 -0.241459 0.991056 0.001547
6 1.932806 -0.206141 -0.000130
6 1.198491 -1.446410 -0.008348
6 -0.159231 -1.412194 -0.010164
1 -0.768114 -2.308864 -0.015116
7 -0.858234 -0.235637 -0.005547
1 -1.890842 -0.252756 -0.001899
7 1.137183 0.942869 0.004048
8 -0.867484 2.060212 0.004830
8 3.172021 -0.091599 0.003364
1 1.616251 1.839624 0.009057
1 1.743883 -2.380123 -0.011816
8 -3.685728 -0.424293 0.049734
1 -3.939784 -1.236839 0.512859
1 -4.048154 -0.524093 -0.843457
U/Uacu.xyz
6 0.434775 -0.777380 -0.023334
6 -1.201602 1.083745 -0.005737
6 -2.256141 0.098851 0.023418

6 -1.936799 -1.218768 0.028236
1 -2.679037 -2.007648 0.050194
7 -0.631673 -1.641281 0.006245
1 -0.412925 -2.632209 0.013862
7 0.094383 0.554537 -0.032381
8 1.610523 -1.172893 -0.040908
8 -1.357150 2.315852 -0.010607
1 0.865794 1.217072 -0.059628
1 -3.284499 0.432220 0.042671
8 3.684834 0.750025 0.056340
1 2.999499 0.051115 0.019810
1 4.525141 0.284089 -0.058060
U/U114_aaf.xyz
6 1.102257 1.064991 -0.019067
6 -0.449783 -0.865736 -0.054736
6 0.691785 -1.739650 0.022053
6 1.935935 -1.200621 0.075491
1 2.836290 -1.799815 0.136718
7 2.132522 0.155319 0.054346
1 3.072148 0.536652 0.095634
7 -0.153581 0.496930 -0.068371
8 1.284098 2.286879 -0.039913
8 -1.639241 -1.237703 -0.105840
1 -0.934816 1.146114 -0.128946
1 0.535333 -2.809302 0.039010
8 -3.683915 0.683070 0.006738
1 -2.980355 -0.001348 -0.008991
1 -3.749893 0.950071 0.934415
U/U114_acw.xyz
6 -0.288921 -0.939058 0.006507
6 1.943961 0.144209 -0.007243
6 1.274103 1.420580 0.009693
6 -0.083525 1.456768 0.022627
1 -0.645205 2.383536 0.033296
7 -0.841680 0.317553 0.022583
1 -1.873228 0.380643 0.022538

7 1.090379 -0.962385 -0.007735
8 -0.970009 -1.974112 0.005200
8 3.175811 -0.033034 -0.021327
1 1.522833 -1.882528 -0.018860
1 1.866428 2.325296 0.010071
8 -3.663582 0.572926 0.020991
1 -3.992960 0.468193 -0.884544
1 -4.030233 -0.182551 0.505137
U/U114_abt.xyz
6 0.287647 0.936973 -0.017376
6 -1.945784 -0.144587 0.013247
6 -1.277008 -1.421614 -0.003722
6 0.080418 -1.459053 -0.025754
1 0.641398 -2.386267 -0.036724
7 0.839054 -0.320253 -0.034885
1 1.869832 -0.382065 -0.041332
7 -1.091483 0.961435 0.005353
8 0.970145 1.970972 -0.020937
8 -3.177377 0.033467 0.034594
1 -1.523169 1.881938 0.016847
1 -1.870071 -2.325813 0.003296
8 3.678503 -0.467193 -0.062354
1 4.017707 -1.284499 0.332820
1 3.989490 0.240160 0.523029
U/Uadn.xyz
6 1.908062 -0.318293 0.055628
6 -0.559501 -0.440432 -0.114706
6 -0.594148 0.999478 -0.134847
6 0.570598 1.691157 -0.057740
1 0.617230 2.773449 -0.063351
7 1.779125 1.051924 0.034641
1 2.638813 1.588268 0.090856
7 0.712297 -1.002004 -0.017602
8 3.001170 -0.888988 0.133485
8 -1.552935 -1.192344 -0.171956
1 0.776114 -2.016874 -0.004098

1 -1.549077 1.500915 -0.208344
8 -4.053782 0.037741 0.174027
1 -3.853103 0.942670 0.452256
1 -3.175635 -0.380594 0.038945
U/Uabj.xyz
6 0.265016 0.964088 -0.013653
6 -1.940119 -0.174575 0.009645
6 -1.238859 -1.433957 0.001054
6 0.119230 -1.436212 -0.013857
1 0.703782 -2.348871 -0.019605
7 0.848771 -0.278543 -0.022350
1 1.880859 -0.318097 -0.028824
7 -1.114356 0.952924 0.001528
8 0.920217 2.015623 -0.018054
8 -3.175869 -0.027571 0.023860
1 -1.569544 1.862040 0.007675
1 -1.808642 -2.352991 0.007499
8 3.681711 -0.454912 -0.065400
1 3.967793 -1.371747 0.064000
1 4.044766 0.027820 0.692623
U/Uabg.xyz
6 0.309792 0.913222 -0.015785
6 -1.947689 -0.116831 0.014409
6 -1.308606 -1.408878 -0.013613
6 0.047433 -1.477624 -0.038733
1 0.587013 -2.417268 -0.056445
7 0.831813 -0.356326 -0.042724
1 1.861222 -0.439422 -0.045046
7 -1.068409 0.969499 0.011164
8 1.015992 1.931244 -0.015763
8 -3.174930 0.088737 0.041171
1 -1.478747 1.899620 0.030216
1 -1.922350 -2.299213 -0.010877
8 3.671328 -0.539901 -0.030786
1 3.962428 -0.709715 0.878037
1 3.941914 0.373822 -0.209608

U/U114_acg.xyz
6 -0.287277 0.941098 -0.000753
6 1.943660 -0.146374 -0.000465
6 1.271287 -1.421507 0.001963
6 -0.086473 -1.455126 0.002811
1 -0.649927 -2.380868 0.004448
7 -0.842562 -0.314510 0.001570
1 -1.874222 -0.376180 0.001366
7 1.092142 0.961797 -0.001754
8 -0.966215 1.977558 -0.001699
8 3.175899 0.028658 -0.001452
1 1.526387 1.881161 -0.003241
1 1.861874 -2.327357 0.002993
8 -3.662546 -0.582445 0.012582
1 -4.020700 -0.217745 -0.811022
1 -4.014766 -0.008738 0.709963
U/Uadu.xyz
6 -0.317051 0.903276 -0.012044
6 1.949199 -0.107217 0.011747
6 1.321145 -1.404861 -0.009400
6 -0.034281 -1.485443 -0.029267
1 -0.565817 -2.429705 -0.043083
7 -0.828025 -0.370824 -0.032680
1 -1.856657 -0.460475 -0.036945
7 1.060753 0.971671 0.009116
8 -1.032401 1.914884 -0.012335
8 3.174806 0.108588 0.032122
1 1.463134 1.905357 0.023092
1 1.942566 -2.289862 -0.006940
8 -3.669007 -0.506805 -0.059193
1 -3.900794 0.430019 0.035403
1 -4.012783 -0.929119 0.742444
U/Uabw.xyz
6 1.911533 0.347043 0.052417
6 -0.558835 0.400199 -0.115132
6 -0.554802 -1.040126 -0.120577

6 0.628597 -1.698819 -0.036240
1 0.704446 -2.779392 -0.031107
7 1.819409 -1.026293 0.049797
1 2.693571 -1.538422 0.108344
7 0.697632 0.997107 -0.028465
8 2.988774 0.948291 0.121201
8 -1.572046 1.124949 -0.176767
1 0.733869 2.013413 -0.026653
1 -1.495451 -1.568742 -0.186687
8 -4.044895 -0.164080 0.136754
1 -4.603929 0.455711 0.625782
1 -3.185428 0.298675 0.028690
U/Uaab.xyz
6 1.904703 -0.355523 0.070879
6 -0.561283 -0.385850 -0.157457
6 -0.544317 1.054266 -0.161648
6 0.643141 1.701861 -0.051084
1 0.728795 2.781684 -0.045045
7 1.825523 1.018668 0.062345
1 2.702504 1.522987 0.142516
7 0.687252 -0.994128 -0.042470
8 2.973789 -0.966631 0.171313
8 -1.578929 -1.101853 -0.244132
1 0.713494 -2.010728 -0.037099
1 -1.478572 1.590987 -0.249358
8 -4.073324 0.103687 0.167155
1 -4.291676 -0.131046 1.080117
1 -3.189717 -0.295808 0.010920
U/Uabe.xyz
6 0.493475 0.490844 -0.095357
6 -1.968867 0.254947 0.053293
6 -1.789886 -1.177059 0.029814
6 -0.538136 -1.690463 -0.057472
1 -0.334131 -2.754103 -0.080980
7 0.567753 -0.879957 -0.119363
1 1.496653 -1.285207 -0.181626

7 -0.782206 0.994983 -0.007270
8 1.495375 1.221108 -0.145906
8 -3.056754 0.850289 0.121043
1 -0.863495 2.008582 0.008798
1 -2.661844 -1.814770 0.077859
8 4.011112 -0.078042 0.094322
1 3.921499 -0.583959 0.914400
1 3.165112 0.407827 0.010637
U/Uabz.xyz
6 1.142418 1.053916 -0.011781
6 -0.454356 -0.840510 -0.040993
6 0.668214 -1.740755 0.009706
6 1.924913 -1.230518 0.051176
1 2.812051 -1.850732 0.094014
7 2.152704 0.120858 0.041191
1 3.101449 0.479753 0.073759
7 -0.126558 0.514718 -0.048838
8 1.350510 2.271671 -0.025489
8 -1.652433 -1.184546 -0.076001
1 -0.891926 1.183248 -0.092959
1 0.487883 -2.806760 0.018420
8 -3.679865 0.755917 0.087968
1 -4.526191 0.296515 -0.006470
1 -2.999111 0.051800 0.026300
U/Uadp.xyz
6 -0.493119 -0.486876 -0.097082
6 1.969726 -0.257577 0.053533
6 1.794276 1.174905 0.034508
6 0.543848 1.691610 -0.052431
1 0.342461 2.755817 -0.072718
7 -0.563952 0.884058 -0.118052
1 -1.491972 1.291617 -0.179409
7 0.781221 -0.994502 -0.009715
8 -1.496933 -1.214516 -0.149506
8 3.056118 -0.855837 0.119783
1 0.860039 -2.008333 0.003429

1 2.667696 1.810378 0.085455
8 -4.002933 0.101472 0.079665
1 -4.075673 0.337504 1.015301
1 -3.161831 -0.395194 0.011604
U/Uadv.xyz
6 -0.491409 -0.487774 -0.066354
6 1.973934 -0.256524 0.032878
6 1.796765 1.175864 0.024705
6 0.544105 1.691604 -0.031243
1 0.341232 2.755674 -0.040874
7 -0.563946 0.883209 -0.076851
1 -1.493627 1.290414 -0.111118
7 0.785074 -0.994384 -0.010477
8 -1.495527 -1.216041 -0.101543
8 3.061986 -0.854068 0.073937
1 0.865223 -2.008193 -0.004533
1 2.670467 1.812025 0.060055
8 -3.980342 0.118300 0.219586
1 -4.400270 0.196046 -0.648648
1 -3.160224 -0.392297 0.060657
U/U114_aav.xyz
6 0.493094 -0.480733 -0.096269
6 -1.970950 -0.262358 0.052324
6 -1.801549 1.170891 0.037883
6 -0.553192 1.693085 -0.045917
1 -0.356297 2.758188 -0.062571
7 0.558140 0.890516 -0.112832
1 1.484475 1.302477 -0.170233
7 -0.779227 -0.994012 -0.011904
8 1.499946 -1.204077 -0.149803
8 -3.054878 -0.865434 0.115429
1 -0.853823 -2.008195 -0.002098
1 -2.677646 1.802594 0.089639
8 4.001860 0.117420 0.072022
1 3.162906 -0.383885 0.012063
1 4.208171 0.144718 1.017028

rd_1m5caC/rd_1m5caC_3_021.xyz
6 -2.162610 -0.882175 -0.026154
6 0.165301 -1.013874 -0.060742
6 0.289791 0.438659 0.010162
6 -0.900875 1.178393 0.052376
1 -0.885035 2.262307 0.090985
7 -2.075885 0.574070 0.059907
7 -1.030277 -1.606475 -0.080137
8 -3.299121 -1.360023 -0.041998
7 1.255254 -1.772569 -0.107711
1 1.133757 -2.778668 -0.153374
1 2.213918 -1.407941 -0.074615
6 1.593303 1.180224 -0.001121
8 2.322016 1.145832 1.018226
8 1.811099 1.773137 -1.091235
6 -3.332920 1.329026 0.152043
1 -3.938724 1.122234 -0.731527
1 -3.872166 1.003307 1.042981
1 -3.100147 2.390547 0.214179
8 4.063242 -0.987471 0.114767
1 3.846726 -0.116420 0.492969
1 4.468186 -0.793865 -0.743468
rd_1m5caC/rd_1m5caC_3_079.xyz
6 -1.967646 -0.610871 -0.240471
6 0.271404 -0.978616 -0.753284
6 0.628670 0.374477 -0.343364
6 -0.417791 1.221729 0.046114
1 -0.227642 2.253957 0.321018
7 -1.659350 0.774506 0.108648
7 -0.982622 -1.417089 -0.683239
8 -3.142653 -0.954794 -0.112350
7 1.218971 -1.795604 -1.205609
1 0.973973 -2.741109 -1.477997
1 2.195103 -1.525910 -1.219699
6 2.039389 0.879356 -0.280173
8 2.829916 0.344398 0.533702

8 2.268622 1.811424 -1.095657
6 -2.773532 1.630302 0.539886
1 -3.529363 1.642762 -0.246770
1 -3.207556 1.215501 1.450979
1 -2.394210 2.634401 0.719746
8 1.422908 -1.168799 2.546059
1 1.935559 -0.680856 1.869915
1 0.501837 -1.087533 2.257942
rd_1m5caC/rd_1m5caC_3_013.xyz
6 2.031231 0.755148 -0.172789
6 -0.222316 0.881768 -0.740937
6 -0.470644 -0.466306 -0.240198
6 0.630296 -1.165245 0.278626
1 0.524524 -2.181507 0.641636
7 1.822360 -0.600816 0.339076
7 0.989512 1.430536 -0.696252
8 3.176367 1.198468 -0.087663
7 -1.229617 1.577302 -1.261418
1 -1.065133 2.521030 -1.593278
1 -2.181255 1.230788 -1.227457
6 -1.795015 -1.162546 -0.334452
8 -2.821599 -0.615024 0.136665
8 -1.715152 -2.265522 -0.941175
6 2.990287 -1.315920 0.875404
1 3.756511 -1.370557 0.100751
1 3.381868 -0.763168 1.730770
1 2.678989 -2.314341 1.176410
8 -2.560055 1.529996 1.955087
1 -2.644045 0.790228 1.317037
1 -3.146775 1.293623 2.687038
rd_1m5caC/rd_1m5caC_3_009.xyz
6 -1.312796 0.606019 -0.431862
6 0.898657 1.203845 -0.040465
6 1.323866 -0.191058 -0.045453
6 0.332805 -1.155134 -0.282746
1 0.577038 -2.211458 -0.301005

7 -0.921974 -0.798890 -0.493542
7 -0.377729 1.546530 -0.213094
8 -2.512066 0.848000 -0.600143
7 1.807708 2.156116 0.141579
1 1.522152 3.129054 0.146894
1 2.794223 1.928890 0.202528
6 2.717596 -0.640300 0.269041
8 3.689243 -0.132195 -0.334760
8 2.743375 -1.507714 1.189845
6 -1.971066 -1.789593 -0.777487
1 -2.728629 -1.742500 0.006021
1 -2.428774 -1.547959 -1.737880
1 -1.518116 -2.778557 -0.808827
8 -4.576287 0.006199 1.224347
1 -4.592962 0.728553 1.868490
1 -3.869448 0.250689 0.598701
rd_1m5caC/rd_1m5caCaci.xyz
6 -2.028538 0.711470 -0.188829
6 0.222981 0.918926 -0.737239
6 0.507466 -0.433394 -0.268706
6 -0.573292 -1.176547 0.230805
1 -0.438426 -2.196362 0.574520
7 -1.781127 -0.647008 0.299656
7 -1.005252 1.429583 -0.691118
8 -3.187581 1.116560 -0.102718
7 1.213877 1.658086 -1.228159
1 1.023153 2.605493 -1.534895
1 2.175258 1.339889 -1.190787
6 1.855762 -1.081332 -0.364322
8 2.853413 -0.511491 0.142347
8 1.825587 -2.165786 -1.006640
6 -2.927214 -1.398812 0.831788
1 -3.319570 -0.872282 1.703359
1 -3.701449 -1.453051 0.065292
1 -2.592514 -2.396791 1.107899
8 2.327229 1.426992 2.118423

1 2.462630 0.959932 2.954839
1 2.506256 0.756491 1.424836
rd_1m5caC/rd_1m5caCaat.xyz
6 -2.010575 0.688559 -0.220126
6 0.248800 0.912272 -0.726554
6 0.534375 -0.437797 -0.252324
6 -0.549364 -1.188118 0.229102
1 -0.413465 -2.206264 0.577383
7 -1.761514 -0.665794 0.277937
7 -0.983498 1.414266 -0.703626
8 -3.174531 1.083955 -0.159186
7 1.243912 1.659175 -1.197253
1 1.051870 2.604350 -1.510056
1 2.206754 1.347898 -1.143879
6 1.891769 -1.071599 -0.316905
8 2.868765 -0.499136 0.225080
8 1.889501 -2.148184 -0.973072
6 -2.910954 -1.423371 0.793693
1 -3.336894 -0.883251 1.640562
1 -3.660925 -1.508167 0.005796
1 -2.569042 -2.409225 1.102648
8 2.187570 1.403241 2.195021
1 2.438941 0.763565 1.494989
1 1.395711 1.838877 1.849101
rd_1m5caC/rd_1m5caC_3_074.xyz
6 2.161139 -0.883384 0.028912
6 -0.166299 -1.009891 0.089654
6 -0.289131 0.442346 0.009006
6 0.902438 1.178536 -0.064810
1 0.888595 2.261827 -0.119139
7 2.075803 0.571123 -0.083613
7 1.028156 -1.604393 0.106176
8 3.296877 -1.362930 0.042674
7 -1.256682 -1.767146 0.150892
1 -1.135491 -2.773126 0.200279
1 -2.214638 -1.404201 0.101132

6 -1.591435 1.186087 0.038493
8 -2.325355 1.157302 -0.978353
8 -1.802960 1.774085 1.131318
6 3.333220 1.321463 -0.203305
1 3.944216 1.134680 0.681193
1 3.865032 0.972684 -1.089892
1 3.101114 2.381397 -0.290149
8 -4.060873 -0.999298 -0.211895
1 -4.536077 -0.806937 0.609788
1 -3.804792 -0.127626 -0.565038
rd_1m5caC/rd_1m5caCacx.xyz
6 2.268784 -0.841467 -0.072476
6 -0.045528 -1.075925 -0.003843
6 -0.232041 0.368887 0.086652
6 0.925375 1.162121 0.100639
1 0.858317 2.243012 0.159626
7 2.123391 0.609780 0.051279
7 1.167614 -1.617334 -0.090118
8 3.421669 -1.266285 -0.148761
7 -1.111709 -1.871327 -0.005728
1 -0.982424 -2.875074 -0.066727
1 -2.047848 -1.505041 0.123680
6 -1.573251 1.036662 0.089273
8 -2.425479 0.684295 0.942943
8 -1.703588 1.888625 -0.829660
6 3.352507 1.414004 0.107539
1 3.932683 1.106672 0.979303
1 3.936739 1.233522 -0.795859
1 3.081233 2.465627 0.181997
8 -4.708555 -0.628116 -0.131814
1 -3.973184 -0.087217 0.218602
1 -4.908040 -0.243170 -0.997024
rd_1m5caC/rd_1m5caC_3_092.xyz
6 -2.029493 0.717745 0.186156
6 0.221984 0.911063 0.740791
6 0.501096 -0.439806 0.264700

6 -0.582414 -1.174699 -0.241033
1 -0.451830 -2.193245 -0.589972
7 -1.787697 -0.639416 -0.308801
7 -1.003680 1.427910 0.694523
8 -3.186290 1.129185 0.099848
7 1.214947 1.642685 1.238827
1 1.027689 2.588831 1.551493
1 2.174781 1.319499 1.204568
6 1.845552 -1.095714 0.359341
8 2.848013 -0.526520 -0.138481
8 1.807657 -2.185962 0.991448
6 -2.936772 -1.384188 -0.844627
1 -3.709754 -1.442223 -0.077096
1 -3.329563 -0.850304 -1.711480
1 -2.605037 -2.380876 -1.128913
8 2.349458 1.444094 -2.090803
1 2.734782 1.084330 -2.901985
1 2.523528 0.762949 -1.406523
rd_1m5caC/rd_1m5caC_3_029.xyz
6 -2.031729 -0.618463 -0.203762
6 0.137889 -1.442781 -0.363571
6 0.681123 -0.272158 0.320039
6 -0.237753 0.702947 0.741239
1 0.097105 1.598699 1.253307
7 -1.529504 0.557198 0.514637
7 -1.164225 -1.563270 -0.612189
8 -3.248141 -0.662767 -0.383750
7 0.963095 -2.406650 -0.760874
1 0.586183 -3.219929 -1.234848
1 1.946773 -2.391362 -0.518079
6 2.146446 -0.042053 0.553056
8 2.772932 -0.938701 1.167947
8 2.600545 1.041985 0.095367
6 -2.508902 1.570954 0.929459
1 -3.028250 1.942153 0.044739
1 -3.231451 1.107094 1.602756

1 -1.983415 2.380558 1.432084
8 1.043867 2.774459 -1.535939
1 1.641944 3.179040 -2.179854
1 1.609489 2.182311 -0.998880
rd_1m5caC/rd_1m5caCacc.xyz
6 -2.512416 0.305276 -0.150058
6 -0.444119 1.366462 -0.077974
6 0.250748 0.098461 0.130510
6 -0.542734 -1.058698 0.204466
1 -0.092110 -2.033506 0.358705
7 -1.855132 -0.984749 0.091069
7 -1.766247 1.423728 -0.222206
8 -3.736401 0.272360 -0.267595
7 0.264800 2.489244 -0.137346
1 -0.210215 3.372911 -0.282458
1 1.259140 2.486772 0.061642
6 1.743055 -0.035186 0.228342
8 2.352036 0.739865 1.003845
8 2.233458 -0.932157 -0.514437
6 -2.711190 -2.175216 0.186916
1 -3.401997 -2.042300 1.021199
1 -3.280656 -2.277948 -0.737731
1 -2.081828 -3.048221 0.347500
8 5.055793 -0.921266 -0.450467
1 5.290215 -0.318413 0.269600
1 4.074349 -0.913868 -0.463061
rd_1m5caC/rd_1m5caC_3_084.xyz
6 -1.994925 -0.679299 -0.188635
6 0.197924 -1.439913 -0.345413
6 0.706978 -0.248433 0.327305
6 -0.241009 0.695431 0.754452
1 0.066554 1.603368 1.262290
7 -1.528184 0.508170 0.533622
7 -1.100301 -1.600517 -0.592235
8 -3.208741 -0.755577 -0.375533
7 1.050376 -2.382671 -0.735658

1 0.695717 -3.209487 -1.203402
1 2.034830 -2.335788 -0.500290
6 2.165613 0.043856 0.526435
8 2.838932 -0.807024 1.156232
8 2.565952 1.130380 0.026598
6 -2.534233 1.498338 0.941038
1 -3.042737 1.871639 0.050675
1 -3.261772 1.013706 1.593739
1 -2.033552 2.310240 1.464923
8 0.953364 2.780557 -1.669159
1 1.492047 2.212652 -1.081518
1 0.097532 2.852225 -1.222708
rd_1m5caC/rd_1m5caCabr.xyz
6 -2.296756 -0.058388 -0.048595
6 -0.607875 1.540437 -0.076575
6 0.426795 0.516808 0.039521
6 -0.003744 -0.818013 0.113547
1 0.708576 -1.632022 0.194204
7 -1.289742 -1.113808 0.093199
7 -1.901001 1.226552 -0.122982
8 -3.468723 -0.431469 -0.087486
7 -0.247235 2.818207 -0.148072
1 -0.958873 3.535364 -0.231715
1 0.721822 3.093661 -0.035719
6 1.897898 0.809758 0.042421
8 2.329844 1.693188 0.817515
8 2.557241 0.118819 -0.788116
6 -1.765678 -2.499501 0.197856
1 -2.432884 -2.577771 1.057561
1 -2.314758 -2.756276 -0.709639
1 -0.905496 -3.154597 0.320861
8 3.133494 -2.595799 -0.077739
1 3.949230 -2.588574 0.442690
1 2.979550 -1.660964 -0.315693
rd_1m5caC/rd_1m5caCadu.xyz
6 -2.509930 0.312605 -0.153974

6 -0.438488 1.366338 -0.072117
6 0.252137 0.095383 0.134252
6 -0.546252 -1.058607 0.210162
1 -0.099598 -2.034761 0.367058
7 -1.857887 -0.979493 0.094703
7 -1.759812 1.428392 -0.223143
8 -3.733224 0.282520 -0.278410
7 0.275372 2.486178 -0.121557
1 -0.194217 3.373187 -0.263948
1 1.268170 2.474581 0.086708
6 1.744835 -0.046422 0.221692
8 2.370838 0.751188 0.960227
8 2.219167 -0.973889 -0.494225
6 -2.718410 -2.166516 0.191498
1 -3.411413 -2.028601 1.023161
1 -3.285235 -2.270159 -0.734672
1 -2.092561 -3.041119 0.356918
8 5.042315 -0.923063 -0.434959
1 5.253925 -0.187418 0.157561
1 4.061099 -0.941997 -0.452950
rd_1m5caC/rd_1m5caC_3_071.xyz
6 -2.152514 -0.894842 -0.028750
6 0.175943 -1.001208 -0.108253
6 0.287689 0.450681 -0.009047
6 -0.909839 1.174886 0.085450
1 -0.904843 2.257386 0.153618
7 -2.078140 0.557875 0.106301
7 -1.014126 -1.604739 -0.124851
8 -3.284270 -1.384072 -0.042835
7 1.270765 -1.750376 -0.187109
1 1.154497 -2.756100 -0.251393
1 2.227586 -1.384682 -0.130848
6 1.580737 1.210002 -0.054391
8 2.343427 1.178662 0.940088
8 1.752036 1.815965 -1.145376
6 -3.340433 1.298085 0.238652

1 -3.947388 1.126377 -0.651756
1 -3.873053 0.928304 1.116027
1 -3.114026 2.357191 0.348257
8 4.075916 -0.952919 0.095453
1 3.821106 -0.122464 0.537739
1 4.440256 -1.512051 0.797361
rd_1m5caC/rd_1m5caC_3_039.xyz
6 1.990524 -0.692637 0.193199
6 -0.200154 -1.462193 0.320710
6 -0.710270 -0.259178 -0.331910
6 0.240094 0.686140 -0.753961
1 -0.066170 1.597401 -1.256220
7 1.526195 0.495515 -0.532858
7 1.096329 -1.621225 0.578371
8 3.201934 -0.761171 0.396297
7 -1.053608 -2.415985 0.677791
1 -0.704668 -3.251731 1.133774
1 -2.031296 -2.361652 0.413682
6 -2.170519 0.052866 -0.495690
8 -2.903839 -0.833247 -0.997425
8 -2.513197 1.196705 -0.085854
6 2.533925 1.487111 -0.931900
1 3.033026 1.862446 -0.037017
1 3.268559 1.002292 -1.576500
1 2.036763 2.297194 -1.461870
8 -0.926250 2.852743 1.619178
1 -1.451324 2.277811 1.026170
1 -0.038085 2.855218 1.234605
rd_1m5caC/rd_1m5caC_3_075.xyz
6 -1.920753 0.702240 0.088740
6 0.320108 1.318502 0.156126
6 0.730320 0.063899 -0.463024
6 -0.286941 -0.842786 -0.801082
1 -0.053934 -1.803293 -1.248426
7 -1.554338 -0.547166 -0.579014
7 -0.957583 1.587535 0.411998

8 -3.120122 0.866934 0.310777
7 1.250586 2.210997 0.483969
1 0.974732 3.083581 0.920346
1 2.226101 2.068075 0.250175
6 2.162710 -0.356813 -0.599686
8 2.918167 0.304966 -1.345874
8 2.450742 -1.362107 0.109976
6 -2.637066 -1.483791 -0.911435
1 -3.132747 -1.791658 0.010921
1 -3.356046 -0.976193 -1.555786
1 -2.208617 -2.344359 -1.421444
8 0.621717 -1.659532 2.436121
1 1.249205 -1.618442 1.690702
1 -0.043642 -0.986864 2.229021
rd_1m5caC/rd_1m5caCadb.xyz
6 -1.437656 0.310730 -0.038524
6 0.410372 -1.097601 -0.029123
6 1.327918 0.034910 -0.045360
6 0.755169 1.314877 -0.092469
1 1.375548 2.203671 -0.116213
7 -0.556816 1.469077 -0.118460
7 -0.910541 -0.924990 -0.005135
8 -2.648747 0.552674 -0.012300
7 0.903034 -2.331942 -0.036790
1 0.268599 -3.122911 -0.031809
1 1.898794 -2.495012 -0.137925
6 2.815683 -0.093028 0.055471
8 3.431668 -0.862812 -0.715532
8 3.292855 0.613408 0.990959
6 -1.178248 2.799648 -0.201773
1 -1.777477 2.851568 -1.112295
1 -1.824853 2.944322 0.664682
1 -0.390125 3.550402 -0.218388
8 -4.554164 -1.528968 0.208103
1 -3.846526 -0.855691 0.138654
1 -4.086021 -2.372984 0.276810

rd_1m5caC/rd_1m5caC_3_012.xyz
6 1.559314 0.708402 0.012622
6 -0.089612 -0.946366 -0.002543
6 -1.150504 0.052862 -0.043277
6 -0.755695 1.398651 -0.076564
1 -1.492607 2.194238 -0.107363
7 0.523224 1.732147 -0.083722
7 1.194188 -0.589898 0.046088
8 2.723568 1.101954 0.052672
7 -0.401390 -2.237471 -0.011416
1 0.338420 -2.931455 0.013373
1 -1.362226 -2.544115 -0.111963
6 -2.609818 -0.277598 0.008540
8 -3.085835 -1.097626 -0.809158
8 -3.211516 0.326094 0.942366
6 0.961220 3.132499 -0.170253
1 1.513976 3.390166 0.734729
1 1.615426 3.241182 -1.036508
1 0.083006 3.766956 -0.273602
8 3.134005 -2.722832 0.219695
1 2.530274 -1.951915 0.141211
1 3.590374 -2.769932 -0.632288
rd_1m5caC/rd_1m5caC_3_081.xyz
6 2.296129 0.051770 0.046129
6 0.602255 -1.541834 0.074536
6 -0.429456 -0.514556 -0.036003
6 0.005077 0.819141 -0.107137
1 -0.705315 1.635515 -0.183062
7 1.292094 1.110765 -0.089715
7 1.896466 -1.232097 0.118284
8 3.469440 0.420867 0.082511
7 0.237675 -2.818647 0.143472
1 0.947157 -3.538381 0.222893
1 -0.732524 -3.090771 0.033006
6 -1.901420 -0.803079 -0.037768
8 -2.337086 -1.681962 -0.815949

8 -2.557661 -0.113501 0.796372
6 1.772059 2.495235 -0.192486
1 2.324789 2.748267 0.713826
1 2.436735 2.573469 -1.054151
1 0.913508 3.153259 -0.311075
8 -3.129907 2.602305 0.082016
1 -2.970120 1.669154 0.322548
1 -3.824026 2.567591 -0.591493
rd_1m5caU/rd_1m5caUadm.xyz
6 1.537050 0.655017 -0.003758
6 -0.229064 -1.051026 0.010170
6 -1.210262 0.037407 -0.030512
6 -0.768640 1.361399 -0.010804
1 -1.472640 2.187066 -0.004076
7 0.527399 1.668923 -0.018733
7 1.091314 -0.637953 0.013275
8 2.717651 0.965976 -0.002953
8 -0.530805 -2.245055 0.035765
6 -2.670037 -0.276703 -0.028968
8 -3.138053 -0.203208 1.146494
8 -3.276858 -0.561749 -1.077139
1 1.837122 -1.367560 0.033683
6 0.996805 3.064142 -0.062429
1 1.585073 3.271211 0.832817
1 1.620225 3.197894 -0.947435
1 0.128799 3.717995 -0.106374
8 3.203181 -2.467217 0.101157
1 3.986248 -1.895542 0.081531
1 3.248135 -2.979254 -0.720728
rd_1m5caU/rd_1m5caUacj.xyz
6 -1.538372 0.657913 0.005902
6 0.226439 -1.049093 -0.011228
6 1.208436 0.038605 0.030382
6 0.767699 1.362902 0.008583
1 1.472331 2.188006 -0.000021
7 -0.528110 1.671295 0.016428

7 -1.093477 -0.635134 -0.013541
8 -2.718933 0.969123 0.010580
8 0.527286 -2.243389 -0.038300
6 2.667980 -0.276680 0.031464
8 3.138574 -0.205561 -1.143019
8 3.272309 -0.560519 1.081484
1 -1.840061 -1.364374 -0.035434
6 -0.996359 3.067011 0.054794
1 -1.587841 3.269635 -0.839334
1 -1.616436 3.206191 0.941289
1 -0.127736 3.720433 0.091995
8 -3.204014 -2.456813 -0.113303
1 -3.973099 -1.940333 0.173893
1 -3.112757 -3.159373 0.548498
rd_1m5caU/rd_1m5caUadk.xyz
6 2.032752 -0.551439 -0.210593
6 -0.329530 -0.921345 -0.830867
6 -0.620320 0.405410 -0.282569
6 0.422285 1.194712 0.205545
1 0.235776 2.198419 0.573263
7 1.676210 0.748746 0.262787
7 1.004704 -1.293317 -0.734790
8 3.184124 -0.943435 -0.142965
8 -1.170316 -1.667105 -1.327302
6 -2.021129 0.923403 -0.293576
8 -2.891099 0.500406 0.489634
8 -2.141024 1.799614 -1.200962
1 1.259120 -2.215341 -1.086723
6 2.768105 1.561678 0.829606
1 3.169662 1.048657 1.704848
1 3.551603 1.675782 0.079407
1 2.363849 2.532014 1.108939
8 -1.898451 -1.477551 2.302265
1 -2.261786 -0.815052 1.679379
1 -1.263463 -1.982426 1.774277
rd_1m5caU/rd_1m5caUacp.xyz

6 2.026895 0.592336 -0.201010
6 -0.349627 0.866930 -0.813110
6 -0.591000 -0.458852 -0.238666
6 0.482523 -1.205065 0.251655
1 0.336424 -2.209970 0.633820
7 1.720389 -0.714198 0.290970
7 0.970653 1.287454 -0.733675
8 3.163068 1.027634 -0.143871
8 -1.219375 1.573246 -1.317345
6 -1.974817 -1.021028 -0.231338
8 -2.072882 -1.920093 -1.118360
8 -2.854701 -0.607199 0.546218
1 1.189616 2.211545 -1.103262
6 2.848058 -1.484815 0.846827
1 3.627549 -1.567626 0.088410
1 3.239312 -0.957659 1.718410
1 2.484938 -2.470259 1.129123
8 -2.050767 1.683837 2.039451
1 -1.747258 2.294451 1.352428
1 -2.342799 0.890283 1.545120
rd_1m5caU/rd_1m5caUaat.xyz
6 -2.029931 -0.565781 -0.209448
6 0.337547 -0.892021 -0.832761
6 0.609089 0.430299 -0.264234
6 -0.445801 1.199289 0.230451
1 -0.275088 2.201243 0.610459
7 -1.693438 0.734768 0.278158
7 -0.990815 -1.285048 -0.743379
8 -3.175026 -0.976295 -0.146327
8 1.189320 -1.617354 -1.340569
6 2.003934 0.963907 -0.262450
8 2.118664 1.857105 -1.153465
8 2.875804 0.535154 0.515904
1 -1.231127 -2.205232 -1.109602
6 -2.799590 1.527746 0.845594
1 -3.583991 1.629453 0.094584

1 -3.193752 1.006463 1.719393
1 -2.412730 2.504397 1.127115
8 1.950456 -1.577589 2.194540
1 1.475287 -2.170841 1.595380
1 2.285934 -0.862323 1.615633
rd_1m5caU/rd_1m5caUaed.xyz
6 2.039631 0.569461 -0.199498
6 -0.318601 0.891672 -0.861003
6 -0.600734 -0.422472 -0.279028
6 0.446457 -1.187964 0.237205
1 0.269188 -2.186240 0.623973
7 1.694112 -0.725322 0.297328
7 1.008680 1.284866 -0.753971
8 3.185064 0.977546 -0.126978
8 -1.159626 1.607843 -1.398434
6 -1.995898 -0.954977 -0.291670
8 -2.103144 -1.854165 -1.176985
8 -2.875709 -0.519196 0.474272
1 1.255921 2.199240 -1.129953
6 2.791736 -1.517737 0.881965
1 3.571136 -1.651907 0.130587
1 3.197445 -0.978199 1.739047
1 2.392078 -2.480498 1.192293
8 -1.933337 1.601346 2.122351
1 -2.298455 0.868001 1.585145
1 -2.388383 1.537907 2.973783
rd_1m5caU/rd_1m5caUacm.xyz
6 1.557341 -1.096924 -0.026264
6 -0.803960 -0.375785 -0.005621
6 -0.325993 1.004604 -0.052631
6 1.045883 1.256388 -0.020999
1 1.424917 2.272246 -0.006243
7 1.946886 0.273747 -0.022470
7 0.202946 -1.327972 -0.009017
8 2.390975 -1.985561 -0.038843
8 -1.993940 -0.695175 0.028020

6 -1.302011 2.134057 -0.043794
8 -1.324254 2.646671 1.120418
8 -1.965948 2.465340 -1.037180
1 -0.078115 -2.308567 0.005519
6 3.395997 0.546289 -0.022880
1 3.845736 0.064178 0.845907
1 3.833618 0.140192 -0.936060
1 3.542585 1.623140 0.021940
8 -2.541141 -3.539841 -0.006890
1 -2.462267 -2.568898 0.042906
1 -3.084368 -3.785951 0.755377
rd_1m5caU/rd_1m5caUaeb.xyz
6 -2.033420 -0.668683 -0.162670
6 0.355947 -0.846287 -0.764949
6 0.538252 0.492812 -0.200896
6 -0.568922 1.195245 0.279513
1 -0.468666 2.209884 0.649515
7 -1.784665 0.653494 0.320436
7 -0.945285 -1.322940 -0.684270
8 -3.150474 -1.151357 -0.108919
8 1.255912 -1.518973 -1.262594
6 1.887705 1.132794 -0.218303
8 1.891960 2.065519 -1.077979
8 2.825056 0.752639 0.505832
1 -1.123588 -2.257559 -1.048834
6 -2.948071 1.386812 0.854043
1 -3.724396 1.417942 0.088633
1 -3.322385 0.863284 1.735195
1 -2.628674 2.393172 1.115003
8 2.467210 -1.760334 1.795915
1 2.076493 -2.308522 1.100181
1 2.594612 -0.888190 1.368672
rd_1m5caU/rd_1m5caUadd.xyz
6 -2.093833 -0.444533 -0.207587
6 0.155447 -1.444209 -0.409252
6 0.676932 -0.271898 0.298442

6 -0.202404 0.722908 0.731295
1 0.160953 1.589580 1.273650
7 -1.509492 0.665421 0.480823
7 -1.219969 -1.427167 -0.597861
8 -3.293248 -0.482685 -0.415025
8 0.848520 -2.371362 -0.820742
6 2.138758 -0.174913 0.591187
8 2.912495 0.471627 -0.141494
8 2.413417 -0.809165 1.649609
1 -1.633893 -2.216038 -1.093193
6 -2.433840 1.737698 0.892099
1 -2.934580 2.128975 0.005556
1 -3.173222 1.320371 1.577517
1 -1.857965 2.520095 1.381530
8 1.657356 2.556315 -1.660078
1 2.073230 1.823879 -1.162411
1 0.717026 2.497200 -1.438637
rd_1m5caU/rd_1m5caUaaq.xyz
6 -2.509601 -0.410272 0.053788
6 -0.223992 -1.338604 -0.068001
6 0.275303 0.036824 -0.001562
6 -0.630352 1.099676 0.013950
1 -0.285809 2.127914 0.005944
7 -1.947150 0.903395 0.055436
7 -1.607091 -1.441583 -0.016074
8 -3.716132 -0.570013 0.104835
8 0.492725 -2.333063 -0.154363
6 1.746098 0.289882 -0.057867
8 2.053997 0.683378 -1.222804
8 2.496479 0.102470 0.916420
1 -2.008794 -2.377800 -0.043218
6 -2.902028 2.025863 0.100428
1 -3.580667 1.942999 -0.749386
1 -3.469471 1.970252 1.030727
1 -2.339594 2.955603 0.052899
8 5.234834 0.368648 0.275460

1 4.279247 0.294915 0.485113
1 5.256983 0.621846 -0.658403
rd_1m5caU/rd_1m5caUaam.xyz
6 2.097791 0.445147 0.184031
6 -0.131363 1.485278 0.402231
6 -0.684048 0.310195 -0.277032
6 0.171656 -0.714821 -0.691047
1 -0.214650 -1.588805 -1.203962
7 1.483635 -0.669872 -0.471450
7 1.246138 1.447705 0.571584
8 3.301269 0.468869 0.366629
8 -0.801369 2.431735 0.807468
6 -2.153035 0.220450 -0.533120
8 -2.513447 0.908803 -1.524635
8 -2.866092 -0.502362 0.197963
1 1.681053 2.239621 1.043797
6 2.384628 -1.761564 -0.883647
1 3.117221 -1.363932 -1.587877
1 2.895800 -2.147417 -0.000615
1 1.788668 -2.541899 -1.352053
8 -1.443377 -2.631125 1.500842
1 -1.942970 -3.440798 1.324038
1 -1.963170 -1.917064 1.081107
rd_1m5caU/rd_1m5caUacy.xyz
6 2.509201 0.413108 0.064611
6 0.222420 1.337063 -0.068246
6 -0.273876 -0.040131 -0.016165
6 0.633929 -1.100994 -0.001179
1 0.291661 -2.129913 -0.018262
7 1.949864 -0.901977 0.051469
7 1.604878 1.442866 -0.005249
8 3.714870 0.575058 0.127238
8 -0.496312 2.330276 -0.151850
6 -1.743953 -0.295772 -0.077951
8 -2.055037 -0.660899 -1.250752
8 -2.490932 -0.136814 0.904243

1 2.004553 2.380213 -0.021171
6 2.906661 -2.022779 0.096811
1 3.590067 -1.934893 -0.748686
1 3.468641 -1.970247 1.030589
1 2.346178 -2.953288 0.041853
8 -5.238777 -0.383386 0.316025
1 -4.276176 -0.319409 0.495033
1 -5.304904 -0.395534 -0.649422
rd_1m5caU/rd_1m5caUaah.xyz
6 1.448673 0.439187 -0.040300
6 -0.438509 -1.151055 -0.016430
6 -1.334790 0.004627 -0.047522
6 -0.799939 1.292021 -0.016253
1 -1.443931 2.164913 0.006461
7 0.516127 1.512081 -0.030830
7 0.911144 -0.820662 -0.026266
8 2.650722 0.662167 -0.055362
8 -0.811197 -2.321849 0.011604
6 -2.812671 -0.202696 -0.017242
8 -3.218286 -0.034770 1.176802
8 -3.481574 -0.497368 -1.018329
1 1.581737 -1.589309 -0.017725
6 1.081247 2.874396 -0.051228
1 1.722284 3.004291 0.821677
1 1.670570 2.996871 -0.961074
1 0.258829 3.585981 -0.030888
8 4.244236 -1.770964 -0.013487
1 3.819069 -0.894176 -0.007340
1 4.465261 -1.945121 0.912591
rd_1m5caU/rd_1m5caUaby.xyz
6 -2.146931 0.806614 0.001049
6 0.301371 0.984916 -0.280042
6 0.362583 -0.473834 -0.187446
6 -0.808574 -1.194625 0.052880
1 -0.783569 -2.272230 0.174787
7 -2.001738 -0.605795 0.129514

7 -0.979478 1.505300 -0.187885
8 -3.244875 1.331265 0.062743
8 1.274906 1.723618 -0.434259
6 1.676555 -1.180267 -0.268799
8 2.059671 -1.470428 0.908543
8 2.257750 -1.408040 -1.339654
1 -1.078656 2.516865 -0.262394
6 -3.241841 -1.376505 0.334888
1 -3.732898 -1.018401 1.240634
1 -3.897534 -1.224518 -0.523613
1 -2.980341 -2.427600 0.434197
8 3.862200 1.206277 0.806007
1 3.774212 0.278281 1.070734
1 3.031098 1.391748 0.332026
rd_1m5caU/rd_1m5caUaaw.xyz
6 -1.323591 0.515377 -0.478349
6 0.989376 1.253401 -0.029842
6 1.375979 -0.158607 -0.021401
6 0.398021 -1.140203 -0.178566
1 0.651079 -2.193750 -0.130816
7 -0.880549 -0.835976 -0.409889
7 -0.360326 1.468844 -0.282846
8 -2.496770 0.780066 -0.694520
8 1.762178 2.190203 0.157391
6 2.796370 -0.538476 0.238618
8 2.906488 -0.899126 1.452989
8 3.683623 -0.481582 -0.625863
1 -0.681943 2.435413 -0.310496
6 -1.905763 -1.875396 -0.619506
1 -2.666538 -1.786970 0.157102
1 -2.361396 -1.729435 -1.599785
1 -1.423617 -2.849012 -0.565402
8 -4.587141 -0.041699 1.200568
1 -3.888752 0.190581 0.564376
1 -5.072087 0.783626 1.343924
rd_1m5caU/rd_1m5caUaca.xyz

6 2.162004 -0.828080 -0.006330
6 -0.299637 -0.984301 0.140522
6 -0.344923 0.473434 0.020199
6 0.849903 1.190959 -0.062854
1 0.846102 2.274542 -0.114666
7 2.038553 0.589306 -0.100039
7 0.979384 -1.516707 0.108501
8 3.255913 -1.364178 -0.027099
8 -1.283900 -1.715433 0.256269
6 -1.653789 1.192288 0.076759
8 -1.878337 1.541673 1.276347
8 -2.365003 1.384305 -0.921924
1 1.064698 -2.529689 0.180389
6 3.294356 1.349483 -0.234256
1 3.921425 1.151642 0.636308
1 3.807060 1.022079 -1.139775
1 3.050658 2.407828 -0.296025
8 -4.064505 -1.170632 -0.356918
1 -4.037546 -0.239337 -0.624490
1 -3.138795 -1.363825 -0.118604
rd_1m5caU/rd_1m5caUadp.xyz
6 -1.267093 0.674308 0.035892
6 1.135785 1.248034 0.030037
6 1.417596 -0.185458 -0.045747
6 0.361741 -1.095589 -0.019129
1 0.547637 -2.164311 -0.027894
7 -0.913596 -0.704416 0.001314
7 -0.218756 1.557544 0.047675
8 -2.436490 1.025813 0.059263
8 1.994237 2.126492 0.071640
6 2.831682 -0.663095 -0.069930
8 3.154386 -1.060470 1.093948
8 3.533990 -0.641870 -1.091807
1 -0.472831 2.543750 0.088383
6 -2.022614 -1.677006 -0.006909
1 -2.618420 -1.541224 0.896373

1 -2.639934 -1.502105 -0.888791
1 -1.597555 -2.677771 -0.035707
8 -5.102256 -0.142029 -0.101685
1 -4.993020 -1.103664 -0.112026
1 -4.192935 0.202772 -0.059417
rd_1m5caU/rd_1m5caUabz.xyz
6 2.083929 -0.474144 -0.180922
6 -0.162591 -1.476490 -0.401673
6 -0.694665 -0.296176 0.285116
6 0.178658 0.710026 0.706898
1 -0.193660 1.587918 1.224698
7 1.488809 0.647579 0.480786
7 1.215680 -1.461666 -0.569987
8 3.286337 -0.515158 -0.367336
8 -0.848743 -2.408035 -0.814062
6 -2.162017 -0.177351 0.538219
8 -2.532474 -0.871031 1.523458
8 -2.861458 0.567980 -0.181646
1 1.636621 -2.257233 -1.048630
6 2.406295 1.727138 0.887719
1 3.136510 1.321134 1.589612
1 2.918978 2.104306 0.001777
1 1.822915 2.516526 1.356645
8 -1.470554 2.657009 -1.589136
1 -1.933902 1.934863 -1.119837
1 -0.801423 2.966953 -0.962225
rd_1m5caU/rd_1m5caUadl.xyz
6 2.133208 -0.875233 -0.011390
6 -0.330620 -0.940477 -0.172803
6 -0.317622 0.521262 -0.137585
6 0.895989 1.190345 0.024240
1 0.930388 2.271403 0.103696
7 2.061482 0.545143 0.075815
7 0.925859 -1.521901 -0.120200
8 3.206179 -1.452242 0.017770
8 -1.345982 -1.633950 -0.247812

6 -1.600874 1.284081 -0.178957
8 -1.900396 1.635390 1.006136
8 -2.231137 1.501297 -1.223761
1 0.973174 -2.539057 -0.161280
6 3.341434 1.262684 0.220009
1 3.847144 0.901129 1.116200
1 3.959734 1.066350 -0.657160
1 3.128276 2.326036 0.305487
8 -4.088389 -1.124655 0.499580
1 -3.160536 -1.225538 0.216907
1 -4.440853 -2.025706 0.510467
rd_1m5caU/rd_1m5caUacd.xyz
6 -2.104034 -0.428675 -0.199599
6 0.126626 -1.468864 -0.399772
6 0.673035 -0.293392 0.283061
6 -0.186023 0.727478 0.697904
1 0.195069 1.597941 1.220863
7 -1.496305 0.683547 0.464890
7 -1.249293 -1.431433 -0.580657
8 -3.305770 -0.451812 -0.394875
8 0.800886 -2.415482 -0.798099
6 2.138570 -0.214072 0.561953
8 2.900529 0.433131 -0.185864
8 2.434921 -0.850233 1.611047
1 -1.680090 -2.222283 -1.058329
6 -2.401926 1.772120 0.875473
1 -2.907514 2.161684 -0.009048
1 -3.138707 1.370303 1.572942
1 -1.810551 2.551137 1.351789
8 1.560645 2.539138 -1.588050
1 1.913934 3.353258 -1.202067
1 2.039881 1.817641 -1.133180
rd_1m5dhmC/rd_1m5dhmC_4_034_b.xyz
6 -2.145995 0.306115 0.194348
6 -0.139987 1.488108 0.414241
6 0.609047 0.403226 -0.172803

6 -0.123094 -0.668472 -0.648829
1 0.354945 -1.498747 -1.153025
7 -1.449188 -0.740694 -0.477067
7 -1.459394 1.397043 0.604795
8 -3.371549 0.170478 0.361185
6 2.102262 0.435920 -0.397302
1 2.633964 1.073365 0.321682
7 0.469555 2.612794 0.814571
1 -0.090464 3.362612 1.199421
1 1.443119 2.798071 0.619930
8 2.092577 0.963694 -1.667756
8 2.633542 -0.884139 -0.325477
1 3.601117 -0.822474 -0.352976
6 -2.220325 -1.898799 -0.950612
1 -2.715659 -2.380382 -0.106152
1 -2.972081 -1.565336 -1.668011
1 -1.536130 -2.599101 -1.427557
8 1.608925 -2.334580 1.905278
1 2.159391 -3.124458 2.000514
1 1.995573 -1.847751 1.149979
rd_1m5dhmC/rd_1m5dhmC_4_026.xyz
6 -1.502401 -0.781851 -0.022567
6 -0.060256 1.072597 0.044956
6 1.098036 0.215059 0.103131
6 0.865987 -1.144746 0.016440
1 1.684236 -1.854274 0.008040
7 -0.376456 -1.648107 -0.031674
7 -1.293865 0.556129 0.008600
8 -2.637422 -1.287049 -0.048011
6 2.523717 0.717038 0.108967
1 2.623402 1.734627 0.512543
7 0.047393 2.406172 0.047410
1 -0.793319 2.968177 -0.001904
1 0.941072 2.875918 0.019448
8 2.737794 0.680102 -1.254499
8 3.350410 -0.163640 0.850958

1 4.248693 0.202307 0.848455
6 -0.619393 -3.098360 -0.066410
1 -1.190745 -3.393785 0.815233
1 -1.183681 -3.353026 -0.964841
1 0.341248 -3.611068 -0.073652
8 -3.688077 2.071018 0.120301
1 -4.392361 1.407081 0.128421
1 -2.853837 1.542820 0.080813
rd_1m5dhmC/rd_1m5dhmC_4_090.xyz
6 1.958775 0.425967 -0.349055
6 -0.213369 1.246498 -0.614023
6 -0.775617 -0.076528 -0.466088
6 0.115205 -1.099863 -0.186641
1 -0.229897 -2.110701 -0.007210
7 1.435046 -0.888226 -0.154245
7 1.106638 1.447998 -0.590853
8 3.192106 0.569267 -0.286694
6 -2.255471 -0.361988 -0.397125
1 -2.870037 0.400892 -0.895354
7 -1.000613 2.312309 -0.802180
1 -0.577469 3.226847 -0.894906
1 -2.008074 2.245954 -0.800867
8 -2.372685 -0.311216 0.976098
8 -2.539961 -1.632222 -0.958377
1 -3.499154 -1.767272 -0.910775
6 2.384264 -1.976366 0.120452
1 3.079471 -2.070353 -0.715326
1 2.942016 -1.750451 1.030819
1 1.825599 -2.902836 0.244403
8 -0.306086 0.683667 2.846539
1 0.473074 0.822058 2.288451
1 -0.992737 0.379010 2.226076
rd_1m5dhmC/rd_1m5dhmC_3_083.xyz
6 -2.123874 -0.383483 0.162370
6 -0.080011 -1.498829 0.365684
6 0.640747 -0.361646 -0.158975

6 -0.127001 0.688105 -0.626801
1 0.316256 1.555854 -1.100405
7 -1.459280 0.700738 -0.482117
7 -1.403080 -1.461284 0.545971
8 -3.353299 -0.291321 0.328482
6 2.159447 -0.337013 -0.295579
1 2.649262 -0.926035 0.490801
7 0.563232 -2.620201 0.720613
1 0.021484 -3.409030 1.049965
1 1.535011 -2.776150 0.494733
8 2.750836 0.952849 -0.204371
1 2.603663 1.429081 -1.039193
8 2.223237 -0.896473 -1.543052
6 -2.269692 1.840253 -0.936258
1 -3.013914 1.491557 -1.654057
1 -2.775403 2.294527 -0.082774
1 -1.611105 2.568195 -1.408101
8 1.465488 2.446690 1.877205
1 1.922873 1.948395 1.170731
1 0.557980 2.110551 1.858274
rd_1m5dhmC/rd_1m5dhmC_4_078.xyz
6 -2.540827 -0.234864 -0.172732
6 -0.526008 -1.419012 -0.084918
6 0.235362 -0.197184 0.014076
6 -0.490491 0.972985 0.120310
1 0.001269 1.928157 0.255796
7 -1.829208 0.981135 0.027876
7 -1.856748 -1.401177 -0.205678
8 -3.777912 -0.169485 -0.299523
6 1.743557 -0.146435 0.129824
1 2.233055 -1.014293 -0.335557
7 0.077706 -2.615902 -0.087111
1 -0.492230 -3.450402 -0.138539
1 1.065924 -2.723166 0.089749
8 1.818335 -0.195390 1.512446
8 2.258493 1.039470 -0.434325

1 3.239340 0.937435 -0.475063
6 -2.600785 2.230512 0.113430
1 -3.122667 2.407449 -0.828634
1 -3.331767 2.150441 0.919406
1 -1.915295 3.052515 0.316484
8 4.955176 0.528513 -0.584103
1 5.510625 1.269411 -0.298215
1 5.111899 -0.166809 0.073061
rd_1m5dhmC/rd_1m5dhmC_4_034.xyz
6 -2.144031 0.314247 0.198346
6 -0.128920 1.482089 0.410074
6 0.610123 0.391961 -0.179862
6 -0.131247 -0.674745 -0.652543
1 0.339256 -1.508565 -1.158087
7 -1.457171 -0.737898 -0.475072
7 -1.448091 1.400281 0.606020
8 -3.369895 0.187649 0.369736
6 2.102636 0.415219 -0.410108
1 2.641109 1.047635 0.308441
7 0.490445 2.602379 0.807582
1 -0.062126 3.356402 1.194959
1 1.464765 2.779937 0.609585
8 2.091717 0.945927 -1.679245
8 2.625125 -0.908583 -0.343066
1 3.592764 -0.853333 -0.380096
6 -2.238376 -1.890664 -0.945533
1 -2.735468 -2.367439 -0.099420
1 -2.989067 -1.552219 -1.661738
1 -1.560573 -2.596706 -1.423192
8 1.586992 -2.323958 1.903460
1 2.327199 -2.750556 2.357496
1 1.993231 -1.845418 1.153015
rd_1m5dhmC/rd_1m5dhmC_4_043.xyz
6 -1.458356 0.367870 -0.112162
6 0.260822 -1.211077 -0.174627
6 1.265562 -0.176465 -0.117312

6 0.809234 1.122048 0.003810
1 1.496535 1.954238 0.092691
7 -0.500554 1.410757 -0.013469
7 -1.043465 -0.912928 -0.185689
8 -2.663826 0.696144 -0.122043
6 2.751089 -0.438676 -0.027320
1 3.044343 -1.413937 -0.439468
7 0.596546 -2.505198 -0.243351
1 -0.136666 -3.201325 -0.286184
1 1.554998 -2.821050 -0.211278
8 2.860508 -0.408217 1.348608
8 3.472438 0.586715 -0.689538
1 4.416326 0.373905 -0.621173
6 -0.984493 2.795289 0.086599
1 -1.538765 3.057095 -0.816334
1 -1.640931 2.888002 0.953029
1 -0.125990 3.455712 0.198201
8 -4.679753 -1.178788 0.172689
1 -3.915392 -0.567227 0.068457
1 -4.300225 -1.994597 0.527957
rd_1m5dhmC/rd_1m5dhmC_4_086.xyz
6 1.979535 0.406518 -0.350614
6 -0.197352 1.163432 -0.735258
6 -0.743586 -0.150004 -0.478672
6 0.154105 -1.126771 -0.079292
1 -0.181045 -2.122381 0.183650
7 1.468290 -0.888553 -0.028207
7 1.118432 1.389052 -0.698535
8 3.210590 0.568972 -0.291030
6 -2.220348 -0.453670 -0.423223
1 -2.831604 0.242415 -1.013866
7 -0.995995 2.191861 -1.046181
1 -0.584977 3.102341 -1.207266
1 -2.002692 2.113153 -1.043568
8 -2.378026 -0.263664 0.934036
8 -2.469964 -1.780389 -0.855571

1 -3.427900 -1.927026 -0.816803
6 2.426132 -1.937382 0.352147
1 3.124677 -2.109398 -0.468253
1 2.979906 -1.618723 1.236617
1 1.874952 -2.851440 0.569423
8 -0.420481 1.072211 2.680198
1 -0.915290 1.452571 3.419586
1 -1.098970 0.682176 2.099354
rd_1m5dhmC/rd_1m5dhmC_4_016.xyz
6 -1.461366 0.348676 0.077097
6 0.268614 -1.217519 0.022404
6 1.257627 -0.174903 -0.105026
6 0.799783 1.124880 -0.010932
1 1.481601 1.966128 -0.028727
7 -0.509752 1.401513 0.077968
7 -1.037900 -0.931626 0.066006
8 -2.668859 0.668538 0.101263
6 2.746032 -0.426155 -0.186874
1 2.991471 -1.417062 -0.596101
7 0.623510 -2.507081 0.080022
1 -0.095114 -3.212569 0.179401
1 1.589783 -2.798491 0.119222
8 3.026883 -0.337239 1.160500
8 3.368031 0.573568 -0.977098
1 4.315375 0.369154 -1.017509
6 -1.002243 2.784757 0.164473
1 -1.628806 3.007238 -0.700572
1 -1.590814 2.899974 1.075884
1 -0.146749 3.458278 0.186904
8 -4.705087 -1.165543 -0.261185
1 -3.928218 -0.574125 -0.134566
1 -4.337967 -1.991178 -0.606586
rd_1m5dhmC/rd_1m5dhmC_4_073.xyz
6 2.546579 0.209572 -0.162890
6 0.550842 1.426961 -0.085044
6 -0.231058 0.217575 0.002890

6 0.474792 -0.964320 0.113924
1 -0.033883 -1.911294 0.243677
7 1.813790 -0.994632 0.032380
7 1.881932 1.387063 -0.196426
8 3.783043 0.124168 -0.284316
6 -1.740765 0.188528 0.103612
1 -2.213729 1.064930 -0.363113
7 -0.032522 2.634059 -0.087507
1 0.553156 3.458355 -0.125698
1 -1.018856 2.757708 0.089394
8 -1.829340 0.234064 1.485408
8 -2.264012 -0.988693 -0.470383
1 -3.247888 -0.901980 -0.472388
6 2.562379 -2.257714 0.122147
1 3.084977 -2.445272 -0.817489
1 3.291218 -2.190082 0.931190
1 1.860746 -3.066463 0.322923
8 -4.991248 -0.679586 -0.492875
1 -5.296633 -0.554651 0.418539
1 -5.185673 0.160088 -0.936674
rd_1m5dhmC/rd_1m5dhmC_4_023.xyz
6 2.312673 0.199387 -0.006773
6 0.756160 -1.544652 -0.006650
6 -0.340221 -0.621554 0.171463
6 -0.024972 0.724196 0.132382
1 -0.786583 1.491580 0.191648
7 1.245331 1.142113 0.049714
7 2.022238 -1.120727 -0.052934
8 3.474744 0.643271 -0.025757
6 -1.786114 -1.053676 0.251980
1 -1.909539 -2.068680 0.653518
7 0.539049 -2.863485 -0.103882
1 1.327650 -3.479753 -0.253261
1 -0.390004 -3.252262 -0.177122
8 -2.079035 -0.973052 -1.089018
8 -2.536263 -0.152156 1.062431

1 -3.401080 -0.557091 1.234437
6 1.579474 2.574078 0.049409
1 2.195111 2.808488 0.919250
1 2.132468 2.819604 -0.858684
1 0.653459 3.146417 0.085223
8 -3.309369 2.202799 -0.383787
1 -2.782282 2.172586 -1.195063
1 -3.068146 1.380227 0.087959
rd_1m5dhmC/rd_1m5dhmC_4_020.xyz
6 -2.555262 0.157469 0.162485
6 -0.596880 1.435091 0.100076
6 0.220816 0.250510 0.002119
6 -0.448669 -0.950796 -0.123186
1 0.087581 -1.881113 -0.262121
7 -1.786121 -1.021886 -0.043958
7 -1.926482 1.354158 0.208515
8 -3.788748 0.032668 0.282051
6 1.730717 0.266813 -0.094324
1 2.176368 1.150859 0.384137
7 -0.049359 2.658913 0.115574
1 -0.660261 3.464534 0.158464
1 0.932292 2.813132 -0.064449
8 1.821665 0.330506 -1.475181
8 2.284079 -0.902157 0.468097
1 3.266867 -0.803898 0.458007
6 -2.494412 -2.306772 -0.144807
1 -3.009940 -2.519693 0.793393
1 -3.225617 -2.255218 -0.952907
1 -1.767299 -3.090475 -0.352810
8 5.009983 -0.574175 0.449164
1 5.353219 -0.849404 -0.414540
1 5.426840 -1.175653 1.084685
rd_1m5dhmC/rd_1m5dhmC_4_066.xyz
6 1.393598 -0.542874 -0.463020
6 -0.742173 -1.352917 0.028928
6 -1.269926 -0.013420 0.129804

6 -0.396311 1.017273 -0.161341
1 -0.722580 2.049646 -0.157860
7 0.896568 0.787421 -0.434721
7 0.552070 -1.570972 -0.233187
8 2.608018 -0.703574 -0.710988
6 -2.725757 0.311649 0.377934
1 -3.263148 -0.480491 0.917263
7 -1.518392 -2.427700 0.214591
1 -1.107080 -3.348557 0.132324
1 -2.511119 -2.359671 0.384811
8 -3.121483 0.396186 -0.941987
8 -2.842536 1.527815 1.097321
1 -3.785848 1.685678 1.259649
6 1.830021 1.887024 -0.723419
1 2.635516 1.884736 0.012073
1 2.251194 1.751371 -1.720955
1 1.284584 2.828367 -0.676056
8 4.514577 0.119842 1.215377
1 3.841382 -0.120077 0.546432
1 4.060065 0.025189 2.064461
rd_1m5dhmC/rd_1m5dhmC_4_005.xyz
6 -1.452148 0.341958 -0.104392
6 0.282703 -1.219684 -0.162208
6 1.277135 -0.174794 -0.113798
6 0.808258 1.119898 -0.000015
1 1.487484 1.959454 0.081849
7 -0.504454 1.395145 -0.015925
7 -1.024583 -0.934989 -0.172142
8 -2.660772 0.658508 -0.111576
6 2.765341 -0.421882 -0.027027
1 3.066196 -1.399502 -0.427872
7 0.632714 -2.510337 -0.223316
1 -0.092310 -3.215339 -0.258400
1 1.594920 -2.814490 -0.191759
8 2.881589 -0.372856 1.347728
8 3.473547 0.601718 -0.706311

1 4.419558 0.396173 -0.645915
6 -1.001961 2.775347 0.077482
1 -1.567084 3.024178 -0.822318
1 -1.651303 2.868473 0.949225
1 -0.149352 3.445494 0.175346
8 -4.636161 -1.271944 0.177097
1 -5.451614 -0.760421 0.273138
1 -3.923827 -0.601220 0.070636
rd_1m5dhmC/rd_1m5dhmC_4_004.xyz
6 2.545816 0.216953 -0.153056
6 0.544550 1.426051 -0.087431
6 -0.232988 0.213259 -0.007846
6 0.476903 -0.965935 0.106806
1 -0.028902 -1.915215 0.230863
7 1.816620 -0.990539 0.035413
7 1.876577 1.391737 -0.189245
8 3.783366 0.136803 -0.266483
6 -1.743366 0.177204 0.080557
1 -2.215939 1.053654 -0.386524
7 -0.043345 2.630948 -0.091649
1 0.539463 3.457557 -0.123263
1 -1.031696 2.750529 0.076323
8 -1.844823 0.216885 1.461356
8 -2.256056 -1.000191 -0.503059
1 -3.240568 -0.923699 -0.491161
6 2.569980 -2.250475 0.128560
1 3.102054 -2.432947 -0.806729
1 3.291124 -2.181818 0.944378
1 1.870257 -3.062973 0.320779
8 -4.979138 -0.677703 -0.385211
1 -5.102032 0.002663 0.294625
1 -5.324877 -0.281461 -1.199292
rd_1m5dhmC/rd_1m5dhmC_4_006.xyz
6 -2.219395 -0.168711 0.105735
6 -0.369473 -1.596896 0.171362
6 0.504482 -0.553174 -0.313557

6 -0.060651 0.700579 -0.478838
1 0.534801 1.550014 -0.789792
7 -1.368191 0.907895 -0.289889
7 -1.678694 -1.386788 0.334955
8 -3.432474 0.073653 0.230444
6 1.993299 -0.720735 -0.498795
1 2.288986 -1.758135 -0.706616
7 0.102376 -2.819147 0.446827
1 -0.534092 -3.522996 0.798615
1 1.087004 -3.039872 0.406256
8 2.370654 -0.318989 0.766439
8 2.456788 0.121034 -1.539690
1 3.413639 -0.014149 -1.623307
6 -1.974917 2.230400 -0.499741
1 -2.706917 2.172466 -1.307139
1 -2.474927 2.548407 0.416331
1 -1.188109 2.936957 -0.759565
8 2.265064 2.453420 1.402620
1 1.357817 2.719178 1.195414
1 2.313034 1.512681 1.131052
rd_1m5dhmC/rd_1m5dhmC_4_084.xyz
6 2.553530 0.157827 -0.175417
6 0.594721 1.433902 -0.099828
6 -0.221210 0.249034 0.009383
6 0.450342 -0.951359 0.131399
1 -0.083764 -1.881764 0.277806
7 1.787125 -1.021590 0.040308
7 1.923376 1.353864 -0.219632
8 3.786176 0.033819 -0.304291
6 -1.730234 0.265839 0.120480
1 -2.181064 1.144475 -0.363087
7 0.045680 2.657049 -0.114454
1 0.655034 3.463339 -0.165776
1 -0.934262 2.810430 0.075427
8 -1.806508 0.343964 1.501542
8 -2.288891 -0.909211 -0.423381

1 -3.271119 -0.804994 -0.423929
6 2.497420 -2.305654 0.138365
1 3.005031 -2.520485 -0.803711
1 3.235516 -2.251377 0.940011
1 1.772834 -3.089482 0.354634
8 -5.012100 -0.576983 -0.453957
1 -5.429533 -1.181628 0.178245
1 -5.358770 -0.844017 -1.318757
rd_1m5dhmC/rd_1m5dhmC_4_076.xyz
6 -2.112785 -0.389554 -0.160461
6 -0.057993 -1.485749 -0.375399
6 0.647166 -0.354520 0.177910
6 -0.127862 0.693367 0.638787
1 0.318544 1.554816 1.119498
7 -1.457983 0.702239 0.481098
7 -1.381719 -1.456990 -0.554763
8 -3.344431 -0.312044 -0.318688
6 2.142492 -0.315026 0.387766
1 2.692157 -0.961631 -0.309374
7 0.596317 -2.592600 -0.753707
1 0.066632 -3.374983 -1.115957
1 1.580716 -2.726938 -0.572861
8 2.166974 -0.788577 1.679247
8 2.620890 1.020528 0.254434
1 3.590261 0.996468 0.279834
6 -2.278079 1.834287 0.936634
1 -2.793434 2.280667 0.084882
1 -3.014457 1.479975 1.659876
1 -1.624693 2.570658 1.402731
8 1.454606 2.304946 -2.015402
1 0.526767 2.043800 -1.926271
1 1.899259 1.872979 -1.258909
rd_1m5dhmC/rd_1m5dhmC_4_071.xyz
6 1.977124 0.393737 -0.340727
6 -0.200359 1.137833 -0.750483
6 -0.745329 -0.169083 -0.459266

6 0.155085 -1.139529 -0.051454
1 -0.178075 -2.131293 0.228190
7 1.470011 -0.901048 -0.012529
7 1.115600 1.365806 -0.716855
8 3.206258 0.564822 -0.265955
6 -2.221378 -0.473029 -0.382809
1 -2.841219 0.219763 -0.967956
7 -0.998239 2.157813 -1.089384
1 -0.586334 3.064250 -1.270065
1 -2.005034 2.081566 -1.085858
8 -2.363789 -0.278359 0.975643
8 -2.476027 -1.801700 -0.805845
1 -3.433453 -1.948463 -0.755571
6 2.430358 -1.944721 0.375907
1 3.134463 -2.114255 -0.440237
1 2.977668 -1.623067 1.263365
1 1.882078 -2.861227 0.590132
8 -0.557089 1.279477 2.663394
1 0.284993 1.197612 2.192316
1 -1.194527 0.811958 2.092162
rd_1m5dhmC/rd_1m5dhmC_4_015.xyz
6 1.320608 0.668842 -0.084099
6 -0.913302 1.353751 -0.092825
6 -1.372739 -0.014328 -0.106341
6 -0.401232 -0.991232 -0.006573
1 -0.660389 -2.041398 0.040734
7 0.905251 -0.685482 0.001834
7 0.391791 1.647535 -0.112035
8 2.545913 0.905368 -0.124621
6 -2.828867 -0.421154 -0.083955
1 -3.495975 0.332380 -0.526417
7 -1.774224 2.379274 -0.085392
1 -1.410997 3.323811 -0.073715
1 -2.772836 2.249814 -0.012964
8 -2.977350 -0.504783 1.285550
8 -3.002404 -1.654238 -0.762038

1 -3.946384 -1.875460 -0.732651
6 1.935134 -1.734066 0.053405
1 2.528001 -1.710108 -0.862237
1 2.584067 -1.563675 0.912996
1 1.440565 -2.699418 0.151551
8 5.038207 -0.369258 0.133395
1 5.653321 0.375725 0.085136
1 4.148366 0.031445 0.050753
rd_1m5dhmU/rd_1m5dhmU_a1_8_abg_a.xyz
6 -2.100183 0.815523 0.103436
6 0.208784 0.942545 0.478081
6 0.323670 -0.527856 0.347552
6 -0.824112 -1.248123 -0.018120
1 -0.788448 -2.318531 -0.196168
7 -1.982779 -0.633333 -0.162636
7 -1.001683 1.525566 0.393825
8 -3.243698 1.271429 0.024385
8 1.256619 1.618414 0.676963
6 1.636325 -1.274982 0.489829
1 1.443203 -2.302678 0.803704
8 2.485033 -0.702204 1.454255
1 2.462456 0.267966 1.320660
6 -3.192970 -1.353154 -0.577324
1 -3.938906 -1.285847 0.216372
1 -3.585543 -0.882365 -1.480071
1 -2.932406 -2.392310 -0.769634
8 2.223520 -1.358622 -0.795539
1 2.445795 -0.448876 -1.126096
8 2.869363 1.171496 -1.615974
1 2.376565 1.568577 -0.870861
1 2.372730 1.420619 -2.409682
rd_1m5dhmU/rd_1m5dhmU_a1_8_aaf.xyz
6 -2.105158 0.826716 -0.087251
6 0.203679 0.945077 -0.464655
6 0.310880 -0.527381 -0.350067
6 -0.840138 -1.245428 0.009678

1 -0.809776 -2.317758 0.176668
7 -1.995152 -0.625835 0.163065
7 -1.003294 1.533899 -0.371187
8 -3.246272 1.287539 -0.002221
8 1.254563 1.617030 -0.660195
6 1.620939 -1.278146 -0.496049
1 1.425037 -2.303164 -0.816869
8 2.204987 -1.371588 0.790162
1 2.433037 -0.464749 1.124536
6 -3.208881 -1.343909 0.570148
1 -3.602136 -0.878291 1.475172
1 -3.952806 -1.268475 -0.224786
1 -2.952629 -2.385421 0.755507
8 2.473553 -0.701500 -1.454725
1 2.452536 0.267832 -1.314540
8 2.807910 1.151158 1.669205
1 3.757428 1.316127 1.570636
1 2.392586 1.554767 0.881893
rd_1m5dhmU/rd_1m5dhmU_a1_8_aal.xyz
6 -1.322715 0.669426 -0.118806
6 0.973434 1.108379 -0.036701
6 1.262873 -0.343309 0.001046
6 0.189356 -1.240815 -0.038625
1 0.342986 -2.315299 -0.020654
7 -1.051219 -0.787376 -0.102805
7 -0.307028 1.533897 -0.089628
8 -2.518840 0.980038 -0.163627
8 1.945961 1.902981 -0.016245
6 2.689107 -0.837097 0.085623
1 2.713650 -1.926920 0.114392
8 3.287640 -0.388998 1.284226
1 3.254468 0.584934 1.297855
6 -2.201111 -1.698106 -0.147370
1 -2.814483 -1.534036 0.740521
1 -2.793183 -1.477257 -1.036791
1 -1.838011 -2.723874 -0.173444

8 3.409214 -0.449945 -1.066014
1 3.379006 0.521606 -1.132856
8 -5.177750 0.028423 0.258794
1 -4.248065 0.286128 0.112320
1 -5.604094 0.828213 0.597613
rd_1m5dhmU/rd_1m5dhmU_a1_8_aas.xyz
6 1.517789 0.201750 0.117555
6 -0.495966 -0.985340 0.109604
6 -1.254647 0.279929 -0.020053
6 -0.544105 1.485553 -0.059900
1 -1.048677 2.442444 -0.150356
7 0.776083 1.478516 0.013928
7 0.853066 -0.952795 0.173134
8 2.749173 0.325912 0.145957
8 -1.144959 -2.059271 0.154420
6 -2.763674 0.262483 -0.108140
1 -3.150068 1.276508 -0.217336
8 -3.315220 -0.250564 1.086631
1 -2.960075 -1.146413 1.229719
6 1.559701 2.718429 -0.033906
1 2.203948 2.763392 0.845196
1 2.180252 2.707748 -0.932264
1 0.878210 3.567198 -0.051680
8 -3.176649 -0.449464 -1.255827
1 -2.816694 -1.353328 -1.201532
8 4.420155 -1.931634 -0.140718
1 3.818285 -1.164362 -0.044643
1 5.116182 -1.629885 -0.741208
rd_1m5dhmU/rd_1m5dhmU_a1_8_aao.xyz
6 -1.763399 -0.309225 0.097069
6 0.297509 0.825305 0.093581
6 1.016140 -0.464670 -0.015976
6 0.268375 -1.646869 -0.079172
1 0.745756 -2.617668 -0.170811
7 -1.052517 -1.602281 -0.028423
7 -1.052381 0.824387 0.156582

8 -2.991892 -0.383130 0.139054
8 0.969746 1.883501 0.126387
6 2.526696 -0.495176 -0.061378
1 2.883351 -1.520562 -0.161944
8 3.057414 -0.002239 1.151430
1 2.737143 0.908538 1.281169
6 -1.872828 -2.816128 -0.109089
1 -2.504451 -2.876243 0.778503
1 -2.507040 -2.751516 -0.995307
1 -1.218328 -3.683539 -0.171737
8 2.996583 0.207188 -1.192516
1 2.665864 1.122625 -1.142118
8 -2.433482 3.284290 -0.177904
1 -1.736205 3.955247 -0.194601
1 -1.953713 2.432079 -0.042084
rd_1m5dhmU/rd_1m5dhmU_a1_8_abp.xyz
6 2.048583 -0.744046 -0.292708
6 -0.194261 -1.414455 -0.355747
6 -0.587607 -0.212911 0.416424
6 0.411509 0.691767 0.803114
1 0.180798 1.592406 1.363081
7 1.671821 0.460955 0.486559
7 1.102719 -1.611206 -0.668586
8 3.253551 -0.846366 -0.536836
8 -1.096102 -2.219382 -0.705550
6 -2.038824 0.111420 0.697150
1 -2.116674 0.849180 1.496735
8 -2.774188 -1.017378 1.088274
1 -2.528407 -1.742741 0.475444
6 2.741032 1.392875 0.859656
1 3.479214 0.861511 1.462967
1 3.221850 1.759689 -0.048883
1 2.307097 2.216286 1.423674
8 -2.581596 0.749822 -0.459067
1 -2.548651 0.138615 -1.215819
8 -0.898521 3.019046 -1.119682

1 -1.520856 2.288614 -0.941169
1 -1.403907 3.654365 -1.646291
rd_1m5dhmU/rd_1m5dhmU_a1_8_abz.xyz
6 -1.711664 -0.341736 0.094598
6 0.308930 0.863734 0.104259
6 1.071619 -0.400471 -0.007888
6 0.365285 -1.608363 -0.063910
1 0.876927 -2.561754 -0.150675
7 -0.956220 -1.610670 -0.012406
7 -1.040466 0.815908 0.158965
8 -2.937768 -0.455936 0.118101
8 0.944640 1.943762 0.144799
6 2.581793 -0.377908 -0.071984
1 2.973056 -1.391190 -0.169634
8 3.113134 0.143421 1.128113
1 2.758354 1.041182 1.258948
6 -1.732091 -2.854260 -0.086022
1 -2.383217 -2.917349 0.786982
1 -2.345491 -2.831948 -0.989059
1 -1.045478 -3.698563 -0.113153
8 3.009681 0.329166 -1.217112
1 2.648413 1.233059 -1.172425
8 -2.761290 3.049797 -0.171487
1 -2.133072 2.300392 -0.034542
1 -3.623101 2.621848 -0.275990
rd_1m5dhmU/rd_1m5dhmU_a1_8_adn.xyz
6 -1.321877 0.676089 -0.092199
6 0.975098 1.113735 -0.012588
6 1.265645 -0.337697 -0.039126
6 0.191294 -1.234327 -0.080365
1 0.345992 -2.308788 -0.095243
7 -1.050291 -0.780818 -0.105827
7 -0.306002 1.539720 -0.046226
8 -2.518144 0.987315 -0.127729
8 1.946575 1.907950 0.044445
6 2.690863 -0.838535 0.008451

1 2.715927 -1.922921 -0.103451
8 3.249891 -0.558055 1.277179
1 3.224178 0.403723 1.425471
6 -2.200322 -1.691229 -0.148577
1 -2.806158 -1.534420 0.745659
1 -2.799703 -1.463870 -1.031526
1 -1.837190 -2.716703 -0.186240
8 3.447125 -0.305573 -1.056006
1 3.390023 0.666749 -1.007898
8 -5.183531 0.012355 0.218278
1 -5.626354 0.804841 0.553075
1 -4.252196 0.278920 0.101620
rd_1m5dhmU/rd_1m5dhmU_a1_8_acy.xyz
6 1.717574 1.066353 -0.007209
6 -0.541800 0.468372 -0.041363
6 -0.179656 -0.965635 -0.013551
6 1.177135 -1.311581 0.001872
1 1.504141 -2.346306 0.014765
7 2.099943 -0.365453 0.000977
7 0.422075 1.407337 -0.032291
8 2.664339 1.855940 0.008742
8 -1.768405 0.755586 -0.075476
6 -1.256065 -2.026907 0.007318
1 -0.810630 -3.022187 0.005015
8 -2.053840 -1.940934 -1.154245
1 -2.434180 -1.044331 -1.196760
6 3.531341 -0.686154 0.014601
1 3.997605 -0.257537 -0.874233
1 3.983957 -0.244576 0.904002
1 3.649985 -1.768131 0.023421
8 -2.006298 -1.929670 1.200980
1 -2.409934 -1.044275 1.244541
8 -2.767346 3.347499 0.056763
1 -2.328733 2.469397 0.005212
1 -2.045112 3.990707 0.029118
rd_1m5dhmU/rd_1m5dhmU_a1_8_aah.xyz

6 -1.711891 -0.351619 -0.083011
6 0.295796 0.875792 -0.154299
6 1.075281 -0.381535 -0.084669
6 0.381142 -1.597589 -0.030500
1 0.906016 -2.546578 0.015579
7 -0.940364 -1.614134 -0.036424
7 -1.054478 0.813217 -0.157016
8 -2.936695 -0.477934 -0.053141
8 0.913091 1.966637 -0.207914
6 2.586252 -0.362239 -0.007098
1 2.985171 -1.365894 -0.161339
8 2.972883 0.012494 1.304741
1 2.655589 0.913185 1.491891
6 -1.700215 -2.868435 0.019291
1 -2.306264 -2.871898 0.927157
1 -2.357942 -2.920941 -0.849945
1 -1.002438 -3.703948 0.022246
8 3.145359 0.460993 -1.002009
1 2.695683 1.327151 -0.949476
8 -2.769353 3.036834 0.261547
1 -2.145151 2.291219 0.090024
1 -3.627234 2.605684 0.383871
rd_1m5dhmU/rd_1m5dhmU_a1_10_aaw.xyz
6 2.074835 0.887870 0.006065
6 -0.210612 0.880585 0.526700
6 -0.269104 -0.582032 0.291650
6 0.924856 -1.258226 0.002302
1 0.951606 -2.335105 -0.131409
7 2.056167 -0.589496 -0.119648
7 0.953809 1.534620 0.342144
8 3.169234 1.413868 -0.209392
8 -1.258340 1.481591 0.880854
6 -1.571580 -1.353808 0.297843
1 -1.375079 -2.414586 0.478513
8 -2.120124 -1.182113 -1.005484
1 -2.739933 -1.910143 -1.176344

6 3.325125 -1.269157 -0.401838
1 3.708893 -0.925708 -1.363955
1 4.040353 -1.010468 0.380761
1 3.148118 -2.342955 -0.421157
8 -2.444617 -0.926319 1.311526
1 -2.439111 0.054712 1.295115
8 -3.156050 1.470025 -1.353046
1 -2.623753 1.853220 -0.636363
1 -2.902868 0.527360 -1.314631
rd_1m5dhmU/rd_1m5dhmU_f2_12_aap.xyz
6 -1.735172 1.015308 0.146682
6 0.547084 0.499485 0.107565
6 0.234560 -0.931637 -0.103128
6 -1.108358 -1.309726 -0.230219
1 -1.396467 -2.340659 -0.410220
7 -2.064497 -0.404123 -0.131122
7 -0.454051 1.389924 0.242815
8 -2.709386 1.761689 0.263415
8 1.758205 0.841539 0.157245
6 1.322946 -1.979225 -0.132915
1 0.923731 -2.924306 -0.509715
8 1.758432 -2.118752 1.208123
1 2.097113 -3.019964 1.331811
6 -3.480867 -0.761215 -0.261814
1 -3.994718 -0.518326 0.670008
1 -3.917514 -0.174921 -1.072223
1 -3.556942 -1.826243 -0.474299
8 2.368279 -1.615375 -1.004552
1 2.657314 -0.719072 -0.741089
8 2.586171 3.476008 -0.194159
1 1.833980 4.081509 -0.136473
1 2.208565 2.582569 -0.037251
rd_1m5dhmU/rd_1m5dhmU_f2_12_aat.xyz
6 2.497720 0.251237 -0.315501
6 0.445881 1.373175 -0.148638
6 -0.210661 0.125973 0.309028

6 0.573832 -1.023267 0.467391
1 0.146820 -1.958184 0.817270
7 1.865029 -0.991076 0.188968
7 1.762656 1.359024 -0.452238
8 3.701541 0.152026 -0.572241
8 -0.247748 2.416733 -0.244563
6 -1.701739 0.060924 0.536839
1 -1.958954 -0.849286 1.086505
8 -2.290178 0.052001 -0.745575
1 -3.099147 -0.521780 -0.715929
6 2.719861 -2.172190 0.349528
1 3.156798 -2.427276 -0.617366
1 3.522639 -1.932368 1.049051
1 2.117832 -2.996808 0.726521
8 -2.152045 1.138326 1.330793
1 -1.814762 1.954192 0.909650
8 -4.456621 -1.566736 -0.604044
1 -5.265743 -1.048736 -0.473894
1 -4.588248 -2.029312 -1.445763
rd_1m5dhmU/rd_1m5dhmU_a1_8_aad.xyz
6 -1.754108 0.936937 -0.001590
6 0.542055 0.494497 -0.005829
6 0.277021 -0.961245 -0.002617
6 -1.053456 -1.398209 0.000571
1 -1.307606 -2.453761 0.002747
7 -2.039772 -0.518308 0.000692
7 -0.484601 1.363249 -0.004941
8 -2.751086 1.661509 -0.000450
8 1.745296 0.870707 -0.009786
6 1.424089 -1.945771 -0.000897
1 1.050435 -2.970025 -0.004254
8 2.184681 -1.800246 1.180483
1 2.509741 -0.882807 1.225459
6 -3.447294 -0.934973 0.003298
1 -3.933241 -0.529768 0.892536
1 -3.935816 -0.531354 -0.885253

1 -3.492789 -2.022967 0.004349
8 2.194577 -1.795365 -1.174895
1 2.515641 -0.876079 -1.214380
8 2.242581 3.621779 0.015411
1 1.388129 4.072664 -0.039314
1 2.017887 2.665013 0.004134
rd_1m5dhmU/rd_1m5dhmU_a1_10_acw.xyz
6 -2.215314 -0.467500 -0.284526
6 -0.108774 -1.495346 -0.253095
6 0.482427 -0.287292 0.369002
6 -0.357656 0.797777 0.652589
1 0.020489 1.702705 1.118766
7 -1.645675 0.735030 0.364790
7 -1.426829 -1.514649 -0.549605
8 -3.423179 -0.398458 -0.533049
8 0.638065 -2.480743 -0.481954
6 1.972852 -0.153922 0.579343
1 2.193122 0.661140 1.274670
8 2.505488 0.134725 -0.707839
1 2.458082 1.117338 -0.841465
6 -2.560093 1.840628 0.670095
1 -3.017979 2.186574 -0.258144
1 -3.342488 1.478133 1.339082
1 -1.997300 2.643902 1.141966
8 2.561428 -1.315373 1.098245
1 2.199978 -2.066370 0.583110
8 2.192687 2.825672 -0.940515
1 2.664233 3.253427 -0.209462
1 1.252824 2.947831 -0.734373
rd_1m5dhmU/rd_1m5dhmU_f2_12_adi.xyz
6 2.499445 0.258849 -0.312925
6 0.443125 1.371083 -0.138315
6 -0.209001 0.117160 0.307444
6 0.580255 -1.029696 0.459122
1 0.157024 -1.969244 0.801173
7 1.871782 -0.989278 0.183348

7 1.760072 1.364821 -0.441352
8 3.703528 0.165877 -0.570695
8 -0.254609 2.412708 -0.225093
6 -1.700627 0.043906 0.528497
1 -1.956752 -0.872227 1.068889
8 -2.282666 0.044307 -0.756860
1 -3.093261 -0.527716 -0.734034
6 2.732352 -2.166801 0.338581
1 3.172102 -2.414369 -0.628989
1 3.532814 -1.926622 1.040642
1 2.134140 -2.996594 0.710121
8 -2.158997 1.112171 1.330259
1 -1.822962 1.933238 0.918213
8 -4.458497 -1.568068 -0.643767
1 -5.101661 -1.177509 -0.032412
1 -4.907792 -1.580711 -1.502762
rd_1m5dhmU/rd_1m5dhmU_f2_12_aaa.xyz
6 2.494629 0.257316 -0.300933
6 0.443058 1.378292 -0.125795
6 -0.218029 0.123150 0.302909
6 0.564683 -1.028918 0.447761
1 0.134901 -1.969691 0.778113
7 1.857928 -0.992467 0.179208
7 1.761403 1.368189 -0.422182
8 3.699755 0.161400 -0.552621
8 -0.248895 2.424386 -0.205175
6 -1.712181 0.054116 0.506318
1 -1.980033 -0.866854 1.032957
8 -2.276418 0.070363 -0.787117
1 -3.078628 -0.514199 -0.784014
6 2.712052 -2.175506 0.329128
1 3.163029 -2.411391 -0.636133
1 3.504068 -1.948228 1.044925
1 2.105948 -3.008191 0.681138
8 -2.176588 1.116032 1.312657
1 -1.830616 1.939663 0.914007

8 -4.423499 -1.572674 -0.729036
1 -5.243867 -1.056597 -0.715605
1 -4.410244 -2.041315 0.119451
rd_1m5dhmU/rd_1m5dhmU_f2_12_aax.xyz
6 -1.702543 -0.378389 0.111302
6 0.278763 0.892376 0.011677
6 1.076259 -0.351774 -0.089201
6 0.400180 -1.576654 -0.159875
1 0.936179 -2.512733 -0.280836
7 -0.918103 -1.618953 -0.076723
7 -1.065538 0.798616 0.136006
8 -2.919501 -0.534285 0.225706
8 0.868033 1.997723 -0.014054
6 2.586656 -0.312114 -0.087748
1 2.992137 -1.292152 -0.353109
8 2.963207 0.046346 1.229276
1 3.855683 -0.295617 1.400292
6 -1.659390 -2.882681 -0.166272
1 -2.204055 -3.041756 0.766154
1 -2.374527 -2.810218 -0.987607
1 -0.954449 -3.693271 -0.341605
8 3.075282 0.591160 -1.053360
1 2.648340 1.452801 -0.878816
8 -2.857949 2.996396 -0.058561
1 -2.208698 2.257198 0.024762
1 -3.717248 2.554811 -0.115561
rd_1m5dhmU/rd_1m5dhmU_a1_8_aco.xyz
6 2.511813 0.586488 -0.080937
6 0.260213 1.213443 0.029611
6 -0.148410 -0.210184 0.035422
6 0.845610 -1.195820 0.005781
1 0.604881 -2.254337 0.025700
7 2.118465 -0.844733 -0.047119
7 1.568918 1.534498 -0.035605
8 3.727425 0.785018 -0.149457
8 -0.646124 2.083425 0.086295

6 -1.610384 -0.581752 0.126756
1 -1.744571 -1.657036 0.005893
8 -2.351604 0.036788 -0.904632
1 -2.189215 0.998786 -0.852096
6 3.190656 -1.844565 -0.074449
1 3.766597 -1.722300 -0.993347
1 3.846950 -1.678963 0.781661
1 2.745894 -2.836706 -0.029382
8 -2.095933 -0.261458 1.415406
1 -1.980115 0.693437 1.565491
8 -5.107652 -0.556074 -0.456512
1 -5.198758 -0.547718 0.506991
1 -4.169229 -0.330772 -0.613747
rd_1m5dhmU/rd_1m5dhmU_a1_10_ads.xyz
6 -2.172219 -0.540373 -0.295684
6 -0.024695 -1.481066 -0.301525
6 0.510753 -0.289167 0.397689
6 -0.372590 0.752339 0.713355
1 -0.033143 1.651297 1.219744
7 -1.652750 0.657882 0.401914
7 -1.339522 -1.539874 -0.606262
8 -3.382506 -0.513562 -0.540141
8 0.764891 -2.417771 -0.586586
6 1.992556 -0.112560 0.644851
1 2.170393 0.648400 1.410381
8 2.529554 0.300899 -0.606815
1 2.362522 1.273816 -0.717428
6 -2.608568 1.719312 0.734762
1 -3.086176 2.064303 -0.183654
1 -3.370446 1.310882 1.401360
1 -2.074875 2.535015 1.218980
8 2.625869 -1.287857 1.068909
1 2.292780 -2.007015 0.491684
8 1.922568 2.943130 -0.864052
1 2.092530 3.235634 -1.772554
1 0.957897 2.971992 -0.769286

rd_1m5fC/rd_1m5fC_3_074.xyz
6 1.134710 0.344540 -0.059500
6 -0.558332 -1.275069 -0.061589
6 -1.586563 -0.275524 0.007292
6 -1.167219 1.045902 0.016131
1 -1.875349 1.866158 0.055007
7 0.132412 1.357407 -0.028199
7 0.748049 -0.955866 -0.089155
8 2.324024 0.705807 -0.064545
7 -0.922002 -2.581061 -0.102601
1 -0.060520 -3.138432 -0.156228
6 -3.017850 -0.592111 0.059398
1 -3.279770 -1.658825 0.032792
8 -3.897125 0.268852 0.132339
6 0.585017 2.756062 -0.041074
1 1.199524 2.943472 0.841099
1 1.185135 2.927309 -0.936226
1 -0.286984 3.407030 -0.041079
8 4.398827 -1.140821 0.270203
1 4.820951 -1.249129 -0.593541
1 3.643413 -0.534449 0.109934
rd_1m5fC/rd_1m5fC_3_059.xyz
6 -2.354990 0.285428 0.004725
6 -0.301492 1.424727 -0.006291
6 0.441505 0.194675 -0.009681
6 -0.299795 -0.977217 -0.007946
1 0.179944 -1.949374 -0.010727
7 -1.636074 -0.950491 -0.001668
7 -1.643628 1.448717 0.000490
8 -3.591451 0.246884 0.013553
7 0.378450 2.600389 -0.009803
1 -0.318671 3.355208 -0.007176
6 1.900247 0.141488 -0.009382
1 2.425239 1.105525 -0.007664
8 2.541319 -0.916415 -0.010053
6 -2.434329 -2.185613 0.002478

1 -3.061593 -2.200963 0.895555
1 -3.070860 -2.201432 -0.884031
1 -1.762845 -3.042573 -0.000694
8 5.316326 -0.413613 0.110513
1 4.362558 -0.626624 0.031868
1 5.718562 -0.765859 -0.695794
rd_1m5fC/rd_1m5fC_3_038.xyz
6 1.142949 0.390209 -0.022546
6 -0.514092 -1.266881 -0.033383
6 -1.564994 -0.289569 0.003676
6 -1.174768 1.040581 0.006296
1 -1.900775 1.845632 0.024061
7 0.118187 1.380860 -0.016548
7 0.785077 -0.919002 -0.042372
8 2.324416 0.776100 -0.013599
7 -0.849653 -2.580591 -0.062235
1 0.023362 -3.121436 -0.090667
6 -2.989391 -0.637888 0.031883
1 -3.226032 -1.710766 0.015142
8 -3.889567 0.203286 0.073987
6 0.537835 2.789853 -0.030078
1 1.116667 3.002102 0.870641
1 1.163829 2.966444 -0.906084
1 -0.349208 3.419278 -0.066746
8 4.385552 -1.132249 0.088065
1 3.635830 -0.499889 0.050377
1 3.972605 -2.005165 0.028647
rd_1m5fC/rd_1m5fC_3_091.xyz
6 -0.976837 0.663800 -0.081188
6 1.294070 1.242356 0.001154
6 1.698394 -0.135027 0.015763
6 0.684429 -1.078738 -0.031059
1 0.897499 -2.141679 -0.028216
7 -0.600551 -0.711261 -0.082621
7 0.000433 1.607806 -0.045602
8 -2.185386 0.947093 -0.116549

7 2.252596 2.201451 0.037022
1 1.778658 3.112722 0.015503
6 3.099697 -0.564069 0.074474
1 3.851660 0.236776 0.103127
8 3.443175 -1.747914 0.091777
6 -1.678359 -1.710841 -0.127688
1 -2.284276 -1.622970 0.775745
1 -2.302809 -1.522858 -1.002125
1 -1.233226 -2.702094 -0.188841
8 -4.742331 -0.227396 0.198454
1 -5.290849 0.550451 0.371567
1 -3.836034 0.124517 0.093451
rd_1m5fC/rd_1m5fC_2_032.xyz
6 -2.341215 0.441530 -0.034487
6 -0.127558 1.245833 0.018349
6 0.399934 -0.100767 0.045659
6 -0.525108 -1.129108 0.048046
1 -0.221377 -2.170387 0.072420
7 -1.843163 -0.886588 0.017151
7 -1.447755 1.480271 -0.017631
8 -3.563961 0.619572 -0.090973
7 0.649717 2.359110 0.028069
1 1.636256 2.069212 0.049924
6 1.807175 -0.464460 0.058091
1 2.014691 -1.546559 0.075994
8 2.747976 0.340544 0.045517
6 -2.824560 -1.981897 0.012181
1 -3.496833 -1.863184 0.863455
1 -3.403297 -1.939736 -0.912472
1 -2.291741 -2.928791 0.081069
8 5.273100 -0.891843 -0.169070
1 5.851236 -0.467525 0.480596
1 4.410542 -0.435548 -0.074927
rd_1m5fC/rd_1m5fC_3_090.xyz
6 2.351819 0.291548 0.032689
6 0.294522 1.422527 -0.019657

6 -0.442977 0.189386 -0.046849
6 0.303172 -0.979418 -0.044598
1 -0.172032 -1.953564 -0.067867
7 1.638924 -0.947041 -0.009732
7 1.635980 1.452142 0.019842
8 3.587657 0.257643 0.077623
7 -0.390380 2.595237 -0.033480
1 0.302992 3.353234 -0.012953
6 -1.901367 0.130643 -0.057995
1 -2.429887 1.092745 -0.050923
8 -2.538750 -0.929452 -0.069115
6 2.442284 -2.178703 0.000655
1 3.095830 -2.186061 -0.873535
1 3.052493 -2.197104 0.905354
1 1.774724 -3.038438 -0.020612
8 -5.302516 -0.434984 0.204490
1 -5.717071 -0.536609 -0.663843
1 -4.354567 -0.638136 0.058499
rd_1m5fC/rd_1m5fC_3_002.xyz
6 -2.362654 0.253076 0.000006
6 -0.334571 1.437840 -0.000002
6 0.435414 0.224760 -0.000006
6 -0.280110 -0.963038 -0.000008
1 0.221710 -1.923820 -0.000012
7 -1.616466 -0.966434 -0.000006
7 -1.677060 1.431639 0.000005
8 -3.598090 0.187405 0.000016
7 0.318601 2.628485 -0.000007
1 -0.395638 3.367188 -0.000001
6 1.895214 0.200795 -0.000012
1 2.401994 1.174525 -0.000027
8 2.555157 -0.845244 -0.000002
6 -2.385457 -2.220213 -0.000004
1 -3.016915 -2.250599 0.889760
1 -3.016931 -2.250594 -0.889756
1 -1.692916 -3.060269 -0.000013

8 5.340664 -0.399327 0.000013
1 4.377184 -0.581480 0.000008
1 5.757122 -1.272763 0.000024
rd_1m5fC/rd_1m5fC_2_098.xyz
6 1.072105 0.490726 -0.593595
6 -1.189965 0.980553 -0.177995
6 -1.485027 -0.411704 0.069464
6 -0.434398 -1.300372 -0.063242
1 -0.566930 -2.366264 0.093845
7 0.798514 -0.884575 -0.390011
7 0.047775 1.387412 -0.503123
8 2.238993 0.834333 -0.845914
7 -2.116938 1.969983 -0.103481
1 -3.020827 1.548633 0.148647
6 -2.786362 -0.958828 0.445999
1 -2.808109 -2.054469 0.573893
8 -3.811907 -0.295721 0.623087
6 1.919125 -1.829458 -0.524554
1 2.350972 -1.728428 -1.521667
1 2.678364 -1.593939 0.222472
1 1.544656 -2.840850 -0.378166
8 3.825196 0.524707 1.571081
1 3.379310 0.621611 0.708888
1 4.745982 0.771906 1.405899
rd_1m5fC/rd_1m5fC_2_070.xyz
6 1.203989 -1.220449 -0.046597
6 -0.785312 0.036454 -0.054261
6 -0.052502 1.279448 0.008521
6 1.325904 1.179725 0.051567
1 1.958525 2.060230 0.102600
7 1.943716 -0.010507 0.027654
7 -0.163447 -1.152006 -0.082281
8 1.816791 -2.294768 -0.072567
7 -2.140260 -0.027640 -0.083867
1 -2.529497 0.924068 -0.071749
6 -0.640667 2.615526 0.021435

1 0.087696 3.442645 0.073119
8 -1.850347 2.857797 -0.024044
6 3.410367 -0.119454 0.067795
1 3.702739 -0.695139 0.947994
1 3.753863 -0.636436 -0.829992
1 3.835543 0.881378 0.114629
8 -3.597521 -2.490621 0.114403
1 -3.077305 -1.660008 0.027643
1 -2.933686 -3.192428 0.172123
rd_1m5fC/rd_1m5fC_2_013.xyz
6 -1.207284 -1.221828 -0.048811
6 0.782503 0.034093 -0.059546
6 0.050682 1.277311 0.009925
6 -1.327679 1.178285 0.055103
1 -1.959684 2.059004 0.109883
7 -1.946202 -0.011534 0.028228
7 0.160122 -1.154092 -0.088763
8 -1.820731 -2.295782 -0.074047
7 2.137153 -0.030232 -0.095684
1 2.526757 0.921304 -0.080971
6 0.639904 2.612889 0.026182
1 -0.087657 3.440395 0.082518
8 1.849677 2.854360 -0.021277
6 -3.412903 -0.119634 0.068964
1 -3.756894 -0.636321 -0.828802
1 -3.705430 -0.695233 0.949167
1 -3.837482 0.881444 0.115891
8 3.615193 -2.476773 0.104414
1 2.978486 -3.165144 0.343147
1 3.079931 -1.655580 0.019089
rd_1m5fC/rd_1m5fC_2_055.xyz
6 1.207429 1.237773 -0.037575
6 -0.771588 -0.034854 -0.044862
6 -0.028559 -1.272347 0.006146
6 1.349148 -1.161972 0.042099
1 1.989423 -2.037430 0.083885

7 1.957133 0.033412 0.023586
7 -0.159843 1.158884 -0.067119
8 1.811721 2.316952 -0.058694
7 -2.127099 0.018783 -0.069813
1 -2.509034 -0.935855 -0.060624
6 -0.605157 -2.613287 0.016178
1 0.130969 -3.434139 0.058059
8 -1.813025 -2.866024 -0.021059
6 3.422997 0.153948 0.057608
1 3.758560 0.678188 -0.839008
1 3.714791 0.727232 0.939540
1 3.856196 -0.843749 0.097200
8 -3.559503 2.499049 0.100008
1 -3.090550 1.637275 0.024204
1 -4.500858 2.275549 0.090560
rd_1m5fC/rd_1m5fC_2_011.xyz
6 2.337721 0.440561 0.059563
6 0.125617 1.246237 -0.023701
6 -0.401633 -0.099509 -0.077992
6 0.522914 -1.128250 -0.072919
1 0.219194 -2.169046 -0.114390
7 1.840017 -0.886819 -0.013154
7 1.444838 1.479710 0.041134
8 3.559560 0.617371 0.135867
7 -0.651400 2.359733 -0.034028
1 -1.637259 2.070128 -0.078802
6 -1.808264 -0.462227 -0.124204
1 -2.015762 -1.544127 -0.153863
8 -2.749104 0.342948 -0.126237
6 2.821036 -1.982339 -0.002930
1 3.500348 -1.862251 -0.848486
1 3.392269 -1.942233 0.926394
1 2.288545 -2.928947 -0.077839
8 -5.294641 -0.846201 0.089143
1 -5.483318 -0.859168 1.038026
1 -4.409063 -0.432504 0.014214

rd_1m5fC/rd_1m5fC_2_034.xyz
6 -1.044421 -0.512739 -0.512555
6 1.227163 -0.988689 -0.133914
6 1.525928 0.410346 0.065744
6 0.470236 1.293317 -0.063261
1 0.603954 2.363104 0.062792
7 -0.769182 0.867403 -0.351642
7 -0.017227 -1.405887 -0.416675
8 -2.213814 -0.866846 -0.735689
7 2.157048 -1.974747 -0.049592
1 3.070525 -1.542953 0.143032
6 2.836280 0.968762 0.390509
1 2.861595 2.068269 0.478124
8 3.866028 0.311284 0.563891
6 -1.894738 1.808382 -0.472679
1 -2.397016 1.643207 -1.426754
1 -2.599532 1.631476 0.341430
1 -1.508815 2.824790 -0.420829
8 -4.249740 -0.409179 1.224279
1 -4.016284 -1.025197 1.933045
1 -3.551394 -0.528429 0.551604
rd_1m5fC/rd_1m5fC_2_001.xyz
6 0.979824 -0.612723 -0.053935
6 -1.340496 -0.997022 -0.004379
6 -1.613387 0.421685 0.008059
6 -0.517108 1.262685 -0.009944
1 -0.626950 2.342632 -0.001904
7 0.736062 0.782520 -0.040171
7 -0.084470 -1.468580 -0.038096
8 2.151634 -1.020207 -0.081177
7 -2.309342 -1.948682 0.018756
1 -3.224099 -1.479071 0.048874
6 -2.936234 1.039870 0.036787
1 -2.930489 2.142924 0.041781
8 -4.007737 0.427554 0.053752
6 1.902848 1.679350 -0.059802

1 2.507778 1.500299 0.830602
1 2.499005 1.472846 -0.949959
1 1.554561 2.710673 -0.072631
8 4.831570 -0.119587 0.137784
1 5.286040 -0.971153 0.068653
1 3.881991 -0.341112 0.067577
rd_1m5fC/rd_1m5fC_2_042.xyz
6 1.045036 -0.528470 -0.519622
6 -1.226688 -0.988354 -0.124280
6 -1.517560 0.414283 0.060734
6 -0.457616 1.290394 -0.080129
1 -0.585474 2.362070 0.035389
7 0.778634 0.854551 -0.367088
7 0.014114 -1.415344 -0.408134
8 2.211135 -0.889607 -0.748333
7 -2.161476 -1.968346 -0.024168
1 -3.072064 -1.529346 0.165777
6 -2.824181 0.982818 0.382871
1 -2.844164 2.083509 0.455659
8 -3.856375 0.332642 0.568698
6 1.908996 1.787459 -0.502793
1 2.624944 1.604097 0.299920
1 2.395745 1.620845 -1.464725
1 1.531576 2.806572 -0.442583
8 4.176197 -0.542285 1.315278
1 4.198499 0.392808 1.563651
1 3.516460 -0.591367 0.596804
rd_1m5fC/rd_1m5fC_2_006.xyz
6 1.778926 0.745357 -0.110053
6 -0.526641 0.884987 -0.569907
6 -0.674091 -0.549264 -0.462413
6 0.463711 -1.265719 -0.143965
1 0.442816 -2.343515 -0.018474
7 1.646460 -0.659586 0.036606
7 0.663531 1.482178 -0.408529
8 2.895583 1.257978 0.039991

7 -1.558765 1.727379 -0.835400
1 -2.423571 1.172136 -0.886878
6 -1.920070 -1.292934 -0.609606
1 -1.824690 -2.385155 -0.486164
8 -3.023809 -0.790565 -0.844065
6 2.854479 -1.422872 0.383820
1 3.590252 -1.318196 -0.415893
1 3.274639 -1.029713 1.310818
1 2.582456 -2.469629 0.508163
8 -2.221514 0.432645 2.502361
1 -1.281818 0.400557 2.271002
1 -2.678630 0.325914 1.655143
rd_1m5fC/rd_1m5fC_3_078.xyz
6 1.793682 0.589273 -0.056403
6 -0.416194 1.224713 -0.532923
6 -0.854631 -0.142613 -0.590556
6 0.106812 -1.111053 -0.347165
1 -0.139152 -2.166739 -0.352961
7 1.374990 -0.777679 -0.087490
7 0.860138 1.557437 -0.277640
8 2.985128 0.832644 0.175017
7 -1.329864 2.207629 -0.738467
1 -0.840756 3.107697 -0.658976
6 -2.243434 -0.536415 -0.845018
1 -2.958150 0.282513 -1.004553
8 -2.617318 -1.711111 -0.880151
6 2.394585 -1.798957 0.192136
1 3.197217 -1.716137 -0.542775
1 2.803635 -1.628426 1.189731
1 1.933124 -2.783148 0.134847
8 -1.862229 0.264894 2.777277
1 -1.770198 -0.451377 3.422227
1 -1.292138 0.002802 2.040075
rd_1m5fC/rd_1m5fC_2_043.xyz
6 0.980277 0.617982 0.136360
6 -1.338641 0.995276 0.012282

6 -1.606113 -0.423920 -0.020587
6 -0.508315 -1.261481 0.027532
1 -0.614606 -2.341489 0.006954
7 0.741482 -0.777865 0.105415
7 -0.086066 1.470700 0.093527
8 2.149413 1.028854 0.202697
7 -2.309736 1.943397 -0.039050
1 -3.221108 1.470523 -0.102542
6 -2.924651 -1.046810 -0.098243
1 -2.914120 -2.149845 -0.111082
8 -3.997469 -0.438444 -0.145274
6 1.909000 -1.672483 0.156475
1 2.529685 -1.502024 -0.724602
1 2.490068 -1.453105 1.053335
1 1.561618 -2.703937 0.175488
8 4.830029 0.206956 -0.233556
1 3.881542 0.398989 -0.096147
1 4.942027 0.196952 -1.194492
rd_1m5fC/rd_1m5fC_2_067.xyz
6 -0.973948 0.653776 -0.078653
6 1.353432 0.990017 -0.005614
6 1.596995 -0.433935 0.009472
6 0.484066 -1.252180 -0.024190
1 0.572052 -2.334217 -0.018482
7 -0.758780 -0.746490 -0.068809
7 0.107894 1.487361 -0.054500
8 -2.136519 1.086676 -0.111118
7 2.341846 1.921048 0.028799
1 3.245876 1.432105 0.073820
6 2.906414 -1.079328 0.056924
1 2.877880 -2.181976 0.063867
8 3.990120 -0.489563 0.087037
6 -1.942063 -1.621382 -0.102005
1 -2.539615 -1.450583 0.795187
1 -2.537992 -1.385162 -0.984670
1 -1.612503 -2.658235 -0.138040

8 -4.816139 0.234928 0.181711
1 -4.822583 -0.709818 0.390839
1 -3.868897 0.456323 0.082402
rd_1m5fC/rd_1m5fC_2_082.xyz
6 -1.184504 1.374935 -0.095164
6 1.142011 1.062028 -0.292667
6 1.059388 -0.237119 0.335578
6 -0.199316 -0.660256 0.718567
1 -0.355607 -1.622419 1.196347
7 -1.286352 0.100152 0.518612
7 0.050938 1.819038 -0.485612
8 -2.214780 2.039955 -0.263134
7 2.304103 1.610105 -0.732857
1 3.068307 0.949703 -0.537299
6 2.179420 -1.135689 0.592724
1 1.906577 -2.083083 1.088097
8 3.357619 -0.907416 0.301605
6 -2.630003 -0.368285 0.888881
1 -3.074519 0.333620 1.596554
1 -3.250131 -0.415721 -0.008143
1 -2.546663 -1.355302 1.340930
8 -1.486427 -3.070910 -1.365925
1 -0.945080 -2.303373 -1.601846
1 -1.736979 -2.915198 -0.443521
rd_1m5fU/rd_1m5fU1_a_009.xyz
6 -1.146032 0.261914 -0.076650
6 0.561345 -1.337290 -0.051625
6 1.583099 -0.266162 -0.004516
6 1.157918 1.079169 0.010370
1 1.870227 1.897646 0.052137
7 -0.125074 1.354733 -0.026473
7 -0.752121 -1.007152 -0.086526
8 -2.312543 0.668133 -0.102775
8 0.949190 -2.527007 -0.057157
6 -0.616563 2.734582 0.009257
1 -1.223940 2.915413 -0.879304

1 -1.242844 2.856976 0.895170
1 0.234516 3.411809 0.039186
6 3.022959 -0.577786 0.037641
1 3.292267 -1.640996 0.005377
8 3.873740 0.306762 0.111535
8 -4.421560 -1.187847 0.205838
1 -5.193757 -0.760614 -0.190805
1 -3.683076 -0.560204 0.062836
rd_1m5fU/rd_1m5fU1_a_017.xyz
6 -1.144528 0.250397 -0.091557
6 0.571828 -1.338793 -0.052369
6 1.587157 -0.261874 -0.000165
6 1.153997 1.080946 0.015730
1 1.861166 1.903543 0.063728
7 -0.130321 1.349021 -0.028401
7 -0.743194 -1.016307 -0.098926
8 -2.312854 0.650177 -0.130150
8 0.966425 -2.526300 -0.050744
6 -0.630146 2.725968 0.006284
1 -1.227774 2.906589 -0.888956
1 -1.267814 2.841830 0.884921
1 0.216751 3.407804 0.048634
6 3.028572 -0.565534 0.046461
1 3.303776 -1.627220 0.013734
8 3.874362 0.323501 0.123722
8 -4.458616 -1.171075 0.110881
1 -5.034660 -0.752370 0.765924
1 -3.692660 -0.566266 0.027333
rd_1m5fU/rd_1m5fU1_a_097.xyz
6 1.038961 0.593024 -0.473975
6 -1.142200 1.328101 -0.043000
6 -1.607644 -0.072170 0.076255
6 -0.676883 -1.115383 -0.110051
1 -0.974533 -2.156947 -0.044829
7 0.576588 -0.830011 -0.376165
7 0.163019 1.578198 -0.307333

8 2.243396 0.720521 -0.715121
8 -1.978672 2.247204 0.103654
6 1.574614 -1.879513 -0.600815
1 1.985883 -1.759566 -1.605185
1 2.379677 -1.760778 0.126662
1 1.097888 -2.852005 -0.491862
6 -3.015812 -0.393488 0.369670
1 -3.699406 0.459157 0.468723
8 -3.400526 -1.554090 0.498123
8 4.187164 0.048912 1.306317
1 5.052306 0.128363 0.880007
1 3.543816 0.240669 0.598678
rd_1m5fU/rd_1m5fU1_a_027.xyz
6 -0.956665 0.736767 0.098580
6 1.312716 1.306998 -0.011972
6 1.686626 -0.125922 -0.013184
6 0.662341 -1.095635 0.025415
1 0.888418 -2.157276 0.014602
7 -0.593113 -0.715776 0.076530
7 0.004833 1.654760 0.051079
8 -2.168859 0.960716 0.161162
8 2.226027 2.160684 -0.075188
6 -1.688266 -1.689627 0.109483
1 -2.294008 -1.506984 0.998419
1 -2.306408 -1.547481 -0.779164
1 -1.267386 -2.692853 0.128053
6 3.094754 -0.558484 -0.057683
1 3.851183 0.236407 -0.070728
8 3.403140 -1.748517 -0.078168
8 -4.742584 -0.246925 -0.206159
1 -3.854338 0.136475 -0.080772
1 -5.310332 0.506580 -0.420678
rd_1m5fU/rd_1m5fU1_a_016.xyz
6 1.064511 -0.564008 -0.532011
6 -1.100517 -1.329706 -0.075928
6 -1.576842 0.063030 0.081222

6 -0.661905 1.119344 -0.109998
1 -0.969725 2.156459 -0.023613
7 0.588028 0.851887 -0.409503
7 0.201884 -1.561254 -0.369462
8 2.268330 -0.674263 -0.786271
8 -1.925284 -2.259887 0.066792
6 1.565972 1.916438 -0.652501
1 2.390427 1.805388 0.053932
1 1.953276 1.807239 -1.667529
1 1.078961 2.881707 -0.526377
6 -2.981935 0.363551 0.408625
1 -3.654696 -0.498225 0.502993
8 -3.376041 1.517027 0.568708
8 4.034951 -0.018879 1.402919
1 3.462983 -0.235523 0.643311
1 4.338031 -0.875362 1.736404
rd_1m5fU/rd_1m5fU1_a_080.xyz
6 0.611111 -1.578003 -0.059977
6 -0.933765 0.180597 -0.057691
6 0.169299 1.168622 -0.033944
6 1.500501 0.701886 0.029714
1 2.338561 1.390412 0.080676
7 1.736789 -0.588409 0.029548
7 -0.647975 -1.137574 -0.084372
8 0.972023 -2.752887 -0.099043
8 -2.111355 0.613346 -0.051018
6 3.098118 -1.123310 0.121482
1 3.164409 -1.750641 1.012726
1 3.296097 -1.736504 -0.759325
1 3.799444 -0.293158 0.179710
6 -0.098091 2.617884 -0.048993
1 -1.150387 2.918183 -0.129787
8 0.809545 3.443560 0.030829
8 -4.361200 -1.024612 0.135573
1 -4.108058 -1.958138 0.113394
1 -3.516899 -0.529584 0.052099

rd_1m5fU/rd_1m5fU1_a_050.xyz
6 0.594707 -1.580211 -0.062690
6 -0.934508 0.192266 -0.066389
6 0.177033 1.170355 -0.033515
6 1.503979 0.691597 0.032267
1 2.347885 1.372533 0.087644
7 1.729010 -0.600686 0.028952
7 -0.660134 -1.128305 -0.091683
8 0.944624 -2.758493 -0.099412
8 -2.108365 0.635338 -0.068652
6 3.085554 -1.147763 0.121283
1 3.147465 -1.771805 1.015201
1 3.276146 -1.766778 -0.757036
1 3.794698 -0.323909 0.174644
6 -0.078070 2.621837 -0.041411
1 -1.128084 2.931196 -0.117827
8 0.836640 3.439612 0.039073
8 -4.350836 -1.018023 0.119516
1 -4.072187 -1.908647 0.374928
1 -3.516725 -0.505615 0.040088
rd_1m5fU/rd_1m5fU1_a_043.xyz
6 1.827935 -0.906766 -0.088421
6 -0.488603 -0.978621 -0.423301
6 -0.556102 0.497712 -0.314904
6 0.602917 1.202022 0.080526
1 0.577413 2.270733 0.268815
7 1.735473 0.557012 0.225803
7 0.713631 -1.588528 -0.361396
8 2.969289 -1.363704 -0.048238
8 -1.556792 -1.618154 -0.574549
6 2.959999 1.232666 0.666116
1 3.334908 0.719761 1.553899
1 3.706352 1.164413 -0.127460
1 2.727176 2.272199 0.888890
6 -1.798404 1.239560 -0.594220
1 -2.644260 0.650907 -0.971867

8 -1.880009 2.452872 -0.413831
8 -3.692361 -1.009380 1.243237
1 -2.992232 -1.207808 0.591316
1 -3.860550 -0.062107 1.137836
rd_1m5fU/rd_1m5fU1_a_020.xyz
6 1.059223 0.582743 -0.526947
6 -1.112558 1.326706 -0.066473
6 -1.577441 -0.071060 0.080104
6 -0.654077 -1.118229 -0.120192
1 -0.953373 -2.158467 -0.042504
7 0.593653 -0.838321 -0.417319
7 0.188082 1.571421 -0.357226
8 2.262457 0.704346 -0.778297
8 -1.945401 2.248818 0.081832
6 1.580847 -1.892540 -0.667436
1 1.964867 -1.774925 -1.682861
1 2.405764 -1.775330 0.037830
1 1.103372 -2.863085 -0.544919
6 -2.979639 -0.385920 0.406440
1 -3.659059 0.469459 0.510506
8 -3.364278 -1.544098 0.555417
8 3.984757 0.126592 1.476595
1 4.818768 -0.240302 1.150708
1 3.449100 0.275480 0.675695
rd_1m5fU/rd_1m5fU1_a_006.xyz
6 -1.760319 0.640372 -0.072383
6 0.462933 1.327535 -0.373580
6 0.902523 -0.087355 -0.420610
6 -0.066901 -1.106848 -0.296604
1 0.210256 -2.155491 -0.330351
7 -1.327759 -0.791357 -0.133325
7 -0.850330 1.606524 -0.200527
8 -2.971540 0.799208 0.092670
8 1.325413 2.226675 -0.491387
6 -2.369425 -1.815852 -0.000823
1 -2.866443 -1.691154 0.962602

1 -3.099697 -1.679010 -0.800091
1 -1.906052 -2.798479 -0.069690
6 2.318243 -0.447852 -0.609625
1 3.027679 0.387831 -0.665919
8 2.686323 -1.617419 -0.698842
8 1.346693 -0.025666 2.277524
1 1.823510 0.811355 2.165212
1 2.039937 -0.703602 2.277224
rd_1m5fU/rd_1m5fU1_a_074.xyz
6 -1.068920 0.994955 -0.000004
6 -0.111158 -1.157810 -0.000009
6 1.246138 -0.563920 0.000000
6 1.376816 0.841962 0.000002
1 2.352365 1.317316 0.000006
7 0.304601 1.599077 0.000000
7 -1.187441 -0.334307 -0.000003
8 -1.990798 1.806802 -0.000006
8 -0.227853 -2.401826 -0.000023
6 0.387095 3.063359 0.000001
1 -0.123971 3.439429 -0.888337
1 -0.123974 3.439428 0.888337
1 1.433989 3.359981 0.000003
6 2.452862 -1.408471 0.000005
1 2.288466 -2.493346 0.000001
8 3.581731 -0.921077 0.000012
8 -3.733698 -1.590718 0.000020
1 -2.852110 -1.145910 0.000010
1 -3.526935 -2.536179 0.000014
rd_1m5fU/rd_1m5fU1_a_035.xyz
6 1.032587 -0.626419 -0.460534
6 -1.159732 -1.324283 -0.023392
6 -1.606471 0.083997 0.071953
6 -0.660171 1.111382 -0.123771
1 -0.942710 2.157908 -0.072471
7 0.590965 0.804701 -0.376803
7 0.142267 -1.596461 -0.282590

8 2.234782 -0.773766 -0.700921
8 -2.008675 -2.229444 0.137360
6 1.608148 1.837248 -0.592968
1 2.390424 1.721973 0.159652
1 2.045842 1.695048 -1.582859
1 1.141807 2.817427 -0.512350
6 -3.010300 0.429437 0.358866
1 -3.703671 -0.412005 0.482324
8 -3.380891 1.597676 0.456008
8 4.262846 -0.104176 1.220506
1 3.570062 -0.288900 0.558961
1 4.276766 0.860388 1.297940
rd_1m5fU/rd_1m5fU1_a_066.xyz
6 1.660809 0.678745 -0.141804
6 -0.565411 1.406015 -0.234144
6 -1.025259 0.013421 -0.445481
6 -0.062678 -1.016406 -0.532533
1 -0.354465 -2.048852 -0.695670
7 1.210958 -0.727747 -0.419431
7 0.756446 1.656101 -0.090556
8 2.876687 0.800764 0.007659
8 -1.425851 2.314467 -0.191126
6 2.244669 -1.764105 -0.496162
1 2.938823 -1.508204 -1.298784
1 2.789890 -1.785732 0.449297
1 1.767419 -2.723190 -0.689818
6 -2.457928 -0.321590 -0.515830
1 -3.159644 0.517101 -0.424800
8 -2.844642 -1.479703 -0.663956
8 -0.508047 -0.674913 2.808633
1 0.055545 -0.012651 2.381491
1 -1.359779 -0.598344 2.354244
rd_1m5fU/rd_1m5fU1_a_007.xyz
6 -2.343582 0.390261 0.071890
6 -0.269801 1.474886 -0.043436
6 0.428434 0.167502 -0.054834

6 -0.338919 -1.017281 -0.073935
1 0.130159 -1.995216 -0.122337
7 -1.648104 -0.941030 -0.028398
7 -1.618737 1.507619 0.044567
8 -3.567809 0.313498 0.167841
8 0.421653 2.516309 -0.113949
6 -2.495586 -2.135320 -0.051946
1 -3.085079 -2.163128 0.866475
1 -3.173776 -2.063385 -0.904425
1 -1.866094 -3.019375 -0.132599
6 1.896298 0.091160 -0.037708
1 2.436172 1.045731 -0.020890
8 2.490722 -0.987639 -0.033646
8 5.330314 -0.511529 0.122975
1 4.373381 -0.697440 0.065151
1 5.393014 0.454327 0.129494
rd_1m5fU/rd_1m5fU1_a_038.xyz
6 1.767991 -0.643460 -0.024526
6 -0.444910 -1.316206 -0.423386
6 -0.882751 0.099903 -0.423675
6 0.092249 1.113258 -0.311120
1 -0.176490 2.163518 -0.360926
7 1.350971 0.790276 -0.131619
7 0.859543 -1.603412 -0.199313
8 2.965806 -0.808104 0.215556
8 -1.298980 -2.208026 -0.624969
6 2.400288 1.808164 -0.015334
1 2.887284 1.704883 0.955563
1 3.136749 1.642108 -0.803656
1 1.947236 2.792919 -0.115678
6 -2.304996 0.469928 -0.554692
1 -3.024459 -0.358214 -0.536294
8 -2.661837 1.637551 -0.688925
8 -1.717541 -0.025313 2.214514
1 -1.056124 0.654557 2.414765
1 -1.254606 -0.866206 2.349745

rd_1m5fU/rd_1m5fU1_a_081.xyz
6 1.767720 0.633998 -0.018317
6 -0.438962 1.352386 -0.367591
6 -0.895715 -0.056242 -0.425090
6 0.065223 -1.086832 -0.352351
1 -0.219315 -2.130819 -0.436130
7 1.328888 -0.789047 -0.166628
7 0.870859 1.612399 -0.143451
8 2.971695 0.774853 0.206277
8 -1.282309 2.263056 -0.526042
6 2.362505 -1.827162 -0.089743
1 3.082505 -1.663499 -0.893700
1 2.874006 -1.746528 0.870378
1 1.888403 -2.801599 -0.192999
6 -2.323320 -0.401386 -0.562410
1 -3.030071 0.436766 -0.517388
8 -2.698828 -1.559349 -0.726113
8 -1.627103 -0.112885 2.233383
1 -1.213770 -0.986363 2.312388
1 -0.912334 0.514615 2.420984
rd_1m5fU/rd_1m5fU1_a_001.xyz
6 -0.954158 0.744816 -0.082257
6 1.318389 1.305549 0.013547
6 1.686464 -0.128832 0.011977
6 0.657835 -1.094134 -0.017106
1 0.879657 -2.156708 -0.006719
7 -0.596359 -0.709243 -0.058559
7 0.011598 1.658704 -0.041732
8 -2.165673 0.973705 -0.139596
8 2.235676 2.155357 0.071160
6 -1.695250 -1.679015 -0.082716
1 -2.313633 -1.526392 0.803885
1 -2.299534 -1.502376 -0.973876
1 -1.278256 -2.683995 -0.091635
6 3.092965 -0.567801 0.042673
1 3.853040 0.223652 0.055912

8 3.396061 -1.759354 0.051132
8 -4.737453 -0.259217 0.181810
1 -5.356255 0.483968 0.210502
1 -3.858060 0.148203 0.071215
rd_1m5fU/rd_1m5fU1_a_091.xyz
6 -1.728689 0.644558 -0.081287
6 0.495331 1.297189 -0.445961
6 0.923820 -0.120860 -0.410175
6 -0.060643 -1.127030 -0.302082
1 0.203072 -2.179457 -0.327167
7 -1.320076 -0.793375 -0.161420
7 -0.811906 1.596601 -0.258191
8 -2.929132 0.820291 0.136049
8 1.360231 2.179439 -0.645219
6 -2.378847 -1.802717 -0.054921
1 -2.889128 -1.682115 0.901835
1 -3.093958 -1.643780 -0.864071
1 -1.929634 -2.791493 -0.129135
6 2.345569 -0.497998 -0.496510
1 3.067820 0.328581 -0.479477
8 2.703722 -1.670038 -0.585065
8 1.313338 0.018738 2.342682
1 0.453326 0.462701 2.399445
1 1.947869 0.736685 2.193888
rd_1m5fU/rd_1m5fU1_a_025.xyz
6 -1.998305 0.121583 0.204309
6 -0.262370 1.695971 0.182234
6 0.682248 0.668877 -0.315164
6 0.202266 -0.634412 -0.566069
1 0.849867 -1.412303 -0.957723
7 -1.060992 -0.913465 -0.347309
7 -1.548944 1.356306 0.426652
8 -3.142839 -0.286590 0.401536
8 0.170713 2.856046 0.369584
6 -1.612113 -2.242395 -0.624943
1 -1.995352 -2.666473 0.305052

1 -2.436156 -2.136735 -1.333087
1 -0.825182 -2.869367 -1.039075
6 2.102598 0.983161 -0.549286
1 2.376600 2.045245 -0.551692
8 2.941171 0.097905 -0.714682
8 2.527228 -2.197470 1.243289
1 2.890841 -1.841557 2.066787
1 2.752809 -1.534534 0.570043
rd_1m5fU/rd_1m5fU1_a_045.xyz
6 -2.075263 0.203531 0.146303
6 -0.654474 -1.659947 0.148418
6 0.500720 -0.800164 -0.202706
6 0.289498 0.583421 -0.385972
1 1.096533 1.252487 -0.665014
7 -0.915303 1.081094 -0.231092
7 -1.877571 -1.104372 0.304921
8 -3.142280 0.803133 0.276197
8 -0.454853 -2.887894 0.292862
6 -1.184620 2.511257 -0.405271
1 -1.946431 2.634301 -1.177247
1 -1.566486 2.910686 0.536442
1 -0.261100 3.011677 -0.690292
6 1.845877 -1.374623 -0.372230
1 1.911394 -2.469352 -0.358442
8 2.845813 -0.672298 -0.517405
8 3.319515 1.894320 0.806792
1 4.250154 1.887172 1.071859
1 3.190067 1.057048 0.327057
rd_1m5hmC/rd_1m5hmC_3_098.xyz
6 1.408317 0.514726 -0.015855
6 -0.477460 -0.877456 0.151590
6 -1.367053 0.248889 0.196253
6 -0.780058 1.479235 0.120691
1 -1.367268 2.390555 0.142262
7 0.566194 1.636338 0.018494
7 0.849711 -0.720594 0.053013

8 2.644084 0.685258 -0.113292
6 -2.859052 0.111917 0.311918
1 -3.156101 -0.502890 1.189996
1 -3.340938 1.084856 0.515463
7 -0.944125 -2.136997 0.217530
1 -0.294693 -2.909117 0.146089
1 -1.932845 -2.340434 0.215779
8 -3.495233 -0.487777 -0.768789
6 1.175815 2.969423 -0.070314
1 1.862140 3.120958 0.765245
1 1.728573 3.060987 -1.007349
1 0.385341 3.718480 -0.035382
8 2.680827 -2.851607 -0.101824
1 3.540203 -2.418426 -0.204103
1 2.032654 -2.103585 -0.045717
rd_1m5hmC/rd_1m5hmC_3_085.xyz
6 -1.036818 -0.603769 -0.035546
6 1.265778 -0.992251 -0.110767
6 1.554365 0.414008 -0.175190
6 0.473757 1.246272 -0.163339
1 0.585552 2.323989 -0.209617
7 -0.801134 0.776527 -0.095765
7 0.011775 -1.457387 -0.044744
8 -2.224715 -1.012569 0.026803
6 2.948192 0.968694 -0.260931
1 3.497449 0.570765 -1.144369
1 2.944208 2.058277 -0.438309
7 2.257979 -1.902513 -0.131578
1 2.025752 -2.882426 -0.042886
1 3.229825 -1.635652 -0.074186
8 3.779486 0.678234 0.813159
6 -1.949039 1.694018 -0.077194
1 -2.606072 1.482864 -0.922559
1 -2.507336 1.574638 0.852861
1 -1.580785 2.716768 -0.151362
8 -4.831897 -0.072267 0.220621

1 -3.884886 -0.325524 0.156757
1 -5.304449 -0.909099 0.331409
rd_1m5hmC/rd_1m5hmC_3_082.xyz
6 -1.097332 0.447811 -0.492558
6 1.113437 1.016987 -0.001343
6 1.465043 -0.352968 0.254808
6 0.459608 -1.264244 0.109897
1 0.621645 -2.322326 0.284499
7 -0.799740 -0.900056 -0.250909
7 -0.126221 1.377271 -0.362064
8 -2.273649 0.748824 -0.827581
6 2.845257 -0.791873 0.659535
1 3.214681 -0.240815 1.552471
1 2.853096 -1.844154 0.994691
7 2.024798 1.998732 0.125288
1 1.755374 2.954018 -0.066881
1 2.990851 1.808832 0.346607
8 3.837067 -0.618283 -0.299436
6 -1.869938 -1.897372 -0.396289
1 -2.662947 -1.705942 0.328394
1 -2.285839 -1.846487 -1.404042
1 -1.449645 -2.887869 -0.225269
8 -4.303646 0.377243 1.040284
1 -3.586474 0.492193 0.378567
1 -4.717241 1.248604 1.114318
rd_1m5hmC/rd_1m5hmC_3_024.xyz
6 1.065262 0.580803 -0.318105
6 -1.227397 0.997567 -0.150122
6 -1.535619 -0.406260 -0.142752
6 -0.468466 -1.252260 -0.225236
1 -0.596233 -2.329157 -0.228879
7 0.810265 -0.797853 -0.316737
7 0.031367 1.447750 -0.237666
8 2.258221 0.973301 -0.398499
6 -2.935745 -0.946855 -0.062857
1 -3.584758 -0.541116 -0.870706

1 -2.960441 -2.035655 -0.244995
7 -2.205786 1.919573 -0.080177
1 -1.958502 2.899443 -0.052254
1 -3.170586 1.666668 0.074280
8 -3.626054 -0.667864 1.110672
6 1.942675 -1.732203 -0.392767
1 2.578326 -1.473902 -1.240642
1 2.529651 -1.684647 0.526376
1 1.553115 -2.741368 -0.523162
8 4.565773 0.134091 0.899249
1 3.728808 0.376243 0.444515
1 4.801904 0.916218 1.417187
rd_1m5hmC/rd_1m5hmC_3_010.xyz
6 -1.086811 0.500637 -0.471532
6 1.138096 1.013335 0.018561
6 1.462016 -0.368431 0.246006
6 0.438588 -1.255984 0.082065
1 0.579628 -2.320479 0.234791
7 -0.813254 -0.859319 -0.270903
7 -0.095706 1.406718 -0.326965
8 -2.259515 0.834740 -0.786549
6 2.832979 -0.843566 0.641022
1 3.215004 -0.314300 1.541813
1 2.819175 -1.901059 0.959078
7 2.070296 1.973217 0.162596
1 1.823576 2.935625 -0.024468
1 3.037676 1.756547 0.351668
8 3.827342 -0.674190 -0.316236
6 -1.905097 -1.832308 -0.418428
1 -2.652030 -1.679677 0.362980
1 -2.376653 -1.711813 -1.394656
1 -1.491077 -2.836616 -0.334516
8 -4.343724 0.473587 1.019469
1 -4.560721 -0.469040 1.044582
1 -3.595376 0.541305 0.386005
rd_1m5hmC/rd_1m5hmC_3_062.xyz

6 -2.363534 0.456297 -0.039103
6 -0.141970 1.180231 0.017382
6 0.359120 -0.170161 0.050457
6 -0.594526 -1.161506 0.047258
1 -0.318735 -2.210504 0.072378
7 -1.913673 -0.886790 0.010028
7 -1.452714 1.453935 -0.026974
8 -3.596083 0.658589 -0.092879
6 1.804664 -0.569469 0.074883
1 1.975431 -1.268653 0.931059
1 2.001990 -1.269937 -0.774013
7 0.715216 2.212816 0.029263
1 0.356120 3.158139 -0.000451
1 1.712935 2.029559 0.058076
8 2.767294 0.383442 0.091534
6 -2.915868 -1.960369 -0.001751
1 -3.591408 -1.840719 0.846872
1 -3.491956 -1.915840 -0.928097
1 -2.401356 -2.918084 0.067860
8 5.222814 -0.936985 -0.106021
1 5.891135 -0.285621 -0.360871
1 4.384534 -0.428574 -0.034864
rd_1m5hmC/rd_1m5hmC_3_034.xyz
6 -2.363906 0.460579 -0.033847
6 -0.139751 1.177014 0.014989
6 0.357003 -0.175136 0.043359
6 -0.599972 -1.163309 0.041966
1 -0.327721 -2.213311 0.063752
7 -1.918355 -0.884028 0.011056
7 -1.449750 1.455231 -0.023488
8 -3.596012 0.666945 -0.082218
6 1.801408 -0.579065 0.062279
1 1.972924 -1.279362 0.917371
1 1.993834 -1.279434 -0.787852
7 0.720728 2.206881 0.025483
1 0.364308 3.153332 -0.000548

1 1.717978 2.020599 0.050107
8 2.766879 0.370922 0.076605
6 -2.924231 -1.954170 0.001540
1 -3.598239 -1.831170 0.850868
1 -3.501350 -1.908824 -0.924143
1 -2.412908 -2.913534 0.071894
8 5.233556 -0.926839 -0.114998
1 4.385527 -0.435107 -0.039774
1 5.918604 -0.243481 -0.109862
rd_1m5hmC/rd_1m5hmC_3_065.xyz
6 1.099903 -0.435167 -0.498843
6 -1.107425 -1.016607 -0.006835
6 -1.464714 0.350538 0.256392
6 -0.462922 1.266686 0.116931
1 -0.629095 2.323148 0.297188
7 0.797923 0.909471 -0.245576
7 0.133090 -1.369563 -0.371823
8 2.276330 -0.728800 -0.840078
6 -2.846785 0.781216 0.663447
1 -3.213279 0.225072 1.554437
1 -2.859505 1.832119 1.002779
7 -2.014186 -2.002987 0.116386
1 -1.740200 -2.956450 -0.078408
1 -2.979885 -1.818970 0.344058
8 -3.838079 0.606935 -0.295879
6 1.864430 1.911397 -0.385610
1 2.667022 1.705939 0.324480
1 2.267543 1.881349 -1.399527
1 1.445235 2.897515 -0.188719
8 4.310707 -0.402285 1.029285
1 3.587436 -0.492871 0.370346
1 4.550348 -1.310471 1.260944
rd_1m5hmC/rd_1m5hmC_3_004.xyz
6 1.433269 -0.512084 -0.012276
6 -0.470048 0.855145 -0.187363
6 -1.347693 -0.281433 -0.195239

6 -0.746038 -1.503441 -0.101819
1 -1.323500 -2.421281 -0.095679
7 0.603079 -1.643403 -0.013718
7 0.860618 0.715224 -0.105034
8 2.671447 -0.667967 0.077956
6 -2.843014 -0.163714 -0.288000
1 -3.162402 0.436659 -1.168142
1 -3.316136 -1.145004 -0.470999
7 -0.952661 2.107754 -0.272522
1 -0.313223 2.889877 -0.223166
1 -1.943846 2.298535 -0.260171
8 -3.468116 0.440021 0.797208
6 1.228633 -2.967914 0.091606
1 1.909344 -3.125065 -0.747467
1 1.790909 -3.038779 1.024796
1 0.446540 -3.726199 0.075208
8 2.494880 2.991456 0.130124
1 1.959464 2.160960 0.050777
1 3.259243 2.745851 0.670009
rd_1m5hmC/rd_1m5hmC_3_063.xyz
6 1.064325 0.584162 -0.312657
6 -1.229032 0.998207 -0.146857
6 -1.535863 -0.405957 -0.143819
6 -0.467658 -1.250635 -0.226147
1 -0.594319 -2.327648 -0.232722
7 0.810786 -0.794785 -0.314181
7 0.029405 1.449823 -0.231712
8 2.256955 0.978070 -0.390839
6 -2.935525 -0.948528 -0.069017
1 -3.582900 -0.541118 -0.877339
1 -2.958380 -2.036817 -0.254379
7 -2.208333 1.919184 -0.075560
1 -1.961778 2.899137 -0.044196
1 -3.172971 1.665164 0.078146
8 -3.629251 -0.673796 1.103494
6 1.944230 -1.727879 -0.390217

1 2.580751 -1.467614 -1.236799
1 2.529723 -1.681761 0.529988
1 1.555834 -2.737196 -0.522838
8 4.559548 0.119515 0.904920
1 3.728128 0.372574 0.446098
1 4.912025 0.949198 1.255888
rd_1m5hmC/rd_1m5hmC_3_003.xyz
6 1.805919 -0.731595 0.039810
6 -0.442296 -0.828664 0.674498
6 -0.592773 0.596739 0.563020
6 0.522271 1.287426 0.185067
1 0.513771 2.365968 0.071606
7 1.703577 0.666203 -0.067813
7 0.716668 -1.444869 0.418124
8 2.907339 -1.270846 -0.218882
6 -1.887288 1.309266 0.829306
1 -2.290807 1.092837 1.842604
1 -1.752224 2.406619 0.850904
7 -1.475045 -1.598709 1.076284
1 -1.363210 -2.603697 1.055693
1 -2.416747 -1.236151 1.110179
8 -2.927872 1.022679 -0.044069
6 2.890629 1.427206 -0.477595
1 3.705654 1.254625 0.227958
1 3.209922 1.114306 -1.473788
1 2.637427 2.486845 -0.490401
8 -2.310392 -0.667180 -2.297217
1 -1.491393 -1.138781 -2.087828
1 -2.500167 -0.140449 -1.496389
rd_1m5hmC/rd_1m5hmC_3_070.xyz
6 1.037374 0.599148 0.066412
6 -1.259528 0.992908 -0.098931
6 -1.548178 -0.412424 -0.180015
6 -0.470759 -1.247361 -0.127046
1 -0.582771 -2.324567 -0.182155
7 0.801469 -0.780406 -0.008386

7 -0.008328 1.455218 0.020497
8 2.222830 1.004667 0.177997
6 -2.937482 -0.963152 -0.334814
1 -3.441408 -0.562935 -1.243926
1 -2.926581 -2.052342 -0.514900
7 -2.248398 1.904926 -0.159917
1 -2.018548 2.884539 -0.062017
1 -3.222097 1.639263 -0.140720
8 -3.821285 -0.673269 0.696262
6 1.947154 -1.699995 0.036487
1 2.602694 -1.518015 -0.817064
1 2.507746 -1.551799 0.960781
1 1.576239 -2.723571 -0.004224
8 4.834621 0.080645 -0.029104
1 3.884153 0.320865 0.034344
1 5.306562 0.919312 0.070710
rd_1m5hmC/rd_1m5hmC_3_012.xyz
6 1.095877 -0.471695 -0.495525
6 -1.119399 -1.014081 0.005657
6 -1.456503 0.361507 0.251088
6 -0.442687 1.261423 0.094653
1 -0.593716 2.322461 0.261222
7 0.811774 0.881689 -0.267632
7 0.115742 -1.389962 -0.354532
8 2.268490 -0.787868 -0.829076
6 -2.830942 0.817681 0.656485
1 -3.203169 0.276830 1.554503
1 -2.827329 1.872524 0.983512
7 -2.040039 -1.985592 0.143862
1 -1.781711 -2.944424 -0.045878
1 -3.005434 -1.783458 0.357264
8 -3.827335 0.646052 -0.298161
6 1.891417 1.867393 -0.420751
1 2.670803 1.690085 0.322539
1 2.323751 1.787815 -1.419434
1 1.475517 2.864812 -0.282135

8 4.300583 -0.533964 1.051591
1 3.575945 -0.557418 0.388194
1 4.392527 0.400699 1.283849
rd_1m5hmC/rd_1m5hmC_3_018.xyz
6 1.096594 -0.442494 -0.493836
6 -1.113205 -1.015696 -0.002779
6 -1.467395 0.353647 0.253181
6 -0.462838 1.266416 0.111286
1 -0.626494 2.324159 0.286269
7 0.797682 0.904103 -0.246977
7 0.126921 -1.373672 -0.364057
8 2.272534 -0.740803 -0.832215
6 -2.848676 0.789933 0.656927
1 -3.217176 0.238921 1.550301
1 -2.858896 1.842465 0.991275
7 -2.022773 -1.999104 0.123927
1 -1.750889 -2.954256 -0.065385
1 -2.988574 -1.811310 0.348055
8 -3.839873 0.613568 -0.302114
6 1.867832 1.901901 -0.387705
1 2.677572 1.680577 0.309276
1 2.259051 1.883368 -1.406734
1 1.457027 2.887574 -0.171936
8 4.269547 -0.500561 1.099696
1 3.581516 -0.536216 0.399311
1 5.102510 -0.374446 0.623943
rd_1m5hmC/rd_1m5hmC_3_014.xyz
6 2.358987 0.437991 0.053054
6 0.148365 1.193523 -0.014982
6 -0.371101 -0.149237 -0.068488
6 0.568571 -1.153810 -0.069500
1 0.277972 -2.198341 -0.109642
7 1.890948 -0.898256 -0.016956
7 1.462236 1.448033 0.045147
8 3.593516 0.622783 0.120750
6 -1.821710 -0.527455 -0.107512

1 -1.995985 -1.215001 -0.972484
1 -2.035526 -1.234422 0.732113
7 -0.694680 2.237599 -0.022756
1 -0.323960 3.177669 0.024742
1 -1.694506 2.067443 -0.060985
8 -2.770283 0.439640 -0.122658
6 2.878337 -1.985598 -0.008949
1 3.558178 -1.870385 -0.854823
1 3.452116 -1.953948 0.919319
1 2.351207 -2.935921 -0.085244
8 -5.241268 -0.843817 0.122914
1 -5.040429 -1.616221 0.670143
1 -4.383002 -0.373837 0.029024
rd_1m5hmC/rd_1m5hmC_3_032.xyz
6 1.436298 -0.515876 0.002902
6 -0.465766 0.852777 -0.173515
6 -1.343104 -0.283767 -0.198616
6 -0.742345 -1.506450 -0.109274
1 -1.319824 -2.424272 -0.115002
7 0.606012 -1.647062 -0.011960
7 0.864270 0.712473 -0.080095
8 2.674097 -0.673229 0.095533
6 -2.837389 -0.165102 -0.304866
1 -3.148168 0.439229 -1.185397
1 -3.309064 -1.145336 -0.497045
7 -0.948421 2.105653 -0.252631
1 -0.309576 2.887965 -0.198224
1 -1.939791 2.295718 -0.251251
8 -3.472786 0.434325 0.776630
6 1.230750 -2.972272 0.088737
1 1.912990 -3.126532 -0.749659
1 1.791133 -3.046952 1.022741
1 0.448275 -3.730077 0.068113
8 2.462634 3.008155 0.211955
1 3.287627 2.875150 -0.275864
1 1.951159 2.167793 0.089233

rd_1m5hmC/rd_1m5hmC_3_048.xyz
6 -1.136367 -0.486274 -0.552546
6 1.126034 -0.977476 -0.253659
6 1.466191 0.412916 -0.126802
6 0.426849 1.292988 -0.216049
1 0.578824 2.363311 -0.130099
7 -0.852444 0.879892 -0.421711
7 -0.133155 -1.385991 -0.462395
8 -2.333317 -0.829331 -0.743302
6 2.870749 0.903192 0.090377
1 3.566394 0.532502 -0.695254
1 2.938120 2.000436 -0.012954
7 2.075296 -1.929099 -0.177713
1 1.802091 -2.901744 -0.217553
1 3.026608 -1.712678 0.080371
8 3.464443 0.519678 1.287070
6 -1.960223 1.841821 -0.508570
1 -2.472559 1.733465 -1.466045
1 -2.671622 1.663209 0.299755
1 -1.555325 2.849591 -0.421706
8 -3.920273 -0.581446 1.562030
1 -3.377152 -0.630470 0.746234
1 -3.909487 0.354556 1.807098
rd_1m5hmC/rd_1m5hmC_3_019.xyz
6 1.062579 -0.603537 0.312994
6 -1.234004 -0.997466 0.137776
6 -1.529584 0.409222 0.148379
6 -0.454826 1.244248 0.241708
1 -0.572760 2.322172 0.258582
7 0.819805 0.777439 0.328754
7 0.020613 -1.459740 0.222128
8 2.251668 -1.007981 0.387955
6 -2.924285 0.964896 0.076982
1 -3.576331 0.554764 0.880214
1 -2.937835 2.051401 0.273225
7 -2.220040 -1.910063 0.053069

1 -1.980872 -2.891576 0.013640
1 -3.182648 -1.647604 -0.099458
8 -3.618938 0.707717 -1.098970
6 1.959648 1.701627 0.411621
1 2.604078 1.420352 1.245132
1 2.534291 1.670822 -0.516343
1 1.579365 2.710493 0.568599
8 4.572874 -0.272659 -0.936158
1 4.542050 0.661463 -1.185345
1 3.726016 -0.436290 -0.465263
rd_1m5hmC/rd_1m5hmC_3_000.xyz
6 1.437798 0.516819 -0.008671
6 -0.464319 -0.851802 0.167541
6 -1.341126 0.284970 0.199612
6 -0.740200 1.507790 0.113371
1 -1.317322 2.425790 0.124198
7 0.607934 1.648256 0.013285
7 0.865542 -0.711785 0.070314
8 2.675324 0.674471 -0.103658
6 -2.835153 0.166280 0.309316
1 -3.143863 -0.440264 1.189041
1 -3.306168 1.146081 0.505328
7 -0.947229 -2.104736 0.243854
1 -0.308607 -2.886843 0.182945
1 -1.938682 -2.294333 0.244074
8 -3.473416 -0.430206 -0.772091
6 1.233045 2.973510 -0.084207
1 1.916297 3.125145 0.753859
1 1.792375 3.050543 -1.018639
1 0.450884 3.731557 -0.060595
8 2.464091 -3.008723 -0.218755
1 1.947546 -2.171584 -0.095656
1 3.155561 -2.977975 0.457527
rd_1m5hmC/rd_1m5hmC_3_047.xyz
6 1.678204 -0.559310 -0.513589
6 -0.647994 -0.764056 -0.628806

6 -0.846073 0.558791 -0.098153
6 0.288387 1.263771 0.186923
1 0.247000 2.268726 0.592824
7 1.528331 0.746578 -0.013385
7 0.571299 -1.279061 -0.820519
8 2.838015 -1.011338 -0.660643
6 -2.202505 1.137314 0.192510
1 -2.761333 0.508021 0.923966
1 -2.129021 2.111878 0.705708
7 -1.703054 -1.546153 -0.930719
1 -1.538661 -2.451353 -1.350124
1 -2.644057 -1.181761 -0.943160
8 -3.069690 1.241224 -0.885399
6 2.737682 1.508732 0.323501
1 3.297226 0.992936 1.106720
1 3.371094 1.608438 -0.559835
1 2.442727 2.496818 0.675550
8 -0.717792 -1.272833 2.796595
1 -0.568690 -0.435484 2.332333
1 -0.942780 -1.895634 2.089643
rd_1m5hmU/rd_1m5hmU654.xyz
6 -1.223005 0.283232 -0.059091
6 0.458225 -1.347797 -0.027093
6 1.497355 -0.288927 -0.000534
6 1.084236 1.049651 0.008720
1 1.795356 1.866797 0.037142
7 -0.205404 1.346804 -0.013746
7 -0.851548 -0.995764 -0.060634
8 -2.394100 0.693557 -0.091882
8 0.823416 -2.544435 -0.018305
6 2.924775 -0.677207 0.027909
1 3.087759 -1.310881 0.915418
1 3.122329 -1.323282 -0.843010
8 3.767897 0.463753 0.037308
1 4.685831 0.156136 0.059043
6 -0.670470 2.737463 0.026499

1 -1.245806 2.949822 -0.876264
1 -1.314491 2.868933 0.897455
1 0.194804 3.394481 0.088277
8 -4.604226 -1.009330 0.104279
1 -3.775475 -0.491426 0.017279
1 -4.322250 -1.934723 0.115662
rd_1m5hmU/rd_1m5hmU786.xyz
6 1.222416 0.286075 0.071372
6 -0.451621 -1.351041 0.039694
6 -1.496390 -0.299121 -0.007827
6 -1.089248 1.040703 -0.020432
1 -1.805469 1.853524 -0.064557
7 0.198246 1.345133 0.015024
7 0.856854 -0.994301 0.080809
8 2.390902 0.703186 0.104898
8 -0.812725 -2.549245 0.038750
6 -2.932853 -0.692991 -0.057676
1 -3.087027 -1.309955 -0.952633
1 -3.144047 -1.347819 0.801061
8 -3.840961 0.394147 -0.128613
1 -3.901849 0.814099 0.742848
6 0.656723 2.737543 -0.030254
1 1.235691 2.953524 0.869252
1 1.296086 2.870045 -0.904619
1 -0.211676 3.390630 -0.088962
8 4.598967 -0.998209 -0.125131
1 4.318284 -1.923998 -0.136389
1 3.770682 -0.481909 -0.025316
rd_1m5hmU/rd_1m5hmU665.xyz
6 2.397686 0.314245 0.083197
6 0.381614 1.511373 -0.031043
6 -0.380100 0.240878 -0.109409
6 0.333377 -0.964504 -0.100424
1 -0.170694 -1.921671 -0.162329
7 1.653864 -0.957952 -0.005889
7 1.732384 1.472195 0.064095

8 3.626447 0.196117 0.171349
8 -0.250652 2.593167 -0.048679
6 -1.857627 0.283522 -0.169113
1 -2.212005 0.820830 0.727529
1 -2.144009 0.921663 -1.023255
8 -2.425783 -1.007544 -0.263933
1 -3.392929 -0.907857 -0.100131
6 2.430438 -2.202487 0.024017
1 3.109431 -2.219358 -0.830461
1 3.015299 -2.231479 0.944681
1 1.745479 -3.047748 -0.018019
8 -5.082213 -0.516401 0.216816
1 -5.287396 -0.681174 1.149697
1 -5.141633 0.446215 0.117082
rd_1m5hmU/rd_1m5hmU609.xyz
6 -2.407142 0.302026 0.061004
6 -0.399264 1.515622 -0.022054
6 0.374545 0.251471 -0.080246
6 -0.328594 -0.960006 -0.077070
1 0.184691 -1.913040 -0.124548
7 -1.650564 -0.964351 -0.006227
7 -1.751387 1.465533 0.047569
8 -3.636223 0.173551 0.127209
8 0.224103 2.602619 -0.034162
6 1.852237 0.306248 -0.116666
1 2.147145 0.939590 -0.971452
1 2.187060 0.854316 0.781260
8 2.431651 -0.980798 -0.191154
1 3.404222 -0.870365 -0.067245
6 -2.416976 -2.215444 0.014200
1 -3.020160 -2.245451 0.922916
1 -3.077701 -2.242367 -0.854266
1 -1.723741 -3.054655 -0.009641
8 5.112516 -0.553508 0.163214
1 5.305660 -0.406844 1.101670
1 5.301272 0.296117 -0.263951

rd_1m5hmU/rd_1m5hmU682.xyz
6 0.430891 -1.692750 -0.046394
6 -0.938418 0.209316 -0.052129
6 0.255469 1.085095 -0.020089
6 1.520477 0.483120 0.024230
1 2.429849 1.070387 0.068414
7 1.630341 -0.835454 0.017471
7 -0.783436 -1.131673 -0.074155
8 0.656869 -2.907641 -0.067478
8 -2.071845 0.751329 -0.057795
6 0.076340 2.553658 -0.016572
1 -0.512568 2.829851 -0.906927
1 -0.549416 2.818275 0.851977
8 1.327040 3.222074 0.014125
1 1.158652 4.175541 0.021082
6 2.938518 -1.497444 0.091733
1 2.977037 -2.100328 1.001029
1 3.057295 -2.150393 -0.774143
1 3.716663 -0.736370 0.105277
8 -4.427764 -0.700565 0.110029
1 -4.228277 -1.647173 0.117183
1 -3.551629 -0.257439 0.037172
rd_1m5hmU/rd_1m5hmU707.xyz
6 -2.074200 0.139489 0.219642
6 -0.290523 1.662967 0.145905
6 0.620338 0.593607 -0.330814
6 0.084564 -0.680620 -0.557288
1 0.693491 -1.493808 -0.935847
7 -1.194027 -0.919827 -0.315299
7 -1.586920 1.368388 0.408325
8 -3.237081 -0.215001 0.450292
8 0.171788 2.817692 0.296535
6 2.062356 0.898097 -0.555594
1 2.133188 1.642255 -1.361326
1 2.445807 1.400661 0.346610
8 2.855154 -0.217204 -0.913395

1 2.910434 -0.811301 -0.128435
6 -1.783883 -2.240823 -0.555939
1 -2.161907 -2.640511 0.386550
1 -2.612585 -2.136459 -1.258225
1 -1.018232 -2.896251 -0.966784
8 2.774670 -1.790119 1.352791
1 1.829485 -1.970287 1.473151
1 3.018790 -1.233481 2.107864
rd_1m5hmU/rd_1m5hmU771.xyz
6 -2.071638 0.161313 0.232617
6 -0.262987 1.654017 0.136574
6 0.624501 0.568190 -0.347218
6 0.064090 -0.696791 -0.566791
1 0.655259 -1.520188 -0.951096
7 -1.215969 -0.913378 -0.311851
7 -1.560895 1.381551 0.414992
8 -3.237623 -0.173298 0.476804
8 0.219562 2.801635 0.277981
6 2.067841 0.848902 -0.595651
1 2.137656 1.574948 -1.417972
1 2.470012 1.364672 0.290422
8 2.840451 -0.284975 -0.939631
1 2.911725 -0.850174 -0.135368
6 -1.834545 -2.220899 -0.554856
1 -2.222068 -2.613740 0.386532
1 -2.660611 -2.096272 -1.257091
1 -1.083880 -2.892636 -0.967228
8 2.758942 -1.742270 1.403742
1 3.233950 -1.282994 2.112651
1 1.821775 -1.657949 1.637933
rd_1m5hmU/rd_1m5hmU748.xyz
6 2.061463 -0.175784 0.216362
6 0.246460 -1.661659 0.134045
6 -0.642172 -0.570159 -0.334791
6 -0.078753 0.692691 -0.558506
1 -0.669545 1.519376 -0.936115

7 1.204806 0.903442 -0.316972
7 1.548509 -1.395208 0.398707
8 3.230711 0.154135 0.451283
8 -0.239058 -2.807887 0.276377
6 -2.090759 -0.842554 -0.559110
1 -2.178618 -1.581750 -1.367729
1 -2.483956 -1.340367 0.341535
8 -2.860037 0.290932 -0.911576
1 -2.900976 0.878694 -0.121487
6 1.825198 2.210073 -0.560304
1 2.220111 2.599031 0.379664
1 2.646202 2.085849 -1.268462
1 1.073293 2.885069 -0.965091
8 -2.690624 1.768513 1.416120
1 -1.799690 2.149080 1.378839
1 -2.636597 1.086190 2.102887
rd_1m5hmU/rd_1m5hmU652.xyz
6 2.406471 0.274081 0.087284
6 0.424543 1.526760 -0.027611
6 -0.371261 0.278411 -0.120059
6 0.308577 -0.946255 -0.109552
1 -0.221442 -1.889066 -0.174995
7 1.627828 -0.976301 -0.007350
7 1.773928 1.450432 0.070310
8 3.631179 0.120354 0.178729
8 -0.178665 2.625088 -0.036643
6 -1.847260 0.359149 -0.188917
1 -2.194819 0.876987 0.721897
1 -2.112955 1.029370 -1.024565
8 -2.443009 -0.915293 -0.325833
1 -3.403279 -0.818061 -0.122840
6 2.366645 -2.243285 0.031878
1 3.058288 -2.279248 -0.811746
1 2.936340 -2.291082 0.961312
1 1.656401 -3.066391 -0.023562
8 -5.086756 -0.607379 0.321748

1 -5.519794 0.006166 -0.290947
1 -5.119319 -0.162912 1.182582
rd_1m5hmU/rd_1m5hmU640.xyz
6 -1.249808 0.788834 0.095341
6 0.015876 -1.201901 0.132943
6 1.266743 -0.413693 0.014377
6 1.172843 0.983374 -0.040578
1 2.054298 1.609240 -0.115726
7 -0.009197 1.578344 0.006496
7 -1.170304 -0.544357 0.171574
8 -2.298994 1.440430 0.096288
8 0.083399 -2.448024 0.196542
6 2.563914 -1.123151 -0.034689
1 2.650622 -1.737604 0.876932
1 2.532927 -1.836984 -0.874516
8 3.643788 -0.212227 -0.158379
1 4.467311 -0.721961 -0.162198
6 -0.137406 3.040197 -0.037362
1 -0.715953 3.320060 -0.919626
1 -0.665029 3.374284 0.857555
1 0.858230 3.477456 -0.080611
8 -3.636441 -1.857415 -0.282839
1 -2.773055 -1.411068 -0.101940
1 -4.279832 -1.135399 -0.319436
rd_1m5hmU/rd_1m5hmU653.xyz
6 2.107389 0.016723 0.188488
6 0.443010 1.670717 0.181998
6 -0.564526 0.670075 -0.248767
6 -0.141864 -0.645560 -0.477983
1 -0.825258 -1.414954 -0.816395
7 1.124430 -0.978731 -0.290602
7 1.724658 1.281166 0.385235
8 3.248392 -0.425712 0.367964
8 0.073601 2.855909 0.348839
6 -1.969954 1.097030 -0.435761
1 -2.320374 1.561736 0.498846

1 -1.991692 1.888541 -1.201330
8 -2.792202 -0.002724 -0.809790
1 -3.688057 0.330304 -0.969378
6 1.600091 -2.342848 -0.542166
1 2.408871 -2.305067 -1.273870
1 1.979612 -2.767437 0.388869
1 0.770920 -2.937299 -0.921664
8 -2.850856 -1.949206 1.257394
1 -3.663177 -1.826468 1.768513
1 -2.890817 -1.269353 0.553866
rd_1m5hmU/rd_1m5hmU789.xyz
6 2.405266 0.253574 0.110291
6 0.446727 1.537304 -0.029013
6 -0.368215 0.303227 -0.141812
6 0.288155 -0.933511 -0.118686
1 -0.257295 -1.867460 -0.190993
7 1.604443 -0.985489 0.001447
7 1.792938 1.440048 0.087993
8 3.624526 0.075370 0.217588
8 -0.142711 2.642815 -0.041687
6 -1.849611 0.412778 -0.250769
1 -2.222984 0.937343 0.638163
1 -2.098775 1.050340 -1.110833
8 -2.519449 -0.840717 -0.314483
1 -2.441820 -1.203620 -1.210738
6 2.320554 -2.264662 0.048465
1 3.031480 -2.304919 -0.778780
1 2.867810 -2.328076 0.990482
1 1.598319 -3.075112 -0.030715
8 -5.235778 -0.541810 0.376141
1 -5.239888 -0.285252 1.309078
1 -4.288469 -0.642677 0.146928
rd_1m5hmU/rd_1m5hmU799.xyz
6 -2.061000 0.184493 0.166883
6 -0.242425 1.667950 0.149772
6 0.660467 0.576347 -0.291923

6 0.111232 -0.695962 -0.498866
1 0.716052 -1.528916 -0.838195
7 -1.177775 -0.908544 -0.290015
7 -1.555485 1.405635 0.358085
8 -3.243681 -0.137282 0.337137
8 0.240783 2.811609 0.317911
6 2.106523 0.866389 -0.509984
1 2.185204 1.627145 -1.298943
1 2.495748 1.345854 0.402529
8 2.890336 -0.247000 -0.894142
1 2.927465 -0.864183 -0.128005
6 -1.783503 -2.225243 -0.515669
1 -2.261287 -2.560760 0.405883
1 -2.541031 -2.137152 -1.296997
1 -1.004833 -2.922612 -0.818862
8 2.594286 -1.803566 1.382720
1 1.985489 -2.498864 1.089252
1 2.028454 -1.174091 1.856549
rd_1m5hmU/rd_1m5hmU615.xyz
6 2.423402 0.245620 -0.042990
6 0.461080 1.533418 0.006813
6 -0.360070 0.299617 0.062974
6 0.297100 -0.937491 0.059493
1 -0.251440 -1.871205 0.093046
7 1.618438 -0.991027 0.005191
7 1.811936 1.433191 -0.040281
8 3.647766 0.069385 -0.085765
8 -0.121942 2.642500 0.002279
6 -1.835491 0.406338 0.094657
1 -2.110728 1.071282 0.931321
1 -2.150339 0.940795 -0.818822
8 -2.454742 -0.859618 0.201274
1 -3.424911 -0.732888 0.073112
6 2.335402 -2.271097 -0.019663
1 2.933883 -2.323071 -0.930710
1 2.998144 -2.325210 0.845832

1 1.608846 -3.081337 0.006590
8 -5.125393 -0.450709 -0.219055
1 -5.613594 -0.458748 0.618175
1 -5.496523 -1.185668 -0.730475
rd_1m5hmU/rd_1m5hmU1256.xyz
6 1.961194 0.683084 0.338079
6 -0.256210 1.163837 -0.248396
6 -0.486958 -0.253582 -0.611174
6 0.601575 -1.135629 -0.536725
1 0.506051 -2.177482 -0.826503
7 1.777441 -0.716273 -0.104594
7 0.946924 1.547552 0.227547
8 3.081747 0.947484 0.785053
8 -1.197405 1.985490 -0.409516
6 -1.787800 -0.731714 -1.167386
1 -1.718563 -0.619873 -2.266083
1 -1.916426 -1.799914 -0.966901
8 -2.934353 -0.051695 -0.671320
1 -2.695965 0.899317 -0.630248
6 2.928347 -1.622712 -0.002783
1 3.762824 -1.198482 -0.562521
1 3.213778 -1.717271 1.046553
1 2.649716 -2.593481 -0.409493
8 -3.130848 -0.851079 2.020006
1 -3.116875 -0.552755 1.085494
1 -4.069104 -0.920324 2.245563
rd_1m5hmU/rd_1m5hmU612.xyz
6 -2.094175 -0.045326 0.165691
6 -0.413470 -1.682747 0.176687
6 0.591799 -0.669206 -0.230269
6 0.159101 0.643294 -0.459217
1 0.839545 1.421927 -0.782666
7 -1.113437 0.962735 -0.291285
7 -1.702295 -1.307265 0.360159
8 -3.242117 0.384969 0.329675
8 -0.034743 -2.864896 0.343051

6 2.004068 -1.083758 -0.392262
1 2.343800 -1.534069 0.553530
1 2.044581 -1.884675 -1.147015
8 2.823491 0.017635 -0.767065
1 3.725710 -0.309496 -0.901446
6 -1.598153 2.323689 -0.542872
1 -2.397713 2.282059 -1.284389
1 -1.992469 2.741175 0.385226
1 -0.769613 2.926820 -0.909964
8 2.846971 1.980900 1.288074
1 2.889034 1.302855 0.583223
1 1.983405 2.400572 1.164946
rd_1m5hmU/rd_1m5hmU646.xyz
6 -1.878963 -1.027265 -0.024651
6 0.463247 -0.966780 -0.126863
6 0.440045 0.514428 -0.071068
6 -0.802063 1.154525 0.038984
1 -0.884639 2.233055 0.099940
7 -1.917666 0.443676 0.073800
7 -0.700221 -1.652500 -0.112647
8 -2.983176 -1.582909 -0.014733
8 1.569895 -1.556703 -0.191079
6 1.713885 1.264613 -0.113101
1 2.313673 0.960581 0.761521
1 2.281323 0.932957 -0.997607
8 1.487841 2.664834 -0.127942
1 2.349356 3.107148 -0.110457
6 -3.229906 1.086807 0.206132
1 -3.837122 0.843339 -0.667452
1 -3.722560 0.703955 1.101384
1 -3.084147 2.162659 0.281304
8 4.264047 -0.980800 0.316616
1 4.648401 -1.867524 0.360966
1 3.314589 -1.127739 0.122837
rd_1m5hmU/rd_1m5hmU726.xyz
6 -2.095542 0.049394 0.197602

6 -0.400255 1.670999 0.164573
6 0.583341 0.653439 -0.281120
6 0.129520 -0.652321 -0.506685
1 0.794903 -1.432778 -0.858288
7 -1.139884 -0.962632 -0.299858
7 -1.686527 1.306374 0.388461
8 -3.241715 -0.371917 0.397027
8 -0.007231 2.849814 0.324921
6 2.009924 1.041141 -0.474797
1 2.048850 1.827395 -1.241235
1 2.358680 1.517035 0.456050
8 2.862817 -0.012016 -0.879276
1 2.953927 -0.642592 -0.126151
6 -1.644254 -2.317953 -0.542101
1 -2.014675 -2.735182 0.395915
1 -2.464611 -2.267637 -1.259958
1 -0.832249 -2.927051 -0.935156
8 2.871045 -1.697556 1.295311
1 2.022574 -2.163905 1.242424
1 3.541751 -2.396269 1.257490
rd_1m5hmU/rd_1m5hmU756.xyz
6 1.234633 0.816719 0.087585
6 0.011526 -1.200229 0.090205
6 -1.256808 -0.439495 -0.014335
6 -1.191566 0.958842 -0.065624
1 -2.088617 1.562597 -0.145734
7 -0.023898 1.579978 -0.012071
7 1.183531 -0.518521 0.147293
8 2.267759 1.492485 0.108679
8 -0.029657 -2.448804 0.124831
6 -2.550670 -1.177045 -0.069971
1 -2.550973 -1.799810 -0.974743
1 -2.586996 -1.877314 0.777856
8 -3.701488 -0.349667 -0.121863
1 -3.855422 0.033712 0.755336
6 0.073111 3.044015 -0.059080

1 0.591283 3.390989 0.836373
1 0.649032 3.333133 -0.940232
1 -0.931164 3.460613 -0.106843
8 3.666424 -1.827487 -0.195660
1 2.797796 -1.375722 -0.056323
1 4.311978 -1.107463 -0.232817
rd_1m5hmU/rd_1m5hmU749.xyz
6 0.593200 -1.670140 -0.037593
6 -0.939956 0.100639 -0.043067
6 0.170188 1.082089 -0.025389
6 1.483701 0.596482 0.009741
1 2.336972 1.265053 0.037894
7 1.711810 -0.706891 0.013526
7 -0.666617 -1.221286 -0.057457
8 0.928291 -2.859182 -0.056545
8 -2.116319 0.542058 -0.047899
6 -0.141362 2.539458 -0.032036
1 -0.748685 2.755573 -0.920517
1 -0.784353 2.758830 0.833982
8 0.994620 3.387868 -0.073655
1 1.403740 3.415953 0.804680
6 3.073668 -1.247387 0.085418
1 3.160820 -1.859938 0.984687
1 3.257328 -1.872093 -0.790053
1 3.778530 -0.418562 0.117438
8 -4.374093 -1.068265 0.071128
1 -3.551446 -0.530182 0.021488
1 -5.065887 -0.444082 0.331246
rd_1m5mC/rd_1m5mC_2_078.xyz
6 0.887530 0.322037 -0.012134
6 -0.909565 -1.187165 -0.000439
6 -1.887545 -0.127096 -0.005476
6 -1.379730 1.164577 -0.005470
1 -2.028579 2.033370 -0.002254
7 -0.058246 1.397550 -0.005722
7 0.430825 -0.932881 -0.007521

8 2.102384 0.638904 -0.019562
7 -1.357787 -2.444728 0.013480
1 -0.544820 -3.071110 0.020118
6 -3.362502 -0.373085 -0.002122
1 -3.661610 -0.962605 -0.874886
1 -3.660338 -0.939804 0.886299
1 -3.911284 0.571569 -0.013583
6 0.469548 2.766471 0.021675
1 1.080783 2.894359 0.917193
1 1.094840 2.929877 -0.857897
1 -0.363694 3.466874 0.028749
8 4.034085 -1.337229 0.003104
1 3.589339 -2.194800 0.053665
1 3.303648 -0.675143 -0.003604
rd_1m5mC/rd_1m5mC_2_003.xyz
6 -0.629945 1.008489 0.002193
6 0.058797 -1.251692 0.001094
6 1.456374 -0.892145 -0.003360
6 1.735329 0.470508 -0.003901
1 2.754403 0.839994 -0.007134
7 0.760358 1.387759 -0.000900
7 -0.920478 -0.298140 0.003179
8 -1.475812 1.925737 0.003980
7 -0.250989 -2.545902 0.003172
1 -1.273120 -2.635000 0.008871
6 2.559056 -1.899864 -0.000488
1 2.523842 -2.508370 0.909821
1 2.462874 -2.585724 -0.847992
1 3.533056 -1.408177 -0.053892
6 1.069975 2.821627 -0.000422
1 0.631893 3.283688 -0.887167
1 0.636935 3.281666 0.889876
1 2.150900 2.950855 -0.003245
8 -3.676986 -0.904604 -0.006068
1 -2.708812 -0.696779 -0.002470
1 -4.109341 -0.038779 0.001188

rd_1m5mC/rd_1m5mC_1_090.xyz
6 -0.837643 -1.234805 -0.007549
6 0.856307 0.390566 -0.006866
6 -0.097068 1.482126 -0.002052
6 -1.438319 1.117119 0.003802
1 -2.229751 1.858286 0.009450
7 -1.811356 -0.166537 0.003408
7 0.458287 -0.911650 -0.010198
8 -1.286052 -2.400817 -0.013367
7 2.170744 0.609498 -0.007935
1 2.381708 1.609294 -0.005835
6 0.299157 2.923804 -0.000400
1 0.901557 3.165255 0.883319
1 0.900519 3.167996 -0.884004
1 -0.583554 3.566918 0.001268
6 -3.229881 -0.537672 0.014473
1 -3.458213 -1.114937 -0.883779
1 -3.431614 -1.157108 0.890262
1 -3.832779 0.368386 0.044945
8 4.177839 -1.363451 0.008951
1 3.732229 -2.220051 0.070784
1 3.446570 -0.695900 0.003532
rd_1m5mC/rd_1m5mC_2_079.xyz
6 -0.739250 -0.469574 -0.513686
6 1.512097 -0.933858 -0.040273
6 1.821436 0.455158 0.190246
6 0.760948 1.340827 0.059762
1 0.886504 2.405629 0.221199
7 -0.468665 0.918112 -0.271277
7 0.257837 -1.347489 -0.383904
8 -1.914527 -0.774818 -0.834524
7 2.499740 -1.821026 0.098756
1 2.116178 -2.753761 -0.090078
6 3.190968 0.937999 0.546396
1 3.547047 0.457166 1.462997
1 3.905545 0.689480 -0.245419

1 3.193884 2.020594 0.693497
6 -1.585533 1.862980 -0.400547
1 -1.994449 1.795968 -1.410343
1 -2.364125 1.597622 0.316397
1 -1.221992 2.870452 -0.206583
8 -3.921311 -0.534930 1.103489
1 -3.953669 -1.405810 1.523553
1 -3.214599 -0.607729 0.426657
rd_1m5mC/rd_1m5mC_2_020.xyz
6 0.732526 -0.482019 -0.501917
6 -1.522518 -0.931620 -0.032146
6 -1.826237 0.460799 0.185045
6 -0.761278 1.340429 0.050345
1 -0.882573 2.407199 0.201664
7 0.467261 0.909195 -0.273467
7 -0.269054 -1.354179 -0.367650
8 1.907429 -0.795152 -0.815946
7 -2.514957 -1.812787 0.111039
1 -2.135275 -2.749094 -0.067638
6 -3.194845 0.953505 0.531071
1 -3.907063 0.702419 -0.262086
1 -3.557946 0.482249 1.449873
1 -3.192714 2.037292 0.669206
6 1.589099 1.847128 -0.408932
1 2.371228 1.573828 0.301215
1 1.990636 1.779728 -1.421823
1 1.233771 2.856638 -0.210325
8 3.876303 -0.617142 1.180663
1 4.719645 -0.474965 0.728259
1 3.207193 -0.641880 0.463676
rd_1m5mC/rd_1m5mC_2_088.xyz
6 -0.734131 -0.496221 -0.509731
6 1.521940 -0.925339 -0.026306
6 1.813781 0.470589 0.184154
6 0.742598 1.340970 0.039755
1 0.855108 2.409455 0.185538

7 -0.481200 0.898249 -0.286906
7 0.273571 -1.359665 -0.365833
8 -1.904768 -0.820284 -0.828573
7 2.520669 -1.797730 0.126962
1 2.149697 -2.738349 -0.047312
6 3.176687 0.976355 0.533699
1 3.535822 0.519803 1.461537
1 3.896485 0.719180 -0.250497
1 3.166739 2.061829 0.657510
6 -1.609323 1.827411 -0.430328
1 -2.020061 1.738367 -1.437624
1 -2.383635 1.565929 0.292802
1 -1.257262 2.842284 -0.254728
8 -3.898665 -0.683166 1.136898
1 -3.973944 0.241020 1.413177
1 -3.202079 -0.680485 0.445980
rd_1m5mC/rd_1m5mC_2_073.xyz
6 0.741948 -0.493553 -0.533757
6 -1.510615 -0.922403 -0.034925
6 -1.798130 0.472519 0.188044
6 -0.727293 1.342631 0.038571
1 -0.837250 2.410340 0.191892
7 0.493098 0.900150 -0.300943
7 -0.265938 -1.356323 -0.388347
8 1.910562 -0.817217 -0.860856
7 -2.509427 -1.794123 0.120841
1 -2.141937 -2.734063 -0.064207
6 -3.156246 0.976703 0.557861
1 -3.887461 0.719970 -0.215727
1 -3.500797 0.517711 1.490114
1 -3.145541 2.061883 0.684066
6 1.621629 1.828243 -0.449881
1 2.408159 1.554631 0.255584
1 2.015339 1.752367 -1.465140
1 1.275640 2.841519 -0.254120
8 3.835232 -0.684116 1.186231

1 3.169370 -0.700648 0.466297
1 3.626239 0.114180 1.691913
rd_1m5mC/rd_1m5mC_2_019.xyz
6 0.730452 -0.504358 -0.500007
6 -1.528730 -0.923215 -0.022230
6 -1.816214 0.474667 0.181065
6 -0.741221 1.340388 0.037122
1 -0.850320 2.409893 0.177830
7 0.481975 0.891892 -0.283778
7 -0.280802 -1.363557 -0.355669
8 1.900694 -0.834205 -0.814483
7 -2.531231 -1.791196 0.131392
1 -2.163309 -2.734038 -0.037187
6 -3.178582 0.987451 0.522336
1 -3.896481 0.727392 -0.262628
1 -3.542844 0.538889 1.452101
1 -3.165157 2.073726 0.638520
6 1.613901 1.816539 -0.426868
1 2.382343 1.558032 0.303596
1 2.030832 1.718674 -1.430765
1 1.263592 2.833697 -0.261262
8 3.904382 -0.669210 1.131227
1 4.128690 0.264348 1.250715
1 3.204817 -0.672084 0.443011
rd_1m5mC/rd_1m5mC_2_023.xyz
6 0.670668 -0.607694 -0.226336
6 -1.646309 -0.905146 0.013167
6 -1.890604 0.513818 0.086699
6 -0.769640 1.325126 -0.010432
1 -0.844561 2.405887 0.031654
7 0.460396 0.808820 -0.161493
7 -0.391059 -1.415502 -0.141974
8 1.850546 -1.011272 -0.365055
7 -2.699561 -1.721329 0.104917
1 -2.358898 -2.687113 0.040104
6 -3.254117 1.102941 0.259559

1 -3.915021 0.803155 -0.560319
1 -3.714894 0.751764 1.188612
1 -3.202295 2.193987 0.285882
6 1.636720 1.682469 -0.257336
1 2.270400 1.528187 0.618239
1 2.200990 1.427183 -1.155306
1 1.306168 2.718620 -0.300847
8 4.384894 -0.234688 0.514877
1 3.478430 -0.460678 0.215290
1 4.827425 -1.086318 0.636042
rd_1m5mC/rd_1m5mC_1_082.xyz
6 -0.750664 -0.467608 -0.518083
6 1.498290 -0.949506 -0.052734
6 1.813560 0.440323 0.182109
6 0.756476 1.328886 0.064211
1 0.885596 2.392313 0.231907
7 -0.477397 0.913111 -0.263706
7 0.242929 -1.351632 -0.393863
8 -1.926198 -0.769910 -0.843661
7 2.412897 -1.922412 0.056605
1 3.319229 -1.520296 0.311110
6 3.185982 0.926538 0.534799
1 3.531158 0.485023 1.476923
1 3.909968 0.653611 -0.241540
1 3.193142 2.013189 0.644534
6 -1.590933 1.863168 -0.381687
1 -1.991854 1.819042 -1.396104
1 -2.376061 1.583018 0.322441
1 -1.227462 2.865471 -0.162116
8 -3.952491 -0.576362 1.077751
1 -3.230402 -0.627963 0.415041
1 -3.709070 -1.217510 1.760139
rd_1m5mC/rd_1m5mC_1_015.xyz
6 -0.904986 0.275301 -0.183509
6 0.945466 -1.165764 -0.101431
6 1.871118 -0.069330 0.067243

6 1.308305 1.196867 0.104256
1 1.914164 2.087207 0.228869
7 -0.016442 1.381267 -0.013328
7 -0.394976 -0.959238 -0.219971
8 -2.129015 0.539717 -0.288743
7 1.344548 -2.444364 -0.152066
1 2.362380 -2.496379 -0.055064
6 3.352686 -0.249604 0.199658
1 3.600127 -0.845197 1.086144
1 3.766595 -0.769580 -0.671861
1 3.851774 0.717795 0.289141
6 -0.598565 2.728357 0.035270
1 -1.123319 2.929304 -0.900763
1 -1.312582 2.781366 0.859325
1 0.200295 3.453409 0.181485
8 -3.976695 -1.411654 0.341530
1 -3.514701 -2.256548 0.433846
1 -3.275106 -0.764488 0.095228
rd_1m5mC/rd_1m5mC_2_011.xyz
6 -1.689386 -0.912424 -0.000006
6 0.643772 -0.648811 0.000003
6 0.552148 0.795556 -0.000003
6 -0.736393 1.323465 0.000003
1 -0.910334 2.393886 0.000008
7 -1.812478 0.530966 0.000005
7 -0.464246 -1.443685 -0.000002
8 -2.754772 -1.563409 -0.000014
7 1.856810 -1.192719 0.000015
1 1.763044 -2.213276 0.000031
6 1.746501 1.691329 -0.000011
1 2.370641 1.503768 0.880301
1 2.370625 1.503771 -0.880336
1 1.443201 2.740444 -0.000006
6 -3.168565 1.089962 0.000010
1 -3.697937 0.739532 -0.888139
1 -3.697937 0.739523 0.888155

1 -3.105274 2.176554 0.000016
8 4.529477 -0.310803 -0.000003
1 3.567939 -0.543125 0.000004
1 4.549326 0.656227 0.000003
rd_1m5mC/rd_1m5mC_2_052.xyz
6 -0.184405 -1.217356 -0.054419
6 -0.422231 1.132478 -0.048676
6 1.005065 1.340663 0.006024
6 1.789475 0.192247 0.035754
1 2.871515 0.247119 0.084159
7 1.245413 -1.030277 0.006438
7 -0.956780 -0.125211 -0.078486
8 -0.607736 -2.390176 -0.078442
7 -1.205385 2.207725 -0.070360
1 -2.182606 1.897997 -0.109358
6 1.629063 2.697539 0.036347
1 1.336737 3.279053 -0.844089
1 1.294769 3.258044 0.915749
1 2.718455 2.623124 0.062024
6 2.080604 -2.236166 0.055355
1 1.819544 -2.816802 0.942428
1 1.888616 -2.841902 -0.832112
1 3.128428 -1.942785 0.092138
8 -3.767465 -0.435787 0.090800
1 -2.782837 -0.359730 0.027338
1 -4.059167 0.441503 0.377400
rd_1m5mC/rd_1m5mC_1_098.xyz
6 -0.670309 -0.626344 -0.054986
6 1.658374 -0.919624 0.004400
6 1.901881 0.503997 0.020687
6 0.775118 1.309064 -0.000510
1 0.847548 2.390962 0.009097
7 -0.462796 0.787589 -0.037089
7 0.398253 -1.430995 -0.034867
8 -1.854345 -1.041314 -0.090950
7 2.652918 -1.819254 0.027486

1 3.556236 -1.337850 0.057529
6 3.271946 1.109781 0.056249
1 3.821117 0.792024 0.950045
1 3.860316 0.806350 -0.817396
1 3.209764 2.200100 0.063417
6 -1.647496 1.654716 -0.060284
1 -2.237212 1.433842 -0.951661
1 -2.254147 1.459167 0.825880
1 -1.327530 2.695260 -0.070598
8 -4.500618 -0.198442 0.118450
1 -4.945875 -1.055442 0.176453
1 -3.546217 -0.417281 0.055184
rd_1m5mC/rd_1m5mC_2_090.xyz
6 -1.677699 -0.928768 0.000235
6 0.649683 -0.620785 -0.008080
6 0.530255 0.821452 -0.005573
6 -0.767694 1.325464 0.001660
1 -0.961235 2.392532 0.004560
7 -1.828469 0.512729 0.004228
7 -0.442958 -1.437125 -0.005543
8 -2.731167 -1.598822 0.002487
7 1.874320 -1.137064 -0.012827
1 1.805506 -2.159457 -0.013832
6 1.709618 1.736183 -0.010010
1 2.338877 1.559519 0.868590
1 2.334180 1.557269 -0.891485
1 1.389816 2.780359 -0.010312
6 -3.194957 1.045666 0.009446
1 -3.720224 0.686337 -0.877590
1 -3.714919 0.683784 0.898562
1 -3.153062 2.133464 0.010766
8 4.525614 -0.158376 0.005510
1 5.030622 -0.975671 0.120535
1 3.579371 -0.445603 -0.000848
rd_1m5mC/rd_1m5mC_2_063.xyz
6 -1.460389 0.765560 -0.229121

6 0.822362 1.110098 0.213393
6 0.981929 -0.203054 0.787952
6 -0.154496 -0.998787 0.802286
1 -0.135462 -2.008076 1.197862
7 -1.325514 -0.557696 0.319772
7 -0.375654 1.550408 -0.263445
8 -2.592390 1.099574 -0.642322
7 1.900627 1.896899 0.159300
1 1.621228 2.783762 -0.274977
6 2.287429 -0.718573 1.303416
1 3.045631 -0.704915 0.513717
1 2.662080 -0.092859 2.120074
1 2.180909 -1.743014 1.668120
6 -2.516212 -1.414988 0.321573
1 -3.304756 -0.934478 0.904060
1 -2.865752 -1.544155 -0.704656
1 -2.260478 -2.380139 0.756302
8 1.877558 -1.404063 -2.286186
1 2.133108 -0.542001 -1.925889
1 1.482196 -1.867030 -1.532923
rd_1m5mC/rd_1m5mC_1_086.xyz
6 -0.458438 -1.132490 0.000016
6 -0.172356 1.211264 0.000012
6 1.269148 1.094528 0.000003
6 1.778820 -0.198869 -0.000008
1 2.846935 -0.386584 -0.000024
7 0.974889 -1.269650 0.000001
7 -0.969410 0.104312 0.000011
8 -1.135273 -2.180758 0.000022
7 -0.802552 2.387792 0.000017
1 -0.118832 3.148686 0.000017
6 2.188230 2.275795 -0.000010
1 2.022128 2.902417 -0.883936
1 2.022076 2.902487 0.883856
1 3.230875 1.951729 0.000034
6 1.519806 -2.631723 -0.000014

1 1.164461 -3.157493 0.888358
1 1.164436 -3.157485 -0.888381
1 2.607038 -2.579626 -0.000031
8 -3.780796 0.264684 -0.000027
1 -3.992302 1.208801 -0.000028
1 -2.790015 0.227443 -0.000020
rd_1m5mC/rd_1m5mC_2_030.xyz
6 -0.043129 -1.297045 -0.030446
6 2.127995 -0.400477 0.036669
6 1.645898 0.958388 -0.011180
6 0.267514 1.109758 -0.052677
1 -0.198623 2.088250 -0.080005
7 -0.552803 0.048228 -0.057178
7 1.283749 -1.470529 0.023104
8 -0.881254 -2.224617 -0.056170
7 3.449679 -0.583794 0.097316
1 3.627822 -1.593957 0.127005
6 2.552272 2.147367 -0.003710
1 3.150832 2.174342 0.913126
1 3.254198 2.109218 -0.842848
1 1.975799 3.073298 -0.069808
6 -2.009652 0.217801 -0.080835
1 -2.414114 -0.263192 -0.973064
1 -2.436442 -0.260285 0.803472
1 -2.243747 1.280525 -0.085841
8 -5.392939 0.789391 0.063843
1 -6.227336 0.302434 0.000371
1 -4.804610 0.199089 0.556594
rd_1m5mC/rd_1m5mC_1_031.xyz
6 0.741720 -0.495189 -0.497918
6 -1.513019 -0.945736 -0.029181
6 -1.816906 0.452335 0.168607
6 -0.749217 1.327094 0.043932
1 -0.868689 2.394788 0.189889
7 0.482793 0.892315 -0.265816
7 -0.260340 -1.366977 -0.356969

8 1.913424 -0.814402 -0.820683
7 -2.435721 -1.908075 0.104716
1 -3.340067 -1.491951 0.343490
6 -3.187601 0.962653 0.492166
1 -3.913128 0.645593 -0.265502
1 -3.534480 0.581039 1.459642
1 -3.190690 2.054032 0.535385
6 1.607338 1.828610 -0.389708
1 2.381096 1.555877 0.329820
1 2.020060 1.760568 -1.397905
1 1.251528 2.838619 -0.194357
8 3.947741 -0.686140 1.089462
1 3.229496 -0.676543 0.420417
1 4.074540 0.242872 1.327988
rd_1m5mC/rd_1m5mC_2_062.xyz
6 1.629587 -0.520351 0.251857
6 -0.493008 -1.523073 0.144223
6 -1.076294 -0.381637 -0.516557
6 -0.217142 0.680068 -0.763666
1 -0.557504 1.584426 -1.255286
7 1.074912 0.630104 -0.410393
7 0.822072 -1.559099 0.503267
8 2.843171 -0.472405 0.551058
7 -1.290600 -2.563847 0.398653
1 -0.727840 -3.283609 0.865939
6 -2.515871 -0.312641 -0.915833
1 -2.773382 -1.131806 -1.594844
1 -3.167549 -0.403610 -0.040289
1 -2.732467 0.635512 -1.414516
6 1.975909 1.756946 -0.676014
1 2.371095 2.131929 0.270329
1 2.808764 1.415535 -1.293367
1 1.423399 2.541719 -1.189584
8 -1.829537 2.674416 1.251368
1 -2.431886 3.105217 1.875162
1 -2.385475 2.042619 0.772305

rd_1m5mU/rd_1m5mU18.xyz
7 1.834970 -0.107277 0.000687
6 0.862330 -1.216349 -0.000521
7 -0.447100 -0.945617 -0.001661
6 -0.903526 0.326490 -0.001392
6 0.055069 1.459740 0.000136
6 1.424883 1.149788 0.000943
8 -2.132268 0.588701 -0.002633
8 1.359300 -2.348548 -0.000424
6 3.263280 -0.445500 0.001968
1 2.180649 1.928633 0.001900
1 3.841120 0.477221 0.004079
1 3.488706 -1.038232 0.889952
1 3.491013 -1.035604 -0.887202
6 -0.423403 2.856755 -0.000198
1 -1.057545 3.032360 -0.879425
1 -1.069708 3.029315 0.870541
1 0.401315 3.571295 0.006127
8 -4.033254 -1.447903 -0.005400
1 -4.871623 -0.970070 0.064743
1 -3.341049 -0.748196 -0.001852
rd_1m5mU/rd_1m5mU12.xyz
7 -1.397568 -0.857797 -0.000677
6 0.027735 -1.231218 0.001023
7 0.952958 -0.265766 0.001282
6 0.622949 1.052102 0.000341
6 -0.810209 1.442679 -0.001090
6 -1.766195 0.413148 -0.001578
8 1.497446 1.945058 0.000935
8 0.260287 -2.444464 0.002090
6 -2.383656 -1.944409 -0.000682
1 -2.829357 0.631374 -0.002729
1 -3.381982 -1.511303 -0.004392
1 -2.231405 -2.561732 -0.887485
1 -2.236053 -2.557476 0.889891
6 -1.198456 2.867225 0.000167

1 -0.779224 3.365364 0.884795
1 -0.753465 3.375613 -0.865354
1 -2.281871 2.996161 -0.013587
8 3.714508 -0.853993 -0.002010
1 4.131250 0.019491 -0.001859
1 2.743446 -0.662526 -0.000725
rd_1m5mU/rd_1m5mU43.xyz
7 -1.817994 -0.126475 0.005120
6 -0.825093 -1.217583 -0.008444
7 0.479128 -0.922964 -0.010004
6 0.911952 0.357691 -0.004891
6 -0.067467 1.472969 -0.006095
6 -1.431140 1.137853 0.002599
8 2.135476 0.642876 0.000694
8 -1.301535 -2.358519 -0.016805
6 -3.239538 -0.490884 0.018943
1 -2.200947 1.902714 0.007927
1 -3.833943 0.420407 0.057307
1 -3.471848 -1.059590 -0.883199
1 -3.438922 -1.113170 0.892514
6 0.383556 2.879016 -0.007756
1 0.979261 3.078220 0.893728
1 1.058769 3.055463 -0.854735
1 -0.455125 3.575952 -0.050134
8 4.084338 -1.342112 0.018844
1 3.351945 -0.684479 0.011008
1 3.643020 -2.201783 -0.028218
rd_1m5mU/rd_1m5mU24.xyz
7 -0.806798 1.378609 -0.000019
6 0.616480 1.001114 0.000008
7 0.945811 -0.295013 -0.000021
6 0.009669 -1.279983 -0.000019
6 -1.430148 -0.912993 -0.000008
6 -1.753844 0.454024 -0.000013
8 0.330089 -2.487789 -0.000023
8 1.418782 1.941091 0.000055

6 -1.128492 2.810499 -0.000021
1 -2.785739 0.788793 -0.000016
1 -2.210661 2.928115 -0.000109
1 -0.695698 3.271553 0.889312
1 -0.695551 3.271583 -0.889264
6 -2.472944 -1.958892 0.000038
1 -2.346025 -2.609822 -0.875185
1 -2.346163 -2.609575 0.875470
1 -3.478230 -1.534827 -0.000090
8 3.711040 -0.845996 -0.000001
1 2.738212 -0.661623 -0.000007
1 4.123144 0.029569 0.000012
rd_1m5mU/rd_1m5mU100.xyz
7 -0.074600 1.410474 -0.007650
6 0.878538 0.288902 -0.030394
7 0.430320 -0.964251 -0.028956
6 -0.899250 -1.239948 -0.009071
6 -1.882145 -0.125099 -0.001198
6 -1.378604 1.186376 0.002936
8 -1.327138 -2.415675 0.002720
8 2.073545 0.627075 -0.048483
6 0.471701 2.772204 0.027588
1 -2.040185 2.046647 0.018174
1 -0.354233 3.481064 0.037077
1 1.081958 2.882804 0.925683
1 1.099797 2.928141 -0.850717
6 -3.333888 -0.396565 0.015994
1 -3.604246 -1.045005 -0.827095
1 -3.594551 -0.961327 0.921801
1 -3.921089 0.522414 -0.020115
8 4.090403 -1.336887 0.034921
1 3.353484 -0.690276 0.004699
1 3.656428 -2.199350 0.098337
rd_1m5mU/rd_1m5mU81.xyz
7 -1.281756 0.532380 -0.378019
6 -1.294489 -0.886045 0.034766

7 -0.158059 -1.586804 0.025659
6 1.022953 -1.013548 -0.314946
6 1.065646 0.424750 -0.686588
6 -0.148740 1.129385 -0.705987
8 2.094906 -1.661886 -0.315950
8 -2.406200 -1.322767 0.360136
6 -2.560163 1.252149 -0.391325
1 -0.189389 2.177717 -0.983587
1 -2.383064 2.275691 -0.716677
1 -3.244619 0.747128 -1.074910
1 -2.985832 1.239995 0.613275
6 2.351527 1.093767 -0.971165
1 2.979019 1.074034 -0.068492
1 2.909096 0.539240 -1.735588
1 2.211317 2.128816 -1.287577
8 1.002569 0.910002 2.658751
1 1.085154 1.607156 1.991793
1 1.546421 0.185645 2.316257
rd_1m5mU/rd_1m5mU53.xyz
7 -0.479020 0.859467 -0.288841
6 -0.678724 -0.594615 -0.437077
7 0.354695 -1.421370 -0.299769
6 1.597397 -0.959747 -0.003710
6 1.819691 0.498258 0.172696
6 0.714089 1.348335 0.007895
8 2.570415 -1.735242 0.128626
8 -1.843492 -0.940988 -0.692236
6 -1.644156 1.735363 -0.463629
1 0.809828 2.424069 0.114220
1 -1.312126 2.771394 -0.432255
1 -2.114153 1.511950 -1.421813
1 -2.356844 1.541648 0.340027
6 3.160659 1.026285 0.496336
1 3.532522 0.562807 1.419363
1 3.871606 0.738033 -0.289614
1 3.155613 2.112063 0.605386

8 -4.091471 -0.551667 1.022576
1 -4.302050 0.392721 1.039605
1 -3.311472 -0.621468 0.438545
rd_1m5mU/rd_1m5mU60.xyz
7 -1.412364 0.613037 -0.016694
6 -1.391612 -0.862511 -0.095187
7 -0.252287 -1.489854 -0.394293
6 0.889738 -0.804729 -0.645798
6 0.877511 0.681863 -0.628886
6 -0.324163 1.317762 -0.278260
8 1.970514 -1.390496 -0.887160
8 -2.475334 -1.413073 0.137773
6 -2.672272 1.259624 0.366980
1 -0.393571 2.398620 -0.212746
1 -2.522738 2.337966 0.382772
1 -3.445148 0.991555 -0.354913
1 -2.967284 0.901353 1.354800
6 2.107309 1.450250 -0.909143
1 2.884289 1.173834 -0.183252
1 2.506482 1.175957 -1.893960
1 1.934197 2.526867 -0.866138
8 2.118686 -0.135694 2.488941
1 1.340825 0.372102 2.214664
1 2.485514 -0.479983 1.661010
rd_1m5mU/rd_1m5mU91.xyz
7 -0.450067 0.812924 -0.000006
6 -0.635710 -0.649963 -0.000006
7 0.427542 -1.451423 -0.000005
6 1.690925 -0.954487 -0.000002
6 1.903793 0.515991 0.000001
6 0.765022 1.337110 -0.000001
8 2.692409 -1.705069 0.000001
8 -1.815518 -1.033954 -0.000007
6 -1.651699 1.656595 -0.000010
1 0.849205 2.419092 -0.000001
1 -1.349919 2.702625 -0.000016

1 -2.242843 1.433394 -0.889662
1 -2.242843 1.433404 0.889644
6 3.267095 1.084060 0.000011
1 3.821207 0.720646 0.875549
1 3.821234 0.720616 -0.875497
1 3.254170 2.175168 -0.000005
8 -4.532962 -0.209180 0.000014
1 -3.576974 -0.409838 0.000009
1 -4.963561 -1.075826 0.000026
rd_1m5mU/rd_1m5mU46.xyz
7 0.468015 0.928151 -0.285148
6 0.713040 -0.506064 -0.529620
7 -0.283088 -1.378740 -0.402688
6 -1.532845 -0.981027 -0.048388
6 -1.805117 0.457395 0.202186
6 -0.735361 1.356395 0.059899
8 -2.471035 -1.799604 0.076021
8 1.879964 -0.790667 -0.845619
6 1.600930 1.850396 -0.432111
1 -0.867724 2.420092 0.230180
1 1.254638 2.865670 -0.248007
1 2.375952 1.576062 0.285031
1 2.000679 1.761048 -1.443339
6 -3.157151 0.913284 0.584208
1 -3.878339 0.626288 -0.192602
1 -3.480030 0.394989 1.496692
1 -3.195471 1.992594 0.740519
8 3.939225 -0.535611 1.134105
1 3.968977 -1.405470 1.556908
1 3.240635 -0.612373 0.456382
rd_1m5mU/rd_1m5mU19.xyz
7 -0.475482 0.782428 -0.053457
6 -0.627176 -0.684551 -0.062209
7 0.453875 -1.462012 -0.038433
6 1.704720 -0.935039 0.004234
6 1.882893 0.539894 0.030273

6 0.726104 1.334566 -0.004369
8 2.723806 -1.661236 0.021778
8 -1.797334 -1.095896 -0.096027
6 -1.693004 1.601453 -0.099531
1 0.785644 2.418144 0.004060
1 -1.410026 2.652180 -0.127402
1 -2.264935 1.337566 -0.990288
1 -2.294745 1.396378 0.787547
6 3.231896 1.138700 0.086488
1 3.765050 0.771034 0.973165
1 3.823581 0.805002 -0.776327
1 3.194427 2.229080 0.104995
8 -4.511311 -0.354231 0.160315
1 -3.556416 -0.543289 0.075566
1 -4.565216 0.611753 0.174068
rd_1m5mU/rd_1m5mU99.xyz
7 -0.571521 0.079892 0.042178
6 -0.041613 -1.299624 0.032305
7 1.276910 -1.497785 -0.022153
6 2.149429 -0.459907 -0.045211
6 1.639105 0.934711 0.017979
6 0.247335 1.118801 0.045050
8 3.386502 -0.641914 -0.120310
8 -0.901617 -2.188749 0.075050
6 -2.028582 0.248674 0.055161
1 -0.191263 2.111134 0.067182
1 -2.258335 1.312564 0.054580
1 -2.447086 -0.232999 -0.829871
1 -2.434373 -0.228622 0.948321
6 2.571475 2.079871 0.030802
1 3.298836 1.963106 0.844017
1 3.160455 2.088913 -0.896828
1 2.047588 3.031681 0.135353
8 -5.438942 0.695983 -0.097065
1 -4.963414 -0.145098 -0.030917
1 -4.740568 1.366857 -0.109923

rd_1m5mU/rd_1m5mU09.xyz
7 0.535964 0.270566 -0.009186
6 0.182778 -1.163420 -0.005338
7 -1.101767 -1.525906 0.000653
6 -2.098258 -0.605015 0.004402
6 -1.764796 0.843917 0.001444
6 -0.406387 1.198954 -0.005549
8 -3.304804 -0.941250 0.011095
8 1.149072 -1.937612 -0.008161
6 1.961164 0.620380 -0.015268
1 -0.092419 2.237599 -0.008228
1 2.059139 1.704610 -0.022625
1 2.431171 0.203160 0.876611
1 2.427070 0.191248 -0.903629
6 -2.831985 1.865876 0.009264
1 -3.495772 1.718565 -0.852644
1 -3.463943 1.735195 0.897878
1 -2.427908 2.879419 -0.006649
8 5.341105 0.557659 -0.051764
1 4.711518 -0.175247 0.010331
1 6.073690 0.298309 0.525599
rd_1m5mU/rd_1m5mU50_a.xyz
7 -0.455137 0.823692 -0.159594
6 -0.646088 -0.637188 -0.218624
7 0.408539 -1.446050 -0.137439
6 1.667107 -0.958150 0.011465
6 1.883431 0.509562 0.089689
6 0.754600 1.338747 -0.009976
8 2.660429 -1.715717 0.086239
8 -1.822096 -1.011469 -0.349785
6 -1.643733 1.679581 -0.260129
1 0.843961 2.419342 0.032492
1 -1.325434 2.719509 -0.303592
1 -2.199822 1.411708 -1.158971
1 -2.274134 1.513974 0.615653
6 3.239253 1.069099 0.263714

1 3.699196 0.660585 1.173004
1 3.883987 0.749434 -0.565996
1 3.225478 2.159025 0.316533
8 -4.423109 -0.239390 0.498600
1 -4.858864 -1.093794 0.626056
1 -3.517398 -0.460579 0.206780
rd_1m5mU/rd_1m5mU01.xyz
7 -1.836605 0.508895 0.010167
6 -1.634543 -0.953287 0.003477
7 -0.393445 -1.449240 -0.015610
6 0.690323 -0.639935 -0.027224
6 0.513152 0.834425 -0.017677
6 -0.801188 1.331298 0.002066
8 1.853912 -1.111731 -0.047167
8 -2.673568 -1.623780 0.015379
6 -3.217605 1.005847 0.023919
1 -0.998931 2.398354 0.008474
1 -3.200095 2.094416 0.022026
1 -3.736525 0.630505 -0.859593
1 -3.719496 0.633070 0.918319
6 1.679937 1.739490 -0.028550
1 2.312075 1.542596 0.847525
1 2.308047 1.528929 -0.903912
1 1.380026 2.788447 -0.034337
8 4.494128 -0.197558 0.053096
1 3.540746 -0.426224 0.023843
1 4.948276 -1.050167 0.109219
rd_1m5mU/rd_1m5mU62.xyz
7 -1.728162 -0.839360 -0.005423
6 -2.114803 0.585833 0.010011
7 -1.168167 1.527389 0.003514
6 0.149423 1.205190 -0.009400
6 0.560577 -0.223216 -0.008967
6 -0.453847 -1.194292 -0.010400
8 1.042409 2.083533 -0.020967
8 -3.335139 0.793172 0.027858

6 -2.802577 -1.838211 -0.003934
1 -0.222819 -2.254739 -0.015331
1 -2.358061 -2.830719 -0.047426
1 -3.393492 -1.723487 0.906448
1 -3.445135 -1.669871 -0.869352
6 1.990349 -0.594630 -0.007369
1 2.489485 -0.156613 -0.882390
1 2.486334 -0.157504 0.869960
1 2.133101 -1.676415 -0.007402
8 5.749173 -0.606146 0.019079
1 6.160559 0.270462 0.019977
1 4.798067 -0.425838 0.011474
rd_1m5mU/rd_1m5mU04.xyz
7 1.263144 -1.032543 0.126761
6 2.080856 0.132757 -0.267845
7 1.522695 1.344364 -0.316020
6 0.215785 1.534298 -0.006796
6 -0.624665 0.375452 0.391310
6 -0.015272 -0.888660 0.433593
8 -0.312055 2.669669 -0.045999
8 3.262548 -0.125700 -0.530743
6 1.921220 -2.342438 0.175169
1 -0.572438 -1.775342 0.718490
1 1.188317 -3.093759 0.463342
1 2.334414 -2.568791 -0.809019
1 2.734449 -2.304251 0.902309
6 -2.050815 0.559513 0.728598
1 -2.151535 1.286350 1.545211
1 -2.580208 1.000271 -0.127551
1 -2.527353 -0.380083 1.014976
8 -5.069472 -1.176474 -0.733062
1 -5.781000 -0.952914 -0.115605
1 -4.356350 -0.559710 -0.513067
rd_1m5mU/rd_1m5mU66.xyz
7 1.727551 0.490730 0.183922
6 1.527023 -0.954612 -0.037551

7 0.309937 -1.412769 -0.348764
6 -0.744959 -0.578296 -0.482145
6 -0.553409 0.886322 -0.333193
6 0.728071 1.340781 0.020552
8 -1.896464 -1.011206 -0.738293
8 2.541325 -1.649609 0.091479
6 3.071692 0.945253 0.558275
1 0.928492 2.397034 0.169822
1 3.045376 2.020357 0.726893
1 3.380602 0.424919 1.466128
1 3.765856 0.701375 -0.247927
6 -1.671028 1.824373 -0.560967
1 -2.059118 1.696903 -1.580559
1 -2.509019 1.583604 0.105702
1 -1.368992 2.862271 -0.411264
8 -3.968665 -0.544063 1.128551
1 -3.257739 -0.634264 0.461242
1 -3.741779 0.258162 1.620132
rd_1m5mU/rd_1m5mU06.xyz
7 1.702895 -0.834542 0.005066
6 2.083567 0.591851 -0.033430
7 1.133472 1.529841 -0.013646
6 -0.182319 1.202548 0.031340
6 -0.587897 -0.227408 0.049711
6 0.430462 -1.194269 0.040468
8 -1.078294 2.077692 0.056246
8 3.302366 0.803364 -0.082131
6 2.781179 -1.828977 -0.007301
1 0.203815 -2.255482 0.061579
1 2.341745 -2.822852 0.054007
1 3.354952 -1.720925 -0.929339
1 3.438745 -1.649892 0.844681
6 -2.016009 -0.604292 0.067384
1 -2.522132 -0.120066 0.912125
1 -2.507856 -0.219975 -0.836960
1 -2.154869 -1.685151 0.122580

8 -5.467497 -0.624675 -0.077371
1 -4.801377 0.078105 -0.095011
1 -6.314087 -0.158609 -0.136579
rd_1m5mU/rd_1m5mU72.xyz
7 -0.484498 0.906459 -0.310027
6 -0.708052 -0.534850 -0.534571
7 0.300519 -1.390800 -0.393269
6 1.541909 -0.970175 -0.035912
6 1.791459 0.475043 0.199078
6 0.710180 1.356670 0.037132
8 2.491369 -1.772941 0.104284
8 -1.870016 -0.840023 -0.849151
6 -1.629025 1.810304 -0.476315
1 0.826401 2.424326 0.193543
1 -1.299042 2.832263 -0.299235
1 -2.019690 1.705315 -1.489592
1 -2.406608 1.532326 0.236736
6 3.133729 0.955807 0.585038
1 3.459238 0.450812 1.503956
1 3.864024 0.673327 -0.184937
1 3.154138 2.037011 0.731533
8 -3.889922 -0.683784 1.200420
1 -3.968925 0.249553 1.443732
1 -3.214331 -0.697357 0.496201
rd_1mC/rd_1mC_2_086.xyz
6 0.449025 -0.327318 -0.009603
6 -1.136255 1.392480 0.000154
6 -2.218748 0.467373 0.008513
6 -1.905499 -0.872739 0.008756
1 -2.658205 -1.652052 0.015333
7 -0.617878 -1.270492 0.000751
7 0.156765 0.988663 -0.008763
8 1.616720 -0.779870 -0.019434
7 -1.439272 2.709107 0.001363
1 -0.557283 3.235978 -0.005586
1 -3.246904 0.803074 0.015084

6 -0.268341 -2.696332 0.001480
1 0.306686 -2.928644 -0.897093
1 0.342241 -2.917987 0.878778
1 -1.185178 -3.283139 0.023374
8 3.789237 0.937606 -0.011089
1 4.532347 0.361223 0.215625
1 3.000253 0.347919 -0.010584
rd_1mC/rd_1mC_1_027.xyz
6 -0.473188 0.317090 0.001481
6 1.154567 -1.361139 -0.000040
6 2.211640 -0.403442 0.001663
6 1.863046 0.926742 0.001130
1 2.594795 1.725842 0.001728
7 0.564372 1.288981 -0.000358
7 -0.146593 -0.991340 0.000076
8 -1.653584 0.736233 0.003956
7 1.397116 -2.693677 -0.002239
1 2.412691 -2.841130 -0.001720
1 3.251457 -0.704955 0.002660
6 0.174406 2.703935 -0.003208
1 -0.433952 2.909641 -0.885907
1 -0.415555 2.919172 0.889858
1 1.073914 3.317008 -0.015952
8 -3.865335 -0.912703 -0.001300
1 -3.024507 -0.399296 0.001082
1 -3.594575 -1.841391 -0.001503
rd_1mC/rd_1mC_1_010.xyz
6 -0.472721 -0.327852 0.002350
6 1.132774 1.371867 -0.001036
6 2.202322 0.428219 -0.004912
6 1.871298 -0.906371 -0.003224
1 2.613427 -1.695845 -0.005154
7 0.577680 -1.285977 0.001170
7 -0.163413 0.984874 0.002607
8 -1.647680 -0.762507 0.003697
7 1.357467 2.707545 -0.000411

1 2.370976 2.869149 -0.003624
1 3.237980 0.743651 -0.008556
6 0.207303 -2.706309 0.004756
1 -0.391009 -2.921797 0.892056
1 -0.386809 -2.927830 -0.883970
1 1.115435 -3.306845 0.009013
8 -3.852411 0.897042 -0.031189
1 -3.021509 0.368005 -0.009525
1 -3.585755 1.792829 0.218537
rd_1mC/rd_1mC_2_042.xyz
6 -0.284337 -0.808851 -0.106292
6 0.714107 1.321692 -0.056393
6 -0.548168 1.967340 0.067460
6 -1.671545 1.168148 0.108121
1 -2.672077 1.573523 0.204820
7 -1.559521 -0.169987 0.024754
7 0.816709 -0.027444 -0.141231
8 -0.254986 -2.051044 -0.180868
7 1.818231 2.095394 -0.088152
1 2.636616 1.480851 -0.182256
1 -0.624788 3.044465 0.129567
6 -2.739949 -1.039181 0.073562
1 -2.785994 -1.634821 -0.840169
1 -2.654330 -1.711547 0.929586
1 -3.632383 -0.423056 0.165882
8 3.252615 -1.441628 0.208590
1 2.406705 -0.953908 0.060159
1 2.996637 -2.374764 0.224288
rd_1mC/rd_1mC_1_026.xyz
6 0.472020 -0.335627 0.006387
6 -1.116502 1.380086 0.003857
6 -2.195306 0.447033 0.001835
6 -1.877486 -0.890691 -0.003552
1 -2.627406 -1.672809 -0.008129
7 -0.587775 -1.283247 -0.003716
7 0.175824 0.980148 0.005440

8 1.642752 -0.781834 0.014867
7 -1.327825 2.718001 0.003464
1 -2.339676 2.890241 0.003652
1 -3.227780 0.772682 0.002559
6 -0.232246 -2.707498 -0.013036
1 0.383933 -2.919991 -0.888604
1 0.338894 -2.944741 0.886807
1 -1.146513 -3.297901 -0.044600
8 3.838746 0.891636 0.045393
1 3.018132 0.346837 0.024714
1 3.623978 1.683138 -0.467739
rd_1mC/rd_1mC_2_001.xyz
6 -0.379927 -0.831252 -0.098273
6 0.788866 1.211326 -0.066173
6 -0.416174 1.958366 0.058481
6 -1.600708 1.253417 0.103395
1 -2.563362 1.741147 0.202257
7 -1.599662 -0.089589 0.022272
7 0.781366 -0.142516 -0.144318
8 -0.452792 -2.072243 -0.155512
7 1.951684 1.893800 -0.102649
1 2.718927 1.215877 -0.192292
1 -0.404863 3.038198 0.119280
6 -2.848235 -0.859729 0.082225
1 -2.920142 -1.496905 -0.800739
1 -2.839292 -1.489363 0.974640
1 -3.690138 -0.170219 0.117926
8 3.240082 -1.543889 0.174549
1 2.372610 -1.092599 0.039567
1 3.831301 -0.841715 0.482002
rd_1mC/rd_1mC_2_072.xyz
6 -0.222557 -0.515102 -0.157050
6 2.108505 -0.473030 0.053945
6 2.128883 0.947208 0.151508
6 0.921428 1.602911 0.077980
1 0.833102 2.681200 0.135964

7 -0.223912 0.906831 -0.072020
7 0.956333 -1.168797 -0.097719
8 -1.321316 -1.098803 -0.290019
7 3.293011 -1.120681 0.116665
1 3.107499 -2.127824 0.033541
1 3.056060 1.490714 0.273629
6 -1.518253 1.595169 -0.153352
1 -2.030323 1.287893 -1.066459
1 -2.129820 1.323337 0.709253
1 -1.343265 2.669495 -0.162097
8 -4.021120 -0.679926 0.314382
1 -3.069495 -0.777423 0.099941
1 -4.370325 -1.581966 0.314662
rd_1mC/rd_1mC_1_080.xyz
6 -0.815395 -0.929772 0.028669
6 0.845484 0.716657 0.036150
6 -0.117647 1.769642 0.009840
6 -1.446692 1.411042 -0.018817
1 -2.249586 2.138531 -0.045870
7 -1.797268 0.111803 -0.015787
7 0.491142 -0.587633 0.046358
8 -1.225525 -2.106933 0.047198
7 2.170869 0.980228 0.053709
1 2.324858 1.993306 0.041783
1 0.178325 2.811036 0.007137
6 -3.206515 -0.291299 -0.067835
1 -3.361471 -0.941136 -0.931117
1 -3.459792 -0.844967 0.838913
1 -3.826556 0.599725 -0.150350
8 4.283691 -0.932864 0.014948
1 4.767721 -0.755579 -0.804049
1 3.552567 -0.270947 0.028377
rd_1mC/rd_1mC_2_013.xyz
6 0.359057 -0.828219 -0.078210
6 -0.768230 1.237383 -0.049550
6 0.453194 1.961393 0.049801

6 1.624274 1.233760 0.083190
1 2.598181 1.702791 0.160620
7 1.595019 -0.109855 0.016538
7 -0.788940 -0.116859 -0.114024
8 0.405960 -2.070918 -0.123589
7 -1.917469 1.942901 -0.075593
1 -2.699443 1.279850 -0.145427
1 0.464069 3.041884 0.098324
6 2.828018 -0.905278 0.058980
1 2.820797 -1.531914 0.953452
1 2.871991 -1.546027 -0.823351
1 3.685075 -0.233997 0.078321
8 -3.236588 -1.553059 0.160398
1 -2.383404 -1.070079 0.046144
1 -3.910394 -0.858236 0.163747
rd_1mC/rd_1mC_2_011.xyz
6 -0.289956 -0.384630 0.518126
6 1.963149 -0.494461 -0.103174
6 1.985814 0.881781 -0.466177
6 0.817494 1.595080 -0.320229
1 0.736298 2.645749 -0.572740
7 -0.292004 0.992469 0.152392
7 0.845303 -1.095580 0.371952
8 -1.356144 -0.869044 0.963643
7 3.105895 -1.200084 -0.247784
1 2.919723 -2.165877 0.049808
1 2.884289 1.350274 -0.844652
6 -1.544240 1.740308 0.325767
1 -2.329434 1.277192 -0.274441
1 -1.838531 1.708317 1.376672
1 -1.385599 2.769643 0.009639
8 -3.409824 -1.246188 -0.916973
1 -3.366543 -0.489253 -1.518199
1 -2.700379 -1.080297 -0.261242
rd_1mC/rd_1mC_2_071.xyz
6 0.268403 -0.426166 0.448311

6 -2.003640 -0.491685 -0.109900
6 -2.023599 0.897939 -0.418118
6 -0.847818 1.597004 -0.265755
1 -0.765735 2.657884 -0.470787
7 0.267784 0.968880 0.157838
7 -0.878732 -1.120292 0.308817
8 1.343926 -0.943194 0.828886
7 -3.157039 -1.181331 -0.248637
1 -2.972698 -2.158709 0.009546
1 -2.927040 1.388084 -0.755166
6 1.525940 1.705371 0.337128
1 1.895059 1.541007 1.350649
1 2.267686 1.338796 -0.375289
1 1.339299 2.764802 0.170591
8 3.595184 -1.132412 -0.812144
1 3.707281 -0.298609 -1.289851
1 2.803455 -0.993984 -0.249757
rd_1mC/rd_1mC_2_036.xyz
6 -1.448286 0.802314 0.000269
6 0.875760 0.537143 -0.001571
6 0.775206 -0.885324 -0.001473
6 -0.491632 -1.429504 -0.000056
1 -0.668766 -2.498681 0.000328
7 -1.571299 -0.627929 0.000747
7 -0.211431 1.343036 -0.000818
8 -2.501336 1.468570 0.000889
7 2.115313 1.066944 -0.002471
1 2.048374 2.090748 -0.002313
1 1.656108 -1.513253 -0.001920
6 -2.929552 -1.182440 0.001832
1 -3.458352 -0.832017 0.890488
1 -3.459549 -0.832347 -0.886243
1 -2.868593 -2.269222 0.001967
8 4.368671 -0.660075 -0.009412
1 3.624405 -0.012126 -0.002320
1 5.170635 -0.128553 0.091981

rd_1mC/rd_1mC_2_054.xyz
6 -1.447222 0.796471 0.007249
6 0.880013 0.561340 -0.013839
6 0.798063 -0.862331 -0.022170
6 -0.461636 -1.422512 -0.014448
1 -0.625869 -2.493749 -0.020120
7 -1.551637 -0.634804 -0.000414
7 -0.217228 1.352815 0.000368
8 -2.508112 1.449950 0.018976
7 2.112076 1.108738 -0.020790
1 2.029945 2.131525 -0.013899
1 1.687139 -1.478809 -0.035132
6 -2.902573 -1.207035 0.005692
1 -3.430863 -0.868366 0.899181
1 -3.442189 -0.859468 -0.877580
1 -2.827029 -2.292803 -0.000208
8 4.421663 -0.541382 0.012758
1 3.625206 0.040969 -0.002892
1 4.072917 -1.430683 0.167722
rd_1mC/rd_1mC_2_039.xyz
6 -0.208077 -0.547219 -0.082683
6 2.127475 -0.437226 0.033154
6 2.112418 0.984915 0.099714
6 0.886024 1.606224 0.048658
1 0.770137 2.682895 0.081601
7 -0.244237 0.876530 -0.049381
7 0.990172 -1.167317 -0.053583
8 -1.294572 -1.164621 -0.142109
7 3.331497 -1.051006 0.057558
1 3.171398 -2.064049 -0.003608
1 3.027979 1.555484 0.177937
6 -1.557668 1.528996 -0.117824
1 -2.079097 1.189070 -1.013871
1 -2.143175 1.258907 0.763198
1 -1.411528 2.607047 -0.152479
8 -4.047041 -0.821769 0.158647

1 -4.233730 0.100353 0.384259
1 -3.072143 -0.860178 0.062375
rd_1mC/rd_1mC_1_033.xyz
6 -0.330665 -0.823132 0.115157
6 0.775419 1.251788 0.070042
6 -0.455835 1.958765 -0.058105
6 -1.615748 1.216887 -0.110850
1 -2.593595 1.672075 -0.218427
7 -1.570728 -0.125984 -0.027005
7 0.809730 -0.098494 0.155936
8 -0.361076 -2.065275 0.193339
7 1.969794 1.884784 0.107644
1 1.803700 2.895247 0.044072
1 -0.484255 3.039274 -0.120963
6 -2.790794 -0.938923 -0.098267
1 -2.733209 -1.595007 -0.969346
1 -2.868666 -1.551600 0.801566
1 -3.653768 -0.280430 -0.180920
8 3.156627 -1.645499 -0.195449
1 2.356848 -1.082900 -0.050130
1 3.852716 -1.024913 -0.452864
rd_1mC/rd_1mC_2_059.xyz
6 -0.202996 -0.554935 0.011780
6 2.134436 -0.431103 -0.005282
6 2.114672 0.992529 -0.026653
6 0.883694 1.606734 -0.016875
1 0.763057 2.683433 -0.027369
7 -0.245094 0.869045 0.009070
7 0.998944 -1.168590 0.011183
8 -1.287612 -1.178299 0.017206
7 3.341883 -1.038876 -0.001107
1 3.184882 -2.054123 0.018050
1 3.029768 1.568866 -0.045333
6 -1.563495 1.513969 0.033226
1 -2.121520 1.229339 -0.860962
1 -2.108336 1.181312 0.918425

1 -1.424338 2.593008 0.061519
8 -4.069216 -0.805336 -0.033856
1 -4.244942 0.146127 -0.023443
1 -3.091947 -0.873097 -0.018883
rd_1mC/rd_1mC_1_078.xyz
6 0.812606 -0.929191 0.012212
6 -0.846105 0.719706 0.007495
6 0.118610 1.771493 0.008165
6 1.447343 1.410999 0.000061
1 2.251630 2.137342 -0.004721
7 1.795976 0.111181 -0.003184
7 -0.493736 -0.585129 0.010365
8 1.220641 -2.107162 0.024699
7 -2.171384 0.984680 0.001222
1 -2.324516 1.997939 0.001956
1 -0.175771 2.813338 0.010359
6 3.205802 -0.292472 -0.023920
1 3.434211 -0.860621 0.880373
1 3.384853 -0.928715 -0.892612
1 3.828199 0.599198 -0.075365
8 -4.242525 -0.966854 -0.061510
1 -5.030162 -0.547946 0.312950
1 -3.538897 -0.276740 -0.021345
rd_1mC/rd_1mC_2_041.xyz
6 -0.275548 0.404429 0.445271
6 1.997356 0.516398 -0.100853
6 2.043065 -0.869325 -0.423986
6 0.879261 -1.590929 -0.284672
1 0.816895 -2.650931 -0.500939
7 -0.248941 -0.986953 0.140163
7 0.859405 1.120623 0.319290
8 -1.362042 0.897386 0.827472
7 3.139217 1.227256 -0.226257
1 2.936811 2.198898 0.040009
1 2.956342 -1.339919 -0.762305
6 -1.494685 -1.746494 0.309206

1 -1.861584 -1.608875 1.327674
1 -2.244512 -1.376356 -0.392487
1 -1.292851 -2.799423 0.120726
8 -3.628730 0.970540 -0.807836
1 -2.828415 0.917495 -0.242912
1 -3.700980 1.904744 -1.049017
rd_1mC/rd_1mC_2_081.xyz
6 0.274095 -0.407707 -0.439440
6 -2.000133 -0.517238 0.101640
6 -2.046536 0.869812 0.419018
6 -0.882604 1.590972 0.278544
1 -0.820816 2.651986 0.489980
7 0.246438 0.985296 -0.141614
7 -0.861321 -1.123232 -0.313528
8 1.361841 -0.902592 -0.815309
7 -3.142286 -1.227558 0.227598
1 -2.939413 -2.200229 -0.034525
1 -2.960499 1.341757 0.753587
6 1.492193 1.744419 -0.312394
1 2.240328 1.380211 0.394199
1 1.862228 1.599478 -1.328688
1 1.289073 2.798555 -0.132221
8 3.631219 -0.957720 0.818588
1 2.832190 -0.910438 0.251433
1 3.830532 -1.901914 0.888605
rd_1mC/rd_1mC_1_048.xyz
6 -0.217205 -0.559976 0.023438
6 2.120479 -0.469503 -0.007839
6 2.115871 0.956663 -0.039083
6 0.893738 1.585378 -0.023645
1 0.785743 2.663382 -0.040075
7 -0.244595 0.861344 0.016327
7 0.977050 -1.189532 0.020547
8 -1.307806 -1.173916 0.035079
7 3.272985 -1.182845 -0.003736
1 4.062640 -0.528185 -0.030074

1 3.035362 1.527794 -0.068860
6 -1.555313 1.521806 0.046991
1 -2.119193 1.248669 -0.847195
1 -2.102044 1.191274 0.931799
1 -1.403258 2.599052 0.080421
8 -4.078536 -0.781954 -0.058164
1 -4.250464 0.170057 -0.041864
1 -3.101554 -0.854060 -0.030600
rd_1mU/rd_1mU82.xyz
7 -1.607530 -0.103094 0.026038
6 -0.318867 -0.829798 -0.114555
7 0.820495 -0.132676 -0.163817
6 0.833861 1.222907 -0.073293
6 -0.440457 1.941475 0.053758
6 -1.636006 1.218029 0.107165
8 1.901791 1.877573 -0.093739
8 -0.413430 -2.057329 -0.177580
6 -2.828939 -0.910460 0.097953
1 -0.448008 3.022820 0.113991
1 -2.598848 1.706233 0.213829
1 -3.691034 -0.248048 0.152818
1 -2.887745 -1.544146 -0.788197
1 -2.777353 -1.544553 0.985633
8 3.251902 -1.533477 0.216657
1 3.927829 -0.841084 0.234536
1 2.404749 -1.047882 0.062981
rd_1mU/rd_1mU04.xyz
7 -0.645434 1.273682 -0.000003
6 0.460537 0.284831 0.000003
7 0.187318 -1.017465 0.000003
6 -1.097156 -1.460286 0.000001
6 -2.199275 -0.490255 -0.000005
6 -1.907903 0.876743 -0.000005
8 -1.377214 -2.683189 0.000004
8 1.592979 0.789459 0.000007
6 -0.291031 2.695990 0.000002

1 -3.225879 -0.834928 -0.000010
1 -2.683209 1.635367 -0.000008
1 -1.205633 3.286264 -0.000031
1 0.306072 2.912547 0.887732
1 0.306133 2.912538 -0.887688
8 3.928148 -0.752512 -0.000009
1 3.065846 -0.284464 0.000003
1 3.701141 -1.693050 0.000013
rd_1mU/rd_1mU47.xyz
7 -1.782914 0.130604 -0.031112
6 -0.740623 -0.929618 0.061018
7 0.550644 -0.587758 0.091950
6 0.924631 0.711813 0.064813
6 -0.096645 1.765968 0.031719
6 -1.447330 1.410261 -0.028607
8 2.133520 1.061357 0.068517
8 -1.183442 -2.080733 0.098048
6 -3.179616 -0.300702 -0.125717
1 0.199429 2.807674 0.041062
1 -2.239412 2.150127 -0.078217
1 -3.814069 0.577364 -0.232170
1 -3.284763 -0.958459 -0.990399
1 -3.442088 -0.856662 0.776663
8 4.199550 -0.803227 -0.140642
1 3.815666 -1.690779 -0.110986
1 3.431611 -0.194697 -0.058554
rd_1mU/rd_1mU58.xyz
7 1.787420 0.134444 0.029763
6 0.750632 -0.931781 -0.056103
7 -0.542587 -0.596995 -0.086221
6 -0.923326 0.700664 -0.063476
6 0.092302 1.760399 -0.036243
6 1.444939 1.412214 0.022888
8 -2.133893 1.044122 -0.066375
8 1.199541 -2.080632 -0.088967
6 3.186825 -0.288529 0.123314

1 -0.209437 2.800420 -0.049566
1 2.233085 2.156557 0.067996
1 3.816408 0.593694 0.224562
1 3.297555 -0.941727 0.990721
1 3.450530 -0.846905 -0.777196
8 -4.222226 -0.786442 0.162502
1 -3.918680 -1.658330 -0.126605
1 -3.438905 -0.200032 0.065739
rd_1mU/rd_1mU41.xyz
7 -1.563178 -0.197590 0.029053
6 -0.214506 -0.803216 -0.117377
7 0.857111 -0.005173 -0.158245
6 0.745957 1.345689 -0.062708
6 -0.588504 1.944160 0.068385
6 -1.713327 1.114828 0.114978
8 1.748422 2.096188 -0.083654
8 -0.196723 -2.033935 -0.191326
6 -2.707916 -1.110188 0.077997
1 -0.694447 3.020067 0.133204
1 -2.718418 1.509631 0.218499
1 -3.621071 -0.527638 0.181635
1 -2.730068 -1.699153 -0.840746
1 -2.582641 -1.784762 0.927200
8 3.287359 -1.415758 0.189483
1 2.997531 -2.305402 0.436179
1 2.448878 -0.912994 0.044711
rd_1mU/rd_1mU05.xyz
7 -1.562518 -0.199932 0.029495
6 -0.212555 -0.803008 -0.115223
7 0.857515 -0.002902 -0.155207
6 0.743614 1.347944 -0.063287
6 -0.592330 1.944065 0.063417
6 -1.715414 1.112437 0.111685
8 1.744679 2.100308 -0.083873
8 -0.192051 -2.033731 -0.188548
6 -2.705500 -1.114730 0.080558

1 -0.700712 3.019986 0.123764
1 -2.721410 1.505420 0.213208
1 -3.619812 -0.533640 0.182190
1 -2.726162 -1.706476 -0.836407
1 -2.579190 -1.786478 0.931865
8 3.286202 -1.410235 0.212625
1 3.017364 -2.339793 0.220734
1 2.447393 -0.910159 0.060102
rd_1mU/rd_1mU64.xyz
7 -1.796250 0.142740 0.028072
6 -0.770398 -0.934494 -0.053059
7 0.526200 -0.613077 -0.081350
6 0.920765 0.680393 -0.059109
6 -0.083763 1.750689 -0.031924
6 -1.440165 1.416664 0.023008
8 2.135003 1.011174 -0.062613
8 -1.231184 -2.078671 -0.084198
6 -3.200750 -0.264417 0.115708
1 0.228639 2.787565 -0.042911
1 -2.220490 2.169277 0.066031
1 -3.820376 0.625047 0.215429
1 -3.467239 -0.818900 -0.786430
1 -3.322689 -0.917264 0.981805
8 4.161520 -0.908794 0.112119
1 4.975160 -0.392679 0.198858
1 3.440485 -0.243362 0.049944
rd_1mU/rd_1mU21.xyz
7 0.204316 0.930489 0.070202
6 0.199615 -0.552487 0.161475
7 -0.953006 -1.216240 0.101667
6 -2.130905 -0.558382 -0.052928
6 -2.135840 0.906058 -0.155991
6 -0.926260 1.602564 -0.081214
8 -3.228383 -1.163951 -0.109753
8 1.316827 -1.069504 0.298253
6 1.496619 1.616954 0.149054

1 -3.070733 1.437535 -0.284895
1 -0.873226 2.683982 -0.144880
1 1.321980 2.691160 0.145151
1 2.003414 1.310853 1.065353
1 2.105432 1.326789 -0.709990
8 4.070842 -0.669919 -0.285730
1 4.341190 -1.564314 -0.537123
1 3.119112 -0.746996 -0.081237
rd_1mU/rd_1mU89.xyz
7 1.580433 0.633838 0.004275
6 1.400336 -0.844358 0.008031
7 0.168453 -1.362328 -0.006710
6 -0.921680 -0.561799 -0.023104
6 -0.755057 0.897557 -0.022407
6 0.533200 1.441550 -0.008078
8 -2.088396 -1.033593 -0.039791
8 2.452943 -1.488264 0.023770
6 2.952465 1.148578 0.012930
1 -1.624542 1.543518 -0.033047
1 0.707840 2.512181 -0.009523
1 2.921154 2.236575 0.003604
1 3.459482 0.785444 0.908848
1 3.474886 0.770805 -0.867926
8 -4.356977 0.591890 -0.029422
1 -4.952499 0.162535 0.600735
1 -3.544676 0.038943 -0.026334
rd_1mU/rd_1mU43.xyz
7 1.579575 -0.173916 0.032146
6 0.247646 -0.814287 -0.123931
7 -0.845155 -0.045004 -0.165057
6 -0.767547 1.308880 -0.073022
6 0.551236 1.942009 0.055941
6 1.696415 1.142020 0.114076
8 -1.787946 2.034803 -0.095219
8 0.265111 -2.044357 -0.205389
6 2.745683 -1.057826 0.098496

1 0.629449 3.020678 0.113568
1 2.690363 1.562985 0.223316
1 3.643790 -0.453035 0.206158
1 2.628659 -1.728214 0.952267
1 2.791270 -1.653772 -0.814735
8 -3.270228 -1.438630 0.328930
1 -3.579569 -1.804861 -0.511451
1 -2.440990 -0.950649 0.105325
rd_1mU/rd_1mU37.xyz
7 -0.231388 0.894179 0.059876
6 -0.180711 -0.591269 0.097362
7 0.995069 -1.214529 0.055300
6 2.154747 -0.513855 -0.037955
6 2.113334 0.952042 -0.108434
6 0.879673 1.606399 -0.046669
8 3.273829 -1.080642 -0.065022
8 -1.283982 -1.148796 0.171060
6 -1.547494 1.535824 0.121899
1 3.032718 1.517120 -0.199958
1 0.792079 2.686769 -0.085101
1 -1.408971 2.614909 0.143796
1 -2.123700 1.242886 -0.758291
1 -2.064861 1.193479 1.019093
8 -4.081384 -0.798268 -0.182159
1 -4.243758 0.127123 -0.414022
1 -3.114277 -0.853040 -0.060001
rd_1mU/rd_1mU85.xyz
7 0.207757 0.924891 0.070289
6 0.196823 -0.558839 0.151061
7 -0.959144 -1.216830 0.092249
6 -2.134871 -0.552587 -0.051570
6 -2.133625 0.912561 -0.144692
6 -0.920456 1.603038 -0.071379
8 -3.235396 -1.152737 -0.106422
8 1.312318 -1.081945 0.278247
6 1.503656 1.605084 0.144607

1 -3.066651 1.449242 -0.265164
1 -0.862561 2.684564 -0.128644
1 1.333594 2.679996 0.148825
1 2.015646 1.290800 1.055137
1 2.104690 1.317992 -0.720947
8 4.064799 -0.655342 -0.297078
1 4.415814 -1.556845 -0.304080
1 3.116253 -0.757528 -0.089031
rd_1mU/rd_1mU51.xyz
7 1.734211 -0.435684 -0.019101
6 0.299394 -0.830971 0.038102
7 -0.644741 0.112000 0.056106
6 -0.317782 1.428383 0.022894
6 1.095712 1.825346 -0.034641
6 2.086508 0.839408 -0.052579
8 -1.189298 2.332144 0.041993
8 0.105469 -2.050730 0.066923
6 2.731519 -1.508457 -0.024780
1 1.362920 2.874605 -0.060802
1 3.143801 1.079542 -0.091548
1 3.723536 -1.066421 -0.094498
1 2.631749 -2.087390 0.895479
1 2.542405 -2.163487 -0.876930
8 -4.763754 -1.015478 -0.120785
1 -5.561894 -0.633793 0.272423
1 -4.060241 -0.387005 0.097805
rd_1mU/rd_1mU30.xyz
7 1.580875 0.633065 0.001990
6 1.400106 -0.845068 0.002700
7 0.167916 -1.362376 -0.003006
6 -0.921987 -0.561387 -0.008884
6 -0.754639 0.897934 -0.008427
6 0.533979 1.441288 -0.002501
8 -2.088948 -1.032752 -0.015027
8 2.452495 -1.489508 0.008277
6 2.953174 1.147176 0.005638

1 -1.623526 1.544809 -0.012309
1 0.709083 2.511843 -0.002546
1 2.922310 2.235223 0.002656
1 3.464799 0.778772 0.896763
1 3.470734 0.774144 -0.880106
8 -4.343083 0.612156 -0.010645
1 -5.070864 0.017926 0.219801
1 -3.541586 0.043628 -0.009144
rd_1mU/rd_1mU57.xyz
7 1.553974 0.644416 -0.001378
6 1.402412 -0.837021 0.004360
7 0.180672 -1.378392 0.001737
6 -0.924871 -0.599020 -0.005105
6 -0.786235 0.863656 -0.008901
6 0.491610 1.432104 -0.007135
8 -2.082113 -1.094047 -0.008714
8 2.467136 -1.460807 0.010860
6 2.915976 1.185184 -0.000609
1 -1.667908 1.493289 -0.014561
1 0.646421 2.505813 -0.011112
1 2.863619 2.272209 -0.006563
1 3.435250 0.829441 0.891236
1 3.439850 0.819787 -0.885812
8 -4.365028 0.502683 -0.010000
1 -4.070771 1.400251 0.200167
1 -3.542286 -0.035003 -0.008686
rd_1mU/rd_1mU25.xyz
7 0.228806 0.990807 0.142491
6 0.240841 -0.467307 0.429345
7 -0.878172 -1.177173 0.306066
6 -2.035343 -0.589227 -0.097445
6 -2.047695 0.841912 -0.422938
6 -0.876344 1.589423 -0.273730
8 -3.106525 -1.233396 -0.204068
8 1.341637 -0.913685 0.783354
6 1.477572 1.735049 0.332204

1 -2.961386 1.311613 -0.765907
1 -0.836755 2.653152 -0.482741
1 1.282517 2.793608 0.171114
1 1.841586 1.558911 1.345328
1 2.220558 1.371693 -0.380686
8 3.726673 -0.895577 -0.775642
1 3.843822 -1.825668 -1.015608
1 2.906746 -0.876595 -0.245170
rd_1mU/rd_1mU45.xyz
7 0.248327 0.965433 -0.160122
6 0.226782 -0.498424 -0.418928
7 -0.909669 -1.177778 -0.285609
6 -2.052716 -0.556231 0.106481
6 -2.030387 0.879169 0.411866
6 -0.841845 1.596227 0.248245
8 -3.139246 -1.173060 0.219526
8 1.317592 -0.978717 -0.758414
6 1.514199 1.676929 -0.359362
1 -2.931775 1.375864 0.749423
1 -0.776785 2.661737 0.441111
1 1.341192 2.743236 -0.226395
1 2.243422 1.315398 0.368831
1 1.882312 1.466482 -1.364279
8 3.701865 -1.043618 0.799468
1 4.043707 -0.141300 0.872778
1 2.889439 -0.957866 0.264198
rd_1mU/rd_1mU28.xyz
7 -1.010837 -0.540530 -0.462647
6 -0.488444 -0.464385 0.930331
7 0.827960 -0.404546 1.140226
6 1.699839 -0.373213 0.101862
6 1.190537 -0.423116 -1.275670
6 -0.187364 -0.504580 -1.497376
8 2.941131 -0.300795 0.275893
8 -1.360538 -0.471670 1.805001
6 -2.463576 -0.603730 -0.638131

1 1.881458 -0.392440 -2.109407
1 -0.611916 -0.542694 -2.494802
1 -2.687405 -0.666057 -1.701429
1 -2.846360 -1.478908 -0.110266
1 -2.909093 0.294330 -0.205344
8 -0.352802 2.979019 0.035060
1 -0.118510 3.704827 -0.561111
1 0.243688 2.258193 -0.214418
rd_1mU/rd_1mU32.xyz
7 0.417517 0.913582 -0.154281
6 0.340438 -0.466007 -0.373476
7 -0.863452 -1.087941 -0.258694
6 -1.925090 -0.381300 0.087439
6 -1.900660 1.006439 0.354934
6 -0.687493 1.617505 0.213696
8 -3.108452 -1.010720 0.197715
8 1.384264 -1.095348 -0.677023
6 1.718272 1.580293 -0.324064
1 -2.788169 1.551964 0.644458
1 -0.547506 2.678553 0.381431
1 1.567140 2.657224 -0.264621
1 2.408651 1.262820 0.460587
1 2.132975 1.319094 -1.298289
8 3.632979 -1.063209 0.845754
1 -2.992722 -1.955931 -0.008236
1 2.798056 -1.080573 0.292745
rd_5caC/rd_5caCacr.xyz
6 1.458690 -1.297935 -0.062577
6 0.032982 0.650483 -0.044686
6 -1.114124 -0.233703 -0.069681
6 -0.840176 -1.624820 -0.143525
1 -1.683961 -2.306411 -0.192720
7 0.361287 -2.155942 -0.147956
7 1.253595 0.078303 -0.008253
8 2.608041 -1.736806 -0.038786
7 -0.092218 1.960095 -0.046041

1 0.720518 2.569005 -0.009604
1 -1.027216 2.353848 -0.148641
6 -2.530487 0.269636 0.041281
8 -2.800070 1.362905 -0.529620
8 -3.303904 -0.459597 0.716206
1 2.106268 0.684216 0.038281
8 3.435799 1.767339 0.134934
1 3.414744 2.227228 0.988360
1 4.250754 1.242237 0.153328
rd_5caC/rd_5caCadu.xyz
6 -1.453139 1.318149 -0.061660
6 -0.043251 -0.641690 -0.034518
6 1.110786 0.232657 -0.072869
6 0.847836 1.625000 -0.161532
1 1.696820 2.299104 -0.222901
7 -0.349325 2.165833 -0.166816
7 -1.258636 -0.058676 0.008886
8 -2.598712 1.766559 -0.033970
7 0.070099 -1.952387 -0.032478
1 -0.747511 -2.553781 0.014886
1 1.001097 -2.355255 -0.135994
6 2.522799 -0.281293 0.041065
8 2.783428 -1.380350 -0.522753
8 3.302098 0.446030 0.711220
1 -2.112511 -0.660444 0.071831
8 -3.401312 -1.780766 0.288889
1 -4.249584 -1.315024 0.229382
1 -3.417484 -2.426713 -0.434346
rd_5caC/rd_5caCabj.xyz
6 -1.457524 1.306393 -0.060101
6 -0.037610 -0.646323 -0.037149
6 1.112011 0.234050 -0.069303
6 0.842036 1.625846 -0.146905
1 1.687783 2.304684 -0.200334
7 -0.357715 2.160839 -0.149202
7 -1.256188 -0.070155 -0.004096

8 -2.605667 1.748371 -0.034241
7 0.081093 -1.956671 -0.028090
1 -0.735458 -2.559762 0.019124
1 1.012726 -2.357711 -0.130586
6 2.526860 -0.273368 0.037795
8 2.791609 -1.366118 -0.536135
8 3.304140 0.452272 0.712194
1 -2.109527 -0.675552 0.037317
8 -3.437887 -1.761641 0.105445
1 -3.259597 -2.444310 0.770828
1 -4.198474 -1.268103 0.449234
rd_5caC/rd_5caCacs.xyz
6 -1.449581 -1.335150 0.054491
6 -0.052507 0.634347 0.014040
6 1.106786 -0.231915 0.066815
6 0.853094 -1.625833 0.157948
1 1.706481 -2.293933 0.223958
7 -0.340072 -2.175216 0.158518
7 -1.264201 0.043131 -0.019250
8 -2.592095 -1.791228 0.029997
7 0.049770 1.946067 -0.012508
1 -0.774479 2.536439 -0.079918
1 0.973743 2.362894 0.091888
6 2.516181 0.290870 -0.029587
8 2.766166 1.381216 0.554956
8 3.304584 -0.421455 -0.705211
1 -2.121748 0.641375 -0.061088
8 -3.448295 1.738716 -0.095657
1 -3.840315 1.756274 -0.982259
1 -3.148877 2.647169 0.065187
rd_5caC/rd_5caCacy.xyz
6 1.441846 1.471432 0.050922
6 0.131795 -0.565912 0.021733
6 -1.062875 0.248160 0.065587
6 -0.873120 1.654080 0.131161
1 -1.756954 2.283323 0.178563

7 0.293032 2.259143 0.130672
7 1.315868 0.086467 -0.015099
8 2.564115 1.975166 0.038917
7 0.103897 -1.880260 0.005240
1 0.977774 -2.407928 -0.046936
1 -0.791007 -2.353468 0.105148
6 -2.444697 -0.341479 -0.016181
8 -2.664543 -1.387684 0.652540
8 -3.243581 0.278234 -0.767028
1 2.177873 -0.480118 -0.060166
8 2.989222 -2.165163 -0.180877
1 3.463064 -2.319875 -1.011898
1 3.620286 -2.381496 0.521843
rd_5caC/rd_5caCadj.xyz
6 2.601081 -0.152485 0.006802
6 0.257937 -0.823391 -0.028756
6 -0.088262 0.576854 0.004678
6 0.983401 1.508040 0.018803
1 0.737268 2.566530 0.030124
7 2.259776 1.194675 0.023457
7 1.584356 -1.109677 -0.030196
8 3.773150 -0.525486 0.019931
7 -0.627424 -1.794032 -0.063854
1 -0.313898 -2.761286 -0.088548
1 -1.650915 -1.617775 -0.061928
6 -1.507713 1.070277 0.033327
8 -2.078320 1.043754 1.153412
8 -1.967997 1.471367 -1.063772
1 1.886636 -2.083042 -0.051132
8 -3.416427 -1.427398 -0.024797
1 -3.490472 -0.721544 0.639032
1 -3.647488 -0.993303 -0.860714
rd_5caC/rd_5caCadn.xyz
6 -1.257675 -1.757366 0.021175
6 -0.241193 0.449335 -0.019081
6 1.053644 -0.194332 0.064648

6 1.059136 -1.610337 0.164410
1 2.018853 -2.110128 0.253022
7 -0.012099 -2.371199 0.151925
7 -1.323276 -0.367585 -0.073347
8 -2.295849 -2.414138 -0.007067
7 -0.389536 1.750349 -0.043827
1 -1.337593 2.171893 -0.090140
1 0.448813 2.318886 0.074046
6 2.345511 0.579524 -0.005080
8 2.371294 1.714248 0.547271
8 3.275292 0.005339 -0.631005
1 -2.256177 0.039482 -0.139069
8 -3.049970 2.602277 -0.122422
1 -3.514496 2.011727 -0.735529
1 -3.447695 2.424446 0.743756
rd_5caC/rd_5caCaaj.xyz
6 2.280636 -0.417927 0.148921
6 0.504505 1.163161 -0.348744
6 -0.410853 0.276029 0.337485
6 0.145197 -0.886606 0.933273
1 -0.523004 -1.546876 1.478325
7 1.409002 -1.238260 0.861819
7 1.788935 0.752788 -0.438735
8 3.470149 -0.694047 0.026295
7 0.123567 2.304940 -0.875087
1 0.781015 2.910607 -1.359089
1 -0.832924 2.621041 -0.727352
6 -1.890707 0.547909 0.401889
8 -2.226608 1.727784 0.685281
8 -2.644463 -0.433507 0.167616
1 2.475938 1.332357 -0.921314
8 -1.837130 -2.756805 -1.195829
1 -2.089437 -1.951506 -0.695499
1 -0.930367 -2.944690 -0.914887
rd_5caC/rd_5caCabp.xyz
6 1.193950 -1.818709 -0.015389

6 0.259184 0.424265 0.039757
6 -1.056921 -0.170466 -0.065322
6 -1.113478 -1.584494 -0.180632
1 -2.090292 -2.046896 -0.285525
7 -0.071515 -2.384929 -0.165711
7 1.309075 -0.432951 0.094072
8 2.208218 -2.511703 0.015929
7 0.457475 1.718262 0.082927
1 1.419728 2.107054 0.129608
1 -0.354671 2.322378 -0.037363
6 -2.319260 0.649606 -0.002351
8 -2.307682 1.775738 -0.571522
8 -3.265417 0.117588 0.636299
1 2.256056 -0.064470 0.175277
8 3.138133 2.517829 0.160617
1 3.303126 3.341874 -0.322669
1 3.633959 1.840560 -0.325302
rd_5caC/rd_5caCaao.xyz
6 -2.793910 -0.165861 0.061929
6 -0.657070 0.984683 0.008343
6 -0.013259 -0.308428 -0.122354
6 -0.867219 -1.438808 -0.216864
1 -0.406398 -2.411381 -0.358574
7 -2.178446 -1.401726 -0.139422
7 -2.004406 0.985732 0.118655
8 -4.010939 -0.065804 0.180173
7 0.028316 2.102725 0.021782
1 -0.425955 3.006090 0.127751
1 1.039039 2.030807 -0.126587
6 1.488629 -0.477553 -0.095198
8 2.188313 0.508705 -0.478221
8 1.899224 -1.584015 0.331222
1 -2.502695 1.868986 0.226351
8 4.868451 0.209243 0.198663
1 3.920825 0.292157 -0.056286
1 4.953519 -0.703002 0.510411

rd_5caC/rd_5caCade.xyz
6 2.287082 -0.475996 -0.138176
6 0.545471 1.144947 0.354721
6 -0.385663 0.283750 -0.344552
6 0.146509 -0.890889 -0.938638
1 -0.533501 -1.532205 -1.491384
7 1.401487 -1.271734 -0.861882
7 1.818413 0.701659 0.453879
8 3.469297 -0.779350 -0.009823
7 0.186509 2.293196 0.882330
1 0.852189 2.880347 1.377871
1 -0.761483 2.629826 0.720773
6 -1.857571 0.593026 -0.427985
8 -2.164635 1.793455 -0.652446
8 -2.636425 -0.384356 -0.267210
1 2.514915 1.261286 0.946359
8 -1.894645 -2.713473 1.117551
1 -2.375378 -2.662946 1.955509
1 -2.145315 -1.897386 0.633782
rd_5caC/rd_5caCadd.xyz
6 2.595684 -0.215529 0.041275
6 0.234350 -0.806616 -0.099992
6 -0.068763 0.600107 0.019711
6 1.031748 1.492967 0.115633
1 0.817432 2.556106 0.183906
7 2.296591 1.139005 0.135954
7 1.551409 -1.133623 -0.089093
8 3.753957 -0.627750 0.063804
7 -0.675783 -1.745673 -0.228285
1 -0.384001 -2.716340 -0.316634
1 -1.695257 -1.559759 -0.201625
6 -1.467624 1.148231 0.022802
8 -2.192495 0.875026 1.014728
8 -1.759152 1.844217 -0.981600
1 1.825898 -2.112542 -0.166194
8 -3.486473 -1.420759 -0.073467

1 -3.427287 -0.588245 0.431638
1 -3.821378 -2.078025 0.554584
rd_5caC/rd_5caCaas.xyz
6 -1.273166 1.740751 -0.020242
6 -0.236067 -0.456656 0.017759
6 1.052627 0.199556 -0.064700
6 1.044694 1.615565 -0.163801
1 1.999709 2.124346 -0.251950
7 -0.033541 2.366419 -0.150613
7 -1.325767 0.350192 0.072066
8 -2.317377 2.387739 0.009569
7 -0.370891 -1.759372 0.041925
1 -1.314122 -2.190269 0.089757
1 0.473594 -2.318768 -0.076605
6 2.352174 -0.561541 0.005605
8 2.388124 -1.697385 -0.543988
8 3.277166 0.023124 0.628844
1 -2.255021 -0.065671 0.136079
8 -3.041020 -2.604516 0.104197
1 -3.461625 -2.113262 0.827002
1 -3.427851 -2.234794 -0.704625
rd_5caC/rd_5caCabc.xyz
6 1.188383 -1.826084 -0.026392
6 0.259658 0.420081 0.014613
6 -1.058321 -0.173381 -0.064796
6 -1.120647 -1.589002 -0.153891
1 -2.100558 -2.050279 -0.232152
7 -0.080495 -2.391903 -0.143489
7 1.308397 -0.438886 0.061124
8 2.202308 -2.520106 -0.003240
7 0.462287 1.714095 0.044351
1 1.425610 2.100222 0.094760
1 -0.345831 2.323409 -0.070253
6 -2.314761 0.653155 0.000017
8 -2.321471 1.745207 -0.631176
8 -3.236074 0.162304 0.704461

1 2.257824 -0.070700 0.108779
8 3.123791 2.557595 0.204553
1 3.716613 1.815384 0.009818
1 3.340711 3.230199 -0.458941
rd_5caC/rd_5caCadv.xyz
6 2.794462 -0.160771 0.060905
6 0.654117 0.983629 0.010344
6 0.014418 -0.311149 -0.122536
6 0.871605 -1.438899 -0.219474
1 0.413523 -2.412564 -0.362757
7 2.182722 -1.398080 -0.142539
7 2.001496 0.988392 0.120164
8 4.011223 -0.057076 0.178909
7 -0.034595 2.099745 0.026420
1 0.417299 3.004078 0.134118
1 -1.044810 2.026418 -0.123235
6 -1.486527 -0.484733 -0.094716
8 -2.189065 0.496020 -0.486035
8 -1.893415 -1.589164 0.340612
1 2.497304 1.872871 0.229188
8 -4.874035 0.211397 0.178029
1 -3.923713 0.283777 -0.069885
1 -4.933087 -0.622852 0.664993
rd_5caC/rd_5caCacj.xyz
6 2.730215 -0.508931 0.058565
6 0.862383 1.040420 0.042087
6 -0.024775 -0.095620 -0.125359
6 0.590169 -1.368728 -0.252221
1 -0.052960 -2.227129 -0.417337
7 1.882294 -1.593631 -0.170808
7 2.183717 0.773031 0.148517
8 3.942654 -0.656914 0.173142
7 0.409387 2.269401 0.093057
1 1.030080 3.063931 0.222920
1 -0.597630 2.394813 -0.053969
6 -1.532866 0.040205 -0.129212

8 -2.015119 1.161223 -0.448519
8 -2.164768 -1.000492 0.201870
1 2.845953 1.537758 0.280684
8 -4.922445 -0.640671 0.279144
1 -3.942962 -0.737698 0.244726
1 -5.083595 0.277473 0.019340
rd_5caC/rd_5caCabo.xyz
6 2.282005 -0.361123 -0.164886
6 0.460662 1.156776 0.364583
6 -0.425478 0.265047 -0.350584
6 0.162862 -0.869980 -0.967970
1 -0.484561 -1.535484 -1.531285
7 1.434951 -1.189805 -0.896386
7 1.756936 0.783921 0.444884
8 3.478383 -0.607408 -0.043880
7 0.044700 2.268507 0.928011
1 0.683409 2.877798 1.432178
1 -0.919473 2.564112 0.793625
6 -1.910720 0.499098 -0.416038
8 -2.274522 1.662719 -0.728748
8 -2.639364 -0.494048 -0.153699
1 2.424482 1.368037 0.948900
8 -1.695172 -2.778388 1.216770
1 -0.729433 -2.722053 1.190426
1 -2.001120 -1.992677 0.716426
rd_5caC/rd_5caCack.xyz
6 -2.599055 -0.197305 0.026401
6 -0.241803 -0.813193 -0.073795
6 0.073034 0.592724 0.022977
6 -1.019914 1.497563 0.085503
1 -0.796066 2.559758 0.135166
7 -2.288331 1.156009 0.096235
7 -1.561808 -1.127367 -0.072695
8 -3.761290 -0.598428 0.043715
7 0.660962 -1.762971 -0.173643
1 0.361515 -2.732535 -0.246472

1 1.681774 -1.581878 -0.153363
6 1.477654 1.125189 0.038944
8 1.798551 1.791660 -0.976327
8 2.179115 0.866712 1.051584
1 -1.844530 -2.104968 -0.134712
8 3.464722 -1.473400 0.032661
1 3.417987 -0.592757 0.449791
1 3.915277 -1.329538 -0.812925
rd_5caC/rd_5caCaba.xyz
6 1.467921 -0.922221 -0.105691
6 -0.221877 0.816862 -0.037054
6 -1.228978 -0.223282 -0.071037
6 -0.762434 -1.560748 -0.180746
1 -1.502850 -2.352560 -0.242342
7 0.501621 -1.918671 -0.205548
7 1.068739 0.412886 -0.029963
8 2.668105 -1.193096 -0.090539
7 -0.529768 2.092395 -0.011663
1 0.185944 2.812762 0.036181
1 -1.516024 2.346210 -0.087493
6 -2.699833 0.073744 0.069596
8 -3.127717 1.145222 -0.441689
8 -3.353901 -0.789475 0.711548
1 1.816763 1.106146 0.007345
8 4.471914 1.033406 0.341605
1 3.984841 0.211762 0.143847
1 5.151171 1.094837 -0.345126
rd_5caC/rd_5caCabd_b.xyz
6 1.462530 -0.929213 -0.001540
6 -0.224275 0.813434 0.054945
6 -1.230226 -0.218465 -0.078708
6 -0.762930 -1.552216 -0.222228
1 -1.501073 -2.336859 -0.357074
7 0.499255 -1.915146 -0.190487
7 1.062791 0.402717 0.120091
8 2.659982 -1.205835 0.052032

7 -0.532326 2.087820 0.116594
1 0.180303 2.804545 0.227144
1 -1.512869 2.345130 -0.010069
6 -2.705060 0.079481 -0.001318
8 -3.102586 1.176561 -0.481957
8 -3.393840 -0.808680 0.566084
1 1.808670 1.091335 0.222437
8 4.478040 1.068480 -0.005621
1 4.021657 0.208259 0.036934
1 5.422278 0.857521 0.006039
rd_5caU/rd_5caUacz.xyz
6 1.499926 -1.230046 0.002040
6 0.022437 0.699584 0.008622
6 -1.098224 -0.251139 -0.000941
6 -0.802550 -1.635473 -0.007907
1 -1.624404 -2.345056 -0.014527
7 0.416698 -2.127109 -0.006576
7 1.263574 0.134628 0.008832
8 2.652157 -1.661070 0.004399
8 -0.142486 1.932591 0.015967
1 2.095865 0.762894 0.014513
6 -2.519504 0.206705 -0.002947
8 -3.444356 -0.595300 -0.016731
8 -2.740474 1.521129 0.010973
1 -1.872539 2.006712 0.017433
8 3.550778 1.748035 0.054657
1 3.743099 2.082718 -0.834621
1 4.278621 1.139236 0.254108
rd_5caU/rd_5caUaau.xyz
6 -1.493066 -1.281566 -0.008205
6 -0.052088 0.675491 -0.010544
6 1.086038 -0.253752 0.001570
6 0.816495 -1.643598 0.004610
1 1.651831 -2.337273 0.010807
7 -0.393202 -2.158116 -0.000364
7 -1.282214 0.086881 -0.017460

8 -2.637090 -1.734050 -0.007441
8 0.088164 1.911686 -0.015393
1 -2.121909 0.705763 -0.024301
6 2.498193 0.230767 0.007558
8 3.438028 -0.553584 0.021980
8 2.694227 1.549257 -0.003501
1 1.817610 2.019004 -0.011591
8 -3.512083 1.769680 -0.071465
1 -4.125736 1.541903 0.643547
1 -3.197285 2.661290 0.142925
rd_5caU/rd_5caUabj.xyz
6 1.491884 -1.287686 0.008919
6 0.055520 0.672942 0.011067
6 -1.084735 -0.253651 -0.001063
6 -0.818581 -1.644141 -0.002649
1 -1.655625 -2.335762 -0.007939
7 0.389866 -2.161537 0.002848
7 1.284390 0.081394 0.016941
8 2.634717 -1.743035 0.007625
8 -0.082102 1.909399 0.016940
1 2.125274 0.698446 0.021365
6 -2.495755 0.234147 -0.008608
8 -3.437358 -0.548061 -0.024022
8 -2.688794 1.553082 0.001833
1 -1.811122 2.020851 0.010926
8 3.516497 1.765873 0.061010
1 4.020442 1.679236 -0.762662
1 3.167562 2.670511 0.046708
rd_5caU/rd_5caUacu.xyz
6 1.498024 1.244070 0.006962
6 0.030301 -0.692794 0.016063
6 -1.095039 0.252153 0.000090
6 -0.806291 1.638033 -0.005464
1 -1.631802 2.343360 -0.012827
7 0.410462 2.135828 -0.001831
7 1.268321 -0.121455 0.021289

8 2.648367 1.680271 0.003148
8 -0.127947 -1.926771 0.025319
1 2.103162 -0.746639 0.029540
6 -2.513855 -0.212967 -0.008317
8 -3.442715 0.584246 -0.028298
8 -2.728142 -1.528531 0.006221
1 -1.857872 -2.009848 0.017743
8 3.551460 -1.734360 0.069352
1 4.281799 -1.193653 -0.269069
1 3.466208 -2.463644 -0.563535
rd_5caU/rd_5caUabn.xyz
6 2.718493 -0.018159 -0.292320
6 0.536077 -0.968249 0.265073
6 0.018909 0.405807 0.224335
6 0.918961 1.457833 -0.073140
1 0.540026 2.474708 -0.101514
7 2.198246 1.285880 -0.322008
7 1.871711 -1.081899 -0.006057
8 3.908481 -0.218193 -0.517032
8 -0.172700 -1.953751 0.522364
1 2.282480 -2.014456 0.007251
6 -1.417466 0.711995 0.488253
8 -1.845194 1.855552 0.435138
8 -2.220280 -0.315603 0.779395
1 -1.704723 -1.166109 0.766676
8 -4.405325 -0.426764 -1.239488
1 -3.729835 -0.430188 -0.540384
1 -4.647344 -1.357109 -1.351793
rd_5caU/rd_5caUaah.xyz
6 -1.495241 -0.870034 0.008737
6 0.228193 0.858642 0.004925
6 1.210207 -0.233658 -0.002049
6 0.733484 -1.567833 -0.007923
1 1.455242 -2.378958 -0.016219
7 -0.539273 -1.896489 -0.004444
7 -1.079093 0.451656 0.008833

8 -2.694323 -1.145040 0.020360
8 0.544006 2.057523 0.007434
1 -1.802956 1.170001 0.016450
6 2.680817 0.030281 -0.005073
8 3.487529 -0.889836 -0.011009
8 3.078599 1.302486 -0.001189
1 2.290372 1.905858 0.002441
8 -4.626111 1.023445 0.077139
1 -4.026652 0.256120 0.024792
1 -5.049799 1.067799 -0.791751
rd_5caU/rd_5caUaaq.xyz
6 -1.496929 -0.877134 0.009254
6 0.222100 0.855796 0.007417
6 1.206900 -0.234015 -0.001985
6 0.733558 -1.569382 -0.009689
1 1.457305 -2.378724 -0.019579
7 -0.538383 -1.901201 -0.006201
7 -1.084164 0.445640 0.011439
8 -2.695330 -1.155228 0.021039
8 0.534883 2.055476 0.011933
1 -1.809699 1.162356 0.022128
6 2.676817 0.033898 -0.005563
8 3.486103 -0.883934 -0.013879
8 3.070932 1.307234 0.000382
1 2.280796 1.908173 0.005434
8 -4.616402 1.022969 0.077216
1 -4.020888 0.252259 0.027756
1 -4.845850 1.217749 -0.842532
rd_5caU/rd_5caUabq.xyz
6 2.720342 -0.021194 0.290176
6 0.535445 -0.966788 -0.265033
6 0.020490 0.407988 -0.221122
6 0.922800 1.458067 0.076386
1 0.545584 2.475514 0.106976
7 2.202246 1.283636 0.322719
7 1.871197 -1.083141 0.004272

8 3.910493 -0.223477 0.511982
8 -0.175242 -1.950690 -0.523239
1 2.280359 -2.016370 -0.011260
6 -1.415955 0.716793 -0.481842
8 -1.842312 1.860607 -0.423628
8 -2.220104 -0.308743 -0.776233
1 -1.705818 -1.160066 -0.766395
8 -4.432197 -0.436873 1.210663
1 -3.737868 -0.431416 0.530223
1 -4.520189 -1.366909 1.463772
rd_5caU/rd_5caUabx.xyz
6 2.774666 -0.453012 0.000012
6 0.871446 1.080876 -0.000001
6 0.014781 -0.111075 -0.000013
6 0.630938 -1.386450 -0.000023
1 0.000103 -2.270017 -0.000040
7 1.931788 -1.576223 -0.000010
7 2.213840 0.817636 0.000009
8 3.994355 -0.591398 0.000030
8 0.424852 2.238360 -0.000001
1 2.853582 1.610977 0.000017
6 -1.472871 -0.004327 -0.000017
8 -2.174968 -1.010263 -0.000018
8 -2.005928 1.212607 -0.000021
1 -1.285323 1.898525 -0.000013
8 -5.023140 -0.634032 0.000043
1 -4.050888 -0.729956 -0.000010
1 -5.172018 0.322312 0.000054
rd_5caU/rd_5caUacy.xyz
6 2.757472 -0.526980 -0.006694
6 0.921164 1.086275 -0.003894
6 0.014746 -0.068515 0.008040
6 0.576587 -1.368624 0.013648
1 -0.091620 -2.224230 0.024112
7 1.868311 -1.613510 0.006804
7 2.251109 0.766284 -0.008587

8 3.970096 -0.717166 -0.016561
8 0.522805 2.261124 -0.009005
1 2.924793 1.530720 -0.018725
6 -1.467729 0.098926 0.017571
8 -2.208448 -0.878672 0.028401
8 -1.952328 1.335705 0.014058
1 -1.206434 1.992780 0.004558
8 -5.060902 -0.576350 -0.003697
1 -4.085942 -0.638709 0.007022
1 -5.359964 -1.453595 -0.282073
rd_5caU/rd_5caUabv.xyz
6 1.952700 -0.155348 -0.722915
6 -0.135185 1.051549 -0.334083
6 -0.787279 -0.262575 -0.254409
6 0.007893 -1.421105 -0.438479
1 -0.470875 -2.394121 -0.390374
7 1.301818 -1.398746 -0.670233
7 1.205499 1.008946 -0.599495
8 3.167440 -0.097139 -0.888360
8 -0.743188 2.121807 -0.177813
1 1.709025 1.893921 -0.643897
6 -2.251115 -0.399677 0.008723
8 -2.784908 -1.499840 0.056219
8 -2.950111 0.720565 0.190928
1 -2.351128 1.508451 0.109566
8 1.837715 -0.088699 2.631663
1 1.630586 -0.164026 3.574342
1 0.993502 -0.246232 2.184349
rd_5caU/rd_5caUabl.xyz
6 -1.233997 -1.482354 -0.230740
6 1.140499 -1.087300 0.193499
6 0.878563 0.325968 -0.111481
6 -0.440318 0.705752 -0.461465
1 -0.637734 1.748113 -0.692140
7 -1.455843 -0.126790 -0.523742
7 0.045257 -1.902571 0.109630

8 -2.160524 -2.286257 -0.275457
8 2.261405 -1.519296 0.502697
1 0.174057 -2.892764 0.312641
6 1.960715 1.355396 -0.067628
8 1.729011 2.526375 -0.336953
8 3.184055 0.951546 0.275972
1 3.183609 -0.025159 0.451119
8 -4.596959 1.979150 0.507249
1 -4.651766 1.013074 0.469469
1 -5.362748 2.245335 1.036524
rd_5caU/rd_5caUabs.xyz
6 -1.561433 0.508928 -0.160941
6 0.789217 1.141831 0.014893
6 1.096548 -0.293642 -0.034293
6 0.020192 -1.208967 -0.139912
1 0.241526 -2.271413 -0.170939
7 -1.244699 -0.856633 -0.198106
7 -0.543857 1.444433 -0.067433
8 -2.733994 0.877789 -0.210026
8 1.657598 2.019199 0.129225
1 -0.811814 2.427307 -0.035412
6 2.499303 -0.803755 0.032169
8 2.739441 -1.999874 -0.059561
8 3.475228 0.089660 0.197214
1 3.099221 1.006438 0.234164
8 -4.931137 -0.895011 0.290163
1 -4.595259 -1.714105 0.680809
1 -4.133837 -0.363295 0.102527
rd_5caU/rd_5caUack.xyz
6 2.825440 -0.035383 0.144999
6 0.624961 1.000302 -0.096936
6 0.074991 -0.362285 -0.112448
6 0.970765 -1.452992 0.007820
1 0.568029 -2.461223 0.001148
7 2.273838 -1.327331 0.128551
7 1.984723 1.064556 0.031372

8 4.037348 0.122512 0.258836
8 -0.078699 2.018045 -0.188679
1 2.417816 1.986808 0.049206
6 -1.390164 -0.615026 -0.240240
8 -1.842011 -1.750677 -0.256946
8 -2.188777 0.449769 -0.334633
1 -1.657740 1.289095 -0.296531
8 -5.008023 0.362744 0.449185
1 -5.067988 -0.141373 1.273078
1 -4.064710 0.339280 0.212364
rd_5caU/rd_5caUacv.xyz
6 -2.305121 0.201716 -0.114890
6 -0.186848 -0.984440 -0.404854
6 0.466516 0.328432 -0.325900
6 -0.350841 1.478042 -0.172856
1 0.127911 2.451388 -0.129608
7 -1.658105 1.446114 -0.070740
7 -1.549823 -0.950423 -0.296257
8 -3.525826 0.128442 -0.001015
8 0.435126 -2.047071 -0.558698
1 -2.050987 -1.836915 -0.332907
6 1.949674 0.474333 -0.403156
8 2.483801 1.574700 -0.356841
8 2.670492 -0.642259 -0.513814
1 2.065555 -1.429378 -0.553351
8 0.718535 -0.381748 2.365828
1 1.283973 0.392735 2.506330
1 1.331742 -1.092682 2.124785
rd_5caU/rd_5caUabm.xyz
6 1.499686 -0.876954 0.010821
6 -0.220924 0.855015 0.011472
6 -1.204857 -0.235496 -0.000561
6 -0.730523 -1.570466 -0.007974
1 -1.453672 -2.380332 -0.018149
7 0.541648 -1.901449 -0.003814
7 1.085632 0.445667 0.017173

8 2.698091 -1.154335 0.018287
8 -0.534357 2.054496 0.016852
1 1.809965 1.163322 0.029612
6 -2.674988 0.031150 -0.006596
8 -3.483418 -0.887411 -0.015135
8 -3.070347 1.304135 -0.002990
1 -2.280976 1.905950 0.003469
8 4.681842 0.970795 -0.009583
1 4.143396 1.748694 -0.215088
1 4.045478 0.231901 0.004220
rd_5caU/rd_5caUaca.xyz
6 1.492715 -0.852577 0.005045
6 -0.241963 0.865128 0.000342
6 -1.216846 -0.233436 -0.001411
6 -0.731494 -1.564505 -0.003121
1 -1.448110 -2.380188 -0.006626
7 0.543332 -1.885024 -0.000937
7 1.067961 0.466338 0.002725
8 2.693498 -1.119808 0.012446
8 -0.565493 2.061917 -0.000335
1 1.786923 1.189302 0.004291
6 -2.689196 0.020332 -0.002777
8 -3.489258 -0.905584 -0.003914
8 -3.096298 1.289597 -0.002640
1 -2.312899 1.899024 -0.001895
8 4.658427 1.018086 0.062060
1 4.038948 0.266391 0.016387
1 5.369795 0.792955 -0.554082
rd_5caU/rd_5caUadp.xyz
6 -1.581691 -0.900722 0.000312
6 0.844819 -1.187579 -0.000283
6 0.943575 0.279395 0.000021
6 -0.255462 1.035722 0.000206
1 -0.194741 2.119944 0.000292
7 -1.453870 0.499073 0.000268
7 -0.433691 -1.677864 -0.000044

8 -2.693382 -1.418355 0.000682
8 1.835652 -1.932293 -0.000685
1 -0.554965 -2.689675 -0.000160
6 2.262665 0.982075 0.000153
8 2.323813 2.204099 0.000567
8 3.363082 0.230801 -0.000128
1 3.120325 -0.730928 -0.000465
8 -4.137677 1.677745 -0.001824
1 -4.733315 0.914950 0.008186
1 -3.239716 1.287922 -0.000781
rd_5caU/rd_5caUaai.xyz
6 -2.763757 -0.505587 -0.000253
6 -0.907368 1.084517 -0.000116
6 -0.015422 -0.081414 0.000218
6 -0.593407 -1.374469 0.000324
1 0.063910 -2.238519 0.000615
7 -1.888089 -1.603045 0.000101
7 -2.241268 0.781220 -0.000295
8 -3.978726 -0.680681 -0.000525
8 -0.494671 2.254437 -0.000220
1 -2.905072 1.554393 -0.000550
6 1.468935 0.067909 0.000637
8 2.198271 -0.918406 0.000871
8 1.967802 1.298973 0.000795
1 1.229173 1.964636 0.000443
8 5.043930 -0.528478 -0.001745
1 5.415084 -1.422066 -0.000229
1 4.075674 -0.658169 0.002820
rd_5caU/rd_5caUabr.xyz
6 1.559029 0.479123 -0.205812
6 -0.780429 1.138588 0.015278
6 -1.106265 -0.292445 -0.040865
6 -0.043815 -1.219134 -0.181866
1 -0.278887 -2.278186 -0.225174
7 1.224120 -0.881493 -0.258780
7 0.554809 1.425753 -0.085009

8 2.735264 0.834166 -0.267180
8 -1.636305 2.025541 0.148833
1 0.835126 2.404907 -0.045497
6 -2.512834 -0.787084 0.053401
8 -2.767212 -1.981127 -0.026748
8 -3.476642 0.117320 0.229693
1 -3.090102 1.029833 0.260997
8 4.862845 -0.999481 0.360991
1 5.502440 -0.474392 0.862641
1 4.141191 -0.377618 0.148033
rd_5dhmC/rd_5dhmC_006.xyz
6 -1.565561 1.208635 0.059548
6 -0.180377 -0.656062 -0.092696
6 0.990124 0.213561 -0.102507
6 0.732243 1.596674 0.019193
1 1.566687 2.289080 0.035322
7 -0.476669 2.109868 0.114143
7 -1.406808 -0.145034 -0.033336
8 -2.711554 1.701809 0.106466
6 2.384151 -0.339233 -0.306934
1 2.439689 -0.789311 -1.306670
7 -0.039644 -1.982094 -0.170181
1 -0.867725 -2.566397 -0.151274
1 0.873201 -2.412249 -0.081385
8 2.664698 -1.407852 0.585005
1 2.566882 -1.092314 1.499903
8 3.311510 0.711339 -0.178394
1 4.161531 0.404181 -0.530203
8 -3.767577 -1.706553 -0.062850
1 -2.943085 -1.161201 -0.052571
1 -4.475427 -1.082353 0.151051
rd_5dhmC/rd_5dhmC_021.xyz
6 2.401440 -0.183322 0.085919
6 0.471604 0.893645 -0.605435
6 -0.284516 -0.343352 -0.442174
6 0.443485 -1.448540 0.048678

1 -0.068022 -2.390332 0.213104
7 1.729362 -1.405777 0.326435
7 1.776990 0.945044 -0.361496
8 3.631419 -0.174203 0.314871
6 -1.752829 -0.427549 -0.801241
1 -1.865983 -0.245228 -1.878371
7 -0.149584 1.997942 -1.038007
1 0.381873 2.854951 -1.132787
1 -1.160565 2.036688 -1.084239
8 -2.502357 0.587864 -0.162726
1 -2.248163 0.616427 0.793378
8 -2.220052 -1.718620 -0.472285
1 -3.087406 -1.834138 -0.889516
8 -1.484313 0.668616 2.391635
1 -1.920519 1.310204 2.972509
1 -0.601270 1.036422 2.230965
rd_5dhmC/rd_5dhmC_066.xyz
6 2.755095 -0.495599 -0.114870
6 1.010410 1.028357 -0.158907
6 0.055807 -0.048668 0.082243
6 0.612634 -1.333747 0.260096
1 -0.046998 -2.169680 0.465732
7 1.902433 -1.586540 0.186232
7 2.312335 0.785945 -0.271172
8 3.973932 -0.757144 -0.225078
6 -1.440532 0.184307 0.051399
1 -1.718242 0.522512 -0.956554
7 0.571684 2.285396 -0.301145
1 1.244319 3.027503 -0.450869
1 -0.386232 2.523318 -0.074112
8 -1.820733 1.265543 0.901179
1 -1.563671 1.050558 1.813854
8 -2.107374 -1.001500 0.382369
1 -3.035242 -0.922870 0.047038
8 -4.616601 -0.669449 -0.617772
1 -4.595358 0.186163 -1.073471

1 -4.798017 -1.318596 -1.314359
rd_5dhmC/rd_5dhmC_005.xyz
6 -2.764430 0.487928 -0.103769
6 -1.015229 -1.030660 -0.147767
6 -0.061722 0.052812 0.066186
6 -0.621407 1.336870 0.242607
1 0.037186 2.176596 0.435740
7 -1.913039 1.583740 0.183247
7 -2.319122 -0.793926 -0.249761
8 -3.984444 0.745561 -0.209848
6 1.434675 -0.176151 0.017691
1 1.699477 -0.538388 -0.985401
7 -0.574535 -2.288579 -0.274424
1 -1.246225 -3.034444 -0.408522
1 0.387310 -2.521226 -0.059452
8 1.830122 -1.235394 0.888194
1 1.579391 -1.001828 1.798069
8 2.100835 1.019900 0.310957
1 3.030759 0.931504 -0.016224
8 4.619086 0.725048 -0.679729
1 4.585608 -0.001586 -1.320997
1 5.217261 0.415018 0.017066
rd_5dhmC/rd_5dhmC_044.xyz
6 2.403546 -0.244096 0.027086
6 0.528811 1.093432 -0.218003
6 -0.276054 -0.083097 -0.524576
6 0.392097 -1.323896 -0.441211
1 -0.164946 -2.238292 -0.613116
7 1.672666 -1.442379 -0.161348
7 1.832622 0.995623 0.018323
8 3.633186 -0.369066 0.221329
6 -1.736410 0.039153 -0.896232
1 -1.827408 0.665723 -1.792838
7 -0.039968 2.305368 -0.203645
1 0.530692 3.109851 0.027154
1 -1.046761 2.408563 -0.227498

8 -2.462003 0.709389 0.116140
1 -2.236867 0.293195 0.986059
8 -2.246567 -1.252544 -1.152416
1 -3.108477 -1.149358 -1.584605
8 -1.624870 -0.466301 2.458727
1 -1.999523 -1.360235 2.476229
1 -0.673866 -0.600542 2.322674
rd_5dhmC/rd_5dhmC_045.xyz
6 -1.563887 1.216594 0.053159
6 -0.184160 -0.651847 -0.102645
6 0.989144 0.214005 -0.103526
6 0.735291 1.597287 0.024746
1 1.571927 2.286827 0.048206
7 -0.472331 2.113953 0.117517
7 -1.409168 -0.136999 -0.046712
8 -2.708559 1.713050 0.098001
6 2.382340 -0.342440 -0.303864
1 2.440889 -0.789726 -1.304669
7 -0.047539 -1.978026 -0.184917
1 -0.877634 -2.559625 -0.172666
1 0.863330 -2.411855 -0.094167
8 2.655765 -1.414485 0.586196
1 2.555430 -1.101085 1.501567
8 3.312231 0.704987 -0.168180
1 4.163072 0.395991 -0.516339
8 -3.753336 -1.721528 -0.041475
1 -4.473064 -1.113980 0.180529
1 -2.941855 -1.156832 -0.047239
rd_5dhmC/rd_5dhmC_029.xyz
6 -1.551803 1.269255 0.045206
6 -0.210645 -0.626326 -0.117046
6 0.980535 0.214445 -0.105179
6 0.755491 1.601778 0.032351
1 1.606266 2.273391 0.065708
7 -0.441558 2.142937 0.122380
7 -1.425505 -0.086453 -0.066125

8 -2.685679 1.789859 0.089350
6 2.362786 -0.371034 -0.297706
1 2.417245 -0.823005 -1.296553
7 -0.100430 -1.954696 -0.205098
1 -0.941981 -2.519932 -0.201386
1 0.800698 -2.407476 -0.111428
8 2.609399 -1.445435 0.597525
1 2.509542 -1.127052 1.511208
8 3.313050 0.657766 -0.160905
1 4.158693 0.331597 -0.505954
8 -3.694800 -1.780631 -0.079802
1 -2.934332 -1.149017 -0.060121
1 -4.197626 -1.592189 0.725330
rd_5dhmC/rd_5dhmC_046.xyz
6 2.769315 -0.479524 -0.101210
6 1.012876 1.030834 -0.139279
6 0.063848 -0.059039 0.062473
6 0.629487 -1.341093 0.234387
1 -0.025447 -2.185160 0.420959
7 1.922600 -1.580836 0.178798
7 2.318303 0.801167 -0.238834
8 3.990397 -0.731467 -0.208718
6 -1.433369 0.161694 0.002957
1 -1.690408 0.519382 -1.003725
7 0.566705 2.287581 -0.256538
1 1.234930 3.037696 -0.383873
1 -0.398086 2.512876 -0.046529
8 -1.842806 1.221099 0.866585
1 -1.599698 0.991383 1.779517
8 -2.095108 -1.037421 0.294916
1 -3.026093 -0.947846 -0.028068
8 -4.606097 -0.670264 -0.704518
1 -4.684405 0.290854 -0.806495
1 -5.288085 -0.907385 -0.057840
rd_5dhmC/rd_5dhmC_091.xyz
6 2.764052 -0.485073 -0.104884

6 1.012374 1.030587 -0.150504
6 0.061033 -0.053835 0.068469
6 0.622909 -1.336604 0.246779
1 -0.034083 -2.176905 0.442896
7 1.914839 -1.581575 0.185811
7 2.316452 0.795681 -0.253915
8 3.984384 -0.740940 -0.211587
6 -1.435588 0.173272 0.022433
1 -1.702048 0.534596 -0.980481
7 0.569261 2.287344 -0.280817
1 1.239589 3.033934 -0.417770
1 -0.392290 2.519288 -0.063771
8 -1.830468 1.232814 0.892831
1 -1.578204 0.999973 1.802466
8 -2.100765 -1.022933 0.317262
1 -3.031369 -0.934644 -0.008389
8 -4.626512 -0.728301 -0.654676
1 -4.553475 -0.259889 -1.500336
1 -5.113778 -0.121717 -0.076559
rd_5dhmC/rd_5dhmC_053.xyz
6 -2.381758 0.207214 0.017974
6 -0.449513 -0.955724 -0.512655
6 0.319083 0.282886 -0.450725
6 -0.403846 1.436005 -0.075609
1 0.119966 2.380429 0.020457
7 -1.695558 1.435736 0.175535
7 -1.761611 -0.965776 -0.303480
8 -3.619203 0.236726 0.199261
6 1.793774 0.320416 -0.788316
1 1.923378 0.039024 -1.841918
7 0.166424 -2.106235 -0.812404
1 -0.373882 -2.963058 -0.825443
1 1.177775 -2.160555 -0.825952
8 2.521519 -0.638141 -0.045091
1 2.232872 -0.580122 0.898444
8 2.269870 1.632072 -0.572429

1 3.147291 1.697529 -0.979439
8 1.332132 -0.478865 2.445736
1 0.605584 -1.108595 2.317375
1 0.901240 0.390146 2.455086
rd_5dhmC/rd_5dhmC_069.xyz
6 -1.116693 1.922751 0.061240
6 -0.460318 -0.296499 -0.074864
6 0.938809 0.117802 -0.108285
6 1.177114 1.502876 0.018900
1 2.199016 1.865765 0.035991
7 0.218430 2.399002 0.122657
7 -1.441644 0.602507 -0.020352
8 -2.015256 2.794026 0.100234
6 2.054410 -0.886005 -0.290593
1 1.877438 -1.467371 -1.203754
7 -0.778391 -1.592294 -0.125322
1 -1.764282 -1.876109 -0.108540
1 -0.062179 -2.304427 -0.074592
8 2.052550 -1.846345 0.756618
1 2.125440 -1.382008 1.607970
8 3.278805 -0.195514 -0.371331
1 3.952542 -0.821312 -0.679403
8 -3.553911 -2.478922 -0.129044
1 -3.935290 -2.397380 0.757897
1 -4.038891 -1.833179 -0.664661
rd_5dhmC/rd_5dhmC_052.xyz
6 1.622229 0.549365 -0.073219
6 -0.598373 1.193611 -0.086272
6 -1.009359 -0.206883 -0.116516
6 0.036129 -1.155475 -0.062388
1 -0.206693 -2.212268 -0.053375
7 1.310204 -0.829797 -0.020773
7 0.686658 1.537304 -0.088033
8 2.841485 0.841162 -0.097327
6 -2.459757 -0.607827 -0.275948
1 -2.817971 -0.252825 -1.250981

7 -1.522260 2.159505 -0.084381
1 -1.222243 3.126960 -0.056455
1 -2.502091 1.939320 0.048904
8 -3.294578 0.040073 0.671961
1 -2.982798 -0.177842 1.566652
8 -2.553426 -2.009606 -0.190659
1 -3.427886 -2.270914 -0.518500
8 4.947472 -0.952266 0.204328
1 4.142537 -0.398617 0.097625
1 4.622102 -1.814545 0.498081
rd_5dhmC/rd_5dhmC_072.xyz
6 1.094497 1.952496 -0.051020
6 0.463364 -0.276226 0.041061
6 -0.941017 0.122091 0.080499
6 -1.193938 1.503768 -0.056750
1 -2.219715 1.854480 -0.089809
7 -0.244496 2.410559 -0.152999
7 1.434436 0.635102 0.009079
8 1.980284 2.837245 -0.029677
6 -2.042897 -0.886106 0.318721
1 -1.871282 -1.376851 1.285298
7 0.794323 -1.569090 0.067545
1 1.782579 -1.843319 0.049295
1 0.079279 -2.281166 -0.010954
8 -2.005317 -1.942318 -0.630623
1 -2.072103 -1.566619 -1.524837
8 -3.281157 -0.216346 0.313294
1 -3.947315 -0.820521 0.676166
8 3.588113 -2.404746 -0.004323
1 4.111623 -1.753313 0.486257
1 3.711646 -3.235502 0.478783
rd_5dhmC/rd_5dhmC_011.xyz
6 2.392061 -0.220343 0.000975
6 0.484946 1.054984 -0.315998
6 -0.305189 -0.160271 -0.476145
6 0.386188 -1.374184 -0.271540

1 -0.161463 -2.308955 -0.320192
7 1.675714 -1.442956 -0.018829
7 1.796396 1.002863 -0.109320
8 3.632102 -0.305148 0.142117
6 -1.769350 -0.103773 -0.853052
1 -1.865372 0.386941 -1.830317
7 -0.109201 2.250914 -0.410238
1 0.447053 3.087364 -0.280918
1 -1.119129 2.326888 -0.417204
8 -2.503017 0.699913 0.051294
1 -2.254817 0.424717 0.967818
8 -2.268697 -1.422580 -0.917723
1 -3.126484 -1.391671 -1.368424
8 -1.453094 -0.164839 2.450248
1 -0.580952 0.260223 2.444489
1 -1.269477 -1.098505 2.260516
rd_5dhmC/rd_5dhmC_041.xyz
6 -2.845191 0.137987 0.083508
6 -0.851888 -1.020843 -0.137343
6 -0.115617 0.238119 -0.139978
6 -0.891419 1.408000 0.008445
1 -0.397497 2.373155 0.024556
7 -2.201878 1.399095 0.129909
7 -2.176814 -1.045319 -0.040871
8 -4.092752 0.151058 0.170981
6 1.381255 0.278779 -0.341696
1 1.628850 -0.167123 -1.313562
7 -0.193127 -2.179918 -0.262237
1 -0.718282 -3.046096 -0.249821
1 0.815308 -2.221028 -0.190601
8 2.053029 -0.545420 0.609090
1 1.830236 -0.242482 1.506694
8 1.818741 1.612741 -0.274907
1 2.740710 1.639301 -0.576950
8 4.833863 -0.051110 0.132438
1 3.909393 -0.298351 0.329728

1 5.228114 -0.844783 -0.256076
rd_5dhmC/rd_5dhmC_073.xyz
6 -1.566016 -0.745781 -0.111524
6 0.089492 0.859438 0.064618
6 1.106856 -0.186698 0.098446
6 0.631978 -1.510130 -0.036126
1 1.341956 -2.329892 -0.043015
7 -0.642173 -1.816761 -0.155065
7 -1.203938 0.562074 -0.020155
8 -2.777553 -1.064277 -0.173233
6 2.566261 0.136437 0.330842
1 2.671435 0.624409 1.308263
7 0.447433 2.144528 0.148038
1 -0.273136 2.856404 0.129665
1 1.418502 2.424568 0.089634
8 3.042502 1.094377 -0.603074
1 2.909224 0.750956 -1.503023
8 3.309617 -1.057854 0.288853
1 4.187996 -0.875022 0.657299
8 -4.795507 0.840553 0.139233
1 -4.069399 0.186937 0.028814
1 -5.599737 0.310759 0.230871
rd_5dhmC/rd_5dhmC_017.xyz
6 1.621608 0.544744 -0.075832
6 -0.598017 1.192399 -0.089486
6 -1.011254 -0.207466 -0.117379
6 0.032913 -1.157728 -0.065871
1 -0.211513 -2.214146 -0.057497
7 1.307590 -0.834074 -0.026253
7 0.687536 1.534032 -0.091699
8 2.841275 0.834952 -0.096248
6 -2.462744 -0.606193 -0.272578
1 -2.822524 -0.252875 -1.247656
7 -1.520221 2.159922 -0.089117
1 -1.218296 3.126836 -0.062737
1 -2.500327 1.941830 0.045508

8 -3.294411 0.045353 0.675570
1 -2.981522 -0.171437 1.570152
8 -2.558646 -2.007612 -0.183714
1 -3.434654 -2.268107 -0.508059
8 4.955898 -0.937633 0.239400
1 4.143550 -0.400022 0.108826
1 4.652986 -1.856254 0.247759
rd_5dhmC/rd_5dhmC_094.xyz
6 1.567304 -0.758937 0.112487
6 -0.079906 0.855320 -0.058574
6 -1.102373 -0.185694 -0.098948
6 -0.634094 -1.512276 0.027715
1 -1.347921 -2.328699 0.027624
7 0.638339 -1.825733 0.146661
7 1.212051 0.551239 0.025804
8 2.776835 -1.083562 0.178068
6 -2.560171 0.145287 -0.330866
1 -2.662128 0.638694 -1.305900
7 -0.431779 2.142548 -0.135585
1 0.291761 2.851228 -0.110838
1 -1.401617 2.426401 -0.074520
8 -3.033043 1.100672 0.607311
1 -2.903449 0.751513 1.505572
8 -3.308725 -1.045919 -0.295410
1 -4.186156 -0.857539 -0.663303
8 4.873935 0.722502 -0.102336
1 4.077360 0.152762 -0.015046
1 4.585305 1.477531 -0.633699
rd_5dhmC/rd_5dhmC_075.xyz
6 -1.568038 -0.765764 -0.113782
6 0.075302 0.852521 0.057817
6 1.100214 -0.186061 0.098440
6 0.635159 -1.513703 -0.029495
1 1.351011 -2.328357 -0.029936
7 -0.636481 -1.830238 -0.149097
7 -1.215802 0.545217 -0.027211

8 -2.776986 -1.092910 -0.178120
6 2.557007 0.148365 0.331733
1 2.656865 0.641684 1.307031
7 0.423825 2.140629 0.135519
1 -0.301530 2.847469 0.110934
1 1.392968 2.426990 0.075120
8 3.028330 1.105279 -0.605691
1 2.900308 0.756157 -1.504197
8 3.308475 -1.040990 0.296456
1 4.185128 -0.850653 0.665197
8 -4.859815 0.726926 0.126931
1 -4.513210 1.568377 0.455022
1 -4.070226 0.150498 0.021465
rd_5dhmC/rd_5dhmC_070.xyz
6 1.569197 -0.790292 0.089631
6 -0.060844 0.841960 -0.078549
6 -1.095833 -0.187120 -0.098219
6 -0.641741 -1.517989 0.036815
1 -1.365247 -2.325695 0.053176
7 0.628155 -1.845761 0.144734
7 1.227990 0.523069 -0.004135
8 2.776038 -1.127336 0.142826
6 -2.551899 0.158982 -0.318735
1 -2.657722 0.644634 -1.297251
7 -0.397528 2.132599 -0.165336
1 0.335021 2.832407 -0.156529
1 -1.363159 2.429505 -0.100510
8 -3.004708 1.128379 0.615008
1 -2.871944 0.785726 1.515352
8 -3.313663 -1.023098 -0.265317
1 -4.192395 -0.827757 -0.626277
8 4.808880 0.767645 -0.103881
1 4.051989 0.146097 -0.017246
1 4.407485 1.647774 -0.092289
rd_5dhmU/rd_5dhmU_a15_aam.xyz
6 1.556508 1.136349 0.070627

6 -0.781145 1.154455 -0.017356
6 -0.794077 -0.327638 -0.114476
6 0.440614 -0.997777 -0.178960
1 0.518497 -2.075435 -0.287993
7 1.551043 -0.300740 -0.100312
7 0.398582 1.808291 0.092504
8 2.685091 1.642037 0.178834
8 -1.880830 1.762136 -0.031013
6 -2.088868 -1.103615 -0.091580
1 2.480858 -0.790069 -0.131754
8 -2.552689 -1.038638 1.245488
1 -3.108621 -1.815907 1.415561
8 -3.018631 -0.578664 -1.011031
1 -3.088421 0.379074 -0.822416
1 -1.914501 -2.141239 -0.389312
8 3.917125 -1.698559 -0.172358
1 4.520935 -1.365474 0.509447
1 3.745170 -2.620951 0.072237
rd_5dhmU/rd_5dhmU_a11_abk.xyz
6 -1.555292 1.139974 0.091298
6 0.780873 1.155652 -0.035589
6 0.791945 -0.328072 -0.104438
6 -0.443553 -0.999352 -0.129854
1 -0.523325 -2.079021 -0.214850
7 -1.552482 -0.300939 -0.043264
7 -0.396995 1.811967 0.080287
8 -2.682060 1.649100 0.201541
8 1.880532 1.762151 -0.076879
6 2.086094 -1.105586 -0.088456
1 -2.482545 -0.790680 -0.056112
8 3.003987 -0.596523 -1.028392
1 3.077112 0.364091 -0.856261
8 2.568594 -1.021819 1.240975
1 3.124889 -1.797950 1.415148
1 1.905661 -2.147245 -0.368068
8 -3.915832 -1.702823 -0.036427

1 -4.517321 -1.391343 -0.730243
1 -3.740316 -2.631421 -0.253086
rd_5dhmU/rd_5dhmU_a25_aas.xyz
6 1.606842 -1.089764 0.015571
6 -0.710529 -1.382552 0.150417
6 -0.900043 0.089894 0.173531
6 0.236570 0.911782 0.063891
1 0.182228 1.994465 0.042706
7 1.420866 0.347340 -0.006433
7 0.543186 -1.897438 0.088509
8 2.791909 -1.457981 -0.042782
8 -1.729545 -2.110198 0.190539
6 -2.296300 0.647823 0.219004
1 2.287079 0.938646 -0.077501
8 -2.237140 2.006416 0.554601
1 -3.136645 2.309424 0.755680
8 -2.835443 0.522860 -1.100776
1 -3.258478 -0.348938 -1.177080
1 -2.909914 0.080069 0.925057
8 3.662579 1.926030 -0.231297
1 3.837967 2.398588 0.597301
1 4.411275 1.318343 -0.337452
rd_5dhmU/rd_5dhmU_a14_aar.xyz
6 -1.547044 1.129619 -0.000797
6 0.787740 1.113362 0.004363
6 0.785605 -0.370845 -0.001242
6 -0.453605 -1.033904 -0.008070
1 -0.543061 -2.116023 -0.012640
7 -1.556067 -0.318269 -0.008302
7 -0.381241 1.791155 0.004805
8 -2.671222 1.654920 -0.000682
8 1.903922 1.692439 0.008066
6 2.090063 -1.131084 -0.001179
1 -2.492140 -0.797180 -0.008969
8 2.815238 -0.844852 -1.179106
1 2.960552 0.118262 -1.218516

8 2.816690 -0.845363 1.175742
1 2.961051 0.117965 1.215286
1 1.907428 -2.206543 -0.001410
8 -3.906472 -1.738170 -0.019084
1 -4.337512 -1.687031 0.848110
1 -4.536967 -1.334324 -0.635324
rd_5dhmU/rd_5dhmU_a11_aaa.xyz
6 -1.563778 1.092996 0.091828
6 0.771092 1.149957 -0.040926
6 0.808535 -0.333698 -0.105741
6 -0.415013 -1.026974 -0.126997
1 -0.476180 -2.108004 -0.209909
7 -1.535495 -0.347707 -0.040383
7 -0.417889 1.785875 0.076414
8 -2.699721 1.581129 0.205000
8 1.859803 1.775795 -0.085339
6 2.116301 -1.088653 -0.086854
1 -2.461389 -0.845578 -0.056762
8 3.029678 -0.560619 -1.020519
1 3.081469 0.401366 -0.847073
8 2.590982 -1.000843 1.245260
1 3.163141 -1.765638 1.418396
1 1.955010 -2.132401 -0.370488
8 -3.932388 -1.692568 -0.049199
1 -4.631119 -1.019460 -0.057523
1 -4.046896 -2.182379 -0.878343
rd_5dhmU/rd_5dhmU_a14_aal.xyz
6 2.480829 0.320320 -0.055650
6 0.245229 0.716740 0.510926
6 -0.101348 -0.691215 0.188774
6 0.916615 -1.518965 -0.321721
1 0.740655 -2.547712 -0.620760
7 2.133483 -1.033973 -0.435541
7 1.521577 1.138704 0.398837
8 3.675986 0.611307 -0.190972
8 -0.672698 1.489845 0.899322

6 -1.506964 -1.245957 0.292376
1 2.892470 -1.615128 -0.794267
8 -2.228121 -0.720402 1.377533
1 -2.056508 0.243532 1.403881
8 -2.125346 -1.020413 -0.962379
1 -2.276459 -0.046618 -1.098999
1 -1.469655 -2.328816 0.424303
8 -2.584600 1.660856 -1.167649
1 -1.956903 1.841403 -0.439218
1 -2.186953 2.065146 -1.953090
rd_5dhmU/rd_5dhmU_a14_aac.xyz
6 -2.476042 0.314555 -0.060279
6 -0.245447 0.720803 0.518817
6 0.107135 -0.688321 0.207643
6 -0.905795 -1.522852 -0.301984
1 -0.725345 -2.553430 -0.591848
7 -2.123130 -1.042101 -0.426745
7 -1.522026 1.138537 0.395042
8 -3.670724 0.601880 -0.206642
8 0.667956 1.499158 0.907575
6 1.515815 -1.235111 0.311940
1 -2.878489 -1.627974 -0.785422
8 2.119495 -1.037851 -0.955130
1 2.233926 -0.065361 -1.129852
8 2.246271 -0.678724 1.375156
1 2.054358 0.282364 1.387931
1 1.484992 -2.314722 0.470494
8 2.401664 1.656300 -1.315328
1 1.930541 1.843097 -0.479139
1 3.324810 1.900443 -1.152110
rd_5dhmU/rd_5dhmU_a25_abj.xyz
6 1.597995 -1.151354 0.014133
6 -0.726539 -1.388390 0.136949
6 -0.879671 0.087906 0.174816
6 0.276253 0.882409 0.070069
1 0.249733 1.966281 0.056070

7 1.446692 0.289844 -0.004647
7 0.514245 -1.933871 0.074534
8 2.773976 -1.548102 -0.034736
8 -1.764085 -2.090172 0.165081
6 -2.262548 0.677854 0.231475
1 2.320197 0.869298 -0.073742
8 -2.168393 2.036725 0.557220
1 -3.057394 2.360762 0.771174
8 -2.821360 0.558136 -1.080342
1 -3.241307 -0.315555 -1.153618
1 -2.880341 0.129028 0.949126
8 3.670928 1.902466 -0.181006
1 3.776444 2.393353 0.648485
1 3.544637 2.582039 -0.861089
rd_5dhmU/rd_5dhmU_a25_aav.xyz
6 2.504342 0.297677 0.093773
6 0.316600 0.890429 -0.501886
6 -0.145168 -0.489631 -0.209279
6 0.778214 -1.398065 0.340317
1 0.510132 -2.404123 0.643025
7 2.029292 -1.016967 0.478359
7 1.629021 1.198006 -0.368425
8 3.721938 0.475153 0.242050
8 -0.520457 1.742910 -0.889291
6 -1.588070 -0.881676 -0.375664
1 2.727136 -1.658501 0.856332
8 -1.654268 -2.247639 -0.688939
1 -2.563028 -2.456521 -0.956963
8 -2.176647 -0.652465 0.899351
1 -2.530320 0.277487 0.911857
1 -2.075460 -0.264636 -1.135092
8 -2.880977 1.955137 0.609370
1 -2.705825 2.486113 1.400465
1 -2.083053 2.055727 0.046926
rd_5dhmU/rd_5dhmU_a11_aaj.xyz
6 2.730369 -0.360579 -0.166939

6 0.866231 1.055427 -0.172363
6 0.006309 -0.051316 0.324332
6 0.607068 -1.298642 0.570654
1 0.054644 -2.155497 0.943876
7 1.894746 -1.435289 0.341138
7 2.183674 0.837374 -0.398008
8 3.918084 -0.667858 -0.340694
8 0.335551 2.175070 -0.376586
6 -1.485601 0.115342 0.480424
1 2.359644 -2.328361 0.510797
8 -1.816413 1.324510 1.125465
1 -1.339024 2.033063 0.647828
8 -2.010912 0.036492 -0.827285
1 -2.893369 -0.417389 -0.775520
1 -1.889785 -0.686369 1.106040
8 -4.429648 -1.131392 -0.583479
1 -4.366046 -2.094149 -0.490723
1 -4.794555 -0.821859 0.259783
rd_5dhmU/rd_5dhmU_a14_abt.xyz
6 2.227033 -0.424663 0.026795
6 0.065545 0.455464 0.048657
6 -0.510554 -0.907097 -0.069232
6 0.374060 -1.998247 -0.145753
1 0.038987 -3.026726 -0.236311
7 1.667068 -1.759358 -0.103337
7 1.401957 0.628968 0.089967
8 3.462574 -0.383598 0.067882
8 -0.742498 1.418954 0.110043
6 -2.008216 -1.110282 -0.060335
1 2.338109 -2.527371 -0.158789
8 -2.517310 -0.762389 1.211992
1 -2.314458 0.173760 1.387471
8 -2.617698 -0.400552 -1.114295
1 -2.374556 0.539906 -1.025901
1 -2.250832 -2.162823 -0.209369
8 0.074580 4.074997 -0.073083

1 -0.125137 3.115812 0.003637
1 1.040319 4.129837 -0.088270
rd_5dhmU/rd_5dhmU_a11_abt.xyz
6 2.479453 0.360103 -0.088874
6 0.246196 0.708425 0.522340
6 -0.073578 -0.707500 0.199722
6 0.974846 -1.545379 -0.224958
1 0.830708 -2.597378 -0.451604
7 2.179194 -1.036775 -0.351088
7 1.504393 1.167932 0.342899
8 3.657002 0.682392 -0.290976
8 -0.674434 1.452779 0.946213
6 -1.481778 -1.257556 0.242642
1 2.960389 -1.620929 -0.654281
8 -2.224046 -0.753836 1.321774
1 -2.069279 0.214381 1.347681
8 -2.051761 -0.927265 -1.019481
1 -2.755896 -1.565489 -1.219266
1 -1.449835 -2.344322 0.361646
8 -2.653360 1.876176 -1.155948
1 -2.029644 2.100643 -0.445199
1 -2.569593 0.904480 -1.199539
rd_5dhmU/rd_5dhmU_a14_abi.xyz
6 2.833043 0.174012 -0.023499
6 0.631902 0.957727 0.055234
6 0.116338 -0.424750 -0.122574
6 1.048043 -1.472739 -0.234539
1 0.757211 -2.511393 -0.357567
7 2.329002 -1.180409 -0.190478
7 1.963706 1.184591 0.088303
8 4.067063 0.267417 -0.002259
8 -0.208176 1.885537 0.172831
6 -1.364353 -0.723137 -0.074494
1 3.030464 -1.917675 -0.275930
8 -1.752155 -0.764464 1.289227
1 -1.611074 0.109678 1.693354

8 -2.113095 0.198196 -0.829042
1 -1.791611 1.093567 -0.587938
1 -1.571047 -1.716597 -0.474563
8 -4.886641 0.068917 -0.094187
1 -3.944880 0.110885 -0.352877
1 -5.363818 0.490769 -0.822585
rd_5dhmU/rd_5dhmU_a14_abr.xyz
6 -1.733002 -1.043366 -0.004437
6 -0.029082 0.573222 -0.027472
6 1.001503 -0.497631 -0.046032
6 0.572230 -1.836848 -0.049922
1 1.259045 -2.677673 -0.065722
7 -0.720194 -2.083953 -0.034306
7 -1.341246 0.237793 -0.006253
8 -2.900583 -1.446676 0.019787
8 0.356555 1.765131 -0.031742
6 2.471519 -0.148598 -0.027978
1 -1.061855 -3.046650 -0.038411
8 2.801205 0.680835 -1.118400
1 2.231672 1.472002 -1.076965
8 2.800785 0.422532 1.222824
1 2.295538 1.247158 1.337132
1 3.077650 -1.049872 -0.123185
8 -3.272004 2.321690 0.054779
1 -2.592236 1.606226 0.031355
1 -2.766400 3.143158 -0.023233
rd_5dhmU/rd_5dhmU_a25_abk.xyz
6 -2.509876 -0.301408 0.093614
6 -0.320522 -0.901459 -0.489974
6 0.140222 0.482011 -0.211320
6 -0.783599 1.395631 0.328850
1 -0.515279 2.404009 0.623661
7 -2.035045 1.016423 0.468082
7 -1.634029 -1.206482 -0.357865
8 -3.728139 -0.476478 0.239987
8 0.517274 -1.758214 -0.865253

6 1.583705 0.872394 -0.373985
1 -2.733054 1.661135 0.840214
8 1.652668 2.230477 -0.719132
1 2.562273 2.431273 -0.990488
8 2.157953 0.673392 0.912500
1 2.508137 -0.257132 0.950801
1 2.079548 0.237853 -1.113088
8 2.821754 -1.954241 0.743937
1 2.104186 -2.053131 0.082696
1 3.646036 -2.096088 0.255262
rd_5dhmU/rd_5dhmU_a14_abx.xyz
6 1.616890 0.640312 -0.020081
6 -0.256061 -0.751919 -0.032519
6 -1.154433 0.431531 -0.035101
6 -0.569910 1.710876 -0.036373
1 -1.150562 2.627888 -0.040155
7 0.742607 1.798406 -0.032251
7 1.087182 -0.583813 -0.023943
8 2.827818 0.914405 -0.006530
8 -0.790337 -1.886873 -0.034839
6 -2.654686 0.252583 -0.002574
1 1.200275 2.712021 -0.033027
8 -3.092068 -0.489489 -1.118877
1 -2.623315 -1.344806 -1.116190
8 -3.026771 -0.329720 1.230578
1 -2.583980 -1.193334 1.313504
1 -3.156971 1.219365 -0.051237
8 4.744129 -1.172744 0.060384
1 4.273036 -2.015822 -0.000456
1 4.040031 -0.492399 0.035081
rd_5dhmU/rd_5dhmU_a14_abf.xyz
6 2.826540 0.152127 0.017602
6 0.634291 0.963905 0.028816
6 0.106377 -0.413574 -0.150292
6 1.027575 -1.473488 -0.236936
1 0.726621 -2.508811 -0.363624

7 2.310469 -1.196441 -0.163471
7 1.967443 1.174133 0.099586
8 4.060304 0.229018 0.079417
8 -0.196753 1.903229 0.114088
6 -1.378336 -0.694287 -0.129865
1 3.004925 -1.942266 -0.230051
8 -1.793670 -0.718470 1.226499
1 -1.647371 0.156932 1.625817
8 -2.101947 0.228232 -0.907255
1 -1.773750 1.121921 -0.668877
1 -1.588678 -1.688994 -0.524710
8 -4.867242 0.091281 -0.133206
1 -3.926744 0.137417 -0.394976
1 -4.844619 -0.194467 0.791319
rd_5dhmU/rd_5dhmU_a14_aap.xyz
6 2.827992 0.136799 -0.076558
6 0.642528 0.950735 0.053418
6 0.108109 -0.434769 -0.004601
6 1.021064 -1.501503 -0.079082
1 0.715175 -2.542390 -0.113131
7 2.305542 -1.220445 -0.112389
7 1.975787 1.164850 0.005073
8 4.062165 0.214911 -0.129851
8 -0.188398 1.888462 0.145643
6 -1.380726 -0.676145 0.071154
1 2.997958 -1.968914 -0.171322
8 -1.825449 -0.371505 1.378268
1 -1.635355 0.565613 1.563363
8 -2.060568 0.058405 -0.923639
1 -1.793391 0.994530 -0.832790
1 -1.614210 -1.727855 -0.096636
8 -4.873916 -0.011927 -0.394678
1 -4.953242 -0.017019 0.569914
1 -3.910716 0.007735 -0.560119
rd_5dhmU/rd_5dhmU_a25_abf.xyz
6 -1.574399 -0.607149 -0.047939

6 0.120739 0.987014 0.173523
6 1.146561 -0.086690 0.148394
6 0.724886 -1.411430 -0.070042
1 1.409058 -2.248116 -0.155432
7 -0.565940 -1.644986 -0.161305
7 -1.197179 0.660854 0.104987
8 -2.743310 -1.025752 -0.114757
8 0.509088 2.171900 0.262870
6 2.602701 0.279330 0.237450
1 -0.910142 -2.595720 -0.304382
8 3.337388 -0.853134 0.604072
1 4.231890 -0.572187 0.852822
8 2.986962 0.670035 -1.085014
1 2.768172 1.610163 -1.204923
1 2.751612 1.110269 0.932022
8 -4.985489 0.648304 0.035135
1 -4.144302 0.147354 -0.021199
1 -4.724495 1.579880 0.028551
rd_5dhmU/rd_5dhmU_a15_aal.xyz
6 -2.512742 0.406812 -0.105954
6 -0.258556 0.747200 0.428021
6 0.026690 -0.693128 0.191332
6 -1.047458 -1.534645 -0.154867
1 -0.928893 -2.601368 -0.318731
7 -2.244431 -1.010116 -0.286520
7 -1.511913 1.220745 0.248737
8 -3.689082 0.738594 -0.299880
8 0.684226 1.495832 0.789543
6 1.423433 -1.268690 0.253582
1 -3.043684 -1.595884 -0.534783
8 2.005548 -0.984987 -1.013778
1 2.665295 -1.668945 -1.213435
8 2.169377 -0.747105 1.322444
1 2.059169 0.226300 1.301997
1 1.371752 -2.350089 0.406044
8 2.989772 1.724870 -0.994459

1 2.283846 1.982406 -0.377754
1 2.819988 0.770253 -1.102499
rd_5fC/rd_5fC_087.xyz
6 -1.229679 0.750374 -0.002909
6 0.546198 -0.732944 -0.001785
6 1.481328 0.393952 0.001001
6 0.901390 1.689234 0.001501
1 1.554634 2.559176 0.003458
7 -0.392225 1.901410 -0.000475
7 -0.766735 -0.527849 -0.003668
8 -2.458921 0.980021 -0.004412
7 1.016817 -1.978441 -0.002495
1 0.365313 -2.754747 -0.004409
1 2.016439 -2.150676 -0.000988
6 2.945977 0.284375 0.003048
1 3.490640 1.241440 0.004999
8 3.553692 -0.786516 0.002637
8 -4.287139 -1.169151 -0.000082
1 -3.622635 -0.447200 -0.000200
1 -5.141725 -0.718608 0.053331
rd_5fC/rd_5fC_073.xyz
6 2.623906 -0.186697 0.057155
6 0.600961 0.940823 -0.037310
6 -0.102226 -0.343244 -0.071462
6 0.709450 -1.505350 -0.091461
1 0.230029 -2.480324 -0.149309
7 2.019542 -1.472431 -0.048990
7 1.925535 0.985083 0.047169
8 3.867126 -0.180440 0.159566
7 -0.094710 2.076739 -0.086532
1 0.397638 2.962007 -0.062175
1 -1.107434 2.059074 -0.122467
6 -1.556471 -0.516722 -0.073625
1 -1.910989 -1.557197 -0.115213
8 -2.357861 0.420053 -0.023068
8 -5.076865 -0.511233 0.049718

1 -5.507922 -0.112437 0.818754
1 -4.166819 -0.156745 0.059373
rd_5fC/rd_5fC_039.xyz
6 2.626358 -0.173101 0.065392
6 0.594242 0.936592 -0.041553
6 -0.097249 -0.353706 -0.082211
6 0.724745 -1.508704 -0.098439
1 0.254536 -2.487913 -0.161021
7 2.034140 -1.464136 -0.046585
7 1.917841 0.992551 0.050973
8 3.868671 -0.156114 0.177286
7 -0.111167 2.066373 -0.093070
1 0.373184 2.955844 -0.062832
1 -1.123522 2.039866 -0.134340
6 -1.550057 -0.538985 -0.091774
1 -1.896576 -1.581987 -0.135447
8 -2.358593 0.391751 -0.044286
8 -5.083619 -0.503097 0.090922
1 -5.527187 0.046876 0.752195
1 -4.166044 -0.169114 0.062371
rd_5fC/rd_5fC_085.xyz
6 -1.225348 0.477616 -0.005267
6 1.055409 0.866904 -0.001116
6 1.297396 -0.577721 -0.000363
6 0.145927 -1.405335 -0.004185
1 0.274555 -2.485809 -0.004802
7 -1.079174 -0.938970 -0.007237
7 -0.182118 1.349817 -0.003052
8 -2.400013 0.904731 -0.005778
7 2.088742 1.706766 0.000189
1 1.915048 2.705217 0.000214
1 3.039488 1.353473 0.003034
6 2.617259 -1.221220 0.004503
1 2.604534 -2.322113 0.004271
8 3.681849 -0.602380 0.008526
8 -4.738668 -0.648977 -0.013668

1 -3.895546 -0.147701 -0.003888
1 -5.419439 0.005183 0.197807
rd_5fC/rd_5fC_003.xyz
6 -0.930206 -1.797352 -0.009184
6 0.316044 0.158661 -0.039213
6 -0.922694 0.940597 0.005552
6 -2.131034 0.201293 0.047922
1 -3.075298 0.740314 0.085922
7 -2.179356 -1.109154 0.045781
7 0.277258 -1.171586 -0.051719
8 -1.000803 -3.043849 -0.016458
7 1.490600 0.781907 -0.065083
1 2.362192 0.239001 -0.068545
1 1.517001 1.795420 -0.054388
6 -1.009533 2.405649 0.014966
1 -2.031345 2.815149 0.048910
8 -0.030473 3.153055 -0.010984
8 4.010854 -0.673735 -0.032626
1 3.816912 -1.614929 0.093025
1 4.518931 -0.419966 0.752498
rd_5fC/rd_5fC_023.xyz
6 1.234698 0.769348 0.004202
6 -0.529878 -0.727852 0.003529
6 -1.473546 0.391980 -0.001343
6 -0.903694 1.691453 -0.000587
1 -1.563498 2.556426 -0.002662
7 0.388269 1.913671 0.003161
7 0.781544 -0.512727 0.005744
8 2.461836 1.009411 0.003590
7 -0.991823 -1.976636 0.005653
1 -0.335537 -2.748829 0.008667
1 -1.990285 -2.155681 0.002986
6 -2.937309 0.272152 -0.005666
1 -3.488378 1.225504 -0.008962
8 -3.537536 -0.802935 -0.005609
8 4.357038 -1.070808 -0.015121

1 3.644371 -0.396335 -0.006344
1 3.895060 -1.919074 0.040720
rd_5fC/rd_5fC_020.xyz
6 -0.899557 1.811060 -0.011913
6 0.315425 -0.164426 -0.048089
6 -0.935283 -0.926658 0.006344
6 -2.131196 -0.168195 0.059833
1 -3.083521 -0.692107 0.107729
7 -2.158392 1.142827 0.058361
7 0.297688 1.166361 -0.062484
8 -0.951251 3.058443 -0.025498
7 1.479560 -0.806788 -0.080794
1 2.359920 -0.278078 -0.082378
1 1.489657 -1.820545 -0.069409
6 -1.045824 -2.390094 0.017590
1 -2.073994 -2.782324 0.061977
8 -0.080059 -3.154192 -0.017815
8 4.029655 0.579608 0.025721
1 3.962456 1.454028 -0.386491
1 4.205325 0.761237 0.961136
rd_5fC/rd_5fC_016.xyz
6 -1.228024 0.514402 -0.000010
6 1.058453 0.866982 -0.000002
6 1.277541 -0.581400 0.000002
6 0.112851 -1.390816 -0.000016
1 0.223798 -2.473331 -0.000018
7 -1.104326 -0.904348 -0.000038
7 -0.171171 1.369797 0.000005
8 -2.395994 0.959489 0.000003
7 2.105311 1.689844 -0.000007
1 1.948232 2.691105 -0.000008
1 3.049892 1.320359 -0.000009
6 2.587523 -1.245001 0.000015
1 2.558579 -2.345545 0.000024
8 3.661536 -0.642638 0.000017
8 -4.763660 -0.529037 0.000026

1 -4.568519 -1.476575 -0.000024
1 -3.885809 -0.090581 0.000014
rd_5fC/rd_5fC_011.xyz
6 2.125933 0.007041 0.003998
6 0.047120 1.024106 -0.126189
6 -0.544763 -0.108437 0.587359
6 0.345206 -1.145708 0.967591
1 -0.051038 -2.008184 1.499703
7 1.629548 -1.127692 0.708958
7 1.347443 1.053747 -0.394763
8 3.348955 -0.000239 -0.242812
7 -0.729451 2.035009 -0.516177
1 -0.316900 2.816398 -1.012033
1 -1.721657 2.028445 -0.306481
6 -1.970553 -0.257753 0.907335
1 -2.244532 -1.205805 1.395744
8 -2.820719 0.601150 0.673308
8 -1.681468 -1.740559 -1.778479
1 -1.068942 -2.343705 -1.332349
1 -1.121508 -1.012908 -2.087408
rd_5fC/rd_5fC_032.xyz
6 2.636700 -0.128376 -0.062309
6 0.573600 0.921929 0.045908
6 -0.080393 -0.388077 0.082647
6 0.774612 -1.518989 0.101772
1 0.333620 -2.511768 0.163748
7 2.082304 -1.436026 0.054394
7 1.895227 1.016473 -0.043287
8 3.877146 -0.076171 -0.183665
7 -0.164116 2.030774 0.095945
1 0.294763 2.933615 0.065020
1 -1.175561 1.975539 0.131918
6 -1.527635 -0.613429 0.082208
1 -1.846740 -1.665187 0.122453
8 -2.359450 0.295893 0.028642
8 -5.116616 -0.448714 -0.167330

1 -4.173854 -0.202678 -0.093045
1 -5.596075 0.385512 -0.061991
rd_5fC/rd_5fC_090.xyz
6 2.161868 0.140787 0.034650
6 -0.002568 0.515794 -0.706059
6 -0.404564 -0.784347 -0.165700
6 0.615016 -1.549824 0.455519
1 0.363827 -2.526750 0.863417
7 1.856858 -1.144426 0.570091
7 1.249051 0.942357 -0.584791
8 3.345342 0.514742 0.160974
7 -0.897295 1.280062 -1.332024
1 -0.611976 2.186707 -1.683057
1 -1.867109 0.990713 -1.391704
6 -1.762973 -1.338354 -0.208893
1 -1.878504 -2.341117 0.230861
8 -2.729162 -0.746590 -0.692472
8 -2.052158 1.420340 2.028293
1 -2.454709 1.047037 1.230497
1 -1.164667 1.685185 1.745583
rd_5fC/rd_5fC_098.xyz
6 2.178901 -0.259925 0.050796
6 0.295084 1.076515 0.237655
6 -0.473445 0.089012 -0.521144
6 0.228321 -1.072941 -0.930501
1 -0.305793 -1.841388 -1.486044
7 1.496319 -1.280723 -0.671485
7 1.582841 0.882136 0.499470
8 3.391064 -0.466320 0.263419
7 -0.304898 2.185937 0.668178
1 0.231020 2.872978 1.185204
1 -1.285944 2.347333 0.467999
6 -1.891855 0.210799 -0.882881
1 -2.305441 -0.632580 -1.456654
8 -2.593516 1.178899 -0.587806
8 -2.430719 -2.006547 1.291265

1 -2.844742 -2.757055 1.741626
1 -1.865600 -2.409745 0.616143
rd_5fC/rd_5fC_051.xyz
6 -1.416477 0.177774 -0.803198
6 0.645428 -0.785465 -0.362626
6 1.166798 0.525733 0.027202
6 0.265352 1.614943 -0.072280
1 0.613443 2.611466 0.191661
7 -0.977848 1.491820 -0.470440
7 -0.614663 -0.924184 -0.757725
8 -2.609064 0.076482 -1.155198
7 1.442850 -1.852787 -0.321229
1 1.076637 -2.755534 -0.600176
1 2.405700 -1.764191 -0.016080
6 2.528960 0.794532 0.501745
1 2.743769 1.847644 0.742093
8 3.393119 -0.073023 0.634836
8 -3.125472 -0.513239 2.012442
1 -3.164570 0.334223 1.545601
1 -3.036376 -1.164415 1.300951
rd_5fC/rd_5fC_047.xyz
6 1.268593 1.226045 0.016167
6 -0.212420 -0.569917 0.047191
6 -1.335298 0.367590 0.000232
6 -1.002977 1.745859 -0.002889
1 -1.806727 2.479076 -0.016403
7 0.228736 2.197380 0.013046
7 1.037802 -0.119337 0.045545
8 2.434033 1.663425 -0.006240
7 -0.438433 -1.880449 0.092256
1 0.346799 -2.521484 0.128549
1 -1.388688 -2.235003 0.083803
6 -2.752383 -0.011473 -0.047462
1 -3.465442 0.827095 -0.078533
8 -3.147591 -1.177813 -0.055921
8 3.154167 -2.025975 -0.119090

1 2.452351 -1.336179 -0.053643
1 2.747009 -2.822371 0.250862
rd_5fC/rd_5fC_079.xyz
6 2.331420 -0.374863 -0.167792
6 0.111752 -0.586658 0.469728
6 -0.106964 0.825939 0.150399
6 1.024927 1.548403 -0.307502
1 0.914775 2.605407 -0.540611
7 2.211274 1.012868 -0.465357
7 1.304404 -1.143590 0.293923
8 3.459482 -0.874102 -0.354527
7 -0.890037 -1.325434 0.946456
1 -0.725896 -2.303342 1.155201
1 -1.817258 -0.933195 1.058417
6 -1.378324 1.546382 0.276328
1 -1.332869 2.623333 0.052323
8 -2.441712 1.018005 0.607000
8 -3.634131 -1.068976 -1.249710
1 -3.322164 -0.341025 -0.688762
1 -3.062057 -1.812722 -1.010784
rd_5fC/rd_5fC_095.xyz
6 2.123184 0.547489 0.023980
6 0.527019 -1.118138 0.246593
6 -0.444542 -0.270441 -0.447155
6 0.020512 0.999668 -0.872810
1 -0.665314 1.662354 -1.396519
7 1.241872 1.429866 -0.665599
7 1.768327 -0.695785 0.457632
8 3.277123 0.979314 0.220303
7 0.160227 -2.326476 0.672587
1 0.833969 -2.909647 1.154732
1 -0.787094 -2.656018 0.523354
6 -1.839491 -0.630938 -0.729037
1 -2.423321 0.130217 -1.269431
8 -2.350535 -1.701873 -0.398574
8 -2.949945 2.394601 0.890653

1 -3.172152 2.468182 1.830180
1 -2.112311 1.909497 0.876857
rd_5fC/rd_5fC_078.xyz
6 1.970327 0.277654 -0.192733
6 -0.198619 0.965957 0.246816
6 -0.704782 -0.100206 -0.618456
6 0.269761 -0.936070 -1.221562
1 -0.061226 -1.747479 -1.866474
7 1.559766 -0.785112 -1.048498
7 1.106646 1.131930 0.427781
8 3.201686 0.398563 -0.031258
7 -1.057403 1.778942 0.860884
1 -0.703976 2.510041 1.466438
1 -2.056107 1.659054 0.732780
6 -2.120322 -0.377301 -0.888502
1 -2.313792 -1.225539 -1.563666
8 -3.050398 0.272017 -0.408282
8 -0.339828 -2.070710 1.825169
1 0.476037 -1.554136 1.747392
1 -0.393876 -2.301431 2.763947
rd_5fC/rd_5fC_076.xyz
6 -2.199412 0.195663 0.097765
6 -0.039226 0.554089 -0.662049
6 0.337139 -0.783328 -0.199622
6 -0.700352 -1.567088 0.366405
1 -0.474922 -2.578848 0.697227
7 -1.931975 -1.141107 0.511157
7 -1.274863 1.008513 -0.489074
8 -3.361049 0.603642 0.299131
7 0.865328 1.325066 -1.265067
1 0.601782 2.258116 -1.559489
1 1.825544 1.013240 -1.357845
6 1.683652 -1.361877 -0.273045
1 1.775882 -2.391761 0.105126
8 2.664656 -0.761974 -0.715082
8 2.436489 1.275770 1.911479

1 1.474655 1.260951 1.800857
1 2.776053 0.926749 1.074067
rd_5fC/rd_5fC_050.xyz
6 2.065957 -0.637106 0.003928
6 0.585359 1.126447 -0.261859
6 -0.453021 0.348319 0.415974
6 -0.079578 -0.946649 0.857380
1 -0.817421 -1.560260 1.371016
7 1.115197 -1.456026 0.678934
7 1.799385 0.621417 -0.448804
8 3.195454 -1.140986 -0.159977
7 0.310153 2.356034 -0.697122
1 1.032846 2.889446 -1.166060
1 -0.614153 2.751968 -0.567262
6 -1.826485 0.801173 0.666605
1 -2.477843 0.071711 1.174580
8 -2.253826 1.910118 0.343470
8 -2.828090 -2.598135 -0.949336
1 -2.599068 -1.879305 -0.342870
1 -3.759191 -2.444618 -1.165902
rd_5fC/rd_5fC_002.xyz
6 1.957668 -0.325196 -0.172763
6 -0.217560 -0.887922 0.395469
6 -0.715503 0.003671 -0.653362
6 0.266448 0.688467 -1.413559
1 -0.056465 1.361304 -2.205294
7 1.555929 0.551216 -1.222181
7 1.086108 -1.029907 0.603875
8 3.188150 -0.426445 0.007743
7 -1.081842 -1.564907 1.150844
1 -0.731630 -2.176037 1.879022
1 -2.079975 -1.462662 1.004779
6 -2.128956 0.245771 -0.964747
1 -2.315868 0.960794 -1.781198
8 -3.064228 -0.297600 -0.375479
8 -0.280896 2.268244 1.699895

1 0.007184 3.034818 2.216513
1 0.538605 1.884633 1.354904
rd_5fU/rd_5fUrd_abo.xyz
6 1.010493 1.359342 -0.029102
6 -0.044816 -0.841014 -0.053193
6 -1.333000 -0.133386 0.001585
6 -1.325230 1.287264 0.042427
1 -2.275747 1.811368 0.086686
7 -0.236208 2.017976 0.028399
7 1.056883 -0.020051 -0.084037
8 2.047683 2.022010 -0.031687
8 0.054332 -2.072675 -0.068615
6 -2.587743 -0.897375 0.015050
1 -2.496264 -1.991868 -0.005662
8 -3.686188 -0.343752 0.046658
1 1.990529 -0.474928 -0.083282
8 3.510342 -1.428228 -0.005020
1 3.230853 -2.306580 0.295965
1 4.078333 -1.091304 0.704460
rd_5fU/rd_5fUda_aai.xyz
6 1.224510 1.258136 -0.001138
6 -0.274323 -0.678871 -0.034124
6 -1.388617 0.290164 -0.010009
6 -1.071425 1.674382 -0.011585
1 -1.887661 2.393927 -0.012729
7 0.146557 2.165573 -0.008425
7 0.975196 -0.098123 -0.020552
8 2.379279 1.685936 0.020222
8 -0.411035 -1.903458 -0.065856
6 -2.808597 -0.093879 0.020751
1 -3.514861 0.752572 0.007686
8 -3.215889 -1.251196 0.063393
1 1.795617 -0.735546 -0.022262
8 3.156190 -1.900894 -0.053849
1 2.741272 -2.777060 -0.039663
1 3.655723 -1.848739 0.775155

rd_5fU/rd_5fUrd_aaf.xyz
6 -2.316479 -0.510895 0.000001
6 0.127559 -0.722329 -0.000002
6 0.168263 0.747339 -0.000003
6 -1.062898 1.459763 -0.000003
1 -1.030876 2.545564 -0.000004
7 -2.246323 0.894671 -0.000001
7 -1.143101 -1.249200 0.000001
8 -3.406709 -1.075996 0.000002
8 1.127873 -1.445591 -0.000004
6 1.457285 1.456260 -0.000003
1 2.364967 0.840267 -0.000001
8 1.516250 2.684725 -0.000004
1 -1.233727 -2.263820 0.000002
8 4.022847 -1.360353 0.000010
1 3.048822 -1.342812 0.000006
1 4.252313 -2.300608 0.000014
rd_5fU/rd_5fUda_abm.xyz
6 1.234030 1.190476 0.001659
6 -0.318185 -0.703118 -0.030585
6 -1.404830 0.297062 -0.007996
6 -1.048968 1.671761 -0.004215
1 -1.844680 2.413837 -0.002304
7 0.182374 2.128723 0.000172
7 0.946826 -0.158170 -0.019549
8 2.400858 1.584783 0.019562
8 -0.489732 -1.923323 -0.058312
6 -2.835465 -0.047065 0.013480
1 -3.517798 0.818873 -0.000876
8 -3.275294 -1.192722 0.048647
1 1.755198 -0.811788 -0.025351
8 3.213020 -1.835429 -0.067866
1 3.196165 -2.486158 0.650662
1 3.956406 -1.249793 0.143201
rd_5fU/rd_5fUrd_abw.xyz
6 -1.170351 -1.130838 -0.081566

6 1.279011 -1.090392 0.064469
6 1.177735 0.374323 -0.018692
6 -0.112411 0.963062 -0.130662
1 -0.191986 2.045153 -0.189845
7 -1.226795 0.274957 -0.164156
7 0.066139 -1.744782 0.022131
8 -2.205045 -1.789958 -0.101550
8 2.347102 -1.697423 0.162546
6 2.396682 1.195037 0.014354
1 3.349981 0.656710 0.100522
8 2.350772 2.422154 -0.051964
1 0.075521 -2.761805 0.079181
8 -3.727391 1.726765 0.100569
1 -3.752543 1.986760 1.032385
1 -2.903882 1.202502 0.007697
rd_5fU/rd_5fUrd_aaz.xyz
6 -1.130421 -0.865370 -0.051356
6 0.415581 1.035834 -0.058544
6 1.498143 0.043713 -0.004457
6 1.156142 -1.336378 -0.004906
1 1.958007 -2.069034 0.016098
7 -0.073170 -1.792568 -0.029647
7 -0.849980 0.488128 -0.080891
8 -2.294589 -1.266731 -0.046949
8 0.600443 2.254115 -0.083141
6 2.897891 0.490238 0.049269
1 3.069588 1.574905 0.029543
8 3.830190 -0.309000 0.116121
1 -1.640676 1.130631 -0.111653
8 -4.418294 0.676775 0.106106
1 -3.726236 -0.010645 0.063583
1 -3.964643 1.455737 0.459053
rd_5fU/rd_5fUrd_abi.xyz
6 -1.167765 -1.140654 -0.080732
6 1.281329 -1.088529 0.066086
6 1.173373 0.375494 -0.020569

6 -0.119454 0.958035 -0.132491
1 -0.204065 2.039713 -0.192824
7 -1.230699 0.264785 -0.163716
7 0.071552 -1.748660 0.024410
8 -2.199372 -1.804545 -0.101595
8 2.352158 -1.690350 0.166455
6 2.388377 1.202045 0.011195
1 3.344192 0.668509 0.099319
8 2.336652 2.428776 -0.058174
1 0.085783 -2.765534 0.082922
8 -3.743280 1.688747 0.141835
1 -3.509272 2.434650 0.712297
1 -2.907037 1.190424 0.024347
rd_5fU/rd_5fUrd_abd.xyz
6 -0.498194 1.863210 -0.012346
6 -0.283641 -0.576094 -0.099687
6 1.167510 -0.366061 -0.015615
6 1.657966 0.965921 0.063766
1 2.731480 1.120022 0.124807
7 0.896453 2.033928 0.068416
7 -1.023372 0.581994 -0.092658
8 -1.240940 2.840834 -0.013125
8 -0.807571 -1.693451 -0.169105
6 2.074566 -1.522917 -0.004041
1 1.608216 -2.514926 -0.073630
8 3.293972 -1.387895 0.081081
1 -2.037596 0.497188 -0.146839
8 -3.690605 -1.766565 0.062125
1 -3.841831 -1.738350 1.017746
1 -2.719924 -1.803130 -0.032654
rd_5fU/rd_5fUda_abc.xyz
6 -1.218421 0.848767 0.001020
6 0.586739 -0.821456 0.000410
6 1.520328 0.321072 0.000187
6 0.975697 1.633786 0.001617
1 1.660894 2.478695 0.002220

7 -0.306742 1.916820 0.002179
7 -0.744944 -0.447702 0.001102
8 -2.428704 1.079051 -0.000009
8 0.916849 -2.005518 0.000071
6 2.986328 0.178630 -0.001199
1 3.539469 1.131997 -0.000468
8 3.579175 -0.895467 -0.003044
1 -1.438280 -1.194724 0.000859
8 -4.322493 -1.105507 -0.013568
1 -3.697299 -0.355951 -0.006300
1 -3.765630 -1.889107 0.100924
rd_5fU/rd_5fUrd_aba.xyz
6 -2.291013 -0.248892 0.145605
6 0.039344 -0.770259 -0.411283
6 0.321364 0.654212 -0.183211
6 -0.748246 1.503511 0.211251
1 -0.536307 2.554666 0.385285
7 -1.988950 1.107638 0.368319
7 -1.279556 -1.116646 -0.241311
8 -3.438556 -0.659649 0.293807
8 0.896878 -1.601556 -0.727440
6 1.680933 1.178990 -0.386459
1 2.420824 0.483706 -0.804149
8 1.973097 2.340340 -0.107294
1 -1.533904 -2.093755 -0.376468
8 3.375456 -1.634147 0.828646
1 3.468294 -0.708990 1.098206
1 2.591357 -1.637840 0.250899
rd_5fU/rd_5fUrd_aal.xyz
6 0.518671 1.852359 -0.012016
6 0.276480 -0.586055 -0.066562
6 -1.173400 -0.356811 -0.012558
6 -1.648405 0.981763 0.048329
1 -2.720769 1.148683 0.093937
7 -0.874628 2.040984 0.051107
7 1.029212 0.563985 -0.069813

8 1.273329 2.820766 -0.016968
8 0.788333 -1.710278 -0.103734
6 -2.096156 -1.501471 -0.012257
1 -1.642931 -2.499798 -0.077984
8 -3.314487 -1.349882 0.058767
1 2.042831 0.468586 -0.111481
8 3.674185 -1.867860 0.082829
1 3.904123 -0.974236 0.376048
1 2.700631 -1.858699 0.013653
rd_5fU/rd_5fUrd_abj.xyz
6 2.291414 -0.329435 0.128066
6 -0.071610 -0.742172 -0.380081
6 -0.282467 0.694361 -0.151299
6 0.831821 1.491356 0.229237
1 0.671289 2.550184 0.411305
7 2.055271 1.038090 0.363196
7 1.231512 -1.150573 -0.228843
8 3.423735 -0.790448 0.241878
8 -0.971559 -1.533337 -0.681126
6 -1.616393 1.284972 -0.344148
1 -2.395170 0.625576 -0.749060
8 -1.846145 2.462320 -0.073486
1 1.437809 -2.137785 -0.372859
8 -3.539929 -1.501843 0.681014
1 -2.682545 -1.507872 0.218261
1 -3.484260 -2.230750 1.315000
rd_5fU/rd_5fUda_abt.xyz
6 -2.625272 -0.263822 0.068325
6 -0.525377 1.017370 -0.039891
6 0.160259 -0.289004 -0.098644
6 -0.632829 -1.467011 -0.102260
1 -0.128764 -2.428855 -0.165742
7 -1.943783 -1.491783 -0.022153
7 -1.899337 0.914060 0.057161
8 -3.851013 -0.244220 0.153765
8 0.034198 2.111688 -0.073765

6 1.621108 -0.442118 -0.130874
1 1.976041 -1.481425 -0.210322
8 2.417403 0.491939 -0.063782
1 -2.428462 1.783461 0.101203
8 5.139093 -0.434398 0.081242
1 5.355875 -0.462775 1.023996
1 4.222373 -0.098909 0.046191
rd_5fU/rd_5fUda_abg.xyz
6 2.625085 -0.271212 0.055788
6 0.531465 1.021797 -0.037124
6 -0.162135 -0.280762 -0.082951
6 0.624179 -1.463262 -0.090166
1 0.113902 -2.422335 -0.144643
7 1.935629 -1.495479 -0.023191
7 1.905746 0.910842 0.047053
8 3.851665 -0.258431 0.129302
8 -0.022207 2.119162 -0.070717
6 -1.623849 -0.427068 -0.099560
1 -1.983368 -1.465196 -0.174474
8 -2.415922 0.510096 -0.025472
1 2.440433 1.777253 0.081195
8 -5.097094 -0.539921 0.140843
1 -5.736403 0.186511 0.133397
1 -4.224181 -0.107979 0.069913
rd_5fU/rd_5fUrd_aao.xyz
6 1.999815 -1.051411 -0.003931
6 1.074595 1.218748 0.070172
6 -0.248343 0.591919 -0.075668
6 -0.322370 -0.824793 -0.173015
1 -1.295793 -1.291498 -0.284912
7 0.719185 -1.621920 -0.141861
7 2.120954 0.324294 0.100898
8 2.995997 -1.769388 0.021441
8 1.250828 2.435808 0.160424
6 -1.442581 1.445152 -0.125685
1 -1.268841 2.528864 -0.106025

8 -2.582375 0.985087 -0.187097
1 3.061688 0.702301 0.197404
8 -3.966262 -1.545361 0.223505
1 -3.481555 -0.714023 0.073857
1 -4.848677 -1.269100 0.508995
rd_5fU/rd_5fUrd_abm.xyz
6 -2.304588 -0.277420 0.132212
6 0.044979 -0.772472 -0.365794
6 0.298977 0.661079 -0.164583
6 -0.790796 1.499897 0.195845
1 -0.598241 2.557236 0.354261
7 -2.027920 1.087395 0.336777
7 -1.271113 -1.136631 -0.212965
8 -3.449006 -0.703011 0.261679
8 0.921358 -1.597046 -0.645740
6 1.650917 1.206547 -0.363729
1 2.402090 0.522730 -0.780468
8 1.924268 2.372679 -0.085828
1 -1.507463 -2.120245 -0.332921
8 3.480059 -1.604324 0.749847
1 3.744027 -0.674225 0.796913
1 2.642447 -1.593014 0.252150
rd_5fU/rd_5fUda_abu.xyz
6 -2.611904 -0.298682 0.050649
6 -0.541114 1.031621 -0.034005
6 0.175706 -0.258464 -0.076536
6 -0.589365 -1.454684 -0.082767
1 -0.061745 -2.404532 -0.132162
7 -1.900123 -1.510740 -0.019381
7 -1.913709 0.895935 0.041768
8 -3.838936 -0.307353 0.116675
8 -0.007003 2.138729 -0.063515
6 1.639569 -0.379268 -0.091069
1 2.018378 -1.411176 -0.160875
8 2.414672 0.572318 -0.021627
1 -2.464122 1.752532 0.071854

8 5.122857 -0.415026 0.150422
1 4.226523 -0.034433 0.077844
1 5.007705 -1.351227 -0.066631
rd_5fU/rd_5fUrd_abv.xyz
6 -1.995973 -1.053422 -0.005416
6 -1.075447 1.218724 0.066544
6 0.249274 0.594089 -0.072830
6 0.326348 -0.822713 -0.166707
1 1.300972 -1.287865 -0.274449
7 -0.713845 -1.621746 -0.138123
7 -2.120130 0.322273 0.095970
8 -2.990869 -1.773235 0.018763
8 -1.254383 2.435657 0.153125
6 1.441868 1.449822 -0.120202
1 1.265284 2.533189 -0.108253
8 2.583226 0.992397 -0.171310
1 -3.061951 0.698549 0.188647
8 3.947917 -1.547843 0.243069
1 4.887558 -1.316319 0.241640
1 3.482414 -0.706055 0.089976
rd_5fU/rd_5fUda_aab.xyz
6 -1.229140 0.864799 -0.047193
6 0.567092 -0.814680 -0.056139
6 1.506616 0.321400 0.010920
6 0.968744 1.636499 0.040206
1 1.657765 2.476886 0.089207
7 -0.311744 1.926660 0.014289
7 -0.762933 -0.434124 -0.072539
8 -2.437513 1.103484 -0.077399
8 0.891195 -1.999727 -0.096183
6 2.971084 0.170728 0.058174
1 3.527891 1.120960 0.101484
8 3.558974 -0.906110 0.054512
1 -1.457502 -1.178552 -0.123821
8 -4.205046 -1.177500 0.072636
1 -3.696980 -1.740122 0.674780

1 -3.675694 -0.360565 0.001771
rd_5fU/rd_5fUda_aat.xyz
6 -2.301058 -0.345141 0.136033
6 0.075498 -0.551556 -0.469719
6 0.192177 0.880152 -0.132322
6 -0.981332 1.576317 0.264107
1 -0.903741 2.639655 0.480432
7 -2.166887 1.029217 0.406870
7 -1.192136 -1.063685 -0.272979
8 -3.393476 -0.894322 0.259950
8 0.990192 -1.250792 -0.900921
6 1.457484 1.628030 -0.194405
1 1.349143 2.723285 -0.145773
8 2.567749 1.110311 -0.279348
1 -1.333882 -2.048280 -0.492084
8 3.336865 -1.398945 0.970604
1 3.018260 -0.628414 0.465680
1 2.716137 -2.101796 0.730059
rd_5hmC/rd_5hmC_045.xyz
6 -2.691800 -0.011727 0.042874
6 -0.546315 0.866024 0.018957
6 0.014080 -0.480664 0.011573
6 -0.930108 -1.531342 -0.045511
1 -0.569469 -2.556492 -0.095697
7 -2.232203 -1.349974 -0.048177
7 -1.859727 1.069591 0.045392
8 -3.931153 0.147521 0.112912
6 1.474809 -0.786403 0.164098
1 1.749236 -0.656594 1.218890
1 1.664793 -1.830981 -0.103994
7 0.277117 1.920429 0.031461
1 -0.113520 2.854829 0.036843
1 1.273000 1.788380 -0.105319
8 2.335658 0.093397 -0.573404
1 2.238452 -0.090634 -1.521240
8 4.983542 -0.227865 0.315779

1 4.070885 -0.088227 -0.012853
1 5.361946 0.659641 0.388395
rd_5hmC/rd_5hmC_078.xyz
6 2.675277 -0.132599 0.030016
6 0.607661 0.905388 -0.095974
6 -0.054055 -0.394429 -0.105471
6 0.802205 -1.515641 -0.026038
1 0.363496 -2.511113 -0.017069
7 2.112287 -1.433430 0.046608
7 1.931416 1.010914 -0.037274
8 3.923356 -0.069490 0.090328
6 -1.534472 -0.576022 -0.263433
1 -1.812049 -0.362740 -1.303125
1 -1.803969 -1.615293 -0.049089
7 -0.132742 2.017660 -0.175837
1 0.326412 2.920237 -0.159925
1 -1.139957 1.967977 -0.076145
8 -2.310533 0.326483 0.540509
1 -2.150920 0.126649 1.477106
8 -5.005064 -0.297503 -0.058981
1 -4.917958 -1.029388 -0.686127
1 -4.083554 -0.058451 0.170452
rd_5hmC/rd_5hmC_003.xyz
6 2.245018 0.020891 -0.174027
6 0.172756 -0.950104 0.190562
6 -0.344809 0.316441 0.693250
6 0.566317 1.396116 0.669881
1 0.227825 2.374088 1.005429
7 1.811075 1.299097 0.257914
7 1.433783 -1.076209 -0.211873
8 3.438876 -0.066236 -0.540399
6 -1.730050 0.497028 1.243874
1 -1.760074 0.075541 2.257802
1 -1.942725 1.569816 1.328885
7 -0.631078 -2.019712 0.164987
1 -0.275918 -2.897881 -0.193618

1 -1.624616 -1.906050 0.336417
8 -2.744421 -0.169664 0.491971
1 -2.689716 0.161652 -0.434972
8 -2.197864 0.807172 -2.048581
1 -1.228286 0.796923 -2.019359
1 -2.431077 0.151272 -2.722948
rd_5hmC/rd_5hmC_035.xyz
6 -2.210756 -0.013034 -0.109755
6 -0.128572 0.969358 0.150854
6 0.404525 -0.277812 0.684926
6 -0.510151 -1.352766 0.747327
1 -0.163498 -2.314067 1.120458
7 -1.768215 -1.269146 0.375240
7 -1.399912 1.079723 -0.222283
8 -3.413308 0.058426 -0.449781
6 1.809528 -0.437345 1.188375
1 1.889940 0.051475 2.168517
1 2.018071 -1.504233 1.334197
7 0.672938 2.037979 0.066271
1 0.307049 2.903853 -0.310731
1 1.671728 1.932138 0.207976
8 2.792687 0.169028 0.347264
1 2.660356 -0.194264 -0.558069
8 1.903726 -0.884166 -2.076419
1 1.214315 -0.233037 -2.281075
1 1.416074 -1.658465 -1.754744
rd_5hmC/rd_5hmC_027.xyz
6 -2.690684 -0.011475 0.046278
6 -0.545028 0.865716 0.015488
6 0.015051 -0.481093 0.008917
6 -0.929571 -1.531691 -0.043005
1 -0.569314 -2.557048 -0.091593
7 -2.231609 -1.350042 -0.042490
7 -1.858332 1.069625 0.044902
8 -3.929872 0.148252 0.118279
6 1.476038 -0.787425 0.157696

1 1.752824 -0.658782 1.212041
1 1.664950 -1.831883 -0.111638
7 0.278617 1.920010 0.024282
1 -0.111910 2.854460 0.029135
1 1.274099 1.787532 -0.115071
8 2.335794 0.092500 -0.580755
1 2.237756 -0.091400 -1.528538
8 4.986131 -0.223371 0.300114
1 4.069292 -0.086395 -0.018043
1 5.270300 0.643120 0.623501
rd_5hmC/rd_5hmC_090.xyz
6 1.391349 1.133295 -0.041170
6 -0.206835 -0.556083 0.082273
6 -1.269135 0.442331 0.104513
6 -0.841771 1.787472 0.008134
1 -1.594025 2.573284 -0.004883
7 0.417087 2.159793 -0.069665
7 1.072112 -0.193965 0.016500
8 2.588945 1.485300 -0.082213
6 -2.719402 0.117114 0.317545
1 -2.873699 -0.090472 1.384685
1 -3.330395 0.989024 0.058111
7 -0.510907 -1.854155 0.159050
1 0.233541 -2.541568 0.152581
1 -1.478320 -2.152607 0.093270
8 -3.164990 -1.049551 -0.380975
1 -3.138638 -0.867745 -1.333474
8 3.170525 -2.090896 -0.014430
1 3.994761 -1.589944 -0.092189
1 2.457659 -1.405277 -0.006127
rd_5hmC/rd_5hmC_028.xyz
6 -2.680742 -0.139492 -0.062286
6 -0.619271 0.906323 0.101835
6 0.046119 -0.391157 0.136104
6 -0.806309 -1.515877 0.057756
1 -0.365049 -2.510160 0.073634

7 -2.114782 -1.438912 -0.041173
7 -1.941572 1.006414 0.008499
8 -3.926843 -0.080223 -0.158554
6 1.523546 -0.572734 0.321814
1 1.775816 -0.386872 1.373374
1 1.799271 -1.606444 0.088168
7 0.113326 2.022775 0.194016
1 -0.350331 2.922615 0.161830
1 1.123485 1.976959 0.126500
8 2.319157 0.349156 -0.438458
1 2.172505 0.183782 -1.383862
8 5.047057 -0.296666 -0.049246
1 5.051122 -1.133333 0.436830
1 4.099347 -0.079006 -0.167145
rd_5hmC/rd_5hmC_029.xyz
6 2.262017 0.156557 -0.159947
6 0.266531 -1.017752 -0.079162
6 -0.342199 0.051353 0.703152
6 0.476879 1.180005 0.929094
1 0.060694 2.029481 1.466461
7 1.724286 1.275771 0.523837
7 1.535472 -0.956454 -0.470164
8 3.466743 0.233819 -0.490420
6 -1.729441 -0.023215 1.273624
1 -1.726844 -0.730369 2.113475
1 -2.010172 0.960594 1.668544
7 -0.460267 -2.100562 -0.380410
1 -0.044367 -2.836795 -0.937782
1 -1.461982 -2.093257 -0.219747
8 -2.699084 -0.499059 0.337989
1 -2.706407 0.135652 -0.416496
8 -2.361732 1.392804 -1.652416
1 -1.535762 1.800566 -1.348644
1 -3.021729 2.100645 -1.600438
rd_5hmC/rd_5hmC_065.xyz
6 -2.676358 -0.135720 -0.042514

6 -0.611529 0.906032 0.098708
6 0.052025 -0.392661 0.119754
6 -0.802453 -1.515712 0.045562
1 -0.362482 -2.510660 0.050276
7 -2.112035 -1.436053 -0.037338
7 -1.934724 1.009077 0.024606
8 -3.923520 -0.074412 -0.121524
6 1.531968 -0.572318 0.284444
1 1.804206 -0.361926 1.326157
1 1.803929 -1.610781 0.068820
7 0.125498 2.020182 0.183508
1 -0.335707 2.921561 0.160556
1 1.133966 1.972708 0.096511
8 2.311598 0.332924 -0.512607
1 2.153088 0.139746 -1.450803
8 5.014107 -0.280064 0.055568
1 4.986351 -1.214077 0.307360
1 4.086078 -0.054324 -0.161526
rd_5hmC/rd_5hmC_053.xyz
6 -1.391413 -1.133766 -0.043254
6 0.206436 0.555996 0.079221
6 1.268811 -0.442285 0.104345
6 0.841777 -1.787541 0.007953
1 1.594188 -2.573224 -0.004006
7 -0.416934 -2.160093 -0.070927
7 -1.072392 0.193646 0.011986
8 -2.588942 -1.486169 -0.082623
6 2.718570 -0.117100 0.320912
1 2.870159 0.090037 1.388540
1 3.330194 -0.988949 0.062778
7 0.510248 1.854189 0.154832
1 -0.234277 2.541509 0.147083
1 1.477797 2.152612 0.090825
8 3.166127 1.049797 -0.375922
1 3.143941 0.867835 -1.328484
8 -3.169803 2.091979 -0.027058

1 -3.995165 1.593322 0.053602
1 -2.457430 1.405973 -0.011814
rd_5hmC/rd_5hmC_092.xyz
6 -2.262122 0.022241 -0.189098
6 -0.173893 0.883065 0.332653
6 0.322697 -0.460631 0.605498
6 -0.600668 -1.506096 0.377016
1 -0.273849 -2.533257 0.524518
7 -1.842759 -1.319532 -0.011163
7 -1.432107 1.096458 -0.041458
8 -3.460723 0.192476 -0.507855
6 1.690556 -0.759434 1.150105
1 1.685829 -0.553570 2.229336
1 1.898109 -1.829188 1.022360
7 0.644830 1.929243 0.494535
1 0.301824 2.862692 0.303504
1 1.633334 1.774193 0.664157
8 2.732103 0.035597 0.585103
1 2.713738 -0.102954 -0.391134
8 2.270974 -0.341699 -2.108996
1 2.846565 0.178053 -2.689970
1 1.406445 0.094988 -2.159230
rd_5hmC/rd_5hmC_080.xyz
6 -1.329053 -0.718733 -0.174846
6 0.487160 0.707661 -0.035792
6 1.381689 -0.431317 0.140409
6 0.763556 -1.701785 0.073654
1 1.384251 -2.591101 0.157195
7 -0.527716 -1.882653 -0.089802
7 -0.826874 0.544840 -0.171089
8 -2.566188 -0.908512 -0.270306
6 2.842683 -0.317879 0.464882
1 2.940916 -0.084234 1.533439
1 3.330037 -1.284644 0.293743
7 0.989992 1.945545 -0.034502
1 0.365552 2.732993 -0.163002

1 1.995188 2.084532 -0.042107
8 3.522825 0.729579 -0.232835
1 3.558394 0.500293 -1.174649
8 -4.357435 1.170851 0.226747
1 -3.697103 0.466933 0.038229
1 -4.994886 0.758087 0.826213
rd_5hmC/rd_5hmC_014.xyz
6 -1.299714 1.000234 -0.029892
6 1.014051 0.912061 0.057795
6 0.971438 -0.546011 0.113780
6 -0.318128 -1.120543 0.028657
1 -0.417995 -2.203861 0.030026
7 -1.421064 -0.412773 -0.055506
7 -0.097687 1.640991 0.004890
8 -2.370109 1.645132 -0.049950
6 2.171948 -1.417215 0.345416
1 2.428026 -1.371838 1.412293
1 1.916886 -2.456939 0.110585
7 2.197410 1.533210 0.090504
1 2.226266 2.544778 0.048470
1 3.055094 0.995709 0.018407
8 3.344000 -1.003716 -0.362189
1 3.197210 -1.144818 -1.310658
8 -4.047358 -1.495191 -0.042177
1 -4.657497 -0.745920 0.008856
1 -3.148431 -1.088072 -0.057198
rd_5hmC/rd_5hmC_067.xyz
6 -1.322683 0.509496 -0.055731
6 0.957231 0.880918 0.069174
6 1.200731 -0.556701 0.101520
6 0.046568 -1.369159 0.009239
1 0.166685 -2.450419 0.002893
7 -1.177239 -0.898411 -0.075907
7 -0.275919 1.376514 -0.002985
8 -2.497829 0.947700 -0.098442
6 2.546968 -1.183484 0.318122

1 2.778748 -1.138232 1.390692
1 2.503224 -2.241358 0.035491
7 1.992950 1.722258 0.141655
1 1.822688 2.720699 0.127616
1 2.941409 1.365172 0.090229
8 3.623762 -0.517799 -0.347297
1 3.506547 -0.620001 -1.304857
8 -4.718122 -0.733203 -0.006705
1 -5.426873 -0.119136 0.230933
1 -3.906360 -0.179272 -0.046731
rd_5hmC/rd_5hmC_038.xyz
6 2.210817 0.029288 -0.101522
6 0.137638 -0.988501 0.083473
6 -0.411164 0.217687 0.692004
6 0.486321 1.303090 0.806493
1 0.123387 2.240504 1.222484
7 1.746866 1.255805 0.436726
7 1.412514 -1.063268 -0.287138
8 3.419958 -0.013237 -0.422350
6 -1.816805 0.327280 1.208655
1 -1.892362 -0.234715 2.148814
1 -2.034115 1.378622 1.432765
7 -0.651286 -2.058113 -0.073673
1 -0.271519 -2.895867 -0.497419
1 -1.651608 -1.974325 0.070389
8 -2.793535 -0.222458 0.321762
1 -2.666368 0.220233 -0.547761
8 -1.898804 1.072710 -1.983178
1 -1.186757 0.463396 -2.233178
1 -1.439107 1.831993 -1.591992
rd_5hmC/rd_5hmC_058.xyz
6 1.319743 0.470379 0.043402
6 -0.955076 0.878245 -0.067371
6 -1.221297 -0.555088 -0.101036
6 -0.079831 -1.385943 -0.017539
1 -0.217843 -2.465052 -0.013456

7 1.152090 -0.935464 0.060900
7 0.286026 1.353929 -0.001238
8 2.500693 0.892296 0.080454
6 -2.578004 -1.159471 -0.315247
1 -2.813416 -1.103955 -1.386544
1 -2.549778 -2.219591 -0.039297
7 -1.977777 1.735956 -0.133892
1 -1.791468 2.731465 -0.119711
1 -2.931619 1.394473 -0.076839
8 -3.641557 -0.480713 0.358396
1 -3.523405 -0.592418 1.314716
8 4.796716 -0.678911 0.005943
1 3.954989 -0.171683 0.042470
1 5.490128 -0.004286 -0.013325
rd_5hmC/rd_5hmC_089.xyz
6 2.243314 0.044641 -0.162569
6 0.177437 -0.968047 0.112684
6 -0.355202 0.254440 0.703244
6 0.546106 1.341043 0.765239
1 0.196498 2.288571 1.169561
7 1.794480 1.284819 0.357004
7 1.441398 -1.052516 -0.292434
8 3.442334 -0.006155 -0.518420
6 -1.743360 0.377906 1.262353
1 -1.772308 -0.127269 2.237226
1 -1.965510 1.438026 1.435467
7 -0.614370 -2.041726 0.008283
1 -0.245945 -2.892721 -0.398729
1 -1.609027 -1.954562 0.187793
8 -2.749080 -0.233952 0.454471
1 -2.686645 0.164509 -0.445236
8 -2.185566 0.852721 -2.026241
1 -1.222764 0.735539 -2.039605
1 -2.316144 1.813064 -2.020640
rd_5hmC/rd_5hmC_046.xyz
6 -1.314404 0.945274 -0.036232

6 1.000339 0.912483 0.060345
6 0.992418 -0.546392 0.111252
6 -0.282408 -1.151465 0.015820
1 -0.355528 -2.236897 0.010732
7 -1.401872 -0.470614 -0.071150
7 -0.128363 1.614632 0.004159
8 -2.399902 1.564369 -0.054477
6 2.212324 -1.389563 0.345173
1 2.463756 -1.340494 1.412983
1 1.983429 -2.434723 0.107333
7 2.168162 1.561956 0.100320
1 2.172601 2.574080 0.061355
1 3.038738 1.045484 0.029768
8 3.376789 -0.946374 -0.357313
1 3.237153 -1.088991 -1.306651
8 -4.089269 -1.414445 -0.034082
1 -4.603068 -0.605736 0.102648
1 -3.153127 -1.104960 -0.062642
rd_5hmC/rd_5hmC_039.xyz
6 -2.685494 -0.101753 -0.045641
6 -0.597789 0.894247 0.093346
6 0.036496 -0.418890 0.124166
6 -0.843353 -1.522873 0.057301
1 -0.426114 -2.527545 0.070287
7 -2.150822 -1.414779 -0.027423
7 -1.918152 1.026015 0.015763
8 -3.930524 -0.011607 -0.131594
6 1.511757 -0.631272 0.294376
1 1.782980 -0.433067 1.338779
1 1.762646 -1.674013 0.074039
7 0.163021 1.992547 0.172603
1 -0.278892 2.903365 0.143345
1 1.170512 1.921930 0.089408
8 2.314628 0.261778 -0.492723
1 2.163725 0.074029 -1.433207
8 5.032703 -0.204510 0.093962

1 5.211535 -1.116751 -0.174627
1 4.091119 -0.056465 -0.133080
rd_5hmC/rd_5hmC_094.xyz
6 -1.334858 -0.765980 -0.076831
6 0.457930 0.692825 0.031788
6 1.382542 -0.432621 0.108657
6 0.786901 -1.712137 0.018101
1 1.428095 -2.590822 0.028763
7 -0.507424 -1.913769 -0.086483
7 -0.857942 0.507379 -0.044280
8 -2.571479 -0.978051 -0.111958
6 2.855817 -0.303209 0.366335
1 3.001468 -0.125103 1.440217
1 3.352167 -1.248329 0.118134
7 0.937538 1.939433 0.062804
1 0.294327 2.720051 0.007596
1 1.938883 2.098206 0.019841
8 3.481705 0.795141 -0.303542
1 3.476964 0.619674 -1.257564
8 -4.384745 1.123101 0.073929
1 -3.681130 0.438113 0.009683
1 -3.909818 1.962109 0.153310
rd_5hmU/rd_5hmU214_aan.xyz
6 1.327823 1.255138 0.064822
6 -0.233467 -0.609256 -0.143786
6 -1.311063 0.385441 -0.156083
6 -0.948811 1.748538 -0.068180
1 -1.737498 2.497363 -0.082387
7 0.287484 2.194378 0.038345
7 1.034856 -0.092806 -0.021762
8 2.499996 1.630557 0.167264
8 -0.433404 -1.829350 -0.243645
1 1.827539 -0.760432 -0.011246
6 -2.731262 -0.064274 -0.313230
1 -3.406128 0.782991 -0.176005
1 -2.863463 -0.439914 -1.339567

8 -3.108840 -1.060288 0.642402
1 -2.583853 -1.857526 0.463516
8 3.121133 -2.030883 -0.062586
1 2.637916 -2.871317 -0.068090
1 3.618713 -2.035986 0.768966
rd_5hmU/rd_5hmU214_aap.xyz
6 -1.547416 1.380641 -0.075255
6 0.086468 -0.436330 0.091365
6 1.122517 0.596897 0.156717
6 0.707952 1.948295 0.107772
1 1.467292 2.724987 0.159530
7 -0.542495 2.351440 -0.002252
7 -1.197454 0.038475 -0.032679
8 -2.731397 1.705687 -0.177602
8 0.332843 -1.650887 0.150660
1 -1.954131 -0.641943 -0.081796
6 2.555710 0.199558 0.337633
1 3.197963 1.075306 0.224753
1 2.677255 -0.178069 1.365176
8 2.998555 -0.770407 -0.614285
1 2.504687 -1.591945 -0.458411
8 -1.874318 -3.464799 -0.050656
1 -1.094188 -2.887583 0.064468
1 -1.976082 -3.921276 0.796465
rd_5hmU/rd_5hmU214_abf.xyz
6 -2.689302 -0.125606 0.081772
6 -0.482445 0.921708 -0.129298
6 0.076595 -0.433476 -0.144493
6 -0.825918 -1.520886 -0.075346
1 -0.423982 -2.530969 -0.103171
7 -2.134529 -1.409706 0.034757
7 -1.851482 0.976374 0.008402
8 -3.907423 0.030018 0.188161
8 0.202222 1.947805 -0.238498
1 -2.289009 1.895579 0.025964
6 1.551250 -0.628912 -0.306934

1 1.821211 -1.658185 -0.062524
1 1.803033 -0.458376 -1.366102
8 2.335838 0.220462 0.536924
1 2.134745 1.143415 0.304480
8 5.043423 -0.314499 -0.017379
1 5.569310 0.387662 0.390732
1 4.113203 -0.093062 0.200648
rd_5hmU/rd_5hmU214_aaf.xyz
6 -1.334840 1.166695 -0.059797
6 0.290410 -0.643136 0.135660
6 1.332690 0.388923 0.147610
6 0.922916 1.739386 0.065993
1 1.685349 2.514931 0.080346
7 -0.328513 2.142451 -0.033799
7 -0.994808 -0.170874 0.018761
8 -2.520544 1.499665 -0.155127
8 0.533691 -1.855694 0.232694
1 -1.773555 -0.855859 0.001233
6 2.767962 -0.008372 0.308926
1 3.412516 0.859004 0.152244
1 2.913033 -0.353598 1.344732
8 3.183894 -1.012377 -0.620923
1 2.672185 -1.817397 -0.437640
8 -3.216117 -1.934134 -0.094676
1 -3.429969 -2.263269 0.791484
1 -3.938523 -1.325507 -0.313243
rd_5hmU/rd_5hmU214_aag.xyz
6 -2.189872 0.134624 -0.062988
6 -0.060670 -1.079286 -0.139864
6 0.465766 -0.058586 0.770900
6 -0.402336 0.980097 1.176311
1 -0.016791 1.747419 1.843281
7 -1.661050 1.099753 0.802423
7 -1.377085 -0.897946 -0.504508
8 -3.363505 0.201288 -0.433418
8 0.605204 -2.036611 -0.552614

1 -1.793713 -1.581796 -1.133163
6 1.891945 -0.144448 1.220328
1 2.147361 0.731441 1.819049
1 2.021462 -1.041210 1.841042
8 2.802188 -0.171038 0.108706
1 2.706441 -1.027306 -0.340678
8 1.815801 1.741144 -1.776737
1 2.209501 1.091289 -1.159316
1 0.886173 1.794848 -1.511246
rd_5hmU/rd_5hmU214_abn.xyz
6 1.296210 1.060605 -0.011754
6 -1.151874 0.902784 0.060459
6 -0.986709 -0.551767 0.151132
6 0.332312 -1.065173 0.167346
1 0.475683 -2.140788 0.241763
7 1.421126 -0.329795 0.086324
7 0.026379 1.612245 -0.044556
8 2.295611 1.777408 -0.067634
8 -2.250260 1.471738 0.084449
1 -0.037514 2.626870 -0.101078
6 -2.181811 -1.437854 0.316105
1 -1.897723 -2.475599 0.126984
1 -2.498561 -1.370018 1.370980
8 -3.264489 -1.129822 -0.558910
1 -3.552582 -0.221110 -0.371630
8 4.023039 -1.561260 -0.063121
1 3.954930 -2.186752 -0.798282
1 3.143260 -1.125839 -0.019120
rd_5hmU/rd_5hmU214_abi.xyz
6 -1.308112 0.865207 -0.032202
6 0.515488 -0.757316 0.177351
6 1.433695 0.384797 0.157473
6 0.873293 1.679448 0.064789
1 1.543287 2.535898 0.062984
7 -0.415703 1.939564 -0.028680
7 -0.819471 -0.424605 0.072905

8 -2.523607 1.067749 -0.129014
8 0.884106 -1.932659 0.287737
1 -1.495770 -1.186311 0.075017
6 2.908289 0.150042 0.278272
1 3.445344 1.088909 0.129516
1 3.119816 -0.204455 1.298618
8 3.403623 -0.774710 -0.694234
1 3.033226 -1.649523 -0.494333
8 -4.266299 -1.220877 -0.156217
1 -3.722346 -0.409202 -0.153509
1 -3.795846 -1.829130 0.431854
rd_5hmU/rd_5hmU214_abx.xyz
6 2.686667 0.143801 0.092066
6 0.492294 -0.927959 -0.126149
6 -0.080426 0.421014 -0.157893
6 0.810394 1.518274 -0.092269
1 0.398444 2.523913 -0.132618
7 2.119219 1.421657 0.028762
7 1.860654 -0.967371 0.023216
8 3.905514 0.001757 0.208573
8 -0.180678 -1.962076 -0.232305
1 2.307194 -1.881919 0.053080
6 -1.555477 0.600297 -0.335051
1 -1.838319 1.627946 -0.098401
1 -1.794903 0.422495 -1.395921
8 -2.339775 -0.252792 0.504898
1 -2.128245 -1.175002 0.279065
8 -5.056125 0.231494 -0.023872
1 -4.115457 0.054755 0.190475
1 -5.440029 0.568182 0.797901
rd_5hmU/rd_5hmU214_abg.xyz
6 -1.466623 1.289830 -0.067221
6 0.153210 -0.531269 0.136606
6 1.199755 0.494430 0.146757
6 0.795240 1.846789 0.060380
1 1.561688 2.618277 0.073171

7 -0.453582 2.256949 -0.042490
7 -1.129406 -0.049890 0.014448
8 -2.649823 1.625415 -0.163652
8 0.386728 -1.746256 0.238125
1 -1.886259 -0.744074 -0.001872
6 2.633324 0.091682 0.309718
1 3.280421 0.956767 0.151017
1 2.776646 -0.250917 1.346587
8 3.045655 -0.915392 -0.617779
1 2.541961 -1.723266 -0.426082
8 -2.406068 -2.672248 0.071289
1 -1.441770 -2.799656 0.122808
1 -2.653146 -2.987465 -0.810640
rd_5hmU/rd_5hmU214_abc.xyz
6 -2.355864 -0.217165 -0.206544
6 -0.046269 -0.716925 0.457858
6 0.205589 0.724962 0.392385
6 -0.864292 1.560814 -0.011593
1 -0.679588 2.630121 -0.085076
7 -2.083682 1.152013 -0.294759
7 -1.338538 -1.086336 0.159450
8 -3.482203 -0.653368 -0.451461
8 0.806571 -1.562773 0.764222
1 -1.569719 -2.077061 0.202662
6 1.570150 1.281656 0.675517
1 1.480343 2.303331 1.050390
1 2.088838 0.669760 1.419026
8 2.308642 1.337113 -0.554941
1 2.686764 0.440258 -0.716398
8 3.270025 -1.247297 -0.648794
1 3.178643 -1.684255 -1.508301
1 2.490104 -1.531338 -0.133092
rd_5hmU/rd_5hmU214_abl.xyz
6 -2.190532 0.141573 -0.062833
6 -0.071558 -1.089365 -0.143537
6 0.467782 -0.067014 0.757826

6 -0.390433 0.979447 1.163852
1 0.003954 1.746841 1.825550
7 -1.650339 1.106347 0.796072
7 -1.388588 -0.900542 -0.501670
8 -3.364990 0.216371 -0.429118
8 0.585419 -2.053757 -0.554122
1 -1.813468 -1.584161 -1.125041
6 1.894879 -0.163472 1.202153
1 2.159886 0.711727 1.797735
1 2.019422 -1.059967 1.824235
8 2.800692 -0.199513 0.087126
1 2.689621 -1.052039 -0.365938
8 1.819062 1.767152 -1.740639
1 1.016597 2.052816 -1.279873
1 2.214191 1.095115 -1.148230
rd_5hmU/rd_5hmU214_aaj.xyz
6 -1.324317 0.452412 0.043258
6 1.077277 0.905767 -0.118117
6 1.272866 -0.546609 -0.145473
6 0.122330 -1.368044 -0.097096
1 0.254994 -2.446766 -0.131265
7 -1.116892 -0.928426 -0.003914
7 -0.237577 1.305733 -0.003915
8 -2.466235 0.916916 0.125850
8 1.999915 1.725432 -0.201905
1 -0.429987 2.305395 0.016161
6 2.651985 -1.109478 -0.296837
1 2.629974 -2.187940 -0.127421
1 2.974827 -0.939882 -1.336702
8 3.595488 -0.558976 0.623502
1 3.693637 0.386308 0.424240
8 -4.853945 -0.595542 0.074057
1 -4.662793 -1.514189 -0.161811
1 -3.982002 -0.151002 0.085173
rd_5hmU/rd_5hmU214_aao.xyz
6 1.453688 -0.192757 -0.832248

6 -0.649136 0.872175 -0.157647
6 -1.131368 -0.469413 0.184648
6 -0.255855 -1.559718 -0.024164
1 -0.604976 -2.559702 0.222004
7 0.968683 -1.463932 -0.503592
7 0.627844 0.908414 -0.675812
8 2.601307 -0.050706 -1.260850
8 -1.315412 1.902088 0.003113
1 1.014773 1.817690 -0.920892
6 -2.503827 -0.633073 0.763099
1 -2.737397 -1.694705 0.863735
1 -2.515221 -0.184971 1.767729
8 -3.520969 -0.056534 -0.062503
1 -3.413403 0.908031 -0.044511
8 3.392894 0.287755 1.840043
1 3.581439 -0.143107 0.992733
1 2.755524 0.981287 1.614481
rd_5hmU/rd_5hmU214_aai.xyz
6 -1.307064 0.860165 -0.015860
6 0.526196 -0.751962 0.194209
6 1.439643 0.392634 0.138882
6 0.872139 1.683827 0.036994
1 1.538704 2.542596 0.011984
7 -0.418976 1.937743 -0.041889
7 -0.811093 -0.425788 0.101599
8 -2.524790 1.055413 -0.097277
8 0.899881 -1.924020 0.323345
1 -1.484654 -1.188903 0.132279
6 2.917100 0.169616 0.242399
1 3.446181 1.101428 0.031805
1 3.147565 -0.121169 1.279414
8 3.405086 -0.806613 -0.680426
1 3.022966 -1.666028 -0.439729
8 -4.316146 -1.201936 -0.117767
1 -3.733960 -0.417402 -0.116087
1 -3.726658 -1.942648 -0.320393

rd_5hmU/rd_5hmU214_abe.xyz
6 -2.046067 -0.899304 0.094513
6 -0.720112 1.118514 -0.337968
6 0.439028 0.373426 0.161893
6 0.237745 -0.968095 0.562421
1 1.091901 -1.534570 0.924827
7 -0.920807 -1.596221 0.547567
7 -1.905997 0.417884 -0.315356
8 -3.151923 -1.443475 0.061263
8 -0.659710 2.281218 -0.759666
1 -2.741196 0.890963 -0.654850
6 1.794136 1.011186 0.167411
1 2.480970 0.405343 0.763502
1 2.166957 1.016594 -0.870208
8 1.812427 2.327266 0.719063
1 1.234028 2.887192 0.175344
8 3.257215 -2.531036 -0.653723
1 4.185003 -2.679879 -0.420647
1 3.077509 -1.623435 -0.368562
rd_5hmU/rd_5hmU214_abu.xyz
6 2.187411 -0.477881 -0.059801
6 0.422025 1.223986 -0.112656
6 -0.441628 0.239230 0.546268
6 0.106860 -1.029574 0.842115
1 -0.525293 -1.766279 1.330981
7 1.342466 -1.399990 0.568618
7 1.702174 0.783045 -0.370223
8 3.350547 -0.778831 -0.336006
8 0.052099 2.365275 -0.414484
1 2.345948 1.431762 -0.818167
6 -1.849100 0.618972 0.893280
1 -2.359480 -0.228045 1.355075
1 -1.826549 1.444491 1.618390
8 -2.604972 0.983517 -0.268586
1 -2.266937 1.833742 -0.592693
8 -2.580821 -2.353699 -0.911270

1 -3.086693 -1.819980 -0.281219
1 -1.881716 -1.755577 -1.213593
rd_5hmU/rd_5hmU214_aas.xyz
6 -2.111032 0.161536 0.190590
6 0.061898 0.947802 -0.631406
6 0.673453 0.060188 0.362239
6 -0.185566 -0.692067 1.195743
1 0.259278 -1.348214 1.940043
7 -1.502782 -0.669994 1.138846
7 -1.316214 0.928239 -0.647557
8 -3.338968 0.215859 0.099630
8 0.714102 1.658924 -1.405017
1 -1.791819 1.529096 -1.317956
6 2.168644 -0.012204 0.448981
1 2.463238 -0.754197 1.192679
1 2.574304 -0.313325 -0.526104
8 2.724677 1.242775 0.866595
1 2.641130 1.871398 0.132137
8 0.935472 -3.158088 -1.124640
1 1.433822 -2.336106 -1.240832
1 0.226373 -2.923672 -0.508423
rd_5hmU/rd_5hmU214_abr.xyz
6 -2.433806 -0.336219 -0.048140
6 -0.009296 -0.689969 0.038846
6 0.118660 0.754437 0.253394
6 -1.068289 1.522087 0.297068
1 -0.983758 2.594384 0.456981
7 -2.287759 1.039064 0.165765
7 -1.303681 -1.138582 -0.092017
8 -3.551469 -0.833787 -0.195427
8 0.949699 -1.471074 -0.021705
1 -1.446425 -2.134212 -0.250981
6 1.481578 1.370883 0.354195
1 1.398826 2.403499 0.696370
1 2.100077 0.809576 1.062553
8 2.094912 1.412966 -0.945291

1 2.373143 0.514927 -1.185011
8 3.795989 -1.301496 0.266891
1 2.820850 -1.309269 0.220481
1 4.031246 -0.362469 0.275456
rd_5hmU/rd_5hmU114_abp.xyz
6 1.207208 1.162998 -0.004257
6 -1.120015 0.922977 0.003614
6 -0.988721 -0.553047 0.003462
6 0.315597 -1.082949 0.008994
1 0.511482 -2.151299 0.014971
7 1.348881 -0.269531 0.004423
7 -0.019882 1.706333 -0.003929
8 2.272669 1.801686 -0.010453
8 -2.281636 1.422144 0.013199
6 -2.135819 -1.497029 0.026767
1 -2.001902 -2.222879 -0.794918
1 -2.038172 -2.102793 0.949055
8 -3.428467 -0.935972 -0.045397
1 -3.315755 0.044757 -0.013916
1 2.318600 -0.664383 0.003938
8 3.800436 -1.546707 0.019583
1 4.232889 -1.485470 -0.846038
1 4.416359 -1.122454 0.636505
rd_5hmU/rd_5hmU114_abc.xyz
6 2.449497 -0.143720 0.341051
6 0.323227 -0.924261 -0.243690
6 -0.158144 0.464790 -0.449805
6 0.766454 1.507754 -0.255267
1 0.511657 2.553673 -0.396241
7 1.991653 1.211479 0.122894
7 1.594779 -1.156245 0.147354
8 3.631485 -0.247489 0.697838
8 -0.483676 -1.871129 -0.453350
6 -1.525081 0.799293 -0.929659
1 -1.884960 1.703148 -0.418553
1 -1.413971 1.089813 -1.994785

8 -2.500584 -0.217081 -0.800704
1 -2.013483 -1.074445 -0.744567
1 2.674987 1.952194 0.283582
8 -3.885445 0.172653 1.614880
1 -4.779601 0.452268 1.373211
1 -3.429616 0.017934 0.760533
rd_5mC/rd_5mC_066.xyz
6 0.888703 -0.786747 0.000004
6 -0.748013 0.845767 0.000002
6 -1.790266 -0.173915 -0.000003
6 -1.319953 -1.509218 0.000001
1 -2.048681 -2.316982 0.000001
7 -0.049074 -1.844269 0.000007
7 0.544710 0.530722 0.000004
8 2.099371 -1.120981 0.000000
7 -1.095313 2.137660 0.000007
1 -0.372246 2.846857 0.000007
1 -2.060600 2.437118 0.000000
6 -3.238243 0.152160 -0.000010
1 -3.502645 0.752386 0.881381
1 -3.502634 0.752405 -0.881390
1 -3.844054 -0.755946 -0.000023
8 4.114688 0.807524 -0.000012
1 3.362684 0.173215 -0.000002
1 3.700077 1.681527 0.000029
rd_5mC/rd_5mC_062.xyz
6 -0.665344 -1.372087 -0.028021
6 0.270542 0.758414 -0.049361
6 1.609396 0.186248 0.014162
6 1.655490 -1.228095 0.057146
1 2.627634 -1.713306 0.107991
7 0.595728 -2.006288 0.040031
7 -0.813197 -0.013304 -0.073245
8 -1.674953 -2.108446 -0.045952
7 0.116880 2.086439 -0.081967
1 -0.815521 2.481338 -0.122058

1 0.907069 2.716454 -0.059369
6 2.838043 1.018012 0.033485
1 2.890107 1.657881 -0.858044
1 2.839279 1.690224 0.902792
1 3.732819 0.393784 0.070895
8 -3.338664 1.271242 0.048772
1 -2.483553 0.778086 -0.008486
1 -3.133539 2.050290 0.585534
rd_5mC/rd_5mC_050.xyz
6 -0.913509 -0.470819 0.000001
6 1.381478 -0.743398 0.000002
6 1.572321 0.702135 0.000001
6 0.377976 1.462339 0.000013
1 0.451573 2.547746 0.000022
7 -0.827859 0.939821 0.000016
7 0.170885 -1.294743 -0.000002
8 -2.069039 -0.962204 -0.000008
7 2.453713 -1.543547 0.000005
1 2.324566 -2.548147 0.000011
1 3.396631 -1.179815 0.000017
6 2.914257 1.336592 -0.000016
1 3.491855 1.024758 0.881094
1 3.491825 1.024764 -0.881149
1 2.834091 2.425241 -0.000012
8 -4.419906 0.514664 -0.000006
1 -5.091271 -0.182117 0.000003
1 -3.560012 0.036080 -0.000009
rd_5mC/rd_5mC_054.xyz
6 0.816359 -1.004236 0.000245
6 -1.462649 -0.595274 -0.000910
6 -1.227300 0.844845 0.000811
6 0.133982 1.230658 -0.000354
1 0.381328 2.290159 -0.000593
7 1.131712 0.376990 -0.001218
7 -0.464162 -1.473943 -0.000714
8 1.786938 -1.792858 0.002406

7 -2.721578 -1.049804 -0.002645
1 -2.890289 -2.048467 -0.004065
1 -3.518598 -0.428341 -0.002682
6 -2.328654 1.839187 0.003581
1 -2.972288 1.707567 -0.877001
1 -2.969676 1.704836 0.885682
1 -1.939115 2.858780 0.004615
8 3.890243 1.048017 -0.008797
1 4.347727 0.198116 0.062047
1 2.931239 0.812304 -0.005070
rd_5mC/rd_5mC_086.xyz
6 1.014544 1.514957 0.013421
6 -0.307106 -0.388999 0.038004
6 0.881483 -1.234274 -0.004881
6 2.111742 -0.537397 -0.059092
1 3.033324 -1.113996 -0.105861
7 2.220600 0.772867 -0.055304
7 -0.221153 0.940394 0.051093
8 1.129039 2.763078 0.037506
7 -1.517970 -0.954030 0.064074
1 -2.361910 -0.372187 0.069561
1 -1.627854 -1.958638 0.049918
6 0.816894 -2.718734 -0.001887
1 0.288624 -3.083239 0.889668
1 0.253794 -3.083915 -0.871963
1 1.817239 -3.155607 -0.022747
8 -4.014618 0.567997 0.025051
1 -4.050190 1.058234 -0.809889
1 -4.744072 -0.067197 -0.031581
rd_5mC/rd_5mC_070.xyz
6 -1.036639 -1.507345 -0.011073
6 0.308792 0.379941 -0.025940
6 -0.869564 1.240054 0.003323
6 -2.109019 0.558670 0.047064
1 -3.023812 1.146709 0.083670
7 -2.233994 -0.750102 0.045644

7 0.206520 -0.948296 -0.036958
8 -1.166798 -2.753961 -0.035900
7 1.526726 0.930081 -0.042100
1 2.364309 0.339199 -0.046680
1 1.648496 1.933348 -0.031070
6 -0.786018 2.723622 -0.003460
1 -0.224206 3.084386 0.869230
1 -0.247144 3.078271 -0.892650
1 -1.780704 3.173604 0.009006
8 4.013278 -0.604647 -0.019065
1 4.208594 -0.858717 0.895299
1 4.742548 -0.019371 -0.272669
rd_5mC/rd_5mC_039.xyz
6 -2.201425 -0.423834 -0.004947
6 0.113353 -0.562412 0.006182
6 0.216695 0.892641 0.002227
6 -1.019256 1.581094 0.011986
1 -1.007281 2.669117 0.019485
7 -2.194726 0.992785 0.012326
7 -1.067088 -1.180812 -0.002415
8 -3.327533 -0.974662 -0.020699
7 1.228416 -1.299463 0.015889
1 1.138127 -2.308511 0.020069
1 2.171194 -0.904522 0.030127
6 1.514825 1.613532 -0.015732
1 2.128096 1.338728 0.852409
1 2.095185 1.342495 -0.908002
1 1.361690 2.694665 -0.011269
8 4.062453 -0.551136 0.096260
1 4.549919 -1.147561 -0.491077
1 4.172343 0.328274 -0.295128
rd_5mC/rd_5mC_060.xyz
6 -0.894604 -1.557654 -0.014539
6 0.293225 0.432773 -0.039962
6 -0.951086 1.193411 0.002110
6 -2.129612 0.413840 0.058838

1 -3.088192 0.926013 0.108167
7 -2.147552 -0.900755 0.057863
7 0.298941 -0.899514 -0.054798
8 -0.924248 -2.810412 -0.040135
7 1.460691 1.083732 -0.063568
1 2.342857 0.562164 -0.052545
1 1.494770 2.093802 -0.043361
6 -0.992112 2.678123 -0.002930
1 -0.447341 3.086676 0.859354
1 -0.504783 3.079218 -0.901927
1 -2.021444 3.040384 0.029315
8 4.025006 -0.313473 0.109726
1 3.855823 -1.127547 0.607487
1 4.322818 -0.616837 -0.760793
rd_5mC/rd_5mC_045.xyz
6 -0.584660 1.179145 -0.307186
6 0.610876 -0.804087 -0.289174
6 1.794287 -0.107840 0.198779
6 1.623108 1.278494 0.423499
1 2.466171 1.850794 0.804745
7 0.504216 1.930090 0.196532
7 -0.529395 -0.166946 -0.534057
8 -1.638708 1.812400 -0.544092
7 0.671183 -2.125151 -0.501859
1 -0.148891 -2.611324 -0.842895
1 1.512139 -2.659086 -0.331550
6 3.089322 -0.792627 0.437914
1 2.981948 -1.584443 1.192211
1 3.446614 -1.279318 -0.479767
1 3.848008 -0.085932 0.779828
8 -4.490350 -0.895171 0.645726
1 -5.150046 -1.603773 0.628075
1 -3.643094 -1.349215 0.528972
rd_5mC/rd_5mC_037.xyz
6 -0.881681 -1.561491 -0.012437
6 0.292446 0.436894 -0.047827

6 -0.956713 1.189076 0.002720
6 -2.129612 0.401638 0.066223
1 -3.091366 0.907298 0.120726
7 -2.138548 -0.913020 0.066055
7 0.307257 -0.895407 -0.060405
8 -0.903284 -2.814447 -0.036579
7 1.455061 1.096174 -0.081092
1 2.340864 0.580605 -0.072329
1 1.481847 2.106492 -0.063636
6 -1.007752 2.673432 -0.000795
1 -0.458669 3.085007 0.857260
1 -0.530987 3.078770 -0.903543
1 -2.039228 3.028712 0.040398
8 4.025706 -0.295127 0.061110
1 4.032753 -0.999528 -0.604475
1 4.018898 -0.762288 0.910136
rd_5mC/rd_5mC_013.xyz
6 -1.790993 -0.088460 0.039514
6 0.307573 -1.017987 -0.265229
6 0.865741 -0.138623 0.754911
6 -0.062603 0.743039 1.357494
1 0.293127 1.425808 2.126151
7 -1.338355 0.797963 1.046035
7 -0.978517 -0.982009 -0.598443
8 -3.005840 -0.022113 -0.257007
7 1.111853 -1.886683 -0.891919
1 0.727315 -2.496421 -1.602973
1 2.097803 -1.953219 -0.681069
6 2.304096 -0.141722 1.121540
1 2.613296 -1.131159 1.485181
1 2.926986 0.081013 0.244332
1 2.512035 0.597808 1.897224
8 1.164613 2.285687 -1.718268
1 1.136313 2.397743 -0.756830
1 1.115188 1.327451 -1.848898
rd_5mC/rd_5mC_051.xyz

6 2.201039 -0.426654 0.008328
6 -0.113950 -0.559854 -0.017559
6 -0.213793 0.895430 -0.013544
6 1.023380 1.580908 0.022472
1 1.013487 2.668857 0.038402
7 2.197308 0.989763 0.038172
7 1.065076 -1.180982 -0.009871
8 3.326011 -0.979949 0.002253
7 -1.230611 -1.294495 -0.029616
1 -1.142534 -2.303747 -0.030363
1 -2.172831 -0.898302 -0.030181
6 -1.509902 1.619637 -0.037380
1 -2.100687 1.335058 -0.917821
1 -2.114646 1.361982 0.842630
1 -1.353672 2.700256 -0.051644
8 -4.074665 -0.577221 -0.056743
1 -4.180262 0.366475 0.135208
1 -4.502683 -1.030021 0.684996
rd_5mC/rd_5mC_049.xyz
6 1.103423 0.025461 -0.865529
6 -0.938283 -0.808796 -0.160507
6 -1.413996 0.540648 0.117317
6 -0.495548 1.573625 -0.185676
1 -0.799612 2.604686 -0.017704
7 0.712158 1.370823 -0.663737
7 0.280146 -1.040982 -0.638226
8 2.269966 -0.160505 -1.280888
7 -1.750689 -1.848201 0.071762
1 -1.421583 -2.788458 -0.108769
1 -2.685322 -1.728716 0.436491
6 -2.763852 0.819743 0.668995
1 -3.545464 0.431904 0.001160
1 -2.901019 0.318286 1.636686
1 -2.918681 1.891841 0.805206
8 2.858836 -0.191996 1.983018
1 2.781760 0.658609 1.526360

1 2.817736 -0.843707 1.267327
rd_5mC/rd_5mC_040.xyz
6 -1.706729 -0.315885 0.093665
6 0.465557 -0.876023 -0.482157
6 0.974754 -0.189942 0.699181
6 -0.015424 0.393152 1.525681
1 0.302230 0.910640 2.428415
7 -1.305847 0.348162 1.278746
7 -0.833586 -0.929070 -0.759901
8 -2.934645 -0.328403 -0.152086
7 1.326896 -1.475774 -1.314064
1 0.974042 -1.962021 -2.128986
1 2.323312 -1.479788 -1.146135
6 2.421664 -0.105941 1.021527
1 2.853897 -1.109563 1.136257
1 2.973027 0.384022 0.207639
1 2.585591 0.453995 1.944272
8 0.542508 2.639582 -1.239307
1 0.147115 3.249424 -1.878861
1 -0.173298 2.028459 -1.012306
rd_5mC/rd_5mC_015.xyz
6 -2.201065 -0.415721 -0.012088
6 0.112586 -0.566159 0.029713
6 0.223093 0.888394 0.022676
6 -1.008092 1.582393 -0.047552
1 -0.989230 2.669820 -0.081844
7 -2.186045 0.999593 -0.074233
7 -1.070969 -1.178377 0.020932
8 -3.330231 -0.960157 0.004867
7 1.223483 -1.309412 0.048599
1 1.127066 -2.317920 0.048596
1 2.168832 -0.920831 0.032569
6 1.523686 1.602715 0.081288
1 2.079736 1.321430 0.985431
1 2.157983 1.331012 -0.772805
1 1.375763 2.684579 0.080524

8 4.073261 -0.623347 -0.021827
1 4.168476 0.250800 0.385077
1 4.300592 -0.483227 -0.953178
rd_5mC/rd_5mC_048.xyz
6 1.741176 -0.204709 -0.113421
6 -0.407005 -0.981455 0.262884
6 -0.936583 -0.052689 -0.728179
6 0.032469 0.756233 -1.367374
1 -0.299428 1.465510 -2.122536
7 1.320449 0.709349 -1.109313
7 0.890508 -1.047937 0.543907
8 2.965489 -0.219871 0.149326
7 -1.251906 -1.792717 0.911843
1 -0.892181 -2.437824 1.604236
1 -2.245499 -1.786051 0.728288
6 -2.386634 0.065694 -1.025869
1 -2.798367 -0.893843 -1.367207
1 -2.944384 0.343505 -0.120452
1 -2.569205 0.818913 -1.794739
8 -0.705865 2.231211 1.827838
1 -1.341152 2.145987 1.102017
1 0.039327 1.673779 1.559780
rd_5mC/rd_5mC_022.xyz
6 1.787122 -0.445281 0.019737
6 0.187871 1.209910 -0.234564
6 -0.748826 0.455246 0.587912
6 -0.246400 -0.762262 1.105226
1 -0.893616 -1.362125 1.741222
7 0.961339 -1.222237 0.867340
7 1.408581 0.758446 -0.504936
8 2.918659 -0.914583 -0.239622
7 -0.193265 2.390474 -0.739944
1 0.452003 2.921742 -1.311369
1 -1.109929 2.776663 -0.562491
6 -2.139593 0.907928 0.846409
1 -2.150008 1.884870 1.348703

1 -2.685840 1.033692 -0.098817
1 -2.676336 0.188042 1.467249
8 -2.255642 -2.239316 -1.094495
1 -2.261964 -1.280471 -1.228818
1 -2.256080 -2.611249 -1.988289
rd_5mC/rd_5mC_063.xyz
6 1.901189 0.291235 -0.096035
6 -0.251775 0.386763 0.749824
6 -0.525210 -0.957047 0.256819
6 0.545971 -1.568531 -0.436589
1 0.399460 -2.568258 -0.840034
7 1.718338 -1.005907 -0.629829
7 0.926134 0.973868 0.576897
8 3.025666 0.814109 -0.267078
7 -1.223461 1.063239 1.383092
1 -1.024518 1.982247 1.758957
1 -2.116182 0.641222 1.598478
6 -1.830742 -1.637230 0.451571
1 -2.049418 -1.755660 1.522087
1 -2.650168 -1.040714 0.027953
1 -1.832509 -2.623056 -0.016995
8 -2.438885 1.322589 -1.763317
1 -2.235473 1.566680 -0.847683
1 -2.169110 0.394409 -1.824276
rd_5mC/rd_5mC_057.xyz
6 -1.506409 -0.398567 -0.486507
6 0.637110 0.445364 -0.727759
6 1.171379 -0.621894 0.108618
6 0.210772 -1.541670 0.591603
1 0.548716 -2.362790 1.220325
7 -1.075157 -1.474699 0.325936
7 -0.657776 0.531609 -1.017819
8 -2.735988 -0.324529 -0.710216
7 1.472847 1.368325 -1.219528
1 1.106742 2.120529 -1.789833
1 2.463264 1.354519 -1.019310

6 2.613945 -0.739340 0.437792
1 3.217858 -0.826737 -0.475546
1 2.966755 0.158760 0.963389
1 2.802373 -1.610374 1.068319
8 -0.821666 1.871377 2.044370
1 -1.076601 1.995555 1.117809
1 -0.508053 0.955750 2.079015
rd_5mC/rd_5mC_069.xyz
6 1.813180 -0.673232 -0.041433
6 0.424969 1.177495 -0.150539
6 -0.641445 0.439845 0.514474
6 -0.321413 -0.895724 0.857807
1 -1.076823 -1.498252 1.358099
7 0.837897 -1.463732 0.612825
7 1.605693 0.625482 -0.410460
8 2.911726 -1.225156 -0.279704
7 0.214167 2.450216 -0.511942
1 0.953174 2.965174 -0.974628
1 -0.666169 2.916836 -0.344473
6 -1.970962 1.033173 0.807836
1 -1.868798 1.922883 1.443942
1 -2.463836 1.360809 -0.117393
1 -2.618111 0.313779 1.313338
8 -3.072098 -1.660834 -1.087267
1 -3.065785 -1.568236 -0.123190
1 -2.340933 -1.098176 -1.381749
rd_5mU/rd_5mU4_aaw.xyz
6 0.738069 -1.243786 0.015857
6 -0.449579 0.893272 0.032710
6 -1.698870 0.122659 -0.009148
6 -1.589969 -1.289073 -0.029213
1 -2.506308 -1.874208 -0.056198
7 -0.459122 -1.966337 -0.017473
7 0.701504 0.137390 0.050276
8 1.824328 -1.835493 0.017356
8 -0.411139 2.131487 0.050790

1 1.611564 0.629636 0.056090
6 -2.997058 0.832377 -0.031323
1 -3.083366 1.495341 0.839422
1 -3.057973 1.482435 -0.914524
1 -3.834448 0.132504 -0.040102
8 3.315830 1.305717 0.034578
1 3.483573 1.738283 -0.816523
1 3.842579 0.492257 0.007120
rd_5mU/rd_5mU4_abt.xyz
6 -0.852802 -0.818753 -0.004336
6 0.841933 0.953416 -0.001056
6 1.852736 -0.111066 0.004895
6 1.387544 -1.449536 0.004053
1 2.124499 -2.249206 0.007683
7 0.121576 -1.817012 -0.000619
7 -0.465781 0.507612 -0.004339
8 -2.054290 -1.116140 -0.007790
8 1.111016 2.159319 -0.002984
1 -1.203408 1.209492 -0.007678
6 3.287203 0.247743 0.004973
1 3.536788 0.801517 -0.910869
1 3.509922 0.926365 0.838075
1 3.920408 -0.638334 0.071964
8 -4.085962 0.901082 0.000221
1 -3.411690 0.193212 0.000361
1 -4.932873 0.437833 0.068411
rd_5mU/rd_5mU4_aab.xyz
6 0.852929 -0.826276 -0.004671
6 -0.834200 0.953014 -0.005796
6 -1.849534 -0.107109 0.004644
6 -1.390052 -1.447549 0.004197
1 -2.130431 -2.244039 0.008550
7 -0.125661 -1.820444 -0.000898
7 0.471594 0.501692 -0.009415
8 2.053211 -1.128768 -0.004036
8 -1.098060 2.160060 -0.011111

1 1.212269 1.200436 -0.016362
6 -3.282479 0.257691 0.008151
1 -3.500733 0.935079 0.843506
1 -3.531609 0.814897 -0.905670
1 -3.919226 -0.625905 0.074472
8 4.069319 0.901477 0.058731
1 3.405643 0.184775 0.019645
1 4.866809 0.525244 -0.339763
rd_5mU/rd_5mU4_abc.xyz
6 0.788114 -1.076649 0.004814
6 -1.610258 -0.547115 -0.005946
6 -1.231036 0.872864 0.006831
6 0.153103 1.175744 0.020560
1 0.457478 2.219691 0.032813
7 1.119925 0.281371 0.021645
7 -0.549128 -1.431987 -0.003256
8 1.669393 -1.938733 -0.002313
8 -2.779994 -0.944465 -0.018661
1 -0.766168 -2.426497 -0.014323
6 -2.285317 1.909409 0.001012
1 -2.901143 1.813683 -0.903684
1 -2.967557 1.761792 0.848218
1 -1.859344 2.912984 0.044690
8 3.883960 1.060838 0.071709
1 4.023596 1.569639 -0.739491
1 2.943057 0.776376 0.033540
rd_5mU/rd_5mU4_aat.xyz
6 0.851448 0.955771 0.003592
6 -1.580371 0.615621 -0.002844
6 -1.311853 -0.829521 0.001995
6 0.043899 -1.240833 0.006879
1 0.264057 -2.305857 0.009996
7 1.077815 -0.424598 0.007363
7 -0.453797 1.414823 -0.001801
8 1.798642 1.744938 0.004965
8 -2.715652 1.102923 -0.007552

1 -0.592148 2.423225 -0.004670
6 -2.445176 -1.778514 0.000950
1 -3.086877 -1.600389 0.874105
1 -3.078531 -1.608075 -0.879855
1 -2.100714 -2.813590 0.006988
8 3.871734 -1.122792 -0.015939
1 2.915818 -0.892960 -0.005962
1 4.324800 -0.269627 0.045242
rd_5mU/rd_5mU4_abm.xyz
6 0.717702 -1.273980 -0.028373
6 -0.423742 0.887521 -0.053347
6 -1.688895 0.145007 0.013874
6 -1.610418 -1.268490 0.044689
1 -2.538771 -1.833370 0.087785
7 -0.494570 -1.970006 0.024574
7 0.710506 0.106986 -0.083022
8 1.790692 -1.889428 -0.030210
8 -0.359244 2.124571 -0.081001
1 1.629867 0.582441 -0.090003
6 -2.970572 0.883305 0.053924
1 -3.010546 1.522233 0.946704
1 -3.048546 1.559926 -0.806719
1 -3.823295 0.202154 0.061411
8 3.306904 1.298326 -0.008966
1 3.872854 0.562078 0.269029
1 3.355620 1.937750 0.717735
rd_5mU/rd_5mU4_abi.xyz
6 2.165910 0.427526 -0.056220
6 -0.279593 0.503352 0.138238
6 -0.263086 -0.961666 0.068869
6 0.999844 -1.588599 -0.074219
1 1.027387 -2.673840 -0.136382
7 2.159526 -0.966165 -0.140296
7 0.959446 1.100483 0.068311
8 3.224888 1.059755 -0.092710
8 -1.309622 1.182616 0.250961

1 0.998019 2.116496 0.119244
6 -1.533141 -1.715133 0.137544
1 -2.100133 -1.429631 1.032237
1 -2.171058 -1.458142 -0.719143
1 -1.355840 -2.791950 0.141862
8 -4.091310 0.706008 -0.168418
1 -4.238486 0.951162 -1.092684
1 -3.123944 0.765774 -0.045174
rd_5mU/rd_5mU4_aap.xyz
6 1.716704 -0.736168 -0.046543
6 0.367928 1.291713 0.272668
6 -0.681813 0.630756 -0.511598
6 -0.412630 -0.675469 -0.989704
1 -1.182046 -1.176305 -1.571568
7 0.703845 -1.346491 -0.789530
7 1.514696 0.542940 0.446512
8 2.781602 -1.321499 0.172494
8 0.258708 2.426708 0.751416
1 2.274008 0.958253 0.982434
6 -1.960443 1.332489 -0.759039
1 -2.442776 1.587596 0.194290
1 -1.778642 2.286906 -1.270501
1 -2.639397 0.722895 -1.357623
8 -2.736685 -1.936073 0.991398
1 -3.512847 -2.170351 1.520590
1 -2.855571 -0.997159 0.786345
rd_5mU/rd_5mU4_abe.xyz
6 -0.736709 -1.615778 -0.012635
6 0.348882 0.583589 -0.052528
6 -0.993277 1.171889 0.011709
6 -2.091313 0.277685 0.069359
1 -3.092679 0.698004 0.124778
7 -2.005612 -1.037588 0.060453
7 0.378493 -0.792674 -0.061571
8 -0.597593 -2.841457 -0.033347
8 1.389246 1.256435 -0.094020

1 1.288106 -1.248341 -0.104549
6 -1.144786 2.642899 0.004320
1 -0.593109 3.087432 0.842928
1 -0.698858 3.062978 -0.907348
1 -2.194255 2.935849 0.061645
8 3.914300 -0.094349 0.099746
1 3.672678 -1.031440 0.071786
1 3.063545 0.380603 0.018208
rd_5mU/rd_5mU4_abn.xyz
6 0.923760 -0.458137 0.007081
6 -1.506444 -0.784105 -0.000326
6 -1.631968 0.679092 -0.001573
6 -0.432405 1.433919 0.002541
1 -0.507218 2.518703 0.002187
7 0.785120 0.929114 0.006845
7 -0.206395 -1.252306 0.004650
8 2.044064 -0.983640 0.009538
8 -2.469851 -1.557868 -0.003403
1 -0.067110 -2.260696 0.004945
6 -2.975945 1.295803 -0.007445
1 -3.542224 0.960239 -0.886371
1 -3.549258 0.962468 0.867761
1 -2.915032 2.385284 -0.008489
8 4.471370 0.452038 -0.037131
1 4.316797 1.378984 0.192616
1 3.586308 0.033686 -0.006811
rd_5mU/rd_5mU4_aar.xyz
6 0.486956 1.514445 0.006089
6 -0.182888 -0.838838 0.008435
6 -1.575475 -0.374580 -0.009266
6 -1.791236 1.025330 -0.014310
1 -2.816779 1.386663 -0.025860
7 -0.844426 1.942833 -0.007035
7 0.765142 0.161303 0.012607
8 1.410157 2.337342 0.011906
8 0.141228 -2.034658 0.019322

1 1.753070 -0.136639 0.020354
6 -2.677026 -1.361889 -0.010868
1 -2.556512 -2.067599 -0.842490
1 -2.642855 -1.965551 0.906560
1 -3.650485 -0.873576 -0.082926
8 3.253897 -1.249575 0.073912
1 2.717142 -2.056955 0.023440
1 3.747165 -1.226970 -0.759680
rd_5mU/rd_5mU4_abr.xyz
6 -0.856980 -0.861496 -0.031501
6 0.797730 0.948476 -0.026455
6 1.831823 -0.092127 0.019572
6 1.396909 -1.440752 0.020705
1 2.151747 -2.223207 0.044169
7 0.139852 -1.837171 -0.003654
7 -0.499183 0.473113 -0.049653
8 -2.051467 -1.185797 -0.042161
8 1.038656 2.160276 -0.044511
1 -1.251078 1.158920 -0.082077
6 3.257566 0.298267 0.054516
1 3.517888 0.857937 -0.854548
1 3.444899 0.980830 0.893303
1 3.907885 -0.573916 0.138308
8 -4.089862 0.831258 0.065121
1 -3.407191 0.132676 0.028566
1 -3.589743 1.655055 0.156807
rd_5mU/rd_5mU4_aag.xyz
6 2.422099 -0.070318 -0.006305
6 0.172499 0.914108 0.005734
6 -0.350490 -0.458140 0.009715
6 0.592983 -1.514941 0.006641
1 0.219715 -2.536272 0.010199
7 1.903038 -1.367672 -0.000709
7 1.549555 1.005790 -0.002643
8 3.641458 0.122740 -0.014581
8 -0.542687 1.922882 0.008995

1 1.961700 1.936538 -0.006225
6 -1.813418 -0.674469 0.015230
1 -2.264252 -0.192358 0.892585
1 -2.269806 -0.194810 -0.860678
1 -2.063362 -1.736791 0.017423
8 -5.185962 -0.009641 -0.013861
1 -4.475798 0.648496 0.014464
1 -4.720863 -0.856912 -0.074814
rd_5mU/rd_5mU4_abl.xyz
6 2.280400 -0.352620 -0.265581
6 0.322138 1.078716 0.119089
6 -0.409721 -0.140716 0.480761
6 0.297721 -1.366519 0.419852
1 -0.234160 -2.278780 0.679878
7 1.562387 -1.501576 0.074212
7 1.641863 0.876331 -0.231323
8 3.468256 -0.424252 -0.594350
8 -0.178731 2.209543 0.122234
1 2.194242 1.693118 -0.483795
6 -1.826244 -0.044347 0.894825
1 -1.904334 0.523049 1.832970
1 -2.403722 0.519297 0.152217
1 -2.268553 -1.032178 1.036746
8 -4.920731 -0.461364 -0.864409
1 -4.207059 0.014364 -1.313891
1 -4.542291 -0.700664 -0.005822
rd_5mU/rd_5mU4_aai.xyz
6 1.566289 -1.248508 0.002066
6 1.082911 1.159361 -0.000465
6 -0.341633 0.802874 -0.003838
6 -0.665817 -0.576150 -0.008475
1 -1.715594 -0.857453 -0.015079
7 0.205699 -1.565053 -0.006180
7 1.947566 0.083112 0.001199
8 2.421773 -2.138656 0.009900
8 1.502113 2.322786 0.000769

1 2.945656 0.282255 0.005521
6 -1.360849 1.875248 -0.005229
1 -1.220678 2.532560 0.862808
1 -1.237591 2.512082 -0.891419
1 -2.372538 1.466529 0.008020
8 -4.408091 -1.001719 -0.052941
1 -5.333618 -0.874962 0.201596
1 -3.950256 -0.223669 0.297235
rd_5mU/rd_5mU4_abp.xyz
6 -1.676465 0.328523 -0.205892
6 0.574434 0.958058 0.547806
6 1.092326 0.205933 -0.601776
6 0.145358 -0.418570 -1.450789
1 0.515958 -0.978804 -2.305864
7 -1.163094 -0.376502 -1.297216
7 -0.802283 0.982668 0.647767
8 -2.893259 0.381127 -0.003246
8 1.292021 1.537604 1.371298
1 -1.209653 1.483601 1.434646
6 2.552304 0.125616 -0.820271
1 2.976916 1.133854 -0.914468
1 3.040602 -0.329164 0.051928
1 2.791229 -0.454629 -1.713096
8 0.348778 -2.782384 1.238815
1 -0.113485 -1.937651 1.138308
1 -0.351994 -3.448511 1.185291
rd_5mU/rd_5mU4_abg.xyz
6 1.765198 0.060911 0.142631
6 -0.429044 1.044636 -0.356242
6 -0.974353 0.187051 0.702885
6 -0.065969 -0.655750 1.390122
1 -0.457305 -1.294684 2.178228
7 1.228320 -0.736624 1.155950
7 0.933466 0.930646 -0.544332
8 2.965724 0.001378 -0.139268
8 -1.112896 1.816057 -1.038874

1 1.356496 1.494172 -1.278682
6 -2.429049 0.188885 0.969164
1 -2.799786 1.213395 1.091436
1 -2.962539 -0.228273 0.102189
1 -2.673601 -0.403858 1.852347
8 -0.983325 -2.214844 -1.694152
1 -1.599452 -2.087553 -0.957899
1 -0.153037 -1.826489 -1.381962
rd_5mU/rd_5mU4_aaq.xyz
6 -1.780081 -0.827234 -0.021954
6 -0.495408 1.254891 -0.240951
6 0.567487 0.596267 0.527770
6 0.337839 -0.737720 0.945512
1 1.119379 -1.238596 1.511665
7 -0.753093 -1.436917 0.702687
7 -1.613149 0.475723 -0.462616
8 -2.825235 -1.435354 -0.271133
8 -0.419882 2.413048 -0.667390
1 -2.380664 0.888909 -0.988274
6 1.820018 1.327713 0.817342
1 2.314135 1.616291 -0.120435
1 1.601365 2.265150 1.344997
1 2.504670 0.723778 1.414895
8 3.574230 -1.477186 -0.907511
1 3.319774 -2.235861 -1.452645
1 2.752987 -0.978873 -0.788743
rd_5mU/rd_5mU4_aao.xyz
6 -1.891997 -0.390892 -0.084872
6 0.412805 -0.216825 0.745620
6 0.464932 1.109137 0.118634
6 -0.701992 1.558948 -0.547084
1 -0.682648 2.544217 -1.006596
7 -1.826137 0.880373 -0.660602
7 -0.783977 -0.885086 0.584191
8 -2.919653 -1.070210 -0.165921
8 1.353329 -0.719961 1.370946

1 -0.866134 -1.810362 1.000114
6 1.709772 1.904622 0.189506
1 2.071566 1.966007 1.222622
1 2.505413 1.406431 -0.383335
1 1.564180 2.908601 -0.213370
8 2.669988 -1.680774 -1.330514
1 2.817814 -0.841916 -0.869481
1 2.070173 -2.162371 -0.741989
rd_5mU/rd_5mU4_aas.xyz
6 -1.753927 -0.181504 0.036827
6 0.495526 -0.124516 1.017250
6 0.975011 -0.744379 -0.224720
6 0.010320 -1.043268 -1.217871
1 0.350385 -1.512998 -2.137839
7 -1.280932 -0.795051 -1.126040
7 -0.866385 0.091852 1.064932
8 -2.947885 0.108378 0.157231
8 1.233287 0.188872 1.958755
1 -1.245539 0.535993 1.898748
6 2.422005 -0.990822 -0.401664
1 2.973268 -0.044025 -0.318583
1 2.802348 -1.635371 0.401464
1 2.633178 -1.448951 -1.369263
8 0.833599 2.627867 -1.061834
1 1.535412 2.170594 -0.575713
1 0.136557 1.963156 -1.163210
rd_C/rd_C56_072.xyz
6 -0.455329 0.744937 0.002529
6 1.152202 -0.905129 0.002930
6 2.190130 0.102484 -0.003364
6 1.772137 1.443727 -0.007861
1 2.511708 2.239769 -0.013626
7 0.500431 1.794091 -0.005438
7 -0.138114 -0.580517 0.005372
8 -1.656409 1.105601 0.007206
7 1.516946 -2.191678 0.006627

1 0.814241 -2.921560 0.012467
1 2.494862 -2.453216 0.005735
1 3.239319 -0.171284 -0.005051
8 -3.802248 -0.669232 0.023354
1 -2.993781 -0.109992 0.009590
1 -3.506772 -1.544048 -0.264930
rd_C/rd_C56_050.xyz
6 -0.045711 -1.182801 -0.000003
6 -0.368230 1.114548 -0.000004
6 -1.799337 0.910208 0.000004
6 -2.253063 -0.419726 0.000006
1 -3.320720 -0.621404 0.000010
7 -1.437536 -1.457420 0.000004
7 0.472575 0.084129 -0.000009
8 0.720449 -2.168249 -0.000006
7 0.108084 2.362561 -0.000004
1 1.108602 2.525681 -0.000006
1 -0.519502 3.156598 0.000004
1 -2.484913 1.750320 0.000008
8 3.296734 0.203642 0.000011
1 3.567064 -0.725607 0.000020
1 2.308194 0.173007 -0.000038
rd_C/rd_C56_013.xyz
6 -0.456496 -0.732775 -0.000353
6 1.169326 0.899367 -0.000704
6 2.196319 -0.119573 0.000391
6 1.763254 -1.456078 0.000932
1 2.493854 -2.260382 0.001742
7 0.487692 -1.792347 0.000397
7 -0.124475 0.588956 -0.000823
8 -1.661734 -1.080147 -0.000519
7 1.547720 2.181994 -0.001633
1 0.852743 2.919203 -0.002667
1 2.528209 2.433721 -0.001639
1 3.248490 0.142633 0.000735
8 -3.716399 0.818903 0.011947

1 -4.525047 0.295909 -0.080080
1 -2.984176 0.163000 0.003293
rd_C/rd_C46_049.xyz
6 -1.222583 -1.034261 0.001170
6 0.393111 0.802079 -0.008440
6 -0.718017 1.702251 0.001357
6 -2.019521 1.163336 0.010951
1 -2.869999 1.839752 0.018680
7 -2.292036 -0.129213 0.010936
7 0.063196 -0.544335 -0.006078
8 -1.431637 -2.254643 -0.000733
7 1.655103 1.136729 -0.019906
1 0.817244 -1.226734 -0.012593
1 1.758497 2.152797 -0.020016
1 -0.545569 2.771746 0.001457
8 3.764481 -0.763482 -0.036771
1 4.531225 -0.366702 0.400102
1 3.064078 -0.068557 -0.012485
rd_C/rd_C56_033.xyz
6 -0.407325 -1.198699 0.020558
6 -0.041896 1.092313 0.019516
6 -1.468876 1.318880 -0.019406
6 -2.291910 0.180639 -0.042480
1 -3.371303 0.299905 -0.076302
7 -1.817158 -1.050471 -0.023863
7 0.459524 -0.139688 0.038874
8 0.034361 -2.366039 0.041415
7 0.780565 2.145488 0.036926
1 1.784104 2.005694 0.068903
1 0.416541 3.089848 0.024364
1 -1.877376 2.322884 -0.033265
8 3.272900 -0.483462 -0.056860
1 2.288791 -0.395582 -0.021701
1 3.600681 0.427157 -0.071125
rd_C/rd_C56_053.xyz
6 0.454488 -0.748674 0.005240

6 -1.147046 0.907116 0.005021
6 -2.188553 -0.096656 -0.006403
6 -1.775424 -1.439411 -0.014830
1 -2.517924 -2.232672 -0.025804
7 -0.505023 -1.794416 -0.010212
7 0.142035 0.577840 0.009657
8 1.654278 -1.113742 0.015483
7 -1.506943 2.194997 0.011614
1 -0.801289 2.922018 0.022355
1 -2.483863 2.460207 0.009966
1 -3.236739 0.180865 -0.009444
8 3.785995 0.681258 0.044085
1 2.990299 0.104231 0.022246
1 3.556054 1.442021 -0.507449
rd_C/rd_C56_097.xyz
6 1.240602 1.025005 -0.003409
6 -0.307833 -0.689727 -0.000515
6 0.765256 -1.660581 0.001581
6 2.076608 -1.158771 0.002549
1 2.917583 -1.847182 0.005142
7 2.347573 0.132839 0.000385
7 -0.062633 0.619947 -0.002865
8 1.521700 2.245422 -0.007421
7 -1.568100 -1.132164 0.000008
1 -2.359521 -0.478382 0.000529
1 -1.755799 -2.126846 0.003072
1 0.560710 -2.725422 0.002999
8 -3.916099 0.566604 0.047675
1 -4.156155 0.856894 -0.844951
1 -3.717297 1.384821 0.527248
rd_C/rd_C46_024.xyz
6 -0.421559 -0.841992 -0.001145
6 1.153768 1.025900 -0.001984
6 2.204942 0.061874 0.003106
6 1.865022 -1.304684 0.008429
1 2.659310 -2.046367 0.013861

7 0.626391 -1.765555 0.007675
7 -0.131129 0.498368 -0.001717
8 -1.601678 -1.236267 -0.008246
7 1.289053 2.327244 -0.007086
1 -0.915862 1.146504 -0.010466
1 2.285609 2.559975 -0.006933
1 3.237263 0.390158 0.003257
8 -3.627675 0.721666 -0.094787
1 -2.950410 0.014539 -0.040985
1 -3.784336 0.985007 0.822988
rd_C/rd_C46_013.xyz
6 1.143996 1.055064 0.016204
6 -0.385965 -0.853276 0.057486
6 0.763593 -1.700463 -0.011335
6 2.037502 -1.103473 -0.080550
1 2.915552 -1.740374 -0.144993
7 2.250923 0.199874 -0.072495
7 -0.117276 0.507288 0.068650
8 1.299662 2.283174 0.046016
7 -1.630681 -1.246085 0.101777
1 -0.899215 1.154656 0.133445
1 -1.686094 -2.266085 0.090025
1 0.639525 -2.776572 -0.019864
8 -3.742428 0.658411 -0.098414
1 -3.273627 1.499749 -0.194640
1 -3.029540 -0.018710 -0.021156
rd_C/rd_C56_017.xyz
6 -1.647810 -0.715666 -0.143150
6 -0.387125 1.206101 0.071820
6 0.726351 0.423088 0.559072
6 0.537681 -0.965691 0.645387
1 1.347789 -1.598695 0.997193
7 -0.595604 -1.553848 0.313308
7 -1.542812 0.642409 -0.266188
8 -2.718006 -1.292204 -0.438790
7 -0.231013 2.531223 -0.036955

1 -0.989641 3.107952 -0.379975
1 0.642945 2.973279 0.217592
1 1.665463 0.890301 0.834362
8 4.074149 -0.517048 -0.439026
1 3.157492 -0.632597 -0.728511
1 4.538229 -1.291700 -0.788060
rd_C/rd_C46_041.xyz
6 -0.510279 -0.347347 -0.126271
6 1.926619 -0.323813 0.056424
6 1.852458 1.100897 0.067334
6 0.586937 1.709965 -0.042535
1 0.521494 2.794618 -0.049662
7 -0.553414 1.049092 -0.140838
7 0.704925 -0.977221 -0.044561
8 -1.557729 -1.015153 -0.186774
7 3.011936 -1.050965 0.126561
1 0.703414 -1.994121 -0.043456
1 3.826059 -0.435948 0.201742
1 2.759697 1.687713 0.147252
8 -4.098442 0.120748 0.177857
1 -4.639580 -0.631352 0.456392
1 -3.200255 -0.250222 0.041219
rd_C/rd_C46_058.xyz
6 -1.129195 1.058837 -0.019095
6 0.384808 -0.862210 -0.055336
6 -0.771712 -1.699605 0.015015
6 -2.040738 -1.092019 0.081696
1 -2.924052 -1.721546 0.146689
7 -2.243422 0.212991 0.069911
7 0.127576 0.500604 -0.069188
8 -1.274710 2.288157 -0.051239
7 1.626258 -1.265511 -0.099385
1 0.915624 1.141104 -0.131632
1 1.673213 -2.285898 -0.084081
1 -0.656605 -2.776692 0.026003
8 3.711216 0.669671 0.071448

1 3.250567 1.442117 0.429518
1 3.017338 -0.028316 0.008786
rd_C/rd_C56_065.xyz
6 -0.482601 -0.439944 -0.060558
6 1.814048 -0.272581 0.024883
6 1.710933 1.170686 0.027339
6 0.416919 1.714368 -0.036084
1 0.283391 2.792655 -0.046177
7 -0.668309 0.966562 -0.082669
7 0.735534 -1.050546 -0.014875
8 -1.528153 -1.132037 -0.086038
7 3.031671 -0.824527 0.064533
1 3.133988 -1.832616 0.058613
1 3.863793 -0.248683 0.096111
1 2.593527 1.798839 0.071278
8 -4.107538 -0.077085 0.079520
1 -3.169594 -0.365304 0.025508
1 -4.067638 0.852485 0.344420
rd_C/rd_C46_011.xyz
6 0.423097 -0.826493 0.008239
6 -1.179018 1.018555 0.005307
6 -2.216337 0.039834 -0.008941
6 -1.856858 -1.321855 -0.009653
1 -2.640395 -2.074874 -0.018015
7 -0.611755 -1.765132 -0.000990
7 0.113115 0.509280 0.015925
8 1.608779 -1.203190 0.010041
7 -1.332583 2.317798 0.010125
1 0.887488 1.169056 0.028357
1 -2.332178 2.536401 0.001174
1 -3.253203 0.353388 -0.018503
8 3.624990 0.768523 -0.094224
1 4.298802 0.550732 0.564987
1 2.962591 0.048776 -0.029667
rd_C/rd_C56_019.xyz
6 -1.962192 -0.306478 -0.000885

6 0.342663 -0.464905 -0.001610
6 0.443862 0.978613 0.002518
6 -0.760243 1.700232 0.005773
1 -0.737934 2.786787 0.009461
7 -1.943534 1.116057 0.004672
7 -0.838395 -1.081310 -0.003535
8 -3.095938 -0.838279 -0.002914
7 1.473952 -1.174755 -0.003614
1 1.425216 -2.186839 -0.006557
1 2.389674 -0.711950 -0.001315
1 1.408240 1.473089 0.003679
8 3.998740 0.232444 0.025618
1 4.435942 0.203848 -0.838657
1 4.627742 -0.182977 0.634320
rd_C/rd_C46_004.xyz
6 1.145062 -1.054713 0.011588
6 -0.386881 0.852084 0.047456
6 0.762658 1.700271 -0.006042
6 2.037744 1.104806 -0.064528
1 2.915892 1.742744 -0.116504
7 2.252157 -0.198397 -0.060277
7 -0.117303 -0.508355 0.053838
8 1.301354 -2.282874 0.035682
7 -1.631872 1.244888 0.082995
1 -0.899004 -1.157303 0.105358
1 -1.687073 2.264945 0.075806
1 0.637876 2.776320 -0.010617
8 -3.742663 -0.658010 -0.094224
1 -3.277097 -1.504117 -0.029035
1 -3.032482 0.022852 -0.023406
rd_C/rd_C46_091.xyz
6 -0.508555 -0.394446 -0.107588
6 1.928158 -0.288440 0.047376
6 1.805906 1.132842 0.055862
6 0.519743 1.698919 -0.040277
1 0.416584 2.780631 -0.046698

7 -0.598404 0.999543 -0.125163
7 0.727963 -0.983126 -0.035355
8 -1.534505 -1.095922 -0.154984
7 3.039190 -0.977121 0.105719
1 0.760403 -1.999479 -0.033470
1 3.831513 -0.332778 0.168637
1 2.693095 1.750756 0.125159
8 -4.093220 -0.012964 0.231256
1 -4.154862 0.829738 -0.239964
1 -3.177696 -0.326109 0.067510
rd_C/rd_C56_023.xyz
6 -0.427324 1.197010 0.000587
6 -0.022710 -1.087603 0.005050
6 -1.446137 -1.338363 0.000391
6 -2.288884 -0.214441 -0.007293
1 -3.366532 -0.352703 -0.013064
7 -1.835274 1.024781 -0.008283
7 0.457807 0.152837 0.005858
8 -0.004973 2.371581 0.003250
7 0.816799 -2.127319 0.008575
1 1.818829 -1.973232 0.012320
1 0.467253 -3.077227 0.009885
1 -1.837623 -2.349201 0.001789
8 3.266861 0.503184 0.022465
1 3.577224 -0.366106 -0.268806
1 2.280753 0.428640 0.016697
rd_C/rd_C46_048.xyz
6 0.408268 -0.910064 -0.021054
6 -1.046374 1.053355 -0.008564
6 -2.156172 0.157752 0.026423
6 -1.902141 -1.227630 0.034265
1 -2.740618 -1.918462 0.060280
7 -0.695220 -1.765194 0.011654
7 0.202506 0.446207 -0.031556
8 1.561869 -1.377700 -0.041303
7 -1.097204 2.360977 -0.021572

1 1.028497 1.042971 -0.055905
1 -2.075466 2.659879 -0.002845
1 -3.165867 0.549811 0.045969
8 3.401728 0.761746 -0.056305
1 3.653526 0.937871 0.861381
1 2.899099 -0.078848 -0.024121
rd_C/rd_C46_061.xyz
6 0.098632 1.207721 0.012669
6 0.397702 -1.211850 0.010284
6 1.806857 -0.981791 -0.013350
6 2.271248 0.347304 -0.025302
1 3.342027 0.532880 -0.044588
7 1.486041 1.410246 -0.014782
7 -0.391993 -0.073201 0.028269
8 -0.672648 2.181578 0.022806
7 -0.193573 -2.381368 0.014914
1 -1.418357 -0.180179 0.041249
1 0.519024 -3.114973 -0.000169
1 2.491365 -1.821661 -0.025166
8 -3.267936 -0.167717 0.074318
1 -3.635859 -0.955435 -0.353322
1 -3.523482 0.570430 -0.499615
rd_U/rd_U4_adc.xyz
6 1.178123 1.039036 -0.010795
6 -0.419989 -0.816298 -0.046206
6 0.734020 -1.699879 0.009356
6 2.023676 -1.137990 0.066041
1 2.885687 -1.796942 0.118151
7 2.267662 0.160360 0.059770
1 -0.876189 1.196001 -0.099224
7 -0.110369 0.526154 -0.050038
8 1.359924 2.257201 -0.036749
8 -1.591488 -1.221713 -0.084900
1 0.570688 -2.770479 0.013894
8 -3.752947 0.637700 -0.017465
1 -3.014230 -0.003359 -0.009709

1 -3.885895 0.874464 0.911297
rd_U/rd_U4_aci.xyz
6 1.209024 1.028490 0.002631
6 -0.424029 -0.796840 0.011100
6 0.714571 -1.701549 -0.000907
6 2.015625 -1.163787 -0.014685
1 2.866515 -1.839017 -0.026791
7 2.283817 0.129858 -0.013796
1 -0.842396 1.224737 0.023762
7 -0.089365 0.539367 0.012336
8 1.411732 2.243587 0.008325
8 -1.603269 -1.180699 0.019037
1 0.532106 -2.769082 -0.001505
8 -3.734953 0.716924 -0.030327
1 -4.563957 0.225646 0.058062
1 -3.032668 0.036765 -0.008413
rd_U/rd_U4_abs.xyz
6 0.382276 0.793449 -0.000011
6 -1.934321 -0.008751 0.000008
6 -1.379216 -1.355756 -0.000005
6 0.019178 -1.524362 -0.000023
1 0.436303 -2.527522 -0.000039
7 0.876246 -0.520742 -0.000017
1 -1.311561 1.962506 -0.000020
7 -0.986787 0.997520 -0.000003
8 1.160944 1.747112 -0.000015
8 -3.146291 0.235800 0.000028
1 -2.059343 -2.198741 0.000012
8 3.783350 -0.477839 0.000030
1 2.802647 -0.508593 0.000021
1 3.994213 0.466852 0.000012
rd_U/rd_U4_aam.xyz
6 -0.386889 -0.825058 -0.025777
6 1.160313 1.073591 -0.007055
6 2.230933 0.088568 0.030855
6 1.902286 -1.280806 0.031241

1 2.701469 -2.016316 0.055620
7 0.666761 -1.747066 0.004454
1 -0.901237 1.170341 -0.072675
7 -0.110479 0.529274 -0.039692
8 -1.556035 -1.225511 -0.041027
8 1.343987 2.296441 -0.012781
1 3.256915 0.434119 0.057470
8 -3.651226 0.718754 0.063934
1 -4.500642 0.275524 -0.072050
1 -2.984152 0.005633 0.021714
rd_U/rd_U4_abt.xyz
6 1.935471 -0.266806 0.044394
6 -0.502902 -0.448971 -0.086622
6 -0.525794 1.006152 -0.100575
6 0.691078 1.710029 -0.023868
1 0.674228 2.796175 -0.020643
7 1.877755 1.133766 0.048061
1 0.839559 -2.012997 -0.001276
7 0.757529 -0.998136 -0.003990
8 3.021775 -0.846510 0.086152
8 -1.518704 -1.158534 -0.138432
1 -1.481665 1.511645 -0.160299
8 -4.047290 0.168144 0.060739
1 -3.190140 -0.300040 0.014310
1 -4.522329 -0.251426 0.791769
rd_U/rd_U4_adt.xyz
6 -1.140499 1.047471 -0.013518
6 0.416988 -0.842663 -0.062659
6 -0.755637 -1.700612 0.006689
6 -2.031883 -1.110662 0.081775
1 -2.907171 -1.750521 0.147809
7 -2.247668 0.192580 0.076416
1 0.914715 1.160374 -0.127681
7 0.135734 0.506551 -0.070128
8 -1.296205 2.269259 -0.041265
8 1.579396 -1.272624 -0.107229

1 -0.615529 -2.774482 0.010453
8 3.735502 0.593333 0.120827
1 3.013499 -0.060949 0.039094
1 3.294655 1.450715 0.033919
rd_U/rd_U4_aap.xyz
6 -0.388150 -0.838710 0.030016
6 1.136008 1.078770 0.006719
6 2.218284 0.106778 -0.035892
6 1.906257 -1.266472 -0.035135
1 2.714022 -1.992426 -0.062433
7 0.676637 -1.747433 -0.003579
1 -0.926386 1.151213 0.080977
7 -0.128175 0.519288 0.044799
8 -1.551710 -1.254744 0.047456
8 1.305163 2.303742 0.011890
1 3.239917 0.464671 -0.066799
8 -3.729541 0.588402 -0.062134
1 -3.295556 1.450346 0.012839
1 -2.996926 -0.058171 -0.025070
rd_U/rd_U4_abv.xyz
6 -0.487136 -0.393893 -0.111347
6 1.954254 -0.305611 0.057025
6 1.814616 1.143510 0.051992
6 0.528738 1.707848 -0.056708
1 0.423621 2.788886 -0.073053
7 -0.585179 1.002894 -0.140792
1 0.786617 -2.008999 -0.017915
7 0.756322 -0.991365 -0.024742
8 -1.505326 -1.092185 -0.159728
8 3.038646 -0.895920 0.126955
1 2.706788 1.753427 0.122816
8 -4.123614 -0.025220 0.158192
1 -3.197675 -0.317228 0.039691
1 -4.057827 0.898683 0.438072
rd_U/rd_U4_abz.xyz
6 1.935949 -0.271141 0.043134

6 -0.503150 -0.445464 -0.084219
6 -0.521160 1.009624 -0.102090
6 0.698144 1.709713 -0.029964
1 0.684904 2.795907 -0.030537
7 1.882944 1.129639 0.042081
1 0.834254 -2.013846 0.003128
7 0.755593 -0.998723 -0.002596
8 3.020376 -0.854267 0.086182
8 -1.521661 -1.151319 -0.131865
1 -1.475210 1.518554 -0.162643
8 -4.033163 0.198172 0.140378
1 -4.709387 -0.493646 0.163274
1 -3.187441 -0.280470 0.031663
rd_U/rd_U4_adq.xyz
6 0.300391 0.906813 -0.061419
6 -1.921674 -0.124617 0.047455
6 -1.238700 -1.408975 -0.037226
6 0.167129 -1.435240 -0.120317
1 0.683831 -2.389489 -0.173547
7 0.919428 -0.351192 -0.131890
1 -1.499047 1.896317 0.083684
7 -1.078905 0.970444 0.032306
8 0.980334 1.932889 -0.079361
8 -3.149192 -0.000607 0.127343
1 -1.831150 -2.315447 -0.027042
8 3.802746 -0.372280 0.124260
1 2.825767 -0.373543 0.026251
1 4.022946 -1.260497 0.438848
rd_U/rd_U4_ads.xyz
6 0.385158 -0.848961 -0.038049
6 -1.120070 1.083342 -0.013817
6 -2.211065 0.122045 0.046633
6 -1.912218 -1.254204 0.052378
1 -2.726578 -1.972109 0.092737
7 -0.687623 -1.747160 0.011787
1 0.942610 1.134495 -0.110108

7 0.138272 0.511373 -0.059229
8 1.544840 -1.275564 -0.063878
8 -1.277902 2.309767 -0.027503
1 -3.228902 0.490015 0.084668
8 3.700901 0.597567 0.038598
1 3.283151 1.394829 0.394980
1 2.981649 -0.064212 0.009221
rd_U/rd_U4_adp.xyz
6 -1.333387 0.008869 -0.403390
6 1.027514 0.663430 -0.496297
6 0.872632 1.093302 0.886588
6 -0.383157 0.957218 1.509767
1 -0.500326 1.285066 2.538786
7 -1.452647 0.449200 0.923408
1 -0.081188 -0.162022 -2.029399
7 -0.129130 0.171091 -1.068703
8 -2.293951 -0.510695 -0.974906
8 2.091385 0.725269 -1.125076
1 1.730801 1.502637 1.405128
8 1.044671 -2.615307 0.728114
1 0.579405 -1.867222 1.129748
1 1.505300 -2.231538 -0.032267
rd_U/rd_U4_aao.xyz
6 1.930037 -0.246209 0.041727
6 -0.504639 -0.476616 -0.080576
6 -0.556543 0.978073 -0.099101
6 0.646938 1.705675 -0.034910
1 0.609603 2.791283 -0.039149
7 1.845076 1.153109 0.033710
1 0.867814 -2.013309 0.008303
7 0.766582 -1.000221 -0.001564
8 3.027258 -0.804524 0.088132
8 -1.505677 -1.207179 -0.126109
1 -1.522453 1.465128 -0.155806
8 -4.057224 0.049357 0.139480
1 -3.183258 -0.377444 0.036891

1 -3.862933 0.997356 0.149865
rd_U/rd_U4_aac.xyz
6 -0.295274 0.914641 -0.066861
6 1.919427 -0.131714 0.052893
6 1.228845 -1.411218 -0.041851
6 -0.176484 -1.427854 -0.136486
1 -0.698803 -2.378506 -0.198635
7 -0.921743 -0.338977 -0.149119
1 1.509336 1.891715 0.095397
7 1.083847 0.968815 0.035409
8 -0.968203 1.945333 -0.082709
8 3.147068 -0.015921 0.142550
1 1.815307 -2.321564 -0.030756
8 -3.806971 -0.424547 0.087267
1 -3.965136 -0.918382 0.904298
1 -2.829672 -0.384186 0.002626
rd_U/rd_U4_abl.xyz
6 -0.838111 1.299545 0.000447
6 -1.410590 -1.085182 -0.000205
6 0.022861 -1.338036 -0.000210
6 0.914427 -0.248074 0.000109
1 1.984906 -0.434148 0.000142
7 0.535578 1.018282 0.000433
1 -2.736378 0.497962 0.000143
7 -1.748174 0.253698 0.000135
8 -1.236290 2.466334 0.000749
8 -2.270369 -1.975462 -0.000487
1 0.369267 -2.364213 -0.000463
8 4.362211 -0.211941 -0.007844
1 4.829213 -1.058521 0.047593
1 5.065225 0.454102 0.008430
rd_U/rd_U4_aal.xyz
6 -0.307045 0.900715 -0.077066
6 1.920619 -0.116112 0.056697
6 1.246960 -1.405025 -0.032078
6 -0.157464 -1.440283 -0.134724

1 -0.667086 -2.397919 -0.194372
7 -0.916565 -0.361226 -0.158722
1 1.484257 1.901951 0.085797
7 1.071017 0.973362 0.028304
8 -0.993440 1.922466 -0.096307
8 3.146308 0.016153 0.150748
1 1.844781 -2.307788 -0.011130
8 -3.800246 -0.472831 0.086153
1 -2.824756 -0.416306 -0.008979
1 -3.957749 -0.426952 1.039879
rd_U/rd_U4_acx.xyz
6 1.579551 -0.785080 -0.111859
6 0.363645 1.337166 0.062037
6 -0.769514 0.549587 0.525147
6 -0.635701 -0.848431 0.631245
1 -1.481721 -1.435079 0.976224
7 0.467013 -1.511964 0.334834
1 2.315578 1.086105 -0.562344
7 1.488498 0.592050 -0.233559
8 2.630410 -1.365783 -0.392464
8 0.345342 2.568227 -0.063062
1 -1.690875 1.059703 0.776873
8 -3.874987 -0.545857 -0.449569
1 -4.553984 0.109924 -0.234230
1 -4.311579 -1.153399 -1.064112
rd_U/rd_U4_acw.xyz
6 -0.886502 -0.835868 -0.381978
6 1.405279 0.033272 -0.364824
6 1.346730 -0.087258 1.084796
6 0.178120 -0.593986 1.684177
1 0.139992 -0.696677 2.764900
7 -0.896191 -0.970747 1.013805
1 0.229690 -0.301397 -2.028477
7 0.262988 -0.391240 -1.015194
8 -1.889230 -1.117308 -1.041502
8 2.382543 0.473123 -0.982994

1 2.207680 0.216804 1.667447
8 -1.459637 2.552567 0.180159
1 -0.686100 2.068104 0.503850
1 -1.990011 1.883055 -0.276328
rd_U/rd_U4_acj.xyz
6 1.780006 -0.630204 0.003234
6 0.230822 1.271049 -0.002514
6 -0.840969 0.286139 -0.013774
6 -0.513122 -1.083145 -0.014989
1 -1.311918 -1.820120 -0.022398
7 0.722152 -1.551278 -0.007090
1 2.293329 1.364993 0.015164
7 1.500303 0.726835 0.006027
8 2.948015 -1.024113 0.010327
8 0.045765 2.494563 0.000080
1 -1.866795 0.631960 -0.022202
8 -4.240567 -0.406300 0.001416
1 -5.078251 -0.889073 0.053867
1 -3.559676 -1.092904 0.056683
rd_U/rd_U4_aax.xyz
6 -1.372693 -0.070083 -0.370818
6 0.918037 0.798152 -0.456454
6 0.786941 1.039069 0.973574
6 -0.423382 0.714016 1.615144
1 -0.522938 0.900797 2.680707
7 -1.472187 0.192831 1.003798
1 -0.177799 0.057747 -2.042102
7 -0.215114 0.275521 -1.048353
8 -2.310127 -0.596969 -0.973415
8 1.945330 1.031168 -1.105608
1 1.628361 1.459059 1.510661
8 1.464233 -2.515653 0.456263
1 1.904450 -1.958840 -0.202492
1 0.730118 -1.972514 0.778517
p_5fC/p_5fC_14a_aaa.xyz
6 1.201944 1.160306 -0.009697

6 -1.215554 0.782187 0.003163
6 -0.986596 -0.637934 -0.003968
6 0.323824 -1.067361 -0.016310
1 0.575507 -2.123436 -0.022394
7 1.359972 -0.216358 -0.019775
7 -0.118270 1.591969 0.000554
8 2.145234 1.939751 -0.009582
7 -2.418407 1.313986 0.012161
1 -2.555466 2.319882 0.016723
1 -3.220153 0.686473 0.014573
6 -2.062329 -1.617602 0.001013
1 -1.738540 -2.670684 -0.005444
8 -3.263534 -1.328956 0.012238
1 2.332307 -0.596561 -0.023006
1 -0.243172 2.603682 0.005900
8 3.822295 -1.472572 -0.049654
1 4.244889 -1.461557 0.822721
1 3.641868 -2.408333 -0.228881
p_5fC/p_5fC_14a_aas.xyz
6 -1.234903 1.049011 -0.016167
6 1.198144 0.795105 0.011338
6 1.041740 -0.634021 -0.014748
6 -0.245773 -1.130670 -0.043061
1 -0.440396 -2.198565 -0.061427
7 -1.323780 -0.333364 -0.045174
7 0.060650 1.546758 0.012807
8 -2.216132 1.780675 -0.015538
7 2.370155 1.392238 0.033796
1 2.448835 2.404161 0.048771
1 3.206831 0.812425 0.031545
6 2.165392 -1.558034 -0.011637
1 1.893260 -2.625595 -0.032378
8 3.350480 -1.210707 0.013568
1 -2.286005 -0.743604 -0.044731
1 0.133664 2.563392 0.029623
8 -3.851813 -1.443272 -0.032872

1 -4.019824 -1.876416 0.818544
1 -4.493428 -0.717138 -0.075569
p_5fC/p_5fC_14a_abl.xyz
6 1.211956 1.125420 -0.008815
6 -1.211564 0.786679 0.007233
6 -1.005623 -0.636639 -0.012362
6 0.297791 -1.087403 -0.033545
1 0.531215 -2.147551 -0.048630
7 1.347659 -0.253250 -0.033435
7 -0.100850 1.578339 0.009201
8 2.167885 1.889550 -0.003971
7 -2.405722 1.338058 0.023433
1 -2.526012 2.346041 0.036224
1 -3.217863 0.724070 0.021909
6 -2.096884 -1.599232 -0.009961
1 -1.789071 -2.657028 -0.025776
8 -3.293294 -1.292098 0.009296
1 2.315575 -0.645387 -0.034072
1 -0.209847 2.591822 0.022871
8 3.787782 -1.548877 -0.027499
1 4.017199 -1.788734 0.883516
1 4.518158 -0.986810 -0.328351
p_5fC/p_5fC_14a_aao.xyz
6 1.207342 1.142549 -0.013817
6 -1.213390 0.785394 0.008621
6 -0.996860 -0.636364 -0.013966
6 0.309648 -1.077061 -0.041005
1 0.551252 -2.135345 -0.058932
7 1.353099 -0.234981 -0.042720
7 -0.108858 1.585567 0.008656
8 2.157736 1.913473 -0.008881
7 -2.411726 1.327216 0.029348
1 -2.540330 2.334123 0.044346
1 -3.218783 0.706528 0.029494
6 -2.080765 -1.607202 -0.009167
1 -1.765341 -2.662694 -0.028251

8 -3.279358 -1.308983 0.015503
1 2.322767 -0.621627 -0.046800
1 -0.225240 2.598185 0.025029
8 3.802180 -1.524187 -0.053995
1 4.523298 -0.981646 0.300511
1 3.724459 -2.268472 0.562607
p_5fC/p_5fC_14a_aba.xyz
6 1.203341 1.151960 -0.005661
6 -1.215512 0.781948 0.001983
6 -0.991110 -0.638872 -0.003725
6 0.317951 -1.072775 -0.011492
1 0.565237 -2.129842 -0.015796
7 1.357207 -0.225458 -0.012972
7 -0.115486 1.587983 0.001004
8 2.148762 1.928739 -0.005102
7 -2.416688 1.317649 0.008159
1 -2.550473 2.323993 0.011984
1 -3.220501 0.692792 0.009094
6 -2.070145 -1.614948 -0.001191
1 -1.749789 -2.669085 -0.006019
8 -3.270421 -1.322361 0.006182
1 2.329449 -0.606951 -0.013849
1 -0.237076 2.600110 0.005176
8 3.847454 -1.430190 -0.001128
1 3.864465 -2.121108 -0.681414
1 3.949948 -1.904504 0.838385
p_5fC/p_5fC_14a_aap.xyz
6 -0.487072 -1.956014 -0.001656
6 -0.080452 0.459181 -0.002143
6 1.340776 0.213176 0.002092
6 1.761838 -1.096404 0.002220
1 2.814618 -1.358953 0.003903
7 0.892526 -2.123003 0.000004
7 -0.901444 -0.635456 -0.002716
8 -1.267532 -2.897150 -0.002016
7 -0.611579 1.656923 -0.005274

1 -1.638232 1.784566 -0.013157
1 0.022077 2.453022 -0.003941
6 2.332758 1.279709 0.004707
1 3.382529 0.944922 0.007200
8 2.056025 2.482867 0.004434
1 1.228928 -3.083235 -0.000238
1 -1.911709 -0.500470 -0.002063
8 -3.436290 1.731429 -0.093146
1 -3.837208 2.529010 0.284345
1 -3.742237 1.006832 0.474358
p_5fC/p_5fC_14a_aaf.xyz
6 -1.288320 0.864171 -0.010861
6 0.574149 -0.717210 -0.012638
6 1.502725 0.381874 0.004976
6 0.980852 1.654980 0.015144
1 1.617136 2.533652 0.028847
7 -0.345606 1.879756 0.007726
7 -0.756573 -0.413299 -0.016626
8 -2.493852 1.080960 -0.021014
7 0.950576 -1.974879 -0.024706
1 0.273155 -2.730684 -0.038811
1 1.947585 -2.182484 -0.020005
6 2.950084 0.204126 0.013825
1 3.536759 1.136616 0.028561
8 3.511343 -0.894091 0.006246
1 -0.706557 2.831676 0.013946
1 -1.442371 -1.169159 -0.031290
8 -4.141706 -1.307303 -0.048656
1 -3.680158 -0.448469 -0.030605
1 -3.807550 -1.776358 0.729329
p_5fC/p_5fC_14a_abm.xyz
6 -2.413595 -0.523932 0.014781
6 0.038594 -0.520020 -0.054884
6 0.030276 0.920423 -0.012692
6 -1.193948 1.547062 0.054669
1 -1.278572 2.627914 0.093227

7 -2.350299 0.861192 0.077932
7 -1.177593 -1.144934 -0.056477
8 -3.465769 -1.145667 0.021880
7 1.123792 -1.257657 -0.089130
1 1.050405 -2.270561 -0.112551
1 2.063617 -0.852455 -0.069245
6 1.231168 1.746383 -0.035880
1 1.045317 2.833132 -0.004326
8 2.379270 1.305160 -0.089105
1 -3.240444 1.352241 0.126073
1 -1.214594 -2.163143 -0.090705
8 4.055803 -1.156843 0.029069
1 4.208400 -0.209679 0.165270
1 4.285179 -1.568347 0.875263
p_5fC/p_5fC_14a_aaj.xyz
6 2.648759 -0.165144 -0.073105
6 0.456225 0.915342 0.033903
6 -0.173720 -0.378666 0.101894
6 0.643801 -1.487524 0.087005
1 0.242693 -2.494583 0.133947
7 1.980517 -1.381645 0.008523
7 1.816304 0.943269 -0.055794
8 3.863686 -0.076920 -0.154690
7 -0.215936 2.043950 0.052132
1 0.253405 2.942930 0.001120
1 -1.231592 2.001163 0.108633
6 -1.613530 -0.561205 0.158347
1 -1.958854 -1.605539 0.185095
8 -2.425933 0.371200 0.168861
1 2.562609 -2.216751 -0.003939
1 2.295827 1.841830 -0.109078
8 -5.139659 -0.323707 -0.246293
1 -5.186048 -1.289939 -0.269233
1 -4.198195 -0.119533 -0.071852
p_5fC/p_5fC_14a_aav.xyz
6 1.279402 0.540728 -0.016290

6 -1.147023 0.815980 0.003018
6 -1.305642 -0.615012 -0.001267
6 -0.163460 -1.382273 -0.011560
1 -0.202299 -2.466754 -0.015288
7 1.063308 -0.828914 -0.017967
7 0.126670 1.304763 -0.007110
8 2.396516 1.042951 -0.023091
7 -2.161761 1.648977 0.015858
1 -2.023690 2.654818 0.019199
1 -3.103550 1.261350 0.021983
6 -2.606829 -1.273046 0.004952
1 -2.575869 -2.374178 0.000193
8 -3.684782 -0.673456 0.014666
1 1.895165 -1.417291 -0.025048
1 0.278116 2.313595 -0.005687
8 4.676739 -0.706180 -0.062085
1 5.150218 -0.600275 0.775373
1 3.937931 -0.069830 -0.015222
p_5fC/p_5fC_14a_abo.xyz
6 -2.649110 -0.156642 0.081038
6 -0.451522 0.912633 -0.037356
6 0.171255 -0.384685 -0.107251
6 -0.651886 -1.489353 -0.087008
1 -0.256351 -2.498532 -0.135327
7 -1.987610 -1.376654 -0.001864
7 -1.811084 0.947428 0.058591
8 -3.863212 -0.061940 0.167689
7 0.226377 2.037866 -0.059167
1 -0.238404 2.939124 -0.006932
1 1.241355 1.990107 -0.123010
6 1.609492 -0.575341 -0.175580
1 1.948857 -1.621586 -0.200236
8 2.426913 0.352547 -0.199108
1 -2.573968 -2.208695 0.014114
1 -2.286099 1.848282 0.113052
8 5.122271 -0.440959 0.179451

1 5.317430 -0.187683 1.092825
1 4.196246 -0.158347 0.035280
p_5fC/p_5fC_14a_abc.xyz
6 2.472198 -0.317499 0.201164
6 0.108248 -0.641655 -0.342733
6 -0.118818 0.771375 -0.179519
6 0.959934 1.553930 0.170231
1 0.864256 2.625096 0.315267
7 2.185055 1.034385 0.351480
7 1.379135 -1.095606 -0.149089
8 3.589471 -0.782076 0.363752
7 -0.839156 -1.492523 -0.665627
1 -0.646423 -2.484143 -0.769011
1 -1.783555 -1.141491 -0.806871
6 -1.413680 1.402655 -0.371645
1 -1.433667 2.492597 -0.216870
8 -2.441785 0.795489 -0.690363
1 2.966342 1.633248 0.610756
1 1.578840 -2.089705 -0.258884
8 -4.156518 -0.790978 1.062875
1 -3.601223 -0.237582 0.487704
1 -4.996446 -0.314128 1.125459
p_5fC/p_5fC_14a_abb.xyz
6 1.784880 0.406832 -0.516126
6 -0.527570 0.896835 0.110210
6 -0.951044 -0.336492 -0.498954
6 0.017121 -1.123852 -1.079195
1 -0.224121 -2.072049 -1.548614
7 1.311747 -0.762859 -1.096142
7 0.797741 1.208473 0.035619
8 2.967856 0.711753 -0.501214
7 -1.353035 1.718506 0.718230
1 -1.023350 2.578572 1.145991
1 -2.340125 1.470894 0.755205
6 -2.334487 -0.792518 -0.500446
1 -2.508296 -1.760176 -0.998336

8 -3.268596 -0.168902 0.011010
1 2.010948 -1.374426 -1.511426
1 1.136517 2.064984 0.473293
8 1.114525 -1.475815 2.224306
1 1.216309 -1.733513 3.152020
1 1.993267 -1.164244 1.962167
p_5fC/p_5fC_14a_abi.xyz
6 -2.224030 0.152986 0.093658
6 0.074006 0.588504 -0.622589
6 0.434895 -0.761265 -0.274030
6 -0.553352 -1.573989 0.233056
1 -0.357775 -2.602852 0.516807
7 -1.813601 -1.137927 0.403206
7 -1.220593 0.963574 -0.414869
8 -3.369981 0.544077 0.253660
7 0.925181 1.450911 -1.129543
1 0.648278 2.402482 -1.350935
1 1.895029 1.161649 -1.243600
6 1.789147 -1.282843 -0.402738
1 1.917832 -2.334276 -0.100298
8 2.747800 -0.621036 -0.810419
1 -2.528049 -1.760872 0.773764
1 -1.508710 1.915115 -0.641916
8 2.301481 1.077758 2.123055
1 1.470871 0.580043 2.106006
1 2.667224 0.956051 1.234103
p_5fC/p_5fC_14a_abn.xyz
6 1.284869 0.568464 -0.006980
6 -1.145071 0.817195 -0.002252
6 -1.287635 -0.615557 -0.000477
6 -0.137086 -1.370178 -0.005025
1 -0.163191 -2.455037 -0.005202
7 1.083721 -0.803563 -0.009566
7 0.123558 1.319464 -0.005754
8 2.396088 1.083309 -0.006185
7 -2.169227 1.638988 -0.000739

1 -2.041988 2.646278 -0.001397
1 -3.106557 1.240666 0.003122
6 -2.581052 -1.288614 0.007138
1 -2.536953 -2.389388 0.009446
8 -3.665896 -0.701604 0.011066
1 1.921625 -1.383397 -0.012928
1 0.264244 2.329888 -0.004972
8 4.686826 -0.660910 0.050331
1 4.309717 -1.481949 -0.297294
1 3.946432 -0.025501 0.025520
p_5fC/p_5fC_14a_abd.xyz
6 -2.417902 -0.360252 0.174934
6 -0.045115 -0.591013 -0.384378
6 0.141430 0.819325 -0.155274
6 -0.961278 1.553592 0.223549
1 -0.899067 2.620145 0.413077
7 -2.170796 0.991480 0.382058
7 -1.302447 -1.089283 -0.208148
8 -3.520613 -0.864547 0.317662
7 0.922919 -1.399571 -0.752142
1 0.757188 -2.389391 -0.906649
1 1.856844 -1.019051 -0.881221
6 1.415872 1.502115 -0.314920
1 1.391513 2.587775 -0.131531
8 2.470429 0.943668 -0.634526
1 -2.968809 1.557583 0.662703
1 -1.474875 -2.082340 -0.363556
8 3.973878 -0.882605 1.123876
1 3.529533 -0.213631 0.576574
1 3.472346 -1.694205 0.958665
p_5fC/p_5fC_14a_abp.xyz
6 -2.406348 0.344493 0.180708
6 -0.042332 0.592284 -0.408263
6 0.160833 -0.813417 -0.164518
6 -0.930801 -1.554659 0.232565
1 -0.855817 -2.618534 0.432406

7 -2.144410 -1.003074 0.396740
7 -1.301837 1.080590 -0.220404
8 -3.511994 0.839975 0.331178
7 0.912276 1.405603 -0.799907
1 0.734102 2.391241 -0.966680
1 1.848033 1.032415 -0.937006
6 1.440114 -1.485354 -0.329970
1 1.428595 -2.568915 -0.133592
8 2.485624 -0.920291 -0.667609
1 -2.933746 -1.573972 0.691869
1 -1.485569 2.070378 -0.383306
8 3.894335 0.896285 1.183445
1 3.234344 1.601357 1.251762
1 3.495343 0.256370 0.570301
p_5fC/p_5fC_14_abd.xyz
6 0.976676 1.514226 0.010472
6 0.033653 -0.739477 0.028391
6 -1.295222 -0.189129 0.003134
6 -1.404247 1.184355 -0.013133
1 -2.372196 1.671300 -0.031278
7 -0.329756 1.989373 -0.008227
7 1.079109 0.138289 0.027438
8 1.951303 2.254554 0.011620
7 0.325954 -2.025602 0.052606
1 1.301440 -2.323544 0.058262
1 -0.397220 -2.732522 0.050090
6 -2.513573 -1.005796 -0.008949
1 -2.389096 -2.099652 -0.001019
8 -3.645884 -0.524970 -0.028343
1 -0.445423 3.000394 -0.018875
1 2.036475 -0.255799 0.026509
8 3.234989 -1.632616 -0.042817
1 3.720251 -1.682508 -0.880276
1 3.911639 -1.652900 0.650693
p_5fC/p_5fC_14a_aaz.xyz
6 -1.969277 -0.364783 -0.209009

6 0.352996 -0.935020 0.304381
6 0.801639 0.071352 -0.621530
6 -0.159093 0.797463 -1.287292
1 0.102026 1.576626 -1.995431
7 -1.472083 0.580821 -1.097377
7 -0.993438 -1.103903 0.442069
8 -3.163816 -0.537524 -0.020369
7 1.175957 -1.683344 1.003604
1 0.833966 -2.387847 1.649591
1 2.177031 -1.539785 0.882723
6 2.207729 0.364042 -0.862979
1 2.400256 1.165010 -1.594703
8 3.141426 -0.219691 -0.305791
1 -2.163383 1.139486 -1.593529
1 -1.347367 -1.803022 1.094202
8 0.079617 2.395495 1.717770
1 -0.551230 1.670570 1.599615
1 -0.286134 2.919376 2.445154
p_5fC/p_5fC_14a_abh.xyz
6 2.264328 0.196895 0.134097
6 -0.042303 0.586915 -0.581408
6 -0.371526 -0.775585 -0.250707
6 0.636144 -1.571794 0.245202
1 0.464791 -2.609398 0.512263
7 1.884279 -1.107327 0.426788
7 1.243890 0.988228 -0.371224
8 3.399217 0.614512 0.304742
7 -0.914593 1.436374 -1.073541
1 -0.659882 2.395680 -1.287509
1 -1.876751 1.125614 -1.194106
6 -1.706627 -1.335616 -0.405538
1 -1.802938 -2.400294 -0.140050
8 -2.685179 -0.692484 -0.797536
1 2.611524 -1.716562 0.795371
1 1.510344 1.948908 -0.585610
8 -2.681137 1.044777 1.901954

1 -2.970257 0.705671 1.041205
1 -1.715163 0.990117 1.861110
p_C/p_C96.xyz
7 -1.861098 -0.806416 0.002093
6 -0.545120 -1.217301 -0.004195
7 0.379496 -0.187983 -0.005800
6 0.062879 1.137511 -0.002113
6 -1.311351 1.503269 0.005817
6 -2.235352 0.504734 0.007439
7 1.045176 2.026533 -0.006292
8 -0.209988 -2.401823 -0.008110
1 -2.564857 -1.538769 0.003282
1 -1.600351 2.545253 0.009649
1 -3.302236 0.691393 0.012825
1 0.826334 3.015174 -0.003441
1 2.017939 1.739974 -0.013992
1 1.383425 -0.463841 -0.007912
8 3.142687 -0.649748 -0.003769
1 3.490314 -0.282335 0.823609
1 3.516486 -0.088493 -0.700685
p_C/p_C12.xyz
7 1.767245 -0.962104 0.012619
6 0.416168 -1.236580 -0.004722
7 -0.398104 -0.119014 -0.014994
6 0.051280 1.166544 -0.010595
6 1.455832 1.392783 0.006183
6 2.272239 0.304749 0.017888
7 -0.836160 2.149357 -0.022376
8 -0.038867 -2.380738 -0.010050
1 2.392906 -1.762467 0.020761
1 1.848057 2.400341 0.009679
1 3.352375 0.382418 0.031584
1 -0.521713 3.111864 -0.020947
1 -1.832758 1.962358 -0.038488
1 -1.424564 -0.298465 -0.021195
8 -3.176167 -0.328697 -0.047230

1 -3.507341 -0.139482 0.844116
1 -3.490680 -1.223738 -0.246541
p_C/p_C57.xyz
7 -1.783967 -0.938841 -0.014450
6 -0.437560 -1.234568 0.007591
7 0.394272 -0.129752 0.019591
6 -0.034572 1.162746 0.011887
6 -1.435348 1.410904 -0.009789
6 -2.268806 0.335858 -0.022913
7 0.867913 2.131809 0.025321
8 -0.000262 -2.385549 0.015350
1 -2.422052 -1.729251 -0.023776
1 -1.811351 2.424601 -0.015860
1 -3.347499 0.430819 -0.040341
1 0.567858 3.098932 0.021096
1 1.861554 1.931159 0.045526
1 1.417131 -0.325695 0.026786
8 3.178191 -0.381880 0.061061
1 3.498906 -1.289139 -0.055378
1 3.522211 0.105858 -0.703228
p_C/p_C03.xyz
7 2.167200 -0.234303 0.007165
6 1.095194 -1.097217 -0.006854
7 -0.140838 -0.469284 -0.014492
6 -0.338949 0.885017 -0.006690
6 0.814225 1.719399 0.006745
6 2.036465 1.124550 0.013879
7 -1.575158 1.344848 -0.011523
8 1.209110 -2.321550 -0.012658
1 3.089591 -0.659997 0.013753
1 0.705388 2.795101 0.012466
1 2.962262 1.686240 0.025089
1 -1.727599 2.346182 -0.004796
1 -2.380096 0.702071 -0.013106
1 -0.947219 -1.092684 -0.029265
8 -3.506118 -0.741611 -0.019835

1 -3.809708 -0.964542 0.872996
1 -4.316599 -0.586409 -0.527732
p_C/p_C02.xyz
7 0.596827 -1.713231 -0.003096
6 -0.467068 -0.844440 0.014972
7 -0.114201 0.495113 0.021882
6 1.166979 0.967578 0.004132
6 2.224689 0.017567 -0.015954
6 1.898099 -1.302579 -0.018137
7 1.370088 2.272813 0.007185
8 -1.642380 -1.213930 0.024905
1 0.371548 -2.704268 -0.007878
1 3.253334 0.350161 -0.029779
1 2.640843 -2.090866 -0.032487
1 2.317707 2.631175 -0.004926
1 0.601499 2.934997 0.021187
1 -0.894630 1.150006 0.041374
8 -3.683211 0.818184 -0.042963
1 -3.043146 0.081336 -0.018201
1 -4.547629 0.401809 0.083290
p_C/p_C07.xyz
7 -1.827087 1.080355 0.012462
6 -1.981268 -0.286767 0.011773
7 -0.788854 -0.995270 -0.004069
6 0.461451 -0.438842 -0.022040
6 0.547449 0.984000 -0.024188
6 -0.608784 1.697586 -0.005951
7 1.524104 -1.219910 -0.035081
8 -3.075521 -0.847596 0.024797
1 -2.680617 1.631143 0.024654
1 1.513377 1.468883 -0.038853
1 -0.630789 2.780415 -0.004650
1 2.463827 -0.796141 -0.036113
1 1.429108 -2.229636 -0.033129
1 -0.889789 -2.009072 -0.002398
8 4.042378 0.114539 -0.030672

1 4.351017 0.208714 0.883296
1 3.838788 1.018053 -0.316563
p_C/p_C13.xyz
7 1.876498 1.051923 0.004513
6 1.988374 -0.319208 -0.000113
7 0.774621 -0.990673 -0.004024
6 -0.457385 -0.395107 -0.002967
6 -0.499996 1.029295 0.000845
6 0.677873 1.706916 0.004638
7 -1.542919 -1.143393 -0.005624
8 3.064594 -0.914174 -0.000580
1 2.746807 1.575770 0.007308
1 -1.451360 1.542077 0.001317
1 0.733555 2.788457 0.007938
1 -2.472285 -0.696536 -0.002792
1 -1.477686 -2.155493 -0.008525
1 0.843477 -2.006966 -0.006407
8 -4.038087 0.214537 0.028207
1 -4.476949 0.192436 -0.835623
1 -4.668222 -0.199021 0.637292
p_C/p_C45.xyz
7 2.173151 0.223324 0.013509
6 1.106969 1.092754 -0.007722
7 -0.133126 0.472531 -0.019764
6 -0.341000 -0.880207 -0.011985
6 0.807098 -1.721666 0.011906
6 2.033132 -1.134660 0.024352
7 -1.579826 -1.332193 -0.026321
8 1.227760 2.316382 -0.016112
1 3.098199 0.643190 0.022885
1 0.691663 -2.796650 0.020434
1 2.955170 -1.702306 0.042732
1 -1.738332 -2.332671 -0.018937
1 -2.382836 -0.687076 -0.034488
1 -0.932134 1.103828 -0.039317
8 -3.585311 0.711547 -0.070220

1 -3.254960 1.414744 0.509979
1 -4.434947 0.450539 0.316100
p_C/p_C43.xyz
7 -1.834772 -1.076354 -0.008997
6 -1.983063 0.291413 -0.005531
7 -0.787553 0.994774 0.002542
6 0.460498 0.432882 0.009572
6 0.540329 -0.990290 0.008693
6 -0.619096 -1.698877 -0.001350
7 1.526254 1.209708 0.016055
8 -3.074958 0.856957 -0.009786
1 -2.690744 -1.623416 -0.015250
1 1.503946 -1.479878 0.014913
1 -0.645763 -2.781594 -0.003852
1 2.464966 0.783798 0.017271
1 1.434682 2.219772 0.017694
1 -0.883904 2.009027 0.003839
8 4.047055 -0.116454 0.060680
1 3.838077 -1.048066 0.227712
1 4.482452 -0.111323 -0.804989
p_C/p_C67.xyz
7 -2.142934 0.270774 0.005089
6 -1.049461 1.106402 -0.001506
7 0.170236 0.447182 -0.005296
6 0.334149 -0.911469 -0.005136
6 -0.839503 -1.716610 0.002966
6 -2.046299 -1.090907 0.007959
7 1.558922 -1.401330 -0.012331
8 -1.131611 2.333262 -0.003908
1 -3.054685 0.718962 0.007865
1 -0.757585 -2.794731 0.004927
1 -2.985901 -1.629191 0.014000
1 1.687542 -2.406027 -0.011474
1 2.375716 -0.775475 -0.017085
1 0.991649 1.050599 -0.009023
8 3.562865 0.650221 -0.003554

1 3.342645 1.291857 -0.695915
1 3.453707 1.135274 0.828463
p_C/p_C49.xyz
7 0.627170 -1.712484 0.004990
6 -0.462103 -0.876278 -0.009033
7 -0.150642 0.473746 -0.009717
6 1.116604 0.983711 -0.000568
6 2.202748 0.065774 0.012332
6 1.915630 -1.263538 0.014662
7 1.279987 2.294607 -0.004587
8 -1.625320 -1.283153 -0.020669
1 0.430503 -2.709590 0.006621
1 3.221178 0.428643 0.019867
1 2.681465 -2.029544 0.024012
1 2.216045 2.682382 0.000645
1 0.491787 2.933199 -0.016550
1 -0.950631 1.106375 -0.020938
8 -3.620991 0.772338 -0.080965
1 -3.012291 0.010264 -0.029741
1 -4.040445 0.815692 0.790004
p_C/p_C62.xyz
7 1.828053 1.078221 0.005412
6 1.981308 -0.288977 0.015516
7 0.788513 -0.996835 0.001744
6 -0.460895 -0.439017 -0.024304
6 -0.546332 0.983720 -0.035823
6 0.610285 1.696609 -0.020087
7 -1.525407 -1.217072 -0.035653
8 3.075198 -0.850399 0.035193
1 2.682039 1.628320 0.015445
1 -1.512668 1.467541 -0.056868
1 0.633501 2.779385 -0.026240
1 -2.462342 -0.786955 -0.041950
1 -1.434647 -2.227079 -0.025510
1 0.888236 -2.010711 0.009858
8 -3.991413 0.194074 -0.053991

1 -3.864262 0.934718 0.558421
1 -4.744449 -0.298828 0.304886
p_C/p_C42.xyz
7 -0.605797 -1.712922 -0.001863
6 0.466456 -0.854628 0.010702
7 0.127404 0.488529 0.019674
6 -1.149578 0.972993 0.005749
6 -2.216666 0.033331 -0.009775
6 -1.903049 -1.289909 -0.012189
7 -1.340377 2.280084 0.007891
8 1.637867 -1.236927 0.014230
1 -0.389495 -2.705957 -0.007540
1 -3.242067 0.375898 -0.020293
1 -2.653456 -2.070975 -0.023307
1 -2.284450 2.647747 -0.001851
1 -0.565438 2.934886 0.018139
1 0.914268 1.136293 0.035089
8 3.660313 0.796960 -0.096124
1 3.029188 0.054155 -0.036556
1 4.354413 0.587139 0.544628
p_C/p_C84.xyz
7 1.057875 -0.971533 0.765248
6 1.028085 -0.633191 -0.568911
7 -0.219886 -0.232043 -1.027103
6 -1.335613 -0.103517 -0.252151
6 -1.227931 -0.432683 1.127351
6 -0.021649 -0.858566 1.590709
7 -2.459988 0.316078 -0.806593
8 2.008246 -0.688249 -1.306906
1 1.954954 -1.272286 1.136108
1 -2.087671 -0.341670 1.776625
1 0.145932 -1.129907 2.625782
1 -3.293173 0.412166 -0.238115
1 -2.516107 0.550193 -1.791770
1 -0.256319 0.020384 -2.013437
8 1.127303 2.530460 0.408603

1 0.476485 2.024860 0.917320
1 1.188150 3.378795 0.871057
p_C/p_C35.xyz
7 -0.639424 0.891550 -0.097741
6 -0.542885 -0.478476 -0.085057
7 0.752236 -0.962157 -0.006602
6 1.877508 -0.188576 0.039437
6 1.709822 1.223774 0.030560
6 0.444885 1.718250 -0.041337
7 3.060287 -0.776985 0.089390
8 -1.516360 -1.232507 -0.135625
1 -1.578238 1.279118 -0.150580
1 2.573038 1.873563 0.074189
1 0.228796 2.779385 -0.055076
1 3.899939 -0.210696 0.126293
1 3.155621 -1.786996 0.093986
1 0.836430 -1.977626 -0.002350
8 -4.073622 0.067313 0.054450
1 -3.240942 -0.441378 0.011491
1 -4.302457 0.089498 0.994493
p_C/p_C15.xyz
7 0.632335 -1.712380 0.006836
6 -0.461088 -0.881775 -0.011965
7 -0.156355 0.469815 -0.015080
6 1.108221 0.986260 -0.002737
6 2.199014 0.073853 0.015837
6 1.918503 -1.256905 0.019741
7 1.264554 2.298042 -0.009325
8 -1.622224 -1.294557 -0.025723
1 0.440729 -2.710464 0.010215
1 3.215579 0.441878 0.026238
1 2.688075 -2.019100 0.032989
1 2.198529 2.690817 -0.001935
1 0.473188 2.932545 -0.025818
1 -0.959589 1.098482 -0.031978
8 -3.619869 0.760484 -0.076812

1 -3.010303 -0.001436 -0.032410
1 -3.881103 0.922927 0.840708
p_C/p_C19.xyz
7 0.609976 0.922670 -0.074304
6 0.542591 -0.449174 -0.067403
7 -0.743218 -0.960304 -0.008951
6 -1.884664 -0.210659 0.029697
6 -1.747102 1.204758 0.026823
6 -0.492157 1.726058 -0.028590
7 -3.054584 -0.824529 0.067874
8 1.532575 -1.181694 -0.105844
1 1.540047 1.331313 -0.114094
1 -2.623529 1.836995 0.062423
1 -0.298344 2.791524 -0.036491
1 -3.907273 -0.277414 0.095581
1 -3.127986 -1.836188 0.064915
1 -0.805923 -1.977268 -0.009863
8 4.090116 0.116729 0.121793
1 3.256785 -0.385628 0.036393
1 4.787463 -0.554369 0.118047
p_C/p_C99.xyz
7 -0.632479 -1.713239 0.005262
6 0.462945 -0.884968 0.008176
7 0.160658 0.467305 0.011607
6 -1.102845 0.986410 -0.001133
6 -2.195593 0.076230 -0.005905
6 -1.917714 -1.255112 -0.002309
7 -1.256018 2.298579 -0.008348
8 1.623192 -1.300141 0.008313
1 -0.443065 -2.711745 0.003518
1 -3.211449 0.446270 -0.014059
1 -2.688912 -2.015783 -0.006202
1 -2.189100 2.693473 -0.016862
1 -0.463214 2.931453 -0.005531
1 0.964673 1.094893 0.012286
8 3.645022 0.732006 -0.094453

1 3.480137 1.220355 0.725101
1 3.019332 -0.017698 -0.061750
p_C/p_C32.xyz
7 1.800069 1.094966 0.002329
6 1.976627 -0.269510 0.004315
7 0.795796 -0.997493 -0.000317
6 -0.463038 -0.461510 -0.007857
6 -0.572738 0.959734 -0.008018
6 0.571564 1.692236 -0.003175
7 -1.514553 -1.257543 -0.014222
8 3.079964 -0.812326 0.009730
1 2.644542 1.659737 0.005508
1 -1.548049 1.425684 -0.011270
1 0.575897 2.775282 -0.003324
1 -2.457241 -0.840546 -0.017682
1 -1.407846 -2.266004 -0.013743
1 0.913667 -2.009417 0.000989
8 -4.008640 0.117645 -0.002459
1 -4.010121 0.602865 0.836796
1 -3.925114 0.804638 -0.681567
p_C/p_C92.xyz
7 1.057839 0.971658 0.765133
6 1.028068 0.633135 -0.568982
7 -0.219884 0.231882 -1.027129
6 -1.335618 0.103449 -0.252170
6 -1.227967 0.432842 1.127278
6 -0.021697 0.858807 1.590593
7 -2.459977 -0.316264 -0.806553
8 2.008234 0.688141 -1.306975
1 1.954904 1.272503 1.135957
1 -2.087722 0.341929 1.776546
1 0.145862 1.130322 2.625625
1 -3.293162 -0.412289 -0.238068
1 -2.516072 -0.550555 -1.791689
1 -0.256292 -0.020715 -2.013420
8 1.127373 -2.530429 0.408890

1 0.476602 -2.024685 0.917526
1 1.188459 -3.378541 0.871723
rc_1m5caC/rc_1m5caCacq.xyz
6 1.679200 0.680198 -0.005333
6 -0.161553 -0.907843 -0.006030
6 -1.095006 0.190786 -0.058023
6 -0.580320 1.490510 -0.061223
1 -1.247421 2.345498 -0.080219
7 0.726858 1.735785 -0.059435
7 1.158448 -0.594893 0.026801
8 2.873659 0.905952 0.009869
7 -0.528673 -2.167055 0.005690
1 0.183203 -2.901946 0.043760
1 -1.510510 -2.418748 -0.057220
6 -2.571856 -0.016733 -0.027894
8 -3.182001 -0.708003 -0.854345
8 -3.049100 0.580366 0.993879
1 1.836924 -1.380787 0.062332
6 1.267411 3.106789 -0.130169
1 1.881225 3.291239 0.752160
1 1.878235 3.194673 -1.029748
1 0.431536 3.801763 -0.165966
8 2.175193 -3.153332 0.112416
1 2.587506 -3.492130 0.921396
1 2.693609 -3.518530 -0.620416
rc_1m5caC/rc_1m5caCaco.xyz
6 -1.591564 0.730373 0.005644
6 0.156478 -0.949037 0.014440
6 1.149917 0.097404 0.058221
6 0.706675 1.422923 0.064004
1 1.418912 2.240903 0.079614
7 -0.585462 1.735952 0.070476
7 -1.143069 -0.572710 -0.024687
8 -2.771534 1.022992 -0.018989
7 0.471092 -2.224574 0.017913
1 -0.248029 -2.942456 -0.025520

1 1.441052 -2.518165 0.086144
6 2.612996 -0.189964 0.013502
8 3.188671 -0.923087 0.829097
8 3.117040 0.389667 -1.005055
1 -1.885444 -1.321234 -0.062249
6 -1.054922 3.132067 0.150541
1 -1.618720 3.369606 -0.752703
1 -1.698924 3.232997 1.024967
1 -0.186471 3.780775 0.239540
8 -2.895793 -2.668849 -0.116075
1 -2.631742 -3.302146 0.569748
1 -2.773119 -3.139338 -0.955398
rc_1m5caC/rc_1m5caCack.xyz
6 -2.192106 -0.815638 -0.019224
6 0.251144 -0.985814 -0.011288
6 0.324858 0.455316 0.060794
6 -0.869987 1.180390 0.059600
1 -0.852415 2.264513 0.082743
7 -2.063965 0.595463 0.048565
7 -1.003127 -1.519247 -0.051490
8 -3.278459 -1.358599 -0.044893
7 1.298771 -1.769462 -0.035132
1 1.169900 -2.777792 -0.087581
1 2.280020 -1.416056 -0.015563
6 1.612381 1.220358 0.037058
8 2.397332 1.299044 0.988741
8 1.723036 1.735737 -1.122239
1 -1.115471 -2.533066 -0.099992
6 -3.319168 1.368839 0.118081
1 -3.918308 1.150010 -0.766614
1 -3.862452 1.069586 1.015613
1 -3.069401 2.426843 0.158030
8 3.983235 -1.028415 0.046220
1 3.964929 -0.130376 0.416894
1 4.337562 -0.923772 -0.849885
rc_1m5caC/rc_1m5caCaar.xyz

6 2.197229 0.805268 0.002180
6 -0.243631 0.993035 0.026979
6 -0.326964 -0.448190 0.086358
6 0.860196 -1.183321 0.030569
1 0.832515 -2.267521 0.021025
7 2.058159 -0.606781 0.003840
7 1.013852 1.519298 0.000554
8 3.287888 1.339992 -0.005363
7 -1.288805 1.779691 0.002995
1 -1.159094 2.788223 -0.040961
1 -2.267941 1.421265 -0.027030
6 -1.624007 -1.195038 0.124481
8 -2.378042 -1.227833 1.103235
8 -1.784086 -1.742896 -1.014391
1 1.133357 2.533213 -0.022964
6 3.308520 -1.390635 -0.020604
1 3.878976 -1.110932 -0.907186
1 3.885814 -1.162512 0.876553
1 3.051613 -2.447283 -0.049848
8 -3.959473 0.998740 -0.155662
1 -4.007353 0.122804 0.261488
1 -4.188678 0.846544 -1.085118
rc_1m5caC/rc_1m5caCabl.xyz
6 -1.279935 -1.433142 -0.029984
6 0.812086 -0.171382 -0.029707
6 0.049259 1.050428 0.080859
6 -1.344168 0.960168 0.126354
1 -1.947330 1.858546 0.195943
7 -1.984250 -0.205344 0.095722
7 0.096349 -1.328602 -0.094212
8 -1.860449 -2.499117 -0.074410
7 2.117626 -0.204840 -0.063912
1 2.634230 -1.110992 -0.113566
1 2.639246 0.663330 0.025433
6 0.692107 2.395635 0.032218
8 1.622342 2.737297 0.775376

8 0.167323 3.067467 -0.918107
1 0.585606 -2.221057 -0.173257
6 -3.456912 -0.298324 0.161560
1 -3.815477 -0.788485 -0.744516
1 -3.734015 -0.888575 1.035983
1 -3.860728 0.708660 0.238302
8 3.365051 -2.677987 -0.126218
1 2.685211 -3.336957 0.083642
1 4.016424 -2.754557 0.587931
rc_1m5caC/rc_1m5caCacc.xyz
6 -1.276362 1.421641 0.025298
6 0.820035 0.167829 0.013535
6 0.061969 -1.057457 -0.086528
6 -1.332422 -0.972680 -0.121273
1 -1.932492 -1.873807 -0.181687
7 -1.976925 0.190340 -0.089199
7 0.100367 1.322206 0.079000
8 -1.860795 2.485482 0.069195
7 2.126154 0.210148 0.038349
1 2.627597 1.122927 0.093406
1 2.655218 -0.653155 -0.052189
6 0.711611 -2.398956 -0.031028
8 1.638818 -2.742960 -0.776704
8 0.196304 -3.064485 0.929091
1 0.592107 2.214908 0.147204
6 -3.450414 0.278018 -0.141820
1 -3.801524 0.776572 0.762612
1 -3.737341 0.858570 -1.019659
1 -3.852045 -0.730788 -0.204238
8 3.168992 2.776980 0.099546
1 4.018119 2.894271 0.552767
1 3.330140 3.061136 -0.813408
rc_1m5caC/rc_1m5caCacw.xyz
6 -2.202491 -0.789978 0.012971
6 0.235833 -1.000124 0.031941
6 0.333659 0.440562 0.076924

6 -0.845951 1.186353 0.009872
1 -0.808472 2.270215 -0.014490
7 -2.049755 0.620408 -0.009199
7 -1.026143 -1.515199 0.018657
8 -3.298330 -1.314254 0.016038
7 1.274017 -1.795671 0.010697
1 1.138035 -2.803663 -0.022489
1 2.254289 -1.440833 -0.027293
6 1.639378 1.174332 0.126605
8 2.325678 1.296997 1.146140
8 1.875986 1.613504 -1.045028
1 -1.154944 -2.528151 0.008325
6 -3.293135 1.414518 -0.052850
1 -3.858363 1.128547 -0.940903
1 -3.879874 1.201705 0.841852
1 -3.027159 2.468568 -0.092288
8 3.933605 -0.994989 -0.188350
1 4.046619 -0.298847 0.479003
1 4.003757 -0.528360 -1.035978
rc_1m5caC/rc_1m5caCadt.xyz
6 -2.204335 -0.784893 0.023515
6 0.233317 -1.001055 0.036203
6 0.335365 0.439684 0.067697
6 -0.842344 1.187596 -0.008913
1 -0.802846 2.271132 -0.046270
7 -2.047858 0.624441 -0.021884
7 -1.029983 -1.512892 0.036398
8 -3.301588 -1.306141 0.037502
7 1.269009 -1.799646 0.017528
1 1.130616 -2.807552 -0.005120
1 2.249553 -1.446026 -0.029406
6 1.643143 1.171328 0.122984
8 2.265729 1.390545 1.166315
8 1.948211 1.505702 -1.066948
1 -1.161364 -2.525541 0.038895
6 -3.289086 1.420660 -0.083971

1 -3.849439 1.121687 -0.970923
1 -3.881209 1.222656 0.810510
1 -3.020293 2.473307 -0.138295
8 3.920160 -0.997691 -0.201120
1 3.953489 -0.341024 -0.914962
1 4.126870 -0.491199 0.600184
rc_1m5caC/rc_1m5caCabt.xyz
6 2.184922 0.829431 -0.020787
6 -0.260373 0.974146 -0.052919
6 -0.320814 -0.466374 0.038111
6 0.881724 -1.178018 0.076909
1 0.875823 -2.261552 0.121275
7 2.069635 -0.580440 0.081662
7 0.989332 1.518704 -0.091869
8 3.265926 1.383297 -0.041530
7 -1.313272 1.750236 -0.093943
1 -1.188784 2.758460 -0.159151
1 -2.293798 1.396531 -0.054420
6 -1.598254 -1.247103 -0.004466
8 -2.401443 -1.325184 0.932103
8 -1.678588 -1.778875 -1.158775
1 1.091906 2.532462 -0.159230
6 3.330348 -1.339697 0.193655
1 3.940085 -1.142882 -0.689005
1 3.856649 -1.007969 1.089701
1 3.087745 -2.397838 0.263206
8 -3.993257 0.988948 0.002051
1 -3.971348 0.134172 0.463549
1 -4.464560 1.589317 0.599327
rc_1m5caC/rc_1m5caCacy.xyz
6 -2.171984 -0.257358 -0.112023
6 -0.131201 -1.588340 -0.256213
6 0.621930 -0.453670 0.224539
6 -0.072864 0.731611 0.493344
1 0.458977 1.611466 0.838310
7 -1.389302 0.834650 0.349886

7 -1.469632 -1.415407 -0.395859
8 -3.374486 -0.170463 -0.244720
7 0.416864 -2.742376 -0.549900
1 -0.142535 -3.519673 -0.894262
1 1.412870 -2.894035 -0.414597
6 2.109253 -0.479915 0.369211
8 2.692062 -1.265240 1.129005
8 2.637857 0.371519 -0.415903
1 -2.047856 -2.188068 -0.730266
6 -2.114471 2.087102 0.642401
1 -2.645711 2.399892 -0.257404
1 -2.826823 1.902968 1.447607
1 -1.387220 2.839414 0.937983
8 1.805416 3.172695 -0.861714
1 2.576693 3.726537 -1.048558
1 2.165323 2.278765 -0.728605
rc_1m5caC/rc_1m5caCacm.xyz
6 2.052624 0.627415 -0.161607
6 -0.309319 0.896769 -0.710405
6 -0.548624 -0.447273 -0.244447
6 0.539206 -1.196125 0.213822
1 0.401181 -2.219295 0.546271
7 1.767881 -0.691958 0.279715
7 0.971731 1.340158 -0.653549
8 3.172079 1.091990 -0.109116
7 -1.259645 1.676851 -1.165125
1 -1.058825 2.621424 -1.486305
1 -2.228775 1.370098 -1.161758
6 -1.898897 -1.079941 -0.315732
8 -2.904689 -0.617460 0.241123
8 -1.818834 -2.101220 -1.072533
1 1.192370 2.289976 -0.957467
6 2.909160 -1.465563 0.809882
1 3.696883 -1.486682 0.056276
1 3.273872 -0.973997 1.713103
1 2.566055 -2.472976 1.033097

8 -2.284993 1.386433 2.200233
1 -2.517245 0.726379 1.517188
1 -1.458706 1.780083 1.885585
rc_1m5caC/rc_1m5caCacj.xyz
6 1.287704 0.620299 0.105381
6 -1.080415 1.191469 -0.047997
6 -1.402738 -0.209047 0.058460
6 -0.351397 -1.122043 0.155023
1 -0.554693 -2.185674 0.210623
7 0.924696 -0.743022 0.196543
7 0.238751 1.513003 -0.018473
8 2.447222 0.986939 0.134552
7 -1.983903 2.134425 -0.168064
1 -1.715897 3.112746 -0.249410
1 -2.974523 1.909199 -0.148832
6 -2.802368 -0.717794 -0.020924
8 -3.719323 -0.357124 0.723916
8 -2.878026 -1.527966 -1.009055
1 0.519049 2.493228 -0.081179
6 2.029169 -1.717455 0.308898
1 2.629847 -1.668847 -0.600383
1 2.639414 -1.460986 1.175102
1 1.596879 -2.708481 0.426916
8 5.107932 -0.203699 -0.288121
1 4.216929 0.151632 -0.128580
1 5.644013 0.568568 -0.517675
rc_1m5caC/rc_1m5caCaca.xyz
6 2.054358 -0.638906 0.153114
6 -0.306449 -0.887799 0.717657
6 -0.539809 0.454021 0.241439
6 0.551595 1.192936 -0.225210
1 0.419509 2.215592 -0.561500
7 1.776801 0.681008 -0.292394
7 0.971477 -1.340274 0.656404
8 3.170041 -1.111894 0.096043
7 -1.257040 -1.656972 1.189183

1 -1.059815 -2.600774 1.514791
1 -2.224410 -1.344776 1.191539
6 -1.884852 1.098580 0.313980
8 -2.894532 0.644739 -0.243755
8 -1.795811 2.119295 1.069587
1 1.187446 -2.289009 0.966895
6 2.922222 1.446901 -0.824860
1 3.711364 1.461940 -0.072563
1 3.282014 0.953477 -1.729036
1 2.585594 2.456837 -1.046535
8 -2.219355 -1.471432 -2.050825
1 -2.513080 -0.736671 -1.475966
1 -2.692411 -1.343006 -2.885088
rc_1m5caC/rc_1m5caCabj.xyz
6 -2.052104 -0.626952 -0.162049
6 0.309817 -0.896732 -0.710716
6 0.549772 0.446626 -0.243619
6 -0.538134 1.196089 0.213603
1 -0.399947 2.219291 0.545888
7 -1.767143 0.692559 0.278634
7 -0.971359 -1.339900 -0.653765
8 -3.171626 -1.091389 -0.109281
7 1.259577 -1.677089 -1.165875
1 1.058295 -2.621594 -1.486939
1 2.228939 -1.371120 -1.162194
6 1.900363 1.078942 -0.314083
8 2.905600 0.617775 0.244629
8 1.820653 2.099048 -1.072639
1 -1.192097 -2.289726 -0.957574
6 -2.908596 1.466779 0.807515
1 -3.696241 1.486387 0.053786
1 -3.273259 0.976613 1.711516
1 -2.565818 2.474685 1.029004
8 2.283774 -1.396248 2.193849
1 2.519429 -0.729943 1.518120
1 1.399260 -1.695570 1.939060

rc_1m5caC/rc_1m5caCadk.xyz
6 -1.918638 -0.638688 0.051050
6 0.430691 -1.291703 -0.035509
6 0.791024 0.010596 0.470451
6 -0.234745 0.927081 0.728157
1 -0.001812 1.925371 1.082496
7 -1.518645 0.619554 0.573921
7 -0.893436 -1.533455 -0.206930
8 -3.085972 -0.909127 -0.139726
7 1.312768 -2.220085 -0.317185
1 1.028387 -3.127693 -0.679462
1 2.303610 -2.059085 -0.156495
6 2.210878 0.467322 0.567532
8 3.060015 -0.062204 1.292494
8 2.369178 1.434544 -0.250318
1 -1.200789 -2.437630 -0.569130
6 -2.600051 1.574079 0.887624
1 -3.177442 1.761109 -0.018426
1 -3.240793 1.133893 1.652977
1 -2.146367 2.493181 1.250590
8 0.320448 1.271321 -2.723993
1 1.021556 1.430876 -2.073147
1 -0.275476 0.649488 -2.281525
rc_1m5caC/rc_1m5caCacr.xyz
6 -0.707959 1.643856 0.198182
6 1.570326 0.929176 -0.299159
6 1.189768 -0.430769 -0.003871
6 -0.138531 -0.681313 0.350701
1 -0.467225 -1.690674 0.571479
7 -1.050326 0.285075 0.420320
7 0.609939 1.876566 -0.157197
8 -1.523264 2.536571 0.306795
7 2.776280 1.262651 -0.689777
1 3.023829 2.232533 -0.871692
1 3.479308 0.543176 -0.841610
6 2.186444 -1.535486 0.045949

8 3.025047 -1.743618 -0.841943
8 2.074834 -2.157986 1.157467
1 0.832950 2.855663 -0.343934
6 -2.462551 0.011009 0.751057
1 -2.706646 0.499546 1.695628
1 -3.089043 0.416384 -0.044306
1 -2.591173 -1.065747 0.833621
8 -5.346432 -1.762739 -0.932294
1 -4.494764 -1.303925 -0.895239
1 -6.004956 -1.053663 -0.894729
rc_1m5caC/rc_1m5caCabs.xyz
6 2.258645 0.076305 0.060707
6 0.461811 -1.572587 0.076698
6 -0.494483 -0.507817 -0.108373
6 -0.014834 0.803759 -0.195031
1 -0.702753 1.635425 -0.312571
7 1.282236 1.088461 -0.141050
7 1.767710 -1.214104 0.163943
8 3.441187 0.335469 0.135399
7 0.115636 -2.834227 0.157648
1 0.809661 -3.565339 0.296550
1 -0.858049 -3.110401 0.061172
6 -1.967098 -0.756876 -0.106927
8 -2.527271 -1.516707 -0.905731
8 -2.498811 -0.116002 0.860596
1 2.482281 -1.931805 0.295678
6 1.785027 2.470351 -0.266970
1 2.314142 2.736521 0.649197
1 2.467053 2.519310 -1.116849
1 0.933369 3.128309 -0.422455
8 -2.886931 2.756394 0.246871
1 -3.159844 2.734304 -0.681660
1 -2.844732 1.820722 0.509450
rc_1m5caC/rc_1m5caCaaq.xyz
6 1.932744 0.778199 -0.091950
6 -0.405054 1.439309 -0.319655

6 -0.796020 0.219060 0.344584
6 0.211569 -0.662183 0.752353
1 -0.044617 -1.598251 1.236582
7 1.500988 -0.406139 0.563624
7 0.925350 1.635532 -0.499706
8 3.108053 1.013109 -0.277256
7 -1.269103 2.333193 -0.733745
1 -0.967221 3.177530 -1.215176
1 -2.263454 2.207700 -0.561579
6 -2.223895 -0.190798 0.484795
8 -3.072672 0.510723 1.053056
8 -2.408441 -1.306782 -0.101151
1 1.253954 2.482135 -0.967303
6 2.554832 -1.365499 0.949867
1 3.064185 -1.703382 0.045839
1 3.264108 -0.864019 1.608840
1 2.083326 -2.201546 1.461110
8 -0.311281 -2.986751 -1.615920
1 -1.014399 -2.472556 -1.186116
1 0.353151 -3.116634 -0.923203
rc_1m5caC/rc_1m5caCaaa.xyz
6 2.517664 0.358289 -0.064916
6 0.269668 1.306654 -0.037070
6 -0.268887 -0.024316 0.109173
6 0.625545 -1.098719 0.159227
1 0.258265 -2.114050 0.257169
7 1.942203 -0.929758 0.099325
7 1.619623 1.410489 -0.128095
8 3.717922 0.516726 -0.141063
7 -0.482194 2.379745 -0.074835
1 -0.073303 3.304849 -0.186105
1 -1.490225 2.307531 0.037170
6 -1.734565 -0.295795 0.077693
8 -2.559473 0.287128 0.797086
8 -1.964316 -1.147307 -0.843711
1 2.051350 2.331022 -0.224025

6 2.885243 -2.063646 0.177498
1 3.480807 -2.087139 -0.735792
1 3.536203 -1.918451 1.040473
1 2.309563 -2.980148 0.282069
8 -5.201411 -0.495821 -0.028847
1 -4.313624 -0.222544 0.277332
1 -5.026247 -1.025016 -0.820403
rc_1m5caC/rc_1m5caCaas.xyz
6 -2.172990 0.684595 0.133119
6 0.176055 1.068340 -0.403635
6 0.387151 -0.358917 -0.409432
6 -0.700701 -1.190288 -0.126103
1 -0.573638 -2.267087 -0.106488
7 -1.918412 -0.711774 0.110743
7 -1.081937 1.496122 -0.129756
8 -3.278156 1.130082 0.361712
7 1.127470 1.935380 -0.649745
1 0.946864 2.936444 -0.621613
1 2.068491 1.623721 -0.875980
6 1.739733 -0.956612 -0.595050
8 2.471669 -0.709492 -1.560343
8 2.013299 -1.679721 0.423782
1 -1.286185 2.496957 -0.118552
6 -3.079574 -1.591688 0.352197
1 -3.512206 -1.341040 1.321246
1 -3.813428 -1.423650 -0.437416
1 -2.735917 -2.623307 0.340725
8 3.457480 0.612463 1.874400
1 3.524205 1.055968 1.016034
1 3.079591 -0.255339 1.660368
rc_1m5caU/rc_1m5caUack.xyz
6 1.617605 0.678120 0.011652
6 -0.147742 -0.998046 0.000899
6 -1.141102 0.090377 0.013727
6 -0.695976 1.427812 0.012075
1 -1.406677 2.248243 0.010859

7 0.590696 1.716333 0.002273
7 1.165865 -0.609086 0.009468
8 2.784456 1.002742 0.018692
8 -0.475721 -2.189236 -0.018477
1 1.901897 -1.367938 -0.002429
6 -2.618262 -0.168149 0.009917
8 -3.406947 0.764998 0.045325
8 -3.020671 -1.431525 -0.032471
1 -2.242660 -2.045707 -0.045035
6 1.077056 3.102430 -0.045913
1 1.698919 3.283518 0.833028
1 1.684496 3.218459 -0.946053
1 0.220062 3.770769 -0.063239
8 3.020673 -2.595264 -0.077653
1 2.538541 -3.436555 -0.103692
1 3.545693 -2.630501 0.736909
rc_1m5caU/rc_1m5caUabr.xyz
6 1.616341 0.675195 -0.030027
6 -0.150064 -0.999856 -0.016928
6 -1.142557 0.089478 -0.011329
6 -0.696341 1.426532 -0.002584
1 -1.406338 2.247439 0.011572
7 0.590583 1.714130 -0.002749
7 1.163641 -0.611697 -0.035297
8 2.783396 0.998940 -0.043386
8 -0.478927 -2.190878 -0.004899
1 1.900578 -1.369979 -0.034723
6 -2.619933 -0.167579 0.001715
8 -3.407763 0.766732 -0.019128
8 -3.023617 -1.430806 0.036954
1 -2.246528 -2.046252 0.038739
6 1.078335 3.099417 0.051868
1 1.699208 3.206640 0.943863
1 1.686821 3.289243 -0.834640
1 0.221959 3.767786 0.088193
8 3.032288 -2.586324 -0.048738

1 3.547198 -2.578924 0.773086
1 2.559826 -3.433425 -0.042480
rc_1m5caU/rc_1m5caUaaf.xyz
6 1.595560 0.639587 -0.021571
6 -0.182208 -1.024226 -0.009199
6 -1.166232 0.073072 -0.012841
6 -0.710596 1.407244 -0.009771
1 -1.415031 2.233026 -0.002140
7 0.578174 1.685880 -0.007399
7 1.134002 -0.644806 -0.022852
8 2.765427 0.953276 -0.028948
8 -0.520983 -2.212254 0.006462
1 1.877992 -1.394042 -0.006991
6 -2.645730 -0.171940 -0.002090
8 -3.425326 0.769228 -0.025785
8 -3.060675 -1.431383 0.034017
1 -2.289448 -2.053895 0.039685
6 1.076214 3.067861 0.040339
1 1.685525 3.178626 0.939946
1 1.698797 3.243614 -0.839144
1 0.224715 3.743180 0.058476
8 3.155271 -2.466572 0.107027
1 3.144444 -3.120731 -0.609142
1 3.976020 -1.965239 -0.020326
rc_1m5caU/rc_1m5caUaan.xyz
6 1.599048 0.653840 -0.020866
6 -0.170642 -1.017899 -0.009158
6 -1.160377 0.074186 -0.012295
6 -0.711587 1.410370 -0.006285
1 -1.419933 2.232777 0.002053
7 0.575868 1.695067 -0.001632
7 1.144376 -0.632859 -0.022347
8 2.766807 0.975119 -0.031906
8 -0.504212 -2.207351 0.006112
1 1.887814 -1.384204 -0.018493
6 -2.638587 -0.178814 -0.004421

8 -3.423412 0.757927 -0.031215
8 -3.046386 -1.440548 0.032312
1 -2.271153 -2.058268 0.039142
6 1.066804 3.079397 0.050408
1 1.679091 3.189468 0.948023
1 1.684687 3.262694 -0.830877
1 0.211885 3.750173 0.074963
8 3.079847 -2.547950 -0.003741
1 3.761242 -2.322403 -0.656027
1 3.535552 -2.529748 0.852271
rc_1m5caU/rc_1m5caUabk.xyz
6 1.596284 0.633574 -0.024685
6 -0.185604 -1.025672 -0.016423
6 -1.166744 0.074195 -0.011863
6 -0.707586 1.407182 -0.002872
1 -1.409842 2.234741 0.009903
7 0.581901 1.682524 -0.001508
7 1.131407 -0.649513 -0.031887
8 2.767062 0.943922 -0.033850
8 -0.527556 -2.212882 -0.005487
1 1.875033 -1.399983 -0.034589
6 -2.646897 -0.166718 -0.000039
8 -3.423767 0.776849 -0.017168
8 -3.065528 -1.425114 0.030091
1 -2.296260 -2.050037 0.031163
6 1.083630 3.063032 0.050176
1 1.695966 3.168890 0.948309
1 1.703871 3.240559 -0.830626
1 0.233846 3.740383 0.073323
8 3.167396 -2.455846 -0.065708
1 3.953030 -1.950321 0.196702
1 3.071855 -3.144303 0.610783
rc_1m5caU/rc_1m5caUada.xyz
6 -1.625107 -0.680950 -0.030036
6 0.143304 0.992296 -0.015433
6 1.135011 -0.097411 -0.011225

6 0.687558 -1.433970 -0.001017
1 1.396839 -2.255488 0.013634
7 -0.599685 -1.720481 -0.000019
7 -1.170975 0.605269 -0.035068
8 -2.792299 -1.004063 -0.045616
8 0.472631 2.183125 -0.000892
1 -1.901363 1.369107 -0.038589
6 2.612506 0.159028 -0.000862
8 3.399884 -0.775585 -0.024506
8 3.016749 1.422065 0.034559
1 2.239919 2.037838 0.038523
6 -1.088001 -3.105482 0.057437
1 -1.708499 -3.210658 0.949953
1 -1.696735 -3.296965 -0.828526
1 -0.231802 -3.774051 0.094909
8 -2.946693 2.667183 -0.064256
1 -3.656729 2.557080 0.587117
1 -2.440814 3.436751 0.241091
rc_1m5caU/rc_1m5caUaat.xyz
6 1.599730 0.643516 0.028682
6 -0.177573 -1.020258 0.017607
6 -1.162152 0.076356 0.012748
6 -0.706838 1.410469 0.000090
1 -1.411367 2.236030 -0.015790
7 0.581945 1.689480 -0.001388
7 1.138518 -0.640678 0.034610
8 2.769396 0.957872 0.044034
8 -0.515817 -2.208553 0.005318
1 1.880578 -1.393522 0.034430
6 -2.641474 -0.169572 0.002008
8 -3.421735 0.771160 0.020720
8 -3.055484 -1.429495 -0.028541
1 -2.283661 -2.051407 -0.030752
6 1.079343 3.071268 -0.059439
1 1.693808 3.257186 0.823697
1 1.696219 3.173221 -0.954894

1 0.227442 3.745579 -0.091933
8 3.114580 -2.509942 0.047828
1 3.128114 -3.007363 -0.784578
1 3.951888 -2.020352 0.062228
rc_1m5caU/rc_1m5caUacb.xyz
6 -2.072518 -0.654163 -0.150360
6 0.300771 -0.912087 -0.757261
6 0.523285 0.445020 -0.236284
6 -0.565235 1.210716 0.215891
1 -0.429720 2.236909 0.542425
7 -1.784229 0.699664 0.276478
7 -1.007713 -1.353332 -0.652340
8 -3.199158 -1.091371 -0.058801
8 1.187308 -1.608864 -1.236688
1 -1.210600 -2.296916 -0.982490
6 1.880157 1.072053 -0.303512
8 1.953564 2.077300 -1.006763
8 2.888918 0.549906 0.329606
1 2.655949 -0.277297 0.908498
6 -2.931175 1.471580 0.786303
1 -3.714250 1.467151 0.026514
1 -3.298665 0.985034 1.692050
1 -2.600090 2.485363 0.997188
8 2.373130 -1.470243 1.785449
1 2.065691 -2.208515 1.234941
1 1.633485 -1.268589 2.380805
rc_1m5caU/rc_1m5caUadt.xyz
6 -2.002418 0.613803 -0.106168
6 0.362547 1.263606 -0.235377
6 0.709213 -0.166348 -0.259390
6 -0.328356 -1.122459 -0.283890
1 -0.097572 -2.180433 -0.352960
7 -1.596254 -0.768636 -0.217155
7 -0.983825 1.533031 -0.141312
8 -3.175293 0.898033 -0.002993
8 1.200927 2.163893 -0.297403

1 -1.265325 2.512860 -0.096252
6 2.123481 -0.645478 -0.394666
8 2.377136 -1.839612 -0.411728
8 3.075066 0.277129 -0.490929
1 2.688025 1.187802 -0.453318
6 -2.686002 -1.761743 -0.223433
1 -3.228134 -1.686931 0.720596
1 -3.357645 -1.535369 -1.052596
1 -2.246043 -2.748955 -0.340726
8 0.890298 -0.089467 2.231689
1 0.029258 -0.454290 2.495998
1 1.522114 -0.813553 2.376992
rc_1m5caU/rc_1m5caUaci.xyz
6 -2.001202 0.614711 -0.120073
6 0.367104 1.248503 -0.258061
6 0.706385 -0.183342 -0.258582
6 -0.335670 -1.134579 -0.257027
1 -0.109921 -2.194839 -0.302872
7 -1.601336 -0.773420 -0.190198
7 -0.977983 1.527074 -0.178145
8 -3.173063 0.906667 -0.029749
8 1.211141 2.142717 -0.330764
1 -1.254568 2.509027 -0.155344
6 2.116411 -0.673473 -0.391623
8 2.362716 -1.869262 -0.397950
8 3.073667 0.242644 -0.498746
1 2.691400 1.155788 -0.470754
6 -2.695413 -1.761598 -0.174683
1 -3.252237 -1.648913 0.756736
1 -3.353147 -1.563617 -1.022290
1 -2.258664 -2.754506 -0.247900
8 0.829445 0.013478 2.241226
1 0.434803 -0.836026 2.499576
1 1.780635 -0.093782 2.409400
rc_1m5caU/rc_1m5caUadx.xyz
6 2.006594 0.616869 -0.112233

6 -0.359499 1.262965 -0.238842
6 -0.704311 -0.167009 -0.266744
6 0.334505 -1.122853 -0.270726
1 0.104511 -2.181625 -0.327928
7 1.600577 -0.768115 -0.195914
7 0.987046 1.534620 -0.158492
8 3.179592 0.903371 -0.019298
8 -1.201141 2.161823 -0.282672
1 1.267734 2.514961 -0.119930
6 -2.116058 -0.649403 -0.408658
8 -2.364571 -1.843891 -0.455939
8 -3.072984 0.270794 -0.476186
1 -2.688991 1.181835 -0.424226
6 2.690461 -1.761326 -0.181125
1 3.354138 -1.560329 -1.023233
1 3.241380 -1.658089 0.754861
1 2.249520 -2.751607 -0.264053
8 -0.794014 -0.169821 2.238621
1 -1.214219 -1.037938 2.359583
1 -1.512646 0.473591 2.359523
rc_1m5caU/rc_1m5caUabe.xyz
6 -2.006686 -0.613722 -0.109233
6 0.360660 -1.246629 -0.263520
6 0.699864 0.185443 -0.260291
6 -0.342651 1.136172 -0.254713
1 -0.117790 2.196532 -0.302182
7 -1.607818 0.774350 -0.182238
7 -0.983777 -1.525803 -0.176089
8 -3.177739 -0.906263 -0.010167
8 1.204705 -2.140350 -0.342552
1 -1.260048 -2.507811 -0.152437
6 2.109966 0.676660 -0.391166
8 2.356830 1.872235 -0.387570
8 3.066671 -0.239189 -0.507188
1 2.683904 -1.152337 -0.485610
6 -2.702551 1.761834 -0.164296

1 -3.363690 1.561213 -1.008627
1 -3.255328 1.651263 0.769762
1 -2.266831 2.754873 -0.241953
8 0.854433 -0.007510 2.223037
1 1.799492 -0.207025 2.332744
1 0.770656 0.933533 2.451432
rc_1m5caU/rc_1m5caUadu.xyz
6 2.000315 -0.622355 -0.119725
6 -0.364731 -1.283887 -0.204617
6 -0.717256 0.142034 -0.268606
6 0.317117 1.103864 -0.298385
1 0.079641 2.159713 -0.374112
7 1.585027 0.758572 -0.227320
7 0.985468 -1.546485 -0.135403
8 3.175732 -0.899931 -0.031792
8 -1.201356 -2.188529 -0.213291
1 1.271736 -2.524211 -0.076316
6 -2.130619 0.615472 -0.420061
8 -2.380893 1.806429 -0.516711
8 -3.088602 -0.306451 -0.441989
1 -2.706403 -1.215480 -0.363109
6 2.668374 1.759214 -0.233474
1 3.216305 1.681344 0.706769
1 3.335889 1.543808 -1.068777
1 2.220404 2.744002 -0.340480
8 -0.764902 0.454112 2.247980
1 -1.695441 0.250814 2.439934
1 -0.274630 -0.335699 2.530776
rc_1m5caU/rc_1m5caUabu.xyz
6 -1.722515 -0.553205 -0.503575
6 0.623398 -1.215390 -0.278634
6 0.978244 0.210016 -0.178931
6 -0.041126 1.180237 -0.245194
1 0.192854 2.235822 -0.155721
7 -1.302957 0.836495 -0.403067
7 -0.707249 -1.474121 -0.500861

8 -2.894431 -0.822199 -0.623743
8 1.457308 -2.117954 -0.182468
1 -0.992783 -2.452205 -0.560337
6 2.391982 0.674481 0.003733
8 2.648798 1.868856 0.019823
8 3.331134 -0.252744 0.143391
1 2.940590 -1.161102 0.089009
6 -2.385076 1.829062 -0.400543
1 -2.959711 1.719316 -1.322119
1 -3.030992 1.621966 0.456496
1 -1.945315 2.820515 -0.326946
8 -1.470149 -0.168653 2.265710
1 -1.358269 -1.110304 2.462045
1 -2.425658 -0.020273 2.322228
rc_1m5caU/rc_1m5caUaac.xyz
6 2.028506 0.613063 -0.092082
6 -0.337594 1.255851 -0.247942
6 -0.683166 -0.174725 -0.254753
6 0.356002 -1.128944 -0.238368
1 0.127172 -2.188377 -0.286005
7 1.622979 -0.772679 -0.159888
7 1.007800 1.528859 -0.146861
8 3.201261 0.901506 -0.000773
8 -1.176785 2.154524 -0.325284
1 1.287766 2.509956 -0.125046
6 -2.091784 -0.659674 -0.423737
8 -2.338714 -1.853953 -0.469678
8 -3.046039 0.259927 -0.524705
1 -2.663583 1.171495 -0.474074
6 2.712742 -1.765563 -0.138767
1 3.370121 -1.576022 -0.988628
1 3.271342 -1.649164 0.791060
1 2.271645 -2.757042 -0.204832
8 -1.201420 -0.179608 2.176481
1 -0.427370 -0.651096 2.526727
1 -1.067203 0.747780 2.433604

rc_1m5caU/rc_1m5caUaal.xyz
6 -2.002722 -0.610976 -0.111763
6 0.364774 -1.240942 -0.284913
6 0.702785 0.191173 -0.258887
6 -0.341420 1.140738 -0.246228
1 -0.116878 2.201626 -0.282033
7 -1.606730 0.777262 -0.185068
7 -0.979515 -1.521610 -0.192357
8 -3.172280 -0.906088 -0.002432
8 1.208872 -2.133401 -0.379367
1 -1.255293 -2.503953 -0.176363
6 2.112941 0.685958 -0.373220
8 2.357460 1.881570 -0.357554
8 3.072392 -0.226946 -0.490344
1 2.691676 -1.140764 -0.489217
6 -2.703278 1.762894 -0.167219
1 -3.366341 1.557156 -1.008804
1 -3.253152 1.655310 0.768929
1 -2.269563 2.756325 -0.250835
8 0.960280 0.122543 2.221850
1 1.424325 -0.723983 2.337357
1 0.036668 -0.065774 2.459088
rc_1m5caU/rc_1m5caUadv.xyz
6 -2.007334 -0.616875 -0.108201
6 0.359071 -1.260850 -0.239967
6 0.702944 0.169533 -0.263896
6 -0.336677 1.123875 -0.271873
1 -0.107819 2.182717 -0.332260
7 -1.602686 0.768237 -0.194750
7 -0.987141 -1.533664 -0.156915
8 -3.179939 -0.904211 -0.012032
8 1.200907 -2.158936 -0.291266
1 -1.266993 -2.514243 -0.119032
6 2.114197 0.654066 -0.404002
8 2.363123 1.848834 -0.435861
8 3.070118 -0.266032 -0.488200

1 2.685788 -1.177401 -0.442056
6 -2.693094 1.760835 -0.181777
1 -3.357756 1.557199 -1.022471
1 -3.242828 1.660056 0.755183
1 -2.252758 2.751122 -0.268013
8 0.815956 0.125471 2.227147
1 1.674358 -0.315846 2.344656
1 1.000842 1.069883 2.365631
rc_1m5caU/rc_1m5caUadh.xyz
6 -2.005207 0.615092 -0.112834
6 0.361709 1.257912 -0.255203
6 0.704412 -0.173047 -0.259042
6 -0.335996 -1.127332 -0.257416
1 -0.106299 -2.186628 -0.304490
7 -1.602295 -0.770792 -0.191964
7 -0.985188 1.531551 -0.174945
8 -3.177155 0.903950 -0.012731
8 1.203489 2.155619 -0.316233
1 -1.265334 2.512359 -0.145619
6 2.114566 -0.660026 -0.396697
8 2.361460 -1.854540 -0.437997
8 3.073241 0.258635 -0.468954
1 2.690188 1.170671 -0.430881
6 -2.693233 -1.763002 -0.179677
1 -3.245806 -1.661038 0.755397
1 -3.355005 -1.560339 -1.022900
1 -2.252710 -2.753409 -0.263829
8 0.923025 -0.257321 2.227368
1 1.607096 0.423893 2.342924
1 0.090265 0.180840 2.471347
rc_1m5caU/rc_1m5caUach.xyz
6 1.995317 -0.615187 -0.107046
6 -0.369154 -1.271483 -0.224893
6 -0.718208 0.157413 -0.258924
6 0.318366 1.114357 -0.302944
1 0.084894 2.170882 -0.384321

7 1.586924 0.764071 -0.236201
7 0.978299 -1.537134 -0.134068
8 3.168448 -0.896406 0.002816
8 -1.205930 -2.174196 -0.272938
1 1.262035 -2.515701 -0.077242
6 -2.133763 0.634757 -0.382251
8 -2.388851 1.828533 -0.398229
8 -3.085122 -0.288978 -0.471857
1 -2.697617 -1.199094 -0.431962
6 2.674090 1.760007 -0.256988
1 3.212076 1.704870 0.690775
1 3.349998 1.520872 -1.078896
1 2.231451 2.743435 -0.394689
8 -0.870670 0.174465 2.225592
1 -1.008866 1.129792 2.341864
1 0.066580 0.029867 2.441563
rc_1m5caU/rc_1m5caUadd.xyz
6 -1.364425 0.614738 -0.518388
6 0.962914 1.239266 -0.071825
6 1.295411 -0.193034 -0.003742
6 0.278115 -1.147319 -0.207591
1 0.503410 -2.208067 -0.170182
7 -0.967569 -0.783306 -0.446133
7 -0.357586 1.522775 -0.339754
8 -2.521064 0.904339 -0.725300
8 1.799839 2.126850 0.094086
1 -0.631224 2.505766 -0.380486
6 2.687556 -0.684442 0.266029
8 2.915297 -1.884093 0.304765
8 3.636483 0.222515 0.454556
1 3.269145 1.138651 0.389231
6 -2.043453 -1.766196 -0.651452
1 -2.775760 -1.645762 0.150186
1 -2.519795 -1.556911 -1.611183
1 -1.611219 -2.763337 -0.634686
8 -4.484002 0.069648 1.476955

1 -4.704769 -0.858737 1.313538
1 -3.922837 0.319963 0.726130
rc_1m5dhmC/rc_1m5dhmCadu.xyz
6 -2.093501 0.780059 -0.063358
6 0.161798 0.909667 -0.628312
6 0.348956 -0.529892 -0.426583
6 -0.792465 -1.264587 -0.055048
1 -0.733407 -2.335415 0.112334
7 -1.960419 -0.676457 0.105075
7 -1.018929 1.494190 -0.436460
8 -3.210033 1.245568 0.152001
6 1.667414 -1.278383 -0.432737
1 1.489480 -2.325134 -0.695629
7 1.191050 1.656928 -1.012570
1 1.053615 2.651220 -1.160706
1 2.092657 1.228293 -1.203687
8 2.159404 -1.286470 0.888624
1 2.269889 -0.359041 1.234908
8 2.548401 -0.675240 -1.351770
1 3.353966 -1.213745 -1.406142
6 -3.160154 -1.430837 0.490625
1 -3.501514 -1.073426 1.463831
1 -3.940674 -1.252746 -0.250436
1 -2.908323 -2.488534 0.535774
8 2.389299 1.157137 2.034901
1 3.311506 1.343913 2.267424
1 2.142041 1.857871 1.412439
rc_1m5dhmC/rc_1m5dhmCacl.xyz
6 -1.620486 1.221920 0.029673
6 0.557170 0.410844 -0.124687
6 0.092313 -0.981308 -0.163171
6 -1.299569 -1.180975 -0.141483
1 -1.721660 -2.179902 -0.184812
7 -2.130698 -0.159522 -0.075183
7 -0.298659 1.430132 -0.019494
8 -2.475448 2.097217 0.152389

6 0.974838 -2.213882 -0.092895
1 0.422778 -3.085080 -0.455172
7 1.853687 0.675492 -0.193257
1 2.191879 1.648400 -0.128597
1 2.517748 -0.084218 -0.311495
8 1.254978 -2.491376 1.267482
1 1.788801 -1.773670 1.651849
8 2.133196 -1.991470 -0.857601
1 2.622380 -2.826702 -0.929562
6 -3.586099 -0.344394 -0.078762
1 -3.990731 0.041354 0.858833
1 -4.007119 0.223970 -0.909844
1 -3.805491 -1.404500 -0.184708
8 2.918986 3.315167 0.072401
1 2.765387 3.604790 0.984522
1 3.883016 3.303309 -0.024902
rc_1m5dhmC/rc_1m5dhmCabd.xyz
6 2.546202 -0.505100 -0.113000
6 0.341192 -1.243160 -0.003729
6 -0.173564 0.129591 -0.001602
6 0.782633 1.158343 -0.080869
1 0.481016 2.200601 -0.084502
7 2.070925 0.887887 -0.150045
7 1.647101 -1.499945 -0.050669
8 3.765327 -0.657379 -0.147309
6 -1.620638 0.529185 0.216408
1 -1.786809 1.535121 -0.180207
7 -0.510946 -2.260224 0.043562
1 -0.152386 -3.209349 0.046209
1 -1.512857 -2.088092 0.021438
8 -1.818460 0.651421 1.621022
1 -1.722686 -0.221447 2.041694
8 -2.470274 -0.405026 -0.377807
1 -3.383196 -0.015897 -0.459010
6 3.080699 1.952140 -0.220687
1 3.701115 1.902197 0.676260

1 3.705393 1.787320 -1.099518
1 2.571098 2.911444 -0.283858
8 -4.938865 0.620873 -0.609232
1 -5.478687 0.035731 -1.162445
1 -4.912534 1.463242 -1.088511
rc_1m5dhmC/rc_1m5dhmCabv.xyz
6 -2.096463 0.790640 -0.049544
6 0.156151 0.909202 -0.628608
6 0.337294 -0.531415 -0.430050
6 -0.806862 -1.261886 -0.058977
1 -0.752856 -2.333728 0.103404
7 -1.971418 -0.668469 0.106738
7 -1.020546 1.499988 -0.425378
8 -3.208930 1.261038 0.177760
6 1.652626 -1.284956 -0.434053
1 1.471154 -2.331741 -0.694440
7 1.183420 1.650584 -1.024739
1 1.052983 2.647941 -1.156753
1 2.087608 1.222330 -1.203156
8 2.142423 -1.290926 0.888328
1 2.266707 -0.361452 1.225127
8 2.537968 -0.687391 -1.352512
1 3.343133 -1.227184 -1.400041
6 -3.174085 -1.419285 0.489730
1 -3.506370 -1.073461 1.470340
1 -3.958332 -1.224283 -0.242991
1 -2.930026 -2.479412 0.518038
8 2.435959 1.176650 1.963734
1 3.065707 1.106869 2.697466
1 2.848774 1.790636 1.337186
rc_1m5dhmC/rc_1m5dhmCabn.xyz
6 2.079483 0.764786 0.075380
6 -0.180416 0.916201 0.618093
6 -0.380627 -0.521507 0.412877
6 0.758670 -1.268454 0.060768
1 0.692508 -2.340297 -0.097584

7 1.934529 -0.692456 -0.084422
7 1.007553 1.489883 0.431277
8 3.203499 1.217612 -0.129528
6 -1.703949 -1.261542 0.408299
1 -1.534739 -2.306026 0.686090
7 -1.200192 1.671434 1.006175
1 -1.056829 2.666653 1.140988
1 -2.114494 1.257726 1.165725
8 -2.181219 -1.282409 -0.918349
1 -2.256897 -0.357286 -1.281394
8 -2.593182 -0.644161 1.310094
1 -3.399064 -1.181961 1.365274
6 3.138706 -1.464002 -0.416516
1 3.556520 -1.084963 -1.350116
1 3.869195 -1.330397 0.383619
1 2.867203 -2.513262 -0.513615
8 -2.299469 1.167062 -2.060512
1 -2.745954 1.778405 -1.454742
1 -1.378908 1.471671 -2.082503
rc_1m5dhmC/rc_1m5dhmCaax.xyz
6 2.085509 -0.782852 -0.053295
6 -0.171509 -0.909566 -0.614266
6 -0.357964 0.529671 -0.407860
6 0.788608 1.265326 -0.055356
1 0.733615 2.338285 0.099325
7 1.957814 0.677047 0.094494
7 1.008071 -1.496659 -0.415565
8 3.201556 -1.248759 0.165866
6 -1.674357 1.282035 -0.400898
1 -1.495375 2.326907 -0.670795
7 -1.195120 -1.652810 -1.015418
1 -1.061041 -2.649245 -1.150696
1 -2.104257 -1.229668 -1.179684
8 -2.150463 1.297338 0.926399
1 -2.268878 0.368339 1.268498
8 -2.569455 0.678814 -1.306097

1 -3.372695 1.221482 -1.352525
6 3.170099 1.437927 0.421864
1 3.578344 1.068039 1.363362
1 3.902539 1.282857 -0.372534
1 2.911205 2.491887 0.501404
8 -2.448050 -1.172508 1.984194
1 -2.960447 -1.703378 1.355128
1 -1.569374 -1.582878 1.989913
rc_1m5dhmC/rc_1m5dhmCaal.xyz
6 -2.551789 0.504657 -0.042671
6 -0.344518 1.242353 -0.022301
6 0.170343 -0.130046 -0.059537
6 -0.787928 -1.156859 -0.138392
1 -0.486799 -2.198238 -0.184342
7 -2.077933 -0.885277 -0.153726
7 -1.651231 1.499222 -0.002187
8 -3.770964 0.654002 -0.000008
6 1.621018 -0.531449 0.128411
1 1.778777 -1.536941 -0.272859
7 0.509437 2.258801 -0.005483
1 0.151655 3.207788 0.024636
1 1.509446 2.085457 -0.071172
8 1.847536 -0.657163 1.528492
1 1.765976 0.215023 1.953588
8 2.459909 0.403922 -0.480503
1 3.381908 0.029616 -0.521244
6 -3.088643 -1.948036 -0.235805
1 -3.684946 -1.935371 0.678409
1 -3.737388 -1.750808 -1.090448
1 -2.580562 -2.903560 -0.350721
8 4.921416 -0.660913 -0.602381
1 5.601229 0.030791 -0.598665
1 5.064716 -1.155484 0.219569
rc_1m5dhmC/rc_1m5dhmCaae.xyz
6 -2.548214 -0.487177 0.098644
6 -0.351133 -1.251792 0.012420

6 0.180077 0.114572 0.017360
6 -0.764278 1.154498 0.089417
1 -0.449861 2.192888 0.098469
7 -2.056455 0.899964 0.143589
7 -1.660533 -1.492765 0.046173
8 -3.769415 -0.624638 0.117410
6 1.632768 0.498126 -0.189146
1 1.808622 1.499851 0.214066
7 0.489616 -2.278526 -0.028419
1 0.120351 -3.223506 -0.035896
1 1.493161 -2.117860 0.003904
8 1.839467 0.625994 -1.592434
1 1.731686 -0.242967 -2.018407
8 2.469428 -0.448807 0.403173
1 3.384474 -0.064355 0.490142
6 -3.052969 1.977209 0.204850
1 -3.667835 1.933591 -0.696181
1 -3.685527 1.821883 1.079834
1 -2.531469 2.929947 0.269831
8 4.927301 0.576969 0.647023
1 5.019490 1.042352 1.492554
1 5.056764 1.258703 -0.030353
rc_1m5dhmC/rc_1m5dhmCabb.xyz
6 1.586839 -1.263204 0.062305
6 -0.572507 -0.397197 -0.062524
6 -0.069166 0.977293 -0.169195
6 1.327775 1.138768 -0.183019
1 1.774147 2.124253 -0.269617
7 2.132974 0.098286 -0.100060
7 0.258840 -1.435264 0.055512
8 2.420560 -2.158223 0.189381
6 -0.909482 2.238514 -0.130132
1 -0.347780 3.072303 -0.559473
7 -1.877480 -0.627267 -0.086641
1 -2.237842 -1.591979 -0.020890
1 -2.522455 0.146194 -0.220398

8 -1.115589 2.587217 1.227459
1 -1.629929 1.889771 1.670570
8 -2.109107 2.019300 -0.828344
1 -2.583003 2.863159 -0.902743
6 3.592684 0.244319 -0.135277
1 4.005893 -0.141658 0.798220
1 3.981676 -0.342863 -0.968990
1 3.837416 1.297258 -0.257219
8 -3.003093 -3.256621 0.048790
1 -3.469877 -3.382218 0.888762
1 -3.687618 -3.348855 -0.631131
rc_1m5dhmC/rc_1m5dhmCaai.xyz
6 -1.645673 1.168916 0.028038
6 0.557803 0.424263 -0.107446
6 0.135288 -0.979955 -0.164648
6 -1.250052 -1.221479 -0.148815
1 -1.639784 -2.233294 -0.197931
7 -2.112777 -0.227118 -0.078867
7 -0.329928 1.416939 -0.008010
8 -2.527307 2.018865 0.142424
6 1.053533 -2.187204 -0.105459
1 0.528168 -3.068665 -0.481707
7 1.846646 0.728539 -0.152174
1 2.151410 1.712477 -0.077885
1 2.536327 -0.008309 -0.267296
8 1.334860 -2.475279 1.252457
1 1.833682 -1.742142 1.654364
8 2.207876 -1.920745 -0.861828
1 2.720686 -2.740659 -0.943225
6 -3.561612 -0.460199 -0.077957
1 -3.971305 -0.110853 0.871930
1 -4.007833 0.113774 -0.891587
1 -3.746456 -1.524186 -0.209257
8 2.851226 3.373833 0.165604
1 2.256979 3.865783 0.752265
1 2.841580 3.868105 -0.667856

rc_1m5dhmC/rc_1m5dhmCadi.xyz
6 2.243050 0.862765 0.027587
6 -0.083154 0.970647 0.188173
6 -0.197998 -0.491328 0.166141
6 0.989544 -1.204793 -0.080243
1 0.974669 -2.284515 -0.188076
7 2.153449 -0.592972 -0.170108
7 1.110761 1.567418 0.168813
8 3.378345 1.335530 0.034722
6 -1.461180 -1.311847 0.380312
1 -1.172381 -2.337549 0.625525
7 -1.163128 1.737828 0.255613
1 -1.032532 2.744497 0.249651
1 -2.112371 1.374899 0.162879
8 -2.186649 -1.394369 -0.828858
1 -2.717736 -0.570376 -0.957080
8 -2.189640 -0.721475 1.427748
1 -2.872420 -1.349114 1.713812
6 3.400818 -1.333469 -0.398304
1 3.902170 -0.913993 -1.272003
1 4.044936 -1.214185 0.474546
1 3.160532 -2.382624 -0.559591
8 -3.718531 0.888820 -0.845447
1 -4.562484 0.727077 -0.397613
1 -3.934635 1.400072 -1.639602
rc_1m5dhmC/rc_1m5dhmCaaj.xyz
6 -1.905831 0.794480 -0.075702
6 0.381922 1.209023 0.051860
6 0.702857 -0.088108 -0.550110
6 -0.385766 -0.925864 -0.855214
1 -0.222713 -1.916794 -1.267203
7 -1.626083 -0.533893 -0.650057
7 -0.877784 1.602285 0.232511
8 -3.092585 1.071915 0.076965
6 2.091361 -0.669775 -0.729762
1 2.076271 -1.418123 -1.527100

7 1.364349 2.017479 0.431844
1 1.135758 2.905658 0.865840
1 2.333815 1.778932 0.242838
8 2.430899 -1.382521 0.444847
1 2.404076 -0.786413 1.213748
8 2.992657 0.367869 -1.024277
1 3.851104 -0.024411 -1.251556
6 -2.771787 -1.407471 -0.931605
1 -3.302272 -1.593885 0.004312
1 -3.440177 -0.897392 -1.626911
1 -2.407464 -2.339150 -1.359256
8 -0.093079 -1.179822 2.655013
1 -0.618990 -0.413657 2.383208
1 -0.412446 -1.389092 3.544810
rc_1m5dhmC/rc_1m5dhmCach.xyz
6 1.551035 0.245181 -0.192102
6 -0.430145 -0.964755 -0.298879
6 -1.239050 0.248365 -0.143949
6 -0.536916 1.466420 -0.095659
1 -1.067850 2.409739 -0.016369
7 0.779480 1.496616 -0.154893
7 0.901766 -0.921425 -0.294055
8 2.774443 0.374534 -0.133518
6 -2.733536 0.293051 0.107346
1 -3.121372 1.283064 -0.146508
7 -1.031392 -2.137139 -0.450907
1 -0.469405 -2.975243 -0.560847
1 -2.045303 -2.195053 -0.496162
8 -2.945177 0.153672 1.500422
1 -2.634177 -0.720600 1.794264
8 -3.361466 -0.711594 -0.647567
1 -4.323526 -0.597771 -0.582883
6 1.530968 2.758509 -0.145180
1 2.151220 2.789582 0.752461
1 2.173028 2.785603 -1.027290
1 0.825645 3.587023 -0.156876

8 4.455411 -1.822202 0.559674
1 3.839098 -1.110577 0.296749
1 3.883840 -2.558028 0.820901
rc_1m5dhmC/rc_1m5dhmCaaq.xyz
6 -1.373753 0.673269 -0.294247
6 0.895030 1.135111 -0.066600
6 1.245900 -0.283877 -0.175634
6 0.179302 -1.183949 -0.351936
1 0.363589 -2.250500 -0.433679
7 -1.067271 -0.760761 -0.417595
7 -0.371165 1.546348 -0.128141
8 -2.564432 0.979015 -0.361203
6 2.626780 -0.880783 0.021305
1 2.667736 -1.869740 -0.441485
7 1.852795 2.039469 0.096943
1 1.603068 3.020361 0.170964
1 2.831373 1.766137 0.083016
8 2.816185 -1.107565 1.406373
1 2.817212 -0.259436 1.884562
8 3.585732 -0.014730 -0.529945
1 4.448660 -0.459865 -0.522989
6 -2.193279 -1.692193 -0.568037
1 -2.788612 -1.663214 0.347057
1 -2.806582 -1.371456 -1.410505
1 -1.799718 -2.693369 -0.731693
8 -4.963071 -0.056375 0.872394
1 -5.360426 0.732403 1.267744
1 -4.152009 0.265046 0.438500
rc_1m5dhmC/rc_1m5dhmCade.xyz
6 -2.096691 0.670256 0.251913
6 0.182613 1.010243 0.583522
6 0.494638 -0.369096 0.196762
6 -0.590371 -1.182278 -0.176900
1 -0.433555 -2.212425 -0.481333
7 -1.822339 -0.714692 -0.173550
7 -1.069571 1.461110 0.601099

8 -3.278054 1.005475 0.249708
6 1.865617 -1.019493 0.242095
1 1.875983 -1.896541 -0.409783
7 1.162052 1.837294 0.931559
1 0.934086 2.790635 1.194230
1 2.133991 1.552474 0.859730
8 2.099489 -1.513797 1.544684
1 2.141135 -0.776148 2.178512
8 2.827135 -0.074079 -0.166191
1 3.685688 -0.519935 -0.256119
6 -2.967720 -1.536857 -0.581353
1 -3.656030 -1.614688 0.262365
1 -3.474247 -1.042709 -1.412037
1 -2.609283 -2.520347 -0.878222
8 1.735613 0.805633 -2.760435
1 0.883453 0.356794 -2.658513
1 2.225809 0.574396 -1.950938
rc_1m5dhmC/rc_1m5dhmCaar.xyz
6 -2.089012 -0.637267 -0.355495
6 0.198924 -0.823886 -0.746796
6 0.469515 0.449298 -0.071908
6 -0.643145 1.135841 0.448353
1 -0.521907 2.088435 0.954371
7 -1.859742 0.642631 0.337503
7 -1.038126 -1.301825 -0.862575
8 -3.257556 -1.010529 -0.416565
6 1.816707 1.143788 0.020520
1 1.804703 1.858517 0.847299
7 1.204171 -1.524661 -1.259197
1 1.009378 -2.404390 -1.725570
1 2.165749 -1.225712 -1.128578
8 2.012431 1.920084 -1.144511
1 2.086250 1.340463 -1.923001
8 2.820495 0.173821 0.202846
1 3.656254 0.622856 0.411454
6 -3.031136 1.346649 0.873977

1 -3.716600 1.560209 0.051957
1 -3.527961 0.697045 1.596816
1 -2.697836 2.266952 1.348765
8 1.911377 -1.619587 2.296662
1 1.058631 -1.872754 1.914444
1 2.314124 -1.041481 1.622568
rc_1m5dhmC/rc_1m5dhmCaba.xyz
6 1.389242 0.560362 -0.455736
6 -0.838323 1.124886 -0.092304
6 -1.202037 -0.268156 0.178499
6 -0.160833 -1.212308 0.114181
1 -0.354867 -2.264007 0.299087
7 1.072436 -0.850740 -0.178031
7 0.411058 1.474297 -0.397309
8 2.565103 0.805312 -0.726529
6 -2.608773 -0.801661 0.375865
1 -2.566931 -1.762831 0.894667
7 -1.768378 2.068875 -0.031605
1 -1.512347 3.032161 -0.223544
1 -2.718371 1.842949 0.250283
8 -3.153075 -1.088911 -0.899496
1 -3.257145 -0.263340 -1.403965
8 -3.364754 0.137760 1.096343
1 -4.224502 -0.257247 1.314985
6 2.169053 -1.826941 -0.231920
1 2.679522 -1.727878 -1.190212
1 2.870953 -1.607534 0.575053
1 1.755030 -2.826336 -0.114233
8 4.906207 0.168022 0.878832
1 4.120518 0.375967 0.341632
1 5.084497 0.976504 1.380154
rc_1m5dhmC/rc_1m5dhmCabw.xyz
6 2.069318 -0.907290 0.038117
6 -0.152070 -1.584255 -0.124639
6 -0.629270 -0.198594 -0.082915
6 0.354523 0.806545 -0.023831

1 0.077860 1.860404 -0.015297
7 1.635547 0.497997 0.011145
7 1.144583 -1.877739 -0.049296
8 3.280650 -1.093255 0.131515
6 -2.080119 0.237666 0.020604
1 -2.167509 1.287669 -0.270956
7 -1.026508 -2.577523 -0.238731
1 -0.690702 -3.534737 -0.260681
1 -2.017317 -2.385135 -0.352399
8 -2.473808 0.197143 1.380280
1 -2.490809 -0.723831 1.695188
8 -2.864090 -0.589368 -0.802251
1 -3.761414 -0.221416 -0.846643
6 2.672972 1.536557 0.038180
1 3.243519 1.440835 0.963599
1 3.339564 1.384039 -0.812194
1 2.190955 2.510460 -0.019345
8 -0.675174 3.862109 -0.184652
1 -1.475368 3.571147 -0.647171
1 -0.076876 4.154610 -0.888152
rc_1m5dhmC/rc_1m5dhmCacf.xyz
6 2.085299 -0.638101 0.357537
6 -0.202797 -0.808965 0.755174
6 -0.468380 0.456693 0.065389
6 0.647470 1.136984 -0.455945
1 0.530078 2.086818 -0.968044
7 1.862358 0.641377 -0.338419
7 1.031783 -1.293116 0.871950
8 3.251368 -1.019569 0.414786
6 -1.814881 1.150033 -0.046425
1 -1.798666 1.847951 -0.887273
7 -1.210921 -1.494899 1.282037
1 -1.020901 -2.370635 1.757816
1 -2.171126 -1.194611 1.144917
8 -2.016087 1.950519 1.101012
1 -2.085372 1.388411 1.892555

8 -2.818086 0.176822 -0.214527
1 -3.652127 0.621399 -0.438700
6 3.037226 1.341365 -0.872493
1 3.721764 1.552089 -0.048961
1 3.533261 0.690797 -1.594967
1 2.707954 2.263253 -1.347070
8 -1.897473 -1.680741 -2.254681
1 -1.109114 -2.024349 -1.809886
1 -2.299684 -1.078966 -1.601514
rc_1m5dhmC/rc_1m5dhmCabc.xyz
6 -1.385105 0.555034 0.436096
6 0.841147 1.124468 0.073895
6 1.210455 -0.269423 -0.185647
6 0.168879 -1.214128 -0.134133
1 0.364392 -2.265270 -0.320443
7 -1.067268 -0.853455 0.146878
7 -0.409331 1.471544 0.376769
8 -2.559557 0.795708 0.715901
6 2.621558 -0.801675 -0.352007
1 2.591776 -1.767249 -0.863371
7 1.767894 2.071074 0.005774
1 1.508776 3.034925 0.190632
1 2.719662 1.844832 -0.270043
8 3.139912 -1.076263 0.936837
1 3.226205 -0.246619 1.438125
8 3.390530 0.132781 -1.065234
1 4.254748 -0.263039 -1.263870
6 -2.164613 -1.829072 0.189725
1 -2.658516 -1.757823 1.159339
1 -2.880952 -1.583145 -0.596530
1 -1.754571 -2.825074 0.035292
8 -4.995152 0.239958 -0.762645
1 -4.820217 0.489081 -1.681273
1 -4.150161 0.386548 -0.300253
rc_1m5dhmU/rc_1m5dhmUabb.xyz
6 2.169016 0.778392 0.022073

6 -0.248362 0.915074 0.477904
6 -0.320010 -0.545954 0.327812
6 0.850484 -1.246804 -0.011647
1 0.824643 -2.320823 -0.166505
7 2.020864 -0.644405 -0.156390
7 1.017440 1.453171 0.333019
8 3.256226 1.301279 -0.105792
8 -1.219834 1.630241 0.722876
1 1.113102 2.461930 0.450289
6 -1.623535 -1.316172 0.429341
8 -2.496295 -0.837872 1.417292
1 -2.626249 0.120006 1.298484
8 -2.169548 -1.314174 -0.873578
1 -2.497878 -0.401664 -1.115393
1 -1.408544 -2.356249 0.678508
6 3.246924 -1.389915 -0.489896
1 3.666277 -0.980112 -1.409818
1 3.962165 -1.263971 0.324936
1 2.987066 -2.438475 -0.615078
8 -3.150737 1.126280 -1.436956
1 -2.805197 1.484687 -2.268675
1 -2.689105 1.619542 -0.737405
rc_1m5dhmU/rc_1m5dhmUadq.xyz
6 -2.168923 0.786726 0.005490
6 0.247215 0.915560 0.471066
6 0.312921 -0.546803 0.329188
6 -0.862197 -1.246190 0.003211
1 -0.841632 -2.322202 -0.137775
7 -2.030271 -0.640192 -0.144763
7 -1.014202 1.459061 0.310060
8 -3.251850 1.314716 -0.137022
8 1.219115 1.627563 0.722755
1 -1.104708 2.469683 0.414506
6 1.615577 -1.319485 0.417469
8 2.147215 -1.313127 -0.891566
1 2.471558 -0.398693 -1.132739

8 2.499991 -0.845736 1.397325
1 2.634103 0.111317 1.276577
1 1.401596 -2.360026 0.665485
6 -3.262077 -1.385008 -0.458486
1 -3.973553 -1.235139 0.355590
1 -3.681825 -0.994230 -1.386448
1 -3.009628 -2.437916 -0.560170
8 3.059659 1.146006 -1.487865
1 2.704992 1.614154 -0.712753
1 4.022269 1.156792 -1.376011
rc_1m5dhmU/rc_1m5dhmUaal.xyz
6 -1.737223 0.449088 0.002956
6 0.319072 -0.872219 0.047485
6 1.088096 0.382885 0.073660
6 0.393857 1.602954 0.066631
1 0.930243 2.546170 0.084977
7 -0.929345 1.652640 0.033193
7 -1.051226 -0.730460 0.014186
8 -2.948391 0.542008 -0.029038
8 0.856460 -1.980923 0.055386
1 -1.636083 -1.602660 -0.002962
6 2.603039 0.363784 0.052762
8 3.126834 -0.446223 1.075138
1 2.775952 -1.349894 0.977650
8 3.035125 -0.006893 -1.240806
1 2.762257 -0.921049 -1.433920
1 2.985226 1.370776 0.223725
6 -1.665374 2.927423 0.023898
1 -2.278678 2.965969 -0.878186
1 -2.310536 2.961959 0.903919
1 -0.946290 3.743171 0.038935
8 -2.616641 -2.995812 0.008937
1 -2.512164 -3.485346 -0.821637
1 -3.541823 -2.705101 0.014562
rc_1m5dhmU/rc_1m5dhmUaak.xyz
6 2.022298 0.681981 -0.091585

6 -0.387703 1.183431 -0.091931
6 -0.643032 -0.254270 -0.260291
6 0.449395 -1.134768 -0.342767
1 0.289275 -2.200427 -0.473695
7 1.701011 -0.711281 -0.266312
7 0.948909 1.531979 -0.027630
8 3.179917 1.038999 -0.013068
8 -1.280920 2.024363 -0.011494
1 1.172798 2.519593 0.093085
6 -2.059139 -0.769505 -0.439424
8 -2.924254 -0.317139 0.574152
1 -2.935342 0.656896 0.575332
8 -2.498328 -0.443490 -1.742072
1 -2.537492 0.522574 -1.852799
1 -2.056846 -1.857956 -0.382298
6 2.847431 -1.634485 -0.353414
1 3.468645 -1.338515 -1.200293
1 3.424461 -1.560080 0.569587
1 2.465266 -2.643668 -0.488549
8 -0.647648 -0.774506 2.417973
1 -0.264796 0.080038 2.669792
1 -1.581044 -0.583467 2.219977
rc_1m5dhmU/rc_1m5dhmUaau.xyz
6 -2.018720 0.643231 -0.202096
6 0.391105 1.123700 -0.325906
6 0.641804 -0.315967 -0.166331
6 -0.453147 -1.193764 -0.102974
1 -0.298289 -2.265299 -0.023630
7 -1.704349 -0.759675 -0.121699
7 -0.942829 1.485191 -0.310490
8 -3.174623 1.014444 -0.180413
8 1.286629 1.954440 -0.465122
1 -1.163985 2.477427 -0.389592
6 2.054092 -0.868480 -0.215250
8 2.432388 -0.981348 -1.574410
1 2.440298 -0.103035 -1.993486

8 2.960108 -0.123558 0.558124
1 2.917183 0.811307 0.284650
1 2.060050 -1.882144 0.185894
6 -2.855596 -1.677141 -0.032775
1 -3.431007 -1.430832 0.861063
1 -3.476915 -1.539103 -0.918754
1 -2.479662 -2.696398 0.019586
8 0.664220 0.129741 2.444254
1 1.625748 0.102555 2.299337
1 0.429807 -0.772312 2.712783
rc_1m5dhmU/rc_1m5dhmUadv.xyz
6 -1.986445 -0.710469 -0.134596
6 0.421008 -1.198798 -0.236756
6 0.664332 0.048601 0.502807
6 -0.430324 0.842264 0.882165
1 -0.279104 1.772421 1.420888
7 -1.677196 0.493147 0.603638
7 -0.908961 -1.479298 -0.488266
8 -3.138429 -0.984372 -0.395013
8 1.325833 -1.939606 -0.613743
1 -1.121829 -2.331241 -1.007294
6 2.082344 0.524717 0.741790
8 2.826042 -0.411279 1.478913
1 2.841561 -1.261448 1.003887
8 2.664829 0.852036 -0.506025
1 2.793897 0.040970 -1.030056
1 2.068166 1.446731 1.323232
6 -2.826806 1.324320 1.000432
1 -3.374832 1.612174 0.101359
1 -3.476424 0.732177 1.647685
1 -2.453249 2.201651 1.522932
8 0.386785 1.857776 -2.261611
1 1.186828 1.657486 -1.745976
1 -0.147056 1.051893 -2.199481
rc_1m5dhmU/rc_1m5dhmUabo.xyz
6 2.023025 -0.683933 -0.180229

6 -0.382422 -1.181864 -0.280417
6 -0.629159 0.048957 0.484748
6 0.461948 0.844071 0.870595
1 0.309382 1.762243 1.429186
7 1.708687 0.513556 0.567397
7 0.947398 -1.446614 -0.550732
8 3.176728 -0.953661 -0.437157
8 -1.284743 -1.924619 -0.658643
1 1.162116 -2.291379 -1.080609
6 -2.051109 0.474138 0.796765
8 -2.742055 0.748488 -0.405554
1 -2.865567 -0.076666 -0.908471
8 -2.690954 -0.483089 1.603454
1 -2.718237 -1.337099 1.136019
1 -2.039212 1.406454 1.361684
6 2.854179 1.350987 0.962433
1 3.512970 0.760193 1.601650
1 3.394105 1.648433 0.061587
1 2.476877 2.221662 1.493227
8 -0.704282 1.801835 -2.311806
1 -0.077339 2.253223 -1.728707
1 -1.444001 1.558570 -1.727933
rc_1m5dhmU/rc_1m5dhmUadp.xyz
6 2.051338 -0.697102 -0.242463
6 -0.328784 -1.303265 -0.380406
6 -0.636319 -0.123033 0.441609
6 0.414944 0.712503 0.855308
1 0.218624 1.601658 1.445829
7 1.677028 0.455364 0.547238
7 1.014417 -1.493909 -0.648733
8 3.217605 -0.901814 -0.503053
8 -1.189071 -2.070619 -0.805399
1 1.271034 -2.304511 -1.212198
6 -2.073710 0.250175 0.750501
8 -2.655593 0.772107 -0.432008
1 -2.708752 0.077437 -1.112818

8 -2.795244 -0.816500 1.305465
1 -2.768486 -1.575827 0.696045
1 -2.094130 1.060561 1.479782
6 2.781059 1.320912 0.995197
1 3.453947 0.729742 1.619290
1 3.320971 1.678633 0.116735
1 2.360408 2.151258 1.557104
8 -0.666644 2.700400 -1.448447
1 -1.418073 2.134759 -1.194932
1 -1.015242 3.286375 -2.135317
rc_1m5dhmU/rc_1m5dhmUadd.xyz
6 -2.572893 0.443057 0.137669
6 -0.251081 1.242763 -0.014841
6 0.187038 -0.160543 -0.080174
6 -0.779692 -1.178537 -0.035535
1 -0.491280 -2.223648 -0.084584
7 -2.073163 -0.912364 0.068282
7 -1.618157 1.423624 0.093760
8 -3.764552 0.647145 0.227399
8 0.526898 2.192873 -0.055367
1 -1.963122 2.382708 0.137986
6 1.652454 -0.507134 -0.246268
8 2.034739 -0.238724 -1.577980
1 1.943470 0.710992 -1.771743
8 2.452227 0.140616 0.712735
1 2.351940 1.106106 0.626834
1 1.796676 -1.577582 -0.096286
6 -3.084959 -1.981131 0.113238
1 -3.655569 -1.880667 1.037983
1 -3.752313 -1.860825 -0.742205
1 -2.571676 -2.938682 0.074973
8 5.186202 -0.667746 0.499306
1 4.256802 -0.379857 0.587611
1 5.535011 -0.661533 1.401850
rc_1m5dhmU/rc_1m5dhmUaad.xyz
6 1.874532 1.043284 -0.135217

6 -0.557209 1.364093 -0.320505
6 -0.738615 0.047079 0.309273
6 0.394932 -0.694012 0.679357
1 0.290996 -1.666840 1.147459
7 1.623514 -0.246974 0.464673
7 0.758403 1.764314 -0.467509
8 3.013321 1.422444 -0.307937
8 -1.494735 2.068613 -0.687471
1 0.927558 2.675365 -0.893530
6 -2.127143 -0.526315 0.498231
8 -2.936284 0.332516 1.263800
1 -3.010789 1.194516 0.816792
8 -2.669595 -0.838066 -0.767317
1 -2.775673 -0.022742 -1.288508
1 -2.065080 -1.466675 1.045979
6 2.811790 -1.051193 0.793221
1 3.390203 -1.199817 -0.120383
1 3.416749 -0.504051 1.518688
1 2.484361 -2.003061 1.203974
8 0.312557 -3.370186 -1.002235
1 1.194792 -2.970145 -1.012607
1 -0.278367 -2.678113 -1.334888
rc_1m5dhmU/rc_1m5dhmUadf.xyz
6 2.572666 0.439518 -0.142440
6 0.252730 1.244449 0.011263
6 -0.187887 -0.157629 0.084788
6 0.776774 -1.177643 0.042430
1 0.486488 -2.222004 0.096515
7 2.070493 -0.914514 -0.065788
7 1.619773 1.422006 -0.103443
8 3.764626 0.640937 -0.234350
8 -0.523364 2.196194 0.049948
1 1.966640 2.380152 -0.152671
6 -1.653416 -0.501121 0.255822
8 -2.031137 -0.229572 1.588201
1 -1.937963 0.720382 1.779805

8 -2.454804 0.146264 -0.701931
1 -2.351634 1.111711 -0.618955
1 -1.800088 -1.571616 0.108326
6 3.080115 -1.985341 -0.109795
1 3.647014 -1.890112 -1.037403
1 3.751257 -1.862510 0.742288
1 2.565249 -2.941761 -0.065008
8 -5.192817 -0.649843 -0.516475
1 -4.257812 -0.375805 -0.589472
1 -5.446928 -0.906109 -1.414360
rc_1m5dhmU/rc_1m5dhmUadm.xyz
6 -2.006849 -0.724617 0.065080
6 0.402361 -1.209355 0.177267
6 0.655382 0.126296 -0.378768
6 -0.437470 0.962670 -0.667575
1 -0.277486 1.960177 -1.063544
7 -1.688195 0.572168 -0.481727
7 -0.931817 -1.530452 0.341238
8 -3.163174 -1.042469 0.249022
8 1.303324 -1.989783 0.478911
1 -1.151498 -2.446757 0.731269
6 2.079868 0.587147 -0.622005
8 2.811233 0.623256 0.584147
1 2.866711 -0.273654 0.959242
8 2.676932 -0.212320 -1.616656
1 2.708506 -1.138790 -1.319303
1 2.071521 1.607114 -1.006175
6 -2.832033 1.460436 -0.753438
1 -3.507304 0.956755 -1.446290
1 -3.349869 1.655320 0.187752
1 -2.455754 2.385145 -1.184143
8 0.468705 1.679493 2.047021
1 0.097262 0.864734 2.418443
1 1.394280 1.457066 1.843249
rc_1m5dhmU/rc_1m5dhmUabf.xyz
6 -1.779923 -1.090478 -0.122783

6 0.659819 -1.245761 -0.390632
6 0.777233 0.061218 0.273514
6 -0.388945 0.704960 0.723207
1 -0.335433 1.662120 1.230118
7 -1.589936 0.171188 0.555361
7 -0.633433 -1.708610 -0.547846
8 -2.897228 -1.533887 -0.282196
8 1.631604 -1.887513 -0.783325
1 -0.758448 -2.606890 -1.014475
6 2.144075 0.679568 0.482397
8 2.871673 -0.097723 1.408105
1 3.009662 -0.991173 1.046466
8 2.806283 0.856774 -0.746886
1 2.929930 -0.008236 -1.177029
1 2.039192 1.670500 0.924594
6 -2.813422 0.857000 1.002842
1 -3.429385 1.069854 0.126692
1 -3.355593 0.193882 1.678801
1 -2.526833 1.776429 1.507349
8 -0.801211 2.875025 -1.270529
1 -0.809071 3.594749 -1.918567
1 -1.682428 2.900265 -0.869170
rc_1m5dhmU/rc_1m5dhmUabs.xyz
6 -1.366315 0.622964 0.174540
6 1.037860 1.114517 0.021153
6 1.296100 -0.332436 0.073595
6 0.208158 -1.215613 0.163963
1 0.368670 -2.288649 0.198919
7 -1.044686 -0.784655 0.214039
7 -0.297243 1.471354 0.089012
8 -2.522869 0.989949 0.219660
8 1.928493 1.954456 -0.078165
1 -0.522251 2.465822 0.053928
6 2.711083 -0.864768 -0.022364
8 3.157038 -0.725717 -1.355861
1 3.214303 0.217256 -1.591000

8 3.559374 -0.260621 0.920476
1 3.560980 0.703103 0.777852
1 2.713597 -1.933712 0.190614
6 -2.187583 -1.710618 0.301005
1 -2.780460 -1.451529 1.179648
1 -2.794496 -1.590531 -0.598586
1 -1.804538 -2.725560 0.375220
8 -5.220339 -0.063163 -0.449406
1 -5.644619 0.721089 -0.825715
1 -4.327077 0.232311 -0.207208
rc_1m5dhmU/rc_1m5dhmUaby.xyz
6 2.573396 0.381796 0.124693
6 0.281870 1.266480 0.018120
6 -0.202222 -0.115140 -0.121590
6 0.726005 -1.168273 -0.126843
1 0.399509 -2.198584 -0.224481
7 2.028253 -0.951452 -0.013157
7 1.653231 1.395876 0.137066
8 3.771272 0.540066 0.221724
8 -0.468649 2.239085 0.027420
1 2.032437 2.338203 0.230698
6 -1.684976 -0.387856 -0.262127
8 -2.362932 -0.011017 0.917540
1 -2.271751 0.947480 1.065837
8 -2.189880 0.229922 -1.421039
1 -2.064820 1.193688 -1.358709
1 -1.854082 -1.458214 -0.382597
6 3.004601 -2.052968 -0.018120
1 3.686989 -1.906351 -0.857383
1 3.565570 -2.021150 0.917675
1 2.463480 -2.990888 -0.116123
8 -5.074569 -0.888316 0.499524
1 -5.147795 -0.880557 -0.465661
1 -4.173901 -0.556759 0.677232
rc_1m5dhmU/rc_1m5dhmUado.xyz
6 -2.014893 -0.769421 -0.149868

6 0.382519 -1.304629 -0.282261
6 0.664761 -0.027536 0.384869
6 -0.410156 0.815565 0.726928
1 -0.233304 1.763945 1.221138
7 -1.667316 0.473031 0.494015
7 -0.958163 -1.577226 -0.481260
8 -3.177383 -1.043782 -0.362458
8 1.263962 -2.082185 -0.643664
1 -1.196527 -2.452837 -0.946559
6 2.097692 0.408722 0.620492
8 2.820018 -0.556099 1.341956
1 2.810378 -1.400161 0.856051
8 2.689381 0.746670 -0.620545
1 2.718784 -0.033715 -1.202177
1 2.110354 1.320692 1.217880
6 -2.788666 1.371806 0.818609
1 -3.275187 1.666578 -0.113508
1 -3.498944 0.833294 1.447364
1 -2.393159 2.241046 1.338337
8 0.468109 2.529434 -1.375633
1 -0.083258 2.014629 -1.982976
1 1.318968 2.056557 -1.354677
rc_1m5dhmU/rc_1m5dhmUacd.xyz
6 -2.021027 -0.726976 -0.220259
6 0.372542 -1.277369 -0.359639
6 0.653523 -0.088790 0.459696
6 -0.417699 0.717668 0.879245
1 -0.241111 1.610185 1.471331
7 -1.673299 0.433281 0.569478
7 -0.966411 -1.502150 -0.623134
8 -3.182126 -0.957622 -0.482377
8 1.250851 -2.023187 -0.786008
1 -1.204250 -2.318584 -1.186469
6 2.082331 0.327903 0.751513
8 2.839439 -0.711718 1.310704
1 2.828963 -1.478972 0.710624

8 2.637583 0.853303 -0.442470
1 2.717401 0.148633 -1.110418
1 2.086552 1.146069 1.472546
6 -2.795799 1.278817 1.010111
1 -3.338135 1.622725 0.127519
1 -3.460012 0.677366 1.633559
1 -2.393661 2.119729 1.569872
8 0.560202 2.535752 -1.739038
1 1.300216 2.054201 -1.328129
1 0.091190 2.940982 -0.995330
rc_1m5dhmU/rc_1m5dhmUaar.xyz
6 2.032350 0.671255 -0.301316
6 -0.385127 0.591442 -0.742475
6 -0.422830 -0.730374 -0.097391
6 0.772968 -1.280075 0.392036
1 0.784045 -2.257937 0.862259
7 1.931037 -0.643211 0.291514
7 0.857974 1.192932 -0.774950
8 3.104723 1.234263 -0.352921
8 -1.379467 1.139280 -1.214680
1 0.920889 2.117668 -1.201053
6 -1.737206 -1.465852 0.070356
8 -2.499279 -0.809263 1.061210
1 -2.706181 0.100733 0.781372
8 -2.409813 -1.610427 -1.155445
1 -2.587987 -0.729326 -1.530517
1 -1.550529 -2.472930 0.444639
6 3.192608 -1.239529 0.761779
1 3.875274 -1.311216 -0.086998
1 3.624653 -0.587258 1.522993
1 2.976535 -2.224075 1.170534
8 -2.069838 2.537481 1.490855
1 -1.445391 1.849606 1.765121
1 -2.101577 2.454818 0.525626
rc_1m5dhmU/rc_1m5dhmUadl.xyz
6 -1.995140 -0.756970 -0.120776

6 0.411511 -1.246951 -0.271013
6 0.672544 0.022353 0.419255
6 -0.415859 0.840831 0.773325
1 -0.251481 1.792500 1.267593
7 -1.668381 0.479806 0.544571
7 -0.925162 -1.539728 -0.469508
8 -3.153011 -1.047098 -0.337517
8 1.305805 -2.000526 -0.650163
1 -1.149922 -2.410006 -0.951361
6 2.097042 0.481228 0.665151
8 2.820101 -0.470609 1.403755
1 2.833717 -1.315840 0.919857
8 2.703194 0.814658 -0.569258
1 2.789892 0.017374 -1.121270
1 2.089093 1.397882 1.255279
6 -2.803375 1.359685 0.873780
1 -3.293661 1.651628 -0.057327
1 -3.506114 0.806891 1.498664
1 -2.421603 2.232320 1.398204
8 0.336439 2.173476 -1.732044
1 1.242214 1.877653 -1.532180
1 -0.077886 1.408784 -2.159405
rc_1m5dhmU/rc_1m5dhmUadt.xyz
6 -2.015238 -0.705401 0.021473
6 0.392953 -1.204243 0.090227
6 0.648521 0.175444 -0.343945
6 -0.443042 1.032440 -0.569435
1 -0.281357 2.058000 -0.885164
7 -1.694028 0.631893 -0.410554
7 -0.942317 -1.530304 0.239382
8 -3.172915 -1.039302 0.167231
8 1.289287 -2.017278 0.306809
1 -1.164933 -2.479318 0.539818
6 2.069680 0.654773 -0.574870
8 2.855415 0.511375 0.586873
1 2.913763 -0.431344 0.823861

8 2.609351 0.000106 -1.700715
1 2.653540 -0.957882 -1.534990
1 2.058563 1.719023 -0.810068
6 -2.838052 1.530363 -0.646314
1 -3.461118 1.098333 -1.431081
1 -3.414687 1.611259 0.276345
1 -2.455506 2.502167 -0.948906
8 0.548162 1.365438 2.231100
1 0.206683 0.524778 2.572438
1 1.476140 1.180889 2.002759
rc_1m5fC/rc_1m5fCaak.xyz
6 -1.170613 -1.290057 -0.006366
6 0.745252 0.030424 -0.014878
6 -0.043837 1.268620 0.000740
6 -1.449224 1.134675 0.008762
1 -2.094443 2.008554 0.018163
7 -2.006597 -0.054992 0.005681
7 0.161535 -1.168885 -0.018261
8 -1.798578 -2.343102 -0.004942
7 2.065657 0.105578 -0.025891
1 2.636445 -0.759149 -0.030312
1 2.516167 1.017231 -0.022046
6 0.498894 2.645181 0.007296
1 -0.254479 3.447922 0.016449
8 1.700107 2.890978 0.002392
6 -3.462210 -0.229150 0.013639
1 -3.748855 -0.793284 -0.876482
1 -3.738412 -0.802408 0.901170
1 -3.935815 0.750041 0.021547
8 3.694635 -2.207054 -0.071544
1 3.189311 -2.989883 0.196561
1 4.407029 -2.135667 0.581835
rc_1m5fC/rc_1m5fCacx.xyz
6 1.174233 -1.290314 0.012527
6 -0.743294 0.027664 0.020553
6 0.044123 1.266872 -0.001139

6 1.449643 1.134802 -0.012566
1 2.093618 2.009519 -0.026997
7 2.008565 -0.054122 -0.007035
7 -0.158063 -1.170929 0.027717
8 1.803728 -2.342431 0.013522
7 -2.063746 0.101508 0.032973
1 -2.634269 -0.763495 0.037852
1 -2.514966 1.012800 0.025411
6 -0.500603 2.642649 -0.011017
1 0.251537 3.446486 -0.023945
8 -1.702151 2.886804 -0.004576
6 3.464384 -0.226265 -0.019155
1 3.754766 -0.786585 0.872170
1 3.738416 -0.802587 -0.905368
1 3.936504 0.753590 -0.032550
8 -3.678299 -2.219985 0.062468
1 -4.550434 -2.030488 -0.315456
1 -3.299604 -2.901985 -0.513232
rc_1m5fC/rc_1m5fCaar.xyz
6 1.167725 1.290957 0.001746
6 -0.746162 -0.032340 0.007972
6 0.044874 -1.269373 -0.000329
6 1.450076 -1.133300 -0.005241
1 2.096684 -2.006199 -0.010045
7 2.005636 0.057220 -0.004484
7 -0.164288 1.167823 0.009095
8 1.794027 2.345001 0.000046
7 -2.066482 -0.109361 0.014797
1 -2.638288 0.754544 0.017770
1 -2.515814 -1.021574 0.014052
6 -0.495540 -2.646843 -0.002451
1 0.259257 -3.448283 -0.007742
8 -1.696342 -2.894634 0.001718
6 3.461002 0.233642 -0.009224
1 3.738552 0.804895 -0.897629
1 3.744579 0.800587 0.880078

1 3.936201 -0.744798 -0.013228
8 -3.719197 2.185526 0.062955
1 -4.166045 2.314802 -0.787433
1 -3.198935 2.992659 0.195736
rc_1m5fC/rc_1m5fCacn.xyz
6 1.174927 -1.297123 0.006229
6 -0.738298 0.027050 0.010574
6 0.053831 1.263591 -0.000717
6 1.459007 1.126680 -0.004781
1 2.106132 1.999193 -0.011216
7 2.013846 -0.064106 -0.001221
7 -0.157075 -1.173235 0.013113
8 1.800526 -2.351543 0.005749
7 -2.058705 0.106028 0.018213
1 -2.634123 -0.755178 0.021643
1 -2.505897 1.019246 0.013303
6 -0.485205 2.641547 -0.008494
1 0.270407 3.442220 -0.014076
8 -1.685781 2.890596 -0.008507
6 3.469096 -0.241591 -0.005166
1 3.751967 -0.807882 0.884762
1 3.746542 -0.813880 -0.892936
1 3.945023 0.736493 -0.009995
8 -3.670943 -2.221097 0.030569
1 -4.525547 -2.068981 0.461511
1 -3.891537 -2.496613 -0.872080
rc_1m5fC/rc_1m5fCacp.xyz
6 -1.162037 -1.288312 0.002608
6 0.751501 0.035537 0.000877
6 -0.040061 1.272292 0.000206
6 -1.445261 1.135767 -0.000553
1 -2.092158 2.008468 -0.001880
7 -2.000445 -0.054913 0.000145
7 0.169941 -1.164806 0.002550
8 -1.787986 -2.342584 0.004330
7 2.071851 0.112711 -0.000296

1 2.642582 -0.752123 -0.001170
1 2.521328 1.024800 -0.001626
6 0.499845 2.649954 -0.000475
1 -0.255261 3.451126 -0.000934
8 1.700569 2.898137 -0.000501
6 -3.455762 -0.231861 -0.001127
1 -3.735159 -0.802310 -0.889488
1 -3.737095 -0.799784 0.888241
1 -3.931336 0.746403 -0.003082
8 3.711570 -2.192724 -0.028539
1 3.681492 -2.637463 0.832212
1 3.333606 -2.832956 -0.650602
rc_1m5fC/rc_1m5fCadq.xyz
6 -1.167589 -1.290347 -0.000189
6 0.746543 0.032582 0.005859
6 -0.044118 1.269810 -0.000822
6 -1.449342 1.134080 -0.003347
1 -2.095758 2.007130 -0.006046
7 -2.005171 -0.056325 -0.002534
7 0.164454 -1.167527 0.005532
8 -1.794205 -2.344203 -0.003220
7 2.066884 0.109158 0.012678
1 2.637947 -0.755292 0.016038
1 2.516574 1.021201 0.012279
6 0.496729 2.647111 -0.003368
1 -0.257820 3.448795 -0.006877
8 1.697614 2.894536 -0.001514
6 -3.460579 -0.232367 -0.004404
1 -3.740242 -0.801704 -0.893390
1 -3.742333 -0.801101 0.884329
1 -3.935538 0.746197 -0.005285
8 3.695615 -2.200144 0.079876
1 4.339192 -2.220192 -0.644654
1 3.172752 -3.008901 -0.029643
rc_1m5fC/rc_1m5fCaag.xyz
6 -1.690217 -0.822587 0.036286

6 0.615977 -1.105010 -0.097186
6 0.816293 0.310967 -0.423201
6 -0.343684 1.120205 -0.504965
1 -0.264199 2.177871 -0.739753
7 -1.532647 0.611163 -0.302725
7 -0.599085 -1.602038 0.114769
8 -2.842117 -1.198987 0.223260
7 1.669246 -1.904833 -0.010025
1 1.531548 -2.884889 0.214021
1 2.604145 -1.545696 -0.179866
6 2.115082 0.948129 -0.725059
1 2.070442 2.025336 -0.946131
8 3.177095 0.335496 -0.731699
6 -2.757371 1.419062 -0.395171
1 -3.397560 0.989643 -1.167440
1 -3.274625 1.378196 0.564654
1 -2.487207 2.443747 -0.644277
8 1.021512 1.183723 2.063750
1 1.896957 1.602581 2.069061
1 1.169505 0.296721 2.428897
rc_1m5fC/rc_1m5fCach.xyz
6 -1.698636 -0.814492 0.031471
6 0.606353 -1.087514 -0.138539
6 0.800870 0.341212 -0.409289
6 -0.362120 1.147512 -0.464205
1 -0.287872 2.211572 -0.670850
7 -1.548576 0.628199 -0.270411
7 -0.605364 -1.593959 0.069077
8 -2.847247 -1.198237 0.224701
7 1.661553 -1.888140 -0.099395
1 1.529017 -2.876258 0.089379
1 2.593926 -1.518907 -0.260874
6 2.096825 0.991976 -0.695317
1 2.047713 2.076420 -0.877182
8 3.160808 0.383901 -0.730797
6 -2.776974 1.432039 -0.340848

1 -3.418669 1.014888 -1.118653
1 -3.290009 1.369599 0.620108
1 -2.512908 2.462745 -0.570953
8 1.111579 0.946763 2.098663
1 1.555678 1.807774 2.035593
1 1.830801 0.305653 2.218350
rc_1m5fC/rc_1m5fCaad.xyz
6 1.607430 -0.974955 -0.127110
6 -0.703436 -1.246675 -0.112231
6 -0.903322 0.113711 0.398766
6 0.260209 0.880936 0.661851
1 0.183193 1.882734 1.070686
7 1.451088 0.385813 0.434602
7 0.514606 -1.721560 -0.356564
8 2.761341 -1.328291 -0.343127
7 -1.760214 -2.015128 -0.336537
1 -1.625481 -2.954784 -0.695321
1 -2.698871 -1.661739 -0.174753
6 -2.209710 0.761075 0.633602
1 -2.166582 1.789709 1.021578
8 -3.279602 0.202105 0.415212
6 2.668814 1.181392 0.635548
1 3.360096 0.617657 1.262523
1 3.127801 1.356632 -0.340133
1 2.398513 2.123879 1.107598
8 -0.450221 2.407097 -1.520464
1 0.437304 2.780433 -1.405144
1 -1.026394 2.981407 -0.993064
rc_1m5fC/rc_1m5fCacd.xyz
6 1.680772 -0.827023 0.034509
6 -0.626236 -1.103160 -0.103346
6 -0.822107 0.315430 -0.420395
6 0.341897 1.118413 -0.510946
1 0.266211 2.176343 -0.746109
7 1.529186 0.604420 -0.313690
7 0.587207 -1.603779 0.109413

8 2.830558 -1.205674 0.230646
7 -1.681116 -1.901999 -0.026175
1 -1.545732 -2.883692 0.191975
1 -2.615180 -1.538967 -0.192054
6 -2.120695 0.962158 -0.699127
1 -2.073075 2.040949 -0.911904
8 -3.186509 0.355760 -0.697092
6 2.756509 1.408294 -0.407855
1 3.269260 1.374833 0.554719
1 3.399219 0.971195 -1.173528
1 2.489568 2.431477 -0.666515
8 -0.950564 1.143704 2.065718
1 -1.660043 1.804885 2.019125
1 -1.385883 0.337483 2.386239
rc_1m5fC/rc_1m5fCaau.xyz
6 -1.909302 -0.987525 0.013426
6 0.401106 -0.688079 0.058302
6 0.260051 0.773451 0.013399
6 -1.053520 1.291161 -0.056396
1 -1.225612 2.363202 -0.102050
7 -2.093129 0.489434 -0.049253
7 -0.667341 -1.485124 0.065459
8 -2.944900 -1.642969 0.010376
7 1.604361 -1.237998 0.092660
1 1.662110 -2.251852 0.118106
1 2.482731 -0.713476 0.057752
6 1.360870 1.760996 0.047570
1 1.047991 2.812835 -0.037631
8 2.538206 1.446661 0.164481
6 -3.467634 0.998131 -0.087330
1 -3.961797 0.601434 -0.976585
1 -3.991609 0.637256 0.800064
1 -3.441712 2.085596 -0.108298
8 4.474172 -0.768615 -0.087550
1 4.614923 0.169641 -0.283452
1 4.816486 -1.238251 -0.862236

rc_1m5fC/rc_1m5fCadr.xyz
6 2.345406 0.461055 0.054574
6 0.153456 1.235856 -0.022355
6 -0.378677 -0.131714 -0.075469
6 0.559405 -1.188940 -0.074332
1 0.235834 -2.224320 -0.117923
7 1.847195 -0.940433 -0.013967
7 1.460978 1.468453 0.040139
8 3.562135 0.581884 0.120849
7 -0.688771 2.258122 -0.036395
1 -0.321933 3.204243 0.000526
1 -1.692204 2.102936 -0.075219
6 -1.807317 -0.503528 -0.106642
1 -2.021946 -1.581491 -0.140451
8 -2.710787 0.327780 -0.088972
6 2.842023 -2.017437 -0.004985
1 3.510541 -1.876728 -0.856675
1 3.420694 -1.943523 0.918185
1 2.325338 -2.973077 -0.066553
8 -5.305306 -0.963268 0.212326
1 -4.471898 -0.475174 0.081411
1 -5.974348 -0.438778 -0.250115
rc_1m5fC/rc_1m5fCaco.xyz
6 -1.438686 0.411210 -0.078247
6 0.549144 -0.814577 -0.070495
6 1.356153 0.408481 0.005263
6 0.660256 1.636727 0.048484
1 1.201062 2.576145 0.112863
7 -0.652687 1.669874 0.011775
7 -0.779852 -0.758606 -0.119758
8 -2.653923 0.542614 -0.109551
7 1.150834 -1.992242 -0.089735
1 0.590091 -2.838348 -0.141584
1 2.165023 -2.053572 -0.054864
6 2.833492 0.464604 0.047328
1 3.267597 1.473690 0.106118

8 3.535162 -0.540608 0.022429
6 -1.401795 2.929993 0.073025
1 -2.045347 2.995446 -0.806218
1 -2.024408 2.916291 0.970490
1 -0.696858 3.758303 0.103290
8 -2.239216 -3.250637 0.201953
1 -1.808493 -2.380150 0.068464
1 -3.164304 -3.110578 -0.045324
rc_1m5fC/rc_1m5fCaah.xyz
6 1.690871 -0.816292 0.026506
6 -0.614536 -1.080725 -0.148404
6 -0.804940 0.350866 -0.406443
6 0.360529 1.151969 -0.467646
1 0.290600 2.216101 -0.675551
7 1.546088 0.627508 -0.276694
7 0.595384 -1.592074 0.058912
8 2.838083 -1.202257 0.223869
7 -1.670903 -1.879446 -0.125638
1 -1.542144 -2.869381 0.056020
1 -2.602626 -1.504294 -0.276851
6 -2.099111 1.009737 -0.684135
1 -2.045694 2.094727 -0.861416
8 -3.165914 0.406976 -0.718526
6 2.777070 1.426068 -0.353890
1 3.294654 1.362095 0.604569
1 3.413347 1.005278 -1.134296
1 2.516179 2.457639 -0.583691
8 -1.165181 0.810177 2.127227
1 -0.510514 1.513243 2.266063
1 -2.022990 1.263768 2.152605
rc_1m5fC/rc_1m5fCacg.xyz
6 1.688962 0.815452 0.022371
6 -0.615952 1.074569 -0.165627
6 -0.803817 -0.362528 -0.394896
6 0.364517 -1.160829 -0.447912
1 0.297273 -2.227782 -0.641607

7 1.548518 -0.630156 -0.269019
7 0.592264 1.590581 0.039001
8 2.834067 1.205482 0.224699
7 -1.673592 1.872573 -0.162710
1 -1.546551 2.865891 0.000593
1 -2.604296 1.493477 -0.310041
6 -2.096999 -1.029576 -0.655754
1 -2.041157 -2.117473 -0.813497
8 -3.165754 -0.430402 -0.699250
6 2.781973 -1.425686 -0.341074
1 3.420136 -1.003534 -1.119047
1 3.295621 -1.361029 0.619457
1 2.524139 -2.457801 -0.571961
8 -1.082854 -0.813744 2.123948
1 -1.247871 -1.769806 2.087544
1 -1.963405 -0.412014 2.199837
rc_1m5fC/rc_1m5fCabu.xyz
6 1.715547 -0.811305 -0.061516
6 -0.586990 -1.112583 0.092142
6 -0.796701 0.301702 0.419827
6 0.356078 1.118297 0.500685
1 0.270463 2.174124 0.742067
7 1.548883 0.619898 0.284120
7 0.630161 -1.598719 -0.133961
8 2.869131 -1.177590 -0.259028
7 -1.634032 -1.921821 0.019065
1 -1.490517 -2.901572 -0.202502
1 -2.569263 -1.571314 0.205283
6 -2.101898 0.927826 0.728349
1 -2.067365 2.009967 0.924491
8 -3.152752 0.299688 0.772197
6 2.767502 1.436932 0.373573
1 3.412720 1.013173 1.144956
1 3.283560 1.398485 -0.587002
1 2.490769 2.460029 0.622106
8 -1.391170 1.199878 -1.982928

1 -1.307086 0.325573 -2.395875
1 -0.561275 1.654997 -2.198383
rc_1m5fC/rc_1m5fCabd.xyz
6 1.294846 -1.299853 -0.109818
6 -1.020481 -1.110807 -0.223882
6 -0.986629 0.237023 0.355639
6 0.282255 0.758765 0.699610
1 0.375777 1.745461 1.140613
7 1.370756 0.052657 0.499025
7 0.094465 -1.805970 -0.430975
8 2.370946 -1.862090 -0.274895
7 -2.189087 -1.642926 -0.551136
1 -2.215757 -2.574724 -0.953309
1 -3.051785 -1.125960 -0.405672
6 -2.160186 1.098316 0.612118
1 -1.937485 2.078935 1.059046
8 -3.308375 0.754990 0.351471
6 2.700422 0.573691 0.835972
1 3.162806 -0.096962 1.563182
1 3.309207 0.587104 -0.070110
1 2.593306 1.575109 1.247385
8 0.864489 2.830160 -1.388446
1 1.759672 2.467768 -1.314830
1 0.993481 3.789651 -1.407574
rc_1m5fC/rc_1m5fCabj.xyz
6 1.693180 -0.813645 0.033197
6 -0.611961 -1.090455 -0.134069
6 -0.810048 0.339657 -0.393869
6 0.352474 1.144370 -0.464719
1 0.277312 2.207750 -0.674747
7 1.542365 0.624030 -0.287282
7 0.600983 -1.594391 0.075461
8 2.841798 -1.193419 0.236093
7 -1.664384 -1.894533 -0.106427
1 -1.530210 -2.883447 0.076790
1 -2.597851 -1.525151 -0.261268

6 -2.110018 0.991682 -0.663288
1 -2.065068 2.080547 -0.818239
8 -3.170253 0.380122 -0.717673
6 2.769726 1.427073 -0.380250
1 3.289931 1.382077 0.577872
1 3.406084 0.997200 -1.155439
1 2.502707 2.453345 -0.626339
8 -1.268548 0.924704 2.070343
1 -0.421611 0.572770 2.389100
1 -1.138140 1.885825 2.027885
rc_1m5fC/rc_1m5fCace.xyz
6 0.968202 0.690662 -0.139010
6 -1.336524 0.961076 -0.019832
6 -1.555310 -0.490110 0.020313
6 -0.409933 -1.313621 -0.046213
1 -0.501725 -2.396158 -0.033488
7 0.790627 -0.786964 -0.132392
7 -0.113028 1.478226 -0.093993
8 2.128419 1.084867 -0.191755
7 -2.383864 1.770329 0.016926
1 -2.236494 2.774945 -0.004660
1 -3.325965 1.393562 0.083062
6 -2.868096 -1.164598 0.124687
1 -2.835102 -2.264063 0.143150
8 -3.928408 -0.551965 0.183910
6 1.996562 -1.618277 -0.210384
1 2.567534 -1.319118 -1.090966
1 2.597630 -1.437979 0.684228
1 1.702039 -2.663718 -0.271655
8 4.851141 0.276569 0.387537
1 4.866944 -0.691143 0.371731
1 3.930369 0.505993 0.169905
rc_1m5fC/rc_1m5fCads.xyz
6 0.371471 1.508692 -0.003680
6 -1.800364 0.679162 0.011593
6 -1.322613 -0.709753 -0.011673

6 0.075734 -0.910465 -0.031543
1 0.495161 -1.912314 -0.049790
7 0.896456 0.114893 -0.027811
7 -0.954752 1.705581 0.012351
8 1.223438 2.388224 0.000278
7 -3.104355 0.910946 0.032320
1 -3.437668 1.869789 0.050488
1 -3.767264 0.140180 0.033448
6 -2.178098 -1.914304 -0.013497
1 -1.643571 -2.875582 -0.040859
8 -3.402989 -1.859856 0.013794
6 2.351843 -0.058851 -0.043497
1 2.752607 0.448840 -0.923098
1 2.765031 0.406196 0.853867
1 2.577102 -1.122219 -0.070298
8 5.594150 -1.232474 0.048117
1 4.952682 -1.210628 0.773367
1 5.139835 -0.798237 -0.688875
rc_1m5fU/rc_1m5fUadl.xyz
6 -1.055549 1.022589 -0.034919
6 -0.033673 -1.198410 -0.038365
6 1.287928 -0.550874 0.001979
6 1.371010 0.859454 0.026309
1 2.335908 1.355257 0.056412
7 0.287663 1.606835 0.009411
7 -1.112511 -0.337965 -0.057435
8 -2.017058 1.760360 -0.046052
8 -0.175653 -2.419120 -0.051014
1 -2.071160 -0.768880 -0.050259
6 2.520215 -1.364808 0.014160
1 2.385434 -2.453667 0.003465
8 3.627558 -0.837807 0.033289
6 0.347439 3.077190 0.023112
1 -0.149735 3.447375 -0.875798
1 -0.188858 3.434242 0.904683
1 1.390226 3.384219 0.047302

8 -3.568451 -1.575179 -0.013177
1 -3.377360 -2.506773 0.177919
1 -4.105902 -1.270751 0.734424
rc_1m5fU/rc_1m5fUaco.xyz
6 -1.111264 0.907853 -0.050344
6 0.072714 -1.231407 -0.055560
6 1.340959 -0.486505 0.002858
6 1.317686 0.926138 0.042891
1 2.244387 1.489607 0.091131
7 0.182716 1.591406 0.012557
7 -1.066042 -0.453024 -0.096512
8 -2.125939 1.570851 -0.057606
8 0.022627 -2.459291 -0.064825
1 -1.997432 -0.941066 -0.079503
6 2.631806 -1.205171 0.013428
1 2.580782 -2.300957 -0.014454
8 3.695603 -0.596045 0.047411
6 0.133201 3.061863 0.031596
1 -0.396824 3.396663 -0.862487
1 -0.420809 3.376138 0.918763
1 1.150587 3.444877 0.049853
8 -3.538828 -1.644507 0.018320
1 -3.563444 -2.294282 0.738016
1 -4.152286 -0.944353 0.290745
rc_1m5fU/rc_1m5fUabb.xyz
6 0.297417 -1.639420 -0.075897
6 -0.875034 0.521091 -0.098416
6 0.435257 1.184379 -0.011209
6 1.609798 0.400903 0.060966
1 2.582565 0.877054 0.138031
7 1.559319 -0.914803 0.040283
7 -0.825902 -0.859863 -0.132102
8 0.302439 -2.847647 -0.114543
8 -1.935981 1.137621 -0.136443
1 -1.713429 -1.361549 -0.194111
6 0.526412 2.659950 -0.006602

1 -0.423150 3.208250 -0.046563
8 1.613806 3.224483 0.031870
6 2.768219 -1.749684 0.129948
1 2.673614 -2.392622 1.007232
1 2.828368 -2.368291 -0.767594
1 3.635797 -1.099867 0.215170
8 -4.309222 -0.567538 0.286616
1 -3.626385 0.095659 0.085224
1 -5.032064 -0.374635 -0.327400
rc_1m5fU/rc_1m5fUacj.xyz
6 -1.765685 0.540601 -0.096221
6 0.558368 1.328744 -0.337349
6 0.979191 -0.080882 -0.357768
6 -0.000512 -1.100316 -0.292709
1 0.293427 -2.143917 -0.336510
7 -1.280235 -0.823545 -0.176464
7 -0.809357 1.514490 -0.202257
8 -2.950628 0.752919 0.043887
8 1.332058 2.274671 -0.428721
1 -1.149486 2.475184 -0.164298
6 2.405044 -0.434557 -0.527088
1 3.111357 0.405471 -0.557686
8 2.767291 -1.600505 -0.623752
6 -2.306436 -1.880428 -0.108236
1 -2.848341 -1.780733 0.833104
1 -2.994219 -1.748259 -0.944741
1 -1.805112 -2.843513 -0.166555
8 1.143277 -0.197997 2.203882
1 1.991823 0.264223 2.302774
1 0.471897 0.463255 2.438813
rc_1m5fU/rc_1m5fUabc.xyz
6 1.808068 0.543253 -0.048782
6 -0.515448 1.305254 -0.368658
6 -0.926649 -0.108443 -0.356319
6 0.056091 -1.115923 -0.240657
1 -0.227451 -2.163076 -0.265898

7 1.333297 -0.825844 -0.098275
7 0.845567 1.506220 -0.194285
8 2.989218 0.768398 0.104186
8 -1.291394 2.241575 -0.517681
1 1.177813 2.470328 -0.180576
6 -2.345620 -0.479171 -0.568597
1 -3.060291 0.353079 -0.602440
8 -2.687882 -1.646650 -0.698592
6 2.366398 -1.871799 0.007942
1 3.067067 -1.750038 -0.819682
1 2.893465 -1.749236 0.955153
1 1.875606 -2.840865 -0.040176
8 -1.668470 0.026164 2.068020
1 -1.113969 -0.685659 2.427567
1 -1.253106 0.847918 2.376941
rc_1m5fU/rc_1m5fUabi.xyz
6 -1.142428 -0.519755 -0.017999
6 0.468767 1.338460 -0.031964
6 1.550225 0.342430 -0.009894
6 1.229384 -1.033967 -0.007474
1 2.015035 -1.783484 -0.000584
7 -0.020420 -1.448350 -0.017204
7 -0.809869 0.804673 -0.030716
8 -2.274466 -0.952022 -0.009527
8 0.666058 2.547784 -0.048428
1 -1.593090 1.459411 -0.031255
6 2.963772 0.772939 0.016184
1 3.147298 1.853975 -0.024057
8 3.870493 -0.048851 0.087584
6 -0.364644 -2.879852 -0.021976
1 -0.949458 -3.097331 0.874092
1 -0.969183 -3.086008 -0.907272
1 0.558093 -3.454867 -0.035839
8 -4.378676 1.215910 0.118662
1 -3.911002 1.937897 -0.326056
1 -3.783391 0.452045 0.038816

rc_1m5fU/rc_1m5fUaad.xyz
6 -1.779936 0.546789 -0.081704
6 0.550522 1.297112 -0.371239
6 0.953374 -0.118606 -0.352171
6 -0.036536 -1.121961 -0.246688
1 0.242771 -2.170391 -0.265346
7 -1.312396 -0.825437 -0.119952
7 -0.812558 1.504399 -0.228312
8 -2.961338 0.778230 0.059573
8 1.334161 2.229418 -0.502790
1 -1.140366 2.469997 -0.218189
6 2.371517 -0.496522 -0.541403
1 3.088192 0.333409 -0.597916
8 2.716436 -1.668503 -0.625212
6 -2.351433 -1.866107 -0.016781
1 -2.888139 -1.733729 0.923598
1 -3.042153 -1.748168 -0.853170
1 -1.863993 -2.837521 -0.051102
8 1.245392 0.125132 2.149851
1 1.452841 -0.806038 2.333999
1 2.103262 0.581265 2.174524
rc_1m5fU/rc_1m5fUaat.xyz
6 1.772308 0.537341 -0.082637
6 -0.547770 1.331247 -0.337577
6 -0.971772 -0.077598 -0.363707
6 0.005478 -1.098964 -0.296121
1 -0.289790 -2.142040 -0.345357
7 1.285163 -0.825054 -0.172404
7 0.817952 1.513517 -0.185851
8 2.957005 0.747213 0.063303
8 -1.318735 2.278870 -0.434424
1 1.160025 2.473292 -0.142625
6 -2.397319 -0.426881 -0.542945
1 -3.099047 0.416054 -0.591457
8 -2.764547 -1.592466 -0.627568
6 2.309347 -1.883824 -0.104393

1 3.001218 -1.749108 -0.937122
1 2.846993 -1.789235 0.839927
1 1.806876 -2.845871 -0.169493
8 -1.110712 -0.141221 2.193109
1 -1.961725 -0.600376 2.285428
1 -1.312071 0.790943 2.377402
rc_1m5fU/rc_1m5fUaae.xyz
6 -1.880305 -0.811069 0.007810
6 0.514958 -0.972227 -0.526274
6 0.603349 0.488403 -0.370414
6 -0.546324 1.219869 0.004957
1 -0.492035 2.294317 0.150527
7 -1.706733 0.625856 0.191332
7 -0.749941 -1.499564 -0.339536
8 -2.971043 -1.306759 0.170776
8 1.480326 -1.679955 -0.795175
1 -0.859738 -2.508365 -0.447138
6 1.887682 1.189661 -0.588774
1 2.729612 0.573983 -0.929497
8 1.987834 2.395179 -0.392122
6 -2.911716 1.371356 0.589461
1 -3.659098 1.267225 -0.200126
1 -3.295938 0.934045 1.512635
1 -2.638708 2.413953 0.732282
8 3.562766 -0.963871 1.465726
1 3.890984 -0.085875 1.223316
1 3.036719 -1.246038 0.701186
rc_1m5fU/rc_1m5fUabj.xyz
6 -1.785293 0.547334 -0.076341
6 0.544294 1.295085 -0.381819
6 0.946998 -0.120474 -0.351970
6 -0.043060 -1.122905 -0.244094
1 0.235801 -2.171462 -0.258477
7 -1.319367 -0.825225 -0.119287
7 -0.817605 1.504251 -0.228012
8 -2.965262 0.780208 0.073661

8 1.327714 2.225923 -0.527584
1 -1.144979 2.470096 -0.221207
6 2.367496 -0.500082 -0.526724
1 3.089027 0.327395 -0.541333
8 2.706964 -1.669660 -0.646174
6 -2.358738 -1.864503 -0.007765
1 -2.885478 -1.733003 0.938496
1 -3.058062 -1.743964 -0.836575
1 -1.873199 -2.836601 -0.048913
8 1.446812 -0.021210 2.142464
1 0.536839 0.166925 2.425936
1 1.898848 0.838601 2.166502
rc_1m5fU/rc_1m5fUaay.xyz
6 -1.769973 0.536815 -0.087199
6 0.549291 1.336623 -0.332779
6 0.976012 -0.070885 -0.364100
6 0.001031 -1.094980 -0.300899
1 0.299247 -2.137045 -0.352434
7 -1.279453 -0.824420 -0.180258
7 -0.817338 1.515383 -0.181945
8 -2.955637 0.743300 0.055167
8 1.318477 2.286201 -0.424909
1 -1.161708 2.474218 -0.136006
6 2.401748 -0.417198 -0.543826
1 3.103332 0.426350 -0.582835
8 2.769406 -1.581845 -0.640019
6 -2.301200 -1.885858 -0.119143
1 -2.841811 -1.795709 0.823916
1 -2.990825 -1.749523 -0.953498
1 -1.796173 -2.846429 -0.186361
8 1.103327 -0.198633 2.213899
1 2.056102 -0.359267 2.311330
1 0.983330 0.731372 2.465875
rc_1m5fU/rc_1m5fUadr.xyz
6 -1.776818 0.544448 -0.082814
6 0.551622 1.301610 -0.367807

6 0.958156 -0.113018 -0.354380
6 -0.029768 -1.119400 -0.253841
1 0.251934 -2.167121 -0.276554
7 -1.306473 -0.826353 -0.130867
7 -0.811778 1.505175 -0.224917
8 -2.958430 0.772709 0.061681
8 1.333766 2.236443 -0.491779
1 -1.141483 2.470043 -0.207610
6 2.378024 -0.485435 -0.540036
1 3.090708 0.347289 -0.604075
8 2.728639 -1.656617 -0.612743
6 -2.343813 -1.869638 -0.035392
1 -2.881153 -1.744854 0.905618
1 -3.034082 -1.746991 -0.871443
1 -1.854384 -2.839769 -0.076224
8 1.198818 0.077391 2.159445
1 1.623380 -0.783152 2.311629
1 1.926083 0.721990 2.181938
rc_1m5fU/rc_1m5fUack.xyz
6 -1.076733 0.529615 0.545980
6 1.195550 1.312983 0.025105
6 1.606325 -0.094126 -0.089474
6 0.656501 -1.117483 0.128144
1 0.946810 -2.160903 0.057541
7 -0.594663 -0.841585 0.432822
7 -0.141168 1.501695 0.339124
8 -2.240968 0.735294 0.810522
8 1.961752 2.255020 -0.139309
1 -0.474196 2.463115 0.416032
6 3.006121 -0.439667 -0.416587
1 3.702478 0.400193 -0.533120
8 3.351589 -1.608130 -0.549991
6 -1.586131 -1.896954 0.698278
1 -1.980314 -1.749941 1.705772
1 -2.397412 -1.800788 -0.026337
1 -1.096749 -2.863702 0.606846

8 -4.138272 -0.078952 -1.425219
1 -3.590797 0.137092 -0.653656
1 -4.241606 0.762098 -1.893396
rc_1m5fU/rc_1m5fUabx.xyz
6 1.798224 -0.534692 -0.054074
6 -0.519867 -1.325535 -0.343524
6 -0.943831 0.084197 -0.358579
6 0.030957 1.103164 -0.271951
1 -0.262936 2.146823 -0.317705
7 1.311272 0.828013 -0.132713
7 0.844407 -1.509565 -0.174841
8 2.982017 -0.746177 0.099226
8 -1.288159 -2.272049 -0.463397
1 1.185942 -2.469875 -0.140348
6 -2.368423 0.435927 -0.560937
1 -3.070554 -0.406836 -0.602693
8 -2.730725 1.599724 -0.669433
6 2.334638 1.886543 -0.055296
1 2.871383 1.786551 0.888945
1 3.028261 1.757856 -0.887689
1 1.832215 2.848916 -0.115497
8 -1.527796 0.000136 2.094489
1 -0.702614 -0.392977 2.423459
1 -1.444338 0.949715 2.283505
rc_1m5fU/rc_1m5fUacw.xyz
6 1.762725 -0.539182 -0.098206
6 -0.559821 -1.333565 -0.332127
6 -0.983373 0.074932 -0.358489
6 -0.005491 1.096913 -0.299433
1 -0.301962 2.139602 -0.347478
7 1.274914 0.823503 -0.185131
7 0.808029 -1.515561 -0.196473
8 2.948327 -0.748660 0.040642
8 -1.331643 -2.281533 -0.418763
1 1.150581 -2.475214 -0.154325
6 -2.409703 0.425141 -0.527629

1 -3.113825 -0.416563 -0.561431
8 -2.774984 1.590672 -0.620938
6 2.298613 1.883155 -0.123405
1 2.841558 1.789306 0.817937
1 2.985744 1.748674 -0.960053
1 1.794742 2.844685 -0.185882
8 -1.084227 0.211729 2.211760
1 -2.019407 -0.017791 2.337551
1 -0.595522 -0.590322 2.459034
rc_1m5fU/rc_1m5fUabw.xyz
6 -1.777291 -0.544780 -0.084421
6 0.552359 -1.299633 -0.375060
6 0.957919 0.114738 -0.350975
6 -0.030783 1.120065 -0.249844
1 0.251127 2.167749 -0.267375
7 -1.307723 0.826376 -0.130290
7 -0.811322 -1.504703 -0.228002
8 -2.958635 -0.774195 0.060200
8 1.333888 -2.233454 -0.511464
1 -1.140980 -2.469719 -0.218117
6 2.379077 0.490376 -0.523469
1 3.097012 -0.339832 -0.554828
8 2.724398 1.660659 -0.620212
6 -2.345176 1.869445 -0.032959
1 -3.033465 1.750209 -0.871155
1 -2.884783 1.740964 0.906243
1 -1.855389 2.839604 -0.068723
8 1.337910 0.046718 2.156328
1 1.985289 -0.677827 2.181860
1 0.497395 -0.361945 2.421695
rc_1m5fU/rc_1m5fUacs.xyz
6 2.350812 0.305721 0.070322
6 0.182167 1.461078 -0.044321
6 -0.465826 0.141517 -0.110400
6 0.325337 -1.029396 -0.094487
1 -0.140530 -2.008908 -0.142946

7 1.637919 -0.967073 -0.008421
7 1.562250 1.423099 0.053499
8 3.557492 0.310014 0.146011
8 -0.439601 2.516916 -0.070756
1 2.055293 2.315653 0.096929
6 -1.937268 0.046615 -0.157787
1 -2.490542 0.993773 -0.158590
8 -2.501949 -1.042937 -0.182549
6 2.481611 -2.170812 0.033399
1 3.179037 -2.133260 -0.805748
1 3.042934 -2.159557 0.970153
1 1.842774 -3.048603 -0.026865
8 -5.339358 -0.464412 0.258756
1 -5.299109 0.502258 0.294603
1 -4.424696 -0.738514 0.064873
rc_1m5fU/rc_1m5fUaah.xyz
6 -1.785493 -0.535707 -0.066655
6 0.535551 -1.321263 -0.348509
6 0.955781 0.089684 -0.354734
6 -0.022303 1.106563 -0.272859
1 0.269568 2.150908 -0.314687
7 -1.302946 0.828226 -0.145862
7 -0.829077 -1.508243 -0.187125
8 -2.968880 -0.750731 0.085332
8 1.306096 -2.265599 -0.469446
1 -1.168612 -2.469299 -0.154022
6 2.380780 0.445116 -0.542391
1 3.084533 -0.396064 -0.590234
8 2.743133 1.610843 -0.631442
6 -2.329285 1.884059 -0.071698
1 -3.019786 1.753662 -0.906378
1 -2.868763 1.782557 0.870840
1 -1.829028 2.847729 -0.129906
8 1.418488 -0.044568 2.117055
1 1.546703 0.905364 2.277437
1 0.508669 -0.225015 2.406942

rc_1m5fU/rc_1m5fUabd.xyz
6 2.364373 0.255734 -0.079755
6 0.236337 1.485629 0.040176
6 -0.454782 0.189407 0.123839
6 0.297006 -1.007427 0.111036
1 -0.202612 -1.969261 0.173494
7 1.609749 -0.991405 0.010440
7 1.613758 1.399089 -0.065718
8 3.570055 0.219253 -0.162512
8 -0.348504 2.562471 0.060272
1 2.136880 2.273736 -0.120760
6 -1.928449 0.141773 0.185227
1 -2.451628 1.105522 0.185457
8 -2.525758 -0.929922 0.220739
6 2.411236 -2.224487 -0.033833
1 2.982718 -2.224048 -0.964248
1 3.100038 -2.219150 0.813544
1 1.740460 -3.079255 0.011343
8 -5.382848 -0.439238 -0.192589
1 -5.483436 -0.428050 -1.155113
1 -4.444857 -0.657557 -0.044188
rc_1m5fU/rc_1m5fUadu.xyz
6 -1.423250 0.528379 -0.528341
6 0.885520 1.306573 -0.199097
6 1.303145 -0.103163 -0.132930
6 0.335522 -1.123681 -0.262916
1 0.624061 -2.168170 -0.200001
7 -0.934634 -0.843261 -0.457501
7 -0.464357 1.498843 -0.433932
8 -2.603513 0.736289 -0.692320
8 1.662979 2.246009 -0.068938
1 -0.800792 2.460814 -0.476415
6 2.724085 -0.451884 0.076681
1 3.422686 0.387445 0.184175
8 3.087348 -1.622081 0.120041
6 -1.963156 -1.891522 -0.524492

1 -2.643829 -1.752446 0.318677
1 -2.515771 -1.780476 -1.458919
1 -1.471500 -2.859640 -0.471671
8 -1.428312 -0.013491 2.291375
1 -1.369511 0.944728 2.417804
1 -2.371619 -0.183340 2.151697
rc_1m5hmC/rc_1m5hmCads.xyz
6 -2.061240 0.118713 -0.206248
6 -0.269794 1.596852 -0.097116
6 0.642242 0.542917 0.351874
6 0.076043 -0.726679 0.558709
1 0.678131 -1.548490 0.927940
7 -1.202254 -0.949025 0.315763
7 -1.548901 1.352222 -0.368810
8 -3.223407 -0.195753 -0.456555
6 2.103387 0.779001 0.569205
1 2.510139 1.262109 -0.335218
1 2.218254 1.510410 1.383768
7 0.189419 2.835920 -0.247868
1 -0.445293 3.565329 -0.554559
1 1.149123 3.093688 -0.055949
8 2.836675 -0.376799 0.907146
1 2.884439 -0.943733 0.099585
6 -1.811430 -2.266143 0.540871
1 -2.639050 -2.154307 1.243130
1 -2.192183 -2.646457 -0.408349
1 -1.054680 -2.935164 0.945388
8 2.676706 -1.786562 -1.437150
1 2.644456 -1.108170 -2.129264
1 1.783778 -2.164098 -1.421360
rc_1m5hmC/rc_1m5hmCacs.xyz
6 -2.106031 -0.091083 -0.138187
6 -0.504904 1.595799 -0.145125
6 0.557022 0.667803 0.254796
6 0.173810 -0.671778 0.438596
1 0.894819 -1.420330 0.742473

7 -1.075010 -1.057354 0.254609
7 -1.762936 1.197436 -0.315096
8 -3.241494 -0.541275 -0.280890
6 1.978436 1.093747 0.454356
1 2.332306 1.552359 -0.485823
1 1.997137 1.892648 1.210635
7 -0.214738 2.878557 -0.341244
1 -0.952753 3.513750 -0.625251
1 0.719833 3.251355 -0.233228
8 2.840999 0.065932 0.886733
1 2.977898 -0.569887 0.141950
6 -1.480640 -2.457753 0.429503
1 -2.242641 -2.509722 1.208979
1 -1.900965 -2.819580 -0.510308
1 -0.604565 -3.041126 0.707528
8 2.950497 -1.683487 -1.209695
1 2.265906 -2.341005 -1.011311
1 3.775643 -2.188687 -1.266352
rc_1m5hmC/rc_1m5hmCaby.xyz
6 -2.410485 0.196494 0.093592
6 -0.470666 1.472839 -0.020712
6 0.371972 0.279287 -0.129754
6 -0.290587 -0.959971 -0.106055
1 0.266349 -1.887265 -0.168805
7 -1.604422 -1.026172 0.002636
7 -1.793994 1.391636 0.078873
8 -3.626086 0.035661 0.181727
6 1.861527 0.353730 -0.236716
1 2.128589 0.970233 -1.108984
1 2.249071 0.876422 0.649501
7 0.094194 2.676307 -0.016018
1 -0.496070 3.496712 0.073692
1 1.094385 2.815039 -0.085475
8 2.494300 -0.913108 -0.284064
1 2.435704 -1.272736 -1.183498
6 -2.312819 -2.311782 0.050623

1 -2.874046 -2.365319 0.984915
1 -3.008482 -2.362181 -0.788951
1 -1.582242 -3.115833 -0.008092
8 5.207796 -0.628472 0.486135
1 4.278177 -0.727385 0.196871
1 5.536387 0.143688 0.004140
rc_1m5hmC/rc_1m5hmCaaq.xyz
6 -2.086291 0.054030 0.179322
6 -0.457281 -1.604254 0.130434
6 0.579484 -0.651472 -0.276819
6 0.158192 0.672608 -0.489040
1 0.857028 1.430352 -0.822527
7 -1.098619 1.028113 -0.297585
7 -1.714124 -1.227143 0.350184
8 -3.217088 0.490118 0.387383
6 2.014183 -1.039751 -0.454594
1 2.360516 -1.503122 0.485735
1 2.067362 -1.827155 -1.221178
7 -0.144292 -2.886811 0.289794
1 -0.866561 -3.537398 0.579828
1 0.790511 -3.246399 0.144152
8 2.860922 0.013573 -0.855874
1 2.961107 0.642205 -0.099822
6 -1.554781 2.404568 -0.528997
1 -2.377181 2.384215 -1.245786
1 -1.908967 2.822791 0.414898
1 -0.721428 2.986344 -0.918072
8 2.840105 1.723240 1.278066
1 2.117292 2.337861 1.078323
1 3.627013 2.281367 1.369253
rc_1m5hmC/rc_1m5hmCabj.xyz
6 2.074585 0.005406 0.170754
6 0.393067 1.610156 0.119885
6 -0.611359 0.623566 -0.287220
6 -0.148074 -0.686284 -0.499144
1 -0.822497 -1.465530 -0.833506

7 1.119281 -1.000610 -0.305097
7 1.661996 1.274868 0.338792
8 3.218971 -0.393086 0.379859
6 -2.057519 0.966130 -0.460067
1 -2.413547 1.424089 0.479317
1 -2.138995 1.747265 -1.230647
7 0.035149 2.880700 0.281607
1 0.733258 3.556076 0.574063
1 -0.910098 3.209867 0.131316
8 -2.874428 -0.114358 -0.851354
1 -2.943678 -0.742170 -0.091831
6 1.618485 -2.362474 -0.531984
1 2.443280 -2.319366 -1.244885
1 1.979391 -2.768982 0.414464
1 0.804640 -2.969088 -0.924078
8 -2.782762 -1.801819 1.313840
1 -1.951754 -1.529890 1.733434
1 -2.604346 -2.691875 0.973137
rc_1m5hmC/rc_1m5hmCaaj.xyz
6 2.160166 -0.130128 0.147379
6 0.568420 1.565102 0.148061
6 -0.496439 0.642184 -0.254714
6 -0.129017 -0.707304 -0.393799
1 -0.860565 -1.456441 -0.670618
7 1.116072 -1.099798 -0.198174
7 1.825319 1.161881 0.313931
8 3.297762 -0.581400 0.268641
6 -1.902074 1.087319 -0.518131
1 -2.294548 1.549781 0.404679
1 -1.871633 1.888139 -1.272106
7 0.283266 2.848442 0.347890
1 1.024808 3.480270 0.630235
1 -0.648936 3.226683 0.238517
8 -2.754922 0.071021 -0.994207
1 -3.014013 -0.515312 -0.240738
6 1.508601 -2.508820 -0.328352

1 2.241876 -2.599807 -1.131669
1 1.961662 -2.832553 0.610031
1 0.621063 -3.098316 -0.550749
8 -3.349051 -1.584858 1.094062
1 -3.261516 -1.088002 1.921949
1 -4.279051 -1.856350 1.064297
rc_1m5hmC/rc_1m5hmCacu.xyz
6 2.125467 0.045501 -0.122107
6 0.468425 -1.584733 -0.150891
6 -0.558428 -0.626127 0.266418
6 -0.141540 0.707619 0.415451
1 -0.843692 1.482400 0.698728
7 1.117015 1.054315 0.216743
7 1.742146 -1.231346 -0.301645
8 3.281590 0.451655 -0.226318
6 -1.979024 -1.023198 0.520695
1 -2.377316 -1.481972 -0.401268
1 -1.981465 -1.816675 1.282993
7 0.130228 -2.850277 -0.379173
1 0.846058 -3.504089 -0.677630
1 -0.818208 -3.192311 -0.289874
8 -2.806461 0.023545 0.974978
1 -2.987379 0.635288 0.219096
6 1.562447 2.447548 0.344379
1 2.280653 2.517124 1.163483
1 2.049539 2.743653 -0.586000
1 0.694737 3.074177 0.540758
8 -3.097311 1.734098 -1.129613
1 -3.485197 1.304818 -1.906941
1 -2.190082 1.954697 -1.390864
rc_1m5hmC/rc_1m5hmCabo.xyz
6 -0.274976 -1.719115 0.080251
6 0.832660 0.327649 0.053536
6 -0.431338 1.069376 -0.001910
6 -1.606422 0.302805 -0.073330
1 -2.576005 0.781079 -0.150808

7 -1.559697 -1.017140 -0.055408
7 0.860350 -1.004133 0.114483
8 -0.337626 -2.946092 0.150767
6 -0.488697 2.565546 0.004362
1 0.062704 2.936071 0.883072
1 0.047203 2.935897 -0.881733
7 1.983725 0.987691 0.046334
1 2.875729 0.470008 0.042548
1 2.016702 1.998966 0.004297
8 -1.799377 3.094646 -0.043790
1 -2.202473 3.024444 0.835710
6 -2.774292 -1.836864 -0.156888
1 -2.677341 -2.496039 -1.021137
1 -2.868471 -2.442743 0.745754
1 -3.635007 -1.180688 -0.271055
8 4.517753 -0.351413 -0.034476
1 4.979931 -0.113612 -0.852413
1 4.398771 -1.311812 -0.088240
rc_1m5hmC/rc_1m5hmCadi.xyz
6 2.049821 0.162981 0.199739
6 0.219493 1.592955 0.089312
6 -0.665295 0.513616 -0.353791
6 -0.066583 -0.742735 -0.552882
1 -0.647285 -1.581886 -0.916849
7 1.217551 -0.930532 -0.311966
7 1.504973 1.382840 0.359968
8 3.221301 -0.119155 0.445377
6 -2.129775 0.713977 -0.587537
1 -2.556725 1.203670 0.303556
1 -2.252897 1.427160 -1.416686
7 -0.270827 2.820570 0.235342
1 0.346849 3.568339 0.532584
1 -1.237912 3.051352 0.045907
8 -2.833855 -0.464478 -0.910851
1 -2.868309 -1.017161 -0.094059
6 1.863278 -2.230220 -0.539356

1 2.673982 -2.097246 -1.257680
1 2.274420 -2.591026 0.404626
1 1.120795 -2.925719 -0.925611
8 -2.578610 -1.760649 1.503970
1 -2.367488 -1.010979 2.081643
1 -1.743636 -2.245837 1.418296
rc_1m5hmC/rc_1m5hmCadk.xyz
6 -1.222694 0.372638 0.009848
6 0.408578 -1.279497 -0.024914
6 1.488181 -0.288942 -0.007682
6 1.096254 1.061039 -0.021606
1 1.835794 1.853214 -0.035471
7 -0.180132 1.399875 -0.024675
7 -0.873077 -0.922165 -0.000555
8 -2.383319 0.787529 0.042779
6 2.934374 -0.669983 0.005010
1 3.116368 -1.336759 0.862539
1 3.141714 -1.262189 -0.898267
7 0.702301 -2.574484 -0.064201
1 -0.055591 -3.249336 -0.082147
1 1.651209 -2.927032 -0.086656
8 3.822557 0.429980 0.003201
1 3.893016 0.784483 0.903126
6 -0.605575 2.805142 -0.071256
1 -1.252186 2.945042 -0.938706
1 -1.167746 3.035318 0.835674
1 0.278289 3.435331 -0.144531
8 -4.498243 -1.151807 0.041887
1 -3.787055 -0.481237 0.049577
1 -5.320122 -0.647415 0.121540
rc_1m5hmC/rc_1m5hmCacg.xyz
6 -1.146505 -0.594875 -0.486507
6 1.016117 -1.321930 -0.049038
6 1.514918 0.050993 0.060172
6 0.578481 1.073876 -0.166604
1 0.877301 2.114716 -0.133691

7 -0.683355 0.796349 -0.437923
7 -0.264106 -1.588452 -0.298977
8 -2.347828 -0.757905 -0.707063
6 2.942596 0.371324 0.376374
1 3.203516 -0.098904 1.338312
1 3.579475 -0.100569 -0.385221
7 1.859281 -2.337317 0.103883
1 1.509732 -3.286875 0.028346
1 2.844494 -2.207246 0.297766
8 3.237878 1.753990 0.374418
1 2.927323 2.149885 1.203655
6 -1.663735 1.855725 -0.713884
1 -2.095552 1.681360 -1.700757
1 -2.453652 1.811481 0.037731
1 -1.157620 2.818477 -0.680248
8 -4.223721 0.152073 1.345555
1 -3.643306 -0.149407 0.624264
1 -4.756315 -0.622927 1.574607
rc_1m5hmC/rc_1m5hmCaav.xyz
6 2.404984 0.194565 -0.104495
6 0.468213 1.473490 0.026977
6 -0.374695 0.281315 0.146295
6 0.284981 -0.959196 0.112109
1 -0.272890 -1.885673 0.179342
7 1.597557 -1.027074 -0.010107
7 1.790531 1.390706 -0.084126
8 3.619700 0.031762 -0.200732
6 -1.861967 0.357612 0.277978
1 -2.114342 0.973767 1.154757
1 -2.265227 0.880710 -0.601008
7 -0.096043 2.677139 0.024824
1 0.493641 3.497125 -0.072362
1 -1.095464 2.816453 0.103677
8 -2.493061 -0.909717 0.334803
1 -2.427730 -1.265477 1.235158
6 2.302902 -2.313677 -0.069223

1 2.857731 -2.363703 -1.007501
1 3.004028 -2.370018 0.765442
1 1.570908 -3.116315 -0.009642
8 -5.211066 -0.617109 -0.421539
1 -5.191224 0.028857 -1.141902
1 -4.274841 -0.725256 -0.158196
rc_1m5hmC/rc_1m5hmCabg.xyz
6 1.067107 0.697416 -0.120928
6 -1.171299 1.304217 0.003344
6 -1.606590 -0.093624 -0.004412
6 -0.592302 -1.063780 -0.076788
1 -0.834531 -2.119846 -0.087477
7 0.680199 -0.715269 -0.133238
7 0.114637 1.642072 -0.055784
8 2.275104 0.932512 -0.172661
6 -3.047770 -0.490063 0.055006
1 -3.516320 -0.002002 0.923986
1 -3.551434 -0.089305 -0.836624
7 -2.081223 2.269376 0.075796
1 -1.777200 3.237639 0.080490
1 -3.074490 2.082383 0.133344
8 -3.259244 -1.887956 0.069185
1 -3.081163 -2.225478 0.960742
6 1.750473 -1.720027 -0.191998
1 2.377134 -1.518335 -1.061207
1 2.352406 -1.639883 0.715257
1 1.297220 -2.706487 -0.265159
8 4.907616 -0.182883 0.222960
1 5.043978 -0.193701 1.180854
1 3.983584 0.103553 0.107165
rc_1m5hmC/rc_1m5hmCadl.xyz
6 -2.056317 -0.135298 0.217345
6 -0.247259 -1.590795 0.091769
6 0.646734 -0.525952 -0.367632
6 0.061143 0.734937 -0.574662
1 0.648929 1.563197 -0.952236

7 -1.217161 0.941725 -0.318445
7 -1.526079 -1.361502 0.377980
8 -3.219199 0.165572 0.480533
6 2.108760 -0.741428 -0.602020
1 2.529950 -1.237711 0.287988
1 2.224572 -1.454042 -1.432834
7 0.228467 -2.824270 0.236970
1 -0.394448 -3.561830 0.548339
1 1.189801 -3.069040 0.036305
8 2.824140 0.431260 -0.920712
1 2.867423 0.980187 -0.100899
6 -1.847594 2.248458 -0.547170
1 -2.676453 2.119933 -1.245137
1 -2.230295 2.627291 0.401802
1 -1.103237 2.927323 -0.958416
8 2.585717 1.772971 1.456676
1 3.264197 1.531118 2.105023
1 1.764905 1.383945 1.796789
rc_1m5hmC/rc_1m5hmCabc.xyz
6 -2.021122 -0.216360 0.179278
6 -0.154387 -1.600161 0.086956
6 0.705827 -0.500519 -0.354110
6 0.075608 0.738906 -0.565047
1 0.636893 1.590886 -0.929448
7 -1.213809 0.896001 -0.331502
7 -1.446595 -1.421320 0.347882
8 -3.200872 0.037190 0.416798
6 2.178478 -0.659637 -0.571114
1 2.606672 -1.159756 0.312946
1 2.331442 -1.347673 -1.416176
7 0.364545 -2.814704 0.242253
1 -0.237039 -3.576114 0.537860
1 1.338566 -3.021650 0.060538
8 2.856965 0.544248 -0.856362
1 2.829371 1.095893 -0.040458
6 -1.891137 2.178635 -0.566317

1 -2.691067 2.024611 -1.292429
1 -2.321595 2.528141 0.373272
1 -1.163241 2.893379 -0.945223
8 2.330379 1.812306 1.560037
1 1.654684 2.470848 1.336524
1 1.834942 1.086451 1.970509
rc_1m5hmC/rc_1m5hmCact.xyz
6 -0.377264 1.718453 -0.053489
6 0.841545 -0.264333 -0.014909
6 -0.379064 -1.076995 0.009399
6 -1.596197 -0.375950 0.045070
1 -2.538434 -0.908623 0.092975
7 -1.624239 0.944277 0.029358
7 0.795898 1.067491 -0.063722
8 -0.505656 2.941384 -0.105417
6 -0.357884 -2.575653 0.010483
1 0.233307 -2.920467 -0.853144
1 0.176489 -2.913053 0.910539
7 2.028669 -0.855397 0.010194
1 2.892521 -0.291217 0.008354
1 2.121388 -1.862843 0.044854
8 -1.640277 -3.172114 0.031282
1 -2.029799 -3.122609 -0.856003
6 -2.886690 1.692362 0.099875
1 -2.875283 2.311232 0.999109
1 -2.966176 2.335939 -0.777405
1 -3.712946 0.984767 0.134027
8 4.495023 0.590803 0.020901
1 5.122932 0.162290 -0.580089
1 4.914307 0.542091 0.893267
rc_1m5hmC/rc_1m5hmCade.xyz
6 -1.645545 0.335033 -0.550766
6 0.525996 1.167295 -0.592524
6 1.091954 -0.142233 -0.259023
6 0.174195 -1.188856 -0.067701
1 0.515463 -2.182380 0.197868

7 -1.123594 -0.993431 -0.211562
7 -0.783075 1.350680 -0.739062
8 -2.867551 0.428816 -0.645425
6 2.562954 -0.366251 -0.100466
1 3.067975 -0.062772 -1.031531
1 2.931317 0.307868 0.686374
7 1.336775 2.208202 -0.753966
1 0.937584 3.113680 -0.977237
1 2.343024 2.140929 -0.666320
8 2.910834 -1.684026 0.275869
1 2.880326 -2.257209 -0.505828
6 -2.093320 -2.079073 -0.013122
1 -2.750312 -1.816745 0.818219
1 -2.688631 -2.187333 -0.920923
1 -1.549847 -2.996876 0.202789
8 -0.611286 1.178472 2.770943
1 -0.885582 1.693661 1.998136
1 0.034568 0.547428 2.421102
rc_1m5hmC/rc_1m5hmCaal.xyz
6 1.058745 0.743851 -0.052415
6 -1.196060 1.296879 0.013448
6 -1.598174 -0.111069 0.001300
6 -0.558649 -1.056659 -0.014731
1 -0.774971 -2.118685 -0.015049
7 0.706450 -0.678333 -0.028886
7 0.082292 1.665332 -0.022911
8 2.260886 1.008041 -0.099467
6 -3.032185 -0.537893 -0.000263
1 -3.530354 -0.109929 0.884128
1 -3.521992 -0.093992 -0.878742
7 -2.129453 2.240791 0.062949
1 -1.848333 3.215879 0.068025
1 -3.119477 2.031783 0.090992
8 -3.217873 -1.937426 -0.076754
1 -3.091488 -2.326178 0.802425
6 1.800736 -1.658908 -0.031884

1 2.395312 -1.520141 -0.936035
1 2.427074 -1.485372 0.844543
1 1.372004 -2.658304 -0.002942
8 4.890499 -0.153536 0.102172
1 3.981325 0.191460 0.039494
1 4.781308 -1.114892 0.124767
rc_1m5hmC/rc_1m5hmCabk.xyz
6 2.117350 -0.166064 -0.115076
6 0.585725 1.583256 -0.167562
6 -0.521834 0.698449 0.207170
6 -0.202977 -0.659827 0.379619
1 -0.965187 -1.383244 0.642178
7 1.034538 -1.093688 0.231369
7 1.829815 1.133968 -0.304825
8 3.239473 -0.658355 -0.213169
6 -1.918171 1.200885 0.405495
1 -1.897400 2.010213 1.152781
1 -2.267964 1.648632 -0.535331
7 0.351170 2.875629 -0.375532
1 1.121967 3.482223 -0.634033
1 -0.569198 3.286942 -0.286391
8 -2.855358 0.202529 0.765315
1 -2.724838 -0.053261 1.692480
6 1.377135 -2.511959 0.399333
1 1.801527 -2.881682 -0.535671
1 2.119734 -2.603779 1.193651
1 0.472373 -3.060683 0.654458
8 -3.036731 -1.869508 -1.167198
1 -3.042765 -1.163550 -0.490347
1 -3.959347 -1.958938 -1.444297
rc_1m5hmC/rc_1m5hmCaao.xyz
6 1.058447 0.745472 0.060990
6 -1.196830 1.295926 -0.001663
6 -1.596401 -0.112450 -0.021060
6 -0.556555 -1.057251 0.001120
1 -0.772305 -2.119166 -0.016997

7 0.707532 -0.677478 0.042862
7 0.080860 1.666060 0.037375
8 2.260579 1.010936 0.099124
6 -3.028986 -0.539917 -0.075643
1 -3.571698 -0.070254 0.759452
1 -3.471259 -0.140271 -0.999720
7 -2.133190 2.237896 -0.024956
1 -1.855414 3.213916 -0.018211
1 -3.122390 2.025243 -0.056265
8 -3.213205 -1.941613 -0.091249
1 -3.118227 -2.283842 0.810801
6 1.802937 -1.656627 0.063491
1 2.415545 -1.514558 -0.828448
1 2.411195 -1.484226 0.952438
1 1.375456 -2.656880 0.079162
8 4.886894 -0.155006 -0.135103
1 4.772261 -1.115834 -0.155066
1 3.980602 0.195058 -0.059690
rc_1m5hmU/rc_1m5hmUabn.xyz
6 1.484817 -0.401799 -0.054536
6 -0.615756 0.848571 0.035384
6 -1.342834 -0.427253 0.051676
6 -0.597946 -1.618590 0.081007
1 -1.099097 -2.579828 0.136703
7 0.725577 -1.625657 0.016985
7 0.755946 0.753460 -0.046677
8 2.699552 -0.443711 -0.115364
8 -1.182237 1.945191 0.095614
1 1.317043 1.639947 -0.046283
6 -2.825128 -0.504974 0.135442
1 -3.181600 -1.383016 -0.418001
1 -3.041721 -0.727376 1.204533
8 -3.558631 0.618840 -0.296272
1 -3.048887 1.425107 -0.091757
6 1.504887 -2.876233 -0.000732
1 2.107024 -2.896211 -0.910290

1 2.159084 -2.893219 0.872557
1 0.814369 -3.716528 0.019638
8 2.347505 3.003966 -0.037181
1 2.257933 3.481865 0.801821
1 3.247446 2.642013 -0.024878
rc_1m5hmU/rc_1m5hmUaab.xyz
6 1.924040 0.804558 -0.116201
6 -0.455748 0.924214 0.512924
6 -0.476892 -0.544645 0.562083
6 0.689532 -1.246204 0.209553
1 0.695976 -2.331492 0.196270
7 1.824862 -0.631251 -0.089659
7 0.772211 1.479155 0.193049
8 2.976029 1.342128 -0.398065
8 -1.432862 1.634460 0.743973
1 0.833665 2.496095 0.157007
6 -1.744387 -1.288998 0.852532
1 -1.538301 -2.224885 1.373749
1 -2.437557 -0.680299 1.436639
8 -2.265277 -1.599577 -0.445959
1 -2.735980 -0.790758 -0.789937
6 3.058766 -1.374798 -0.400784
1 3.390814 -1.100248 -1.403192
1 3.823906 -1.097339 0.325941
1 2.839824 -2.438491 -0.345047
8 -3.472958 0.704511 -1.151374
1 -3.049197 1.248238 -0.463987
1 -4.413975 0.686910 -0.920421
rc_1m5hmU/rc_1m5hmUaav.xyz
6 1.920491 -0.790455 0.099812
6 -0.455911 -0.937720 -0.534946
6 -0.492667 0.530332 -0.585450
6 0.658712 1.245556 -0.211700
1 0.648128 2.330548 -0.184334
7 1.798745 0.643709 0.095434
7 0.779714 -1.478754 -0.221435

8 2.979862 -1.315723 0.377153
8 -1.428101 -1.657256 -0.758447
1 0.854930 -2.495104 -0.194084
6 -1.762844 1.262220 -0.894748
1 -1.559926 2.183080 -1.443495
1 -2.454119 0.635117 -1.461361
8 -2.286349 1.611847 0.392579
1 -2.736888 0.806996 0.769534
6 3.011564 1.400634 0.453451
1 3.310346 1.121354 1.465237
1 3.806478 1.140746 -0.247001
1 2.780617 2.461819 0.399421
8 -3.485972 -0.685784 1.144418
1 -3.234084 -0.974357 2.034610
1 -2.976297 -1.252964 0.539351
rc_1m5hmU/rc_1m5hmUabw.xyz
6 -1.802828 -0.700095 -0.089100
6 0.592262 -1.169373 0.237540
6 0.782797 0.246673 0.586933
6 -0.340044 1.092899 0.607639
1 -0.234635 2.139638 0.872748
7 -1.554115 0.665477 0.295475
7 -0.707990 -1.523039 -0.081685
8 -2.923824 -1.057862 -0.390406
8 1.505004 -1.991506 0.228170
1 -0.882827 -2.494804 -0.335977
6 2.146143 0.740110 0.969838
1 2.127059 1.823851 1.093468
1 2.408751 0.286598 1.937208
8 3.134597 0.441826 -0.017172
1 3.309076 -0.513618 -0.004495
6 -2.722201 1.564885 0.280993
1 -3.112232 1.611794 -0.737152
1 -3.484001 1.157728 0.947314
1 -2.405015 2.549734 0.617104
8 1.193459 1.071893 -2.002086

1 2.055458 0.823631 -1.614196
1 0.842440 0.252973 -2.383663
rc_1m5hmU/rc_1m5hmUacn.xyz
6 1.563976 0.274395 0.074464
6 -0.651381 -0.755744 -0.012456
6 -1.247631 0.586649 -0.046689
6 -0.386255 1.697929 -0.079493
1 -0.783549 2.706212 -0.144679
7 0.930864 1.568023 -0.015511
7 0.723129 -0.800570 0.075617
8 2.776480 0.197410 0.141202
8 -1.324000 -1.791077 -0.064117
1 1.172208 -1.747255 0.057429
6 -2.715279 0.801177 -0.152771
1 -2.995584 1.721903 0.373949
1 -2.896244 1.015545 -1.229917
8 -3.556285 -0.237595 0.295570
1 -3.130606 -1.092927 0.098151
6 1.833539 2.730625 -0.024552
1 2.410353 2.731353 0.901734
1 2.510629 2.638786 -0.875146
1 1.235515 3.635163 -0.104105
8 1.763898 -3.345130 -0.174837
1 0.972625 -3.904154 -0.218477
1 2.264137 -3.675838 0.586761
rc_1m5hmU/rc_1m5hmUadc.xyz
6 -2.239386 -0.626771 0.208269
6 0.147430 -1.060991 -0.198260
6 0.360193 0.384187 -0.315606
6 -0.750469 1.236226 -0.175017
1 -0.631058 2.311904 -0.260736
7 -1.971361 0.777947 0.060609
7 -1.149104 -1.447192 0.077062
8 -3.365841 -1.020575 0.432482
8 1.049504 -1.893878 -0.328295
1 -1.331736 -2.446559 0.167680

6 1.694272 0.991886 -0.528655
1 1.613546 1.758001 -1.318591
1 1.928059 1.569779 0.389763
8 2.768291 0.129412 -0.828853
1 2.477229 -0.800636 -0.731107
6 -3.132988 1.677301 0.198618
1 -3.878704 1.398630 -0.546894
1 -3.549834 1.554324 1.199614
1 -2.797535 2.700404 0.046069
8 4.777289 0.296441 1.187573
1 4.126852 0.267212 0.458022
1 5.578186 0.669441 0.793142
rc_1m5hmU/rc_1m5hmUaam.xyz
6 -2.391490 -0.459771 -0.056859
6 -0.050320 -1.205799 0.095830
6 0.364208 0.205955 0.101446
6 -0.632926 1.198613 0.086486
1 -0.368167 2.250301 0.128158
7 -1.922016 0.903487 0.007149
7 -1.414786 -1.417820 -0.005005
8 -3.579237 -0.692467 -0.143995
8 0.738523 -2.143987 0.189167
1 -1.737743 -2.385188 -0.022040
6 1.800878 0.588654 0.230186
1 1.950165 1.609539 -0.131407
1 2.019159 0.600211 1.316074
8 2.713882 -0.246188 -0.462130
1 2.600935 -1.164178 -0.160372
6 -2.956726 1.951130 -0.030923
1 -3.533449 1.838247 -0.950157
1 -3.612963 1.820681 0.831320
1 -2.463055 2.919332 -0.000152
8 5.291798 0.824774 -0.003450
1 5.949176 0.116627 -0.055539
1 4.432079 0.385027 -0.164627
rc_1m5hmU/rc_1m5hmUaaq.xyz

6 1.302130 0.376255 -0.084412
6 -0.747294 -0.984039 -0.137251
6 -1.528965 0.249659 -0.005005
6 -0.841225 1.475260 0.019816
1 -1.388551 2.410535 0.079738
7 0.481898 1.551114 -0.017690
7 0.625831 -0.811895 -0.150746
8 2.515597 0.475822 -0.082948
8 -1.251379 -2.104148 -0.236123
1 1.204154 -1.650815 -0.214093
6 -3.012895 0.264225 0.009517
1 -3.369455 1.047626 0.692137
1 -3.304787 0.622605 -1.004687
8 -3.680122 -0.935553 0.319331
1 -3.129410 -1.691411 0.041461
6 1.194125 2.842003 0.047177
1 1.796818 2.861508 0.956725
1 1.842890 2.926691 -0.825537
1 0.456316 3.640928 0.057275
8 3.886581 -2.094934 0.308868
1 3.559314 -1.194821 0.137056
1 4.617948 -2.217049 -0.313099
rc_1m5hmU/rc_1m5hmUada.xyz
6 2.442824 0.662783 -0.008762
6 0.006894 1.022216 0.037444
6 -0.174751 -0.435171 0.007924
6 0.968904 -1.252881 0.034035
1 0.872922 -2.334145 0.056083
7 2.197929 -0.756328 0.019268
7 1.322185 1.450538 -0.001563
8 3.579110 1.089900 -0.033078
8 -0.925817 1.823081 0.104050
1 1.488788 2.456442 0.004013
6 -1.518195 -1.071117 0.068425
1 -1.496009 -2.035202 -0.452327
1 -1.667116 -1.318589 1.143411

8 -2.609348 -0.330442 -0.429136
1 -2.520944 0.597161 -0.146726
6 3.393840 -1.619129 0.025339
1 3.979353 -1.411749 -0.871749
1 3.987628 -1.385192 0.910542
1 3.072716 -2.658246 0.042831
8 -6.252024 -0.197350 0.076912
1 -6.646935 0.515989 -0.445247
1 -6.763656 -0.207668 0.898802
rc_1m5hmU/rc_1m5hmUacb.xyz
6 1.064907 0.645019 0.025519
6 -1.372823 0.981980 0.060505
6 -1.540396 -0.476101 0.032208
6 -0.388561 -1.281704 0.065474
1 -0.473995 -2.363824 0.089762
7 0.836568 -0.773569 0.056964
7 -0.060738 1.422334 0.030193
8 2.195028 1.093733 0.001459
8 -2.312846 1.775215 0.117352
1 0.096606 2.429934 0.033075
6 -2.874600 -1.128500 0.082464
1 -2.845802 -2.075698 -0.470028
1 -3.007748 -1.419221 1.149415
8 -3.982849 -0.387031 -0.371733
1 -3.870863 0.546189 -0.115271
6 2.038833 -1.628986 0.071049
1 2.610049 -1.442194 -0.839531
1 2.640497 -1.369246 0.943291
1 1.723905 -2.669006 0.118102
8 4.988396 0.201720 -0.156471
1 4.981518 -0.766167 -0.155853
1 4.049039 0.448518 -0.111236
rc_1m5hmU/rc_1m5hmUaaj.xyz
6 -1.801869 0.705927 0.055652
6 0.600797 1.115768 -0.291221
6 0.771686 -0.324209 -0.540055

6 -0.364872 -1.150265 -0.522003
1 -0.276421 -2.212023 -0.728104
7 -1.574175 -0.684434 -0.241622
7 -0.693771 1.508884 0.004253
8 -2.919345 1.101388 0.322658
8 1.522035 1.925998 -0.349524
1 -0.853538 2.497170 0.196574
6 2.120437 -0.864301 -0.912047
1 2.093763 -1.955285 -0.909842
1 2.335732 -0.530687 -1.939492
8 3.157403 -0.464391 -0.018649
1 3.300937 0.491781 -0.114070
6 -2.759801 -1.559151 -0.193195
1 -3.178404 -1.521487 0.813739
1 -3.494941 -1.190689 -0.910261
1 -2.454328 -2.572530 -0.444188
8 1.227825 -0.589125 2.082886
1 2.122359 -0.585537 1.690776
1 0.978850 -1.525426 2.124697
rc_1m5hmU/rc_1m5hmUaah.xyz
6 -1.396650 -1.126668 0.053808
6 0.813098 -0.054428 0.161291
6 0.155786 1.247607 0.020315
6 -1.247981 1.281062 -0.059376
1 -1.771013 2.229311 -0.134864
7 -1.986502 0.180597 -0.052434
7 -0.028620 -1.147306 0.151454
8 -2.095116 -2.119591 0.055181
8 2.035229 -0.185400 0.284674
1 0.399892 -2.069943 0.235549
6 0.904053 2.529969 0.066137
1 0.420960 3.265931 -0.589335
1 0.738464 2.915352 1.098648
8 2.278948 2.500624 -0.234790
1 2.653079 1.650601 0.062001
6 -3.457158 0.220629 -0.143963

1 -3.766752 -0.342000 -1.026237
1 -3.872314 -0.244483 0.751488
1 -3.772529 1.258662 -0.219091
8 3.098177 -2.887529 -0.149728
1 3.083782 -2.984544 -1.112654
1 2.827502 -1.965783 0.009391
rc_1m5hmU/rc_1m5hmUacs.xyz
6 2.389824 0.416153 -0.054329
6 0.075049 1.241808 0.098936
6 -0.386124 -0.155386 0.099122
6 0.576204 -1.181533 0.080476
1 0.275021 -2.223358 0.115731
7 1.874889 -0.930943 0.005975
7 1.446348 1.406590 -0.002291
8 3.584748 0.609115 -0.140091
8 -0.681851 2.205253 0.194621
1 1.803700 2.361763 -0.017127
6 -1.834122 -0.494640 0.221480
1 -2.009861 -1.511106 -0.141221
1 -2.059399 -0.499563 1.305686
8 -2.719283 0.364333 -0.477975
1 -2.602408 1.275752 -0.159269
6 2.874202 -2.012701 -0.029329
1 3.442446 -1.930930 -0.957377
1 3.545426 -1.891137 0.822463
1 2.350072 -2.964052 0.021645
8 -5.273632 -0.782483 -0.024853
1 -4.423256 -0.327530 -0.190726
1 -5.020460 -1.671321 0.262655
rc_1m5hmU/rc_1m5hmUacm.xyz
6 1.389293 -1.142685 -0.053862
6 -0.809402 -0.046509 -0.156571
6 -0.137408 1.248140 -0.018289
6 1.266658 1.265864 0.060063
1 1.799657 2.208626 0.134609
7 1.993318 0.157778 0.052059

7 0.020656 -1.148433 -0.148053
8 2.076895 -2.143191 -0.057813
8 -2.033365 -0.163871 -0.276410
1 -0.416525 -2.066837 -0.230588
6 -0.869140 2.539825 -0.060521
1 -0.386108 3.261881 0.610975
1 -0.682492 2.936905 -1.084969
8 -2.248819 2.525432 0.217433
1 -2.623164 1.669963 -0.064054
6 3.464642 0.183049 0.140026
1 3.771026 -0.384695 1.020075
1 3.872811 -0.283969 -0.757679
1 3.790468 1.217752 0.217118
8 -3.068009 -2.844766 0.278329
1 -3.999448 -2.971903 0.048837
1 -2.865510 -1.928073 0.020250
rc_1m5hmU/rc_1m5hmUadn.xyz
6 -2.218532 -0.168045 -0.264621
6 -0.116818 -1.441030 -0.109127
6 0.546771 -0.225936 0.368182
6 -0.222321 0.940648 0.512769
1 0.235676 1.855651 0.875609
7 -1.513704 0.986719 0.214016
7 -1.462471 -1.305692 -0.391521
8 -3.402957 -0.105481 -0.529304
8 0.461364 -2.522622 -0.252215
1 -1.955191 -2.131430 -0.730893
6 1.975093 -0.176376 0.748198
1 2.430673 0.724506 0.297248
1 2.001249 0.032282 1.840353
8 2.782788 -1.289207 0.452592
1 2.215112 -2.049071 0.215443
6 -2.309488 2.219579 0.365817
1 -2.761293 2.463406 -0.596437
1 -3.092336 2.039806 1.104419
1 -1.648794 3.018821 0.693699

8 3.793808 2.076907 -1.041481
1 4.007559 2.763246 -0.392706
1 4.392320 1.345787 -0.828244
rc_1m5hmU/rc_1m5hmUaag.xyz
6 1.130744 -0.561657 -0.529290
6 -1.264125 -0.972658 -0.149070
6 -1.450141 0.471272 0.024484
6 -0.341200 1.315560 -0.164301
1 -0.452127 2.393115 -0.091445
7 0.872034 0.846956 -0.423400
7 0.034864 -1.371352 -0.410992
8 2.261042 -0.969785 -0.719926
8 -2.181840 -1.791541 -0.075632
1 0.208967 -2.371935 -0.505346
6 -2.778295 1.082194 0.284037
1 -2.661124 1.960444 0.932335
1 -3.096853 1.497816 -0.699662
8 -3.787843 0.262193 0.822194
1 -3.666520 -0.650506 0.500861
6 2.034228 1.739157 -0.597384
1 2.729604 1.574521 0.227163
1 2.519219 1.492806 -1.542563
1 1.681112 2.767468 -0.601475
8 4.252567 -0.428510 1.489845
1 3.654892 -0.542272 0.732747
1 4.395867 0.527239 1.545422
rc_1m5hmU/rc_1m5hmUabd.xyz
6 -1.306157 -0.192062 -0.775565
6 1.022043 -0.886367 -0.392953
6 1.300726 0.452645 0.138161
6 0.241434 1.374597 0.215053
1 0.401484 2.367788 0.623141
7 -0.979157 1.088646 -0.214795
7 -0.273394 -1.088193 -0.833980
8 -2.436616 -0.426236 -1.156957
8 1.862997 -1.785674 -0.449879

1 -0.500976 -2.008556 -1.209704
6 2.630985 0.844882 0.670112
1 2.834543 1.893890 0.413518
1 2.508634 0.845757 1.777261
8 3.746354 0.071355 0.297989
1 3.463421 -0.850326 0.150202
6 -2.071847 2.076771 -0.178507
1 -2.382419 2.286514 -1.203707
1 -2.908673 1.652793 0.377897
1 -1.702909 2.977301 0.306406
8 -2.870920 -1.330328 1.926000
1 -3.111094 -2.252115 2.098885
1 -3.151764 -1.171957 1.012493
rc_1m5hmU/rc_1m5hmUabp.xyz
6 -1.794992 -0.700045 -0.106439
6 0.594177 -1.219245 0.183721
6 0.802211 0.166123 0.632945
6 -0.306200 1.031941 0.684685
1 -0.183688 2.062728 0.999786
7 -1.525847 0.639764 0.350769
7 -0.713360 -1.539177 -0.143048
8 -2.920575 -1.022409 -0.429114
8 1.499632 -2.045164 0.102507
1 -0.901045 -2.490022 -0.459747
6 2.182458 0.619358 1.009067
1 2.166018 1.675615 1.279758
1 2.515953 0.038666 1.879623
8 3.098374 0.476483 -0.082026
1 3.305595 -0.465326 -0.195940
6 -2.670803 1.567589 0.351365
1 -3.004691 1.705096 -0.679050
1 -3.476698 1.130437 0.941518
1 -2.347741 2.513615 0.779838
8 1.088064 1.315172 -1.926548
1 0.689056 0.510852 -2.291468
1 1.946629 1.027259 -1.558977

rc_1m5hmU/rc_1m5hmUacp.xyz
6 -1.589297 -1.034214 -0.107026
6 0.864704 -1.225536 -0.142022
6 0.945292 0.130440 0.411387
6 -0.254587 0.802277 0.717435
1 -0.236180 1.787670 1.166933
7 -1.443375 0.267501 0.481640
7 -0.417540 -1.693568 -0.369772
8 -2.693308 -1.486831 -0.336002
8 1.852235 -1.920468 -0.386080
1 -0.511623 -2.627895 -0.766957
6 2.245881 0.771834 0.748152
1 2.154147 1.860486 0.668264
1 2.396422 0.561517 1.830111
8 3.371511 0.372839 -0.003263
1 3.351139 -0.594270 -0.115124
6 -2.687272 1.021074 0.716148
1 -3.167267 1.205285 -0.247200
1 -3.348021 0.424603 1.345985
1 -2.434146 1.959056 1.205094
8 -0.182908 2.768180 -1.366217
1 0.041804 2.000757 -1.913054
1 -1.148436 2.740255 -1.289068
rc_1m5hmU/rc_1m5hmUadp.xyz
6 1.381852 1.146190 -0.095447
6 -0.817012 0.045020 -0.062319
6 -0.134563 -1.248610 0.045362
6 1.270114 -1.257075 0.119970
1 1.807519 -2.193773 0.229811
7 1.992651 -0.148745 0.046335
7 0.011158 1.145536 -0.152357
8 2.066918 2.146272 -0.155578
8 -2.045746 0.162328 -0.063313
1 -0.432078 2.061971 -0.226784
6 -0.875391 -2.529840 0.195098
1 -0.296805 -3.348045 -0.249990

1 -0.870933 -2.731677 1.290534
8 -2.186830 -2.588271 -0.316165
1 -2.633290 -1.738162 -0.150755
6 3.466088 -0.169713 0.091588
1 3.847624 0.253334 -0.839112
1 3.796078 0.440019 0.934087
1 3.795659 -1.199511 0.209244
8 -3.126716 2.864343 0.125430
1 -3.601585 2.953278 0.964076
1 -2.846390 1.931821 0.092537
rc_1m5mC/rc_1m5mCaaj.xyz
6 0.846669 -1.240940 0.043799
6 -0.799604 0.401125 0.030688
6 0.192260 1.482734 0.017087
6 1.541074 1.081971 -0.019397
1 2.337718 1.818063 -0.051276
7 1.877295 -0.194931 -0.022285
7 -0.447179 -0.885896 0.057647
8 1.262042 -2.399557 0.079239
7 -2.089134 0.708066 0.017531
1 -2.801110 -0.037449 0.011756
1 -2.403050 1.670368 -0.000424
6 -0.172862 2.916473 0.025292
1 -0.780615 3.159620 -0.858009
1 -0.790495 3.150172 0.903362
1 0.712909 3.552791 0.031717
6 3.278979 -0.625256 -0.097601
1 3.528725 -1.182855 0.806895
1 3.397130 -1.277201 -0.964455
1 3.911802 0.255147 -0.190963
8 -4.180047 -1.253938 0.011185
1 -4.663807 -1.218619 -0.827740
1 -3.811138 -2.149385 0.046283
rc_1m5mC/rc_1m5mCacs.xyz
6 -0.860209 -1.240479 0.045633
6 0.797129 0.390506 0.032570

6 -0.187315 1.478749 0.012865
6 -1.538752 1.086936 -0.021372
1 -2.330469 1.828170 -0.054623
7 -1.883758 -0.187520 -0.019814
7 0.436021 -0.893987 0.059902
8 -1.283292 -2.396280 0.080510
7 2.088858 0.688520 0.024027
1 2.795790 -0.061476 0.023286
1 2.409559 1.648587 0.004638
6 0.186982 2.910076 0.012816
1 0.805285 3.145503 0.889966
1 0.797316 3.143621 -0.871270
1 -0.694623 3.552192 0.014245
6 -3.288976 -0.607290 -0.089564
1 -3.415262 -1.261353 -0.953603
1 -3.540359 -1.159794 0.817642
1 -3.914985 0.277887 -0.184081
8 4.156560 -1.306397 0.022363
1 4.984305 -0.884367 -0.252838
1 3.976296 -1.967629 -0.662832
rc_1m5mC/rc_1m5mCacf.xyz
6 0.846599 -1.240933 -0.044180
6 -0.799868 0.400909 -0.031657
6 0.191823 1.482659 -0.017364
6 1.540669 1.082095 0.019847
1 2.337193 1.818291 0.052329
7 1.877062 -0.194761 0.022709
7 -0.447284 -0.886088 -0.058623
8 1.262199 -2.399473 -0.079703
7 -2.089440 0.707579 -0.019165
1 -2.801216 -0.038267 -0.013057
1 -2.403554 1.669817 -0.001269
6 -0.173537 2.916338 -0.025437
1 -0.790621 3.150178 -0.903858
1 -0.781940 3.159131 0.857510
1 0.712125 3.552815 -0.031089

6 3.278768 -0.624892 0.098656
1 3.396562 -1.277036 0.965411
1 3.529061 -1.182211 -0.805862
1 3.911412 0.255585 0.192583
8 -4.182165 -1.248847 -0.004255
1 -4.611302 -1.262687 0.864499
1 -3.827084 -2.143212 -0.119172
rc_1m5mC/rc_1m5mCadt.xyz
6 0.047208 -1.253324 -0.030268
6 -0.588024 0.991337 -0.034785
6 0.803461 1.448224 0.015181
6 1.783444 0.435694 0.032389
1 2.838503 0.686706 0.061868
7 1.452504 -0.842883 0.008803
7 -0.905927 -0.302564 -0.054591
8 -0.171806 -2.461660 -0.040276
7 -1.564905 1.889043 -0.062864
1 -2.530545 1.575606 -0.088974
1 -1.381495 2.884733 -0.048571
6 1.176082 2.878357 0.044337
1 0.761738 3.398180 -0.830682
1 0.743810 3.366083 0.929725
1 2.258565 3.008309 0.058981
6 2.467961 -1.904769 0.010573
1 2.301839 -2.548996 0.875088
1 2.360889 -2.492159 -0.902935
1 3.452318 -1.444303 0.058341
8 -3.722041 -0.910740 -0.005147
1 -2.746374 -0.797749 0.003283
1 -3.920962 -1.435499 0.783256
rc_1m5mC/rc_1m5mCacd.xyz
6 0.851699 -1.240665 -0.043151
6 -0.798788 0.397183 -0.030594
6 0.190192 1.481385 -0.015613
6 1.540034 1.084096 0.019474
1 2.334804 1.822193 0.050943

7 1.879625 -0.191882 0.021229
7 -0.443080 -0.888946 -0.056884
8 1.270079 -2.398224 -0.077523
7 -2.089131 0.700605 -0.019201
1 -2.799019 -0.046832 -0.015425
1 -2.405792 1.662028 -0.001553
6 -0.178556 2.914186 -0.021583
1 -0.796169 3.147901 -0.899680
1 -0.787623 3.154207 0.861646
1 0.705570 3.552808 -0.026290
6 3.282740 -0.618058 0.093885
1 3.404580 -1.270096 0.960128
1 3.532585 -1.174442 -0.811344
1 3.912988 0.264278 0.186544
8 -4.173927 -1.268537 -0.023536
1 -3.827671 -2.150007 0.181937
1 -4.799293 -1.075145 0.691045
rc_1m5mC/rc_1m5mCaab.xyz
6 0.839639 -1.241049 -0.046770
6 -0.801279 0.406258 -0.033764
6 0.194065 1.484619 -0.018174
6 1.541529 1.079531 0.021076
1 2.340447 1.813069 0.055556
7 1.873550 -0.198501 0.023753
7 -0.453038 -0.881894 -0.062224
8 1.251391 -2.400908 -0.084061
7 -2.089830 0.717351 -0.020986
1 -2.803617 -0.026580 -0.013930
1 -2.400802 1.680559 -0.001814
6 -0.166662 2.919488 -0.026136
1 -0.782708 3.155508 -0.904693
1 -0.774476 3.164024 0.856755
1 0.721083 3.553047 -0.031317
6 3.273416 -0.633896 0.102746
1 3.386534 -1.287027 0.969415
1 3.523787 -1.191557 -0.801530

1 3.909188 0.244078 0.198806
8 -4.181020 -1.242926 0.016854
1 -3.845629 -2.093605 -0.304266
1 -4.435793 -1.409246 0.937011
rc_1m5mC/rc_1m5mCabs.xyz
6 -0.897328 0.329071 -0.010741
6 0.894139 -1.145744 -0.018962
6 1.872029 -0.054282 0.025479
6 1.338866 1.249829 0.024852
1 1.991847 2.116157 0.039144
7 0.035072 1.457085 0.000006
7 -0.419292 -0.924415 -0.026104
8 -2.094360 0.625260 -0.008139
7 1.335240 -2.397718 -0.051652
1 0.669074 -3.161852 -0.084918
1 2.322468 -2.623135 -0.051534
6 3.332597 -0.280846 0.050024
1 3.608530 -0.916870 0.902389
1 3.645122 -0.821183 -0.855644
1 3.880696 0.659818 0.110164
6 -0.532760 2.812250 -0.033450
1 -1.126970 2.916727 -0.942902
1 -1.179050 2.946077 0.835245
1 0.282914 3.532401 -0.021798
8 -4.121297 -1.385104 0.041455
1 -3.678255 -2.243392 0.100082
1 -3.393523 -0.732321 0.024282
rc_1m5mC/rc_1m5mCace.xyz
6 0.655940 -0.659674 0.036962
6 -1.657234 -0.847392 -0.011473
6 -1.839069 0.607508 -0.004525
6 -0.656535 1.373334 -0.000404
1 -0.700896 2.457290 -0.008354
7 0.533187 0.800784 0.013566
7 -0.453285 -1.414932 0.018194
8 1.801373 -1.112203 0.072274

7 -2.731109 -1.627967 -0.043433
1 -2.613921 -2.635699 -0.048679
1 -3.671825 -1.254874 -0.070695
6 -3.168489 1.255456 0.001066
1 -3.740129 0.958466 -0.890259
1 -3.751408 0.919909 0.870314
1 -3.083095 2.342124 0.023693
6 1.772092 1.591792 0.010032
1 2.350623 1.343575 0.901143
1 2.350105 1.334633 -0.879291
1 1.514424 2.649073 0.004181
8 4.575595 -0.420828 -0.041612
1 4.629791 0.521760 -0.253933
1 3.618805 -0.603338 -0.001652
rc_1m5mC/rc_1m5mCacg.xyz
6 -0.084441 -1.254553 -0.046270
6 0.616387 0.970887 -0.048739
6 -0.760371 1.467837 0.015656
6 -1.768106 0.483945 0.049466
1 -2.814505 0.764928 0.094729
7 -1.476193 -0.803645 0.018876
7 0.895759 -0.331459 -0.077593
8 0.099758 -2.468419 -0.070615
7 1.618684 1.840242 -0.080133
1 2.573982 1.499500 -0.120308
1 1.464225 2.840775 -0.063886
6 -1.095383 2.907242 0.041199
1 -0.639583 3.389898 0.917539
1 -0.679758 3.411107 -0.842552
1 -2.174146 3.064210 0.068982
6 -2.525615 -1.832104 0.030214
1 -2.457969 -2.408658 -0.894132
1 -2.360972 -2.493622 0.881735
1 -3.493357 -1.340175 0.107030
8 3.671094 -1.146289 0.113481
1 2.720707 -0.908049 0.047326

1 4.131990 -0.297750 0.183396
rc_1m5mC/rc_1m5mCaba.xyz
6 0.143631 -1.227558 -0.011364
6 0.434279 1.088055 -0.019342
6 -1.010930 1.330191 0.003683
6 -1.826823 0.182113 0.008977
1 -2.907785 0.274016 0.022573
7 -1.309358 -1.033020 -0.001819
7 0.941558 -0.143464 -0.020986
8 0.543730 -2.388372 -0.010491
7 1.266755 2.121421 -0.039557
1 2.267599 1.952657 -0.056200
1 0.941873 3.080525 -0.034439
6 -1.600094 2.685718 0.025513
1 -1.240085 3.241219 0.903056
1 -1.275625 3.254984 -0.857362
1 -2.689375 2.645927 0.046105
6 -2.151597 -2.237850 0.005338
1 -1.927407 -2.828584 -0.884396
1 -1.917122 -2.825323 0.894626
1 -3.197603 -1.937292 0.010835
8 3.811702 -0.442209 0.071865
1 4.048161 -1.336356 -0.212943
1 2.830435 -0.415696 0.036851
rc_1m5mC/rc_1m5mCadh.xyz
6 -0.141346 -1.231452 -0.021686
6 2.053022 -0.469383 0.017754
6 1.634113 0.935592 -0.008771
6 0.243946 1.161476 -0.026838
1 -0.151866 2.171450 -0.038206
7 -0.613623 0.157557 -0.030397
7 1.181098 -1.474709 0.004976
8 -1.013540 -2.098622 -0.038852
7 3.351369 -0.749569 0.056665
1 3.651409 -1.718067 0.078053
1 4.059951 -0.027273 0.070485

6 2.590855 2.063395 -0.007843
1 3.198666 2.041827 0.908555
1 3.293497 1.973157 -0.847769
1 2.075958 3.022665 -0.070662
6 -2.065284 0.382128 -0.043848
1 -2.487715 -0.107063 -0.922279
1 -2.494403 -0.058276 0.857693
1 -2.252928 1.452876 -0.073202
8 -5.381297 0.651631 0.111222
1 -4.901452 -0.183679 0.210211
1 -6.156167 0.424821 -0.423158
rc_1m5mC/rc_1m5mCaay.xyz
6 0.179254 1.252370 -0.115545
6 -0.688656 -0.914662 -0.088902
6 0.646035 -1.511590 0.022016
6 1.720927 -0.603116 0.098074
1 2.739595 -0.961383 0.201236
7 1.528039 0.702021 0.040428
7 -0.864967 0.403765 -0.165657
8 0.089446 2.474787 -0.189215
7 -1.756754 -1.702498 -0.114781
1 -2.680684 -1.288848 -0.193817
1 -1.686339 -2.711470 -0.070526
6 0.881991 -2.971726 0.038529
1 0.347437 -3.439606 0.877501
1 0.481434 -3.429036 -0.877446
1 1.944684 -3.202144 0.118312
6 2.645438 1.654009 0.115963
1 2.661901 2.250734 -0.797254
1 2.487449 2.311409 0.972486
1 3.572691 1.095798 0.226452
8 -3.581225 1.331792 0.198776
1 -2.643051 1.089386 0.040713
1 -3.945033 0.577799 0.685119
rc_1m5mC/rc_1m5mCabh.xyz
6 0.215130 -1.159650 0.005253

6 -2.023274 -0.534888 -0.000489
6 -1.690682 0.893291 -0.002599
6 -0.316639 1.204027 0.009470
1 0.017643 2.235667 0.012870
7 0.601313 0.255235 0.017019
7 -1.090167 -1.484137 0.001337
8 1.139894 -1.971162 -0.000195
7 -3.302220 -0.896703 -0.001992
1 -3.539284 -1.882920 -0.001506
1 -4.057391 -0.222933 -0.004627
6 -2.713941 1.961251 -0.019818
1 -3.353188 1.858314 -0.908147
1 -3.377078 1.867339 0.851769
1 -2.258738 2.952001 -0.018567
6 2.037125 0.566000 0.028532
1 2.490705 0.093186 0.900152
1 2.488788 0.161193 -0.878854
1 2.163845 1.645912 0.069253
8 5.336115 0.535993 0.020389
1 4.842793 -0.200075 -0.370217
1 6.265037 0.272715 -0.050326
rc_1m5mC/rc_1m5mCabx.xyz
6 0.213425 -1.167913 0.011045
6 -2.020785 -0.528450 -0.004904
6 -1.679086 0.897530 -0.003074
6 -0.302930 1.199298 0.011539
1 0.037433 2.228931 0.015368
7 0.608731 0.244564 0.020566
7 -1.093886 -1.483730 0.000350
8 1.132798 -1.985501 0.013841
7 -3.301929 -0.882036 -0.013614
1 -3.545323 -1.866691 -0.015646
1 -4.052613 -0.203339 -0.018805
6 -2.695277 1.972262 -0.019707
1 -3.331143 1.877667 -0.911471
1 -3.363196 1.878669 0.848207

1 -2.233492 2.959920 -0.011910
6 2.046628 0.545301 0.034069
1 2.492981 0.076239 0.911974
1 2.498281 0.128243 -0.867567
1 2.181539 1.624524 0.065195
8 5.449529 0.490749 -0.055255
1 4.916300 0.606616 0.744808
1 4.878356 -0.012512 -0.653758
rc_1m5mC/rc_1m5mCacl.xyz
6 1.721674 -0.907691 0.003882
6 -0.594895 -0.682283 0.030049
6 -0.513361 0.782836 0.032678
6 0.785273 1.328154 -0.002916
1 0.932955 2.402950 -0.012988
7 1.855173 0.555170 -0.020905
7 0.495373 -1.452549 0.026798
8 2.776478 -1.542958 -0.000406
7 -1.785809 -1.266073 0.032712
1 -1.816864 -2.280337 0.026710
1 -2.683028 -0.762826 0.013806
6 -1.700318 1.664494 0.071877
1 -2.353967 1.464183 -0.787478
1 -2.298963 1.455211 0.968782
1 -1.411271 2.715993 0.067512
6 3.213338 1.113041 -0.055018
1 3.753654 0.773855 0.830306
1 3.721148 0.746266 -0.948505
1 3.145498 2.198837 -0.070159
8 -4.449457 -0.218403 -0.065022
1 -4.500175 0.488288 0.596667
1 -4.528588 0.241313 -0.914786
rc_1m5mC/rc_1m5mCaan.xyz
6 -0.437704 -1.238181 -0.036045
6 0.829452 0.722081 -0.024494
6 -0.367198 1.568422 0.000009
6 -1.600205 0.886583 0.021335

1 -2.533272 1.438844 0.050603
7 -1.661134 -0.432447 0.008308
7 0.751961 -0.609226 -0.049607
8 -0.584924 -2.457399 -0.056839
7 2.030314 1.286966 -0.021260
1 2.858335 0.695288 -0.029424
1 2.153770 2.291752 -0.004393
6 -0.312139 3.047111 0.007539
1 0.240627 3.399843 0.890400
1 0.240797 3.410531 -0.870407
1 -1.311467 3.483493 0.011405
6 -2.942502 -1.151877 0.047234
1 -3.012599 -1.793230 -0.832586
1 -2.967892 -1.769919 0.946556
1 -3.751950 -0.424704 0.058481
8 3.462666 -1.563692 -0.034332
1 3.708150 -1.825827 0.864557
1 2.487351 -1.459231 -0.011386
rc_1m5mC/rc_1m5mCabb.xyz
6 -1.714548 -0.919301 -0.005041
6 0.598787 -0.662369 0.002387
6 0.497355 0.801509 0.001127
6 -0.809040 1.329586 0.002881
1 -0.970649 2.402433 0.005771
7 -1.868523 0.542072 0.001275
7 -0.480955 -1.447534 -0.003488
8 -2.760789 -1.568660 -0.010170
7 1.797283 -1.230004 0.008875
1 1.841880 -2.243792 0.008910
1 2.689272 -0.717220 0.019722
6 1.673570 1.698626 -0.005131
1 2.315756 1.493639 0.861661
1 2.287175 1.512031 -0.897239
1 1.371067 2.746095 0.009315
6 -3.235031 1.080360 0.004230
1 -3.754034 0.723435 -0.886838

1 -3.753297 0.715178 0.892395
1 -3.183666 2.167199 0.009226
8 4.440329 -0.154845 0.096244
1 4.490567 0.702751 -0.351895
1 5.028430 -0.735914 -0.408963
rc_1m5mC/rc_1m5mCadj.xyz
6 -0.895855 -1.354362 -0.155000
6 1.371601 -0.843808 -0.140405
6 1.135258 0.470269 0.463988
6 -0.206895 0.774869 0.764963
1 -0.469508 1.718125 1.230894
7 -1.179552 -0.076335 0.500503
7 0.379868 -1.684541 -0.426464
8 -1.872174 -2.056133 -0.416668
7 2.619179 -1.210163 -0.416550
1 2.788144 -2.116626 -0.838674
1 3.414816 -0.616164 -0.219983
6 2.213034 1.445796 0.734349
1 2.970321 1.008821 1.400323
1 2.731637 1.703725 -0.200030
1 1.820788 2.356713 1.188182
6 -2.584728 0.261506 0.763379
1 -3.109561 0.350078 -0.190074
1 -3.037707 -0.538190 1.349965
1 -2.620536 1.203282 1.307125
8 -0.828030 2.731084 -1.500611
1 -1.632893 2.649564 -0.967419
1 -0.184810 3.152718 -0.912140
rc_1m5mC/rc_1m5mCacq.xyz
6 1.452682 -1.127519 -0.020032
6 -0.669444 -0.182504 -0.051670
6 -0.131709 1.181769 -0.016099
6 1.271103 1.289214 0.038114
1 1.750943 2.261241 0.075294
7 2.041425 0.216616 0.040444
7 0.115252 -1.257571 -0.058149

8 2.248805 -2.065057 -0.028798
7 -1.986626 -0.348011 -0.077729
1 -2.384136 -1.280732 -0.088063
1 -2.630553 0.432569 -0.060917
6 -0.987222 2.387180 -0.046640
1 -1.608649 2.389832 -0.953340
1 -1.680610 2.388265 0.806284
1 -0.390072 3.299449 -0.021121
6 3.507005 0.312799 0.080755
1 3.869619 -0.222484 0.959737
1 3.912305 -0.155166 -0.817844
1 3.789266 1.362690 0.125518
8 -4.949105 -0.807782 0.096706
1 -5.021279 0.124918 -0.155210
1 -5.849293 -1.060750 0.347869
rc_1m5mC/rc_1m5mCade.xyz
6 0.739106 -0.485638 -0.557445
6 -1.493695 -0.882386 -0.061851
6 -1.751366 0.535078 0.209002
6 -0.650705 1.400167 0.051931
1 -0.757791 2.465110 0.229827
7 0.534635 0.945139 -0.309586
7 -0.294172 -1.330960 -0.428593
8 1.882103 -0.819896 -0.876663
7 -2.488479 -1.752904 0.064415
1 -2.316213 -2.735167 -0.122248
1 -3.424977 -1.473962 0.331388
6 -3.072247 1.051684 0.626725
1 -3.829536 0.822293 -0.136508
1 -3.399502 0.552316 1.549760
1 -3.044902 2.129229 0.792137
6 1.691896 1.837079 -0.469107
1 2.468722 1.533192 0.233927
1 2.070024 1.742273 -1.488065
1 1.376026 2.859169 -0.270436
8 3.918424 -0.611586 1.232089

1 3.265029 -0.679586 0.513886
1 4.147082 -1.527841 1.443750
rc_1m5mU/rc_1m5mUabi.xyz
6 -0.514640 1.092936 0.001789
6 -0.040735 -1.309863 0.004503
6 1.404118 -0.999239 -0.000730
6 1.790991 0.353773 -0.004390
1 2.841086 0.626665 -0.008267
7 0.908090 1.344231 -0.003707
7 -0.888315 -0.218222 0.005806
8 -1.291385 2.030422 0.002745
8 -0.475678 -2.459665 0.007245
6 2.385236 -2.097607 -0.001023
1 2.226763 -2.732637 0.881971
1 2.209833 -2.748794 -0.868592
1 3.410827 -1.728636 -0.013643
6 1.320259 2.758533 -0.007389
1 0.912795 3.239328 -0.898159
1 0.917613 3.242113 0.884066
1 2.407482 2.802556 -0.010500
1 -1.919190 -0.396702 0.013242
8 -3.636181 -0.593853 0.081850
1 -3.957273 -1.079820 -0.693211
1 -4.033787 0.287438 0.007116
rc_1m5mU/rc_1m5mUadf.xyz
6 -0.524552 -1.087334 -0.002262
6 -0.028853 1.311180 -0.003848
6 1.413213 0.987685 0.000218
6 1.787624 -0.368745 0.004775
1 2.834952 -0.651883 0.009094
7 0.895768 -1.351106 0.004596
7 -0.886648 0.227110 -0.004803
8 -1.309335 -2.018070 -0.005619
8 -0.453022 2.464937 -0.005996
6 2.404971 2.076508 -0.002318
1 2.256257 2.706937 -0.890422

1 2.232805 2.735382 0.859930
1 3.426722 1.697130 0.016181
6 1.295427 -2.768942 0.009951
1 0.882648 -3.245519 0.900529
1 0.890093 -3.249810 -0.881739
1 2.382209 -2.822448 0.014811
1 -1.915803 0.415027 -0.010697
8 -3.631797 0.644465 -0.064458
1 -4.025744 -0.218506 -0.265948
1 -3.961719 0.868897 0.819203
rc_1m5mU/rc_1m5mUabg.xyz
6 0.098143 -1.246670 -0.005568
6 0.483283 1.171976 -0.002879
6 -0.979400 1.380043 0.003463
6 -1.810870 0.244747 0.004194
1 -2.890576 0.351923 0.008810
7 -1.324379 -0.989726 -0.000119
7 0.901485 -0.145290 -0.006959
8 0.502754 -2.394640 -0.008021
8 1.288832 2.100663 -0.004758
6 -1.520493 2.749159 0.009276
1 -1.140304 3.291942 0.886031
1 -1.143708 3.298469 -0.864748
1 -2.610551 2.754672 0.011629
6 -2.198232 -2.175641 0.001902
1 -1.986300 -2.768499 -0.889470
1 -1.981301 -2.769143 0.891676
1 -3.234546 -1.844920 0.005043
1 1.932498 -0.321852 -0.013777
8 3.655251 -0.485319 -0.080530
1 3.988720 -0.965198 0.693034
1 4.017038 0.410399 0.005461
rc_1m5mU/rc_1m5mUacc.xyz
6 0.122524 -1.242354 0.000942
6 0.460779 1.183464 0.000806
6 -1.005778 1.363756 -0.001099

6 -1.815044 0.212745 -0.000784
1 -2.896621 0.298701 -0.001578
7 -1.304700 -1.011912 0.000534
7 0.905118 -0.125879 0.001092
8 0.547814 -2.382716 0.001039
8 1.247080 2.128349 0.002149
6 -1.573823 2.722159 -0.003533
1 -1.206449 3.277111 0.870952
1 -1.205662 3.273956 -0.879741
1 -2.663786 2.706539 -0.004077
6 -2.156202 -2.213887 0.001127
1 -1.930750 -2.803507 -0.889095
1 -1.931109 -2.802313 0.892236
1 -3.198571 -1.902837 0.000696
1 1.939350 -0.282984 0.001371
8 3.661665 -0.485028 0.011788
1 4.021442 0.086667 0.707440
1 4.022027 -0.126931 -0.814147
rc_1m5mU/rc_1m5mUaaq.xyz
6 -0.490061 1.106375 -0.006091
6 -0.069262 -1.306163 -0.008615
6 1.381976 -1.026726 0.001981
6 1.799189 0.317417 0.006394
1 2.855332 0.566043 0.013375
7 0.937976 1.326723 0.002828
7 -0.892530 -0.196440 -0.012867
8 -1.245662 2.060900 -0.007504
8 -0.529253 -2.446150 -0.013237
6 2.337211 -2.147524 0.009284
1 2.150213 -2.784709 0.885053
1 2.160165 -2.788618 -0.865526
1 3.371164 -1.802788 0.014950
6 1.380146 2.731758 0.006955
1 0.988918 3.223224 -0.885294
1 0.981168 3.221200 0.896875
1 2.467955 2.752559 0.011858

1 -1.926926 -0.351875 -0.022992
8 -3.653362 -0.503517 -0.083686
1 -4.033197 0.313971 0.273951
1 -3.941896 -1.201685 0.523995
rc_1m5mU/rc_1m5mUadj.xyz
6 -0.883627 0.394995 -0.211396
6 0.947232 -1.247297 -0.126556
6 1.891774 -0.134415 0.091905
6 1.378249 1.176372 0.132118
1 2.040212 2.022499 0.283933
7 0.085216 1.438605 -0.012672
7 -0.382813 -0.874189 -0.265692
8 -2.064040 0.674150 -0.318947
8 1.290029 -2.422808 -0.182156
6 3.324419 -0.422482 0.266056
1 3.462528 -1.068427 1.145530
1 3.693012 -1.006772 -0.588138
1 3.911183 0.488642 0.381033
6 -0.438525 2.815572 0.025922
1 -0.945417 3.023457 -0.917883
1 -1.150662 2.896847 0.848777
1 0.397222 3.495785 0.170410
1 -1.070094 -1.617094 -0.394560
8 -3.948025 -1.473519 0.340600
1 -4.425838 -1.178575 1.128920
1 -3.369815 -0.726325 0.106246
rc_1m5mU/rc_1m5mUadl.xyz
6 -2.129738 0.003510 -0.175137
6 -0.216627 1.544847 0.010981
6 0.653062 0.383501 0.284777
6 0.066820 -0.896663 0.302923
1 0.672288 -1.776076 0.494411
7 -1.228355 -1.089496 0.089085
7 -1.554948 1.244538 -0.195894
8 -3.311264 -0.209472 -0.360922
8 0.194642 2.699250 -0.033769

6 2.089498 0.592841 0.526888
1 2.532490 1.141216 -0.315506
1 2.222067 1.243939 1.403250
1 2.618172 -0.347224 0.680462
6 -1.827517 -2.436251 0.103119
1 -2.615182 -2.459837 0.857915
1 -2.258849 -2.636406 -0.879339
1 -1.047714 -3.157226 0.336976
1 -2.185521 2.023209 -0.384635
8 5.236227 -0.938026 -0.379409
1 5.482762 -0.783052 -1.302794
1 5.292780 -1.898561 -0.271580
rc_1m5mU/rc_1m5mUadn.xyz
6 0.879245 0.471026 -0.013499
6 -0.856527 -1.274053 -0.017530
6 -1.880262 -0.210234 0.007273
6 -1.447210 1.129268 0.012770
1 -2.168098 1.939973 0.029099
7 -0.162576 1.462008 -0.003562
7 0.457768 -0.828402 -0.021406
8 2.046496 0.816906 -0.015461
8 -1.125876 -2.469562 -0.031814
6 -3.305694 -0.575001 0.028195
1 -3.527934 -1.256347 -0.803870
1 -3.523974 -1.146871 0.942466
1 -3.950916 0.302314 -0.017603
6 0.275488 2.868407 -0.001991
1 0.844724 3.057551 0.909870
1 0.913684 3.036298 -0.870446
1 -0.606501 3.502584 -0.044072
1 1.193976 -1.534520 -0.036283
8 4.057130 -1.357820 0.012574
1 3.447865 -0.599284 -0.006190
1 3.498585 -2.109610 0.258101
rc_1m5mU/rc_1m5mUacd.xyz
6 1.715662 -0.875492 0.019174

6 -0.728525 -0.643581 -0.171543
6 -0.561581 0.822558 -0.150084
6 0.742298 1.339156 -0.020767
1 0.905371 2.411122 0.010480
7 1.813173 0.561152 0.064325
7 0.445393 -1.373481 -0.102980
8 2.716399 -1.560020 0.084909
8 -1.818860 -1.204180 -0.247892
6 -1.741035 1.698008 -0.242017
1 -2.399478 1.365020 -1.052724
1 -2.331919 1.602102 0.682848
1 -1.459193 2.742420 -0.377550
6 3.170421 1.116421 0.202173
1 3.600200 0.759125 1.139486
1 3.777655 0.766120 -0.633946
1 3.100767 2.201623 0.199293
1 0.367079 -2.389867 -0.123279
8 -4.502358 -0.314512 0.417346
1 -3.588302 -0.536911 0.163977
1 -4.527021 0.652824 0.405468
rc_1m5mU/rc_1m5mUace.xyz
6 0.679650 -0.591648 -0.167958
6 -1.753858 -0.916438 0.022445
6 -1.913589 0.550335 0.069443
6 -0.755606 1.347114 -0.004873
1 -0.832346 2.428734 0.026954
7 0.461478 0.830074 -0.121024
7 -0.446209 -1.363047 -0.104705
8 1.806424 -1.040745 -0.262828
8 -2.693600 -1.700666 0.088677
6 -3.259255 1.133393 0.190719
1 -3.886065 0.791423 -0.645081
1 -3.747394 0.752195 1.098805
1 -3.229301 2.222814 0.211909
6 1.663420 1.679312 -0.203499
1 2.284593 1.489722 0.673859

1 2.215321 1.415729 -1.106670
1 1.349684 2.719919 -0.232867
1 -0.294989 -2.370871 -0.135784
8 4.563327 -0.191662 0.374349
1 3.650375 -0.427290 0.137651
1 5.009467 -1.039387 0.512079
rc_1m5mU/rc_1m5mUabl.xyz
6 -0.879204 0.470987 0.021341
6 0.856412 -1.274348 0.012135
6 1.880191 -0.210329 0.001503
6 1.447114 1.129174 -0.002788
1 2.168141 1.939949 -0.010129
7 0.162403 1.462010 0.000198
7 -0.457772 -0.828503 0.022115
8 -2.046358 0.816828 0.036733
8 1.125536 -2.470001 0.012494
6 3.305837 -0.574414 -0.018474
1 3.523886 -1.278217 0.795324
1 3.529592 -1.120296 -0.947630
1 3.950293 0.301743 0.053175
6 -0.275901 2.868303 -0.020373
1 -0.934305 3.015445 -0.877457
1 -0.823677 3.079513 0.899918
1 0.604833 3.500951 -0.097980
1 -1.194079 -1.534500 0.036429
8 -4.056581 -1.358154 -0.013918
1 -3.496149 -2.105581 -0.268297
1 -3.448433 -0.599177 0.017914
rc_1m5mU/rc_1m5mUaav.xyz
6 1.409003 -0.795070 0.000557
6 -1.026426 -0.967589 -0.330984
6 -1.063833 0.488751 -0.566208
6 0.149032 1.201436 -0.509568
1 0.164017 2.272898 -0.678176
7 1.310379 0.615118 -0.251395
7 0.232150 -1.489679 -0.075059

8 2.485805 -1.300917 0.253552
8 -2.021407 -1.684579 -0.360161
6 -2.345010 1.142423 -0.890814
1 -2.652915 0.844931 -1.905211
1 -3.133151 0.790496 -0.217368
1 -2.263118 2.229327 -0.852484
6 2.568028 1.377801 -0.165938
1 2.965225 1.281295 0.846014
1 3.279628 0.962802 -0.881035
1 2.355074 2.419095 -0.396049
1 0.296901 -2.490344 0.107431
8 -1.250683 0.680814 2.202954
1 -1.691457 1.528343 2.039218
1 -1.972395 0.034023 2.229807
rc_1m5mU/rc_1m5mUads.xyz
6 -1.364873 -0.820852 0.012544
6 1.079702 -1.003756 -0.236839
6 1.119493 0.431778 -0.573344
6 -0.098852 1.139508 -0.607575
1 -0.110829 2.195961 -0.852875
7 -1.265017 0.566433 -0.346989
7 -0.184422 -1.513855 0.017242
8 -2.445776 -1.310844 0.278193
8 2.078187 -1.715037 -0.183582
6 2.412445 1.071376 -0.874699
1 3.137594 0.839690 -0.085797
1 2.823783 0.641604 -1.800153
1 2.311128 2.150500 -0.992454
6 -2.531264 1.321223 -0.375426
1 -3.193633 0.865142 -1.112720
1 -2.991640 1.268282 0.612298
1 -2.311987 2.352994 -0.640215
1 -0.250461 -2.499910 0.266772
8 0.980208 1.135246 2.193945
1 0.307972 0.449873 2.328249
1 1.823280 0.657230 2.208706

rc_1m5mU/rc_1m5mUaai.xyz
6 0.869260 -0.399191 -0.795318
6 -1.473157 -0.861030 -0.189805
6 -1.609425 0.547787 0.229539
6 -0.488155 1.390115 0.106875
1 -0.548758 2.430190 0.409060
7 0.674022 0.963946 -0.370556
7 -0.223197 -1.213130 -0.676161
8 1.947169 -0.756968 -1.226964
8 -2.385876 -1.676900 -0.119391
6 -2.895189 1.026135 0.762723
1 -3.691161 0.850095 0.025703
1 -3.174258 0.431133 1.644051
1 -2.857036 2.083543 1.024913
6 1.848197 1.847707 -0.474036
1 2.641113 1.446183 0.160247
1 2.182866 1.863610 -1.512148
1 1.560677 2.843955 -0.145990
1 -0.088935 -2.179956 -0.969787
8 2.620766 -1.087023 1.974082
1 2.874037 -0.462467 2.669135
1 2.980019 -0.704011 1.160154
rc_1m5mU/rc_1m5mUabv.xyz
6 -1.362837 -1.014702 -0.165579
6 0.878498 -0.109015 -0.627061
6 0.399105 1.217080 -0.194267
6 -0.940801 1.334678 0.223578
1 -1.332847 2.292360 0.547863
7 -1.775629 0.304144 0.237863
7 -0.061689 -1.121976 -0.581363
8 -2.146164 -1.941623 -0.125633
8 2.030303 -0.319243 -0.999710
6 1.327757 2.358082 -0.198329
1 2.189727 2.132093 0.445947
1 1.743334 2.493398 -1.206805
1 0.845102 3.277124 0.134187

6 -3.174319 0.440701 0.681119
1 -3.830204 0.137323 -0.136552
1 -3.332924 -0.213447 1.539853
1 -3.348622 1.480653 0.948643
1 0.235851 -2.055713 -0.864544
8 3.802218 -1.219558 1.208660
1 4.167208 -0.397517 1.566512
1 3.269331 -0.938993 0.446088
rc_1m5mU/rc_1m5mUabb.xyz
6 0.784579 -1.232995 -0.053145
6 -0.922322 0.538980 -0.055508
6 0.159456 1.541065 -0.003856
6 1.489787 1.081253 0.031869
1 2.313556 1.786200 0.076847
7 1.796409 -0.209663 0.018411
7 -0.507853 -0.781112 -0.077703
8 1.101300 -2.404687 -0.085843
8 -2.112832 0.841689 -0.078382
6 -0.179277 2.972856 0.017544
1 -0.733716 3.233776 -0.895874
1 -0.868841 3.178086 0.847747
1 0.708576 3.599173 0.101901
6 3.189904 -0.679833 0.091500
1 3.286584 -1.343639 0.952024
1 3.425037 -1.229550 -0.821758
1 3.839516 0.185556 0.197196
1 -1.229642 -1.501021 -0.123994
8 -3.984984 -1.404053 0.071098
1 -3.470215 -1.972330 0.662402
1 -3.451376 -0.592362 -0.006860
rc_1m5mU/rc_1m5mUaax.xyz
6 0.042855 -1.327842 0.041632
6 -2.194772 -0.305300 -0.080881
6 -1.559142 1.022828 0.025003
6 -0.157508 1.082391 0.140165
1 0.346291 2.039694 0.220160

7 0.604071 -0.004592 0.144494
7 -1.320421 -1.380871 -0.062548
8 0.768563 -2.302179 0.050618
8 -3.406310 -0.467284 -0.180170
6 -2.397076 2.231692 -0.011990
1 -2.972711 2.249860 -0.948509
1 -3.145584 2.190752 0.791642
1 -1.802443 3.140953 0.076101
6 2.073064 0.067857 0.227533
1 2.402024 -0.483278 1.109904
1 2.490792 -0.394933 -0.668048
1 2.364303 1.113110 0.294738
1 -1.724164 -2.314280 -0.132757
8 5.311592 0.767057 -0.116000
1 6.164306 0.734469 -0.573502
1 4.656345 0.811386 -0.827691
rc_1m5mU/rc_1m5mUacg.xyz
6 -1.700417 -0.907099 -0.000001
6 0.739652 -0.582975 0.000008
6 0.516523 0.876388 0.000005
6 -0.812429 1.344695 -0.000002
1 -1.016948 2.410259 -0.000003
7 -1.856651 0.526024 -0.000004
7 -0.407089 -1.356634 0.000004
8 -2.676864 -1.628857 -0.000004
8 1.852111 -1.103064 0.000014
6 1.671510 1.788242 0.000010
1 2.304295 1.582246 0.874949
1 2.304298 1.582251 -0.874928
1 1.362869 2.833300 0.000012
6 -3.242999 1.025493 -0.000007
1 -3.747582 0.649657 -0.891591
1 -3.747588 0.649653 0.891571
1 -3.217771 2.112690 -0.000004
1 -0.289373 -2.369419 0.000008
8 4.597448 -0.197658 -0.000018

1 3.645733 -0.407452 -0.000009
1 5.035641 -1.060749 -0.000017
rc_1m5mU/rc_1m5mUacf.xyz
6 -0.907975 -0.438118 -0.749632
6 1.457999 -0.844627 -0.200065
6 1.580193 0.576576 0.181376
6 0.442637 1.397210 0.066236
1 0.493662 2.445757 0.339688
7 -0.724305 0.939227 -0.368262
7 0.200894 -1.231045 -0.638747
8 -1.989096 -0.823238 -1.148228
8 2.388546 -1.641032 -0.139935
6 2.869631 1.087329 0.673921
1 3.181000 0.511680 1.557161
1 3.650403 0.914179 -0.079883
1 2.818466 2.147891 0.920434
6 -1.915732 1.799593 -0.462156
1 -2.284455 1.774159 -1.488511
1 -2.679063 1.406583 0.212469
1 -1.635391 2.811291 -0.177771
1 0.075488 -2.207159 -0.904192
8 -2.350324 -1.038181 1.990400
1 -2.916839 -0.744672 1.261633
1 -2.592920 -0.465149 2.732060
rc_1m5mU/rc_1m5mUacm.xyz
6 -1.372967 0.909533 -0.133222
6 1.069490 1.235801 -0.079456
6 1.204410 -0.117337 0.487005
6 0.030102 -0.867762 0.704310
1 0.088616 -1.861884 1.130967
7 -1.178002 -0.393659 0.436894
7 -0.231989 1.636716 -0.343167
8 -2.492575 1.305228 -0.395554
8 2.025308 1.970086 -0.311879
6 2.541783 -0.649474 0.798384
1 3.179271 -0.601358 -0.094332

1 3.027912 -0.007124 1.546597
1 2.493000 -1.673768 1.168603
6 -2.390531 -1.207993 0.630911
1 -3.095263 -0.649778 1.247783
1 -2.834420 -1.407328 -0.346447
1 -2.106880 -2.137572 1.119224
1 -0.364901 2.563122 -0.746678
8 0.670999 -2.405892 -1.525484
1 -0.074873 -2.950445 -1.231163
1 1.433894 -2.747253 -1.034906
rc_1mC/rc_1mCaar.xyz
6 -1.472581 -0.830305 0.001777
6 0.826440 -0.538305 0.000634
6 0.694514 0.907262 -0.003424
6 -0.595534 1.448202 -0.004125
1 -0.765528 2.519269 -0.006776
7 -1.644398 0.642707 -0.001524
7 -0.234111 -1.348404 0.003240
8 -2.515217 -1.480945 0.003011
7 2.052875 -1.043019 0.001890
1 2.170736 -2.051727 0.005428
1 2.890601 -0.436347 -0.001505
1 1.562622 1.555742 -0.006172
6 -3.016279 1.160038 -0.001833
1 -3.530306 0.786746 -0.889648
1 -3.529334 0.790122 0.887962
1 -2.982392 2.247685 -0.003909
8 4.336227 0.615149 -0.022337
1 4.991701 0.277391 -0.651387
1 4.783901 0.597152 0.837190
rc_1mC/rc_1mCaad.xyz
6 1.472870 0.829496 -0.002940
6 -0.826660 0.541671 0.003661
6 -0.697323 -0.904160 0.006999
6 0.591743 -1.447423 0.005278
1 0.759858 -2.518789 0.008052

7 1.642024 -0.643804 0.000822
7 0.235336 1.349883 -0.001317
8 2.516686 1.478238 -0.007115
7 -2.052261 1.048483 0.005816
1 -2.168491 2.057369 0.002573
1 -2.890736 0.442829 0.008966
1 -1.566599 -1.551043 0.011292
6 3.012960 -1.163626 -0.000305
1 3.525268 -0.796197 -0.891564
1 3.529045 -0.789751 0.886062
1 2.977065 -2.251185 0.003699
8 -4.327697 -0.617614 0.007799
1 -4.804694 -0.540913 -0.832466
1 -4.969853 -0.354977 0.684505
rc_1mC/rc_1mCaby.xyz
6 0.835082 -0.943156 -0.041928
6 -0.810554 0.686413 -0.036272
6 0.173338 1.752693 -0.029195
6 1.525496 1.398255 0.007326
1 2.312434 2.144418 0.033747
7 1.866943 0.120697 0.017041
7 -0.463126 -0.602002 -0.053529
8 1.268381 -2.092740 -0.072345
7 -2.092461 1.028642 -0.024982
1 -2.838610 0.313595 -0.009940
1 -2.350609 2.009580 -0.014391
1 -0.120290 2.795843 -0.040450
6 3.268007 -0.303205 0.093261
1 3.387563 -0.952456 0.962469
1 3.516807 -0.867156 -0.808124
1 3.898899 0.579142 0.180905
8 -4.194286 -0.889917 0.053861
1 -4.881599 -0.680454 -0.596422
1 -4.645056 -0.858623 0.911221
rc_1mC/rc_1mCacv.xyz
6 -0.247124 0.517588 -0.198684

6 2.051346 0.467988 0.054974
6 2.057804 -0.974930 0.199657
6 0.831628 -1.644415 0.107470
1 0.760444 -2.723397 0.188617
7 -0.280878 -0.955210 -0.083320
7 0.929112 1.159740 -0.131160
8 -1.334961 1.068179 -0.360781
7 3.216057 1.103636 0.111974
1 3.249311 2.113209 0.008289
1 4.083177 0.597070 0.254799
1 2.975866 -1.527636 0.363543
6 -1.586159 -1.615635 -0.192825
1 -2.214789 -1.294672 0.640542
1 -2.052311 -1.310725 -1.131056
1 -1.433950 -2.692451 -0.166168
8 -4.108181 0.767485 0.361279
1 -4.348834 1.667690 0.622402
1 -3.168785 0.824869 0.109034
rc_1mC/rc_1mCaau.xyz
6 0.817973 -0.939846 0.038023
6 -0.814065 0.703445 0.031221
6 0.178542 1.761715 0.036967
6 1.527820 1.396296 0.003607
1 2.321061 2.136010 -0.015758
7 1.858548 0.115828 -0.012674
7 -0.477418 -0.588266 0.044489
8 1.242020 -2.092999 0.066586
7 -2.093031 1.055228 0.011331
1 -2.842632 0.343448 -0.014946
1 -2.344766 2.037911 0.004418
1 -0.107016 2.807068 0.054187
6 3.255890 -0.319982 -0.089321
1 3.497460 -0.894353 0.807354
1 3.371268 -0.962359 -0.964244
1 3.895149 0.557160 -0.167452
8 -4.204749 -0.837923 -0.155827

1 -3.935969 -1.696460 0.204895
1 -4.966375 -0.570349 0.380480
rc_1mC/rc_1mCadf.xyz
6 -0.392755 0.842767 -0.104423
6 0.786539 -1.160904 -0.060273
6 -0.434169 -1.932221 0.070406
6 -1.647453 -1.234503 0.109439
1 -2.594926 -1.752208 0.210821
7 -1.655461 0.085138 0.020399
7 0.771703 0.167835 -0.147729
8 -0.498458 2.062459 -0.162097
7 1.943322 -1.810227 -0.091240
1 2.813939 -1.293598 -0.177395
1 1.978597 -2.821344 -0.023254
1 -0.416261 -3.013569 0.141929
6 -2.900787 0.859879 0.072151
1 -2.884517 1.484613 0.968016
1 -2.953010 1.500179 -0.809616
1 -3.743248 0.171601 0.099735
8 3.296021 1.536536 0.172706
1 2.407189 1.137891 0.051778
1 3.126531 2.445154 0.459309
rc_1mC/rc_1mCadp.xyz
6 1.473295 -0.822380 0.002763
6 -0.830344 -0.574132 -0.008997
6 -0.726786 0.873871 -0.012649
6 0.552590 1.439586 -0.005175
1 0.703101 2.513609 -0.006517
7 1.616345 0.653817 0.001902
7 0.245138 -1.364310 -0.002712
8 2.528263 -1.452730 0.008569
7 -2.047557 -1.100386 -0.012479
1 -2.149213 -2.110811 -0.008902
1 -2.892904 -0.503855 -0.012106
1 -1.608197 1.504453 -0.021440
6 2.978211 1.196855 0.007058

1 3.494529 0.837089 0.899129
1 3.502447 0.832775 -0.878613
1 2.923719 2.283466 0.004131
8 -4.360470 0.508950 -0.002816
1 -4.276107 1.207726 -0.669480
1 -4.419001 0.979145 0.842792
rc_1mC/rc_1mCacm.xyz
6 0.807052 -0.939063 -0.042934
6 -0.816176 0.712980 -0.034377
6 0.182475 1.765798 -0.031973
6 1.529689 1.392882 0.003706
1 2.326874 2.128177 0.028496
7 1.853327 0.110524 0.015274
7 -0.486341 -0.580475 -0.051843
8 1.224822 -2.094446 -0.074958
7 -2.093413 1.071655 -0.018372
1 -2.846870 0.363308 0.001561
1 -2.339844 2.055635 -0.007963
1 -0.097415 2.812783 -0.045124
6 3.247807 -0.333673 0.094051
1 3.356991 -0.980274 0.966692
1 3.488590 -0.905868 -0.804235
1 3.891936 0.539368 0.177438
8 -4.232905 -0.790101 0.091320
1 -4.301719 -1.272079 -0.746717
1 -4.033979 -1.470150 0.752701
rc_1mC/rc_1mCadu.xyz
6 -0.832845 -0.942968 0.037291
6 0.810446 0.688989 0.035802
6 -0.175091 1.753846 0.035987
6 -1.526789 1.397558 -0.000367
1 -2.314893 2.142657 -0.022351
7 -1.866339 0.119522 -0.015850
7 0.464895 -0.599997 0.048456
8 -1.264413 -2.093310 0.063616
7 2.091845 1.033128 0.022403

1 2.838747 0.319106 0.000797
1 2.348657 2.014455 0.016758
1 0.117031 2.797365 0.052414
6 -3.266718 -0.306520 -0.093327
1 -3.385767 -0.950246 -0.966722
1 -3.513379 -0.876820 0.804596
1 -3.899479 0.575093 -0.174542
8 4.188961 -0.889024 -0.088884
1 4.665524 -0.943196 0.753207
1 4.860355 -0.633743 -0.739383
rc_1mC/rc_1mCaac.xyz
6 -1.472875 -0.822577 0.001873
6 0.831251 -0.577995 -0.002634
6 0.730067 0.870217 0.002452
6 -0.548494 1.437885 0.004305
1 -0.697324 2.512147 0.006177
7 -1.613515 0.653818 0.002777
7 -0.245602 -1.366394 -0.001514
8 -2.528848 -1.451294 0.003738
7 2.047408 -1.106625 -0.008868
1 2.147146 -2.117216 -0.013592
1 2.893451 -0.511391 -0.011563
1 1.612087 1.500071 0.005684
6 -2.974533 1.199021 0.001632
1 -3.494635 0.837349 -0.887477
1 -3.496115 0.838498 0.890306
1 -2.918274 2.285528 0.000887
8 4.332137 0.544676 -0.074621
1 4.018892 1.454811 -0.191165
1 4.797930 0.548248 0.775270
rc_1mC/rc_1mCabe.xyz
6 1.472417 -0.823704 0.007762
6 -0.831209 -0.575552 -0.017569
6 -0.727623 0.872463 -0.026936
6 0.551716 1.438138 -0.012525
1 0.702174 2.512155 -0.016882

7 1.615428 0.652470 0.003236
7 0.244325 -1.365623 -0.002393
8 2.527376 -1.454009 0.020639
7 -2.048259 -1.102043 -0.025151
1 -2.150075 -2.112404 -0.016792
1 -2.893039 -0.505007 -0.029872
1 -1.608314 1.503905 -0.044817
6 2.977211 1.195603 0.012856
1 3.489611 0.839061 0.908471
1 3.505340 0.828363 -0.869193
1 2.922706 2.282198 0.005720
8 -4.320926 0.567930 -0.063523
1 -4.017591 1.464450 0.146564
1 -4.937933 0.340591 0.648496
rc_1mC/rc_1mCabo.xyz
6 1.565852 -0.340503 0.207968
6 0.367511 1.620796 -0.069196
6 -0.816399 0.897390 -0.492049
6 -0.749745 -0.500396 -0.538169
1 -1.599495 -1.102894 -0.839066
7 0.374060 -1.113236 -0.208643
7 1.497684 1.001456 0.260823
8 2.559676 -1.007121 0.481875
7 0.301656 2.947262 -0.019500
1 1.113797 3.485404 0.265394
1 -0.549430 3.439393 -0.267393
1 -1.729806 1.412939 -0.766666
6 0.496105 -2.574122 -0.245546
1 1.282187 -2.840564 -0.954630
1 0.776970 -2.928509 0.747920
1 -0.459037 -2.996770 -0.550135
8 -3.900432 -0.578647 0.540169
1 -3.678015 0.186781 -0.009724
1 -4.824862 -0.437013 0.791132
rc_1mC/rc_1mCaaz.xyz
6 0.384484 -0.840822 -0.105247

6 -0.780891 1.171063 -0.064284
6 0.445034 1.934146 0.066025
6 1.653372 1.227930 0.108323
1 2.604256 1.739151 0.210811
7 1.652303 -0.091960 0.021484
7 -0.775037 -0.157779 -0.149937
8 0.481606 -2.061205 -0.163324
7 -1.933525 1.827573 -0.096839
1 -2.806840 1.315512 -0.183104
1 -1.962856 2.838980 -0.030752
1 0.435097 3.015732 0.135334
6 2.891507 -0.876060 0.077910
1 2.867929 -1.498700 0.975070
1 2.941038 -1.518650 -0.802327
1 3.739703 -0.194835 0.106214
8 -3.283867 -1.543876 0.236610
1 -3.156444 -2.474887 0.005381
1 -2.411020 -1.124039 0.077766
rc_1mC/rc_1mCabz.xyz
6 0.232075 -0.547830 -0.035323
6 -2.078365 -0.450727 0.015086
6 -2.067814 0.997204 0.091908
6 -0.826754 1.643476 0.049401
1 -0.742208 2.724151 0.075787
7 0.283618 0.929140 -0.030828
7 -0.957881 -1.168704 -0.029413
8 1.319048 -1.122993 -0.048984
7 -3.256884 -1.062781 -0.009491
1 -3.302606 -2.075224 -0.071593
1 -4.122813 -0.535545 0.031228
1 -2.984094 1.571487 0.165347
6 1.603749 1.564656 -0.100858
1 2.178051 1.280983 0.783527
1 2.116435 1.203229 -0.993703
1 1.467996 2.643098 -0.141235
8 4.178196 -0.693592 0.092497

1 3.212774 -0.813205 0.043073
1 4.539197 -1.590554 0.126316
rc_1mC/rc_1mCacb.xyz
6 -0.857592 0.940795 0.309713
6 1.328315 0.584273 -0.368755
6 0.981714 -0.686570 -0.975828
6 -0.366089 -1.068752 -0.965724
1 -0.697693 -1.989064 -1.433796
7 -1.263857 -0.301435 -0.376265
7 0.427107 1.339336 0.254408
8 -1.755257 1.545588 0.891557
7 2.591847 0.988924 -0.449480
1 2.869430 1.876238 -0.042920
1 3.292439 0.421012 -0.912184
1 1.727219 -1.313559 -1.449123
6 -2.687142 -0.659180 -0.344090
1 -3.007393 -0.742761 0.695473
1 -3.258547 0.132191 -0.832542
1 -2.821436 -1.605973 -0.863642
8 1.309974 -1.907840 1.531857
1 2.143953 -1.427378 1.645052
1 0.633375 -1.303861 1.873831
rc_1mC/rc_1mCabp.xyz
6 -0.345951 1.291825 -0.269576
6 -1.781006 -0.508637 -0.022696
6 -0.788327 -1.261407 0.720591
6 0.454626 -0.660732 0.948760
1 1.241407 -1.169077 1.494614
7 0.687552 0.557965 0.493720
7 -1.542880 0.716353 -0.484192
8 -0.020846 2.409193 -0.660744
7 -2.960438 -1.082541 -0.239946
1 -3.680509 -0.588461 -0.757592
1 -3.157682 -2.015915 0.103765
1 -0.992042 -2.260617 1.088388
6 1.980757 1.220610 0.690365

1 2.431359 1.400179 -0.287939
1 1.812531 2.177559 1.187295
1 2.616785 0.578939 1.296783
8 2.970685 -1.809091 -0.938012
1 3.433517 -2.378052 -1.570119
1 2.285685 -2.377753 -0.556896
rc_1mC/rc_1mCaaa.xyz
6 0.515558 1.151356 -0.008123
6 2.254742 -0.369454 -0.164843
6 1.383109 -1.479651 0.170248
6 0.035044 -1.200027 0.422108
1 -0.670152 -1.980707 0.684097
7 -0.403456 0.044480 0.339709
7 1.812276 0.883074 -0.243806
8 0.006158 2.267273 -0.054221
7 3.535691 -0.636540 -0.398109
1 4.176249 0.112431 -0.641179
1 3.887848 -1.586229 -0.350486
1 1.753361 -2.496492 0.228478
6 -1.802331 0.389598 0.617302
1 -1.828936 1.081617 1.461589
1 -2.225367 0.882886 -0.259494
1 -2.347590 -0.522864 0.851453
8 -5.148311 -0.770365 -0.605206
1 -4.549673 -0.164728 -0.144959
1 -5.986817 -0.289209 -0.658807
rc_1mC/rc_1mCaca.xyz
6 -0.257939 -0.495471 0.385436
6 2.005520 -0.448083 -0.091120
6 1.967219 0.970800 -0.385621
6 0.747080 1.637496 -0.223652
1 0.652289 2.703260 -0.400031
7 -0.325088 0.964297 0.159676
7 0.918686 -1.129625 0.266586
8 -1.320824 -1.039634 0.681108
7 3.173858 -1.071625 -0.191967

1 3.240339 -2.063883 0.013681
1 4.011118 -0.570910 -0.469701
1 2.850600 1.509510 -0.708849
6 -1.616382 1.629255 0.367321
1 -2.324160 1.270587 -0.383000
1 -1.986804 1.369294 1.359771
1 -1.473354 2.704018 0.275108
8 -3.836304 -1.069995 -0.749578
1 -3.002827 -0.989149 -0.251766
1 -4.155359 -0.161002 -0.841704
rc_1mC/rc_1mCaao.xyz
6 0.870191 -0.951082 0.303900
6 -1.328914 -0.611947 -0.340667
6 -1.005337 0.670452 -0.936423
6 0.338026 1.066632 -0.941928
1 0.653252 1.995313 -1.404723
7 1.251967 0.303992 -0.370317
7 -0.410734 -1.363771 0.261790
8 1.782973 -1.552495 0.866167
7 -2.587473 -1.032138 -0.413129
1 -2.849638 -1.927115 -0.013627
1 -3.301311 -0.465417 -0.856442
1 -1.763280 1.289614 -1.399557
6 2.671040 0.678939 -0.351511
1 3.000053 0.767901 0.684991
1 3.247932 -0.105464 -0.844439
1 2.789116 1.626863 -0.872910
8 -1.424091 1.850865 1.493368
1 -0.970238 2.658991 1.209750
1 -0.723290 1.297808 1.872039
rc_1mC/rc_1mCadv.xyz
6 -0.433373 -0.998093 -0.476768
6 1.670109 -0.211831 0.086607
6 1.140227 0.563088 1.192922
6 -0.239107 0.513389 1.424862
1 -0.704351 1.080690 2.223252

7 -1.007029 -0.239869 0.657319
7 0.893150 -0.955722 -0.697167
8 -1.240149 -1.632066 -1.150569
7 2.979998 -0.162522 -0.132971
1 3.388631 -0.692446 -0.896137
1 3.588742 0.393967 0.457061
1 1.782304 1.170655 1.820324
6 -2.464721 -0.257591 0.812633
1 -2.913375 0.154270 -0.095072
1 -2.797744 -1.289122 0.936856
1 -2.732292 0.342980 1.679895
8 -0.780846 2.484817 -1.439503
1 -0.008623 1.925107 -1.270972
1 -1.536971 1.944912 -1.166437
rc_1mU/rc_1mUabh.xyz
6 -0.348619 -0.844618 0.045665
6 0.783004 1.321899 0.036144
6 -0.528978 1.962709 -0.023989
6 -1.691506 1.181303 -0.046715
1 -2.675823 1.634822 -0.091010
7 -1.623276 -0.142632 -0.010946
7 0.766135 -0.060806 0.078251
8 -0.341657 -2.059476 0.062130
8 1.837307 1.958436 0.045832
1 -0.587941 3.043512 -0.050879
6 -2.822613 -0.990532 -0.037643
1 -2.795254 -1.601313 -0.942569
1 -2.808377 -1.640885 0.838651
1 -3.703252 -0.352466 -0.030809
1 1.683027 -0.567473 0.059431
8 3.160128 -1.421406 -0.160840
1 3.871084 -0.776833 -0.299650
1 3.422571 -1.920303 0.627949
rc_1mU/rc_1mUacq.xyz
6 -0.292339 -0.830546 -0.056789
6 0.736172 1.386430 -0.034221

6 -0.604409 1.963696 0.038393
6 -1.728766 1.128104 0.059606
1 -2.733770 1.532566 0.112353
7 -1.597715 -0.190594 0.013159
7 0.783935 0.004946 -0.087050
8 -0.227634 -2.043641 -0.083920
8 1.758937 2.072210 -0.046062
1 -0.713985 3.040308 0.073088
6 -2.755479 -1.093733 0.041918
1 -2.726916 -1.724701 -0.848266
1 -2.681659 -1.721890 0.932155
1 -3.663933 -0.496826 0.064532
1 1.719327 -0.465867 -0.083023
8 3.179992 -1.387360 0.001217
1 3.750201 -1.042283 0.705500
1 2.925759 -2.275155 0.297573
rc_1mU/rc_1mUacg.xyz
6 0.292545 -0.830279 0.056337
6 -0.737027 1.386107 0.033215
6 0.603295 1.963987 -0.039730
6 1.728101 1.128962 -0.059846
1 2.732937 1.533903 -0.112154
7 1.597671 -0.189768 -0.012785
7 -0.784095 0.004682 0.086963
8 0.228293 -2.043427 0.082566
8 -1.760136 2.071410 0.044760
1 0.712336 3.040625 -0.075335
6 2.755803 -1.092448 -0.039299
1 2.728187 -1.720013 0.853371
1 2.681583 -1.724069 -0.926997
1 3.663987 -0.495243 -0.064711
1 -1.719201 -0.466975 0.083938
8 -3.172170 -1.398899 0.002694
1 -3.794483 -0.996814 -0.622874
1 -2.924560 -2.246454 -0.398706
rc_1mU/rc_1mUacn.xyz

6 -0.767075 -0.928851 -0.034491
6 0.910798 0.865415 -0.034086
6 -0.184671 1.829511 -0.015982
6 -1.513116 1.383079 0.013724
1 -2.341203 2.083547 0.040939
7 -1.801258 0.088344 0.014503
7 0.519659 -0.462863 -0.047416
8 -1.076064 -2.100425 -0.058219
8 2.096510 1.194671 -0.037767
1 0.046129 2.887458 -0.014389
6 -3.184751 -0.404116 0.080296
1 -3.393771 -0.983472 -0.821430
1 -3.278454 -1.051097 0.954633
1 -3.852464 0.450450 0.154654
1 1.251194 -1.174074 -0.070496
8 4.069718 -0.979835 0.004923
1 4.767879 -0.774430 0.642828
1 3.483464 -0.202261 0.015396
rc_1mU/rc_1mUadp.xyz
6 -0.924883 -0.902951 -0.238917
6 1.431871 -0.606614 0.416378
6 1.074980 0.732181 0.880133
6 -0.263066 1.151471 0.849594
1 -0.552407 2.120939 1.239664
7 -1.216346 0.386925 0.333332
7 0.373109 -1.325446 -0.123077
8 -1.808428 -1.549955 -0.766329
8 2.563139 -1.078482 0.484055
1 1.841564 1.360579 1.312166
6 -2.622826 0.819158 0.271478
1 -2.931915 0.855240 -0.774299
1 -3.234749 0.091846 0.807507
1 -2.703189 1.802167 0.729762
1 0.571795 -2.250650 -0.501306
8 1.576189 1.603124 -1.479670
1 0.856216 1.134658 -1.932374

1 1.231677 2.497903 -1.329352
rc_1mU/rc_1mUacz.xyz
6 -0.287906 -0.396815 0.504625
6 2.078589 -0.531442 -0.147821
6 2.024394 0.892682 -0.460118
6 0.831251 1.607364 -0.284176
1 0.769736 2.665658 -0.514165
7 -0.265056 1.013236 0.169052
7 0.882409 -1.071664 0.308068
8 -1.307920 -0.899055 0.932290
8 3.090598 -1.215870 -0.271401
1 2.915913 1.385658 -0.827186
6 -1.529277 1.743873 0.350684
1 -2.272314 1.325477 -0.331812
1 -1.865391 1.607423 1.379659
1 -1.352434 2.794628 0.134844
1 0.870590 -2.062649 0.549705
8 -3.626207 -0.988860 -0.990956
1 -2.955314 -0.946291 -0.289999
1 -4.386340 -1.424598 -0.579513
rc_1mU/rc_1mUadh.xyz
6 0.287290 -0.402243 0.475164
6 -2.088707 -0.535915 -0.142045
6 -2.040160 0.889913 -0.448231
6 -0.847800 1.606974 -0.276042
1 -0.792625 2.668162 -0.493994
7 0.254794 1.012284 0.161167
7 -0.886236 -1.076296 0.297061
8 1.317365 -0.909774 0.871460
8 -3.101037 -1.221356 -0.256368
1 -2.936364 1.383154 -0.803398
6 1.516074 1.748122 0.345225
1 1.869674 1.582190 1.363760
1 2.251748 1.360231 -0.362775
1 1.326659 2.803409 0.164900
1 -0.867674 -2.070058 0.526657

8 3.742699 -0.916112 -0.877175
1 2.984087 -0.884590 -0.271152
1 3.952204 -1.857578 -0.959348
rc_1mU/rc_1mUacy.xyz
6 -0.996252 0.878213 -0.242332
6 1.369094 0.671067 0.415921
6 1.064497 -0.679681 0.881894
6 -0.247706 -1.167951 0.808281
1 -0.497331 -2.157534 1.174540
7 -1.227446 -0.443159 0.283603
7 0.285443 1.347830 -0.127201
8 -1.910181 1.505048 -0.741784
8 2.482093 1.185553 0.483172
1 1.848026 -1.266788 1.340650
6 -2.611306 -0.941343 0.198556
1 -3.257629 -0.279780 0.777904
1 -2.922773 -0.933337 -0.846759
1 -2.637273 -1.951997 0.599041
1 0.446233 2.286866 -0.488617
8 1.677662 -1.529082 -1.426026
1 1.513858 -2.468014 -1.242118
1 2.634353 -1.416092 -1.306279
rc_1mU/rc_1mUaaa.xyz
6 -1.011631 -0.868073 -0.250055
6 1.356090 -0.703775 0.413797
6 1.072818 0.647825 0.889021
6 -0.231191 1.158959 0.814428
1 -0.464593 2.150511 1.185744
7 -1.221884 0.452865 0.286961
7 0.263858 -1.356327 -0.143034
8 -1.935459 -1.475902 -0.754255
8 2.459772 -1.238612 0.482239
1 1.864436 1.219119 1.353848
6 -2.598228 0.972666 0.208173
1 -2.907571 0.988698 -0.837726
1 -3.254969 0.309697 0.773884

1 -2.609967 1.975739 0.627643
1 0.410369 -2.295171 -0.511061
8 1.686445 1.689210 -1.369263
1 2.531549 2.030334 -1.036227
1 1.923712 0.902128 -1.885539
rc_1mU/rc_1mUaaq.xyz
6 0.488615 1.115827 0.002804
6 2.391123 -0.444520 0.006810
6 1.430451 -1.543444 -0.009291
6 0.054971 -1.274865 -0.020046
1 -0.675862 -2.076504 -0.032889
7 -0.401505 -0.029670 -0.015470
7 1.824880 0.822912 0.010988
8 0.026922 2.237073 0.009580
8 3.608426 -0.609567 0.016864
1 1.796207 -2.562369 -0.012925
6 -1.842650 0.266083 -0.026821
1 -2.092997 0.818670 0.881000
1 -2.064400 0.883621 -0.899033
1 -2.382868 -0.676000 -0.068557
1 2.454751 1.624813 0.025094
8 -5.059201 -0.588839 0.102231
1 -4.722992 -0.003434 -0.592146
1 -6.019702 -0.585325 -0.019291
rc_1mU/rc_1mUaah.xyz
6 -0.929734 0.898451 0.237849
6 1.420729 0.598828 -0.435218
6 1.059063 -0.737773 -0.899658
6 -0.277616 -1.160614 -0.848961
1 -0.569470 -2.132270 -1.231669
7 -1.225242 -0.395928 -0.324996
7 0.366361 1.321988 0.106751
8 -1.809061 1.546464 0.770305
8 2.555008 1.064804 -0.502823
1 1.821753 -1.367231 -1.337230
6 -2.630844 -0.828930 -0.247055

1 -2.930319 -0.857045 0.801680
1 -3.247859 -0.105988 -0.783226
1 -2.714115 -1.815502 -0.697063
1 0.569039 2.246960 0.483581
8 1.504998 -1.696801 1.472437
1 0.864562 -1.128049 1.929247
1 2.361439 -1.258802 1.601297
rc_1mU/rc_1mUadu.xyz
6 -0.989669 0.880526 -0.239694
6 1.374867 0.659352 0.417177
6 1.063090 -0.690921 0.879862
6 -0.252355 -1.170311 0.809990
1 -0.507573 -2.158256 1.176821
7 -1.228475 -0.439450 0.286304
7 0.294005 1.343977 -0.121846
8 -1.899767 1.512081 -0.740311
8 2.490270 1.168103 0.485859
1 1.844260 -1.283767 1.335361
6 -2.614934 -0.930107 0.200783
1 -3.258506 -0.263159 0.776958
1 -2.924731 -0.923459 -0.845087
1 -2.647350 -1.939484 0.604095
1 0.459912 2.282270 -0.482848
8 1.684318 -1.494818 -1.434938
1 1.271595 -2.372267 -1.387022
1 2.619118 -1.647730 -1.223077
rc_1mU/rc_1mUacj.xyz
6 0.887015 1.209255 0.013380
6 1.699711 -1.112926 -0.001665
6 0.307534 -1.550016 -0.031706
6 -0.729830 -0.605105 -0.042150
1 -1.777647 -0.905269 -0.058055
7 -0.467869 0.695280 -0.026586
7 1.875915 0.264125 0.019850
8 1.077965 2.407014 0.038344
8 2.655119 -1.885543 0.006085

1 0.089028 -2.610454 -0.045094
6 -1.543780 1.698795 -0.033170
1 -1.482437 2.277752 0.890764
1 -1.394883 2.361685 -0.887121
1 -2.495990 1.178516 -0.105320
1 2.831395 0.619360 0.046038
8 -3.865709 -1.256411 -0.008826
1 -4.192627 -0.449203 -0.432867
1 -4.096055 -1.148726 0.925850
rc_1mU/rc_1mUaco.xyz
6 0.724893 -0.921383 -0.049214
6 -0.918224 0.905476 -0.040640
6 0.196074 1.847355 -0.003333
6 1.515311 1.374402 0.033150
1 2.356716 2.057932 0.076629
7 1.778274 0.074467 0.018832
7 -0.552291 -0.430520 -0.062017
8 1.010663 -2.098518 -0.087137
8 -2.096929 1.258263 -0.052048
1 -0.013539 2.909649 0.008554
6 3.152061 -0.444884 0.082314
1 3.233924 -1.098007 0.953365
1 3.350169 -1.023432 -0.822302
1 3.835469 0.396829 0.160689
1 -1.296134 -1.127975 -0.103981
8 -4.041388 -0.969193 0.046438
1 -3.551577 -1.548429 0.648233
1 -3.496355 -0.164405 -0.010574
rc_1mU/rc_1mUaac.xyz
6 -0.302096 -0.364936 0.728701
6 1.996496 -0.384547 -0.144019
6 1.761288 0.958506 -0.664094
6 0.525578 1.586458 -0.457046
1 0.335075 2.588628 -0.825319
7 -0.449728 0.980697 0.207154
7 0.917606 -0.946307 0.525461

8 -1.232851 -0.895577 1.299257
8 3.057850 -0.987585 -0.280237
1 2.554095 1.460480 -1.204274
6 -1.747713 1.626949 0.451464
1 -2.526093 1.029252 -0.026959
1 -1.923187 1.653311 1.528638
1 -1.716998 2.631479 0.037261
1 1.026329 -1.891409 0.892671
8 -2.896045 -1.415172 -1.385528
1 -3.219074 -0.536469 -1.632516
1 -2.638235 -1.323906 -0.455777
rc_1mU/rc_1mUadr.xyz
6 -0.922196 1.196662 0.008938
6 -1.659041 -1.150711 -0.000272
6 -0.253621 -1.542394 -0.033168
6 0.753128 -0.565103 -0.039492
1 1.810049 -0.833868 -0.058756
7 0.448738 0.726058 -0.014366
7 -1.880181 0.220031 0.016998
8 -1.151238 2.387973 0.020276
8 -2.588597 -1.954226 0.011462
1 -0.001502 -2.595250 -0.050751
6 1.491403 1.764217 -0.010775
1 1.387923 2.364593 -0.916921
1 1.345008 2.399871 0.863981
1 2.461254 1.274057 0.023096
1 -2.847104 0.543506 0.035989
8 3.875852 -1.219562 -0.045819
1 4.195643 -0.323965 -0.230584
1 4.122658 -1.381072 0.876787
rc_1mU/rc_1mUace.xyz
6 1.470033 -0.769895 0.064848
6 -0.969810 -0.593327 -0.172981
6 -0.812802 0.856371 -0.245249
6 0.462355 1.430748 -0.152399
1 0.601424 2.505205 -0.200328

7 1.544652 0.677833 -0.007266
7 0.213162 -1.302260 -0.044346
8 2.483278 -1.418866 0.208806
8 -2.060537 -1.160574 -0.218358
1 -1.694112 1.474544 -0.364304
6 2.891852 1.258574 0.096291
1 3.309284 0.990928 1.069158
1 3.513201 0.834090 -0.694427
1 2.810297 2.337398 -0.009964
1 0.153163 -2.318404 0.020091
8 -4.379205 0.584100 0.315280
1 -3.650175 -0.023143 0.096286
1 -3.945839 1.438270 0.455891
rc_1mU/rc_1mUabd.xyz
6 -1.398465 -0.801715 -0.000003
6 -0.924948 1.613290 0.000002
6 0.497465 1.285836 0.000009
6 0.913218 -0.053620 0.000007
1 1.970924 -0.304645 0.000014
7 0.032106 -1.044923 -0.000001
7 -1.772865 0.513907 0.000001
8 -2.168573 -1.738779 -0.000004
8 -1.358074 2.763124 -0.000005
1 1.224532 2.088755 0.000017
6 0.447369 -2.456639 0.000003
1 0.040576 -2.939106 0.890756
1 0.040553 -2.939117 -0.890734
1 1.534617 -2.496512 -0.000010
1 -2.776783 0.691747 -0.000009
8 4.013154 0.331136 -0.000012
1 4.906726 -0.041478 0.000010
1 4.144275 1.290703 0.000017
rc_1mU/rc_1mUado.xyz
6 0.278011 -0.424803 -0.487969
6 -2.094421 -0.503356 0.153787
6 -2.014641 0.924624 0.442903

6 -0.808234 1.613909 0.256923
1 -0.728290 2.674933 0.467699
7 0.278921 0.991965 -0.180959
7 -0.907425 -1.072800 -0.291015
8 1.292654 -0.956187 -0.892350
8 -3.118981 -1.167036 0.286573
1 -2.897933 1.440387 0.798478
6 1.557251 1.696492 -0.366005
1 2.278907 1.296908 0.350233
1 1.913068 1.512595 -1.380511
1 1.391645 2.757472 -0.195575
1 -0.912728 -2.068174 -0.514459
8 3.692818 -1.091789 0.902477
1 2.955230 -0.980721 0.280540
1 3.959899 -0.188663 1.125982
rc_1mU/rc_1mUaca.xyz
6 0.475942 1.109635 0.001272
6 2.398490 -0.426418 0.000847
6 1.451646 -1.537281 -0.001224
6 0.072748 -1.286277 -0.001824
1 -0.647330 -2.097673 -0.003035
7 -0.399570 -0.046864 -0.000866
7 1.815972 0.833632 0.002035
8 0.000466 2.225149 0.002211
8 3.617811 -0.576113 0.001603
1 1.830310 -2.551511 -0.002190
6 -1.844756 0.230382 -0.000722
1 -2.089507 0.807662 0.892804
1 -2.087299 0.816592 -0.889037
1 -2.371365 -0.720293 -0.003904
1 2.435270 1.643900 0.003985
8 -5.073806 -0.528454 0.102790
1 -6.021350 -0.521911 -0.095975
1 -4.643734 -0.389046 -0.753762
rc_5caC/rc_5caCacr.xyz
6 -1.520415 1.225658 -0.069863

6 -0.016861 -0.674851 -0.010469
6 1.118477 0.220045 -0.083692
6 0.864069 1.590103 -0.114690
1 1.665059 2.320670 -0.143380
7 -0.383951 2.049617 -0.127364
7 -1.256123 -0.126207 -0.000328
8 -2.646546 1.689707 -0.079188
7 0.113121 -1.980821 0.042752
1 -0.702350 -2.585276 0.108724
1 1.030189 -2.413774 -0.012301
6 2.523162 -0.269527 -0.027362
8 2.999569 -1.126163 -0.777902
8 3.105722 0.302553 0.962308
1 -2.100564 -0.761101 0.058928
1 -0.563456 3.052943 -0.175663
8 -3.297098 -1.889948 0.189767
1 -3.806420 -1.943160 -0.633847
1 -3.937559 -1.626175 0.868697
rc_5caC/rc_5caCabd.xyz
6 1.523307 -1.215552 -0.073055
6 0.011475 0.677772 -0.011932
6 -1.120075 -0.221768 -0.084501
6 -0.859549 -1.590714 -0.115597
1 -1.657441 -2.324740 -0.143024
7 0.390553 -2.044731 -0.129880
7 1.252498 0.134807 -0.003996
8 2.651997 -1.673561 -0.082648
7 -0.122829 1.983363 0.042447
1 0.689906 2.591109 0.109145
1 -1.041344 2.413575 -0.009705
6 -2.526488 0.262185 -0.026078
8 -3.007487 1.116585 -0.776222
8 -3.105276 -0.311631 0.964815
1 2.095140 0.772457 0.060505
1 0.574515 -3.047270 -0.177679
8 3.334328 1.838017 0.259535

1 4.181597 1.368873 0.206788
1 3.355562 2.485105 -0.462892
rc_5caC/rc_5caCaay.xyz
6 -1.555452 1.039251 -0.057806
6 0.881587 1.160334 -0.011616
6 0.936239 -0.280616 0.071086
6 -0.273309 -0.980291 0.071748
1 -0.300064 -2.063853 0.113747
7 -1.439434 -0.350188 0.036315
7 -0.350875 1.729365 -0.084194
8 -2.635184 1.599350 -0.107446
7 1.956557 1.912666 -0.014304
1 1.889970 2.925290 -0.084697
1 2.879580 1.498039 0.076239
6 2.223094 -1.031744 0.037441
8 3.147804 -0.881253 0.841968
8 2.200063 -1.794593 -0.991087
1 -0.434439 2.745492 -0.143219
1 -2.344366 -0.903232 0.068702
8 -3.710193 -1.802939 0.086404
1 -3.702473 -2.448179 0.810964
1 -4.444814 -1.202577 0.289703
rc_5caC/rc_5caCaaw.xyz
6 -1.526955 1.207106 -0.074747
6 -0.008727 -0.681435 -0.032053
6 1.120054 0.223196 -0.084181
6 0.854446 1.591593 -0.101002
1 1.649822 2.328758 -0.115658
7 -0.397118 2.041232 -0.116901
7 -1.251787 -0.143252 -0.024453
8 -2.657185 1.661521 -0.081714
7 0.130336 -1.987290 0.005741
1 -0.680739 -2.599317 0.051427
1 1.050908 -2.412921 -0.047642
6 2.528629 -0.254423 -0.019765
8 3.023077 -1.091995 -0.779911

8 3.092249 0.305244 0.987743
1 -2.094737 -0.783720 0.028371
1 -0.584791 3.043538 -0.153650
8 -3.336710 -1.849031 0.119416
1 -4.174243 -1.359659 0.121969
1 -3.312371 -2.313642 0.970694
rc_5caC/rc_5caCaaj.xyz
6 1.555778 1.035658 0.056394
6 -0.881101 1.159403 0.011045
6 -0.937229 -0.281412 -0.072723
6 0.271705 -0.982277 -0.077550
1 0.297201 -2.065756 -0.121940
7 1.438593 -0.353401 -0.042945
7 0.351938 1.727009 0.083630
8 2.635834 1.594775 0.109824
7 -1.955330 1.912825 0.014695
1 -1.887638 2.925331 0.085723
1 -2.878690 1.499071 -0.076465
6 -2.224959 -1.030695 -0.035081
8 -3.152950 -0.877353 -0.835316
8 -2.199129 -1.795089 0.992261
1 0.436646 2.742909 0.144887
1 2.343462 -0.907036 -0.078163
8 3.692898 -1.828679 -0.111869
1 4.433146 -1.258797 0.150258
1 3.891082 -2.094059 -1.023679
rc_5caC/rc_5caCacf.xyz
6 -1.521709 1.362503 -0.061313
6 -0.098728 -0.610487 -0.035639
6 1.073213 0.235210 -0.091896
6 0.877670 1.615299 -0.105032
1 1.709444 2.310731 -0.124028
7 -0.348560 2.132544 -0.111751
7 -1.312428 0.000372 -0.009106
8 -2.626999 1.872637 -0.060922
7 -0.043412 -1.921027 -0.012078

1 -0.907562 -2.465312 0.044167
1 0.849021 -2.402587 -0.067392
6 2.456019 -0.313359 -0.026461
8 2.914078 -1.169581 -0.787620
8 3.040432 0.214962 0.987016
1 -2.153488 -0.609251 0.042803
1 -0.479429 3.143958 -0.146626
8 -2.931541 -2.219881 0.184950
1 -3.397986 -2.351631 1.024473
1 -3.575753 -2.429223 -0.508210
rc_5caC/rc_5caCaao.xyz
6 1.547208 1.089007 0.038733
6 -0.892964 1.160628 0.024802
6 -0.920170 -0.279977 -0.073826
6 0.303649 -0.953660 -0.102024
1 0.352070 -2.035807 -0.158019
7 1.458114 -0.301064 -0.076792
7 0.328391 1.752159 0.094072
8 2.614056 1.673054 0.083800
7 -1.980663 1.893900 0.041886
1 -1.932219 2.906807 0.122374
1 -2.897143 1.464326 -0.044237
6 -2.191591 -1.057006 -0.036212
8 -3.121352 -0.925540 -0.838103
8 -2.149847 -1.820403 0.991152
1 0.392514 2.768779 0.164796
1 2.367407 -0.845047 -0.114536
8 3.720870 -1.767841 -0.155420
1 3.829969 -2.239676 0.685019
1 3.659908 -2.462464 -0.829830
rc_5caC/rc_5caCaap.xyz
6 -1.521256 1.256139 -0.069878
6 -0.036318 -0.660041 -0.018853
6 1.108365 0.223246 -0.084142
6 0.867225 1.596065 -0.107001
1 1.675295 2.318992 -0.129401

7 -0.375949 2.068473 -0.118804
7 -1.269970 -0.098827 -0.009857
8 -2.642212 1.731948 -0.078270
7 0.077442 -1.967877 0.028747
1 -0.746257 -2.561501 0.093051
1 0.989058 -2.412260 -0.025871
6 2.508383 -0.280099 -0.027168
8 2.978695 -1.136294 -0.782004
8 3.093498 0.279724 0.967974
1 -2.115573 -0.731148 0.042586
1 -0.544593 3.074022 -0.159825
8 -3.344632 -1.835911 0.168860
1 -3.249071 -2.525076 -0.507433
1 -3.270709 -2.303009 1.016058
rc_5caC/rc_5caCacq.xyz
6 -1.522485 1.230944 -0.072695
6 -0.021588 -0.671267 -0.013666
6 1.115179 0.222158 -0.081975
6 0.862441 1.592757 -0.109605
1 1.664364 2.322448 -0.134294
7 -0.384842 2.053879 -0.123157
7 -1.259948 -0.121689 -0.010183
8 -2.647812 1.696786 -0.081907
7 0.105634 -1.977656 0.041347
1 -0.711077 -2.579927 0.107965
1 1.021762 -2.412975 -0.010157
6 2.519305 -0.269030 -0.025297
8 2.995546 -1.123527 -0.778426
8 3.101344 0.298973 0.966931
1 -2.106198 -0.756565 0.038254
1 -0.563122 3.057618 -0.167329
8 -3.374003 -1.801546 0.103089
1 -3.081385 -2.715851 -0.038041
1 -3.767949 -1.795353 0.989485
rc_5caC/rc_5caCadf.xyz
6 -2.582445 -0.291412 -0.011269

6 -0.191505 -0.825925 -0.001539
6 0.108779 0.588511 -0.077652
6 -0.956961 1.486126 -0.059068
1 -0.797188 2.558790 -0.070242
7 -2.218176 1.061745 -0.046213
7 -1.513195 -1.168350 0.010102
8 -3.743942 -0.651570 0.002689
7 0.719681 -1.761746 0.052437
1 0.433173 -2.736960 0.112131
1 1.745148 -1.567733 0.051566
6 1.501462 1.135182 -0.091414
8 2.282109 1.082456 -1.042414
8 1.698802 1.638932 1.068179
1 -1.770035 -2.155689 0.046578
1 -2.987096 1.732442 -0.057017
8 3.478056 -1.482766 0.055424
1 3.656219 -0.635013 -0.383320
1 3.805423 -1.368680 0.960643
rc_5caC/rc_5caCaat.xyz
6 -1.359359 1.663560 -0.024675
6 -0.217298 -0.497018 0.005089
6 1.053890 0.187939 -0.100336
6 1.047904 1.578995 -0.161680
1 1.964889 2.152691 -0.233323
7 -0.095679 2.260144 -0.148762
7 -1.335716 0.283457 0.059576
8 -2.383690 2.318721 0.002285
7 -0.334194 -1.798040 0.039859
1 -1.277128 -2.241844 0.090586
1 0.494709 -2.379989 -0.043168
6 2.351163 -0.537503 -0.033975
8 2.665439 -1.493845 -0.747788
8 3.034958 -0.032020 0.928104
1 -2.251891 -0.162280 0.131588
1 -0.088471 3.277755 -0.218898
8 -2.940730 -2.738372 0.096437

1 -3.391401 -2.273597 -0.626064
1 -3.357209 -2.403363 0.905725
rc_5caC/rc_5caCabh.xyz
6 -2.578681 -0.323599 -0.023179
6 -0.179577 -0.817369 0.027592
6 0.098042 0.599422 -0.082621
6 -0.982420 1.479029 -0.099499
1 -0.839626 2.553359 -0.136747
7 -2.236571 1.034035 -0.088909
7 -1.495453 -1.181909 0.027383
8 -3.733937 -0.703395 -0.010825
7 0.746396 -1.734799 0.125130
1 0.475983 -2.712767 0.210025
1 1.768648 -1.530036 0.113784
6 1.480244 1.169784 -0.080026
8 2.308063 1.052255 -0.984585
8 1.619067 1.766892 1.044083
1 -1.738003 -2.171679 0.086412
1 -3.016298 1.691298 -0.125036
8 3.512024 -1.433800 0.159527
1 3.907489 -2.136993 -0.377689
1 3.673815 -0.613701 -0.335188
rc_5caC/rc_5caCabn.xyz
6 -2.573349 -0.483822 0.001051
6 -0.131321 -0.766761 -0.004790
6 0.014577 0.667105 0.002024
6 -1.129584 1.435532 0.007277
1 -1.089447 2.519392 0.012324
7 -2.352924 0.889145 0.006693
7 -1.414509 -1.241384 -0.002779
8 -3.689018 -0.978013 0.000242
7 0.849540 -1.637946 -0.014148
1 0.635466 -2.631360 -0.021298
1 1.849894 -1.395530 -0.017743
6 1.291017 1.360531 0.002732
8 2.441557 0.829346 0.013536

8 1.413674 2.633504 -0.007994
1 -1.570691 -2.248959 -0.007432
1 -3.180629 1.482285 0.010776
8 3.690579 -1.758779 -0.069499
1 4.114542 -0.912153 -0.275775
1 3.983738 -1.966356 0.830738
rc_5caC/rc_5caCaby.xyz
6 2.570231 -0.496703 -0.005972
6 0.126585 -0.760662 0.005413
6 -0.008005 0.674026 0.006058
6 1.141442 1.434141 0.012617
1 1.109924 2.518312 0.016835
7 2.360409 0.877870 0.010588
7 1.405698 -1.245687 -0.007223
8 3.682185 -0.999047 -0.016982
7 -0.862532 -1.622367 0.018123
1 -0.658446 -2.617869 0.018761
1 -1.860235 -1.370340 0.037196
6 -1.281265 1.371824 -0.012211
8 -2.430640 0.843933 -0.075140
8 -1.402306 2.645177 0.030002
1 1.553558 -2.254599 -0.011530
1 3.192844 1.464359 0.012840
8 -3.706842 -1.718147 0.119424
1 -4.158433 -0.918638 -0.189996
1 -3.937327 -2.401013 -0.528391
rc_5caC/rc_5caCaar.xyz
6 -1.839998 -0.328089 -0.658824
6 0.253545 0.913485 -0.520798
6 0.909053 -0.269247 -0.010739
6 0.157014 -1.433601 0.113552
1 0.593956 -2.359288 0.470469
7 -1.138004 -1.446823 -0.191660
7 -1.060873 0.803468 -0.844841
8 -3.030974 -0.361352 -0.898223
7 0.875518 2.056458 -0.675366

1 0.399148 2.870854 -1.056160
1 1.848513 2.155611 -0.398442
6 2.366875 -0.296828 0.275824
8 2.964411 0.514125 0.988245
8 2.890972 -1.259226 -0.395675
1 -1.565733 1.622120 -1.186881
1 -1.689373 -2.294402 -0.056767
8 -2.295252 0.554100 2.090815
1 -3.176113 0.500654 1.691239
1 -2.459075 0.517234 3.044225
rc_5caC/rc_5caCabo.xyz
6 -2.786034 -0.125956 -0.059242
6 -0.625772 1.010433 -0.037499
6 0.029325 -0.261965 0.165905
6 -0.762849 -1.406459 0.228937
1 -0.332422 -2.392587 0.363338
7 -2.087891 -1.324620 0.144433
7 -1.980327 0.999728 -0.142362
8 -3.996415 -0.085918 -0.150925
7 0.030295 2.141685 -0.115821
1 -0.453654 3.023202 -0.269945
1 1.039420 2.165272 0.004837
6 1.511316 -0.384852 0.193356
8 2.265136 0.272159 0.918566
8 1.841398 -1.223840 -0.718933
1 -2.481705 1.878679 -0.279243
1 -2.660541 -2.166196 0.221459
8 4.912867 0.056825 -0.226930
1 4.050488 0.139620 0.224210
1 4.724073 -0.506560 -0.991373
rc_5caC/rc_5caCadk.xyz
6 1.545431 0.659949 -0.099871
6 -0.844233 1.110384 0.094464
6 -1.112925 -0.295288 -0.107762
6 -0.032240 -1.148730 -0.301332
1 -0.164964 -2.213328 -0.458238

7 1.216543 -0.682192 -0.313983
7 0.460457 1.496976 0.095450
8 2.696185 1.060052 -0.089363
7 -1.791313 2.000287 0.261996
1 -1.572939 2.982680 0.410977
1 -2.769047 1.724747 0.217266
6 -2.484978 -0.853824 -0.031004
8 -3.473409 -0.390927 -0.607199
8 -2.490093 -1.855178 0.778539
1 0.696259 2.481588 0.228936
1 2.004324 -1.315215 -0.462343
8 4.683842 -1.130349 0.088860
1 4.700899 -1.348836 1.031794
1 4.147127 -0.320867 0.033701
rc_5caC/rc_5caCado.xyz
6 -2.748867 -0.376656 0.118208
6 -0.786337 1.064296 -0.078750
6 0.062275 -0.104569 -0.026202
6 -0.546497 -1.355329 0.065118
1 0.031803 -2.272031 0.083323
7 -1.869265 -1.467018 0.151557
7 -2.128059 0.856193 -0.010739
8 -3.953570 -0.510181 0.196912
7 -0.316595 2.283811 -0.176841
1 -0.939247 3.087845 -0.212293
1 0.684878 2.456429 -0.189946
6 1.545500 -0.024781 -0.140327
8 2.279686 0.636307 0.600524
8 1.886228 -0.719434 -1.161350
1 -2.763119 1.655795 -0.033515
1 -2.302994 -2.386735 0.239868
8 4.965893 -0.436436 0.413824
1 4.814873 -1.221158 -0.132739
1 4.088910 -0.010853 0.469900
rc_5caC/rc_5caCacg.xyz
6 2.756136 -0.361451 -0.123558

6 0.781311 1.061444 0.082143
6 -0.057044 -0.114943 0.029720
6 0.562901 -1.360105 -0.065529
1 -0.007017 -2.282046 -0.083654
7 1.886321 -1.459709 -0.155807
7 2.124566 0.865416 0.009664
8 3.961709 -0.483904 -0.206543
7 0.301452 2.276770 0.184174
1 0.917174 3.086229 0.219113
1 -0.701401 2.440851 0.200143
6 -1.540832 -0.049560 0.148260
8 -2.282789 0.601541 -0.593801
8 -1.872554 -0.744070 1.171888
1 2.752820 1.670386 0.032225
1 2.327989 -2.375333 -0.247155
8 -4.988599 -0.407753 -0.445768
1 -4.942112 -1.071485 0.257436
1 -4.090801 -0.024763 -0.476753
rc_5caC/rc_5caCabv.xyz
6 -2.282091 0.089013 -0.159835
6 -0.289218 -1.198850 0.407865
6 0.512412 -0.293519 -0.386790
6 -0.133675 0.766087 -1.024857
1 0.407661 1.487262 -1.626766
7 -1.449951 0.920182 -0.926141
7 -1.621827 -0.944425 0.487096
8 -3.480203 0.268044 -0.080010
7 0.221846 -2.231484 1.030040
1 -0.357626 -2.854805 1.588347
1 1.211331 -2.448743 0.939037
6 1.994706 -0.404190 -0.451829
8 2.602077 -1.441106 -0.738076
8 2.511901 0.714761 -0.104614
1 -2.224096 -1.564625 1.031414
1 -1.915143 1.688018 -1.412714
8 1.131712 3.007854 1.332104

1 0.284853 3.053590 0.865479
1 1.605838 2.281723 0.895690
rc_5caU/rc_5caUaaq.xyz
6 -1.622082 0.986371 0.039924
6 0.825600 1.264567 0.073987
6 0.937400 -0.206322 -0.006768
6 -0.240664 -0.991004 -0.019485
1 -0.199451 -2.075499 -0.052731
7 -1.422063 -0.422144 -0.019189
7 -0.478066 1.740400 0.071529
8 -2.751044 1.438287 0.057099
8 1.787574 2.018373 0.146295
1 -0.603619 2.750451 0.134279
6 2.247953 -0.912239 0.019496
8 2.368643 -1.900858 0.727441
8 3.180238 -0.373116 -0.745398
1 4.014922 -0.876454 -0.665984
1 -2.324450 -1.019562 -0.077353
8 -3.627847 -1.831572 -0.260968
1 -4.363821 -1.200875 -0.204608
1 -3.772426 -2.462992 0.461346
rc_5caU/rc_5caUacr.xyz
6 -2.817040 0.071157 0.130902
6 -0.654718 -1.074170 -0.176522
6 0.006342 0.237386 -0.028341
6 -0.781164 1.400178 0.098355
1 -0.343401 2.392064 0.143971
7 -2.090150 1.298699 0.191558
7 -2.039661 -1.040033 -0.059738
8 -4.026325 0.074947 0.237715
8 -0.050035 -2.116818 -0.386767
1 -2.534337 -1.928721 -0.139944
6 1.481910 0.386019 -0.147468
8 1.861211 1.212130 -0.979096
8 2.212064 -0.375668 0.605555
1 3.233092 -0.236179 0.446967

1 -2.661858 2.137599 0.321763
8 4.705205 -0.097518 0.240195
1 4.989537 0.804145 0.460623
1 5.196701 -0.679575 0.841501
rc_5caU/rc_5caUaba.xyz
6 1.621609 -0.989940 0.038197
6 -0.826518 -1.264536 0.076697
6 -0.936311 0.206326 -0.006976
6 0.242910 0.989334 -0.019694
1 0.203267 2.073890 -0.052592
7 1.423574 0.418882 -0.019146
7 0.476468 -1.742236 0.072576
8 2.749840 -1.443741 0.051424
8 -1.789402 -2.016836 0.152415
1 0.600570 -2.752434 0.135896
6 -2.245888 0.914088 0.017390
8 -2.365232 1.905187 0.722091
8 -3.178996 0.373619 -0.745577
1 -4.013047 0.878224 -0.667629
1 2.325935 1.016724 -0.073546
8 3.614673 1.854760 -0.243313
1 4.337683 1.234127 -0.429907
1 3.863413 2.297402 0.583774
rc_5caU/rc_5caUadc.xyz
6 -2.814862 0.069595 0.113131
6 -0.648241 -1.072371 -0.172368
6 0.008733 0.239811 -0.015595
6 -0.780775 1.401742 0.099150
1 -0.345248 2.394497 0.148095
7 -2.090867 1.298273 0.178870
7 -2.034222 -1.040560 -0.071685
8 -4.025031 0.070985 0.210219
8 -0.038300 -2.113172 -0.377308
1 -2.526865 -1.929945 -0.156127
6 1.486412 0.383780 -0.113597
8 1.879754 1.175146 -0.972076

8 2.203285 -0.344684 0.682922
1 3.227356 -0.210122 0.525606
1 -2.665122 2.136371 0.302669
8 4.691004 -0.023124 0.353431
1 4.848085 0.426985 -0.492792
1 5.144111 -0.878334 0.280433
rc_5caU/rc_5caUaaw.xyz
6 -2.814413 -0.087812 -0.124617
6 -0.662059 1.078605 0.173774
6 0.010924 -0.227083 0.025487
6 -0.766097 -1.397653 -0.095856
1 -0.318512 -2.385113 -0.141385
7 -2.076201 -1.308800 -0.184217
7 -2.047141 1.030860 0.062328
8 -4.023788 -0.103330 -0.228926
8 -0.067009 2.127470 0.380126
1 -2.550055 1.914945 0.142492
6 1.488581 -0.362928 0.134951
8 1.882659 -1.195315 0.953688
8 2.207257 0.415612 -0.612057
1 3.229913 0.272575 -0.460983
1 -2.640457 -2.153268 -0.310815
8 4.697567 0.122661 -0.259740
1 5.160507 0.063525 -1.110682
1 4.866897 -0.722645 0.187430
rc_5caU/rc_5caUabx.xyz
6 1.616619 1.030609 -0.029541
6 -0.835711 1.269193 -0.067785
6 -0.923846 -0.203063 0.011560
6 0.266632 -0.968183 0.028999
1 0.244209 -2.053341 0.059888
7 1.439198 -0.380611 0.034654
7 0.460031 1.765794 -0.065192
8 2.737402 1.501826 -0.048391
8 -1.810136 2.007104 -0.138964
1 0.569248 2.777687 -0.129170

6 -2.222457 -0.929879 -0.029676
8 -2.322088 -1.909732 -0.752965
8 -3.166585 -0.418806 0.739576
1 -3.992180 -0.935618 0.651040
1 2.343548 -0.974190 0.089652
8 3.631373 -1.822336 0.232455
1 3.434608 -2.757273 0.401965
1 4.138825 -1.810070 -0.594645
rc_5caU/rc_5caUadr.xyz
6 -1.619787 0.999115 0.037580
6 0.829476 1.264880 0.074801
6 0.934020 -0.206184 -0.010710
6 -0.247999 -0.984892 -0.025753
1 -0.212431 -2.069538 -0.060084
7 -1.426732 -0.410219 -0.024760
7 -0.471789 1.747096 0.072913
8 -2.746122 1.457300 0.053875
8 1.795086 2.013727 0.150181
1 -0.592228 2.757601 0.138471
6 2.240955 -0.918545 0.018684
8 2.353884 -1.908595 0.725970
8 3.178684 -0.382962 -0.741898
1 4.010901 -0.889949 -0.659823
1 -2.328640 -1.007274 -0.077145
8 -3.603773 -1.878574 -0.227081
1 -4.310178 -1.311944 -0.577238
1 -3.919836 -2.170443 0.642778
rc_5caU/rc_5caUade.xyz
6 -1.615879 1.039847 0.034426
6 0.837529 1.270355 0.064135
6 0.920608 -0.202485 -0.009542
6 -0.272442 -0.963645 -0.020572
1 -0.254121 -2.048999 -0.047602
7 -1.443204 -0.372311 -0.024582
7 -0.456586 1.771121 0.065477
8 -2.734731 1.515397 0.053479

8 1.814865 2.005141 0.127583
1 -0.562314 2.783675 0.124690
6 2.216934 -0.933345 0.031015
8 2.316096 -1.908215 0.761054
8 3.159247 -0.431508 -0.746398
1 3.983269 -0.951031 -0.658544
1 -2.347245 -0.965519 -0.077789
8 -3.630929 -1.822598 -0.217957
1 -3.463547 -2.650639 -0.695268
1 -3.974405 -2.089254 0.649392
rc_5caU/rc_5caUacs.xyz
6 -1.619914 1.001698 0.039222
6 0.829367 1.266412 0.069483
6 0.932907 -0.205114 -0.008520
6 -0.249427 -0.983095 -0.022099
1 -0.214647 -2.067854 -0.054884
7 -1.427806 -0.407692 -0.023071
7 -0.471599 1.749387 0.069619
8 -2.746149 1.460099 0.060294
8 1.795839 2.014906 0.137635
1 -0.591458 2.760153 0.132024
6 2.239483 -0.918051 0.024111
8 2.353250 -1.901521 0.740350
8 3.175484 -0.390851 -0.744378
1 4.007251 -0.898311 -0.660431
1 -2.331138 -1.003295 -0.082328
8 -3.624774 -1.830968 -0.267716
1 -4.377683 -1.316636 0.065339
1 -3.620198 -2.650354 0.251783
rc_5caU/rc_5caUaal.xyz
6 -1.621965 0.993864 0.042343
6 0.826612 1.266497 0.062994
6 0.934977 -0.205631 0.000034
6 -0.244940 -0.987746 0.005301
1 -0.206847 -2.072770 -0.011380
7 -1.425200 -0.416059 0.004283

7 -0.475986 1.745290 0.060549
8 -2.749412 1.449336 0.057162
8 1.790981 2.018447 0.121352
1 -0.599022 2.756380 0.110053
6 2.244502 -0.913417 0.025338
8 2.370595 -1.889374 0.749600
8 3.169068 -0.390702 -0.759931
1 4.003227 -0.895160 -0.681637
1 -2.325795 -1.014701 -0.043668
8 -3.612313 -1.883468 -0.133532
1 -3.791975 -2.109037 -1.060426
1 -4.357751 -1.324645 0.139968
rc_5caU/rc_5caUacp.xyz
6 1.623571 -0.985671 0.043721
6 -0.823971 -1.266360 0.063117
6 -0.937199 0.205574 0.001898
6 0.240139 0.991542 0.009923
1 0.198438 2.076459 -0.005014
7 1.422084 0.423605 0.009321
7 0.480235 -1.741029 0.060339
8 2.752940 -1.436548 0.057132
8 -1.785857 -2.021528 0.120526
1 0.606551 -2.751789 0.108200
6 -2.248938 0.909447 0.024042
8 -2.379605 1.886569 0.745895
8 -3.170550 0.382207 -0.761797
1 -4.006283 0.884385 -0.685858
1 2.322908 1.022496 -0.037655
8 3.630763 1.854727 -0.116561
1 4.365860 1.220055 -0.118898
1 3.693158 2.319770 -0.966169
rc_5caU/rc_5caUaau.xyz
6 -1.581345 1.178676 0.082702
6 -0.052473 -0.729517 -0.040333
6 1.077507 0.222008 0.036715
6 0.813316 1.610955 0.055284

1 1.608363 2.350643 0.055966
7 -0.429551 2.032621 0.094940
7 -1.310165 -0.154592 0.007369
8 -2.689539 1.676354 0.129453
8 0.100042 -1.941660 -0.145233
1 -2.138063 -0.802094 -0.051024
6 2.497240 -0.217252 -0.028179
8 3.257837 0.387468 -0.769529
8 2.791377 -1.256467 0.729372
1 3.730352 -1.506447 0.614849
1 -0.632493 3.035524 0.135722
8 -3.437055 -1.865681 -0.241255
1 -4.025494 -1.849300 0.529364
1 -3.071422 -2.763846 -0.260644
rc_5caU/rc_5caUabk.xyz
6 -1.582698 1.138465 0.087072
6 -0.028463 -0.747685 -0.047465
6 1.088263 0.218720 0.037705
6 0.805766 1.603673 0.067302
1 1.590677 2.354093 0.074952
7 -0.442788 2.007933 0.108749
7 -1.293809 -0.190478 0.001957
8 -2.698010 1.620342 0.134661
8 0.141933 -1.956761 -0.159651
1 -2.118023 -0.842902 -0.062036
6 2.513471 -0.201610 -0.032143
8 3.263529 0.415418 -0.774082
8 2.823760 -1.238422 0.722048
1 3.765863 -1.474925 0.604810
1 -0.659470 3.007523 0.157818
8 -3.462810 -1.841877 -0.244344
1 -3.485008 -2.514063 0.454502
1 -4.263124 -1.310897 -0.108875
rc_5caU/rc_5caUaca.xyz
6 1.584682 1.120139 -0.086531
6 0.019002 -0.757068 0.039665

6 -1.091411 0.217532 -0.034740
6 -0.800417 1.600814 -0.055672
1 -1.580575 2.356238 -0.056726
7 0.450587 1.997403 -0.096759
7 1.287646 -0.207878 -0.012373
8 2.702981 1.595085 -0.134037
8 -0.159363 -1.965473 0.146141
1 2.110858 -0.863136 0.032734
6 -2.519282 -0.193399 0.034401
8 -3.262123 0.420228 0.786382
8 -2.840322 -1.217496 -0.732439
1 -3.784843 -1.445594 -0.617598
1 0.673755 2.995842 -0.139112
8 3.493481 -1.827784 0.076694
1 4.248590 -1.219546 0.105729
1 3.531896 -2.315071 0.914217
rc_5caU/rc_5caUadk.xyz
6 2.291685 0.093003 0.158158
6 0.148432 -1.128748 0.319716
6 -0.543341 0.178635 0.272218
6 0.230184 1.362906 0.277342
1 -0.233279 2.342918 0.312717
7 1.540453 1.303274 0.214770
7 1.535275 -1.045774 0.230524
8 3.502223 0.127170 0.065175
8 -0.415026 -2.207963 0.428917
1 2.049602 -1.926627 0.231224
6 -2.022469 0.331695 0.390325
8 -2.498016 1.304032 0.956493
8 -2.712660 -0.654202 -0.165381
1 -3.669162 -0.493873 -0.045952
1 2.093089 2.163618 0.190613
8 -0.560992 0.365135 -2.226201
1 0.296257 -0.047404 -2.426532
1 -1.217784 -0.339444 -2.357695
rc_5caU/rc_5caUabw.xyz

6 2.286007 -0.094028 -0.169925
6 0.143029 1.127812 -0.314363
6 -0.550108 -0.179208 -0.265905
6 0.224018 -1.363641 -0.279124
1 -0.239152 -2.343924 -0.313407
7 1.534441 -1.303666 -0.228080
7 1.528617 1.044965 -0.224883
8 3.497339 -0.128550 -0.088296
8 -0.419773 2.207624 -0.422234
1 2.042874 1.925864 -0.227036
6 -2.028964 -0.330195 -0.385836
8 -2.504240 -1.312855 -0.935162
8 -2.721060 0.667827 0.145566
1 -3.676669 0.507611 0.018580
1 2.087460 -2.163947 -0.211426
8 -0.387666 -0.299161 2.242715
1 -1.211018 -0.786269 2.416694
1 -0.605587 0.628056 2.437540
rc_5caU/rc_5caUacw.xyz
6 2.304589 0.094365 0.143260
6 0.163448 -1.132429 0.300675
6 -0.531618 0.174002 0.271779
6 0.240789 1.359318 0.276849
1 -0.224093 2.338403 0.318356
7 1.550760 1.302653 0.204335
7 1.549192 -1.046007 0.199650
8 3.515543 0.131365 0.056976
8 -0.395559 -2.214381 0.405546
1 2.065398 -1.925733 0.199409
6 -2.009444 0.327295 0.409221
8 -2.480840 1.313344 0.954721
8 -2.706515 -0.674224 -0.110139
1 -3.661216 -0.509501 0.016891
1 2.101126 2.164544 0.181999
8 -0.669824 0.384953 -2.205025
1 0.200501 0.025772 -2.447209

1 -1.290415 -0.353767 -2.324666
rc_5caU/rc_5caUaaz.xyz
6 -2.281363 -0.087570 0.177599
6 -0.137034 1.133511 0.308890
6 0.552900 -0.173926 0.267925
6 -0.220166 -1.358406 0.285829
1 0.243599 -2.338324 0.322296
7 -1.530838 -1.297834 0.237331
7 -1.523145 1.051914 0.225404
8 -3.492940 -0.121252 0.100217
8 0.430783 2.211721 0.409013
1 -2.037571 1.932710 0.227719
6 2.032036 -0.317799 0.393015
8 2.504552 -1.245801 1.031375
8 2.720435 0.624429 -0.234509
1 3.677669 0.479206 -0.101555
1 -2.084234 -2.157905 0.221639
8 0.413401 -0.413683 -2.255290
1 -0.055699 0.411450 -2.463884
1 1.346029 -0.223870 -2.451362
rc_5caU/rc_5caUacn.xyz
6 -2.290932 -0.101660 0.162290
6 -0.145728 1.114424 0.333970
6 0.546340 -0.192310 0.251325
6 -0.228814 -1.374875 0.244222
1 0.232769 -2.356297 0.259672
7 -1.541054 -1.312870 0.197266
7 -1.532319 1.034940 0.243663
8 -3.502400 -0.133440 0.077565
8 0.416747 2.190074 0.472752
1 -2.045291 1.916203 0.266514
6 2.025120 -0.349951 0.376727
8 2.495822 -1.352846 0.892569
8 2.722351 0.665737 -0.111581
1 3.676868 0.497611 0.012894
1 -2.094616 -2.172268 0.165969

8 0.547356 -0.040226 -2.219116
1 -0.317346 -0.404792 -2.473489
1 1.186299 -0.743104 -2.426786
rc_5caU/rc_5caUacy.xyz
6 -2.586658 -0.429615 -0.002536
6 -0.157645 -0.842312 0.016964
6 0.040125 0.620658 0.060479
6 -1.079545 1.478575 -0.010084
1 -0.975029 2.558585 -0.040653
7 -2.291467 0.970106 -0.019323
7 -1.484375 -1.243500 0.009818
8 -3.738947 -0.806622 -0.007868
8 0.761322 -1.650290 -0.017734
1 -1.668986 -2.246893 -0.008434
6 1.397179 1.228543 0.067330
8 1.640826 2.088715 -0.765321
8 2.212914 0.746968 0.979785
1 3.098860 1.155036 0.901986
1 -3.108399 1.587872 -0.043175
8 3.730227 -1.580516 -0.274268
1 2.782463 -1.432064 -0.116437
1 3.770517 -2.429915 -0.736418
rc_5dhmC/rc_5dhmCadu.xyz
6 1.656761 1.235942 -0.005438
6 0.042900 -0.562144 -0.001417
6 -1.000872 0.427884 -0.183101
6 -0.588673 1.783002 -0.242464
1 -1.353946 2.545632 -0.362977
7 0.655828 2.197551 -0.161770
7 1.311096 -0.108938 0.070498
8 2.840594 1.560738 0.064784
6 -2.483959 0.119233 -0.187910
1 -3.017335 0.916413 -0.712155
7 -0.199940 -1.852733 0.092018
1 0.558301 -2.516828 0.228159
1 -1.152836 -2.191462 -0.024127

8 -2.970481 0.158654 1.141486
1 -2.548404 -0.543559 1.666589
8 -2.690873 -1.128403 -0.805434
1 -3.646283 -1.273124 -0.899238
1 2.098680 -0.798459 0.132005
8 3.267499 -2.047984 0.132257
1 4.147007 -1.639499 0.138342
1 3.215072 -2.517808 -0.714588
rc_5dhmC/rc_5dhmCabw.xyz
6 -1.718522 -1.324176 -0.027487
6 -0.114559 0.487877 -0.134741
6 0.943084 -0.503365 -0.183809
6 0.541033 -1.862313 -0.170398
1 1.314534 -2.624929 -0.216874
7 -0.703760 -2.281853 -0.110551
7 -1.383369 0.024882 -0.053256
8 -2.898177 -1.655488 0.063912
6 2.418853 -0.169230 -0.106074
1 3.001010 -0.999516 -0.514165
7 0.105415 1.782572 -0.166981
1 -0.684784 2.431018 -0.099601
1 1.060428 2.120551 -0.268569
8 2.798771 -0.077984 1.254568
1 2.318754 0.654497 1.679046
8 2.655150 1.022295 -0.817624
1 3.612441 1.181320 -0.847787
1 -2.145359 0.718017 0.019124
8 -2.651361 2.512488 0.205065
1 -2.981295 2.764397 1.080667
1 -3.278128 2.892195 -0.428638
rc_5dhmC/rc_5dhmCabj.xyz
6 -2.858907 -0.188868 -0.128239
6 -0.712936 0.940150 0.029961
6 -0.073144 -0.354301 -0.101920
6 -0.927556 -1.473592 -0.268653
1 -0.467816 -2.451623 -0.386422

7 -2.240777 -1.427374 -0.294322
7 -2.064963 0.951887 0.010663
8 -4.080399 -0.077969 -0.106722
6 1.415111 -0.576839 0.074051
1 1.699403 -1.514125 -0.412850
7 -0.035547 2.057547 0.158998
1 -0.506301 2.953707 0.256714
1 0.983178 2.017880 0.112416
8 1.664830 -0.788697 1.458884
1 1.500965 0.038772 1.945288
8 2.122719 0.512852 -0.443466
1 3.089252 0.276354 -0.481927
1 -2.562829 1.837706 0.101244
8 4.706252 -0.198986 -0.576697
1 5.265273 0.541505 -0.857960
1 5.025252 -0.431492 0.308931
rc_5dhmC/rc_5dhmCabf.xyz
6 -1.662071 -1.242101 -0.052986
6 -0.043813 0.548962 -0.143327
6 1.005622 -0.453011 -0.183493
6 0.590027 -1.807761 -0.175389
1 1.356223 -2.578154 -0.214800
7 -0.659660 -2.213403 -0.127979
7 -1.315728 0.104335 -0.076398
8 -2.846465 -1.561074 0.030218
6 2.484368 -0.136920 -0.089666
1 3.060416 -0.971584 -0.497468
7 0.202018 1.841163 -0.172427
1 -0.555761 2.518203 -0.129029
1 1.164153 2.161888 -0.267038
8 2.852515 -0.059072 1.274925
1 2.371839 0.671335 1.702017
8 2.742789 1.055739 -0.791923
1 3.701977 1.204909 -0.808697
1 -2.093809 0.800970 0.006459
8 -3.164917 2.120555 0.262653

1 -3.754094 2.252731 -0.495704
1 -3.753526 1.910845 1.004071
rc_5dhmC/rc_5dhmCaad.xyz
6 1.665316 1.247723 -0.054215
6 0.048645 -0.545051 -0.145813
6 -1.001674 0.456381 -0.181086
6 -0.587289 1.811455 -0.170374
1 -1.354268 2.581219 -0.206485
7 0.662034 2.218249 -0.124244
7 1.320088 -0.099334 -0.082687
8 2.849425 1.567003 0.029316
6 -2.480281 0.139508 -0.087046
1 -3.056648 0.975139 -0.492419
7 -0.197319 -1.837335 -0.175469
1 0.558801 -2.516271 -0.136685
1 -1.159889 -2.157339 -0.268467
8 -2.847866 0.057935 1.277456
1 -2.367644 -0.674163 1.702137
8 -2.738735 -1.051296 -0.792423
1 -3.698023 -1.199616 -0.810997
1 2.100198 -0.793697 -0.005541
8 3.267214 -2.046517 0.195877
1 3.467993 -2.163987 1.137252
1 2.907256 -2.899433 -0.092605
rc_5dhmC/rc_5dhmCabc.xyz
6 -2.846636 -0.213252 -0.186741
6 -0.722739 0.944460 0.047406
6 -0.062630 -0.344214 -0.025841
6 -0.898387 -1.480118 -0.173843
1 -0.424386 -2.456858 -0.226218
7 -2.209244 -1.451003 -0.259433
7 -2.072783 0.939371 -0.033066
8 -4.067346 -0.114723 -0.257796
6 1.426424 -0.544297 0.169208
1 1.724388 -1.487753 -0.297075
7 -0.065959 2.073887 0.178435

1 -0.551991 2.966014 0.230566
1 0.953766 2.050620 0.170333
8 1.667546 -0.724518 1.559077
1 1.482811 0.109186 2.026199
8 2.124964 0.541473 -0.368136
1 3.083412 0.290542 -0.462752
1 -2.583290 1.822064 0.004775
8 4.690984 -0.209199 -0.612021
1 5.265524 0.522727 -0.883673
1 4.780302 -0.872071 -1.313826
rc_5dhmC/rc_5dhmCadf.xyz
6 -2.843439 -0.230697 -0.168085
6 -0.728371 0.947118 0.042437
6 -0.056373 -0.334667 -0.043399
6 -0.882368 -1.478363 -0.185268
1 -0.399554 -2.450170 -0.247423
7 -2.194419 -1.461591 -0.254417
7 -2.079303 0.929330 -0.021551
8 -4.066006 -0.145123 -0.221060
6 1.436635 -0.519749 0.136206
1 1.741203 -1.456205 -0.340128
7 -0.080394 2.081788 0.170887
1 -0.573852 2.969267 0.232574
1 0.939542 2.066656 0.150889
8 1.693186 -0.708395 1.522510
1 1.498953 0.117966 1.998990
8 2.118346 0.578262 -0.397736
1 3.081710 0.340988 -0.482594
1 -2.597861 1.806737 0.027248
8 4.694827 -0.131436 -0.603867
1 4.927464 -0.298648 -1.530005
1 4.781884 -0.991592 -0.164095
rc_5dhmC/rc_5dhmCaal.xyz
6 2.456427 -0.072507 -0.094737
6 0.261642 0.653751 0.663446
6 -0.242000 -0.661468 0.319058

6 0.696232 -1.582242 -0.216041
1 0.339183 -2.573652 -0.482391
7 1.970054 -1.337617 -0.419490
7 1.577856 0.873069 0.438305
8 3.634116 0.225964 -0.264794
6 -1.700104 -1.071004 0.348830
1 -1.767381 -2.137853 0.582920
7 -0.489815 1.602203 1.173268
1 -0.104162 2.518153 1.389728
1 -1.474118 1.403006 1.353543
8 -2.231939 -0.918851 -0.947311
1 -2.061368 0.003824 -1.280098
8 -2.382581 -0.305456 1.319012
1 -3.302068 -0.612856 1.364266
1 1.983520 1.779788 0.671330
8 -1.674285 1.532631 -1.951621
1 -1.908512 2.207626 -1.296269
1 -0.707437 1.574895 -2.013249
rc_5dhmC/rc_5dhmCabg.xyz
6 2.522383 0.098368 -0.169697
6 0.286622 0.593679 0.646888
6 -0.107847 -0.740532 0.238934
6 0.901283 -1.557290 -0.332136
1 0.629781 -2.565699 -0.633779
7 2.149203 -1.196058 -0.527843
7 1.574021 0.940008 0.415626
8 3.663003 0.512042 -0.351389
6 -1.535613 -1.247221 0.237921
1 -1.529830 -2.339161 0.298151
7 -0.537777 1.439091 1.220440
1 -0.235248 2.374294 1.483050
1 -1.496480 1.143458 1.404746
8 -2.117690 -0.926900 -1.005653
1 -2.148371 0.059545 -1.136212
8 -2.229656 -0.684620 1.330471
1 -3.127657 -1.053442 1.346593

1 1.901922 1.869911 0.677669
8 -2.220958 1.752695 -1.477264
1 -2.851046 1.922877 -2.194126
1 -2.599759 2.199166 -0.704424
rc_5dhmC/rc_5dhmCadd.xyz
6 -2.854710 -0.207896 -0.104632
6 -0.718313 0.943751 0.010502
6 -0.067414 -0.345367 -0.119576
6 -0.912663 -1.474459 -0.266296
1 -0.444874 -2.448681 -0.383941
7 -2.226525 -1.441348 -0.272070
7 -2.070506 0.942505 0.009202
8 -4.076690 -0.110038 -0.060919
6 1.424538 -0.551848 0.044232
1 1.714622 -1.488564 -0.440717
7 -0.049337 2.067752 0.123058
1 -0.526367 2.960610 0.221085
1 0.969340 2.035135 0.065059
8 1.687294 -0.755689 1.427782
1 1.514287 0.070504 1.913154
8 2.117895 0.542520 -0.483409
1 3.089992 0.324520 -0.491794
1 -2.575542 1.824201 0.100356
8 4.726114 -0.074504 -0.459755
1 4.884832 -0.543960 0.373993
1 4.932757 -0.719519 -1.153495
rc_5dhmC/rc_5dhmCach.xyz
6 -1.658905 -1.246913 -0.041661
6 -0.045398 0.549227 -0.119690
6 1.004702 -0.450174 -0.188277
6 0.592425 -1.805941 -0.196620
1 1.359863 -2.573762 -0.256118
7 -0.655776 -2.214879 -0.141444
7 -1.315306 0.100745 -0.046522
8 -2.842100 -1.568887 0.046337
6 2.483301 -0.132364 -0.107001

1 3.056837 -0.957156 -0.537686
7 0.199451 1.842074 -0.129610
1 -0.556122 2.519889 -0.068353
1 1.160226 2.164714 -0.232474
8 2.866542 -0.080204 1.254795
1 2.382520 0.635240 1.702860
8 2.731121 1.074380 -0.788653
1 3.689813 1.224955 -0.815949
1 -2.096705 0.792183 0.043470
8 -3.229962 2.066286 0.306291
1 -4.100645 1.831605 -0.049852
1 -2.955933 2.851142 -0.193522
rc_5dhmC/rc_5dhmCadt.xyz
6 -1.706105 -1.489161 -0.008630
6 -0.185232 0.405634 -0.120796
6 0.914293 -0.538494 -0.177713
6 0.576627 -1.914642 -0.155879
1 1.385391 -2.639716 -0.202708
7 -0.645623 -2.394088 -0.086488
7 -1.432315 -0.123505 -0.033932
8 -2.868559 -1.874124 0.077551
6 2.375914 -0.141542 -0.123285
1 2.985654 -0.948188 -0.538610
7 -0.028715 1.705763 -0.147897
1 -0.854217 2.334640 -0.067089
1 0.912572 2.080222 -0.252558
8 2.772426 -0.031015 1.231246
1 2.280038 0.693293 1.655547
8 2.552573 1.056600 -0.840303
1 3.502771 1.249245 -0.893272
1 -2.237111 0.501457 0.017334
8 -2.375922 3.178516 0.150452
1 -3.080690 2.741979 -0.352776
1 -2.644956 3.099277 1.078602
rc_5dhmC/rc_5dhmCacc.xyz
6 -1.686942 -1.528148 -0.012384

6 -0.197862 0.392442 -0.117296
6 0.917072 -0.533198 -0.176472
6 0.602892 -1.914911 -0.151879
1 1.424229 -2.625868 -0.195423
7 -0.610884 -2.414867 -0.081972
7 -1.436323 -0.157683 -0.037336
8 -2.843075 -1.932992 0.066469
6 2.372469 -0.113727 -0.127018
1 2.992520 -0.909263 -0.548505
7 -0.061150 1.695149 -0.134659
1 -0.892830 2.315223 -0.052237
1 0.874572 2.083892 -0.236634
8 2.773971 -0.002733 1.226078
1 2.271715 0.711265 1.656101
8 2.528404 1.089621 -0.840073
1 3.475804 1.293551 -0.901070
1 -2.252922 0.452742 0.002956
8 -2.459127 3.074447 0.143170
1 -2.652957 3.235769 1.079256
1 -2.528792 3.942997 -0.281538
rc_5dhmC/rc_5dhmCabx.xyz
6 -2.542477 0.134176 0.185239
6 -0.304192 0.590598 -0.647275
6 0.075553 -0.742456 -0.222826
6 -0.940852 -1.538828 0.363830
1 -0.680530 -2.546327 0.678308
7 -2.183637 -1.159914 0.559289
7 -1.587184 0.955238 -0.417533
8 -3.676677 0.564870 0.368344
6 1.496158 -1.269179 -0.228893
1 1.472705 -2.362170 -0.255561
7 0.529697 1.418163 -1.232073
1 0.240751 2.354936 -1.504005
1 1.485147 1.110230 -1.410354
8 2.104717 -0.921308 0.994452
1 2.196595 0.066618 1.079122

8 2.178262 -0.749933 -1.349611
1 3.067060 -1.139783 -1.374819
1 -1.904579 1.886017 -0.689125
8 2.447187 1.748530 1.354449
1 3.371406 1.982690 1.179640
1 2.306250 1.970242 2.287500
rc_5dhmC/rc_5dhmCacs.xyz
6 2.491920 0.045800 -0.137177
6 0.260989 0.554510 0.685575
6 -0.160180 -0.750736 0.213570
6 0.836077 -1.565447 -0.382342
1 0.545624 -2.553581 -0.729726
7 2.093209 -1.223624 -0.553149
7 1.557428 0.882265 0.477922
8 3.642508 0.441530 -0.293966
6 -1.599812 -1.218688 0.175133
1 -1.625280 -2.311979 0.173128
7 -0.548986 1.386372 1.297354
1 -0.229007 2.300544 1.608810
1 -1.515683 1.102688 1.456906
8 -2.160199 -0.809844 -1.052003
1 -2.113278 0.179783 -1.143205
8 -2.286517 -0.700280 1.294166
1 -3.196049 -1.039849 1.281108
1 1.906731 1.788561 0.790412
8 -2.052315 1.890371 -1.394368
1 -2.480914 2.102594 -2.237633
1 -1.125490 2.149286 -1.513874
rc_5dhmC/rc_5dhmCaaw.xyz
6 -1.653795 0.487598 0.065303
6 0.711244 1.029254 0.006379
6 0.982395 -0.384256 -0.167191
6 -0.137673 -1.253187 -0.178366
1 0.045487 -2.319286 -0.285611
7 -1.390842 -0.872699 -0.073195
7 -0.590303 1.387144 0.103439

8 -2.803100 0.913675 0.154354
6 2.373550 -0.983429 -0.206798
1 2.336243 -1.947188 -0.720676
7 1.657629 1.936807 0.073985
1 1.435051 2.920389 0.210819
1 2.627408 1.655094 -0.065304
8 2.785600 -1.278973 1.114174
1 2.867169 -0.453982 1.623595
8 3.236534 -0.085148 -0.861286
1 4.095230 -0.517751 -0.996064
1 -0.837931 2.371906 0.204392
8 -5.082326 -0.827977 -0.087904
1 -4.792328 -1.706450 -0.371693
1 -4.259695 -0.309985 0.000276
rc_5dhmC/rc_5dhmCacy.xyz
6 -1.652291 0.476919 0.056986
6 0.711076 1.027234 0.011240
6 0.987774 -0.384045 -0.171518
6 -0.129267 -1.257053 -0.190868
1 0.058100 -2.321836 -0.303679
7 -1.384157 -0.881811 -0.087107
7 -0.592100 1.379729 0.105596
8 -2.803216 0.899248 0.142150
6 2.381305 -0.977591 -0.210113
1 2.349689 -1.937700 -0.731128
7 1.653853 1.937718 0.089577
1 1.426908 2.919206 0.234382
1 2.625252 1.660603 -0.047516
8 2.789030 -1.281086 1.110429
1 2.863262 -0.459666 1.626720
8 3.243493 -0.071353 -0.854538
1 4.103938 -0.500124 -0.990402
1 -0.843456 2.363012 0.211697
8 -5.104767 -0.801920 -0.101117
1 -4.848031 -1.734914 -0.099178
1 -4.266734 -0.309930 -0.007112

rc_5dhmC/rc_5dhmCadr.xyz
6 -1.700986 -1.522409 -0.009444
6 -0.198629 0.387704 -0.126124
6 0.910036 -0.545844 -0.173602
6 0.586405 -1.925132 -0.138481
1 1.403104 -2.641936 -0.173385
7 -0.630778 -2.416797 -0.068359
7 -1.440548 -0.154144 -0.046363
8 -2.860240 -1.918079 0.070099
6 2.367956 -0.135730 -0.123659
1 2.983853 -0.938960 -0.536521
7 -0.054697 1.689144 -0.153528
1 -0.881883 2.316946 -0.072610
1 0.883761 2.071376 -0.255942
8 2.766495 -0.015894 1.229580
1 2.267416 0.704638 1.652387
8 2.533200 1.060457 -0.846496
1 3.481913 1.258441 -0.906138
1 -2.253521 0.460426 -0.016309
8 -2.371456 3.223033 0.097551
1 -3.071146 2.658635 0.461876
1 -2.264026 3.935350 0.746381
rc_5dhmC/rc_5dhmCaam.xyz
6 -1.605715 -0.908663 -0.006536
6 0.196388 0.716861 -0.007821
6 1.126606 -0.380616 -0.185179
6 0.570263 -1.682206 -0.260940
1 1.249190 -2.520570 -0.393698
7 -0.711281 -1.961805 -0.184293
7 -1.115780 0.394155 0.073184
8 -2.815097 -1.109944 0.076375
6 2.633862 -0.228665 -0.172599
1 3.087393 -1.081023 -0.684283
7 0.577637 1.970188 0.071060
1 -0.098743 2.718752 0.202801
1 1.564536 2.197596 -0.046608

8 3.091764 -0.306236 1.164682
1 2.749164 0.448644 1.675407
8 2.975483 0.985138 -0.796914
1 3.940443 1.023159 -0.897493
1 -1.809877 1.133569 0.189437
8 -4.498430 1.264666 0.074172
1 -5.417752 1.106481 -0.183151
1 -4.076572 0.386376 0.069860
rc_5dhmC/rc_5dhmCacu.xyz
6 -2.462443 -0.030545 -0.075985
6 -0.228730 0.363161 0.800463
6 0.275302 -0.549723 -0.209162
6 -0.678118 -1.121804 -1.087810
1 -0.319805 -1.802499 -1.855662
7 -1.971588 -0.890346 -1.056980
7 -1.566278 0.561278 0.817141
8 -3.659394 0.219688 0.018395
6 1.720926 -0.993642 -0.303906
1 1.918556 -1.377542 -1.307512
7 0.543054 0.978121 1.667452
1 0.154650 1.607425 2.365916
1 1.553555 0.867894 1.601487
8 1.935244 -2.085081 0.567643
1 1.785120 -1.810579 1.489521
8 2.548380 0.109168 -0.006994
1 3.470115 -0.123065 -0.206964
1 -1.972797 1.195209 1.505287
8 1.506942 2.547888 -1.250114
1 1.966251 1.757018 -0.914477
1 1.997032 2.804766 -2.043941
rc_5dhmU/rc_5dhmUadn.xyz
6 1.643926 1.027264 -0.030877
6 -0.810131 1.102268 0.031404
6 -0.817206 -0.372779 0.037820
6 0.418302 -1.051703 0.010424
1 0.475302 -2.136223 0.015151

7 1.555587 -0.388471 -0.019811
7 0.442337 1.691099 -0.011039
8 2.732511 1.571653 -0.055771
8 -1.842550 1.768731 0.053575
1 0.486256 2.710017 -0.014360
6 -2.130084 -1.124707 0.024998
1 2.489575 -0.918828 -0.019008
8 -2.926708 -0.780557 1.131762
1 -3.110898 0.175528 1.112895
8 -2.766315 -0.915729 -1.217674
1 -2.975034 0.029044 -1.327937
1 -1.941832 -2.196520 0.097095
8 3.843646 -1.739641 -0.029328
1 4.563769 -1.115129 0.153291
1 3.873880 -2.383986 0.695687
rc_5dhmU/rc_5dhmUada.xyz
6 1.641802 1.040488 -0.031999
6 -0.812814 1.104420 0.028887
6 -0.812551 -0.370558 0.048651
6 0.426012 -1.043823 0.020430
1 0.487853 -2.128035 0.029441
7 1.560158 -0.375536 -0.016568
7 0.436983 1.698767 -0.015182
8 2.727755 1.590063 -0.057430
8 -1.848363 1.766301 0.042443
1 0.476042 2.717860 -0.023890
6 -2.121460 -1.129460 0.037327
1 2.495072 -0.903961 -0.019247
8 -2.937121 -0.767681 1.123809
1 -3.124285 0.187148 1.082412
8 -2.739426 -0.946531 -1.219211
1 -2.952530 -0.005129 -1.348481
1 -1.929323 -2.198760 0.132551
8 3.836010 -1.748819 -0.032623
1 3.775160 -2.492793 0.587349
1 4.555253 -1.192018 0.306436

rc_5dhmU/rc_5dhmUacw.xyz
6 -1.638361 -1.057201 0.026091
6 0.817082 -1.104925 -0.021551
6 0.806465 0.369639 -0.057957
6 -0.436905 1.034311 -0.054104
1 -0.506145 2.117820 -0.079078
7 -1.566976 0.358933 -0.017632
7 -0.428607 -1.706812 0.025201
8 -2.719602 -1.615408 0.058475
8 1.857139 -1.759881 -0.025281
1 -0.460666 -2.725932 0.048843
6 2.110608 1.136424 -0.038642
1 -2.503250 0.885181 -0.011225
8 2.722077 0.960156 1.221824
1 2.938934 0.020091 1.354760
8 2.934895 0.775276 -1.119142
1 3.127304 -0.178358 -1.072777
1 1.913100 2.204345 -0.138307
8 -3.841825 1.729382 0.050295
1 -3.672026 2.671535 -0.107414
1 -4.442983 1.454789 -0.660174
rc_5dhmU/rc_5dhmUaci.xyz
6 -1.644163 -1.028530 0.030872
6 0.810003 -1.102446 -0.032676
6 0.816618 0.372589 -0.035257
6 -0.419177 1.050914 -0.005844
1 -0.476597 2.135414 -0.008351
7 -1.556247 0.387230 0.023297
7 -0.442278 -1.691837 0.008679
8 -2.732514 -1.573437 0.055310
8 1.842645 -1.768489 -0.056848
1 -0.485751 -2.710777 0.009614
6 2.129168 1.125053 -0.022795
1 -2.489908 0.918307 0.021287
8 2.768395 0.913145 1.217785
1 2.977651 -0.031831 1.325212

8 2.923321 0.784279 -1.132430
1 3.108385 -0.171700 -1.116507
1 1.940222 2.196962 -0.091596
8 -3.832481 1.759037 0.018704
1 -3.925339 2.248005 -0.814364
1 -4.568609 1.126102 0.024897
rc_5dhmU/rc_5dhmUact.xyz
6 -1.635488 -1.075985 0.014049
6 0.821003 -1.104746 -0.011694
6 0.799426 0.369398 -0.058242
6 -0.449103 1.024093 -0.075252
1 -0.526403 2.106795 -0.109357
7 -1.574596 0.340384 -0.046464
7 -0.420198 -1.715334 0.032313
8 -2.711702 -1.643529 0.044000
8 1.865881 -1.752010 -0.006695
1 -0.444408 -2.734436 0.064929
6 2.098469 1.144496 -0.036161
1 -2.511121 0.864667 -0.041205
8 2.713311 0.970954 1.222669
1 2.933990 0.031724 1.355358
8 2.922866 0.788412 -1.118696
1 3.122400 -0.163771 -1.071799
1 1.894221 2.211204 -0.135046
8 -3.854083 1.708849 0.004921
1 -3.981382 2.194135 -0.825554
1 -3.819759 2.389389 0.695923
rc_5dhmU/rc_5dhmUaan.xyz
6 1.637261 -1.065032 0.019304
6 -0.818741 -1.104726 -0.013752
6 -0.803389 0.369475 -0.061690
6 0.442296 1.029636 -0.074379
1 0.515148 2.112653 -0.108841
7 1.570467 0.350828 -0.040548
7 0.425045 -1.710087 0.032972
8 2.716367 -1.627200 0.051868

8 -1.860850 -1.756448 -0.009512
1 0.453714 -2.729046 0.066229
6 -2.105375 1.139741 -0.040114
1 2.507126 0.876613 -0.033034
8 -2.932086 0.777199 -1.118588
1 -3.127344 -0.175717 -1.068560
8 -2.715525 0.968131 1.221439
1 -2.933634 0.028749 1.357280
1 -1.905484 2.206892 -0.143183
8 3.845613 1.715983 0.050828
1 4.315717 1.707633 -0.797763
1 3.665699 2.651146 0.236420
rc_5dhmU/rc_5dhmUabq.xyz
6 1.642795 1.032749 -0.008910
6 -0.812600 1.101959 0.004454
6 -0.815651 -0.371832 0.067601
6 0.421917 -1.046043 0.105435
1 0.481720 -2.129227 0.155959
7 1.557969 -0.380527 0.076987
7 0.438859 1.692103 -0.039182
8 2.730009 1.579131 -0.047078
8 -1.846539 1.766231 -0.013326
1 0.479958 2.709988 -0.088367
6 -2.126169 -1.127292 0.039778
1 2.491229 -0.911981 0.080116
8 -2.956365 -0.749554 1.110516
1 -3.139841 0.205428 1.055662
8 -2.725389 -0.955812 -1.226905
1 -2.934335 -0.015143 -1.368091
1 -1.939502 -2.196088 0.150626
8 3.827551 -1.764760 0.073713
1 4.569964 -1.139455 0.089520
1 3.917115 -2.243685 -0.765570
rc_5dhmU/rc_5dhmUadi.xyz
6 1.714176 1.076617 0.013542
6 -0.040762 -0.621149 0.032131

6 -1.051373 0.456741 0.085930
6 -0.608350 1.794489 0.092914
1 -1.297932 2.632979 0.124504
7 0.682221 2.064077 0.063455
7 1.279291 -0.216981 0.008689
8 2.879073 1.427932 -0.020152
8 -0.354834 -1.810781 0.009680
1 2.022611 -0.959232 -0.029473
6 -2.530264 0.134025 0.072139
1 1.004035 3.035381 0.072959
8 -2.880185 -0.269661 -1.236592
1 -2.434685 -1.106703 -1.459552
8 -2.875329 -0.799367 1.062296
1 -2.359043 -1.614518 0.926309
1 -3.104349 1.037682 0.276924
8 3.224296 -2.159589 -0.091210
1 3.695668 -2.191718 0.755582
1 3.898372 -1.916161 -0.744388
rc_5dhmU/rc_5dhmUadc.xyz
6 -1.724878 1.084353 0.001651
6 0.036721 -0.606046 0.005125
6 1.043551 0.476540 0.036633
6 0.595720 1.812532 0.044978
1 1.282164 2.653890 0.064260
7 -0.696413 2.076628 0.030067
7 -1.284404 -0.207125 -0.005620
8 -2.891868 1.429845 -0.013175
8 0.356307 -1.794366 -0.008953
1 -2.022499 -0.955002 -0.029273
6 2.521350 0.149437 0.033243
1 -1.022132 3.046697 0.038089
8 2.861152 -0.645351 1.143174
1 2.369501 -1.485504 1.105062
8 2.868715 -0.427848 -1.205669
1 2.412814 -1.281710 -1.310970
1 3.100663 1.068521 0.118849

8 -3.212714 -2.164570 -0.115094
1 -4.021009 -1.869322 0.331966
1 -2.921289 -2.946680 0.378837
rc_5dhmU/rc_5dhmUadq.xyz
6 2.527885 0.252504 -0.103999
6 0.192341 0.694840 0.539681
6 -0.126926 -0.710342 0.222423
6 0.896603 -1.532963 -0.285113
1 0.714396 -2.565435 -0.567606
7 2.126533 -1.072548 -0.423696
7 1.519397 1.054026 0.367406
8 3.682804 0.600293 -0.252660
8 -0.638927 1.505218 0.944979
1 1.775804 2.015677 0.591454
6 -1.535227 -1.257770 0.296998
1 2.868574 -1.682928 -0.773903
8 -2.307222 -0.771962 1.357615
1 -2.243098 0.199827 1.384223
8 -2.060950 -0.967976 -0.986085
1 -2.250070 0.014830 -1.089639
1 -1.508076 -2.343610 0.391745
8 -2.550708 1.632163 -1.297194
1 -3.492891 1.829531 -1.183729
1 -2.114184 2.002250 -0.511709
rc_5dhmU/rc_5dhmUaag.xyz
6 1.711845 1.053328 0.020651
6 -0.055030 -0.632446 0.042216
6 -1.057570 0.453708 0.086655
6 -0.604720 1.788353 0.088944
1 -1.288358 2.631901 0.113138
7 0.687842 2.048810 0.064085
7 1.267068 -0.237069 0.022505
8 2.879969 1.393718 -0.013799
8 -0.377233 -1.820071 0.023666
1 2.010859 -0.977763 -0.021852
6 -2.538740 0.142352 0.065711

1 1.016923 3.017748 0.069302
8 -2.883765 -0.263227 -1.243776
1 -2.442130 -1.103583 -1.461806
8 -2.897131 -0.784642 1.057093
1 -2.384417 -1.603288 0.928887
1 -3.107122 1.051104 0.263560
8 3.317860 -2.059180 -0.174252
1 3.323528 -2.713950 0.541180
1 4.134042 -1.548896 -0.055068
rc_5dhmU/rc_5dhmUabk.xyz
6 1.716692 1.047744 -0.005400
6 -0.060683 -0.626684 -0.010821
6 -1.056448 0.466597 -0.035293
6 -0.595900 1.798254 -0.046518
1 -1.274346 2.646034 -0.064796
7 0.698686 2.050094 -0.035448
7 1.263976 -0.240030 -0.001068
8 2.887174 1.380913 0.013452
8 -0.393060 -1.811401 -0.000999
1 2.004136 -0.985390 0.022512
6 -2.537540 0.155102 -0.024401
1 1.034311 3.016815 -0.044676
8 -2.892264 -0.628853 -1.137479
1 -2.409587 -1.474539 -1.107658
8 -2.884445 -0.426084 1.212714
1 -2.435198 -1.284187 1.311764
1 -3.107180 1.081022 -0.100778
8 3.305809 -2.082356 0.108676
1 3.346196 -2.617748 -0.698666
1 4.120598 -1.556281 0.101585
rc_5dhmU/rc_5dhmUaat.xyz
6 -1.734392 1.113302 0.011081
6 0.010736 -0.594811 0.037762
6 1.028622 0.475945 0.086312
6 0.593650 1.816401 0.086049
1 1.288639 2.650655 0.112250

7 -0.695270 2.094063 0.056086
7 -1.306595 -0.182100 0.012657
8 -2.896965 1.471996 -0.023846
8 0.314729 -1.787334 0.020652
1 -2.041873 -0.931303 -0.032398
6 2.504986 0.142217 0.071736
1 -1.010217 3.067643 0.060151
8 2.844581 -0.786708 1.068049
1 2.323420 -1.599596 0.937617
8 2.848952 -0.273866 -1.234583
1 2.396060 -1.108888 -1.450158
1 3.086649 1.042775 0.268690
8 -3.111691 -2.250500 -0.169188
1 -3.835697 -2.176158 0.471495
1 -2.612398 -3.035900 0.104829
rc_5dhmU/rc_5dhmUaca.xyz
6 -1.716883 1.045477 0.006630
6 0.061588 -0.627802 0.021948
6 1.056600 0.466528 0.031774
6 0.595194 1.797928 0.027014
1 1.273163 2.646276 0.032222
7 -0.699528 2.048833 0.017086
7 -1.263247 -0.241917 0.014253
8 -2.887789 1.377427 -0.007830
8 0.394650 -1.812410 0.021099
1 -2.003957 -0.987374 0.012270
6 2.538007 0.156690 0.021179
1 -1.035748 3.015390 0.015147
8 2.896762 -0.619854 1.137953
1 2.415548 -1.466591 1.114451
8 2.882260 -0.431881 -1.213399
1 2.432897 -1.290631 -1.306029
1 3.106622 1.083814 0.090131
8 -3.325821 -2.059432 0.060980
1 -4.105152 -1.552789 -0.216693
1 -3.231482 -2.760229 -0.602563

rc_5dhmU/rc_5dhmUaam.xyz
6 2.524375 0.245786 -0.097474
6 0.189606 0.685702 0.550752
6 -0.133136 -0.713775 0.214887
6 0.887942 -1.531954 -0.304702
1 0.702879 -2.559790 -0.601852
7 2.119153 -1.072898 -0.437433
7 1.517770 1.043647 0.384397
8 3.680604 0.592185 -0.239458
8 -0.640305 1.493336 0.964887
1 1.777341 2.000685 0.624126
6 -1.542427 -1.259100 0.283042
1 2.859667 -1.680464 -0.795651
8 -2.311805 -0.789691 1.352550
1 -2.244451 0.181257 1.397152
8 -2.068672 -0.945807 -0.994708
1 -2.267414 0.037148 -1.077745
1 -1.518127 -2.346511 0.358150
8 -2.651616 1.647140 -1.196618
1 -2.297426 2.023468 -2.016974
1 -2.094736 2.011696 -0.488207
rc_5dhmU/rc_5dhmUabo.xyz
6 -2.867060 -0.062149 -0.168648
6 -0.651735 0.979380 0.051414
6 -0.050334 -0.369707 0.059926
6 -0.902859 -1.490256 -0.020712
1 -0.524109 -2.507611 -0.005067
7 -2.206871 -1.326137 -0.130741
7 -2.030041 1.018574 -0.079784
8 -4.075388 -0.007849 -0.273490
8 0.017511 2.003285 0.151625
1 -2.473938 1.937181 -0.092497
6 1.446395 -0.540338 0.209605
1 -2.822833 -2.140766 -0.201628
8 2.160275 0.225849 -0.726672
1 1.946214 1.169817 -0.612313

8 1.783438 -0.258185 1.551122
1 1.618890 0.681567 1.748860
1 1.719449 -1.580867 0.035435
8 4.974848 -0.317270 -0.531309
1 5.435642 0.416735 -0.961785
1 4.027155 -0.091319 -0.597051
rc_5dhmU/rc_5dhmUads.xyz
6 2.340751 0.163153 0.135570
6 0.078460 0.982893 -0.383214
6 -0.472960 -0.154463 0.374362
6 0.423639 -1.047849 0.993449
1 0.076309 -1.894408 1.577206
7 1.727543 -0.883296 0.874126
7 1.461473 1.038437 -0.450349
8 3.551908 0.237538 0.057106
8 -0.627552 1.826637 -0.930870
1 1.875006 1.808016 -0.976244
6 -1.970952 -0.294125 0.576858
1 2.371324 -1.544842 1.312481
8 -2.388139 0.658474 1.532809
1 -2.267453 1.557120 1.179821
8 -2.682154 -0.221112 -0.634607
1 -2.474431 0.619490 -1.081264
1 -2.188493 -1.272854 1.005424
8 -0.464501 -1.913435 -1.561340
1 -1.348920 -1.533748 -1.709590
1 0.123436 -1.427227 -2.161208
rc_5dhmU/rc_5dhmUabr.xyz
6 2.324790 -0.178426 0.151488
6 0.059139 -0.994430 -0.358648
6 -0.486229 0.164500 0.370370
6 0.413764 1.057101 0.985450
1 0.069684 1.914778 1.554681
7 1.716751 0.879787 0.876701
7 1.441763 -1.060976 -0.418321
8 3.535588 -0.258167 0.071309

8 -0.651026 -1.844694 -0.890907
1 1.852405 -1.841351 -0.930346
6 -1.983760 0.328189 0.546801
1 2.363480 1.542976 1.307950
8 -2.679425 0.233101 -0.671130
1 -2.466152 -0.615310 -1.099355
8 -2.422476 -0.596879 1.521622
1 -2.293749 -1.505821 1.198935
1 -2.196114 1.318837 0.950009
8 -0.345049 1.877837 -1.582449
1 0.303317 1.396720 -2.121415
1 -1.209595 1.506302 -1.829444
rc_5dhmU/rc_5dhmUabx.xyz
6 2.862846 -0.091766 0.117670
6 0.659864 0.986816 -0.040764
6 0.036361 -0.352474 -0.035564
6 0.871775 -1.486873 0.016491
1 0.475705 -2.497697 0.006088
7 2.180651 -1.344601 0.092286
7 2.041739 1.002888 0.053119
8 4.074044 -0.058463 0.192822
8 0.004557 2.021822 -0.116168
1 2.500681 1.914244 0.059593
6 -1.466456 -0.499675 -0.130811
1 2.784706 -2.169763 0.141028
8 -2.128822 0.257926 0.850807
1 -1.906431 1.201072 0.747326
8 -1.858831 -0.191415 -1.450788
1 -1.674786 0.744373 -1.648710
1 -1.746727 -1.539334 0.037553
8 -4.939786 -0.280081 0.505207
1 -4.002197 -0.049052 0.646492
1 -4.983319 -0.546340 -0.424384
rc_5dhmU/rc_5dhmUaab.xyz
6 2.860037 -0.088777 0.181820
6 0.660751 0.986377 -0.036615

6 0.041762 -0.353599 -0.073868
6 0.877940 -1.487015 -0.003677
1 0.484742 -2.498489 -0.037695
7 2.183071 -1.342955 0.120109
7 2.038453 1.004802 0.104064
8 4.068228 -0.052883 0.295446
8 0.005735 2.020779 -0.123612
1 2.494486 1.917060 0.134908
6 -1.455467 -0.502462 -0.244812
1 2.787202 -2.166928 0.184716
8 -2.170614 0.282255 0.673939
1 -1.937336 1.221462 0.556364
8 -1.768263 -0.223957 -1.593417
1 -1.588619 0.712496 -1.794233
1 -1.747369 -1.537522 -0.068020
8 -4.959410 -0.318089 0.747645
1 -4.017485 -0.079373 0.649989
1 -5.341834 -0.193604 -0.132348
rc_5fC/rc_5fCaba.xyz
6 1.283268 1.212231 -0.006632
6 -0.302897 -0.602942 -0.015312
6 -1.369345 0.390031 0.005314
6 -0.982214 1.754407 0.013843
1 -1.761906 2.512306 0.025381
7 0.262520 2.175834 0.009167
7 0.960265 -0.140617 -0.025117
8 2.461084 1.554840 -0.005366
7 -0.549356 -1.891296 -0.022617
1 0.209124 -2.569701 -0.033765
1 -1.517399 -2.210129 -0.016669
6 -2.802436 0.048460 0.013711
1 -3.492682 0.904814 0.027902
8 -3.212888 -1.109304 0.004556
1 1.766880 -0.819051 -0.043008
8 3.049473 -1.898099 -0.071194
1 3.836336 -1.420853 0.235179

1 2.906043 -2.597437 0.585440
rc_5fC/rc_5fCaca.xyz
6 1.277500 1.264990 0.000926
6 -0.270447 -0.583902 -0.003912
6 -1.358202 0.385366 0.001905
6 -1.000175 1.757336 0.009381
1 -1.795693 2.498646 0.015361
7 0.235305 2.205135 0.010052
7 0.983465 -0.094644 -0.008316
8 2.447038 1.634270 0.000376
7 -0.490329 -1.876937 -0.003861
1 0.282355 -2.539624 -0.005389
1 -1.452139 -2.214215 -0.003866
6 -2.783851 0.013784 -0.000814
1 -3.491695 0.855805 0.002074
8 -3.170024 -1.152347 -0.006568
1 1.794480 -0.764062 -0.018763
8 2.930519 -2.020284 -0.077030
1 2.883840 -2.529835 0.746937
1 3.830555 -1.660145 -0.100628
rc_5fC/rc_5fCaah.xyz
6 -1.278708 -1.263180 -0.001068
6 0.271096 0.583932 -0.003143
6 1.357740 -0.386728 0.002504
6 0.998279 -1.758285 0.007126
1 1.792965 -2.500495 0.011854
7 -0.237722 -2.204634 0.006647
7 -0.983299 0.096305 -0.008699
8 -2.448694 -1.631022 -0.001502
7 0.493764 1.876506 -0.001925
1 -0.276091 2.542266 -0.002656
1 1.456564 2.211134 -0.000553
6 2.783858 -0.016812 0.001813
1 3.490695 -0.859666 0.004222
8 3.171530 1.148844 -0.001828
1 -1.794810 0.765278 -0.019778

8 -2.962511 1.989771 -0.092595
1 -3.788901 1.701020 0.324863
1 -2.665806 2.748926 0.433901
rc_5fC/rc_5fCadh.xyz
6 1.277871 1.255887 -0.002198
6 -0.276077 -0.587490 -0.009802
6 -1.360398 0.385863 -0.000590
6 -0.997799 1.756576 0.001772
1 -1.790718 2.500679 0.005673
7 0.239227 2.199993 -0.001664
7 0.979449 -0.103086 -0.005151
8 2.448676 1.621146 -0.000535
7 -0.502032 -1.879398 -0.022632
1 0.266184 -2.546866 -0.035099
1 -1.465584 -2.211803 -0.021954
6 -2.787395 0.019479 0.008446
1 -3.492017 0.864128 0.019414
8 -3.178143 -1.145148 0.004666
1 1.791564 -0.772080 0.007515
8 3.006673 -1.940867 0.108011
1 2.640069 -2.819888 -0.076002
1 3.679125 -1.799680 -0.576331
rc_5fC/rc_5fCaab.xyz
6 1.273564 1.273357 -0.001671
6 -0.266194 -0.582783 -0.005617
6 -1.358324 0.381419 0.000946
6 -1.006652 1.754982 0.004337
1 -1.805536 2.492684 0.008377
7 0.226744 2.208402 0.002909
7 0.985931 -0.087744 -0.007555
8 2.441082 1.648775 -0.000956
7 -0.480015 -1.876693 -0.009455
1 0.297247 -2.534128 -0.014704
1 -1.440178 -2.218518 -0.008345
6 -2.782299 0.003467 0.004205
1 -3.493784 0.842412 0.009625

8 -3.163447 -1.164316 0.000968
1 1.798438 -0.754574 -0.010080
8 2.896737 -2.047074 -0.005812
1 3.579042 -1.943477 -0.687114
1 3.380602 -2.073882 0.834151
rc_5fC/rc_5fCabw.xyz
6 -1.278045 1.257059 -0.002412
6 0.274887 -0.587167 0.001701
6 1.359777 0.385635 0.001804
6 0.997978 1.756512 0.000351
1 1.791270 2.500226 0.000341
7 -0.238821 2.200546 -0.000223
7 -0.980481 -0.102208 -0.004973
8 -2.448519 1.623288 -0.001945
7 0.500693 -1.879165 0.008096
1 -0.267575 -2.546743 0.011540
1 1.464323 -2.211413 0.010429
6 2.786630 0.018590 0.001352
1 3.491699 0.862936 -0.000975
8 3.176834 -1.146233 0.002918
1 -1.792991 -0.771012 -0.014980
8 -3.019109 -1.929839 -0.083764
1 -2.635913 -2.820282 -0.114149
1 -3.541562 -1.909420 0.733050
rc_5fC/rc_5fCact.xyz
6 -1.401405 -0.086967 -0.818033
6 0.762737 0.819705 -0.198885
6 1.219303 -0.550067 -0.006262
6 0.283510 -1.589178 -0.247818
1 0.610258 -2.616608 -0.107569
7 -0.958772 -1.401998 -0.632154
7 -0.521345 0.972336 -0.586332
8 -2.546845 0.148063 -1.179817
7 1.543984 1.852509 -0.009577
1 1.204081 2.802767 -0.144892
1 2.508893 1.695835 0.282595

6 2.585560 -0.896560 0.428641
1 2.783194 -1.971123 0.553339
8 3.450738 -0.051671 0.640893
1 -0.898874 1.909410 -0.731283
8 -3.361254 0.174511 1.858740
1 -4.118722 -0.211734 2.321549
1 -3.575244 0.082699 0.918313
rc_5fC/rc_5fCacz.xyz
6 -2.117890 -0.009874 -0.088885
6 0.108406 0.966963 -0.066625
6 0.617143 -0.322329 -0.505295
6 -0.336485 -1.355979 -0.728462
1 0.029419 -2.320051 -1.073427
7 -1.627169 -1.236742 -0.544891
7 -1.226094 1.049596 0.116724
8 -3.309994 0.159218 0.131346
7 0.888650 1.997268 0.146350
1 0.509535 2.886661 0.464040
1 1.891373 1.896992 -0.009856
6 2.047888 -0.590063 -0.747203
1 2.287481 -1.626047 -1.028312
8 2.916652 0.270404 -0.648052
1 -1.640193 1.925018 0.439305
8 1.283156 -1.148217 1.956717
1 0.412927 -1.546685 2.114251
1 1.228877 -0.270293 2.365452
rc_5fC/rc_5fCaam.xyz
6 -1.233759 -0.897975 0.007378
6 0.565610 0.727812 0.009339
6 1.500113 -0.388732 -0.001415
6 0.951244 -1.697280 -0.002517
1 1.634362 -2.543012 -0.007822
7 -0.334161 -1.968144 0.002601
7 -0.747537 0.409798 0.012410
8 -2.442784 -1.099072 0.006968
7 0.962048 1.974212 0.016306

1 0.291346 2.740055 0.024299
1 1.961376 2.179146 0.012177
6 2.966523 -0.226501 -0.010434
1 3.543177 -1.162753 -0.019298
8 3.516614 0.870571 -0.008488
1 -1.449252 1.152501 0.022217
8 -4.205787 1.231288 -0.104008
1 -4.869007 1.203780 0.600551
1 -3.717174 0.392985 -0.021232
rc_5fC/rc_5fCabq.xyz
6 -2.086122 0.006905 -0.134662
6 0.155787 0.946773 -0.110232
6 0.649905 -0.370384 -0.479947
6 -0.318386 -1.393928 -0.687256
1 0.036212 -2.375951 -0.991059
7 -1.609960 -1.244682 -0.535824
7 -1.180036 1.059160 0.042791
8 -3.278613 0.204852 0.057978
7 0.946855 1.975430 0.059318
1 0.576473 2.886480 0.320264
1 1.951433 1.850368 -0.060041
6 2.076867 -0.672310 -0.689543
1 2.301035 -1.717796 -0.948901
8 2.963484 0.169718 -0.583481
1 -1.584570 1.954190 0.319513
8 0.982599 -0.817610 2.117762
1 0.037039 -0.986428 2.254190
1 1.376301 -1.698242 2.013799
rc_5fC/rc_5fCaad.xyz
6 1.266912 0.415135 -0.000542
6 -1.120564 0.837053 -0.001989
6 -1.319159 -0.606569 -0.000454
6 -0.158746 -1.423534 -0.001806
1 -0.289715 -2.502887 -0.002329
7 1.074134 -0.969959 -0.002192
7 0.160273 1.264026 -0.001139

8 2.392499 0.899221 0.001354
7 -2.118761 1.682428 -0.004351
1 -1.956870 2.687822 -0.005456
1 -3.073529 1.322788 -0.003412
6 -2.648551 -1.246658 0.003768
1 -2.642182 -2.346128 0.004366
8 -3.695426 -0.605856 0.006444
1 0.363980 2.264514 -0.001015
8 4.825541 -0.661346 -0.021976
1 3.984678 -0.167717 -0.002879
1 5.503855 -0.002565 0.184063
rc_5fC/rc_5fCaau.xyz
6 -2.421746 -0.431644 0.009142
6 0.011389 -0.551556 -0.067844
6 0.025863 0.902131 0.025301
6 -1.227071 1.556368 0.171907
1 -1.227218 2.637571 0.285186
7 -2.396080 0.959180 0.165283
7 -1.216400 -1.124814 -0.103356
8 -3.480441 -1.045433 -0.021733
7 1.086384 -1.294806 -0.108393
1 0.985635 -2.307485 -0.160857
1 2.053178 -0.932892 -0.044519
6 1.240604 1.733318 -0.061718
1 1.079350 2.810256 0.100132
8 2.350471 1.277867 -0.309153
1 -1.293017 -2.140209 -0.175372
8 3.881386 -1.141335 0.156170
1 4.120967 -1.597231 0.976924
1 4.178224 -0.227439 0.280776
rc_5fC/rc_5fCabk.xyz
6 2.422105 -0.445128 0.000274
6 -0.012462 -0.546533 -0.038098
6 -0.013917 0.909728 0.016020
6 1.246888 1.557528 0.116826
1 1.258766 2.641926 0.191977

7 2.410581 0.949665 0.114716
7 1.209803 -1.131155 -0.072300
8 3.475145 -1.068763 -0.029394
7 -1.093515 -1.281734 -0.048739
1 -1.001775 -2.296311 -0.073778
1 -2.056709 -0.909664 0.000909
6 -1.224431 1.748735 -0.048807
1 -1.044785 2.830574 0.047530
8 -2.350482 1.294005 -0.208373
1 1.277250 -2.148657 -0.118484
8 -3.886817 -1.112783 0.251496
1 -4.293819 -1.826544 -0.261700
1 -4.238880 -0.294403 -0.129311
rc_5fC/rc_5fCacc.xyz
6 -1.233614 -0.904272 -0.028971
6 0.562149 0.725842 -0.024884
6 1.499241 -0.388180 0.010381
6 0.953315 -1.697842 0.020917
1 1.638207 -2.541794 0.046062
7 -0.331289 -1.971919 0.001555
7 -0.750178 0.404747 -0.036331
8 -2.441816 -1.108133 -0.048519
7 0.956029 1.972949 -0.045510
1 0.283896 2.737115 -0.071982
1 1.954882 2.179960 -0.032237
6 2.965056 -0.221986 0.036140
1 3.543898 -1.156434 0.066333
8 3.512408 0.876431 0.025499
1 -1.452828 1.145753 -0.064961
8 -4.163914 1.257905 0.107604
1 -3.735868 0.388483 0.015468
1 -5.084425 1.115480 -0.154860
rc_5fC/rc_5fCabs.xyz
6 -2.089008 0.009474 -0.131375
6 0.146636 0.963609 -0.076078
6 0.649023 -0.334562 -0.497354

6 -0.312147 -1.358995 -0.731980
1 0.049517 -2.328712 -1.065556
7 -1.604222 -1.225198 -0.572118
7 -1.190415 1.062999 0.075950
8 -3.282295 0.191717 0.071246
7 0.932629 1.986732 0.145492
1 0.556727 2.882064 0.449781
1 1.937829 1.873488 0.018918
6 2.078798 -0.622137 -0.710922
1 2.309832 -1.659271 -0.996287
8 2.959357 0.222696 -0.581133
1 -1.600963 1.943517 0.388813
8 1.027886 -0.982628 2.065264
1 1.227501 -1.909368 1.858112
1 0.074210 -0.972063 2.244180
rc_5fC/rc_5fCaao.xyz
6 1.269400 0.450302 0.009358
6 -1.123861 0.835646 -0.011660
6 -1.301139 -0.610727 -0.002932
6 -0.128335 -1.410338 -0.004131
1 -0.242491 -2.491668 -0.007313
7 1.097116 -0.937514 -0.000124
7 0.150342 1.282329 -0.000862
8 2.387767 0.950500 0.026018
7 -2.135137 1.664969 -0.029836
1 -1.990534 2.672917 -0.036151
1 -3.083697 1.289190 -0.029818
6 -2.621127 -1.270037 0.012849
1 -2.599052 -2.369256 0.019700
8 -3.677619 -0.645203 0.018078
1 0.337261 2.286216 0.001003
8 4.846748 -0.562103 0.059465
1 4.785334 -1.258470 -0.609762
1 3.982135 -0.112045 0.028698
rc_5fC/rc_5fCadq.xyz
6 -1.184453 -1.134832 -0.000011

6 1.219706 -0.792337 0.000007
6 0.963942 0.643665 -0.000007
6 -0.391735 1.064894 -0.000025
1 -0.606990 2.131063 -0.000038
7 -1.418373 0.247209 -0.000024
7 0.134141 -1.595575 0.000007
8 -2.112508 -1.929512 -0.000015
7 2.430823 -1.287306 0.000020
1 2.589787 -2.293413 0.000029
1 3.225991 -0.648132 0.000018
6 2.035076 1.658564 -0.000006
1 1.696485 2.704238 -0.000018
8 3.227181 1.365557 0.000006
1 0.255089 -2.609369 0.000013
8 -4.102318 1.450929 0.000032
1 -3.220381 1.028291 0.000008
1 -3.920180 2.401509 0.000030
rc_5fC/rc_5fCadf.xyz
6 -2.105488 0.002698 -0.108625
6 0.136477 -0.937120 -0.116572
6 0.628420 0.386058 -0.467826
6 -0.339325 1.418929 -0.625990
1 0.014812 2.409766 -0.900356
7 -1.629117 1.268015 -0.464842
7 -1.197740 -1.051343 0.047537
8 -3.299229 -0.204116 0.065838
7 0.927809 -1.970062 0.024897
1 0.558723 -2.885784 0.270832
1 1.930692 -1.845146 -0.107666
6 2.048811 0.680958 -0.725867
1 2.270831 1.725607 -0.989549
8 2.933861 -0.167016 -0.654044
1 -1.601503 -1.953916 0.299280
8 0.979420 0.790845 2.117282
1 1.124780 1.745137 2.020793
1 1.869216 0.411223 2.190193

rc_5fC/rc_5fCaak.xyz
6 2.113138 -0.007790 -0.093563
6 -0.117943 0.958666 -0.090768
6 -0.620406 -0.342993 -0.499939
6 0.338388 -1.375957 -0.702389
1 -0.023280 -2.348926 -1.026209
7 1.628609 -1.246063 -0.524817
7 1.216381 1.051741 0.088887
8 3.304119 0.171519 0.124458
7 -0.901562 1.991258 0.095899
1 -0.525408 2.890250 0.388968
1 -1.904868 1.881032 -0.048444
6 -2.049206 -0.624596 -0.733275
1 -2.281949 -1.664100 -1.007103
8 -2.925470 0.228275 -0.631917
1 1.626495 1.935249 0.393680
8 -1.201240 -1.107772 1.999121
1 -0.271394 -0.989062 2.249754
1 -1.626708 -0.263153 2.215875
rc_5fC/rc_5fCabn.xyz
6 -2.081543 -0.006897 -0.137170
6 0.167860 -0.926193 -0.157210
6 0.649747 0.411088 -0.467478
6 -0.327839 1.432447 -0.635349
1 0.016410 2.429014 -0.901146
7 -1.618164 1.264699 -0.489563
7 -1.165506 -1.055858 0.000722
8 -3.272452 -0.223349 0.044929
7 0.966702 -1.956858 -0.051037
1 0.605407 -2.883593 0.163066
1 1.969561 -1.817877 -0.168325
6 2.073156 0.734415 -0.664890
1 2.287087 1.788247 -0.897380
8 2.969352 -0.100150 -0.576387
1 -1.561827 -1.967484 0.230787
8 0.876856 0.623879 2.186163

1 0.202155 1.316477 2.262486
1 1.721641 1.099141 2.224600
rc_5fU/rc_5fUabg.xyz
6 1.341799 1.152505 -0.002661
6 -0.361719 -0.573133 -0.001393
6 -1.375311 0.459664 0.003637
6 -0.996616 1.788976 0.004204
1 -1.710493 2.606544 0.006479
7 0.300309 2.099664 0.001697
7 0.937985 -0.170896 -0.003573
8 2.512567 1.491434 -0.004987
8 -0.683952 -1.785230 -0.003650
1 1.685695 -0.910845 -0.014449
6 -2.698153 -0.044813 0.004776
1 -3.566529 0.632369 0.007094
8 -2.907941 -1.273713 0.002779
1 0.590076 3.076568 0.001900
8 2.736336 -2.231352 -0.089089
1 2.214842 -3.026181 0.105406
1 3.402287 -2.188131 0.614906
rc_5fU/rc_5fUacf.xyz
6 -1.320337 1.104117 -0.000885
6 0.401700 -0.602433 -0.006476
6 1.403040 0.442851 0.003319
6 1.009498 1.767770 0.010699
1 1.714040 2.593325 0.017881
7 -0.290883 2.063761 0.008503
7 -0.902216 -0.215197 -0.007012
8 -2.495346 1.428438 -0.003731
8 0.740079 -1.810093 -0.013498
1 -1.656947 -0.949600 -0.012618
6 2.731795 -0.045508 0.002872
1 3.591748 0.642442 0.009021
8 2.956796 -1.271743 -0.004949
1 -0.592146 3.037161 0.013754
8 -2.866227 -2.116644 -0.051784

1 -3.681151 -1.685440 -0.353226
1 -3.060454 -2.418277 0.849273
rc_5fU/rc_5fUaab.xyz
6 -1.166822 1.049978 0.000011
6 1.255089 0.741286 0.005456
6 1.032519 -0.687049 -0.003126
6 -0.262533 -1.181397 -0.010919
1 -0.481466 -2.244482 -0.018389
7 -1.296175 -0.344656 -0.008869
7 0.140052 1.527991 0.007600
8 -2.134966 1.790083 0.000523
8 2.410967 1.218636 0.010396
1 0.254352 2.540838 0.012806
6 2.226847 -1.441505 -0.006180
1 2.209223 -2.542562 -0.013227
8 3.339746 -0.875338 -0.000947
1 -2.280968 -0.720713 -0.011986
8 -3.814898 -1.373769 -0.056309
1 -4.421879 -0.672585 -0.340349
1 -4.103793 -1.608610 0.839272
rc_5fU/rc_5fUaaw.xyz
6 -1.320331 1.105028 0.000839
6 0.401116 -0.602113 0.006460
6 1.402818 0.442823 -0.003284
6 1.009734 1.767877 -0.010614
1 1.714560 2.593191 -0.017742
7 -0.290546 2.064315 -0.008442
7 -0.902667 -0.214426 0.006982
8 -2.495232 1.429742 0.003592
8 0.739072 -1.809891 0.013448
1 -1.657545 -0.948660 0.012570
6 2.731402 -0.046000 -0.002846
1 3.591598 0.641642 -0.008963
8 2.955966 -1.272316 0.004925
1 -0.591469 3.037820 -0.013676
8 -2.864184 -2.118523 0.051836

1 -3.680548 -1.690191 0.353472
1 -3.057527 -2.420820 -0.849190
rc_5fU/rc_5fUadb.xyz
6 1.150995 1.095498 -0.002300
6 -1.264059 0.733470 -0.001198
6 -1.010480 -0.689753 0.004070
6 0.295417 -1.154876 0.005033
1 0.537662 -2.212904 0.007840
7 1.311025 -0.296247 0.002027
7 -0.166365 1.544008 -0.003694
8 2.101897 1.857317 -0.004144
8 -2.429931 1.186130 -0.003221
1 -0.303419 2.554079 -0.007619
6 -2.188531 -1.469912 0.006313
1 -2.147280 -2.570307 0.010493
8 -3.313211 -0.927591 0.003789
1 2.300863 -0.657028 -0.003266
8 3.840995 -1.298272 -0.070435
1 3.809684 -2.248740 -0.260282
1 4.291827 -1.226662 0.785089
rc_5fU/rc_5fUabq.xyz
6 -1.166271 1.051954 -0.003312
6 1.255342 0.741135 0.007198
6 1.031546 -0.686959 -0.005047
6 -0.263952 -1.180076 -0.017909
1 -0.483773 -2.242968 -0.028293
7 -1.296834 -0.342488 -0.016883
7 0.140988 1.528795 0.008588
8 -2.133804 1.792899 -0.002441
8 2.411629 1.217475 0.015976
1 0.256172 2.541526 0.016385
6 2.225195 -1.442476 -0.006719
1 2.206611 -2.543497 -0.015980
8 3.338592 -0.877315 0.002540
1 -2.282114 -0.717639 -0.021175
8 -3.819233 -1.358685 -0.065099

1 -4.450326 -0.623476 -0.112277
1 -4.024286 -1.814558 0.766330
rc_5fU/rc_5fUaai.xyz
6 1.342501 1.151196 0.008241
6 -0.361992 -0.573434 0.010334
6 -1.374933 0.459930 -0.005887
6 -0.995438 1.789006 -0.011144
1 -1.708787 2.606973 -0.021216
7 0.301626 2.098964 -0.003063
7 0.937960 -0.172031 0.015113
8 2.513415 1.489566 0.011322
8 -0.685090 -1.785298 0.018682
1 1.685722 -0.912314 0.023511
6 -2.698142 -0.043587 -0.013346
1 -3.565847 0.634370 -0.025219
8 -2.908995 -1.272262 -0.006281
1 0.592017 3.075672 -0.007330
8 2.751963 -2.219659 0.064463
1 3.259525 -2.261186 -0.760704
1 2.217934 -3.029493 0.071934
rc_5fU/rc_5fUaay.xyz
6 -1.321783 1.106245 0.003662
6 0.399960 -0.600554 0.011337
6 1.401523 0.444386 -0.004799
6 1.008155 1.769375 -0.014656
1 1.712839 2.594763 -0.025845
7 -0.292179 2.065692 -0.009780
7 -0.903728 -0.212946 0.013852
8 -2.496859 1.430425 0.006340
8 0.737716 -1.808402 0.021471
1 -1.658104 -0.947712 0.021982
6 2.730139 -0.044395 -0.007647
1 3.590299 0.643234 -0.018660
8 2.954804 -1.270669 0.002727
1 -0.593203 3.039155 -0.017205
8 -2.869669 -2.111297 0.065011

1 -2.874672 -2.621253 -0.759804
1 -3.721714 -1.648206 0.079265
rc_5fU/rc_5fUacv.xyz
6 1.146790 1.108459 -0.003681
6 -1.266072 0.730758 0.001230
6 -1.003540 -0.690890 -0.001230
6 0.305417 -1.147396 -0.005170
1 0.554690 -2.203784 -0.007020
7 1.315708 -0.282397 -0.006608
7 -0.173525 1.548188 -0.000237
8 2.092329 1.876770 -0.004051
8 -2.434731 1.176221 0.004654
1 -0.317280 2.557352 0.001620
6 -2.176843 -1.478395 0.000380
1 -2.128744 -2.578507 -0.001212
8 -3.304794 -0.942988 0.003743
1 2.306803 -0.639541 -0.008091
8 3.847798 -1.286871 -0.007526
1 4.014302 -1.746912 0.829947
1 3.935632 -1.969421 -0.691067
rc_5fU/rc_5fUaae.xyz
6 1.210301 -0.537696 0.008035
6 -1.216928 -0.775951 -0.001163
6 -1.323028 0.667962 -0.000167
6 -0.179336 1.443211 0.004068
1 -0.201471 2.528078 0.004943
7 1.017134 0.850713 0.007660
7 0.048463 -1.292380 0.003915
8 2.320690 -1.040382 0.012086
8 -2.234962 -1.498664 -0.005620
1 0.166161 -2.305480 0.003427
6 -2.659568 1.137627 -0.003791
1 -2.886640 2.215739 -0.002738
8 -3.614968 0.337985 -0.008161
1 1.867526 1.414336 0.010281
8 4.685129 0.639398 0.012372

1 3.919510 0.036293 0.007600
1 5.439985 0.085089 -0.231838
rc_5fU/rc_5fUace.xyz
6 -1.212404 -0.578958 -0.012135
6 1.218444 -0.773657 0.003666
6 1.298297 0.671944 -0.000050
6 0.141212 1.426830 -0.009091
1 0.143069 2.511973 -0.012677
7 -1.044070 0.812558 -0.014480
7 -0.037389 -1.313115 -0.004161
8 -2.313953 -1.101100 -0.017522
8 2.249539 -1.477479 0.011926
1 -0.136985 -2.328222 -0.001784
6 2.625725 1.166079 0.004649
1 2.833163 2.248148 0.000976
8 3.595965 0.384358 0.012768
1 -1.905431 1.359964 -0.020621
8 -4.628717 0.653268 -0.070698
1 -5.105714 0.566919 0.767041
1 -3.908201 -0.000686 -0.016483
rc_5fU/rc_5fUadk.xyz
6 2.199325 -0.557544 -0.041300
6 -0.165257 -0.318134 0.517323
6 -0.119851 1.028378 -0.009762
6 1.065129 1.529634 -0.516640
1 1.155378 2.534553 -0.915966
7 2.156060 0.762773 -0.519504
7 0.994539 -1.033949 0.461488
8 3.215360 -1.224423 -0.069325
8 -1.219930 -0.789170 0.996547
1 0.999170 -1.990411 0.815769
6 -1.355913 1.712617 0.080504
1 -1.467046 2.747002 -0.281898
8 -2.355766 1.158778 0.577742
1 3.032705 1.126855 -0.892364
8 -3.258058 -1.570938 -1.167321

1 -3.869721 -2.206951 -0.769482
1 -2.698129 -1.276491 -0.431845
rc_5fU/rc_5fUabf.xyz
6 -2.134132 0.289732 -0.039896
6 0.219707 0.293666 -0.688542
6 0.384398 -0.953021 0.027607
6 -0.696774 -1.526206 0.671002
1 -0.627764 -2.457649 1.223031
7 -1.885869 -0.923210 0.623366
7 -1.029639 0.843362 -0.675046
8 -3.234045 0.807989 -0.051456
8 1.179791 0.830058 -1.281177
1 -1.185581 1.721388 -1.169540
6 1.707278 -1.454865 -0.014416
1 1.981660 -2.393251 0.493304
8 2.600239 -0.839333 -0.629583
1 -2.689194 -1.337346 1.095343
8 2.214025 1.893244 1.576528
1 1.418531 2.286789 1.189284
1 2.547958 1.307503 0.881318
rc_5fU/rc_5fUado.xyz
6 -1.215003 0.592241 0.011013
6 1.217052 0.774191 -0.003532
6 1.289471 -0.671838 0.003298
6 0.128667 -1.420701 0.015142
1 0.124616 -2.505827 0.021938
7 -1.053362 -0.800156 0.019608
7 -0.035929 1.320257 0.002658
8 -2.313837 1.119597 0.011952
8 2.251873 1.472490 -0.013131
1 -0.130273 2.335871 -0.003076
6 2.614286 -1.172994 -0.001738
1 2.816177 -2.256106 0.003195
8 3.588554 -0.396335 -0.011933
1 -1.916562 -1.343932 0.029741
8 -4.683762 -0.578840 -0.007033

1 -4.292766 -1.444672 -0.193250
1 -3.925622 0.033261 0.001648
rc_5fU/rc_5fUadc.xyz
6 -1.235387 0.829196 -0.000002
6 0.651830 -0.717965 -0.000002
6 1.540119 0.424243 0.000000
6 1.019486 1.704390 0.000000
1 1.641218 2.593550 0.000002
7 -0.304134 1.876299 0.000001
7 -0.687034 -0.443972 -0.000001
8 -2.436654 1.035913 -0.000003
8 1.096488 -1.884466 -0.000004
1 -1.346419 -1.222816 -0.000007
6 2.911798 0.072085 0.000002
1 3.701069 0.841592 0.000004
8 3.259597 -1.124551 0.000003
1 -0.698713 2.816556 0.000005
8 -4.047206 -1.417615 0.000006
1 -5.004541 -1.276264 -0.000003
1 -3.659313 -0.524853 -0.000002
rc_5fU/rc_5fUabu.xyz
6 -2.167266 -0.338692 0.016151
6 0.175591 -0.339917 -0.670852
6 0.329803 0.946196 -0.025812
6 -0.748887 1.532812 0.609373
1 -0.686733 2.492426 1.111653
7 -1.926242 0.906562 0.619296
7 -1.064700 -0.906613 -0.609795
8 -3.260386 -0.870482 0.044250
8 1.136196 -0.893969 -1.246639
1 -1.214964 -1.810349 -1.057411
6 1.642872 1.467275 -0.112763
1 1.906239 2.436312 0.340110
8 2.539569 0.834114 -0.703920
1 -2.728944 1.331817 1.082320
8 2.502834 -1.719815 1.584743

1 2.800393 -0.821047 1.381536
1 2.122231 -2.033639 0.751231
rc_5fU/rc_5fUaaj.xyz
6 -1.241935 0.839281 -0.003828
6 0.640884 -0.713689 -0.006943
6 1.532539 0.425793 0.001952
6 1.015578 1.707457 0.009436
1 1.640177 2.594588 0.017341
7 -0.307530 1.883664 0.006868
7 -0.696911 -0.435268 -0.009112
8 -2.442726 1.048529 -0.007978
8 1.081691 -1.881632 -0.012338
1 -1.359243 -1.211348 -0.020176
6 2.903127 0.069434 0.004644
1 3.694800 0.836434 0.011155
8 3.247242 -1.128288 -0.000819
1 -0.699015 2.825207 0.011864
8 -4.148704 -1.369654 -0.037105
1 -3.576772 -1.982807 0.447023
1 -3.670036 -0.522155 -0.017142
rc_5fU/rc_5fUacg.xyz
6 2.290364 -0.643249 0.051507
6 -0.121147 -0.397296 -0.241918
6 0.027691 1.022064 -0.003927
6 1.281897 1.551676 0.240729
1 1.451096 2.608995 0.417895
7 2.343532 0.744948 0.262245
7 1.017089 -1.147642 -0.182533
8 3.285944 -1.340545 0.074667
8 -1.236848 -0.898666 -0.493704
1 0.951175 -2.150848 -0.353377
6 -1.188558 1.740842 -0.086302
1 -1.223307 2.829916 0.077800
8 -2.259147 1.156313 -0.342639
1 3.272393 1.128744 0.431989
8 -4.052602 -1.358656 0.560891

1 -3.132084 -1.205794 0.297265
1 -4.483883 -0.503956 0.416185
rc_5fU/rc_5fUabm.xyz
6 -2.129597 -0.286069 -0.044804
6 0.226273 -0.289209 -0.686279
6 0.388072 0.957777 0.030284
6 -0.695054 1.530078 0.671066
1 -0.627911 2.461090 1.224045
7 -1.883783 0.926607 0.619788
7 -1.023000 -0.839282 -0.676567
8 -3.229406 -0.804532 -0.060092
8 1.188258 -0.825753 -1.275416
1 -1.177193 -1.717186 -1.171791
6 1.711125 1.459467 -0.006347
1 1.983695 2.397834 0.502359
8 2.606470 0.843176 -0.617482
1 -2.688481 1.340007 1.090052
8 2.159660 -1.927691 1.572987
1 2.540253 -1.239966 1.007070
1 1.512341 -2.366918 1.002226
rc_5fU/rc_5fUaas.xyz
6 -2.589557 0.131819 0.082593
6 -0.391685 -0.926592 -0.029666
6 0.200902 0.390887 -0.118693
6 -0.607375 1.511664 -0.105946
1 -0.216204 2.521875 -0.167308
7 -1.930249 1.368640 -0.012079
7 -1.752806 -0.976551 0.069222
8 -3.799559 0.048054 0.170605
8 0.316027 -1.956170 -0.041273
1 -2.207119 -1.887354 0.132009
6 1.613601 0.369579 -0.201306
1 2.200405 1.299231 -0.262829
8 2.230919 -0.713679 -0.200992
1 -2.533331 2.190419 -0.000361
8 5.021021 0.581638 0.297932

1 4.603631 0.487369 -0.570864
1 4.431426 0.100944 0.897278
rc_5hmC/rc_5hmCacg.xyz
6 2.388942 0.065469 -0.285696
6 0.218426 -0.861393 0.288510
6 -0.249088 0.486132 0.540066
6 0.688208 1.529058 0.319280
1 0.358785 2.552016 0.483412
7 1.934173 1.363884 -0.065314
7 1.503919 -1.000864 -0.110845
8 3.544052 -0.161449 -0.634529
6 -1.606727 0.799033 1.102371
1 -1.501356 0.837781 2.197219
1 -1.895657 1.807528 0.778487
7 -0.555693 -1.909771 0.450662
1 -0.209970 -2.853707 0.297615
1 -1.534779 -1.744560 0.698663
8 -2.614094 -0.152127 0.801776
1 -2.839726 -0.058382 -0.159624
1 1.877700 -1.931836 -0.293619
8 -3.003254 0.163194 -1.860343
1 -3.929883 0.313492 -2.101794
1 -2.554108 0.998189 -2.064302
rc_5hmC/rc_5hmCaaz.xyz
6 1.473423 1.308074 -0.042440
6 -0.160107 -0.476850 0.055601
6 -1.206916 0.524303 0.069785
6 -0.780565 1.877662 0.014555
1 -1.544281 2.651908 0.016206
7 0.469987 2.279709 -0.037525
7 1.117369 -0.034664 -0.001862
8 2.662124 1.619761 -0.087891
6 -2.662930 0.203448 0.246663
1 -2.868212 0.190235 1.326754
1 -3.260958 1.013818 -0.186092
7 -0.404052 -1.767387 0.111204

1 0.374207 -2.431696 0.117609
1 -1.374564 -2.078838 0.087206
8 -3.059458 -1.066199 -0.261487
1 -3.113345 -1.012230 -1.228907
1 1.870200 -0.741341 -0.016190
8 2.383396 -2.537878 0.018451
1 2.801902 -2.882638 -0.784563
1 2.965999 -2.798120 0.747692
rc_5hmC/rc_5hmCacm.xyz
6 2.395204 -0.038421 -0.315082
6 0.208591 -0.753897 0.465031
6 -0.212631 0.630935 0.441823
6 0.768831 1.579788 0.049220
1 0.484635 2.629104 0.039353
7 2.000679 1.297813 -0.309353
7 1.479313 -1.015184 0.080601
8 3.524506 -0.375894 -0.659370
6 -1.568466 1.098601 0.884514
1 -1.479333 1.402978 1.938734
1 -1.825303 2.004765 0.319192
7 -0.588092 -1.722964 0.854388
1 -0.273164 -2.689257 0.885177
1 -1.559003 -1.482715 1.070192
8 -2.594302 0.124992 0.803253
1 -2.824085 -0.004825 -0.152939
1 1.815244 -1.978176 0.069309
8 -3.081292 -0.212759 -1.837482
1 -4.007296 -0.054784 -2.076135
1 -2.915472 -1.137488 -2.076584
rc_5hmC/rc_5hmCabj.xyz
6 1.400029 1.250617 -0.040136
6 -0.224840 -0.534907 0.049033
6 -1.275341 0.463754 0.069010
6 -0.853058 1.818094 0.015133
1 -1.618611 2.590485 0.017716
7 0.396645 2.222161 -0.036874

7 1.050073 -0.094424 -0.002679
8 2.588537 1.565559 -0.081938
6 -2.730407 0.141116 0.252082
1 -2.929479 0.124061 1.333321
1 -3.331377 0.952431 -0.174583
7 -0.479646 -1.825538 0.094251
1 0.273548 -2.508737 0.090129
1 -1.452825 -2.130230 0.066411
8 -3.129750 -1.127055 -0.258158
1 -3.191991 -1.069077 -1.224875
1 1.827488 -0.793397 -0.011873
8 2.896937 -2.164081 -0.021549
1 3.695688 -1.964801 -0.533650
1 3.213967 -2.343552 0.876938
rc_5hmC/rc_5hmCaab.xyz
6 -2.400676 -0.033590 0.321969
6 -0.213993 -0.769653 -0.439366
6 0.206379 0.615362 -0.455288
6 -0.779211 1.575371 -0.099516
1 -0.497383 2.625133 -0.125937
7 -2.010835 1.303302 0.267331
7 -1.481506 -1.021260 -0.037638
8 -3.528939 -0.362051 0.678189
6 1.564693 1.073678 -0.900654
1 1.476026 1.377844 -1.954965
1 1.826314 1.979970 -0.337013
7 0.578983 -1.748616 -0.811157
1 0.262683 -2.714957 -0.815798
1 1.547920 -1.514404 -1.041695
8 2.585942 0.095951 -0.818271
1 2.825833 -0.023532 0.136559
1 -1.817394 -1.983509 0.001329
8 3.112530 -0.255375 1.817252
1 3.972862 -0.677892 1.962994
1 3.177223 0.602156 2.264544
rc_5hmC/rc_5hmCabp.xyz

6 -2.421187 -0.058660 -0.307862
6 -0.248561 0.851834 0.285873
6 0.217040 -0.500827 0.507579
6 -0.725408 -1.537234 0.272133
1 -0.399208 -2.563674 0.420894
7 -1.970394 -1.362079 -0.109655
7 -1.533865 1.001425 -0.111504
8 -3.575210 0.177806 -0.654300
6 1.573678 -0.837732 1.058622
1 1.453896 -0.971067 2.144876
1 1.879909 -1.812726 0.656393
7 0.526093 1.896288 0.470300
1 0.181658 2.843265 0.334145
1 1.502074 1.722737 0.726364
8 2.572652 0.145424 0.850775
1 2.871800 0.077637 -0.091852
1 -1.906059 1.936727 -0.273807
8 3.213567 -0.128336 -1.771360
1 4.153753 0.010536 -1.961630
1 3.047901 -1.056311 -1.998366
rc_5hmC/rc_5hmCack.xyz
6 -2.175513 -0.412787 -0.093663
6 -0.354831 1.150358 0.281798
6 0.542350 0.198333 -0.341415
6 -0.045169 -1.007099 -0.809144
1 0.601339 -1.734780 -1.292442
7 -1.315701 -1.324179 -0.703151
7 -1.659625 0.801385 0.363715
8 -3.372202 -0.649647 0.042200
6 2.031819 0.372709 -0.406634
1 2.453224 -0.104482 0.489577
1 2.416564 -0.177688 -1.273233
7 0.061171 2.299323 0.763123
1 -0.580508 2.949327 1.210068
1 1.042660 2.550729 0.632558
8 2.479200 1.723541 -0.405278

1 2.340776 2.099106 -1.289225
1 -2.328989 1.443149 0.788178
8 2.226104 -2.893019 0.855866
1 1.262696 -2.983254 0.817271
1 2.534580 -3.733910 1.223472
rc_5hmC/rc_5hmCade.xyz
6 1.465821 1.498824 -0.044038
6 -0.070257 -0.383176 0.067075
6 -1.167397 0.563090 0.075338
6 -0.816518 1.937483 0.023257
1 -1.621398 2.668568 0.028136
7 0.409142 2.409209 -0.032125
7 1.182565 0.135700 -0.000652
8 2.634407 1.874433 -0.095835
6 -2.606580 0.165272 0.237468
1 -2.820339 0.130287 1.315345
1 -3.242057 0.948102 -0.192818
7 -0.243401 -1.680432 0.134950
1 0.571561 -2.326803 0.128614
1 -1.200678 -2.032274 0.112305
8 -2.933762 -1.117675 -0.286200
1 -2.964328 -1.062334 -1.254686
1 1.985635 -0.491895 -0.009516
8 2.083723 -3.228989 0.103268
1 2.000968 -4.069586 -0.372450
1 2.747141 -2.726524 -0.394609
rc_5hmC/rc_5hmCacj.xyz
6 1.401439 1.239853 -0.044979
6 -0.230505 -0.537805 0.052187
6 -1.276670 0.465560 0.069983
6 -0.848624 1.817907 0.013018
1 -1.610713 2.593709 0.015600
7 0.402836 2.216248 -0.040772
7 1.045573 -0.103851 -0.007407
8 2.591413 1.548965 -0.087559
6 -2.732620 0.148724 0.255508

1 -2.930273 0.130907 1.336954
1 -3.331026 0.962842 -0.169409
7 -0.490810 -1.826944 0.107780
1 0.258622 -2.513423 0.119710
1 -1.465030 -2.128365 0.082137
8 -3.137216 -1.117416 -0.255797
1 -3.196547 -1.059033 -1.222685
1 1.823346 -0.803492 -0.022785
8 3.013565 -2.056777 -0.067526
1 3.528432 -2.051707 0.754013
1 2.599782 -2.933214 -0.097998
rc_5hmC/rc_5hmCacc.xyz
6 -2.267075 0.070604 0.122922
6 -0.079190 -0.905350 -0.272567
6 0.407096 0.418750 -0.609019
6 -0.530511 1.478404 -0.513555
1 -0.187058 2.484512 -0.739701
7 -1.794109 1.346630 -0.174416
7 -1.385255 -1.011895 0.062472
8 -3.436645 -0.123796 0.441389
6 1.804953 0.671153 -1.094510
1 1.830273 0.462078 -2.173692
1 2.037340 1.735152 -0.965006
7 0.697572 -1.963648 -0.302306
1 0.338658 -2.891193 -0.090229
1 1.694706 -1.826988 -0.477132
8 2.785023 -0.156379 -0.482007
1 2.750721 0.038762 0.484847
1 -1.775354 -1.922542 0.304884
8 2.105404 0.496655 2.104326
1 1.641024 1.339636 1.982863
1 1.400340 -0.150229 2.263631
rc_5hmC/rc_5hmCabk.xyz
6 1.460153 1.490196 -0.051383
6 -0.075259 -0.392167 0.053871
6 -1.172215 0.553847 0.080102

6 -0.821815 1.928622 0.037960
1 -1.626559 2.659639 0.056132
7 0.403622 2.400676 -0.021898
7 1.177104 0.126811 -0.020598
8 2.628331 1.866510 -0.108117
6 -2.609252 0.152678 0.252024
1 -2.810908 0.099146 1.331474
1 -3.250156 0.941820 -0.158106
7 -0.247082 -1.690063 0.113033
1 0.571540 -2.331578 0.107326
1 -1.203567 -2.043881 0.094665
8 -2.940839 -1.121683 -0.289568
1 -2.979523 -1.050361 -1.256734
1 1.978574 -0.502701 -0.049634
8 2.081183 -3.231422 0.089602
1 2.336320 -3.450969 -0.819452
1 2.809698 -2.689366 0.429788
rc_5hmC/rc_5hmCaby.xyz
6 -1.448971 -1.487173 -0.049294
6 0.079914 0.401235 0.039291
6 1.179768 -0.540615 0.082929
6 0.834308 -1.916946 0.050827
1 1.641502 -2.644933 0.078498
7 -0.389306 -2.393649 -0.009697
7 -1.170563 -0.122514 -0.032370
8 -2.615730 -1.868128 -0.103452
6 2.613710 -0.132372 0.262946
1 2.805285 -0.065639 1.343526
1 3.261219 -0.923718 -0.132285
7 0.247132 1.700644 0.079663
1 -0.575328 2.335336 0.067180
1 1.202020 2.058742 0.061626
8 2.945570 1.136863 -0.290387
1 2.994255 1.054243 -1.256209
1 -1.973235 0.505283 -0.070681
8 -2.131538 3.179050 0.021878

1 -2.566563 3.132427 0.886936
1 -2.738788 2.729839 -0.586267
rc_5hmC/rc_5hmCaax.xyz
6 -2.292256 -0.057711 -0.168682
6 -0.102780 0.870101 0.323931
6 0.368008 -0.477018 0.579336
6 -0.578468 -1.518585 0.406018
1 -0.247526 -2.540175 0.574363
7 -1.835704 -1.354161 0.057518
7 -1.400662 1.009400 -0.030597
8 -3.455091 0.167900 -0.491390
6 1.752547 -0.771985 1.080196
1 1.741952 -0.663981 2.174563
1 1.990622 -1.820654 0.862690
7 0.678595 1.918448 0.446758
1 0.327717 2.860995 0.296575
1 1.670557 1.761614 0.635637
8 2.750370 0.105771 0.578478
1 2.765151 -0.005241 -0.403813
1 -1.779108 1.938857 -0.212007
8 2.323646 -0.247720 -2.107962
1 1.451666 0.171150 -2.180476
1 2.149655 -1.194778 -2.221089
rc_5hmC/rc_5hmCadf.xyz
6 2.600439 0.204093 -0.149398
6 0.244934 0.700615 0.187306
6 -0.028247 -0.717119 0.077436
6 1.089473 -1.558918 -0.167458
1 0.908570 -2.626246 -0.268088
7 2.335134 -1.157768 -0.277973
7 1.539235 1.081554 0.082144
8 3.741273 0.648849 -0.233835
6 -1.402230 -1.318342 0.106710
1 -1.747095 -1.404970 -0.933943
1 -1.338462 -2.335755 0.509420
7 -0.701506 1.594257 0.364320

1 -0.486425 2.587206 0.411798
1 -1.660466 1.271932 0.495069
8 -2.379329 -0.548921 0.797133
1 -2.283120 -0.671135 1.754873
1 1.782950 2.069351 0.156573
8 -4.317399 0.689476 -0.862251
1 -5.052624 0.074932 -0.995789
1 -3.715948 0.231179 -0.245298
rc_5hmC/rc_5hmCabl.xyz
6 2.475286 -0.290158 -0.085423
6 0.082711 -0.678087 0.116656
6 -0.092961 0.756795 0.252868
6 1.081555 1.547706 0.200230
1 0.972551 2.624695 0.298254
7 2.304655 1.087623 0.044086
7 1.354678 -1.118893 -0.041844
8 3.588023 -0.785883 -0.238706
6 -1.444958 1.388922 0.425154
1 -1.979340 0.917143 1.254919
1 -1.323347 2.451710 0.651064
7 -0.907723 -1.535065 0.162757
1 -0.714293 -2.531187 0.081669
1 -1.906069 -1.263307 0.224684
8 -2.253925 1.195120 -0.741698
1 -1.891455 1.721313 -1.471451
1 1.536473 -2.117216 -0.143109
8 -3.692727 -1.064801 0.145767
1 -3.615804 -0.238013 -0.364135
1 -4.080740 -1.707337 -0.466708
rc_5hmC/rc_5hmCadd.xyz
6 2.478372 -0.269446 -0.075352
6 0.088209 -0.688341 0.099575
6 -0.106021 0.743392 0.247487
6 1.059675 1.548186 0.213733
1 0.936818 2.622774 0.320896
7 2.289907 1.104980 0.064691

7 1.367191 -1.111399 -0.051463
8 3.598337 -0.751017 -0.220787
6 -1.465278 1.361801 0.415144
1 -2.000761 0.879421 1.238445
1 -1.354435 2.423965 0.649212
7 -0.891196 -1.558861 0.127627
1 -0.683360 -2.551369 0.038608
1 -1.893171 -1.299151 0.190360
8 -2.267174 1.169514 -0.756706
1 -1.907423 1.703246 -1.482351
1 1.562413 -2.106502 -0.158801
8 -3.667205 -1.128294 0.019532
1 -3.568292 -0.250623 -0.394743
1 -4.094514 -0.959970 0.872550
rc_5hmC/rc_5hmCadu.xyz
6 2.688526 -0.097287 -0.117530
6 0.455725 0.850715 0.013965
6 -0.068427 -0.496856 0.105306
6 0.884573 -1.548912 0.080819
1 0.517970 -2.569742 0.152422
7 2.185062 -1.393334 -0.023282
7 1.798420 0.978259 -0.093497
8 3.892569 0.118890 -0.216883
6 -1.532093 -0.823542 0.110845
1 -1.850922 -0.938973 -0.935274
1 -1.684342 -1.789532 0.604464
7 -0.320562 1.910107 0.019346
1 0.065505 2.847563 -0.060871
1 -1.321393 1.778731 0.171038
8 -2.362744 0.186345 0.680960
1 -2.311618 0.130182 1.648944
1 2.214709 1.907726 -0.152600
8 -4.914661 -0.112950 -0.534129
1 -4.067574 0.008580 -0.061380
1 -4.693913 -0.682744 -1.284728
rc_5hmC/rc_5hmCacu.xyz

6 2.692490 -0.114147 -0.105594
6 0.468436 0.856850 0.004133
6 -0.069847 -0.485414 0.093883
6 0.872837 -1.546947 0.086201
1 0.495675 -2.563689 0.160531
7 2.175823 -1.404675 -0.006759
7 1.813164 0.970428 -0.091516
8 3.899026 0.089887 -0.199803
6 -1.537235 -0.794587 0.078049
1 -1.844211 -0.891973 -0.973231
1 -1.707667 -1.764395 0.557920
7 -0.297600 1.923813 -0.000273
1 0.098965 2.856931 -0.080328
1 -1.300972 1.803369 0.142973
8 -2.361974 0.219357 0.650330
1 -2.311098 0.160260 1.618235
1 2.239337 1.895334 -0.151631
8 -4.934130 -0.121066 -0.507195
1 -4.076838 0.014000 -0.056640
1 -4.816359 -0.936746 -1.014676
rc_5hmC/rc_5hmCaau.xyz
6 -2.697831 -0.080370 0.110022
6 -0.455215 0.843600 -0.020895
6 0.056465 -0.509967 -0.090170
6 -0.906781 -1.552414 -0.054837
1 -0.549792 -2.577713 -0.108282
7 -2.206259 -1.382459 0.038707
7 -1.797139 0.986092 0.074743
8 -3.900269 0.148760 0.199381
6 1.516677 -0.851456 -0.080615
1 1.823531 -0.961905 0.969401
1 1.663224 -1.823136 -0.564843
7 0.331628 1.895026 -0.034851
1 -0.045012 2.837512 0.029743
1 1.332412 1.750917 -0.175169
8 2.363643 0.144646 -0.650362

1 2.317698 0.084662 -1.618500
1 -2.204499 1.920428 0.117954
8 4.958181 -0.153314 0.470934
1 4.833877 0.094831 1.397951
1 4.078607 -0.033297 0.060895
rc_5hmC/rc_5hmCach.xyz
6 -1.362387 0.408213 -0.027911
6 1.022815 0.842834 0.058420
6 1.244095 -0.588143 0.062118
6 0.082484 -1.405682 0.035006
1 0.220022 -2.483927 0.047612
7 -1.155106 -0.967359 -0.006286
7 -0.262471 1.262919 -0.001983
8 -2.492654 0.891159 -0.071152
6 2.593865 -1.229414 0.195956
1 2.757102 -1.426235 1.265951
1 2.573034 -2.202955 -0.308396
7 2.007258 1.708852 0.126829
1 1.828782 2.710113 0.147948
1 2.965479 1.354608 0.110032
8 3.684187 -0.433229 -0.250345
1 3.710048 -0.455095 -1.220289
1 -0.470977 2.261353 -0.014457
8 -4.887612 -0.676928 0.001999
1 -4.051252 -0.173923 -0.039093
1 -5.576605 -0.029685 -0.204769
rc_5hmU/rc_5hmUaas.xyz
6 1.297857 1.107760 -0.021795
6 -1.152938 0.897445 0.032198
6 -0.998428 -0.564306 0.023192
6 0.310618 -1.089099 0.044985
1 0.492187 -2.159698 0.073864
7 1.369914 -0.303505 0.015151
7 0.024341 1.623627 -0.013167
8 2.311426 1.783757 -0.051660
8 -2.248857 1.461269 0.083259

1 -0.053948 2.640080 -0.016738
6 -2.149482 -1.495608 0.085738
1 -1.956277 -2.364134 -0.560412
1 -2.125993 -1.911716 1.119685
8 -3.430082 -0.994209 -0.209320
1 -3.459230 -0.041660 0.000898
1 2.343274 -0.737115 0.004417
8 3.790276 -1.465679 -0.035417
1 3.814860 -2.155974 -0.716897
1 3.957471 -1.928883 0.800493
rc_5hmU/rc_5hmUaav.xyz
6 -1.302084 1.098577 -0.020500
6 1.150089 0.900817 0.029131
6 1.003088 -0.561656 0.023786
6 -0.303430 -1.092762 0.049787
1 -0.479049 -2.164182 0.081761
7 -1.366506 -0.312415 0.020409
7 -0.031371 1.620954 -0.017614
8 -2.319855 1.768698 -0.049038
8 2.242720 1.470797 0.078415
1 0.042120 2.637762 -0.022394
6 2.157376 -1.489077 0.086431
1 2.134855 -1.905930 1.120036
1 1.966527 -2.357742 -0.560247
8 3.436647 -0.984771 -0.209184
1 3.467463 -0.034476 0.009309
1 -2.336324 -0.753005 0.011729
8 -3.705445 -1.619750 0.002629
1 -4.344329 -1.222309 0.614971
1 -4.128889 -1.575069 -0.869109
rc_5hmU/rc_5hmUabf.xyz
6 -1.428139 1.063091 0.000625
6 0.319077 -0.646637 -0.008593
6 1.335851 0.415756 0.002521
6 0.896712 1.751077 0.008492
1 1.591147 2.586043 0.013931

7 -0.394670 2.036519 0.008344
7 -0.996706 -0.234646 -0.005648
8 -2.597336 1.408794 -0.000281
8 0.613978 -1.848084 -0.019633
1 -1.748195 -0.965879 -0.016449
6 2.789023 0.152654 -0.001358
1 3.216922 0.679433 -0.880220
1 3.232565 0.697880 0.856551
8 3.231277 -1.179330 0.010624
1 2.455219 -1.777114 -0.006504
1 -0.702897 3.010520 0.013708
8 -3.092431 -2.035763 -0.076589
1 -3.142342 -2.552228 0.742381
1 -3.881835 -1.472353 -0.065363
rc_5hmU/rc_5hmUaab.xyz
6 -1.428903 1.065570 -0.003606
6 0.317062 -0.645002 0.016242
6 1.334572 0.416762 0.000448
6 0.896257 1.752447 -0.000641
1 1.591210 2.587001 -0.001636
7 -0.394943 2.038618 -0.003701
7 -0.998291 -0.232244 0.007796
8 -2.598003 1.411544 -0.012661
8 0.611210 -1.846547 0.036098
1 -1.750628 -0.963074 0.019663
6 2.787565 0.152107 0.007826
1 3.245020 0.724363 -0.823064
1 3.201658 0.648525 0.912032
8 3.228312 -1.179486 -0.044658
1 2.453481 -1.777346 -0.000547
1 -0.702622 3.012803 -0.008659
8 -3.097465 -2.021920 0.086542
1 -3.010566 -2.731072 -0.568974
1 -3.866660 -1.505842 -0.201660
rc_5hmU/rc_5hmUacj.xyz
6 -1.438684 1.118410 -0.001630

6 0.279755 -0.619455 -0.019547
6 1.315155 0.424642 0.000539
6 0.898291 1.767198 0.000869
1 1.606693 2.590442 0.004340
7 -0.387995 2.074023 0.000288
7 -1.029199 -0.185884 -0.012961
8 -2.601291 1.485447 0.006203
8 0.553044 -1.826048 -0.041307
1 -1.782502 -0.916219 -0.019475
6 2.763628 0.135070 -0.004499
1 3.186269 0.618601 -0.911827
1 3.231562 0.703209 0.823105
8 3.179176 -1.204393 0.055371
1 2.392723 -1.786575 0.006245
1 -0.679642 3.053109 0.005448
8 -2.992752 -2.122557 -0.065375
1 -2.541618 -2.980977 -0.068955
1 -3.507414 -2.113345 0.756286
rc_5hmU/rc_5hmUaai.xyz
6 -2.468479 -0.148542 0.335163
6 -0.219323 -0.841450 -0.379997
6 0.189479 0.572017 -0.388485
6 -0.784643 1.545591 -0.092543
1 -0.560797 2.607800 -0.121275
7 -2.011725 1.193183 0.247856
7 -1.520970 -1.089218 0.019511
8 -3.612660 -0.393405 0.664609
8 0.532542 -1.760224 -0.710524
1 -1.824533 -2.062532 0.045435
6 1.546325 1.017383 -0.776153
1 1.421283 1.537689 -1.753053
1 1.880986 1.811332 -0.090431
8 2.557361 0.044394 -0.868755
1 2.151726 -0.831432 -1.028061
1 -2.706147 1.908216 0.476782
8 3.840718 -0.175097 1.677206

1 4.796395 -0.177960 1.525718
1 3.446100 -0.116207 0.785119
rc_5hmU/rc_5hmUabt.xyz
6 -1.321599 0.596845 -0.018570
6 1.118541 0.889253 -0.004322
6 1.270730 -0.571735 -0.010468
6 0.106822 -1.359406 -0.031418
1 0.148030 -2.444343 -0.044669
7 -1.093318 -0.799466 -0.035538
7 -0.184086 1.359276 -0.010269
8 -2.453454 1.049725 -0.013835
8 2.077175 1.665675 0.006620
1 -0.318374 2.370134 0.000422
6 2.582375 -1.247611 -0.004826
1 2.585293 -1.980543 0.828255
1 2.619228 -1.901162 -0.903562
8 3.743501 -0.465094 0.054317
1 3.498083 0.482889 0.043426
1 -1.932273 -1.384388 -0.052823
8 -4.696607 -0.807047 0.061883
1 -4.021969 -0.105685 0.026629
1 -5.542318 -0.345710 -0.031286
rc_5hmU/rc_5hmUaci.xyz
6 -1.323437 -0.619640 0.009675
6 1.119090 -0.888751 0.005111
6 1.257919 0.573530 0.009247
6 0.086483 1.350466 0.025752
1 0.116834 2.435828 0.037746
7 -1.108099 0.778767 0.027055
7 -0.178977 -1.371333 0.006090
8 -2.451150 -1.082879 0.000443
8 2.084966 -1.656264 0.000192
1 -0.303108 -2.383500 -0.003733
6 2.563574 1.260887 0.005901
1 2.564091 1.986791 -0.833560
1 2.589701 1.922769 0.898716

8 3.732126 0.488734 -0.040596
1 3.495421 -0.461417 -0.028070
1 -1.953818 1.354539 0.041417
8 -4.658609 0.818961 -0.105654
1 -4.016046 0.091577 -0.031335
1 -5.363989 0.594006 0.517603
rc_5hmU/rc_5hmUabn.xyz
6 -2.659779 0.041067 -0.178321
6 -0.373051 0.908006 0.093577
6 0.113533 -0.475254 0.204435
6 -0.821012 -1.519855 0.075783
1 -0.523141 -2.563003 0.120418
7 -2.105875 -1.262480 -0.093509
7 -1.738848 1.052971 -0.079263
8 -3.855912 0.198663 -0.328248
8 0.372757 1.888509 0.142473
1 -2.103290 2.002848 -0.149466
6 1.538951 -0.821799 0.383429
1 1.626054 -1.605340 1.155037
1 1.847755 -1.338194 -0.553900
8 2.441202 0.213730 0.680210
1 2.038074 1.076090 0.453288
1 -2.776668 -2.029725 -0.172930
8 4.914484 -0.211716 -0.681826
1 4.959799 0.441022 -1.394752
1 4.072369 -0.023612 -0.222578
rc_5hmU/rc_5hmUaay.xyz
6 2.572841 0.097511 0.292341
6 0.300976 0.880513 -0.228426
6 -0.135515 -0.519359 -0.330543
6 0.822999 -1.529745 -0.128406
1 0.569625 -2.583236 -0.200364
7 2.075888 -1.225089 0.161943
7 1.633451 1.076215 0.085928
8 3.740311 0.298442 0.565556
8 -0.459731 1.835304 -0.407748

1 1.959475 2.039651 0.160679
6 -1.523393 -0.914624 -0.645880
1 -1.485340 -1.550023 -1.555117
1 -1.865815 -1.614409 0.141303
8 -2.481496 0.098523 -0.816667
1 -2.046763 0.974907 -0.755946
1 2.763347 -1.967125 0.309128
8 -4.437563 -0.086641 1.265806
1 -5.180264 0.483950 1.022872
1 -3.799251 0.007595 0.532264
rc_5hmU/rc_5hmUaap.xyz
6 -2.574365 -0.080473 -0.291067
6 -0.308723 -0.887120 0.221215
6 0.139776 0.507993 0.334165
6 -0.810041 1.528143 0.140477
1 -0.547303 2.578771 0.220188
7 -2.065650 1.236672 -0.151383
7 -1.643652 -1.068880 -0.091608
8 -3.743565 -0.268873 -0.565768
8 0.444348 -1.849662 0.390704
1 -1.977933 -2.028821 -0.174281
6 1.531293 0.889440 0.649555
1 1.877892 1.591748 -0.133601
1 1.500379 1.518406 1.563448
8 2.481709 -0.132546 0.811156
1 2.041082 -1.005310 0.740959
1 -2.746695 1.985821 -0.291985
8 4.432310 0.010399 -1.276142
1 5.232457 0.399728 -0.896049
1 3.799169 -0.037331 -0.533419
rc_5hmU/rc_5hmUacn.xyz
6 1.328156 0.647962 -0.038152
6 -1.117101 0.890690 -0.007537
6 -1.240854 -0.573084 -0.017136
6 -0.061450 -1.337490 -0.053642
1 -0.079651 -2.423035 -0.073914

7 1.126702 -0.752663 -0.061052
7 0.175777 1.387318 -0.022317
8 2.451169 1.122252 -0.036869
8 -2.090756 1.647824 0.011708
1 0.288720 2.400746 -0.007584
6 -2.539495 -1.273763 -0.013485
1 -2.579654 -1.879813 -0.946309
1 -2.515600 -2.047516 0.779576
8 -3.714545 -0.519475 0.108555
1 -3.495484 0.434223 0.081400
1 1.978428 -1.319355 -0.083838
8 4.687967 -0.776066 0.162729
1 4.372521 -1.464173 -0.440817
1 4.027144 -0.065821 0.085794
rc_5hmU/rc_5hmUace.xyz
6 2.260170 -0.222110 0.085583
6 -0.047710 -0.558714 -0.698586
6 -0.389069 0.784444 -0.193847
6 0.648782 1.576628 0.339751
1 0.478059 2.594893 0.676088
7 1.870828 1.092215 0.463822
7 1.255787 -0.972009 -0.467492
8 3.403510 -0.598675 0.254251
8 -0.847094 -1.276610 -1.295136
1 1.512158 -1.906199 -0.786874
6 -1.756431 1.358875 -0.306441
1 -1.951536 2.010285 0.553319
1 -1.723994 2.034618 -1.188561
8 -2.826735 0.445649 -0.407729
1 -2.615804 -0.222227 -1.084782
1 2.613534 1.668506 0.867080
8 -2.269586 -1.320005 1.835546
1 -2.564438 -0.753267 1.097600
1 -1.609486 -1.905668 1.437596
rc_5hmU/rc_5hmUaam.xyz
6 2.653248 0.009790 0.177643

6 0.381149 0.922787 -0.067646
6 -0.128923 -0.448505 -0.213280
6 0.787495 -1.511941 -0.110752
1 0.472090 -2.548465 -0.182395
7 2.076436 -1.281594 0.065288
7 1.750189 1.039538 0.101850
8 3.852206 0.142831 0.329054
8 -0.347757 1.916843 -0.088336
1 2.131660 1.980674 0.194658
6 -1.558159 -0.765732 -0.415989
1 -1.649242 -1.521227 -1.214807
1 -1.886436 -1.307526 0.500009
8 -2.440769 0.293363 -0.689165
1 -2.025075 1.142622 -0.437246
1 2.733530 -2.062532 0.123706
8 -4.889016 -0.142378 0.718786
1 -4.062639 0.025058 0.224651
1 -4.706426 -0.937886 1.238895
rc_5hmU/rc_5hmUacw.xyz
6 2.660610 0.027249 -0.174833
6 0.379277 0.913705 0.080842
6 -0.117546 -0.464846 0.203765
6 0.809668 -1.517449 0.087594
1 0.504119 -2.557938 0.141794
7 2.096789 -1.271381 -0.080592
7 1.747021 1.046697 -0.087205
8 3.858374 0.174486 -0.322411
8 -0.359466 1.899971 0.116803
1 2.118943 1.992983 -0.165793
6 -1.545375 -0.799624 0.386839
1 -1.858678 -1.321314 -0.546066
1 -1.637257 -1.575623 1.165380
8 -2.439811 0.244808 0.676483
1 -2.032412 1.102048 0.438251
1 2.761991 -2.044493 -0.149893
8 -4.894870 -0.068675 -0.756590

1 -4.067489 0.022793 -0.244807
1 -5.129506 -1.004608 -0.683810
rc_5hmU/rc_5hmUabb.xyz
6 -2.577254 -0.089984 0.289248
6 -0.306726 -0.884172 -0.220507
6 0.135165 0.513459 -0.329079
6 -0.819944 1.528420 -0.134366
1 -0.562318 2.580547 -0.210965
7 -2.074731 1.229996 0.154191
7 -1.641208 -1.073262 0.089517
8 -3.746181 -0.285028 0.560491
8 0.451340 -1.842651 -0.391052
1 -1.970960 -2.035065 0.168787
6 1.525178 0.902068 -0.642753
1 1.491890 1.532540 -1.555575
1 1.868119 1.604874 0.141534
8 2.480107 -0.115415 -0.805910
1 2.042982 -0.990079 -0.737408
1 -2.759757 1.975388 0.295435
8 4.449308 0.079295 1.259710
1 3.806028 -0.011106 0.529852
1 5.280489 -0.262586 0.901216
rc_5hmU/rc_5hmUadi.xyz
6 1.326970 0.651985 -0.015750
6 -1.118795 0.889207 -0.001004
6 -1.239325 -0.574702 -0.007168
6 -0.057805 -1.336642 -0.025427
1 -0.073292 -2.422384 -0.036850
7 1.129036 -0.749233 -0.027762
7 0.172879 1.388761 -0.006449
8 2.448715 1.129543 -0.015977
8 -2.094323 1.644372 0.008179
1 0.283310 2.402537 0.001293
6 -2.536309 -1.278254 -0.009750
1 -2.555383 -1.924977 -0.914244
1 -2.526431 -2.018084 0.816907

8 -3.714917 -0.522496 0.052066
1 -3.492477 0.430942 0.040103
1 1.983179 -1.313464 -0.038316
8 4.602826 -0.850231 -0.053521
1 5.061311 -0.877043 0.798392
1 4.019543 -0.073293 0.000807
rc_5hmU/rc_5hmUadf.xyz
6 -1.326282 0.615503 0.002474
6 1.115581 0.889061 0.000681
6 1.256787 -0.572984 0.006173
6 0.086766 -1.352046 0.017869
1 0.119269 -2.437348 0.028877
7 -1.109055 -0.782600 0.015590
7 -0.183287 1.369220 -0.001501
8 -2.454509 1.077533 -0.003172
8 2.080108 1.658241 -0.002185
1 -0.309529 2.381166 -0.009170
6 2.563807 -1.257653 0.011545
1 2.585923 -1.918231 0.905476
1 2.571049 -1.984806 -0.826815
8 3.730905 -0.483103 -0.028983
1 3.492047 0.466607 -0.017834
1 -1.953722 -1.360008 0.025549
8 -4.749308 -0.713960 0.047322
1 -4.477987 -1.423396 -0.552827
1 -4.018186 -0.071292 0.011813
rc_5hmU/rc_5hmUabe.xyz
6 -2.379603 -0.362245 0.091559
6 0.023669 -0.621259 -0.435400
6 0.221959 0.805210 -0.109061
6 -0.904607 1.546739 0.307958
1 -0.826150 2.597093 0.572620
7 -2.102198 0.996797 0.389227
7 -1.283456 -1.083674 -0.293704
8 -3.511092 -0.799863 0.190051
8 0.907890 -1.378909 -0.818882

1 -1.447393 -2.064355 -0.519361
6 1.514743 1.541238 -0.198641
1 1.362603 2.407577 -0.862894
1 1.666409 1.997917 0.804427
8 2.657705 0.856022 -0.621889
1 2.846270 0.099774 -0.010127
1 -2.904962 1.557608 0.682263
8 3.145226 -1.182169 1.121844
1 4.023798 -1.571394 0.995818
1 2.524200 -1.834827 0.761116
rc_5hmU/rc_5hmUabo.xyz
6 2.673805 0.037986 -0.152473
6 0.383454 0.909216 0.071777
6 -0.107086 -0.473225 0.178994
6 0.828222 -1.519644 0.069510
1 0.527861 -2.562223 0.110186
7 2.116465 -1.264500 -0.075028
7 1.752837 1.051363 -0.074827
8 3.872916 0.193182 -0.279675
8 -0.361491 1.890861 0.102133
1 2.120192 2.000413 -0.140922
6 -1.536873 -0.817157 0.328424
1 -1.829086 -1.314508 -0.624736
1 -1.642476 -1.613149 1.083852
8 -2.439183 0.217830 0.627650
1 -2.038927 1.079819 0.395295
1 2.787531 -2.032911 -0.139906
8 -4.918502 -0.142921 -0.738468
1 -4.083315 -0.011365 -0.248043
1 -5.605948 -0.148788 -0.057253
rc_5mC/rc_5mCaaf.xyz
6 -0.263110 1.649673 -0.008684
6 0.069370 -0.747572 -0.023503
6 1.502372 -0.521860 0.002011
6 1.915219 0.837051 0.020193
1 2.982796 1.040472 0.039569

7 1.112194 1.878938 0.014711
7 -0.733727 0.340309 -0.029539
8 -1.065837 2.581829 -0.012021
7 -0.465842 -1.948692 -0.040366
1 -1.485885 -2.046107 -0.048883
1 0.113261 -2.781329 -0.036694
1 -1.754209 0.189910 -0.036221
6 2.465530 -1.646642 0.017034
1 2.304467 -2.276838 0.903139
1 2.319897 -2.292913 -0.859546
1 3.492616 -1.280779 0.023798
8 -3.180700 -1.055965 0.031880
1 -3.673280 -1.048441 0.866423
1 -3.852028 -1.028675 -0.666403
rc_5mC/rc_5mCaco.xyz
6 -0.633899 -1.429373 0.004542
6 0.314867 0.788869 0.008704
6 1.639454 0.195401 -0.001390
6 1.681062 -1.223723 -0.010952
1 2.656450 -1.702573 -0.020797
7 0.630999 -2.015341 -0.009176
7 -0.746904 -0.041961 0.013420
8 -1.654924 -2.116877 0.008257
7 0.132750 2.093204 0.011707
1 -0.803201 2.491081 0.017553
1 0.922359 2.729528 0.006896
1 -1.710170 0.362903 0.014053
6 2.865170 1.024668 -0.003154
1 2.886727 1.681493 -0.884314
1 2.890830 1.680499 0.878448
1 3.757572 0.397725 -0.005903
8 -3.158385 1.332310 -0.002902
1 -3.635811 1.198604 -0.836109
1 -3.776114 1.050907 0.689181
rc_5mC/rc_5mCadd.xyz
6 0.638874 -1.425175 -0.002998

6 -0.318548 0.789392 -0.008094
6 -1.640869 0.190852 0.001210
6 -1.676792 -1.228445 0.010165
1 -2.650250 -1.711201 0.018837
7 -0.623722 -2.016030 0.008939
7 0.746318 -0.037403 -0.011213
8 1.662526 -2.108799 -0.005859
7 -0.140856 2.094341 -0.012102
1 0.793728 2.495359 -0.018900
1 -0.932408 2.728207 -0.008319
1 1.708046 0.371575 -0.010879
6 -2.870078 1.015043 0.002318
1 -2.894570 1.671858 0.883416
1 -2.897835 1.670770 -0.879315
1 -3.759862 0.384373 0.004659
8 3.159209 1.335877 -0.008846
1 3.596613 1.269681 0.853940
1 3.794963 0.966389 -0.640778
rc_5mC/rc_5mCabj.xyz
6 -0.659583 1.406807 -0.016079
6 0.334364 -0.791704 -0.019525
6 1.646485 -0.171040 0.006906
6 1.657920 1.248615 0.027593
1 2.622882 1.747610 0.052355
7 0.592165 2.018686 0.019308
7 -0.743449 0.017394 -0.038742
8 -1.694431 2.073277 -0.025986
7 0.178170 -2.099723 -0.021030
1 -0.748185 -2.518097 -0.031871
1 0.979262 -2.721061 -0.003836
1 -1.701331 -0.403376 -0.043537
6 2.890596 -0.973403 0.014382
1 2.922557 -1.627841 0.897156
1 2.933202 -1.630313 -0.865642
1 3.768780 -0.326639 0.019707
8 -3.219782 -1.232991 -0.050237

1 -3.101597 -2.183695 0.100051
1 -3.768768 -0.929025 0.688986
rc_5mC/rc_5mCacc.xyz
6 0.573259 -1.680104 0.001491
6 -0.233028 0.609499 -0.002515
6 1.145760 1.060875 0.000894
6 2.135688 0.042356 -0.004016
1 3.178266 0.348424 -0.007151
7 1.897937 -1.251241 -0.005211
7 -0.446435 -0.729265 0.002691
8 0.285375 -2.875252 0.006017
7 -1.251696 1.434764 -0.010708
1 -2.223334 1.068091 -0.015465
1 -1.095599 2.437174 -0.014300
1 -1.402150 -1.086411 0.007627
6 1.486358 2.501646 0.008523
1 1.059022 3.006097 -0.869080
1 1.062108 2.994716 0.894104
1 2.567679 2.644273 0.007511
8 -3.747304 0.137732 -0.014415
1 -3.800167 -0.345299 0.824744
1 -3.697263 -0.552338 -0.694458
rc_5mC/rc_5mCaau.xyz
6 0.590241 1.469199 -0.003686
6 -0.276385 -0.782435 -0.003945
6 -1.621539 -0.238277 0.001064
6 -1.715739 1.178281 0.006299
1 -2.708751 1.619744 0.012304
7 -0.695509 2.008176 0.005283
7 0.754149 0.086273 -0.009128
8 1.585603 2.192957 -0.006462
7 -0.045736 -2.079253 -0.002596
1 0.905038 -2.440407 -0.001257
1 -0.811075 -2.744729 0.000756
1 1.730211 -0.285006 -0.014142
6 -2.814588 -1.113109 0.001577

1 -2.816166 -1.767648 -0.881248
1 -2.813172 -1.771175 0.881800
1 -3.729841 -0.519891 0.004390
8 3.244983 -1.165018 -0.006369
1 3.304427 -1.652322 0.830009
1 3.232365 -1.850412 -0.692729
rc_5mC/rc_5mCaaw.xyz
6 2.231591 -0.364609 -0.003632
6 -0.195732 -0.524588 0.011839
6 -0.255450 0.926131 0.009658
6 0.994727 1.600941 -0.012194
1 0.980780 2.687632 -0.022470
7 2.177021 1.025824 -0.020377
7 1.038841 -1.087391 0.009709
8 3.302383 -0.968180 -0.002457
7 -1.269710 -1.278103 0.015792
1 -1.172894 -2.291067 0.015582
1 -2.234733 -0.897099 0.003340
1 1.129907 -2.103241 0.013109
6 -1.541354 1.660145 0.029160
1 -2.150335 1.395786 -0.845958
1 -2.129464 1.383724 0.914809
1 -1.371976 2.737608 0.034074
8 -3.979488 -0.589308 -0.123156
1 -4.483782 -1.172615 0.464054
1 -4.176416 0.308751 0.183507
rc_5mC/rc_5mCaam.xyz
6 -2.226461 -0.395096 0.009507
6 0.203336 -0.501683 -0.022093
6 0.231416 0.949971 -0.017060
6 -1.033147 1.597170 0.016700
1 -1.042456 2.683843 0.030111
7 -2.202419 0.996093 0.033524
7 -1.018011 -1.091528 -0.014669
8 -3.283485 -1.022465 0.011198
7 1.292535 -1.232859 -0.031401

1 1.217150 -2.247582 -0.031405
1 2.250087 -0.834348 -0.023357
1 -1.086968 -2.108899 -0.021002
6 1.501272 1.710609 -0.038315
1 2.116189 1.458803 0.836009
1 2.095664 1.446176 -0.922687
1 1.308610 2.784285 -0.042911
8 4.002440 -0.502974 0.035709
1 4.368363 -0.854913 0.861550
1 4.458487 -0.991625 -0.666177
rc_5mC/rc_5mCabg.xyz
6 -2.230740 -0.370109 -0.003678
6 0.197033 -0.519709 0.008311
6 0.250811 0.931083 0.003443
6 -1.002075 1.600752 -0.013879
1 -0.992703 2.687487 -0.023421
7 -2.181964 1.020597 -0.018657
7 -1.034970 -1.087991 0.005921
8 -3.299057 -0.978041 -0.000465
7 1.274315 -1.268185 0.013815
1 1.182235 -2.281603 0.016761
1 2.238160 -0.883771 0.013551
1 -1.121469 -2.104201 0.011747
6 1.534206 1.669461 0.014309
1 2.127790 1.399157 0.897997
1 2.139659 1.403376 -0.862825
1 1.361123 2.746390 0.015527
8 3.992441 -0.617931 0.072500
1 4.439797 -1.045951 -0.673160
1 4.181266 0.327060 -0.031058
rc_5mC/rc_5mCads.xyz
6 -2.232541 -0.356173 -0.002498
6 0.194086 -0.531873 -0.001210
6 0.262562 0.918493 0.002872
6 -0.983501 1.601239 0.009796
1 -0.962794 2.687870 0.016447

7 -2.169495 1.033892 0.007786
7 -1.044280 -1.086431 -0.006400
8 -3.307027 -0.953182 -0.007123
7 1.263641 -1.292040 -0.001273
1 1.160447 -2.304332 -0.004638
1 2.230736 -0.915153 0.007697
1 -1.142414 -2.101623 -0.009673
6 1.552592 1.645063 -0.005605
1 2.165381 1.358634 0.859073
1 2.133903 1.384088 -0.900526
1 1.389477 2.723354 0.011170
8 3.960951 -0.544365 0.095477
1 4.172352 0.183550 -0.508668
1 4.513259 -1.284457 -0.198636
rc_5mC/rc_5mCabe.xyz
6 2.231655 0.339794 -0.006251
6 -0.192767 0.541164 0.015107
6 -0.276581 -0.908454 0.009334
6 0.961964 -1.604442 -0.022637
1 0.929559 -2.690717 -0.039431
7 2.153821 -1.049478 -0.031318
7 1.051368 1.082342 0.009202
8 3.312378 0.925436 -0.000914
7 -1.254456 1.311949 0.024970
1 -1.141229 2.323133 0.027655
1 -2.226322 0.944317 0.019592
1 1.160898 2.096336 0.017040
6 -1.574688 -1.620191 0.040451
1 -2.142607 -1.342019 0.938234
1 -2.194822 -1.335814 -0.820057
1 -1.423262 -2.700300 0.033160
8 -3.967083 0.652674 -0.024904
1 -4.129070 0.191865 -0.862581
1 -4.148133 -0.012594 0.656913
rc_5mC/rc_5mCada.xyz
6 2.230079 -0.355888 -0.011284

6 -0.196155 -0.528791 0.028815
6 -0.263645 0.921585 0.017458
6 0.982407 1.602726 -0.036388
1 0.961918 2.689036 -0.064243
7 2.167610 1.033785 -0.053213
7 1.041548 -1.084725 0.020180
8 3.304222 -0.953486 -0.006643
7 -1.265846 -1.288096 0.044945
1 -1.162738 -2.300417 0.050360
1 -2.234478 -0.913504 0.032422
1 1.138491 -2.099963 0.033161
6 -1.553171 1.648307 0.059418
1 -2.183075 1.369228 -0.795837
1 -2.117162 1.379732 0.962924
1 -1.389813 2.726669 0.047638
8 -3.982912 -0.657182 -0.015363
1 -4.236276 -0.513883 -0.940094
1 -4.147621 0.194065 0.418225
rc_5mC/rc_5mCaai.xyz
6 -0.869190 0.922569 -0.009004
6 0.767571 -0.864794 -0.003908
6 1.815915 0.138295 0.008067
6 1.380462 1.491335 0.010667
1 2.142010 2.266656 0.019520
7 0.127891 1.890876 0.003201
7 -0.510191 -0.424839 -0.009580
8 -2.061189 1.229055 -0.020038
7 1.022153 -2.153720 -0.008832
1 0.269863 -2.838663 -0.018293
1 1.975579 -2.501626 -0.005294
1 -1.278450 -1.096641 -0.017727
6 3.248375 -0.231882 0.012652
1 3.483138 -0.867947 0.877348
1 3.498110 -0.815707 -0.884782
1 3.878269 0.657774 0.043053
8 -4.014841 -0.929907 -0.076945

1 -3.435047 -0.148224 -0.022248
1 -4.462994 -0.968152 0.779926
rc_5mC/rc_5mCacp.xyz
6 0.723028 -1.645113 -0.008502
6 -0.271430 0.571487 -0.017422
6 1.066505 1.134358 0.008012
6 2.136227 0.199888 0.032189
1 3.149691 0.591582 0.057923
7 2.008109 -1.109104 0.025885
7 -0.370419 -0.780785 -0.025785
8 0.531166 -2.859226 -0.022616
7 -1.356816 1.307425 -0.029668
1 -2.295864 0.861675 -0.036919
1 -1.290202 2.319445 -0.025228
1 -1.290264 -1.220173 -0.047471
6 1.292294 2.598241 0.002078
1 0.858810 3.050224 -0.901153
1 0.796133 3.072929 0.859848
1 2.357967 2.827456 0.035804
8 -3.752811 -0.146007 -0.060664
1 -3.636564 -0.879455 0.563006
1 -4.522400 0.342259 0.269280
rc_5mC/rc_5mCadc.xyz
6 -0.867555 -0.897272 0.005604
6 0.794890 0.866816 0.000606
6 1.828402 -0.151453 -0.004420
6 1.373439 -1.498283 -0.003793
1 2.123907 -2.284420 -0.007347
7 0.115232 -1.879996 0.000773
7 -0.488562 0.444438 0.003908
8 -2.064211 -1.184779 0.011763
7 1.067185 2.152068 0.001744
1 0.324501 2.847321 0.005545
1 2.025395 2.486297 -0.000949
1 -1.247600 1.126218 0.006003
6 3.265841 0.199298 -0.007577

1 3.511945 0.820445 -0.880063
1 3.520514 0.790844 0.883238
1 3.883446 -0.699240 -0.024837
8 -4.064810 0.935243 0.034354
1 -4.887685 0.598848 -0.348067
1 -3.449340 0.179771 0.010059
rc_5mC/rc_5mCabz.xyz
6 0.872872 -0.961790 -0.016936
6 -0.725706 0.859922 -0.013086
6 -1.794983 -0.120901 0.011594
6 -1.388403 -1.482357 0.017732
1 -2.166057 -2.241356 0.034583
7 -0.144710 -1.908300 0.005123
7 0.542999 0.393803 -0.023198
8 2.057663 -1.294957 -0.032294
7 -0.955009 2.153772 -0.025184
1 -0.189224 2.823506 -0.044799
1 -1.901465 2.520505 -0.021078
1 1.326746 1.048632 -0.041347
6 -3.219796 0.277072 0.027897
1 -3.435781 0.907572 0.901658
1 -3.463823 0.877189 -0.860122
1 -3.867116 -0.600129 0.052927
8 3.886796 0.980491 -0.056864
1 4.154405 1.147637 0.858210
1 3.419769 0.125563 -0.027166
rc_5mC/rc_5mCadt.xyz
6 -0.867757 -0.907013 -0.006482
6 0.784739 0.866155 -0.000220
6 1.824071 -0.146214 0.005796
6 1.376690 -1.495516 0.005931
1 2.131501 -2.277458 0.010881
7 0.120635 -1.884185 0.000435
7 -0.496486 0.436916 -0.003694
8 -2.062658 -1.201815 -0.015483
7 1.050072 2.152869 -0.002053

1 0.303601 2.844108 -0.006941
1 2.006424 2.492478 0.000679
1 -1.259182 1.114783 -0.006095
6 3.259557 0.212295 0.008094
1 3.511573 0.798447 -0.887136
1 3.501822 0.841742 0.875605
1 3.882127 -0.682670 0.032324
8 -4.044189 0.934142 -0.059573
1 -4.761123 0.726185 0.556247
1 -3.445308 0.166333 -0.016646
rc_5mC/rc_5mCadb.xyz
6 -0.870852 -0.924584 0.010636
6 0.766531 0.863106 0.010284
6 1.814662 -0.139992 -0.007615
6 1.379024 -1.493021 -0.009657
1 2.140650 -2.268266 -0.019061
7 0.126427 -1.892752 -0.001382
7 -0.510976 0.422750 0.019298
8 -2.062978 -1.230066 0.014901
7 1.020767 2.152089 0.017152
1 0.268330 2.836814 0.029818
1 1.974061 2.500331 0.011811
1 -1.278854 1.094267 0.034429
6 3.246982 0.230639 -0.018610
1 3.477769 0.862336 -0.887656
1 3.500106 0.819337 0.874609
1 3.877221 -0.658807 -0.047308
8 -4.109191 0.861068 -0.003537
1 -3.462378 0.131868 0.001301
1 -3.591164 1.642603 -0.244565
rc_5mC/rc_5mCadp.xyz
6 -0.875296 0.956844 -0.000004
6 1.516561 0.546358 0.000011
6 1.242602 -0.880456 0.000013
6 -0.128635 -1.252216 0.000009
1 -0.371486 -2.311706 0.000005

7 -1.137575 -0.411085 0.000007
7 0.454635 1.382731 0.000007
8 -1.781801 1.782906 -0.000021
7 2.739399 1.026535 0.000011
1 2.912007 2.029329 -0.000005
1 3.545618 0.409649 0.000001
1 0.602442 2.392118 0.000004
6 2.332953 -1.879867 -0.000019
1 2.975451 -1.748435 -0.882076
1 2.975673 -1.748302 0.881852
1 1.931482 -2.893521 0.000107
8 -3.977151 -1.039454 0.000011
1 -4.405921 -0.171619 -0.000066
1 -3.017974 -0.836374 0.000022
rc_5mC/rc_5mCaan.xyz
6 -2.442640 -0.191050 -0.086111
6 -0.280605 0.903131 0.063104
6 0.340431 -0.407740 0.122106
6 -0.545543 -1.518314 0.094194
1 -0.110220 -2.512459 0.152077
7 -1.855021 -1.449297 -0.002162
7 -1.626533 0.942951 -0.046957
8 -3.657958 -0.049695 -0.191952
7 0.416604 2.017209 0.112061
1 -0.038132 2.926059 0.071940
1 1.427907 1.998198 0.191702
1 -2.106166 1.841815 -0.098429
6 1.810175 -0.568819 0.193092
1 2.293689 -0.076985 -0.661663
1 2.209784 -0.089745 1.098551
1 2.084903 -1.623757 0.199065
8 5.114650 -0.180548 -0.323208
1 4.685888 -0.278937 0.539635
1 6.062548 -0.221539 -0.130500
rc_5mU/rc_5mUaaq.xyz
6 -0.813022 -1.071308 -0.000976

6 1.601704 -0.596206 0.005772
6 1.286710 0.850789 -0.003562
6 -0.075435 1.226066 -0.010595
1 -0.373120 2.269767 -0.018301
7 -1.040388 0.326780 -0.008905
1 -2.062987 0.633038 -0.014514
7 0.505132 -1.447505 0.004533
8 -1.754553 -1.846213 -0.000080
8 2.748246 -1.032049 0.014010
1 0.692023 -2.449352 0.010981
6 2.381740 1.832191 -0.006440
1 3.017515 1.677440 0.877251
1 3.035497 1.653588 -0.871995
1 2.009987 2.856632 -0.023569
8 -3.600776 1.111728 -0.073958
1 -3.856433 1.575515 0.738933
1 -4.149208 0.311524 -0.093150
rc_5mU/rc_5mUaat.xyz
6 -0.808931 1.079145 -0.001385
6 1.603480 0.592209 0.004064
6 1.281524 -0.853240 -0.001627
6 -0.082425 -1.221525 -0.010121
1 -0.385398 -2.263701 -0.017275
7 -1.042873 -0.317562 -0.010462
1 -2.067106 -0.619325 -0.018740
7 0.511010 1.449065 0.002388
8 -1.747242 1.858012 0.002204
8 2.752182 1.022429 0.009931
1 0.702974 2.449965 0.007787
6 2.370935 -1.840993 -0.000464
1 3.022278 -1.673401 -0.870128
1 3.011049 -1.683247 0.879414
1 1.992696 -2.863216 -0.008515
8 -3.602621 -1.091527 -0.085790
1 -3.795037 -1.774601 0.575538
1 -4.164473 -0.337887 0.154869

rc_5mU/rc_5mUaau.xyz
6 -0.809653 1.077870 -0.006924
6 1.603221 0.592700 -0.000468
6 1.282279 -0.852976 -0.002581
6 -0.081519 -1.222338 -0.007920
1 -0.383613 -2.264757 -0.008972
7 -1.042855 -0.319155 -0.010750
1 -2.066380 -0.623152 -0.009012
7 0.510108 1.448569 -0.004266
8 -1.747860 1.856810 -0.005807
8 2.751575 1.023838 0.004962
1 0.701250 2.449632 -0.001243
6 2.372439 -1.839739 0.007825
1 3.042804 -1.661361 -0.844582
1 2.992759 -1.691753 0.903961
1 1.995555 -2.862158 -0.018065
8 -3.586061 -1.147709 0.050265
1 -3.993221 -1.131033 -0.830133
1 -4.101757 -0.517930 0.578207
rc_5mU/rc_5mUach.xyz
6 0.698125 -1.334333 -0.005946
6 -0.300697 0.899695 -0.016137
6 -1.649001 0.280376 -0.002218
6 -1.725144 -1.128994 0.002882
1 -2.674971 -1.653069 0.009116
7 -0.626257 -1.862334 -0.000294
1 -0.691889 -2.882438 0.003214
7 0.775319 0.027532 -0.015124
8 1.651488 -2.093869 -0.002597
8 -0.137346 2.116544 -0.025442
1 1.733004 0.451113 -0.011341
6 -2.843781 1.132994 0.021079
1 -2.846672 1.728631 0.947043
1 -2.798179 1.866779 -0.794885
1 -3.761907 0.548650 -0.040483
8 3.274523 1.233469 0.004544

1 3.361394 1.701031 0.849707
1 3.249442 1.935334 -0.664452
rc_5mU/rc_5mUadt.xyz
6 0.748503 1.280441 -0.036898
6 -0.352944 -0.904674 -0.065110
6 -1.669413 -0.225025 0.006981
6 -1.678633 1.185859 0.055112
1 -2.602404 1.752422 0.108839
7 -0.547852 1.868738 0.033136
1 -0.565185 2.889984 0.067493
7 0.760600 -0.082078 -0.094595
8 1.737109 1.993900 -0.041550
8 -0.245162 -2.127457 -0.092553
1 1.700503 -0.543473 -0.090697
6 -2.901433 -1.022877 0.052778
1 -2.896699 -1.769979 -0.751406
1 -2.920107 -1.603176 0.988999
1 -3.793102 -0.398327 -0.003864
8 3.223584 -1.350823 0.006485
1 3.115711 -2.081890 0.634462
1 3.871326 -0.759484 0.420147
rc_5mU/rc_5mUaak.xyz
6 -0.672007 -1.367575 -0.016725
6 0.269746 0.891622 -0.021861
6 1.632698 0.307440 0.013968
6 1.744812 -1.099461 0.025971
1 2.708010 -1.597994 0.047635
7 0.665370 -1.861011 0.011542
1 0.756851 -2.879286 0.019802
7 -0.783464 -0.008429 -0.031207
8 -1.606023 -2.150733 -0.025501
8 0.074169 2.103642 -0.041133
1 -1.748595 0.394891 -0.035462
6 2.804705 1.191117 0.024642
1 2.814781 1.795432 -0.895292
1 2.718735 1.916066 0.845715

1 3.736229 0.631134 0.106932
8 -3.238946 1.285439 -0.041304
1 -2.955493 2.213151 -0.037876
1 -3.717189 1.167050 0.793752
rc_5mU/rc_5mUabn.xyz
6 -0.798433 -1.206354 -0.041807
6 0.417889 0.917524 -0.055468
6 1.696027 0.166489 0.010042
6 1.629638 -1.243626 0.043926
1 2.522485 -1.858433 0.090809
7 0.464045 -1.864448 0.015101
1 0.423561 -2.885573 0.039135
7 -0.738498 0.155615 -0.089319
8 -1.823019 -1.867090 -0.046279
8 0.377405 2.144318 -0.076483
1 -1.658197 0.654935 -0.066561
6 2.969358 0.898254 0.056383
1 3.014518 1.621650 -0.768860
1 3.006271 1.503730 0.975337
1 3.827935 0.227479 0.025286
8 -3.219944 1.351472 0.180807
1 -3.529180 1.856379 -0.586783
1 -3.848624 0.618334 0.268349
rc_5mU/rc_5mUadp.xyz
6 0.775949 1.241932 -0.034451
6 -0.387483 -0.910777 -0.061582
6 -1.684043 -0.193005 0.006446
6 -1.652896 1.217852 0.053237
1 -2.560445 1.810345 0.104402
7 -0.502903 1.867555 0.032263
1 -0.488884 2.888964 0.064821
7 0.749573 -0.120625 -0.091374
8 1.783910 1.927766 -0.038059
8 -0.316333 -2.136189 -0.086530
1 1.678698 -0.603296 -0.083761
6 -2.938122 -0.957035 0.047947

1 -2.951983 -1.700120 -0.759990
1 -2.973532 -1.541316 0.981097
1 -3.812739 -0.308928 -0.007866
8 3.222871 -1.371540 0.013842
1 3.900741 -0.680581 0.074581
1 3.277447 -1.847668 0.856891
rc_5mU/rc_5mUaab.xyz
6 2.067745 -0.509259 0.130495
6 -0.323869 -0.478897 -0.447831
6 -0.342773 0.983242 -0.208033
6 0.862557 1.598673 0.198886
1 0.921105 2.663729 0.398435
7 1.967662 0.891044 0.352187
1 2.833076 1.348584 0.648635
7 0.901233 -1.104368 -0.272767
8 3.124609 -1.085268 0.301109
8 -1.320398 -1.115008 -0.776599
1 0.943268 -2.109700 -0.439608
6 -1.593240 1.735653 -0.365434
1 -2.121175 1.418539 -1.271866
1 -2.265814 1.480783 0.471446
1 -1.421063 2.812353 -0.362601
8 -3.816667 -0.757295 0.804746
1 -3.024798 -0.833491 0.245525
1 -3.869727 -1.603440 1.271560
rc_5mU/rc_5mUaca.xyz
6 0.706509 -1.608012 0.025569
6 -0.396285 0.589973 0.071497
6 0.933761 1.237769 -0.016752
6 2.069514 0.401152 -0.087647
1 3.074226 0.804822 -0.158247
7 1.944273 -0.914059 -0.069401
1 2.774764 -1.508934 -0.123339
7 -0.395462 -0.796362 0.091696
8 0.682789 -2.823257 0.043038
8 -1.440308 1.233146 0.124163

1 -1.300020 -1.265791 0.149582
6 1.029653 2.702509 -0.013763
1 0.449098 3.112409 -0.853385
1 0.545298 3.099050 0.890755
1 2.063070 3.043720 -0.068754
8 -3.918333 -0.284714 -0.032730
1 -3.970662 -0.539529 -0.965066
1 -3.129548 0.285458 0.023197
rc_5mU/rc_5mUado.xyz
6 -0.891095 1.550335 0.015379
6 0.436819 -0.519287 0.084965
6 -0.816488 -1.303941 -0.003217
6 -2.034570 -0.591514 -0.074850
1 -2.990628 -1.100300 -0.140095
7 -2.049417 0.729605 -0.063222
1 -2.938734 1.232675 -0.112456
7 0.289909 0.858530 0.089642
8 -0.994316 2.761372 0.013874
8 1.543068 -1.047290 0.146516
1 1.139085 1.421738 0.143127
6 -0.752936 -2.770150 -0.028034
1 -0.188036 -3.092882 -0.916579
1 -0.172133 -3.132950 0.831268
1 -1.745174 -3.220977 -0.037708
8 3.921075 0.603795 -0.101046
1 3.156446 0.007379 -0.002090
1 4.646731 0.032704 -0.390616
rc_5mU/rc_5mUaai.xyz
6 -2.456490 0.026275 -0.075417
6 -0.209552 -0.969648 0.064961
6 0.368730 0.395648 0.109071
6 -0.521533 1.491496 0.066843
1 -0.169704 2.517386 0.095354
7 -1.826749 1.300155 -0.017652
1 -2.461133 2.101560 -0.052277
7 -1.593696 -1.034999 -0.034543

8 -3.667326 -0.056812 -0.156939
8 0.476249 -1.983497 0.108240
1 -2.018003 -1.961534 -0.066144
6 1.826122 0.558050 0.183210
1 2.202854 0.080016 1.099790
1 2.300082 0.007896 -0.641284
1 2.123631 1.605812 0.160579
8 5.228494 0.124141 -0.175405
1 5.689204 0.003298 -1.018554
1 5.933189 0.107889 0.488727
rc_5mU/rc_5mUacv.xyz
6 -0.714630 1.606224 -0.026097
6 0.396347 -0.587728 -0.075424
6 -0.931478 -1.240432 0.012258
6 -2.070093 -0.408023 0.086191
1 -3.073213 -0.815451 0.157830
7 -1.949721 0.907622 0.069425
1 -2.782283 1.499378 0.125567
7 0.390208 0.798727 -0.094921
8 -0.695499 2.821592 -0.041961
8 1.442847 -1.226719 -0.127599
1 1.292853 1.271925 -0.150647
6 -1.022497 -2.705539 0.011159
1 -0.521914 -3.103964 -0.883265
1 -0.454684 -3.109960 0.862454
1 -2.055538 -3.049812 0.052279
8 3.868559 0.333252 0.225599
1 3.135705 -0.273480 0.014751
1 4.502517 0.224900 -0.497335
rc_5mU/rc_5mUaba.xyz
6 -1.176297 -0.104194 -0.824382
6 0.993169 0.893802 -0.224927
6 1.487097 -0.456080 0.138605
6 0.610028 -1.549441 -0.035076
1 0.909806 -2.566385 0.195852
7 -0.618696 -1.367586 -0.486559

1 -1.247846 -2.166052 -0.597725
7 -0.316211 0.951474 -0.684406
8 -2.325554 -0.024560 -1.215251
8 1.683263 1.901717 -0.133326
1 -0.687815 1.869028 -0.928212
6 2.852834 -0.612873 0.653676
1 3.570672 -0.215894 -0.079252
1 2.981197 0.011011 1.550132
1 3.084773 -1.652858 0.882159
8 -2.542365 0.251431 2.020139
1 -2.174306 1.086367 2.343907
1 -3.015869 0.491585 1.210025
rc_5mU/rc_5mUacy.xyz
6 0.727804 -1.602533 -0.022923
6 -0.401377 0.581919 -0.074819
6 0.920742 1.245973 0.012834
6 2.066308 0.423248 0.086181
1 3.065978 0.839280 0.156421
7 1.957007 -0.893326 0.070314
1 2.794736 -1.477920 0.124999
7 -0.383910 -0.804312 -0.092186
8 0.718902 -2.817997 -0.036560
8 -1.453250 1.212112 -0.128642
1 -1.282834 -1.284724 -0.147640
6 0.999828 2.711754 0.014914
1 0.481317 3.110115 -0.868942
1 0.442743 3.107334 0.877785
1 2.030537 3.064170 0.042415
8 -3.912666 -0.330798 0.029605
1 -4.118199 -0.391635 0.973474
1 -3.129676 0.248145 -0.017759
rc_5mU/rc_5mUacn.xyz
6 -1.799762 -0.061760 -0.071574
6 0.451997 -1.053440 -0.179435
6 0.991221 0.272595 -0.561239
6 0.077001 1.336796 -0.706806

1 0.399638 2.329990 -1.000660
7 -1.215276 1.161883 -0.482490
1 -1.864249 1.944033 -0.584737
7 -0.919494 -1.105594 0.031277
8 -2.995191 -0.133742 0.151080
8 1.153479 -2.050549 -0.054943
1 -1.316937 -1.999332 0.317227
6 2.430940 0.421174 -0.833923
1 3.018160 -0.091422 -0.065484
1 2.666974 -0.078355 -1.786729
1 2.719914 1.470275 -0.904800
8 0.997292 0.782306 2.053333
1 1.462448 1.622113 1.915123
1 1.704417 0.132359 2.190650
rc_5mU/rc_5mUabc.xyz
6 2.360524 -0.396499 -0.193588
6 0.463828 1.155780 0.039456
6 -0.414280 0.001878 0.348255
6 0.162986 -1.287171 0.343648
1 -0.422793 -2.177000 0.550560
7 1.449053 -1.451581 0.087755
1 1.856644 -2.389684 0.088414
7 1.798021 0.850972 -0.200215
8 3.533278 -0.639485 -0.405134
8 0.054672 2.309483 -0.003631
1 2.424894 1.627835 -0.408205
6 -1.836027 0.230122 0.634692
1 -1.942620 1.005892 1.404633
1 -2.325799 0.641634 -0.262428
1 -2.342513 -0.686216 0.939825
8 -5.363981 -0.645935 -0.525483
1 -5.592127 0.116823 -1.076573
1 -4.599138 -0.352190 -0.009797
rc_5mU/rc_5mUack.xyz
6 -0.716142 -1.606407 0.022839
6 0.396459 0.586694 0.068520

6 -0.931482 1.240539 -0.009523
6 -2.070972 0.408984 -0.078700
1 -3.074247 0.817121 -0.143565
7 -1.951272 -0.906761 -0.065263
1 -2.784577 -1.497870 -0.117170
7 0.389536 -0.799657 0.088241
8 -0.697693 -2.821791 0.036089
8 1.443861 1.224872 0.112940
1 1.292580 -1.273209 0.135206
6 -1.021614 2.705705 -0.005806
1 -0.504788 3.103934 0.879199
1 -0.469112 3.109650 -0.867564
1 -2.055056 3.050371 -0.029314
8 3.891130 -0.309519 -0.222888
1 3.132517 0.271329 -0.030353
1 4.358959 -0.397982 0.619608
rc_5mU/rc_5mUacm.xyz
6 -1.793759 0.067536 0.083276
6 0.463635 1.049842 0.172747
6 0.999418 -0.278574 0.551966
6 0.081343 -1.336721 0.713323
1 0.400718 -2.329447 1.012160
7 -1.212742 -1.156320 0.498329
1 -1.864884 -1.934279 0.611506
7 -0.908788 1.106031 -0.033304
8 -2.990531 0.144066 -0.131332
8 1.166318 2.046089 0.052397
1 -1.304349 2.001012 -0.317827
6 2.438934 -0.431449 0.822218
1 3.027329 0.092912 0.063147
1 2.672489 0.053088 1.783498
1 2.727301 -1.481677 0.879240
8 1.046477 -0.682765 -2.065122
1 0.384159 -1.379686 -2.192457
1 1.892424 -1.152817 -2.003178
rc_5mU/rc_5mUacu.xyz

6 -1.797761 0.067744 0.077563
6 0.460316 1.046762 0.178257
6 0.993326 -0.282611 0.557971
6 0.074191 -1.342200 0.704300
1 0.392018 -2.337358 0.996716
7 -1.218357 -1.160342 0.483040
1 -1.870933 -1.939457 0.585458
7 -0.911764 1.106111 -0.029004
8 -2.994110 0.146752 -0.138103
8 1.165126 2.041804 0.059653
1 -1.305597 2.003057 -0.309653
6 2.429743 -0.435781 0.845197
1 3.027792 0.102615 0.103724
1 2.647509 0.033518 1.817860
1 2.720145 -1.486032 0.890126
8 1.040197 -0.679128 -2.076091
1 0.653386 -1.565566 -2.146264
1 1.997923 -0.830065 -2.059618
rc_C/rc_Caar.xyz
6 1.149073 -1.065076 0.004069
6 -0.345410 0.841774 0.018917
6 0.816611 1.686967 0.001510
6 2.083934 1.072734 -0.026321
1 2.970612 1.698993 -0.048042
7 2.272287 -0.233565 -0.027568
7 -0.124611 -0.495587 0.022325
8 1.264316 -2.287439 0.015819
7 -1.564479 1.324711 0.028978
1 -2.385659 0.684398 0.026777
1 -1.698150 2.332025 0.026315
1 0.699330 2.763984 0.002686
1 -0.914581 -1.141263 0.041398
8 -3.541411 -0.630000 -0.016999
1 -3.318877 -1.284070 0.663609
1 -3.443547 -1.101853 -0.858495
rc_C/rc_Caab.xyz

6 -0.823534 1.154581 0.011146
6 0.235198 -1.011792 0.017969
6 -1.075054 -1.603615 -0.009650
6 -2.186524 -0.739138 -0.028209
1 -3.184247 -1.166667 -0.051807
7 -2.098144 0.578091 -0.018425
7 0.302676 0.336217 0.031668
8 -0.683177 2.375482 0.018853
7 1.330341 -1.740925 0.026712
1 2.248466 -1.291834 0.037432
1 1.261888 -2.753917 0.018072
1 -1.184388 -2.681543 -0.016827
1 1.238074 0.779571 0.034167
8 3.059012 0.629315 -0.031424
1 3.443223 0.993615 -0.843192
1 3.525674 1.068498 0.695501
rc_C/rc_Cadd.xyz
6 -0.192372 -1.245327 0.014441
6 -0.294462 1.157443 0.009292
6 -1.729703 1.052658 -0.006111
6 -2.294670 -0.237435 -0.017847
1 -3.375530 -0.338158 -0.034816
7 -1.586922 -1.351371 -0.007949
7 0.411655 0.010664 0.025005
8 0.513274 -2.252431 0.023069
7 0.307909 2.328744 0.006293
1 1.322076 2.403603 0.011984
1 -0.241139 3.183364 -0.010255
1 -2.341134 1.947164 -0.014137
1 1.461515 0.026151 0.013830
8 3.154380 0.000165 -0.116125
1 3.560808 0.446811 0.642480
1 3.410919 -0.931099 -0.026735
rc_C/rc_Cacu.xyz
6 -0.823580 -1.154567 0.011201
6 0.235229 1.011768 0.018051

6 -1.074999 1.603638 -0.009683
6 -2.186498 0.739201 -0.028338
1 -3.184203 1.166765 -0.052038
7 -2.098165 -0.578030 -0.018519
7 0.302659 -0.336242 0.031841
8 -0.683268 -2.375472 0.018925
7 1.330396 1.740865 0.026826
1 2.248504 1.291743 0.037521
1 1.261979 2.753858 0.018082
1 -1.184296 2.681570 -0.016878
1 1.238040 -0.779630 0.034303
8 3.058999 -0.629344 -0.031572
1 3.525874 -1.068597 0.695188
1 3.443113 -0.993573 -0.843430
rc_C/rc_Caaw.xyz
6 -1.200566 1.050840 0.002565
6 0.348708 -0.812032 -0.001486
6 -0.788699 -1.690440 -0.008072
6 -2.073440 -1.113057 -0.005388
1 -2.942044 -1.764540 -0.007894
7 -2.299103 0.187347 0.000517
7 0.089513 0.518471 0.000165
8 -1.351684 2.269267 0.006283
7 1.579839 -1.261978 0.003801
1 2.390702 -0.607150 0.014340
1 1.739745 -2.265507 0.000756
1 -0.640111 -2.763587 -0.012709
1 0.858206 1.188870 0.001571
8 3.556043 0.686002 0.093211
1 4.408140 0.430059 -0.292042
1 3.242731 1.420964 -0.457070
rc_C/rc_Cacq.xyz
6 -0.583748 1.217308 0.002691
6 0.045707 -1.105801 0.000647
6 -1.353121 -1.440437 -0.003754
6 -2.282241 -0.382439 -0.004353

1 -3.342885 -0.614433 -0.007611
7 -1.944945 0.893911 -0.001374
7 0.371479 0.202147 0.003603
8 -0.217209 2.390544 0.005448
7 0.976736 -2.037468 0.002118
1 1.964675 -1.794117 0.006237
1 0.713502 -3.018298 -0.000439
1 -1.662355 -2.478887 -0.006577
1 1.380812 0.473646 0.005605
8 3.119641 0.610366 0.010362
1 3.467438 0.256484 -0.822896
1 3.476888 0.026410 0.697389
rc_C/rc_Cact.xyz
6 0.464400 1.239680 0.003771
6 0.055608 -1.130931 -0.001600
6 1.479575 -1.335726 -0.001390
6 2.304756 -0.195086 0.000006
1 3.382468 -0.326487 -0.000976
7 1.850088 1.044031 0.002261
7 -0.391025 0.140204 0.003135
8 -0.010003 2.373947 0.005457
7 -0.782885 -2.145873 -0.006466
1 -1.789464 -1.997568 -0.010839
1 -0.428954 -3.097705 -0.009098
1 1.883783 -2.340887 -0.003358
1 -1.422134 0.322707 0.002610
8 -3.137539 0.286620 -0.040093
1 -3.492464 1.032976 -0.547075
1 -3.512169 0.386278 0.848583
rc_C/rc_Caao.xyz
6 -1.989874 0.238313 0.011861
6 0.424473 0.443104 -0.017424
6 0.493953 -0.993858 -0.031191
6 -0.714853 -1.716098 -0.019543
1 -0.678301 -2.801206 -0.027147
7 -1.910346 -1.156948 0.001593

7 -0.814539 0.991974 0.005189
8 -3.074750 0.812605 0.026770
7 1.512659 1.174815 -0.024152
1 1.454355 2.190528 -0.013362
1 2.445811 0.710741 -0.028446
1 1.459037 -1.484911 -0.049733
1 -0.924867 2.005844 0.013967
8 3.907321 -0.227501 -0.027147
1 4.668241 0.301511 -0.311662
1 4.118546 -0.500990 0.878777
rc_C/rc_Caay.xyz
6 1.992370 0.245452 -0.003685
6 -0.423655 0.432612 0.004412
6 -0.482668 -1.004744 0.010564
6 0.731465 -1.718126 0.007952
1 0.702526 -2.803494 0.011493
7 1.923007 -1.150310 0.000932
7 0.811472 0.990537 -0.003090
8 3.073079 0.827740 -0.008237
7 -1.516601 1.157200 0.005201
1 -1.464171 2.173278 0.000183
1 -2.448377 0.690052 0.006479
1 -1.443970 -1.503235 0.016947
1 0.914351 2.005209 -0.006512
8 -3.919552 -0.229984 0.015728
1 -4.567121 0.153673 0.626678
1 -4.351667 -0.210681 -0.851965
rc_C/rc_Caad.xyz
6 -1.187954 -1.054708 -0.005801
6 0.348751 0.818431 -0.011276
6 -0.794410 1.689290 -0.001943
6 -2.075195 1.103353 0.015064
1 -2.948032 1.748995 0.029326
7 -2.292203 -0.198523 0.015139
7 0.098250 -0.513729 -0.015928
8 -1.331072 -2.274138 -0.015175

7 1.577401 1.275369 -0.013105
1 2.390952 0.623293 -0.005091
1 1.731335 2.279832 -0.009901
1 -0.652973 2.763410 -0.001902
1 0.872527 -1.177691 -0.032687
8 3.594703 -0.625403 0.074470
1 3.860245 -0.906449 -0.814488
1 3.205607 -1.415066 0.481376
rc_C/rc_Cacy.xyz
6 -1.225957 -1.043280 0.002485
6 0.352784 0.794384 -0.001516
6 -0.770518 1.691177 0.002336
6 -2.064206 1.134334 0.003751
1 -2.922224 1.799743 0.004876
7 -2.310654 -0.162391 0.003604
7 0.072567 -0.531936 0.000142
8 -1.396739 -2.259118 0.003446
7 1.589943 1.226339 -0.007256
1 2.397218 0.565057 -0.014601
1 1.762978 2.227748 -0.008456
1 -0.604842 2.761845 0.002650
1 0.829344 -1.215579 -0.000529
8 3.640741 -0.637588 -0.082027
1 3.213147 -1.507463 -0.111841
1 4.156748 -0.641480 0.738773
rc_C/rc_Cabi.xyz
6 0.458893 -1.239391 0.004690
6 0.064190 1.133294 -0.001378
6 1.489302 1.329938 -0.006546
6 2.307878 0.184868 -0.006379
1 3.386323 0.310006 -0.010843
7 1.845588 -1.051413 -0.001310
7 -0.390582 -0.134733 0.005399
8 -0.021698 -2.370836 0.008545
7 -0.766532 2.154337 -0.002954
1 -1.773757 2.016956 -0.001963

1 -0.404969 3.103324 -0.007344
1 1.898737 2.332995 -0.010959
1 -1.422042 -0.312439 0.012321
8 -3.147860 -0.409717 0.080601
1 -3.495054 -0.896628 -0.682468
1 -3.573664 0.460618 0.037821
rc_C/rc_Cace.xyz
6 1.186459 1.058276 -0.002902
6 -0.343314 -0.820062 -0.005507
6 0.802827 -1.686964 0.001859
6 2.081615 -1.096676 0.009847
1 2.956777 -1.739274 0.017856
7 2.293938 0.205951 0.007898
7 -0.098023 0.512965 -0.006687
8 1.325472 2.278176 -0.008862
7 -1.570306 -1.282610 -0.010567
1 -2.384549 -0.633176 -0.013167
1 -1.720383 -2.287560 -0.008566
1 0.665074 -2.761561 0.003035
1 -0.876183 1.173046 -0.013666
8 -3.482013 0.724372 -0.002784
1 -3.963292 0.780457 0.836945
1 -4.169900 0.666090 -0.683558
rc_C/rc_Cabh.xyz
6 0.477588 1.236472 -0.009602
6 0.045603 -1.129954 -0.001792
6 1.467439 -1.348194 0.011617
6 2.303810 -0.215996 0.014837
1 3.380099 -0.358255 0.026423
7 1.861221 1.027443 0.004534
7 -0.389223 0.144878 -0.012256
8 0.014341 2.375073 -0.018427
7 -0.800138 -2.138670 -0.004697
1 -1.805718 -1.987580 -0.015704
1 -0.452359 -3.092779 0.002732
1 1.861407 -2.357390 0.020039

1 -1.417686 0.339614 -0.019669
8 -3.141054 0.461654 -0.052012
1 -3.481159 0.663277 0.833218
1 -3.540538 -0.390232 -0.286948
rc_C/rc_Cabc.xyz
6 1.986069 -0.225867 0.015221
6 -0.425718 -0.460675 -0.023196
6 -0.512483 0.975643 -0.036608
6 0.687334 1.712596 -0.018911
1 0.637542 2.797164 -0.025179
7 1.889540 1.168331 0.006510
7 0.820052 -0.993653 0.003533
8 3.077719 -0.787006 0.033415
7 -1.504799 -1.205928 -0.033822
1 -1.434463 -2.220913 -0.024222
1 -2.441620 -0.750423 -0.039903
1 -1.482739 1.456170 -0.059215
1 0.943648 -2.005978 0.012875
8 -3.903422 0.199334 -0.053885
1 -4.661752 -0.303525 0.280304
1 -3.799766 0.937458 0.566517
rc_C/rc_Cabm.xyz
6 0.418355 -0.889625 0.017720
6 -1.140052 0.957318 0.005254
6 -2.206411 -0.006026 -0.023270
6 -1.861859 -1.372041 -0.020489
1 -2.655416 -2.112964 -0.035939
7 -0.620841 -1.820377 -0.000667
7 0.121465 0.474917 0.030198
8 1.592463 -1.252967 0.024344
7 -1.381479 2.248037 0.007992
1 -0.628792 2.933138 0.027379
1 -2.341508 2.581561 -0.010204
1 -3.236414 0.329258 -0.044919
1 0.915280 1.115542 0.054586
8 3.688159 0.787210 -0.048037

1 4.553874 0.375452 0.085095
1 3.053779 0.048270 -0.024404
rc_C/rc_Cacz.xyz
6 -0.669860 0.325238 0.794422
6 1.460958 -0.480793 -0.017094
6 1.629678 0.784563 -0.678567
6 0.607432 1.743783 -0.544423
1 0.722392 2.703357 -1.039100
7 -0.494017 1.554861 0.156977
7 0.326658 -0.650968 0.695799
8 -1.685244 0.081932 1.439419
7 2.369044 -1.426564 -0.103929
1 2.255692 -2.323716 0.362519
1 3.214981 -1.264178 -0.643422
1 2.524171 0.972618 -1.260911
1 0.149493 -1.528994 1.183948
8 -2.798619 -1.027562 -1.314312
1 -2.927595 -0.685292 -0.417028
1 -3.649271 -0.886802 -1.754824
rc_C/rc_Cadq.xyz
6 0.417924 0.902689 0.005279
6 -1.116870 -0.963367 0.004831
6 -2.195895 -0.013784 -0.006269
6 -1.868633 1.356362 -0.004641
1 -2.671405 2.087424 -0.008993
7 -0.633203 1.819769 0.001589
7 0.139390 -0.465927 0.015705
8 1.586816 1.282813 -0.000203
7 -1.342700 -2.256918 0.004744
1 -0.581371 -2.932724 0.011654
1 -2.298518 -2.602729 -0.002937
1 -3.221669 -0.362285 -0.015036
1 0.940755 -1.097946 0.025951
8 3.648907 -0.768927 -0.083847
1 4.351897 -0.555553 0.546124
1 3.030954 -0.017135 -0.033830

rc_C/rc_Cack.xyz
6 -0.415074 -0.918467 -0.011356
6 1.091156 0.970809 -0.001003
6 2.184596 0.037706 0.015523
6 1.877928 -1.337233 0.018368
1 2.691443 -2.056197 0.031469
7 0.649762 -1.819076 0.006185
7 -0.157736 0.454648 -0.011923
8 -1.577602 -1.317589 -0.026995
7 1.296963 2.267767 -0.006343
1 0.525017 2.931322 -0.020659
1 2.246835 2.629539 0.000725
1 3.204971 0.401738 0.025880
1 -0.969274 1.074596 -0.025487
8 -3.619277 0.746187 -0.074908
1 -3.020043 -0.021050 -0.028448
1 -4.058476 0.771017 0.787115
rc_C/rc_Cadh.xyz
6 -0.543560 -0.337429 -0.129577
6 1.865623 -0.266424 0.051120
6 1.771157 1.168391 0.068675
6 0.491404 1.748215 -0.041559
1 0.403291 2.830194 -0.042928
7 -0.626784 1.054829 -0.141059
7 0.706247 -0.954477 -0.040028
8 -1.550844 -1.038479 -0.193268
7 3.024233 -0.880898 0.120794
1 3.094811 -1.895796 0.097726
1 3.881704 -0.338450 0.188200
1 2.668539 1.769327 0.156193
1 0.712191 -1.974496 -0.053563
8 -4.201493 0.057544 0.165417
1 -3.298957 -0.283537 0.019959
1 -4.676502 -0.672459 0.587314
rc_U/rc_U14_ack.xyz
6 0.292756 0.948590 -0.018701

6 -1.927100 -0.103869 0.015329
6 -1.259144 -1.405554 -0.015629
6 0.148167 -1.464156 -0.040551
1 0.686610 -2.405972 -0.053824
7 0.861128 -0.356692 -0.042004
1 1.936108 -0.403894 -0.034647
7 -1.077021 0.994202 0.012229
8 1.017359 1.927175 -0.026054
8 -3.149036 0.023175 0.041375
1 -1.501160 1.921192 0.025990
1 -1.860910 -2.305620 -0.013188
8 3.504726 -0.561996 0.028643
1 3.920581 0.040391 -0.608416
1 3.817573 -0.265563 0.898117
rc_U/rc_U14_aar.xyz
6 -0.296317 0.944514 -0.029569
6 1.926088 -0.100915 0.024953
6 1.262697 -1.404519 -0.019243
6 -0.143885 -1.467454 -0.059757
1 -0.679084 -2.411030 -0.080517
7 -0.860353 -0.362293 -0.065042
1 -1.936180 -0.408725 -0.059556
7 1.072809 0.994624 0.018361
8 -1.024528 1.920491 -0.041109
8 3.147326 0.029801 0.064491
1 1.493688 1.922941 0.039163
1 1.867393 -2.302602 -0.013356
8 -3.506858 -0.482708 -0.021991
1 -3.849262 0.358542 0.320367
1 -3.802761 -1.155874 0.611233
rc_U/rc_U14_abi.xyz
6 -0.304609 0.934754 -0.011772
6 1.928346 -0.089129 0.011937
6 1.276819 -1.399328 -0.014906
6 -0.129686 -1.476098 -0.033277
1 -0.656044 -2.424700 -0.045700

7 -0.856446 -0.377765 -0.031555
1 -1.931668 -0.432835 -0.026164
7 1.064628 0.998249 0.011134
8 -1.041922 1.903855 -0.016034
8 3.148722 0.053200 0.032661
1 1.476959 1.930525 0.023787
1 1.890046 -2.291662 -0.015853
8 -3.502773 -0.545889 -0.006834
1 -3.865554 0.216328 -0.485846
1 -3.828457 -0.451567 0.902484
rc_U/rc_Uabn.xyz
6 -0.277976 0.964297 -0.030795
6 1.924213 -0.124057 0.022924
6 1.235410 -1.414625 -0.010191
6 -0.172218 -1.450245 -0.047136
1 -0.725753 -2.383362 -0.060895
7 -0.867329 -0.331489 -0.057711
1 -1.943180 -0.363124 -0.052869
7 1.092032 0.987642 0.013155
8 -0.987032 1.954149 -0.044305
8 3.147859 -0.016753 0.056875
1 1.530831 1.907847 0.026966
1 1.822594 -2.324181 -0.000086
8 -3.515678 -0.437812 -0.021017
1 -3.820338 -1.347683 0.123153
1 -3.854840 0.068549 0.734381
rc_U/rc_U14_aak.xyz
6 -0.306290 0.932669 0.017356
6 1.927878 -0.087799 -0.016996
6 1.278524 -1.398915 0.015551
6 -0.127595 -1.477705 0.043422
1 -0.652427 -2.427117 0.060014
7 -0.855914 -0.380454 0.044719
1 -1.931603 -0.434778 0.042143
7 1.062639 0.998419 -0.013688
8 -1.045701 1.900255 0.022440

8 3.147942 0.056312 -0.044277
1 1.473478 1.931293 -0.029554
1 1.893122 -2.290303 0.013153
8 -3.501551 -0.500926 0.038046
1 -3.841672 0.404894 -0.039355
1 -3.828592 -0.964376 -0.749293
rc_U/rc_U14_abc.xyz
6 0.264222 0.979147 0.028743
6 -1.922143 -0.141898 -0.020416
6 -1.214387 -1.422109 0.010447
6 0.193764 -1.436718 0.043709
1 0.760961 -2.361650 0.055485
7 0.872720 -0.308082 0.053123
1 1.948225 -0.329117 0.045393
7 -1.106152 0.981877 -0.012934
8 0.958250 1.979406 0.041730
8 -3.147302 -0.052550 -0.051008
1 -1.558197 1.895748 -0.025500
1 -1.788104 -2.340212 0.001461
8 3.522507 -0.399444 -0.018083
1 3.815220 -0.477699 -0.939961
1 3.859556 -1.193460 0.425793
rc_U/rc_U14_abz.xyz
6 0.267771 -0.975735 0.035198
6 -1.922175 0.137283 -0.025335
6 -1.219347 1.420102 0.011756
6 0.188477 1.439959 0.053268
1 0.752307 2.366902 0.068309
7 0.871277 0.313635 0.065222
1 1.946557 0.336814 0.058164
7 -1.102273 -0.983602 -0.015112
8 0.965772 -1.973252 0.050681
8 -3.146807 0.043548 -0.063124
1 -1.550957 -1.899052 -0.030799
1 -1.796425 2.336072 0.000242
8 3.522786 0.416540 -0.005347

1 3.822522 1.326342 0.149240
1 3.820607 0.198353 -0.902936
rc_U/rc_U14_aal.xyz
6 0.028232 -1.153292 0.005757
6 0.313051 1.272369 -0.001716
6 1.765247 1.069903 -0.004548
6 2.286703 -0.238557 0.000526
1 3.351593 -0.445878 -0.000635
7 1.457775 -1.263846 0.006079
1 1.820100 -2.221313 0.009047
7 -0.457040 0.120903 0.004839
8 -0.645507 -2.167141 0.006147
8 -0.195541 2.392654 -0.004442
1 -1.502385 0.211552 0.000565
1 2.418433 1.933688 -0.011159
8 -3.211212 0.172175 -0.081315
1 -3.592732 0.398671 0.780979
1 -3.441469 -0.760175 -0.218464
rc_U/rc_Uacq.xyz
6 -0.311372 1.191081 -0.004820
6 -0.017200 -1.234121 0.002882
6 -1.475637 -1.382002 0.007561
6 -2.289958 -0.233351 0.005606
1 -3.373583 -0.283899 0.008852
7 -1.727133 0.958734 -0.000147
1 -2.306042 1.803431 -0.001946
7 0.459876 0.067613 -0.002262
8 0.101945 2.335783 -0.009954
8 0.741479 -2.202847 0.002663
1 1.500099 0.203062 -0.007570
1 -1.907120 -2.374990 0.012304
8 3.211228 0.304383 -0.069839
1 3.563009 0.515560 0.808713
1 3.522224 -0.595784 -0.253821
rc_U/rc_Uadu.xyz
6 -0.043850 -1.156819 0.006919

6 -0.296654 1.272342 0.000973
6 -1.751444 1.089302 -0.003295
6 -2.290009 -0.212303 -0.003279
1 -3.357457 -0.405923 -0.007866
7 -1.474497 -1.248264 0.002381
1 -1.850053 -2.200640 0.002370
7 0.458179 0.110792 0.007036
8 0.616597 -2.179382 0.009907
8 0.226954 2.385769 -0.000621
1 1.504715 0.188015 -0.000542
1 -2.393392 1.961438 -0.008406
8 3.210650 0.129655 -0.099491
1 3.612991 0.579300 0.659388
1 3.455557 -0.803343 0.002862
rc_U/rc_Uade.xyz
6 0.109414 -1.172222 -0.035913
6 0.226280 1.267311 -0.036048
6 1.687440 1.166561 0.019383
6 2.296408 -0.102561 0.063790
1 3.371784 -0.235360 0.115246
7 1.540803 -1.182406 0.039968
1 1.968609 -2.112401 0.063805
7 -0.462700 0.065006 -0.076585
8 -0.490629 -2.230979 -0.058265
8 -0.357805 2.350333 -0.042642
1 -1.512186 0.090487 -0.071438
1 2.279768 2.072715 0.038837
8 -3.214911 0.105892 0.058820
1 -3.459577 -0.219625 0.938907
1 -3.605607 -0.530524 -0.559611
rc_U/rc_Uadd.xyz
6 0.317688 -1.191439 -0.002936
6 0.010613 1.232117 0.002883
6 1.468188 1.387903 0.005403
6 2.288521 0.243463 0.003988
1 3.371831 0.300085 0.005775

7 1.732243 -0.951709 0.000619
1 2.315747 -1.793234 -0.000435
7 -0.459186 -0.072043 -0.001198
8 -0.089938 -2.338249 -0.006548
8 -0.753963 2.196259 0.003801
1 -1.499019 -0.211429 -0.008516
1 1.894492 2.383145 0.007973
8 -3.207720 -0.285025 -0.089612
1 -3.519697 0.628329 0.006978
1 -3.571843 -0.756768 0.675128
rc_U/rc_Uaah.xyz
6 -1.540672 -0.107914 0.174267
6 0.706090 -0.999883 -0.307611
6 1.060855 0.312472 -0.856374
6 0.086390 1.323321 -0.918009
1 0.287344 2.296977 -1.351083
7 -1.122059 1.110008 -0.425081
1 -1.830420 1.844973 -0.460160
7 -0.587220 -1.092911 0.197939
8 -2.670135 -0.221109 0.614229
8 1.474711 -1.956212 -0.280708
1 -0.872359 -1.983417 0.603808
1 2.034672 0.449414 -1.305356
8 1.917669 1.227841 1.256438
1 1.842516 0.460432 1.848208
1 2.849263 1.239821 0.981275
rc_U/rc_Uaav.xyz
6 1.536110 -0.156644 0.176186
6 -0.744021 -0.942455 -0.331335
6 -1.041070 0.397742 -0.849779
6 -0.023459 1.363978 -0.895312
1 -0.178812 2.352690 -1.312804
7 1.173818 1.089148 -0.400356
1 1.915001 1.791294 -0.423821
7 0.542814 -1.102034 0.172125
8 2.657020 -0.326605 0.620437

8 -1.552432 -1.864684 -0.329471
1 0.786369 -2.012325 0.560542
1 -2.005991 0.583133 -1.300811
8 -1.917950 0.989383 1.328202
1 -1.552129 1.879084 1.466091
1 -2.839334 1.135843 1.056515
rc_U/rc_Uaaa.xyz
6 1.537895 -0.151679 0.178593
6 -0.736933 -0.948769 -0.334247
6 -1.039669 0.389189 -0.854616
6 -0.028030 1.362452 -0.892418
1 -0.188758 2.351996 -1.305978
7 1.168634 1.094176 -0.394203
1 1.904551 1.802114 -0.410523
7 0.550968 -1.103695 0.167448
8 2.658447 -0.315669 0.625526
8 -1.544070 -1.872474 -0.326542
1 0.798756 -2.013295 0.554893
1 -2.005647 0.570893 -1.304903
8 -1.883001 1.042881 1.333735
1 -2.753646 0.622153 1.236697
1 -2.043059 1.987711 1.171479
rc_U/rc_U14_abb.xyz
6 -1.533554 -0.137527 -0.165054
6 0.738516 -0.966458 0.313119
6 1.065944 0.366311 0.832580
6 0.063523 1.347027 0.900063
1 0.239645 2.329448 1.324052
7 -1.146211 1.094432 0.422288
1 -1.875374 1.808087 0.461716
7 -0.553927 -1.097242 -0.185504
8 -2.662299 -0.286847 -0.597520
8 1.526964 -1.905724 0.303398
1 -0.818362 -2.001142 -0.575247
1 2.037026 0.531325 1.277444
8 1.975912 1.013250 -1.284882

1 1.186376 1.285483 -1.782593
1 2.400471 1.844912 -1.015074
rc_U/rc_U14_aba.xyz
6 -1.527438 0.142306 0.162092
6 0.746730 0.969978 -0.311650
6 1.070985 -0.359711 -0.840654
6 0.065495 -1.337296 -0.913892
1 0.240001 -2.317282 -1.344277
7 -1.143331 -1.086268 -0.434648
1 -1.873461 -1.798841 -0.477412
7 -0.548069 1.102164 0.181104
8 -2.653680 0.289307 0.601535
8 1.539752 1.905233 -0.288442
1 -0.808789 2.002318 0.581873
1 2.043157 -0.526313 -1.282129
8 1.971213 -1.065614 1.280233
1 2.054468 -2.020879 1.121072
1 1.191515 -0.973344 1.853684
rc_U/rc_U14_abe.xyz
6 1.532708 0.141819 0.174111
6 -0.734915 0.969785 -0.321230
6 -1.052753 -0.356199 -0.861013
6 -0.052161 -1.340714 -0.912459
1 -0.225392 -2.323515 -1.337037
7 1.148550 -1.091938 -0.415441
1 1.875910 -1.808431 -0.440769
7 0.555659 1.103885 0.179918
8 2.656089 0.287518 0.620089
8 -1.532422 1.901898 -0.297400
1 0.813603 2.004560 0.581331
1 -2.023522 -0.523840 -1.306410
8 -1.836944 -1.123651 1.331525
1 -2.574048 -0.495024 1.410376
1 -2.247081 -1.951638 1.030995
rc_U/rc_Uadp.xyz
6 -1.517040 -0.177545 0.163813

6 0.772884 -0.945383 -0.326834
6 1.054760 0.387714 -0.868050
6 0.025173 1.341815 -0.931777
1 0.172207 2.326112 -1.362487
7 -1.170545 1.060110 -0.441644
1 -1.918525 1.755010 -0.473287
7 -0.518336 -1.117013 0.161937
8 -2.630504 -0.346200 0.625944
8 1.598687 -1.852255 -0.290880
1 -0.747434 -2.018323 0.579049
1 2.025103 0.584614 -1.301870
8 1.854384 1.264150 1.289437
1 2.560510 0.609846 1.421434
1 1.135113 0.975897 1.876202
rc_U/rc_Uaad.xyz
6 1.526687 0.147233 0.165051
6 -0.746460 0.973969 -0.310572
6 -1.062891 -0.347694 -0.860288
6 -0.059027 -1.328596 -0.928260
1 -0.233012 -2.307396 -1.361442
7 1.143688 -1.080989 -0.437064
1 1.872554 -1.795543 -0.472764
7 0.548608 1.108246 0.181441
8 2.650491 0.289831 0.610940
8 -1.546850 1.903323 -0.271694
1 0.805684 2.004661 0.592762
1 -2.036645 -0.515619 -1.298707
8 -1.881982 -1.275281 1.256673
1 -1.282272 -0.844671 1.888628
1 -2.695510 -0.744699 1.277947
d_1m5caC/d_1m5caCads.xyz
6 -1.571647 0.657687 -0.007741
6 0.139957 -0.957199 -0.002140
6 1.169090 0.055222 -0.002374
6 0.720990 1.350449 -0.000048
1 1.418283 2.178998 0.001775

7 -0.594319 1.670472 0.000806
7 -1.165653 -0.632874 -0.007917
8 -2.778853 0.986779 -0.014359
7 0.452696 -2.258068 0.003775
1 -0.279494 -2.954856 0.005250
1 1.441174 -2.499965 0.007214
6 2.651738 -0.222114 -0.000832
8 3.029112 -1.440537 0.004730
8 3.439657 0.772581 -0.004808
6 -1.039441 3.069617 0.013492
1 -1.612184 3.289294 -0.889722
1 -1.668489 3.251200 0.886695
1 -0.161886 3.713526 0.054937
8 -3.179189 -2.589541 -0.000319
1 -2.481609 -1.883615 -0.002475
1 -4.014801 -2.107517 0.075583
d_1m5caC/d_1m5caCacv.xyz
6 1.579356 -0.691156 0.024339
6 -0.114286 0.942391 0.025510
6 -1.154755 -0.058226 0.004863
6 -0.721222 -1.358314 -0.004746
1 -1.427422 -2.179105 -0.020361
7 0.590275 -1.692964 0.003794
7 1.188308 0.604060 0.037723
8 2.781978 -1.035893 0.030093
7 -0.412950 2.246582 0.035596
1 0.327061 2.935346 0.042849
1 -1.398571 2.499221 0.020470
6 -2.634106 0.235380 -0.011557
8 -2.997940 1.457896 -0.019589
8 -3.433252 -0.750452 -0.017858
6 1.019915 -3.096733 -0.023926
1 1.648971 -3.275115 -0.897800
1 1.588192 -3.332737 0.878013
1 0.135390 -3.730430 -0.074389
8 3.105658 2.660320 0.032281

1 3.602168 2.579998 -0.794203
1 2.463829 1.904053 0.019317
d_1m5caC/d_1m5caCack.xyz
6 2.524352 0.238694 0.068909
6 0.474117 1.366933 -0.007767
6 -0.270215 0.130041 -0.074387
6 0.486848 -1.013206 -0.078372
1 0.020142 -1.988829 -0.131324
7 1.837424 -0.991837 -0.014548
7 1.814837 1.388010 0.066741
8 3.775248 0.212236 0.141100
7 -0.160329 2.547355 -0.020097
1 0.372045 3.403461 0.047412
1 -1.176105 2.537371 -0.052171
6 -1.769438 0.030080 -0.117231
8 -2.450178 1.103477 -0.113935
8 -2.280901 -1.138182 -0.148275
6 2.626964 -2.230878 -0.021886
1 3.276938 -2.252881 -0.899023
1 3.240084 -2.283968 0.879510
1 1.946311 -3.081350 -0.050587
8 -4.966875 -1.041920 0.245309
1 -5.190748 -0.101520 0.287818
1 -3.986308 -1.051856 0.084492
d_1m5caC/d_1m5caCadp.xyz
6 -2.272315 0.141142 0.004594
6 -0.636710 -1.534936 0.008413
6 0.439640 -0.568444 -0.007747
6 0.045805 0.744931 -0.014046
1 0.766574 1.550837 -0.022978
7 -1.253865 1.116507 -0.006545
7 -1.927949 -1.164340 0.012073
8 -3.462963 0.533218 0.008104
7 -0.376726 -2.849356 0.019949
1 -1.140584 -3.510876 0.030587
1 0.597989 -3.138269 0.020031

6 1.904053 -0.913648 -0.018210
8 2.230505 -2.142786 0.004517
8 2.751020 0.037751 -0.050010
6 -1.637604 2.533758 -0.013553
1 -2.241549 2.752535 -0.896127
1 -2.217361 2.768545 0.881247
1 -0.732158 3.139420 -0.031314
8 3.000124 2.770267 -0.014723
1 3.794906 2.921357 0.515512
1 2.875262 1.792343 -0.015113
d_1m5caC/d_1m5caCadk.xyz
6 -2.536194 0.166063 -0.050527
6 -0.535801 1.382940 -0.001200
6 0.262572 0.179999 0.052160
6 -0.443932 -0.995088 0.059195
1 0.065870 -1.949358 0.100525
7 -1.794644 -1.033311 0.013214
7 -1.877280 1.344750 -0.053790
8 -3.785949 0.085743 -0.101457
7 0.044540 2.590985 0.001294
1 -0.528951 3.421163 -0.052792
1 1.059653 2.630314 0.024886
6 1.764951 0.143522 0.084029
8 2.399401 1.244365 0.079152
8 2.323726 -1.002756 0.110949
6 -2.526844 -2.307190 0.027039
1 -3.164349 -2.359853 0.912025
1 -3.148752 -2.386226 -0.866435
1 -1.806598 -3.124585 0.045817
8 5.028455 -0.970064 -0.144095
1 4.038891 -0.954691 -0.054005
1 5.242355 -1.873521 -0.415617
d_1m5caC/d_1m5caCadg.xyz
6 -2.535023 0.162068 -0.062757
6 -0.538022 1.384130 -0.004218
6 0.262835 0.183394 0.060971

6 -0.441021 -0.993299 0.069988
1 0.070598 -1.946124 0.121256
7 -1.791242 -1.035119 0.013860
7 -1.878906 1.342283 -0.067999
8 -3.784198 0.078452 -0.122656
7 0.039032 2.593751 -0.002043
1 -0.536319 3.422044 -0.064548
1 1.053743 2.636230 0.030652
6 1.765080 0.150637 0.103318
8 2.396593 1.253131 0.101901
8 2.326254 -0.994376 0.134960
6 -2.520365 -2.310646 0.038677
1 -3.154786 -2.358835 0.926227
1 -3.145163 -2.397500 -0.851933
1 -1.798008 -3.126078 0.060675
8 5.027091 -0.992865 -0.155412
1 5.202025 -1.786466 -0.680020
1 4.038888 -0.962114 -0.055262
d_1m5caC/d_1m5caCaah.xyz
6 -2.495512 -0.487343 0.044207
6 -0.288947 -1.269013 -0.032538
6 0.246700 0.072975 -0.056794
6 -0.685264 1.078644 -0.037510
1 -0.382791 2.118245 -0.054241
7 -2.014999 0.839016 0.006344
7 -1.610278 -1.506288 0.016722
8 -3.735009 -0.665173 0.103823
7 0.525188 -2.333564 -0.061625
1 0.134131 -3.264661 -0.028217
1 1.527040 -2.166141 -0.073665
6 1.708025 0.426721 -0.089888
8 2.552247 -0.536660 -0.069581
8 2.029987 1.649144 -0.132333
6 -2.987869 1.938588 0.034456
1 -3.669694 1.851765 -0.813418
1 -3.563603 1.901281 0.961619

1 -2.447605 2.882936 -0.023594
8 5.130508 0.283051 0.201347
1 5.121076 1.250490 0.209030
1 4.177411 0.025618 0.094745
d_1m5caC/d_1m5caCaak.xyz
6 2.152838 -0.833724 0.033103
6 -0.140652 -0.994350 -0.418365
6 -0.331902 0.429007 -0.258132
6 0.798743 1.141910 0.048641
1 0.757900 2.215162 0.187622
7 2.009971 0.560741 0.196738
7 1.063774 -1.571463 -0.272290
8 3.294320 -1.331130 0.179391
7 -1.168537 -1.797326 -0.728154
1 -1.013441 -2.790606 -0.829552
1 -2.088023 -1.380754 -0.832090
6 -1.639691 1.159036 -0.405443
8 -2.688273 0.466739 -0.657343
8 -1.637807 2.417045 -0.273710
6 3.202665 1.354340 0.520750
1 3.646792 0.997909 1.451800
1 3.938253 1.266716 -0.281455
1 2.907988 2.397231 0.632338
8 -3.834957 -0.866513 1.494384
1 -3.436609 -0.361902 0.750538
1 -3.367598 -1.713853 1.481631
d_1m5caC/d_1m5caCaas.xyz
6 2.263160 -0.745351 0.117109
6 -0.011579 -1.102680 -0.309277
6 -0.297008 0.314175 -0.288998
6 0.777642 1.127267 -0.034652
1 0.661553 2.203188 0.009095
7 2.023715 0.644679 0.170271
7 1.228579 -1.581491 -0.114920
8 3.436919 -1.148261 0.292847
7 -0.987748 -1.996467 -0.520398

1 -0.770618 -2.983254 -0.522637
1 -1.928149 -1.644565 -0.676759
6 -1.651720 0.929553 -0.508304
8 -2.628184 0.141617 -0.770837
8 -1.762329 2.186605 -0.420510
6 3.155846 1.539555 0.444496
1 3.603675 1.284380 1.406743
1 3.910432 1.439148 -0.338174
1 2.791862 2.565996 0.471532
8 -4.189351 -0.518377 1.415445
1 -3.646526 -0.247190 0.640578
1 -4.748529 0.247459 1.607146
d_1m5caC/d_1m5caCadr.xyz
6 1.456555 0.341361 -0.020952
6 -0.399495 -1.080323 -0.011100
6 -1.321561 0.031224 -0.004171
6 -0.743222 1.273561 -0.004051
1 -1.349367 2.170713 0.004625
7 0.598553 1.454080 -0.014336
7 0.933270 -0.899111 -0.022264
8 2.697136 0.557173 -0.024428
7 -0.848682 -2.342123 -0.006484
1 -0.191422 -3.109425 -0.008488
1 -1.855436 -2.484705 0.004383
6 -2.826310 -0.086764 0.007613
8 -3.333488 -1.256949 0.014916
8 -3.501019 0.987732 0.009773
6 1.190033 2.799033 0.017584
1 1.790103 2.916555 0.922104
1 1.828586 2.945281 -0.855371
1 0.385541 3.533767 0.011453
8 4.475029 -1.528700 0.040624
1 3.771480 -0.835461 0.013506
1 4.001267 -2.369254 -0.027236
d_1m5caC/d_1m5caCabw.xyz
6 -1.357351 0.542090 -0.487425

6 0.859244 1.186845 -0.116241
6 1.299621 -0.184072 -0.004432
6 0.320893 -1.129641 -0.161648
1 0.551507 -2.185767 -0.102360
7 -0.972424 -0.807746 -0.399965
7 -0.427597 1.505200 -0.346092
8 -2.572820 0.797132 -0.698406
7 1.730042 2.196406 0.009386
1 1.406414 3.151512 -0.054170
1 2.702376 1.958331 0.189654
6 2.718548 -0.621944 0.267466
8 3.609527 0.283002 0.384243
8 2.941634 -1.866989 0.366323
6 -1.996581 -1.846831 -0.577774
1 -2.754268 -1.765649 0.203483
1 -2.474246 -1.732479 -1.552464
1 -1.516374 -2.822858 -0.517642
8 -4.376038 0.258527 1.361276
1 -4.373300 -0.692691 1.537556
1 -3.736921 0.380522 0.625494
d_1m5caC/d_1m5caCaax.xyz
6 1.454592 0.337117 0.026326
6 -0.403492 -1.081844 0.013907
6 -1.323980 0.030956 0.003596
6 -0.743873 1.272467 0.004132
1 -1.348701 2.170480 -0.006496
7 0.598113 1.451071 0.017571
7 0.929510 -0.902556 0.028224
8 2.695409 0.551546 0.031402
7 -0.854370 -2.343040 0.009682
1 -0.198093 -3.111181 0.013582
1 -1.861262 -2.484335 -0.003828
6 -2.828858 -0.084894 -0.011569
8 -3.337662 -1.254363 -0.019016
8 -3.502043 0.990560 -0.016139
6 1.191517 2.795215 -0.013891

1 1.828800 2.940858 0.860088
1 1.793306 2.911596 -0.917419
1 0.388045 3.531082 -0.009340
8 4.487151 -1.519864 -0.084819
1 4.086581 -2.322126 0.277712
1 3.780275 -0.831842 -0.029069
d_1m5caC/d_1m5caCadh.xyz
6 -2.262837 -0.742603 0.120048
6 0.010544 -1.105409 -0.308951
6 0.298808 0.310916 -0.292287
6 -0.774018 1.126792 -0.039185
1 -0.655782 2.202594 0.001440
7 -2.020808 0.647179 0.168300
7 -1.230243 -1.581330 -0.111481
8 -3.436964 -1.142846 0.299329
7 0.984721 -2.001440 -0.519627
1 0.765828 -2.987838 -0.519810
1 1.925531 -1.651485 -0.678095
6 1.654604 0.923010 -0.514066
8 2.629298 0.132697 -0.775926
8 1.767898 2.179999 -0.428302
6 -3.150809 1.544965 0.441616
1 -3.907562 1.442361 -0.338665
1 -3.596556 1.294608 1.406112
1 -2.785223 2.570967 0.463228
8 4.195576 -0.511867 1.414072
1 3.646899 -0.248572 0.640722
1 4.566954 0.316620 1.748283
d_1m5caC/d_1m5caCaan.xyz
6 1.355473 0.542623 -0.482370
6 -0.861525 1.187116 -0.112954
6 -1.302326 -0.183929 -0.004214
6 -0.323205 -1.129342 -0.159938
1 -0.553835 -2.185531 -0.101906
7 0.970651 -0.807236 -0.395061
7 0.425596 1.505636 -0.340888

8 2.570753 0.797992 -0.693750
7 -1.732264 2.196595 0.013752
1 -1.408460 3.151769 -0.047851
1 -2.704964 1.958281 0.191746
6 -2.721910 -0.621938 0.264079
8 -3.612870 0.283052 0.380720
8 -2.945559 -1.867085 0.360293
6 1.995545 -1.846182 -0.569341
1 2.477624 -1.730945 -1.541720
1 2.749675 -1.765701 0.215464
1 1.515006 -2.822219 -0.512274
8 4.403201 0.254346 1.336176
1 3.750313 0.378185 0.612766
1 4.350242 -0.685341 1.560066
d_1m5caC/d_1m5caCacz.xyz
6 1.338942 0.545674 -0.433971
6 -0.884088 1.188694 -0.098399
6 -1.326293 -0.182772 -0.000458
6 -0.344379 -1.127380 -0.143670
1 -0.575151 -2.183863 -0.092009
7 0.953018 -0.804422 -0.356861
7 0.406422 1.508181 -0.303685
8 2.556112 0.803774 -0.628097
7 -1.757006 2.197812 0.016694
1 -1.432617 3.153120 -0.039626
1 -2.732457 1.958788 0.177941
6 -2.749387 -0.621345 0.247513
8 -3.641598 0.283680 0.354306
8 -2.974692 -1.866571 0.338566
6 1.980795 -1.843127 -0.516591
1 2.482101 -1.722636 -1.478544
1 2.718644 -1.767370 0.283709
1 1.498174 -2.818874 -0.475130
8 4.545135 0.047487 1.174480
1 3.832970 0.290793 0.542999
1 5.018099 0.873631 1.347044

d_1m5caC/d_1m5caCaca.xyz
6 1.356822 0.524685 -0.486883
6 -0.856899 1.185470 -0.125968
6 -1.304858 -0.181768 -0.000805
6 -0.331237 -1.134194 -0.148812
1 -0.567797 -2.188440 -0.080161
7 0.963826 -0.821911 -0.389780
7 0.432160 1.494468 -0.356061
8 2.573976 0.771816 -0.697813
7 -1.722342 2.201315 -0.013389
1 -1.393142 3.153695 -0.088296
1 -2.696204 1.971104 0.168433
6 -2.726284 -0.609615 0.273753
8 -3.612707 0.300921 0.381559
8 -2.956011 -1.852571 0.383421
6 1.982566 -1.868221 -0.558161
1 2.459763 -1.765452 -1.534414
1 2.740642 -1.782425 0.221905
1 1.496966 -2.840994 -0.488925
8 4.421520 0.114287 1.291731
1 4.346963 0.836298 1.931316
1 3.762423 0.325356 0.594836
d_1m5caC/d_1m5caCact.xyz
6 -1.338993 -0.549803 -0.434623
6 0.885278 -1.188544 -0.098933
6 1.324746 0.183744 -0.000503
6 0.341173 1.126451 -0.144788
1 0.570030 2.183363 -0.093383
7 -0.955513 0.801066 -0.358760
7 -0.404668 -1.510459 -0.303917
8 -2.555743 -0.810534 -0.628228
7 1.760184 -2.196003 0.015435
1 1.437686 -3.151913 -0.041573
1 2.735189 -1.955144 0.176694
6 2.746827 0.624980 0.248554
8 3.640651 -0.278423 0.355484

8 2.969666 1.870584 0.340430
6 -1.984605 1.838100 -0.521236
1 -2.484800 1.715156 -1.483465
1 -2.722959 1.762809 0.278580
1 -1.503332 2.814571 -0.481171
8 -4.549364 -0.040192 1.161102
1 -4.887805 -0.875179 1.513493
1 -3.832264 -0.296927 0.540390
d_1m5caC/d_1m5caCadb.xyz
6 1.359286 0.518959 -0.492809
6 -0.852618 1.184789 -0.130082
6 -1.302911 -0.181311 -0.000927
6 -0.331311 -1.135937 -0.148164
1 -0.569773 -2.189572 -0.076701
7 0.963908 -0.826635 -0.392014
7 0.436642 1.490830 -0.363050
8 2.576685 0.763174 -0.705690
7 -1.715940 2.202578 -0.018621
1 -1.384987 3.154106 -0.096573
1 -2.689950 1.974810 0.165354
6 -2.724745 -0.606012 0.276319
8 -3.609500 0.306253 0.383199
8 -2.956487 -1.848346 0.388872
6 1.980734 -1.874963 -0.559384
1 2.454821 -1.776849 -1.537649
1 2.741604 -1.786442 0.217689
1 1.494215 -2.846865 -0.484424
8 4.417885 0.139253 1.302014
1 4.203033 0.759173 2.013078
1 3.759492 0.328389 0.598143
d_1m5caC/d_1m5caCabz.xyz
6 -2.180122 -0.365873 -0.127399
6 -0.151107 -1.533317 -0.191259
6 0.599466 -0.368449 0.218044
6 -0.145729 0.758727 0.451862
1 0.321680 1.679426 0.777191

7 -1.488934 0.788456 0.299804
7 -1.483556 -1.499742 -0.356358
8 -3.422580 -0.289600 -0.273354
7 0.470919 -2.695727 -0.434584
1 -0.070193 -3.508294 -0.695268
1 1.469867 -2.744892 -0.255046
6 2.092160 -0.327063 0.399023
8 2.735147 -1.422236 0.360982
8 2.636424 0.811726 0.585358
6 -2.258191 2.015383 0.547789
1 -2.786299 2.312101 -0.360377
1 -2.985482 1.846847 1.344257
1 -1.569218 2.806221 0.843298
8 2.057975 2.831654 -1.191738
1 2.238521 2.105919 -0.547155
1 1.097524 2.822964 -1.307278
d_1m5caC/d_1m5caCabd.xyz
6 -1.279209 0.683153 0.079707
6 1.003424 1.191212 0.017603
6 1.372576 -0.205054 -0.000937
6 0.325961 -1.088168 0.027037
1 0.498448 -2.156968 0.017319
7 -0.967419 -0.688412 0.068146
7 -0.280638 1.587604 0.059178
8 -2.489907 1.022912 0.112783
7 1.941180 2.147344 -0.000269
1 1.667146 3.119864 0.005041
1 2.913052 1.852997 -0.055360
6 2.787537 -0.728441 -0.045885
8 3.732092 0.121905 -0.150563
8 2.955285 -1.984314 0.022571
6 -2.063133 -1.667167 0.083367
1 -2.706171 -1.488632 0.946299
1 -2.652163 -1.581651 -0.831763
1 -1.635407 -2.666954 0.149018
8 -5.037099 -0.011387 -0.203376

1 -5.001880 -0.977434 -0.240498
1 -4.100794 0.266884 -0.096114
d_1m5caU/d_1m5caUaab.xyz
6 -2.523694 0.399722 0.019578
6 -0.215304 1.345173 0.015996
6 0.296099 -0.015452 -0.022054
6 -0.614833 -1.037388 -0.037979
1 -0.276351 -2.065559 -0.060879
7 -1.966848 -0.869133 -0.029930
7 -1.613681 1.427756 0.033896
8 -3.744380 0.589105 0.048407
8 0.428410 2.405188 0.046392
6 1.766364 -0.367759 -0.035523
8 2.074474 -1.584449 0.136370
8 2.606797 0.569221 -0.225011
1 -2.009510 2.363533 0.075751
6 -2.890400 -2.014784 -0.039953
1 -3.477419 -2.023180 0.880433
1 -3.560319 -1.939137 -0.897916
1 -2.304625 -2.929522 -0.110191
8 5.184058 -0.232985 0.047076
1 5.159709 -1.163225 0.311992
1 4.227955 0.021007 -0.053215
d_1m5caU/d_1m5caUacj.xyz
6 -2.171570 -0.310060 -0.103855
6 -0.036822 -1.584621 -0.287452
6 0.657413 -0.407770 0.204417
6 -0.088303 0.702127 0.488543
1 0.387659 1.601967 0.857228
7 -1.443489 0.776257 0.354269
7 -1.422368 -1.423581 -0.400713
8 -3.400015 -0.279561 -0.235399
8 0.458455 -2.670650 -0.627151
6 2.149638 -0.364341 0.441280
8 2.706911 0.783566 0.398200
8 2.752183 -1.444856 0.692605

1 -1.943377 -2.222803 -0.752219
6 -2.185832 1.999121 0.700140
1 -2.746491 2.347552 -0.168649
1 -2.875720 1.795519 1.521173
1 -1.469516 2.760997 1.002675
8 1.935102 2.767715 -1.320297
1 2.171412 2.038276 -0.695022
1 0.968800 2.753312 -1.362190
d_1m5caU/d_1m5caUada.xyz
6 -2.149449 -0.359429 0.116142
6 0.028628 -1.558368 0.289731
6 0.674844 -0.368539 -0.233582
6 -0.111492 0.708654 -0.533396
1 0.328713 1.617097 -0.924984
7 -1.467523 0.737122 -0.385765
7 -1.358195 -1.439030 0.428646
8 -3.375608 -0.366867 0.270991
8 0.565759 -2.621768 0.638010
6 2.163665 -0.275868 -0.476688
8 2.684552 0.888351 -0.413850
8 2.797334 -1.333616 -0.747477
1 -1.846500 -2.245882 0.808593
6 -2.261410 1.917678 -0.763213
1 -2.988169 1.641674 -1.529148
1 -2.784613 2.307214 0.111677
1 -1.584914 2.674870 -1.156248
8 1.785334 2.687356 1.452497
1 0.856469 2.477015 1.622570
1 2.071367 2.028957 0.772041
d_1m5caU/d_1m5caUabq.xyz
6 -2.543338 0.116087 -0.008214
6 -0.445146 1.463976 0.057291
6 0.304396 0.219684 0.024832
6 -0.404043 -0.950587 0.002270
1 0.116314 -1.899724 -0.010657
7 -1.764506 -1.030935 -0.016467

7 -1.834988 1.292533 0.036799
8 -3.778065 0.081671 -0.037485
8 -0.001274 2.621223 0.115761
6 1.813653 0.151149 -0.003084
8 2.336158 -0.979954 0.279000
8 2.467921 1.184745 -0.315894
1 -2.394750 2.141346 0.055571
6 -2.464040 -2.325522 -0.073730
1 -3.124997 -2.425155 0.788949
1 -3.052556 -2.385699 -0.991089
1 -1.720142 -3.120225 -0.059905
8 5.032155 -1.039603 -0.005275
1 4.044005 -0.976780 0.086985
1 5.304531 -0.158329 -0.297229
d_1m5caU/d_1m5caUadd.xyz
6 -2.266489 -0.187235 -0.017196
6 -0.567343 1.637052 0.037664
6 0.468675 0.617340 0.033235
6 0.070886 -0.691385 0.031935
1 0.800223 -1.490385 0.045459
7 -1.228282 -1.104689 0.002350
7 -1.871167 1.128139 0.006628
8 -3.454158 -0.527063 -0.050854
8 -0.424926 2.868547 0.087165
6 1.947216 0.935970 0.016830
8 2.747797 0.009308 0.376398
8 2.311053 2.085424 -0.359465
1 -2.621752 1.813969 0.010323
6 -1.579492 -2.533849 -0.016026
1 -2.202951 -2.771221 0.847617
1 -2.122905 -2.771535 -0.932385
1 -0.658936 -3.113618 0.026230
8 3.053308 -2.684341 -0.018367
1 2.917843 -1.714361 0.094077
1 3.279305 -2.799365 -0.951815
d_1m5caU/d_1m5caUabo.xyz

6 2.546904 0.069646 0.015618
6 0.485830 1.474445 0.056642
6 -0.297183 0.251669 0.002072
6 0.378974 -0.937503 -0.021683
1 -0.166242 -1.872235 -0.052646
7 1.737012 -1.055149 -0.017795
7 1.870771 1.264819 0.060138
8 3.780666 0.000890 0.007535
8 0.072967 2.643013 0.115635
6 -1.807429 0.225074 -0.044047
8 -2.363059 -0.892859 0.226427
8 -2.429936 1.277683 -0.357417
1 2.452837 2.097895 0.096692
6 2.400811 -2.367615 -0.087717
1 2.986096 -2.435963 -1.006706
1 3.060624 -2.492537 0.772384
1 1.635700 -3.142073 -0.079041
8 -5.071710 -0.929274 0.016738
1 -4.080822 -0.885549 0.082266
1 -5.301540 -1.827142 0.293997
d_1m5caU/d_1m5caUabx.xyz
6 2.523187 0.394441 0.058890
6 0.221581 1.347958 -0.066739
6 -0.295487 -0.011281 -0.075031
6 0.611417 -1.036487 -0.042129
1 0.269372 -2.063632 -0.057440
7 1.962593 -0.873120 0.016749
7 1.618058 1.425655 0.012611
8 3.742740 0.579807 0.130846
8 -0.414786 2.411009 -0.128027
6 -1.766890 -0.358709 -0.099907
8 -2.075512 -1.577731 -0.253671
8 -2.608474 0.584470 0.049447
1 2.017029 2.360929 0.025345
6 2.881099 -2.021988 0.058628
1 3.580846 -1.963534 -0.776603

1 3.436425 -2.016171 0.998527
1 2.294538 -2.935909 -0.015293
8 -5.182545 -0.240169 0.232656
1 -4.224338 0.015200 0.156633
1 -5.179252 -1.207323 0.211024
d_1m5caU/d_1m5caUaau.xyz
6 -2.066416 -0.737226 -0.084333
6 0.364993 -0.897797 -0.604209
6 0.490378 0.501383 -0.241420
6 -0.640258 1.169022 0.137873
1 -0.594081 2.219484 0.397884
7 -1.876364 0.598576 0.231508
7 -0.932513 -1.404711 -0.481715
8 -3.171360 -1.286993 -0.016172
8 1.256093 -1.653614 -1.022685
6 1.801652 1.254364 -0.260621
8 1.747803 2.501667 -0.468474
8 2.872463 0.601485 -0.042865
1 -1.064103 -2.379705 -0.738705
6 -3.051425 1.364691 0.676484
1 -3.813857 1.353352 -0.104267
1 -3.456973 0.922267 1.588189
1 -2.740553 2.389477 0.871677
8 2.888148 -1.623220 1.545107
1 2.852942 -0.830619 0.953218
1 2.340029 -2.272533 1.081538
d_1m5caU/d_1m5caUaan.xyz
6 -2.096256 -0.878790 -0.018066
6 0.376971 -0.905071 -0.289881
6 0.373838 0.533944 -0.127123
6 -0.829235 1.146268 0.090664
1 -0.879945 2.219413 0.226486
7 -2.027447 0.495499 0.142448
7 -0.883606 -1.497597 -0.213947
8 -3.162348 -1.502679 0.013017
8 1.360407 -1.649170 -0.463532

6 1.619457 1.389831 -0.239900
8 1.594336 2.517675 0.344335
8 2.582462 0.944208 -0.930398
1 -0.917618 -2.508407 -0.319034
6 -3.293113 1.222613 0.331808
1 -3.933230 1.079358 -0.540606
1 -3.801906 0.849690 1.222258
1 -3.066552 2.280316 0.454773
8 3.785449 -1.356537 0.946308
1 2.966483 -1.324064 0.409080
1 3.817722 -2.262366 1.284694
d_1m5caU/d_1m5caUadk.xyz
6 -2.083168 0.731047 -0.068635
6 0.334465 0.908048 -0.644665
6 0.480392 -0.486375 -0.269443
6 -0.635956 -1.162100 0.136109
1 -0.575134 -2.210053 0.403331
7 -1.876063 -0.602429 0.246693
7 -0.964374 1.405515 -0.495571
8 -3.190455 1.272714 0.023870
8 1.208226 1.664645 -1.094457
6 1.801736 -1.219312 -0.305272
8 1.765193 -2.471470 -0.487480
8 2.865109 -0.543125 -0.124615
1 -1.109417 2.378132 -0.754355
6 -3.036498 -1.376079 0.716160
1 -3.417711 -0.944552 1.643591
1 -3.820035 -1.357991 -0.043149
1 -2.718450 -2.402290 0.891272
8 2.861440 1.621454 1.562903
1 2.824725 0.875743 0.914065
1 3.457153 1.314284 2.260094
d_1m5caU/d_1m5caUacz.xyz
6 2.186037 0.270421 0.110158
6 0.077248 1.587565 0.288103
6 -0.638963 0.425510 -0.206588

6 0.084025 -0.700667 -0.485985
1 -0.408006 -1.590850 -0.856097
7 1.436983 -0.801852 -0.347356
7 1.459228 1.399602 0.403123
8 3.413363 0.214798 0.244428
8 -0.397197 2.682764 0.628505
6 -2.130629 0.412469 -0.446780
8 -2.709700 -0.725166 -0.411779
8 -2.713130 1.505267 -0.691843
1 1.995273 2.188053 0.756439
6 2.154790 -2.042532 -0.681283
1 2.850346 -1.860869 -1.502693
1 2.706908 -2.394127 0.191695
1 1.423700 -2.792496 -0.977716
8 -1.921096 -2.774034 1.212587
1 -2.190326 -2.008940 0.645071
1 -2.424347 -2.670661 2.031999
d_1m5caU/d_1m5caUadg.xyz
6 -2.191691 0.253064 0.111572
6 -0.093928 1.587653 0.290089
6 0.632060 0.431745 -0.205269
6 -0.081532 -0.700597 -0.483841
1 0.418126 -1.587324 -0.852667
7 -1.433520 -0.813298 -0.344650
7 -1.474325 1.388288 0.404513
8 -3.418587 0.187356 0.245073
8 0.371354 2.686355 0.631516
6 2.123440 0.432043 -0.448383
8 2.711306 -0.701223 -0.421328
8 2.696714 1.530771 -0.688611
1 -2.016888 2.172107 0.758246
6 -2.140765 -2.060163 -0.677870
1 -2.692739 -2.413955 0.194278
1 -2.835389 -1.885763 -1.501684
1 -1.402980 -2.805078 -0.970348
8 1.946517 -2.735779 1.235669

1 2.729837 -2.919914 1.772329
1 2.211008 -1.987322 0.644465
d_1m5caU/d_1m5caUacx.xyz
6 -1.361941 0.497897 -0.466082
6 0.969377 1.275689 -0.054243
6 1.378490 -0.118449 0.014547
6 0.410577 -1.070343 -0.156237
1 0.669564 -2.120549 -0.114813
7 -0.907739 -0.805469 -0.388587
7 -0.400045 1.457521 -0.296960
8 -2.551973 0.778668 -0.673374
8 1.668993 2.290696 0.079264
6 2.805711 -0.576205 0.259070
8 2.987031 -1.826233 0.403371
8 3.721081 0.298046 0.299278
1 -0.725318 2.419982 -0.342445
6 -1.891912 -1.883174 -0.587101
1 -2.664364 -1.824669 0.181035
1 -2.350609 -1.784480 -1.572491
1 -1.374597 -2.838235 -0.516277
8 -4.463136 0.026164 1.337449
1 -3.826737 0.227176 0.624380
1 -5.331237 0.065181 0.911806
d_1m5caU/d_1m5caUacq.xyz
6 -2.144484 0.779713 -0.009367
6 0.319406 0.995614 -0.279249
6 0.421908 -0.444907 -0.165399
6 -0.731413 -1.148438 0.048489
1 -0.700278 -2.225220 0.160822
7 -1.973071 -0.588475 0.123848
7 -0.982275 1.490141 -0.201053
8 -3.253694 1.321402 0.042583
8 1.247316 1.816488 -0.411137
6 1.724465 -1.209032 -0.301126
8 1.781180 -2.345852 0.261032
8 2.652493 -0.683829 -0.985186

1 -1.089891 2.499049 -0.270627
6 -3.179981 -1.403847 0.334453
1 -3.674699 -1.095051 1.257039
1 -3.863759 -1.274033 -0.506159
1 -2.882193 -2.448086 0.408977
8 3.556875 1.258149 1.135872
1 2.764620 1.491665 0.609500
1 3.800851 0.394542 0.768767
d_1m5caU/d_1m5caUabw.xyz
6 -1.461692 0.448419 -0.081140
6 0.422907 -1.182623 -0.084850
6 1.340941 -0.056970 -0.012881
6 0.800584 1.200045 0.010379
1 1.448128 2.066158 0.061654
7 -0.535641 1.472316 -0.023138
7 -0.931202 -0.814460 -0.109288
8 -2.682756 0.664825 -0.106852
8 0.690813 -2.392475 -0.131181
6 2.851993 -0.189145 0.051860
8 3.519013 0.893065 0.043304
8 3.356830 -1.349106 0.117105
1 -1.602829 -1.577110 -0.161202
6 -1.044890 2.851886 0.042764
1 -1.601845 3.087340 -0.865829
1 -1.699174 2.959830 0.909370
1 -0.195361 3.525771 0.136468
8 -4.371089 -1.583219 0.149017
1 -3.822790 -0.777578 0.047681
1 -3.819780 -2.183795 0.670907
d_1m5caU/d_1m5caUack.xyz
6 1.376997 0.481502 0.502748
6 -0.944903 1.274500 0.068941
6 -1.359885 -0.116761 -0.016187
6 -0.398848 -1.074999 0.156824
1 -0.662574 -2.123470 0.102703
7 0.917588 -0.818863 0.409301

7 0.422404 1.447457 0.329147
8 2.566304 0.754093 0.726020
8 -1.637769 2.293857 -0.066468
6 -2.787587 -0.564601 -0.275189
8 -2.971325 -1.809578 -0.455274
8 -3.701368 0.312083 -0.287887
1 0.752248 2.407772 0.385916
6 1.892560 -1.903478 0.615917
1 2.672449 -1.849887 -0.144989
1 2.342004 -1.808879 1.605866
1 1.369684 -2.855070 0.539205
8 4.414650 -0.009507 -1.337354
1 4.323715 0.704296 -1.984399
1 3.788589 0.220513 -0.624055
d_1m5caU/d_1m5caUadp.xyz
6 2.109488 -0.807908 -0.039164
6 -0.353573 -0.942998 -0.356689
6 -0.419788 0.491937 -0.171424
6 0.745664 1.151993 0.102221
1 0.741505 2.222233 0.268312
7 1.970307 0.553933 0.173054
7 0.933039 -1.476759 -0.287139
8 3.204045 -1.380207 -0.005231
8 -1.304289 -1.726571 -0.540575
6 -1.698766 1.293412 -0.314326
8 -1.742826 2.413524 0.282877
8 -2.615963 0.815849 -1.044912
1 1.016685 -2.482254 -0.414475
6 3.193748 1.328634 0.437058
1 3.688129 0.944143 1.330677
1 3.872894 1.251237 -0.413934
1 2.914842 2.369337 0.592110
8 -3.513353 -1.303890 1.212321
1 -2.800042 -1.354874 0.543901
1 -3.438981 -2.130102 1.710108
d_1m5caU/d_1m5caUadv.xyz

6 -1.373272 0.491247 -0.496438
6 0.951728 1.275797 -0.063148
6 1.363140 -0.117054 0.014482
6 0.398933 -1.071727 -0.160897
1 0.659741 -2.121128 -0.111509
7 -0.917295 -0.810813 -0.409287
7 -0.415170 1.453670 -0.322546
8 -2.562432 0.768183 -0.714857
8 1.647211 2.292729 0.076972
6 2.789631 -0.570548 0.270803
8 2.971687 -1.818693 0.429439
8 3.703910 0.305106 0.304107
1 -0.742385 2.415178 -0.374027
6 -1.897492 -1.892239 -0.608395
1 -2.660814 -1.848958 0.169871
1 -2.368561 -1.784279 -1.586740
1 -1.373470 -2.844844 -0.556146
8 -4.394167 -0.048349 1.343382
1 -4.722067 0.785395 1.709166
1 -3.790862 0.213980 0.621434
d_1m5caU/d_1m5caUaaz.xyz
6 -1.461249 0.455893 -0.056201
6 0.421998 -1.172113 -0.112395
6 1.338424 -0.048515 -0.022835
6 0.802888 1.208259 0.023390
1 1.449496 2.075002 0.080119
7 -0.536005 1.479188 0.015539
7 -0.931235 -0.807206 -0.117189
8 -2.683137 0.670194 -0.066567
8 0.700133 -2.377378 -0.203625
6 2.846264 -0.197893 0.047697
8 3.536408 0.825554 -0.253608
8 3.319133 -1.311523 0.423616
1 -1.602756 -1.567972 -0.192251
6 -1.046168 2.856899 0.107961
1 -1.609693 3.105798 -0.793051

1 -1.694010 2.950313 0.981081
1 -0.196706 3.530289 0.206350
8 -4.354780 -1.608355 0.083998
1 -3.818264 -0.791422 0.017966
1 -3.812382 -2.209011 0.615119
d_1m5caU/d_1m5caUabv.xyz
6 -2.111497 0.798488 -0.041670
6 0.350428 0.948290 -0.359024
6 0.425234 -0.486177 -0.174135
6 -0.735526 -1.152840 0.103001
1 -0.724859 -2.222841 0.270484
7 -1.963582 -0.561679 0.175154
7 -0.939278 1.474294 -0.291312
8 -3.209726 1.363838 -0.009670
8 1.297129 1.737396 -0.540801
6 1.708347 -1.280101 -0.322322
8 1.765717 -2.394894 0.283463
8 2.614589 -0.802298 -1.066328
1 -1.029025 2.479138 -0.419798
6 -3.182079 -1.342850 0.442736
1 -3.679865 -0.955880 1.333363
1 -3.861030 -1.275582 -0.409314
1 -2.896495 -2.380652 0.604775
8 3.498631 1.310554 1.219867
1 2.782596 1.376952 0.555692
1 3.268534 1.964913 1.894153
d_1m5dhmC/d_1m5dhmC_3_020.xyz
6 2.520905 0.190069 -0.167135
6 0.495007 1.357345 -0.164728
6 -0.269568 0.158345 0.043906
6 0.457565 -0.989294 0.147168
1 -0.026099 -1.946579 0.293427
7 1.818829 -0.999654 0.063443
7 1.830447 1.351415 -0.271733
8 3.773295 0.140916 -0.268066
6 -1.800042 0.130223 0.075855

1 -2.171234 0.572614 -0.869642
7 -0.129786 2.550211 -0.294629
1 0.442825 3.383831 -0.317284
1 -1.078372 2.630505 0.050603
8 -2.262033 1.143200 1.055540
1 -1.942762 0.849390 1.924359
8 -2.317685 -1.095076 0.325583
6 2.578150 -2.249787 0.190961
1 3.115320 -2.461185 -0.736374
1 3.298455 -2.166278 1.007236
1 1.883005 -3.062267 0.401615
8 -4.629549 -1.217515 -0.811449
1 -3.716376 -1.161018 -0.349536
1 -4.845524 -0.306422 -1.052994
d_1m5dhmC/d_1m5dhmC_3_042.xyz
6 2.527926 0.173927 -0.158691
6 0.513110 1.360377 -0.169106
6 -0.264179 0.168760 0.034834
6 0.451525 -0.985452 0.144123
1 -0.041991 -1.938029 0.287974
7 1.813200 -1.008716 0.069617
7 1.849096 1.341536 -0.267929
8 3.780358 0.112751 -0.253320
6 -1.795128 0.154532 0.053014
1 -2.153417 0.600815 -0.895104
7 -0.098948 2.559220 -0.302723
1 0.481713 3.387280 -0.323234
1 -1.049889 2.649143 0.033315
8 -2.256848 1.170063 1.030005
1 -1.944859 0.874357 1.900830
8 -2.325703 -1.066515 0.296927
6 2.559137 -2.266308 0.201579
1 3.097533 -2.484423 -0.723525
1 3.277235 -2.189155 1.020413
1 1.854759 -3.071172 0.410590
8 -4.635498 -1.219120 -0.830659

1 -3.725952 -1.142143 -0.363082
1 -5.191392 -0.543395 -0.419074
d_1m5dhmC/d_1m5dhmC_3_016.xyz
6 2.517535 0.141952 -0.174345
6 0.524646 1.365048 -0.188666
6 -0.271899 0.193793 0.054427
6 0.423738 -0.970934 0.179926
1 -0.085141 -1.910592 0.352200
7 1.783455 -1.021800 0.087666
7 1.858911 1.319130 -0.301990
8 3.767534 0.054833 -0.281586
6 -1.802767 0.207991 0.094694
1 -2.166923 0.636781 -0.859246
7 -0.067141 2.571095 -0.345788
1 0.527691 3.387730 -0.395794
1 -1.012587 2.686791 -0.002259
8 -2.231134 1.255338 1.052505
1 -1.914418 0.973583 1.926249
8 -2.350484 -0.997337 0.376442
6 2.506048 -2.291997 0.226466
1 3.026609 -2.532199 -0.703449
1 3.237209 -2.218947 1.034071
1 1.788737 -3.079757 0.455147
8 -4.639994 -1.221635 -0.776031
1 -3.721874 -1.128763 -0.327635
1 -4.457069 -1.498305 -1.684161
d_1m5dhmC/d_1m5dhmC_4_018.xyz
6 -2.435944 0.686295 0.019009
6 -0.152595 1.182687 0.040200
6 0.215512 -0.207507 0.061783
6 -0.821221 -1.090325 0.040131
1 -0.666002 -2.160889 0.056770
7 -2.123226 -0.677620 -0.002053
7 -1.427339 1.592711 0.029217
8 -3.646972 1.026268 0.019939
6 1.682585 -0.615542 0.159221

1 2.031331 -0.371791 1.184618
7 0.815648 2.126424 0.064286
1 0.546352 3.088002 -0.097121
1 1.746473 1.821216 -0.212735
8 2.472876 -0.014274 -0.783068
8 1.716434 -2.072131 0.060514
1 2.654304 -2.310005 0.112757
6 -3.221917 -1.650066 -0.023226
1 -3.821147 -1.561895 0.885960
1 -3.862674 -1.470273 -0.888626
1 -2.800971 -2.653224 -0.086396
8 4.838625 0.236786 0.196366
1 4.778393 0.935420 0.862165
1 3.892143 0.146380 -0.190266
d_1m5dhmC/d_1m5dhmC_3_058.xyz
6 2.319669 -0.161739 0.165075
6 0.368347 -1.439538 0.323246
6 -0.458784 -0.348739 -0.114770
6 0.201176 0.807262 -0.403692
1 -0.332109 1.692366 -0.726405
7 1.555653 0.919200 -0.292272
7 1.697500 -1.329738 0.457570
8 3.562705 -0.011562 0.283512
6 -1.990029 -0.415723 -0.164022
1 -2.345510 -0.690322 0.848693
7 -0.192749 -2.626960 0.647371
1 0.418484 -3.409875 0.838185
1 -1.127558 -2.820122 0.309465
8 -2.384023 -1.610647 -0.937240
1 -2.076336 -1.466516 -1.846997
8 -2.572366 0.711320 -0.640757
6 2.238392 2.179466 -0.606582
1 2.764885 2.550884 0.275077
1 2.960399 2.026248 -1.411563
1 1.493464 2.909625 -0.921120
8 -2.732878 2.492023 1.235583

1 -3.555589 2.317233 1.712564
1 -2.683094 1.777946 0.509098
d_1m5dhmC/d_1m5dhmC_4_071.xyz
6 2.357911 -0.781615 0.018650
6 0.045852 -1.120131 0.020678
6 -0.227191 0.291420 0.052917
6 0.868101 1.101147 0.050147
1 0.786204 2.179756 0.075889
7 2.139883 0.600996 0.016571
7 1.289378 -1.615553 0.015136
8 3.542027 -1.205777 0.016571
6 -1.666837 0.793258 0.132470
1 -2.054860 0.535587 1.140792
7 -0.982088 -1.999422 0.029096
1 -0.771531 -2.974864 -0.136819
1 -1.889539 -1.640790 -0.255659
8 -2.472538 0.279132 -0.847731
8 -1.601593 2.251329 0.086538
1 -2.523117 2.549406 0.109478
6 3.305578 1.493833 0.003989
1 3.923058 1.317910 0.887460
1 3.906998 1.312433 -0.889243
1 2.958145 2.526741 0.003665
8 -4.623739 -0.655732 0.240863
1 -4.539770 -0.457621 1.183555
1 -3.779543 -0.269771 -0.187775
d_1m5dhmC/d_1m5dhmC_4_054.xyz
6 -2.134376 -0.587889 -0.045254
6 0.082508 -1.180049 -0.484477
6 0.518997 0.190094 -0.477264
6 -0.444739 1.111122 -0.199969
1 -0.231471 2.170760 -0.155916
7 -1.743275 0.753700 0.032595
7 -1.194356 -1.533463 -0.291463
8 -3.347248 -0.873236 0.128408
6 1.979013 0.535779 -0.773878

1 2.163321 0.327355 -1.846709
7 0.981572 -2.161472 -0.724713
1 0.687882 -3.119530 -0.586295
1 1.958762 -1.921226 -0.573805
8 2.867734 -0.150101 0.011886
8 2.096560 1.982076 -0.614532
1 3.020185 2.191357 -0.818797
6 -2.761310 1.765367 0.339047
1 -3.533512 1.768423 -0.433396
1 -3.224668 1.545743 1.303254
1 -2.282887 2.743326 0.381168
8 1.935151 -0.388667 2.432377
1 0.974643 -0.401795 2.315402
1 2.302017 -0.282883 1.485824
d_1m5dhmC/d_1m5dhmC_3_031.xyz
6 -2.262738 -0.317184 0.153503
6 -0.249546 -1.500786 0.032074
6 0.491803 -0.329290 -0.347701
6 -0.237884 0.817392 -0.446412
1 0.223084 1.751191 -0.741509
7 -1.576956 0.854456 -0.191376
7 -1.569529 -1.476283 0.263172
8 -3.502311 -0.251820 0.354842
6 1.983262 -0.348096 -0.709577
1 2.120347 -1.050798 -1.552773
7 0.374852 -2.696082 0.139257
1 -0.145774 -3.469063 0.532476
1 1.382893 -2.694233 0.241335
8 2.712866 -1.054544 0.371316
1 2.611345 -0.513309 1.171227
8 2.504885 0.878074 -0.957501
6 -2.337454 2.104694 -0.307796
1 -3.096233 2.012378 -1.088089
1 -2.828821 2.332020 0.640487
1 -1.649439 2.910911 -0.560739
8 2.363087 2.338054 1.207765

1 1.429674 2.336549 1.461265
1 2.401481 1.771225 0.363017
d_1m5dhmC/d_1m5dhmC_3_060.xyz
6 -2.294880 0.250045 -0.175701
6 -0.320177 1.492727 -0.026167
6 0.451245 0.342138 0.355316
6 -0.243045 -0.827100 0.438376
1 0.244832 -1.748305 0.729894
7 -1.577752 -0.902360 0.170242
7 -1.636465 1.430432 -0.270673
8 -3.529715 0.148239 -0.391450
6 1.937978 0.402759 0.725853
1 2.054924 1.124829 1.555589
7 0.270731 2.706302 -0.121768
1 -0.267140 3.465906 -0.517857
1 1.278908 2.734676 -0.216107
8 2.656007 1.105739 -0.366325
1 2.546953 0.562022 -1.163520
8 2.486759 -0.805063 1.001790
6 -2.302507 -2.174174 0.278363
1 -3.075796 -2.101999 1.046436
1 -2.772609 -2.418919 -0.676326
1 -1.594829 -2.958352 0.545355
8 2.528089 -2.280837 -1.154391
1 2.500251 -1.701757 -0.317416
1 3.188109 -1.861727 -1.723886
d_1m5dhmC/d_1m5dhmC_4_026.xyz
6 2.135878 -0.576687 0.044496
6 -0.076140 -1.210394 0.448668
6 -0.528749 0.153786 0.487921
6 0.422989 1.094631 0.237618
1 0.195903 2.152157 0.220343
7 1.726225 0.760817 -0.003141
7 1.206983 -1.541024 0.258797
8 3.353848 -0.840525 -0.126971
6 -1.995321 0.470985 0.782935

1 -2.192038 0.200871 1.839476
7 -0.965957 -2.211475 0.639065
1 -0.658314 -3.158661 0.460563
1 -1.943737 -1.979056 0.482515
8 -2.866629 -0.177104 -0.052895
8 -2.123692 1.923096 0.703225
1 -3.052299 2.112771 0.904304
6 2.728583 1.792031 -0.295242
1 3.506146 1.789973 0.471734
1 3.187674 1.597875 -1.267051
1 2.236859 2.764131 -0.314027
8 -1.863614 -0.219329 -2.457690
1 -0.908699 -0.179347 -2.305112
1 -2.264996 -0.194159 -1.519523
d_1m5dhmC/d_1m5dhmC_4_087_b.xyz
6 -2.132670 -0.614383 -0.050155
6 0.095173 -1.116561 -0.543879
6 0.495654 0.261731 -0.448379
6 -0.493784 1.138788 -0.122301
1 -0.310330 2.200203 -0.022085
7 -1.781072 0.732580 0.091298
7 -1.168900 -1.517266 -0.356157
8 -3.334756 -0.943975 0.120303
6 1.939482 0.670836 -0.745935
1 2.094291 0.582946 -1.840175
7 1.013750 -2.054146 -0.868026
1 0.747642 -3.027086 -0.791664
1 1.988154 -1.794379 -0.726338
8 2.867956 -0.070916 -0.065611
8 2.032073 2.095210 -0.435828
1 2.946368 2.344275 -0.637258
6 -2.830709 1.699339 0.434729
1 -3.592029 1.721376 -0.348260
1 -3.301823 1.418901 1.378968
1 -2.379909 2.686372 0.533845
8 2.104353 -0.715390 2.332307

1 2.387373 -0.440836 1.390439
1 1.177917 -0.977875 2.238881
d_1m5dhmC/d_1m5dhmC_3_002.xyz
6 2.282067 -0.236565 0.141237
6 0.299592 -1.462491 0.321911
6 -0.504306 -0.348073 -0.099155
6 0.182992 0.788343 -0.401718
1 -0.329202 1.687075 -0.720210
7 1.541540 0.862684 -0.311334
7 1.632982 -1.388554 0.438356
8 3.529407 -0.117886 0.249473
6 -2.037538 -0.374388 -0.121252
1 -2.381290 -0.654966 0.893947
7 -0.288794 -2.636279 0.647244
1 0.302916 -3.436181 0.828650
1 -1.233414 -2.802800 0.322884
8 -2.475289 -1.547432 -0.904029
1 -2.177293 -1.399082 -1.816339
8 -2.598831 0.774305 -0.571372
6 2.253060 2.101628 -0.645360
1 2.795034 2.471351 0.227589
1 2.964417 1.920479 -1.453932
1 1.523968 2.845922 -0.964093
8 -2.319454 2.649567 1.212920
1 -3.170784 2.715451 1.666235
1 -2.436313 1.908636 0.525493
d_1m5dhmC/d_1m5dhmC_3_072.xyz
6 2.245573 0.329472 -0.122484
6 0.217079 1.475729 -0.313894
6 -0.545017 0.327141 0.092863
6 0.185594 -0.779551 0.403947
1 -0.292366 -1.697199 0.721924
7 1.546880 -0.798331 0.326825
7 1.553139 1.455996 -0.419157
8 3.496865 0.259685 -0.226902
6 -2.078219 0.289883 0.092747

1 -2.417559 0.569136 -0.924343
7 -0.414320 2.627526 -0.635967
1 0.147725 3.450942 -0.806151
1 -1.368777 2.753449 -0.322385
8 -2.574387 1.434656 0.882429
1 -2.286214 1.285230 1.797657
8 -2.598354 -0.886325 0.520361
6 2.305886 -2.005926 0.672536
1 2.865917 -2.360415 -0.195297
1 3.005201 -1.789916 1.483005
1 1.605464 -2.776225 0.994140
8 -2.143213 -2.739750 -1.262107
1 -1.180486 -2.784860 -1.342947
1 -2.311461 -2.013092 -0.572054
d_1m5dhmC/d_1m5dhmC_4_087.xyz
6 -2.340347 -0.806534 -0.001048
6 -0.022998 -1.104607 -0.049423
6 0.224527 0.311384 -0.081143
6 -0.884706 1.101603 -0.058698
1 -0.821328 2.181436 -0.083467
7 -2.146625 0.579409 -0.004243
7 -1.257320 -1.621836 -0.019724
8 -3.516712 -1.251114 0.025834
6 1.653322 0.841367 -0.172433
1 2.045078 0.579189 -1.177347
7 1.018217 -1.968393 -0.085201
1 0.824751 -2.947686 0.079061
1 1.926740 -1.603590 0.184054
8 2.470094 0.356570 0.813771
8 1.557734 2.298145 -0.143010
1 2.472710 2.615507 -0.170389
6 -3.326872 1.452149 0.032360
1 -3.957537 1.267613 -0.840024
1 -3.908597 1.259143 0.936165
1 -2.996708 2.490646 0.028239
8 4.631674 -0.628317 -0.187250

1 3.774678 -0.229132 0.205379
1 4.380447 -1.511825 -0.489940
d_1m5dhmC/d_1m5dhmC_4_098.xyz
6 -2.374541 -0.772773 -0.009698
6 -0.065088 -1.126241 -0.047375
6 0.216848 0.283679 -0.075000
6 -0.872986 1.100449 -0.052240
1 -0.784442 2.178614 -0.072723
7 -2.147477 0.608391 -0.003072
7 -1.311574 -1.613572 -0.026569
8 -3.561168 -1.189399 0.007931
6 1.658764 0.777057 -0.169847
1 2.032724 0.527054 -1.184454
7 0.956878 -2.012142 -0.076752
1 0.741941 -2.986992 0.086856
1 1.869150 -1.660355 0.201536
8 2.472363 0.248306 0.796102
8 1.602956 2.234968 -0.108381
1 2.525317 2.528464 -0.148832
6 -3.306974 1.508629 0.033816
1 -3.937412 1.343975 -0.842666
1 -3.897269 1.323379 0.933641
1 -2.952688 2.539182 0.037933
8 4.646192 -0.675677 -0.234297
1 3.796855 -0.276726 0.175113
1 5.302139 0.034276 -0.210419
d_1m5dhmC/d_1m5dhmC_4_064.xyz
6 -2.485388 0.593850 0.017962
6 -0.237567 1.231050 0.055825
6 0.215651 -0.134785 0.063082
6 -0.764824 -1.079375 0.026616
1 -0.544590 -2.138535 0.035204
7 -2.089648 -0.747221 -0.018102
7 -1.535843 1.561633 0.040885
8 -3.715645 0.857431 0.020619
6 1.702545 -0.459090 0.177501

1 2.013389 -0.247898 1.222110
7 0.669160 2.231816 0.096557
1 0.343234 3.179430 -0.041382
1 1.622940 1.984680 -0.161734
8 2.478451 0.231136 -0.714408
8 1.824577 -1.905644 0.010409
1 2.769074 -2.098779 0.108765
6 -3.126637 -1.784811 -0.042588
1 -3.719178 -1.747155 0.874663
1 -3.786434 -1.631885 -0.898620
1 -2.644201 -2.758316 -0.126395
8 4.893351 0.136173 0.195962
1 5.033235 -0.790872 0.434258
1 3.938292 0.171927 -0.173285
d_1m5dhmC/d_1m5dhmC_3_078.xyz
6 2.525917 0.523815 0.018215
6 0.305475 1.243790 -0.123638
6 -0.198790 -0.102762 -0.128662
6 0.745920 -1.080554 -0.042662
1 0.470484 -2.127732 -0.051307
7 2.078064 -0.802370 0.048929
7 1.614702 1.523950 -0.056704
8 3.763666 0.742507 0.070545
6 -1.679093 -0.459494 -0.322368
1 -2.024223 0.028229 -1.257110
7 -0.545947 2.290782 -0.217182
1 -0.169674 3.221278 -0.091796
1 -1.521082 2.132311 0.005026
8 -2.461645 0.348666 0.687719
1 -2.232432 -0.014662 1.559101
8 -1.960723 -1.764674 -0.247306
6 3.073129 -1.879309 0.113016
1 3.710921 -1.854413 -0.773678
1 3.695163 -1.760696 1.002184
1 2.549627 -2.833674 0.160178
8 -5.083416 -0.126436 0.002113

1 -4.149730 0.032549 0.281318
1 -5.055199 -0.983138 -0.447185
d_1m5dhmC/d_1m5dhmC_3_000.xyz
6 -2.528502 -0.516266 0.031306
6 -0.312988 -1.247080 -0.132132
6 0.197924 0.096936 -0.141784
6 -0.741747 1.079539 -0.054869
1 -0.461462 2.125342 -0.069138
7 -2.074651 0.808180 0.044222
7 -1.622886 -1.520751 -0.051568
8 -3.766179 -0.729020 0.105366
6 1.679247 0.447234 -0.340399
1 2.019497 -0.038806 -1.277725
7 0.532144 -2.298338 -0.235127
1 0.152602 -3.226961 -0.105798
1 1.510119 -2.144986 -0.021977
8 2.461152 -0.367854 0.664761
1 2.227148 -0.014449 1.538970
8 1.966845 1.750899 -0.261978
6 -3.063675 1.890166 0.115247
1 -3.744157 1.831662 -0.736980
1 -3.642485 1.810645 1.037862
1 -2.536610 2.843532 0.097985
8 5.090201 0.141654 0.036922
1 5.083112 1.055196 -0.282380
1 4.152285 -0.033420 0.291709
d_1m5dhmC/d_1m5dhmC_3_012.xyz
6 -2.302381 -0.864992 0.031728
6 0.023503 -1.048619 -0.139745
6 0.198748 0.377747 -0.162249
6 -0.947323 1.109182 -0.079747
1 -0.922395 2.191745 -0.102234
7 -2.176750 0.529473 0.031035
7 -1.183772 -1.625570 -0.049113
8 -3.454498 -1.364810 0.111684
6 1.560924 1.065039 -0.324439

1 2.078482 0.597726 -1.187446
7 1.088265 -1.875992 -0.244633
1 0.934963 -2.868142 -0.122763
1 2.018357 -1.517886 -0.079659
8 2.429975 0.564842 0.796918
1 2.077347 0.938096 1.620792
8 1.517508 2.403540 -0.374089
6 -3.393482 1.342886 0.132473
1 -4.052719 1.139494 -0.714300
1 -3.923523 1.109108 1.058178
1 -3.112488 2.395573 0.130371
8 4.714386 -0.788707 0.063241
1 4.366389 -1.325855 -0.662694
1 3.942493 -0.261613 0.368580
d_1m5dhmC/d_1m5dhmC_3_025.xyz
6 -1.632303 0.655930 0.016938
6 0.086866 -0.938346 -0.138228
6 1.107030 0.072660 -0.140497
6 0.658655 1.356886 -0.062205
1 1.349922 2.190554 -0.073797
7 -0.667977 1.667536 0.025439
7 -1.218498 -0.633890 -0.065854
8 -2.846831 0.964904 0.092058
6 2.602710 -0.237733 -0.313316
1 2.712325 -0.826002 -1.247987
7 0.410447 -2.244627 -0.239354
1 -0.323212 -2.933658 -0.137224
1 1.363228 -2.516760 -0.030319
8 2.958817 -1.281622 0.698511
1 2.858337 -0.859967 1.567529
8 3.409329 0.834811 -0.248392
6 -1.121374 3.060367 0.119562
1 -1.766697 3.301732 -0.727733
1 -1.679861 3.208480 1.046130
1 -0.249456 3.713564 0.111603
8 -3.217359 -2.595408 0.015865

1 -2.521568 -1.887095 -0.013497
1 -4.051968 -2.114049 0.105803
d_1m5dhmU/d_1m5dhmU_a1_8_acw.xyz
6 -1.784509 0.290032 -0.042099
6 0.323588 -0.793005 0.001615
6 0.989318 0.496923 0.066616
6 0.214819 1.612489 0.093651
1 0.637498 2.610083 0.152114
7 -1.148929 1.537706 0.045811
7 -1.031813 -0.835985 -0.058883
8 -3.040899 0.261590 -0.099772
8 1.005682 -1.871926 0.008990
6 2.495422 0.569612 0.085198
1 2.832686 1.581228 0.318467
8 3.055590 0.273281 -1.197836
1 2.717887 -0.593907 -1.477931
6 -1.980691 2.745580 0.081840
1 -2.589425 2.810242 -0.822555
1 -2.641109 2.721962 0.951481
1 -1.330745 3.618027 0.144284
8 3.047259 -0.285439 1.075606
1 2.597588 -1.147284 0.956604
8 -2.221714 -3.335203 -0.018025
1 -1.476507 -3.950145 0.035047
1 -1.797701 -2.430452 -0.038622
d_1m5dhmU/d_1m5dhmU_a1_8_aah.xyz
6 -1.731859 -0.345852 -0.054009
6 0.321816 0.837161 -0.124462
6 1.050996 -0.419523 -0.085431
6 0.331163 -1.571359 -0.056004
1 0.803830 -2.547563 -0.034717
7 -1.035325 -1.563731 -0.048677
7 -1.035127 0.814671 -0.106891
8 -2.988896 -0.373162 -0.015076
8 0.947908 1.948478 -0.183421
6 2.559852 -0.430447 -0.043540

1 2.946765 -1.425132 -0.273080
8 3.057921 -0.136061 1.267089
1 2.672607 0.707070 1.557414
6 -1.806799 -2.811714 -0.004391
1 -2.409315 -2.848836 0.905902
1 -2.467677 -2.875649 -0.871424
1 -1.115019 -3.653923 -0.015118
8 3.114717 0.460752 -0.996953
1 2.596357 1.289736 -0.907928
8 -2.558318 3.099418 0.245245
1 -2.004800 2.280498 0.100250
1 -3.467253 2.772218 0.299620
d_1m5dhmU/d_1m5dhmU_f2_12_acd.xyz
6 -1.816659 0.851275 0.074695
6 0.513040 0.512900 0.018614
6 0.317742 -0.919744 -0.109258
6 -0.961372 -1.377208 -0.162969
1 -1.194518 -2.431444 -0.270550
7 -2.026154 -0.529700 -0.082155
7 -0.554181 1.336832 0.110013
8 -2.831166 1.591866 0.170859
8 1.703939 0.996535 0.027558
6 1.486164 -1.870040 -0.159656
1 1.190143 -2.828373 -0.598986
8 1.928914 -2.083725 1.187623
1 2.645463 -2.738029 1.160218
6 -3.404010 -1.030437 -0.136979
1 -3.929796 -0.788369 0.789267
1 -3.936045 -0.574321 -0.974517
1 -3.379236 -2.112076 -0.268541
8 2.547322 -1.378450 -0.958763
1 2.623993 -0.426094 -0.722431
8 2.152054 3.674022 0.002321
1 1.306624 4.136638 0.085623
1 1.917781 2.709693 0.021448
d_1m5dhmU/d_1m5dhmU_a1_8_ado.xyz

6 1.731541 -0.339069 -0.066445
6 -0.327380 0.834406 -0.134603
6 -1.050551 -0.424949 -0.075321
6 -0.326235 -1.573532 -0.037862
1 -0.794515 -2.551429 -0.001353
7 1.040466 -1.559811 -0.041882
7 1.029785 0.818155 -0.128492
8 2.988917 -0.360593 -0.034900
8 -0.960507 1.941104 -0.199944
6 -2.558749 -0.438116 -0.025578
1 -2.943997 -1.440988 -0.219327
8 -3.120915 0.417222 -1.007975
1 -2.614115 1.254598 -0.943202
6 1.817582 -2.804055 0.007207
1 2.479095 -2.867540 -0.859300
1 2.419836 -2.836344 0.917919
1 1.129702 -3.649475 -0.001925
8 -3.050566 -0.097298 1.275576
1 -2.670198 0.760300 1.528236
8 2.561493 3.095079 0.243159
1 2.002087 2.282023 0.089430
1 3.435716 2.748228 0.470416
d_1m5dhmU/d_1m5dhmU_f2_12_abh.xyz
6 -1.537982 0.257400 0.030269
6 0.463936 -0.981767 0.074745
6 1.233005 0.251530 0.109567
6 0.547668 1.424572 0.130055
1 1.048699 2.385946 0.168382
7 -0.816055 1.456470 0.099804
7 -0.891356 -0.924190 0.030592
8 -2.800207 0.343595 -0.025028
8 1.056767 -2.115646 0.100938
6 2.740323 0.235664 0.092187
1 3.146399 1.168442 0.497341
8 3.154787 0.097060 -1.274472
1 4.122942 0.164041 -1.294218

6 -1.550092 2.727395 0.116376
1 -2.136701 2.834989 -0.798556
1 -2.224164 2.759874 0.974795
1 -0.833495 3.545585 0.186938
8 3.274771 -0.810788 0.881644
1 2.692859 -1.586174 0.697234
8 -4.391849 -1.853698 -0.223511
1 -3.740636 -1.111104 -0.146291
1 -3.859332 -2.660506 -0.194171
d_1m5dhmU/d_1m5dhmU_a1_8_abz.xyz
6 -1.717982 0.372161 -0.048115
6 0.309984 -0.855342 0.005682
6 1.062293 0.384444 0.104950
6 0.363073 1.549228 0.140691
1 0.853276 2.513397 0.224708
7 -1.000404 1.571761 0.069030
7 -1.043821 -0.802430 -0.071471
8 -2.972248 0.429902 -0.124122
8 0.911810 -1.982272 0.001668
6 2.572210 0.375314 0.136966
1 2.957012 1.324323 0.515780
8 3.131943 0.255307 -1.176854
1 2.745375 -0.526557 -1.603576
6 -1.746665 2.834790 0.106184
1 -2.326406 2.955446 -0.811473
1 -2.428509 2.843590 0.959277
1 -1.039207 3.658625 0.199064
8 3.070166 -0.644291 0.985844
1 2.519820 -1.435326 0.788745
8 -2.688746 -3.032639 -0.041842
1 -2.076535 -2.243565 -0.060992
1 -3.576125 -2.646869 -0.030134
d_1m5dhmU/d_1m5dhmU_a1_8_ace.xyz
6 -1.787513 -0.272491 -0.058111
6 0.334475 0.783636 -0.078646
6 0.984028 -0.513724 0.002150

6 0.197874 -1.620070 0.043483
1 0.608606 -2.622478 0.103706
7 -1.165788 -1.528218 0.010866
7 -1.021752 0.844154 -0.108597
8 -3.044820 -0.227908 -0.073343
8 1.034442 1.848640 -0.121844
6 2.487886 -0.594476 0.044652
1 2.819375 -1.632654 0.103189
8 3.016486 0.050670 1.201150
1 2.645524 0.950721 1.218914
6 -2.010947 -2.726020 0.062318
1 -2.642751 -2.704515 0.953143
1 -2.649274 -2.773536 -0.822484
1 -1.370051 -3.606814 0.093780
8 3.080577 -0.060785 -1.136609
1 2.713868 0.832905 -1.259162
8 -2.190837 3.340962 0.129526
1 -1.442178 3.952113 0.177024
1 -1.771937 2.438940 0.029879
d_1m5dhmU/d_1m5dhmU_a1_8_abd.xyz
6 -1.772737 -0.330317 0.046687
6 0.301833 0.816590 -0.011775
6 1.005909 -0.451383 -0.104905
6 0.261770 -1.588350 -0.137902
1 0.713871 -2.571439 -0.218534
7 -1.101727 -1.557207 -0.067956
7 -1.053012 0.816287 0.066815
8 -3.028108 -0.339832 0.123464
8 0.944537 1.921170 -0.014690
6 2.514974 -0.500065 -0.133752
1 2.863929 -1.464242 -0.508983
8 3.076715 -0.397164 1.180579
1 2.717542 0.398419 1.605892
6 -1.898099 -2.789171 -0.104514
1 -2.488479 -2.881649 0.809512
1 -2.573999 -2.774733 -0.962315

1 -1.224453 -3.641727 -0.188370
8 3.053006 0.497193 -0.984944
1 2.532757 1.309307 -0.791829
8 -2.348822 3.265076 0.058579
1 -1.881585 2.382103 0.061548
1 -1.636946 3.915029 -0.025872
d_1m5dhmU/d_1m5dhmU_a1_8_adu.xyz
6 -1.806190 0.873264 0.011413
6 0.513504 0.484980 -0.043482
6 0.289563 -0.948642 -0.063726
6 -0.996721 -1.385989 -0.057900
1 -1.252463 -2.440169 -0.077540
7 -2.044229 -0.510812 -0.024726
7 -0.533630 1.336023 -0.004355
8 -2.803716 1.641750 0.053664
8 1.718342 0.930782 -0.071205
6 1.458196 -1.901976 -0.067204
1 1.125076 -2.924362 -0.254984
8 2.108602 -1.946560 1.206047
1 2.339539 -1.036137 1.455066
6 -3.432403 -0.985937 -0.020232
1 -3.940867 -0.655143 0.888027
1 -3.967139 -0.592518 -0.887396
1 -3.430747 -2.075334 -0.058840
8 2.391228 -1.583885 -1.090012
1 2.550412 -0.621450 -1.011501
8 2.224217 3.590088 0.077384
1 1.386461 4.065580 0.165520
1 1.969660 2.631460 0.024972
d_1m5dhmU/d_1m5dhmU_f2_12_adm.xyz
6 -1.816811 0.851164 0.078518
6 0.512952 0.512831 0.025121
6 0.317772 -0.919245 -0.109109
6 -0.961276 -1.376365 -0.167122
1 -1.194344 -2.430104 -0.279652
7 -2.026132 -0.529107 -0.084608

7 -0.554351 1.336470 0.118156
8 -2.831426 1.591420 0.176089
8 1.703899 0.996280 0.038121
6 1.486179 -1.869430 -0.161692
1 1.190703 -2.825922 -0.605377
8 1.927047 -2.088716 1.185291
1 2.643040 -2.743562 1.156241
6 -3.403935 -1.029473 -0.144061
1 -3.931536 -0.790715 0.782006
1 -3.934238 -0.570244 -0.981002
1 -3.379034 -2.110627 -0.279485
8 2.548499 -1.374641 -0.957287
1 2.624868 -0.423265 -0.716963
8 2.152958 3.673189 -0.009218
1 1.918539 2.709098 0.018470
1 1.308289 4.136660 0.077044
d_1m5dhmU/d_1m5dhmU_f2_12_abi.xyz
6 1.774775 -0.344812 -0.034981
6 -0.286052 0.828911 0.034023
6 -1.005119 -0.431129 0.116636
6 -0.274777 -1.577218 0.142944
1 -0.740574 -2.554415 0.216255
7 1.088255 -1.564221 0.075545
7 1.069670 0.810094 -0.045062
8 3.029567 -0.370675 -0.116122
8 -0.908188 1.944585 0.050748
6 -2.511467 -0.468345 0.141347
1 -2.872604 -1.408661 0.570783
8 -2.968183 -0.362423 -1.214130
1 -3.934639 -0.451015 -1.203503
6 1.868544 -2.806762 0.102493
1 2.454035 -2.902041 -0.814409
1 2.547974 -2.805274 0.957555
1 1.184250 -3.651006 0.184835
8 -3.061369 0.569406 0.932404
1 -2.523396 1.366157 0.715481

8 2.383079 3.245865 -0.116566
1 1.674579 3.901608 -0.050123
1 1.910226 2.365601 -0.095694
d_1m5dhmU/d_1m5dhmU_a1_8_ade.xyz
6 -1.395080 -0.431940 -0.503313
6 0.846510 -1.068494 -0.174357
6 1.202493 0.309955 0.120880
6 0.209559 1.236910 0.109026
1 0.388523 2.284234 0.327854
7 -1.079424 0.895828 -0.187490
7 -0.437767 -1.380679 -0.480757
8 -2.597812 -0.695132 -0.802916
8 1.742386 -1.981109 -0.134819
6 2.636796 0.693839 0.397012
1 2.692823 1.682526 0.856884
8 3.397473 0.807681 -0.811890
1 3.308525 -0.024142 -1.305425
6 -2.144624 1.905757 -0.203618
1 -2.612191 1.941861 -1.189677
1 -2.905247 1.666165 0.541656
1 -1.706730 2.876843 0.026511
8 3.256394 -0.216511 1.289762
1 2.988338 -1.107070 0.969849
8 -4.663583 -0.414290 1.000439
1 -4.838046 -1.323738 1.280117
1 -3.908435 -0.484011 0.371579
d_1m5dhmU/d_1m5dhmU_f2_12_acs.xyz
6 -1.775967 -0.342647 0.032220
6 0.286509 0.828153 -0.038185
6 1.003912 -0.433161 -0.116376
6 0.272038 -1.578317 -0.140702
1 0.736542 -2.556367 -0.210800
7 -1.091083 -1.563235 -0.075164
7 -1.069330 0.811346 0.039655
8 -3.030832 -0.366585 0.112980
8 0.910194 1.942888 -0.057637

6 2.510225 -0.472529 -0.138614
1 2.870696 -1.415004 -0.563843
8 2.964967 -0.361941 1.217135
1 3.931319 -0.451914 1.208432
6 -1.873091 -2.804739 -0.099576
1 -2.458173 -2.897781 0.817833
1 -2.553031 -2.803640 -0.954218
1 -1.190025 -3.650054 -0.181036
8 3.062847 0.561364 -0.932850
1 2.526108 1.359830 -0.719464
8 -2.371868 3.252772 0.123593
1 -1.907787 2.368059 0.095987
1 -1.656977 3.901540 0.057294
d_1m5dhmU/d_1m5dhmU_f2_12_aax.xyz
6 1.720061 0.386416 0.048087
6 -0.292995 -0.867739 -0.013389
6 -1.059556 0.362435 -0.118556
6 -0.374066 1.535344 -0.156824
1 -0.876670 2.492613 -0.245997
7 0.987786 1.576505 -0.081974
7 1.060583 -0.795671 0.070176
8 2.972734 0.461575 0.135133
8 -0.873666 -2.005297 -0.014300
6 -2.566265 0.342397 -0.153987
1 -2.959155 1.258548 -0.607146
8 -3.028959 0.251307 1.200736
1 -3.997627 0.308936 1.182027
6 1.716750 2.849473 -0.120928
1 2.280526 2.988244 0.804171
1 2.411402 2.858533 -0.963526
1 0.999030 3.661781 -0.234040
8 -3.071191 -0.732773 -0.924482
1 -2.501778 -1.502769 -0.689894
8 2.718076 -3.015601 0.073772
1 2.102184 -2.228961 0.080416
1 3.603968 -2.626401 0.083288

d_1m5dhmU/d_1m5dhmU_f2_12_acg.xyz
6 -1.816228 0.852964 0.054543
6 0.512859 0.513188 -0.016071
6 0.316575 -0.921973 -0.109640
6 -0.962938 -1.380432 -0.139995
1 -1.196845 -2.436866 -0.220891
7 -2.027030 -0.531259 -0.067779
7 -0.553531 1.339003 0.066710
8 -2.829818 1.595821 0.142539
8 1.703666 0.996971 -0.029032
6 1.484871 -1.873049 -0.148483
1 1.185587 -2.841159 -0.563485
8 1.938500 -2.055768 1.199810
1 2.657857 -2.707279 1.181270
6 -3.405270 -1.032895 -0.099975
1 -3.923554 -0.769903 0.824778
1 -3.944112 -0.595734 -0.943262
1 -3.381488 -2.117255 -0.207314
8 2.539293 -1.399587 -0.967127
1 2.618360 -0.442311 -0.752342
8 2.153837 3.671313 0.062615
1 1.306848 4.132180 0.139446
1 1.918232 2.707301 0.036582
d_1m5dhmU/d_1m5dhmU_f2_12_aav.xyz
6 -1.398074 0.589861 -0.394704
6 0.879438 1.110485 -0.093846
6 1.212146 -0.304701 -0.118343
6 0.200335 -1.189249 -0.318132
1 0.369469 -2.259829 -0.366406
7 -1.091431 -0.775750 -0.468315
7 -0.416321 1.495493 -0.221056
8 -2.613782 0.926789 -0.511053
8 1.800382 1.990512 0.029867
6 2.627652 -0.779942 0.089266
1 2.766697 -1.785821 -0.321041
8 2.870487 -0.810172 1.503036

1 3.761296 -1.171397 1.638487
6 -2.174415 -1.740328 -0.697209
1 -2.862383 -1.748990 0.150388
1 -2.726002 -1.472619 -1.600302
1 -1.738030 -2.731428 -0.819205
8 3.572192 0.052002 -0.558752
1 3.249697 0.970209 -0.396038
8 -4.576588 0.066902 1.225538
1 -4.910392 0.886115 1.616974
1 -3.860114 0.350543 0.611464
d_1m5dhmU/d_1m5dhmU_a1_8_acq.xyz
6 1.533018 0.256755 0.053510
6 -0.474645 -0.970016 0.003089
6 -1.234505 0.264790 -0.107007
6 -0.541375 1.432497 -0.150374
1 -1.033208 2.394975 -0.242834
7 0.821826 1.456372 -0.076336
7 0.878345 -0.920916 0.082678
8 2.794236 0.336393 0.135968
8 -1.081547 -2.097196 0.013748
6 -2.744552 0.249289 -0.140418
1 -3.133602 1.192324 -0.529860
8 -3.304309 0.141691 1.174876
1 -2.915614 -0.635058 1.608968
6 1.565988 2.720634 -0.122239
1 2.138149 2.853698 0.798383
1 2.254251 2.720916 -0.970042
1 0.857122 3.541441 -0.230555
8 -3.236461 -0.783052 -0.976891
1 -2.676183 -1.566073 -0.770477
8 4.379769 -1.870279 0.030514
1 3.730176 -1.123516 0.074953
1 3.840636 -2.671046 -0.028014
d_1m5dhmU/d_1m5dhmU_a1_8_aaf.xyz
6 2.106930 0.800092 0.065696
6 -0.212257 0.855680 0.469172

6 -0.305863 -0.576018 0.252797
6 0.844254 -1.235629 -0.052391
1 0.868327 -2.306206 -0.226725
7 2.036854 -0.583800 -0.162529
7 0.980010 1.481989 0.376984
8 3.232936 1.357737 -0.028249
8 -1.269947 1.534591 0.761138
6 -1.618609 -1.329283 0.352306
1 -1.426794 -2.380764 0.578893
8 -2.337633 -1.347108 -0.879186
1 -2.513779 -0.420735 -1.160346
6 3.267440 -1.307714 -0.499856
1 3.708245 -0.896103 -1.410117
1 3.991369 -1.222715 0.313667
1 3.023056 -2.358045 -0.658519
8 -2.424652 -0.822331 1.405089
1 -2.315793 0.155704 1.371240
8 -2.810506 1.374117 -1.493953
1 -3.740382 1.560806 -1.300204
1 -2.325259 1.561927 -0.654125
d_1m5dhmU/d_1m5dhmU_a1_8_aad.xyz
6 1.804577 0.868193 -0.044958
6 -0.518477 0.495909 0.014186
6 -0.304261 -0.937224 0.096821
6 0.980796 -1.379740 0.119840
1 1.229852 -2.433506 0.188772
7 2.033626 -0.514380 0.054410
7 0.534785 1.337559 -0.055469
8 2.807233 1.627574 -0.116234
8 -1.719123 0.956473 0.019542
6 -1.468350 -1.898216 0.127988
1 -1.146221 -2.884664 0.467386
8 -2.010234 -2.121109 -1.179781
1 -2.216936 -1.259204 -1.576865
6 3.418596 -0.997964 0.080757
1 3.935227 -0.710627 -0.837614

1 3.949807 -0.569754 0.933479
1 3.409476 -2.084482 0.167364
8 -2.481511 -1.465627 1.020395
1 -2.587897 -0.504876 0.845714
8 -2.188301 3.629492 -0.061026
1 -1.346048 4.097987 0.021984
1 -1.947932 2.666705 -0.033786
d_1m5dhmU/d_1m5dhmU_a1_8_aas.xyz
6 1.522833 0.241496 0.079225
6 -0.490189 -0.975625 0.022783
6 -1.242882 0.261465 -0.108462
6 -0.543653 1.425185 -0.158640
1 -1.029738 2.388929 -0.267308
7 0.819038 1.443058 -0.071959
7 0.862135 -0.932328 0.116083
8 2.783788 0.316216 0.173693
8 -1.102923 -2.099588 0.039218
6 -2.752544 0.251716 -0.158632
1 -3.133255 1.193574 -0.559024
8 -3.328313 0.154977 1.150470
1 -2.954272 -0.625186 1.591357
6 1.569905 2.702976 -0.126337
1 2.133499 2.845134 0.798206
1 2.266475 2.689274 -0.967226
1 0.866397 3.525908 -0.252170
8 -3.238579 -0.784500 -0.993913
1 -2.685581 -1.568741 -0.773305
8 4.337366 -1.920374 0.043821
1 3.735771 -1.134642 0.084023
1 5.040968 -1.666480 -0.569354
d_1m5fC/d_1m5fC_2_082.xyz
6 -1.245996 -1.189140 0.062770
6 0.834307 -0.048993 0.074814
6 0.121462 1.237520 -0.003965
6 -1.257808 1.206522 -0.061574
1 -1.836484 2.123536 -0.124807

7 -1.948795 0.060142 -0.039956
7 0.084940 -1.203093 0.116903
8 -1.962491 -2.224491 0.093892
7 2.139915 -0.156103 0.106476
1 2.571488 0.764979 0.065819
6 0.737053 2.537892 -0.022908
1 0.030878 3.385946 -0.087755
8 1.960036 2.774893 0.029619
6 -3.415309 0.041859 -0.096203
1 -3.747041 -0.534011 -0.962080
1 -3.817975 -0.413707 0.810471
1 -3.776623 1.066856 -0.180976
8 3.519506 -2.459556 -0.130751
1 2.891570 -3.131312 -0.430705
1 2.973098 -1.609632 -0.033616
d_1m5fC/d_1m5fC_2_056.xyz
6 1.245094 -1.193162 -0.059546
6 -0.832373 -0.047527 -0.070287
6 -0.115686 1.237360 0.001186
6 1.263537 1.202691 0.058291
1 1.844777 2.118365 0.117590
7 1.951334 0.054287 0.041465
7 -0.085857 -1.203477 -0.112782
8 1.958631 -2.230594 -0.090380
7 -2.138403 -0.151308 -0.095453
1 -2.567034 0.771350 -0.058912
6 -0.727425 2.539628 0.015062
1 -0.018682 3.385917 0.074789
8 -1.949772 2.780024 -0.036663
6 3.417778 0.031997 0.098775
1 3.747152 -0.543033 0.966118
1 3.819921 -0.426544 -0.806619
1 3.781821 1.056156 0.181950
8 -3.542410 -2.438946 0.178511
1 -2.987610 -1.598311 0.056494
1 -2.947005 -3.170209 -0.036653

d_1m5fC/d_1m5fC_2_023.xyz
6 -1.253082 -1.204806 -0.063429
6 0.820074 -0.052310 -0.075437
6 0.099554 1.230110 0.000924
6 -1.279501 1.190800 0.060793
1 -1.863544 2.104444 0.123981
7 -1.963430 0.040202 0.041988
7 0.077774 -1.210634 -0.119398
8 -1.963378 -2.244403 -0.095209
7 2.126463 -0.151945 -0.103517
1 2.552404 0.771752 -0.062949
6 0.707205 2.534163 0.017918
1 -0.004025 3.378068 0.081772
8 1.928765 2.778624 -0.035090
6 -3.429627 0.012663 0.103270
1 -3.832553 -0.446672 -0.801355
1 -3.754705 -0.564132 0.971082
1 -3.797052 1.035477 0.188137
8 3.489113 -2.462989 0.190882
1 4.406506 -2.262737 -0.041333
1 2.983577 -1.593136 0.058254
d_1m5fC/d_1m5fC_2_042.xyz
6 -1.248725 -1.189969 -0.069354
6 0.832025 -0.050889 -0.084126
6 0.120277 1.235586 0.003945
6 -1.258743 1.205044 0.067254
1 -1.836624 2.122007 0.137885
7 -1.950429 0.059151 0.042389
7 0.081988 -1.204316 -0.129242
8 -1.965858 -2.224805 -0.102466
7 2.137551 -0.158275 -0.121151
1 2.569280 0.762544 -0.076163
6 0.736667 2.535520 0.027231
1 0.031231 3.383535 0.100119
8 1.959517 2.772206 -0.029646
6 -3.416584 0.041403 0.106117

1 -3.824443 -0.408552 -0.801058
1 -3.744251 -0.539193 0.970388
1 -3.776821 1.066121 0.198665
8 3.526861 -2.454062 0.127186
1 2.967935 -3.062346 0.630354
1 2.976266 -1.606916 0.028853
d_1m5fC/d_1m5fC_3_086.xyz
6 1.822569 -0.938233 -0.016687
6 -0.531259 -0.906377 -0.306495
6 -0.521703 0.557260 -0.198670
6 0.684951 1.174292 0.047119
1 0.751709 2.252535 0.144219
7 1.830092 0.484427 0.165654
7 0.670864 -1.573872 -0.244235
8 2.939416 -1.514377 0.050107
7 -1.663377 -1.555805 -0.450667
1 -1.464230 -2.555436 -0.507374
6 -1.705026 1.365702 -0.391133
1 -2.597960 0.828658 -0.743603
8 -1.772983 2.596864 -0.209860
6 3.113155 1.161399 0.388372
1 3.585778 0.779612 1.295138
1 3.779680 0.988055 -0.459002
1 2.927963 2.230040 0.497140
8 -4.014234 -0.995330 0.804605
1 -4.088497 -0.033575 0.874888
1 -3.151201 -1.154646 0.309485
d_1m5fC/d_1m5fC_3_061.xyz
6 1.805672 -0.985827 -0.015920
6 -0.553166 -0.866569 -0.242285
6 -0.484917 0.596449 -0.147040
6 0.752635 1.169369 0.047511
1 0.863412 2.245205 0.129244
7 1.874547 0.438163 0.135778
7 0.623525 -1.579745 -0.195290
8 2.900690 -1.604379 0.029704

7 -1.709518 -1.475632 -0.366711
1 -1.546637 -2.482034 -0.419043
6 -1.644467 1.448622 -0.288883
1 -2.584770 0.941646 -0.548955
8 -1.645634 2.687801 -0.153374
6 3.188167 1.067430 0.314927
1 3.668339 0.681281 1.215925
1 3.825794 0.856303 -0.545948
1 3.047524 2.144009 0.410552
8 -4.170650 -0.823179 0.592424
1 -4.593928 -1.677235 0.756579
1 -3.258401 -1.047463 0.225320
d_1m5fC/d_1m5fC_3_002.xyz
6 -1.799421 -0.989796 -0.016378
6 0.558014 -0.858234 -0.251762
6 0.483211 0.603793 -0.147533
6 -0.756809 1.170121 0.050618
1 -0.872374 2.245117 0.136615
7 -1.875215 0.433450 0.137451
7 -0.614817 -1.577355 -0.201151
8 -2.891006 -1.614121 0.032157
7 1.716106 -1.461650 -0.389267
1 1.555938 -2.468134 -0.448789
6 1.638623 1.462276 -0.285556
1 2.582127 0.959654 -0.542206
8 1.632908 2.701238 -0.148173
6 -3.191704 1.055683 0.320277
1 -3.830486 0.840892 -0.538823
1 -3.667553 0.667300 1.222624
1 -3.056689 2.133057 0.415085
8 4.201449 -0.876104 0.539049
1 3.270450 -1.060687 0.197680
1 4.297773 -1.429478 1.326318
d_1m5fC/d_1m5fC_3_071.xyz
6 -1.796241 0.981490 -0.015320
6 0.557659 0.861070 -0.286133

6 0.492885 -0.600308 -0.167349
6 -0.740994 -1.172404 0.050990
1 -0.849230 -2.247358 0.146743
7 -1.862284 -0.441311 0.147320
7 -0.617979 1.574359 -0.223079
8 -2.890204 1.600240 0.047410
7 1.710884 1.468224 -0.448056
1 1.545433 2.473456 -0.513669
6 1.652063 -1.452265 -0.314248
1 2.588278 -0.944810 -0.587534
8 1.656586 -2.690056 -0.166494
6 -3.173259 -1.069275 0.349622
1 -3.639485 -0.680722 1.256914
1 -3.823914 -0.859494 -0.501768
1 -3.031765 -2.145749 0.445479
8 4.150536 0.919056 0.614532
1 4.068702 1.192649 1.538597
1 3.239614 1.079354 0.212982
d_1m5fC/d_1m5fC_3_023.xyz
6 1.801753 -0.983636 -0.016611
6 -0.554753 -0.863836 -0.262774
6 -0.488401 0.598305 -0.155309
6 0.747266 1.170767 0.051895
1 0.856880 2.246120 0.141316
7 1.868888 0.439483 0.143553
7 0.621494 -1.577016 -0.210339
8 2.896536 -1.602035 0.035147
7 -1.710034 -1.471651 -0.403806
1 -1.546750 -2.477513 -0.464690
6 -1.647549 1.450336 -0.301999
1 -2.584115 0.943923 -0.576247
8 -1.651740 2.688262 -0.156121
6 3.181425 1.068055 0.333088
1 3.656722 0.678357 1.235142
1 3.823768 0.860137 -0.525066
1 3.040243 2.144257 0.432218

8 -4.159870 -0.849454 0.595000
1 -3.247014 -1.055635 0.218357
1 -4.380024 -1.609492 1.151181
d_1m5fC/d_1m5fC_3_052.xyz
6 1.821539 0.947506 -0.009212
6 -0.534345 0.902010 -0.285534
6 -0.515056 -0.562110 -0.182945
6 0.698928 -1.173136 0.041427
1 0.773769 -2.251705 0.128219
7 1.841283 -0.477493 0.151531
7 0.663620 1.577121 -0.221097
8 2.934206 1.531471 0.059497
7 -1.669352 1.546374 -0.428833
1 -1.474596 2.547091 -0.480888
6 -1.696365 -1.377729 -0.357244
1 -2.603418 -0.844536 -0.677213
8 -1.749293 -2.611841 -0.190568
6 3.130942 -1.148787 0.351025
1 3.788568 -0.954819 -0.498800
1 3.608229 -0.780241 1.260864
1 2.954356 -2.220570 0.440987
8 -4.072047 0.986639 0.719020
1 -4.049318 0.060228 0.996126
1 -3.183236 1.145865 0.270810
d_1m5fC/d_1m5fC_3_080.xyz
6 -1.802854 -0.985755 -0.016193
6 0.554622 -0.863950 -0.253228
6 0.486036 0.598517 -0.151053
6 -0.751105 1.170046 0.050013
1 -0.862290 2.245518 0.135916
7 -1.872196 0.437740 0.139788
7 -0.621138 -1.578279 -0.203184
8 -2.897164 -1.605262 0.032805
7 1.710960 -1.471142 -0.387124
1 1.548874 -2.477427 -0.444177
6 1.644666 1.451657 -0.294862

1 2.583675 0.945838 -0.561917
8 1.646216 2.690202 -0.154115
6 -3.185876 1.065359 0.324371
1 -3.825910 0.854914 -0.534885
1 -3.662962 0.677231 1.226163
1 -3.046074 2.141930 0.421351
8 4.166091 -0.835049 0.594256
1 4.493619 -1.671879 0.952079
1 3.253606 -1.048739 0.221251
d_1m5fC/d_1m5fC_2_094.xyz
6 1.397590 0.357897 -0.105376
6 -0.616660 -0.931563 -0.099630
6 -1.390674 0.320103 0.010143
6 -0.697193 1.512066 0.057385
1 -1.219565 2.460412 0.142588
7 0.640981 1.571275 -0.001902
7 0.762005 -0.812383 -0.160858
8 2.649535 0.476631 -0.141195
7 -1.133493 -2.128254 -0.141729
1 -2.147961 -2.048691 -0.087883
6 -2.824798 0.413356 0.076149
1 -3.221365 1.442125 0.158702
8 -3.628696 -0.539819 0.048702
6 1.361735 2.847755 0.082085
1 1.969530 2.875559 0.989056
1 2.011629 2.967180 -0.786180
1 0.633736 3.658621 0.106620
8 2.430977 -2.956980 0.226284
1 3.291765 -2.533176 0.352086
1 1.801250 -2.192849 0.061567
d_1m5fC/d_1m5fC_2_036.xyz
6 -1.434809 0.330586 -0.100153
6 0.621502 -0.889612 -0.099902
6 1.353089 0.387653 0.007604
6 0.620047 1.555902 0.060324
1 1.111360 2.520743 0.146553

7 -0.719648 1.569186 0.006500
7 -0.760451 -0.817626 -0.156562
8 -2.689484 0.407575 -0.138336
7 1.178647 -2.068338 -0.143594
1 2.190058 -1.954604 -0.094947
6 2.783646 0.527138 0.067596
1 3.147918 1.567823 0.148222
8 3.617294 -0.400062 0.036006
6 -1.485727 2.819037 0.092162
1 -2.138967 2.916667 -0.776230
1 -2.093949 2.823817 0.999192
1 -0.788139 3.656326 0.118150
8 -2.268289 -3.087173 0.210220
1 -1.637825 -3.787612 0.428611
1 -1.702947 -2.272658 0.057138
d_1m5fC/d_1m5fC_3_092.xyz
6 -1.814073 -0.981695 -0.017401
6 0.548226 -0.872651 -0.218322
6 0.483831 0.591481 -0.134351
6 -0.753787 1.169751 0.044997
1 -0.861452 2.246489 0.118683
7 -1.879067 0.443431 0.126559
7 -0.632302 -1.580569 -0.180404
8 -2.911847 -1.595940 0.019791
7 1.702387 -1.489088 -0.323706
1 1.532830 -2.494689 -0.372363
6 1.646054 1.440177 -0.271016
1 2.586715 0.928845 -0.520812
8 1.650608 2.679948 -0.141608
6 -3.191512 1.078690 0.292513
1 -3.823429 0.866483 -0.572287
1 -3.679742 0.698126 1.191535
1 -3.047191 2.155072 0.384530
8 4.218226 -1.000800 0.586502
1 3.282175 -1.110367 0.229161
1 4.504630 -0.126558 0.288403

d_1m5fC/d_1m5fC_2_034.xyz
6 0.979544 0.622424 0.218104
6 -1.348213 1.050703 0.011179
6 -1.593574 -0.404601 -0.026966
6 -0.504481 -1.248954 0.041917
1 -0.627186 -2.327738 0.008146
7 0.750954 -0.792647 0.155185
7 -0.035700 1.470965 0.144035
8 2.185641 0.986070 0.349457
7 -2.282935 1.958611 -0.075929
1 -3.185248 1.493804 -0.167377
6 -2.880736 -1.036980 -0.135844
1 -2.855636 -2.142140 -0.152239
8 -3.984521 -0.460614 -0.207830
6 1.897482 -1.708856 0.207069
1 2.523914 -1.574293 -0.676651
1 2.491481 -1.514284 1.100940
1 1.525626 -2.733151 0.233023
8 4.664274 0.140642 -0.510773
1 5.120748 0.984627 -0.632671
1 3.756789 0.383467 -0.215793
d_1m5fC/d_1m5fC_3_039.xyz
6 1.140128 0.351072 -0.003594
6 -0.534083 -1.332536 -0.013993
6 -1.565881 -0.283942 0.002533
6 -1.155568 1.029523 0.000370
1 -1.875854 1.840651 0.006991
7 0.142888 1.375081 -0.010315
7 0.791505 -0.930225 -0.005395
8 2.340553 0.755073 0.003660
7 -0.868268 -2.595491 -0.036110
1 -0.008200 -3.147722 -0.043987
6 -2.979281 -0.584521 0.018469
1 -3.235817 -1.654501 0.022322
8 -3.896867 0.259702 0.029117
6 0.562449 2.780741 -0.019526

1 1.136776 3.010703 0.880195
1 1.181093 2.982200 -0.895853
1 -0.327892 3.408548 -0.052225
8 4.347571 -1.100738 0.031020
1 3.578692 -0.476803 0.020177
1 3.951680 -1.982945 0.009196
d_1m5fC/d_1m5fC_3_041.xyz
6 -1.806735 -1.002272 -0.020688
6 0.558370 -0.856288 -0.162067
6 0.467098 0.607541 -0.103048
6 -0.784210 1.166845 0.036413
1 -0.910839 2.242426 0.095073
7 -1.899297 0.423120 0.098357
7 -0.610905 -1.583596 -0.140455
8 -2.894643 -1.634806 -0.002043
7 1.723650 -1.456708 -0.231172
1 1.571049 -2.465419 -0.266812
6 1.619923 1.474135 -0.203963
1 2.583548 0.974434 -0.376738
8 1.593097 2.717481 -0.114996
6 -3.224426 1.037429 0.240823
1 -3.843161 0.804459 -0.628100
1 -3.716205 0.659312 1.139016
1 -3.099905 2.117220 0.320832
8 4.321434 -0.885699 0.309608
1 3.344018 -1.048958 0.119589
1 4.438125 -1.103324 1.244653
d_1m5fC/d_1m5fC_3_037.xyz
6 -1.141556 -0.827019 -0.085358
6 0.094508 1.221043 -0.107494
6 1.340129 0.447127 -0.004173
6 1.251358 -0.925596 0.039333
1 2.144899 -1.537059 0.108884
7 0.075167 -1.575139 -0.005297
7 -1.092088 0.506399 -0.145285
8 -2.213550 -1.484292 -0.097250

7 0.114939 2.525140 -0.164071
1 -0.848337 2.859358 -0.233902
6 2.638383 1.079730 0.048868
1 2.628635 2.179798 0.016537
8 3.730632 0.483712 0.126430
6 -0.002576 -3.040308 0.036975
1 -0.559753 -3.360967 0.919598
1 -0.506444 -3.411861 -0.857275
1 1.009483 -3.442420 0.079103
8 -3.501391 1.797038 0.230333
1 -4.128772 1.093605 0.448819
1 -2.632844 1.323212 0.075793
d_1m5fC/d_1m5fC_2_000.xyz
6 2.321647 0.440369 0.055964
6 0.114990 1.323295 -0.022193
6 -0.417103 -0.055027 -0.068280
6 0.486679 -1.102137 -0.070812
1 0.151087 -2.134421 -0.110602
7 1.807809 -0.904396 -0.022084
7 1.487666 1.473695 0.048130
8 3.576452 0.543424 0.130725
7 -0.625913 2.399079 -0.038648
1 -1.606241 2.126299 -0.085750
6 -1.801666 -0.416381 -0.097610
1 -2.009737 -1.500249 -0.120819
8 -2.770534 0.377318 -0.092833
6 2.754975 -2.024844 -0.021108
1 3.437714 -1.935775 -0.867938
1 3.333651 -2.023974 0.904711
1 2.194448 -2.956439 -0.099971
8 -5.245385 -0.822817 0.127622
1 -5.074356 -1.722337 0.440006
1 -4.354893 -0.406803 0.048697
d_1m5fC/d_1m5fC_2_022.xyz
6 1.188599 -0.207471 -0.170112
6 -0.812044 1.068823 -0.107785

6 -1.582930 -0.183293 0.030583
6 -0.886557 -1.373970 0.068333
1 -1.402284 -2.323502 0.177729
7 0.448988 -1.426699 -0.028714
7 0.564990 0.960697 -0.208325
8 2.446016 -0.342910 -0.255363
7 -1.342447 2.261220 -0.140252
1 -2.354259 2.172760 -0.057680
6 -3.014322 -0.281638 0.135362
1 -3.404224 -1.311435 0.236035
8 -3.824101 0.667265 0.121872
6 1.178228 -2.698742 0.052712
1 1.807524 -2.826396 -0.829158
1 1.807690 -2.712655 0.945120
1 0.454912 -3.512621 0.105389
8 4.116343 1.728922 0.322641
1 3.451154 1.031446 0.096056
1 3.606858 2.547411 0.399787
d_1m5fU/d_1m5fU190.xyz
6 -0.115579 -1.211074 -0.026344
7 1.076788 -0.545338 -0.036546
6 1.157605 0.798514 -0.021438
7 -0.041816 1.570163 -0.000982
6 -1.234256 0.954605 0.009019
8 2.242955 1.421035 -0.025152
8 -0.133742 -2.472780 -0.038775
1 -2.113988 1.589545 0.025999
6 0.073346 3.034562 0.011859
1 0.621820 3.354905 0.899442
1 0.606574 3.371454 -0.878836
1 -0.928647 3.462112 0.024446
6 -2.649616 -1.049155 0.010342
8 -3.737426 -0.447487 0.031602
1 -2.638616 -2.150435 -0.000183
6 -1.347032 -0.418700 -0.002087
8 3.516234 -1.864143 0.056569

1 2.632944 -1.400572 0.018164
1 4.164129 -1.146291 0.091619
d_1m5fU/d_1m5fU219.xyz
6 0.599127 -0.867717 -0.023668
7 -0.766547 -0.809155 -0.038476
6 -1.451726 0.348452 -0.022245
7 -0.733460 1.579454 0.005738
6 0.607747 1.569719 0.016012
8 -2.700925 0.410535 -0.031176
8 1.172986 -1.988902 -0.032645
1 1.097774 2.538508 0.035851
6 -1.501313 2.832319 0.020685
1 -2.124788 2.893851 -0.873018
1 -2.140967 2.862919 0.904464
1 -0.805900 3.670945 0.041661
6 2.781025 0.538628 0.014829
8 3.621688 -0.373658 0.005778
1 3.132856 1.587710 0.034659
6 1.341058 0.399524 0.002033
8 -2.084740 -3.249072 0.051676
1 -1.366033 -3.896603 0.076677
1 -1.620482 -2.366192 0.013941
d_1m5fU/d_1m5fU270.xyz
6 -0.573404 0.957372 0.000006
7 0.782229 0.781119 0.000015
6 1.363554 -0.433078 0.000009
7 0.542516 -1.596978 -0.000005
6 -0.792759 -1.471526 -0.000006
8 2.603220 -0.601993 0.000017
8 -1.053426 2.121622 0.000006
1 -1.362479 -2.395991 -0.000011
6 1.195846 -2.912958 -0.000010
1 1.820200 -3.016095 -0.889226
1 1.820179 -3.016114 0.889218
1 0.426231 -3.684455 -0.000028
6 -2.867541 -0.257422 -0.000001

8 -3.625729 0.723824 -0.000001
1 -3.308463 -1.272545 0.000001
6 -1.421126 -0.242426 -0.000000
8 2.635100 2.850501 -0.000030
1 1.949837 2.125144 -0.000011
1 3.480548 2.379668 0.000076
d_1m5fU/d_1m5fU1100.xyz
6 0.929407 0.237918 0.000008
7 0.664126 -1.093990 0.000007
6 -0.597770 -1.561939 0.000003
7 -1.694202 -0.645892 0.000001
6 -1.463354 0.674674 -0.000001
8 -0.881189 -2.782013 0.000002
8 2.131829 0.648717 0.000014
1 -2.330893 1.326415 -0.000005
6 -3.058290 -1.190302 -0.000007
1 -3.212048 -1.804532 -0.889032
1 -3.212056 -1.804536 0.889014
1 -3.763676 -0.359873 -0.000008
6 0.036245 2.616847 0.000000
8 -0.859984 3.478894 -0.000007
1 1.091629 2.931231 0.000006
6 -0.183326 1.186456 0.000002
8 4.266306 -1.027426 -0.000016
1 3.971270 -1.948521 -0.000003
1 3.433141 -0.488315 -0.000002
d_1m5fU/d_1m5fU167.xyz
6 0.029247 -1.166906 -0.054545
7 1.150415 -0.386311 -0.076179
6 1.098395 0.958623 -0.043989
7 -0.172274 1.606625 0.005689
6 -1.297166 0.876093 0.022650
8 2.113579 1.688636 -0.056491
8 0.137134 -2.424598 -0.077376
1 -2.234932 1.420263 0.059481
6 -0.202720 3.075187 0.037777

1 0.328530 3.436925 0.919898
1 0.278091 3.474970 -0.856798
1 -1.241258 3.402589 0.074407
6 -2.508103 -1.257019 0.012956
8 -3.648995 -0.764578 0.052767
1 -2.389274 -2.351709 -0.011298
6 -1.274127 -0.501594 -0.005955
8 3.564448 -1.738769 0.120748
1 2.700700 -1.245231 0.038173
1 3.308666 -2.671831 0.139021
d_1m5fU/d_1m5fU269.xyz
6 -0.779335 -1.075899 -0.000001
7 0.581115 -0.945581 -0.000009
6 1.179560 0.252351 -0.000011
7 0.399577 1.442593 0.000004
6 -0.938616 1.356853 0.000006
8 2.429373 0.406189 -0.000025
8 -1.299354 -2.224895 0.000002
1 -1.482508 2.296575 0.000013
6 1.090791 2.739467 0.000004
1 1.717901 2.824615 0.889087
1 1.717855 2.824643 -0.889109
1 0.343822 3.532849 0.000037
6 -3.043454 0.199288 0.000004
8 -3.829873 -0.759827 -0.000001
1 -3.455878 1.226415 0.000009
6 -1.597843 0.144481 0.000003
8 4.161098 -1.713237 0.000029
1 3.471573 -1.005193 0.000022
1 3.665820 -2.544070 -0.000091
d_1m5fU/d_1m5fU186.xyz
6 -1.166290 -1.311608 0.030312
7 0.155195 -1.549457 0.280577
6 1.034198 -0.549862 0.430555
7 0.603049 0.805598 0.336031
6 -0.688246 1.076894 0.092375

8 2.257892 -0.738822 0.662022
8 -1.967633 -2.279363 -0.108471
1 -0.966313 2.123620 0.030537
6 1.593593 1.876833 0.514163
1 2.086125 1.766017 1.481357
1 2.343354 1.827482 -0.277037
1 1.079412 2.836535 0.471709
6 -3.007360 0.405125 -0.330979
8 -3.465766 1.557077 -0.425240
1 -3.681413 -0.457850 -0.448119
6 -1.621474 0.077120 -0.071360
8 4.266978 0.044015 -1.096643
1 4.690463 -0.792164 -1.336218
1 3.542369 -0.206891 -0.482221
d_1m5fU/d_1m5fU245.xyz
6 0.846932 -0.049292 -0.075488
7 0.108307 -1.190210 -0.084492
6 -1.235947 -1.171920 -0.036869
7 -1.923385 0.077604 0.017925
6 -1.225245 1.221208 0.033057
8 -1.941886 -2.206642 -0.039045
8 2.111932 -0.117652 -0.127511
1 -1.800917 2.140711 0.079670
6 -3.391670 0.066934 0.063199
1 -3.785742 -0.427815 -0.826207
1 -3.730423 -0.471176 0.950260
1 -3.748930 1.095735 0.099483
6 0.778313 2.547939 0.029409
8 1.992367 2.800786 0.017785
1 0.059007 3.387892 0.074768
6 0.155024 1.242567 -0.007013
8 3.402987 -2.502903 0.113133
1 2.865647 -1.674505 0.020016
1 2.759258 -3.195933 0.315311
d_1m5fU/d_1m5fU120.xyz
6 -0.276623 1.478608 -0.007489

7 -1.639752 1.464478 0.003914
6 -2.337142 0.316399 0.009080
7 -1.645470 -0.938615 0.003121
6 -0.307371 -0.962337 -0.007517
8 -3.588763 0.265270 0.019266
8 0.346639 2.579639 -0.012870
1 0.169096 -1.937049 -0.011862
6 -2.442033 -2.172994 0.008631
1 -3.070027 -2.203317 0.900722
1 -3.079786 -2.205151 -0.876506
1 -1.766238 -3.027854 0.005905
6 1.877869 0.137194 -0.023720
8 2.549677 -0.915038 -0.026948
1 2.397946 1.106461 -0.028895
6 0.439436 0.198559 -0.012859
8 5.293550 -0.530419 -0.043297
1 5.495719 0.067345 0.689938
1 4.316203 -0.649666 -0.014507
d_1m5fU/d_1m5fU133.xyz
6 -1.144618 -1.312879 -0.045619
7 0.175803 -1.538136 -0.312753
6 1.045369 -0.530551 -0.463275
7 0.604780 0.820587 -0.352739
6 -0.685632 1.079730 -0.092151
8 2.268571 -0.707421 -0.707671
8 -1.936501 -2.288260 0.094115
1 -0.971178 2.123678 -0.018073
6 1.584883 1.900667 -0.534803
1 2.352393 1.841610 0.238432
1 2.056273 1.809349 -1.514602
1 1.066351 2.856348 -0.464240
6 -2.994557 0.386373 0.351349
8 -3.460385 1.533889 0.461715
1 -3.660951 -0.482680 0.467410
6 -1.609230 0.071288 0.073387
8 4.202656 0.006387 1.165698

1 4.413251 -0.833453 1.597440
1 3.507763 -0.216544 0.507892
d_1m5fU/d_1m5fU112.xyz
6 -0.556177 -0.850306 -0.423756
7 0.602430 -1.552627 -0.329043
6 1.772150 -0.950051 -0.044568
7 1.807403 0.461328 0.175337
6 0.683345 1.183298 0.068650
8 2.860477 -1.562918 0.047222
8 -1.644424 -1.461509 -0.669298
1 0.769703 2.252729 0.229037
6 3.097020 1.087100 0.496765
1 3.498128 0.656439 1.415937
1 3.805096 0.915449 -0.315839
1 2.941010 2.157391 0.629584
6 -1.713745 1.416797 -0.387461
8 -1.761548 2.645721 -0.204624
1 -2.623443 0.880837 -0.697948
6 -0.527930 0.599425 -0.236147
8 -3.804393 -1.011080 0.976315
1 -3.031675 -1.154441 0.380154
1 -3.956525 -1.868590 1.397201
d_1m5fU/d_1m5fU181.xyz
6 0.253253 1.469383 -0.019949
7 1.615897 1.474525 0.020666
6 2.329202 0.336290 0.031666
7 1.655893 -0.928204 -0.000430
6 0.318588 -0.971041 -0.034266
8 3.580904 0.302681 0.069665
8 -0.385464 2.561409 -0.036174
1 -0.143552 -1.952551 -0.052729
6 2.469940 -2.150941 0.012614
1 3.124613 -2.168647 -0.860499
1 3.081368 -2.177927 0.916226
1 1.806523 -3.015138 -0.007889
6 -1.881527 0.098761 -0.067940

8 -2.540156 -0.961690 -0.088544
1 -2.414120 1.061318 -0.064596
6 -0.444352 0.179278 -0.043637
8 -5.274519 -0.519739 0.060041
1 -4.303375 -0.675765 0.010469
1 -5.370732 0.272799 0.606536
d_1m5fU/d_1m5fU164.xyz
6 -0.258411 1.471314 -0.006319
7 -1.621607 1.472603 0.004854
6 -2.331843 0.332415 0.008563
7 -1.654432 -0.930252 0.000719
6 -0.316630 -0.969251 -0.008640
8 -3.583964 0.295433 0.018858
8 0.377357 2.565212 -0.011468
1 0.148651 -1.949422 -0.013163
6 -2.465131 -2.155321 0.005041
1 -3.092642 -2.179906 0.897662
1 -3.103973 -2.178845 -0.879566
1 -1.799132 -3.017768 0.000337
6 1.880747 0.105965 -0.019169
8 2.541356 -0.953339 -0.024248
1 2.411466 1.069600 -0.019278
6 0.443126 0.183208 -0.012000
8 5.283546 -0.539722 0.005801
1 5.402061 0.386364 0.259528
1 4.308328 -0.677139 -0.000942
d_1m5fU/d_1m5fU199.xyz
6 -0.555650 -0.851281 -0.425668
7 0.603857 -1.552092 -0.331145
6 1.772600 -0.948165 -0.045485
7 1.805668 0.462951 0.176366
6 0.680768 1.183576 0.069173
8 2.861784 -1.559578 0.045886
8 -1.643111 -1.463847 -0.671403
1 0.765585 2.252978 0.230566
6 3.094205 1.090096 0.499468

1 3.495453 0.658510 1.418143
1 3.802966 0.920783 -0.313037
1 2.936597 2.159946 0.633949
6 -1.716074 1.414350 -0.389879
8 -1.765843 2.642984 -0.205688
1 -2.624431 0.877538 -0.702780
6 -0.529404 0.598386 -0.237425
8 -3.801975 -1.015004 0.975515
1 -3.900019 -1.846053 1.460626
1 -3.028339 -1.157925 0.380401
d_1m5fU/d_1m5fU143.xyz
6 -1.314248 1.289233 -0.004935
7 0.010735 1.616732 0.020715
6 0.967974 0.678034 0.041112
7 0.617365 -0.703205 0.034798
6 -0.675492 -1.061983 0.011419
8 2.195018 0.954724 0.067485
8 -2.191387 2.199694 -0.025132
1 -0.887478 -2.125947 0.008245
6 1.690065 -1.706571 0.054124
1 2.313556 -1.601369 -0.835058
1 2.306175 -1.573440 0.944524
1 1.236262 -2.696957 0.067475
6 -3.072560 -0.547750 -0.030016
8 -3.464128 -1.727892 -0.033624
1 -3.811591 0.268719 -0.043385
6 -1.687362 -0.127807 -0.008270
8 4.700627 -0.235473 -0.106700
1 3.791272 0.131324 -0.047046
1 5.283806 0.535614 -0.080180
d_1m5fU/d_1m5fU229.xyz
6 0.498483 -0.766815 -0.373491
7 -0.667513 -1.462654 -0.322153
6 -1.845851 -0.864795 -0.068145
7 -1.885972 0.541343 0.172467
6 -0.758979 1.262159 0.099078

8 -2.936536 -1.478701 -0.021687
8 1.585349 -1.387250 -0.581231
1 -0.857675 2.330179 0.269476
6 -3.181785 1.164965 0.473027
1 -3.869452 1.012043 -0.360548
1 -3.607042 0.717469 1.373035
1 -3.026556 2.231929 0.631385
6 1.587408 1.594948 -0.294220
8 2.754703 1.312686 -0.602146
1 1.332350 2.651835 -0.086763
6 0.465467 0.687728 -0.182581
8 3.658631 -0.932305 1.172444
1 3.896224 -0.005029 1.026811
1 2.920921 -1.073825 0.533357
d_1m5fU/d_1m5fU147.xyz
6 -0.266622 1.474996 -0.020167
7 -1.629169 1.468470 0.023360
6 -2.332832 0.324262 0.034608
7 -1.648869 -0.934503 0.000040
6 -0.311386 -0.965790 -0.038153
8 -3.584113 0.280017 0.075095
8 0.362695 2.572470 -0.035211
1 0.159146 -1.943197 -0.059466
6 -2.452353 -2.164268 0.014842
1 -3.062296 -2.195816 0.919296
1 -3.108045 -2.188145 -0.857372
1 -1.781626 -3.022850 -0.005841
6 1.879568 0.122290 -0.077220
8 2.546372 -0.932945 -0.101747
1 2.404411 1.089008 -0.073854
6 0.441817 0.190942 -0.048262
8 5.282453 -0.516463 0.049357
1 4.308498 -0.656502 0.005581
1 5.417772 0.060513 0.814011
d_1m5fU/d_1m5fU174.xyz
6 -0.256076 1.470341 0.000001

7 -1.619318 1.473663 0.000007
6 -2.331236 0.334515 -0.000000
7 -1.655655 -0.929144 -0.000003
6 -0.317878 -0.970150 -0.000010
8 -3.583452 0.299370 -0.000004
8 0.381364 2.563281 0.000009
1 0.145951 -1.951027 -0.000014
6 -2.468188 -2.152990 0.000019
1 -3.101401 -2.176109 0.888660
1 -3.101422 -2.176131 -0.888606
1 -1.803438 -3.016404 0.000022
6 1.881091 0.101827 -0.000018
8 2.540183 -0.958432 -0.000024
1 2.413250 1.064697 -0.000010
6 0.443572 0.181192 -0.000016
8 5.282242 -0.539897 0.000021
1 4.307504 -0.680817 0.000003
1 5.393963 0.421182 0.000035
d_1m5fU/d_1m5fU189.xyz
6 -0.303790 1.487849 -0.000010
7 -1.666645 1.451251 0.000008
6 -2.344897 0.291677 0.000015
7 -1.632408 -0.951592 -0.000015
6 -0.294104 -0.953004 -0.000017
8 -3.595576 0.219434 0.000045
8 0.301323 2.598860 -0.000021
1 0.198483 -1.919558 -0.000024
6 -2.407528 -2.199536 -0.000007
1 -3.039716 -2.241808 0.888668
1 -3.039823 -2.241748 -0.888607
1 -1.716752 -3.042390 -0.000078
6 1.873051 0.182081 -0.000018
8 2.561606 -0.859177 -0.000018
1 2.377032 1.159766 -0.000019
6 0.433466 0.219923 -0.000014
8 5.298998 -0.400398 0.000049

1 5.715096 -1.273789 -0.000041
1 4.331052 -0.581785 0.000020
d_1m5fU/d_1m5fU159.xyz
6 1.161113 -1.311579 -0.033821
7 -0.160233 -1.545615 -0.288305
6 -1.036226 -0.543500 -0.439685
7 -0.601435 0.810661 -0.341903
6 0.689720 1.078324 -0.094181
8 -2.259879 -0.728322 -0.674504
8 1.959481 -2.281711 0.105938
1 0.970386 2.124270 -0.030410
6 -1.588262 1.884063 -0.526087
1 -2.053946 1.792440 -1.508784
1 -2.359751 1.815897 0.242243
1 -1.077150 2.843276 -0.449637
6 3.005558 0.399814 0.335888
8 3.466588 1.550466 0.433728
1 3.677129 -0.465025 0.453515
6 1.619752 0.075783 0.071273
8 -4.210830 -0.050598 1.204679
1 -3.524881 -0.244475 0.529031
1 -5.052922 -0.137810 0.736452
d_1m5hmC/d_1m5hmC_3_004.xyz
6 2.128989 -0.772012 -0.027131
6 -0.200623 -0.891309 0.074460
6 -0.338945 0.536635 0.172072
6 0.827163 1.238773 0.124813
1 0.847238 2.322155 0.171872
7 2.044278 0.622955 0.019331
7 0.986625 -1.503999 -0.008503
8 3.267645 -1.304518 -0.094689
6 -1.695869 1.203219 0.314673
1 -2.121423 0.884867 1.288331
1 -1.516726 2.290049 0.408530
7 -1.311994 -1.662993 0.098005
1 -1.215350 -2.648825 -0.107272

1 -2.191891 -1.213280 -0.129059
8 -2.577047 0.897794 -0.740341
6 3.284197 1.403717 -0.051899
1 3.928782 1.168858 0.798102
1 3.819780 1.172577 -0.975169
1 3.032587 2.463806 -0.035040
8 -4.723130 -0.115892 0.114164
1 -3.826605 0.301400 -0.234741
1 -4.557975 -1.066554 0.177608
d_1m5hmC/d_1m5hmC_3_000.xyz
6 2.021168 0.813149 0.073846
6 -0.307136 0.862486 -0.176334
6 -0.388833 -0.571878 -0.279429
6 0.799061 -1.232373 -0.178544
1 0.856024 -2.313966 -0.236054
7 1.989238 -0.583240 -0.007666
7 0.860800 1.505656 -0.015472
8 3.133720 1.380960 0.233701
6 -1.696268 -1.320486 -0.459059
1 -2.217423 -0.894808 -1.338708
1 -1.440462 -2.357726 -0.734369
7 -1.417510 1.622191 -0.268029
1 -1.320134 2.617620 -0.117403
1 -2.354421 1.224253 -0.192424
8 -2.529257 -1.283920 0.681013
6 3.246527 -1.327638 0.124644
1 3.954946 -1.008962 -0.642622
1 3.685994 -1.150094 1.109047
1 3.037321 -2.390548 0.006603
8 -4.127223 0.542009 0.283782
1 -3.458814 -0.304288 0.471907
1 -4.635767 0.314320 -0.506532
d_1m5hmC/d_1m5hmC_3_027.xyz
6 -1.990759 0.539516 -0.303048
6 0.208248 1.220270 0.074323
6 0.569211 -0.039988 0.665096

6 -0.413147 -0.982032 0.677115
1 -0.249591 -1.979614 1.070078
7 -1.668991 -0.726071 0.196388
7 -1.024260 1.490553 -0.372635
8 -3.171661 0.752251 -0.684089
6 1.960273 -0.278501 1.231639
1 2.053761 0.333631 2.149419
1 2.000707 -1.331455 1.567769
7 1.154891 2.182816 -0.011533
1 0.951152 3.005743 -0.563778
1 2.117857 1.848008 0.042018
8 2.993959 0.034273 0.326633
6 -2.709670 -1.759229 0.204773
1 -3.528940 -1.466617 0.865823
1 -3.101311 -1.899930 -0.804711
1 -2.273491 -2.693697 0.557117
8 2.775505 -1.591717 -1.611587
1 2.874261 -0.909192 -0.825276
1 3.396769 -1.296635 -2.291046
d_1m5hmC/d_1m5hmC_3_076.xyz
6 -2.296881 0.544189 -0.018345
6 -0.032657 1.100932 0.085958
6 0.374013 -0.277340 0.137102
6 -0.636015 -1.187569 0.072149
1 -0.449399 -2.255758 0.089918
7 -1.948748 -0.810401 -0.009852
7 -1.314805 1.480894 0.019525
8 -3.516693 0.850281 -0.072437
6 1.831239 -0.669783 0.292203
1 2.136731 -0.401619 1.324992
1 1.881764 -1.773516 0.244979
7 0.923636 2.056274 0.134368
1 0.654664 3.013314 -0.053830
1 1.863357 1.748607 -0.118203
8 2.674213 -0.055618 -0.654246
6 -3.020383 -1.810608 -0.061543

1 -3.683174 -1.693883 0.798708
1 -3.603915 -1.691262 -0.976885
1 -2.572880 -2.804036 -0.043232
8 5.045620 -0.630738 -0.017681
1 4.056510 -0.380648 -0.268763
1 5.154749 -0.358884 0.903796
d_1m5hmC/d_1m5hmC_3_072.xyz
6 -1.984946 0.576720 -0.289580
6 0.232613 1.169816 0.132245
6 0.552840 -0.130577 0.655819
6 -0.458921 -1.039862 0.617955
1 -0.328640 -2.059573 0.963752
7 -1.705750 -0.721068 0.150648
7 -0.988809 1.498372 -0.306568
8 -3.155572 0.845381 -0.667966
6 1.925697 -0.440346 1.234930
1 1.990256 0.064250 2.218285
1 1.951110 -1.524645 1.450578
7 1.206815 2.106452 0.104889
1 1.033573 2.963526 -0.403925
1 2.158741 1.735785 0.163019
8 2.993702 -0.035386 0.409962
6 -2.781322 -1.717651 0.123482
1 -3.593748 -1.414933 0.788351
1 -3.172118 -1.818359 -0.891205
1 -2.380877 -2.676089 0.453326
8 2.882072 -1.199319 -1.826039
1 3.158714 -2.113708 -1.677818
1 2.919805 -0.736559 -0.883911
d_1m5hmC/d_1m5hmC_3_018.xyz
6 -1.860529 -0.714038 0.098162
6 0.401400 -0.989186 -0.405701
6 0.597064 0.420230 -0.612644
6 -0.498254 1.199465 -0.400264
1 -0.464843 2.278461 -0.505290
7 -1.709556 0.668919 -0.047063

7 -0.781835 -1.519960 -0.072729
8 -2.996920 -1.166127 0.396992
6 1.942929 0.981142 -1.047291
1 2.092500 0.696380 -2.107366
1 1.852611 2.083370 -1.042995
7 1.456592 -1.817097 -0.580393
1 1.361146 -2.780896 -0.287719
1 2.373215 -1.369372 -0.523622
8 3.020691 0.526671 -0.261795
6 -2.883097 1.525948 0.153613
1 -3.667959 1.260034 -0.557896
1 -3.268822 1.404719 1.168129
1 -2.589485 2.563968 -0.000602
8 2.376705 0.350254 2.196376
1 1.530359 -0.118371 2.202349
1 2.623990 0.430916 1.188463
d_1m5hmC/d_1m5hmC_3_021.xyz
6 -1.968277 0.575225 -0.288526
6 0.247529 1.185610 0.114320
6 0.571051 -0.098259 0.674815
6 -0.436978 -1.012572 0.659625
1 -0.302437 -2.023308 1.029186
7 -1.683771 -0.710625 0.181922
7 -0.975481 1.500158 -0.329559
8 -3.140467 0.830217 -0.670995
6 1.951382 -0.389819 1.243665
1 2.047807 0.179839 2.188013
1 1.966907 -1.457252 1.532161
7 1.220480 2.123184 0.055512
1 1.041933 2.963531 -0.478870
1 2.173479 1.758280 0.108294
8 3.000663 -0.058173 0.363706
6 -2.752241 -1.714996 0.165008
1 -3.567346 -1.412186 0.826558
1 -3.141460 -1.827344 -0.849026
1 -2.344753 -2.667514 0.503229

8 2.661343 -1.357550 -1.778753
1 2.814572 -0.821621 -0.889604
1 3.535590 -1.668533 -2.050173
d_1m5hmC/d_1m5hmC_3_070.xyz
6 1.975073 0.567203 0.290552
6 -0.237704 1.190190 -0.109413
6 -0.568496 -0.090852 -0.672195
6 0.434757 -1.010473 -0.659648
1 0.294779 -2.019725 -1.031227
7 1.683243 -0.716261 -0.181696
7 0.987271 1.497404 0.334403
8 3.149137 0.815074 0.671909
6 -1.950272 -0.374264 -1.241436
1 -2.043779 0.197723 -2.184645
1 -1.971180 -1.440920 -1.532302
7 -1.205489 2.132988 -0.048240
1 -1.022218 2.971435 0.487541
1 -2.160527 1.773876 -0.101189
8 -2.998619 -0.039235 -0.361555
6 2.746530 -1.726093 -0.167518
1 3.562176 -1.426709 -0.829977
1 3.136649 -1.841423 0.845826
1 2.333513 -2.676133 -0.506021
8 -2.717695 -1.332521 1.789541
1 -3.407406 -2.010040 1.791584
1 -2.839101 -0.807827 0.887933
d_1m5hmC/d_1m5hmC_3_025.xyz
6 2.132698 0.768325 0.018891
6 -0.197548 0.892622 -0.054359
6 -0.339974 -0.535126 -0.147975
6 0.824693 -1.239998 -0.113679
1 0.841852 -2.323487 -0.159274
7 2.044367 -0.626492 -0.024003
7 0.991719 1.503013 0.013281
8 3.273376 1.298417 0.071154
6 -1.700587 -1.196735 -0.278885

1 -2.119084 -0.900863 -1.262763
1 -1.528758 -2.286890 -0.343208
7 -1.309677 1.664041 -0.064921
1 -1.209600 2.649133 0.142790
1 -2.181852 1.208330 0.185056
8 -2.584497 -0.856127 0.762860
6 3.283619 -1.410017 0.025953
1 3.913419 -1.177860 -0.835833
1 3.836596 -1.179529 0.939024
1 3.029185 -2.469550 0.014658
8 -4.730798 0.098346 -0.160915
1 -4.512174 0.994855 -0.449814
1 -3.836491 -0.297591 0.214399
d_1m5hmC/d_1m5hmC_3_010.xyz
6 2.127142 -0.769919 -0.017984
6 -0.203852 -0.889919 0.041546
6 -0.343986 0.538088 0.134627
6 0.822289 1.240627 0.109247
1 0.841265 2.323981 0.156859
7 2.041345 0.625015 0.026699
7 0.984733 -1.502359 -0.020147
8 3.267075 -1.302236 -0.063694
6 -1.703642 1.202714 0.258993
1 -2.125364 0.910719 1.242859
1 -1.530235 2.292772 0.320141
7 -1.317130 -1.659741 0.046857
1 -1.216960 -2.645228 -0.158956
1 -2.188055 -1.203739 -0.207353
8 -2.585736 0.859700 -0.783512
6 3.282785 1.406185 -0.003848
1 3.899325 1.171521 0.866856
1 3.848718 1.176334 -0.909037
1 3.030153 2.466203 0.005391
8 -4.702576 -0.122089 0.180918
1 -3.823985 0.289132 -0.214100
1 -4.412023 -0.871770 0.718298

d_1m5hmC/d_1m5hmC_3_085.xyz
6 2.180030 0.717008 -0.015664
6 -0.142953 0.944732 -0.021826
6 -0.351307 -0.476624 -0.078537
6 0.779993 -1.233304 -0.064109
1 0.746174 -2.317232 -0.087330
7 2.028340 -0.673458 -0.026479
7 1.073238 1.503016 -0.001817
8 3.344564 1.195473 -0.008234
6 -1.745077 -1.070806 -0.171234
1 -2.142819 -0.817845 -1.176419
1 -1.634877 -2.170749 -0.163018
7 -1.226185 1.755518 -0.014848
1 -1.085173 2.739172 0.175971
1 -2.099622 1.319124 0.277960
8 -2.613336 -0.609575 0.837097
6 3.233619 -1.510423 -0.015043
1 3.843273 -1.306053 -0.897978
1 3.826362 -1.304385 0.878752
1 2.933678 -2.558145 -0.016659
8 -4.818592 -0.035220 -0.262788
1 -4.633539 -0.030147 -1.211665
1 -3.908123 -0.278200 0.192271
d_1m5hmC/d_1m5hmC_3_011.xyz
6 2.159910 0.744133 0.006620
6 -0.167442 0.917858 -0.053224
6 -0.340972 -0.507297 -0.131496
6 0.807900 -1.236979 -0.097073
1 0.800753 -2.321071 -0.131352
7 2.041211 -0.648905 -0.021943
7 1.034906 1.503567 0.001724
8 3.312446 1.249490 0.046519
6 -1.717073 -1.137939 -0.250647
1 -2.126524 -0.853681 -1.241848
1 -1.571998 -2.233125 -0.290899
7 -1.265110 1.708930 -0.063927

1 -1.147127 2.693970 0.134562
1 -2.142529 1.265641 0.194888
8 -2.594472 -0.752119 0.780834
6 3.263939 -1.458177 0.023933
1 3.893769 -1.240756 -0.841604
1 3.826510 -1.238084 0.933740
1 2.987482 -2.512225 0.015369
8 -4.739885 0.172744 -0.174099
1 -3.857043 -0.239748 0.209817
1 -5.202619 -0.556574 -0.608350
d_1m5hmC/d_1m5hmC_3_073.xyz
6 -1.893740 0.641209 -0.217440
6 0.350644 1.172096 0.137915
6 0.645782 -0.130385 0.668923
6 -0.390116 -1.013318 0.658384
1 -0.275824 -2.036379 0.999614
7 -1.640674 -0.660165 0.229331
7 -0.872933 1.534155 -0.265607
8 -3.063533 0.938251 -0.575170
6 2.036339 -0.484885 1.171918
1 2.213670 0.093847 2.098660
1 2.010509 -1.546872 1.479873
7 1.350649 2.079881 0.062860
1 1.181043 2.933846 -0.452551
1 2.294486 1.693596 0.077891
8 3.049130 -0.225242 0.226425
6 -2.737452 -1.633930 0.211024
1 -3.549038 -1.300635 0.861539
1 -3.121028 -1.746561 -0.805438
1 -2.361120 -2.593705 0.564511
8 2.240380 -1.365750 -1.897690
1 1.278434 -1.359860 -1.789557
1 2.593022 -0.897171 -1.033493
d_1m5hmC/d_1m5hmC_3_034.xyz
6 -2.009119 0.514161 -0.304796
6 0.179211 1.234965 0.061510

6 0.566304 -0.018507 0.650003
6 -0.399891 -0.977118 0.670377
1 -0.216920 -1.970795 1.064715
7 -1.663168 -0.743963 0.197728
7 -1.059921 1.481667 -0.380523
8 -3.194798 0.705356 -0.682105
6 1.964397 -0.233006 1.207691
1 2.053716 0.379754 2.125285
1 2.025833 -1.285371 1.542070
7 1.106068 2.216533 -0.026696
1 0.883735 3.035553 -0.577637
1 2.075803 1.901869 0.021302
8 2.987006 0.098617 0.296602
6 -2.686273 -1.794567 0.218280
1 -3.506988 -1.510716 0.881346
1 -3.080392 -1.949172 -0.788188
1 -2.232189 -2.718656 0.575187
8 2.865199 -1.529605 -1.635066
1 2.915025 -0.854517 -0.833966
1 3.454494 -2.258122 -1.397503
d_1m5hmC/d_1m5hmC_3_064.xyz
6 -2.150404 0.756891 -0.015736
6 0.178350 0.905448 0.059327
6 0.335440 -0.521252 0.146658
6 -0.821201 -1.238426 0.107194
1 -0.826643 -2.322322 0.146648
7 -2.047360 -0.637125 0.019682
7 -1.017308 1.503854 -0.006101
8 -3.296940 1.274856 -0.066169
6 1.703995 -1.166653 0.275706
1 2.111991 -0.880842 1.267042
1 1.546717 -2.260024 0.322397
7 1.283878 1.685319 0.072304
1 1.177081 2.670975 -0.129173
1 2.159649 1.233821 -0.177013
8 2.588402 -0.796554 -0.755479

6 -3.278071 -1.433305 -0.037674
1 -3.914198 -1.209010 0.821496
1 -3.829188 -1.207295 -0.953042
1 -3.012858 -2.490196 -0.026497
8 4.710471 0.140198 0.261125
1 3.846062 -0.260148 -0.169264
1 5.382794 0.096498 -0.432456
d_1m5hmC/d_1m5hmC_3_065.xyz
6 1.867565 -0.689841 -0.153387
6 -0.390591 -1.067511 0.299087
6 -0.620132 0.306570 0.653850
6 0.456391 1.129775 0.527834
1 0.394493 2.191525 0.740372
7 1.683296 0.666517 0.135784
7 0.807121 -1.532805 -0.075344
8 3.014469 -1.082616 -0.491732
6 -1.984111 0.786219 1.126954
1 -2.148835 0.369530 2.139575
1 -1.917184 1.882068 1.259762
7 -1.429396 -1.931990 0.369493
1 -1.309564 -2.853079 -0.031758
1 -2.353748 -1.498229 0.339235
8 -3.035863 0.412021 0.266807
6 2.832057 1.571499 0.012375
1 3.623634 1.270548 0.702169
1 3.220480 1.545312 -1.007920
1 2.508830 2.584198 0.252186
8 -2.287123 0.761660 -2.139755
1 -2.593604 0.628887 -1.151605
1 -1.350589 0.518395 -2.134391
d_1m5hmC/d_1m5hmC_3_086.xyz
6 -1.864279 0.701902 -0.135441
6 0.394794 1.032988 0.346730
6 0.608042 -0.359366 0.634897
6 -0.478408 -1.162674 0.472398
1 -0.431154 -2.233223 0.640089

7 -1.698379 -0.667332 0.098534
7 -0.794987 1.528792 -0.015155
8 -3.004965 1.121163 -0.463761
6 1.964653 -0.876933 1.090827
1 2.115805 -0.535571 2.133412
1 1.892378 -1.979231 1.141841
7 1.441803 1.880803 0.470157
1 1.336582 2.822821 0.116209
1 2.361333 1.435196 0.429964
8 3.030271 -0.445651 0.275758
6 -2.861763 -1.548946 -0.051298
1 -3.648600 -1.253072 0.646096
1 -3.249534 -1.489214 -1.070438
1 -2.555757 -2.572951 0.161528
8 2.341358 -0.582240 -2.171719
1 2.618485 -0.538010 -1.168240
1 1.409866 -0.320591 -2.176119
d_1m5hmC/d_1m5hmC_3_048.xyz
6 1.916422 -0.662633 -0.218795
6 -0.326541 -1.083360 0.271391
6 -0.569723 0.280273 0.656406
6 0.494349 1.119984 0.534794
1 0.424107 2.174553 0.778375
7 1.720790 0.682599 0.110865
7 0.869451 -1.521798 -0.140152
8 3.061089 -1.031581 -0.590934
6 -1.922568 0.725515 1.191517
1 -2.022967 0.314732 2.215212
1 -1.880674 1.823996 1.312222
7 -1.348140 -1.965941 0.352237
1 -1.224387 -2.880209 -0.062819
1 -2.280359 -1.546742 0.368760
8 -3.009375 0.314358 0.394836
6 2.856852 1.604771 -0.001587
1 3.661939 1.292946 0.667520
1 3.231122 1.614909 -1.027474

1 2.525398 2.605750 0.273922
8 -2.609090 0.724778 -2.071316
1 -2.754131 0.586061 -1.044384
1 -2.608496 1.682243 -2.205031
d_1m5hmC/d_1m5hmC_1_078.xyz
6 1.934355 -0.911006 -0.107501
6 -0.437073 -0.815845 -0.033545
6 -0.375962 0.624261 0.204585
6 0.854885 1.196580 0.272550
1 0.981709 2.259312 0.452180
7 2.008849 0.475602 0.123054
7 0.739014 -1.515322 -0.180383
8 3.029325 -1.537666 -0.237228
6 -1.606074 1.469281 0.348009
1 -2.351743 0.976922 0.974684
1 -1.344814 2.428888 0.811158
7 -1.602526 -1.429585 -0.098426
1 -1.443493 -2.425205 -0.257540
8 -2.280719 1.712465 -0.913578
1 -1.643628 2.140916 -1.504866
6 3.321206 1.119761 0.211189
1 3.900836 0.696303 1.035180
1 3.877919 0.981546 -0.718782
1 3.175421 2.185890 0.386909
8 -4.207662 -0.879909 0.308979
1 -4.432340 -1.386193 1.101683
1 -3.212809 -1.030550 0.172580
d_1m5hmC/d_1m5hmC_1_020.xyz
6 -1.934803 0.911608 -0.109428
6 0.436036 0.816916 -0.018807
6 0.373864 -0.624814 0.209635
6 -0.857266 -1.197298 0.269690
1 -0.985008 -2.260827 0.443846
7 -2.010544 -0.475365 0.118658
7 -0.739269 1.516532 -0.172283
8 -3.029020 1.538104 -0.246550

6 1.603648 -1.470622 0.351673
1 2.346531 -0.984142 0.986185
1 1.340627 -2.434068 0.805743
7 1.601719 1.431077 -0.068111
1 1.445234 2.427989 -0.221393
8 2.283269 -1.703175 -0.909445
1 1.649590 -2.130569 -1.505155
6 -3.323438 -1.119067 0.201662
1 -3.905847 -0.695136 1.023454
1 -3.876776 -0.980968 -0.730347
1 -3.178696 -2.185178 0.378370
8 4.201176 0.834894 0.329307
1 3.211480 1.011741 0.187730
1 4.557867 1.666527 0.670669
d_1m5hmU/d_1m5hmU1289.xyz
6 -1.453898 0.380737 0.029905
6 0.555698 -0.868306 -0.155069
6 1.323675 0.366209 -0.215337
6 0.643148 1.538545 -0.120889
1 1.142622 2.501334 -0.144737
7 -0.719758 1.573620 0.002640
7 -0.799648 -0.801932 -0.043541
8 -2.708371 0.451450 0.129168
8 1.142829 -1.997426 -0.194948
6 2.813949 0.299824 -0.379092
1 3.068343 -0.194898 -1.326121
1 3.244309 1.304294 -0.391090
8 3.456218 -0.408063 0.706225
1 3.060303 -1.296447 0.711717
6 -1.443017 2.846152 0.094505
1 -2.133011 2.950832 -0.746060
1 -2.012233 2.889662 1.025589
1 -0.720461 3.661837 0.073861
8 -2.450857 -3.014230 0.040339
1 -3.334797 -2.625552 0.103046
1 -1.835102 -2.225692 0.009686

d_1m5hmU/d_1m5hmU1234.xyz
6 -1.534107 -0.259191 -0.027845
6 0.592065 0.777368 0.167569
6 1.232255 -0.528342 0.210436
6 0.435796 -1.624985 0.112211
1 0.834369 -2.633925 0.125920
7 -0.924432 -1.520584 -0.002035
7 -0.762876 0.849893 0.054472
8 -2.788727 -0.202799 -0.132489
8 1.288611 1.842282 0.224048
6 2.723133 -0.612209 0.363051
1 3.030382 -0.172241 1.321490
1 3.053957 -1.653332 0.342481
8 3.422675 0.062690 -0.707443
1 3.106207 0.982535 -0.686354
6 -1.773928 -2.712557 -0.093870
1 -2.473286 -2.743338 0.744804
1 -2.342045 -2.700328 -1.026609
1 -1.139475 -3.598385 -0.068399
8 -1.884886 3.367155 -0.069650
1 -1.129027 3.967387 -0.001667
1 -1.482600 2.451340 -0.023769
d_1m5hmU/d_1m5hmU1246.xyz
6 -1.380385 1.116741 -0.048328
6 0.759368 0.152385 0.143300
6 0.175541 -1.175806 0.216799
6 -1.177466 -1.269509 0.127651
1 -1.697293 -2.221103 0.160618
7 -1.964597 -0.158516 -0.000540
7 -0.035059 1.241897 0.018927
8 -2.148240 2.112283 -0.158875
8 2.034355 0.280288 0.187457
6 1.066354 -2.371275 0.388080
1 1.623194 -2.295948 1.331683
1 0.475355 -3.290130 0.411779
8 2.006785 -2.518138 -0.700884

1 2.522814 -1.693858 -0.716744
6 -3.424964 -0.268680 -0.079870
1 -3.890147 0.267729 0.750242
1 -3.785412 0.155577 -1.019626
1 -3.700379 -1.322051 -0.029172
8 3.237935 2.688193 -0.009392
1 2.564376 3.375438 -0.107437
1 2.727703 1.836540 0.057698
d_1m5hmU/d_1m5hmU1231.xyz
6 -1.254118 0.259527 0.028315
6 0.730395 -0.996622 -0.142707
6 1.511109 0.229929 -0.210262
6 0.841966 1.408663 -0.120355
1 1.347345 2.368225 -0.146880
7 -0.520814 1.450558 0.001793
7 -0.624314 -0.929080 -0.036746
8 -2.516013 0.356979 0.122008
8 1.319269 -2.128339 -0.171648
6 3.000725 0.148953 -0.376997
1 3.247428 -0.333187 -1.332811
1 3.443927 1.148036 -0.373650
8 3.633902 -0.586111 0.695068
1 3.209038 -1.461944 0.692684
6 -1.236597 2.728018 0.087409
1 -1.922854 2.834725 -0.755842
1 -1.808557 2.778148 1.016362
1 -0.508389 3.538719 0.066857
8 -4.123901 -1.826742 0.034462
1 -3.464941 -1.086116 0.064997
1 -3.594037 -2.634051 -0.018590
d_1m5hmU/d_1m5hmU1222.xyz
6 -1.939028 0.887692 -0.080080
6 0.416144 0.767422 -0.123505
6 0.363939 -0.666037 0.114443
6 -0.866153 -1.237184 0.218562
1 -0.998181 -2.301728 0.381981

7 -2.011875 -0.498214 0.129718
7 -0.732360 1.483532 -0.208606
8 -3.023597 1.530488 -0.144841
8 1.542360 1.359526 -0.269843
6 1.627477 -1.467441 0.227096
1 2.249660 -1.085430 1.045897
1 1.394846 -2.514463 0.435961
8 2.402628 -1.466893 -0.995074
1 2.630031 -0.541632 -1.180712
6 -3.330956 -1.125863 0.258513
1 -3.868269 -0.705330 1.111577
1 -3.918985 -0.957677 -0.646416
1 -3.195012 -2.196755 0.408575
8 4.064318 0.992999 0.699585
1 4.270875 0.053043 0.599741
1 3.130466 1.072250 0.386817
d_1m5hmU/d_1m5hmU1277.xyz
6 -1.136355 0.431730 0.558692
6 1.145251 0.971356 0.333278
6 1.465375 -0.432068 0.119169
6 0.437095 -1.319940 0.116618
1 0.585330 -2.382045 -0.047326
7 -0.854025 -0.919616 0.331848
7 -0.145744 1.343399 0.551528
8 -2.348738 0.749806 0.763672
8 2.072459 1.847672 0.303468
6 2.896125 -0.840157 -0.080850
1 3.483322 -0.609910 0.818716
1 2.968122 -1.914117 -0.270219
8 3.499753 -0.179652 -1.216288
1 3.400424 0.772988 -1.041869
6 -1.955788 -1.888643 0.346370
1 -2.473047 -1.855418 1.307591
1 -2.670242 -1.663403 -0.447809
1 -1.545069 -2.886201 0.190738
8 -4.022177 0.574309 -1.428431

1 -4.050266 1.474004 -1.782787
1 -3.420797 0.626870 -0.649691
d_1m5hmU/d_1m5hmU1278.xyz
6 1.907856 0.905805 0.102635
6 -0.441189 0.721484 0.189625
6 -0.357216 -0.700504 -0.099023
6 0.883915 -1.234458 -0.253665
1 1.039292 -2.288610 -0.458451
7 2.011676 -0.467655 -0.166053
7 0.689077 1.465187 0.276624
8 2.976967 1.573177 0.174998
8 -1.581398 1.275296 0.378921
6 -1.604972 -1.528151 -0.200927
1 -2.251815 -1.149263 -1.001964
1 -1.353779 -2.567169 -0.427999
8 -2.354428 -1.555993 1.036500
1 -2.599056 -0.635760 1.228520
6 3.343688 -1.052475 -0.351682
1 3.853256 -0.571372 -1.189524
1 3.944742 -0.918204 0.550436
1 3.233077 -2.116883 -0.558686
8 -4.002369 0.928212 -0.839902
1 -4.362859 1.825578 -0.821801
1 -3.110792 1.003230 -0.420435
d_1m5hmU/d_1m5hmU1125.xyz
6 1.399016 -0.529540 0.033271
6 -0.411693 0.995859 -0.211889
6 -1.345147 -0.122868 -0.278800
6 -0.830356 -1.379739 -0.189673
1 -1.459475 -2.262665 -0.234717
7 0.508367 -1.609647 -0.040101
7 0.919080 0.730460 -0.060434
8 2.626342 -0.775312 0.183672
8 -0.810926 2.197037 -0.296806
6 -2.820441 0.105682 -0.412258
1 -3.023092 0.900931 -1.133363

1 -3.313503 -0.811148 -0.757604
8 -3.434826 0.554603 0.821563
1 -3.270278 -0.124209 1.493528
6 1.042896 -2.972476 0.048707
1 1.754711 -3.152144 -0.760058
1 1.550499 -3.117876 1.004944
1 0.216127 -3.678090 -0.033266
8 2.815249 2.720426 0.150952
1 2.126118 1.997124 0.073860
1 3.654415 2.246843 0.239232
d_1m5hmU/d_1m5hmU1003.xyz
6 -1.923585 -0.858954 0.079866
6 0.397370 -0.853751 -0.364035
6 0.408142 0.601676 -0.385508
6 -0.780205 1.231237 -0.167509
1 -0.867201 2.312829 -0.175245
7 -1.935450 0.544463 0.068126
7 -0.764675 -1.517792 -0.137843
8 -3.014842 -1.456628 0.294773
8 1.467680 -1.528026 -0.570719
6 1.660053 1.396206 -0.639540
1 2.264500 0.904842 -1.410772
1 1.393409 2.394113 -0.998327
8 2.467265 1.603012 0.542312
1 2.964676 0.774291 0.712951
6 -3.203314 1.245830 0.295231
1 -3.932266 0.964899 -0.468218
1 -3.605204 0.989773 1.278025
1 -3.022646 2.319582 0.246108
8 3.705882 -0.910421 0.742050
1 2.906579 -1.183441 0.213471
1 4.440374 -0.900549 0.111671
d_1m5hmU/d_1m5hmU1148.xyz
6 -1.257769 -1.216814 0.096587
6 0.830046 -0.140186 -0.144220
6 0.165874 1.147714 -0.284696

6 -1.194608 1.156933 -0.231725
1 -1.768095 2.072486 -0.333529
7 -1.918273 0.014092 -0.050808
7 0.091097 -1.264490 0.040547
8 -1.967277 -2.245180 0.277016
8 2.104591 -0.217050 -0.201015
6 0.943251 2.418646 -0.450713
1 1.775365 2.275510 -1.143804
1 0.292078 3.207188 -0.847213
8 1.557431 2.868785 0.783173
1 0.848134 3.012615 1.428075
6 -3.383806 0.041078 -0.000903
1 -3.800532 -0.572860 -0.802542
1 -3.735931 -0.345293 0.958107
1 -3.719860 1.071004 -0.121180
8 3.390525 -2.579881 0.003375
1 2.850388 -1.745978 -0.060012
1 2.748596 -3.279501 0.187559
d_1m5hmU/d_1m5hmU1101.xyz
6 -1.398309 -0.526187 -0.031463
6 0.414342 0.996874 0.213755
6 1.346652 -0.122874 0.278674
6 0.830388 -1.379076 0.188673
1 1.458555 -2.262747 0.232230
7 -0.508710 -1.607334 0.040055
7 -0.916865 0.733169 0.063251
8 -2.625970 -0.770599 -0.181170
8 0.814944 2.197554 0.299527
6 2.822310 0.103973 0.410965
1 3.026483 0.898408 1.132533
1 3.314717 -0.813668 0.755095
8 3.436069 0.553238 -0.823049
1 3.270340 -0.124923 -1.495383
6 -1.044743 -2.969501 -0.049729
1 -1.756324 -3.149156 0.759243
1 -1.552992 -3.113512 -1.005836

1 -0.218669 -3.676061 0.031180
8 -2.826217 2.710093 -0.155963
1 -2.122568 2.001617 -0.072963
1 -3.654966 2.217659 -0.239260
d_1m5hmU/5OHMe_meur1ng_10_kicked07_SMD_opt_freq_b.xyz
6 1.883261 0.597192 0.308485
6 -0.341243 1.321264 0.006435
6 -0.663588 0.078100 -0.679222
6 0.318054 -0.854428 -0.791089
1 0.156187 -1.816627 -1.265488
7 1.578447 -0.625020 -0.312122
7 0.920123 1.532539 0.464577
8 3.069207 0.770706 0.707505
8 -1.251012 2.200190 0.184375
6 -2.053372 -0.138396 -1.198159
1 -2.308420 0.616446 -1.951889
1 -2.151083 -1.127886 -1.650313
8 -3.041685 -0.076520 -0.135417
1 -2.936121 0.805203 0.266493
6 2.649159 -1.609864 -0.497914
1 3.451035 -1.185712 -1.106791
1 3.061603 -1.909955 0.467686
1 2.235856 -2.483601 -1.001797
8 -2.051287 -1.945163 1.705831
1 -2.446247 -1.280579 1.098374
1 -1.108213 -1.926828 1.486968
d_1m5hmU/d_1m5hmU1212.xyz
6 1.092529 -0.498524 -0.446932
6 -1.154659 -1.021040 0.028434
6 -1.439876 0.385420 0.269981
6 -0.426525 1.272857 0.094244
1 -0.558045 2.340765 0.233541
7 0.829572 0.863436 -0.264520
7 0.106691 -1.405060 -0.307888
8 2.278271 -0.832888 -0.754166
8 -2.088927 -1.885925 0.120591

6 -2.821463 0.797477 0.689308
1 -3.068184 0.353680 1.663582
1 -2.888559 1.884364 0.782321
8 -3.824961 0.402377 -0.273402
1 -3.725648 -0.562102 -0.364209
6 1.915205 1.832420 -0.454366
1 2.683840 1.702524 0.310447
1 2.368004 1.696684 -1.438219
1 1.501331 2.838175 -0.383013
8 4.363620 -0.350932 0.979678
1 4.709507 -1.233384 1.172871
1 3.608628 -0.502050 0.363907
d_1m5hmU/d_1m5hmU695.xyz
6 1.231425 0.900687 -0.000008
6 0.026497 -1.147896 -0.000009
6 -1.229916 -0.414687 -0.000001
6 -1.169799 0.941338 0.000002
1 -2.057187 1.561762 0.000008
7 0.028876 1.610880 -0.000003
7 1.197786 -0.451616 -0.000013
8 2.315740 1.547868 -0.000007
8 0.040573 -2.419519 -0.000012
6 -2.514406 -1.192907 0.000004
1 -2.549839 -1.845167 -0.883761
1 -2.549834 -1.845165 0.883771
8 -3.634628 -0.295833 0.000006
1 -4.440734 -0.831211 0.000009
6 0.077490 3.076240 0.000007
1 0.600320 3.438000 0.888391
1 0.600326 3.438010 -0.888370
1 -0.942165 3.461031 0.000005
8 3.558300 -1.871540 0.000022
1 2.698377 -1.356431 0.000008
1 3.286468 -2.800139 -0.000005
d_1m5hmU/d_1m5hmU1223.xyz
6 1.907355 -0.905592 -0.102589

6 -0.441576 -0.721063 -0.192031
6 -0.357795 0.700889 0.096821
6 0.883212 1.234736 0.252740
1 1.038477 2.288870 0.457687
7 2.011002 0.467825 0.166327
7 0.688715 -1.464819 -0.278112
8 2.976475 -1.573088 -0.173696
8 -1.581718 -1.274748 -0.382332
6 -1.605587 1.528709 0.197649
1 -2.251997 1.151738 0.999998
1 -1.354269 2.568212 0.422375
8 -2.355687 1.553713 -1.039330
1 -2.601133 0.633015 -1.228303
6 3.342838 1.052442 0.353863
1 3.850979 0.571527 1.192687
1 3.945296 0.917729 -0.547245
1 3.232123 2.116946 0.560330
8 -3.990451 -0.932782 0.863415
1 -4.411324 -1.790601 0.712856
1 -3.105803 -1.003959 0.428922
d_1m5hmU/d_1m5hmU1091.xyz
6 1.469373 0.505988 -0.064332
6 -0.406150 -0.939139 0.178683
6 -1.288265 0.217815 0.270498
6 -0.718910 1.451412 0.192350
1 -1.307634 2.360409 0.254313
7 0.626850 1.623461 0.034429
7 0.934020 -0.731883 0.015143
8 2.704212 0.701954 -0.223418
8 -0.855819 -2.123410 0.252270
6 -2.765324 0.050114 0.426257
1 -2.981512 -0.751235 1.141702
1 -3.214685 0.980014 0.795239
8 -3.344671 -0.291809 -0.860735
1 -4.264397 -0.558098 -0.711092
6 1.219602 2.963148 -0.035394

1 1.946671 3.096253 0.768799
1 1.723593 3.104211 -0.994194
1 0.425352 3.702497 0.068530
8 2.484970 -3.009405 -0.096162
1 1.835587 -3.719763 0.005223
1 1.939442 -2.170012 -0.059531
d_1m5hmU/d_1m5hmU1210.xyz
6 1.043595 -0.605263 0.100884
6 -1.277675 -1.010268 0.151505
6 -1.536131 0.421416 0.192622
6 -0.464702 1.255470 0.159953
1 -0.567298 2.335458 0.173647
7 0.817473 0.775662 0.117508
7 0.004142 -1.462534 0.114721
8 2.244012 -1.012798 0.071100
8 -2.252920 -1.834194 0.138289
6 -2.951485 0.914920 0.272846
1 -3.415546 0.579803 1.210583
1 -2.980937 2.007106 0.248030
8 -3.754718 0.455266 -0.837932
1 -3.690714 -0.515987 -0.815844
6 1.966957 1.687138 0.090662
1 2.625950 1.486023 0.937531
1 2.529570 1.560196 -0.836623
1 1.603038 2.712659 0.153299
8 4.805463 -0.111554 -0.349789
1 5.263584 -0.953616 -0.478754
1 3.862990 -0.357778 -0.201646
d_1m5hmU/d_1m5hmU640.xyz
6 -1.261127 0.783576 0.016151
6 0.069162 -1.185332 0.022285
6 1.277368 -0.374222 0.006212
6 1.132876 0.975239 -0.002135
1 1.978551 1.651399 -0.013512
7 -0.105983 1.567905 0.003546
7 -1.143746 -0.564217 0.027128

8 -2.384230 1.361261 0.017388
8 0.137223 -2.455019 0.032328
6 2.607723 -1.071131 0.000116
1 2.697306 -1.702255 0.895396
1 2.669124 -1.737673 -0.871411
8 3.670624 -0.107570 -0.035953
1 4.507920 -0.592838 -0.034188
6 -0.246523 3.027218 -0.008128
1 -0.793530 3.348085 -0.897723
1 -0.788738 3.362413 0.879037
1 0.747103 3.474918 -0.014340
8 -3.534833 -1.934043 -0.046901
1 -2.658791 -1.448634 -0.015939
1 -4.197983 -1.230363 -0.083935
d_1m5hmU/d_1m5hmU1041.xyz
6 1.410649 -0.516264 0.039347
6 -0.419751 0.985912 -0.202311
6 -1.338678 -0.144430 -0.268691
6 -0.808125 -1.394583 -0.180039
1 -1.426224 -2.285084 -0.223303
7 0.533288 -1.607587 -0.034249
7 0.914590 0.737341 -0.052313
8 2.641001 -0.747195 0.187678
8 -0.833449 2.182311 -0.286487
6 -2.811610 0.067615 -0.409943
1 -3.010439 0.870483 -1.128654
1 -3.293385 -0.850340 -0.767823
8 -3.366693 0.435550 0.880503
1 -4.280730 0.724646 0.738616
6 1.084870 -2.963773 0.051735
1 1.798334 -3.132872 -0.757846
1 1.594940 -3.104440 1.007363
1 0.266896 -3.679517 -0.030957
8 2.781714 2.757118 0.130796
1 3.627718 2.296773 0.223804
1 2.103030 2.022942 0.064221

d_1m5hmU/d_1m5hmU1250.xyz
6 1.097294 -0.484645 -0.463509
6 -1.145279 -1.021194 0.017482
6 -1.435157 0.381656 0.273883
6 -0.426223 1.275013 0.102596
1 -0.561443 2.340941 0.253067
7 0.829940 0.874236 -0.265529
7 0.116084 -1.396713 -0.328379
8 2.282987 -0.810616 -0.780111
8 -2.075470 -1.890796 0.105715
6 -2.816653 0.783780 0.702892
1 -3.058321 0.327921 1.672845
1 -2.887324 1.869270 0.808596
8 -3.822223 0.396271 -0.260796
1 -3.720364 -0.566735 -0.363122
6 1.910780 1.848998 -0.453390
1 2.691666 1.704450 0.296135
1 2.347980 1.734119 -1.447037
1 1.496645 2.852347 -0.354823
8 4.346279 -0.386060 0.994778
1 4.511843 -1.263352 1.367162
1 3.595992 -0.513659 0.368114
d_1m5mC/d_1m5mC_1_038.xyz
6 -0.856645 0.825833 -0.093935
6 0.343117 -1.241278 -0.090467
6 1.604463 -0.501325 0.027437
6 1.535973 0.852055 0.071542
1 2.424675 1.468608 0.155137
7 0.342302 1.537865 0.006859
7 -0.830642 -0.514788 -0.151542
8 -1.941736 1.486130 -0.128835
7 0.255800 -2.552222 -0.143936
1 1.188219 -2.958889 -0.085189
6 2.921126 -1.225940 0.091409
1 2.969479 -1.901576 0.955385
1 3.085262 -1.840403 -0.803365

1 3.752460 -0.519158 0.170590
6 0.304445 3.000531 0.057408
1 -0.173394 3.402101 -0.839653
1 -0.252127 3.341470 0.934402
1 1.326212 3.376963 0.115387
8 -3.258180 -1.698486 0.206877
1 -3.853458 -0.951325 0.360257
1 -2.355096 -1.274187 0.052674
d_1m5mC/d_1m5mC_2_077.xyz
6 -1.659184 -0.904310 -0.000417
6 0.692412 -0.602893 -0.007632
6 0.521082 0.847195 -0.005399
6 -0.750966 1.318394 0.001202
1 -0.971166 2.380579 0.003610
7 -1.845270 0.483189 0.004799
7 -0.417500 -1.415240 -0.005977
8 -2.700446 -1.634387 0.000737
7 1.910196 -1.114843 -0.011913
1 1.843873 -2.133699 -0.013668
6 1.709481 1.766747 -0.009968
1 2.343720 1.601736 0.868518
1 2.340080 1.597314 -0.890382
1 1.392368 2.814215 -0.011890
6 -3.206952 1.019334 0.010661
1 -3.752887 0.688143 -0.876546
1 -3.746944 0.683576 0.899766
1 -3.156900 2.108688 0.013147
8 4.440398 -0.189335 0.003037
1 4.952059 -0.998750 0.139013
1 3.468962 -0.490567 -0.000797
d_1m5mC/d_1m5mC_1_027.xyz
6 0.904058 -1.189868 -0.002409
6 -0.901668 0.343627 -0.001976
6 0.037109 1.466391 0.002968
6 1.359937 1.165685 0.002321
1 2.125508 1.934402 0.004571

7 1.820394 -0.129609 -0.001556
7 -0.413322 -0.939460 -0.002845
8 1.377165 -2.370266 -0.002818
7 -2.213962 0.502903 -0.005529
1 -2.468022 1.487659 -0.002284
6 -0.447209 2.890094 0.007207
1 -1.061446 3.110796 -0.875523
1 -1.065948 3.103723 0.888505
1 0.395407 3.587927 0.012155
6 3.256088 -0.417057 -0.001572
1 3.534661 -0.989999 0.886638
1 3.534362 -0.990093 -0.889777
1 3.801785 0.527264 -0.001763
8 -4.055234 -1.432353 -0.036924
1 -3.325448 -0.716528 -0.014970
1 -3.647972 -2.224266 0.340660
d_1m5mC/d_1m5mC_1_080.xyz
6 -0.897008 -1.189683 -0.000760
6 0.903885 0.349639 -0.002311
6 -0.038430 1.469412 -0.000748
6 -1.360266 1.164487 0.000901
1 -2.128298 1.930727 0.002326
7 -1.816667 -0.132272 0.001056
7 0.419516 -0.935049 -0.002746
8 -1.366400 -2.371578 -0.000631
7 2.215641 0.512606 -0.003422
1 2.467223 1.497996 -0.003434
6 0.441506 2.894571 -0.000537
1 1.058506 3.113090 0.880795
1 1.056122 3.114191 -0.883267
1 -0.403264 3.589824 0.001012
6 -3.251471 -0.424035 0.002750
1 -3.529478 -0.996933 -0.885656
1 -3.527013 -0.998779 0.890719
1 -3.799993 0.518653 0.004527
8 4.041905 -1.438721 0.013339

1 3.320072 -0.714826 0.004315
1 3.563349 -2.274888 -0.072979
d_1m5mC/d_1m5mC_2_048.xyz
6 -1.669619 -0.890410 0.000007
6 0.687211 -0.629408 -0.000002
6 0.540401 0.823695 0.000009
6 -0.723710 1.316275 0.000003
1 -0.925786 2.382042 0.000004
7 -1.831985 0.500077 -0.000008
7 -0.436870 -1.422333 0.000005
8 -2.723461 -1.602204 0.000014
7 1.894867 -1.164642 -0.000018
1 1.807641 -2.181999 -0.000028
6 1.741631 1.726763 0.000009
1 2.371363 1.551487 0.880161
1 2.371347 1.551495 -0.880157
1 1.438529 2.778349 0.000015
6 -3.184112 1.059684 -0.000013
1 -3.732959 0.735916 -0.888168
1 -3.732968 0.735913 0.888136
1 -3.114768 2.147887 -0.000011
8 4.453136 -0.344774 0.000003
1 4.484437 0.621484 -0.000008
1 3.462875 -0.578055 -0.000009
d_1m5mC/d_1m5mC_2_069.xyz
6 0.638622 1.003881 -0.001856
6 -0.074079 -1.280255 -0.002110
6 -1.469996 -0.843590 0.001104
6 -1.706263 0.490790 0.001357
1 -2.712384 0.895671 0.003489
7 -0.692470 1.426587 -0.000505
7 0.915021 -0.311524 -0.003530
8 1.548747 1.889536 -0.001593
7 0.211877 -2.563059 -0.003435
1 1.229127 -2.665864 -0.005286
6 -2.591016 -1.843341 0.004295

1 -2.546211 -2.496547 -0.875404
1 -2.540140 -2.497112 0.883243
1 -3.562963 -1.339902 0.007872
6 -0.991045 2.859209 -0.000671
1 -0.573664 3.340848 0.887559
1 -0.573887 3.340340 -0.889275
1 -2.073398 2.991231 -0.000633
8 3.557938 -0.996578 -0.006009
1 4.037367 -0.161991 0.093042
1 2.584341 -0.734531 -0.004219
d_1m5mC/d_1m5mC_2_047.xyz
6 -0.769831 0.892456 -0.118510
6 0.257112 -1.267944 -0.112137
6 1.571049 -0.640684 0.028993
6 1.615018 0.712909 0.083942
1 2.549837 1.252737 0.188720
7 0.482262 1.497757 0.008829
7 -0.855287 -0.447450 -0.186437
8 -1.794254 1.641407 -0.165856
7 0.157069 -2.577254 -0.165203
1 -0.831894 -2.819160 -0.259559
6 2.816886 -1.475300 0.117489
1 2.774349 -2.166527 0.967666
1 2.948968 -2.091672 -0.779894
1 3.703384 -0.843506 0.231917
6 0.566191 2.957296 0.074106
1 0.167109 3.407224 -0.838875
1 -0.000292 3.336684 0.928500
1 1.612148 3.245626 0.183438
8 -3.379662 -1.397713 0.271189
1 -2.452528 -1.055901 0.078262
1 -3.906594 -0.598818 0.413540
d_1m5mC/d_1m5mC_1_090.xyz
6 -0.401931 1.155170 -0.073879
6 -0.243187 -1.228836 -0.059576
6 1.218951 -1.125429 0.005617

6 1.758750 0.117886 0.038535
1 2.830469 0.277989 0.091309
7 0.990335 1.261053 0.001962
7 -0.974763 -0.057881 -0.109995
8 -1.079898 2.228689 -0.103370
7 -0.904509 -2.365781 -0.073416
1 -0.246325 -3.142117 -0.030730
6 2.079409 -2.358535 0.042679
1 1.861189 -2.977731 0.922706
1 1.914735 -2.989735 -0.840246
1 3.140219 -2.092648 0.073347
6 1.605465 2.587160 0.076211
1 1.360037 3.177964 -0.809986
1 1.255788 3.123216 0.962427
1 2.687574 2.467433 0.134832
8 -3.683249 -0.150287 0.145328
1 -2.678852 -0.127672 0.039733
1 -3.901838 -1.090142 0.213566
d_1m5mC/d_1m5mC_2_027.xyz
6 0.228255 -1.197206 -0.093248
6 0.390334 1.189371 -0.078869
6 -1.066342 1.286506 0.012819
6 -1.767846 0.127659 0.054054
1 -2.849898 0.112840 0.126421
7 -1.162870 -1.111161 -0.004442
7 0.960251 -0.070932 -0.136285
8 0.755646 -2.352075 -0.128040
7 1.119402 2.282939 -0.102680
1 2.103638 2.012958 -0.164773
6 -1.747101 2.624709 0.061778
1 -1.409373 3.214256 0.922317
1 -1.522644 3.218791 -0.832382
1 -2.833351 2.508637 0.131070
6 -1.950186 -2.340769 0.093979
1 -1.770592 -2.981103 -0.772860
1 -1.688914 -2.895202 0.999829

1 -3.008208 -2.078698 0.129164
8 3.670479 -0.311396 0.176912
1 3.955597 0.580689 0.420368
1 2.674582 -0.232939 0.050645
d_1m5mC/d_1m5mC_2_085.xyz
6 -0.883847 0.323854 -0.034894
6 0.932537 -1.203271 -0.013610
6 1.875712 -0.084278 0.006298
6 1.357446 1.168049 -0.001523
1 1.985022 2.052727 0.012914
7 -0.001412 1.405392 -0.033400
7 -0.423766 -0.930183 -0.036539
8 -2.134012 0.597539 -0.036115
7 1.392114 -2.435890 -0.007899
1 0.592001 -3.072540 -0.021938
6 3.358467 -0.325141 0.035618
1 3.652289 -0.899014 0.922770
1 3.685803 -0.905407 -0.835377
1 3.908904 0.621177 0.043149
6 -0.529178 2.769168 0.026700
1 -1.197354 2.960699 -0.816020
1 -1.082727 2.928210 0.956480
1 0.305687 3.469603 -0.014757
8 -3.939204 -1.392999 0.057348
1 -3.486370 -2.245222 -0.004857
1 -3.212906 -0.712066 0.021093
d_1m5mC/d_1m5mC_2_080.xyz
6 0.760088 -0.399721 -0.536889
6 -1.499026 -0.954986 -0.068047
6 -1.793984 0.454219 0.192420
6 -0.769959 1.335777 0.084562
1 -0.900829 2.396863 0.267551
7 0.506676 0.945447 -0.262229
7 -0.212127 -1.308802 -0.434688
8 1.954770 -0.710457 -0.880428
7 -2.454289 -1.851379 0.051760

1 -2.054057 -2.766636 -0.166785
6 -3.180231 0.893914 0.569384
1 -3.904788 0.636113 -0.212485
1 -3.519460 0.397486 1.486358
1 -3.218594 1.976021 0.730539
6 1.592813 1.922016 -0.366545
1 2.408111 1.663809 0.313055
1 1.983707 1.956511 -1.386977
1 1.206213 2.905928 -0.098823
8 3.739472 -0.654922 1.184071
1 3.819487 -1.577309 1.464217
1 3.086245 -0.669917 0.440993
d_1m5mC/d_1m5mC_1_052.xyz
6 0.889115 0.245105 -0.183743
6 -0.999939 -1.185058 -0.091066
6 -1.872934 -0.014782 0.065686
6 -1.284416 1.206388 0.092263
1 -1.857278 2.120194 0.208147
7 0.077589 1.372070 -0.027668
7 0.359948 -0.978044 -0.215051
8 2.148130 0.453465 -0.295699
7 -1.439532 -2.426056 -0.121974
1 -2.453662 -2.431569 -0.022676
6 -3.363956 -0.168253 0.195842
1 -3.796900 -0.667268 -0.680977
1 -3.633623 -0.772340 1.072045
1 -3.849214 0.807043 0.299610
6 0.682591 2.703719 0.038233
1 1.378794 2.769529 0.878411
1 1.223782 2.930833 -0.883734
1 -0.109210 3.441188 0.175187
8 3.872380 -1.541542 0.269535
1 3.205144 -0.833594 0.060124
1 4.439287 -1.157914 0.952745
d_1m5mC/d_1m5mC_2_053.xyz
6 -0.687129 -0.565292 -0.326338

6 1.636138 -0.922690 0.013201
6 1.846375 0.521708 0.117621
6 0.758525 1.319547 -0.011002
1 0.826154 2.400241 0.053661
7 -0.506206 0.816955 -0.239925
7 0.354365 -1.394167 -0.209293
8 -1.875995 -0.995618 -0.526479
7 2.661814 -1.737376 0.135881
1 2.317386 -2.695126 0.037138
6 3.216898 1.089034 0.357667
1 3.641007 0.724732 1.301150
1 3.915235 0.793734 -0.434494
1 3.185903 2.182660 0.396339
6 -1.657926 1.717347 -0.329269
1 -2.268967 1.467013 -1.197980
1 -2.276418 1.651902 0.570247
1 -1.293899 2.739959 -0.435453
8 -4.170076 -0.299271 0.752511
1 -3.317425 -0.507845 0.294389
1 -4.607512 -1.153963 0.868829
d_1m5mC/d_1m5mC_1_028.xyz
6 0.748327 -0.454911 -0.490369
6 -1.524594 -0.966587 -0.049492
6 -1.798108 0.457412 0.176256
6 -0.757870 1.319893 0.066816
1 -0.875818 2.386867 0.223639
7 0.515313 0.902773 -0.251752
7 -0.238229 -1.345005 -0.381299
8 1.941178 -0.792944 -0.813441
7 -2.438887 -1.909425 0.047537
1 -3.334447 -1.491144 0.294973
6 -3.183805 0.936685 0.511572
1 -3.898233 0.681061 -0.282089
1 -3.557663 0.478827 1.436703
1 -3.200980 2.022638 0.643977
6 1.614542 1.862685 -0.377800

1 2.412510 1.631124 0.330927
1 2.026219 1.843012 -1.390166
1 1.232031 2.862128 -0.168217
8 3.889101 -0.560174 1.076894
1 3.924607 -1.438107 1.481273
1 3.171213 -0.620931 0.398055
d_1m5mC/d_1m5mC_2_026.xyz
6 -0.763352 -0.393212 -0.542900
6 1.494397 -0.954301 -0.074294
6 1.791095 0.453029 0.194277
6 0.768601 1.336735 0.089381
1 0.900713 2.396646 0.278169
7 -0.508150 0.950145 -0.261278
7 0.207397 -1.304273 -0.444179
8 -1.958229 -0.700729 -0.888866
7 2.448129 -1.852819 0.041592
1 2.046706 -2.766052 -0.183171
6 3.177248 0.888412 0.576651
1 3.513563 0.386461 1.491709
1 3.903179 0.633548 -0.204895
1 3.217022 1.969565 0.743750
6 -1.592545 1.928990 -0.361683
1 -1.980770 1.971459 -1.382860
1 -2.409834 1.666759 0.313959
1 -1.205289 2.910405 -0.085789
8 -3.740475 -0.683419 1.177699
1 -3.082359 -0.682744 0.438665
1 -3.597584 -1.522157 1.638273
d_1m5mC/d_1m5mC_1_064.xyz
6 0.899698 0.305643 -0.036285
6 -0.933593 -1.197826 -0.014505
6 -1.861276 -0.060049 0.006712
6 -1.325294 1.185336 -0.000093
1 -1.940860 2.078591 0.015210
7 0.034619 1.402761 -0.032095
7 0.422575 -0.939599 -0.037277

8 2.154139 0.561632 -0.040344
7 -1.322084 -2.456337 -0.011795
1 -2.340046 -2.498880 0.006524
6 -3.349765 -0.274020 0.035361
1 -3.691991 -0.842267 -0.839499
1 -3.658621 -0.839657 0.924319
1 -3.882456 0.681715 0.044316
6 0.584196 2.758054 0.027874
1 1.151376 2.902433 0.951652
1 1.245791 2.943029 -0.821545
1 -0.239898 3.471857 -0.000231
8 3.927426 -1.458210 0.059475
1 3.465510 -2.305755 0.000055
1 3.209103 -0.769046 0.019942
d_1m5mC/d_1m5mC_2_070.xyz
6 -0.734177 -0.469184 0.490645
6 1.546977 -0.940436 0.034322
6 1.801533 0.484212 -0.179900
6 0.753599 1.332211 -0.039649
1 0.856112 2.402503 -0.183115
7 -0.510431 0.896246 0.300062
7 0.265319 -1.345204 0.364126
8 -1.924933 -0.832193 0.791593
7 2.533071 -1.801565 -0.093603
1 2.161038 -2.735875 0.091309
6 3.175794 0.976567 -0.534912
1 3.903956 0.715340 0.242456
1 3.534462 0.520820 -1.465479
1 3.182690 2.063993 -0.660887
6 -1.627823 1.838237 0.398433
1 -2.338778 1.685368 -0.418128
1 -2.150545 1.710660 1.348379
1 -1.235291 2.854144 0.341639
8 -3.868986 -0.518004 -1.089849
1 -3.921388 -1.389838 -1.505424
1 -3.152038 -0.601515 -0.412434

d_1m5mC/d_1m5mC_2_082.xyz
6 -0.762197 -0.435450 -0.552799
6 1.504805 -0.937141 -0.062800
6 1.766704 0.479981 0.189371
6 0.726124 1.339009 0.060926
1 0.833551 2.404228 0.235025
7 -0.538423 0.918903 -0.296307
7 0.228311 -1.322072 -0.434523
8 -1.949214 -0.776609 -0.894303
7 2.479360 -1.810395 0.070058
1 2.103014 -2.736728 -0.144065
6 3.138873 0.950990 0.579672
1 3.474368 0.472289 1.507424
1 3.879259 0.697317 -0.188511
1 3.154436 2.035450 0.728331
6 -1.643447 1.871866 -0.421676
1 -2.040219 1.868500 -1.440166
1 -2.451078 1.619658 0.269772
1 -1.273797 2.870379 -0.186366
8 -3.623804 -0.808479 1.268434
1 -3.021326 -0.767518 0.485136
1 -3.643975 0.096552 1.609609
d_1m5mC/d_1m5mC_1_058.xyz
6 -0.771549 -0.402513 -0.545083
6 1.483909 -0.971012 -0.086740
6 1.780380 0.440024 0.187768
6 0.758583 1.325741 0.088219
1 0.892717 2.384868 0.281014
7 -0.516782 0.941981 -0.261605
7 0.196609 -1.313925 -0.452411
8 -1.969013 -0.706581 -0.887307
7 2.377055 -1.934991 -0.002620
1 3.276040 -1.543558 0.274648
6 3.166683 0.879449 0.572401
1 3.504782 0.383908 1.491947
1 3.897303 0.633766 -0.209400

1 3.202377 1.960058 0.740190
6 -1.601630 1.920795 -0.356381
1 -1.994811 1.962627 -1.375590
1 -2.415433 1.658673 0.323475
1 -1.212865 2.902297 -0.082869
8 -3.726757 -0.676540 1.198660
1 -3.797570 -1.601121 1.474051
1 -3.082819 -0.682910 0.447057
d_1m5mC/d_1m5mC_1_088.xyz
6 0.901283 0.308202 -0.038893
6 -0.930070 -1.197395 -0.017001
6 -1.859024 -0.060687 0.007324
6 -1.324525 1.185340 0.002332
1 -1.941078 2.077867 0.020244
7 0.035094 1.404348 -0.031084
7 0.425826 -0.937658 -0.041163
8 2.155480 0.565508 -0.044958
7 -1.317213 -2.456305 -0.015913
1 -2.335104 -2.499964 0.003502
6 -3.347238 -0.276405 0.037306
1 -3.689815 -0.843279 -0.838305
1 -3.654484 -0.844158 0.925477
1 -3.880958 0.678728 0.048799
6 0.583402 2.760074 0.030670
1 1.153349 2.902519 0.953031
1 1.242147 2.947998 -0.820321
1 -0.241525 3.473063 0.007009
8 3.914049 -1.466115 0.048969
1 3.201276 -0.770722 0.014749
1 3.441043 -2.304658 0.140411
d_1m5mU/d_1m5mU24.xyz
7 -0.539869 1.478763 -0.000096
6 0.740937 0.923995 -0.002409
7 0.875506 -0.421414 -0.004109
6 -0.201170 -1.260455 -0.002453
6 -1.544459 -0.694058 0.001168

6 -1.644064 0.661256 0.001920
8 -0.021023 -2.519423 -0.004183
8 1.737024 1.701497 -0.002921
6 -0.677246 2.938320 0.000487
1 -2.602201 1.169446 0.004155
1 -1.737373 3.191339 0.001677
1 -0.203297 3.363776 0.888227
1 -0.205237 3.364150 -0.888105
6 -2.746134 -1.595466 0.003702
1 -2.752677 -2.249076 -0.876650
1 -2.748923 -2.249068 0.884095
1 -3.672253 -1.012848 0.005707
8 3.469782 -1.340745 -0.000393
1 2.519856 -1.018422 -0.002023
1 3.999195 -0.532924 0.057852
d_1m5mU/d_1m5mU12.xyz
7 -1.352956 0.884131 0.000002
6 -0.003545 1.240612 0.000006
7 0.933684 0.266058 0.000010
6 0.604949 -1.058921 0.000006
6 -0.800356 -1.444646 -0.000001
6 -1.716179 -0.440623 -0.000003
8 1.525241 -1.936650 0.000010
8 0.296911 2.467809 0.000006
6 -2.364090 1.945067 -0.000006
1 -2.783792 -0.632540 -0.000008
1 -3.352123 1.484768 -0.000017
1 -2.257476 2.571990 0.888360
1 -2.257457 2.571994 -0.888367
6 -1.186319 -2.896264 -0.000007
1 -0.785295 -3.412294 -0.880449
1 -0.785309 -3.412297 0.880441
1 -2.274375 -3.011963 -0.000015
8 3.620891 0.854015 -0.000015
1 4.041726 -0.017371 -0.000013
1 2.637894 0.653649 0.000003

d_1m5mU/d_1m5mU43.xyz
7 -1.793120 -0.125520 0.000333
6 -0.876700 -1.181213 -0.000070
7 0.448156 -0.914279 -0.000556
6 0.908596 0.362569 -0.000612
6 -0.023058 1.479913 -0.000310
6 -1.346960 1.171517 0.000164
8 2.170433 0.585013 -0.000989
8 -1.330961 -2.362410 0.000026
6 -3.227241 -0.428674 0.000887
1 -2.119214 1.933270 0.000447
1 -3.783403 0.508983 0.001112
1 -3.493515 -1.006634 -0.887231
1 -3.492822 -1.006638 0.889213
6 0.478599 2.895756 -0.000531
1 1.101159 3.095887 0.879626
1 1.100530 3.095822 -0.881148
1 -0.354335 3.605346 -0.000263
8 3.944733 -1.437784 0.006228
1 3.229020 -0.743749 0.002285
1 3.474274 -2.281450 -0.041767
d_1m5mU/d_1m5mU18.xyz
7 1.812481 -0.107444 0.001650
6 0.916207 -1.180222 -0.002111
7 -0.413700 -0.938991 -0.005735
6 -0.897811 0.329031 -0.005140
6 0.012537 1.463985 -0.000057
6 1.342097 1.180962 0.002896
8 -2.163554 0.528406 -0.009271
8 1.393303 -2.352344 -0.002124
6 3.251905 -0.383985 0.004632
1 2.099694 1.957388 0.006430
1 3.790383 0.563910 0.006758
1 3.526590 -0.957753 0.892902
1 3.530510 -0.955984 -0.883545
6 -0.515503 2.870244 0.001485

1 -1.138628 3.060805 -0.880416
1 -1.144141 3.056728 0.880343
1 0.303986 3.595306 0.005743
8 -3.908072 -1.526610 0.003364
1 -4.761141 -1.073665 0.055038
1 -3.228725 -0.797400 -0.000642
d_1m5mU/d_1m5mU100.xyz
7 0.001368 1.392480 -0.000311
6 0.882792 0.313169 -0.000690
7 0.411198 -0.946721 -0.000677
6 -0.929557 -1.199436 -0.000133
6 -1.874389 -0.088336 0.000368
6 -1.353072 1.166295 0.000211
8 -1.348767 -2.402552 -0.000093
8 2.127863 0.576366 -0.001103
6 0.540837 2.755570 -0.000469
1 -1.976991 2.053601 0.000501
1 -0.291070 3.459964 -0.000090
1 1.155078 2.921272 0.887655
1 1.154334 2.921355 -0.889094
6 -3.353974 -0.346723 0.000988
1 -3.655034 -0.927320 -0.879177
1 -3.654273 -0.927367 0.881384
1 -3.915754 0.592306 0.001263
8 3.976628 -1.402122 0.005897
1 3.243939 -0.731849 0.002082
1 3.530199 -2.259049 -0.036864
d_1m5mU/d_1m5mU91.xyz
7 -0.478365 0.785180 -0.029750
6 -0.661875 -0.596644 -0.036657
7 0.403835 -1.418888 -0.022697
6 1.676792 -0.930286 0.002185
6 1.897035 0.511123 0.014821
6 0.793802 1.303635 -0.002733
8 2.664526 -1.736000 0.014271
8 -1.851816 -1.045387 -0.057865

6 -1.651850 1.664407 -0.044469
1 0.858479 2.386419 0.002928
1 -1.314335 2.700720 -0.062072
1 -2.259137 1.469357 -0.930813
1 -2.261049 1.500249 0.847086
6 3.291268 1.068815 0.045449
1 3.833596 0.729950 0.936209
1 3.870421 0.735530 -0.823933
1 3.275781 2.162946 0.048567
8 -4.456875 -0.204809 0.085942
1 -3.496161 -0.423312 0.039532
1 -4.903599 -1.062631 0.099255
d_1m5mU/d_1m5mU32.xyz
7 -1.835973 0.478514 0.005568
6 -1.652590 -0.907065 0.001048
7 -0.400497 -1.415175 -0.010594
6 0.689513 -0.604121 -0.016398
6 0.526180 0.841943 -0.011788
6 -0.748829 1.314351 -0.001048
8 1.858060 -1.128581 -0.026555
8 -2.683620 -1.641255 0.008165
6 -3.201105 1.011316 0.017510
1 -0.970322 2.376211 0.002682
1 -3.153024 2.100327 0.015858
1 -3.748507 0.672539 -0.865212
1 -3.732541 0.674359 0.910684
6 1.721546 1.751797 -0.019632
1 2.354754 1.583304 0.858793
1 2.347300 1.576860 -0.902129
1 1.411590 2.801113 -0.021926
8 4.424846 -0.243762 0.043886
1 3.461410 -0.473886 0.018745
1 4.882052 -1.094728 -0.004432
d_1m5mU/d_1m5mU46.xyz
7 -0.495009 -0.881270 -0.270778
6 -0.728840 0.479205 -0.463404

7 0.278832 1.361754 -0.344277
6 1.544032 0.954454 -0.035101
6 1.813959 -0.461491 0.183498
6 0.767013 -1.317438 0.050555
8 2.480590 1.813650 0.058880
8 -1.913795 0.841450 -0.757387
6 -1.605098 -1.827808 -0.423160
1 0.873772 -2.387933 0.189107
1 -1.215950 -2.841611 -0.329408
1 -2.361700 -1.659937 0.346367
1 -2.067728 -1.708238 -1.404972
6 3.199519 -0.927577 0.528797
1 3.914600 -0.657697 -0.257448
1 3.555201 -0.460636 1.454924
1 3.226518 -2.013667 0.659551
8 -3.948829 0.473745 1.046119
1 -4.094589 1.357203 1.412005
1 -3.204126 0.582289 0.407259
d_1m5mU/d_1m5mU94.xyz
7 -1.822655 -0.496422 0.004577
6 -1.664830 0.892320 0.000883
7 -0.422344 1.423284 -0.008908
6 0.682885 0.632827 -0.013705
6 0.545997 -0.816352 -0.010231
6 -0.720421 -1.311789 -0.001283
8 1.840502 1.180696 -0.021897
8 -2.709352 1.607143 0.006995
6 -3.177421 -1.054986 0.014854
1 -0.922255 -2.377543 0.001592
1 -3.108161 -2.142759 0.013937
1 -3.716423 -0.727825 0.907132
1 -3.729955 -0.727295 -0.868869
6 1.755124 -1.708295 -0.016343
1 2.380737 -1.522785 -0.896992
1 2.382976 -1.532249 0.864676
1 1.460539 -2.761991 -0.021331

8 4.429091 0.398487 0.032748
1 4.503269 -0.565609 0.027712
1 3.454347 0.577060 0.014642
d_1m5mU/d_1m5mU50_a.xyz
7 -0.488605 0.802901 -0.200031
6 -0.680306 -0.576154 -0.273509
7 0.371899 -1.409047 -0.174542
6 1.637517 -0.935871 0.011271
6 1.864703 0.501791 0.101669
6 0.775602 1.306318 -0.010440
8 2.611454 -1.752501 0.106240
8 -1.865475 -1.008951 -0.439443
6 -1.644377 1.701141 -0.294007
1 0.847355 2.387356 0.042005
1 -1.284044 2.726372 -0.379082
1 -2.240010 1.455997 -1.174779
1 -2.271705 1.613058 0.596189
6 3.249645 1.044675 0.310841
1 3.685976 0.669827 1.244411
1 3.923148 0.739524 -0.498820
1 3.238785 2.138130 0.352616
8 -4.271565 -0.257377 0.628170
1 -4.711770 -1.110505 0.745954
1 -3.382815 -0.477513 0.258826
d_1m5mU/d_1m5mU72.xyz
7 -0.504305 0.859634 -0.281468
6 -0.721605 -0.507139 -0.449424
7 0.297889 -1.374318 -0.318305
6 1.558408 -0.945875 -0.019211
6 1.811034 0.477011 0.174024
6 0.753049 1.317129 0.029274
8 2.505981 -1.791144 0.087758
8 -1.902157 -0.890048 -0.733848
6 -1.625980 1.790475 -0.443046
1 0.846540 2.391122 0.148966
1 -1.244614 2.810371 -0.394279

1 -2.108914 1.631503 -1.409093
1 -2.364549 1.644911 0.348669
6 3.191601 0.966373 0.506712
1 3.555785 0.520749 1.440011
1 3.907714 0.691229 -0.276759
1 3.205323 2.054913 0.617653
8 -3.963776 -0.618644 1.051223
1 -4.066532 0.317443 1.272247
1 -3.205259 -0.648615 0.419946
d_1m5mU/d_1m5mU53.xyz
7 -0.506780 0.829448 -0.273861
6 -0.705342 -0.544642 -0.403287
7 0.331365 -1.391643 -0.271463
6 1.588949 -0.936830 -0.001384
6 1.821826 0.494148 0.153664
6 0.748050 1.313331 0.006847
8 2.551113 -1.764398 0.113050
8 -1.883389 -0.956219 -0.655608
6 -1.645659 1.742164 -0.420259
1 0.825611 2.391165 0.100456
1 -1.271012 2.764721 -0.467619
1 -2.190188 1.515361 -1.338215
1 -2.327160 1.648577 0.428896
6 3.199071 1.013542 0.452669
1 3.584625 0.596951 1.390817
1 3.907732 0.730808 -0.334864
1 3.196454 2.104543 0.536892
8 -4.049507 -0.541315 0.964763
1 -4.235232 0.407181 1.003342
1 -3.250031 -0.618772 0.390420
d_1m5mU/d_1m5mU60.xyz
7 -1.396837 0.594155 -0.087554
6 -1.413711 -0.804003 -0.037854
7 -0.279051 -1.496564 -0.274691
6 0.890944 -0.861835 -0.561970
6 0.928776 0.594990 -0.636602

6 -0.232218 1.255818 -0.385447
8 1.952712 -1.540243 -0.758669
8 -2.512404 -1.373406 0.232877
6 -2.634691 1.331277 0.181422
1 -0.299050 2.338317 -0.408972
1 -2.431279 2.399060 0.097998
1 -3.407292 1.053588 -0.539479
1 -2.997858 1.110612 1.187992
6 2.212621 1.303836 -0.959820
1 2.989943 1.066227 -0.223963
1 2.598107 0.997071 -1.939431
1 2.069766 2.388675 -0.970707
8 1.935133 0.152476 2.511295
1 1.345577 0.639614 1.914907
1 2.349463 -0.507414 1.934963
d_1m5mU/d_1m5mU19.xyz
7 -0.499980 0.755368 -0.033530
6 -0.653519 -0.630394 -0.037003
7 0.430135 -1.429587 -0.022417
6 1.692094 -0.912583 0.002642
6 1.880681 0.533386 0.014928
6 0.760525 1.301415 -0.004996
8 2.697900 -1.695376 0.015587
8 -1.833197 -1.105650 -0.055892
6 -1.690114 1.611831 -0.050056
1 0.801843 2.385330 -0.000214
1 -1.372083 2.654025 -0.077936
1 -2.297108 1.397916 -0.932218
1 -2.292010 1.443843 0.845918
6 3.262294 1.121669 0.047487
1 3.811000 0.793736 0.938451
1 3.849582 0.802047 -0.821588
1 3.223031 2.215163 0.051848
8 -4.455959 -0.350489 0.090386
1 -3.486405 -0.526015 0.043182
1 -4.530645 0.613657 0.105530

d_1m5mU/d_1m5mU17.xyz
7 1.375484 -0.589725 0.139548
6 1.405520 0.803971 0.015433
7 0.273610 1.517265 0.197811
6 -0.905530 0.908536 0.503633
6 -0.956591 -0.542227 0.657804
6 0.202221 -1.224797 0.458325
8 -1.963491 1.604695 0.650477
8 2.513127 1.348395 -0.268954
6 2.610031 -1.350107 -0.073296
1 0.259470 -2.304864 0.542349
1 2.394605 -2.411018 0.055254
1 2.992205 -1.179427 -1.082474
1 3.373155 -1.046746 0.647434
6 -2.248493 -1.223607 1.009310
1 -2.634382 -0.868189 1.972194
1 -3.021470 -1.014408 0.260441
1 -2.115161 -2.307928 1.071353
8 -1.788866 -0.359017 -2.544420
1 -1.343458 -0.783306 -1.793981
1 -2.177728 0.439910 -2.158156
d_1m5mU/d_1m5mU69.xyz
7 0.757722 0.644264 0.483709
6 -0.049189 1.607445 -0.129773
7 -1.311761 1.291887 -0.489314
6 -1.817778 0.044079 -0.281741
6 -0.985613 -0.984001 0.333350
6 0.275075 -0.624633 0.690519
8 -3.017456 -0.221337 -0.624897
8 0.445425 2.757540 -0.324343
6 2.135741 0.992586 0.842042
1 0.964027 -1.318246 1.160779
1 2.602151 0.132495 1.322702
1 2.705773 1.260566 -0.051178
1 2.145471 1.839761 1.531548
6 -1.526985 -2.366931 0.557238

1 -2.414068 -2.346115 1.201611
1 -1.833607 -2.830541 -0.388052
1 -0.776707 -3.010216 1.027214
8 3.397774 -1.930641 -0.990462
1 3.101426 -2.722708 -1.461784
1 2.590349 -1.413830 -0.855799
d_1m5mU/d_1m5mU71.xyz
7 1.314379 0.529897 -0.363065
6 1.476812 -0.711493 0.263631
7 0.419480 -1.540886 0.390990
6 -0.812617 -1.198005 -0.077146
6 -1.007157 0.093692 -0.729960
6 0.082772 0.900858 -0.838331
8 -1.791770 -2.003260 0.055816
8 2.630292 -1.010708 0.691219
6 2.470138 1.424000 -0.478437
1 0.032466 1.878363 -1.305735
1 2.161624 2.333628 -0.994357
1 2.850678 1.682387 0.512709
1 3.268495 0.940328 -1.046021
6 -2.361956 0.489639 -1.245125
1 -2.716753 -0.213519 -2.007881
1 -3.105733 0.487262 -0.439390
1 -2.335916 1.491052 -1.685082
8 -1.767127 1.727089 2.032326
1 -1.278274 1.367065 1.273959
1 -2.672722 1.413236 1.893648
d_1m5mU/d_1m5mU66.xyz
7 1.406649 -0.596490 0.076516
6 1.434573 0.800442 0.002769
7 0.305828 1.505894 0.229992
6 -0.866445 0.885590 0.538391
6 -0.911846 -0.568519 0.654056
6 0.241452 -1.242978 0.403977
8 -1.924059 1.574680 0.719837
8 2.536793 1.356067 -0.281741

6 2.637010 -1.348094 -0.186703
1 0.301386 -2.325105 0.451717
1 2.420140 -2.413453 -0.106220
1 3.007021 -1.130328 -1.191217
1 3.409976 -1.080864 0.538018
6 -2.194875 -1.261712 1.014116
1 -2.559801 -0.934994 1.995174
1 -2.984279 -1.032067 0.288793
1 -2.059339 -2.347223 1.041703
8 -2.067088 -0.230348 -2.439670
1 -2.497030 0.397214 -1.838920
1 -1.429807 -0.690573 -1.871648
d_1m5mU/d_1m5mU07.xyz
7 -1.366905 0.586710 -0.161452
6 -1.399894 -0.804431 -0.011117
7 -0.268185 -1.522866 -0.173707
6 0.913688 -0.921856 -0.484452
6 0.968397 0.526056 -0.663365
6 -0.190695 1.213988 -0.484294
8 1.970728 -1.622550 -0.615140
8 -2.510000 -1.341528 0.277212
6 -2.601054 1.352896 0.031884
1 -0.245504 2.292405 -0.588625
1 -2.383504 2.410941 -0.115227
1 -3.361520 1.037461 -0.686461
1 -2.987895 1.201296 1.042342
6 2.263452 1.199079 -1.019512
1 3.032101 1.001737 -0.263025
1 2.653951 0.826523 -1.974038
1 2.132338 2.282368 -1.101165
8 1.728563 0.428063 2.562305
1 2.028122 -0.439231 2.251551
1 1.339850 0.833314 1.770875
d_1m5mU/d_1m5mU81.xyz
7 -1.244107 0.370981 -0.486360
6 -1.367389 -0.819948 0.238471

7 -0.269781 -1.558891 0.504775
6 0.966248 -1.171091 0.082182
6 1.117832 0.065264 -0.677823
6 -0.010732 0.783902 -0.920299
8 1.986046 -1.886761 0.353903
8 -2.525676 -1.160763 0.621097
6 -2.444763 1.169313 -0.748208
1 0.007759 1.719878 -1.468077
1 -2.161717 2.054687 -1.318088
1 -3.170213 0.587799 -1.321914
1 -2.907768 1.478560 0.191987
6 2.475035 0.511987 -1.142008
1 3.157092 0.650272 -0.294524
1 2.934687 -0.234258 -1.800893
1 2.410083 1.458283 -1.687615
8 1.063950 2.209903 1.984450
1 0.411426 2.648647 1.418847
1 1.303921 1.415905 1.481888
d_1mC/d_1mC_2_002.xyz
6 1.410353 -0.794861 -0.000004
6 -0.941082 -0.515164 0.000007
6 -0.764627 0.925595 0.000006
6 0.493509 1.426662 0.000002
1 0.699537 2.491235 0.000001
7 1.590059 0.600076 -0.000001
7 0.173522 -1.321036 0.000001
8 2.457319 -1.511375 -0.000011
7 -2.162147 -1.016710 0.000015
1 -2.123240 -2.036227 0.000014
1 -1.623618 1.585907 0.000007
6 2.950344 1.140359 0.000002
1 3.493700 0.808335 0.888251
1 3.493702 0.808343 -0.888249
1 2.895754 2.229464 0.000006
8 -4.375415 0.492524 -0.000015
1 -3.528103 -0.076546 -0.000003

1 -4.063979 1.408431 -0.000002
d_1mC/d_1mC_1_061.xyz
6 0.800574 -0.903536 0.001775
6 -0.925045 0.716650 0.001331
6 0.075676 1.770202 -0.000552
6 1.385561 1.425671 -0.001896
1 2.184377 2.158926 -0.003454
7 1.776458 0.110906 -0.001425
7 -0.504719 -0.591416 0.002511
8 1.217146 -2.102328 0.003824
7 -2.224147 0.959037 0.002122
1 -2.397666 1.962974 0.000702
1 -0.219206 2.813440 -0.001217
6 3.193518 -0.255858 -0.002055
1 3.442921 -0.834760 0.890839
1 3.436471 -0.852844 -0.884557
1 3.790842 0.656570 -0.013621
8 -4.168477 -0.877616 0.004424
1 -3.408426 -0.194993 0.002366
1 -3.733518 -1.738222 -0.071110
d_1mC/d_1mC_2_046.xyz
6 1.408516 -0.800617 0.007405
6 -0.937293 -0.477608 -0.010346
6 -0.734649 0.959088 -0.016503
6 0.532537 1.436949 -0.010830
1 0.757836 2.497591 -0.015265
7 1.613986 0.590663 -0.000057
7 0.161954 -1.303515 0.003294
8 2.441257 -1.537205 0.017421
7 -2.167256 -0.958253 -0.018101
1 -2.144557 -1.978288 -0.010880
1 -1.581851 1.633825 -0.026441
6 2.984154 1.105917 0.002942
1 3.517591 0.768130 0.894987
1 3.524491 0.759286 -0.881537
1 2.949835 2.195942 -0.002632

8 -4.402596 0.503867 -0.078177
1 -4.568519 0.794524 0.829185
1 -3.544494 -0.048946 -0.033329
d_1mC/d_1mC_2_022.xyz
6 -1.408376 0.804094 -0.000942
6 0.935847 0.468202 0.004701
6 0.725322 -0.967495 0.003884
6 -0.544453 -1.438379 -0.000076
1 -0.775497 -2.497785 -0.001070
7 -1.621455 -0.586181 -0.002425
7 -0.159286 1.300109 0.002139
8 -2.437126 1.546518 -0.002672
7 2.168265 0.941381 0.008537
1 2.151992 1.961546 0.009902
1 1.568711 -1.647199 0.005986
6 -2.994390 -1.094186 -0.004583
1 -3.530782 -0.749205 0.882939
1 -3.528175 -0.749147 -0.893643
1 -2.965838 -2.184429 -0.004528
8 4.367343 -0.589809 0.021974
1 5.100808 -0.016690 -0.240768
1 3.540674 0.008662 0.011101
d_1mC/d_1mC_2_053.xyz
6 -1.410767 -0.792880 0.003106
6 0.942138 -0.526262 -0.001321
6 0.773586 0.915499 -0.004252
6 -0.481670 1.423559 -0.004676
1 -0.681864 2.489243 -0.007300
7 -1.582741 0.602995 -0.001751
7 -0.176816 -1.326018 0.003315
8 -2.461768 -1.503473 0.006670
7 2.160852 -1.033783 -0.002881
1 2.117378 -2.053082 0.000944
1 1.636427 1.570693 -0.006732
6 -2.939842 1.151158 -0.003158
1 -3.485257 0.819915 -0.890439

1 -3.485195 0.825110 0.886083
1 -2.878704 2.239889 -0.006284
8 4.350581 0.512055 -0.023274
1 3.518831 -0.078611 -0.010990
1 4.038146 1.389391 0.238562
d_1mC/d_1mC_1_032.xyz
6 0.808379 0.904958 -0.005828
6 -0.923435 -0.708575 -0.003093
6 0.073258 -1.765888 0.004515
6 1.384427 -1.426384 0.007294
1 2.180400 -2.162708 0.013032
7 1.780411 -0.113177 0.002971
7 -0.498126 0.597784 -0.007689
8 1.229355 2.102164 -0.011746
7 -2.223394 -0.946720 -0.006096
1 -2.399739 -1.950187 -0.000261
1 -0.225602 -2.807982 0.008464
6 3.199022 0.247809 0.004403
1 3.454547 0.814635 -0.894587
1 3.440631 0.854669 0.880423
1 3.792445 -0.666896 0.029907
8 -4.187616 0.867933 -0.029519
1 -3.821189 1.684208 0.337870
1 -3.417552 0.196770 -0.012768
d_1mC/d_1mC_1_048.xyz
6 0.811602 -0.905752 0.005615
6 -0.922928 0.704537 0.001599
6 0.071681 1.763684 -0.008128
6 1.383469 1.426623 -0.009502
1 2.178086 2.164403 -0.015967
7 1.781809 0.114189 -0.002543
7 -0.495555 -0.601027 0.006238
8 1.234750 -2.102153 0.012167
7 -2.223331 0.940661 0.006522
1 -2.401399 1.943803 -0.000499
1 -0.229189 2.805185 -0.013896

6 3.201161 -0.243994 -0.000962
1 3.455659 -0.810753 0.898340
1 3.446063 -0.849838 -0.876786
1 3.792760 0.671958 -0.024487
8 -4.187236 -0.869777 0.054064
1 -3.416571 -0.199280 0.022628
1 -3.915898 -1.597384 -0.522427
d_1mC/d_1mC_2_031.xyz
6 -0.446109 0.322613 0.000012
6 1.162886 -1.419473 0.000000
6 2.218134 -0.419402 0.000000
6 1.882083 0.891078 0.000001
1 2.618481 1.686690 0.000001
7 0.566112 1.292742 0.000007
7 -0.150150 -0.981127 0.000018
8 -1.647404 0.754517 0.000013
7 1.474836 -2.697175 -0.000013
1 0.606102 -3.236831 -0.000010
1 3.258777 -0.720794 -0.000004
6 0.208820 2.712425 -0.000016
1 -0.376768 2.963869 -0.887933
1 -0.376684 2.963922 0.887943
1 1.125862 3.302745 -0.000077
8 -3.818241 -0.840258 -0.000009
1 -2.966283 -0.327390 0.000002
1 -3.554790 -1.770811 -0.000026
d_1mC/d_1mC_2_003.xyz
6 -0.388895 -0.803412 -0.107040
6 0.873306 1.226639 -0.062609
6 -0.369341 1.969816 0.060033
6 -1.541048 1.293885 0.094419
1 -2.499011 1.792614 0.189342
7 -1.585605 -0.079092 0.003678
7 0.787776 -0.153011 -0.148872
8 -0.466181 -2.066367 -0.168750
7 2.022963 1.862906 -0.087007

1 2.775129 1.175916 -0.177026
1 -0.355019 3.050950 0.126544
6 -2.853717 -0.806139 0.096060
1 -2.978309 -1.468163 -0.763547
1 -2.889201 -1.406489 1.009690
1 -3.671062 -0.084408 0.111307
8 3.058490 -1.642861 0.206086
1 2.221319 -1.105413 0.055782
1 3.759906 -0.981535 0.289457
d_1mC/d_1mC_2_036.xyz
6 0.464786 0.245144 -0.206052
6 -1.323199 -1.302890 -0.029464
6 -2.234699 -0.194901 0.204598
6 -1.747382 1.067160 0.214018
1 -2.373372 1.936927 0.378544
7 -0.409617 1.319481 0.012404
7 0.016692 -1.013947 -0.227343
8 1.695277 0.539049 -0.382780
7 -1.785817 -2.533900 -0.047560
1 -1.003156 -3.167559 -0.225301
1 -3.290169 -0.379182 0.365659
6 0.111627 2.688040 0.011433
1 0.846885 2.822390 0.808997
1 0.585853 2.918662 -0.945690
1 -0.718270 3.376990 0.172672
8 3.656627 -1.203535 0.248216
1 3.864609 -1.003899 1.171429
1 2.896775 -0.605196 0.020490
d_1mC/d_1mC_2_042.xyz
6 0.330573 0.788662 -0.111016
6 -0.833361 -1.298769 -0.056710
6 0.442446 -1.980959 0.078224
6 1.580831 -1.249859 0.106542
1 2.561416 -1.701398 0.207840
7 1.560434 0.123022 0.002195
7 -0.813093 0.082145 -0.151879

8 0.345372 2.053878 -0.175884
7 -1.952885 -1.985979 -0.085065
1 -2.731241 -1.330338 -0.187047
1 0.479119 -3.060801 0.156726
6 2.792740 0.910771 0.077312
1 2.878320 1.569544 -0.789443
1 2.808170 1.519665 0.985764
1 3.644590 0.229886 0.090432
8 -3.099316 1.543744 0.191230
1 -2.256543 1.013537 0.044729
1 -2.792845 2.425534 0.445356
d_1mC/d_1mC_1_035.xyz
6 -0.472502 0.335270 0.000005
6 1.127463 -1.411265 0.000000
6 2.186007 -0.409834 0.000003
6 1.854347 0.902119 0.000004
1 2.593868 1.695105 0.000003
7 0.540969 1.306088 0.000002
7 -0.181264 -0.967478 0.000002
8 -1.673787 0.770218 0.000007
7 1.352258 -2.708617 -0.000005
1 2.361450 -2.863436 -0.000007
1 3.227846 -0.710736 0.000003
6 0.186640 2.726485 -0.000009
1 -0.398199 2.979070 -0.888032
1 -0.398203 2.979084 0.888009
1 1.105143 3.314639 -0.000011
8 -3.752442 -0.949062 -0.000001
1 -3.416573 -1.856037 -0.000016
1 -2.940965 -0.373537 0.000000
d_1mC/d_1mC_1_007.xyz
6 -0.472614 -0.325583 -0.001216
6 1.148235 1.401539 0.001569
6 2.194761 0.387509 0.003161
6 1.847345 -0.920473 0.001246
1 2.577316 -1.722264 0.001948

7 0.529117 -1.308505 -0.001700
7 -0.165462 0.973342 -0.000423
8 -1.678922 -0.746204 -0.002023
7 1.387967 2.696273 0.001861
1 2.398925 2.839038 0.003380
1 3.240205 0.675727 0.005401
6 0.156938 -2.724148 -0.004222
1 -0.427405 -2.972271 0.885437
1 -0.434989 -2.966484 -0.890332
1 1.067705 -3.324113 -0.010164
8 -3.761476 0.968819 0.030777
1 -2.945459 0.400414 0.011615
1 -3.462464 1.848193 -0.238721
d_1mC/d_1mC_2_000.xyz
6 0.444981 0.332426 0.003858
6 -1.141226 -1.430682 -0.001165
6 -2.209324 -0.444329 -0.003697
6 -1.890311 0.870328 -0.002470
1 -2.637033 1.656301 -0.004361
7 -0.579723 1.289279 0.001177
7 0.166102 -0.975078 0.003154
8 1.640781 0.779548 0.007365
7 -1.437441 -2.712145 -0.002844
1 -0.562923 -3.242240 -0.000627
1 -3.245901 -0.759316 -0.006746
6 -0.241483 2.713799 0.001837
1 0.336381 2.973771 0.892377
1 0.344596 2.972339 -0.883697
1 -1.166599 3.291366 -0.002979
8 3.808691 -0.821486 -0.031903
1 3.553830 -1.722371 0.210858
1 2.963489 -0.297986 -0.009106
d_1mC/d_1mC_2_070.xyz
6 0.330601 -0.789536 -0.105629
6 -0.831722 1.298947 -0.055823
6 0.444978 1.980802 0.072173

6 1.582890 1.248954 0.100666
1 2.564096 1.700208 0.197048
7 1.561219 -0.124369 0.002256
7 -0.812680 -0.082399 -0.144402
8 0.344376 -2.054920 -0.167614
7 -1.950881 1.986771 -0.084182
1 -2.729945 1.331202 -0.181051
1 0.482704 3.061002 0.145082
6 2.793160 -0.912708 0.076953
1 2.810020 -1.519004 0.987116
1 2.876579 -1.574017 -0.788086
1 3.645502 -0.232377 0.086421
8 -3.103816 -1.534633 0.206795
1 -2.818715 -2.458674 0.238173
1 -2.257771 -1.010686 0.056102
d_1mC/d_1mC_2_058.xyz
6 -0.361093 -0.796933 -0.122105
6 0.853166 1.261701 -0.065401
6 -0.405178 1.973794 0.079629
6 -1.560458 1.270320 0.114798
1 -2.528917 1.745104 0.225640
7 -1.573619 -0.102117 0.005373
7 0.800068 -0.118818 -0.167058
8 -0.409564 -2.060511 -0.196165
7 1.987588 1.923816 -0.096047
1 2.751083 1.252032 -0.203308
1 -0.415209 3.053917 0.161865
6 -2.823721 -0.860221 0.095235
1 -2.933687 -1.519240 -0.768499
1 -2.844019 -1.466534 1.005408
1 -3.658570 -0.158827 0.115506
8 3.106776 -1.563283 0.142721
1 2.250981 -1.047225 0.025948
1 3.086090 -1.881000 1.056189
d_1mC/d_1mC_1_082.xyz
6 -0.313623 -0.788644 -0.115054

6 0.855040 1.292295 -0.060783
6 -0.423965 1.976441 0.072422
6 -1.564237 1.247866 0.105141
1 -2.543753 1.701817 0.208239
7 -1.544835 -0.123377 0.003444
[truncated: 145,194 more chars]
